# Combined transcriptome studies identify *AFF3* as a mediator of the oncogenic effects of β-catenin in adrenocortical carcinoma

**DOI:** 10.1038/oncsis.2015.20

**Published:** 2015-07-27

**Authors:** L Lefèvre, H Omeiri, L Drougat, C Hantel, M Giraud, P Val, S Rodriguez, K Perlemoine, C Blugeon, F Beuschlein, A de Reyniès, M Rizk-Rabin, J Bertherat, B Ragazzon

**Affiliations:** 1 Inserm, U1016, Institut Cochin, Paris, France; 2 Cnrs, UMR8104, Paris, France; 3 Université Paris Descartes, Sorbonne Paris Cité, France; 4 Endocrine Research Unit, Medizinische Klinik und Poliklinik IV, Klinikum der Universität München, Munich, Germany; 5 Clermont Université, Université Blaise Pascal, GReD, Clermont-Ferrand Cedex 1, France; 6 CNRS, UMR 6293, GReD, Aubière Cedex, France; 7 Inserm, U1103, GReD, Aubière Cedex, France; 8 Ecole Normale Supérieure, Institut de Biologie de l'ENS, IBENS, Plateforme Génomique, Paris, France; 9 Inserm, U1024, Paris, France; 10 CNRS, UMR 8197, Paris, France; 11 Programme Cartes d'Identité des Tumeurs, Ligue Nationale Contre Le Cancer, Paris, France; 12 Department of Endocrinology, Referral Center for Rare Adrenal Diseases, Assistance Publique Hôpitaux de Paris, Hôpital Cochin, Paris, France

## Abstract

Adrenocortical cancer (ACC) is a very aggressive tumor, and genomics studies demonstrate that the most frequent alterations of driver genes in these cancers activate the Wnt/β-catenin signaling pathway. However, the adrenal-specific targets of oncogenic β-catenin-mediating tumorigenesis have not being established. A combined transcriptomic analysis from two series of human tumors and the human ACC cell line H295R harboring a spontaneous β-catenin activating mutation was done to identify the Wnt/β-catenin targets. Seven genes were consistently identified in the three studies. Among these genes, we found that *AFF3* mediates the oncogenic effects of β-catenin in ACC. The Wnt response element site located at nucleotide position −1408 of the AFF3 transcriptional start sites (TSS) mediates the regulation by the Wnt/β-catenin signaling pathway. *AFF3* silencing decreases cell proliferation and increases apoptosis in the ACC cell line H295R. AFF3 is located in nuclear speckles, which play an important role in RNA splicing. AFF3 overexpression in adrenocortical cells interferes with the organization and/or biogenesis of these nuclear speckles and alters the distribution of CDK9 and cyclin T1 such that they accumulate at the sites of AFF3/speckles. We demonstrate that *AFF3* is a new target of Wnt/β-catenin pathway involved in ACC, acting on transcription and RNA splicing.

## Introduction

Adrenocortical cancer (ACC) is a very aggressive tumor with a 5-year survival rate below 35% in most series.^1^ There are few effective treatments available.^2^ At present, surgery is the only curative therapy available and is only effective if complete tumor removal is possible. In progressive patients, medical therapy is of very limited efficacy. Several studies show the importance of the Wnt/β-catenin signaling pathway in the development and maintenance of numerous organs, and that alterations of the Wnt/β-catenin signaling are involved in a wide range of human diseases, and especially malignancies.^3, 4, 5, 6^

The Wnt/β-catenin pathway is required for normal adrenal (NA) gland formation, and adrenocortical cell-specific knockout of *Ctnnb1* in mice results in adrenal gland aplasia.^7^ Gene alterations leading to a constitutive activation of this pathway are the most frequent events in ACC.^8^ Constitutive activation of the Wnt/β-catenin pathway in the adrenal cortex of transgenic mice leads to the development of adrenocortical tumors with malignant characteristics.^9, 10^ Inhibition of the Wnt/β-catenin pathway in the adrenocortical cell line H295R by PKF115-584 or a shRNA against β-catenin messenger RNA (mRNA) increases apoptosis^11, 12^ and is associated with a complete absence of tumor growth in a xenograft model.^12^

These observations implicate alterations of Wnt/β-catenin in ACC pathogenesis. Few Wnt/β-catenin target genes, including the canonical target *AXIN2*, have been shown to be upregulated in ACC,^13^ but there has been no comprehensive and integrative study to identify all specific Wnt/β-catenin targets in ACC. A better understanding of the biological processes affected by Wnt/β-catenin pathway alterations in ACC may allow the identification of therapeutic targets and thereby contribute to the development of new therapeutic strategies. The aim of this study was to use a combination of transcriptome analysis and cellular experiments to identify the targets of the Wnt/β-catenin pathway involved in adrenocortical tumorigenesis.

## Results

### Identification of Wnt/β-catenin targets in ACC

We performed a combined transcriptomic analysis on cohorts of ACC and on human cell models to identify alterations of gene expression due to aberrant Wnt/β-catenin pathway activation.

**Figure 1:**
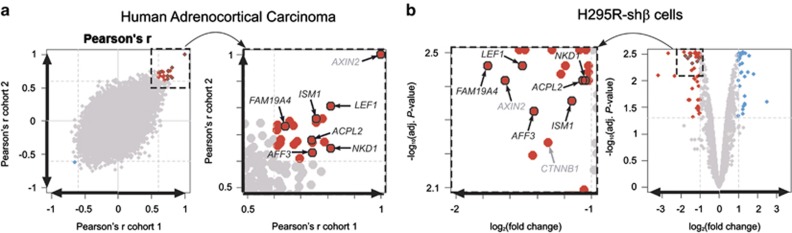
Identification of Wnt/βcatenin target genes in adrenocortical cancer. (**a**) To identify gene's expression that is closely correlated with *AXIN2* expression and that might also be Wnt/β-catenin targets, we performed Pearson's *r* correlation tests with expression data sets from two independent cohorts of ACC (cohort 1^8^ and cohort 2^10^). Each dot in left panels represents the Pearson correlation coefficient (*r*) for each gene in the two cohorts (detailed in figure S01-A and Supplementary Table S1). The genes best correlated with *AXIN2* are represented in red (positively; *r*>0.6 in both cohorts) or blue (negatively; *r*<–0.6 in both cohorts). (**b**) The volcano plot in the right panels shows the differential expression of genes in three stable clones of the human adrenocortical cell line H295R, which carries a construct encoding a doxycyclin-inducible shRNA targeting *CTNNB1* mRNA (shβ). Dots in gray represent genes that did not show significant changes in expression, dots in red on the left indicate the genes with significantly downregulated expression (adjust *P*-value<0.05 and log2 ratio<–1) and dots in blue on the right indicate the genes with significantly upregulated expression (adjust *P*-value<0.05 and log2 ratio>1). Black circles represent the genes common to both analyses.

Two independent ACC microarray data sets^8, 10^ were analyzed for genes whose expression is correlated with the common Wnt/β-catenin pathway target gene, *AXIN2* (Supplementary Figure S1-A and Supplementary Table S1); combining results of the two cohorts, 21 mRNAs were positively correlated (adjust *P*-value<0.05 and Pearson's *r*>0.6 in both cohorts) and 1 mRNA was negatively correlated (adjust *P*-value<0.05 and Pearson's *r*<−0.6 in both cohorts) (Figure 1a).

We used the H295R cell line, human adrenocortical cells, harboring a heterozygous *CTNNB1* (β-catenin) gene mutation affecting the GSK3β phosphorylation site (S45P) and leading to constitutive transcriptional activity of β-catenin-lymphoid enhancer binding factor (LEF)/T-cell factor (TCF). Whole-transcript gene expression was analyzed in three stable clones of H295R cells expressing a doxycyclin-inducible small hairpin RNA (shRNA)-targeting *CTNNB1* mRNA (shβ).^12^ We established a list of genes showing similar expression profiles in the three clones after *CTNNB1* silencing. A control clone was used to eliminate from this list those genes whose expression was sensitive to doxycyclin treatment. We thereby identified 44 genes significantly downregulated (adjusted *P*-value<0.05 and log2 ratio<−1) and 29 genes significantly upregulated (adjusted *P*-value<0.05 and log2 ratio>1) by specific *CTNNB1* inactivation (Figure 1b, Supplementary Figure S1-B and Supplementary Table S1).

We further studied only the genes common to the two lists. In addition to *AXIN2*, there were six genes positively associated in both experiments with Wnt/β-catenin pathway activation: lymphoid enhancer binding factor 1 (*LEF1*); AF4/FMR2 family, member 3 (*AFF3*); family with sequence similarity 19 (chemokine (C–C motif)-like), member A4 (*FAM19A4*); isthmin 1; angiogenesis inhibitor (*ISM1*), acid phosphatase-like 2 (*ACPL2*); and naked cuticle homolog 1 (Drosophila) (*NKD1*) (Figure 1, black circles, 2a and Supplementary Table S1). Two of these genes are already known to be transcriptional targets of the Wnt/β-catenin pathway; one, *NKD1*, acts as a negative feedback regulator, and the other, *LEF1*, mediates, with β-catenin, a nuclear response.

**Figure 2:**
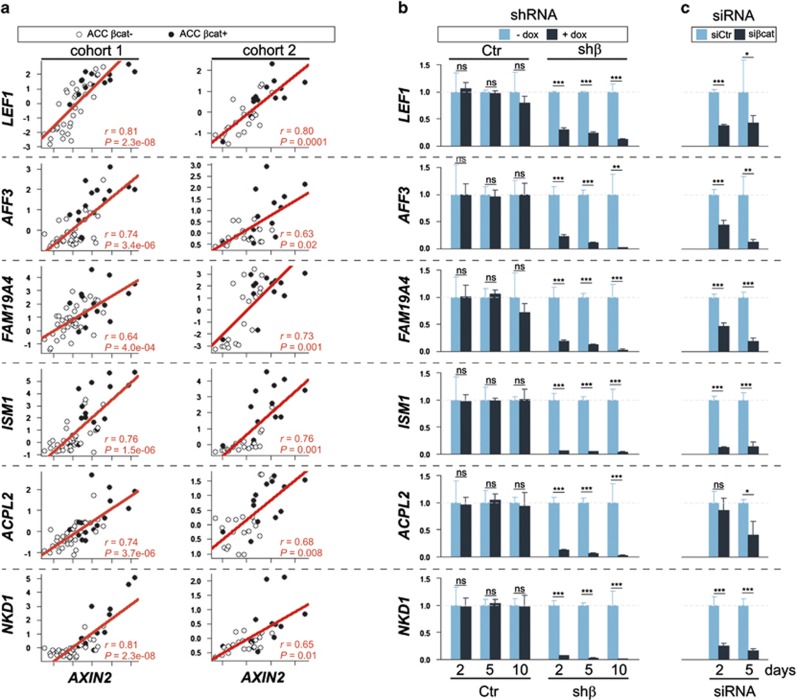
β-catenin-dependent genes expression in adrenocortical carcinoma and cell lines. (**a**) Pearson correlation between log2 values of *AXIN2* expression and expression of genes identified in Figure 1 (*LEF1*, *AFF3*, *FAM19A4*, *ISM1*, *ACPL2*, *AXIN2* and *NKD1*) in both cohorts of ACC. Each dot represents ACC without (white) or with (black) Wnt/β-catenin pathway activation. (**b**) Histograms represent levels of the mRNAs of *LEF1*, *AFF3*, *FAM19A4*, *ISM1*, *ACPL2*, *AXIN2* and *NKD1* in Ctr and shβ clones 2, 5 and 10 days after addition of doxycyclin (dox, 0.2 mg/ml) to the culture medium and (**c**) in parental H295R cells after transient transfection with siCtr or siβcat.

We validated the Wnt/β-catenin pathway-dependent expression of all these six genes by real time-PCR in H295R-shβ cells in parental H295R cells transiently transfected with a small interfering RNA construct targeting another region of the *CTNNB1* mRNA, and in established tumors with *CTNNB1* inactivation from a subcutaneous xenograft model (Figures 2b and c and Supplementary Figure S2-A and B).

**Figure 3:**
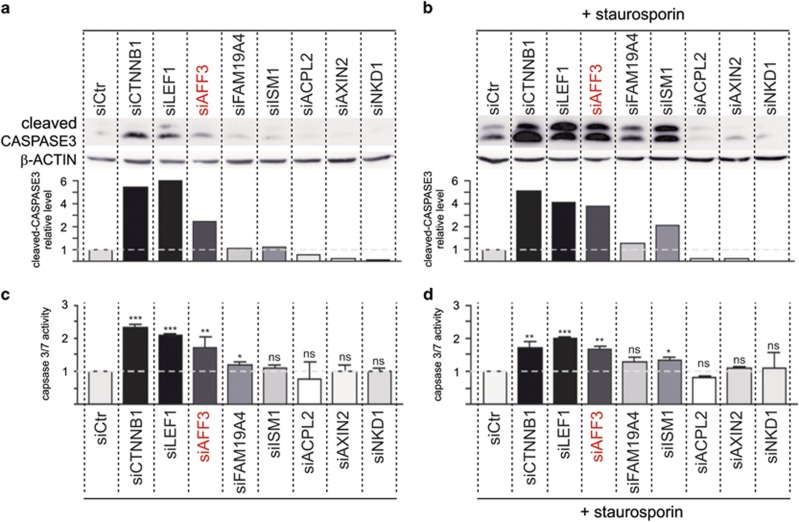
Genes silencing and apoptosis. Apoptosis was analyzed by quantification of cleaved caspase-3 level (**a** and **b**) and caspase-3/7 activity (**c** and **d**) in H295R cells after gene silencing for 5 days without (**a** and **c**) and with staurosporin (b and **d**) for the 6 last hours (50 ng/ml). Histograms in **a** an **b** represent the quantification of cleaved caspase-3 proteins from one experiment representative of three independent experiments. Histograms in **c** and **d** represent caspase-3/7 activity from three independent experiments.

*CTNNB1* (β-catenin) silencing in adrenocortical cells H295R leads to increased apoptosis (reference 12 and Figure 3). We therefore investigated if silencing of the potential β-catenin target genes mimicked this effect on apoptosis. LEF1 silencing had similar effects to CTNNB1 silencing on apoptosis, as expected since LEF1 is involved in nuclear response of Wnt/β-catenin pathway. Of the other genes (*AFF3, FAM19A4*, *ISM1*, *ACPL2*, *AXIN2* and *NKD1*), *AFF3* silencing had the largest effect on apoptosis (Figure 3 and Supplementary Figure S2-C). We therefore focused our analysis on *AFF3* and investigated its role, if any, in adrenocortical oncogenesis.

### AFF3, a new Wnt/β-catenin target

**Figure 4:**
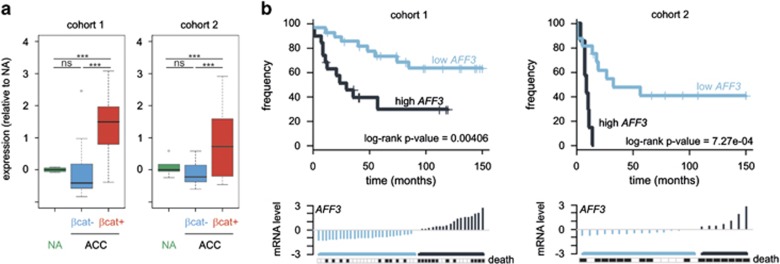
*AFF3* expression in ACC. (**a**) Boxplots representing the expression level of *AFF3* in ACC positive for nuclear β-catenin staining (βcat+, in red) compared with ACC negative for such nuclear staining (βcat–, blue) and in normal adrenal tissue (NA, in green) from two independent expression data sets (cohort 1^8^ and cohort 2^10^). (**b**) The Kaplan–Meier estimates of overall survival of patients with ACC according to *AFF3* expression. The *P*-value of the log-rank test for differences between survival curves is shown. Below, ACC are ordered by their *AFF3* expression value centered on the cutoff value and the specific death is indicated by black rectangles.

The dysregulation of Wnt/β-catenin pathways allows β-catenin to accumulate and translocate to the nucleus, where it may activate the transcription of target genes. This nuclear accumulation can be detected by immunohistochemistry. *AFF3* was more abundant in ACC positive for nuclear β-catenin staining (βcat+) than in ACC without such nuclear staining (βcat−) and in NA in both cohorts (Figure 4a). *AFF3* is not more strongly expressed in NA than in other tissues (Supplementary Figure S3). *AFF3* expression was significantly associated with a poor overall survival in the two cohorts of ACC, whether analyzed as a continuous variable (cohort 1: *P*=0.00927, hazard ratio=1.68, 95% confidence interval=1.12–2.51; cohort 2: *P*=0.00649, hazard ratio=2.2, 95% confidence interval=1.19–4.09) or after dichotomization between ACC with low and high *AFF3* expression (Figure 4b).

**Figure 5:**
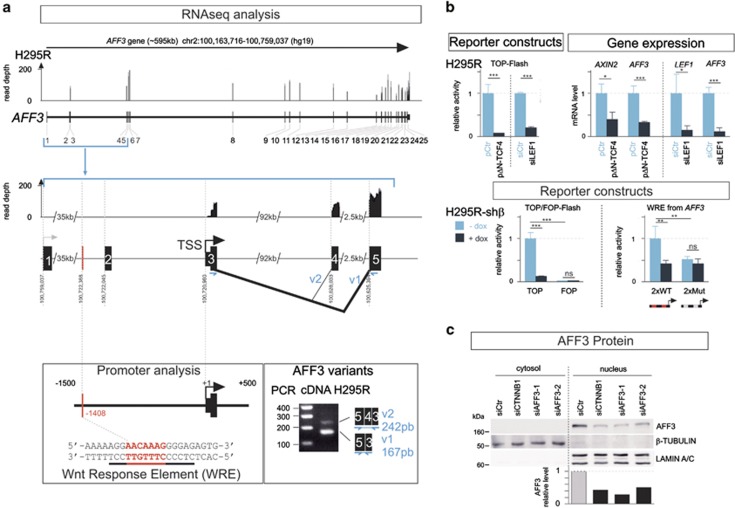
*AFF3* is a β-catenin target gene in adrenocortical carcinoma cells. (**a**) Genomic structure of the *AFF3* gene with the two putative transcription start sites (TSS) at the beginning of exons 1 and 3 indicated by two arrows. Each black rectangle represents an exon. *Y* axis values show the read depth of RNA-seq analysis on adrenocortical cells H295R. Finer details of the *AFF3* TSS regions are shown and demonstrate that only the TSS at exon 3 is used. Promoter analysis (nucleotide positions –1500 to +500) by Genomatix software identified one Wnt Response Element (WRE) shown in the lower left box. The AFF3 variant mRNAs produced were analyzed by producing cDNA from total mRNA from H295R cells, then PCR using oligonucleotides corresponding to sequences in exon 3 and exon 5, followed by agarose gel electrophoresis; 167 bp: isoform 1 and 242 bp: isoform 2. (**b**) The upper histograms represent the effects in H295R cells of ΔN-TCF4 (an inhibitor of the β-catenin/TCF pathway) and *LEF1* silencing on the β-catenin-LEF/TCF-dependent luciferase reporter construct Top-flash (left panel) and expression of other genes as indicated (right panel). The lower histograms represent the effects of β-catenin silencing by dox treatment of the shβ line on the expression of Top-Flash and luciferase constructs, with two copies of the putative *AFF3* WRE wild-type sequence (2xWT) or mutant sequence (2xMut). (**c**) Western blot analysis of AFF3 protein accumulation in H295R cells (cytosol/nucleus) after *CTNNB1* (siCTNNB1) or *AFF3* (siAFF3-1 and 2) silencing. Histograms represent the quantification of AFF3 proteins from one experiment representative of three independent experiments.

Wnt/β-catenin regulation of gene expression generally involves binding of LEF/TCF transcription factors to Wnt response elements (WREs) and recruitment of the activator β-catenin. The *AFF3* gene consists of 23 coding exons and two 5′ non-coding exons, with two major transcriptional start sites (TSS).^14^ To assess the transcriptional activity of *AFF3* in human adrenal cells, we mapped transcriptome sequencing reads with mRNA (RNA-seq) from H295R cells and we examined the regions surrounding the two putative TSSs (Figure 5a). There was no transcription of exons 1 or 2 in the RNA-seq from H295R, whereas there were high levels of exon 3 reads; these findings suggest that only the TSS located at the beginning of exon 3 is used in adrenocortical cells. Analysis of the *AFF3* promoter region from positions –1500 to +500 bp from this TSS revealed one putative WRE (Figure 5a). In H295R cells, ΔN-TCF4, an inhibitor of β-catenin/TCF, decreased the transcriptional activity of the β-catenin-LEF/TCF-dependent luciferase reporter construct top-flash and decreased *AFF3* mRNA levels, similarly to *AXIN2* (Figure 5b). *LEF1* silencing had a similar effect and led to decreases *AFF3* mRNA levels (Figure 5b). Two copies of the putative *AFF3* WRE (wild type or mutant) were inserted in tandem into the reporter plasmid, pGL3Tk, where luciferase expression is driven by the minimal Tk promoter (2xWT or 2xMut). In transfected cells the transcriptional activity of the reporter construct (2xWT) was higher than that of the mutated construct (2xMut) (Figure 5b, shβ-MUT-Luc 0.5±0.07 vs shβ-WT-Luc: 1±0.3, *P*<0.01). The transcriptional activity of the 2xWT was lower (0.4±0.07, *P*<0.01) in H295R cells expressing a shRNA-β-catenin (shβ with dox). Doxycyclin treatment did not affect activity of the 2xMut construct with mutated LEF/TCF sites. These results are consistent with the profiles of the canonical reporter constructs TOP/FOP-Flash (Figure 5b).

Two nuclear protein isoforms can be produced from the *AFF3* gene, and differ owing to the alternative splicing of exon 4 without frameshift. PCR analysis of cDNA using oligonucleotides corresponding to parts of exon 3 and exon 5 showed that the mRNA for isoform 1 (v1), without exon 4 (NP_002276.2, 1226 amino acid), was the most abundant of the two mRNAs in adrenocortical cells (Figure 5a). Consistent with this result, only one protein was detected in nuclear protein extracts of adrenocortical cells, and its abundance was β-catenin-dependent (Figure 5csiCTNNB1 vs siCtr).

### AFF3 silencing alters apoptosis and proliferation

**Figure 6:**
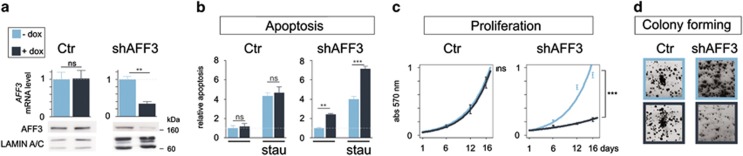
*AFF3* silencing alters apoptosis, proliferation and colony forming. (**a**) Histogram of *AFF3* mRNA levels and western blotting showing protein accumulation in Ctr and shAFF3 clones 5 days after addition of doxycyclin (dox) to the culture medium (0.2 mg/ml). (**b**) In the apoptosis illustration, histograms represent caspase-3/7 activity, after vehicle or dox treatment for 5 days, without or with staurosporin co-treatment for the last 6 h (50 ng/ml). (**c**) Cell survival, reported in graph labeled proliferation, was assessed by the MTT assay without or with dox (0.2 mg/ml) treatment for 1, 6, 12 or 16 days. (**d**) Colony formation by Ctr and shAFF3 clones without or with dox (0.2 mg/ml) treatment for 30 days was visualized by crystal-violet staining.

Adrenocortical cell lines carrying constructs encoding a doxycylin-inducible shRNA targeting *AFF3* were generated (shAFF3). Five days following shRNA-AFF3 induction by doxycyclin (dox) treatment, *AFF3* mRNA and protein levels were significantly decreased (Figure 6a). In contrast, doxycyclin treatment had no effect on *AFF3* expression in control lines (Ctr) (Figure 6a). *AFF3* silencing by shRNA (+dox) increased the apoptosis rate and increased caspase activity (Figure 6b), a result that agrees with the findings of the transient knockdown experiment (Figure 3). Likewise, *AFF3* silencing increased the apoptotic effect of staurosporin (staurosporin had no significant effect on controls). The effect of *AFF3* silencing on H295R cell proliferation was measured by MTT (3-(4, 5-dimethylthiazolyl-2)-2,5-diphenyltetrazolium bromide) and colony-formation assays: silencing significantly decreased proliferation (−75% at 16 days, *P*<0.001, Figure 6c) and reduced the rate of colony formation (Figure 6d), with respect to the shAFF3 cell line without *AFF3* silencing (–dox) or the control line.

### AFF3 alters nuclear speckles and interacts with P-TEFb

**Figure 7:**
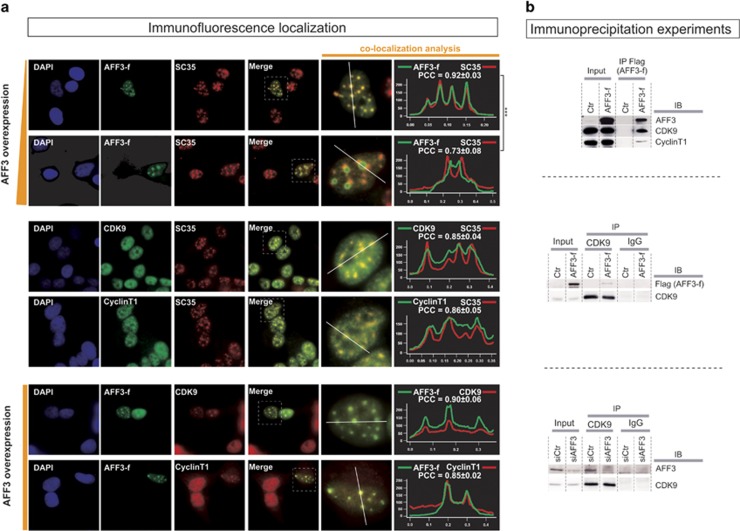
*AFF3* overexpression alters nuclear speckles and pTEFb. (**a**) Lines 1 and 2: H295R cells were transfected with AFF3-Flag vector. The AFF3 protein was revealed with an anti-Flag antibody and nuclear speckles were detected using an anti-SC35 antibody. After 8 h of expression of the vector, AFF3 colocalized with SC35 in nuclear speckles; after 24 h, the distribution of SC35 was altered. Lines 3 and 4: CDK9 and cyclin T1 protein were detected using anti-CDK9 and anti-cyclin T1 antibodies. In H295R cells, CDK9 and cyclin T1 colocalized with SC35 in nuclear speckles. Lines 5 and 6: H295R cells were transfected with AFF3-Flag vector. AFF3 overproduction resulted in CDK9 and cyclin T1 being found colocalized with AFF3. The white dashed rectangle in the merge column represents the enlarged area used for colocalization analysis using ImageJ software. Pearson's colocalization coefficients (PCC) were determined with JACoP plugin^49^ in cells from different experiments (*n*⩾10). *t*-test was used to evaluate the difference between PCC for AFF3/SC35 colocalization analysis after 8 and 24 h of transfection (lines 1 and 2). (**b**) Protein lysates from H295R cells overexpressing AFF3-Flag or Ctr vectors were immunoprecipitated with Anti-Flag M2 Affinity Gel and analyzed by western blot using the indicated antibodies. Overproduced AFF3 associated with CDK9 and Cyclin T1. Protein lysates from H295R cells overexpressing AFF3-Flag or silenced for AFF3 by siAFF3 were immunoprecipitated with anti-CDK9 antibodies and analyzed by western blot using the indicated antibodies. CDK9 associated with overproduced and endogenous AFF3. *AFF3* silencing in H295R cells confirmed the specificity of the protein band of endogenous AFF3.

AFF (AF4/FMR2) proteins have been found in nuclear speckles,^15^ which serve as a reservoir of factors participating in mRNA splicing. AFF (AF4/FMR2) proteins have been also described in the super elongation complex (SEC), which contains the positive transcription elongation factor b (P-TEFb) and other elongation factors,^16^ and positively regulates transcriptional elongation by the RNA polymerase II. In adrenocortical cells, AFF3 colocalized with the SC35 protein (a nuclear speckle marker) (Figure 7a, line 1). Strong overproduction of AFF3 altered the nuclear distribution of SC35 protein (Figure 7a, line 2) although the overproduction of another speckle component (acinus) had no effect on the distribution of SC35 (Supplementary Figure S4). To determine whether AFF3 is present in SEC in adrenocortical cells, AFF3 was immunoprecipitated from nuclear extracts. CDK9 and cyclin T1, both components of P-TEFb, co-immunoprecipitated with AFF3 (Figure 7b, top). The interactions were confirmed by reciprocal co-IP of endogenous CDK9 and overproduced AFF3-flag (Figure 7b, middle) or endogenous AFF3 (Figure 7b, bottom). CDK9 and cyclin T1 were present in the nucleus where spots of high concentration were observed; these spots colocalized with SC35 (Figure 7a, lines 3 and 4). AFF3 overproduction altered the nuclear distribution of CDK9 and cyclin T1, and seemed to concentrate them at the sites of AFF3 accumulation (Figure 7a, lines 5 and 6), consistent with immunoprecipitation results. Similar results were obtained in HeLa cells (data not shown).

## Discussion

**Figure 8:**
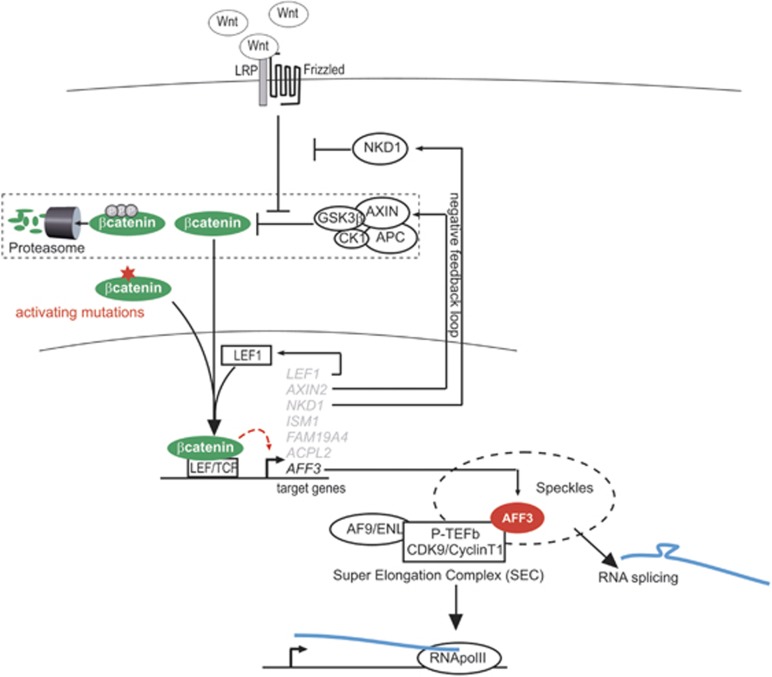
Target genes of the Wnt/β-catenin pathway in adrenocortical carcinoma. In the absence of Wnt factors, β-catenin is phosphorylated by GSK3β and CK1α as part of a multiprotein complex. Phosphorylated β-catenin is then degraded by proteasomes. In the presence of Wnt factors, Wnt binds to its frizzed receptor inhibiting the degradation complex that leads to cytoplasmic accumulation of β-catenin. Then, β-catenin migrates to the nucleus and associates with the LEF/TCF transcription factor to induce transcription of specific target genes. The CTNNB1 mutation leads to a constitutive transcriptional activity of β-catenin-LEF/TCF. Here, we have identified candidate genes regulated by the Wnt/β-catenin pathway in adrenocortical carcinoma. Among these genes, we identified *AFF3* as a direct target of the Wnt/β-catenin pathway. AFF3 is localized in nuclear speckles and is associated with P-TEFb. AFF3 may have a role in adrenocortical tumorigenesis by acting on transcription and RNA splicing. Two genes may regulate the Wnt/β-catenin pathway: *LEF1* may participate in transcriptional activation and *NKD1* has been described as negative regulator. It might be also interesting to study the roles, if any, of *FAM19A4, ACPL2* and *ISM1* in adrenocortical tumorigenesis.

In many cancers, the Wnt/β-catenin signaling pathway has an important role in regulating cell growth, motility and differentiation.^6, 17^ We and others have demonstrated the importance of the Wnt/β-catenin signaling pathway activation in adrenal cortex tumorigenesis.^9, 10, 13, 18, 19, 20, 21, 22, 23^ Using combined transcriptomic analysis we have identified new target genes of the Wnt/β-catenin signaling pathway. We show that one of these genes, *AFF3*, mediates some of the effects of Wnt/β-catenin signaling pathway activation in ACC (Figure 8). We have identified the *AFF3* TSS used in adrenocortical cells at the beginning of exon 3. This predominantly produces a mature mRNA devoid of exon 4, which encodes isoform 1 of the AFF3 protein (1226 amino acid). We also report that the WRE site located at nucleotide position –1408 of the AFF3 TSS, mediates the regulation by the Wnt/β-catenin signaling pathway.

The AFF (AF4/FMR2) family of genes includes four members: *AFF1* (or AF4, acute lymphoblastic leukemia-1 fused gene from chromosome 4), *AFF2* (or FMR2, Fragile X mental retardation 2), *AFF3* and *AFF4* (or AF5Q31, acute lymphoblastic leukemia-fused gene from 5q31). *AFF2* is silenced in cases of Fragile XE (FRAXE) intellectual disability. Proteins encoded by *AFF1, AFF3* and *AFF4* are produced as fusions with that encoded by *MLL* in acute lymphoblastic leukemia patients.^24, 25, 26^ In the mouse, *Aff3* shows strong regional expression in the developing brain, somites and limb buds.^14, 27, 28, 29^ This expression pattern is consistent with mesomelic dysplasia and central nervous system abnormalities observed in a female infant with a microdeletion on chromosome 2, only affecting the *AFF3* gene.^28^ A recent study identified a CGG repeat expansion, associated with intellectual disability, in the promoter of *AFF3* at an autosomal folate-sensitive fragile site named FRA2A.^14^ This polymorphic repeat is hypermethylated in FRA2A, leading to silencing of *AFF3* in the nervous system.^14^ *Aff3* is expressed in the mouse adrenal cortex during embryonic development (E14.5) and thus may be implicated in adrenal development (Supplementary Figure S5). *AFF3* has been identified as a novel susceptibility locus for several autoimmune diseases, notably rheumatoid arthritis,^30, 31, 32, 33^ type I diabetes^34, 35^ and Graves' disease.^36^ *AFF3* has been found to be abnormally expressed in ~20% of breast cancers, suggesting that it may act as a proto-oncogene.^37^ A gene set enrichment analysis of several expression data sets from breast cancers shows a link between genes correlated to *AFF3* expression and the Wnt/β-catenin pathway (Supplementary Figure S6-A). Moreover, *AFF3* expression is positively correlated with *CCND1* expression (Supplementary Figure S6-B), which is a direct target of the Wnt/β-catenin pathway in breast cancer cells.^38^ These observations suggest that *AFF3* expression is Wnt/β-catenin-dependent in breast cancers as it is in ACC.

In H295R adrenocortical cells, AFF3 is located in nuclear speckles; its overproduction interferes with the organization and/or biogenesis of these nuclear speckles, confirming previous findings in HeLa cells.^15^ Several lines of evidence point to speckles being storage/assembly/modification compartments that supply splicing factors to active transcription sites.^39^ CDK9 and cyclin T1, two major components of P-TEFb involved in transcriptional elongation via phosphorylation of the RNA polymerase II, are diffusely distributed throughout the nucleoplasm with an overlap with nuclear speckles.^39^ We report that in H295R adrenocortical cells, AFF3 interacts with CDK9 and cyclin T1, and its overproduction alters the nuclear distribution of these two proteins such that they accumulate at the sites of AFF3/speckles. AFF3 and P-TEFb form the SEC-like 3 recently described in HEK293 cells.^16^ The SEC consists of the RNA polymerase II elongation factors eleven-nineteen Lys-rich leukemia (ELL) proteins, EAF1/2 (ELL-associated factor) proteins, P-TEFb and several frequent myeloid/lymphoid or mixed-lineage leukemia translocation partners, such as AF9, ENL, AFF1, AFF4 and AFF3. It is one of the most active P-TEFb-containing complexes and is required for rapid induction of transcription by paused RNA polymerase II. The significant overlap of genes downregulated by *AFF3* silencing with genes controlled by CDK9 (determined by inhibition of CDK9 by Flavopiridol^40^) suggests that AFF3 modulates P-TEFb activity in adrenocortical cells (Supplementary Figure S6–C).

Two recently developed inhibitors (JQ1 and I-BET151) of bromodomain and extraterminal family members block cell proliferation of leukemia cell lines. They do so by indirectly interfering with the recruitment of SEC to chromatin from key cancer-related genes such as v-myc avian myelocytomatosis viral oncogene homolog (*MYC*), B-cell lymphoma 2 (*BCL2*) and cyclin-dependent kinase 6 (*CDK6*).^41, 42, 43^ It would therefore be worth pursuing the development of inhibitors that more directly interfere with the organization or stability of the SEC. Such inhibitors would be potential anticancer drugs for use against ACCs and other cancers, such as breast cancer, with overexpression of SEC components and in particular members of the AFF family.

## Materials and methods

### Cell culture, generation of clones and cells transfection

ACC cells (H295R, ATCC CRL-2128) and HeLa cells (ATCC CCL-2) were cultured, transfected and stimulated with staurosporin as previously described.^12, 44, 45, 46^ H295R stably transfected with the Tet repressor (H295R/TR) was kindly provided by Dr Lalli.^47^ H295R/TR/shRNA-βcatenin (shβ) clones carrying constructs encoding doxycyclin-inducible shRNAs targeting either *CTNNB1* (βcatenin) are described in.^12^ The pSuperior.puro vector (Oligoengine), which can expresses a doxycyclin-inducible shRNA, was used to created the H295R/TR/shRNA-AFF3 clones. Silencing hairpin targeted *AFF3* mRNA (targeted sequence: 5′-TAAGGACTCTCAGCTTGTA-3′) was cloned into pSuperior.puro vector following the manufacturer's instructions to give finally the pSuperior.puro/shRNA-*AFF3* vector. H295R/TR cells were further transfected with the pSUPERIOR.puro/shRNA-*AFF3* vector and clones were selected with puromycin (5 μg/ml, Sigma-Aldrich P9620, Saint-Quentin Fallavier, France). Three shRNA-*AFF3* clones (shAFF3 clones) were selected in which *AFF3* expression was downregulated at least 0.5-fold in a doxycyclin (0.2 mg/ml, Sigma)-dependent manner in comparison with control clone (Ctr) transfected with pSuperior.puro vector. All cell clones were investigated for their ability to express specific adrenocortical genes (*StAR* and *CYP11B1*). S45P *CTNNB1* (*β-catenin*) gene activating mutation, previously identified in the parental H295R cell line, was confirmed by direct sequencing in all Ctr and shAFF3 clones (data not shown). The data presented are from a single Ctr clone and a single shRNA clone, but all results from *in vitro* experiments were confirmed by similar findings with three independent shRNA clones and two independent Ctr clones.

### Small interfering RNA, plasmids and constructs

The following small interfering RNA were used: siCTNNB1 (5′-AGCUGAUAUUGAUGGACAG-3′); siLEF1 (5′-CUACAGGAAUCUGCAUCAG-3′); siAFF3-1 (5′-AGAUGACCUUAAGCUAAGC-3′); siAFF3-2 (5′-UAAGGACUCUCAGCUUGUA-3′); si-FAM19A4 (5′-GGATGAGAGTCTGTGCTAA-3′); siISM1 (5′-AUAUCCAGGUCACCAUAGA-3′); siACPL2 (5′-GAAUGGAAUGAGUAGCAAG-3′); siAXIN2 (5′-UCAAGAAGCAGCAGAUUGA); siNKD1 (5′-GGAUGUGGCACAUAUAUAC-3′); siCtr (5′-AGCUGAUAUUGAUGGACAG-3′). The AFF3-Flag-tagged vector from origene (RC221621, Origene, Rockville, MD, USA) was used to overexpress AFF3. As a Wnt/β-catenin pathway reporter construct-driving expression of luciferase gene the Top plasmid was used, which contains two copies of the β-catenin/TCF-binding sites, whereas the Fop plasmid contains two mutated copies of the β-catenin/TCF-binding sites. Two copies of the putative *AFF3* WRE (wild type or mutant) were tandemly inserted in the *Xho*I site of a pGL3Tk reporter plasmid, where luciferase expression is driven by the minimal Tk promoter. Double-stranded oligonucleotides with 5′ and 3′ *Xho*I overhangs were ligated in the open pGL3Tk vector. 2xWT-F, 5′-tcgaAGGAACAAAGGGGAGAGGAACAAAGGGGAG-3′ 2xWT-R, 5′-tcgaCTCCCCTTTGTTCCTCTCCCCTTTGTTCCT-3′ 2xMut-F, 5′-tcgaAGGGCCAAAGGGGAGAGGGCCAAAGGGGAG-3′ 2xMut-R, 5′-tcgaCTCCCCTTTGGCCCTCTCCCCTTTGGCCCT-3′. Inserted copy number was verified by DNA sequencing of the inserts. Rous sarcoma virus (RSV)-Renilla (Promega, Charbonnieres, France) was used as a control of transfection efficiencies. Cell tranfection was performed using effecten (Qiagen 301427). Firefly and Renilla luciferase activities were sequentially measured with the Dual Luciferase Reporter Assay System (Promega) and results are expressed as firefly luciferase activity normalized to Renilla luciferase activity of the same sample.

### RNA extraction, RT-qPCR and PCR experiments

RNA extractions from cell lines were performed using Promega kit (Z6012). Reverse transcription of RNA were performed using High-capacity cDNA reverse transcription kit (Applied biosystems, Foster City, CA, USA 4368813). The qPCR were performed using the light cycler 480 SYBR green (Roche, Meylan, France, 04887352001). The PCR conditions for all target genes were: activation at 95 °C for 5 min followed by 45 cycles of 95 °C for 5 s, annealing at 60 °C for 5 s and 72 °C for 1 s/25 bases with the primer pairs (forward and reverse) described below: *CTNNB1*: (5′-CATTACAACTCTCCACAACC-3′ and 5′-CAGATAGCACCTTCAGCAC-3′, 281 bp); *LEF1* (5′-CAGTCATCCCGAAGAGGAAG-3′ and 5′-GCTCCTGAGAGGTTTGTGCT-3′, 122 bp); *AFF3* (5′-ACTCAACAGGATGATGGCAC-3′ and TGCCTAAAGTGTTCTGGATC-3′, 109 bp); *FAM19A4* (5′-TCGCACTGGCTCTTTCTAGC-3′ and 5′-ACCTCACAGGTCCCTTGCTT-3′, 119 bp); *ISM1* (5′-CCCCAGATCCTTTCTCCTTG-3′ and 5′-GTCGACCACCTCTATGGTGA-3′, 103 bp); *ACPL2* (5′-TGACTGCACTCTGGTGGCTA-3′ and 5′-AGGAGTTCAAGGGGCTTTCG-3′, 110 bp); *AXIN2* (5′-AGTGTGAGGTCCACGGAAAC-3′ and 5′-CTTCACACTGCGATGCATTT-3′, 250 bp); *NKD1* (5′-GACAACAACGGCAAGGTCAC-3′ and 5′-GATGTTGGGGAGTGGTTGAC-3′, 95 bp); *PPIA (CYCLO)* (5′-ATGGCACTGGTGGCAAGTCC-3′ and 5′-TTGCCATTCCTGGACCCAAA-3′, 241 bp). The *AFF3* variant mRNAs produced were analyzed by producing complementary DNA from total mRNA from H295R cells, then PCR using oligonucleotides (AFF3-iso-F, 5′-AATGGGACCTCGAGTCACTG-3′ and AFF3-iso-R, 5′-GGAGAGTTCATCCCCCTTGT-3′) corresponding to sequences in exon 3 and exon 5, followed by agarose gel electrophoresis

### Protein extraction, immunoprecipitation experiments and fluorescence immunostaining

H295R cells were washed with cold phosphate-buffered saline (PBS) once and lysed in a low-salt lysis buffer (20 mM Tris HCl (pH 7.4), 0,5% NP40, 20 mM NaCl, 1 mM ethylenediaminetetraacetic acid (EDTA) (pH 7.8)) containing proteinase and phosphatase inhibitors (Roche) for 5 min at 4 °C. After centrifugation at 5000 *g* for 5 min, the supernatant was removed (cytoplasmic extract). The nuclear buffer (50 mM Tris HCl (pH 7.4), 1% NP40; 400 mM NaCl; 1 mM EDTA (pH 7.8)) containing proteinase and phosphatase inhibitors (Roche) was added to the pellet. Then samples were vortexed 15 s every 10 min for a total of 30 min at 4 °C. After centrifugation at 16000 *g* for 5 min, the balance buffer (50 mM Tris HCl (pH 7.4), 1% NP40, 1 mM EDTA) containing proteinase and phosphatase inhibitors (Roche) was added to the supernatant to make the final NaCl concentration 150 mM (nuclear extract). Flag immunoprecipitation: the nuclear extract was incubated with anti-Flag M2 affinity gel (Sigma A2220) overnight at 4 °C with gentle rotation. The affinity gel were spun down and washed three times with wash buffer (50 mM Tris HCl (pH 7.4), 1% NP40, 150 mM NaCl, EDTA 1 mM (pH 7.8)) containing proteinase and phosphatase inhibitors (Roche). The elution was made with the peptide 3X Flag (sigma F4799) at 4 °C for 30 min without agitation. CDK9 immunoprecipitation: the lysate was then incubated with antibodies and protein A coupled magnetic sepharose beads (GE healthcare 28-9537-63 AA, Velizy-Villacoublay, France) overnight at 4 °C with gentle rotation. The beads were washed three times with wash buffer (50 mM Tris HCl (pH 7.4), 1% NP40, 150 mM NaCl, EDTA 1 mM (pH 7.8)) containing proteinase and phosphatase inhibitors (Roche) before boiling at 70 °C in 2% of sodium dodecyl sulfate-loading buffer for 10 min. Fluorescence immunostaining: H295R cells were washed with cold PBS once and were permeabilized using a solution of PBS with 0.5% Triton for 10 min. The cells were washed with PBS for three times before incubated with 10% normal goat serum blocking solution (Invitrogen 50-062Z) at room temperature for 1 h. The cells were then incubated with primary antibodies overnight at 4 °C. After three washes with PBS, secondary fluorescent antibodies were added to the cells for 1 h at room temperature. The nuclei were stained with 4′,6-diamidino-2-phenylindole and after mounting with Fluoromount G (DAKO, Les Ulis, France), images were taken with a fluorescence microscope (Zeiss Axiovert 200M). The following antibodies were used: Anti-cleaved Caspase3 (9661) and anti-LaminA/C (2032) (Cell Signaling, Saint Quentin Yvelines, France), Anti-β-ACTIN (A-2066, Sigma-Aldrich), Anti-AFF3 (ARP 34737-P050, Aviva, San Diego, CA, USA), Anti-Flag (F1804) and anti-Sc35 (s4045) (Sigma-Aldrich), Anti-CDK9 (ab 38840) and anti-Cyclin T1 (ab 2098) (Abcam, Paris, France), Anti-b-Tubulin (sc5274) and normal rabbit IgG (sc2027) (Santa Cruz, Dallas, TX, USA), Alexa Fluor 488 Anti-mouse IgG (A11001), Alexa Fluor 488 Anti-rabbit IgG (A11008), Alexa Fluor 647 Anti-rabbit IgG (A21244), Alexa Fluor 647 Anti-mouse IgG (A21235) (Life technologies, Saint Aubin, France).

### Apoptosis, cell proliferation and colony-formation assay

Apoptosis was analyzed by western blot testing of the amounts of cleaved caspase-3 and by Caspase-Glo 3/7 Assay (Promega G8091). Proliferation was measured with the MTT assay (CellTiter 96 Non-Radioactive Cell Proliferation Assay, Promega). For colony-formation assays, cells from each clone were seeded at low density in individual wells of a standard six-well plate and grown for 30 days in normal serum medium. Colonies were visualized by crystal-violet staining.

### Correlation, expression and statistical analysis

Pearson's *r* correlation test was used to evaluate the correlation between gene expression and *AXIN2* expression in two, previously described, independent cohorts of ACC: Cohort 1^8^ included 47 ACC and four NA (Gene Expression Omnibus data set GSE49280 and ArrayExpress data set E-TABM-311); and Cohort 2,^10^ 33 ACC and 10 NA (Gene Expression Omnibus data set GSE33371). All samples were normalized in batches, independently for each chip type using the RMA algorithm (Bioconductor *affy* package), and probe set intensities were then averaged per gene symbol. Both final matrices were row-centered using the NA median. Differential expression was measured with moderated *t*-test (limma R package) and survival curves were obtained by the Kaplan–Meier method. Differences in survival were assessed with the log-rank test. Gene expression profiles for three H295R/TR/shβ and one control clones with and without doxycycline treatment were analyzed using Affymetrix Human Gene 1.0 ST arrays (ArrayExpress data set E-MTAB-3330) and with the moderated paired *t*-test (limma R package). All *P*-values were adjusted using the Benjamini–Hochberg correction method. All *in vitro* data reported with statistical analyses represent means from at least three experiments. The error bars indicate the s.d. Control conditions were set as one and data were analyzed by one way analysis of variance followed by Fisher's LSD *post* *hoc* test. Gene set enrichment analysis^48^ were performed with several data sets from breast cancers (ArrayExpress data set E-MTAB-365; Gene Expression Omnibus (GEO) data sets GSE6532, GSE4922 and GSE1456; and The Cancer Genome Atlas breast carcinoma data set) and with a data set with inhibition of CDK9 by Flavopiridol.^40^ Significance was set at *P*<0.05 (represented by * in figures); *P*<0.01 (**) and *P*<0.001 (***). Analyses were performed using R 3.0.3 with custom scripts, which can be provided on request.

### RNA-seq library preparation and analysis

Total RNA from H295R cells was extracted, and complementary DNA library was created using Epicentre ScriptSeq strand-specific after a RNA depletion optimized with the Ribo-Zero kit (Epicentre, Madison, WI, USA). A paired-end 10- bp sequencing was performed on a HiSeq 1500 device (Illumina, San Diego, CA, USA). Reads were mapped to the UCSC (University of California, Santa Cruz) reference transcripts from hg19 genome using RSEM (RNA-Seq by Expectation Maximization) and the Bowtie alignment software. Wiggle files in genomic coordinate were generated for visualization.

## Notes

The authors declared no conflict of interest.
