# Supplementary figures and images for: Combined transcriptome studies identify AFF3 as a mediator of the oncogenic effects of β-catenin in adrenocortical carcinoma
Source: Oncogenesis. 2015 Jul 27;4(7):e161–. doi: 10.1038/oncsis.2015.20 (PMC4521181; doi:10.1038/oncsis.2015.20)

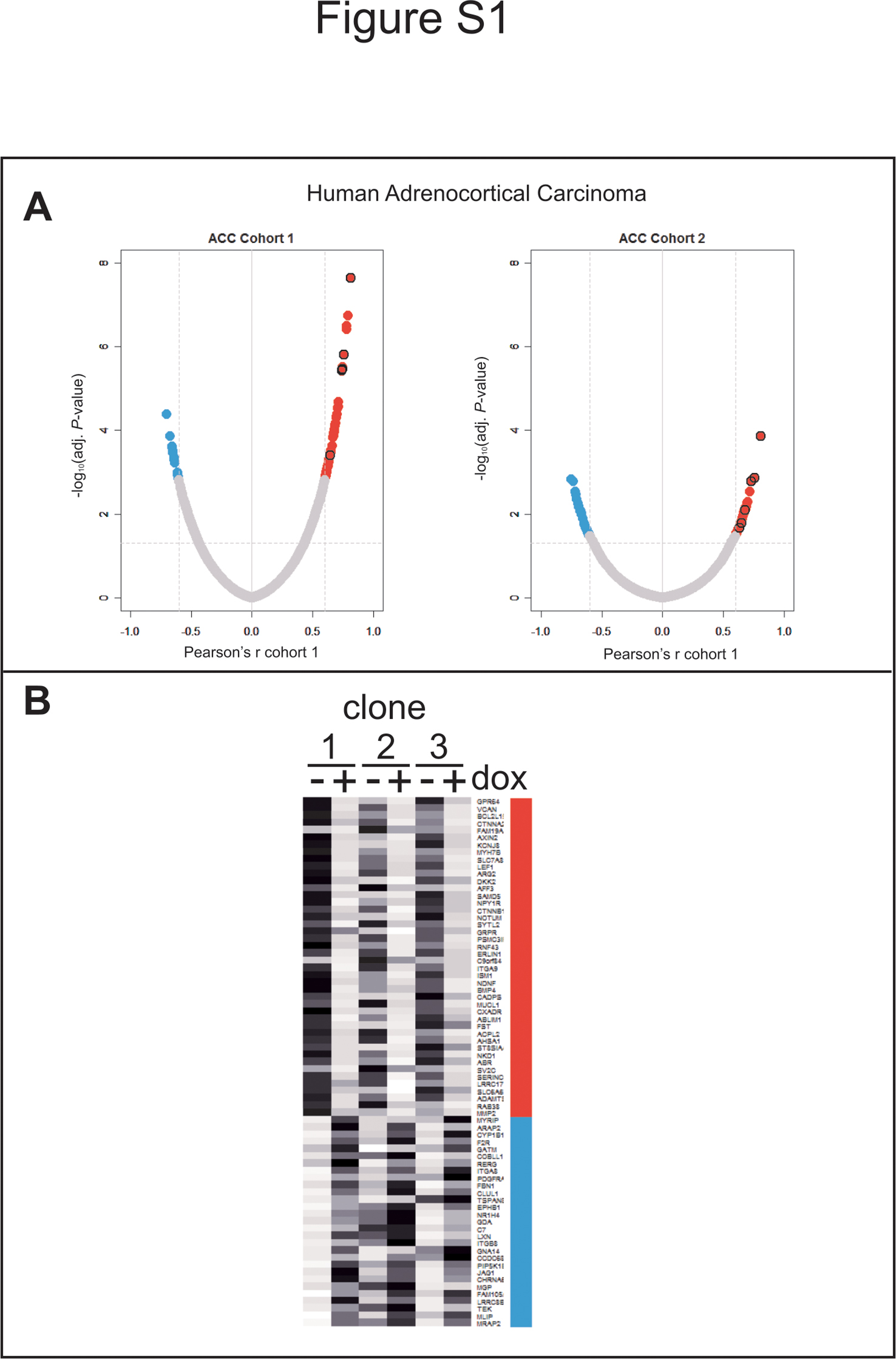

Supplement: Supplementary Figure S1 [file oncsis201520x1.tif]

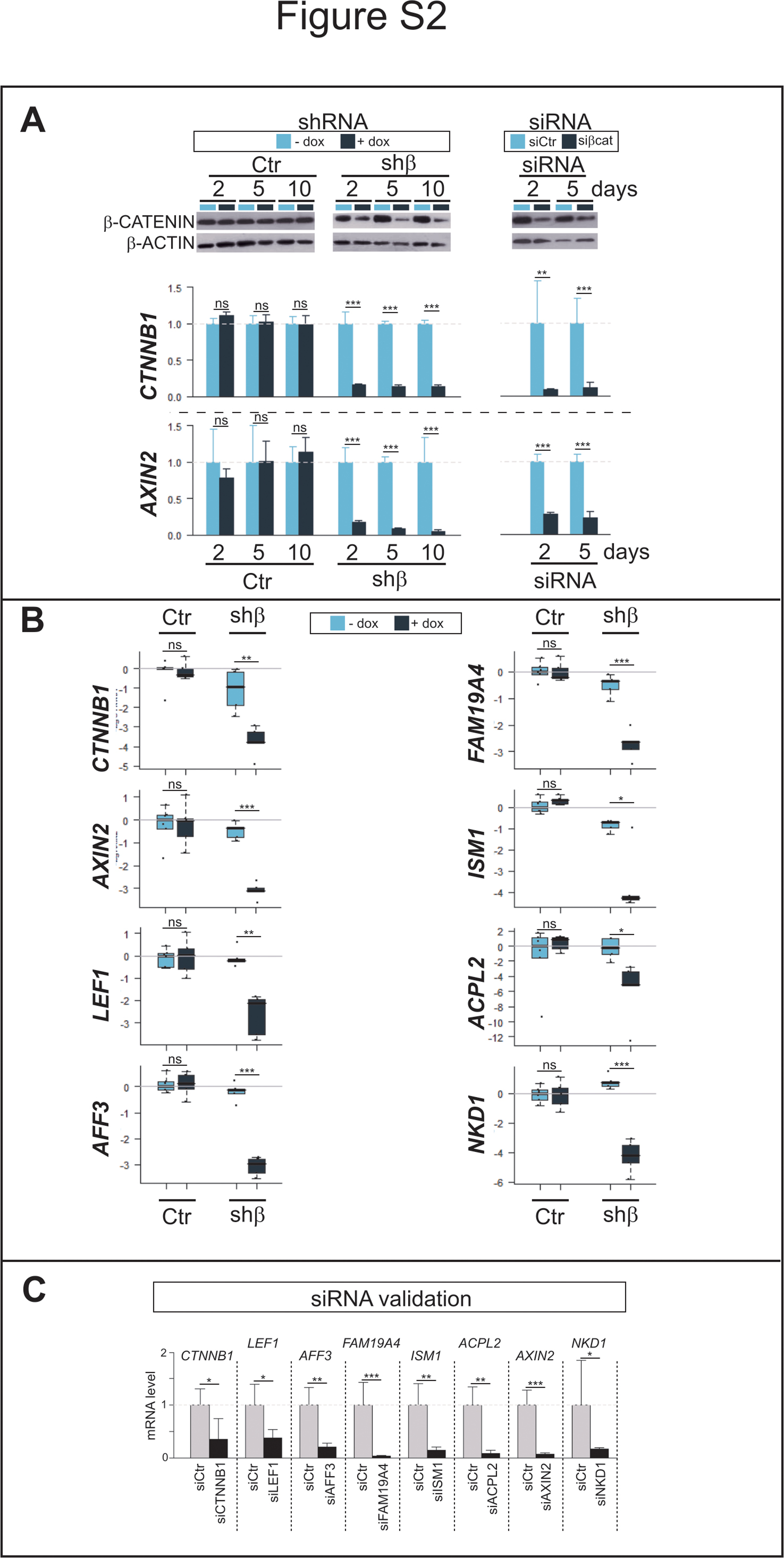

Supplement: Supplementary Figure S2 [file oncsis201520x2.tif]

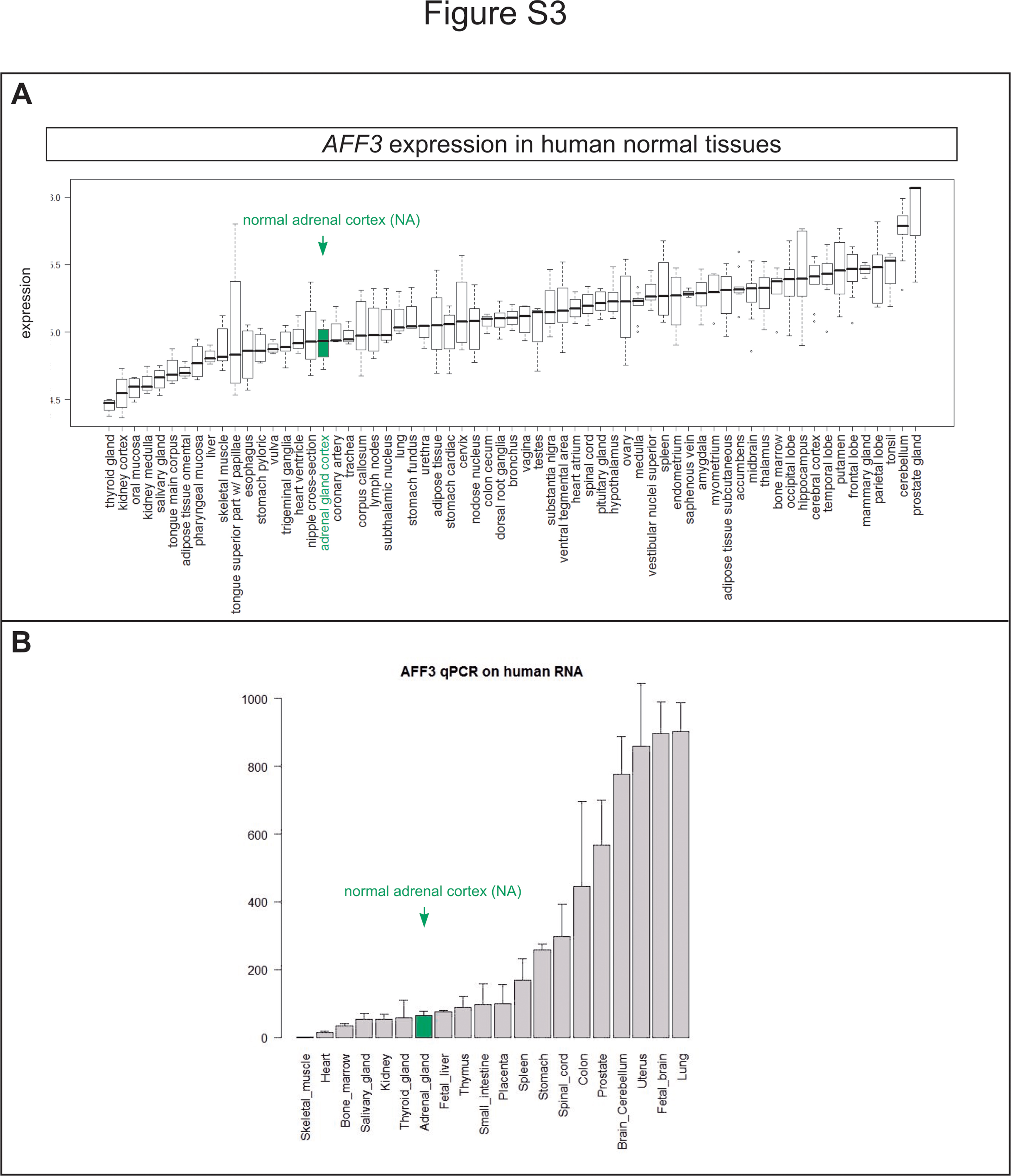

Supplement: Supplementary Figure S3 [file oncsis201520x3.tif]

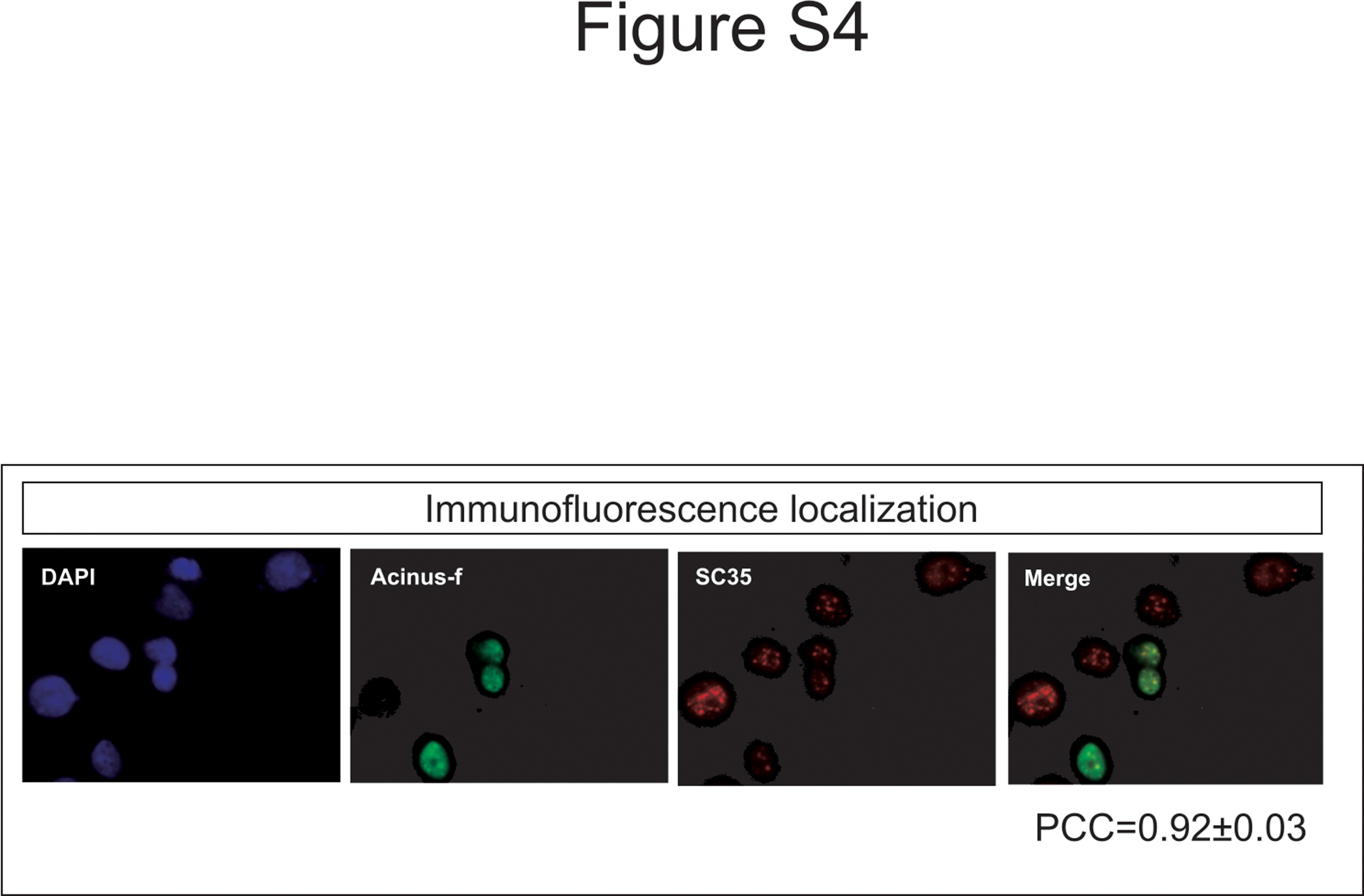

Supplement: Supplementary Figure S4 [file oncsis201520x4.tif]

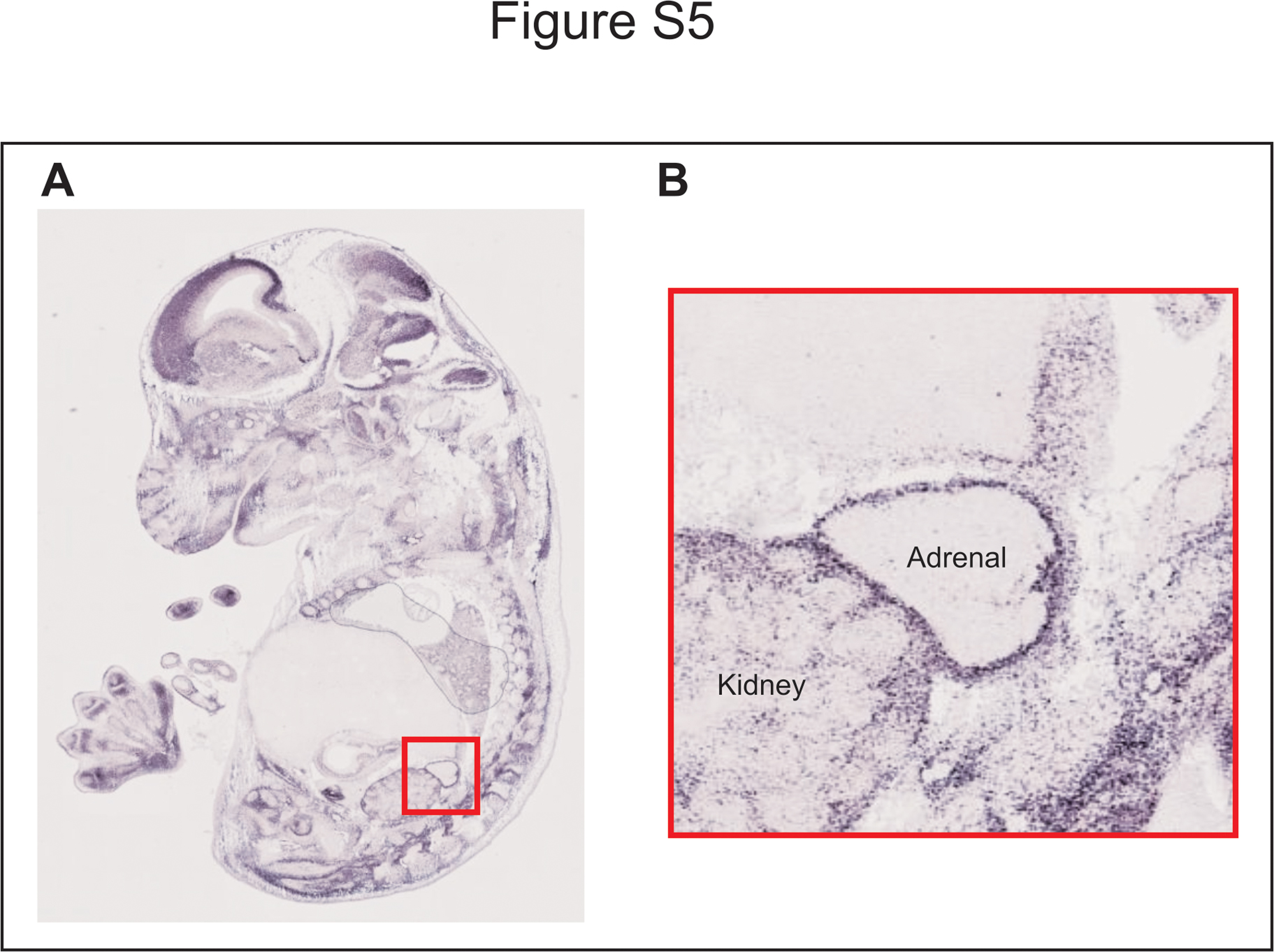

Supplement: Supplementary Figure S5 [file oncsis201520x5.tif]

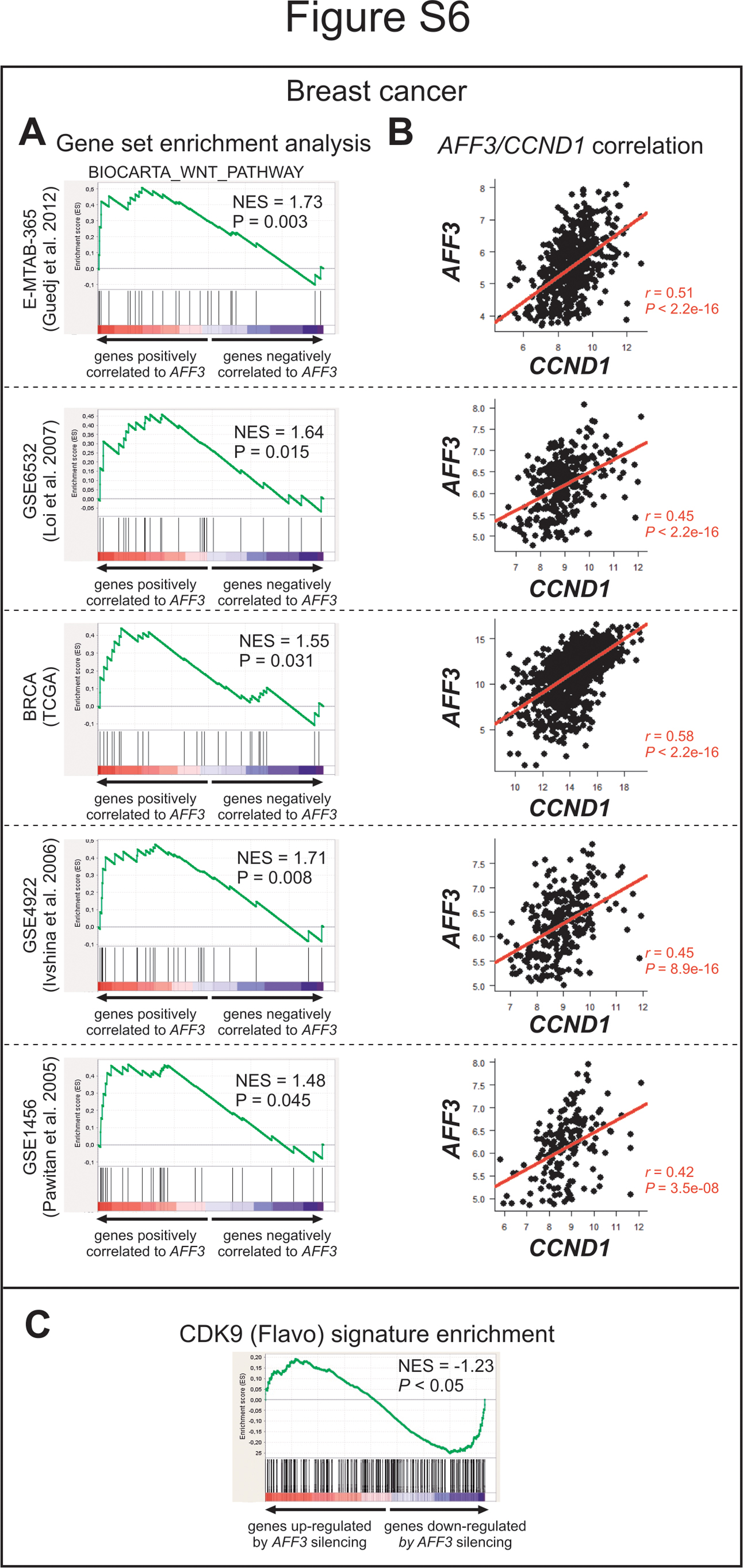

Supplement: Supplementary Figure S6 [file oncsis201520x6.tif]
