# Supplementary material for: Combined transcriptome studies identify AFF3 as a mediator of the oncogenic effects of β-catenin in adrenocortical carcinoma
Source: Oncogenesis. 2015 Jul 27;4(7):e161–. doi: 10.1038/oncsis.2015.20 (PMC4521181; doi:10.1038/oncsis.2015.20)
Supplement: Supplementary Information [file oncsis201520x7.doc]

**Figure S1 Identification of Wnt/β-catenin targets in adrenocortical cancer**

A- Two independent ACC microarray datasets (cohort 1 ^8^ and cohort 2 ^10^) were analyzed for genes that expression is correlated with *AXIN2* expression (Supplementary Table S1). The genes correlated with *AXIN2* are represented in red (adjust *P*-value < 0.05 and Pearson's r > 0.6) or blue (adjust *P*-value < 0.05 and Pearson's r < -0.6). B- The heatmap from microarray data represents expression of significant genes in the 3 H295R- shβ clones. Each line represents a gene and for each clone, 2 columns represent expression without (-dox) and with doxycyclin (+dox) treatment.

**Figure S2 Genes Expression in established tumors with *CTNNB1* inactivation from a subcutaneous xenograft model and siRNA validation**

A- Western-blots representing the levels of β-catenin proteins in Ctr and shβ clones 2, 5 and 10 days after addition of doxycyclin (dox, 0.2 mg/ml) to the culture medium and in parental H295R cells after transient transfection with siCtr or siβcat. Histograms, represent levels of the mRNAs of *CTNNB1* and *AXIN2* in the same conditions. B- Boxplots represent *LEF1*, *AFF3*, *FAM19A4*, *ISM1*, *ACPL2*, *AXIN2* and *NKD1* mRNA accumulation in xenograft for both Ctr (-dox n = 6; +dox, n = 5) and shβ (-dox, n = 5; +dox, n = 5) clones on established tumors and after 9 days of dox treatment ^12^. C- Efficient silencing of genes by siRNA. H295R cells were transiently transfected with siCtr or siRNAs targeting *CTNNB1*, *LEF1*, *AFF3*, *FAM19A4*, *ISM1*, *ACPL2*, *AXIN2* or *NKD1*.

**Figure S3 AFF3 expression in normal human tissues**

A- Boxplots representing the expression level of *AFF3* in 65 normal human tissues which derived from published data (GEO accession GSE3526). B- Histograms, represent levels of the mRNA of *AFF3* on a panel of human normal total RNA (Master Panel II, Clontech).

**Figure S4 Acinus immunofluorescence localization in adrenocortical cells**

H295R cells were transfected with Acinus-Flag vector (obtain from Dr Mireille Lambert, Cochin Institute, Paris, France). The Acinus protein was revealed with an anti-Flag antibody and nuclear speckles were detected using an anti-SC35 antibody.

**Figure S5 *Aff3* in situ hybridization of mouse embryo*Aff3* in situ hybridization of mouse embryo day E14.5**

(A) was obtained from the public database Genepaint  ([http://www.genepaint.org](http://www.genepaint.org/); genepaint set ID : ES1269; Entrez Gene ID : 16764; Embryo_S434_5_2B). The red rectangle represents the enlarged area of the adrenal (B).

**Figure S6 Gene set enrichment analysis (GSEA) analysis**

A- Gene set enrichment analysis (GSEA) showed a significant enrichment of genes positively correlated to *AFF3* expression and the Wnt/β-catenin pathway in several expression datasets from breast cancers [ArrayExpress dataset E-MTAB-365; Gene Expression Omnibus (GEO) datasets GSE6532, GSE4922 and GSE1456; and The Cancer Genome Atlas (TCGA) Breast carcinoma dataset (BRCA)]. B- Pearson correlation between log2 values of *AFF3* and *CCND1* expressions. NES, Normalized Enrichment Score. *P*, *P*-value. r, Pearson correlation coefficient. C- Gene set enrichment analysis (GSEA) showed a significant enrichment of genes impacted by the inhibition of CDK9 with Flavopiridol ^40^ in H295R cells with *AFF3* silencing. NES, Normalized Enrichment Score. *P*, *P*-value.
