# Supplementary material for: Combined transcriptome studies identify AFF3 as a mediator of the oncogenic effects of β-catenin in adrenocortical carcinoma
Source: Oncogenesis. 2015 Jul 27;4(7):e161–. doi: 10.1038/oncsis.2015.20 (PMC4521181; doi:10.1038/oncsis.2015.20)
Supplement: Supplementary Tables [file oncsis201520x8.pdf]

	ACC_cohort_1		ACC_cohort_2		H295R-shbcatenin	
Gene	Pearson correlation coefficientr	p values adjusted	Pearson correlation coefficientr	p values adjusted	Fold Change (log2) [-dox/+dox]	p_values_adjusted
AXIN2	1	0	1	0	-1.63576633333333	0.00383531955221498
LEF1	0.813487991696536	2.32758740429754e-08	0.804840143132419	0.00013848121570347	-1.50921633333333	0.00347244136732606
NKD1	0.812386544683742	2.32758740429754e-08	0.647500364771451	0.0162832424572938	-1.04345366666667	0.00383531955221498
APCDD1	0.789366017450001	1.81843155533379e-07	0.669659657304083	0.0093245701466468	-0.360012000000000	0.233270157863328
WNT4	0.780991053238034	3.17889772905033e-07	0.757668635145505	0.00139063377276949	-0.376455666666666	0.160589525668831
TWSG1	0.776808089830052	3.86518383965750e-07	0.558646710145363	0.0544735712126689	0.0894026666666681	0.766776438263996
NOV	0.757865789054692	1.5380103173257e-06	0.743597258516805	0.00145724921561569	0.123056000000000	0.669530887411007
ISM1	0.757200543274512	1.5380103173257e-06	0.757751337996553	0.00139063377276949	-1.1443200	0.00441604774813744
DAB2	0.746985704324074	3.06911761535942e-06	0.585343445420517	0.0393647293636686	0.104197	0.648806832470653
ERN1	0.744941023475748	3.23272307869438e-06	0.669373527233699	0.0093245701466468	-0.243441666666666	0.190569948330175
AFF3	0.742942807850498	3.42230452368949e-06	0.630095710879757	0.0210748733485634	-1.42359766666667	0.00472058765700093
ACPL2	0.740580515833043	3.74929782422558e-06	0.677520354072249	0.00806573230105558	-1.059745	0.00383531955221498
SLC12A2	0.73940337469492	3.7797033089866e-06	0.508989016144648	0.101906025861329	-0.0227763333333327	0.946627610228776
CTNNB1	0.71392627682077	2.12150882393315e-05	0.388892469367715	0.284305090520190	-1.31956433333333	0.00586308454938864
ABR	0.708969778869235	2.74807283186481e-05	0.556373062403746	0.0555982011257914	-1.03942933333333	0.00383531955221498
PRKD3	0.706919909640266	2.9445365049674e-05	0.552320689841045	0.0593736581182735	-0.278459999999999	0.223260757343954
TGFB2	0.699929952697128	4.14823675615428e-05	0.575496341476614	0.0441256193514982	-0.517693666666666	0.267735810842620
UGCG	0.695887605286069	4.85144567129336e-05	0.609746823503929	0.0286528292075898	-0.476449	0.0432054383604454
ZNF711	0.695525249450336	4.85144567129336e-05	0.501346724436162	0.112878524194511	0.00626466666666687	0.986471172041113
SYTL2	0.694221571547403	5.00801390377971e-05	0.535590610530987	0.074032576515667	-1.28957	0.00311122808725912
ETV5	0.688365158668756	6.75174145693065e-05	0.673501860357514	0.00838388668812464	0.096069666666667	0.775273488714107
PGM5	0.687816506320746	6.75174145693065e-05	0.477421052007996	0.141622491285205	0.411397555555556	0.246561548460872
FBXL7	0.685476654901015	7.44221537239687e-05	0.390157390282120	0.282383744168943	-0.292245333333333	0.194593860853488
MAML3	0.683205369442345	7.96567413366721e-05	0.577157400417561	0.0433421970607609	-0.338022333333333	0.121952230275805
SPOCK1	0.68297794094612	7.96567413366721e-05	0.575663200713908	0.0441256193514982	-0.342299333333333	0.546434057395997
FGF20	0.678614226413619	9.89926571525314e-05	0.317671098070123	0.431016140580857	-0.0487976666666662	0.880270138913017
PDZRN3	0.675859241938789	0.00011188840708322	0.661277207733466	0.0112040463929264	-0.222138999999999	0.477169587518779
MYO9A	0.674183575051094	0.00011888923002464	0.447643364874849	0.178249202877872	0.0212393333333333	0.954865522683112
CCDC170	0.673548809087807	0.00011915362925898	0.731650058312902	0.00165671448978994	-0.763100999999999	0.0166728696318732
RALBP1	0.669742477423373	0.00013847056751469	0.410723600217933	0.245354538163071	-0.201865666666665	0.351839993215702
VCAN	0.667797516960857	0.00014967140179613	0.248499495608358	0.57746120283043	-2.652893	0.00311122808725912
PHLDA1	0.658820727310825	0.00023486429486969	0.475179274781182	0.144197096534399	-0.359014666666666	0.0917235990272545
TRPM3	0.658475636166423	0.00023486429486969	0.747995203942222	0.00145724921561569	0.225264	0.455326357137964
SV2C	0.65207378531134	0.00030288420353906	0.367946651830324	0.326100609372639	-1.03841966666667	0.00311122808725912
SLC44A5	0.647432996204118	0.00034226039979804	0.355846367420999	0.346552539524285	-0.361440333333333	0.320225451789804
SERINC5	0.647416439700406	0.00034226039979804	0.58326642204418 9	0.0407754597786276	-1.035958	0.00383531955221498
PLCL2	0.647407327015034	0.00034226039979804	0.551609741710482	0.059751793998918	0.424863333333333	0.0755898373594719
MME	0.645546706974469	0.00036804132089795	0.169224786786785	0.744909303161203	-0.339116666666665	0.234508116088724
SESN3	0.64436134536704	0.00037983502996154	0.354016278521171	0.351611207120972	0.538249333333335	0.0200880262280604
ITGA9	0.644058876363969	0.00037983502996154	0.551392577673661	0.059751793998918	-1.15585566666667	0.0105638761565426
FAM19A4	0.642548667225481	0.00040139690062994	0.730714431983082	0.00165671448978994	-1.76340866666667	0.00347244136732606
BMP4	0.640903279501547	0.00042709671443177	0.563893454664984	0.0501997179061873	-1.12272933333333	0.0305931016495141
LPPR1	0.639307357107651	0.00044774363426457	0.442126301934229	0.185713473308125	0.0363103333333338	0.887363245747406
C11orf45	0.635596721416072	0.00049470409723740	-0.0735070734253988	0.905838142287595	-0.098738333333333	0.68171195530621
ZNF652	0.634933740226662	0.00050186654282899	0.314271905394406	0.437675147805387	0.259368666666669	0.257540944081527
NTM	0.634318233089169	0.00050505186959894	-0.00685195559699837	0.99129507664977	-0.466206333333333	0.0584908310916042
C3orf70	0.634079605605739	0.00050505186959894	0.450012476143852	0.177025296973857	0.127674333333334	0.583061615740711
PDE5A	0.630649445081164	0.00058689317679817	0.498428910960487	0.114105676690072	0.497125	0.0629160804357479
PDE2A	0.626799525971268	0.00068308515621059	0.679128930697678	0.00781198449038317	-1.131113	0.194593860853488
PRKCQ	0.625969459974888	0.00069910107930011	0.00280057554949891	0.995579670844626	-0.0646169999999998	0.809409373518692
CTHRC1	0.624895094077468	0.00072397328086428	0.35419924298218	0.351010711749563	-0.168478	0.459936388684371
ASTN1	0.624026117718759	0.00074251373720346	0.666822608979916	0.00952091781516912	-0.430490333333333	0.182794399904883
RNF217	0.623367737263173	0.00075407431947426	0.510966591274686	0.0988814622573226	0.3414	0.179451818843724
NPNT	0.621924197860347	0.00078585787449629	0.326401979799203	0.409206882509236	-0.425920333333332	0.137453276113987
KLHL5	0.621841294576913	0.00078585787449629	0.378866165285138	0.304578046752771	0.111600333333333	0.768318188305035
BTAF1	0.621381188208381	0.00079021002342940	0.255298694495418	0.566022340784541	-0.0493279999999996	0.85013576481729
WDFY3	0.621088916034751	0.00079021002342940	0.578835513500867	0.0427567474426921	0.105999333333334	0.646350580563869
C9orf84	0.620691570737848	0.00079341407328683	0.566542655189767	0.0473734415377791	-1.171353	0.0326806169766676
LRRC49	0.618177156034435	0.00085888656033578	0.264171502245108	0.544525995334757	-0.372215333333333	0.0917903726055603
SMAGP	0.61790730772887	0.00085888656033578	0.50813809651321	0.103157700646924	-0.0891689999999992	0.704370299831112
LAMP5	0.617797040310742	0.00085888656033578	0.524334942790454	0.0861537957700314	-0.206505000000000	0.307774837877941
HLF	0.617784264180858	0.00085888656033578	0.586037043923272	0.0388405345077531	0.123086000000000	0.635267430402483
SIAH2	0.614267226418693	0.00099656116442380	0.65575457171057	0.0128550328155255	-0.648251666666666	0.0184916897836653
DAAM2	0.613817069673446	0.00100376825714799	0.735062409501052	0.00165671448978994	-0.246628666666666	0.40610776759241
FGF12	0.613002525082698	0.00102815127996601	0.513878020487204	0.0962522730025878	-0.0803183333333332	0.772263402899903
LGR5	0.612618870426835	0.00103112592709358	0.716032389278893	0.00294561714618298	-0.558951666666667	0.359771290730514
SP1	0.611971736626223	0.00103669016824409	0.145753366629267	0.787804113309913	-0.00290966666666662	0.993756838831997
KHDRBS3	0.607635852695671	0.00120262298688088	0.461638765075056	0.160569275770171	-0.332040333333333	0.226499104403529
PLCB1	0.607610254834613	0.00120262298688088	0.187272803454585	0.709833349594307	0.229422000000001	0.310277378475854
SEMA6D	0.607590496228629	0.00120262298688088	0.475704118275289	0.143137411589934	0.794209333333334	0.0645400135120987
MFHAS1	0.607135046524198	0.00121279588498349	0.333952461500941	0.395721715641199	0.141706333333333	0.516040564500327
PTPRS	0.606331055980905	0.00124237811585880	0.309458694659278	0.447639110710496	0.192862333333334	0.309421300309741
SLC30A1	0.605335605775774	0.00126279518105789	0.457416511703299	0.166671273245692	-0.177486333333333	0.353471671280201
HTR4	0.605186490674426	0.00126279518105789	0.340410822488068	0.378706407610944	0.0802620000000005	0.724879647151162
RELN	0.602525073296089	0.00138908773800389	0.58659791669828	0.0386126735067483	-0.822631333333333	0.008129182209396
SLIT2	0.600251932625149	0.00151796103142844	0.246594576721811	0.581691803416376	-0.529523666666666	0.0632396912595744
KCNJ5	0.59907204157692	0.00156011658557439	0.681020060195421	0.00775246230110297	-0.502679333333333	0.428953946992621

FAM171A1	0.598880422101362	0.00156011658557439	0.616906126889087	0.0257474416965149	0.102222000000001	0.646934482100399
TMEM108	0.597600579850337	0.00163192253173961	0.320137429286152	0.423328320249032	-0.273094333333333	0.187231535349616
DACH1	0.596400278308574	0.00166625301056567	0.57762975502911	0.0432751280177754	-0.8728	0.0185304620090284
WIF1	0.595309235695065	0.00172880080968670	0.256065729409490	0.564792882906668	-0.910069	0.131369372059974
ANO4	0.594887722465336	0.00174279610243234	0.223838465952176	0.633743682471632	-0.923490666666666	0.00841828254294244
NAGS	0.593322224200526	0.00184520270918418	0.407167735811183	0.252790779847156	-0.105932333333334	0.639837290428782
TNFSF4	0.592598252628478	0.00188442982136235	0.432800571832031	0.202313952940475	-0.181332666666666	0.406211996897819
ADAM22	0.590169085967865	0.00206879539008999	0.590723488654411	0.0365855475433461	-0.698472666666666	0.0191141200358598
GABBR2	0.588706330046321	0.00215850402211448	0.316803632947219	0.431921760942787	0.328768666666668	0.450233254347116
FAM178A	0.586769360161783	0.00229402017911543	0.521409950706199	0.0885204961957005	-0.334035333333333	0.190569948330175
ZNF462	0.58646586871636	0.00229402017911543	0.403756921923647	0.257216918236696	-0.246299666666665	0.254350940121719
ASXL1	0.586360867450054	0.00229402017911543	0.219429650454157	0.641226345931986	-0.093965	0.693228432049376
ZNF148	0.584411803087935	0.00244296534966365	0.5163831566578	0.0923726727099053	0.128628000000001	0.529987361613616
ZNF660	0.584034826550033	0.00245366429754527	0.0734404745158692	0.905838142287595	0.151217666666666	0.509471764056684
ETV4	0.58366039202571	0.00245366429754527	0.204073945545516	0.675344930297902	0.232632333333333	0.313220411537404
SLC39A10	0.580900439645008	0.0026787354061596	0.353117174529267	0.352407344218756	-0.199643333333332	0.315385252030064
MUCL1	0.57975412868856	0.00278357423751385	0.666836005519152	0.00952091781516912	-1.08786033333333	0.00311122808725912
TEC	0.578429298001554	0.00291329017294414	0.498411580870914	0.114105676690072	-0.457102666666666	0.0647596980686847
SIN3A	0.577497236455471	0.00293171366667684	-0.162635681352677	0.757524249707797	-0.0574576666666662	0.819757926662461
TMEM2	0.577493102596086	0.00293171366667684	0.490086953997214	0.124999442942958	-0.254083999999998	0.388303383752735
HNRNPM	0.57628581037452	0.00302509756865399	0.183063733136999	0.718112136382915	-0.127743333333332	0.540605664435963
EBF4	0.574588897303505	0.0031880040010521	0.452118500439281	0.176189305981262	-0.0518269999999996	0.870489343487753
SYNE1	0.573025411871893	0.00331569021976937	0.623652354112127	0.0233850729680282	-0.183402999999999	0.49971129098408
QRFPR	0.571340231347890	0.00346594942734817	0.207879466030054	0.667695745454683	0.0765451666666667	0.732086971105268
TASP1	0.569237242936335	0.00365691643289855	0.06659726835246	0.915849132404917	-0.265282999999999	0.202884869911086
FSCN1	0.567974739749071	0.00381546411412576	0.312093237004862	0.440558341460703	-0.208614666666666	0.297888842945994
CADM1	0.567725651289327	0.00381791560295233	0.152515832463426	0.775863712880333	-0.546237999999999	0.0637025835299783
ANTXR2	0.567578529959791	0.00381791560295233	0.490703393993075	0.124219065863018	-0.386570333333333	0.0894144790631108
AJAP1	0.567410610435173	0.00381791560295233	0.267930667473703	0.53559545997875	-0.109942666666666	0.617867263424706
KCNK9	0.565754538987397	0.00404476622330774	0.511674624494779	0.098679015044573	-0.131251666666666	0.707538975864338
SLC23A2	0.565155176930482	0.00405678835329295	0.468231964526927	0.151936428121882	-0.287721333333332	0.1780215342276
RAPH1	0.565142010774626	0.00405678835329295	0.263241935906551	0.546219336675390	-0.126587999999999	0.68149943269806
SV2A	0.564715035875866	0.00406863288244464	0.321114813521604	0.421618886526906	-0.07559	0.777568084061469
APP	0.563043116398278	0.00431183797836431	0.235175060779187	0.607340367904098	0.0905266666666675	0.66807888867384
HS3ST5	0.561636249178444	0.00446657476912868	0.30986786921737	0.4470577615309	0.217134333333334	0.390298381662209
VSTM4	0.561047513326598	0.00453411029274013	0.537638840197471	0.072332911210698	-0.0893986666666666	0.688511260713706
AATF	0.560689683331702	0.00453715128836952	0.258930277382602	0.558709815549469	-0.126039333333333	0.600950363504414
CALN1	0.56023065234448	0.00457947027671716	0.593401052081506	0.0353469575243278	-0.677994666666666	0.099537455213777
TSPAN7	0.559560135633337	0.00461787113382232	0.467619446886064	0.151936428121882	0.220306666666667	0.422658139524950
ANKRD20A5P	0.558568963707864	0.00476582480967628	0.55820107008594	0.0548810795331286	0.0743336666666667	0.777406710539424
RGS7	0.557406281525299	0.00492921358906083	0.253290586222931	0.567925260831937	0.627025333333334	0.0185304620090284
RFX3	0.557173346465007	0.00492921358906083	0.119448976266641	0.836013196419542	0.241456333333334	0.307927332709227
MID1	0.556548053598986	0.00492921358906083	0.183864951473853	0.718067440349544	-0.128774999999999	0.648442399981194
ANKRD50	0.556240487217574	0.00493651574389029	-0.0553708571178473	0.933409139126562	0.300284666666668	0.120319574336004
CGNL1	0.555968398894615	0.00493651574389029	0.693839825100462	0.00553622192861942	-0.502992666666667	0.120319574336004
NHS	0.555879606248799	0.00493651574389029	0.498817473562914	0.114105676690072	-0.243789	0.220370487708255
GAB2	0.555504420952211	0.00497505153968768	-0.0422015026858886	0.95189442152251	-0.323305999999999	0.191847341402838
FZD6	0.553618578733864	0.00512224479437559	0.474877419893966	0.144197096534399	0.104106333333336	0.677277330785112
SMURF2	0.552834584037026	0.00519911559426316	0.513622886594887	0.0962522730025878	0.125175000000000	0.566075188962
PIP4K2B	0.5524280078094	0.00523451389052223	0.0804417588817644	0.896952038996626	0.00356433333333421	0.993756838831997
MCOLN3	0.551104544934007	0.00539683861746734	0.545092911501576	0.0643777421914996	0.0959029999999999	0.756314439453732
AGTR1	0.550933495963657	0.00540225542000636	0.436564829672678	0.195288910691951	1.06945766666667	0.0979662489851624
EGR3	0.550663830046904	0.0054276760272809	-0.041749023310917	0.951989245458556	0.00764533333333386	0.983098154995337
MLLT1	0.549983514345184	0.00552994205398107	0.524942770277298	0.0854578873278813	0.0509560000000009	0.858412246021085
GLI3	0.549875966074618	0.00552994205398107	0.365545362208597	0.329800721443396	0.0441543333333342	0.889547401003742
VSNL1	0.549429247490235	0.00559279539890474	0.741875416711122	0.00145724921561569	-0.418352333333333	0.294683389709549
UGGT1	0.549274897360747	0.00559554422099808	0.404503342305708	0.255449342443462	0.0459739999999999	0.843213673943937
DEPDC5	0.548293043355658	0.00577220287583316	0.463805501880329	0.157277647666176	0.00666150000000009	0.985956426653124
NLGN2	0.547793601561791	0.00584914797216621	0.223861792891774	0.633743682471632	-0.0419523333333321	0.870575403371786
ACTG1	0.547653725577284	0.00584920298818388	0.158264178699604	0.767214373310291	0.0493900000000019	0.84100404224146
HNRNPU	0.547364314533155	0.00586178337165379	0.270899483510175	0.52859316924857	-0.0595166666666665	0.808204808435554
CD44	0.547318021830642	0.00586178337165379	-0.116840393274819	0.841562044027187	-0.703538333333333	0.0748141981193211
RORB	0.547166227210986	0.00586481968313834	0.621353903442522	0.0237528986370316	-0.433597999999998	0.0878554819019113
TNFRSF19	0.546905051036825	0.00587045912418234	0.595091525212975	0.0344667539792754	-0.985321	0.00311122808725912
NAP1L1	0.546292279809065	0.00596563011745496	0.436474828765613	0.195288910691951	-0.0308799999999994	0.90595687094025
NR4A3	0.543840326535866	0.00636603125191048	0.300288632394836	0.465914681155138	-0.0406233333333331	0.873663396186202
PIP5K1B	0.543685436787978	0.00637106132167595	0.201440536343073	0.680054545720343	1.33497066666667	0.0285314814086805
TUBBP5	0.543314564473769	0.00642644859413115	0.581411316103713	0.0410486958316017	0.199026333333334	0.29098458066948
AMOTL1	0.542731382987275	0.0065153278986157	0.506155825385306	0.105880225573204	0.0500756666666677	0.867268897859745
ARPC2	0.542672127543729	0.0065153278986157	0.250781391424866	0.57315146228686	0.0596943333333338	0.789228734235839
FAM114A1	0.542232684563126	0.00655263931652204	0.335016181371293	0.392352903739145	0.301116666666667	0.120903394140597
NRXN2	0.541937726275233	0.00655263931652204	0.586840367937506	0.0386126735067483	-0.224361666666666	0.281792723503127
MED12L	0.540874600692881	0.0066974424969353	-0.00926461537708774	0.98909166696601	-0.117334999999999	0.662666815705326
PLEKHA4	0.540091840441397	0.00682290227992673	0.225845856661822	0.629179277225701	-0.107664333333333	0.617169282107924
EXPH5	0.539853310379048	0.00682290227992673	0.0300334681428414	0.966396286514145	0.0941706666666668	0.780717071872908
CLIP3	0.539220021602377	0.00691739867848149	0.0427793383474335	0.95069188943636	-0.00998666666666659	0.982906624460486
INPP4B	0.538567926520648	0.00701816494278058	0.278579853503148	0.511025440770733	-0.00549966666666475	0.987163226136842
CTNNA2	0.537110507327441	0.00729603095893503	0.430684027210429	0.206533702654214	-1.83836866666667	0.00295083342264811
TBX3	0.536943919447964	0.00730751718884367	0.574956561223397	0.0442317804409017	-0.238913333333333	0.243615883115952
ANKFN1	0.53637699934657	0.00739428436272458	0.0291484818735238	0.967804297028818	-0.477147666666666	0.0749561445182193

ANXA5	0.536368463484062	0.00739428436272458	0.402766262212367	0.259218173878417	0.0460933333333344	0.852346905702153
ABCB4	0.536033801684879	0.00743417165782344	0.606750045821879	0.0292131998244602	0.230634666666667	0.218141405461970
LRP12	0.535975160718731	0.00743417165782344	0.308685908064594	0.449759364777049	-0.302760333333333	0.150604214474289
LDB2	0.535636842160258	0.00749188483024846	0.406832338095775	0.253220889638452	0.320723666666668	0.0928571414757748
DBN1	0.535063072519777	0.00758114301805284	0.0473233100731895	0.943981149721771	0.130580333333334	0.560413476606931
LTBP1	0.534926552333528	0.00758583336063338	0.287002089248081	0.494586265581052	-0.475870333333332	0.0286129614738536
WWTR1	0.534794148717711	0.0075895403811529	0.380987749592724	0.298110142491852	0.207848666666667	0.282765572761291
PRR12	0.53352580243826	0.00783846988059224	0.216847325002900	0.647348808045933	-0.0447986666666666	0.870662337224757
ZMYM2	0.53290814955692	0.00794497207321964	0.0094377069315538	0.98909166696601	0.126221666666668	0.523453039510001
NR1D2	0.532253132803111	0.00808402454398782	0.504359683558587	0.107082087256657	-0.0172433333333332	0.953217548324318
ODAM	0.532181284018692	0.00808402454398782	0.609469268472247	0.0286528292075898	-0.0143843333333328	0.96018064860593
TMEFF2	0.531182340115231	0.00823777155943032	-0.0150012911863051	0.982921609770846	0.150995000000000	0.619996256183009
ZCCHC12	0.529800154623689	0.00847509992992055	0.557524912267985	0.0549063676899021	-0.0793239999999993	0.75048955525919
LUZP1	0.529146402734089	0.0086023783351915	0.15698357540636	0.768401006769299	-0.0139639999999992	0.972130516588353
PEA15	0.529143122174708	0.0086023783351915	0.477259933780627	0.141859002997729	0.154006333333334	0.503217861354116
RPS6KC1	0.528688707630626	0.00862945947786633	0.172550332514253	0.738421727152922	-0.0703253333333323	0.830093119549048
VIM	0.528611005970729	0.00862945947786633	0.237313362098629	0.60323620145965	0.278273333333334	0.175583831213141
WBP1L	0.527062740039066	0.00892931627789106	0.616561983682381	0.0257474416965149	-0.320964	0.0917903726055603
PTMA	0.526916171644874	0.00892931627789106	0.183794977281375	0.718067440349544	-0.0366883333333327	0.890094615232462
GTF3C1	0.525216795052473	0.00934315520819623	0.144104039217696	0.791452594076419	-0.261184666666667	0.216391179549026
MERTK	0.524983097115037	0.00938370873812034	0.650775328980452	0.0147738810455881	-0.926407333333333	0.0351167468854759
C6orf211	0.524280508434172	0.00950734910626468	0.338287990251027	0.384709616676852	-0.610730666666666	0.0469041914734957
RORA	0.522979385989563	0.009867383644288	0.594941241110947	0.0344667539792754	0.23929	0.210845715069665
BRPF3	0.522329466690104	0.0100517821158242	0.442695188606225	0.185211009605428	-0.629201666666665	0.0185304620090284
USP34	0.522181898903253	0.0100661191594718	0.188510738680709	0.707361603786947	-0.077815666666666	0.750961152956367
CHML	0.521296793674958	0.0103360183542293	0.146058523092171	0.787402233709509	-0.335030333333333	0.259184854253064
JUNB	0.520527643968733	0.0105708983929834	0.0602320094935615	0.925649366494154	0.0867416666666663	0.689501283534577
HNRNPUL1	0.519065769833031	0.0110658582538963	0.295804425510648	0.47761182409076	-0.059278666666666	0.808239992878146
EP400	0.51890288067453	0.0110698484592705	0.405907499355161	0.254381601905451	-0.228707999999999	0.359908305595919
NFYA	0.518758677028185	0.0110698484592705	0.202179415157250	0.679836983534512	-0.0862356666666654	0.729368274679147
KCTD1	0.518657564070598	0.0110698484592705	0.423863703101964	0.217939507033383	0.277134333333334	0.278205568286522
R3HDM1	0.518636275780895	0.0110698484592705	0.370860648215343	0.320399684386315	-0.226813333333332	0.390752942547553
CASZ1	0.518492737544849	0.0110754498090562	0.265594406289000	0.540074581830336	-0.187382333333333	0.44900567252947
C12orf68	0.51841381384207	0.0110754498090562	0.430182934924881	0.20761765766777	-0.374028333333333	0.07521501709354
HK1	0.517512959850205	0.0112975221569011	0.168532572999481	0.746758286599055	-0.0914749999999988	0.660591067381541
TAB2	0.516734051695778	0.0114983018301941	0.157789849501718	0.767678318854251	-0.00111133333333161	0.997144862160692
RAB38	0.516697937840455	0.0114983018301941	0.529286812714157	0.0799297163112453	-1.01769166666667	0.00383531955221498
ARID5B	0.515782431843614	0.0117676487503521	-0.237951884265956	0.602142642649819	-0.229183999999998	0.246462634148331
ZNF503	0.515403092050929	0.0117785452504603	0.645011709936256	0.0167517957225989	-0.238142666666667	0.220405584107617
FAM84A	0.515375304053294	0.0117785452504603	0.434223014396463	0.199899755114597	-0.184109333333332	0.52333876394493
DESI2	0.515287998477103	0.0117785452504603	0.130993214571830	0.812271254601992	0.102718000000000	0.630332384171743
NOTUM	0.515285346015553	0.0117785452504603	0.592352186500661	0.0355984111824064	-1.31216966666667	0.00917425090054953
TSC22D1	0.51514172728823	0.0117824401770728	0.357521168585383	0.34460968444079	-0.189904999999998	0.338519927099693
CAMK1	0.514974353415681	0.0118090718219063	0.499221843037268	0.113777333040600	-0.072931666666666	0.791934055525056
ATP8B1	0.514579865728174	0.0118863804520393	-0.0546999372318619	0.934435680487605	0.587886333333333	0.0227211206883655
KRAS	0.514410273215393	0.0118863804520393	0.435586725646225	0.196965277981193	0.0240346666666677	0.930620998877894
FAM169A	0.514347382096628	0.0118863804520393	0.684527380845222	0.00696399491453285	-0.433456	0.092995664718085
DNM3OS	0.514284747133911	0.0118863804520393	-0.179023660974311	0.725806648980606	-0.100791499999999	0.621410665168104
COL18A1	0.514112118206313	0.0119159126481347	0.604961539358434	0.0302117295841319	-0.0919653333333326	0.677990095007544
LMO7	0.512770289890128	0.0123765432982116	0.416876965259788	0.234235464483992	-0.0522133333333335	0.890511682678135
MOXD1	0.512515058397328	0.0124411994433651	-0.103660057942062	0.861872724387044	-0.389704666666665	0.289347186230224
ST8SIA5	0.512125620247681	0.0125190302693418	0.430154406629444	0.20761765766777	-0.323528999999999	0.338754799084584
ESR1	0.511695728792301	0.0126197304731773	0.479823189007099	0.138290570952161	-0.169100166666666	0.410984651167943
ELAVL1	0.511334734260341	0.0127122380374643	0.232872846293334	0.611711680668619	-0.108176666666665	0.637602236970228
KCNK2	0.510659821216584	0.0127942722545686	0.400907558612632	0.262138265619497	0.213706000000001	0.355387672697857
GDF10	0.510442352309654	0.0128251991139824	0.174823087185452	0.733637477114155	-0.333901000000000	0.289347186230224
MMP2	0.510040382057213	0.0129551621538331	-0.110974309471435	0.849357727620213	-1.017295	0.0233091007249086
SMARCA4	0.509850574029952	0.0129891632030135	0.457371144977072	0.166671273245692	-0.0638786666666666	0.790420447081709
REV1	0.506985915296017	0.0138833193384497	0.540307728548483	0.0699440614682481	-0.109116999999998	0.601018880186027
CAMSAP2	0.506780166647292	0.0139346158082935	0.194994485982175	0.695318250718419	0.259911666666667	0.255532956343297
KCNC2	0.504576740897554	0.0145060689390390	0.226463866534008	0.62700289306617	0.144856000000001	0.576277303044571
FZD7	0.504554891436199	0.0145060689390390	0.455572623039854	0.168217575057610	-0.550011999999999	0.0414548221592696
CCND1	0.504402978297618	0.0145263884110030	0.275949012403049	0.517549833373363	-0.184505666666666	0.399848490147388
FKBP10	0.504291069062688	0.0145263884110030	0.200056735795570	0.682061051920062	-0.0340729999999994	0.891803584952102
PTBP3	0.504205636934008	0.0145263884110030	0.0690583613765723	0.911928203725685	-0.117615666666665	0.68414816517367
CHRNA3	0.503778383695573	0.0145379065809406	0.247115123306598	0.580507768773699	-0.0857089999999997	0.760951989611575
GFRA2	0.503760440473317	0.0145379065809406	0.369739674225743	0.322635646591244	-0.189420666666666	0.528409919089401
TNFSF18	0.502013538339997	0.0151186295100801	-0.139722012576487	0.797957231657366	0.125890000000000	0.548643967646122
SAP130	0.501489664758366	0.0153309749700524	0.263392838477552	0.545874639137454	0.0516806666666661	0.824674571093737
CSRP2	0.501279507073155	0.0153742583788850	0.427947367556431	0.211586765714307	-0.0983163333333317	0.692043732062832
TOB1	0.500824718978444	0.0154883612406437	0.632433811101854	0.0208232988311238	-0.0601399999999983	0.794326423532765
TCF7L2	0.500742374204837	0.0154883612406437	0.416627407415858	0.234235464483992	0.387560333333333	0.086617607905191
GPR75	0.5005422992944	0.0154883612406437	0.276500579518119	0.517266722508946	-0.0390656666666663	0.909918896189467
PTMS	0.500505529423954	0.0154883612406437	0.309501366807495	0.447629642677855	0.0389443333333344	0.88683920165247
GNA13	0.500427883050297	0.0154883612406437	0.0893928339226195	0.883460564717377	0.113760000000001	0.587396808178803
CEP170	0.500397092242988	0.0154883612406437	0.0300136477941477	0.96642837651223	0.428522	0.123133456370455
SLC35F1	0.500103325246654	0.0155915080832135	0.243632119006191	0.590086964098531	-0.116828666666666	0.676662919155189
ATP1B2	0.498925208160529	0.0159264978424877	0.383151653205380	0.294230588180528	-0.102550000000000	0.662751649046716
DZIP1	0.498783591980251	0.0159264978424877	0.405305901177232	0.25480455106032	-0.00987199999999868	0.974451420920923
ZNF521	0.498589791539688	0.0159264978424877	0.236489600217274	0.604858516667951	0.223318666666667	0.378344763124493

DUSP6	0.498533261368744	0.0159264978424877	0.119931348746256	0.835280975936648	-0.934075999999999	0.0132656263961273
MCAM	0.498511746442434	0.0159264978424877	0.0104628408488988	0.987849393233007	-0.103921333333333	0.687270356148619
NFXL1	0.497886923540304	0.0161174161004334	0.18811773898461	0.707635058271509	-0.212463999999999	0.352237134604623
NOSTRIN	0.496642839733387	0.0165043729006466	0.447460228601565	0.178249202877872	0.0147929999999995	0.96622567326855
SLC33A1	0.496031678509009	0.0167811353449255	0.2142800729587	0.652991165086813	-0.0317006666666663	0.915761046438965
JAM2	0.495491385960232	0.0169202177523791	0.459259861051022	0.164444235253496	-0.331264333333333	0.137947869718874
LRP6	0.495444848954593	0.0169202177523791	0.522554535663489	0.0873718619016661	0.081383000000001	0.74164843446719
TSC22D2	0.495325176268672	0.0169410184595016	0.596306358714491	0.0342864566106277	0.120147666666667	0.557104392196544
LMNB1	0.494451283689451	0.0172796441672268	0.122385844608628	0.829398647947819	0.0933936666666681	0.696217349388802
CHRNA5	0.4940576878917	0.0174494967372755	0.472684023486502	0.145443641689931	0.0753800000000007	0.780717071872908
FBN1	0.49379953837179	0.0175032940488472	0.211822354966981	0.658506848169835	1.12137933333333	0.00347244136732606
CHN1	0.493418793867768	0.0175474109778976	0.485227754177743	0.132945047074832	-0.108759999999999	0.621406232563173
LRRFIP2	0.493402027923689	0.0175474109778976	0.233405296967673	0.610610966181986	0.266193333333333	0.191847341402838
TRAK2	0.493275924169681	0.0175664190851276	0.313013806152141	0.438608852266634	0.0250370000000013	0.942092082409632
TCF7	0.49313480285545	0.0175664190851276	0.570041088584627	0.0451779801563352	-0.241616666666667	0.270098077393495
CHERP	0.492530477124748	0.0178538361141547	0.348642500278077	0.361261311685225	-0.146234666666665	0.48854839402226
DOC2B	0.492420473260191	0.0178714679441944	0.320097720592926	0.423328320249032	-0.185236666666666	0.512908873161269
ZNF236	0.492140659714643	0.0179274965264759	0.312336618150773	0.440068551920157	0.136147	0.50393099142638
CNP	0.491868478275527	0.0180019891197313	0.114673154009782	0.844162087612813	-0.0600783333333324	0.795703940655181
CDH2	0.491418101016444	0.0181274997752744	0.739647649199105	0.00147294292509128	0.0466103333333337	0.843676733449892
SHROOM4	0.491165604810332	0.0181828957519071	0.411202327653689	0.243981291680416	-0.077281	0.764848773122078
RIF1	0.490044070110481	0.0187339067158953	0.259119841147921	0.558209290204909	0.0169046666666672	0.956567100065073
MARCH8	0.489904048162852	0.0187705386477488	0.191750946360424	0.701544026774214	0.206434	0.305655404008506
KCNK3	0.489469708045006	0.0189338031451471	0.630265201121216	0.0210748733485634	-0.494624	0.0837916958855762
PARVB	0.48922542253639	0.0190318372802025	0.446032682349411	0.180228349426063	-0.168692333333332	0.375553176012692
SNRNP200	0.488661697272961	0.0192120033990857	0.054714966360673	0.934435135880088	-0.206353333333332	0.333622638790673
RGS12	0.488615035874294	0.0192120033990857	0.0457452174715371	0.94547820490174	-0.205757666666666	0.275739244875649
ESRRG	0.488190138016071	0.0194187070437633	0.285877457567225	0.496429042723156	0.0835590000000002	0.708294071875696
NXN	0.487586273564061	0.0196976781372742	0.118297007636943	0.838117566591658	0.0600643333333345	0.832650560777779
B3GNT9	0.487545223565686	0.0196976781372742	0.401633683664334	0.261512734265668	0.0640506666666681	0.814001431217602
VGLL4	0.487453735580666	0.0196976781372742	0.121771049948431	0.83097116342043	-0.0365256666666663	0.907374738017166
MYLIP	0.486738044537815	0.0199970156460819	0.38223251654307	0.295576295884376	0.183623000000001	0.348000484523924
AKAP5	0.486664081666754	0.0199970156460819	-0.0578544298895625	0.929715247340158	0.242779	0.411784621423647
PRRX1	0.486356871395718	0.0201181263770869	0.304250207187336	0.459461419734961	0.0118200000000007	0.974895360062947
TM9SF3	0.485222190624372	0.0206370837998719	0.223600514050818	0.634154463155482	0.0444133333333344	0.856502682928004
UACA	0.48513532478215	0.0206452347743593	0.304170971588704	0.459627758172839	0.249206333333334	0.207611745628393
CEP68	0.484819717362295	0.020750832283089	0.188837268431805	0.707236456264125	0.169207666666667	0.384477482312904
TATDN2	0.484152736329302	0.0210334323205570	0.290960466750689	0.486880079690803	-0.154177666666665	0.451746572873908
ZDHHC20	0.483533506021454	0.0213361431693904	-0.148540066677836	0.783532632807022	0.1231	0.544685122345675
VEZF1	0.48330069912102	0.0213922753581846	0.474557607044695	0.144197096534399	0.125623666666667	0.525143015861964
sept-08	0.483219041655151	0.021394989252267	0.229720503659048	0.619603028959455	0.161048333333335	0.439061344563948
KIF16B	0.483151333996886	0.021394989252267	0.534435958139406	0.0751352200098442	0.0166520000000016	0.965544587103044
NPPC	0.482270769623247	0.0219115737364704	0.196940050438625	0.690061983084583	-0.173651666666665	0.588052519326463
C11orf82	0.481759784183811	0.0221338455051386	-0.120374217334010	0.834588275061672	-0.143660333333333	0.632554412921941
PEAK1	0.481733431514258	0.0221338455051386	-0.0486336853916384	0.94236319435931	0.0340443333333343	0.940972575250573
NFIX	0.481357153644757	0.0221544431194789	0.573914280757299	0.0442317804409017	0.0298793333333343	0.910190419163007
OVOL3	0.481353178009605	0.0221544431194789	0.194151708358896	0.696755619051339	-0.184090333333332	0.343729765439488
IQCA1	0.480949999226107	0.0223488559288475	0.531499206838621	0.0782036966829635	-0.0129806666666663	0.96681311917387
SH3BP4	0.480909245145283	0.0223488559288475	0.639307858693968	0.0185595110823276	-0.182962333333332	0.399012880214421
PPP4R1	0.480660521305331	0.0224679218394927	0.123734920858559	0.826475556843795	0.213797666666669	0.279049509382533
ANKRD12	0.480509731675927	0.0225215592611382	0.0515263591260039	0.938728344711776	0.135943333333334	0.500586905345825
GNL1	0.480332299898248	0.0225933624390969	0.127951638815996	0.817799920299584	-0.0614025555555555	0.84030869240936
LSM14A	0.479435616736051	0.0229763415690932	0.264373281646428	0.544042395849675	0.00816433333333397	0.98025379113353
HNRNPD	0.47921977844298	0.0230154680979866	0.0215279329358536	0.97536722461972	0.0274166666666678	0.923293854892615
PCP4	0.478249082230023	0.0233474443325047	0.40370474868208	0.257216918236696	-0.840506999999999	0.00757447647002903
EXOC7	0.477746063885641	0.0236014328486517	0.501065428477658	0.112878524194511	-0.047772333333333	0.836692897504117
SNRPA	0.477240683548269	0.0238699625991943	0.154681954008118	0.771310656070265	-0.0847349999999983	0.721454370213253
MYB	0.477170704825723	0.0238699625991943	0.395828362050402	0.272027908323936	-0.423528999999999	0.148288164588800
KDM5B	0.477044677067901	0.0238699625991943	0.128243685874177	0.816855571576772	0.0899463333333338	0.689267979966846
HAO2	0.476996460858533	0.0238699625991943	0.225576025815708	0.629710977741725	-0.0101973333333331	0.983159241812861
RASL10B	0.476883188812319	0.0238699625991943	0.501133712316874	0.112878524194511	-0.0621259999999984	0.820041994125866
C1QTNF1	0.476822315614812	0.0238699625991943	0.390020323649854	0.28255597438291	-0.173409666666666	0.426233942525061
RNF43	0.476510891035724	0.0239405354373014	0.745633021245756	0.00145724921561569	-1.22054966666667	0.0036651923426154
CHL1	0.476211253333634	0.0241049825490732	0.586472967968047	0.0386126735067483	-1.448867	0.127449171495251
CHST14	0.475970256284058	0.0242285267902541	0.297881133173133	0.472550555499985	-0.0877006666666664	0.752992371482229
LAMC3	0.47586718893779	0.0242538638549144	0.594757802095646	0.0344667539792754	-0.00240766666666612	0.994012486997982
SUPT6H	0.475755289766454	0.0242856215678313	0.224290313864158	0.632829553438263	-0.076876	0.78960166957155
MBOAT2	0.475396913152249	0.0244946599028276	-0.0474875672467887	0.94394191186189	0.0401576666666668	0.903352632801889
PHF12	0.474657870839095	0.0248274707247140	-0.0554042749672379	0.933409139126562	-0.194335999999997	0.328724837942497
NDNF	0.474601482786662	0.0248274707247140	0.414031749451971	0.238232786801887	-1.13688366666667	0.00841828254294244
COX20	0.474468712584072	0.0248274707247140	0.472626954505465	0.145443641689931	0.283300333333334	0.223260757343954
MYH7B	0.474295084956468	0.0248828661786959	0.416460908986591	0.234235464483992	-1.54786266666667	0.0230379245071538
H1FX	0.473921779628727	0.0250118017158169	0.543750080062469	0.0658108061761627	0.105579000000000	0.60378773986659
TMEM132E	0.473837766371611	0.0250118017158169	0.197374667740769	0.689176078372462	-0.451557333333333	0.0351167468854759
CLDN1	0.473264636058111	0.0253047264600363	0.482299413284314	0.135560525489292	-0.882317666666667	0.008129182209396
TET2	0.472923742314992	0.0254781784186107	0.34994197139628	0.358969704130952	0.194743500000001	0.35135795138426
IL4	0.472657934271173	0.0255338104084811	0.155864620978302	0.76972820876143	0.0473833333333332	0.862509125961694
TMEM74B	0.47241543298473	0.0256621268051285	0.358494931966932	0.343101042014771	0.00568100000000067	0.991175862027014
CCDC41	0.471679776827965	0.0261187639366898	0.261234937861149	0.552474056735769	-0.271988333333333	0.431227317416467
EOGT	0.471349986716877	0.0262635168158843	0.302712259294372	0.462105017205825	0.304785666666668	0.135004030972507

KLHL8	0.471113673857297	0.0263028456061064	-0.0406265996011426	0.952426531595115	-0.509194	0.0471454030933192
TAS2R19	0.470912137587869	0.0263582155436303	0.371082031000873	0.320357523974285	0.00502366666666692	0.987796346951675
CORIN	0.47021165983083	0.0267349718952203	0.0121154494401898	0.985742962398217	0.0299836666666675	0.928785692303482
EDNRA	0.470165698211534	0.0267349718952203	0.209766517234138	0.66376283098013	-0.270533666666667	0.15725962112112
CALML4	0.470023771674212	0.0267953257740962	0.482468188612633	0.135560525489292	-0.129941333333333	0.552298643114782
GJC1	0.469639427750992	0.0269324816158244	0.0124458205230213	0.985220352735461	0.503503000000000	0.0271417780747615
VPS13A	0.46956726359995	0.0269324816158244	0.359290846195751	0.342292001700374	0.133654000000001	0.516040564500327
NFIC	0.469225076650032	0.0271198106754241	0.552331681187464	0.0593736581182735	-0.133893666666666	0.525143015861964
ZFHX4	0.468483056412411	0.0275552374470877	0.495632363049665	0.116827176967180	0.150055666666668	0.523453039510001
FAM95B1	0.4674951903949	0.0281669671672249	0.128925811756885	0.815386814587013	-0.0931959999999994	0.769893749154778
CDK6	0.466627712213155	0.0284930815138688	0.500543858665049	0.113191327408217	-0.672150666666666	0.0368932712496524
L3MBTL3	0.466454137934617	0.0285331056870949	0.504234756731538	0.107082087256657	0.0320853333333339	0.914215855540678
ARHGAP12	0.466346907452816	0.0285699740190408	0.464411157966976	0.156705484651498	-0.0356669999999995	0.887363245747406
XRCC5	0.466140861131983	0.0286614547832846	0.110795307192841	0.849357727620213	-0.0863466666666656	0.680455232178275
DDX10	0.465687356176602	0.0287065121889664	-0.524109819846333	0.0861537957700314	-0.0786859999999993	0.73899161599654
POLR2A	0.465553773602083	0.0287065121889664	0.310506965172026	0.445142823947765	-0.101509333333332	0.689901977245828
GATAD2B	0.465465237903845	0.0287065121889664	0.163620121895656	0.755036658968701	0.121990666666668	0.541939010010167
TXK	0.465332074244992	0.0287065121889664	0.307920244778976	0.451652892672575	-0.356160666666667	0.103455104221207
NOTCH2	0.465297329591325	0.0287065121889664	0.116924062233558	0.841512197646315	-0.0732763333333323	0.791330178898253
CAPN5	0.464970860295449	0.0288437206743345	0.369412909886795	0.323285574998587	-0.151788999999999	0.568393891086758
DNAJC10	0.464750306267454	0.0289756226394537	-0.106533138051810	0.858072018696671	0.0619153333333344	0.780323861357089
POLR1A	0.464412647159295	0.0291052491194405	-0.0904549836058102	0.881797721301484	-0.444285	0.0667771856938434
TCF3	0.464284389073862	0.0291118665431137	0.381833755734262	0.296920946139734	-0.0260586666666656	0.922463263763
HNRNPR	0.464152616672280	0.0291386438784342	0.0843145429724454	0.892396784625427	-0.0952599999999986	0.661383805087653
MIB1	0.464080724796148	0.0291386438784342	0.0499153324495867	0.941458038227918	0.177190333333335	0.368449159208846
RERE	0.464072349141741	0.0291386438784342	0.283020925229524	0.502129505146473	-0.219746666666665	0.243404019091646
PTBP2	0.463799316015458	0.0293006477381062	0.123447034743847	0.827264208621148	0.295269000000002	0.190246103576351
YME1L1	0.463733089688765	0.0293006477381062	0.067322708439677	0.914582007514328	-0.215208000000001	0.268942614418113
TRIM32	0.463466442175109	0.0293948700221888	0.206276341093694	0.671467763213228	-0.258329666666665	0.16995621050072
PHACTR2	0.463309369803266	0.0294765417923311	0.60029571250694	0.0331255554751519	-0.363550999999998	0.130046102419308
RC3H2	0.46314054071942	0.0295683225653156	-0.0863433637070275	0.88794718020464	-0.0243933333333330	0.926246672706072
ENTPD7	0.462859639827978	0.0297049512183461	0.0962833549770764	0.871674033799072	0.227737666666667	0.279049509382533
EGR1	0.461992198206563	0.0303417840341953	-0.0471740497966395	0.944081286115546	0.363682000000001	0.0691995526792515
ZFYVE9	0.461542309376428	0.0306276477254737	-0.0333732181862706	0.960784250392482	-0.00941700000000005	0.988133186272453
NMT2	0.461053714659696	0.0308985290528507	0.402605454261199	0.259322048328011	0.266864333333334	0.217082119591531
SPTLC2	0.460674401587242	0.0310761782145502	-0.00665392119470584	0.991309497300929	-0.153000999999999	0.538968176654309
ANKRD36B	0.460518128179187	0.0311618653776382	0.0213285312891183	0.97554687597484	0.167426333333335	0.545628708794389
ZC3HAV1L	0.459815740719813	0.0315241535454221	0.240962492742261	0.596648444172109	-0.0114823333333326	0.97184501043952
ZBTB7C	0.459690124158987	0.0315568926495927	0.466267957661259	0.153152263339311	-0.309101000000000	0.255595078844894
TLR6	0.459497269600389	0.0315580445363886	-0.0814155114093028	0.89615326639288	-0.0140589999999994	0.965661221820942
FBXW7	0.459475939913186	0.0315580445363886	-0.0774998035360714	0.90079793355475	-0.197932666666666	0.576277303044571
CEP112	0.459430472152517	0.0315580445363886	0.0360954323867835	0.958023954102604	0.379601333333334	0.135033058827820
EPM2AIP1	0.458410282959874	0.0322220638146523	0.309077994051961	0.449020848361897	-0.0280496666666656	0.919420614501031
DPP10	0.458193314465051	0.0323568430801933	0.355732695612183	0.346896495980390	-0.803284	0.00501765297023503
SLC7A8	0.458014597944838	0.0323837202885224	0.58696635828756	0.0386126735067483	-1.53140433333333	0.00311122808725912
RAB30	0.457992224084926	0.0323837202885224	0.500540755800133	0.113191327408217	0.267241	0.313220411537404
PCOLCE	0.457755401910378	0.0325467531682154	0.0879013056223947	0.885848000380851	0.0528643333333344	0.843451607586348
SRSF1	0.456530323635653	0.0336233960004106	0.266366755113176	0.538833471023229	-0.0303533333333311	0.906093399285191
DCBLD2	0.456273756747350	0.0337982771581418	0.140355257422200	0.797441782082887	0.0152083333333345	0.961509682688243
STAG1	0.456229680928235	0.0337982771581418	-0.114841910290530	0.844162087612813	0.084576333333334	0.724915779411004
TBL1XR1	0.456102870333773	0.0338105567670545	0.194850785054458	0.695517703853906	-0.136542333333331	0.502353732434934
TLE3	0.456021631987068	0.033833557875949	0.163261191758611	0.755849656740422	-0.0333756666666663	0.895093529187836
STK24	0.455956345157010	0.0338416498915081	-0.0527072265870996	0.937357975161644	0.0289973333333336	0.913846966562145
CLDN2	0.455816427269594	0.0339200016709571	0.241642235932656	0.595201219333254	0.025318666666667	0.922697964464038
MED1	0.455624419301505	0.0340472637290141	-0.135565624920395	0.80669742201648	-0.174858999999999	0.419500365886586
GRPR	0.455416964702346	0.0341371118587178	0.180080910373325	0.723716289903531	-1.26757233333333	0.0093299183713845
NF1	0.454944181416034	0.0344793314671696	0.218505520575526	0.642691336921378	-0.0274396666666662	0.925109901158611
FAM5B	0.454063830767628	0.0352189339042013	0.400531203301633	0.262685489461853	-1.84412633333333	0.0711528556656884
BRWD3	0.453916425642832	0.035253021947738	-0.335318429214681	0.391197106326829	0.115258666666667	0.575424509084104
SMNDC1	0.453598023363634	0.0354541891108543	0.137788200862426	0.801860085345265	0.0777723333333335	0.727255353478337
PARG	0.453365582595303	0.0356270317632932	0.351308188439598	0.355959518877195	-0.323170666666666	0.134149817255630
OSBPL10	0.453258082242752	0.0356331473785568	0.398508537166324	0.267349827447097	0.544556000000001	0.0583806133794157
AMPD2	0.452791168542616	0.0358456747153192	0.146101854449108	0.787402233709509	-0.322052333333333	0.134309227552143
ZRANB1	0.45256393759219	0.0359233938372133	0.448733735226274	0.177860736739947	-0.00249899999999991	0.994118714439223
ANKRD20A12P	0.452287142023896	0.0360878830911139	-0.179590983533309	0.72471205425263	0.183636333333333	0.426518032099216
KANK2	0.452181045357162	0.0361383946668023	0.155430762848986	0.770224269831267	0.0953570000000012	0.66632805122137
NFATC2	0.451844562352516	0.0363946914244752	0.00054476764765980	0.999260526969206	-0.725059999999999	0.00629285391915396
NT5DC2	0.451569154625301	0.0365288729158546	0.267122817924007	0.536890241773352	-0.0946789999999995	0.714244999058301
CPT1A	0.451419541499225	0.0366238112677826	-0.00164525936428704	0.997963920501446	0.152339000000001	0.466793078197373
DDX42	0.451061466229097	0.0368880448108579	0.0345282777986892	0.959404955370931	-0.0994299999999999	0.629376045476803
FGD6	0.45076065971184	0.0369121738156248	0.193222382004802	0.698676320436036	-0.494254999999999	0.0351167468854759
LOC375295	0.450092955034673	0.0374142995250402	0.535516839087772	0.074032576515667	-0.0950526666666666	0.714244999058301
PLD5	0.449931346335748	0.0374769151413555	0.230891951396640	0.616633154263053	0.167366666666668	0.745852770783688
PBX3	0.449824896233239	0.0374769151413555	0.249765891884698	0.57536723546666	0.229036000000001	0.237868383284494
ZNF827	0.449815247991119	0.0374769151413555	0.517716267763448	0.0911964988720938	-0.0571143333333317	0.822390330537826
TICAM1	0.449618869343821	0.037622649187345	0.0709538434193463	0.909400532983434	-0.160260666666666	0.446756640099031
HMCN1	0.449260873979642	0.0377692641221208	0.165982210087640	0.751869806279813	-0.827160333333332	0.085481985741103
APOBEC3F	0.448312692049015	0.0385859424814640	0.411208397217204	0.243981291680416	0.266086333333333	0.232020628857636
LTBP4	0.448213176308279	0.0386340962553086	0.0870400525955222	0.887055939766881	0.0235280000000012	0.93948504599535
MDC1	0.448014770768829	0.0387477661080561	0.220772334059583	0.6389751639565	0.0684410000000002	0.809979063354947

PPP3CA	0.447792818606205	0.0389074165568188	0.278714668943731	0.511025440770733	0.243662	0.266979834423163
CTBP2	0.447180175760368	0.0393871555697832	0.35370964232304	0.352236748429479	-0.00159433333333224	0.99628178244892
SUSD1	0.446750237525492	0.0397330753545568	0.152215680150808	0.775863712880333	0.474667333333334	0.0583198419957867
ZNF248	0.446565189574281	0.0398178133477837	-0.100515210509434	0.866538548597168	-0.00308033333333142	0.993756838831997
SLC12A1	0.446253823518762	0.0400391180478914	0.215579874008409	0.649490682443484	-0.147409000000000	0.524845938106282
FARP1	0.445876185762698	0.0402925174804839	0.142685605268632	0.794004622916083	-0.0163426666666653	0.956567100065073
ARL5B	0.445777601886519	0.0403025262810187	0.0234887202103245	0.975348968561756	0.134514000000001	0.546219696447415
SMAD2	0.445212718960823	0.0407701219078882	0.279596253233266	0.509655886591018	0.0838066666666676	0.707538975864338
RBFOX2	0.444859327733221	0.0410431019270338	0.396287980236732	0.271136524502442	-0.152466000000000	0.436132693279062
PRDM5	0.444791696879598	0.0410598653505066	0.431429990192051	0.205580120912679	0.0290206666666669	0.931467600666044
QPCT	0.444614613902891	0.0411397525864445	0.539614596926517	0.0705285823494608	-0.0654633333333327	0.766569862858904
CNOT3	0.444506083915317	0.0412019223794412	0.203908552178549	0.675615338672331	-0.150504999999999	0.477235955589408
PIKFYVE	0.44418093357941	0.0414953654796567	0.302904902587854	0.462105017205825	-0.132807666666665	0.544685122345675
SLC16A2	0.443889888780969	0.041596644818236	0.492462234121567	0.121640558692374	-0.275216666666667	0.285116883835521
TP53BP2	0.442805889517907	0.0425452869202817	-0.118686438034045	0.83731413798382	0.195985666666667	0.319483495880312
NFATC4	0.442771980165159	0.0425452869202817	0.473428896994384	0.145443641689931	-0.197073999999999	0.336481835976065
GOLGA7B	0.442610639733507	0.0426281648753193	0.654717098689854	0.0130668403975633	-0.215662333333333	0.248864745773935
ANO1	0.442094871173908	0.0431587031128673	0.101238958735457	0.866005093242444	-0.00152666666666624	0.99628178244892
ARHGDIA	0.441870061423335	0.0433581246769011	0.302454727078585	0.462229407853213	0.0637115000000001	0.775519275409347
KPNB1	0.441776687583442	0.0434063571905561	-0.0974169975464366	0.869861570992203	0.0423966666666687	0.864222734539396
CCNI	0.441459860002339	0.0437136344461206	-0.0261937642310305	0.970632644458507	0.0581466666666666	0.798752256977623
ICK	0.441113206975866	0.0440576942954212	0.370743603089748	0.320399684386315	-0.0139133333333319	0.969513647149429
NACAP1	0.440999809527408	0.04413015311733	0.122379268315677	0.829398647947819	-0.169359666666665	0.467798588118316
SMYD2	0.440729231391797	0.0443269844789221	0.567081536855435	0.0470952455251712	-0.00448533333333399	0.988942941807152
NES	0.4406190479241	0.0443961970797074	0.130381853027104	0.812271254601992	-0.172157333333333	0.570550813984136
SCAF1	0.440499485491457	0.0444766112485002	0.209955003394969	0.66322371639456	0.0168680000000007	0.95197590164177
MAPK14	0.440298862414317	0.0445166758905642	0.279143523692565	0.510839080271478	-0.0935763333333332	0.762269789916409
PBRM1	0.440260914096424	0.0445166758905642	0.143702427593479	0.792224028641432	-0.0168339999999998	0.956024791104257
ARHGAP1	0.440035172090439	0.0446669929714434	0.235549965711672	0.6067637636079	0.133490000000001	0.52333876394493
SPRED1	0.440031635238196	0.0446669929714434	0.0934197190805754	0.8764557345753	0.080121333333334	0.786438669493529
ROPN1L	0.439691000949091	0.0449501185979482	0.154513420817256	0.771310656070265	-0.167134333333333	0.534206202440822
PRPF3	0.439602551525103	0.0449818006934044	0.039988184648313	0.953370115695118	-0.203429333333332	0.304850986720083
SUPT5H	0.439557503278013	0.0449818006934044	-0.0143671023171641	0.983444353656142	0.0353846666666671	0.888476605628154
EXOG	0.439247220473271	0.0451164170488317	0.060108971333261	0.925649366494154	-0.144715999999999	0.464393642723292
ZNF460	0.43857548900362	0.0457438282534666	-0.151863556556866	0.776343233798271	-0.196658333333332	0.395459325761047
LNPEP	0.438213889720758	0.0458619216233238	-0.0501491451033155	0.941137137350672	0.233009333333334	0.245948767014956
PLEKHA1	0.438141091155017	0.0458619216233238	0.164337650441312	0.754189116283047	0.561234333333334	0.0238147039217215
RUNX2	0.438075060069726	0.0458619216233238	-0.134171996023347	0.808009477165608	0.0565403333333341	0.82667899725046
PER3	0.438065474129436	0.0458619216233238	0.242177175998048	0.59449178505628	0.0648100000000001	0.778995157976597
HELZ	0.43792146210526	0.0458861793637293	0.508544880437605	0.102449576952564	-0.0353243333333322	0.90945379498923
GAS2L3	0.437896947988027	0.0458861793637293	0.211296191524635	0.659974817413141	-0.270647666666667	0.333881200421942
sept-04	0.437322321870426	0.0464703276807627	0.425031285585577	0.215733128998344	-0.0892563333333326	0.764848773122078
BRPF1	0.436965088307592	0.0466774784242703	-0.113096396234370	0.846200067907049	0.0167650000000002	0.962219686887808
ADAMTSL1	0.436958635150272	0.0466774784242703	0.414141682967169	0.238217967147046	-1.01820216666667	0.00347244136732606
COIL	0.436947568451851	0.0466774784242703	0.228233637269019	0.623884329309077	-0.124205666666666	0.588509953674261
KCNA4	0.43687236442546	0.0466774784242703	0.166119262754281	0.751830402832405	-0.207666666666666	0.337307065334106
MORC3	0.436457380909909	0.0468631660829815	0.0452093439339317	0.946577404106596	0.344958666666668	0.126863181258387
TMEM221	0.436381200589876	0.0468813141056959	0.407006905324686	0.252941668074154	-0.184821333333333	0.336373925530627
SEMA5A	0.436230288090671	0.0469612306864138	-0.0770285014462708	0.901637911607815	0.160969333333334	0.427283471646461
BICC1	0.435550931199097	0.0475586985264262	0.0612784145826379	0.924274691992681	0.332398000000001	0.258700580229750
ZBTB20	0.435423849217541	0.0476559617314659	0.590739905683325	0.0365855475433461	0.0264836666666669	0.939515352878445
MYH10	0.435211826886196	0.0478593793534009	0.141060654659969	0.796353139796933	0.209772000000002	0.288747111194017
C10orf12	0.435081499133205	0.047961310256444	0.0192402954344621	0.97820434113681	-0.209753999999999	0.376224594108747
FANCC	0.434784829936462	0.0482110793767942	0.287635875165951	0.492887950509948	0.0388040000000005	0.874243298833041
HTRA1	0.43365404467206	0.0493995519101321	0.406844677053706	0.253220889638452	0.256535333333334	0.175763076550523
SEMA3C	0.433412673484668	0.0495849384170874	-0.267812137893424	0.53559545997875	0.434635000000001	0.0802641563431964
ATRN	0.43316545887439	0.0498411868571223	0.308296606657436	0.450974903594884	0.0940866666666677	0.661476512366005
PGR	0.432880801735296	0.0500460107382038	0.372638409856039	0.317205263174137	0.0459460000000005	0.843676733449892
CBL	0.432468971538603	0.0503603353508425	-0.262171616581385	0.549752906834892	-0.483886666666666	0.05368634135411
ZNF333	0.432406059241054	0.0503603353508425	0.159740456538981	0.763150272733332	-0.0615666666666666	0.784944347000133
FOXL2	0.432402519211235	0.0503603353508425	0.486573526232511	0.131139154244725	-0.133009666666666	0.641044966033937
ZMYM4	0.432331068608506	0.0503603353508425	0.033066797091668	0.961277493638149	0.0972650000000007	0.68171195530621
RARG	0.432106727414083	0.0504849427717616	0.210445123888868	0.661670522548494	-0.0821826666666653	0.727065902741195
DNM3	0.431588321621319	0.0509028813239603	0.296390603530668	0.476715216862939	-0.555341333333332	0.0230379245071538
PPFIBP1	0.43148177577581	0.0509080726862465	0.174488019203721	0.734267769889068	0.0466236666666667	0.855321176509099
COL4A3BP	0.431042616014682	0.051119892594992	0.266457491451596	0.538833471023229	-0.264352666666667	0.282721762943589
RAB11B	0.431035809775563	0.051119892594992	0.221160567585984	0.638281520637887	-0.0335196666666652	0.895093529187836
PTPN4	0.43097782812842	0.0511294578667047	0.281111791106722	0.506886267079183	0.325059666666667	0.145808308743157
ADRA2C	0.430933967425943	0.0511294578667047	0.0893375634869293	0.883460564717377	-0.113423333333333	0.637426515050706
RHBDL3	0.42921214651695	0.0531827644171954	0.380032784241379	0.300906670900397	-0.325106666666666	0.228292762690892
RAI1	0.429048929808653	0.0533409921159771	0.632237948202758	0.0208232988311238	-0.00743399999999938	0.982416094939651
LUZP2	0.428767408913211	0.0535767902759637	0.236320666496156	0.60521053717541	0.065158333333334	0.783190318471728
FAM13C	0.428635976482646	0.0535837438331936	0.606649124269784	0.0292131998244602	0.00359566666666734	0.993724132855888
SS18	0.428511684319188	0.0536897913396675	0.11623483142872	0.842218159791738	0.0455356666666674	0.861677398924522
DZIP1L	0.428365655774058	0.0536925702038891	0.150233781526728	0.779711456187392	-0.00060200000000012	0.998281147912498
DPYSL2	0.428342903785869	0.0536925702038891	0.330536800181181	0.402284079938133	0.0466703333333348	0.86521320388291
MICAL2	0.428111991662246	0.0539172877004033	0.203042594568559	0.6772404821028	0.165924333333334	0.587661126214482
HOXA11	0.42791792391672	0.0540003867534886	0.3924490409413	0.279133352313495	0.245064333333335	0.206251535561712
EFNA3	0.427886816474544	0.0540003867534886	0.597104710913947	0.0342685027740703	-0.758241666666666	0.0185304620090284
GNAI3	0.42777953458335	0.0540003867534886	0.00141071255590229	0.998303187767855	0.055746000000002	0.805830951784663

CPN2	0.427742196464908	0.0540003867534886	0.679600509244525	0.00781198449038317	-0.173356000000000	0.684569287653569
MGC16025	0.427645126768707	0.0540475125542724	0.0163185656099058	0.981907598420222	-0.0775069999999993	0.77130466329802
SLC44A2	0.427181781975668	0.0545604669034606	0.286180178995665	0.49631168283336	0.203530666666667	0.343611277724373
ANKRD13B	0.427010488962481	0.0546699793854333	0.170543542744631	0.741949507784887	-0.395264	0.0836149295154894
ATL2	0.426784033015136	0.0548702749368331	0.392806692431076	0.278297610043666	-0.0106289999999991	0.972130516588353
RAB36	0.426750096440768	0.0548702749368331	0.269440919620880	0.532367863371851	0.0353473333333334	0.911970751063452
SLC5A8	0.42672826187091	0.0548702749368331	0.280490302305942	0.507460240190768	-0.0941063333333324	0.66399337759607
RAB11FIP2	0.426525858425915	0.0549978509739766	0.377581183972004	0.306568453195763	0.437053666666667	0.0704370579064744
KDM6B	0.426497520094932	0.0549978509739766	-0.145664869628119	0.787804113309913	0.126726666666667	0.590101586536522
FN1	0.42645286041486	0.0549978509739766	0.271940189342729	0.527284725671175	0.641271333333335	0.132969129744691
UTRN	0.426295575265769	0.0550894768211521	0.147337500894049	0.78561060123541	0.181776333333334	0.476307520847818
SGCB	0.425915264882173	0.055258533304948	0.166671955018346	0.750443995356965	0.159278333333334	0.514076428873743
HECTD2	0.42590138941467	0.055258533304948	0.315984528570041	0.433782024195460	0.456972000000002	0.0338372724876968
SEMA6B	0.425861325277332	0.055258533304948	0.545909817597159	0.0635875421378674	-0.278562000000000	0.313220411537404
PDCD11	0.425838609860531	0.055258533304948	0.0479650092907102	0.94314236584312	-0.411765666666665	0.091806882215603
STX6	0.425016739135831	0.0561205969700506	-0.0772794356736285	0.901065188226888	-0.00624366666666566	0.985884500368235
TPD52L1	0.424737344483577	0.0563889535722484	0.539415217143651	0.0705285823494608	-0.288754333333332	0.188232886284507
TCF12	0.424646885406316	0.0563889535722484	0.136442686297868	0.804725680646266	0.123451333333334	0.551352991056569
LIFR	0.424230410169538	0.0567905176474756	0.634532367214295	0.0208232988311238	0.587683666666667	0.0155160072151846
LAMA2	0.423934445990085	0.0570213321847583	0.560198824815966	0.0531223791775081	0.209897333333334	0.315385252030064
CSN1S2AP	0.423696732590566	0.0572990291627036	-0.0488426912375497	0.942180113539637	0.0569333333333332	0.824327514351812
LAMA4	0.42355732320084	0.0573297194650224	0.144800290458512	0.789598951980813	-0.127973666666666	0.624457186990323
YBX2	0.423540705591981	0.0573297194650224	0.151524936896175	0.777101208632856	-0.0521173333333328	0.864688646629146
DLG4	0.423422875303947	0.0573809053923292	0.181387709362389	0.721174140548176	0.0471360000000017	0.841069468066102
KHDRBS2	0.422788263855195	0.0578987083604403	0.338428391318847	0.384701467775014	0.0927290000000004	0.730330043083783
LEPREL2	0.422759025976279	0.0578987083604403	0.271942324014538	0.527284725671175	-0.123670666666666	0.630112460203515
SOX12	0.42268905950779	0.0579168141704047	0.36338137103866	0.335749276958773	-0.0668599999999997	0.80758784150395
RNF152	0.422550150134597	0.0580543927679845	0.219588735686685	0.641226345931986	-0.0229029999999996	0.944218475609619
SLC22A17	0.422272964015669	0.0582456946626809	0.686638114437475	0.00671590527978087	-0.0711829999999995	0.756066672359783
MPRIP	0.422181870658229	0.0582456946626809	0.52952486177566	0.0799297163112453	-0.125577333333332	0.539562166114593
CACNB2	0.422169158641348	0.0582456946626809	0.361762352437237	0.338556116268406	-0.738781333333333	0.0300090638729873
PIAS2	0.421936238058441	0.0584338415133411	0.190521120780899	0.703920416197528	-0.0104216666666653	0.97435171941994
PRKD2	0.421351043394631	0.0591004762295528	-0.106795984352041	0.857800958803773	0.108259666666668	0.613258123073115
SH3PXD2A	0.420907484635016	0.0594535045440439	0.305159907119250	0.458013550008763	-0.00597666666666534	0.985884500368235
HOXD11	0.420376831649544	0.0598312071319686	-0.0133325747774232	0.98419257407872	0.107088333333334	0.605121621910816
H2AFX	0.420124158044739	0.0599598319012316	0.219403316138345	0.641226345931986	0.0183573333333329	0.948778075888142
CACNB4	0.420116410170785	0.0599598319012316	0.557614655969382	0.0549063676899021	-0.164353000000000	0.528409919089401
SULF2	0.419904069862268	0.0602126949907241	0.172500726178728	0.738421727152922	0.0512620000000001	0.839703258597695
GPSM2	0.419735727067224	0.0603349825548376	0.163523177871635	0.755132173727907	0.0928153333333344	0.720071550811076
KDM5A	0.419631339070629	0.0603614986535103	0.288384997511933	0.490238022687012	-0.103678999999999	0.648209367206976
C10orf137	0.419476571052039	0.0604974907640451	0.137387574152697	0.802126299522881	0.0694390000000013	0.750936504909256
EIF4B	0.419435116824893	0.0604974907640451	0.0225439321896043	0.97536722461972	-0.0282666666666649	0.91680951061779
SRP68	0.419357394425962	0.0604974907640451	0.364287228845504	0.333818991745846	-0.140861333333333	0.484208511056468
PUM1	0.41913475711372	0.0605889199989663	0.158623896216732	0.765791046741822	-0.0939553333333316	0.664067525814732
TSHZ3	0.418990080064208	0.060661233344196	-0.0680824440599642	0.912640418606148	0.336121666666667	0.274121181062751
LRRC8D	0.418897024912618	0.060661233344196	0.451216396400276	0.176663460992485	-0.0394389999999999	0.874960823800592
SF3B3	0.418872123788523	0.060661233344196	0.288047348326932	0.491376246980382	-0.36458	0.0740548894463108
GYG2	0.418809294873433	0.0606914017685531	0.0752779253956028	0.904173044514532	0.270421000000001	0.181157330290383
SOCS7	0.418747163637823	0.0607205840573575	0.297851692760387	0.472550555499985	-0.128470666666666	0.548138764076282
PHF23	0.418620603976929	0.0607474483256769	0.00111229056364851	0.99847747736415	0.0342323333333337	0.903788355106891
SBSPON	0.418601259526682	0.0607474483256769	0.572022278560861	0.0443803345694495	-0.00206133333333225	0.995146554639975
BMPR2	0.418512252770507	0.0608174161356337	0.0295348021889718	0.967226229119616	0.388930000000001	0.120936464750026
ZNF184	0.418018066373427	0.0612386804243887	0.0351347576423973	0.958931053746098	0.159375000000001	0.502766729725258
ITGBL1	0.417777595273707	0.0614800295005997	0.0795089241526912	0.89819077139711	-0.0182003333333314	0.951160188394976
BUB3	0.417691577086069	0.0615464994929744	0.37702323334652	0.308142256146131	0.0446466666666678	0.85013576481729
CSGALNACT2	0.417621908780621	0.0615763108699075	0.101076138163190	0.866005093242444	0.217972666666668	0.261011849103251
REV3L	0.417587385211151	0.0615763108699075	0.399310994764044	0.265509658507366	0.0855980000000002	0.690641668678273
RRP1	0.417290390124586	0.0617908521848227	0.222717247294582	0.635495054621817	-0.149444999999999	0.49679816436215
TMEM246	0.417245831737728	0.0617908521848227	0.263808194545853	0.545053560535766	0.133651666666668	0.57883453172984
PTCD3	0.41723581418462	0.0617908521848227	0.328818922708835	0.406249092981339	0.0110943333333344	0.972327702257384
KLHL11	0.417039582003502	0.062026658380999	0.223875479382906	0.633743682471632	-0.0586226666666654	0.791344461598316
NETO2	0.416746872758613	0.0623469712455893	0.414547692185006	0.237522444879778	0.0675756666666675	0.779668393144765
SPTBN1	0.416516223779756	0.0623868723517108	0.346917826795870	0.364568189046910	-0.0435166666666652	0.870662337224757
ECM2	0.416007310433003	0.0630160885386588	0.349815375079665	0.358977715627648	0.226994333333334	0.338777202459218
CCAR1	0.415957074901561	0.0630160885386588	0.201487845718150	0.680054545720343	-0.0283486666666657	0.91680951061779
MYADM	0.415898888629257	0.0630160885386588	0.0440266642481014	0.948520928173175	0.143270666666666	0.583036318848746
BMPR1A	0.41589515110163	0.0630160885386588	0.100142129053868	0.867048047646049	0.1884625	0.354076683624850
KIAA1671	0.415815761291044	0.0630745989198405	0.083542176030331	0.892833228561937	-0.0327923333333334	0.902208543897795
ATXN1	0.415737929753384	0.0631018729733093	0.0211703159080630	0.975855937594293	-0.0759873333333324	0.749395159463515
SF3B4	0.415689345569985	0.0631018729733093	0.108356198397267	0.854386098374291	0.0343520000000014	0.890073383340154
PDGFD	0.415554931221529	0.0631567846095574	0.183140113676880	0.718067440349544	0.115173666666669	0.720810534891902
PELP1	0.415381531250048	0.0633619678888261	0.239320553890069	0.599678129905115	-0.0386726666666654	0.874353540431336
PBX1	0.414979466351433	0.0638049163743112	0.354539582834893	0.349943361775722	-0.186345999999999	0.436132693279062
SF3A2	0.414973651704669	0.0638049163743112	0.339114972704013	0.382869626487208	0.0800903333333352	0.73097308185803
KAT6B	0.414909052657024	0.0638256845749997	0.121947296009444	0.830347805834363	0.0504290000000009	0.849578856396924
RBBP4	0.414857010048397	0.0638256845749997	-0.0232252490086536	0.975348968561756	0.0772600000000006	0.745685276815889
SMAD4	0.414836373867589	0.0638256845749997	0.137674413261976	0.801934376886443	0.160595333333335	0.419500365886586
SEMA4C	0.414486587166031	0.0641225024670377	0.0543482421968819	0.934486928733702	0.208694333333335	0.295708667353848
NDE1	0.414482318303469	0.0641225024670377	0.104955566895271	0.86067831050617	0.0266676666666678	0.941559580750404
TMEM151B	0.414299126298582	0.0641989350398051	0.0631757952744181	0.921359867071909	-0.0778579999999993	0.724001791100896

ASAP1	0.414232188927894	0.0641989350398051	0.362804244450249	0.337032280901653	0.0071240000000003	0.985484787924072
CBX3	0.41422833337234	0.0641989350398051	0.456650503614874	0.16754724558121	-0.146242333333332	0.459624813256835
ILF3	0.413896123293562	0.0645935387546893	0.239708035860167	0.598516926091973	-0.101233333333332	0.639837290428782
MGC57346	0.413694753430520	0.064625066345431	0.195053450218438	0.695157516663803	-0.150200666666666	0.479014333115247
ST6GAL2	0.413650696598818	0.064625066345431	-0.0492547106592583	0.941842562680095	-0.092882333333333	0.660199472343126
PGF	0.413574515419716	0.064625066345431	0.282964084616973	0.502129505146473	-0.172374333333333	0.421541794258528
SNX25	0.413511901848429	0.064625066345431	0.47484247468635	0.144197096534399	0.316186333333335	0.138581436793350
GPCPD1	0.413509101308976	0.064625066345431	0.0843945962564389	0.892302911210018	0.048863000000001	0.831076445763244
BCAS3	0.412543205455601	0.0656452307109125	0.468910570706081	0.151936428121882	-0.168199666666666	0.4261850174154
ARHGEF12	0.412328059733369	0.0659252952543439	0.143446470333088	0.792638117310597	0.00701800000000144	0.983159241812861
TNFAIP6	0.412060615457176	0.0662907240947266	0.0881385855476173	0.885569889492087	0.0325176666666672	0.910696784037738
DCAF7	0.411667173063256	0.0667165227848441	0.180198509541498	0.723415882806093	-0.157921999999999	0.479067209456976
ZNF609	0.411538401611436	0.0667165227848441	0.578151442286206	0.0431529075008732	0.0237586666666680	0.944097469332832
DACT3	0.411495507005147	0.0667165227848441	0.230966305835052	0.616488238055513	-0.157181666666666	0.669640159813955
HSPA13	0.41143493323699	0.0667165227848441	0.111803844883145	0.848202794339188	0.0370463333333342	0.880254449461876
BPTF	0.41112871838476	0.0669750509608712	0.231885444922913	0.613578980603213	0.097466666666668	0.680436423395844
PAQR8	0.411056274317134	0.0670061449314348	0.0057889075899431	0.99251028338798	0.448007000000001	0.0647596980686847
HS3ST1	0.410895270469803	0.0670881194925936	0.393430830215065	0.276819922369918	-0.101505333333332	0.706087144562033
SLC30A10	0.4108126528444	0.0670881194925936	0.375042184349182	0.312923979918102	-0.467698666666666	0.0584824376865046
MOB1B	0.410764182707432	0.0670881194925936	-0.0170848628515226	0.98105020869911	0.226877666666667	0.370122454788249
RFTN2	0.410752101662944	0.0670881194925936	0.470896401737749	0.148253719428246	0.0128233333333336	0.964995783450258
ABLIM1	0.410726855152161	0.0670881194925936	0.333973573306932	0.395721715641199	-1.08523033333333	0.0126478660661103
ADARB1	0.410225303602471	0.0676474417244594	0.183420279893509	0.718067440349544	-0.173184333333333	0.522694084864109
LONRF2	0.410162417508810	0.0676855193415906	0.435154474838114	0.197130044837380	-0.228593333333333	0.343572153228906
RNF169	0.40981955261598	0.0681185429118887	0.0622211290101256	0.92301259447339	0.207330333333334	0.312600912762022
INVS	0.409551529227896	0.0683649009524232	0.194571559305415	0.696017817587257	0.320835666666667	0.158485868172990
MBTD1	0.409265640316262	0.0687073201570783	0.274942394694639	0.520064783279337	0.217725333333334	0.297072964963319
CBX1	0.409182153573262	0.0687765112730797	0.148653176890154	0.78320766974403	-0.00752766666666705	0.98173288785111
PPAT	0.409137676918578	0.0687765112730797	0.117779109364997	0.838911179690148	-0.077915	0.728429595651334
ANKRD36C	0.409105682197547	0.0687765112730797	0.377992480214471	0.305766089617986	0.327514666666668	0.189835504217186
STT3B	0.408855336770874	0.0690617878912772	0.107308297142686	0.857128584932911	0.106206666666667	0.624073652680538
TEX10	0.408423020880834	0.069586893190559	0.0750583779273474	0.904466414650249	-0.0858913333333315	0.699711167221797
GTF3C4	0.408203095068141	0.0697583696958119	0.0755457394469744	0.903517361619051	-0.147778999999999	0.452846213844736
ARID1B	0.407832715196591	0.070225587586176	0.341716216281723	0.377337219880391	-0.124548333333333	0.560542533059595
ANKRD36	0.407808028405606	0.070225587586176	0.0339772447218979	0.95993706312687	0.177624333333336	0.449419820315226
MAPRE2	0.407721354879247	0.0702740736604308	0.38291393244814	0.294588782643652	-0.125080000000000	0.556572512115849
ARHGEF25	0.407430243841753	0.070666384054255	0.219485896231726	0.641226345931986	0.130693333333334	0.514889931507188
WNK2	0.40725572548823	0.070830172313981	0.211926242750908	0.658364968650984	-0.212507666666665	0.321008129295804
ATXN7L3	0.407216167707088	0.0708311574585712	0.0195137201342611	0.977673457110824	0.0928526666666675	0.673701601183362
FOXRED2	0.406704456739239	0.0714381894925142	0.460951305563075	0.161789531338644	-0.0148039999999999	0.95805797537369
SNAI2	0.406611300837047	0.071531128117574	0.47986840142039	0.138290570952161	0.310809666666667	0.141967552361024
FUBP1	0.406463477840091	0.0716979739283541	-0.0440594893392816	0.948520928173175	-0.103351999999999	0.616036866881905
CHSY1	0.406436321615278	0.0716979739283541	0.112877259322671	0.846769743490891	-0.0245233333333323	0.925845244747948
KIAA0355	0.406375159437661	0.071717162117565	0.0981148801599706	0.868852648210739	0.119543000000000	0.549659494702918
CRTAP	0.406347601291095	0.071717162117565	0.221585782557086	0.637480881894777	0.112773333333335	0.576725159099457
FLNA	0.406270496998404	0.0717393844130941	-0.478371562980426	0.140664651993598	0.112420000000002	0.62979571408014
GPR63	0.406257235721616	0.0717393844130941	-0.0704161472371223	0.91037453717015	0.147328333333333	0.467798588118316
S100PBP	0.406141846149972	0.0718119595087393	0.0617613258046941	0.923246185283376	0.272516333333334	0.148262582993822
CHST10	0.406137657642118	0.0718119595087393	0.424566578139553	0.216673177302171	-0.214441333333333	0.262756877023918
JARID2	0.405899001902304	0.0721376441150086	0.363512358687267	0.335533086114911	-0.347393000000000	0.0749561445182193
CACNA1C	0.405871129918785	0.0721376441150086	0.348462477721714	0.361311804770494	-0.2499395	0.203889537242039
MTMR3	0.405653891891447	0.0722873733758562	0.215131743537803	0.650993480683134	-0.0565913333333325	0.815309621716298
NOLC1	0.405547015416458	0.0722873733758562	-0.0413663563447843	0.952098348256047	-0.352030	0.120319574336004
ITGA1	0.405518441301652	0.0722873733758562	0.263845177675961	0.544971884819175	0.208315333333334	0.319443086223212
ANKLE1	0.405406382341061	0.0722873733758562	-0.148136355367879	0.784080588385358	-0.0279203333333328	0.938563429198324
ACAN	0.405397949654285	0.0722873733758562	0.388429304789536	0.28486220160813	-0.124209	0.703624525872923
FMNL2	0.405200424961735	0.0724998267190525	0.32630049262021	0.409206882509236	-0.0370976666666661	0.909076889745945
CCDC71L	0.405050290143127	0.0726046811967787	0.294747814896918	0.478800228795472	0.058679666666668	0.796894030375034
C10orf88	0.404733705195875	0.0728456912630123	-0.0130881301430589	0.984547546298896	0.0265773333333347	0.919420614501031
PTPN9	0.40460758061346	0.0729986064967984	0.0978362939547263	0.868852648210739	0.419488333333334	0.0392329313827969
FNDC3A	0.404277456082032	0.073374923309689	0.161630744811433	0.758955920988521	-0.0727686666666661	0.741134448923316
UNC5C	0.40413236453284	0.0734957693888411	0.305314647971952	0.458013550008763	0.428817666666667	0.174051133730666
TSPAN2	0.404023674604363	0.0736195110920687	-0.147292653834659	0.78561060123541	0.0624680000000002	0.77718596107678
ATP10A	0.403522222435013	0.0741191384409635	0.264484716250377	0.543860754965843	-0.0336453333333329	0.912165477430182
SLC44A4	0.403511900460188	0.0741191384409635	0.396802984072597	0.270862229167822	-0.330050333333332	0.28487334684302
sept-11	0.403396170026785	0.0742110470922246	0.0452575207338483	0.946556809762233	-0.183492333333332	0.449828629541335
CHD6	0.403353058886528	0.0742110470922246	0.0771714833936957	0.90125257838581	-0.059280333333332	0.821421133422134
PRELP	0.403346553729837	0.0742110470922246	0.231858329859144	0.613578980603213	-0.190846333333333	0.533922589751973
LIX1	0.403037949887798	0.0744897621801499	0.0327735718921551	0.961351562552925	-0.194865666666666	0.552125911260862
ITPR2	0.402896866371311	0.0746063232903876	0.300656496890001	0.465222095816255	-0.818585999999998	0.0802641563431964
PHLPP1	0.402726817543964	0.0747744399102773	0.457480553445687	0.166671273245692	-0.111539666666665	0.723993424337833
SLK	0.402525130989977	0.0750655503923879	0.211100218481046	0.660389781435267	-0.407637333333332	0.0833725664556335
ZNF280A	0.402412917514816	0.0751984056310111	0.0232320408182939	0.975348968561756	0.0483306666666673	0.846906282289456
TET3	0.402214784708686	0.0754266755980398	0.366221860888939	0.328771652750088	0.0553283333333336	0.843134844119548
ARHGEF3	0.401720857754521	0.0760086628538109	0.385145931077047	0.291074182838454	-0.749195666666666	0.0126478660661103
ST6GALNAC5	0.40166042558323	0.0760086628538109	0.304386947227199	0.4594472826444	-0.103911666666666	0.619415826722781
SOBP	0.401572296418276	0.076099960174365	0.495791729357317	0.116658734321069	0.0684220000000003	0.769217868422514
UST	0.401488367817553	0.0761838381792092	0.604316283701258	0.0305362664392858	0.161101000000001	0.488602547609567
ANKRD26	0.401410839623766	0.076256323162581	0.113360144818567	0.845747219093509	0.186866666666667	0.376020235114735
SMAD1	0.401359479739210	0.076281841749082	-0.118322315831013	0.838117566591658	0.0108526666666672	0.9715865162904

CTDSPL2	0.40120821998845	0.0764203843562086	0.119052296409025	0.836828045978697	0.182381000000002	0.385678201128102
WAPAL	0.401075150238981	0.0765643385929815	0.105554920149821	0.85956218633532	-0.0762713333333326	0.748579513438275
GNG2	0.400592729512005	0.0770395942623986	-0.119999175307796	0.835280975936648	-0.161464499999999	0.417125112561133
HSDL1	0.400516161534221	0.0770395942623986	0.324392798619595	0.412678992507379	-0.0359599999999989	0.892345321570547
SRSF12	0.400495487475292	0.0770395942623986	-0.162625448992292	0.757524249707797	0.00712000000000048	0.986692200659221
CRABP2	0.400452459824587	0.0770507875249428	0.385852079679211	0.289470192870154	-0.0362889999999994	0.894836430170129
TGFBR1	0.400398967469711	0.0770809854017239	0.118701934398613	0.83731413798382	-0.0880666666666657	0.68135180147671
CHST1	0.400280411654845	0.0772108328569556	0.436009389958971	0.196057392976856	-0.128642333333332	0.634587745028953
FCHSD2	0.400192574670622	0.0772108328569556	0.0138813915581813	0.983802420779736	-0.0457369999999991	0.856627065232437
SETX	0.399946200958758	0.0774361067206228	-0.181793510765309	0.720390591747688	-0.0792123333333318	0.73097308185803
MAGI1	0.399535358272141	0.0777846335147784	0.468258482968081	0.151936428121882	-0.214056999999998	0.427283471646461
CLIC6	0.399408884400759	0.0779058890214267	0.0450452064471379	0.94678213523172	0.496486	0.0235170204605595
ING1	0.399395956462081	0.0779058890214267	-0.203676514968022	0.676117692782917	0.130323000000001	0.519406653645538
EMILIN3	0.399344914790009	0.0779325588598044	-0.120575277696082	0.833850618726547	-0.100434000000000	0.718749100405876
ACOX1	0.399111091258073	0.0779972057332175	0.36699816443983	0.327869177739765	0.132236333333335	0.509869431796637
BOK	0.398903178890906	0.0780258155904676	0.336082602214139	0.389476012645306	-0.0459259999999989	0.878111320125249
STEAP1	0.39885869348019	0.0780258155904676	-0.067704882561376	0.913809294920045	0.0581963333333338	0.862744795576892
CORO1C	0.398697834062832	0.0781878411523636	0.00401172359857738	0.994485841304070	0.00244000000000107	0.994114279008565
SMAD9	0.398326541769071	0.078736440485224	0.233291992445977	0.610971609231637	-0.185606666666666	0.370208571402686
C10orf2	0.398175928182467	0.0787969446249323	0.240778751427285	0.596773408433565	-0.478875999999999	0.0649438674882112
H2AFY2	0.39816902959501 7	0.0787969446249323	0.129694027013106	0.813320950297213	0.0132103333333347	0.963466120529304
SMARCC1	0.398162860768919	0.0787969446249323	-0.0411808681660863	0.952098348256047	0.0371396666666676	0.883878395614985
SLC22A9	0.398149690807904	0.0787969446249323	-0.270323854966600	0.529999252995877	-0.031561	0.910190419163007
ZFYVE20	0.398045244817018	0.0789230593960687	-0.0969695990146953	0.870463314702701	0.113909666666667	0.593929171138774
RMND1	0.397985878303303	0.07892311467094	0.356824595277211	0.345138088652208	-0.300331333333333	0.217830538526318
RPL32P3	0.397209238028122	0.0800065235613037	0.308721566177287	0.449759364777049	-0.0809163333333330	0.794075220327397
C14orf37	0.397160395939582	0.0800308401375799	0.218054714887586	0.643730951697261	-0.259135666666666	0.235770973926881
GNB1L	0.397069566646507	0.0800943404847788	0.110158447581039	0.850412437492421	-0.0986846666666657	0.648963524582188
RPSA	0.396863895656638	0.0802989145335364	0.165525045404461	0.752352242644389	-0.110189333333334	0.631192833987568
FGFR2	0.396709436913507	0.0802989145335364	0.467633133697212	0.151936428121882	0.324917666666668	0.108684800104187
DLL1	0.396685048459642	0.0802989145335364	0.385844381225807	0.289470192870154	-0.00750166666666607	0.983159241812861
IER5L	0.396660082341740	0.0802989145335364	0.0784568282121454	0.899281198071059	-0.140411666666666	0.618886975439257
CLSTN1	0.396433722143273	0.0806557003812942	0.143831231718267	0.791963790519804	0.126633333333333	0.544685122345675
SORCS1	0.396301542827382	0.0808369272037257	0.539745117290611	0.0705285823494608	-0.262371666666667	0.390561931914011
MARCH7	0.395582450548884	0.0817506508577696	0.271374800307994	0.527984832801825	0.174445000000000	0.361781227674926
WDR82	0.395361831281358	0.082075941462793	0.39909184041131	0.266086446019874	-0.574427333333332	0.0739218716203512
LCA5	0.395212744074521	0.0822249519928466	0.611675359163719	0.0277519681937494	0.111925000000001	0.687939779688318
SACS	0.395115962003120	0.0823419434793774	-0.0769969721978944	0.901685964013167	-0.026231666666666	0.946966858255103
SMAD3	0.394905710933781	0.082608292573247	0.0978325984167479	0.868852648210739	-0.0918606666666654	0.66758573421638
SERPINB3	0.394682886746928	0.0828995465444179	-0.210262785452605	0.662040976946707	-0.0296926666666663	0.913456370971874
ZNF615	0.394558539137042	0.0830703463531559	0.0365886349839676	0.95721448412629	0.284488333333334	0.167461485379690
PDZK1	0.394477691894782	0.083086516829308	0.367597849557975	0.326948811683298	-0.031132999999999	0.90232248061402
NDEL1	0.394068206199746	0.0836074825274493	0.315026858765323	0.436065505708053	-0.0122493333333328	0.967695194723143
USP54	0.39391726719726	0.0837632822332062	0.471369469839366	0.147051558498479	-0.0839859999999991	0.728239972363265
ZFAND4	0.393683798070822	0.0840791572755912	0.131530750209627	0.811042872465441	0.136747000000001	0.535171605667144
XKRX	0.393557031826268	0.0842569639005698	0.488481196548769	0.127979276934222	-0.605079999999999	0.0427664381032382
NRP1	0.393446939865851	0.0843872596179865	0.255270396554041	0.566072097489443	0.624546666666667	0.0493233390534422
SAP30BP	0.393419897273749	0.0843872596179865	0.281333842645287	0.506585071809407	-0.155649333333334	0.443072320689246
YY1AP1	0.393336390944959	0.0844815592416697	0.121195780987550	0.83256316766566	0.0549406666666669	0.818849076927228
COL11A1	0.393146799021915	0.0846465047642521	0.496387070271732	0.115856499197964	-0.0722106666666666	0.792168081595258
PDZD8	0.392779451249086	0.0852273971328808	0.291624713736063	0.485747109971932	-0.263902333333333	0.212873167238858
CABIN1	0.3923760425387	0.0859514890402818	0.104177033965476	0.861275170239332	-0.311237666666666	0.218478495014124
DDX18	0.392234085720404	0.0860251984335728	-0.142713052226499	0.794004622916083	-0.0639649999999992	0.791599529156989
POGZ	0.392050744579884	0.0862042603848057	0.176268024452324	0.730407919148629	-0.00845733333333201	0.980779858267938
ACADVL	0.391749904110183	0.086320593467158	0.38452184267203	0.291180853572672	-0.125882666666666	0.575589336285762
TCF20	0.391545232861446	0.086320593467158	0.327809115720656	0.407901035323684	-0.289892333333333	0.135559179623298
FAM60A	0.391530152987124	0.086320593467158	-0.203967235770585	0.675452833287	0.141018666666667	0.592716584553539
SPON1	0.391228771476024	0.0867808648536482	0.164253611613519	0.754189116283047	-0.300653999999999	0.151960561010493
BBS10	0.391175005602784	0.086819241600439	0.259804589684076	0.5559952440425	0.0652326666666672	0.777406710539424
GPR162	0.391032827961511	0.0869649811018238	0.276159413329525	0.517549833373363	-0.257160333333332	0.277510060203337
B3GALNT2	0.390962465596923	0.0870365333986352	0.378891076669247	0.304578046752771	-0.0449286666666659	0.87290879338693
GCNT4	0.390821753922133	0.0871405987551675	0.0542207702123004	0.934737989077027	-0.144661333333333	0.750399431506523
MED13	0.390716123014958	0.0872536179356933	0.469626670308086	0.150981051310306	0.0372120000000007	0.88588039554932
ATP2B3	0.390420598760338	0.087344687060283	0.217413780845156	0.645724712341553	-0.261077666666666	0.216858796679299
ANP32A-IT1	0.390292107386994	0.0874177641256448	0.266155721914230	0.538986305036309	-0.123188333333333	0.535492155031224
SYNRG	0.390232869306078	0.0874679929278079	0.195560319937411	0.693823574367047	-0.203317666666666	0.332241648424953
FAM212B	0.390048048188641	0.0877011110139052	0.253830525395030	0.567694846153007	-0.0230946666666655	0.938640008704917
RPL37A	0.38988560815623	0.0879007384837027	0.397652726527735	0.269536689655461	-0.166579333333332	0.498456714564834
LRRC17	0.389880268728120	0.0879007384837027	0.416731250061752	0.234235464483992	-1.02241133333333	0.0411983064548969
ID2B	0.389719923382942	0.0879501253712977	0.26464695065688	0.54347643041286	-0.107055000000000	0.605719554165406
ANKS6	0.3895917253065	0.0880033196100157	0.132461782042095	0.809866082339655	0.0673293333333341	0.77740028876925
CWF19L2	0.389506896857293	0.0880033196100157	-0.166217359830264	0.751661811727111	0.0962633333333334	0.686516408582019
FARSA	0.389447810356587	0.0880033196100157	0.172229858128163	0.738567943867944	-0.192562666666666	0.317596397166712
VN1R10P	0.389423065143582	0.0880033196100157	-0.313584451005514	0.438046311563112	-0.144816333333333	0.471680039257208
CASC3	0.389256648262405	0.0880781477214702	0.09807496223397	0.868852648210739	0.031659333333335	0.900904966423356
CCDC138	0.389188210142634	0.0880781477214702	-0.146363529372446	0.787103461358382	-0.0358803333333313	0.895524919093865
PPP1R9B	0.389183584919671	0.0880781477214702	0.0428609187776016	0.950491677163598	-0.0528216666666656	0.843152489417868
LRFN3	0.388943693700497	0.0881565807099983	0.27125818585208	0.527984832801825	0.0514706666666673	0.843152489417868
NUCKS1	0.388520924087503	0.0884596478334427	0.214680199032572	0.652003104177839	0.00678566666666682	0.983606684744139
MACF1	0.388473016913322	0.0884596478334427	0.049687809590794	0.941458038227918	-0.0278623333333312	0.937545603686416

NOB1	0.388459095099165	0.0884596478334427	0.47575890549105	0.143137411589934	-0.157896333333333	0.512796060754107
RHCG	0.388360054042638	0.0885195474883208	0.0648717369193606	0.918590305362052	-0.168647333333333	0.408649882702122
DTX3	0.388245343800667	0.0885195474883208	0.585014269365461	0.0394762869288458	-0.124871000000000	0.657217907037306
ENAH	0.388245033478460	0.0885195474883208	0.207085447059828	0.669674823913575	-0.0789436666666662	0.76409396614905
PPRC1	0.387351071935359	0.0899607930333406	0.121055547683153	0.83265693609154	-0.202136999999999	0.346165986086181
GLCE	0.387193781062194	0.090157180760585	0.317762786532776	0.43078084496581	0.0195800000000013	0.948469012194168
PCGF2	0.387054802863279	0.0903622781144224	-0.0014284887159301	0.998303187767855	0.0403770000000014	0.870996259555823
RFX7	0.386752579670786	0.0907776614373789	0.190294913116710	0.704208582824765	-0.323230333333333	0.187680490274970
IRS1	0.386597683772691	0.0908205898627958	0.34790382742634	0.361983566065810	0.0425993333333349	0.888285531317364
HEPH	0.386590463356744	0.0908205898627958	0.450766429353524	0.176663460992485	-0.0989179999999996	0.650983465855075
KLF14	0.386495047664272	0.0908332907179195	0.447077281243368	0.178525985283524	-0.146388666666666	0.474709570893085
EHMT2	0.386148710909313	0.0912151767285861	0.176192269569187	0.73052037591204	-0.0402386666666647	0.8754328609757
MBD5	0.386037780257743	0.0913679729165923	0.203874070085327	0.675615338672331	0.110995333333335	0.602084409940976
ZNF280C	0.385738772901136	0.091714409814852	-0.0576778478053107	0.929758936765496	-0.0291503333333326	0.947020470290463
FAM168A	0.385630358523614	0.0917360623110668	0.121109965669006	0.83265693609154	-0.0403453333333325	0.880347900939311
EXT1	0.385559969430897	0.0917617535955077	0.335936934779338	0.389782452857155	0.347631666666668	0.0846385443172634
SPRED2	0.385552726056377	0.0917617535955077	0.0361913990157739	0.957959538423961	0.082441666666667	0.718962395682555
CHMP1B	0.385163270465016	0.0923824973441003	-0.245816891331799	0.583500894076193	0.0871610000000022	0.710004432586908
PTBP1	0.3851433921716	0.0923824973441003	0.472513366876786	0.145443641689931	-0.0182299999999987	0.94871117680979
PFKFB3	0.384940513918627	0.0925194948261704	0.153651993587754	0.773358453580566	-0.187905666666665	0.45053278513855
SLC18B1	0.384889035258037	0.0925194948261704	0.43397131026338	0.200238395353932	-0.243215666666666	0.228292762690892
SCARNA17	0.384878336152396	0.0925194948261704	0.123300672242861	0.827531665631642	-0.228132333333332	0.353471671280201
NFATC1	0.384279021989403	0.09311925063447	0.0511889594415265	0.939111418302491	0.0669806666666666	0.762898679429892
CPSF6	0.384250215728586	0.09311925063447	0.332717235196566	0.397958145642584	-0.102100666666667	0.617967324583825
CDYL	0.38411975022951	0.0933242753590603	0.271913492315127	0.527284725671175	0.076991333333334	0.717906134812783
SPIN1	0.383960811352294	0.0935892899603202	-0.081152472030557	0.89635426614511	0.0705433333333343	0.762898679429892
ATP8A2	0.383781043954252	0.0938986658362268	-0.111201198699208	0.849127967004266	-0.0025026666666670	0.993824047902985
DSTYK	0.383564037353932	0.0942195504142274	0.0812017904619362	0.896346444707454	-0.229516333333333	0.218972857603525
ABI2	0.383336861551046	0.0945628978670474	0.114074130897021	0.844272085764738	-0.431472999999998	0.0425543345284076
CCP110	0.382858997638687	0.09526968743483	0.280775689056533	0.507365125605885	0.228254000000001	0.312960077512277
UBE2S	0.382842537352925	0.09526968743483	0.107583706418171	0.85680004995345	0.157076666666667	0.479138506340968
ATP1B3	0.382619868306867	0.0955967614368503	0.0100870508348864	0.988421999436642	0.167733000000000	0.410252743069801
SCUBE3	0.382592578551813	0.0955967614368503	0.166797514544619	0.750325668175428	-0.0531909999999999	0.82667899725046
BTBD2	0.382008993690153	0.096413544393492	0.312683707730309	0.439245998265575	0.112397333333335	0.578893615511221
ST3GAL5	0.381986440850649	0.096413544393492	0.657011720733761	0.0127495293055252	-0.321635333333333	0.295142799341623
CA11	0.381738440663173	0.0968777386092835	0.250541417804334	0.573763059271745	-0.487156333333332	0.0654528404724121
HOXB1	0.381341543470784	0.0974172532368198	0.140207163847597	0.797441782082887	-0.0156426666666661	0.961993177180408
RPL15	0.381267849018853	0.0974172532368198	0.305094712789321	0.458198595388891	0.121298000000001	0.589826330962094
HNRNPH3	0.381229216215622	0.0974172532368198	0.258282920437063	0.560815056132889	-0.0233703333333317	0.946623914607177
EP300	0.381223851313312	0.0974172532368198	0.140232593726452	0.797441782082887	0.140328000000000	0.590147690656472
RAVER1	0.38109512925373	0.097578700729986	0.523255715823126	0.0869156803871297	-0.0683226666666654	0.755697432495201
SOWAHA	0.380863650675945	0.0980117023373435	0.236463602096266	0.604903671615182	-0.089301666666666	0.683854807591317
UAP1	0.380667013400784	0.0981904839622752	0.142981519805896	0.793357892012091	-0.139383333333331	0.49000765832939
NHSL2	0.380537182544956	0.0983767016081567	0.0262527367356214	0.970632644458507	-0.165239999999999	0.409335260696998
GNAT2	0.3804013930656	0.098558180421945	-0.0720915540554428	0.90824872013293	0.0865860000000007	0.69523606597876
SNAI3	0.380390567982842	0.098558180421945	-0.0367152207552713	0.95697345043597	-0.117015999999999	0.65692454979738
DGKA	0.379988334044619	0.0992997698675293	-0.0344203049393615	0.959404955370931	0.322970666666668	0.147305400857476
SPAG9	0.379542681488886	0.100002238233353	0.166371458697326	0.751360233962663	-0.0155133333333317	0.95787549045386
ZBTB5	0.379401185979393	0.100105452381584	0.108779285789537	0.853552381097052	0.102350333333335	0.692494286263795
FRRS1L	0.379279015398917	0.100305782088258	0.45000315330104	0.177025296973857	-0.0485456666666658	0.889343227773276
WDFY3-AS2	0.379057807418872	0.10052231746096	0.504675056692988	0.106882834148277	0.213683666666668	0.266449764451170
FAM171B	0.378923514950166	0.100688379308502	0.332085136705493	0.399068746068573	-0.0705899999999983	0.843134844119548
TMEM237	0.378794166922661	0.100892022136014	0.344859946444209	0.369280599599459	0.243072666666668	0.210325099284251
SMARCA1	0.378437431643624	0.101336573146582	0.0289698318350696	0.96782691865619	0.340760333333336	0.130006790529838
NR4A2	0.378374735499151	0.101406461243018	-0.0369367175578573	0.956918184553224	-0.161763666666666	0.505048087568739
PRAME	0.378319047394750	0.101460779509821	0.0670125044922648	0.914971726375863	-0.117796999999999	0.561477177160103
INHBA	0.378269736751689	0.101491522735275	0.118792748359655	0.837182040755817	-0.552928999999999	0.0286129614738536
C21orf15	0.378167762494953	0.101523480922815	0.452043146587344	0.176223482565411	0.213754333333334	0.3398957099824 1
SORBS3	0.378007504706620	0.101667593558085	0.421984992922973	0.222319600995852	0.222644333333333	0.319316647933142
MSS51	0.377787987937105	0.102017925705622	0.0457632238970596	0.94547820490174	0.00251500000000015	0.995321141422816
LMOD1	0.377547409208918	0.102209230370998	-0.0447355326036723	0.947776877450408	-0.156806333333333	0.463362442026763
CCDC86	0.377458600264776	0.102338723260591	0.181090305363018	0.721631445609392	-0.457898333333332	0.114599211633843
GGTLC1	0.377386148136533	0.102431621708224	-0.108840550226777	0.85348092441742	-0.0594969999999994	0.7996066102903
WDR63	0.377008248651177	0.102793157639910	0.273645771428576	0.522974581803368	-0.156625999999999	0.45895048781174
PRRC2C	0.376896822259485	0.102938571606785	-0.0160573315066225	0.982057247916055	-0.0168593333333316	0.962781154083931
ZNF792	0.376722864841568	0.103013228709236	0.483188823512855	0.135538365320535	0.0278629999999997	0.921373678345685
TGFB1I1	0.376656709158987	0.103013228709236	0.381325629757715	0.297930089233645	0.153065666666667	0.443072320689246
NQO1	0.376596330605965	0.103013228709236	0.449346722992663	0.177764116411209	0.164803333333335	0.405044878280701
ARL4C	0.376585726194834	0.103013228709236	-0.0697405686456385	0.911167570018484	-0.569869666666666	0.120319574336004
DAPL1	0.376570963599766	0.103013228709236	0.289128567541696	0.489048761714482	0.112547333333334	0.717743913529804
BCL2L15	0.376482201807832	0.103143991525684	-0.0057328672435139	0.99251028338798	-2.346362	0.00812139402984378
GPR125	0.376389304175758	0.103212608170703	0.364087762897724	0.334465690107878	0.0421823333333351	0.870996259555823
PRSS35	0.376359569802398	0.103212608170703	0.633015107545282	0.0208232988311238	-0.166762333333333	0.519933704213964
LAMC1	0.375967155002571	0.103664022094523	0.170026568609674	0.742972229508731	0.410874333333334	0.0777324007395152
ORC2	0.37594521386416	0.103664022094523	0.378444656550087	0.304660593775452	0.0741920000000004	0.736441343012057
AOC3	0.375826938771358	0.103861481323428	0.361746916157439	0.338556116268406	-0.0891808333333325	0.66866470442489
PNMA2	0.375796859426166	0.103861481323428	-0.112581921489565	0.847191484945575	-0.0284406666666659	0.919420614501031
USP36	0.375670104337383	0.104078251026435	0.177271773210103	0.728735147897621	-0.142728333333333	0.499957077244054
LRRC4C	0.375418111160432	0.104352900653005	0.293778154095106	0.480614908214975	-0.0272189999999997	0.936597933380236
HERC1	0.3753972376313	0.104352900653005	0.201377692354275	0.680054545720343	-0.0416179999999999	0.903864363916236

ARHGAP35	0.375211750156983	0.104497713946850	0.0571228514517846	0.930775493791569	-0.218373666666666	0.316406201267963
GSTT2	0.375050840937682	0.104725757071942	0.124243341244627	0.825382714207684	0.0710560000000019	0.799660020384009
NBPF10	0.373960441279462	0.106462789375348	-0.0467729408573979	0.944274732245152	0.0934746666666674	0.662897939907853
KCNJ8	0.373905971796858	0.106518702808703	0.416962477043511	0.234235464483992	-1.60697466666667	0.0230379245071538
AKT3	0.373611176217024	0.107061429412557	-0.105848746289799	0.85941024686388	0.219448333333334	0.271815033755243
KCNMB4	0.373378833219188	0.107460982039930	0.370929122573833	0.320399684386315	0.346460333333334	0.0757983407969203
ZNF587B	0.372951102841211	0.108244824324918	-0.113574681935743	0.845326910026624	-0.371412333333333	0.0619126099646093
AR	0.372922324019578	0.108244824324918	0.442590818816613	0.185282268884974	0.197861666666668	0.422658139524950
PLCG1	0.372891846215712	0.108245486758038	0.229133154382398	0.62075895683145	-0.00871433333333187	0.979710875456576
ELK1	0.372796700916929	0.108397730506784	-0.00993118343605738	0.9888083127414	0.0290870000000016	0.913846966562145
LRP2BP	0.372694410824928	0.108566943609832	0.174321023663801	0.734267769889068	-0.177288333333333	0.462293374281597
PLSCR4	0.372382425754726	0.108951046788686	0.430759452885018	0.206533702654214	0.586859333333334	0.0139341065577702
KRT76	0.372362432952522	0.108951046788686	-0.14516418380589	0.788615482689992	-0.0973683333333333	0.750929580167368
DUSP5	0.372320208607055	0.108951046788686	0.258226600287572	0.56103645723532	0.0227666666666684	0.932154136447394
TAOK1	0.372244134333153	0.109059388513826	0.225767261142976	0.629331969778772	0.121645333333333	0.559408774818177
GIGYF2	0.372138623989301	0.109166447178385	0.250404941181728	0.573888735446956	-0.195808333333333	0.376224594108747
RELA	0.372076170320976	0.109242921742274	-0.270403347088707	0.529999252995877	0.0228180000000007	0.936634943217749
ZNRF3	0.371402717304562	0.110195874734938	0.155376160699941	0.770468719772519	-0.648466666666666	0.0351167468854759
PLXDC1	0.371324576255719	0.110242164875977	-0.0275217899383805	0.969866457453454	0.120482333333334	0.637602236970228
FAR2	0.371264522521948	0.110243028963620	0.460953378115645	0.161789531338644	0.103939666666667	0.610847413222267
TET1	0.370652840746404	0.111405356784997	0.395225367596703	0.272692742937895	0.300765666666667	0.139567607482897
SF3B1	0.370597675217867	0.111405356784997	0.101402652762758	0.865954921256394	0.0257876666666682	0.92469922248949
CHD3	0.370568363190972	0.111405356784997	0.238617979377573	0.600861960738847	0.178770000000001	0.383186367186593
JMJD1C	0.370484208758718	0.111535642342826	0.194620586701556	0.69591561649809	0.195957000000001	0.320225451789804
SLC25A28	0.370335323973293	0.111750038234126	0.463731008454695	0.157277647666176	-0.0531616666666652	0.834978816259208
GOSR1	0.370271334496812	0.111832301499939	0.0268508793582058	0.970207612777188	-0.128638333333332	0.516040564500327
COL1A1	0.370149961377469	0.111972980650452	0.165075101150897	0.7529423475214	-0.0372783333333327	0.880347900939311
COL16A1	0.370046587277145	0.112015791405991	0.0958280003445074	0.872499931194687	-0.0737373333333328	0.826163946239814
ULK4	0.369955180375202	0.112164327307503	-0.121816058264542	0.830876156053538	-0.103478833333333	0.707966006319856
CBFB	0.369835266811655	0.112381710108467	0.0943606734350287	0.874489825505267	0.108367666666668	0.669179536566923
FGF9	0.369774591520942	0.112420889767473	0.109591134822130	0.851509079273215	-0.685096333333333	0.0667771856938434
SLC38A10	0.369467236309123	0.112802690469409	0.167024679638601	0.749860124947635	0.0634406666666673	0.800216081516012
ETV1	0.369348379088310	0.112802690469409	0.381088992540633	0.298110142491852	0.121930000000001	0.609858095838542
ITCH	0.369339146617415	0.112802690469409	-0.00630506605557406	0.991853352023739	-0.0734373333333331	0.750408632709156
RPTOR	0.369282928563691	0.112802690469409	0.542240290808958	0.0675663993915656	-0.481874666666666	0.0906008535785511
CCDC80	0.369247570415398	0.112802690469409	0.276666893038172	0.51671109845278	0.292403000000001	0.131638590307817
TRIM37	0.368440935808247	0.114230783127189	-0.081986738989294	0.895276371558631	0.096383000000001	0.710855214469315
HOXB5	0.368413144380405	0.114230783127189	-0.042388413370217	0.951464871947228	0.153061000000001	0.483277436143082
QSER1	0.368383653898432	0.114230783127189	0.111359451867069	0.848968422336654	0.142852333333334	0.606657486378823
ACTN1	0.368222896257661	0.114292809751477	0.326415756364819	0.409206882509236	-0.00608733333333287	0.9879739922351
ZNF469	0.368165243652131	0.114362124979439	0.533186914226812	0.0759909410633651	-0.0968133333333335	0.720071550811076
USH1C	0.367977635507821	0.114749045267863	0.221990650005270	0.636647953876148	0.0669596666666673	0.825958051615143
MYO9B	0.367812438796387	0.114972998879115	0.43379669297285	0.200472406424481	-0.0608866666666656	0.815309621716298
SP5	0.367798539275611	0.114972998879115	0.693811539632335	0.00553622192861942	-0.250941666666667	0.378344763124493
SALL2	0.367601836129508	0.115312332819754	0.0433776710319095	0.949668716947184	-0.231460999999999	0.27071513514891
MCMBP	0.367462094445463	0.115512427810662	-0.0125679692894753	0.985220352735461	0.118381000000002	0.55248070124039
HSP90B1	0.367317748479280	0.115652583536823	-0.217739682887404	0.644743872041817	0.09693	0.638783129327698
MYRIP	0.367249225537811	0.115749634465092	0.0726253480964164	0.907556175729082	1.01251533333333	0.0454558903953922
PRRC2A	0.367177842324864	0.115808664361563	0.320643412751801	0.421921269743406	-0.0207581666666650	0.944425994813353
ATN1	0.367030415890081	0.115933408219958	0.283210038475082	0.502129505146473	0.0306153333333334	0.910190419163007
ZFR	0.366957595216059	0.115958473521989	-0.27808108612964	0.512275963576091	0.0849826666666687	0.6846855637534
BGN	0.366947053311966	0.115958473521989	0.326083969062575	0.409206882509236	-0.154463666666666	0.625510328921698
GPR87	0.366932192415371	0.115958473521989	-0.087624790446521	0.886302037299497	-0.154395000000000	0.575378149225683
COL4A2	0.366886035246048	0.116000840941030	-0.0463402943671614	0.945000118027662	0.498178	0.0262046791709735
ZFAND6	0.366535239295987	0.11662139364643	0.161828278451235	0.758930446817487	0.135534000000001	0.530602707379549
SFPQ	0.366501587281337	0.11662139364643	-0.0278041651825112	0.969317858168394	-0.101883333333332	0.625510328921698
SMS	0.366150310290238	0.117010546464980	0.514615710357418	0.0952714966527572	-0.183646333333333	0.352907257643214
NFRKB	0.366136938222215	0.117010546464980	-0.0430134294278314	0.950146638225747	-0.0271089999999987	0.928295508521289
IL8	0.366136591837858	0.117010546464980	-0.264589508794979	0.54347643041286	0.175446333333334	0.373233057186249
IMPA2	0.366107003635816	0.117010546464980	-0.0415008601548278	0.952098348256047	-0.0938536666666665	0.760617383358761
ACOX2	0.366102267472899	0.117010546464980	0.448591839496098	0.177860736739947	-0.251369333333333	0.359827323900633
ORC4	0.365858672960907	0.117472690476800	-0.0402104779782990	0.952930192037229	-0.0097790000000001	0.974451420920923
ROBO1	0.365761470816945	0.117643055778866	0.136372897723092	0.804864448119512	-0.0204736666666659	0.93931605271847
ATAD1	0.365694209273629	0.117667321153380	0.306179913599897	0.456406236471471	0.146384666666668	0.525218637875881
PDGFB	0.365183598742813	0.118728556910849	-0.0406546229365576	0.952426531595115	-0.0551630000000001	0.825260826676022
PATL1	0.36491780169748	0.119107856493597	0.0793410021893851	0.898323801514594	0.0184446666666666	0.948469012194168
TNS1	0.364807487052203	0.119241145600060	0.336238570957033	0.389442467555813	0.0935896666666668	0.724915779411004
KIAA1644	0.364737159506398	0.119346094752773	0.391739198182139	0.280141098619812	-0.685578666666666	0.0324128117394227
COL12A1	0.364461284989104	0.119594992087809	0.0965071241639032	0.871093247497475	0.393383333333334	0.187638103900419
MYH8	0.364420201261401	0.119594992087809	0.243251369171224	0.591755192777173	-0.0491523333333324	0.838927397112197
AFAP1L2	0.364354550134356	0.119594992087809	0.159758470570168	0.763150272733332	0.0500893333333334	0.880427463662501
ARHGEF4	0.364175508649784	0.119898041846824	0.288448071514459	0.490230108819486	-0.128232666666666	0.680647240841787
ZDHHC8	0.364066151446526	0.120030476507993	0.0508886092939582	0.939774863060813	-0.0804876666666666	0.72802494241369
CXCR7	0.363976736205556	0.120184687184084	0.52082703331761	0.0890016678141832	-0.143848666666666	0.484747721903707
PDS5B	0.363817769857368	0.120515435117461	0.00293078143012374	0.995261259442339	0.111227666666667	0.635267430402483
SCARA3	0.363735896320250	0.120578828889000	0.6392721778922	0.0185595110823276	-0.130209000000000	0.519504252412068
RFC3	0.363418352754022	0.121169507454497	0.0153291363028018	0.982725338620588	0.0283606666666664	0.920830042057353
RBPMS2	0.362636904478525	0.122734733989555	0.279924043361611	0.508570618447507	-0.0126003333333326	0.97002499251073
CTDNEP1	0.362583805484592	0.122783585581279	0.101445333290117	0.865885289830816	-0.0258126666666654	0.932469157246082
SGPL1	0.362560843081883	0.122783585581279	0.277397800325266	0.5144731963081	0.0180990000000013	0.954180927004992

ZC3H4	0.362461726383016	0.122783585581279	0.185067002444352	0.715658781310868	0.0855096666666675	0.6846855637534
ARPC4	0.362425996909681	0.122783585581279	-0.154541059357718	0.771310656070265	0.0202233333333346	0.944218475609619
MREG	0.362362908534520	0.122783585581279	0.573650638920388	0.0442317804409017	0.00126800000000119	0.99704422037925
F5	0.362358148229396	0.122783585581279	0.0141336915369595	0.983444353656142	-0.0622723333333331	0.819757926662461
MYC	0.362159224596104	0.123018003355817	-0.0347033456048797	0.959404955370931	-0.835169666666665	0.0252836057158779
TNKS2	0.362028264694439	0.123280281634975	-0.0193487833051580	0.9780685294577	0.166803666666667	0.384248044508942
CEP97	0.361960435980348	0.123312679051701	0.222199840907369	0.636477566065103	0.0351386666666677	0.895093529187836
CYYR1	0.361831057698348	0.123501429695289	0.149508845708448	0.78215339162259	-0.0302859999999998	0.942393983671961
RPRD2	0.361647839192443	0.123784434912521	0.256719552183086	0.564159017531156	0.112547333333334	0.621414476984274
FAM208A	0.361454808685206	0.124038838342149	-0.0206856788624759	0.976330597778846	0.340726333333334	0.0896797344789652
CENPC1	0.361425343152696	0.124038863607691	-0.191512038333701	0.701635983922402	0.227074000000000	0.281614059742716
ZNF70	0.361221765150093	0.124279669362712	-0.112839830002551	0.846886684524482	-0.0828019999999995	0.707538975864338
SEZ6L	0.361062552966259	0.124475071907297	0.124629896612757	0.824945010244247	-0.200370333333333	0.43923010114809
AP2B1	0.361010881524898	0.124480738138799	0.102790370265534	0.86257932725575	-0.0400826666666666	0.878139763624324
IL34	0.361004446955086	0.124480738138799	0.235356898335267	0.60678764664537	0.00522500000000055	0.99117823918328
GPR171	0.360842210308002	0.124532615246582	-0.320874740762341	0.421618886526906	-0.100259333333333	0.640799282945291
LEMD3	0.360800366756233	0.124532615246582	0.0989804520335247	0.868042453226125	-0.472923666666666	0.100679552334348
RPL18	0.360797583993583	0.124532615246582	0.579704501106464	0.0423056676199601	-0.0373899999999984	0.883775402587518
TJP1	0.360761326322263	0.124532615246582	-0.054590334648151	0.9344692810234	0.249911666666669	0.346165986086181
CPNE8	0.360729287170967	0.124543541919242	0.389396592200555	0.283343454325927	-0.142987999999999	0.646350580563869
HEATR1	0.360636929615084	0.124706154771632	-0.104745657808983	0.86067831050617	-0.249644333333332	0.322494916341941
NCKAP5L	0.360459821730487	0.124882592745293	0.43644863204249	0.195288910691951	-0.133670999999999	0.54292132372707
KLF2	0.360432143123351	0.124882592745293	0.265724217327548	0.539878668275766	0.0256330000000012	0.945609249176696
PAQR4	0.360248314101547	0.125197418013525	0.305224187747811	0.458013550008763	-0.175193666666666	0.41666677641248
RC3H1	0.360228066001862	0.125197418013525	0.187535517467321	0.709248042430951	-0.0710199999999981	0.76307213318662
ELK4	0.360179070958827	0.125247854530309	0.267980737385275	0.53559545997875	-0.0674939999999995	0.760951989611575
KATNAL1	0.360141007207718	0.125247854530309	0.00380285160554575	0.994588964700772	0.184743666666667	0.520709741182581
SRRM4	0.359963599583608	0.12549181276789	0.380358229950951	0.300705577749487	0.168615666666667	0.443152920974238
ZNF75D	0.359921473126929	0.12549181276789	0.110714531524777	0.849357727620213	-0.208711222222221	0.408086593806833
SERBP1	0.359461692233432	0.126173402449558	-0.0155963389194733	0.982442849983933	-0.152115333333332	0.572359444665845
NKTR	0.359401886416388	0.126173402449558	0.0936044175049363	0.875932944246867	-0.0349129999999997	0.896186784012643
TNPO2	0.359385120360776	0.126173402449558	0.518745663928723	0.0903775728003269	-0.0177469999999982	0.953723188802355
ASB7	0.359154753388579	0.126489141361571	-0.00718177082997481	0.991025604847553	0.0321740000000014	0.89779682840784
MAZ	0.35879393608306	0.127221855469451	0.240380809615025	0.597862631330416	0.114735666666666	0.575589336285762
NLK	0.358488255473779	0.12778824492746	0.478361260093718	0.140664651993598	-0.395566666666666	0.0811348977551862
ASH1L	0.358470745764156	0.12778824492746	0.162549461656674	0.757614806491095	-0.049793999999999	0.8754328609757
FN3KRP	0.358468651498352	0.12778824492746	0.457794600457583	0.166671273245692	0.0311930000000001	0.903274690789156
RALGPS2	0.358401530695197	0.127823880493837	0.130816858468424	0.812271254601992	0.220539666666668	0.310277378475854
TRIM14	0.358295917823744	0.127907192710065	0.0763653497055311	0.902928663998623	-0.0599996666666663	0.788201431210286
ECT2	0.358175696574877	0.127987116855542	0.131804043027485	0.810671652076995	0.115056666666668	0.605491766505367
EDC3	0.358081115754224	0.128064704778766	-0.195714323161328	0.693267493630778	0.0968460000000014	0.657217907037306
ATXN7	0.358064481073661	0.128064704778766	0.119173186352251	0.836632262138615	0.0234773333333338	0.938187566801362
PHEX	0.357915749659481	0.128290057046090	-0.180338487631928	0.722985510661031	-0.0969459999999998	0.64069646762551
SPRY3	0.357897659484006	0.128290057046090	-0.111994030697692	0.847604542536677	0.242124333333334	0.340341227423177
U2AF2	0.357727837289536	0.128595422054425	0.225778583694115	0.629331969778772	0.0483060000000009	0.840247972976129
PLCD4	0.357700927207718	0.128595422054425	0.173796129375047	0.735311029057091	-0.141133666666667	0.614221661998915
KAT7	0.357361001318513	0.129071147545201	0.240164653353856	0.597971745721753	0.0313529999999998	0.902208543897795
DSP	0.357014645906336	0.129660896191902	0.373061683587094	0.316692858754573	-0.179713333333332	0.572207354314478
AHNAK	0.356950213521054	0.129660896191902	-0.0058996173838881	0.99251028338798	0.349846666666667	0.320225451789804
SAFB2	0.356948131728092	0.129660896191902	0.375693650725235	0.310953589377137	-0.0722006666666663	0.76564992393702
MAP4K5	0.356442423983969	0.130601370380239	0.0696990646122129	0.911167570018484	0.292150000000000	0.146938363443388
CNOT6L	0.356441169739204	0.130601370380239	0.114753843434708	0.844162087612813	0.0170410000000010	0.953217548324318
MYLK2	0.35638081766086	0.130601370380239	0.257263010598638	0.563116080558022	-0.0738849999999999	0.744485202271404
CSTF3	0.356220508174762	0.130718905802255	0.128729455311016	0.815407702581246	-0.0554683333333324	0.824700707442131
GLB1L3	0.356167258024821	0.130740492056584	0.219709567973894	0.641035290381005	0.102252333333334	0.665071722126052
CHIC2	0.355926133174766	0.131227279143989	0.00385615405068082	0.994588964700772	0.253542000000001	0.245948767014956
SELE	0.355905723353864	0.131227279143989	-0.180296200768746	0.723079097675399	0.0837190000000004	0.723478323564712
AGPS	0.355724337338413	0.131571699941586	0.0231755207986346	0.97535657852997	0.0638733333333346	0.78197285216323
IER2	0.355673350436699	0.131581352824498	-0.0894076919623392	0.883460564717377	0.138520666666668	0.482760288609588
TUBD1	0.355666974900855	0.131581352824498	-0.139786517400523	0.797957231657366	0.278928000000001	0.334220238753961
RPS3	0.355599125188498	0.131621294870554	0.301779854441383	0.463020287545295	-0.0367466666666667	0.887363245747406
FOSB	0.355431764745498	0.131920378820309	-0.40024930682347	0.263139454802378	-0.178249333333333	0.583036318848746
FUT2	0.355379214596934	0.131920378820309	0.371286206448805	0.31973535243631	-0.0460779999999993	0.866115937003813
CERCAM	0.355369891776709	0.131920378820309	0.425486912709123	0.215369960032963	0.524567333333334	0.030025317565228
MCM5	0.354923503655753	0.132870172652411	0.112968550018812	0.846616993271308	-0.0806043333333315	0.712601873916151
ITGA2	0.354611105891017	0.133430072247325	0.549510036025046	0.0608648191629941	0.806534333333333	0.0315936444843357
CASP3	0.354396853157878	0.133809166291857	0.141295818314571	0.796285336930078	-0.113455333333332	0.593008996011892
DIP2A	0.354322768216931	0.133809166291857	0.107502268587038	0.856954951691796	0.00736566666666813	0.983159241812861
ZNF443	0.354230299025754	0.133809166291857	-0.00441118808855108	0.99405344216434	-0.089035666666666	0.906093399285191
MAPK1	0.354229351710838	0.133809166291857	-0.0940263569717496	0.875355472414883	0.0881700000000013	0.6846855637534
PARD3	0.354187962126463	0.133809166291857	0.218189247438845	0.643548513668828	0.244480666666667	0.271872170281604
BNIP2	0.354104982642811	0.133809166291857	0.060036668958489	0.925677399480702	0.169087666666668	0.386947456618367
TNRC6C	0.354091464799613	0.133809166291857	0.291836521500385	0.485255752311035	0.144598666666668	0.657217907037306
WLS	0.35402318455911	0.133870817674449	0.363729564602188	0.335033160801809	-0.112726666666666	0.623988109816431
CMTM3	0.353922817252251	0.133975474665145	-0.0311299123671695	0.965118873711308	-0.197332999999999	0.483769337971167
PARD6G	0.353918792000394	0.133975474665145	-0.131614463492614	0.811042872465441	-0.104961333333333	0.761078552848883
TRIM28	0.353852198740166	0.133975474665145	0.213122765831901	0.655185554924368	0.0268066666666671	0.922148106708426
DIP2C	0.353793224098335	0.134064333421083	0.396155013638558	0.271439858675643	0.123963333333333	0.590841338570121
DNMT3A	0.353389423164921	0.134864355099337	0.168633395153229	0.746758286599055	-0.202493000000000	0.343729765439488
FAM69B	0.353170034618152	0.135291321104154	0.57267360479041	0.0443803345694495	0.117276000000000	0.55586869237591

HSD17B1	0.353080243800193	0.135369063301092	0.0591853694703292	0.927427607950847	-0.0360613333333331	0.902194685260117
HAS2	0.353030319541189	0.135417531761834	0.190167325817967	0.704208582824765	0.232313666666668	0.228394243234014
IBSP	0.352722905413108	0.135918545980398	-0.0467586616227459	0.944280538921282	0.0491180000000002	0.893706914393728
SUFU	0.352625309482478	0.136115717078429	0.101846268870995	0.864616357134757	0.0323886666666672	0.905413794391475
UPP2	0.352539308778395	0.136137376106152	0.0145711729000468	0.983444353656142	-0.0328909999999999	0.904507944803624
SAMD5	0.352538418913127	0.136137376106152	-0.137695252929937	0.801934376886443	-1.401857	0.0143541040572610
VMP1	0.352473630457317	0.136143863427219	-0.154540311760956	0.771310656070265	-0.0192466666666653	0.949173055617325
MMP3	0.352251067005498	0.136355726080166	0.253889155281548	0.567694846153007	0.0163586666666667	0.962620058410129
ADNP2	0.352249259685153	0.136355726080166	0.268991116484880	0.534063021353341	-0.171086666666667	0.36523677764789
ATF2	0.352188594115555	0.136451159054183	0.306176916166920	0.456406236471471	0.111121333333333	0.583699608015136
LRCH2	0.352136454908442	0.136523009982613	0.140749289390705	0.796714214272984	-0.0335999999999997	0.942298301774487
CYP24A1	0.352008447400634	0.136805613798541	0.270773186109645	0.529098041076523	-0.507991333333334	0.064805818136654
CREBBP	0.351919923544884	0.136978831418926	0.101463844314211	0.865856941328066	-0.0378403333333315	0.899912734474539
PSMD5	0.351617680910365	0.137528869556326	0.143291911781811	0.79287526984225	0.175847333333335	0.373594956320212
ZNF428	0.351159739420003	0.138146073118981	-0.0106180989835357	0.98774587825743	0.134591666666668	0.541939010010167
CDC42EP3	0.351138913504657	0.138146073118981	0.228112142783275	0.623935503350068	-0.0540443333333329	0.867354667762818
MAB21L1	0.351131288489326	0.138146073118981	-0.212885233943223	0.655695431812946	-0.236610666666666	0.231059485569270
PDLIM3	0.351118680780393	0.138146073118981	0.249126019093439	0.576190341561914	0.00093900000000004[illegible]	0.997144862160692
UBXN7	0.351109356791361	0.138146073118981	0.152381080582758	0.775863712880333	0.206728666666667	0.295467950792479
NKIRAS2	0.351039321151842	0.138196183273130	0.27105077909535	0.528536444071912	0.0232886666666669	0.930547487203917
P2RY10	0.350917599288831	0.138391179172244	-0.110673112959303	0.849359866036793	-0.0248003333333332	0.925805102015594
FLJ13197	0.350681054464238	0.138805311596342	0.429571843346595	0.208887609473370	-0.0888686666666652	0.881198673456728
EPC2	0.350586450349597	0.138805311596342	-0.0418686752356327	0.951989245458556	0.222869666666669	0.260015811789666
FAP	0.350565026059707	0.138805311596342	0.32229088864669	0.417538034812104	0.148331333333335	0.677647679541427
COPA	0.35048951826248	0.138878470454014	-0.0459872331622807	0.945364757579857	0.0565933333333343	0.804411624264825
DISP1	0.350473629695665	0.138878470454014	0.340892087518615	0.378272273903863	-0.548322666666666	0.0777629484027457
NUP188	0.350458091603234	0.138878470454014	0.154455214647804	0.771310656070265	-0.205066333333331	0.328243714258385
RAD54B	0.350315129178405	0.139061729457709	0.241473346711842	0.595717891213491	0.0410060000000011	0.890073383340154
GBE1	0.350244626198979	0.139187151245889	0.571618771035942	0.0443803345694495	-0.145709999999999	0.496323393705678
LARP6	0.350034515806923	0.139633043407335	0.317416498543923	0.431166148274203	0.189726000000001	0.350299411813049
C3orf17	0.349674075994987	0.140212900179343	-0.0146641472447919	0.983444353656142	-0.0713053333333321	0.748603801948204
ZNF519	0.349583531925797	0.140322994765906	0.320836003831295	0.421618886526906	-0.0638176666666662	0.772520347452753
CCNY	0.349288401695175	0.140832989375854	0.284884921681832	0.498546593922243	-0.100844666666666	0.633561720825521
HMG20A	0.349190315266113	0.140853084685271	0.192261289187359	0.700684773739383	-0.142096999999999	0.480601492957535
TRMT6	0.348978634151986	0.140981608131202	0.21248209039961	0.656785033957219	-0.194952333333333	0.32631478390653
PACSIN3	0.348884817594664	0.141136968508310	0.491784335399589	0.122707160872791	-0.150548000000000	0.483277436143082
CCDC97	0.348781831596298	0.141299072310875	0.269697253297587	0.531924063959685	-0.0384640000000002	0.899845504615556
BCAM	0.348564635484291	0.141710201392643	0.395383314701837	0.272692742937895	0.266395333333333	0.29986081041577
PRR16	0.348517283445356	0.141745168996786	-0.166715957195086	0.750443995356965	-3.99999999988703e-0[illegible]	0.999822702792133
SSBP4	0.348444550820134	0.141806617405171	0.460336275531666	0.162787294570657	-0.161742666666666	0.451528670919448
ALYREF	0.348349723537883	0.141901692361326	0.106357512580154	0.858284876027025	-0.306221666666664	0.144237446651127
SOAT1	0.347726222776713	0.143063070718800	0.346046916067317	0.366042223836959	0.0811766666666670	0.829384095613517
SYNDIG1	0.347399940076841	0.143629010858587	-0.0189849925021995	0.97832242527852	-0.0680339999999995	0.758341714501375
PLEKHF1	0.347270810371272	0.143707944297577	0.47744647389232	0.141622491285205	0.00775500000000055	0.98256318768706
ZNF331	0.34681288463536	0.144626383449650	-0.263360820276926	0.545874639137454	0.0065996666666671	0.983159241812861
B4GALT2	0.346741991420881	0.144647853626462	0.355478933682462	0.347343484863428	0.0755083333333349	0.752992371482229
LRRC66	0.346419273458174	0.145347002723160	0.247365475321821	0.579745218830252	-0.105948000000000	0.727809376593958
RUFY2	0.345942242808047	0.146032047481695	-0.128417363819851	0.816345918941366	0.295745333333335	0.136756149735032
GDNF	0.345936238400233	0.146032047481695	0.367785189892618	0.326542639565994	0.0547636666666671	0.812792750346224
LRRC38	0.34587981160637	0.146070859518748	-0.138314298073212	0.799981150504262	-0.132659333333333	0.527603023191717
TIAL1	0.345796856798305	0.146070859518748	0.336868845733838	0.388626001432611	0.0748490000000008	0.729912257977231
TUG1	0.345686643579131	0.146134121318583	0.0104501420715874	0.987849393233603	0.0621866666666675	0.777406710539424
CYP27B1	0.345653864151462	0.146134121318583	0.239017056400863	0.600032272579353	-0.124192333333333	0.622867915780328
HSD3B2	0.345647295306826	0.146134121318583	0.462687624447143	0.15879071503051	0.473632	0.107866708545887
KLF11	0.345614198495331	0.146134121318583	0.201399567546797	0.680054545720343	0.473076666666667	0.0473853084547341
ZBTB12	0.3455957691192	0.146134121318583	-0.0382683242544566	0.954545200586067	-0.112618333333333	0.59949012219089
HIST1H2AC	0.345573988345727	0.146134121318583	-0.267474410971916	0.536168282496512	-0.0274313333333324	0.919359825156123
ITPRIPL2	0.345504188295848	0.146164046969427	0.189684932077773	0.704928430617595	0.181795000000001	0.340052241399374
PID1	0.3453341233341	0.146394592693037	0.552034590346645	0.0595755126363067	0.0142236666666672	0.961509682688243
KIF3B	0.345116583610644	0.146717048500738	-0.148928024617402	0.783041584899534	0.0363240000000016	0.899614658619676
DSG1	0.345092200838078	0.146717048500738	0.124508851911874	0.824974271393593	0.0228026666666666	0.943250972379106
WASF2	0.345048402804707	0.146717048500738	0.0760369639839475	0.903428608807779	0.0817716666666673	0.727809376593958
TAS2R14	0.345038716224692	0.146717048500738	0.480404297321563	0.137946523725028	-0.00861433333333298	0.980483056915922
IRF2	0.345023490160126	0.146717048500738	0.443521269878219	0.1843534401377	0.162741000000001	0.467798588118316
TRAF5	0.34493825953144	0.146760500791237	0.202000729359986	0.680054545720343	0.00128166666666718	0.996579657157409
DVL2	0.344922463234438	0.146760500791237	0.183498701011419	0.718067440349544	-0.200059666666665	0.328554791536866
ZNF362	0.344918078540317	0.146760500791237	0.124499459926219	0.824974271393593	0.164416000000001	0.415364554474667
GMPS	0.344908807058342	0.146760500791237	0.154051766981929	0.772253934458676	0.117777333333334	0.600146208272329
KIN	0.34477116142755	0.147016846630996	0.169301451399369	0.744676064883446	0.281283666666667	0.261668636036981
SNAPC2	0.344577221182739	0.147438371202596	0.294710918232669	0.478800228795472	-0.0703173333333326	0.754764831450489
STK38	0.344466002309215	0.147623673164783	0.133897655546116	0.808567812988011	0.122517000000001	0.6846855637534
PAF1	0.344464175079633	0.147623673164783	0.183908612995083	0.718067440349544	0.0354083333333346	0.902290576370005
ABCE1	0.344388172257779	0.147746263980143	-0.0222355585477733	0.97536722461972	-0.246433666666665	0.365126455342703
DDX17	0.344313654990976	0.147846224249054	0.0196701168157267	0.97763669370967	-0.0735599999999995	0.73097308185803
TCF4	0.343972705302269	0.148351451545371	0.255391296708097	0.566022340784541	-0.275177999999999	0.248826844950506
AFAP1L1	0.343961094515906	0.148351451545371	0.390644098714387	0.282005640075858	-0.128395666666666	0.631511678860383
NFAT5	0.343893466636347	0.148351451545371	0.0180371229514177	0.979613786029786	-0.166091333333333	0.428358173426632
SLC19A1	0.343857579003701	0.148384150376108	0.48268222140252	0.135560525489292	-0.159425999999999	0.470065542518108
ANTXR1	0.343742965255811	0.148648543804718	0.358308415266595	0.343186459025557	-0.0752853333333321	0.760951989611575
WDPCP	0.343595444871574	0.148841031368746	-0.00929847744234619	0.98909166696601	-0.092392999999999	0.712601873916151

KDELC2	0.34348320553868	0.149027432347936	-0.164387293189877	0.754189116283047	0.454693666666668	0.0372437947587881
SPOCK2	0.343356843284712	0.149238524753554	0.0736097594321372	0.905838142287595	-0.0474069999999998	0.842409429977709
PLTP	0.343345081775667	0.149238524753554	-0.258094328810287	0.561580373803451	-0.182272666666666	0.357189933873914
UBA2	0.34327197699939	0.149312801683032	0.147418008591025	0.785497496933202	0.119823333333334	0.575195976222091
MCF2L2	0.343270565342109	0.149312801683032	0.240286361451321	0.597862631330416	0.035172666666667	0.899614658619676
ERF	0.343010843071121	0.149597771836218	-0.0445951616056108	0.947920610570169	-0.0321353333333334	0.900101096868846
COL6A3	0.342972383631509	0.149611543990051	0.274851375581486	0.520095539214775	-0.111081000000000	0.592716584553539
ARHGAP32	0.342819689354213	0.149772349335459	0.399171729045089	0.265929249534649	0.0921630000000014	0.7108031545527
ZBTB10	0.342743479648385	0.149899689060216	0.278559235099102	0.511025440770733	-0.0093073333333329	0.979527173532206
SGCE	0.342692584942606	0.14990005911232	0.151279539613832	0.777604486471648	0.161385000000001	0.401558725985789
KIRREL	0.342627880024508	0.149976884638037	0.250423893184562	0.573888735446956	-0.139532666666665	0.656222957956058
IKZF5	0.342495907082041	0.150222639896089	0.237361489935377	0.603170893739822	-0.574600666666666	0.0234650314672839
HIST1H1E	0.342318911714877	0.150464736496511	0.436065024729102	0.196057392976856	0.0523500000000004	0.840657236939036
CHAF1A	0.342230435475692	0.150647136021576	0.152194438596307	0.775863712880333	-0.157577999999999	0.447114454029064
AEBP2	0.34203670961858	0.151150605031738	0.390363887563768	0.282383744168943	-0.288057999999999	0.167526000762082
RPA1	0.341468529527446	0.152249830085910	-0.0995789091447285	0.867603998426271	-0.0110729999999995	0.97201219963332
CYTH1	0.341427287248339	0.152249830085910	0.182670624479862	0.71862020095107	-0.333409666666666	0.124833742621232
KCNJ13	0.341367671467900	0.152249830085910	-0.102645703597447	0.862817525027433	0.0569473333333336	0.822657709336468
ESYT2	0.341350935693579	0.152249830085910	-0.0385266048678048	0.954545200586067	0.214221333333334	0.301793253300179
ITGB3	0.341330659505538	0.152249830085910	-0.240911184450863	0.596648444172109	0.0907696666666673	0.710483929190267
JAM3	0.341117707967496	0.152730742513264	-0.1653315096898	0.752543829181	-0.0763703333333329	0.742738366366583
HNRNPU-AS1	0.34104518747276	0.152875385350008	0.21501583571636	0.651257878577277	-0.231230999999999	0.337994438406310
DLC1	0.340931050035509	0.153048794606314	0.482093097162331	0.135560525489292	-0.0377323333333333	0.87796421789874
ARCN1	0.340889059627207	0.153048794606314	-0.0344300609093933	0.959404955370931	0.0470873333333343	0.840251723747548
FNDC1	0.340779620737872	0.153048794606314	0.107048722937463	0.8576027597496	0.279690000000001	0.344663423870532
B4GALT6	0.340714348596952	0.153048794606314	0.453027442130914	0.173768739912551	0.288658333333334	0.288747111194017
ATM	0.340710138315239	0.153048794606314	-0.221428145913988	0.637715957775489	-0.0291126666666664	0.921546617328678
MGA	0.3406313106923	0.153048794606314	-0.117883206189371	0.838686136084666	-0.111451333333332	0.696217349388802
IFFO2	0.340629321401303	0.153048794606314	0.317264248345637	0.431301545026907	-0.374683666666666	0.120319574336004
EDNRB	0.340623387831504	0.153048794606314	0.225604788795299	0.629710977741725	-0.0870016666666662	0.703878203604573
MDM4	0.340512534101407	0.153102060340524	0.263730670436573	0.545144326127723	0.203411666666669	0.344586016841321
SMC3	0.340305548617904	0.153103943264842	-0.0779943756479365	0.899705053748212	0.180721333333334	0.343572153228906
DBP	0.34029811609419	0.153103943264842	0.533204051262088	0.0759909410633651	-0.0524279999999985	0.822657709336468
TMEM131	0.34025673914779	0.153103943264842	0.333425802376037	0.396821740334178	0.182502000000001	0.403606718433947
SEC1P	0.34014965356399	0.153240054631406	0.119459488861121	0.836013196419542	0.0580776666666674	0.794486775271372
PHLDB3	0.340005445213579	0.153456701350364	0.214603839328504	0.652098715879581	-0.102479666666666	0.645689070400576
KCTD15	0.339893757128770	0.153652169909843	-0.229260724810823	0.620409044581731	0.00598200000000029	0.985884500368235
F2RL2	0.339844428479326	0.153652169909843	0.178122846124676	0.727939088352802	-0.00047033333333313	0.998309716374356
FAM222B	0.339397575532789	0.154493425984459	0.311147247407051	0.443419531318142	0.146031333333334	0.5293518047673
PKDREJ	0.339332214013735	0.154495518990287	0.00813559294105519	0.990248011892148	-0.0940156666666658	0.674028958837018
DOCK1	0.339325110684684	0.154495518990287	0.333226177342473	0.396894500849691	0.0194980000000002	0.96038769215064
MTOR	0.339290108733641	0.154511861551480	-0.0602297807081898	0.925649366494154	-0.00613999999999899	0.9863022112014
ZNF45	0.339239594489508	0.154511861551480	-0.0901431124805335	0.882249601181978	0.116691666666668	0.643792824295256
FBN2	0.33923736578357	0.154511861551480	0.0391686719658356	0.953905313456582	0.471787666666668	0.0395176104364946
EGR2	0.3389708785473	0.155061021238989	-0.233211323727861	0.611287729493682	-0.182535333333332	0.417579137533511
CASP8	0.338944603242655	0.155068353684259	-0.0691120125192427	0.911922995899228	-0.0128343333333325	0.96438709754972
TOP2B	0.338871081583866	0.155069099399248	-0.178972574493009	0.725831949422059	0.143819333333336	0.550660741372463
B4GALNT3	0.338807067121969	0.155069099399248	0.628267441968123	0.0220882270537442	-0.330701333333332	0.0894613209101226
SETD5	0.338682588698436	0.155069099399248	0.101693647743405	0.865162779486656	0.0943376666666682	0.6751260712201
EML4	0.33852666263146	0.155205454025086	0.326269481402109	0.409206882509236	0.0257823333333339	0.921546617328678
CSNK1D	0.338374716324094	0.155256513845752	0.147286526822148	0.78561060123541	-0.0615413333333322	0.789377975672973
CD97	0.338366251069736	0.155256513845752	0.220509264128004	0.639292937038778	-0.0588539999999995	0.874908057954497
WAC	0.3383545227038	0.155256513845752	0.151273103691667	0.777604486471648	0.113601333333334	0.630389991874517
C4orf32	0.338266599062941	0.155357112971882	0.470684307120494	0.148668281829074	-0.0440406666666665	0.870662337224757
LPCAT1	0.338232189567837	0.155357112971882	-0.129193845470027	0.814793489052246	-0.00216233333333177	0.99529569362957
SSRP1	0.338067984668162	0.155711444568134	-0.107939216541603	0.856008265865111	-0.0808586666666646	0.727809376593958
COL5A1	0.337346586195906	0.157117127646178	0.232689305489645	0.61200913457472	0.0545463333333343	0.81725103791607
TLK2	0.337271981886437	0.157201211866418	0.261533194160356	0.551468938712409	0.104228666666667	0.638289738241453
BMS1	0.337020189730186	0.15770623585735	0.0572190889217499	0.93071299016553	-0.166619666666665	0.479014333115247
KIF3A	0.336989237214346	0.15770623585735	0.210800339083517	0.66147186925907	0.123027333333334	0.578893615511221
ELAVL3	0.336958582492734	0.157728180262924	-0.219268101213	0.641531120405902	-0.119161	0.562399033626288
MMS19	0.336829461626704	0.158051754212012	0.182484445073376	0.719419078472232	-0.187061999999999	0.324437274165154
GYLTL1B	0.336804951181480	0.158054931332587	0.421669684867054	0.222348587392621	-0.120652333333332	0.592716584553539
NAB2	0.336718001819571	0.158195253577357	0.100708678041536	0.86628046156792	-0.0164693333333329	0.964241302457077
RUNX1T1	0.336701012443881	0.158195253577357	0.372646121869093	0.317205263174137	-0.323293666666666	0.121660357743656
NOVA2	0.336665371335288	0.158195253577357	0.293654965715895	0.480614908214975	-0.0110839999999993	0.974484855799046
ELF1	0.336636923792386	0.158210633124736	-0.494517409614813	0.117780147190022	-0.275456333333333	0.262910099772857
LINC00523	0.336508669493519	0.158388580390476	0.203734892948717	0.676117692782917	-0.0131913333333330	0.97983049046704
ZSWIM4	0.336485112892553	0.158389057546418	0.213128221101560	0.655185554924368	0.0189120000000014	0.945904449997854
BCL11B	0.336383140981324	0.158558495374121	0.165890992604126	0.751869806279813	-0.0955766666666655	0.735492009132547
NAV3	0.336352778353239	0.158578022288567	0.333003737679391	0.397658808533403	-0.446033666666667	0.0353807205776334
KRT35	0.336209542457889	0.158804700378771	-0.091807368220444	0.879153312143409	0.0843746666666674	0.712420423900096
CTCF	0.336097567201231	0.158933701572501	0.146056567982655	0.787402233709509	0.0307386666666679	0.903788355106891
PRKCI	0.33586419269537	0.159283198450136	-0.0616810305740136	0.923246185283376	-0.0456463333333318	0.867254646889164
LTBP2	0.335830202446039	0.159283198450136	-0.201803566770987	0.680054545720343	0.144208666666667	0.518836392563847
GALNT10	0.335820895539648	0.159283198450136	0.0373340216309929	0.955937551268326	-0.128803666666666	0.591142517306808
KCTD4	0.335531629444373	0.159765014750852	-0.0037187424964299	0.994588964700772	-0.183536333333333	0.333881200421942
BHLHE40	0.335525091458749	0.159765014750852	0.0702863527178613	0.910394157189308	-0.174459333333332	0.460374458843217
MCM8	0.335456831552036	0.159882702972512	0.1197477860533	0.835531661413366	-0.441137666666665	0.0351167468854759
H1F0	0.335221241552613	0.160308359830670	0.451559507675422	0.176663460992485	-0.0287513333333323	0.910190419163007

NFIA	0.335209975822312	0.160308359830670	-0.0763076815828672	0.903025028995418	0.0449796666666671	0.883206615493913
GATA3	0.335067388189522	0.160561578898578	0.513696959658874	0.0962522730025878	-0.127334666666666	0.58391025729515
PTGIS	0.335022575280749	0.160561578898578	0.045618421250562	0.945663463098983	-0.234364999999999	0.219457597945103
N4BP2	0.334854154242121	0.160830986233534	0.196766528790354	0.690061983084583	0.431736666666668	0.0758522568433912
CARD6	0.334840517720884	0.160830986233534	0.111362518339103	0.848968422336654	-0.131939999999999	0.528409919089401
FKBP2	0.33462273217612	0.161267646614100	-0.199793413215344	0.682777037002814	-0.0791363333333321	0.771373465365664
MFAP2	0.334159370971046	0.162120549018632	-0.134918278988096	0.807867938002244	0.094607666666667	0.669530887411007
UBTF	0.334140986203411	0.162120549018632	0.0103612061891571	0.988029495198616	0.0450983333333349	0.861945621270498
EHD2	0.334104379686948	0.162162832179723	0.187089334072951	0.709833349594307	0.285176666666668	0.28322823752779
ZNF503-AS2	0.333927852796466	0.162498133395052	0.567067551409212	0.0470952455251712	-0.053175	0.865655368707012
PER2	0.333822446804817	0.162683654546101	0.41016829828924	0.246706088283721	-0.00955599999999995б	0.974895360062947
CBX5	0.333651038634548	0.162943357068602	-0.00513218533342298	0.99251028338798	-0.131199999999998	0.551352991056569
FRS2	0.333647422640634	0.162943357068602	0.183538450296223	0.718067440349544	0.343822000000001	0.106191896591867
NAA50	0.333345522296259	0.163472359647758	0.152726712383232	0.775138534286213	-0.502879999999999	0.0257919352862336
PRRC2B	0.333340793002781	0.163472359647758	0.0302040665028148	0.966272853760157	0.0395314444444454	0.872566183743608
ZNF280B	0.333173315334655	0.163700165326081	0.0845703369057571	0.891600137914975	0.048110000000001	0.83616423402744
DIS3	0.333153561241536	0.163700165326081	-0.106351462043563	0.858284876027025	-0.082995999999999	0.710456701900109
SERP1	0.333051625835684	0.163804543218723	0.349010625385589	0.360528943904524	-0.138271333333332	0.484317289991301
REST	0.33302631843892	0.163812158814858	0.0255793960367721	0.97204937337922	-0.119121999999999	0.601896528443988
POU6F1	0.332913654901651	0.163996688451620	0.224554075897705	0.632644967401566	0.0764690000000009	0.75106495230718
AMOT	0.332908984208664	0.163996688451620	-0.038888207290588	0.954165422885126	0.187699666666668	0.372440180394709
SMCHD1	0.332847737315568	0.164014105875384	-0.0345092971955931	0.959404955370931	-0.137290666666664	0.490929966167076
CRYBA4	0.332802904314012	0.164083238822683	0.230257549453947	0.618707227156037	-0.139988999999999	0.554188413823318
C1orf200	0.332729472696268	0.164202051267498	0.234786641233603	0.607957283325678	-0.183953666666666	0.484125153753832
DCAF6	0.33271946292185	0.164202051267498	0.158715499178480	0.765791046741822	0.215136666666668	0.281579610467498
CCDC17	0.332677189394937	0.16426325068478	-0.237658757048714	0.602664863375033	-0.140828000000000	0.570550813984136
COG1	0.332416066808439	0.164798408097335	0.421744397776667	0.222348587392621	-0.0347593333333327	0.895093529187836
CACNA1S	0.332303307435376	0.164991189804489	0.0847758459568788	0.891309254245872	-0.085202333333333	0.721039266334933
TP53	0.332201048728061	0.165117576877661	-0.0863538634693333	0.88794718020464	-0.378988666666666	0.0906212303943537
ZBTB2	0.332127276438934	0.165238175189831	0.311777299349525	0.441314096241515	-0.0426826666666661	0.87443027347283
TMEM200A	0.331922679833723	0.165279248505394	0.449741929306432	0.177277037186644	-0.103520333333332	0.76276832167361
MAN1A2	0.331839564148643	0.165470195822331	-0.495439891339471	0.116831680799555	0.101051666666667	0.626750668682093
ZNF614	0.331660393196224	0.165821867070994	-0.171206701632239	0.739841869018052	0.138328666666667	0.713523955539733
SERHL2	0.331623712640047	0.165866278109396	0.19397908538978	0.696962749474912	0.288577000000001	0.307774837877941
RHOA	0.331538455924118	0.165879534413178	-0.087534835916245	0.886302037299497	0.0386700000000003	0.873663396186202
AK7	0.331515565089464	0.165879534413178	-0.137430491383165	0.802039543563725	0.358420666666667	0.0949909994193578
CCDC9	0.331436236760553	0.165978251698401	0.180059935443943	0.723716289903531	-0.0771956666666655	0.732554472781933
ZNF358	0.331407407303037	0.165978251698401	0.411480811500964	0.243528138193926	0.201781666666668	0.307927332709227
DCN	0.330863268306205	0.167204343149162	0.363136828591274	0.336332757540532	0.071883666666667	0.80845521378406
PDLIM2	0.33078794890295	0.167228823201215	0.51042307893932	0.0998179014114546	-0.0064363333333321З	0.985544323496696
RANBP2	0.330671663409335	0.167455742225053	-0.131621224898122	0.811042872465441	0.160191000000001	0.428953946992621
NOS1AP	0.330567707566175	0.167643627790574	0.351510594331344	0.355583906553889	-0.114335999999999	0.616961986237355
LZTS2	0.330344922098658	0.168074020341944	0.386144750782637	0.289470192870154	-0.0314976666666662	0.918192039123086
ADAM19	0.330250277471000	0.168074020341944	0.142240261451862	0.79450771779567	0.0629590000000006	0.780732284998362
CASS4	0.330242072042061	0.168074020341944	0.104134650654214	0.861362476037143	0.0259083333333332	0.935223865987462
SLC20A2	0.330221293544236	0.168074020341944	0.135625822320373	0.806637220506547	-0.0393823333333327	0.883478349705756
DRD1	0.330191293928448	0.168074020341944	0.060977306062079	0.924888576360497	-0.120347666666666	0.554188413823318
EIF4A3	0.330012958197846	0.16838757276012	0.128358119438569	0.816429366704753	-0.104358333333332	0.668862273626329
GPR52	0.329901485795102	0.168552874021785	0.0127609830281534	0.985156239185327	-0.0425723333333327	0.857473694247732
PLK1S1	0.32979097250997	0.168704937371774	0.328483503116642	0.406864543297514	-0.365224999999999	0.333881200421942
DAZL	0.329776070064223	0.168704937371774	0.173315217787424	0.736740209571778	-0.0579669999999996	0.85900837597792
HIPK3	0.329716131977730	0.168704937371774	0.0300940453349478	0.966396286514145	0.620828666666668	0.0211138722972481
CBX8	0.329696703397886	0.168704937371774	0.492752287255255	0.121594840868068	-0.012007999999999	0.9681321732097
PMP22	0.329639575082448	0.168704937371774	0.249271733111822	0.576018809235522	0.423343666666667	0.0577241178957641
MORC1	0.329638132796257	0.168704937371774	-0.145773261750093	0.787804113309913	-0.0442763333333328	0.868340672756718
FGF14	0.329615974893293	0.168704937371774	-0.087127811759462	0.887055939766881	0.00187500000000035	0.995621063297892
POLR3A	0.329608130200265	0.168704937371774	0.0866189691615885	0.887493600482322	-0.00305066666666624	0.993756838831997
JDP2	0.329404053905535	0.16921592028786	0.0244666435524151	0.973562415846915	-0.169076666666666	0.462444913819465
JRKL	0.329288973474652	0.169442164116187	-0.0118368689608158	0.986418801521542	-0.147455999999999	0.458006502146897
ZNF543	0.329188398314623	0.169567949024438	0.00321497220154696	0.994730189431381	0.217350000000001	0.446926946255299
TAF15	0.329139790710046	0.169614552473637	0.163388355877025	0.755593196913251	-0.716672999999999	0.035274715818792
MSANTD4	0.329123937684560	0.169614552473637	-0.286880313449581	0.494604746861742	0.0302840000000016	0.910190419163007
MYH11	0.329024438108607	0.169754158366434	-0.120842385709284	0.833217514204468	-0.111545666666667	0.721236226686999
ZNF680	0.329013760954742	0.169754158366434	0.032551717530868	0.961790688145	0.315618000000001	0.162264423966844
OSBPL8	0.328665736253972	0.17066041233134	0.103149771423348	0.862401456058756	0.0681853333333339	0.769217868422514
SETD4	0.328637900591472	0.170678518030613	0.402078065690573	0.260476951140907	0.0376573333333341	0.900467460403227
EVX1	0.328545129045484	0.170869618199708	0.336053248028000	0.389476012645306	-0.219469999999999	0.346439305749204
SLC1A2	0.328376579791717	0.171164146872516	0.168714450873246	0.746469057428344	-0.562850666666666	0.0647596980686847
CCDC62	0.328318829058203	0.171181277972922	-0.100401964909775	0.866570192459381	0.0514060000000005	0.851568943534661
PLEKHG2	0.328174524696452	0.171387472803140	-0.202212905344606	0.679731500360009	0.088996333333334	0.74499160363205
GORAB	0.327963366793256	0.171928279557339	0.0818661013240872	0.89548747312664	0.289107000000001	0.160783308783403
MIA3	0.327902292681091	0.171982774615029	0.108765066842009	0.853552381097052	0.0622096666666674	0.801063448666597
INADL	0.327729432446791	0.172400552526843	0.284090114041682	0.500557068353025	0.197254666666667	0.46297633209186
NSL1	0.327638922208771	0.172576924430945	-0.00265374778560325	0.99580901762095	0.273957333333334	0.156315769387234
POU2F1	0.327630970191237	0.172576924430945	0.274359310611145	0.520772283174073	-0.00105633333333367	0.997098037104877
CCDC24	0.327543374701289	0.172646179794421	0.188700204241925	0.707361603786947	0.0915260000000012	0.690826917301738
PAPOLG	0.327410866828509	0.172933832345376	0.0480469415606557	0.94314236584312	0.164375666666668	0.483840860674189
LINC00597	0.327369391346228	0.172997038163856	0.0547726282264502	0.934331742930062	0.216257	0.443378989641463
TPM4	0.327321137287324	0.173082083829157	-0.117645417679069	0.83940078178718	-0.00994166666666650ϐ	0.978504228748436
ZACN	0.327260073765481	0.173082083829157	0.247400479976233	0.579745218830252	-0.105040666666666	0.686147984639302

ARNT	0.327116861545084	0.173389026970973	-0.0393244438140973	0.953905313456582	-0.087035666666666	0.711082533709514
ATAD5	0.327078926866952	0.173440925693788	-0.183158724446669	0.718067440349544	0.0264036666666673	0.945904449997854
CSPG4P5	0.326992414149144	0.173651545658592	-0.00562491360461929	0.99251028338798	0.00266566666666706	0.993756838831997
KCTD3	0.326809529306104	0.174162029801561	0.312000307932774	0.440789236061843	0.208824333333336	0.284483455012453
LOC339524	0.326724047734058	0.174162029801561	0.103436955176429	0.861943478503987	0.0200291111111121	0.94441651434893
KIFAP3	0.32664512761278	0.174168989399594	-0.0827287637615286	0.893753725133293	0.342852000000001	0.131549958663763
RPL23AP32	0.326401203966079	0.174795448910406	0.526447389068687	0.0838370074507502	0.123619333333334	0.581570366864416
LOXL2	0.326278445948472	0.175054420942903	0.369092045656272	0.323487930700643	0.220540333333334	0.318538641774025
EMILIN2	0.325963407221414	0.175668276967558	-0.0323012127082405	0.962296733304814	-0.197916666666666	0.420804920248363
GPS1	0.325694356672309	0.17618403931812	0.250552642036561	0.573763059271745	-0.112599333333333	0.580149967309238
NBEAL1	0.325616844618532	0.176266456865035	0.262110710207478	0.549752906834892	0.120523333333335	0.617967324583825
LRRC37BP1	0.325603731213849	0.176266456865035	0.0862520690998236	0.88794718020464	0.158777666666669	0.48608244495005
FRY	0.325315646071091	0.176987763450508	0.419442103080829	0.228997998833993	0.208758000000001	0.343143699851351
RBM42	0.325204828302358	0.177224852017419	-0.0209543439556464	0.976125137187145	-0.0162039999999991	0.957164262608967
SERPINH1	0.32517466873525	0.177252386725416	0.0976797101305682	0.86925341204377	-0.0309956666666661	0.915761046438965
PTPN23	0.324959635948573	0.177734048671540	0.276594948143061	0.516955031095084	0.0852603333333348	0.688296747794568
TM4SF20	0.324875971853663	0.177881750151654	0.255162530155673	0.566363733990286	-0.123049333333333	0.562674320626673
C11orf49	0.324833135970759	0.177951629705198	0.270934340748244	0.52859316924857	-0.096588999999999	0.655063476133482
ZNF207	0.324496688342900	0.178636537317619	0.372889095184549	0.317134467774557	-0.0345886666666648	0.890094615232462
ZNF572	0.324473607804213	0.178640842153941	0.0256947089753229	0.97179002490987	0.218967666666667	0.326398785133011
HSPA5	0.324448144923546	0.178653118207819	-0.235010569314474	0.60755186112057	-0.0763199999999984	0.724603790871296
GANAB	0.324393561781239	0.178696655911175	-0.0611893172344094	0.924604287084523	0.0476466666666679	0.838927397112197
UBAP2L	0.324055546760667	0.179601594247684	-0.0533264081720071	0.936629055455007	0.0064860000000009	0.987499363290182
HIST2H2BE	0.323882031111543	0.179782218245222	0.0158653284621441	0.982102356159698	-0.0411516666666656	0.879939647520798
TFAM	0.323861081178372	0.179782218245222	-0.119929903123834	0.835280975936648	0.0152786666666678	0.955763320558376
FAM149A	0.323742123854815	0.180021930621737	0.222365864588317	0.636287617796138	-0.0132489999999998	0.966813448118178
LINC00337	0.323734547402076	0.180021930621737	0.166411495551907	0.75130598980695	-0.142126666666667	0.492720036901389
HBS1L	0.323580371486967	0.18032091564501	0.196821869129620	0.690061983084583	0.0555346666666682	0.812792750346224
FUS	0.323517680748806	0.180373062223738	-0.0451915940886977	0.946577404106596	-0.202689999999999	0.350434342294005
PTGFRN	0.323497648121405	0.180373062223738	0.313618195606606	0.438046311563112	0.0296746666666666	0.923019543204612
TMPRSS3	0.323447068090386	0.180373062223738	0.550605232995329	0.0603608035588276	0.0367643333333344	0.903759271170814
DGKD	0.323411602085894	0.180373062223738	0.113214884497072	0.845978328141518	0.113707333333335	0.591142517306808
ZNF805	0.323368375135876	0.180373062223738	0.0749530041200801	0.904466414650249	0.172382666666668	0.48712540376516
TLE1	0.32336212709742	0.180373062223738	0.268228380960042	0.53532907159569	0.0339333333333341	0.898979600726666
BRSK1	0.323361202787268	0.180373062223738	0.448563523961234	0.177860736739947	-0.0228726666666663	0.947020470290463
FUT11	0.323301095608479	0.180447124888071	0.372313075148863	0.318024489042558	-0.185198999999999	0.461494497197158
PRICKLE1	0.32328304069598	0.180447124888071	-0.220963508688615	0.638522059865506	-0.100850333333332	0.704417585200123
CABP7	0.323115064949594	0.180939079008098	0.302737761474332	0.462105017205825	-0.230605666666666	0.320225451789804
BMP6	0.32285840315222	0.181512686032052	0.392827929039153	0.278297610043666	-0.720972333333333	0.0098546599387472
DYRK1A	0.322735513883891	0.181781715448072	0.0475788572852238	0.943718457020225	-0.243282999999999	0.305101612380486
TAS2R13	0.322682050711534	0.181889402575607	0.0661112941851072	0.916792605561869	-0.0541103333333325	0.832957272763895
ARID4B	0.322514994460672	0.182235392712084	0.060460274297448	0.925565513580637	0.133846333333334	0.514889931507188
VTI1A	0.32245097391289	0.182379071426783	0.176768642725342	0.729837424743184	-0.116053999999999	0.583036318848746
ZNF445	0.322110652259897	0.182721720424423	0.183144067506682	0.718067440349544	-0.117826333333332	0.575837271491686
RPL32	0.322091770946977	0.182721720424423	0.171780256484025	0.739223710318873	-0.330498666666666	0.100026144425143
IRX3	0.322072683899725	0.182721720424423	0.573867136762138	0.0442317804409017	-0.306440666666665	0.134584461343945
C19orf47	0.322006485399159	0.182721720424423	0.118033956696490	0.838500126709059	0.090589333333335	0.684995039710994
GLRA2	0.321944577473846	0.182721720424423	0.267738221949538	0.53559545997875	-0.0361389999999994	0.904507944803624
SRSF3	0.321933015910764	0.182721720424423	0.0194591175437929	0.97779981827904	-0.0399866666666656	0.870662337224757
CAMTA2	0.321921435328927	0.182721720424423	0.136028691557924	0.805641810268282	-0.105365333333333	0.629657237462109
MXD1	0.321877843728112	0.182721720424423	0.0344130367061985	0.959404955370931	-0.000694999999999858	0.997953056544486
YES1	0.321860566360503	0.182721720424423	0.0155056570454073	0.98256069835494	0.147884666666668	0.4736509731531
NOG	0.321764453753939	0.18284403001976	-0.0947612230267504	0.873818349270144	-1.03201700000000	0.125393652266973
SNRNP70	0.321738247072165	0.18284403001976	0.194274853440823	0.696504707499023	-0.0170189999999989	0.950564200758787
FOXK2	0.32138958874087	0.183795449963979	0.237748218332721	0.602639741374674	0.165823000000001	0.409905616261873
PRM2	0.321357429169174	0.183832148319412	0.262082589581687	0.549752906834892	0.0325990000000013	0.900899806056542
ASPRV1	0.321131381484735	0.184165471081052	0.0315446876262729	0.963990276384993	0.0139849999999998	0.963383328073994
GNB4	0.320834604302568	0.184818068557498	-0.344239329852834	0.371106177422032	0.264516000000001	0.293015200389829
TNRC6B	0.320611080353446	0.185243821783514	0.145046006239094	0.789126578958976	0.172435333333334	0.468473414001593
HDGFRP2	0.320597873576152	0.185243821783514	0.28803008304377	0.491376246980382	0.0318843333333332	0.91131019859749
CAMK1G	0.320580945091161	0.185243821783514	0.197932472686974	0.687681948030762	-0.0453753333333335	0.879265091272724
PTHLH	0.320498827787776	0.185379160376085	0.0631741276621754	0.921359867071909	0.00011800000000041	0.999722166207899
RPS4X	0.320408727947767	0.185548673222231	-0.137496348781184	0.802039543563725	-0.137289999999999	0.501881126763681
ITGAV	0.320385518383215	0.185548673222231	0.137447728973972	0.802039543563725	0.270097666666667	0.188232886284507
SRCIN1	0.320296661586697	0.185707421391526	0.113154524280141	0.846173791956369	-0.133985999999999	0.634105753368746
KIF26B	0.320215192650377	0.185748379088795	0.306429543063168	0.455683898979353	-0.271591666666667	0.342476110074442
CDK12	0.320057947839251	0.185770229012258	0.0919643709008175	0.879153312143409	-0.162905999999999	0.47453552474236
ASPA	0.320044478742792	0.185770229012258	0.296568232134857	0.476296748214996	0.0914876666666672	0.673995132867941
YAP1	0.319930499588440	0.185945615217252	-0.00864002915919644	0.98946753270812	0.157927666666668	0.441730896500427
TMTC1	0.319743235599263	0.186381789459866	0.243972336309543	0.589307560917581	0.402141666666668	0.0928571414757748
SEPT5-GP1BB	0.31963633665196	0.186447069912715	0.242894040984033	0.592178030679976	-0.120311416666667	0.575589336285762
SEMA4G	0.319296020650008	0.187383869202056	0.676436003066729	0.00816048914572686	-0.311895333333333	0.390195381635312
BAI1	0.319279374830803	0.187383869202056	0.268925545200714	0.534079394978669	-0.191474999999999	0.362587998731204
NCOR1	0.319125610343255	0.187791095468958	0.167457566238168	0.748960978799932	0.102227333333334	0.635069698575895
LIN9	0.319076881390930	0.187791095468958	0.207288424587427	0.66888525756447	0.111296000000001	0.670071773006358
CREBZF	0.319027713881376	0.187855771101463	0.291043558528527	0.486880079690803	-0.0744756666666655	0.766132481270445
HOXB6	0.318994942214486	0.187855771101463	0.0254921465169720	0.972182886218393	0.311517666666667	0.112146551360288
QRICH1	0.318826353829386	0.188292444248948	0.185738827252050	0.714043644840406	-0.245524666666666	0.206187117033303
COL1A2	0.318691558409532	0.188665505794964	0.163419001326394	0.755496860446204	-0.0184563333333326	0.954180927004992
R3HCC1L	0.318672394958183	0.188665505794964	-0.0150013599072508	0.982921609770846	-0.108107000000000	0.674780899769326

ZNF292	0.318545721643896	0.188899408075159	0.346320632888211	0.365962081836316	0.0325146666666676	0.9061074113295
PDZD4	0.318410777831908	0.189221379948315	-0.104370912380052	0.861121906453898	0.0827926666666675	0.762118416653633
VGF	0.318346181773145	0.18929966923629	0.155131220719403	0.77066107821265	-0.130890666666667	0.546219696447415
OCLM	0.318242427281593	0.189513958146543	-0.0105691674693884	0.98774587825743	0.114795666666667	0.662828468002357
ACBD6	0.318088237138397	0.189830829408251	0.252201435291978	0.570465803488487	0.023652000000001	0.93278021276477
CPB1	0.317933913666799	0.190221684837789	0.161750324427894	0.758930446817487	0.241905666666668	0.458006502146897
ROCK2	0.317863752221831	0.190392972816580	0.0151213873248311	0.982921609770846	-0.167921666666665	0.462956926523536
PRPF8	0.317836896113830	0.190413479616217	-0.0112902920711788	0.98731277141449	0.0168366666666682	0.952916086133035
EEF2	0.317803918338586	0.190445736156446	0.335676675574006	0.389971284120091	-0.0267433333333334	0.921835451781365
MAPKAP1	0.317632279574008	0.190907715355850	0.130802959467220	0.812271254601992	0.0284466666666680	0.91148338318481
RIMBP2	0.317591460771796	0.190954265268131	0.096548285051865	0.870936619256698	-0.0929529999999996	0.664617185770066
PRCP	0.31752475513863	0.191063108838264	0.0339333115120782	0.96004817357407	0.116879000000000	0.576088341851643
USP29	0.317495625826608	0.191063108838264	-0.118092486412925	0.838500126709059	0.0169616666666671	0.952448105846014
PTEN	0.317316221016306	0.191390832705995	0.478110388555791	0.140790518966316	-0.00910916666666582	0.980779858267938
SYNCRIP	0.317263486304459	0.191391960730331	0.164861108998505	0.753300653779567	-0.272434666666666	0.158105452461472
CORO6	0.317048812632075	0.191690144184427	0.205563326198168	0.672695616352514	-0.0462866666666669	0.866671423175521
KCNMA1	0.317032116883313	0.191690144184427	-0.0824993938270335	0.8939835772794	-0.158834333333332	0.577269890738407
XYLT1	0.316762089755029	0.192343951230395	0.42485018925227	0.216149711053392	-0.628438666666666	0.0243055281020217
GTF2F1	0.316451146479596	0.192933519933365	0.0266382211630141	0.970207612777188	-0.0729336666666665	0.758341714501375
CXCL6	0.316448435959088	0.192933519933365	-0.227783493767052	0.624564310654617	-0.176669333333333	0.459236736587605
NR2C2	0.316365931755842	0.193150481569601	-0.0747339720013104	0.904466414650249	0.0678273333333336	0.78036180126224
ACTR3	0.316217028675264	0.193455029637665	0.129047564512104	0.815246175658132	-0.0111299999999997	0.97222097877396
ITGA8	0.315986723524149	0.193900666015668	0.356342252028988	0.345997909309632	1.08793833333333	0.0128046281952114
RPS24	0.315864439190017	0.194185719617492	0.149700346782255	0.78143277453973	-0.145357999999999	0.510372461062965
TIMM22	0.315790529189328	0.194281115249608	-0.0781237821393092	0.899652641295827	-0.174308999999998	0.408086593806833
PTGIR	0.315726350471460	0.194380421308222	0.055165822246882	0.93374866322822	-0.100388333333333	0.673034330904667
SEC24C	0.315654396541400	0.194446454411951	0.277878030666314	0.51282951825352	-0.558074999999999	0.0234650314672839
TADA3	0.315637502158268	0.194446454411951	0.110558471391816	0.84940885580427	0.0747046666666671	0.73097308185803
SERPINB9	0.315624790296491	0.194446454411951	-0.238499784443148	0.601159664824234	-0.106509000000000	0.748388402768556
GPATCH2	0.315567006519303	0.194504349123635	0.139365640876480	0.798615265561297	0.192727666666668	0.33989570998241
TRERF1	0.315497528608144	0.194622603589634	0.131504329067170	0.811042872465441	0.0464558333333334	0.862509125961694
DSERG1	0.315492170897006	0.194622603589634	0.25570083012589	0.565656702091657	-0.0816489999999994	0.705698790800484
POLI	0.315217578495795	0.195082285437809	0.250863608642826	0.572924277154653	0.290017333333334	0.128957627033936
DDX11	0.314902507440092	0.195792776740501	0.107712648760951	0.856450662791083	-0.103850888888888	0.64069646762551
SRPX2	0.31488007396735	0.195792776740501	0.13989452966437	0.797863910048852	-0.106729666666666	0.709574076502276
MSL1	0.314836492803595	0.195792776740501	0.176270983946075	0.730407919148629	0.289104333333334	0.126863181258387
TACC1	0.314826970134773	0.195792776740501	0.186565957743897	0.711334804690432	-0.118160999999999	0.631408228698652
MYH9	0.314810923701493	0.195792776740501	0.11995588755164	0.835280975936648	-0.0646733333333308	0.813402975148615
HELLS	0.314686175005249	0.196016380749519	0.299792501759014	0.467332983744709	-0.0747773333333325	0.771373465365664
MUC20	0.314660745808999	0.196033613675653	0.444564695466653	0.183005576574781	0.0936520000000007	0.659124451108183
RUSC1	0.314602667261054	0.196055395694612	-0.00861913842252057	0.98946753270812	-0.057296333333332	0.796708478797823
HOXB7	0.314554344337623	0.196056325338883	0.150883077674527	0.778253782413218	0.345631666666667	0.0913180596225537
C17orf70	0.314420031659699	0.196068754062959	0.587018133516011	0.0386126735067483	-0.0857496666666651	0.707538975864338
MIB2	0.314407317398649	0.196068754062959	-0.149041955903020	0.782947197211795	0.0117446666666674	0.974451420920923
SMAD7	0.314404416187215	0.196068754062959	0.159846972997671	0.763150272733332	0.159564666666668	0.419447135794164
USP3	0.314192337027465	0.196676706942611	0.288665636843295	0.489813374112312	-0.0302493333333319	0.910190419163007
SHROOM1	0.314072529152100	0.196913153993582	0.516679508393658	0.0919737522070575	-0.108715333333333	0.706965493695243
UNG	0.314064438296004	0.196913153993582	0.321086538236636	0.421618886526906	0.00206100000000055	0.99529569362957
GDAP2	0.313907920417502	0.197314978609862	-0.305200316529396	0.458013550008763	0.050807666666667	0.888307855721476
ABCG5	0.313857963118795	0.197314978609862	-0.0402034469127459	0.952930192037229	-0.0696073333333329	0.772139923817113
SIM1	0.313849317434471	0.197314978609862	-0.0270518503685356	0.970207612777188	0.0946010000000002	0.719909781429035
PSMF1	0.313673491021509	0.197869584358950	0.163256481054111	0.755849656740422	-0.110288999999999	0.587396808178803
PPP3R1	0.313631460993737	0.197883600099076	-0.12331971791234	0.827531665631642	-0.0303722222222213	0.910190419163007
PDHA2	0.312897650682229	0.199772425122298	-0.0131182956429249	0.984547546298896	-0.242933333333333	0.217667994730431
FEM1A	0.312816601295238	0.199917223333229	0.190996555242236	0.702403218972767	-0.0551699999999992	0.81269393719335
UFSP2	0.312740854679590	0.200116575391322	0.293037092888213	0.481961219375454	0.101068333333334	0.710581219632272
HIST1H1C	0.312628758871164	0.20033784314213	-0.0585407611953228	0.928622896197546	0.211495666666669	0.36523677764789
SUZ12	0.312587590731078	0.200374983399979	0.197369196343535	0.689176078372462	0.0299766666666676	0.91148338318481
CRTC3	0.312507835830181	0.200450457690973	-0.0707913688590352	0.909764940148113	0.205136333333334	0.320462604748547
FAM208B	0.312447606157584	0.200528019522819	0.0902930116886731	0.882127593406303	-0.175962999999999	0.459236736587605
ZNF709	0.312443301561939	0.200528019522819	-0.120904925158235	0.832982209969764	0.118866333333334	0.7108031545527
CCDC148-AS1	0.312305335715837	0.200879134987305	0.208182820402899	0.66730832144774	0.287074	0.274121181062751
SLC7A1	0.312249647706927	0.201003308100011	0.120934035478024	0.832982209969764	-0.465799333333333	0.0757640455290112
NANS	0.312228333142689	0.201003308100011	0.294999675502036	0.478655242507888	-0.187758999999998	0.360870827033104
SULF1	0.312155666117202	0.201111188168727	-0.162869885909298	0.75703912930782	0.38676	0.18951095179740 8
CBX4	0.312060238614844	0.201201384724975	0.576620171941819	0.0437485312286241	0.202272333333334	0.370079759630929
SH3KBP1	0.311715739525159	0.202204374155796	-0.357714212267877	0.344139573942358	-0.409090999999999	0.198090248389223
TP53I3	0.311688336404861	0.202230138041153	0.256586652066137	0.564398995459794	0.0191483333333335	0.950884054684253
UBIAD1	0.311508741853042	0.202735172527675	0.0305810906287482	0.965652334162082	-0.378333000000000	0.0697146597616042
ADAR	0.311387324993104	0.203023389009975	-0.114741749599616	0.844162087612813	-0.419409666666666	0.0587663770637753
MAP3K12	0.311199898389304	0.203351298329506	-0.281347054132247	0.506585071809407	-0.0522546666666665	0.880347900939311
TRA2B	0.310944199292362	0.204053963326665	-0.0303291582140883	0.966100909843628	-0.0390766666666657	0.88384908879607
PTPN7	0.310811761547328	0.204315999891462	0.111823749333651	0.848166799200802	0.0584510000000003	0.856256796859322
MYO5B	0.310674125001354	0.204431181296336	0.0688360418332384	0.912149839732916	0.210045333333333	0.4261850174154
CACNA2D2	0.310652007861484	0.204431181296336	0.00349820223810761	0.994730189431381	-0.00676466666666642	0.983159241812861
ZNF516	0.310631327651433	0.204431181296336	0.364614631451876	0.332583029577968	-0.148544000000000	0.516856313887476
LRRC3B	0.310606272775583	0.204431181296336	0.244545340983094	0.587653287154633	-0.131989000000000	0.5530742296054
SRP72	0.310598278100614	0.204431181296336	-0.338723995015028	0.383991827871268	-0.071960999999999	0.754015160951226
WDR48	0.310187987750040	0.205249440557360	-0.127639452346133	0.818717515586031	-0.0236989999999996	0.927709670800671
TMCC3	0.310177152948413	0.205249440557360	0.337644407807092	0.386063654475806	0.194014	0.443378989641463

TOMM20	0.310143278076910	0.205284975812529	0.142371655716795	0.794346532964905	-0.00445533333333296	0.988681127335607
MOB3A	0.309836533853257	0.206041706068384	0.110484025009356	0.849626303351656	0.0525540000000008	0.818849076927228
MBD2	0.309800903628606	0.206098730715848	-0.315832389157153	0.433880593421022	0.0549940000000013	0.81742145099016
DDX50	0.309738984908086	0.206101609316637	0.106572369752258	0.858072018696671	0.0617433333333348	0.783223894269194
HCFC1	0.309674459910113	0.206101609316637	0.0390790047819129	0.95397534100479	-0.120831666666665	0.593549842863452
TOB2	0.309671307151505	0.206101609316637	0.193525335890584	0.698131592467487	-0.119108499999999	0.690641668678273
SF1	0.309374482264514	0.206728962961715	-0.227550698134481	0.62531125089249	0.0345236666666665	0.889610038918956
FAH	0.308992603019145	0.2077465675079	0.340609145130141	0.378350877728096	-0.140110333333333	0.69523606597876
OGN	0.308930329821894	0.20782866410878	0.0829198503733603	0.89362638370288	0.0773320000000004	0.752272447179795
PPP6R3	0.30886163230434	0.207861672356597	0.0451136383362957	0.94659867551232	0.0617983333333347	0.777568084061469
ERCC1	0.308780157561608	0.207861672356597	0.0225794972209768	0.97536722461972	0.119994000000000	0.575378149225683
SNX10	0.308747135825898	0.207861672356597	0.353233012419539	0.352407344218756	0.390738333333333	0.23765426508992
C2CD2	0.30870919727613	0.207861672356597	0.389899870062214	0.282726287492993	-0.0199016666666663	0.954849922195008
METTL9	0.308696050818489	0.207861672356597	0.481530067862387	0.135560525489292	-0.0613456666666659	0.811401072147441
MMGT1	0.308630768458777	0.207974845008236	-0.491025539445503	0.123841586691124	0.0293363333333339	0.907648578460912
PPARA	0.308496525959496	0.208171372738391	0.342478877179987	0.375699706552901	0.136156000000001	0.559387719874282
POU5F2	0.308478004079889	0.208171372738391	0.0443940082090527	0.948327980852394	-0.239536666666666	0.545628708794389
RPRM	0.308371494882815	0.208491862384714	0.0553628014890176	0.933409139126562	-0.056872333333333	0.824110405462623
SSC5D	0.308330330048606	0.208570527433216	0.145614806956851	0.787804113309913	-0.0732120000000002	0.798344353938564
COL3A1	0.308210195453067	0.208650149131104	0.229119217766746	0.62075895683145	-0.305426333333331	0.159176629475828
ZNF778	0.308189263527918	0.208650149131104	-0.384688599246095	0.291180853572672	0.0310796666666678	0.92469922248949
ZNF865	0.308043005235656	0.208650149131104	0.239134874899702	0.599801655225887	0.00169133333333371	0.996285985364494
CEP70	0.308034529375039	0.208650149131104	0.0131686906997099	0.984482935754244	0.177333000000001	0.477169587518779
IGSF10	0.308020154608084	0.208650149131104	0.382605740003158	0.294988445038215	0.127138000000001	0.568393891086758
OBP2B	0.307926210177004	0.208815618742662	0.400546842982927	0.262685489461853	-0.0568836666666659	0.852346905702153
SRC	0.307829498402129	0.209027055591916	0.0991231960742153	0.868042453226125	-0.155424666666667	0.484317289991301
BICD1	0.307773544166707	0.209087540298265	0.0129851267406267	0.98456132886228	-0.0470413333333325	0.854573398968221
MT4	0.307520447987973	0.209425107090409	0.0867324448039846	0.887450470745992	0.0881000000000009	0.67320648871569
FUT1	0.307505394386683	0.209425107090409	0.328827725783893	0.406249092981339	-0.163948999999999	0.523762186913243
RELB	0.307458702814527	0.209494616217219	-0.556489695830836	0.0555982011257914	-0.154293333333332	0.476702574318922
CAPRIN1	0.307428969684786	0.209494616217219	0.147246531706256	0.785649719908014	0.0462193333333341	0.848860592348058
LRP1	0.307425513850761	0.209494616217219	0.376501744892677	0.309333317779353	0.0931140000000014	0.729912257977231
GSN-AS1	0.307266391782989	0.209769585370277	0.0565870336085707	0.93176886339657	-0.0304509999999991	0.929594956567283
SLC2A4	0.307261779583663	0.209769585370277	0.108946828974582	0.85318214208878	0.097843	0.68641735550035
IZUMO1	0.307250302108319	0.209769585370277	0.378625306852792	0.304578046752771	0.0412053333333339	0.87629402535202
RIT1	0.307233893850248	0.209769585370277	0.140666722769727	0.796714214272984	0.302875000000002	0.136902334935621
LRRTM1	0.307075101570063	0.210213154176107	-0.117492191683114	0.839675491536757	-0.0351699999999997	0.911600329626215
OTUD4	0.306902675603183	0.210561434069450	-0.275037125619517	0.51990180188597	0.358769333333334	0.120319574336004
NCKAP5	0.306863055521465	0.210597211699544	-0.160799844787203	0.761016253411733	-0.0499879999999995	0.910696784037738
SAFB	0.306769091434446	0.210597211699544	0.124670140599404	0.82479991054473	-0.0317119999999985	0.904788907158467
MESDC1	0.306710223035951	0.210691784560765	-0.0072663722350709	0.990932082705863	0.0348930000000019	0.896186784012643
SLC25A12	0.306689672480777	0.210695113109457	-0.154543399922984	0.771310656070265	-0.0999409999999992	0.657217907037306
PHF3	0.306579047361972	0.210932738112836	0.224944835621653	0.631321197631073	0.0196626666666680	0.945904449997854
ZNF507	0.306545845358910	0.210932738112836	0.312922691545585	0.438827570495502	-0.0556509999999996	0.818849076927228
MRE11A	0.306527667601502	0.210932738112836	-0.248933511805446	0.576871285544251	0.0157196666666675	0.962126225694904
CNKSR1	0.306443624181989	0.211099675740574	-0.182384693203246	0.719419078472232	-0.104341333333333	0.64240152094964
EFHC1	0.306297550253376	0.211498235195113	-0.280537588600423	0.507460240190768	-0.154785000000000	0.442294185443201
PSPN	0.305993786835113	0.212047373984906	0.320920426325573	0.421618886526906	-0.0919413333333331	0.710456701900109
KIAA1024	0.305942194978237	0.212093894628381	0.193840862072869	0.697189653813355	-0.374053999999999	0.0660125264725009
C1orf110	0.305763861458349	0.212146137856256	0.0940669196700143	0.875237089894291	0.0327316666666675	0.899614658619676
TTLL5	0.30575982550054	0.212146137856256	0.385640335584656	0.289712954934028	-0.426778666666666	0.0809474355460827
PHF21A	0.305741570418109	0.212146137856256	0.157947197631539	0.767373487136803	0.0430250000000003	0.880844178274306
C14orf23	0.3057303702908	0.212146137856256	-0.203446441801644	0.676507174634579	-0.0297296666666663	0.909450927730061
ACTR2	0.305716683713812	0.212146137856256	-0.155264764833480	0.77058108906698	-0.118503333333334	0.573757026246692
C17orf85	0.305446445527929	0.212617338791248	-0.025945750534952	0.971011584434977	-0.0419083333333317	0.878857979526584
LINC00328	0.305403388380571	0.212617338791248	-0.0266805474627498	0.970207612777188	0.496281000000001	0.227445926265811
DHX33	0.305362463594272	0.212617338791248	0.098681763518551	0.868080433714765	-0.349567666666665	0.140860001683260
CLK2	0.305264425114626	0.212617338791248	0.302193324669512	0.462229407853213	-0.164342666666665	0.401207235916543
C22orf23	0.305252116246463	0.212617338791248	0.104673674984561	0.86067831050617	-0.0734219999999998	0.848896273601345
FRA10AC1	0.305219948630556	0.212617338791248	0.188883851709166	0.707236456264125	0.0340970000000007	0.894077464638887
TRDMT1	0.305208197211545	0.212617338791248	0.337363078335413	0.386769126296059	0.123440000000001	0.60814016771842
MCM3	0.305091579505587	0.212652907456724	0.116914412670913	0.841512197646315	-0.0386286666666653	0.877597838720525
EIF3L	0.304795626125521	0.213470975235571	0.246855340162598	0.581279386777	0.152683333333335	0.509869431796637
VAV2	0.304694717209291	0.213588697385157	0.282784772052127	0.502507045934724	0.113232666666669	0.639837290428782
ACD	0.304686398271609	0.213588697385157	0.188145638319912	0.707635058271509	-0.223441	0.246475973365341
TUBB	0.304667436239278	0.213588697385157	0.182712838336364	0.718559738568628	-0.0216966666666654	0.939045817001727
PIK3C3	0.304623818518475	0.213679753761604	0.271917098354507	0.527284725671175	-0.0901379999999986	0.698638671208267
ANGPTL4	0.304593270436649	0.213721803032061	0.235407946943668	0.60678764664537	-0.204539666666666	0.29098458066948
SOCS4	0.30430779513935	0.214304488496227	-0.0364596229874879	0.957567496533117	-0.0376953333333315	0.888762687775546
USP37	0.304302854902704	0.214304488496227	0.352973423679489	0.352407344218756	0.117028000000001	0.649977903605799
NEB	0.304283178815492	0.214305870670173	0.075172521455516	0.904173044514532	0.102950000000000	0.625258262671142
MARCH6	0.303966618770878	0.215061602531843	-0.108533326265834	0.85395367181085	0.118383333333334	0.565719642595747
GSK3A	0.303927140776330	0.215137716701275	0.175474356937902	0.731991966468784	0.00529333333333398	0.987937064305561
CSTF1	0.303729521496069	0.215664006251265	0.210059130849813	0.663000357794695	0.0354363333333340	0.902049890102179
ADNP	0.303710546344482	0.215664006251265	-0.0487879974906358	0.942180113539637	0.150020333333335	0.466277136667929
FTSJ3	0.303648345453292	0.215767644245604	0.121746367434395	0.83097116342043	-0.157801333333332	0.442294185443201
COL15A1	0.303577445656621	0.215875768069889	0.237855469558344	0.60243420134202	0.0959090000000008	0.710483929190267
ATE1	0.3033699274537	0.216223895675687	0.065351500730902	0.918295693698803	0.184270333333334	0.340052241399374
PVRL3	0.303293362720829	0.216433981453287	0.192996051053861	0.699138815299348	0.483608000000001	0.0285444246472189
CDC123	0.303032497044428	0.217065210712423	0.0811713891731218	0.896346444707454	0.0186836666666678	0.950884054684253

PDK4	0.302940629037357	0.217195211816904	0.699176158252689	0.00504930650245257	-0.176473333333334	0.351895650223938
MMP14	0.302512747740156	0.218384257066199	0.306981576069476	0.453768045270662	0.0598806666666668	0.798344353938564
TCL1B	0.302304590492774	0.218993141522434	0.0566816054199276	0.931729752152998	0.00590333333333426	0.987937064305561
FOXD1	0.30229533515087	0.218993141522434	0.243732426814055	0.590086964098531	0.152545666666667	0.471989789810989
SP2	0.302276385870427	0.218993141522434	0.0716789448682455	0.908481403751751	0.0680570000000013	0.829662675541773
LINC00312	0.302180725042443	0.219212075901201	-0.357797957225768	0.344139573942358	0.0592096666666672	0.823956428025517
DVL3	0.302049477507192	0.219420861270241	0.511547093696435	0.0987227738109781	0.0504126666666678	0.830320848383177
BTG1	0.301823448817471	0.219561029860245	0.245452467845161	0.584678706753837	0.0233900000000004	0.933734186466081
INTS4	0.301821625564566	0.219561029860245	0.016470622792295	0.981892085795004	0.107623000000001	0.732554472781933
GORASP2	0.301767103795967	0.219564783459621	0.288211954052117	0.490849570883573	0.103190666666667	0.63113882160721
PPIC	0.301741757487779	0.219574551534238	0.250899845271391	0.572908931754283	0.525127666666668	0.0782543184284344
SYDE2	0.301649092139903	0.219637279265372	0.0577731967191039	0.929745210212238	-0.0228770000000002	0.96142941340171
MLLT6	0.301589060202056	0.219721106713606	0.256524663457338	0.564430426783556	-0.287562999999999	0.161778216065929
LOC284788	0.301460656789780	0.220066498590128	-0.216227002003902	0.648391670659269	0.292679666666667	0.324937679238988
BCOR	0.301188878181196	0.220524431743203	0.146731551042495	0.786187758946602	0.262453333333335	0.206257290355168
UBQLN4	0.301038797903136	0.220954105102955	-0.0556770124352611	0.933409139126562	-0.616846999999999	0.0143541040572610
TRAM2	0.301002159601668	0.221020907901769	-0.123177881840026	0.827789978865629	-0.0203849999999987	0.961509682688243
CELA2B	0.300983395149836	0.221020907901769	0.165513228990667	0.752352242644389	-0.110549666666666	0.673538419974363
TRIP12	0.300939233455115	0.221076433038256	-0.0383543773232674	0.954545200586067	0.0382083333333334	0.884696468393321
PTPN13	0.300930951886625	0.221076433038256	0.497200149657261	0.114508695522455	0.122565666666667	0.578893615511221
ZNF143	0.300874816368260	0.221198393306705	0.0462843405787836	0.945019358156718	0.142409333333333	0.575576625147906
RSF1	0.300802074719217	0.221346853799722	0.0530839696786444	0.936712858731269	0.189142666666667	0.332315132116478
VAPA	0.300669020375055	0.221654938801276	0.316345570757384	0.432990247663455	-0.0382729999999993	0.88629962370345
LZTR1	0.300631700536225	0.221654938801276	0.216324184394775	0.648391670659269	-0.0527499999999991	0.82616467676523
VASH2	0.300536479135454	0.221785611526073	0.227396828902433	0.625706135443686	-0.0280553333333327	0.914066650169675
YTHDC1	0.300527854525697	0.221785611526073	0.0199800056775984	0.977282473659462	-0.0136789999999986	0.961827122082291
KIF21B	0.300408714348933	0.221964567988195	0.145887373590698	0.787741385926872	-0.108005333333333	0.719304894613107
SHOC2	0.300396616154818	0.221964567988195	0.204820581587069	0.673728362275223	-0.0461193333333325	0.844766787725698
ZNF682	0.300233499927374	0.222302806411708	-0.0864448822376886	0.88771097663173	0.169304666666668	0.523453039510001
SPIN4	0.30022333856477	0.222302806411708	0.0297471645831403	0.96695070705759	0.0875046666666671	0.766132481270445
CHD9	0.300086318841548	0.222437542505229	0.222570035413737	0.635668764102271	0.0675241666666674	0.809769116280204
TIMP3	0.300084799042326	0.222437542505229	0.397525133390731	0.269713236868058	-0.409652999999999	0.337886644302564
NNAT	0.299879579353482	0.223156062400876	0.131453043022807	0.811042872465441	-0.0314330000000001	0.903182673566864
GAS5	0.299740511202352	0.223555102253166	-0.0207709252397434	0.976330597778846	-0.107137666666665	0.716517864791776
STEAP2	0.299738543242859	0.223555102253166	0.114901634753257	0.844162087612813	0.0722816666666667	0.739677496053195
KIF19	0.299709174418697	0.223595690999658	0.138875858395768	0.79879462261805	-0.0358525000000001	0.922799610581502
XPO1	0.299640948466702	0.223667668942928	0.0842056337903593	0.892396784625427	0.00562666666666802	0.986471172041113
RHOH	0.299633975122531	0.223667668942928	-0.26775587384809	0.53559545997875	-0.0949299999999994	0.716268843468132
TBC1D5	0.299403692950865	0.22435318999479	-0.197327880171899	0.689256317041816	0.0443163333333334	0.86260982919627
STK39	0.299378490717015	0.22435318999479	0.0518655973547384	0.938067483409541	0.489487333333334	0.0601168126740641
HDAC2	0.299166309696068	0.224705994055049	0.269958126831187	0.5309172412154	-0.026781666666666	0.922148106708426
ARL3	0.299139874495333	0.224705994055049	0.440619135694656	0.188962459353093	-0.0433433333333327	0.867268897859745
GSG2	0.298934355194772	0.225256904600399	0.083958323941025	0.892699875421172	-0.0695329999999994	0.785009062971273
MPG	0.298916733893924	0.225256904600399	0.563400074456076	0.0506240759292818	-0.0876663333333329	0.776538187294065
KIF7	0.298846240450492	0.225262537965351	0.544376263385381	0.0651638396693706	-0.0038896666666666537	0.990930928353104
SYMPK	0.298846143358282	0.225262537965351	0.242061831380367	0.59465236431643	-0.0265903333333311	0.915761046438965
CELSR1	0.298826608841843	0.225265484745873	-0.317197184406129	0.43145661636151	-0.0544069999999994	0.852687970849616
IRF2BP1	0.298749321197727	0.225291643711322	0.521078251757861	0.0887204144095562	0.00286066666666778	0.993756838831997
GATAD2A	0.298635717285921	0.225602602288269	-0.0506953719497606	0.940271086498237	-0.0576169999999993	0.798344353938564
ITGB1BP2	0.298583714596752	0.225678728597816	-0.429868722959019	0.207968627016989	-0.191681333333333	0.320462604748547
SFXN3	0.298332785160578	0.226092532376004	0.368598614678195	0.324129110126502	-0.128574666666667	0.653376501568506
IL17RD	0.298126580423179	0.226558906277037	-0.106696998262836	0.857937967416038	0.0797133333333342	0.728708142852036
WIPF1	0.298075799530358	0.226558906277037	-0.0812782885970445	0.896346444707454	0.302185666666667	0.124096550131011
UBA6	0.298067785170820	0.226558906277037	-0.242538845277853	0.593306823292819	0.136227000000001	0.499957077244054
WNT11	0.298063486779124	0.226558906277037	0.290198924928765	0.488647763802364	-0.465130666666666	0.0854838695526452
AAK1	0.297986221143016	0.226787333179039	0.438942122548478	0.191636246400456	-0.0117523333333324	0.979790222743567
ZNF385A	0.297850418182014	0.227102332890549	0.462267918194443	0.159445854532777	-0.328521333333332	0.138534086847106
NENF	0.297785904079904	0.227131981789437	0.213697219005736	0.653708954135733	0.188934000000000	0.344576368488222
NPM3	0.297668427071405	0.227518033079541	0.102994926711653	0.86257932725575	-0.0357053333333326	0.887363245747406
GSK3B	0.297500790435070	0.228027940547950	0.0674156859282272	0.914318618000915	0.0957640000000002	0.640267000021599
MAP2K6	0.297374585328807	0.228449397671064	-0.199590734511003	0.683529917975725	0.0175213333333344	0.96003296204918
AIG1	0.297272119930213	0.228705037506177	0.0267624369354830	0.970207612777188	-0.0815686666666667	0.707966006319856
MEIS2	0.296991302599382	0.229368918164159	0.240300719924209	0.597862631330416	0.401913666666667	0.0546896912427088
INPPL1	0.296906342192072	0.229410144080668	0.100427099890320	0.866539444904289	0.153030333333334	0.481094083070727
MORC2	0.29680137667756	0.229749906143038	0.0487296465630056	0.942209609547615	-0.0904643333333325	0.720663453207678
C4orf46	0.296728814993220	0.229850965234755	0.174930700057699	0.733518674801325	0.0203613333333331	0.94375604597120
CLTC	0.29672638381315	0.229850965234755	0.100092977042983	0.867175669847129	0.00681000000000257	0.983159241812861
PACRG	0.296719897786330	0.229850965234755	-0.257771581932172	0.562492892055072	-0.0643063333333331	0.787091496664561
PCDH18	0.296650860275284	0.229863889195821	0.358318224599592	0.343186459025557	-0.0698383333333326	0.777406710539424
ARNT2	0.296512672255446	0.230228020293523	0.113018814347712	0.846505494838606	-0.0314646666666656	0.919359825156123
ZNF121	0.29643226136823	0.230303155426860	0.087932364112191	0.885848000380851	-0.145478666666666	0.558494409261702
NARG2	0.296254714217036	0.23053322897815	-0.0198220256249042	0.977526645675126	0.186029333333335	0.330463225389927
NPAT	0.296184707791936	0.230716645414619	-0.403250852812325	0.258453301425503	0.0433040000000006	0.890073383340154
ATIC	0.296057237104934	0.231042225934468	0.144003440839597	0.791666231156359	-0.0540933333333332	0.843134844119548
MBTPS1	0.295979108069051	0.231042225934468	0.0783751464266616	0.899281198071059	0.0778266666666665	0.716517864791776
SRPR	0.295943755028563	0.231042225934468	0.0110503493504011	0.98774001793194	0.144186666666668	0.481899892112649
TTYH3	0.295935638320172	0.231042225934468	0.0698113575619647	0.910931201239754	0.126517666666666	0.541649967621301
DCUN1D4	0.295707621839178	0.231524966084487	0.0540274723513818	0.935109055935003	-0.0963153333333318	0.6715789263798
PAX6	0.295700066605357	0.231524966084487	0.382850608205874	0.294588782643652	-0.0035116666666666628	0.991175862027014
B3GALT2	0.295672806246373	0.231524966084487	0.00269050948843456	0.99580901762095	0.0066493333333333335	0.983159241812861

PAK4	0.295645442003122	0.231524966084487	0.309782760906875	0.4470577615309	-0.0220696666666667	0.93278021276477
STATH	0.295614858042993	0.231524966084487	0.0802567032127403	0.897478730716615	-0.0321623333333328	0.920908349612552
EML6	0.295604427826574	0.231524966084487	0.536767244202183	0.0732739991995906	-0.610040666666666	0.0739218716203512
DDX11L2	0.295591852864503	0.231524966084487	-0.0187064667157207	0.978981596219275	0.148788000000001	0.544685122345675
ADAMTS19	0.295461747305575	0.231966606218206	0.332320532538336	0.39832322900678	-0.0444896666666659	0.850157189407213
UBE2G1	0.295399110728332	0.231995628768264	-0.0253232229183674	0.972285580751433	-0.0268563333333321	0.915761046438965
DDI1	0.295300792844837	0.232287520282706	-0.274456167059098	0.520583045401476	0.067043666666667	0.792514737228328
ANKRD17	0.295282320063326	0.232287520282706	-0.0443261575763241	0.948385499132766	-0.191219666666666	0.351182087586052
RHBDF1	0.295206174204954	0.232448129634437	-0.058259362377831	0.928856173352116	0.0564426666666685	0.85045155392574
TLE2	0.295192755443133	0.232448129634437	0.402034447452131	0.260476951140907	0.0494063333333347	0.849249789604201
KIF22	0.295155991641028	0.232472983001624	0.219823342618105	0.641035290381005	-0.0392059999999982	0.888285531317364
SLC25A15	0.295109129697432	0.232474310068372	0.120962608718900	0.832982209969764	-0.200632666666665	0.310277378475854
LRRN3	0.295102745382679	0.232474310068372	-0.346632465318493	0.365167159367406	-0.0083843333333333308	0.981191596773742
FEN1	0.295094152746942	0.232474310068372	0.0544102444445611	0.9344692810234	-0.21202	0.274914613771951
ANKFY1	0.294828009864072	0.233212542396274	-0.00426469725848269	0.994074534582414	-0.134920666666666	0.513543635781495
SHPRH	0.294757569599620	0.233372198274243	0.267232657724120	0.536453197697168	-0.181747333333332	0.410296457977492
MCM6	0.294598865777479	0.233857154899516	0.294906591004413	0.478695647537866	0.0905766666666682	0.70916032632167
SAMD11	0.294481954400707	0.233988447684391	0.138760650996018	0.798926084240744	-0.169979333333333	0.551125574415782
CCNJ	0.294456484978971	0.233988447684391	0.225309199572382	0.630392692544437	0.0997580000000004	0.63263903123177
KIAA0753	0.294445513921455	0.233988447684391	0.133660686203293	0.808933084408149	0.134940000000000	0.591142517306808
HDAC4	0.294437390769856	0.233988447684391	0.425205185950698	0.215733128998344	-0.0861196666666665	0.76648935876098
PNMA3	0.294394318295714	0.234013927994345	-0.147512533049065	0.785453680209733	-0.0753606666666669	0.766247450031517
DZANK1	0.294264510470364	0.234174094512463	0.35266510891415	0.352560848501456	-0.180753666666666	0.376224594108747
PUM2	0.294262476755870	0.234174094512463	0.26619107366183	0.538963077070747	-0.108670999999998	0.600146208272329
JPH2	0.293996009345416	0.235017325844105	0.239592503092468	0.598769383991889	0.134200666666667	0.513141966218084
C18orf54	0.293820182787277	0.235372391159659	-0.0481798041664508	0.943089870033472	0.282606333333335	0.352897567720880
ELN	0.293815604990123	0.235372391159659	0.0221141755346722	0.97536722461972	-0.0843543333333335	0.761257561656748
IQGAP2	0.293733568222476	0.235592326867697	0.250619186127207	0.573664917766258	-0.846504333333332	0.048170505761132
RFC1	0.293724001963822	0.235592326867697	0.127250408823888	0.819791676572254	0.184912000000001	0.365126455342703
ZNF33B	0.29369818550637	0.235622451946834	-0.00041325172309131	0.999513431881539	0.222032000000000	0.32370226799913
sept-06	0.293671818601821	0.23565479604250	0.125400304130573	0.824013463206763	-0.0354393333333323	0.896699926823174
SYT11	0.293293224911855	0.236781111507549	-0.393784567303341	0.275799518638330	-0.0923626666666659	0.773561338021488
LATS1	0.293248280062178	0.236781111507549	0.259380952303024	0.557249797489529	0.114580000000001	0.657217907037306
EIF3A	0.293243972855485	0.236781111507549	0.0947348338922417	0.873818349270144	-0.0595816666666664	0.814959196808566
RBM7	0.293226234907711	0.236781111507549	-0.224144417672539	0.633180978990857	0.493357000000001	0.075243913539028
AKAP11	0.293066507024808	0.237139874948912	0.0596314185165697	0.92656593131102	0.0719880000000001	0.765276224002577
TAF3	0.292999011986566	0.237180746106415	0.341966328934624	0.377158872804021	0.0118173333333336	0.9681321732097
IL4I1	0.292913309338758	0.237451844130391	0.090558799095456	0.88168896154906	-0.0717993333333328	0.74812728952148
CANT1	0.292851580580669	0.237626756426153	0.157958616764824	0.767373487136803	-0.0495846666666655	0.827433678445379
MARCH9	0.292622476279376	0.238402511224091	0.416596172883336	0.234235464483992	0.0869480000000009	0.697721138055031
INTS7	0.292510415841338	0.238541401546978	0.174070299638757	0.734757646402866	0.0215360000000019	0.937545603686416
CTGF	0.292486937367371	0.238541401546978	-0.205543589876728	0.672695616352514	0.403342000000001	0.320462604748547
LTN1	0.292477206772234	0.238541401546978	-0.0761299302298413	0.903296922694955	-0.0257159999999984	0.924168962796313
WDR26	0.29246057310091	0.238541401546978	0.048333601832564	0.942820948997969	0.00572200000000113	0.987134800094338
GPR173	0.292396667399363	0.238548457822536	0.130578521564990	0.812271254601992	-0.114866666666667	0.626500880282759
GEMIN4	0.292336592197195	0.238548457822536	-0.0922130338738124	0.879153312143409	-0.0910666666666668	0.666405059390122
EMILIN1	0.292313425221198	0.238548457822536	0.0874012249144933	0.88655203337859	-0.108328333333333	0.68135180147671
PDE9A	0.292157521453506	0.239011146480269	0.291369002548483	0.486757482084406	0.494202333333335	0.0660125264725009
ACTR8	0.292115135936597	0.239055370690217	-0.404387221965942	0.255554450438873	2.20000000018627e-05	0.999822702792133
CC2D2A	0.291944564780906	0.239500044209751	0.086234991490573	0.88794718020464	-0.0314806666666653	0.922710832267249
UHRF1BP1L	0.291710937735307	0.239620455057557	-0.0382989405139681	0.954545200586067	-0.507613666666666	0.0285444246472189
ERVMER34-1	0.291629299337383	0.239819500227376	0.103634095622333	0.861872724387044	-0.114053333333333	0.571851976728397
SF3B2	0.291603718152686	0.239819500227376	-0.0912661868132247	0.880426986815658	-0.0308126666666655	0.904955321847437
GABPA	0.291599977588773	0.239819500227376	0.0196913530043796	0.97763669370967	-0.128977999999999	0.524845938106282
C1orf51	0.29153854882673	0.239994809645717	0.295113912163861	0.478475354656325	-0.160793666666666	0.490399896328103
ATRNL1	0.291385346379746	0.240273132753807	0.269442342414659	0.532367863371851	-0.0649706666666674	0.816337381174918
KIF5C	0.291378351532556	0.240273132753807	0.0526345837361933	0.937357975161644	0.609806	0.030025317565228
TFPT	0.291326576029000	0.240297094244808	0.283095245979262	0.502129505146473	-0.00131533333333301	0.996290446076447
ZFP1	0.291295743827692	0.240319714124718	-0.0437035970538725	0.949043721172002	0.0808853333333344	0.716057356951225
ZNF641	0.290997804064895	0.241234852837641	0.230638002553799	0.617417844559199	-0.125314333333333	0.641044966033937
ISG20L2	0.290883590695044	0.241625464358241	-0.108779188349641	0.853552381097052	-0.867770666666665	0.0216371053461493
ABRA	0.290764307969585	0.241885375264049	-0.131677651869246	0.811042872465441	-0.135197666666666	0.646350580563869
SUMO4	0.290747546476122	0.241885375264049	0.365861697251648	0.32927628703889	0.0283088333333346	0.91524082920515
GNAZ	0.290636103474593	0.242107236707697	-0.095290604791878	0.872962271153999	-0.200567666666666	0.383716544590226
ZSCAN23	0.290620951448278	0.242107236707697	-0.202699661955835	0.678079790105593	0.177052000000001	0.354653836868638
LRRC37A2	0.290578340531679	0.242192262692465	0.191685289745740	0.701544026774214	0.305315333333333	0.270327918481764
POLDIP3	0.290540589750608	0.242214509072358	-0.0168847347009561	0.981160061237418	0.0114413333333337	0.969513647149429
HN1L	0.290424656557804	0.242444135262861	0.247748997029113	0.579471329472972	-0.154490999999999	0.512869954128574
DMD	0.290394515564597	0.242444135262861	0.192104548772051	0.700820605802046	-0.168118999999999	0.514420666507349
SF3A1	0.290385010978644	0.242444135262861	-0.136310830657175	0.804864448119512	0.0935273333333333	0.667697330885416
RARS2	0.290329813772037	0.242560179253102	0.323796642055179	0.414201624246883	0.0744203333333345	0.745967084434416
TSHB	0.290270782717632	0.242727453700995	-0.0429932099355689	0.950146638225747	0.0200613333333335	0.942092082409632
ATXN10	0.290224341079466	0.242770314562955	0.235609990453001	0.6067637636079	0.185655333333333	0.328724837942497
FOXF1	0.289932989836972	0.243446208162207	-0.0585125485765281	0.928701484237695	0.00608300000000031	0.987937064305561
SUGP2	0.289678755884131	0.244192066426951	0.131464919018372	0.811042872465441	-0.0735653333333322	0.759818201272615
TERF2	0.28965575517496	0.244212826813235	0.138707269920819	0.799075616170278	-0.129374333333331	0.592975387754524
ZNF526	0.289615704619842	0.244303371550393	-0.0108728808809723	0.98774587825743	0.0182953333333336	0.94692575385424
AGAP9	0.289535874168761	0.244483457411266	-0.146309902305065	0.787289066547754	-0.113604333333333	0.586271602862987
FAM160A1	0.289434373584062	0.244601442168451	0.00093600457304045	0.998745415362821	-0.163018666666665	0.762898679429892
PTCHD4	0.289421836509121	0.244601442168451	-0.0517617445471884	0.938263273501766	-0.384393666666667	0.112146551360288

SCAF4	0.289051049156047	0.245737799228939	0.159555033608188	0.763788326309862	-0.122388666666666	0.589703432017128
ARID2	0.2888030117413	0.246316646008510	0.161765837217466	0.758930446817487	0.011784333333334	0.96888828719750
ZNF17	0.288496748097584	0.246989715937021	0.243630816771933	0.590086964098531	-0.0923706666666657	0.748579513438275
CACNB1	0.288281709511125	0.247656257197752	0.317161961423840	0.43145661636151	-0.0947169999999995	0.6846855637534
ANKRD37	0.288153965806757	0.247970849968847	0.417400267924265	0.234235464483992	-0.0531613333333328	0.843134844119548
PRPF19	0.288152078564922	0.247970849968847	-0.305855974023193	0.457192231066199	-0.0716473333333329	0.755884457996427
EIF2B2	0.288076578974952	0.248061891921296	0.199080877819898	0.684963476191413	-0.149587666666666	0.445566557024313
RNF20	0.288048369892285	0.248104856399705	0.0937634687922506	0.875355472414883	0.0736103333333344	0.742738366366583
MEX3D	0.287910037325792	0.248320024063879	0.0988713045784431	0.868042453226125	0.0456393333333335	0.855533429360007
APOBEC3G	0.287882894669228	0.248320024063879	-0.0648594356981392	0.918590305362052	0.102645333333334	0.664617185770066
FOS	0.287788543007977	0.248516993912163	-0.22212825786349	0.636477566065103	0.0632716666666676	0.806863588982305
CACHD1	0.287637430198274	0.248848252316391	0.508868315815199	0.101940287625209	-0.314805999999999	0.29920573922735
CRLF3	0.287604643329443	0.248910462838056	-0.302796419532007	0.462105017205825	0.00256166666666762	0.993756838831997
SDCCAG8	0.287441697665538	0.249390320277258	0.184779008447277	0.716020884288485	0.072793666666668	0.733650645007746
INTS12	0.287368874433217	0.249584950785432	-0.01724402644226	0.980777168787742	-0.0180590000000000	0.96101988265498
SYT17	0.287353190285386	0.249584950785432	0.20289729468932	0.677532075317915	0.320801000000001	0.186172794183622
TPH1	0.287207118794901	0.250044215158464	0.20403089163659	0.675344930297902	0.0119943333333338	0.97184501043952
CLASP2	0.287040182295861	0.250517095964976	0.117627992018444	0.83940078178718	0.193524000000001	0.32631478390653
TSN	0.287006626963067	0.250521607237365	-0.130245756772659	0.812271254601992	-0.125881666666666	0.568802695733480
C6orf201	0.286936258246272	0.250728164490027	-0.133984490640357	0.808567812988011	-0.00653766666666577	0.985263856374185
MON1A	0.286894635086521	0.250827655145651	-0.183544765885085	0.718067440349544	-0.200294000000000	0.408407091406947
EHBP1	0.286791602585562	0.251091781268573	0.300464435233990	0.465764240927686	0.0256526666666683	0.942250108726196
SP3	0.286777965910317	0.251091781268573	0.341445692351393	0.377754622823514	0.0130236666666675	0.96497349601609
ZCCHC24	0.286760335350597	0.251091781268573	0.145251907288048	0.788344418401815	-0.0762823333333327	0.761550371673734
MSL2	0.286543900680541	0.251630370675846	-0.146008143083886	0.787402233709509	0.0499556666666673	0.843134442499413
CNTN6	0.286361742920804	0.25216420876451	-0.0221076124822277	0.97536722461972	0.195819000000000	0.443378989641463
HES1	0.286226725642259	0.252526228862115	0.274913784422165	0.520064783279337	0.943358666666667	0.0190324754730064
NOL9	0.28609510761467	0.252983957516669	-0.237632718188009	0.602664863375033	0.0358210000000008	0.898450944704634
SPPL2B	0.285972982318812	0.253347647731075	0.450238972197977	0.176663460992485	0.0199593333333345	0.94221243164399
LAMB2	0.285901879246500	0.253555093123838	0.208176918702877	0.66730832144774	0.323745333333334	0.187348818220853
KDM1A	0.285888167789455	0.253555093123838	-0.218755472395898	0.642137448592891	-0.0978576666666656	0.646350580563869
PCBP2	0.285250410778760	0.255271866212707	0.105549816814210	0.85956218633532	0.0549199999999996	0.809505390888975
AUTS2	0.285165982230812	0.255479092623222	0.497925181678277	0.114179995542754	0.283532333333333	0.136216968914598
COL4A3	0.285147852806574	0.255481120580589	0.143035173774597	0.793304811422389	-0.314669666666666	0.120319574336004
CDKL1	0.28504875081082	0.255729063259619	0.0465518990288418	0.944795388116619	0.0704680000000005	0.772139923817113
HS6ST1	0.284855332172848	0.256418913252207	0.460184434834898	0.162787294570657	-0.00397099999999988	0.991175862027014
NOL11	0.284805924509537	0.256500978347920	0.102840116516658	0.86257932725575	-0.0566603333333319	0.83255406161 9867
CX3CL1	0.284634395233106	0.256831731107395	0.295399205943081	0.478306608395718	-0.245908666666666	0.311354677197697
VEGFA	0.284515220127456	0.257112745162566	0.268846117244786	0.534295192322247	-0.431380666666666	0.0679471854203871
ZNF638	0.284487315765474	0.257132539179842	0.0295758911119041	0.967205787110068	0.00794666666666686	0.981909411893533
NOTCH1	0.284437741654627	0.257132539179842	-0.095330079283557	0.872962271153999	-0.314841666666666	0.158095716859654
GPR137B	0.284405054350966	0.257132539179842	-0.0458832148291488	0.945372627844176	0.0757960000000008	0.805201268688175
PKI55	0.284404574684367	0.257132539179842	0.192383213234489	0.700450711372106	-0.0622853333333319	0.78326014479459
SOX8	0.284391005921291	0.257132539179842	0.473892129851238	0.145016003120677	-0.0818653333333334	0.764197723764219
SGK196	0.284345397130216	0.257235923419905	-0.0701591994318469	0.910436470094634	-0.169387666666666	0.396454946335441
FAM175B	0.284309692598376	0.257312749479576	0.180801547076706	0.722159001254958	0.178868000000001	0.351081282449499
NHLRC2	0.284239382050824	0.257365628307745	0.350777051965503	0.357367124206561	0.144439000000001	0.485561467820582
HOXC11	0.284226944478445	0.257365628307745	-0.0773917546359793	0.90079793355475	0.0634056666666667	0.833930463530884
CYP4Z1	0.284041400750050	0.258019107130313	-0.269444063199538	0.532367863371851	-0.464466333333333	0.0802890385571452
PHF20L1	0.284033019777376	0.258019107130313	0.395911390267711	0.271850185265546	0.0717269999999994	0.766132481270445
EIF5A	0.283995669924377	0.258019107130313	-0.260377153443042	0.554121616398363	-0.0595233333333308	0.816134952712854
C1QTNF7	0.283928534011925	0.258019107130313	0.256976494437276	0.5632303733857	-0.191239000000000	0.35135795138426
PEG10	0.283924575574783	0.258019107130313	0.349342710659607	0.359794558968334	0.130886666666667	0.542447274777
SHC3	0.283914547296544	0.258019107130313	0.0996153386856079	0.867603998426271	-0.0842449999999992	0.704417585200123
MSI2	0.283805461329657	0.258260811242302	0.572229833098862	0.0443803345694495	-0.270196999999998	0.174804244891111
STRN4	0.283617788415279	0.258843178554683	0.184650509321388	0.716351619471715	0.0760346666666678	0.727239075127513
TSPAN8	0.283595128926204	0.258843178554683	0.161507114094330	0.759189376564965	1.14683433333333	0.00711541332977091
TSPYL4	0.283590385729223	0.258843178554683	0.352796090598605	0.352560848501456	-0.178378666666667	0.404357603184072
ADAMTS12	0.283553559689513	0.258884889635789	0.366783524401029	0.328151450414815	0.0318443333333338	0.90778598212344
RTTN	0.283296627424838	0.259529083268336	-0.183397793892813	0.718067440349544	0.042159333333334	0.882342951312785
PALM3	0.283279782681527	0.259529083268336	-0.251869644614136	0.570718103608174	-0.234438666666665	0.238291935423886
ZZEF1	0.28324433519877	0.259531138268909	0.582513558435872	0.0409876056955995	-0.186409166666665	0.322472191147023
COL4A1	0.283185978253022	0.259632929343909	-0.0619221887691071	0.923034515346352	0.694112333333334	0.00809874206560261
GLA	0.283185496595707	0.259632929343909	-0.122461695926325	0.829281453520812	-0.140136666666665	0.561395335630266
IPO9	0.283103404340554	0.259759480737971	0.207244954567233	0.66888525756447	-0.00769933333333318	0.98147292539484
PDE7B	0.283017456683677	0.259794375234072	0.506022013261703	0.105941223458825	-0.187446333333333	0.343572153228906
LMBR1	0.283011258412912	0.259794375234072	0.355633256629690	0.347014488563088	-0.268457	0.220511040537878
HMGA2	0.282985351199526	0.259800429964464	0.33651135834212	0.389099686779674	0.143837333333334	0.646350580563869
MKL1	0.282971663858022	0.259800429964464	0.0454944721451607	0.94582511262151	0.0371670000000007	0.879843042066371
KREMEN2	0.282890058079881	0.260022393410511	0.0393330246119881	0.953905313456582	-0.186328333333333	0.502766729725258
CACNA2D1	0.28272457360812	0.260022393410511	-0.185908011868265	0.713531731974471	0.536160666666668	0.0185304620090284
FLJ42627	0.282720266452566	0.260022393410511	0.229996133010279	0.619254341957051	-0.124129833333332	0.64675322310909
RBMS3	0.282714460432521	0.260022393410511	0.190350360759559	0.704208582824765	0.321968666666667	0.102141633674994
HOXD9	0.28269325889475	0.260022393410511	0.545718609314482	0.0636634544605188	-0.0458313333333328	0.86183426299397
FRMD7	0.282401544968485	0.261044756527075	0.203525877240743	0.676207233014711	-0.204137333333333	0.3468384033101
TAF1B	0.282286293859331	0.261314570067605	-0.0136100071803221	0.98412883278237	0.151505333333334	0.4423818632121
CHD4	0.282166720600335	0.261627405026628	-0.189955726517012	0.704446739027045	-0.0117499999999985	0.970481529511925
ZNF618	0.282004571360461	0.262140461694217	0.209659551859249	0.663896123742545	0.134866666666667	0.567901106219822
ZNF304	0.281931917718852	0.262140461694217	0.135143105070100	0.807470535721699	0.0568636666666674	0.816134952712854
IL17RE	0.281919755806088	0.262140461694217	0.310590143370839	0.445114770999321	-0.0167383333333331	0.968785090200212

NSFL1C	0.281900112867218	0.262150303934184	0.115435058139598	0.843614348588468	-0.177625333333333	0.356927068534765
TNC	0.281851577448555	0.262258997988652	-0.222358683353979	0.636287617796138	0.0794380000000002	0.856868322249419
GAN	0.281823338446963	0.262258997988652	-0.0241753179126087	0.97420021343239	0.0342626666666668	0.90945379489823
HECTD1	0.281797402702858	0.262258997988652	0.128974227913700	0.815386814587013	0.0733366666666666	0.742839967278
CCDC172	0.281794934376104	0.262258997988652	-0.205456937466823	0.672877031333227	-0.0446049999999999	0.850827510392157
CWF19L1	0.281757246988702	0.262316868039486	-0.0327620069450350	0.961351562552925	-0.115846000000000	0.590504748502192
HNRNPA2B1	0.281546922984842	0.262996273666535	0.384817353454378	0.291180853572672	-0.0838266666666648	0.718456537087952
FRMD4A	0.281496391372227	0.263138929747225	0.422762391051968	0.220881685540025	-0.175460000000000	0.465677858260496
DLG1	0.281473505109675	0.263162861128454	0.392161733709743	0.279492631038186	0.00408200000000169	0.990496921312903
C19orf43	0.281152677654799	0.264058229483333	0.450564470004685	0.176663460992485	-0.0967426666666665	0.639002829892966
JAG1	0.281126898032989	0.264058229483333	0.0684989499499386	0.912149839732916	1.34277466666667	0.00841828254294244
PRKAR1A	0.281009962715166	0.264128833735171	0.267381553378488	0.536380012714446	0.0773066666666674	0.731003031373733
NPHP1	0.280956089264289	0.264128833735171	-0.168078619385763	0.748024524669236	0.126630666666667	0.559387719874282
LINC00158	0.280795110459032	0.264369781202308	0.0725854442313897	0.907556175729082	0.0488753333333339	0.891780340140922
LIMS1	0.280725933469505	0.264469823623908	0.165541882993972	0.752352242644389	0.396732555555556	0.137305556420538
FAM105A	0.280690334594576	0.264469823623908	-0.402358292724307	0.259895916604029	1.37163766666667	0.00311122808725912
WDR1	0.280668951501211	0.264469823623908	-0.319500208263814	0.42581212454843	0.0341300000000021	0.903788355106891
DDX21	0.280636537719603	0.264489781068333	0.0216639604121454	0.97536722461972	-0.363561999999999	0.0683509607150879
ZBTB45	0.280629890257935	0.264489781068333	-0.111239055468256	0.849127967004266	-0.0321316666666659	0.915761046438965
EXOSC2	0.280539381151663	0.264546593089509	0.0292676475246498	0.967657386725432	-0.172369999999999	0.385678201128102
MKL2	0.280533261327411	0.264546593089509	0.116016462834857	0.842733236705133	0.139173000000001	0.523453039510001
UNC5B	0.280515806933156	0.264546593089509	-0.06514240049145	0.918489100363712	0.0678523333333335	0.780828073315043
ARID1A	0.280514790464442	0.264546593089509	0.190801041997128	0.702817138659917	-0.00142466666666528	0.996285985364494
FSD2	0.280254909149025	0.26506870705492	-0.00970934956284096	0.98909166696601	-0.0321383333333328	0.90945379489823
COTL1	0.280229969209252	0.265102237990551	-0.0032610934853255	0.994730189431381	-0.31977	0.167904678405192
MAP3K11	0.280162189084365	0.265246526906541	0.0187411631629947	0.978981596219275	0.00723700000000105	0.98256318768706
BEAN1	0.279907051146005	0.265866825970844	0.00455039884350154	0.993733225898948	0.0765410000000006	0.770296367591727
S1PR5	0.279798580991579	0.266076173627559	-0.0562904444503137	0.932258604719412	-0.0266509999999983	0.92469922248949
PPM1E	0.27966266533906	0.266256434726935	0.474905412298971	0.144197096534399	-0.0345583333333328	0.930518235697105
SDE2	0.279654171744967	0.266256434726935	-0.145237760045131	0.788344418401815	0.111735666666668	0.585260484981764
C20orf78	0.279616278405833	0.266346286792945	-0.156433112660570	0.768909201237015	0.0996396666666668	0.656222957956058
KRBA1	0.279522919214126	0.266474722312921	0.267930246297882	0.53559545997875	-0.0274593333333327	0.9123656811945
LMAN1	0.279518185138475	0.266474722312921	0.0130448505787545	0.984547546298896	0.0389200000000019	0.880254449461876
BBX	0.279429091610933	0.266564041897205	0.481584673421472	0.135560525489292	0.101690000000001	0.634114491543653
CCDC102B	0.279412125656505	0.266564041897205	0.154058737140785	0.772253934458676	0.827266666666667	0.0822813557674986
SMG7	0.279374740032991	0.266596148769044	-0.020985417106181	0.976103466334817	-0.0761779999999985	0.74512135615444
KLC1	0.279321952829580	0.266596148769044	-0.119867721859996	0.83548380271035	-0.0158079999999977	0.954736472265512
TUBB4B	0.279160690147691	0.266840821580040	0.0878882771369857	0.885848000380851	0.180089500000001	0.344959227986463
ANP32E	0.2789038793173	0.267583244478875	0.057529696623271	0.930191499459057	0.0252536666666683	0.936634943217749
SMAD5	0.278871763402374	0.267648647567799	-0.230969754514437	0.616488238055513	-0.0620533333333323	0.839243391791351
YAF2	0.278793963236858	0.267838337057856	0.226533616568644	0.626993722302945	0.0593226666666671	0.831203732262456
CLDN7	0.278709829643358	0.267979770131987	-0.0995205830308373	0.867603998426271	0.128071666666667	0.530088635403439
YLPM1	0.278705667378817	0.267979770131987	0.0641935994928557	0.919923865590619	0.113203333333335	0.664714409479409
FXYD1	0.278664807271051	0.267979770131987	-0.00673143603124263	0.99129507664977	-0.0996333333333323	0.715853170342195
ESYT3	0.2786593135588	0.267979770131987	0.139191669086913	0.79864602540281	0.0311693333333339	0.903788355106891
CSNK1G1	0.278376201499625	0.268904679266447	-0.133517082275242	0.808933084408149	-0.00707766666666629	0.98256318768706
CILP2	0.278361302236944	0.268904679266447	0.312372811677154	0.440033473850785	-0.102095999999999	0.709565024652319
PRR14L	0.278311220645554	0.268974723654341	0.0467879744207311	0.944274732245152	0.00259200000000003	0.994969303295258
MYEF2	0.278195941661176	0.26928559743223	0.0906692354645727	0.881370595149313	-0.0839439999999988	0.750003369924731
RXFP3	0.278182608861874	0.26928559743223	0.164985290983300	0.753093885671651	-0.0564643333333331	0.863745180020314
TNFAIP8L1	0.278171831510609	0.26928559743223	-0.254829808733816	0.566909791913503	0.049846	0.849168149250465
C8orf47	0.278100108755779	0.269455236733309	-0.118788610094762	0.837182040755817	-0.162707666666666	0.447070880722204
PLXNB1	0.278098908924996	0.269455236733309	0.138377415459833	0.799981150504262	-0.0861283333333336	0.724384319568895
RBM15B	0.277912475473755	0.270040263683657	-0.159363729732951	0.764323101548959	-0.00812866666666634	0.980770032498656
CEP192	0.277909270522575	0.270040263683657	-0.145601168829668	0.787804113309913	0.138136333333335	0.513024230069645
WHSC1L1	0.277903463634924	0.270040263683657	0.294728113027418	0.478800228795472	0.213144666666666	0.320462604748547
CACNA2D4	0.27789673686777	0.270040263683657	-0.103041217013781	0.862493899350713	-0.0932673333333325	0.759627452336978
HAUS8	0.277872870555625	0.270070388179998	0.174731622286864	0.733916687337453	-0.125373999999999	0.588666179294878
DEFB108B	0.277741340219642	0.270496146422345	0.0450310676739339	0.94678213523172	-0.0925986666666661	0.705698790800484
HNRNPL	0.277583283289625	0.270970958431909	0.217874715234393	0.644309798627935	0.0108600000000014	0.971208229448032
GDF11	0.277503453834086	0.271068655881579	0.18190022051926	0.720096350499662	-0.437113	0.226499104403529
FLVCR2	0.277491636334339	0.271068655881579	0.429126964934093	0.209103669076588	-0.372856333333333	0.0667771856938434
RPS9	0.277409337248187	0.271228578025712	0.241913739236410	0.595018350907538	0.0809433333333346	0.741134448923316
ITGB1	0.277404825311339	0.271228578025712	0.0344114204244991	0.959404955370931	0.248878000000003	0.211495399841293
AEBP1	0.277314176218586	0.271548482989318	-0.00901235783111208	0.98909166696601	0.0249663333333339	0.92484471634727
OTUB2	0.277195602206696	0.271887452510717	0.124314789308914	0.825324566780441	-0.0902219999999994	0.733679380704515
BMP7	0.277185964483273	0.271887452510717	0.328599731739632	0.406698358321253	-0.0675019999999994	0.766563380016891
LEPREL4	0.277059777095001	0.272366193281667	0.22473356076286	0.632196674777516	-0.261188666666666	0.337307065334106
CDK2	0.276984110063612	0.272401363387609	2.11187682206193e-05	0.999906934715157	-0.0524633333333316	0.826922793139517
SHF	0.276794438747177	0.272917828639371	0.326251634898401	0.409206882509236	-0.0269666666666662	0.935737101155951
ZNF417	0.276781879841863	0.272917828639371	-0.262659347767633	0.548256555997615	-0.119023333333333	0.560413476606931
C12orf57	0.276722521079476	0.273084871715238	0.0157113930255471	0.982266426585123	0.151168000000000	0.524210311918454
MRC2	0.276710050901901	0.273084871715238	-0.264384931726191	0.544042395849675	0.418781000000001	0.128957627033936
LENG8	0.276600030277999	0.273494044376041	-0.0773828644851428	0.90079793355475	-0.0158059999999995	0.963537990151403
DHX9	0.276482029530845	0.273864483394364	-0.0472469945511493	0.943981149721771	-0.0716219999999982	0.760989915219907
NPY2R	0.276399977381475	0.273949517052422	-0.0225604633869046	0.97536722461972	-0.0530016666666665	0.821587043214193
ID4	0.27639519149413	0.273949517052422	-0.161593831229809	0.758955920988521	-0.0736579999999995	0.735779719565421
CYBRD1	0.27636568269638	0.273965160991352	0.383682870715382	0.293447764611617	0.100620333333335	0.688145991467449
PICALM	0.276335594224137	0.273989112071959	-0.168269601578415	0.747723416813184	0.0377346666666679	0.883189085233712
JPH3	0.276206833217872	0.274418607833627	0.0669402998276394	0.915145594114645	0.0875630000000003	0.74511187686951

CHD8	0.276066334303868	0.274729601717233	0.292158247129284	0.484381575140535	0.00434866666666735	0.991175862027014
RYK	0.275900365274443	0.275313159119075	0.0166542952165424	0.981482946825001	-0.0397913333333319	0.910190419163007
PPP3CB	0.275878349515979	0.275335983646510	0.180928527516278	0.721866914445283	-0.165155	0.476702574318922
RBM6	0.275818233654462	0.275527091439993	0.160800113916711	0.761016253411733	-0.0363543333333323	0.88529622507326
POU5F1P4	0.275767080855979	0.275674541960369	0.0387229690292259	0.954263089081373	-0.144801333333333	0.596725125925172
DIP2B	0.275751179459063	0.275674541960369	0.257714436544979	0.56255485087873	0.0447330000000011	0.872930585008323
IMPDH2	0.275600012285327	0.276119614938647	0.356849525928025	0.345138088652208	-0.157056333333332	0.441705375012433
ZNF410	0.275423164416544	0.27630674073912	0.179895336586127	0.723716289903531	0.0225253333333342	0.933518025413783
HTN3	0.275399877287814	0.276335425473387	0.0425644397603755	0.95117598189383	0.0152710000000002	0.95784037718285
LPHN1	0.275134726734295	0.276963133136439	-0.0419122857916643	0.951989245458556	-0.237238333333333	0.254350940121719
SLC35F2	0.275068385949121	0.276985553361606	-0.0650456963611591	0.918489100363712	-0.192020999999999	0.32370226799913
MBD3	0.275023660214477	0.277109426705334	0.320877541783907	0.421618886526906	-0.0630203333333327	0.779668393144765
GIP	0.274950248742765	0.277360534160196	0.328767299531926	0.406249092981339	-0.0989543333333337	0.646934482100399
ATP2A3	0.274895002443228	0.277456846907581	0.0982479936968633	0.868703615287103	-0.0201603333333321	0.949287545570912
NFE2L2	0.274841831860157	0.277618370234316	0.00884249751881166	0.98946753270812	0.174096666666668	0.365181410092519
HDGFRP3	0.274746538361	0.277675265971723	0.146941804660881	0.78589154083134	-0.0440939999999986	0.863199007777634
ARL14EP	0.274579048576685	0.278156946255882	0.163367309756554	0.755630158946364	0.128574000000001	0.568393891086758
GSTCD	0.274569793168514	0.278156946255882	0.173172394048804	0.736877912871223	0.0440016666666683	0.903274690789156
TNFSF13B	0.274436589678758	0.278674660494381	0.253733333852795	0.567705016149998	0.185492666666667	0.459936388684371
CCDC94	0.274335143613678	0.27900921090713	0.0971136055390235	0.870020870354202	-0.251887333333333	0.212222064067543
PLAT	0.274305568503065	0.279034326966251	-0.115725681974943	0.842992311631688	0.0293513333333335	0.927888560840831
BCLAF1	0.274230490978594	0.279169417322417	0.129715536026370	0.813305249846504	-0.0955599999999996	0.666642292286073
SLC12A8	0.274186739067757	0.279169417322417	0.270618689186492	0.529513496698646	-0.0855396666666661	0.717743913529804
SCNN1A	0.274160781643015	0.279169417322417	0.428230169969219	0.210915834110259	-0.164232333333332	0.551352991056569
UTP6	0.274124784778918	0.279169417322417	-0.103694470632389	0.861872724387044	-0.401674999999999	0.048170505761132
SPEN	0.273952935020449	0.279748262944542	-0.312654558182052	0.439245998265575	0.0592486666666666	0.830987625642722
TRIM45	0.273944760610652	0.279748262944542	-0.0626443891131481	0.922350559812086	0.340100000000001	0.124096550131011
SAMD4B	0.273908287114221	0.279836451086049	0.0530576487797497	0.936712858731269	-0.166576333333332	0.417596927032144
HTN1	0.273841360446914	0.279911632251395	-0.0133160145722712	0.984201843421075	-0.0192543333333332	0.944428790510578
CNN1	0.273819048282762	0.279936737090994	-0.15461070835514	0.771310656070265	0.101369666666667	0.669391045681572
USP11	0.273779790515865	0.280009528923005	-0.099632564385634	0.867603998426271	-0.0206283333333326	0.938282767814398
IPPK	0.273630006971546	0.280333823392464	0.533533072410431	0.0759909410633651	-0.606616333333333	0.0185304620090284
AFAP1	0.273580451703145	0.280362408714442	-0.394542211261345	0.273928897734692	-0.0887619999999999	0.688145991467449
C17orf96	0.273573623474008	0.280362408714442	0.0752628564034941	0.904173044514532	-0.134013666666667	0.551352991056569
GLG1	0.273416143586904	0.280916982884072	0.265983825856106	0.539310262140605	0.113253333333334	0.609972059115841
RNF24	0.273337115461393	0.281038660449459	0.205684262109296	0.672625944768829	-0.206646333333332	0.274121181062751
LINC00238	0.273336758734917	0.281038660449459	-0.0173835523961301	0.980670285500112	-0.0113049999999997	0.972678883626955
ACYP1	0.273322330562907	0.281038660449459	0.259174690800480	0.558100878511014	0.0384546666666669	0.891410328517388
MMD2	0.273169336030723	0.281478607990865	0.400936901138567	0.262138265619497	0.00168533333333379	0.996390456305606
REL	0.273159950901847	0.281478607990865	-0.281725970513327	0.505724230030045	0.00683266666666799	0.986350009478474
GNAL	0.273159336035256	0.281478607990865	0.0784767673143257	0.899281198071059	-0.0486319999999997	0.865083643091658
DBIL5P	0.273157413079661	0.281478607990865	0.0124533113578874	0.985220352735461	-0.100757000000000	0.656324975591247
SCNN1G	0.273130585514577	0.281524236043837	0.133699583579498	0.808933084408149	-0.0078806666666657	0.981191596773742
TAF4B	0.273072513561529	0.281679791338077	-0.0150325396488743	0.982921609770846	-0.217255999999999	0.461494497197158
RETNLB	0.273022231869991	0.281679791338077	0.175573607729295	0.73187914820204	0.149938666666667	0.479067209456976
PIBF1	0.272960187170571	0.281679791338077	-0.108616037019948	0.85395367181085	0.0839513333333337	0.73899161599654
ZNF107	0.272946291542289	0.281679791338077	0.0417364930136154	0.951989245458556	0.0714716666666675	0.754488794029141
TBCB	0.272548385263074	0.282926093852729	0.00859034049741178	0.989535713399827	-0.0725383333333317	0.772520347452753
HOXC8	0.272535465053057	0.282926093852729	-0.0235797014381729	0.975348968561756	-0.0593383333333327	0.824700707442131
TIGD1	0.272350012034820	0.283370573178198	0.348400020601857	0.361311804770494	0.115709000000001	0.596924734519002
CLDN20	0.272176110612126	0.283795991704076	-0.184815545119292	0.716020884288485	0.00954400000000035	0.978192116090894
FOXN3	0.272104096318803	0.284045462030920	0.374248158214801	0.314207741629911	-0.126937333333332	0.600950363504414
EIF3D	0.272061960451612	0.284160572964075	0.208603918872301	0.666278181721133	-0.0102366666666669	0.974895360062947
FBLN1	0.271893540946975	0.284547098847854	0.172285689737713	0.738567943867944	0.249459333333334	0.348661840021751
AMMECR1L	0.271889696088269	0.284547098847854	0.229096936960610	0.62075895683145	0.00556066666666785	0.988133186272453
FERMT2	0.271876817745821	0.284547098847854	-0.144066857768264	0.79159060381656	0.504346	0.0229237543527331
DDX5	0.271813310965020	0.284745471160953	-0.0570226897570575	0.930784376757936	0.0334366666666682	0.892414900592065
LINC00341	0.271780516399717	0.284757447108052	0.0990215828649583	0.868042453226125	-0.134433666666666	0.523041310312964
PLAUR	0.271578953863378	0.285156355419801	-0.223118121687094	0.635247978070818	-0.00206399999999878	0.995621063297892
RASGRF1	0.271576357173026	0.285156355419801	0.0810673606308661	0.896361013063453	-0.106097666666666	0.664841649345171
SEC22C	0.271553320951656	0.285185769673556	-0.0400753348441458	0.953165088013058	-0.0787193333333332	0.729912257977231
ACRV1	0.271332425314745	0.285661248662547	-0.177266355261649	0.728735147897621	0.125571333333334	0.549664615421682
C17orf62	0.271216784090550	0.285915022742951	-0.0805125100680101	0.896949986708202	-0.0899906666666661	0.704417585200123
SBF2	0.271202053734839	0.285915022742951	0.445314086686936	0.181246002449877	0.0930143333333344	0.708811767049238
RPP14	0.271193789098475	0.285915022742951	-0.0459966553419151	0.945364757579857	-0.00247999999999915	0.993756838831997
TRPC4	0.271095106886181	0.286063164085840	0.190468200406504	0.703993799601654	-0.056571666666666	0.83023589034143
DOCK10	0.271062165108589	0.286063164085840	0.0411167208981435	0.952102539249605	-0.0702139999999999	0.825289459849152
ZNF518A	0.271008401702883	0.286083006881321	0.457138590515637	0.166847523900473	0.161571666666667	0.488602547609567
ZNF24	0.270988398724913	0.286099053536704	0.00770715818952455	0.990405674923721	-0.113046999999999	0.595031194861729
PSPC1	0.270897383082553	0.286317256158407	-0.134232203765932	0.808009477165608	-0.00866433333333224	0.978037992435615
C1orf174	0.270733779034193	0.286506937713597	-0.417475516857856	0.234235464483992	0.0440500000000001	0.883478349705756
HOXC10	0.270576310459830	0.287038553947308	-0.0423569254972719	0.951504349503947	-0.0738286666666658	0.841143605305531
LPAR1	0.270318084590686	0.287794215341129	0.485855141100596	0.131934855037098	-0.65339	0.0312984093958667
RSPO1	0.270278691551226	0.287855999650776	0.338039707657135	0.385233273277464	-0.108622333333333	0.793132439192351
ITGB4	0.270271648327231	0.287855999650776	0.194083468647418	0.696755619051339	-0.0562126666666668	0.823248725853867
SORBS1	0.270235792467628	0.287944218610916	0.198919571545530	0.68526131681093	-0.106196333333332	0.772520347452753
EHMT1	0.270183354969294	0.287958980637516	0.187272151827279	0.709833349594307	0.0702106666666685	0.745703398753986
AZI1	0.2701506009059	0.287979732852095	0.467218766869816	0.151936428121882	-0.475042999999999	0.0285444246472189
IL26	0.270114292413239	0.287979732852095	0.105455811717504	0.859800688625324	0.0802380000000005	0.730330043083783
SEC61A1	0.269971833549589	0.288187997457741	0.0105883419953727	0.98774587825743	0.00145866666666693	0.99628178244892

SON	0.269969113810835	0.288187997457741	0.139941286306098	0.7976681602188	-0.0935469999999995	0.667050322639065
FBXO31	0.269936809993851	0.288260317969474	0.513072783389104	0.0967475930522153	-0.344537333333332	0.118156637757681
GPR18	0.269907874992518	0.288317379657705	-0.179823024103466	0.723850002962143	0.00583033333333355	0.98764466753384
POC5	0.269846731733931	0.288446408052970	0.219004481968875	0.641856878959206	-0.200685333333332	0.407919803052257
TMCC1	0.269803582448688	0.288519836413787	0.159742136529886	0.763150272733332	0.203331	0.364462438552017
MAP4K2	0.269632566068476	0.288825245649515	0.132815630726114	0.809588765729788	-0.232180000000000	0.365126455342703
SLC22A16	0.269597238556801	0.288888635170179	0.0259022323212614	0.971054398685485	0.0429736666666668	0.875624463432848
MAL2	0.269578732482806	0.288888635170179	-0.0738395605307536	0.905506084101768	-0.624390999999999	0.131549958663763
ITIH3	0.269537255276521	0.288888635170179	0.0746212242776031	0.904649210128014	-0.182184	0.40500623952892
NEXN-AS1	0.269537015350016	0.288888635170179	-0.159747778584486	0.763150272733332	0.00465100000000044	0.988942941807152
IGF2BP1	0.269471087842890	0.289113939133	-0.133644760993913	0.808933084408149	-0.169720333333333	0.386293615344476
FBXO11	0.269421643292967	0.289140435618476	0.167355339427986	0.749061933609388	0.105534333333333	0.617967324583825
BDNF	0.269394681011183	0.289164795200313	0.0674403052916113	0.914318618000915	-0.0766953333333326	0.724603790871296
LMNB2	0.269363894088732	0.289230618452131	0.191242291743014	0.70194618646891	-0.170747333333333	0.421535331289692
RRN3	0.269179392165740	0.289847143042419	0.085985080299079	0.888306347447303	-0.153351666666665	0.436241738226451
ZNF627	0.269095855176154	0.290132534928617	0.222528581315856	0.635668764102271	0.353747333333334	0.190246103576351
KLHL18	0.269067816199877	0.290132534928617	-0.244192248104581	0.588660529799743	-0.411319666666666	0.0479590226714844
NBPF1	0.268752414275843	0.290976368806032	0.108489348402969	0.85395367181085	0.120593333333334	0.543819286485342
SNAP25	0.268705446336874	0.290989854084311	0.132687496969009	0.809646895498189	0.455987000000001	0.0351167468854759
CEP57	0.268662854387585	0.291088177683084	0.0479054949517679	0.94314236584312	0.150536333333335	0.446756640099031
TRAF3	0.268559819882155	0.291222224339989	-0.300387566144501	0.465824202471203	0.179988333333334	0.384248044508942
SRRM2	0.26851316997144	0.291326323407385	-0.100882813077393	0.866005093242444	-0.0256473333333326	0.931859006463093
ADAMTS5	0.268439529246633	0.291529605890928	-0.139604007990150	0.797957231657366	0.0177706666666668	0.964514058161652
CRYGC	0.268436154205342	0.291529605890928	0.416022458386781	0.234713120194856	-0.156976666666667	0.462293374281597
TSPAN32	0.26841912650232	0.291533235283336	0.080466405681302	0.896952038996626	-0.0262686666666665	0.926170496188608
PHLDB1	0.268359465790052	0.291657423511015	0.381720313380832	0.297058043587116	-0.0883353333333326	0.736406752959809
ISM2	0.268191738735126	0.291705608323769	0.208367709456618	0.666751401997762	-0.0343089999999993	0.891410328517388
ALCAM	0.268147948869635	0.291705608323769	0.249751288821164	0.57536723546666	0.167580333333334	0.721528696907774
SPRY4	0.268118671603164	0.291705608323769	0.100227423653017	0.86681045025669	0.404852000000002	0.0698861985917494
UBA1	0.268112431033128	0.291705608323769	-0.0200179646868664	0.977166445018469	0.0546966666666677	0.831328608591573
PRDM16	0.268105884119598	0.291705608323769	0.321545630053097	0.420418265925367	-0.193906666666667	0.343729765439488
ETV3	0.267799197977741	0.292515441660339	0.132055533266976	0.810300414153187	-0.114354333333332	0.575589336285762
HSD11B2	0.267753081584467	0.292578523977335	0.568001630021656	0.0464030100952796	0.0417413333333337	0.87921343891193
PLD6	0.267694830084957	0.292770999421345	0.210687190545656	0.66164244512346	0.150957000000000	0.449436921437102
NUMA1	0.267599717607025	0.29305833272293	0.0220478195622528	0.97536722461972	0.0408150000000015	0.885075170442055
TMEM45B	0.267525189093541	0.293144590401814	0.0680884664477244	0.912640418606148	-0.111381666666666	0.765086485876632
SENP1	0.267512245581349	0.293144590401814	-0.106883643987079	0.857688820254626	0.104210666666668	0.639000698768934
FOXB1	0.267374047694827	0.293278884939845	0.222611203270351	0.635668764102271	-0.0472289999999997	0.870662337224757
SLC47A1	0.267123854806944	0.294120431578585	0.425509483285830	0.215369960032963	0.248227000000001	0.271985858153698
FEM1B	0.266763444111794	0.295059712641432	-0.100809694776369	0.866035166924059	0.130895666666666	0.516635548538775
RBM34	0.266743923973064	0.295059712641432	0.0327857664420848	0.961351562552925	0.0744086666666674	0.748799811961987
EDEM1	0.266670269279577	0.295105795680037	0.160660692086418	0.761367458467566	0.0866488333333343	0.691482999769299
NTNG2	0.266647310152128	0.295105795680037	0.261559191416750	0.551436731196274	-0.452181	0.117157637576458
THUMPD3	0.266505744856425	0.295268954114020	-0.244152778744606	0.588785864927734	0.0889396666666677	0.675221998008387
CALML5	0.266481608696221	0.295306096287679	0.34554012843835	0.367445209343266	-0.0442096666666654	0.904018282943947
ACTA2	0.266421686212035	0.295433963819217	0.091818258096728	0.879153312143409	0.206239666666667	0.683149536498155
PCGF5	0.266335012208290	0.29548643514339	0.429269926564886	0.209103669076588	0.113498666666668	0.588590281849842
CLASP1	0.266298267533280	0.29548643514339	0.280365567367699	0.507669977518963	-0.0127230000000003	0.967460065937822
HIVEP2	0.266298020005439	0.29548643514339	-0.0833968131715314	0.892833228561937	0.082277000000001	0.86098720418783
CLSTN2	0.266235516316884	0.295582735573979	-0.166009280377117	0.751830402832405	0.178092333333334	0.40500623952892
STON2	0.266216592978545	0.295582735573979	0.0968165921733256	0.870711896934693	0.157740333333334	0.522830837265953
KANSL1L	0.266132738288353	0.295593460866941	0.49925653973202	0.113777333040600	0.0976400000000004	0.715853170342195
FNDC3B	0.266130626596906	0.295593460866941	0.114144629489708	0.844272085764738	0.249943333333334	0.203479260516837
KIF1C	0.265986044406221	0.296184639719317	0.288697359255659	0.489813374112312	-0.397648333333332	0.133094907391771
FAM107B	0.265968459442823	0.296191935140515	0.369426703147863	0.323285574998587	-0.0746306666666666	0.727809376593958
MB21D2	0.265795650800536	0.296653962586001	0.350974728698337	0.356846278147136	0.54969	0.0300090638729873
TBX2	0.265736056523651	0.29674654579056	0.388348087362051	0.284913841222491	-0.110429333333333	0.635069698575895
VAX2	0.265698226086314	0.296841270277965	0.242952978691598	0.592178030679976	-0.169237666666666	0.527320166803475
RIPPLY1	0.265643745751968	0.296841270277965	0.0981598484492153	0.868852648210739	0.0814303333333338	0.762981436878551
NUP88	0.265615916073518	0.296841270277965	0.246136227047424	0.582912574644681	-0.0552849999999988	0.855857615773956
C18orf12	0.26559582433172	0.296841270277965	0.210363118560038	0.661841800852107	0.0333236666666667	0.896557410231352
XXYLT1	0.265298285321054	0.297848384622863	0.39673461977893	0.270862229167822	-0.0659183333333319	0.766497698403098
ADD3	0.265254394203051	0.297874606857331	0.161655102932331	0.758955920988521	0.566660333333335	0.0402148307844276
C11orf30	0.265236009070965	0.297874606857331	-0.0468603708412505	0.944274559688373	0.114562666666667	0.587066400015764
BAI3	0.265216573588454	0.297890728389287	0.129240450804532	0.81476637011684	-0.163733666666666	0.438602663638137
SNAPC1	0.265166702753503	0.297909067355924	-0.0709958044016551	0.909387602167883	0.239706333333333	0.279049509382533
C14orf132	0.265117005942780	0.297909067355924	0.0682340620467558	0.912364243071055	-0.229179666666666	0.234508116088724
NOTCH2NL	0.265035213427919	0.298066156313083	0.166712525504727	0.750443995356965	0.135369666666668	0.578139856834541
KAZN	0.265014202396291	0.298089684585174	0.48112489599331	0.136346306110762	-0.149881333333333	0.48712543076516
RPL38	0.2648264697318	0.29870138049837	0.071159740570118	0.90935479990339	-0.0262633333333330	0.924482212111412
SPARC	0.264818147294185	0.29870138049837	0.252111427623237	0.570473184967855	0.128690000000000	0.535492155031224
CRKL	0.264736677270776	0.298952203302698	0.0459999173553444	0.945364757579857	-0.0451596666666656	0.869860418086136
FAM172A	0.264642855135442	0.299217940058029	-0.0781215510312825	0.899652641295827	0.0268430000000002	0.920295464978618
MAPK1IP1L	0.264545821328197	0.299298016235916	0.082997512823047	0.893446201797764	-0.0025799999999996	0.993756838831997
SEPHS1	0.264532541975065	0.299298016235916	0.408801723527556	0.249852262368516	0.00163433333333359	0.99597023247862
DNAJC9	0.264495510233559	0.299298016235916	0.384541828464324	0.291180853572672	-0.0224323333333332	0.934016415336754
ALDH1A2	0.264461055786069	0.299298016235916	0.266099439161226	0.53910027569054	0.0777203333333336	0.721528696907774
TAF9B	0.264420854157698	0.299298016235916	-0.121724080382263	0.83097116342043	0.170186333333335	0.418637913017074
DHX36	0.264403238123075	0.299298016235916	0.245018432869416	0.58629105649071	0.0929076666666666	0.654911833752806
LOC148696	0.264334699772443	0.299468397349935	0.0710947551308447	0.90935479990339	0.0843073333333334	0.788086071683574

APLP2	0.264290808358875	0.299598161529182	-0.132246258806728	0.810300414153187	0.239006666666667	0.233528575266801
ZNF202	0.264271648674966	0.299613477212094	0.0693091938214078	0.911837389834658	-0.00480133333333287	0.988133186272453
BICD2	0.264243220313550	0.299621317307826	0.09683236759871	0.870711896934693	0.0870593333333332	0.683303078753456
DYNC2H1	0.264226904967190	0.299621317307826	0.00128311479619099	0.998348925592729	0.155957166666667	0.589826330962094
CDR2L	0.26410661061345	0.300010864533983	0.222841186193255	0.635391356864593	-0.0542126666666669	0.81725103791607
HOXA13	0.264024853104970	0.300242933136423	0.185697511220267	0.714043644840406	0.129757666666668	0.524845938106282
EPS15	0.263901527356162	0.300310190487752	0.252495913624416	0.569562951596314	0.362861000000001	0.143222645831961
ASB1	0.263717262444182	0.301009014346210	0.399436352396198	0.265307951044717	-0.212290333333332	0.371858613427745
SBK1	0.263649533111005	0.301166025338068	0.261868105847613	0.550431790513942	-0.0891563333333333	0.676024903789639
GNAI2	0.263636006006164	0.301166025338068	-0.188652884726641	0.707361603786947	-0.0244878333333327	0.927461430814143
RBM12B	0.263462532718799	0.301751709711737	0.229880887943050	0.619381301776937	0.0980320000000007	0.677570340702693
FBLN5	0.263110313902561	0.302508798032481	0.405167220978628	0.25480455106032	0.0305446666666681	0.907646594344291
EMP1	0.263093091924606	0.302515529221596	-0.31419818613386	0.437675147805387	0.351391333333334	0.0720351459642534
DACT2	0.262943202811336	0.302993337241414	0.283132564417643	0.502129505146473	-0.158995999999999	0.580733005287472
C2CD4C	0.262885273593821	0.303189879295787	0.159411994111756	0.764209767000699	-0.151053000000000	0.657217907037306
SNORA65	0.2628523247979	0.303269995557694	-0.0169986457657777	0.98105020869911	-0.126689666666667	0.60814016771842
CDHR5	0.26282956072825	0.303281519064891	0.194587374492158	0.696017817587257	-0.0938326666666665	0.671492528470281
SERP2	0.262798023476388	0.303281519064891	0.0642814139828459	0.919923865590619	-0.0636246666666659	0.791014715933453
PTPRF	0.262733220644386	0.303457953920954	0.250783288513701	0.57315146228686	0.276836000000001	0.162908301904803
RNF122	0.262587221266942	0.303992375993136	0.168933059519910	0.74568001679609	-0.0774053333333332	0.815309621716298
SAMM50	0.262468633301160	0.304104990420902	0.366847044381692	0.328151450414815	-0.0492129999999986	0.855317148872886
XAB2	0.2623929105521	0.30433808747513	0.358735432465907	0.343089107932071	0.00656466666666741	0.98349947509937
BHLHB9	0.262248434264352	0.30472248274705	-0.240923591252111	0.596648444172109	-0.152150666666666	0.447660795892047
MBNL3	0.262164864372420	0.30472248274705	0.439887190345582	0.190324143836679	-0.338953	0.167277848166228
FOXS1	0.262152698854909	0.30472248274705	0.237104673269058	0.603531426598988	-0.196806666666666	0.528409919089401
ZNF136	0.262101892847621	0.304863271426514	-0.0649509905768009	0.918489100363712	0.116677000000000	0.632532249588464
CCDC28B	0.262086295069093	0.304863271426514	0.137766716835344	0.801903553301576	-0.152386333333332	0.446756640099031
CRLS1	0.261928446179663	0.305087699133631	0.287556224960692	0.493085126937196	-0.0946903333333325	0.66807888867384
PTAR1	0.261904942104837	0.305115606637337	0.0131863261970542	0.984482935754244	-0.042978	0.87629402535202
BTN2A2	0.261891113988902	0.305115606637337	0.105063464220243	0.8606536389692	-0.0934106666666664	0.657217907037306
KPNA2	0.261864249418486	0.305164491909189	0.0847455519147162	0.891309254245872	0.0482133333333338	0.842627267790666
RASIP1	0.261833634835766	0.305164491909189	0.097711767685441	0.8692242996093	0.0425406666666669	0.86011149680202
STAM2	0.261572893309961	0.305992373833073	0.161451506088044	0.759200086793837	0.219411333333334	0.285041822446361
CDC42BPA	0.261547161507980	0.305992373833073	0.133315180445076	0.808992949393043	0.131235000000001	0.528501553687416
KIF18A	0.261463776467782	0.306309720964855	0.0497887721940337	0.941458038227918	0.0560616666666686	0.862509125961694
CDH11	0.261373869372194	0.306511098771663	-0.0343662998779112	0.959538629769734	0.275899666666669	0.1409133773217
TRPS1	0.261294915318049	0.306734681541711	-0.0219275578164495	0.97536722461972	0.067193666666667	0.800532222303294
ZNF283	0.261275234092036	0.306753606222885	0.102746114237529	0.86257932725575	-0.108168333333332	0.630743046685015
CLTC-IT1	0.261216943029666	0.306931130850923	0.0195290619222084	0.977673457110824	0.176375333333333	0.590841338570121
RAPGEF2	0.261206124486456	0.306931130850923	-0.289537493564496	0.488870852256136	0.146274333333334	0.621414476984274
CALML3	0.261028807963119	0.307543737215194	0.1227932278491	0.828721578450338	-0.169336333333333	0.489276198144674
FAM65B	0.260990408327319	0.307618758899095	0.145715017265549	0.787804113309913	-0.0472146666666659	0.902194685260117
ANO3	0.260943992268743	0.307618758899095	-0.282693987696255	0.502643073626719	-0.0231946666666659	0.931859006463093
ARHGEF6	0.260934478528241	0.307618758899095	-0.160023881630586	0.762902034786947	0.342183333333333	0.0802641563431964
RCHY1	0.260907491009558	0.307618758899095	-0.278230209416994	0.511880330989176	0.0872215000000006	0.686041857997316
EXOSC6	0.260872192612882	0.307618758899095	-0.00963290549751215	0.98909166966601	-0.0524023333333323	0.818849076927228
CCDC36	0.260792248681632	0.307715923622699	-0.0724311653079057	0.907556175729082	-0.0727599999999995	0.825896989586212
KDM4A	0.260758175214658	0.307715923622699	-0.00636110680254834	0.991853352023739	0.0351340000000014	0.889452949584609
AHSG	0.260664407691222	0.307939999079416	-0.0668890848345367	0.91517239775041	-0.0814186666666664	0.721236226686999
S100A2	0.260622268311611	0.307986903403344	-0.486912676016639	0.130936656112786	0.0489903333333333	0.85409823183973
ADAM10	0.260311274905687	0.308937341959339	0.118893875331925	0.83702538133036	0.0733263333333339	0.748388402768556
PLA2G12B	0.260258979446229	0.309110392729097	0.187718768255447	0.707361603786947	-0.0292063333333331	0.911623909907545
CYP2C8	0.260111024687442	0.309661332766142	-0.00981289827193173	0.989090482697721	0.400848	0.17383106625275
RNF214	0.260032711986654	0.309805014409379	-0.213384576527027	0.654550434157114	0.178596666666667	0.385678201128102
PML	0.260018300797518	0.309805014409379	-0.371229718652152	0.319831054720447	0.079348333333334	0.733776177121002
SHB	0.259921560184755	0.309885300840273	0.0780202403563183	0.899705053748212	-0.219280666666665	0.346020200924341
TROVE2	0.259900124533079	0.309885300840273	0.200906542235927	0.68016888532461	-0.0697306666666658	0.772139923817113
BRWD1	0.259892397096489	0.309885300840273	0.183326846874773	0.718067440349544	-0.0179136666666661	0.964562526717628
ITGB5	0.259671452706495	0.310635305762062	0.376740381978327	0.308846814162417	0.440714333333335	0.0647596980686847
FAM168B	0.25963071396186	0.310680967928277	0.203885920055796	0.675615338672331	-0.0473856666666658	0.845737357656109
AKIRIN2	0.259429826393453	0.311527984386604	0.241732317139881	0.595201219333254	-0.147240666666666	0.528409919089401
FHL3	0.2594206721194	0.311527984386604	0.124584148049937	0.824974271393593	0.0511486666666666	0.827303077786372
SSR2	0.259182829432359	0.312287379715134	-0.0220556897792028	0.97536722461972	-0.0296466666666649	0.90945379489823
RAB13	0.259125806351060	0.312484313503126	0.0084422752108413	0.989783000376216	0.0109360000000008	0.972209175539945
DKK1	0.259032539812898	0.312690301332064	-0.204072519030045	0.675344930297902	0.161846000000001	0.415110347922019
RANBP3	0.258996394309012	0.312690301332064	0.34316993619499	0.374497295817252	-0.0801973333333327	0.707538975864338
PROK1	0.258404423920858	0.314735510359053	0.147447939594492	0.785497496933202	-0.891348333333332	0.0140473413958925
TMEM156	0.258386506731754	0.314746991944476	0.0619934177648403	0.923034515346352	0.093643666666667	0.703580291685151
WTIP	0.258347611391318	0.314773872951073	-0.0447264872794504	0.947776877450408	0.0972769999999998	0.650393862025154
NDP	0.258307671401882	0.314773872951073	0.104858793036811	0.86067831050617	-0.473901666666666	0.0351167468854759
MTA2	0.258303360963654	0.314773872951073	0.088954726560293	0.883865906764221	0.0230800000000006	0.929907694303715
PABPC1L	0.258240384904421	0.314826064051786	0.153441971939015	0.773731863302057	-0.109448999999999	0.612347714154769
KIAA1984	0.258188121506582	0.314826064051786	0.219695811206826	0.641035290381005	-0.110935333333333	0.616472837170869
PCDHB7	0.258168545652158	0.314826064051786	-0.136318962094622	0.804864448119512	0.044160333333333	0.850980105164978
C9orf9	0.257990540761164	0.315453985709264	0.0342658703084458	0.959623239761825	-0.0655716666666662	0.790848042413284
RPL28	0.257948021315768	0.315583168968772	0.119781924027061	0.8354985445494	-0.0947046666666663	0.65865472196943
ITPR1	0.257598140536540	0.316812785045953	0.21888033516379	0.642137448592891	0.153935666666668	0.578893615511221
SPINK6	0.257581244006435	0.316819646483915	0.251156023651431	0.572882168047464	-0.231153333333333	0.28487334684302
MAP4K4	0.257488951731006	0.317010623839367	-0.122286013030419	0.829569971668386	0.097646666666668	0.750936504909256
UBA3	0.257479543587039	0.317010623839367	-0.00509348643177728	0.99251028338798	-0.195136666666667	0.29986081041577

NUDT11	0.25742251895509	0.317200478226005	0.36173816312083	0.338556116268406	-0.0553996666666652	0.872002060489333
KDELR1	0.257376878279392	0.317200478226005	0.169098739840193	0.745220283795449	0.101880000000002	0.625258262671142
YWHAE	0.257362683114363	0.317200478226005	-0.0437248530998956	0.949043721172002	-0.191023333333332	0.374814376822818
STK32B	0.257198151356823	0.317693232569304	-0.185528622473041	0.714210278087368	-0.00264533333333324	0.994738356997797
SLCO6A1	0.257104979991436	0.317819558632523	-0.333514055736039	0.396821740334178	-0.130803000000000	0.580149967309238
BRD4	0.256968471496737	0.31798129409601	0.351756583158849	0.354483223108826	0.124588666666668	0.549659494702918
PHIP	0.256850717640683	0.318257413064343	0.233555937405255	0.610436189314218	0.043005000000001	0.865571667607644
NR2E1	0.256833921624305	0.318257413064343	0.0425985019542868	0.951071128215665	-0.0559316666666664	0.819757926662461
DLL3	0.256818052747566	0.318259689898599	0.12874861490624	0.815407702581246	0.00979300000000084	0.974451420920923
METTL16	0.256784998678796	0.318333230358188	-0.148296838488813	0.784080588385358	-0.160635333333332	0.465948865963889
NOC3L	0.256629308171405	0.318796542246302	0.0355728951537751	0.958689318480951	-0.223962333333332	0.305101612380486
TTPAL	0.256561591203285	0.319027391751791	-0.295593649883026	0.478008201657401	-0.289876666666667	0.203287190925869
LPAR6	0.256430043384365	0.319384772093522	-0.375522738298551	0.311514528071764	8.50000000001567e-05	0.999729972650062
SMARCC2	0.25633126180375	0.319712125535400	0.146275835103356	0.787339147446412	0.0323816666666686	0.896906769316595
NXT2	0.25626706038177	0.319873192294108	-0.201001942909477	0.680143077995217	0.104055000000001	0.621414476984274
FAM198A	0.256213990051369	0.319980735493157	0.340096292852527	0.379571667536824	-0.0612563333333338	0.784674530137123
ARIH1	0.256112624412403	0.320066892990322	-0.115108112172238	0.84409286304878	0.0598066666666687	0.808204808435554
SCN2A	0.256090134264755	0.320066892990322	0.214255918681834	0.652999504750719	0.0119900000000005	0.970094446214144
DOCK7	0.256082060290482	0.320066892990322	0.138744368920709	0.798938201476624	0.0643370000000009	0.814814057921361
OR8G2	0.256019791627829	0.320205382008313	-0.0717539552425686	0.90837606217316	0.154446666666667	0.440315785128901
HSDL2	0.255960038498077	0.320210590768495	0.222932714470637	0.635247978870818	0.00499566666666675	0.987937064305561
SH3RF2	0.255951384037609	0.320210590768495	0.119388426303137	0.836099954217028	-0.0580733333333331	0.797384658979621
QKI	0.255896966114075	0.320355556669308	0.150899254853802	0.778253782413218	-0.00547466666666543	0.987577907335088
ADAM17	0.255767884898394	0.320799313692974	0.179085057683084	0.725727384118373	-0.0430729999999984	0.856640814502011
DCAF16	0.255701896773730	0.321043633107275	-0.174311900489758	0.734267769889068	-0.00189566666666575	0.99597023247862
ZNF397	0.255655503311128	0.321049970985946	-0.191835892791798	0.701517842429758	0.122814333333334	0.549323798856043
SNORA37	0.255646727173377	0.321049970985946	0.188849934463748	0.707236456264125	-0.107520666666666	0.612347714154769
TGFB1	0.255528092723251	0.321438795632009	0.00810983000540452	0.990260061305292	0.116048333333334	0.635069698575895
SPOP	0.2555090532312	0.321456793088018	-0.090981331220299	0.881187233358984	-0.0927266666666651	0.6846855637534
CHEK1	0.255456173068595	0.321564274013404	-0.0618898424413375	0.92306630614464	0.173255333333335	0.523174709089214
PAICS	0.255358900444876	0.321738428180403	0.187168019547422	0.709833349594307	-0.123183333333332	0.598927587414486
MAP1B	0.255320152510221	0.321777835304585	0.0063138071844197	0.991853352023739	0.195116666666669	0.370079759630929
TUBB6	0.255266029115779	0.321965387234982	0.115678676170502	0.843095614322683	0.0142156666666679	0.963537990151403
KHSRP	0.25520610963843	0.322181030739754	0.410061884821987	0.246933084622221	0.0192686666666684	0.94375604597120
CUEDC2	0.255119558699213	0.322494361786547	0.23282186958352	0.611711680668619	-0.144061666666667	0.523453039510001
TNRC18	0.255060228347886	0.322528086409901	0.181273007929038	0.721196149497303	0.0450232500000013	0.847964572928708
TAS2R50	0.255057898470933	0.322528086409901	0.291302816469152	0.486802739867898	-0.197313666666667	0.338184384427605
C12orf54	0.255035708973603	0.322561508498149	0.0966539303862185	0.870857630743202	-0.0356836666666663	0.885345988320645
ZFP37	0.254912031340956	0.323012082188945	0.0821190721317872	0.894840896036786	0.325282666666668	0.122514080403437
CAPZA1	0.254786208018034	0.323334939054748	-0.359233627873121	0.342292001700374	0.153190333333335	0.435157099583407
PAFAH1B1	0.254713920386538	0.323527808570995	-0.0823734960304966	0.894172508150787	0.0270576666666679	0.919456505549688
OTUD3	0.254661028788914	0.323636187652962	0.270338177721513	0.529999252995877	-0.215314666666667	0.310277378475854
RPS15	0.254619683288831	0.323707023007804	0.385700641359717	0.289620259399938	-0.00455666666666581	0.988133186272453
PLEKHA5	0.254605291525683	0.323707023007804	0.313358704562669	0.438100620341757	0.453719666666667	0.0351167468854759
ETV6	0.254591196602036	0.323707023007804	0.365756272430652	0.32927628703889	-0.0131569999999991	0.974451420920923
THBS1	0.254463357457014	0.324025077557245	-0.085182202051525	0.890458025676316	0.889551	0.0353807205776334
VPREB3	0.254427091745008	0.324025077557245	0.24778984794139	0.579471329472972	-0.034895333333333	0.910190419163007
CLCF1	0.254402577170380	0.324025077557245	-0.0614770867192466	0.923853031655103	-0.0328369999999989	0.902194685260117
SLC2A1	0.254366617661895	0.324025077557245	0.27981728204673	0.508808219915286	-0.118731666666666	0.615807897956118
SETD2	0.254162053186104	0.324799604431103	-0.164107568340371	0.754243267462143	0.0490133333333352	0.849233115893056
PRPF38A	0.254073663862187	0.325097605948050	-0.094790464794941	0.873804326353411	-0.0621226666666666	0.809755698910303
THBS2	0.254021345577052	0.325261277751901	0.114252419907235	0.844272085764738	0.0621930000000002	0.816337381174918
LMX1B	0.253973262776912	0.325347023875332	0.289515878078459	0.488870852256136	-0.155408000000000	0.552949876182027
FAM20C	0.25392802520606	0.325492967276651	0.105992356410815	0.85897514543105	0.100122333333334	0.653376501568506
FAM118B	0.253872502013463	0.325598779407747	0.0389466906191873	0.954136075928463	-0.00800933333333274	0.981191596773742
IL13RA2	0.25386688895148	0.325598779407747	0.154713894727750	0.771310656070265	0.457256000000001	0.0521767146109064
FTO	0.253860623020102	0.325598779407747	0.237485066758426	0.603017682513881	-0.0742099999999988	0.729912257977231
CABYR	0.253779836383486	0.325833330273798	-0.00324484743365469	0.994730189431381	-0.020826	0.938640008704917
C10orf25	0.253747487461172	0.325840066335586	0.0643018075411614	0.919923865590619	0.155875000000000	0.452494769641845
COPG1	0.253715798505873	0.325859483588445	0.0557549440553752	0.93339378096251	0.0977716666666675	0.663776173049665
HYAL4	0.253647685540002	0.325998021218746	0.233322954861673	0.610893912893531	-0.000669333333333286	0.997953056544486
SEC62	0.253643173728421	0.325998021218746	0.194774332397317	0.695696493534439	0.0663276666666675	0.777406710539424
ZNF146	0.253641740765876	0.325998021218746	-0.0555043052062628	0.933409139126562	-0.0747739999999998	0.735063355984464
PHC1	0.253598764063776	0.326081792844878	0.173821873993539	0.735246930194452	0.0559766666666677	0.809505390888975
PHF2	0.253275937395194	0.327188172189616	0.197536819969523	0.688744573728648	0.179433666666668	0.388428924555697
DGKH	0.253226853258067	0.327332273398900	-0.109446218433316	0.851952536285005	0.129593333333333	0.516040564500327
CDC42SE1	0.252904556130389	0.328483075275912	-0.228902434033156	0.621520262932208	-0.023624999999999	0.927888560840831
PYGM	0.252861328975701	0.328540043810414	0.289744307695558	0.488870852256136	-0.383062999999999	0.0895935206991096
PPM1M	0.252853443132508	0.328540043810414	-0.00378329078713218	0.994588964700772	0.140506333333335	0.536810332635776
COX10	0.252847392818921	0.328540043810414	0.110851864400852	0.849357727620213	-0.0309276666666666	0.902859284837213
MALT1	0.252694796883028	0.329064092130832	-0.355183963922095	0.347833413629182	0.0612310000000004	0.828649000874796
NEK8	0.252620763072934	0.329260849668460	0.303176896596726	0.462105017205825	-0.123293333333332	0.579412709059421
PCDH10	0.252609128731949	0.329260849668460	-0.318306763957635	0.429394034332441	-0.0794299999999991	0.761537039620744
PHF10	0.252563432972467	0.329410427473051	0.396089815300649	0.271439858675643	0.0832083333333355	0.700662810263276
FBXL20	0.252516812288707	0.329531831344002	-0.163873576877694	0.754916750929288	0.0702196666666662	0.761837433189273
RAB3GAP1	0.252478039426065	0.329531831344002	0.125640589028028	0.8236721238761	-0.130499000000000	0.541939010010167
DYRK3	0.252315501739372	0.330180189361716	0.089515513342612	0.883258404969667	-0.0223246666666657	0.948768037250156
PTK7	0.252191423822665	0.33066786584114	0.102897701875215	0.86257932725575	0.0340320000000007	0.895043648815056
RNMT	0.252170711307554	0.33066786584114	0.0469687267932333	0.944081286115546	-0.265226666666665	0.240683249939396
CORO2B	0.252115638550123	0.330852457383852	0.0393528051789935	0.953905313456582	0.0910726666666662	0.74499160363205

ITGA11	0.252046021797581	0.330908476137250	0.109058308154145	0.852975887741054	0.473624666666667	0.0474700208071304
TADA2A	0.251978038082863	0.331093799632078	0.160737810261928	0.761107376045903	0.0714193333333341	0.761257561656748
PRICKLE2	0.251874314528370	0.331474656026083	0.0499785634238172	0.941458038227918	0.210210333333334	0.51528814031066
FAM161B	0.251566657436790	0.332448310749887	0.377663622420834	0.306357482082007	0.0821240000000012	0.731303939999377
BRIP1	0.251460783445031	0.33277931959564	0.160901129082436	0.761016253411733	-0.0127586666666660	0.973686596735315
CXorf22	0.251333292566888	0.33277931959564	-0.085674215814725	0.889114985071002	-0.0019626666666664	0.99628178244892
SUV420H1	0.251284888562298	0.332815045497043	-0.106522992256749	0.858072018696671	0.0071120000000004	0.985263856374185
CST8	0.251280765839658	0.332815045497043	-0.0182008121298835	0.979327298872732	-0.0049483333333331	0.987937064305561
ID1	0.251182637697367	0.33301755680607	0.0349317127328208	0.959281756747498	-0.0060916666666659	0.987851590533963
RCN2	0.251162979652875	0.33301755680607	0.00743192581719741	0.990848272164343	0.290029000000001	0.147699085494653
CACNA1E	0.251106588283693	0.33301755680607	0.116228976178396	0.842218159791738	-0.137581333333333	0.60627373290346
ALDH18A1	0.250909113071029	0.333607110215859	-0.186470766408253	0.711403774834748	-0.201002333333333	0.327835090482928
APEX1	0.250846714574057	0.333840762042187	0.147226230486275	0.785649719908014	-0.120176666666666	0.601189290968165
FAM65A	0.250707624886055	0.334035231961692	0.173840746935831	0.735225645715852	0.227656000000000	0.307304414570957
IL17RA	0.250700778169453	0.334035231961692	-0.241948468844003	0.594959617426643	-0.111924666666667	0.625510328921698
KIAA1958	0.250640915096881	0.334113872777126	0.10700166134297	0.857671866148626	-0.0738343333333337	0.764973469318093
DNAJC18	0.250421033429637	0.334858177672006	0.0997468127811071	0.867558388686851	0.0162463333333346	0.953335531487028
PLXNB3	0.250351147871624	0.335025897492755	0.139035724151730	0.79864602540281	-0.181214666666667	0.396821451196405
SSPN	0.250301258366572	0.335198373807590	0.489961965073814	0.124999442942958	-0.0742303333333328	0.774104133965233
RIMS3	0.250268284546247	0.33528719093398	0.329481031185164	0.404377515671201	-0.0487479999999991	0.838336524351522
RCC2	0.250204340301221	0.335380627197405	-0.127580661519773	0.818717515586031	-0.891882333333333	0.00347244136732606
ANKRD44	0.250021801760579	0.335987160846791	0.422092729668313	0.222319600995852	0.277067000000001	0.158105452461472
SNX11	0.249909327791827	0.336158521554701	-0.089172867848723	0.883460564717377	0.0112440000000000	0.975988780928093
TMEM98	0.249873693034747	0.336158521554701	0.336387850093763	0.389281981918153	0.574422000000001	0.0185304620090284
GTF2A1	0.249854380052818	0.336158521554701	0.0105323830243661	0.98774587825743	0.307010333333335	0.122557841857133
EIF4G3	0.249848664744415	0.336158521554701	-0.266336233949243	0.538833471023229	0.00529166666666764	0.987937064305561
DPYSL5	0.249837199906301	0.336158521554701	0.497286967997661	0.114508695522455	-0.288058	0.223260757343954
PCF11	0.249702642520911	0.336602636259823	0.235110067244504	0.607525465293757	0.097572333333335	0.687939779688318
SUV39H2	0.249680671202453	0.3366372812408	0.0908469412302764	0.881323965029954	-0.140864666666666	0.49038083653267
TACR2	0.249453129100366	0.337171770755572	0.352552895479077	0.352560848501456	0.0586159999999996	0.870002591217953
SNHG10	0.249358380594844	0.337567926314783	0.238264781901046	0.601773482805025	0.0298586666666682	0.91962770573477
SPATA6	0.249147611674478	0.338017532217371	-0.0152515784404109	0.982921609770846	0.168954333333334	0.527527579392479
EFTUD2	0.249133253322979	0.338017532217371	-0.189377996557478	0.705512498752921	-0.0267866666666667	0.919456505549688
LGALS3	0.249020847456211	0.338407008998992	0.120082365992404	0.835124910268666	-0.0357503333333331	0.902859284837213
DCHS1	0.248981448152645	0.338407008998992	-0.179693016991538	0.724171988216416	0.199806333333334	0.294008795728502
SEC23A	0.248980188119754	0.338407008998992	0.209048493640521	0.665595909869988	0.170433000000001	0.370307138257961
KRBA2	0.248933923176361	0.338562830713635	0.0694852601220102	0.911617959392541	-0.0228533333333330	0.93433854319414
TLK1	0.248897997710011	0.338667248372503	0.185037840182687	0.715658781310868	0.188984666666668	0.426518032099216
ANKRD18A	0.248836176999395	0.338751898114942	0.336709066108440	0.38867878932888	-0.093215	0.729912257977231
CASP7	0.248744465565954	0.33913026052092	-0.0374529709942356	0.955609938182749	0.229379000000001	0.236989286061345
TUBB1	0.248682461010101	0.339219876081509	-0.275639822979446	0.518318935959533	-0.205081666666666	0.387687154733619
HIF1AN	0.248609815635353	0.339507456348909	0.32588561629593	0.409206882509236	0.0493883333333347	0.864165276005277
ZFC3H1	0.248562098027303	0.339651165457778	0.103505967343426	0.861943478503987	-0.103113333333332	0.677667606291399
FAM196A	0.248481488657096	0.339775202474396	0.078905202941784	0.899154242391172	0.0793666666666671	0.739738232644097
NCAPD3	0.248372530795068	0.339886979722670	0.0619237561060334	0.923034515346352	-0.0307896666666648	0.922697964464038
ELK3	0.248343609823553	0.339886979722670	-0.349757737491041	0.358977715627648	-0.0083013333333331	0.979527173532206
MYOZ3	0.248322261151704	0.339886979722670	0.272865025141015	0.525056200978613	0.0277583333333352	0.92469922248949
OMD	0.248259188927592	0.339886979722670	-0.0025250920621481	0.99580901762095	0.0957236666666675	0.688145991467449
NR4A1	0.248256237589382	0.339886979722670	0.141704325056025	0.795109963623224	-0.265437999999999	0.275779229553450
MINPP1	0.248256081513137	0.339886979722670	0.383676512864598	0.293447764611617	0.192294333333334	0.380431002643559
OMG	0.248221847428065	0.339886979722670	0.30075013727857	0.465222095816255	0.0335003333333339	0.896906769316595
RAB39B	0.248205808193854	0.339886979722670	-0.17715709700377	0.729164008872041	-0.0915606666666664	0.710083364777824
ZNF114	0.248161569749963	0.339999972903208	-0.277267732800445	0.514767290867356	0.024554666666667	0.956567100065073
FAM199X	0.248118508717878	0.339999972903208	-0.342243577679803	0.376457834410759	-0.161668666666666	0.423030362322111
UBB	0.248082744575523	0.339999972903208	-0.235373756419682	0.60678764664537	0.0731666666666682	0.754488794029141
PPP1R10	0.248072507240630	0.339999972903208	-0.153020355845858	0.774511250442036	0.0842720000000012	0.687448770010242
TRHDE-AS1	0.247919816301088	0.34064574862624	-0.318231719735992	0.429664745401356	0.0185810000000003	0.956567100065073
TMEM184B	0.247862265339579	0.340759184118839	-0.0640869141088216	0.920005468161721	0.0607170000000004	0.785877544359186
SRP9	0.247719793182514	0.341076741697524	0.109660983973084	0.851352116283305	-0.0635066666666656	0.773301604360926
ASB4	0.247704092163188	0.341076741697524	0.419916772257789	0.227417142008707	-0.118450999999999	0.578893615511221
SRRM1	0.247684948254771	0.341076741697524	-0.115425902126614	0.843614348588468	-0.0120533333333318	0.967256128104685
INTS6	0.247463052139024	0.341610381450793	0.173250183812700	0.736740209571778	0.156424000000002	0.564018770666257
BNIPL	0.247352000631043	0.341957363278934	-0.0740312049834814	0.905506084101768	-0.0569629999999999	0.86218518452039
SEC23IP	0.247345526565334	0.341957363278934	-0.148027134657680	0.784080588385358	-0.0051096666666662	0.987499363290182
TRAF4	0.247241045747821	0.342331640580918	0.422099254204737	0.222319600995852	0.0704496666666664	0.774104133965233
FAM211A	0.247203114532335	0.342444478350648	0.0766658348089585	0.902228403382432	0.203937000000001	0.289688235940250
COL2A1	0.247188856660776	0.342444478350648	0.303814543509127	0.460052080110635	-0.0782113333333333	0.786970336786627
SLC18A2	0.247171375176074	0.342457819324506	0.0310644030116883	0.965118873711308	-0.129269666666666	0.578893615511221
NBEA	0.247127588053687	0.342602814061242	-0.110870954407603	0.849357727620213	-0.205110000000000	0.32631478390653
KCNH5	0.247104811929904	0.342642676051148	-0.198558594178435	0.685952362965345	-0.0355046666666665	0.890073383340154
RPPH1	0.247033295253961	0.342778295450497	0.137541217544542	0.802039543563725	0.186680000000001	0.324937679238988
C11orf58	0.246980254799608	0.342969744787706	-0.0824033498052645	0.894154569859631	0.0602383333333345	0.795446186721078
CTSZ	0.246865854969970	0.343380552655733	0.0404086349194251	0.952720585102517	-0.025021333333333	0.938451042306877
MRFAP1	0.246853849257853	0.343380552655733	0.0601280419159997	0.925649366494154	-0.0753699999999976	0.73689879358434
RNF41	0.246811307547392	0.343519609417085	0.0685679949159865	0.912149839732916	-0.084248333333333	0.717966142527826
FHL1	0.246784616961127	0.343579229041867	0.111754029108671	0.848253236274008	0.177457333333334	0.370523714900868
ASAP2	0.246754183311156	0.343657631806875	0.145602217175136	0.787804113309913	0.0480220000000018	0.870489343487753
IL20RB	0.24667620139645	0.343900341644868	-0.194264731642433	0.696504707499023	-0.240394333333333	0.449950389559863
ALKBH5	0.246583469909261	0.34408921955622	0.051898793457791	0.938067483409541	-0.0468346666666656	0.861945621270498
SLMAP	0.246579397296060	0.34408921955622	0.0892356493569517	0.883460564717377	0.0311433333333344	0.903788355106891

LINC00314	0.24655812308537	0.344121784481633	0.299424229522685	0.468245148036717	0.0354994444444447	0.890073383340154
USP21	0.246506070390276	0.344234645075320	0.0295188745439691	0.967226229119616	0.0621013333333342	0.809409373518692
DPF2	0.246448943203962	0.344373033589398	-0.057737597557685	0.929758936765496	-0.0334199999999972	0.895093529187836
SALL3	0.246432053765658	0.344383649133474	0.458388565090199	0.165946301748737	-0.02990966666666	0.92590541082869
PLD2	0.246280337875517	0.344848824934066	0.00932408402817556	0.98909166696601	-0.232048333333332	0.320592704550458
KDM2A	0.246116406006739	0.345375931055354	-0.151833481299013	0.776343233798271	0.0411700000000021	0.898979600726666
ATXN2	0.246046093075528	0.345467048182168	0.194077456292481	0.696755619051339	0.0289536666666678	0.928950720764733
PCNP	0.246039251374487	0.345467048182168	0.0370228264558242	0.95664230789783	0.0116416666666683	0.9681321732097
SIK2	0.245769540357208	0.346092169442257	0.058475362692667	0.928725686060612	0.047525666666668	0.896699926823174
PDHX	0.24574599971445	0.346092169442257	0.271650312393617	0.527831579272828	0.0433533333333335	0.858648220144221
DCTN1	0.245667166427494	0.346092169442257	0.0286865726018296	0.96782691865619	-0.0343586666666654	0.891220340561155
GNG12	0.2456644565904	0.346092169442257	0.149219375738751	0.782603647278817	0.254616000000001	0.206187117033303
DCDC2	0.245621174621092	0.346235913535102	0.359179036238964	0.342395636333703	-0.0725933333333329	0.774104133965233
SV2B	0.245595128578137	0.346292880146663	0.152306945629325	0.775863712880333	-0.546447666666667	0.216591326893487
COL10A1	0.245580424930983	0.346292880146663	0.0107312004604132	0.98774587825743	-0.0582013333333326	0.791595191224138
LOC619207	0.245488680822681	0.346384505654818	-0.0701877134288314	0.910398028515729	0.0648236666666671	0.786973845403943
ZKSCAN3	0.245417980406516	0.346666429586264	0.169787549513382	0.743328907676018	0.112666666666667	0.577104991740119
ANKH	0.245397319851553	0.346696439454282	0.0500040708624073	0.941458038227918	-0.615246333333332	0.0253065800497287
MYL6B	0.24537373363187	0.346741195078567	0.0545691318276231	0.9344692810234	0.0593633333333339	0.794404527150583
CCL22	0.245356096305048	0.346756001785196	0.200797722426352	0.680489843835436	-0.0645593333333331	0.799204212638847
PRUNE	0.245335229198843	0.346787085856898	-0.131348314642744	0.811328589649422	-0.0294093333333322	0.930211818640573
WDHD1	0.245271688751235	0.346886591253061	0.0286223895550304	0.96782691865619	-0.0478543333333328	0.850364148347928
ZNF264	0.2452594882946	0.346886591253061	0.076807672233606	0.901856698918763	0.142017333333334	0.477811214517565
SCAND1	0.245243286642856	0.346886591253061	0.467169049372931	0.151936428121882	0.00425100000000088	0.989115664059601
AFP	0.245222168319665	0.346886591253061	0.196601230313564	0.690838714578657	-0.0578169999999995	0.850980105164978
YEATS2	0.245197991308775	0.346886591253061	0.237077866665229	0.603531426598988	0.192738666666668	0.407801370651148
TTC28	0.245148764643330	0.346986700150183	-0.00863396136612893	0.98946753270812	0.0941636666666671	0.739054294581317
CELF3	0.245106101507747	0.347031794328994	0.092127452449369	0.879153312143409	-0.174410999999999	0.43400381736769
ADCY6	0.245105094790172	0.347031794328994	-0.0849801095984955	0.890886878560183	-0.00554100000000005	0.987163226136842
G3BP1	0.245037390150038	0.347113991513429	-0.158477944188028	0.7661940703205	-0.162493333333331	0.426237423530267
NCAM1	0.244945253903935	0.347273278483283	0.035723257414444	0.958366440880488	0.197014333333334	0.359908305595919
CTXN1	0.244814803235496	0.347709174204466	-0.0910337252246774	0.881053992915981	0.0946343333333344	0.720336393581905
MCF2	0.244688251608824	0.348125803663	0.0392503449108635	0.953905313456582	0.609862	0.056912810469412
LYG2	0.244648460453365	0.348142522920088	0.0904882617922132	0.881794677754899	-0.106168000000000	0.614081498359193
PSMG1	0.244642885868671	0.348142522920088	0.162646656153391	0.757524249707797	-0.215160999999999	0.258700580229750
GABRQ	0.244611745770009	0.348142522920088	0.107414944813671	0.857065724607536	0.0638663333333334	0.805736889998156
C2CD3	0.244583115587521	0.348155735953097	-0.460099588137624	0.162838224001977	0.119586500000002	0.62979571408014
CTTNBP2	0.244404149648413	0.348479140739777	0.467276855906987	0.151936428121882	-0.401874333333333	0.0647596980686847
FNBP1	0.244357334109152	0.348479140739777	-0.0149707845532740	0.982945511134086	0.219383333333334	0.439061344563948
ANKRD1	0.244321865245367	0.348498833946937	-0.119965893105964	0.835280975936648	0.257597666666667	0.524845938106282
NCKAP1	0.244288547688385	0.34853380412794	0.0809461736498331	0.896361013063453	0.208773333333335	0.278630599310579
RPL34	0.244197602145409	0.348629752009377	0.216699980395918	0.647357580911858	-0.0125886666666661	0.970481529511925
TMEM194B	0.244193765322128	0.348629752009377	0.374732040472108	0.313141424748220	0.425532333333334	0.121660357743656
NXF1	0.244088164453271	0.348958174371701	-0.171595100042862	0.739419313713722	-0.0818453333333323	0.704370299831112
PCCA	0.244084926398131	0.348958174371701	0.315720532478102	0.434100332645522	-0.160984666666666	0.413956090921311
SAMD8	0.244041228129735	0.349105155326953	0.0110282084013416	0.98774001793194	0.214341333333334	0.260859460677011
GRM3	0.24381928003270	0.349858219558482	0.449773284349812	0.177277037186644	-0.0581033333333329	0.844065049770895
ECEL1	0.243598935300236	0.350604355093302	0.21363963680241	0.653708954135733	-0.0902649999999987	0.75829995960291
RBBP6	0.243396025554798	0.35126327317345	0.149318106249449	0.782416617297835	-0.0241613333333334	0.931467600666044
RPL10A	0.243298124982215	0.35160904058329	0.284921584474367	0.498464716905649	-0.0807869999999989	0.759682045828681
OLFM2	0.243188085927648	0.351948654265997	0.0921454791493265	0.879153312143409	0.0314446666666669	0.910704531889447
TP73-AS1	0.243147310820229	0.352081724099359	-0.188424343102778	0.707361603786947	0.0476503333333352	0.880586178469642
ARL8A	0.242947183135055	0.352799684690769	0.204380903840207	0.674837470309368	0.088044	0.69414649287771
RHBDD1	0.242863217076762	0.35286994362405	0.349216005534504	0.359794558968334	0.0143556666666676	0.963383328073994
MTMR11	0.242861178733303	0.35286994362405	0.234362854514988	0.60868879500174	-0.0251829999999995	0.92865132951951
ATF5	0.242683532692043	0.353620302630615	0.318646632048442	0.428704672123663	0.0650086666666678	0.789228734235839
ARPC5	0.242651063201106	0.353620302630615	-0.0828945174942524	0.89362638370288	0.0160950000000018	0.953336983324579
CEP350	0.242393180604935	0.354398122572591	-0.118238231168512	0.838218827550598	0.0764166666666676	0.789105818888855
ADAM12	0.2423848312868	0.354398122572591	0.358931911428875	0.342742323643068	0.0195563333333336	0.949492888401158
PGBD5	0.242371965644227	0.354398122572591	0.0505252759240594	0.940625181975713	-0.165037333333332	0.458006502146897
WISP1	0.242329223680778	0.35441645884311	0.418017547989734	0.233134096593584	0.00074400000000014	0.998275219056485
TRHR	0.242248971234367	0.35441645884311	0.0643195363493246	0.919923865590619	0.170305666666667	0.443072320689246
EFNA5	0.242188452746102	0.35441645884311	0.0227855071199846	0.97536722461972	0.199334333333334	0.316427161487764
DMKN	0.242184714308736	0.35441645884311	0.263878899749257	0.544971884819175	0.0200160000000006	0.957844701580613
METTL10	0.242179691500854	0.35441645884311	0.255554123364780	0.565822042878982	0.0169596666666682	0.951767328727707
PIGN	0.242090644194951	0.354722439504709	-0.193418357943551	0.698131592467487	-0.0846343333333324	0.704370299831112
PCDHB12	0.241990928561627	0.355009105312798	0.0158549945592797	0.982102356159698	-0.026556999999999	0.915761046438965
TRIB1	0.241920616069272	0.355173260515214	0.180813508508500	0.722159001254958	-0.166816666666665	0.387687154733619
SKA2	0.241882538121695	0.355266324531256	0.169632895680958	0.743463590096836	-0.101728333333332	0.6299281511525
FAM135B	0.241720367865928	0.355872245015112	0.18615075464807	0.712609598901976	-0.0818266666666656	0.778995157976597
CCDC14	0.241664353158689	0.355874669345157	0.612425206365868	0.0277519681937494	0.232250666666667	0.25535432469149
TSPAN14	0.241609106451902	0.355990169723410	0.340000302057421	0.379921396456958	0.0597576666666667	0.816337381174918
PLA2G5	0.241528894667809	0.356258715888835	0.131970498146509	0.81030790818999	-0.0779399999999998	0.791934055525056
SORBS2	0.241484777707182	0.356386685633928	0.577341125766195	0.0433421970607609	-0.445322166666667	0.128450477696566
ZNF611	0.241449808559682	0.356427291717585	-0.104566417250565	0.86067831050617	0.0406823333333347	0.875244017858826
TMEM101	0.241287987627072	0.356898721371097	0.255484647812080	0.565900677438537	-0.294802999999998	0.120010783822305
LDOC1L	0.241137967903327	0.357321874764282	0.0289944959703013	0.96782691865619	-0.0204999999999996	0.94622978991441
CYGB	0.241078962367201	0.357524575183337	0.247259775597452	0.580200878259622	0.196636000000000	0.353448189358519
HILS1	0.240986845757569	0.357700381940505	0.284337568721794	0.50044511320899	0.0189010000000005	0.9681321732097
RPS11	0.240960381255338	0.357761955506548	0.0629796209690705	0.921760991904672	-0.0517266666666645	0.824044031896077

LINC00324	0.240909777704960	0.357914958831651	0.142479650998418	0.794346532964905	0.153723666666667	0.515767116469415
RNF187	0.240857499026862	0.357914958831651	0.0739773950388757	0.905506084101768	-0.0467383333333322	0.868395192409465
ROCK1	0.240854994972966	0.357914958831651	-0.0833729515704244	0.892833228561937	0.195287833333334	0.307774837877941
ADCY9	0.240833034420792	0.357914958831651	0.113496029277904	0.84538474875759	-0.129689000000000	0.64069646762551
NUP85	0.240829477227325	0.357914958831651	0.0357956018037514	0.958366440880488	0.0203686666666669	0.946953175174742
PCGF6	0.240800313848851	0.357990443443155	0.103578665847638	0.861943478503987	0.165008666666668	0.492100665301584
NAPG	0.240781685872815	0.35801198246553	-0.242994998086222	0.592178030679976	0.0406560000000002	0.867956695056015
ATF7	0.240750707441227	0.358096791674694	-0.145601320026762	0.787804113309913	0.0620140000000006	0.801063448666597
INTS3	0.240711627582155	0.358149265551826	-0.068674233804955	0.912149839732916	-0.191805166666666	0.447498161237612
OAS3	0.240572267166758	0.358641842282902	-0.0700188033705138	0.910821504793275	-0.0250803333333335	0.944218475609619
STX5	0.240403349675257	0.359286609496307	-0.164594985413004	0.754105947879839	0.00757400000000158	0.982375192575954
CIC	0.24036213638628	0.359354870569428	0.0382828176402345	0.954545200586067	0.165433333333334	0.428953946992621
B3GALTL	0.240321687250495	0.359483924318469	-0.177766299918111	0.728089108754591	0.271132	0.313220411537404
C4orf21	0.239987799628722	0.360607836719231	0.208972835946423	0.665595909869988	0.0783985	0.773270959529093
BAIAP2L2	0.239829795709725	0.36122604231926	0.0869922934988425	0.887055939766881	-0.0438726666666665	0.903464119339974
TNFAIP3	0.239792597062718	0.361316328934229	-0.154762628301785	0.771310656070265	0.520192	0.035274715818792
POLG	0.239711210839265	0.361624450501335	0.386692803914051	0.288212923135036	-0.241620999999998	0.230634542108013
B3GNT5	0.239704014535191	0.361624450501335	-0.394201289975486	0.274555703975637	0.00205766666666710	0.995146554639975
DDB1	0.239640386857907	0.361804066880140	0.167351154165951	0.749061933609388	-0.118883333333332	0.5531802635914
BEND5	0.239443682790786	0.362447699877011	0.395437030279303	0.272692742937895	0.0628370000000003	0.778247870247617
RASSF3	0.239389751169554	0.362651873380434	0.293550388959779	0.480680991686121	0.174093000000001	0.591181007584872
LOC646903	0.239295122888438	0.362882428524456	0.153489095136545	0.773604308443274	0.111557666666666	0.734593656619209
ATP13A3	0.239278901189615	0.362882428524456	-0.0304308857670854	0.96583351705897	0.00855466666666675	0.982375192575954
LRRC6	0.239231208743709	0.362950803739833	0.242961547944371	0.592178030679976	0.102126666666667	0.672980097690282
LONRF3	0.239201166163408	0.363031750493035	0.219177688777342	0.641655489120978	0.0313466666666675	0.911600329626215
IL10RB	0.239146776185889	0.363164267623624	0.0835878472321518	0.892833228561937	0.0504406666666689	0.835752276645858
RNF25	0.239102182831769	0.363239702888745	0.281102524909513	0.506886267079183	-0.0555206666666659	0.841976773189511
CDO1	0.239096157485515	0.363239702888745	0.268581063275810	0.535014466593144	0.062823666666668	0.851568943534661
PKP4	0.239014356703712	0.363239702888745	-0.387149848651428	0.287581226843956	0.0349033333333336	0.935347456642894
SPTLC3	0.238977789870042	0.363239702888745	-0.0560144569204392	0.93280363479932	-0.346436999999999	0.210845715069665
WDR77	0.238963910384359	0.363239702888745	0.0459785639253358	0.945364757579857	-0.165563999999999	0.499844881120878
MEP1B	0.238954936570107	0.363239702888745	-0.0742540938301805	0.905205116779936	-0.0697859999999995	0.759162700398522
KLHL28	0.238853845283987	0.363557175748413	0.135559791759242	0.80669742201648	0.143589333333334	0.532458701847411
USP46	0.238841087945349	0.363557175748413	-0.143502505691520	0.792614473650425	0.246519000000001	0.253356882479072
SRSF7	0.238758184961927	0.363880296724168	0.247637926324416	0.579577896184371	0.0359546666666681	0.888319685310725
KALRN	0.238683045564947	0.364077802741685	0.438773455230612	0.192001550682067	0.0898453333333337	0.669640159813955
C2orf27A	0.238632695123925	0.364264129792480	0.0918897664458119	0.879153312143409	0.086909333333334	0.772139923817113
FAM53B	0.238567690259160	0.364452198178843	0.302880412923044	0.462105017205825	0.105813000000000	0.629657237462109
TRIM13	0.238431100710131	0.364862665821139	0.0972010618171073	0.870020870354202	0.0601346666666674	0.80682025061975
BHLHE41	0.238305334077832	0.365217428127883	0.367639785362964	0.326948811683298	0.114765333333333	0.60217016768875
B3GALNT1	0.238278735745828	0.365281066758916	-0.0900070027352032	0.882422779091356	0.515397	0.0525641773262741
OR2B6	0.238192234546985	0.365474686835051	-0.364227666434034	0.333940239988472	-0.0597949999999994	0.813694870801638
ZBTB11	0.238184123930055	0.365474686835051	-0.0389884768200969	0.954097938114723	0.094099333333334	0.695654544311453
TEX2	0.238101354018147	0.365773853489392	0.566932866454369	0.0470952455251712	-0.157618999999999	0.471741080987819
KIAA0226L	0.238084417718259	0.365773853489392	-0.0735287984789847	0.905838142287595	-0.0700456666666667	0.789149377548591
PGM3	0.238083498658864	0.365773853489392	0.120139954803307	0.835124910268666	0.0412123333333337	0.87290879338693
PTP4A1	0.238065203632111	0.365794544564037	0.459325295165983	0.164444235253496	-0.0300593333333321	0.91680951061779
KIAA1549	0.238040566426004	0.365848137932993	0.325530647720941	0.409206882509236	-0.041413333333333	0.910190419163007
C11orf57	0.237953325131976	0.366083469604504	-0.201546440801933	0.680054545720343	0.0626583333333338	0.779668393144765
ZNF608	0.237947702471269	0.366083469604504	0.0685111817488136	0.912149839732916	-0.133143666666666	0.499957077244054
TNFRSF10B	0.237811610276814	0.366442676342728	0.211477907855501	0.659412013514194	-0.166628666666666	0.541270965348748
SLC22A3	0.237602793127361	0.367304784265763	0.301666363329125	0.463258004901552	0.494539666666667	0.0782543184284344
PITPNB	0.237473994593439	0.367826106793198	-0.119326402066757	0.836360753951164	-0.120606333333332	0.562399033626288
H1FNT	0.237206663806352	0.368846522868977	0.163667126186207	0.754968203901817	-0.173970333333332	0.606083038776023
CEP135	0.237071665389366	0.36947633094974	-0.262917233464416	0.547282180809647	0.233900333333334	0.393584757087412
FAM57A	0.23688554732099	0.370273712684461	0.23577657967288	0.606716022115166	0.114858000000001	0.597707307645867
HLX	0.236861878084225	0.370273712684461	-0.0052352887169022	0.99251028338798	-0.228769999999999	0.435157099583407
ZNF74	0.236756355836674	0.370735896319354	0.302158422765876	0.462229407853213	0.0582243333333331	0.805063265422246
C3orf35	0.236678333979322	0.370809540333996	-0.192341922174869	0.700450711372106	-0.0421929999999998	0.865655368707012
POSTN	0.236628764744377	0.370925363594616	-0.0039411775764861	0.994501615176666	0.0324803333333332	0.904788907158467
CHD1	0.236623015371534	0.370925363594616	-0.178121821578731	0.727939088352802	0.166632000000001	0.426518032099216
TTC7B	0.236527389917741	0.371128852212289	-0.188549071767722	0.707361603786947	0.251894000000001	0.25178638718411
C9	0.236526269978521	0.371128852212289	-0.0919803719887397	0.879153312143409	-0.000467333333333289	0.998309716374356
ZFP36L2	0.236489068465041	0.371128852212289	0.00797640878402772	0.990405674923721	0.0889823333333334	0.725673883230848
ASPSCR1	0.236420184765308	0.371240045081902	0.40040818737042	0.262833489019055	-0.0637449999999986	0.814273596067945
RAB8A	0.236296488821119	0.371572380017735	0.129419597794888	0.81427296395351	-0.0111213333333324	0.97201219963332
SYT8	0.236193993016251	0.371752702770693	0.213708969516276	0.653708954135733	-0.0952149999999992	0.748579513438275
MYH1	0.236160406246586	0.37184468103949	0.0702905875750942	0.910394157189308	0.109740333333334	0.745967084434416
LPPR5	0.236129279571036	0.371847819630972	0.0509368798707645	0.939677282439105	-0.0147853333333328	0.965329851466378
RNF11	0.236118863821737	0.371847819630972	0.180916744271066	0.721866914445283	0.0177176666666674	0.965329851466378
PITPNA	0.236053642027671	0.371891210945978	-0.114074017665472	0.844272085764738	-0.199151999999998	0.342825265121927
TUBGCP2	0.235968023693116	0.372116108265135	0.182344731472596	0.719419078472232	0.075211333333334	0.724384319568895
MUC4	0.235884596488109	0.372256657317942	-0.156648458086568	0.768843563796587	-0.0973039999999998	0.65857887074962
HYOU1	0.235870092527204	0.372256657317942	-0.255376789017014	0.566022340784541	-0.214229666666666	0.264927993735684
ZNF628	0.235822818310441	0.372373912065631	0.346460613857910	0.365394536440656	-0.0357403333333330	0.91402300740264
DKFZP434I0714	0.235790863994762	0.372373912065631	-0.0347868025777637	0.959404955370931	-0.000510999999999864	0.998281147912498
KANSL3	0.235729184962464	0.372485675800260	-0.0740430539989602	0.905506084101768	0.0329826666666664	0.90945379489823
SH3BP1	0.235726916851322	0.372485675800260	0.142481424807896	0.794346532964905	-0.112427333333333	0.705010402761235
HIST1H1D	0.235707084211772	0.372515180298699	-0.0518824066701268	0.938067483409541	0.00784333333333383	0.987134800094338
FMOD	0.235658119423641	0.372697373990385	0.292687218725144	0.483287060229305	0.0122426666666672	0.974895360062947

TBX18	0.235634090300878	0.372748898738101	0.141904303025736	0.794736459940131	0.0660253333333341	0.85013576481729
SOCS3	0.235573842578195	0.372808107799978	-0.082043780011583	0.895064720363003	-0.0169459999999999	0.951509314215492
WIPF2	0.235566015759209	0.372808107799978	-0.284265289686723	0.500550458443644	0.281777666666667	0.183134786321360
FAM198B	0.235541376513346	0.372862881690395	0.274658923263008	0.520282450211655	0.528777000000001	0.035274715818792
SUGP1	0.235448015770758	0.372966438620156	0.27394551193526	0.522049608558573	-0.147641999999999	0.458006502146897
LUC7L3	0.235416632724241	0.372966438620156	0.326776438837471	0.409206882509236	-0.0357794999999996	0.895510661232872
GRWD1	0.235386643546514	0.372966438620156	0.025921235373225	0.971054398685485	-0.544324333333332	0.0205866975799116
PLEKHA3	0.235378921097336	0.372966438620156	-0.0289110725506851	0.96782691865619	0.175652666666668	0.443378989641463
BPIFA1	0.235340107003623	0.373026136740713	0.0614171437249889	0.924016783210879	0.00674433333333365	0.987134800094338
HOXB-AS3	0.235289242917563	0.373144262080447	-0.0378812213421033	0.954958350556272	-0.118464333333333	0.669530887411007
SLC6A8	0.235246653715918	0.373220408386412	0.110723019909187	0.849357727620213	-0.183359333333332	0.439061344563948
SNORA68	0.235246396657325	0.373220408386412	0.121986404020834	0.830347805834363	-0.202481333333332	0.384519727812872
CCRL1	0.235211697290737	0.373253891391860	-0.204334615628348	0.674837470309368	-0.0594208333333326	0.816337381174918
MDK	0.235163241994172	0.373359523779459	-0.188192455928675	0.707635058271509	-0.126637666666665	0.614221661998915
HMGXB4	0.235143592033153	0.373388359066728	-0.0908579983962955	0.881323965029954	0.0181776666666675	0.954608215091566
ZNF536	0.23509179576544	0.373564616906327	0.153743457191787	0.773238461355017	0.144404333333334	0.547637880318521
ZNF594	0.235079839489687	0.373564616906327	-0.172530575164919	0.738421727152922	0.317114333333335	0.220511040537878
CCDC43	0.23503631048541	0.373580041938661	0.0743189702224277	0.905140158515919	-0.0138676666666657	0.964514058161652
PI4K2A	0.23499271333122	0.373674453953311	-0.183098199370838	0.718067440349544	-0.185543333333333	0.329908083538015
NUP35	0.234975320174949	0.373674453953311	0.178095112439638	0.727939088352802	0.0433473333333334	0.880830734199068
CEP95	0.234970627691556	0.373674453953311	0.262562570321083	0.548594673068337	0.164656000000000	0.449436921437102
KLK6	0.234921440004436	0.373674453953311	0.0809183666614465	0.896361013063453	-0.177468	0.367206442400058
MFAP4	0.234895466786480	0.373674453953311	-0.200750125314080	0.680524286175603	-0.155059666666666	0.462444913819465
RSPH9	0.234877934537154	0.373674453953311	0.0205904510268567	0.976404959469803	0.158414666666668	0.459702144856649
CMTM6	0.234877734428177	0.373674453953311	0.0492951759648260	0.941842562680095	0.0893653333333343	0.678230502146332
MGEA5	0.234877007634464	0.373674453953311	0.150764830357228	0.778702262208655	0.0181720000000022	0.953246145975406
ADRA2A	0.234820388937071	0.37389727324029	0.242221257266024	0.594404778895365	0.00690166666666804	0.983159241812861
ZNF780B	0.234795777189268	0.373952302341872	-0.283164096211531	0.502129505146473	0.0918833333333344	0.791014715933453
MAS1	0.234749862335991	0.374045043657601	0.0159981297925650	0.982102356159698	0.137087333333334	0.525143015861964
DRD4	0.234311113024591	0.375587076251692	0.486748839919105	0.130950940658109	-0.112075666666666	0.681255554040718
HUNK	0.234288799180901	0.375587076251692	0.252772854052300	0.569078031057771	0.056340000000001	0.861945621270498
LENG1	0.234288672491503	0.375587076251692	0.259460036213020	0.557000836743775	-0.183869666666666	0.471741080987819
FAM89B	0.234246826271454	0.375681843708681	-0.0203477544779884	0.976488095728823	-0.330834666666665	0.209585689318078
LMNA	0.234226607466428	0.375681843708681	0.0518295290501907	0.938168091114785	0.0838720000000014	0.704370299831112
ZMYND11	0.234205865709981	0.375716791430094	0.0416755769847198	0.951989245458556	0.065757000000001	0.778247870247617
CNN2	0.234125554047127	0.37599095290938	-0.298896533279433	0.469994970380678	0.237025333333334	0.258960340011801
MEX3C	0.233908830945127	0.376446676267346	-0.179481525363331	0.724770967840177	0.317096666666669	0.112438115304648
SUPT4H1	0.233904493925905	0.376446676267346	0.321627739344812	0.420267449900952	0.0168536666666668	0.953217548324318
C1orf74	0.233883131876286	0.376446676267346	0.143320984331656	0.79287526984225	0.359601666666667	0.077796064540853
RALY	0.233881994921539	0.376446676267346	-0.137039791556609	0.803104295581883	-0.0202076666666658	0.945449455238123
PGAM1	0.233869956572206	0.376446676267346	0.153631484327167	0.773358453580566	0.0278780000000009	0.914418029309221
LEFTY2	0.233850168993359	0.376476762252089	0.174746909141972	0.733916687337453	-0.126589666666667	0.618200125888358
PHC3	0.233817115707259	0.376504422955736	0.165293907880663	0.752544465367249	0.101236333333334	0.635069698575895
LYZL4	0.2338167769236	0.376504422955736	0.148655290683423	0.78320766974403	-0.484198999999999	0.223260757343954
HIST1H2BK	0.233764023295087	0.376708065729755	-0.173222537872682	0.736740209571778	0.202344166666668	0.359908305595919
HRH1	0.233653601424227	0.377215595780716	-0.169785458817340	0.743328907676018	-0.0120016666666668	0.969156872792422
MED13L	0.233604358910149	0.377262771452597	0.255722280691390	0.565656702091657	-0.130581999999999	0.589826330962094
ODF3	0.23359284479368	0.377262771452597	0.177530240913567	0.728529406441478	-0.276638333333333	0.324937679238988
ADHFE1	0.233578313607834	0.377262771452597	0.310216033591686	0.446321819288896	0.206143	0.341218327677039
TBCEL	0.233486319947057	0.377491595345758	0.0691543879406086	0.911837389834658	-0.00940366666666497	0.982375192575954
SKP2	0.233469678191666	0.377491595345758	0.173024896957381	0.737325433935202	0.162399666666669	0.440618402525625
PDGFA	0.233461786444679	0.377491595345758	-0.327652998199332	0.408290478248454	0.398259333333334	0.0618388608777955
ZYG11A	0.233460640302677	0.377491595345758	-0.322890042085213	0.415927283236740	-0.0341466666666665	0.907096813604642
NUP160	0.233344181523198	0.37795747478193	0.16662417444879	0.750457342031334	-0.0757786666666657	0.76564992393702
ADCY8	0.233208637203001	0.378376190403124	0.0424311222489460	0.951446425515852	-0.176267333333334	0.459936388684371
MAU2	0.233158256830795	0.37844741424104	0.291849330959132	0.485255752311035	-0.0825926666666656	0.748388402768556
LRRN4	0.233154192499313	0.37844741424104	0.0654821982751548	0.918111672450639	-0.0661743333333333	0.82667899725046
QSOX1	0.232980937957382	0.379208010705086	0.161170417988397	0.760287664335002	-0.132791666666665	0.5488879250835
NID2	0.232911003363315	0.379503527204908	0.245057410850964	0.58629105649071	0.534441333333334	0.0431903372471131
CHRM5	0.232891344197685	0.379533236626602	0.0167979955470520	0.981160061237418	0.0446630000000002	0.867268897859745
CALHM1	0.232775531171235	0.379611757132558	0.0530313207531537	0.936712858731269	-0.103745000000000	0.626653302222468
KDELC1	0.232762490417240	0.379611757132558	-0.025404730302365	0.972264538231849	0.193567666666667	0.381152785222697
ANGEL2	0.232755561608549	0.379611757132558	0.188022072373291	0.70790719815331	-0.643752333333333	0.0093299183713845
TPR	0.232729483368753	0.379611757132558	-0.157504675624215	0.767770230680061	0.186359	0.341218327677039
PRRT3	0.232721238895166	0.379611757132558	-0.00178940180290534	0.99760061742039	-0.134401333333333	0.520245449442202
KREMEN1	0.232706435778469	0.379611757132558	0.456394695378617	0.16754724558121	0.169045666666667	0.397798624983652
NUDT3	0.232706421726981	0.379611757132558	0.266619538276483	0.538667546206922	-0.0476679999999995	0.853673730448935
OR8D2	0.232701505430486	0.379611757132558	0.156805276608783	0.768426887946077	-0.0150416666666668	0.962371956441728
XPNPEP3	0.232680122634195	0.379611757132558	-0.0153405267466509	0.982725338620588	0.0351783333333344	0.886930360251063
IFT46	0.232645475212718	0.379646786038914	0.00941552815594057	0.98909166696601	0.0398843333333342	0.899912734474539
PARM1	0.232613043139938	0.379698624027678	0.151487611299815	0.777104180604402	-0.256621999999999	0.302732927341247
MED17	0.232435529021922	0.38028345581132	-0.249982687992193	0.574968685145169	0.123859666666667	0.580149967309238
GYS2	0.232383583257239	0.38028345581132	0.142077318655405	0.79450771779567	-0.0209866666666667	0.955460918122607
NACC1	0.232144157612399	0.38104033097597	0.304009449203014	0.460052080110635	-0.245384666666666	0.210845715069665
DHX57	0.232023672506256	0.381456418368037	0.317467022343626	0.431166148274203	-0.193147000000000	0.374814376822818
EIF5B	0.231996366980653	0.381524383203159	-0.0833742751082806	0.892833228561937	-0.0739976666666649	0.741438256509195
LAMB4	0.231873198553164	0.381781539217797	0.114348211079999	0.844272085764738	-0.183185000000000	0.426484252681993
DACT1	0.231814779858764	0.381861323684531	0.291417857375837	0.48675097519627	-0.764167333333333	0.0105585104334125
BTBD7	0.231727877396078	0.381987601182243	0.00250741927952545	0.99580901762095	-0.0117903333333319	0.974895360062947
NSMCE4A	0.231605735498835	0.382277154100897	0.151569213580543	0.777101208632856	-0.0300993333333335	0.910920430866321

ADCY10P1	0.231603501606102	0.382277154100897	-0.239224473654081	0.599801655225887	-0.0626303333333323	0.828424497817305
PRPF40A	0.231539362462426	0.382416506067663	0.0971655291707502	0.870020870354202	-0.0240433333333326	0.926730454971853
SPC25	0.231521438823618	0.382416506067663	0.164810080736061	0.753523107566577	0.258531333333334	0.245623058897326
ANXA11	0.231473403024479	0.382504187972498	0.170637696613895	0.741623909905464	0.117137666666667	0.576277303044571
INO80D	0.231391935605533	0.382585921397521	0.367570678174652	0.326948811683298	0.0716616666666676	0.834476346642783
TRPC6	0.231110644170694	0.383635641192995	-0.206929316112269	0.669907948309556	-0.0694246666666665	0.760810252329096
TMEM134	0.231081589679969	0.383716030483807	0.218466447216808	0.642691336921378	0.123900333333334	0.616036866881905
PAK2	0.231025567056436	0.383915991003148	0.118693600262456	0.83731413798382	-0.0755053333333315	0.758885780300811
PNRC1	0.231016119372161	0.383915991003148	0.0769114641116113	0.901842329205355	0.0769786666666672	0.741809049318412
BACE1	0.230992756204814	0.383966173483672	0.0780702606336444	0.899705053748212	0.266678	0.178162638616424
CD248	0.230947418459880	0.384059188433047	0.141240765662805	0.796285336930078	-0.160048000000000	0.575589336285762
FBXO38	0.230804946941437	0.384594935748193	0.066565661874637	0.915858484030651	0.0163936666666681	0.957844701580613
PRRX2	0.23078739057692	0.384614300321166	0.56631891699607	0.0474373345172437	-0.150718333333333	0.5683587002004
TSPAN6	0.230732347161032	0.384833214920536	-0.0269441422810045	0.970207612777188	0.0764790000000006	0.719500645075484
EPHB2	0.230683072391730	0.384885653409818	-0.248676872828443	0.577025280706283	-0.187168333333333	0.376882447735926
EIF4ENIF1	0.230641841285387	0.384885653409818	-0.0418223340886416	0.951989245458556	0.154923000000001	0.442294185443201
ASCC1	0.230625127768668	0.384885653409818	0.0565414675991122	0.93176886339657	0.119502333333334	0.576277303044571
KCNB1	0.230561130232497	0.385152358130436	-0.0775823137360312	0.900701176765499	-0.230907999999999	0.258700580229750
NFASC	0.230536887230635	0.385207426218627	0.103697961451291	0.861872724387044	-0.105199999999999	0.68417809000625
PDGFRB	0.230455659700099	0.385543540117475	0.241688338713432	0.595201219333254	-0.0331049999999994	0.904018282943947
GRK6	0.230438734954882	0.385543540117475	-0.0922205256349527	0.879153312143409	0.0531403333333334	0.815063497380215
DMWD	0.230352859670739	0.385744829004273	0.114568670879647	0.844272085764738	0.0977610000000006	0.63969794643309
TUB	0.230332772234101	0.385744829004273	0.206288378398813	0.671467763213228	-0.142302333333333	0.575589336285762
TXLNA	0.23031095584753	0.385744829004273	0.0649534897742121	0.918489100363712	-0.187112333333333	0.352237134604623
STMN1	0.230295208271764	0.385754742386751	-0.0380883270968761	0.95468180561467	0.00969566666666735	0.974451420920923
DOCK3	0.230239452996255	0.385884205352783	-0.0694164928293628	0.91172160664395	-0.284734333333333	0.152067315618299
KIAA1524	0.230206489979117	0.385884205352783	0.0514608178825349	0.938728344711776	0.0753673333333352	0.76564992393702
ARHGAP18	0.230206250542193	0.385884205352783	0.421720420988329	0.222348587392621	-0.0953903333333328	0.727809376593958
HECW2	0.230201504688084	0.385884205352783	-0.252125896626209	0.570473184967855	-0.0171783333333325	0.956112529438896
STT3A	0.230182586019365	0.385911075159166	-0.147186080976744	0.785691396687707	-0.00401999999999894	0.989265037176288
SYDE1	0.230159601831027	0.385959620462592	0.31685012597415	0.431921760942787	0.178855666666667	0.374814376822818
MXRA8	0.230017271751892	0.386570591244116	0.0713241961757672	0.909119908757507	-0.144530666666666	0.713714407278061
ZRANB3	0.229967687774455	0.386687099605629	0.455414694086865	0.168217575057610	0.0853366666666673	0.73097308185803
GLB1L	0.229937440774587	0.386774481834319	-0.114017257124858	0.844272085764738	0.0805440000000003	0.714244999058301
ESYT1	0.229727594559964	0.387450645607565	0.334441536219656	0.394347836736941	0.186189333333334	0.386271329500871
FLT3LG	0.229626430694617	0.387822589005760	-0.0373398636461429	0.955937551268326	0.00676233333333357	0.98349947509937
ARHGAP20	0.229568221256471	0.388006047069007	0.260665291649923	0.553540075799904	0.313957666666668	0.357227810557244
TRAPPC12	0.229342606476525	0.388489480836173	0.280374146203109	0.507669977518963	-0.0398149999999999	0.880131955403697
DRD5	0.229340691565796	0.388489480836173	-0.0373116535558647	0.955983075178843	0.0155465555555553	0.955460918122607
TRAF6	0.229339742028726	0.388489480836173	0.262880810373422	0.547282180809647	0.0717223333333338	0.797534995180308
HGSNAT	0.229325445423516	0.388489480836173	0.0328629464107106	0.961351562552925	0.495874000000001	0.0392329313827969
MESDC2	0.229304853916976	0.388525579618751	-0.0592620220899535	0.927328520016798	0.218571333333334	0.248864745773935
POLR2B	0.229234362617062	0.388529249342852	-0.172854099251125	0.737853882640005	-0.129263333333332	0.534196070908831
SMARCD1	0.229203425143193	0.388529249342852	0.532580652806342	0.0768868109577917	0.095793	0.643792824295256
RABL6	0.229199925092712	0.388529249342852	0.223152061121801	0.635247978870818	-0.141110333333331	0.536063442230912
HERC2P4	0.229167112178454	0.388529249342852	-0.328011983525293	0.407465492446943	0.0386153333333337	0.895093529187836
GEM	0.229151036772700	0.388529249342852	-0.155203231082775	0.77066107821265	0.207556000000001	0.274121181062751
ACSS1	0.229146845666495	0.388529249342852	0.357200370145049	0.345098975152893	-0.182656666666666	0.513530370066639
CBY1	0.229101322689531	0.388529249342852	0.200858987924431	0.680363224840146	0.152877000000000	0.518836392563847
PRKDC	0.229057607325155	0.388664707963392	0.091821863678268	0.879153312143409	-0.118129666666666	0.62979571408014
NAGK	0.228945014430343	0.389119202968171	-0.072187492351833	0.908125883214154	-0.0805550000000002	0.70610473471702
PNN	0.228910486535316	0.389156168388652	0.0837722662160022	0.892833228561937	-0.110621666666666	0.608679471465191
C1orf112	0.228893318092796	0.389174154383032	-0.0449109673904087	0.947178127421114	0.130259666666667	0.743139552172972
PODN	0.228852320966863	0.389245793438402	-0.00885789443316934	0.98946753270812	-0.0503879999999994	0.854461589542622
MAP1S	0.228754124071536	0.389623658535913	-0.0464774665505127	0.944839700668385	0.0909483333333347	0.707538975864338
HOXD10	0.228737743332445	0.389636662187087	0.022930731336333	0.97536722461972	0.0537066666666675	0.895043648815056
PPP1R14A	0.228724094888276	0.389636662187087	0.374413874910885	0.314207741629911	-0.0183099999999999	0.948469012194168
IWS1	0.228674715853304	0.389647712939183	0.0702624333301288	0.910394157189308	0.175827666666667	0.445344763359555
C10orf114	0.228639393564218	0.389720893015232	0.0886041609882282	0.884539076391469	-0.0762656666666663	0.787230398823895
SERPIND1	0.228578782311085	0.389826376425784	0.174311414720531	0.734267769889068	-0.280162333333333	0.277812190641212
CCDC71	0.228578331512096	0.389826376425784	0.0678039125389167	0.91349245220019	0.0399803333333331	0.873713119973631
LRRFIP1	0.228560586590274	0.389847587331413	-0.139223275926280	0.79864602540281	-0.131526266666666	0.568393891086758
KLHDC8B	0.228409242147205	0.390418313244989	0.5229359302794	0.0873299331180663	-0.548259	0.133614476543628
ABCG1	0.228376218182577	0.390418313244989	0.171699112604495	0.739343956096839	-0.151927333333333	0.527465067495661
TUBB2A	0.228361054102746	0.390418313244989	0.122315406925376	0.829569971668386	-0.00612644444444263	0.986471172041113
FAM126B	0.228357586428551	0.390418313244989	0.242426764027619	0.593510912860895	-0.128752333333332	0.534196070908831
ATL1	0.228329570086873	0.390459037337855	0.242520838675564	0.593306823292819	0.512955666666668	0.0282821352636199
SPSB3	0.228294906434410	0.390459037337855	0.359753983502090	0.341510139061518	-0.0806063333333329	0.738876306321397
LINC00094	0.228273806583633	0.390498334515381	0.0126431639453150	0.985214990982244	-0.130823999999999	0.5293518047673
CCDC40	0.228218024994097	0.390589301752613	0.180503659666846	0.722641197261641	-0.204413333333333	0.372351777769947
CEACAM19	0.228147871666455	0.390804487989279	0.248405863674872	0.577610313927744	-0.0645739999999996	0.772520347452753
FAM83F	0.228098293463991	0.390843395459405	-0.0518064863700128	0.938168091114785	-0.119717000000000	0.661127823811077
NUDT9P1	0.227912162983279	0.391404734978229	0.505508718008236	0.105941223458825	-0.0228666666666666	0.946627610228776
PKMYT1	0.227886886789490	0.391466592474387	0.390159495155426	0.282383744168943	-0.0475776666666659	0.867354667762818
KIAA1109	0.227860151425635	0.391536289974224	-0.171842493719455	0.739167054766667	0.228306000000000	0.324937679238988
SAGE1	0.227719253747545	0.391717843322044	-0.00014532953713752	0.999860038008296	0.143135666666667	0.488602547609567
ZNF506	0.227682630139407	0.391794905590096	-0.167110602913003	0.749664019651347	0.159557333333335	0.549659494702918
C5orf42	0.227604357671053	0.392024584912303	0.30549949285328	0.458013550008763	0.311814000000001	0.147939960258863
CD81	0.227447136599006	0.392596495699220	-0.122640224256739	0.828930912293926	-0.109385333333332	0.615722818909839
ZNF687	0.227431881993062	0.392596495699220	0.371325516266012	0.31973535243631	0.115578333333335	0.563120908487291

ALS2CR12	0.227410936788580	0.392621100737382	-0.208337070534701	0.666836876948506	0.265731000000001	0.159240262526426
ALDH1L2	0.22738918112698	0.392664287438336	0.125148804009129	0.824230922676958	-0.0748529999999995	0.730499650352488
PSME2	0.227214327610602	0.393247190998558	0.256423208838566	0.564430426783556	-0.0213873333333330	0.943336059867727
SHISA6	0.227091351349949	0.393638887768747	0.404946130934083	0.25480455106032	-0.136132166666666	0.536063442230912
NUPL1	0.227070205409796	0.393638887768747	-0.222969238584990	0.635247978870818	0.132622666666667	0.59717035375984
ZNF586	0.227062088838736	0.393638887768747	0.0586123846145296	0.928586335857223	-0.133449666666665	0.571851976728397
LINC00470	0.226959454296212	0.393721944331195	-0.0198555139129882	0.977526645675126	0.168833333333334	0.376224594108747
F2R	0.226922793433815	0.393845495732364	0.0650366337915675	0.918489100363712	1.03671666666667	0.00311122808725912
RPL10L	0.226904357003116	0.393871031550509	-0.0986141173371024	0.868080433714765	0.0556110000000008	0.853541852582193
LEPRE1	0.226660960598485	0.39503426795052	0.12020754880784	0.835118069372686	0.142982333333334	0.491578070092
EDIL3	0.226594145741524	0.395320696172523	0.313078186930673	0.438459883259885	0.266306666666667	0.227881106249801
CNR1	0.226485445294145	0.395719835433369	0.150156900500427	0.779734405920598	-0.121557333333332	0.828418045423507
ZFP36	0.226475190934091	0.395719835433369	-0.229471824710505	0.620183562399927	0.159545	0.436636858796753
NAP1L5	0.226363803224469	0.39604650865713	0.0174273238398257	0.980603313490195	-0.294761666666667	0.311888164974831
KCNAB3	0.226268367664458	0.396414088933018	0.191099233302861	0.702165449732036	-0.193771333333333	0.344576368488222
AK8	0.226173029543716	0.396781372708525	0.150163332537336	0.779734405920598	-0.178374666666666	0.461494497197158
SBF1	0.226086411349093	0.397027719159566	0.101020417595439	0.866005093242444	-0.00291166666666644	0.993724132855888
BZRAP1	0.226005198123813	0.397318972175077	0.554744594955592	0.0573563601870839	-0.145108999999999	0.503217861354116
WDR18	0.225920298155155	0.397630328172276	0.0472876820459809	0.943981149721771	0.00552033333333491	0.987134800094338
NREP	0.225888571476677	0.397675695183782	0.23229687227246	0.612658112747496	-0.134509333333331	0.496883548839873
AGFG1	0.225814134148969	0.397908874045319	-0.0197200982356831	0.97763669370967	0.117700000000001	0.609972059115841
CDKN2A	0.225738396794019	0.398245006275925	0.183592624843900	0.718067440349544	-0.158642333333332	0.442294185443201
USP10	0.225667802587836	0.398535919362169	-0.158606323533985	0.765791046741822	0.0628733333333335	0.791595191224138
LRRC16A	0.225640556000553	0.398535919362169	0.126077052720856	0.822164329801713	0.0701583333333342	0.853099188701412
WDR76	0.225630015985786	0.398535919362169	0.114195669422478	0.844272085764738	0.215163000000000	0.284033466227373
OR7E91P	0.225547329958762	0.398710590262227	-0.121076127267692	0.83265693609154	-0.0692449999999993	0.75583456990156
AKAP8	0.225512836674585	0.398736518016279	0.406126248614214	0.254041953733213	-0.050152666666667	0.82616467676523
RHBG	0.225454679620869	0.398736518016279	0.484819465422489	0.133687121162475	-0.151029333333333	0.626750668682093
SRSF4	0.225432079178630	0.398736518016279	-0.0353166980414821	0.95869292666698	-0.0590806666666656	0.798170083215805
GUCY1A3	0.225429076885164	0.398736518016279	-0.0485800351910234	0.94236319435931	-0.00289566666666643	0.993677488355417
PI4KA	0.225283406543554	0.399378284814651	0.0634408486526395	0.920855211745173	0.0880660000000003	0.709400163383053
ZBTB37	0.225197688138169	0.399481117996288	0.340182077877124	0.379495306350778	0.198002333333334	0.309421300309741
DEK	0.225196215000282	0.399481117996288	-0.130356604800687	0.812271254601992	0.0951166666666682	0.648963524582188
IL31RA	0.225178643798726	0.399488376192033	0.0681967622469058	0.912364243071055	0.0964003333333334	0.64675322310909
AKT2	0.225140321239158	0.399488376192033	0.220898237911026	0.638522059865506	0.0178550000000011	0.95417877753931
KDM3A	0.225027518408503	0.399898699844754	0.0875148588407228	0.886302037299497	0.0618696666666682	0.793596726500656
EFNB1	0.225023788225531	0.399898699844754	0.563065984198485	0.0506240759292818	-0.0306406666666661	0.918363743182112
CTDSP2	0.224954832847686	0.400199014067726	0.220909073433179	0.638522059865506	0.144576666666668	0.488854255999561
AQP9	0.224932450393061	0.400246524320387	-0.174541060562539	0.734267769889068	-0.118043333333334	0.583728961001425
ENG	0.22474938054513	0.400796786994708	-0.0898408802572585	0.88308427646146	-0.077893666666666	0.732554472781933
KIAA0907	0.224732776330722	0.400812979001603	0.0820612546758204	0.895064720363003	0.0326386666666679	0.903274690789156
C16orf78	0.224533385093549	0.401600882235195	0.113324354747469	0.845747219093509	0.00719933333333385	0.983159241812861
ZNF512	0.224493745427340	0.401606737710959	-0.0921313111625062	0.879153312143409	-0.0636109999999999	0.781760032958938
NR5A1	0.224491448913133	0.401606737710959	0.36054483191617	0.34034906917442	0.193217333333334	0.339281891357904
MARCKSL1	0.224412695650771	0.401795734624349	0.382837666268578	0.294588782643652	-0.0827889999999998	0.732554472781933
EFNA4	0.224402255736939	0.401795734624349	0.625582224259281	0.0231547287769424	-0.379284333333333	0.218822168421039
ABCC9	0.224330780373490	0.401962515788963	-0.110678772648417	0.849359866036793	-0.353714333333333	0.0895671235838677
NAB1	0.224231400717511	0.402281306471014	-0.443681489267392	0.18420454346518	-0.0957356666666663	0.702709435280393
IGDCC4	0.224087906615952	0.402766528471267	0.107919101871877	0.856045571852207	-0.309736999999999	0.165412506462955
SRCAP	0.224058738773036	0.402851357504293	0.174846465752033	0.733598828800337	-0.049001	0.880429228421837
ZNF286A	0.224043010870667	0.402862960573447	0.313871996040907	0.438046311563112	0.155700166666667	0.535492155031224
GATA4	0.223973571967072	0.403167327068926	0.499533364833136	0.113777333040600	0.0459893333333334	0.848273418377467
UBE2N	0.223616751353284	0.404227329222235	-0.101918182885549	0.864616357134757	-0.121962333333332	0.575378149225683
NPTX1	0.223615943028102	0.404227329222235	0.347183585559349	0.363894788429779	-0.262846666666666	0.397798624983652
NF2	0.223520669876690	0.404508192854289	0.188158721051397	0.707635058271509	-0.0218283333333323	0.955115242974667
RUVBL1	0.223510423097869	0.404508192854289	-0.0339974025360868	0.95993706312687	-0.0619146666666664	0.840175825309652
ARL15	0.223411726933522	0.404654407024462	-0.090498077851275	0.881794677754899	-0.126268333333333	0.549659494702918
UPF2	0.223372867851327	0.404654407024462	-0.0101270900322481	0.988384322540392	-0.117403666666666	0.589306764532592
BPIFB2	0.223361501658240	0.404654407024462	0.0267924929333885	0.970207612777188	-0.0440166666666667	0.888217263833458
API5	0.223331688160999	0.404718416463957	-0.320923859872696	0.421618886526906	0.152577333333334	0.434092150903519
ZNF584	0.223314064416869	0.404718416463957	0.360075726931181	0.34143422019372	-0.0259866666666661	0.924701921028515
PNLIPRP2	0.223303124052761	0.404718416463957	0.209981544983695	0.663167662486675	0.0139426666666669	0.97184501043952
FRMD6	0.223295536446566	0.404718416463957	0.200795411618676	0.680489843835436	0.236691000000000	0.259184854253064
TMEM136	0.223011920545922	0.406046491719101	0.269193213542706	0.533126014175154	0.126652333333334	0.620354231432682
ENPP6	0.222912733677377	0.40636675409717	0.0560597379025928	0.932663091036367	-0.220871666666666	0.373444155303416
NSUN3	0.222859227727269	0.406545472295097	-0.222160072499339	0.636477566065103	0.094152333333334	0.656222957956058
NCOA1	0.222580623668119	0.407443837984004	0.0488333212291966	0.942180113539637	0.281262000000000	0.187209474302320
SPIRE1	0.222545940357312	0.407559740420304	-0.000681851801027280	0.999016032077037	-0.102802000000000	0.623357154900356
TMEM201	0.222527270105538	0.407586532029621	-0.114594799640523	0.844272085764738	-0.0585683333333329	0.828547778731026
DLX3	0.222500414353721	0.407586532029621	0.231534372706740	0.614724037644417	-0.233734333333333	0.28322823752779
LYPD6B	0.222409314240724	0.407937694179576	0.259455929105396	0.557000836743775	-0.0699459999999993	0.748989588811491
GAP43	0.222241475095032	0.408315434946706	0.448135739749263	0.178249202877872	-0.156020666666666	0.459236736587605
DHX15	0.222195540651767	0.408315434946706	-0.126926116101002	0.820608873323365	-0.0455323333333336	0.853219850381138
COL25A1	0.222191596898172	0.408315434946706	0.377402120563443	0.307201191432389	-0.159850000000000	0.512796060754107
METTL15	0.222160214583834	0.408413444268327	0.216144772516221	0.64844945813838	0.346288333333334	0.0885610157478744
TARDBP	0.222085083110762	0.408720859262330	-0.185683681098146	0.714043644840406	-0.0133999999999989	0.962966537315174
ESPL1	0.222069209173921	0.408720859262330	0.08599621990112	0.888306347447303	0.118276000000001	0.637426515050706
FAM35A	0.222029726319124	0.408720859262330	0.115941122157209	0.842733236705133	-0.0209545555555552	0.943839037582684
NOSIP	0.222022960402677	0.408720859262330	0.0416315300876481	0.951989245458556	0.0877013333333345	0.675221998008387
HSPD1	0.221959519130597	0.408720859262330	0.282734512330877	0.502589413566017	-0.119150000000000	0.575526309068521

IFNW1	0.221945412890256	0.408720859262330	0.215536143274167	0.649546282524329	0.0916313333333334	0.719839702715467
HMBS	0.221938856613768	0.408720859262330	0.0323589194035490	0.962269716253437	0.0466670000000008	0.846917872248932
HIST1H2BD	0.221922314909239	0.408720859262330	-0.280574172891094	0.507460240190768	0.228513000000001	0.451528670919448
RAD21	0.221915013063409	0.408720859262330	0.332391644976040	0.398142341492687	0.130510000000001	0.523453039510001
NOX3	0.221891068003358	0.408778181450326	0.0571828779839574	0.930775493791569	0.0315800000000007	0.919420614501031
CEBPZ	0.221795572496006	0.409046409965906	0.251018078953384	0.572882168047464	0.0852646666666673	0.697187294779476
CBX6	0.221774720198088	0.409046409965906	-0.0823417627932548	0.894273983655509	-0.271950333333333	0.171804245444023
ARHGAP42	0.221759044717248	0.409058391137291	-0.0620063955299035	0.923034515346352	0.345411000000002	0.0917903726055603
PGS1	0.221710431373655	0.409122458004943	0.0683953058302676	0.912232638501567	0.0665973333333341	0.789917107854685
SLFN12	0.221706905744296	0.409122458004943	-0.0515083654538962	0.938728344711776	-0.0671973333333325	0.85013576481729
CDC73	0.221683593891026	0.409176401269062	0.183440269042644	0.718067440349544	0.0818206666666674	0.707685424800127
FAM188A	0.221642596744147	0.409327470059802	0.240262943902541	0.597862631330416	0.0378176666666671	0.905322709877338
NECAB1	0.221619502388297	0.409380244708409	-0.150124434116351	0.7798433776922	-0.128918000000000	0.578402578384935
YWHAH	0.221588826637554	0.409400624711454	0.0639119956380816	0.920312799135406	0.130644333333335	0.520709741182581
KLK7	0.221566797391157	0.409447576236157	-0.0797865134807752	0.897892885174022	0.0324606666666675	0.924900975298637
ATG13	0.221484831620001	0.409591831550925	-0.039958781893112	0.953417942824155	-0.115512999999998	0.568393891086758
SNX22	0.221459763671113	0.409591831550925	0.262991981916206	0.546984029997242	-0.0704516666666657	0.789377975672973
LMF2	0.221427913090383	0.409591831550925	0.0743781796747179	0.904945422294164	0.165895333333333	0.435157099583407
MCC	0.221410397968201	0.409591831550925	0.28087707993318	0.507306683802255	-0.0489693333333318	0.849305028345367
PCDHB18	0.221387086990116	0.409591831550925	0.036170445561845	0.95797846477565	0.103163000000001	0.657353585270084
CGGBP1	0.221378899754665	0.409591831550925	-0.238637369216640	0.600861960738847	-0.0197246666666664	0.943391353769848
LINC00208	0.221319186783203	0.409845838934924	0.0451614790502148	0.946577404106596	-0.16736	0.471741080987819
GMEB1	0.221254349620084	0.41012810877367	-0.313314136619242	0.438100620341757	-0.0307189999999986	0.90945379489823
DDX43	0.221212135448316	0.410286144162918	-0.099750506302526	0.867558388686851	0.180002666666667	0.409335260696998
CDC37	0.221137555567167	0.410400235925056	0.020568783010681	0.976412247012224	-0.151628333333332	0.446926946255299
ZC3H12B	0.220924504648139	0.411042197135469	-0.447658841073935	0.178249202877872	-0.0932526666666671	0.692043732062832
NAA15	0.220777236535452	0.411716737196677	-0.161418970874961	0.759262381997816	-0.184625333333333	0.37261882477296
PXDN	0.220676880148679	0.412094937536418	-0.0851499067271017	0.890458025676316	-0.0775679999999996	0.73097308185803
RBM25	0.220671262929649	0.412094937536418	0.0108876417344561	0.98774587825743	0.0583826666666677	0.793596726500656
TRIM42	0.220569478162727	0.412333837085274	0.208858765204236	0.665595909869988	-0.100906666666666	0.702709435280393
TMEM63A	0.220521783323043	0.412333837085274	0.342725580686528	0.374882411235977	0.0922426666666668	0.764848773122078
FAM193A	0.220520412580126	0.412333837085274	-0.01501770898249	0.982921609770846	0.135895666666667	0.536810332635776
NAT14	0.220457795576195	0.412333837085274	0.405562034532787	0.25473834018135	0.0192683333333338	0.94375604597120
CELA1	0.220440332291579	0.412333837085274	0.0737228514067066	0.905506084101768	0.0325473333333333	0.90945379489823
MED4	0.220403262628225	0.412333837085274	-0.0293773046952356	0.967591608870015	0.111601333333334	0.772139923817113
MAVS	0.220383149539825	0.412333837085274	0.0273206627010246	0.970207612777188	-0.0776406666666666	0.760564116489878
MTMR2	0.220319707420286	0.412409159873773	-0.0190513530484607	0.97832242527852	0.125091000000001	0.551352991056569
GZF1	0.220315829562980	0.412409159873773	-0.0150320440549497	0.982921609770846	-0.211057333333332	0.343247780221912
RAB1A	0.220261560732403	0.412560401636275	0.0879698845039546	0.885848000380851	-0.110318333333333	0.649977903605799
ZNF221	0.220167333918145	0.41285803608584	0.124942912445895	0.824457842812434	0.194022000000001	0.428289389183764
VPS53	0.220052564185033	0.413266552696229	-0.0288660272069172	0.96782691865619	-0.117680333333333	0.601564043046757
HNRNPF	0.220023193274623	0.413266552696229	-0.0567675556905806	0.931488114781041	-0.223920999999999	0.259184854253064
EWSR1	0.219999439496902	0.413266552696229	-0.0273346741744715	0.970207612777188	-0.165749000000000	0.525421163436372
BPY2	0.219958516838315	0.413281314867321	0.0653526439890257	0.918295693698803	0.112030666666667	0.666054079859859
SART3	0.219936155912725	0.413281314867321	-0.0944545661583595	0.874384897652873	-0.0639176666666657	0.779485206901945
KIAA1704	0.219920747390585	0.413281314867321	-0.0235712729590427	0.975348968561756	-0.0310259999999995	0.90616464808104
ZNF562	0.219813733988709	0.413700012203841	-0.0142492986845171	0.983444353656142	-0.0596139999999993	0.804464256710878
LINGO1	0.21976227078532 5	0.413729604290818	0.0281217574960489	0.968668367660302	-0.191502999999999	0.466277136667929
PNKP	0.219752538068755	0.413729604290818	0.24104958717233	0.596648444172109	-0.179060333333333	0.387687154733619
LIN7C	0.219459359122805	0.414770049702684	0.0991312682035593	0.868042453226125	-0.285621666666666	0.158095716859654
PPAP2A	0.219438909936551	0.41480915360533	0.213063459967506	0.655185554924368	0.425657333333334	0.0446858233787091
LPGAT1	0.219292050705022	0.415047408647271	0.140163388277838	0.797441782082887	-0.349886666666667	0.0852927646341535
DARS	0.219288873995307	0.415047408647271	0.404744728425554	0.25480455106032	0.0594466666666676	0.799725367501923
DALRD3	0.219232408691011	0.415285590357455	0.0311292022136898	0.965118873711308	-0.129522000000000	0.537186705207113
DHX40	0.219194802207092	0.415377842950378	0.111672114590370	0.848451264169	0.130112333333333	0.512796060754107
CXXC1	0.219179346123384	0.415377842950378	-0.365049303227167	0.331244161601006	-0.053304999999999	0.823910138699963
PAN3	0.219152064143954	0.415377842950378	-0.131985839968034	0.810300414153187	-0.0943663333333332	0.671825284118867
CCDC50	0.219090282728641	0.415380214500838	-0.0547131913186234	0.934435135880088	-0.0417823333333322	0.874464844016715
ZCCHC14	0.219081795886942	0.415380214500838	0.118885958817923	0.83702538133036	-0.447432	0.12148388169181
C3orf58	0.219045647103679	0.415432539880121	0.401471420512043	0.261687361201831	-0.458762666666666	0.097635308788899
SAV1	0.218922218000700	0.415893567000567	-0.0624527477022191	0.92264749701958	0.118941333333334	0.568903633175651
MEST	0.218695131286911	0.416707201153352	0.0727463758424192	0.907353570590859	-0.0018366666666655	0.99597023247862
PDE3A	0.218633051830177	0.416829475978515	-0.211568911908915	0.659412013514194	-0.156296333333334	0.699705925827209
SLC2A10	0.218576291227268	0.417069944429896	0.0833801714483877	0.892833228561937	0.265067333333334	0.226499104403529
ZNF234	0.218550633983954	0.417089182724873	-0.206065189874212	0.672143058738341	0.112272000000000	0.70610473471702
WDR37	0.218546164639182	0.417089182724873	0.472821490462851	0.145443641689931	-0.443724	0.0372437947587881
SLC9A3R2	0.218402470876366	0.41738365531347	0.256065858096813	0.564792882906668	-0.105184333333333	0.626750668682093
THUMPD2	0.218399233100983	0.41738365531347	0.240186155156235	0.59795653507991	-0.0494939999999992	0.853077816975915
TRIM8	0.218372227823555	0.417388636872549	0.0795642869469638	0.89819077139711	0.0469153333333344	0.843152489417868
PPIEL	0.218256123408463	0.417810614809462	-0.118980579591255	0.836828045978697	-0.181032333333333	0.40500623952892
CD99	0.218112416039946	0.418350994037738	-0.029323815329164	0.967655960277738	0.333480000000002	0.216848931674793
ARHGEF11	0.218014349345096	0.418782356504615	-0.0707540321982016	0.909764940148113	-0.0238333333333322	0.933857541221467
ZBTB26	0.217967040260959	0.418869564287831	-0.0608620770657212	0.92506536457632	0.0883700000000006	0.68717273201429
USP32	0.217956313851347	0.418869564287831	0.00618599426721965	0.992184446520533	0.172573666666668	0.441763847514718
TTC40	0.217945413055407	0.418869564287831	0.00397018470499121	0.994491789164038	0.0281275000000000	0.913644671932166
EPN1	0.217915901142620	0.418940607981883	0.182274226127204	0.719419078472232	-0.219343666666666	0.324443655582244
KLHL25	0.217844226202842	0.418988325705718	0.171741391417458	0.739274192393074	-0.145036333333333	0.516217427447249
ZEB2	0.217763975561347	0.419212665693885	-0.195843704117957	0.692857583007357	0.487250666666667	0.0402148307844276
TTBK2	0.217753605067152	0.419212665693885	0.0885239912267585	0.884688034148025	-0.152899666666666	0.446756640099031
FER1L4	0.217639213707091	0.419453048146725	0.176895582906351	0.729794187782856	-0.0716553333333323	0.777568084061469

ANO7	0.217636384888928	0.419453048146725	0.304502438546501	0.459334784763935	-0.0484553333333327	0.873329245675404
OGG1	0.21761868892656	0.419453048146725	-0.171439848511437	0.739541495555634	0.0624610000000005	0.807091648382304
ALMS1	0.217601079251094	0.419453048146725	0.063382432937097	0.920885810365418	0.0488620000000013	0.876556550350876
CYP4X1	0.217570956170011	0.419456713055060	-0.138135944428182	0.800478820487963	0.211849000000001	0.291481585177291
SALL1	0.21756713739532	0.419456713055060	-0.261086830406214	0.552696861759865	-0.00269899999999947	0.993756838831997
TOP3A	0.217450685912768	0.419772230038917	-0.133847560236327	0.808626799405643	0.0119763333333342	0.97184501043952
VPS13B	0.21730105825203	0.420456040850794	0.181752302330670	0.720487308022801	0.198581000000001	0.351868455836016
BCORL1	0.217204914380473	0.420916862413646	-0.212572101464267	0.656504774622482	0.0151213333333342	0.96433869918248
ZNF30	0.217184532546188	0.420956424050432	0.260577662285296	0.553540075799904	0.0931196666666674	0.720071550811076
CEP250	0.217140403326943	0.421054296605614	0.155643480821371	0.769837060156515	-0.223163666666666	0.354488694820037
OR1J2	0.217026300936293	0.421320172104062	-0.00405273163028803	0.994485841304070	-0.122083999999999	0.550660741372463
ZNF559	0.216968315177394	0.421495294692649	-0.236801020909857	0.604321165518422	-0.0573966666666664	0.813345796866388
MSRB3	0.216708931098447	0.4225709025566	-0.216929704447219	0.647212711808862	-0.365646333333332	0.117157637576458
DNA2	0.216655094043256	0.42259103057904	0.0377186964984552	0.955335956465076	0.0113106666666661	0.974451420920923
ADAMTS2	0.216602599607246	0.422646515900977	0.100134360295748	0.867048047646049	-0.0175303333333321	0.952522040665323
CD46	0.216557847682380	0.422748307025385	0.248337309954819	0.577670812328515	0.33341666666668	0.0895407923270921
CNOT2	0.216506475348758	0.422960886906981	0.346283843145343	0.365990108876469	0.158987666666668	0.431802976936949
CEP164	0.216380814288129	0.423404634889896	-0.0203479850109979	0.976488095728823	-0.243984666666666	0.359771290730514
MED15	0.216269273776728	0.42386715314272	0.165377563132472	0.752352242644389	-0.100400333333333	0.653123035053983
CCDC110	0.216264605190500	0.42386715314272	0.57023885373	0.0451673954411607	0.0752750000000002	0.758098310478085
UBN1	0.216149782283218	0.424277824500044	0.0871376116144766	0.887055939766881	-0.413216333333333	0.0909544784653449
SOGA1	0.216136900893408	0.424277824500044	0.151666339548042	0.777016024293745	-0.0836959999999992	0.759970711908908
WNT1	0.216123141460802	0.424277824500044	0.134277522054709	0.808009477165608	-0.150812999999999	0.577086981531336
SMARCA5	0.216001072156169	0.424378212690633	-0.169626081031303	0.743463590096836	0.00174666666666802	0.995621063297892
ZNF330	0.215973844869298	0.424413817265487	0.146230318664516	0.787402233709509	-0.105718666666667	0.6846855637534
ABHD6	0.21596451171461	0.424413817265487	-0.0924972764109799	0.878406821206854	0.227818333333334	0.408369243753251
PSRC1	0.21595503485816	0.424413817265487	-0.0219281347778571	0.97536722461972	-0.0590779999999983	0.812358226571296
GFRA3	0.215719540338254	0.425360605390087	-0.183838453095143	0.718067440349544	-0.0798636666666667	0.777568084061469
SELP	0.215695342227908	0.425422023718374	-0.278815869260821	0.511025440770733	-0.0341916666666662	0.895093529187836
GMFB	0.215505714866815	0.426229240095483	0.030812105882044	0.96529904314994	0.279122666666668	0.188549331106419
POLRMT	0.215501125605491	0.426229240095483	0.300803579303779	0.465086886291629	0.0454946666666677	0.87709801222343
LHFPL1	0.215486468720624	0.426229240095483	0.194947876017166	0.695325543127847	-0.216322333333333	0.32573269852051
RNF144A	0.215451218071172	0.426344594835493	0.360563135671678	0.34034906917442	0.781290333333334	0.0425543345284076
RGS7BP	0.215380995334511	0.426589928771589	-0.162043365405437	0.75854722544558	0.553206000000001	0.117157637576458
MMP21	0.215257570195610	0.427207711068115	0.11076398326797	0.849357727620213	-0.0327126666666653	0.906093399285191
DEPDC1B	0.215221539078618	0.427335681265534	-0.0214329885795677	0.97536722461972	0.0365680000000008	0.893856182973485
SLC26A11	0.215063402223273	0.427852321033139	0.201530348060463	0.680054545720343	0.230935000000001	0.263247994535131
HNRNPC	0.215009559587647	0.428036591750010	0.104006540100088	0.861570109612412	0.0777200000000022	0.719909781429035
SGK223	0.214979940368881	0.428036591750010	0.0608507617017292	0.92506536457632	-0.0440243333333327	0.86183426299397
NSUN6	0.21497774390193	0.428036591750010	0.25176376629886	0.571012147147691	-0.154205666666666	0.514420666507349
MAGEA4	0.214924231110622	0.428150318458195	-0.305543523715207	0.458013550008763	0.0139456666666671	0.96701817021473
GRB2	0.214897995925657	0.428187823105458	0.0514459666214213	0.938729151196048	-0.0345636666666656	0.895093529187836
RNF5	0.214838802404378	0.428371969354586	0.227038826676395	0.62581495163125	0.0274055000000022	0.919359825156123
PITPNM2	0.214790527821132	0.428544876700904	0.257163478568119	0.563116080558022	0.0205786666666669	0.946623914607177
EREG	0.214781621077397	0.428544876700904	-0.234376878233816	0.60868879500174	-0.145807333333333	0.60378773986659
THADA	0.214746493931666	0.428615783155932	-0.0580251614619594	0.92929701257827	-0.0279689999999989	0.933502474556197
KCTD20	0.214733494600999	0.428615783155932	0.0730790957706981	0.906592046281011	-0.0933726666666653	0.729303008652613
SMU1	0.214726267258448	0.428615783155932	0.291266726300275	0.486802739867898	-0.00133099999999940	0.996285985364494
TIMM23	0.214628561086160	0.428960073234790	0.374278353626123	0.314207741629911	-0.0856064444444435	0.721454370213253
WDR3	0.214533996072354	0.429417505005689	0.0738772126299198	0.905506084101768	-0.272162333333333	0.301793253300179
MXI1	0.214502355743820	0.42944709090507	0.239482133369215	0.599103609129687	0.0487810000000005	0.852969361639689
DKFZP434L187	0.214457845277985	0.429542937801181	-0.22047803201298	0.63932304081136	-0.170489	0.566282142073858
ALDH8A1	0.214390411432330	0.429780050337647	-0.0143300275713029	0.983444353656142	0.0783866666666669	0.727809376593958
PIAS4	0.214358613465366	0.429884770493384	0.387032918076922	0.287581226843956	0.00657966666666653	0.983690244124512
ZNF551	0.21419479938845	0.430732820076983	0.00574573686945093	0.99251028338798	-0.0280599999999995	0.919460556406827
PDLIM7	0.21414948148779	0.430835130712432	-0.290235855478588	0.488568550695131	0.0679216666666673	0.794486775271372
WDR74	0.214141866998504	0.430835130712432	0.0939346035599833	0.875355472414883	-0.115599944444444	0.607103440720429
ID2	0.214115173887337	0.430835130712432	0.467752161481188	0.151936428121882	-0.0416839999999990	0.877940692255388
OXR1	0.214099253415535	0.430835130712432	0.515104367210326	0.0944627871415069	0.0611296666666675	0.84207712482186
INPP4A	0.214029217225891	0.430855002598601	-0.073767805664573	0.905506084101768	0.000987333333334549	0.997462381243084
ELMO1	0.213946485832513	0.431240822582304	-0.423712102399482	0.218176955176854	0.206347333333333	0.309000521568989
PPM1A	0.213789678340885	0.431753615665365	-0.0980640261777958	0.868852648210739	0.131730000000001	0.518178391451896
NCBP1	0.213735098556715	0.431863859826178	-0.0801050276329621	0.897577172229156	-0.105998666666665	0.629243851768459
MDM2	0.213502523904131	0.432631161008946	0.286873124704819	0.494604746861742	0.082161666666668	0.719839702715467
STX18	0.213425623877066	0.432986679032818	0.258147054682634	0.561401773641347	-0.108426000000000	0.594374666543557
ZBTB44	0.213400093106813	0.432986679032818	0.0775943259486243	0.900701176765499	0.119118333333335	0.563120908487291
CYLC1	0.213285343221802	0.433263384644225	-0.0203255108726987	0.976530412432191	0.0709650000000003	0.76368723681351
ERCC6	0.213248556556341	0.433302682570747	0.289818318423591	0.488870852256136	0.0478530000000012	0.864574541922917
ZDHHC6	0.213191257942866	0.433497835889688	0.0565071171949501	0.93176886339657	0.123695333333334	0.540564661517137
RUVBL2	0.213158615210535	0.433511716921192	0.156305934421207	0.768909201237015	-0.311293333333332	0.173286092065797
ATAD2	0.213155808926648	0.433511716921192	0.240776452017313	0.596773408433565	0.0413066666666674	0.893151737593092
CXCL1	0.213096803178133	0.433660277449764	-0.418495075591297	0.231599266507158	-0.0232480000000001	0.954021566744527
MORF4L1	0.212902284296475	0.43453735023526	-0.0578076917784806	0.929715247340158	0.130389666666669	0.516715160469872
PDLIM1	0.212868410602877	0.434569961880974	0.06668589627923	0.915588178522349	0.450582	0.0546896912427088
DCP1A	0.212843972614083	0.434569961880974	0.075808353735273	0.903510413986113	-0.113694	0.605121621910816
HS2ST1	0.212830563008011	0.434571526603041	-0.062681914798939	0.922350559812086	-0.0253689999999988	0.928295508521289
RBM10	0.21264407883041	0.435181128757085	0.00581609211385277	0.99251028338798	0.000705666666666765	0.99792238320329
PCDP1	0.212543691911073	0.435419972497334	0.358029087178173	0.343653896213616	0.197749	0.399848490147388
RRP15	0.212481495472582	0.435419972497334	0.0624415958170645	0.92264749701958	-0.00288133333333324	0.993756838831997
BRD3	0.212462253909732	0.435419972497334	0.162479002402563	0.757685565672998	-0.0467243333333318	0.863479649214073

SNORD8	0.212457583835809	0.435419972497334	0.114839561384936	0.844162087612813	0.232253666666667	0.218103570052522
SSTR2	0.212200222399421	0.436432418663097	0.0988362200547348	0.868042453226125	-0.0686233333333332	0.843152489417868
EFNB3	0.212119268127097	0.436742824753973	0.236950784415352	0.603972819955905	-0.197911666666667	0.458006502146897
TPP2	0.212076095229317	0.436823879524112	-0.273258969500628	0.523997406191732	0.00487100000000064	0.987937064305561
ZSCAN20	0.211931975043902	0.43721067028432	-0.247386603235622	0.579745218830252	0.0463843333333337	0.886500454815894
RNF168	0.211811650052573	0.437314314062237	0.0750183578430438	0.904466414650249	-0.0442033333333331	0.887780036680668
RACGAP1P	0.211795880446752	0.437314314062237	0.162323597321269	0.758164869707887	0.204496666666668	0.332613856483318
UNK	0.211716629146375	0.437467053354377	0.407445921931214	0.252285038466352	0.138568000000001	0.551905208517154
CDK5RAP3	0.211703284161021	0.437468562960131	0.0243074847448856	0.973908542117633	-0.186663	0.338735790842072
ZNF808	0.211681285660018	0.437519176635945	0.0375661995694830	0.955365651737247	-0.118432666666666	0.608311546956177
ZNF592	0.211493555258778	0.438213688865535	0.0141991818350126	0.983444353656142	-0.0329260000000001	0.904236796123256
CACNA1I	0.211364997356738	0.438691668299098	0.274576476758658	0.520384310345519	-0.00477666666666601	0.988133186272453
F13A1	0.211336042891037	0.438691668299098	-0.274592607624441	0.520384310345519	0.0202226666666671	0.945904449997854
NCK2	0.21127457649885	0.438691668299098	0.30042372473843	0.465824202471203	-0.296668999999999	0.158751033455501
HERC2	0.21127247316841	0.438691668299098	0.0967977957494387	0.870742142315919	-0.0116171666666658	0.97482729795816
GBF1	0.211197760064665	0.438800976282356	0.170665358675626	0.741573453363653	0.0380440000000011	0.894769940876883
PKNOX1	0.211194191604113	0.438800976282356	0.352339497525455	0.352921806679009	-0.103891333333333	0.633166039956913
CCNT1	0.211105904718003	0.438976500223113	0.126740449488358	0.820923134671156	-0.128129333333332	0.535171605667144
PTPN14	0.211101319018713	0.438976500223113	-0.0746959672525692	0.904466414650249	0.284128333333334	0.195927187569286
TESK2	0.211072999221212	0.438976500223113	-0.159964412135693	0.763108489884256	0.295553666666667	0.183344879272687
TPBG	0.211032819894353	0.438976500223113	0.486229674725974	0.131857988484294	-0.134603666666666	0.501839684921699
SEC24B	0.210985875295962	0.438999399524847	0.107787498335034	0.856450662791083	0.0189366666666682	0.94871117680979
SATB2	0.210963581399111	0.438999555698935	0.272070592068238	0.527284725671175	-0.0745283333333323	0.739693825693123
ST8SIA3	0.210871756223457	0.439299072790232	-0.0875306313292587	0.886302037299497	-0.153774	0.476612562280949
OR10H2	0.210788685285117	0.439548993628594	0.0364230891261714	0.957597361653107	0.0590100000000008	0.829369385827456
CENPF	0.210722674327873	0.439715421610713	0.116609800926998	0.841809730226979	0.00437100000000067	0.990930928353104
ZC3H14	0.210720325225919	0.439715421610713	-0.156766097580867	0.768487306347322	0.129442333333334	0.536810332635776
FAM83E	0.210648127292827	0.439944451721686	0.0990054151267118	0.868042453226125	-0.0420346666666657	0.911623909907545
SSX2	0.21064096694867	0.439944451721686	-0.0531082045094037	0.936712858731269	0.0458535000000009	0.852608879235174
ABLIM2	0.210624871616329	0.43996193817696	-0.165849661947287	0.751869806279813	-0.290780666666666	0.141475366389837
OR2F2	0.210527982180737	0.440364824528036	0.244605313071534	0.587653287154633	-0.243756666666667	0.408369243753251
MEGF8	0.210434893741186	0.440741510907494	0.114124799974227	0.844272085764738	-0.124038499999998	0.649810088586073
TRO	0.210410082188422	0.440741510907494	0.0205975144515580	0.976404959469803	0.183088000000001	0.442294185443201
ANKRD13C	0.210400767246534	0.440741510907494	-0.0387393608473377	0.954263089081373	0.161174333333334	0.455750758726527
SLC25A36	0.210396690134163	0.440741510907494	0.232609337455961	0.61211578778651	0.241078333333333	0.317651195904626
TUBA8	0.210303318226893	0.441124825032821	0.332860154236410	0.397658808533403	0.0973800000000015	0.638001921634905
SCUBE2	0.210286076760304	0.441148901667171	0.0327183349985637	0.961351562552925	-0.087604	0.748799811961987
URI1	0.210216336381093	0.441471951607164	0.117014195509622	0.841189837368881	0.119246666666666	0.5488879250835
KIF11	0.210183243142929	0.441571870902861	0.0821739893542287	0.894840896036786	-0.0610676666666655	0.797408129996359
SMC1A	0.210098175779688	0.441688903551785	-0.0400888216669812	0.953163386277443	-0.110975000000000	0.65150906147497
C3orf62	0.210077559138076	0.441688903551785	0.06498183322368	0.918489100363712	0.124130111111111	0.662061383945888
SRRT	0.210055534915475	0.441688903551785	-0.040330697318651	0.952720585102517	-0.0785663333333329	0.718749100405876
C9orf50	0.209978823567864	0.441688903551785	0.0542365900727582	0.934724166908893	-0.0154443333333324	0.970094446214144
ELOVL4	0.209977461049035	0.441688903551785	0.343332264007802	0.374265187515147	-0.265306666666667	0.438562514747617
NFS1	0.209975136668523	0.441688903551785	0.267765812446162	0.53559545997875	-0.234729333333332	0.247218027506970
DNAJC22	0.209922702422401	0.441688903551785	0.411849738596307	0.243463779196104	-0.161278	0.501234066666842
C2orf68	0.209905303674820	0.441688903551785	0.214653209092398	0.652031766366328	-0.152613333333332	0.525218637875881
LRP1B	0.209889877461492	0.441702827945854	0.229932376310445	0.619309047495672	-0.106384666666666	0.628477077807168
ACVR1	0.209843349665749	0.44178190332389	-0.120180721903974	0.835124910268666	0.108500333333334	0.609871998781997
GABRG3	0.209762507259391	0.442058604113971	-0.144471178081274	0.790414495161718	-0.0485580000000001	0.834476346642783
RRP7A	0.209716155774895	0.442199617717621	0.336470125668456	0.389160743581898	-0.273389666666665	0.148479926866127
INPP5A	0.209590991781481	0.442626402478662	0.348652111468388	0.361261311685225	0.0929600000000013	0.761837433189273
SMCR7L	0.209585004653233	0.442626402478662	0.0951004336303356	0.872962271153999	-0.130329666666666	0.532226677211706
XAGE3	0.209471727756712	0.442785848013971	0.187660155514333	0.708767689702884	-0.0328679999999997	0.905071365396373
HOXA2	0.209456491864372	0.442785848013971	-0.232418569751229	0.612658112747496	-0.0192193333333336	0.947975216802271
PHLPP2	0.209439173029266	0.442785848013971	0.153279928456677	0.774003008542746	0.00638833333333428	0.988133186272453
C3orf38	0.209437404895289	0.442785848013971	-0.408233654770025	0.250856680899233	0.0323516666666673	0.90945379489823
TTC30A	0.209427402538348	0.442785848013971	0.0983269819596142	0.86848759013952	0.288170333333335	0.130824456840031
MUC15	0.209327457689004	0.443133952643124	-0.2919773983937	0.484940697058018	-0.174747666666665	0.402383488486404
CRYBB3	0.209245470239500	0.443379850415257	0.159232506362235	0.764789523460873	-0.146381000000000	0.591142517306808
BIRC6	0.209222741806013	0.443435608565087	0.135869212951005	0.806017283558846	-0.187990333333333	0.383593963545918
GGT5	0.20919532487096	0.443458808641621	0.248799881346477	0.576871285544251	-0.0851403333333333	0.721484595160786
OLFML2B	0.209087297058839	0.443765068822972	0.121589475514098	0.831310831659304	-0.267847	0.291501950143291
UBE2Q2	0.209043899008515	0.443837458770733	0.177908411487163	0.727982464250053	0.0865620000000015	0.698522934873187
DPY19L2P4	0.208986123941452	0.443862895832274	0.261759901264646	0.550803906509978	0.381412333333333	0.174531226684713
CKAP2	0.208964813529704	0.443862895832274	-0.113933882528468	0.844272085764738	0.118711000000001	0.627861222373769
ZC3H10	0.208867306954737	0.444282077492737	0.0742057562390256	0.905252113490377	-0.151440000000000	0.440032364849398
LRRTM4	0.208828737567635	0.444370825616373	-0.202911625730638	0.677532075317915	-0.0946143333333329	0.760951989611575
ENC1	0.208825895374211	0.444370825616373	0.293861441342666	0.480614908214975	-0.475264666666665	0.164159088342059
CCNT2	0.208725533552794	0.444501038601925	0.326609048756481	0.409206882509236	0.296056333333334	0.226059303638588
DTHD1	0.208647885266308	0.444870467996613	0.113862361092740	0.8443707707356	0.0780260000000003	0.77895955075587
RCAN2	0.208587084626033	0.445109310246671	-0.066323645318822	0.916518252419605	0.198096000000001	0.583036318848746
HOXB3	0.20858021540591	0.445109310246671	0.102119924959032	0.864337135688791	0.0498196666666672	0.861165447055876
HHIP	0.208547218945474	0.445223974956706	0.210600838816061	0.661670522548494	-0.207468666666666	0.458006502146897
IFITM10	0.208512660182169	0.445347584465993	0.217341990410791	0.645781950047533	0.530597000000001	0.0453495824797086
TANC1	0.208414654183276	0.445700766544151	0.161599967655613	0.758955920988521	0.143327333333334	0.492100665301584
CREB3L4	0.208359687164434	0.445785214429039	-0.0734331787294005	0.905838142287595	0.0590833333333346	0.855317148872886
LHX4	0.208358576662006	0.445785214429039	0.110913432589123	0.849357727620213	-0.0904576666666663	0.68171195530621
GREM2	0.208200987800842	0.446300065946442	0.195036398572259	0.695157516663803	-0.959681666666667	0.00462417727395729
IREB2	0.208123636414162	0.446411432856291	0.156632237290246	0.768851997174833	-0.131066333333333	0.512796060754107

COL5A2	0.208105861784707	0.446411432856291	0.0633188968745217	0.921012753856558	0.0689646666666671	0.859412082013537
PFAS	0.208056389402725	0.446411432856291	0.0626642561749849	0.922350559812086	-0.294277999999999	0.158485868172990
DNAJB12	0.208008156096149	0.446411432856291	0.289317822545231	0.488889656845544	-0.138956333333332	0.524210311918454
FUT8	0.2079375975642	0.446716913688695	-0.0583029236294261	0.928766151140997	0.0243880000000006	0.934144092662458
SIK3	0.207901444313025	0.446776269449135	0.153886407439813	0.77284838227453	0.101544000000001	0.702088376583556
RAPSN	0.207843743038794	0.446885212978983	-0.0526362933617579	0.937357975161644	-0.158815333333332	0.536320838519271
SORCS2	0.207803832656421	0.446955625484715	0.278731703176972	0.511025440770733	-0.123846999999999	0.66132749915999
SLC27A1	0.207792791371456	0.446955625484715	0.497805682911506	0.114179995542754	-0.0220953333333333	0.942800137888472
ERAL1	0.207720242660281	0.447223280407224	0.295848237202731	0.47761182409076	-0.0626926666666667	0.801352759417909
XPO6	0.207690297604312	0.447299157964814	0.172762340191525	0.738192208453856	-0.0732400000000001	0.75829995960291
KISS1	0.207527051084612	0.447886843068816	0.315870684537276	0.433880593421022	0.00886933333333406	0.98256318768706
KLF16	0.207455345162056	0.448145891362268	0.164170367115770	0.754189116283047	-0.228441999999999	0.25178638718411
NASP	0.207447191052311	0.448145891362268	-0.0153586659222086	0.982725338620588	-0.00846066666666609	0.98025379113353
MMP16	0.207352466263133	0.448370768485554	0.199409732133036	0.684056115356325	0.395589166666668	0.0714536337053717
IRX4	0.207322042106431	0.448397666922222	0.0328236376534331	0.961351562552925	-0.163484666666666	0.525218637875881
PLXNA3	0.207243380843162	0.448700946471924	-0.21627685914151	0.648391670659269	0.0738976666666668	0.811816884537132
PAWR	0.207089482469914	0.449362023871284	-0.0237424097074016	0.974934434225626	0.143948666666668	0.553997328691896
SLAIN2	0.206879680841240	0.450270852435049	-0.352768438737884	0.352560848501456	-0.0906119999999999	0.675445304679433
ID3	0.206847242296286	0.450310473930399	0.0915707578017026	0.87943055126106	-0.182957000000000	0.477467429367264
EED	0.206847057775306	0.450310473930399	0.182970595330099	0.718289071039953	0.0776113333333338	0.758250508013307
XRN1	0.206773275409762	0.450347958869976	0.0651386039508072	0.918489100363712	0.222451000000000	0.312556712937762
SULT1B1	0.206745920331496	0.450347958869976	0.178748479052944	0.726057281492174	-0.0617506666666661	0.82667899725046
FAM177A1	0.206708074486316	0.450370486588258	0.0458536356738342	0.945372627844176	0.210593333333334	0.502832431909902
RAB26	0.206661013893461	0.450493184415040	0.145498300405049	0.787804113309913	-0.122629666666666	0.544685122345675
RAD23A	0.206624785527119	0.450553722245452	0.456467431064992	0.16754724558121	0.00516233333333444	0.987851590533963
ST3GAL2	0.206596565883438	0.450560986694626	0.173977683195581	0.734793595952544	0.0136703333333343	0.963626568322179
WDR43	0.206582710777228	0.450560986694626	0.0114221870414495	0.987215779667937	-0.183110666666666	0.344663423870532
CRY2	0.206440564003861	0.451141273619072	0.163729398880059	0.754968203901817	-0.0992906666666666	0.634587745028953
ZBTB6	0.206394822832441	0.451284707299653	-0.133153761781707	0.809318769887813	-0.0269299999999982	0.921927799352682
NTRK3	0.206335024627184	0.451480857694211	0.131782751734989	0.810671652076995	0.0876706666666667	0.695654544311453
TARS	0.206319741002790	0.451494947470856	0.40850417898524	0.250484125644429	-0.154699999999998	0.442322325044862
ATP13A4	0.206203291946551	0.451743089308255	0.0336629247423631	0.96061727828906	-0.0639089999999998	0.7703486664151
SRY	0.206131435742265	0.451987544024575	0.218779568768347	0.642137448592891	-0.0103443333333328	0.983159241812861
FAM19A1	0.206072513759699	0.452226269592337	-0.168078708282590	0.748024524669236	-0.226204333333333	0.23765426508992
UNC80	0.206039835031456	0.452288136083943	0.104879263240051	0.86067831050617	-0.0624989999999995	0.831432210225512
PRPF4	0.206008339071900	0.452326815515029	-0.0488845748954697	0.942180113539637	-0.213436666666667	0.277510060203337
TERF1	0.205945298787847	0.452615748121717	0.464339287733472	0.156706752619037	0.0568268333333345	0.851568943534661
BCAN	0.205916479599931	0.452707826645227	-0.0750154737531474	0.904466414650249	-0.320540666666666	0.281579610467498
DIO1	0.205821372964260	0.453176868516581	-0.155651146740146	0.769837060156515	-0.0855729999999997	0.697299941610908
NSD1	0.205770918468665	0.453176868516581	0.0783761895222174	0.899281198071059	0.0747756666666672	0.77720587295293
MYBL1	0.205699030596845	0.453517097155771	0.479787266221741	0.138290570952161	0.0134950000000004	0.979527173532206
BLMH	0.205634448635405	0.45374153065952	0.139095824352541	0.79864602540281	-0.263805999999998	0.288015995370752
KLHL14	0.205503393126593	0.454201531148586	0.353890470979791	0.351918563636471	0.0236766666666673	0.937717709824356
PPIL4	0.205461152984457	0.454297357957358	0.0380519722686697	0.95472785695334	-0.253634666666665	0.194593860853488
IFT81	0.205384002304521	0.454668451826201	-0.0695837025086861	0.911313423064389	0.0464990000000006	0.895093529187836
SLC26A4	0.205338833976206	0.454855155032421	0.350128530569325	0.358738646007386	-0.0294476666666665	0.907648578460912
FRYL	0.205240177994899	0.454907196375431	-0.161615593209982	0.758955920988521	0.0167795000000000	0.96087920699545
C9orf16	0.205128086653412	0.455286748412102	0.369481980227626	0.323285574998587	0.0224543333333353	0.953335531487028
ECD	0.205123210026774	0.455286748412102	0.158777056191281	0.765730464375784	-0.0692796666666662	0.775011972945107
NID1	0.205069463147937	0.455523141946677	-0.0716905666344815	0.908481403751751	0.523212000000002	0.0214024833516911
PDE11A	0.20504139662151	0.455611308835235	0.100932851131087	0.866005093242444	0.410365666666668	0.41785973815832
XPO5	0.204937929669465	0.456025669436935	-0.00529307988219112	0.99251028338798	-0.138324666666667	0.615689623138822
CTTNBP2NL	0.204852463368424	0.456407134193479	-0.530919974556238	0.079070436079089	0.0582970000000005	0.811199233675225
KCNK7	0.204794191114916	0.456415729889017	0.132711930797411	0.809588765729788	-0.0542973333333333	0.821421133422134
EPHA4	0.204689912778395	0.456829192172613	0.337009880192658	0.388337351990747	-0.404690999999999	0.0932478593898661
ANKRD6	0.204664772612999	0.456900618245089	0.205497617339081	0.672727218951913	0.0724826666666673	0.735938662411184
DES	0.204394147893227	0.457847695225033	0.140374010411379	0.797441782082887	-0.0931466666666655	0.67262320052782
CEP72	0.204360467250415	0.457847695225033	0.04090935435604	0.952426531595115	-0.0920846666666656	0.694234871713013
ZSWIM5	0.204297065829764	0.458066802721256	0.0205930187521566	0.976404959469803	-0.00619633333333193	0.985484787924072
RBM14	0.204255897625471	0.458231213738889	0.0233723414820201	0.975348968561756	-0.129615555555555	0.556248360059405
SIM2	0.20423766751989	0.458262796705568	0.495884650745087	0.116658734321069	-0.0771646666666665	0.767099449353307
BAG5	0.204109931906995	0.458484808895989	-0.111526032905997	0.848875498955953	-0.323031333333333	0.100026144425143
RABGAP1	0.204075122137806	0.458612487738251	0.074715916411664	0.904466414650249	-0.255259999999999	0.216663334151092
GON4L	0.203972955562007	0.459036038196187	-0.181725134679462	0.720487308022801	-0.00920333333333265	0.979790222743567
SSR1	0.203848779732403	0.459554468652949	0.104703851880482	0.86067831050617	0.0590333333333337	0.789330177521372
C10orf55	0.203828064396674	0.459600575605096	0.304404947734827	0.4594472826444	0.140985666666667	0.477871006905274
TMEM184C	0.203798775173639	0.459622388350914	0.232647168408435	0.61200913457472	-0.0734996666666659	0.733867173296405
CWC22	0.203719321047835	0.460009305835715	0.0764328270511702	0.90277020200366	0.108615666666668	0.619996256183009
LOC100129455	0.203678321800071	0.460130679329741	0.389061935314985	0.283906329711198	-0.0857146666666665	0.729303008652613
MARVELD1	0.203672881030547	0.460130679329741	-0.104610559003641	0.86067831050617	-0.132347999999999	0.529843823569029
STAC2	0.203599494221992	0.460408521295266	0.125192861103566	0.824230922676958	-0.00150366666666620	0.99628178244892
PTK2	0.203565229056387	0.460533381780119	0.280544794894743	0.507460240190768	0.0905493333333339	0.672980097690282
DDX39A	0.203478998794801	0.460662833375828	0.29368539952565	0.480614908214975	0.00604300000000048	0.987937064305561
ASCC2	0.203476667100325	0.460662833375828	0.0263158149649958	0.970631762839502	0.0609193333333343	0.780474051101386
NET1	0.203466393746587	0.460662833375828	-0.1339248729463	0.808567812988011	0.0538206666666678	0.825979744677476
HIST1H2BC	0.203337120934895	0.461007435085684	-0.429221625069339	0.209103669076588	0.228057666666666	0.302861002390456
MAGEB2	0.203330554918547	0.461007435085684	-0.0613132121741476	0.924230096951398	-0.0289789999999994	0.920830042057353
LLGL1	0.203244136303451	0.461287321691037	0.0292580924949224	0.967657386725432	-0.172137666666665	0.367206442400058
SPZ1	0.203186426820133	0.4615486078897	-0.0135416205631974	0.98412883278237	0.104911000000000	0.639378811691804
CYB5B	0.203150652508441	0.461608395672996	0.226474935713627	0.62700289306617	-0.142603333333334	0.481422642183253

TRIB2	0.203085391367003	0.461692577347343	0.23718215116826	0.603350164283107	-0.0393243333333322	0.883739719585572
DCX	0.203069353212424	0.461692577347343	-0.0982735683570453	0.86863497725203	-0.0971273333333331	0.714961810216865
WDR19	0.202974030935035	0.462015828833737	0.0608749768696181	0.92506536457632	-0.0623749999999993	0.7920412344971
PDE10A	0.202948626673889	0.462015828833737	0.0641830023290322	0.919923865590619	0.0630066666666667	0.774210549173745
POLN	0.202936477426317	0.462015828833737	-0.139127787265823	0.79864602540281	-0.0783723333333333	0.729912257977231
KCNJ4	0.202909063382035	0.462050369864595	-0.24169517462603	0.595201219333254	-0.0100136666666658	0.978109825558428
RCOR3	0.202882025810264	0.462128832741706	0.168372873405688	0.747452909400868	0.254683666666667	0.274914613771951
ST6GAL1	0.202834945431680	0.462128832741706	0.116152241214489	0.842507799216635	-0.645536666666666	0.0142639134811873
USP38	0.202831948134906	0.462128832741706	-0.114683215264262	0.844162087612813	-0.156654333333332	0.441460337585051
POU4F1	0.202716632728294	0.462636921212931	0.205686589527344	0.672625944768829	-0.0157929999999991	0.960210715969161
PRKRA	0.202704799766627	0.462636921212931	0.393917634786817	0.275391712905480	-0.0712446666666652	0.752734121013624
U2SURP	0.202676767390051	0.462636921212931	0.11707374367867	0.840993954510842	-0.0401769999999988	0.895093529187836
MCM10	0.202567461251278	0.463075264091958	0.0846963199397142	0.891309254245872	-0.148480333333332	0.514375987243015
PCDHB2	0.202455945879249	0.463354309996972	-0.348503769693493	0.361311804770494	-0.0621483333333328	0.788228330273453
DDAH2	0.202403951259421	0.46336077480947	0.177429496547986	0.728541130314952	0.258066444444445	0.210845715069665
ABHD13	0.202386114313018	0.46336077480947	0.0288134442680838	0.96782691865619	-0.0361453333333324	0.883478349705756
TFB1M	0.202341158801209	0.463430839944666	0.308883024756171	0.449515205044353	0.138829000000001	0.524145049711131
KLF6	0.202300992347289	0.463506877978689	0.0171370770472460	0.98105020869911	0.195968333333333	0.456391360723819
CLDN18	0.20226518829319	0.463506877978689	-0.113015163384375	0.846505494838606	-0.0242933333333334	0.951326596208643
NXPH3	0.202250288227111	0.463506877978689	0.125563580545565	0.82367707928204	-0.407434999999999	0.0647596980686847
SPRTN	0.202226542177873	0.463506877978689	-0.00612321195626508	0.992286213306377	-0.0134573333333325	0.966342921970461
LOC96610	0.202164391279256	0.463647033557886	-0.519342769575946	0.0902262536464163	-0.179659999999998	0.342002927568267
RIBC1	0.202146285785600	0.463678574709966	-0.0409276100498758	0.952426531595115	0.0295730000000009	0.92127681920952
FAM184B	0.202109801359604	0.463713594189307	-0.041457494758425	0.952098348256047	-0.0436793333333324	0.906090978358452
CELA3B	0.202097474608606	0.463713594189307	0.248700243238203	0.5770020684641	0.0153390000000013	0.979355014372038
CSNK1E	0.202089577125202	0.463713594189307	0.106584294516104	0.858072018696671	0.0817586666666679	0.704169495024335
CARM1	0.20204995208922	0.463794603504122	0.223995756166519	0.633436730790476	-0.0770366666666657	0.727809376593958
HIST1H1T	0.202017740538318	0.463794603504122	-0.0270032072795902	0.970207612777188	0.393701333333334	0.0549655661708422
KIFC1	0.201992276841542	0.463794603504122	-0.0304461397451063	0.96583351705897	0.115221333333335	0.618038241012329
SNRPD3	0.201990888207441	0.463794603504122	-0.0892998549936092	0.883460564717377	-0.0855523333333326	0.697261622653146
TPM2	0.201964112958568	0.463794603504122	-0.126719270824066	0.820923134671156	-0.0367260000000001	0.88616294579686
RNF26	0.201872865323439	0.464163254043601	0.110345060889687	0.85007214344374	0.173548666666668	0.441730896500427
LOC440896	0.201765655428747	0.464492364108428	-0.108169211915010	0.855230880438744	0.192689666666667	0.558807324932864
FBXO18	0.201426073207352	0.465511701858781	0.085459259830542	0.889704423033556	-0.0326069999999998	0.91143191097679
NRF1	0.201396830178525	0.465534680801173	-0.0362853083887814	0.95779371752526	0.0596320000000006	0.816562434074848
COL4A5	0.201291595480432	0.465926802776661	0.109713695041510	0.851195083614214	0.0129936666666671	0.969387309763954
HEXIM2	0.201180575879562	0.466405619443364	0.147268500665399	0.78561060123541	-0.0462866666666667	0.871724803785777
PPIB	0.201010696467487	0.466975081774104	0.0285388303643329	0.967880249275467	0.0841633333333342	0.696194439936211
C12orf4	0.200922253789922	0.467269938707	-0.31123716902661	0.443354053992282	-0.551810666666667	0.0235679723094208
CUX2	0.200874930688990	0.467397067224662	-0.0097572705610386	0.98909166696601	-0.361383999999999	0.241273257416922
WNT5A	0.200862930327327	0.467397067224662	0.155708307690439	0.769837060156515	0.364537666666667	0.1409133773217
SOX4	0.200764288544226	0.467550152449231	0.0276376199262869	0.969740023371175	-0.316863666666665	0.233528575266801
TAC3	0.200752661130062	0.467550152449231	0.0466196223795035	0.944599178367537	-0.00664999999999958	0.98349947509937
METTL8	0.200751697070803	0.467550152449231	0.171372770615921	0.73955239116035	-0.000959999999999928	0.997144862160692
TUBB2B	0.200747905063179	0.467550152449231	-0.152121580165780	0.775920338550028	0.127020000000000	0.551905208517154
LINC00052	0.200653545864983	0.467953655368110	0.0208540672679619	0.976330597778846	0.0793220000000001	0.821421133422134
VPS13C	0.200631361893231	0.467963057507189	0.0287262449410934	0.96782691865619	-0.112972666666666	0.666054079859859
TLCD1	0.200625111628499	0.467963057507189	0.218057950056248	0.643730951697261	0.0128500000000004	0.970481529511925
UNC93A	0.200526405004946	0.468212047491129	0.256875029260919	0.563568369086911	-0.153829333333333	0.459836297629535
LRRC37B	0.200495913723891	0.468212047491129	0.264902833719448	0.542477070548696	0.252133000000001	0.328178286792292
LGALS14	0.200211280631312	0.46912108770892	-0.0328317619055099	0.961351562552925	0.031168	0.910190419163007
FREM2	0.200088618413484	0.469338166860963	0.278683495452271	0.511025440770733	-0.0290059999999990	0.946623914607177
MNT	0.200020610770285	0.469623805463508	-0.0441792214937888	0.948493391127553	-0.0416876666666663	0.874353540431336
FREM3	0.199981073063179	0.469623805463508	-0.155851998857641	0.76972820876143	-0.204189999999999	0.551352991056569
MICAL1	0.199979173483715	0.469623805463508	0.206987107974549	0.669713208801491	-0.190042333333332	0.340052241399374
LRIT1	0.19997676935055	0.469623805463508	0.226192337597233	0.627911885602788	-0.0742696666666666	0.820041994125866
DKK3	0.199833278750533	0.470242163341617	0.472656144670929	0.145443641689931	-0.108085666666666	0.691940834235362
FAM20B	0.19979206358294	0.470335936599847	-0.0109792385867754	0.98774001793194	0.198522666666668	0.297888842945994
FAM24A	0.19976430347473	0.470424700192004	-0.0782891534410541	0.899605628119637	0.0945833333333339	0.771373465365664
GDI2	0.199717006519137	0.470520370661667	0.0116452531036509	0.986654146365942	-0.00379666666666523	0.990402175833635
CPEB1	0.199710180425321	0.470520370661667	-0.182377856519793	0.719419078472232	0.213973333333333	0.33651414986765
BCR	0.199675110393800	0.470636108597492	-0.454574734356789	0.169906238158850	-0.0213819999999980	0.938187566801362
GTPBP1	0.19966522857289	0.470636108597492	0.326203474329056	0.409206882509236	-0.230303999999999	0.367081032943231
DDX52	0.199525054597393	0.471007965963812	0.0915097506852771	0.879662611652185	0.00107800000000117	0.997144862160692
CLEC18A	0.199513615819244	0.471007965963812	0.226204172998302	0.627911885602788	0.0442896666666669	0.851245530347329
MNDA	0.199447252819626	0.471322950110419	-0.152344914423609	0.775863712880333	0.063236666666667	0.83559019443239
CDR1	0.199383430583324	0.471469403017844	0.205240862193648	0.673296856274784	0.516566000000001	0.0917235990272545
PTPRE	0.199308221260196	0.471575858329526	0.294138338384406	0.480066023124667	0.132958333333334	0.525143015861964
PIAS1	0.199287505719103	0.471575858329526	0.31287164631376	0.438968251704482	0.0209100000000019	0.937717709824356
MYLK	0.199284146810194	0.471575858329526	-0.141196203908826	0.796285336930078	0.741707000000001	0.0584908310916042
KCNG2	0.199278309954051	0.471575858329526	0.151680143283505	0.777016024293745	-0.0438463333333322	0.910244184747474
LMAN2L	0.199080015081068	0.472443368362046	0.258667032082508	0.559935260647793	-0.0369366666666658	0.886909407509907
SLC10A4	0.199040803511770	0.472599614131383	0.0592032936809696	0.927427607950847	-0.164034666666666	0.436069495373885
ATF1	0.198977836274231	0.472747772578229	0.0394544982133233	0.953905313456582	0.191472000000001	0.390967882720418
LOC100507388	0.198857807962473	0.473157089637854	-0.0652879336131268	0.918295693698803	-0.0293939999999997	0.928897428735123
DGCR2	0.198833879945312	0.473223776870705	0.195189757093813	0.694852194384891	-0.128978666666667	0.550568975738867
NLRP14	0.198789467724452	0.473379856425847	0.0711192845233547	0.90935479990339	-0.0255183333333328	0.941559580750404
BCL11A	0.198717185352295	0.47346633197412	-0.00928593915966524	0.98909166696601	0.323990666666668	0.375553176012692
SOS1	0.198649001516937	0.473719168971443	-0.120777382370036	0.833478079604586	0.212003000000000	0.32631478390653
FANCD2	0.198529351637314	0.474274432078101	0.068827057519902	0.912149839732916	0.0683300000000023	0.789377975672973

METTL1	0.198514500607848	0.474287905023017	0.313656994582196	0.438046311563112	-0.145171999999999	0.459624813256835
RCN1	0.198396514079016	0.474686144087388	-0.0125028404916239	0.985220352735461	0.0971093333333353	0.639002829892966
CIDEB	0.198343237442393	0.47485174011631	0.446733251267017	0.178661417730793	0.0130710000000019	0.981710634034817
LMCD1	0.198228838497517	0.475271887531312	-0.257744963510247	0.562492892055072	0.193035666666668	0.358607902953009
CEP89	0.198105509260801	0.475733290609846	0.194499752426267	0.696263722143486	0.076051333333333	0.741121617241292
NLRP10	0.19807254246959	0.475822824838838	0.421829132304836	0.222348587392621	0.116541000000001	0.60217016768875
PCNXL2	0.198040156700086	0.475822824838838	-0.0387300047556561	0.954263089081373	0.0330080000000002	0.896699926823174
CAPN7	0.197991622681722	0.475960506413825	-0.0756645549071821	0.903510413986113	0.128553333333335	0.523762186913243
CSMD3	0.197953820345254	0.476063212021981	-0.28683720581354	0.494604746861742	-0.0717426666666664	0.739188468398639
BPI	0.197888946059153	0.476281337262419	0.270368204552527	0.529999252995877	-0.036190333333333	0.89052375260746
HNRNPH1	0.197880089206387	0.476281337262419	0.153699383146053	0.773358453580566	-0.0804933333333322	0.718623590071188
KIR3DL1	0.197874414119115	0.476281337262419	-0.179739980208261	0.724074699453211	0.238853833333334	0.216858796679299
PDGFC	0.197846772652306	0.476303756647883	0.399947490654386	0.264271262947169	0.124739333333334	0.626750668682093
HDLBP	0.197808413341846	0.476360566098201	0.0837204583052805	0.892833228561937	-0.154561333333333	0.464894078628082
ECM1	0.197756527664092	0.47649303188639	0.0254213999085733	0.972264538231849	-0.166501999999999	0.458006502146897
VRK1	0.197749988318564	0.47649303188639	-0.091331649632361	0.880285315850344	0.0392906666666681	0.888181586513563
C19orf26	0.197637567923610	0.476809857936961	0.251654735608613	0.571309031755718	-0.166382666666666	0.461494497197158
MGAT5B	0.197504121522486	0.47687245681861	0.211005152130129	0.660645714735488	0.0912733333333344	0.741438256509195
TEAD4	0.197500058366403	0.47687245681861	-0.372552301096404	0.317268019532456	-0.0886066666666656	0.694407056566956
NXPE3	0.197498437122333	0.47687245681861	-0.157765855802287	0.767726202151986	0.0336923333333346	0.899614658619676
ZBTB40	0.197417323392300	0.477276405535069	0.249451994606782	0.575390588182915	0.0596400000000008	0.837845337512374
MIR142	0.197357277844827	0.477429355795668	0.114109947756878	0.844272085764738	0.00801866666666693	0.981158983767931
TMEM47	0.197306453453658	0.477535081302344	0.00848658171004518	0.989705545062205	-0.586895999999999	0.0185304620090284
HEXIM1	0.197275012637926	0.477570010368482	0.100481618193304	0.866539251141083	-0.0895083333333324	0.691326558186115
USPL1	0.197267399342119	0.477570010368482	-0.130715640020377	0.812271254601992	0.00522200000000143	0.987937064305561
DNAJC13	0.197119200115485	0.478001178731258	0.020121983682222	0.976988909057692	0.218536666666667	0.288133796028617
PKM	0.197055760161007	0.478227730852761	0.295339301983685	0.478306608395718	-0.006086666666665	0.985544323496696
KRTAP3-3	0.197034840575456	0.478237188217809	-0.08680540820466	0.88734162241363	-0.0339873333333334	0.891803584952102
GPR133	0.197012400852792	0.478262312537398	0.178983441031054	0.725831949422059	-0.125034000000000	0.733933387497966
HPDL	0.196685659452698	0.479807820116055	-0.0639580435640059	0.920208600812857	-0.0509763333333317	0.823910138699963
KCTD6	0.196655829459677	0.479807820116055	0.00836031140502923	0.989949154584002	-0.0276089999999988	0.912119509131266
CDC14A	0.196650469654063	0.479807820116055	-0.389267433205871	0.283516069208756	-0.134005333333331	0.567241234616033
YOD1	0.196484743385945	0.480323409317405	0.0264667915001132	0.970351183565168	-0.0421864999999999	0.873022275431289
MED19	0.196471868893259	0.480323409317405	-0.0134740227226703	0.98412883278237	-0.0610119999999983	0.785482517274791
GTF2E1	0.196383904609301	0.480510710663098	0.304987042985803	0.458359470895422	0.221483333333334	0.238780185222418
TP73	0.196371769478277	0.480510710663098	0.251489369772407	0.571839986662516	-0.0322503333333335	0.904178262468472
C2orf70	0.196369108137803	0.480510710663098	0.0820385008539405	0.895064720363003	0.0681500000000003	0.774104133965233
UBE4B	0.196267420286335	0.480802943046876	-0.130707671117414	0.812271254601992	-0.0981116666666665	0.649977903605799
FXYD7	0.196257246549603	0.480802943046876	-0.0512626950839054	0.93900293341482	-0.146655333333332	0.466793078197373
ADAMTS6	0.196209465568508	0.481011589444425	0.178091690361304	0.727939088352802	0.752034333333335	0.00629285391915396
C17orf53	0.196149680949724	0.481105804958761	0.0363662377896422	0.957597361653107	-0.166669333333333	0.388303383752735
PSMC5	0.196121905382951	0.481105804958761	0.0381008642872413	0.95468180561467	0.00228500000000068	0.994819507544822
IFNGR2	0.196118985606893	0.481105804958761	-0.0153248189031732	0.982725338620588	0.100082333333334	0.713527803794788
HIST1H2BM	0.196118675143552	0.481105804958761	0.0529379725266497	0.937009489777134	-0.091299999999998	0.719304894613107
SENP7	0.195996318680569	0.481502702291456	-0.0168875390889210	0.981160061237418	0.353411333333334	0.130834595594896
FAM175A	0.195976631684947	0.481502702291456	-0.282937641659590	0.502129505146473	0.217142	0.412758598920913
ZNF33A	0.195939735848437	0.481502702291456	-0.0216612512252900	0.97536722461972	0.0634650000000004	0.84207712482186
MAGEF1	0.195907823201325	0.481541620547529	0.437300289404691	0.194979332046875	0.0352463333333343	0.888285531317364
BAP1	0.195893376712487	0.481553369236008	-0.0608131894663212	0.925128522530622	-0.148871999999999	0.471680039257208
RCOR1	0.195875123501557	0.481587627077617	0.294931234026825	0.478695647537866	-0.00629166666666656	0.984682291807697
PDE3B	0.195745363560208	0.482133938334717	0.0631842052581848	0.921359867071909	0.218380333333334	0.361284662118081
AAMP	0.195720622120828	0.482206606942403	0.134317906399248	0.808009477165608	-0.0285826666666656	0.915761046438965
MAMDC2	0.195636926118167	0.482554419438853	-0.301047633597143	0.464782507068736	-0.178534666666666	0.359908305595919
CPT1C	0.195610434808731	0.482637486035589	-0.082613849919163	0.893753725133293	0.0582443333333339	0.864974134126057
STK4	0.195493534763248	0.483034603971085	-0.0876881585681557	0.886302037299497	-0.135565333333333	0.541939010010167
PLA2R1	0.195425967074733	0.483123891693579	0.0610098047396264	0.924825206377811	0.110571333333333	0.631408228698652
C10orf11	0.195425378854199	0.483123891693579	0.521137916921317	0.0887204144095562	0.086552666666667	0.692043732062832
MFF	0.195416204521581	0.483123891693579	0.118422752401472	0.837937752076601	-0.442616999999999	0.0474020780670919
RFX5	0.195276615948777	0.483673682779917	0.0495383415711135	0.94156962266198	-0.268005999999999	0.165578686104596
NUP107	0.195209568204462	0.483831787395704	-0.124538924676157	0.824974271393593	0.0160170000000004	0.965918707629255
SNX33	0.195196493517982	0.483835535858326	0.277608252649989	0.513917729737286	-0.106285999999999	0.64090250408923
ORMDL3	0.195169324065209	0.483849099031502	0.162264931046870	0.758164869707887	-0.0735773333333318	0.735938662411184
KRT16	0.194976995379921	0.484546678015786	0.238300106521983	0.601773482805025	0.0222823333333343	0.949194996342177
VCPIP1	0.194913775811707	0.484774068049404	0.0443936350003133	0.948327980852394	-0.280067666666666	0.172629239571161
PRMT1	0.194810402675158	0.485120009157726	0.208389368628027	0.666722617010087	-0.135754666666665	0.580149967309238
APPBP2	0.194805716796442	0.485120009157726	0.198214485208290	0.687345015143924	-0.236634666666665	0.262334522628498
ABCA1	0.19479034977596	0.485137423991072	0.0989812685289107	0.868042453226125	-0.0831716666666658	0.731303939999377
GMEB2	0.194723828658511	0.485266929499885	0.161865130329352	0.758930446817487	-0.110431000000000	0.669530887411007
KIDINS220	0.194706780442522	0.485266929499885	0.266279856234243	0.538833471023229	-0.0274226666666657	0.933502474556197
ST3GAL3	0.194706378841994	0.485266929499885	0.198859173136432	0.68535210394429	0.225300666666668	0.243957990539057
PLAGL2	0.194615563738270	0.485658323618742	-0.278980123046353	0.511025440770733	0.055322000000001	0.813828958467063
CWH43	0.194482371119643	0.486293848768887	-0.187120359092428	0.709833349594307	-0.00335566666666671	0.993756838831997
ARHGEF15	0.194478675799221	0.486293848768887	0.0479792867672075	0.94314236584312	-0.134252999999999	0.678776599957621
KIAA0430	0.194269948490473	0.487047000839996	0.0088086186743798	0.98946753270812	-0.098367666666665	0.657217907037306
CSN2	0.194044822093625	0.488016590657955	0.316717236180334	0.432105820849792	-0.0236980000000000	0.934293360524785
ISYNA1	0.193993216080927	0.488087618570815	0.448574379797346	0.177860736739947	-0.0945186666666658	0.671893093390202
AFG3L2	0.193983202041930	0.488087618570815	0.0801711293791127	0.897577172229156	-0.0232553333333333	0.931305042071124
OXCT2	0.193950863387858	0.488206174074209	0.0130947234247391	0.984547546298896	-0.075273666666666	0.783223894269194
SLCO3A1	0.19387594012699	0.488446496818671	-0.159875838161163	0.763150272733332	0.062587	0.776378054800461
SEC23B	0.193838120764994	0.488581658297915	0.134415288613690	0.808009477165608	-0.0392449999999995	0.8754328609757

LOC100129726	0.193797532784825	0.488645815509843	0.0961120626393401	0.872262037328287	-0.155762999999999	0.673538419974363
COX1	0.193781644166698	0.488645815509843	-0.134219172009495	0.808009477165608	0.0559566666666682	0.821421133422134
ELFN1	0.193637300939004	0.489308080593936	0.124521232224406	0.824974271393593	-0.103678000000000	0.714244999058301
SPACA1	0.193629267360198	0.489308080593936	0.0181518933946665	0.979327298872732	-0.0634536666666666	0.774210549173745
TRHDE	0.193614087525217	0.489324577167932	-0.295189663441912	0.478475354656325	-0.0286596666666667	0.91402300740264
TTC30B	0.193568719708982	0.489425837866597	0.173227469715819	0.736740209571778	0.511700333333334	0.031687055072673
HS3ST3A1	0.193461200914461	0.489741870944763	0.201239268997920	0.680054545720343	-0.176508666666666	0.619996256183009
TSHZ1	0.193457212106275	0.489741870944763	-0.356812247914859	0.345138088652208	-0.602974999999999	0.0183513693848513
CIRBP	0.193437188222415	0.489787288715535	0.302515256985842	0.462229407853213	0.00366999999999976	0.992215039552133
NSUN7	0.193378105449473	0.48999161664851	0.296129683533501	0.477051680229433	-0.0232586666666662	0.93721833131196
NISCH	0.193350845638060	0.490006268701849	0.141946066857084	0.794657772812593	-0.0150066666666660	0.963626568322179
VPS72	0.193318530006674	0.49003691774007	-0.073509680838221	0.905838142287595	-0.0692869999999981	0.754015160951226
MUC6	0.193308510494115	0.49003691774007	-0.00709623073053288	0.991086607331007	-0.0765069999999998	0.799532708684155
CBR1	0.193218594706758	0.490259352870944	0.429342458358875	0.209103669076588	0.049001666666667	0.830320848383177
C11orf16	0.193209228572356	0.490259352870944	0.137659546401141	0.801934376886443	-0.0122669999999992	0.973759319973741
KAT5	0.193115973621016	0.49054375891092	-0.0953708292672	0.872881588028418	0.0612800000000005	0.786579063266167
VPS26A	0.193072925543436	0.49054375891092	-0.109879456919976	0.851013616427418	0.0848293333333351	0.68988329099201
CHAF1B	0.193064401890389	0.49054375891092	0.360125736331466	0.34143422019372	0.0854030000000017	0.710449166505851
RAB3IP	0.193050046425991	0.49054375891092	0.207415636974599	0.668625825887966	0.125628666666667	0.582111650674021
SOCS5	0.192809032577278	0.49140044772686	0.0902732921195427	0.882163033437356	0.0395310000000001	0.878111320125249
CNTN2	0.192806552302954	0.49140044772686	0.158963950800193	0.765499183785873	-0.0493940000000003	0.855240892932328
SCAF11	0.192794998481197	0.49140044772686	0.0125908899447448	0.985220352735461	-0.177642333333332	0.400064357572091
CD276	0.192651706341476	0.491988728439896	-0.00735406693746501	0.990873555460625	0.195102000000000	0.310717409362183
GPX7	0.192600301357709	0.49204553667483	-0.0761637961210778	0.903296922694955	-0.085725666666666	0.69414649287771
IRF1	0.192525816404166	0.492268698139862	-0.552673887116274	0.0593132549115835	0.000365000000000100	0.99868162314374
SPATA3	0.192499731505249	0.492350608008612	0.164430172224302	0.754189116283047	-0.195623999999999	0.524845938106282
ACBD3	0.192455040867604	0.492543655982236	-0.175969281808149	0.731162861882915	-0.0118246666666658	0.967460065937822
UBP1	0.192438671983789	0.49256091848193	-0.0430053789464268	0.950146638225747	0.113723333333334	0.639002829892966
OR2K2	0.192367089914295	0.492847436884753	-0.082609137081365	0.893753725133293	-0.00284499999999995	0.993756838831997
LOC100128288	0.192317355835025	0.492884897985742	0.260162029998304	0.554683096193634	0.203354333333334	0.353471671280201
GDAP1L1	0.192311364299303	0.492884897985742	0.0841864016002478	0.892396784625427	-0.100385333333333	0.818853338419942
BAZ2B	0.192273631445609	0.493036489545704	0.302133663207114	0.462229407853213	0.197039000000000	0.32631478390653
VAT1L	0.192254163024230	0.493078961168481	0.196568404253595	0.690838714578657	0.381115666666667	0.324443655582244
KATNA1	0.192199152507372	0.493242227168817	0.0436861011174437	0.949043721172002	0.137671666666668	0.483245973056047
ATP10D	0.192164512000299	0.49331929865733	0.240788909155618	0.596773408433565	0.0811289999999997	0.714382809933119
UBR5	0.192058217982924	0.493733119384798	0.308939116674366	0.44937281099696	0.0365713333333338	0.88751492208835
PLK2	0.192009176029597	0.493859573825982	-0.0355962406459012	0.958689318480951	0.0331836666666683	0.919456505549688
POLA2	0.191998873194282	0.493859573825982	0.0692153971336145	0.911837389834658	-0.0741669999999987	0.731974410873657
PNMA1	0.191992379310148	0.493859573825982	0.0776759807828133	0.900452756580846	0.0482193333333341	0.850409337098466
ZCCHC13	0.191984864772080	0.493859573825982	0.086527026395104	0.887493600482322	0.0141946666666665	0.963224271516007
DCLK1	0.191950605867067	0.493859573825982	-0.0622422282724218	0.92301259447339	0.643305	0.389396795598359
NT5M	0.191857073902773	0.494027353567239	0.35258499787219	0.352560848501456	0.109351333333334	0.63979843704752
GREB1	0.191852491822908	0.494027353567239	0.232358451680203	0.612658112747496	-0.354741666666665	0.500226891090192
PLXNC1	0.191848726784413	0.494027353567239	-0.258911344145738	0.558709815549469	0.568228333333334	0.0273713913650908
C12orf61	0.191848476085163	0.494027353567239	0.0336776053955392	0.96061727828906	-0.0947313333333324	0.746473454296225
ZNF484	0.19177873512427	0.494222991771503	0.395146764736540	0.272692742937895	0.0806096666666668	0.748579513438275
LOC284757	0.191730633432975	0.494335932863974	-0.048789299579896	0.942180113539637	0.0334276666666672	0.921128981205696
C16orf13	0.191689642660714	0.494386904812511	0.578688446460289	0.0427567474426921	-0.0596126666666656	0.791240721091965
FBXL5	0.191670765820792	0.494426035137083	-0.110476680052848	0.849626303351656	0.230714333333334	0.217583016023457
HIST1H1B	0.191591436523302	0.494826743650959	0.08044947038569	0.896952038996626	0.127506666666668	0.62979571408014
DUOX1	0.191487464229848	0.495300276509354	0.266742151880243	0.538390048324742	-0.0561349999999995	0.809505390888975
SMYD3	0.191389317795809	0.49541630442645	0.0403092292811594	0.952720585102517	-0.288502333333332	0.131549958663763
DIRAS1	0.191389071718292	0.49541630442645	0.0640754501548156	0.920005468161721	0.00831033333333491	0.982375192575954
SLC1A3	0.191199257117903	0.495982294494015	0.304664757699062	0.459197740121543	-0.00355799999999999	0.991175862027014
ZFHX2	0.191176495456376	0.495982294494015	-0.289664674757549	0.488870852256136	-0.0275166666666660	0.928988192507851
KRT79	0.19106566078927	0.496357866358074	0.0308290809990339	0.96529904314994	-0.0809266666666664	0.834476346642783
PCDHB14	0.191052197672225	0.496357866358074	0.0288739979191764	0.96782691865619	0.197546000000001	0.2984053803031
SPAG8	0.191029842623139	0.496418006370664	0.07050656482718	0.910169586639542	0.0526390000000005	0.853353908846638
RPL36	0.190969472590254	0.496705908366597	0.262464704030964	0.548787322027919	0.0219636666666672	0.93901998985171
SNRK	0.190927132647714	0.496706730064261	-0.547874786277394	0.0620641062369145	0.140343000000002	0.476790385924269
SIRPA	0.190907776256251	0.496706730064261	0.130218379425919	0.812271254601992	0.046060666666667	0.88629962370345
CSMD2	0.190695880049343	0.497755630286159	-0.0107410847163993	0.98774587825743	0.0122236666666672	0.974451420920923
RPL3L	0.190643961483429	0.497919385973029	0.0484946200658015	0.94253651106066	-0.0902239999999995	0.758250508013307
CLOCK	0.190582089731669	0.498062242335872	0.0190648369300247	0.97832242527852	-0.0860526666666657	0.708342792016055
CRP	0.190496809488392	0.498211569846507	-0.117432055003036	0.839825318851589	-0.0697576666666664	0.748388402768556
KRTAP7-1	0.190445446359949	0.498260466091655	0.0247470986816086	0.973137512042764	0.0596886666666668	0.791595191224138
CPA4	0.190427160987680	0.498260466091655	0.0520154908236519	0.938067483409541	-0.179639666666666	0.453167962703614
ANGPTL2	0.190402108409383	0.498336997719278	0.150885645523461	0.778253782413218	-0.200779333333332	0.482760288609588
CD2BP2	0.190365196551982	0.498484699647565	0.21690402286371	0.647212711808862	-0.278299666666666	0.180114381586694
EFHD1	0.190340096701677	0.498561538227417	0.079743119157018	0.897892885174022	-0.0437493333333324	0.882517847404652
ACTL7A	0.190302223803093	0.498617863020497	0.303705131301215	0.460279516720084	-0.0808213333333329	0.78061644993475
HSD17B12	0.190295594333431	0.498617863020497	0.361160258199752	0.339627732901974	0.173381333333336	0.373312492166648
IPO5	0.190274887370979	0.498617863020497	-0.189639145162818	0.704928430617595	-0.0657230000000002	0.771373465365664
EIF2S3	0.190248486533913	0.498617863020497	-0.333342090033331	0.396894500849691	-0.152343333333332	0.45895048781174
MYO1C	0.1902294774085	0.498617863020497	-0.0686651751123533	0.912149839732916	0.173043666666668	0.374843323203395
OIP5	0.190218829200477	0.498617863020497	0.0496573702476894	0.941500056942293	0.067068333333334	0.788201431210286
SUSD5	0.190041441096400	0.499247795572908	-0.0774399271411978	0.900797933355475	-0.0549689999999992	0.8694087001794
STRN	0.189996954752210	0.499349928154591	0.167326906631454	0.749084102223547	0.272042333333333	0.187638103900419
HIST1H3F	0.189923948655652	0.499512797993475	-0.124829440107522	0.824472060590171	0.137361333333335	0.548643967646122
DIXDC1	0.189828715819274	0.49978792464102	-0.132787495802501	0.809588765729788	-0.00856466666666642	0.981191596773742

UTS2	0.189684085889905	0.500288124422093	-0.248477398138365	0.57746120283043	0.0911780000000002	0.690641668678273
PLEKHM3	0.189469795128576	0.501059950524392	0.212710272538112	0.656111996220908	-0.132969166666666	0.55753236093702
LINC00176	0.189373680528312	0.50143165996929	0.311349536395125	0.443010534230695	-0.152883333333333	0.610847413222267
ZNF552	0.189340794519344	0.501540721007545	0.131406179527578	0.811227868886268	0.0943581666666669	0.811816884537132
GABPB1	0.189282511165202	0.501766272726072	0.0577304320480069	0.929758936765496	0.199836333333335	0.398349038294794
COL4A4	0.189249734696526	0.501793455100577	0.0994186232112884	0.867651972215247	-0.528267	0.0326806169766676
LRRC27	0.189231403336578	0.501829981538222	0.0056150233827931	0.99251028338798	-0.123430333333332	0.549323798856043
PRPF31	0.18919370714432	0.501909271671179	-0.0616698990295784	0.923246185283376	-0.0661763333333316	0.787248874966057
KAT6A	0.189179963651169	0.501918212635578	0.085840214207487	0.88869811752796	-0.0778186666666663	0.723861793428072
CKAP2L	0.189053911934116	0.502342490057537	0.149031630505507	0.782947197211795	0.108302000000001	0.656324975591247
H2AFZ	0.189011648103215	0.502342490057537	-0.0413564612143772	0.952098348256047	0.204780000000000	0.313220411537404
OTUB1	0.188994989220199	0.502367709966593	0.284190475929883	0.500557068353025	-0.0435646666666658	0.85576203728561
RANBP3L	0.188971139284067	0.502437531949645	0.135314933160697	0.807470535721699	0.207304666666667	0.73097308185803
IFT88	0.188912287767615	0.50264432748132	-0.238103752335533	0.601952506488164	0.303785000000001	0.139486617045524
NUP155	0.188886456117255	0.502726108265664	-0.00058504577393279	0.999231019369277	-0.0150999999999998	0.960071163568134
FAM195B	0.188843906908330	0.502784749457456	0.108529865836391	0.85395367181085	-0.0693509999999991	0.76648935876098
GNL3	0.188839981217015	0.502784749457456	-0.0528859295522036	0.937009489777134	-0.253718999999999	0.190961185202689
COX15	0.188817036225960	0.502847952270357	0.128877988061479	0.815391045978843	-0.00672000000000044	0.9835653863402
CHD5	0.188804998361929	0.502847952270357	0.235186629407248	0.607340367904098	-0.165353	0.460428849906031
LHX6	0.188653637800982	0.503464586971793	0.335865578052795	0.38984602770636	-0.225065333333332	0.298978462850944
NEMF	0.188423819963115	0.504295286474228	0.0538507160589266	0.935404504538153	0.162237666666669	0.410917713955087
CSNK1G2	0.188377245341226	0.504295286474228	0.286813734015249	0.494604746861742	-0.214050666666666	0.351086080682962
OR2L2	0.188375268373441	0.504295286474228	0.264371159656123	0.544042395849675	-0.0501729999999997	0.827433678445379
TH	0.188313144719023	0.504484268304965	0.302209616974342	0.462229407853213	-0.199380000000000	0.50195900391388
CKAP4	0.188263501457204	0.504674062180860	0.16605953321883	0.751830402832405	-0.376841333333332	0.119723060761242
GCH1	0.188220351730078	0.504722984119448	0.0812649186676766	0.896346444707454	-0.0428409999999989	0.880254449461876
AGAP4	0.188193113350253	0.504765641740842	0.217999291526663	0.643858306038742	-0.115017333333332	0.587396808178803
RALYL	0.188135467971367	0.504965830712371	0.278379884550721	0.511415146817954	0.0102303333333338	0.982398683576954
SLC35B1	0.188066308675308	0.505099877503240	0.0864876558850921	0.88755074549681	0.201809666666668	0.315859935065171
NIPBL	0.188064355488756	0.505099877503240	0.177578746181966	0.728492631264289	0.0748126666666679	0.761257561656748
ATP9B	0.187927575961277	0.505703908822651	0.394284895548708	0.274555703975637	0.0659680000000001	0.766247450031517
TTC36	0.187720824831406	0.50658337977313	0.377911677981996	0.305766089617986	0.0398413333333342	0.872930585008323
RILPL1	0.187644278029177	0.506824386496485	0.396675609322121	0.270862229167822	-0.0146359999999999	0.967241125442187
CRK	0.18759341815697	0.507028439022694	-0.175419287373969	0.73213520578756	0.0750063333333347	0.743076926886124
RPS6KB1	0.187383762755874	0.507956302932282	0.0814613693006737	0.89615326639288	0.00976566666666857	0.974451420920923
ZNF791	0.187370880951815	0.507960318344999	0.112194337983243	0.847454473507881	0.0173903333333339	0.950884054684253
USP1	0.187196837099437	0.508702927018347	-0.26596556680197	0.539310262140605	-0.205730999999998	0.294710292981557
LRRC41	0.187081117685119	0.509196859871649	0.0524074201561049	0.937831566264837	0.0225426666666683	0.93235605813448
SCYL1	0.186981376187245	0.509643470225914	0.224326672181341	0.632829553438263	-0.0268133333333328	0.91894315004511
MORC4	0.186970768000097	0.509643470225914	0.141736176394548	0.795109963263224	-0.361760833333333	0.120308223419948
WDR83	0.186881202923663	0.509845514473723	0.208090091263545	0.66739374020401	-0.0843446666666661	0.718749100405876
SHC1	0.186815827951164	0.509845514473723	-0.229850133052645	0.619381301776937	-0.249515333333332	0.219950821176406
AKAP12	0.186779317930924	0.509845514473723	-0.0213073300467898	0.975562088587469	-0.108712666666666	0.605491766505367
LCN8	0.186776410038156	0.509845514473723	0.154278210809400	0.771685265099002	-0.254254333333333	0.365126455342703
C15orf48	0.186748627798445	0.509845514473723	-0.189806231294773	0.704724567131716	0.0546493333333336	0.832903171531715
TRIM17	0.186736715367225	0.509845514473723	0.0624497616830573	0.92264797401958	-0.178868999999999	0.369150831955280
SLC41A1	0.186678565810424	0.510040952152954	0.352625810923029	0.352560848501456	0.0405353333333348	0.890094615232462
HOXC9	0.186645671061864	0.510040952152954	0.00674676364195209	0.99129507664977	0.122748000000001	0.554188413823318
LHFP	0.186632850120495	0.510040952152954	0.156765753129092	0.768487306347322	0.0133653333333346	0.96797990593595
DRP2	0.186624479174948	0.510040952152954	-0.191782173429003	0.701544026774214	0.0441360000000007	0.861165447055876
SERTAD2	0.186485925254890	0.5104373643077	-0.00506997240915401	0.99251028338798	-0.143666333333333	0.514889931507188
SLC4A1	0.186436770840671	0.510587563669006	0.239880233856308	0.598134271398487	-0.166430999999999	0.401558725985789
MEA1	0.186350959967355	0.510960040168297	0.212915955957294	0.655695431812946	0.166016666666667	0.391106728390508
TAS2R7	0.186327237597736	0.511029999601443	0.0625246990486054	0.922632631649811	0.00031000000000011	0.99868162314374
NAF1	0.186081069098472	0.51230208687415	0.0424567378796959	0.951446425515852	0.0435086666666673	0.87629402535202
SAA3P	0.185889905214002	0.512871106283484	0.0854288461590966	0.889732337553966	-0.0360499999999997	0.889343227773276
LPL	0.185714292568331	0.513336257292002	0.197611403176583	0.688668717091024	0.220499666666667	0.448208117903793
GABRR2	0.185688825135330	0.513336257292002	0.191771992603625	0.701544026774214	-0.0248216666666661	0.92484471634727
AP2M1	0.185570456509923	0.513557798456639	0.252596184644899	0.569357224558971	-0.0518933333333323	0.820422171682127
PSME4	0.185536346223617	0.513557798456639	0.250274743402922	0.574107101982055	0.253454666666669	0.263562775503683
PTH2R	0.185533403087130	0.513557798456639	0.543702728407954	0.0658108061761627	0.158857000000000	0.474455297935757
FERD3L	0.185493368148800	0.513579340052052	0.0971427110536528	0.870020870354202	-0.00741633333333237	0.986471172041113
SARDH	0.18543923411544	0.513834411360582	0.223862744644266	0.633743682471632	-0.101322000000000	0.634966811516658
GNGT2	0.185390079712495	0.514059281251269	-0.0613625631509609	0.924178139205947	0.024011666666667	0.936899235817306
MARK4	0.185206076761592	0.514791669555662	0.263565010923494	0.545610060822567	0.091614000000001	0.684937448608093
ZNF780A	0.185196689720385	0.514791669555662	-0.114072926410656	0.844272085764738	0.198419333333335	0.313220411537404
THOC5	0.185106683958629	0.515228246641464	-0.0775565961245793	0.900701176765499	-0.169469999999999	0.422927358730594
EIF3G	0.185049369282670	0.515318223282424	0.301189826880582	0.464501110297982	0.00805066666666748	0.981191596773742
WNT10B	0.184934497801268	0.515647516205703	0.143864518476865	0.791853933422817	-0.126896666666667	0.578893615511221
COL6A1	0.184892369379254	0.515830032446595	0.194621896555878	0.69591561649809	0.118824666666667	0.624595423091977
FBXO34	0.184857930628638	0.51596574891765	-0.118969592951596	0.836828045978697	0.0365036666666668	0.898053037530613
EFEMP2	0.184775120703433	0.516384669654958	-0.144785235841893	0.789598951980813	-0.115098000000000	0.66866470442489
GAST	0.184734568914837	0.516495171983006	0.0866340506194282	0.887493600482322	0.140453333333335	0.6846855637534
MTA1	0.184701311985294	0.516623784328558	0.0616376886808998	0.923246185283376	0.0902070000000006	0.704370299831112
SYCP2	0.184660451870368	0.516650620374677	-0.335121132173425	0.392042077410332	0.170746333333334	0.585260484981764
ZNF830	0.184610339336991	0.516793597097072	0.0476887846021242	0.943560556552289	0.0280149999999995	0.929907694303715
ENGASE	0.184594972963043	0.516793597097072	0.0134342641865043	0.98412883278237	-0.186617666666666	0.336373925530627
MAMSTR	0.184588397086585	0.516793597097072	0.384986024275793	0.291180853572672	0.0585500000000002	0.798170083215805
UNC45B	0.184561027037722	0.516862460766073	-0.0547464146488419	0.934399317980705	-0.0659823333333326	0.82811815913847
MYOD1	0.184538147135312	0.516862460766073	0.139325291141528	0.798615265561297	-0.156332000000000	0.68135180147671

MOBP	0.184528519058361	0.516862460766073	0.0261144751828714	0.970632644458507	0.0669803333333334	0.770112696597648
SYNJ2	0.184359287616062	0.517598072843129	0.152740454946321	0.775138534286213	-0.193302333333333	0.399873592558586
ENOX1	0.184173936801493	0.518210362399209	0.325832306381871	0.409206882509236	0.0523793333333334	0.87152108397136
MBD6	0.184148869205577	0.518289261262682	0.152853722063693	0.774725957219946	-0.0729409999999995	0.757446522571541
ULK2	0.184124532648441	0.51833968715537	-0.0283066509369118	0.96843394074672	0.458888666666668	0.0353807205776334
TUBA4B	0.18409212988145	0.51833968715537	-0.110894028396195	0.849357727620213	-0.0051646666666663	0.987937064305561
RING1	0.183998089642994	0.518617011127265	0.0132822450891822	0.984234840490814	0.239730000000001	0.243957990539057
TMEM119	0.18374413904885	0.519945839055722	0.0572815326922556	0.93071299016553	-0.195938666666667	0.43441407814235
NRIP2	0.183691047364332	0.520122032823158	-0.0247824043781874	0.973095304248227	-0.0635929999999992	0.852953504248302
PIK3R2	0.183674479174042	0.52013625048762	0.201042709182688	0.680143077995217	0.0325180000000005	0.904507944803624
PTRF	0.183660939124369	0.52013625048762	-0.199251054371196	0.684227048797313	-0.186170333333333	0.550446282371936
DIS3L2	0.183652320903490	0.52013625048762	0.257312413358684	0.563116080558022	0.0649923333333336	0.787739636244196
PODXL	0.183598995629007	0.520388118310287	-0.212154458354200	0.657918861205747	-0.106661999999999	0.692828037753004
KIAA2018	0.183502117754157	0.520906515502082	-0.18597942474728	0.713284034966074	-0.149638333333333	0.549323798856043
ZAR1	0.183475106867905	0.520997549325001	0.263870153604136	0.544971884819175	-0.0851346666666658	0.737381684730589
SLC10A1	0.183460511202575	0.521012614586606	0.0543498645170383	0.934486928733702	-0.0825953333333332	0.695654544311453
FBXO9	0.183421567781822	0.521176389314667	0.195385490456426	0.694303704398562	0.0521233333333341	0.817735562413299
TOR1AIP1	0.183406560963529	0.521176389314667	-0.141514160755139	0.795705351419283	0.0681626666666673	0.760010763598572
ILF2	0.183397353444077	0.521176389314667	-0.0144521540689046	0.983444353656142	-0.0267899999999994	0.919349518409758
LOC100128262	0.183360315742326	0.521328833176149	0.0671687931391692	0.914700759157169	-0.0664499999999997	0.790420447081709
SPAG6	0.18331238974802	0.521547963379162	-0.349600715028213	0.359178920097907	0.196265333333334	0.348487071607332
SMOC2	0.183281865187805	0.521660585631766	-0.264124140774972	0.544525995334757	-0.0356899999999998	0.911796922065088
PRMT2	0.183207649589925	0.52189222542416	-0.0269410647763873	0.970207612777188	0.104817333333334	0.656222957956058
DPYSL4	0.183177161093627	0.522004673260376	0.354141676676065	0.351136684029519	-0.127908666666667	0.521702512887465
CHRM4	0.183128136181741	0.522156394207648	0.299775476850411	0.467332983744709	-0.0290253333333324	0.924469572710745
EGFL6	0.183091833330109	0.52230449881985	-0.160801951672445	0.761016253411733	0.0064860000000003	0.985263856374185
MAGEA11	0.183065490324612	0.522391600212859	0.063539711253765	0.920855211745173	0.113040000000000	0.66758573421638
LOC202181	0.182998272028839	0.52272921580937	0.0170975060326436	0.98105020869911	-0.0012039999999996	0.996945419390193
C1orf86	0.182947632130958	0.522965308870481	0.0471210417322442	0.944081286115546	-0.140968833333333	0.549323798856043
ZNF284	0.182912872503577	0.523073097213132	0.290924287844084	0.486880079690803	0.386231666666667	0.0928866723506115
GPR12	0.182905797940926	0.523073097213132	0.0801658769404191	0.897577172229156	0.00680033333333332	0.984211006514154
CCDC77	0.182735280609467	0.523970392518088	0.0602209734494317	0.925649366494154	-0.050439333333332	0.870662337224757
VPS13D	0.182712144953419	0.524037950224375	-0.518922217899563	0.0903775728003269	-0.115652999999999	0.673514912541661
TSPAN5	0.182680505322949	0.524157721875038	0.0210873026675823	0.97607156937294	-0.0050473333333321	0.987937064305561
HOXD13	0.182648451659474	0.524205590574305	0.64508065782116	0.0167517957225989	-0.0096093333333323	0.98025379113353
SETD7	0.182590761560870	0.524410881411505	-0.0377869269590896	0.955190852940141	-0.0879006666666654	0.679958892307394
SNRNP40	0.182563866590687	0.524446143314271	-0.234492872022126	0.608446568003374	-0.131185999999999	0.523041310312964
FIBCD1	0.182560768324541	0.524446143314271	0.272597674235481	0.526081777027135	-0.103853333333332	0.707459630816261
WDR5	0.182535313591929	0.52447120080529	-0.0167477242903063	0.981295472004255	-0.0847589999999998	0.688145991467449
SLC25A25	0.182520758912948	0.52447120080529	-0.259678018716931	0.55626686147478	-0.165434666666666	0.523453039510001
RBM4B	0.182444767913604	0.524750915097822	0.0535893911715681	0.935847524102816	0.00779566666666649	0.982462715783175
PDS5A	0.182260315899142	0.525155433180681	-0.297529266781952	0.473742322122506	0.0842980000000011	0.718749100405876
HOXB9	0.182251389495138	0.525155433180681	0.0163619365827493	0.981892085795004	0.0689766666666672	0.817735562413299
TAOK2	0.182225825796021	0.525238063109405	0.2505191662124	0.573763059271745	-0.167821333333333	0.484583000340636
TEAD2	0.182085384910610	0.525834989854053	-0.0943265254348883	0.874531031957077	-0.135946666666666	0.564757468001238
ARHGAP4	0.182065041732502	0.525834989854053	-0.257280345198312	0.563116080558022	0.0678060000000011	0.791240721091965
CCBL1	0.18205562478622	0.525834989854053	-0.261984884532391	0.5499549817547	0.0801206666666677	0.757015566207214
OPRL1	0.182055044483364	0.525834989854053	0.267740404181756	0.53559545997875	0.0504339999999999	0.843134844119548
ODF2	0.182043907994273	0.525834989854053	-0.0351045578656067	0.95895015491824	0.0387216666666678	0.8754328609757
HNRNPA0	0.182019094595175	0.525913083164943	0.185193566247326	0.715110052196475	-0.225065333333332	0.303929656050658
CD83	0.181965934499981	0.526016631042154	0.292510855589525	0.483519471626455	0.373617666666667	0.113589886597990
KIAA1841	0.181863071640304	0.526276981986147	-0.114105942595396	0.844272085764738	0.256548333333334	0.173667234667710
MLF2	0.181595522670549	0.527529243337058	0.104407514371538	0.861121906453898	-0.016767333333333	0.952455466071018
LOC441179	0.181578852171934	0.527529243337058	-0.326561432252344	0.409206882509236	0.00662366666666684	0.984334402946184
PRPF18	0.181533445326300	0.527560228342234	0.00794795538301163	0.990405674923721	-0.101068666666666	0.730924200776612
UBE2O	0.18149180315669	0.527613362142924	-0.0358826996616501	0.958354725588542	0.0191226666666677	0.952100146370651
GAPVD1	0.181488530138824	0.527613362142924	0.00057837775849383	0.999231019369277	-0.457913666666665	0.0326806169766676
SLC7A5	0.181220314861458	0.52850274846159	-0.199036434584252	0.685138880840339	-0.0641219999999992	0.774448337721913
CROCCP3	0.181217552538521	0.52850274846159	0.273342476785759	0.523887082296009	0.0548730000000007	0.835752276645858
CNIH2	0.181186969684125	0.52850274846159	0.191695318679937	0.701544026774214	-0.168032666666666	0.508602469366771
KANSL1	0.181181063043055	0.52850274846159	0.170902166913627	0.740768646795758	-0.0576943333333325	0.798344353938564
NUCB1	0.181171859973516	0.52850274846159	-0.29093257837754	0.486880079690803	0.0371906666666679	0.890094615232462
RBBP9	0.181151190642696	0.52850274846159	0.162189280176173	0.758353674758641	-0.0130646666666658	0.975538996157203
KANSL2	0.181138578555505	0.52850274846159	0.0415242993300683	0.952098348256047	-0.0243609999999993	0.935261323444314
TIMM17A	0.181019611987814	0.528980446020242	0.104195802253164	0.86124543980304	-0.123332999999999	0.533597777826448
SART1	0.180963585855125	0.529059740063638	0.0335299153195926	0.960782399177614	-0.234655666666666	0.343572153228906
ZC3H15	0.180914114272661	0.529215715986132	0.0191742742749461	0.97832242527852	-0.0429176666666668	0.870363204357393
TPPP	0.180696957710715	0.529832451881243	0.212148820338698	0.657918861205747	-0.0345813333333327	0.936557228489982
SALL4	0.180676821877740	0.529841157089316	-0.311837311283027	0.441314096241515	-0.0798889999999999	0.710083364777824
ZBTB3	0.180660130131566	0.529841157089316	0.00665906290004118	0.991309497300929	-0.101908666666666	0.618241861684285
PPP1R8	0.180637235809020	0.529844605820954	-0.195828713871824	0.692857583007357	-0.0197056666666664	0.94678260229888
NUP210	0.180543521043644	0.530186306866615	0.132794776616055	0.809588765729788	-0.122857999999999	0.661447114784096
ESCO1	0.180541944963687	0.530186306866615	-0.0104421797374677	0.987849393233007	0.158001666666668	0.512563877338275
TGFB3	0.180527334128686	0.530186306866615	0.249567887195677	0.575370998761354	0.665557666666668	0.0725137013180337
ATP8B2	0.180491111874836	0.530212723284643	-0.184741423253834	0.716042213808415	0.170747	0.46297633209186
RPS23	0.180441112205363	0.530212723284643	0.125861121481300	0.822613266045532	-0.220934666666666	0.254795675806330
MAPKBP1	0.180425323686739	0.530219114477558	-0.115124002873502	0.84409286304878	-0.197680666666666	0.307304414570957
POLH	0.180256530460967	0.530503553968386	0.225595937034608	0.629710977741725	0.141289000000000	0.539366851549484
LCOR	0.180251653090873	0.530503553968386	0.0147937640602970	0.98327723682819	0.0929086666666681	0.686516408582019
NUMB	0.180216867704487	0.530540509097309	0.164163455171952	0.754189116283047	-0.153227666666666	0.481986611994676

ZNF654	0.180173008347984	0.530736541917423	-0.174005543005046	0.734793595952544	0.0831270000000005	0.756760013435331
FAM189B	0.18011117020147	0.530861883293047	0.179475064175729	0.724770967840177	-0.135267999999999	0.552476486760774
RNF19A	0.180017854661006	0.53111470620826	0.00867291384739183	0.98946753270812	0.156459666666667	0.493924482756516
ANGPTL1	0.180010336360769	0.53111470620826	-0.075513969855328	0.903537954146139	0.395096333333333	0.46297633209186
sept-12	0.179669687478271	0.532639815649576	0.00198050643294491	0.997312205311242	-0.00648333333333341	0.988133186272453
DNAJC24	0.179668280763763	0.532639815649576	0.0657358568281975	0.917495226662096	-0.0902583333333336	0.704871474914214
LPA	0.179655515608850	0.532639815649576	0.456029444999353	0.167648587973919	0.0704106666666673	0.78036180126224
OR52A1	0.17962380341513	0.532639815649576	-0.0843618229913406	0.892381223757468	0.0624726666666672	0.789377975672973
BTBD6	0.179595137332621	0.532667653170197	-0.163539894426036	0.755130122263395	-0.124538333333332	0.581502485736275
MFAP3	0.179397728281235	0.53351512426611	-0.0834799902252657	0.892833228561937	0.0685540000000006	0.811816884537132
FEV	0.179292844458546	0.533985936406026	-0.07255398587231	0.907556175729082	-0.0409026666666657	0.921623871569288
MED6	0.179273413955031	0.533985936406026	-0.102950407214850	0.86257932725575	-0.241460999999999	0.313340135008504
IAPP	0.179170412407132	0.534491378815025	-0.190238075476842	0.704208582824765	-0.0290583333333327	0.918192039123086
TMEM14A	0.179122692816315	0.534587444941604	0.309807598661185	0.4470577615309	0.137526000000000	0.512869954128574
RPS19	0.179100971192738	0.534605873512927	0.368074455764381	0.325826074795982	-0.0834239999999992	0.701388877286178
RPL14	0.179081830574311	0.534649892930663	0.314021643701788	0.438046311563112	-0.0734498333333323	0.761837433189273
FAM127B	0.178979330000015	0.534964598434281	-0.305388610968087	0.458013550008763	0.0629850000000009	0.780904457213926
UBXN1	0.178946442532572	0.534964598434281	0.183345573107542	0.718067440349544	-0.0510726666666667	0.824700707442131
TMEM177	0.178871766261532	0.535154478377253	0.275244709046154	0.519655146864016	0.0924016666666674	0.696194439936211
DLG2	0.178837219107732	0.535270851838471	-0.170988385765470	0.740570923133528	0.786836	0.0385547594472152
MSX1	0.178763886028079	0.535575823306511	0.365908785812891	0.32927628703889	-0.121176999999999	0.5531802635914
DHX8	0.178699769559351	0.5358981737737	-0.234491205039309	0.608446568003374	-0.00745866666666647	0.983159241812861
TSR1	0.178636661206594	0.536168930743189	-0.161732639314204	0.758946930122467	-0.203611999999999	0.287956132210494
TRIM40	0.178592131331601	0.536254941469553	0.220105195531779	0.64064333199733	-0.0318038888888876	0.914929120258681
USP7	0.178582356943260	0.536254941469553	-0.0646360643605212	0.919198375420231	-0.0951073333333323	0.641178599075931
PCBP4	0.178513878183448	0.536310392396584	0.233672009397437	0.610436189314218	0.226604666666668	0.25809881761054
C21orf128	0.178507810845997	0.536310392396584	-0.00153686289262688	0.998205782293583	-0.168611333333332	0.535487400963825
INE1	0.178497896088997	0.536310392396584	0.0785096836337607	0.899281198071059	-0.0646266666666657	0.803131392712617
VEGFB	0.178476297354222	0.536310392396584	0.0493507765521117	0.941842562680095	0.159800666666668	0.443378989641463
GSC	0.178475263389138	0.536310392396584	0.235653096271285	0.6067637636079	-0.0289896666666667	0.93368986054197
TGOLN2	0.178432275629346	0.536439184862519	-0.273224700274409	0.524050233203661	-0.133593999999999	0.535171605667144
USP6NL	0.17833141180422	0.536876416100451	0.229451360127406	0.620183562399927	-0.0479049999999997	0.894919379351752
TBX5	0.178326081592002	0.536876416100451	0.148350679060586	0.78389646294903	-0.0221483333333328	0.935529447810053
WNT8A	0.178291438544583	0.536970033391514	0.109414158869589	0.851975798726468	-0.0418283333333325	0.861945621270498
KRI1	0.178236795518285	0.537112236273957	0.242875029368519	0.592178030679976	-0.251432	0.313260450520079
SLC35F5	0.178156104019724	0.537406241783363	0.157974037940671	0.767373487136803	0.102558000000001	0.639002829892966
CCDC54	0.178076135067760	0.53760444615845	0.205897106521052	0.672561084372914	-0.103128666666666	0.669225752878437
MMP19	0.178009821118688	0.537883827145728	-0.177418287480204	0.728541130314952	-0.154162666666666	0.514016836574791
NBAS	0.177969776911747	0.537891957199365	0.415356565242484	0.236224795296402	0.117899666666667	0.641709143980398
ZNF768	0.17794307142045	0.537908903184623	0.273010162301858	0.524713596820663	-0.0559699999999991	0.823910138969963
XRCC1	0.177879308628492	0.538106190163949	-0.0176773077447000	0.980278729770454	0.00208866666666717	0.994819507544822
CXorf48	0.177875272923031	0.538106190163949	-0.200453154207008	0.680830337398347	0.158550333333334	0.633166039956913
PHF13	0.177828608009969	0.538246830544444	-0.158494938848953	0.766181314951192	0.0800983333333337	0.72457851707683
VRK2	0.177750410934738	0.538508652434944	-0.135431095914329	0.80704358190743	0.0386370000000008	0.888476605628154
ATP9A	0.177720806263097	0.538617875478383	0.105615942338998	0.85956218633532	-0.259875666666667	0.315859935065171
MTMR7	0.177701443203125	0.538663640505567	-0.184788645999589	0.716020884288485	-0.0882393333333328	0.705312045318091
LPHN3	0.177510035286591	0.539627629889059	0.204889144326131	0.673728362275223	0.558413000000001	0.0601168126740641
KLHL34	0.177433663699045	0.539928832141098	0.0260576936466237	0.970728888293842	-0.160093333333333	0.667293862238229
MYBBP1A	0.177373309295882	0.540178700526722	0.0353126981738219	0.95869292666698	-0.311950999999999	0.131549958663763
HIF1A	0.177355380699743	0.540215625046121	0.235454151360055	0.60678764664537	0.151266666666668	0.4423818632121
FOXL1	0.177316237499956	0.540309887949769	0.271017020745031	0.528536444071912	-0.143746333333333	0.590147690656472
MYH3	0.177281509025384	0.5404511196287	0.139439615965297	0.798450177373409	-0.029913333333333	0.908373940759161
SNED1	0.177263604016687	0.540487920351612	0.444230936516651	0.183294194447278	0.133970000000001	0.516635548538775
TOX	0.177233683472841	0.540552270450162	0.0332564236993228	0.960784250392482	0.984387	0.0219597843188960
LOC100130557	0.177127258441883	0.540888379319458	0.0628107291214273	0.922304271744625	0.113420666666667	0.60814016771842
GPR116	0.177093814470840	0.540947372276554	-0.038755618478868	0.954263089081373	0.29851	0.309421300309741
RASSF2	0.177059302247859	0.541013020882887	-0.169369889758011	0.744580501024877	-0.125894333333332	0.741121617241292
MXRA5	0.177019138500991	0.541043277613793	0.108445848493683	0.85410453602115	-0.0170606666666665	0.958293826181342
YTHDF3	0.177018526554279	0.541043277613793	-0.124904878013146	0.824457843812434	-0.182624666666666	0.421541794258528
NEK2	0.177006074456895	0.541046298771151	-0.0858274864173651	0.88869811752796	0.0683320000000009	0.814959196808566
RAB11FIP5	0.176950609894659	0.541167829228148	0.299411892763558	0.468245148036717	0.225845333333334	0.241273257416922
IMPAD1	0.176922601161672	0.54126746640631	0.388957766425846	0.284194872368231	0.0195915000000014	0.942680762825505
ARL6IP6	0.176891651128957	0.541305307692555	0.298469542476455	0.471239854755259	0.182153333333335	0.346020200924341
CDRT1	0.176887417093872	0.541305307692555	0.0879746708237956	0.885848000380851	0.375686416666667	0.0583806133794157
CTDSP1	0.176873299777958	0.541305307692555	0.0766485640334905	0.902228403382432	0.148738000000001	0.453167962703614
SH3RF1	0.176846009005937	0.541305307692555	0.0344067668204472	0.959404955370931	0.181105666666667	0.466209102755128
ZNF142	0.176831244328454	0.541314957991523	0.104222336321376	0.86124543980304	-0.104241333333332	0.736441343012057
SEC24D	0.176677710481797	0.542045732492862	-0.211058785405978	0.660493649750691	0.321177333333334	0.147433353861458
ZNF250	0.176640807634674	0.54205218851441	0.457602339048339	0.166671273245692	0.0244136666666675	0.949376459031393
ZNF563	0.176585812052920	0.542245345113508	-0.0594621645519638	0.92694538699714	-0.0133933333333323	0.966813448118178
ANKRD2	0.176423561553454	0.54280805500418	0.0437998586014324	0.948934431299106	-0.396461666666666	0.0909486750308436
CLCN3	0.176328544886146	0.543101630982182	0.0458252571560326	0.945372627844176	-0.024609333333333	0.927671110581394
GPX2	0.176273024757734	0.543372572633832	0.126317810826482	0.822011156288607	0.0779530000000004	0.785951697546817
LRTM2	0.176198415136955	0.54368801738441	0.165160787207421	0.752756950243373	-0.161096666666667	0.574486578500537
PTX3	0.176168159273445	0.543801919749328	-0.185283070587544	0.714946606559307	-0.0585093333333333	0.816134952712854
CCDC65	0.176097890097222	0.54401614645393	0.104946114494734	0.86067831050617	-0.105773666666666	0.697625171872868
NFKB1	0.176078014181243	0.544065497024012	-0.46383153331124	0.157277647666176	-0.0243626666666662	0.929594956567283
NKAIN3	0.176033963945390	0.544136104622159	0.187436352769834	0.709286896761914	0.0588336666666677	0.824700707442131
ERCC6L	0.176033062800568	0.544136104622159	-0.253630444162303	0.567705016149998	-0.0293663333333319	0.942680762825505
PRIMA1	0.17596616762281	0.544385633360112	0.156855173195332	0.768401006769299	-0.167417666666667	0.516467297561779

RRAS2	0.175961368337831	0.544385633360112	0.117859356606723	0.838686136084666	-0.0078749999999991	0.983159241812861
NFE2L3	0.175940433959555	0.544385633360112	-0.226813378943792	0.626060671348933	-0.155256666666666	0.663515191078113
TMEM9	0.175931038283281	0.544385633360112	0.223704047602038	0.63401557109874	0.0466536666666675	0.850157189407213
SZT2	0.175871506035930	0.544520527858095	-0.0437441597596417	0.949043721172002	-0.0258736666666656	0.92469922248949
LYZL1	0.175837750193119	0.544520527858095	-0.0420152327588273	0.951989245458556	0.193118666666667	0.375553176012692
AGAP2	0.175753754461983	0.544820489592269	0.252864762038606	0.568985221714455	0.00164500000000022	0.99628178244892
PLAC9	0.175736729896543	0.544835986480307	0.103537342837128	0.861943478503987	-0.273088333333333	0.263703023102649
CBX2	0.175727404788839	0.544835986480307	0.561263716260993	0.05252006717346	-0.149013999999999	0.544260745396866
SLC22A13	0.175641085910599	0.545150526323097	0.0751686021817636	0.904173044514532	-0.0799763333333329	0.739465662522697
HDGFL1	0.175573583685489	0.545389861832931	-0.146426085783570	0.786957117162244	-0.118730999999999	0.626806418302655
NDRG3	0.175566861970134	0.545389861832931	-0.377249767904749	0.307297021174057	0.226707333333335	0.238912992766543
INTU	0.175497624040783	0.545631125100736	-0.0529916529746096	0.936805758168909	0.109939333333334	0.706176049994687
DDX49	0.175494805788220	0.545631125100736	0.0315300595115676	0.963997329973776	-0.123304666666667	0.542096490709595
TAS2R5	0.175491165844791	0.545631125100736	0.16538273069029	0.752352242644389	0.0605870000000002	0.838900963549555
NEK11	0.175468205221462	0.545631125100736	0.166570471627497	0.750601948056403	0.236548000000001	0.399873592558586
FXN	0.175463309978992	0.545631125100736	0.133310428884487	0.808992949393043	0.0365216666666669	0.90232248061402
B3GALT1	0.175444651476177	0.545631125100736	0.0244465440853106	0.973562415846915	0.172847000000000	0.426518032099216
HACL1	0.175330303033158	0.546106949800054	-0.268589835772486	0.535014466593144	-0.026701666666665	0.922854901626297
DTL	0.175330117941340	0.546106949800054	0.0980091446965162	0.868852648210739	-0.316800666666665	0.124096550131011
CUL2	0.175094375253099	0.546839080131259	0.07482772390329	0.904466414650249	0.0857326666666691	0.701993852257083
PRM3	0.175029823190798	0.547101854781136	0.0301217704952176	0.966396286514145	-0.132501666666666	0.707538975864338
CELSR2	0.174990440440438	0.547198832935928	-0.390803945642219	0.282005640075858	-0.0629713333333324	0.800216081516012
GSX2	0.174912645240228	0.547398601488697	0.0387182559183247	0.954263089081373	0.0392149999999998	0.873398028647672
EDN3	0.174908889005558	0.547398601488697	0.105900324548574	0.85919141651157	-0.0666436666666653	0.7833393970818
SH2D6	0.174893406515392	0.547398601488697	-0.224470018675420	0.632710354180004	0.135366000000002	0.567355756887702
SLFN13	0.174874347656599	0.547398601488697	0.0106087214122703	0.98774587825743	0.202318666666668	0.390966211084762
AP3D1	0.17482182959946	0.547564809580266	-0.0571068952033869	0.930775493791569	0.0434883333333346	0.870002591217953
LDB1	0.174621428737241	0.548311936291283	-0.140644476378681	0.796714214272984	-0.110785333333333	0.590147690656472
PER1	0.174540430049837	0.548668781741091	-0.0739180201216468	0.905506084101768	0.0642616666666671	0.814959196808566
DOT1L	0.174491752905716	0.548824007709882	0.266225460716483	0.538910238472542	-0.195621000000000	0.349860712152174
TPH2	0.174401335715739	0.549091248563476	-0.0950875209523405	0.872962271153999	-0.0634586666666661	0.821421133422134
RAB31	0.174324978130249	0.54949368428356	-0.0181687240526979	0.979327298872732	0.0237896666666681	0.950884054684253
LOC100133315	0.174293951879569	0.54961312807494	0.120104380132893	0.835124910268666	-0.00246333333333245	0.99628178244892
MATR3	0.174273243570372	0.54966814159345	-0.0627385030492031	0.922350559812086	0.00687100000000104	0.983159241812861
CST1	0.174251355719845	0.549730528108994	0.260110053053133	0.554748923785473	0.510499666666667	0.0441412799487989
USP4	0.174215669912602	0.549879108018232	-0.126039543102499	0.822164329801713	-0.0441873333333322	0.870002591217953
MAP3K14	0.174113699661065	0.550434184555867	-0.0950707718827006	0.872962271153999	0.306451666666668	0.160856802716546
BCAT1	0.174076021177403	0.550528675137516	-0.0997439320960417	0.867558388686851	-0.157794000000000	0.469591696339734
SLC24A5	0.173692772895226	0.552281901761527	-0.0556123979525554	0.933409139126562	0.124496333333334	0.536810332635776
DOCK6	0.173688683190054	0.552281901761527	-0.100594467767035	0.866414040673375	-0.00498833333333238	0.987937064305561
CCDC102A	0.173609091620354	0.552631302298568	0.0725024490627033	0.907556175729082	0.0483510000000005	0.839998150752089
KCNJ2	0.173572260988373	0.55277471684713	0.164364572663239	0.754189116283047	-0.0477513333333327	0.883189085233712
CDT1	0.173419662943443	0.553222435032236	0.280034155137372	0.508074118826263	0.126180333333334	0.543123897584579
KIF14	0.173403434980554	0.55324880254017	0.04065774220293	0.952426531595115	-0.0538029999999988	0.852927816968262
BCL2A1	0.173363066232861	0.553427234506552	-0.272399798256809	0.526175116911121	0.0554070000000007	0.80841656016322
STAT1	0.173226954798332	0.553907612807686	-0.234212127007387	0.608813237170423	0.0236000000000005	0.93461792157888
TSSC1	0.173107630862714	0.554332767746228	0.13041134021773	0.812271254601992	0.009096666666668	0.976450310053666
GTF2F2	0.173055633102468	0.554332767746228	0.127696414605114	0.818611985817821	0.0323679999999998	0.899614658619676
ENPP2	0.17303406570572	0.554332767746228	0.30291632386773	0.462105017205825	0.0217803333333335	0.98147292539484
CACNG4	0.173006532895362	0.554332767746228	0.131001135796930	0.812271254601992	0.0872400000000005	0.789228734235839
KLRG2	0.172998589675901	0.554332767746228	0.166294351666533	0.751561339294618	-0.119921333333332	0.663931919724114
CNBP	0.172802431152009	0.555078904678241	0.140190968842847	0.797441782082887	-0.0314899999999988	0.903788355106891
IER5	0.172743878092920	0.555371739084904	-0.0109211581276391	0.98774587825743	-0.0855583333333325	0.697728706272367
BCL2L1	0.172524714411741	0.556225465140092	-0.0956711884222622	0.87262667899888	-0.0384989999999991	0.898676790082553
MEOX1	0.172484592444936	0.556235850599864	-0.0834531253065321	0.892833228561937	-0.144909333333333	0.472370507912389
SCN4B	0.172462113848078	0.556235850599864	-0.164907492086631	0.75320079407004	-0.182557999999999	0.61487566217891
ZNF8	0.172445152739510	0.556235850599864	-0.232267322355982	0.612658112747496	-0.0552146666666662	0.814211123519969
GABRG1	0.172438607959900	0.556235850599864	-0.209353052982273	0.664703270325548	-0.120221999999998	0.594040749373457
ATP4B	0.172420812868097	0.556235850599864	0.0753554101488649	0.904110561436003	0.0140603333333336	0.965329851466378
SPATA5	0.172417492397907	0.556235850599864	0.167003853856322	0.749894913110563	0.00480266666666716	0.991175862027014
ZBTB41	0.172381694620930	0.556235850599864	-0.0616249260397355	0.923246185283376	0.264848666666666	0.214329708466551
MURC	0.172355600164707	0.556235850599864	-0.250590749835076	0.573718375959741	-0.252359666666666	0.397798624983652
WEE1	0.172350835337650	0.556235850599864	-0.0312647574063632	0.96494188520489	0.0213903333333342	0.946623914607177
MMP12	0.17233018118227	0.556235850599864	-0.0742116972698856	0.905252113490377	0.170357000000000	0.374843323203395
ARHGEF17	0.172311767379948	0.556235850599864	-0.216670682704389	0.647357580911858	0.179200333333335	0.355902971812274
PTGS2	0.172299001119493	0.556235850599864	0.0325499495215388	0.961790688145	0.0102733333333339	0.974895360062947
ZNF581	0.172290673969783	0.556235850599864	0.114814798925604	0.844162087612813	0.0471653333333346	0.843152489417868
CSRNP2	0.172269255889289	0.556264377964236	0.251987253335986	0.570584539772622	0.097091666666668	0.732554472781933
PTPRG	0.172072926121513	0.557024379667748	0.0133738041487466	0.98412883278237	-0.206735666666666	0.486949340358522
ARL9	0.172029885582121	0.557024379667748	0.158127673817911	0.767373487136803	-0.139275666666666	0.502520286871303
C2orf16	0.172020354844341	0.557024379667748	0.211967196588147	0.658364968650984	-0.000244666666666615	0.99883107916854
UGT3A1	0.172001109394079	0.557024379667748	-0.0185929322154032	0.97902968742576	0.0449796666666671	0.87755642632551
TMUB2	0.171983727631923	0.557024379667748	0.084819331340607	0.891309254245872	-0.0293383333333327	0.908590774398726
ESR2	0.171978356160812	0.557024379667748	-0.161822416950853	0.758930446817487	0.0032116666666674	0.993756838831997
TOPBP1	0.171946662849372	0.557024379667748	0.0283425263657687	0.968433247556251	-0.0186659999999984	0.949295980201336
GRK1	0.171900253124515	0.55712048479011	0.230773822796501	0.617027559487982	0.0943233333333336	0.76409396614905
SPECC1L	0.171883974594383	0.55712048479011	-0.0676819932805087	0.913861056515065	-0.0249213333333319	0.932021796104822
QARS	0.171858320067917	0.557195801087788	0.0282993526447563	0.96843394074672	-0.0907216666666655	0.675145197288683
AGBL5	0.171840366028316	0.557195801087788	-0.0589319172358695	0.928159941640702	0.168175666666667	0.396454946335441
ZAP70	0.171635720569976	0.557907551793801	-0.108884787944741	0.853383071635398	-0.102952666666666	0.650128617876381

TXLNG2P	0.171628360265821	0.557907551793801	0.166136063094496	0.751830402832405	-0.00099566666666635	0.997134925912045
C12orf40	0.171548419718793	0.558010367408551	0.191199668078167	0.702044031084227	-0.0209356666666662	0.93901998985171
RGL1	0.171530969801040	0.558010367408551	0.205568978683630	0.672695616352514	-0.0178739999999985	0.954865522683112
RBPMS	0.171520676571807	0.558010367408551	0.298551187938042	0.471239854755259	-0.146602999999999	0.66399337759607
PHF19	0.171470091195069	0.558232671338916	-0.030883899642181	0.96529904314994	0.118911000000002	0.5683587002004
SNX19	0.171414478389003	0.558433331141246	0.156826021174243	0.768401006769299	0.0154490000000021	0.964514058161652
SYNGR1	0.171354277822735	0.558737135685822	0.114478688750928	0.844272085764738	-0.0619080000000001	0.805741750163024
SPICE1	0.171320666756903	0.558873972653314	0.00435870114168564	0.99405344216434	0.0550103333333341	0.855888714554296
PPP2R5C	0.171285529936201	0.559020413541789	0.130193156493608	0.812271254601992	0.0571260000000015	0.811816884537132
SMTN	0.171247955483416	0.559117343433338	-0.0824822963819907	0.8939835772794	0.0267806666666680	0.924674647382585
ADAMTS9	0.171158883496547	0.559518993491259	-0.135248515789538	0.807470535721699	0.756945	0.0309320552705188
SLC7A6OS	0.171135752426843	0.559590049669918	-0.0657167964238988	0.917495226662096	0.105337666666667	0.651315737851928
ZNF740	0.171044586052769	0.559996579875335	0.0410958661241812	0.9521410112665	-0.504112666666665	0.0338015945178966
GDPD5	0.171035612759677	0.559996579875335	0.149839675137261	0.781136269724702	-0.139814999999999	0.541939010010167
POP1	0.170973502697552	0.560284017217623	0.221733694854722	0.637186258054942	-0.339251666666666	0.128450477696566
TCHH	0.170957701576421	0.560284017217623	-0.259374359858904	0.557249797489529	-0.081511333333333	0.702709435280393
ARL5A	0.170883873658926	0.56051712072331	0.285245980426142	0.498216546497375	0.0235036666666677	0.930547487203917
TOX4	0.170854037537057	0.56051712072331	0.149401771954500	0.782293033916778	-0.0538323333333312	0.815275141941482
MDN1	0.170846432932151	0.56051712072331	-0.0777076215483607	0.900452756580846	-0.199895666666665	0.592845916952892
ACSL1	0.170618690994447	0.561364054139834	0.214046361999813	0.653131926870065	0.112424666666667	0.674531431277108
SRGAP2	0.170617274041009	0.561364054139834	0.0646922835341101	0.91904023678194	-0.101609999999999	0.712688984009924
GPR64	0.170588810638643	0.56146882794773	-0.0341006715083211	0.959808677091882	-3.19520833333333	0.00809874206560261
ASH2L	0.170531997707285	0.561677690952078	0.196373544492439	0.691445158350448	0.125246333333334	0.535492155031224
PARVA	0.170490113797473	0.561828920430304	0.119444775596500	0.836013196419542	0.0392580000000007	0.896186784012643
LOC645188	0.170332772311270	0.562611307518057	0.165562172903035	0.752352242644389	-0.0144526666666662	0.972327702257384
KLF3	0.170324681693594	0.562611307518057	0.282184621879389	0.504065779877366	0.0410830000000016	0.891220340561155
SLC16A1	0.170278901826592	0.562731032077964	-0.0349903556135301	0.959140268168928	-0.533261333333332	0.031687055072673
KIF18B	0.170259963224457	0.562731032077964	0.100271062383761	0.866792907657842	0.0592525000000008	0.84207712482186
SHMT2	0.170218511706977	0.562833597069082	0.354253617253613	0.350901200328296	-0.225799333333333	0.271985858153698
STIP1	0.170180796100372	0.562837385752006	-0.079744456830077	0.897892885174022	-0.100756666666665	0.639383542555836
PLK1	0.170170666070086	0.562837385752006	0.0296517853661567	0.967094033447048	0.131646666666668	0.559246628238873
PCNT	0.170141363217820	0.562888927395928	0.202627699492742	0.678279231957132	0.0425826666666677	0.872291063736238
TRAPPC11	0.170118772054639	0.562888927395928	0.173758467001914	0.735353104758227	0.00588300000000011	0.987937064305561
C6orf48	0.170115256505823	0.562888927395928	0.181144486038468	0.721390952113546	-0.054264666666666	0.854011634317602
RAN	0.170069511583192	0.563102755721342	0.191112491537841	0.702165449732036	-0.0651759999999996	0.773652969892764
AHCTF1	0.169921355947412	0.563738915153322	0.0739000193398962	0.905506084101768	0.0909904999999998	0.720071550811076
SPECC1	0.169891488404211	0.563826155298314	0.221685162016399	0.637352459162288	0.191118000000001	0.320462604748547
TRAF3IP2	0.169879595814315	0.563826155298314	0.0807928297927831	0.896509784694312	0.145122000000001	0.49971129098408
EI24	0.169798786871896	0.564103977676077	-0.219930106646524	0.641001372559671	0.140076000000001	0.490929966167076
BRCA1	0.169789572351557	0.564103977676077	-0.21077607386536	0.661480633411348	0.233618000000001	0.258700580229750
ASPN	0.169780832802664	0.564103977676077	0.0150656606815863	0.982921609770846	0.144158666666667	0.484317289991301
DHRS7	0.169666012741841	0.564565144882512	0.351865206142167	0.354413251589651	0.280079333333334	0.189582434602863
C19orf12	0.169633034038588	0.564565144882512	0.175053051228735	0.733276438413475	-0.148353666666666	0.546434057395997
PP2672	0.169628932135642	0.564565144882512	-0.0204445314577065	0.976412247012224	0.192572	0.442640840368423
LRRC32	0.169554968479141	0.564775090476322	0.097509927036279	0.869681361812339	-0.236060333333333	0.367368801980436
ANKRD10	0.169544644863168	0.564775090476322	-0.116602758767589	0.841809730226979	0.122603666666668	0.60814017771842
TCF7L1	0.169493048919322	0.564810888255011	-0.43777666848367	0.194173491891936	-0.070881333333333	0.756063400921947
HAT1	0.169468222940100	0.564810888255011	0.133747017410431	0.808879359838378	0.135824000000002	0.514889931507188
FLOT2	0.169449450659191	0.564841427394517	-0.232840464423837	0.611711680668619	-0.172597999999998	0.51888228204193
MCU	0.169375659307253	0.564994441048022	0.123171019781396	0.827789978865629	0.168149666666667	0.390967882720418
TRIM16	0.169355855319489	0.564994441048022	0.208868786578988	0.665595909869988	0.199209833333334	0.417125112561133
CCBL2	0.169350297002186	0.564994441048022	0.263372566851823	0.545874639137454	0.353083666666668	0.0802641563431964
ARHGDIG	0.169321278943601	0.564994441048022	-0.0165803411949898	0.981654072941606	-0.143892666666667	0.64770351920705
CERK	0.169304887028057	0.565023528258436	0.144769738717142	0.789605229873979	-0.180119333333332	0.487761541713127
ALOX12	0.169274612147659	0.565089189880341	-0.217494536013018	0.645392535370888	-0.265116000000000	0.270000135172916
RARA	0.169207989118824	0.565411742764455	0.082556125511622	0.893825951190383	0.0234886666666675	0.931859006463093
CYB5D1	0.169179701175743	0.565441603545533	0.130506678989607	0.812271254601992	-0.317021666666666	0.107160585319744
CCDC3	0.169115293478536	0.565773583330077	0.0193563204960230	0.9780685294577	-0.103803666666666	0.615689623138822
ELP4	0.168992303166174	0.566475464990173	0.111938319358744	0.847677166378871	0.0382983333333344	0.891339318634358
AARS	0.168831394680734	0.56700839212582	0.180794061369797	0.722159001254958	-0.149598666666665	0.458097980931337
ZNF175	0.168825457436739	0.56700839212582	0.324209621248455	0.413072828507872	0.00505166666666728	0.992055117828222
GLI2	0.168797357215595	0.56711149121242	-0.00896187039048253	0.989225199722122	0.0310823333333333	0.910004971096712
FAM76B	0.168761780510731	0.567187443975173	0.0505800322796974	0.940547229035666	0.103344666666668	0.66091480846429
SLC35C1	0.168697665700868	0.567443692101873	-0.00419616312396007	0.994201024031647	-0.0632859999999985	0.819757926662461
EID2	0.168583765011376	0.567791410741928	0.139650607922521	0.797957231657366	-0.0696606666666652	0.780732284998362
SGCG	0.168538767837676	0.56789770070593	0.00428281928021861	0.99405344216434	0.149267000000001	0.575076472049916
TBX1	0.168528411220325	0.56789770070593	0.259305121645491	0.557422749225218	-0.166209666666666	0.616036866881905
SYT7	0.168517508815413	0.56789770070593	0.168969430495089	0.745643994160493	-0.132204333333332	0.580149967309238
DLGAP5	0.168508094318895	0.56789770070593	-0.087650903677662	0.886302037299497	0.118963333333335	0.584153892885306
FAM219B	0.168483858942276	0.567902137561487	0.157684958703342	0.767770230680061	-0.542708999999999	0.0584908310916042
TNFAIP1	0.168386392156976	0.567923455745915	0.330256776004184	0.402653172067709	-0.495044666666665	0.101980812453755
PRC1	0.16837452461786	0.567924154307819	0.106308515045557	0.858284876027025	0.0546966666666667	0.822401190583587
MPP4	0.168317076109519	0.568064251013372	0.287425039084797	0.493442130049965	0.100374666666667	0.704370299831112
DUSP21	0.168284195602485	0.568123451927638	0.0538300252777456	0.935404504538153	-0.0041140000000001б	0.99209221913287
FERMT1	0.168093565471615	0.568956589843534	-0.100160517693536	0.867048047646049	0.00406700000000094	0.988942941807152
IRGC	0.168053185957565	0.569137537359182	0.355200559555547	0.347833413629182	0.00635500000000067	0.984700985286246
ZNF580	0.167959449871707	0.56943535764532	0.274711773019865	0.520095539214775	-0.0838463333333326	0.741383694227137
ABCD2	0.167927079543202	0.56943535764532	0.372691800878268	0.317205263174137	-0.0948686666666656	0.716268843468132
LEPREL1	0.167926052289217	0.56943535764532	0.203624229403505	0.676207233014711	0.29219	0.147433353861458
HIC2	0.167923819693081	0.56943535764532	0.196795606319961	0.690061983084583	-0.0371656666666661	0.883960545140055

COX7B2	0.167900717767795	0.569507137479082	-0.288619677597101	0.489813374112312	0.141439333333334	0.521771474977985
ZNF224	0.167870383735735	0.56962464916831	-0.0693875325820139	0.91172160664395	0.190778000000001	0.367756122563697
C11orf24	0.167789129941959	0.56982193825055	-0.14531247348762	0.788242201248065	-0.00976966666666657	0.974484855799046
ADSSL1	0.167780177073371	0.56982193825055	0.294779088365158	0.478800228795472	0.0159156666666667	0.955460918122607
CFH	0.167775896109196	0.56982193825055	-0.0988629566169987	0.868042453226125	0.0466246666666665	0.892176227034965
ZSCAN2	0.167758676850448	0.56982193825055	0.0434818505175966	0.949521794694691	-0.0847026666666663	0.692828037753004
TRIML1	0.167519312458168	0.570656703162153	0.0694202351514833	0.91172160664395	-0.104790666666666	0.637426515050706
MRGPRX3	0.167494103969773	0.570667673127201	0.222103209716143	0.636477566065103	-0.066447333333333	0.884755004977469
RQCD1	0.16748118405615	0.570675163855811	0.194428820635452	0.696294554451457	0.0921916666666672	0.669391045681572
STK36	0.167466732931338	0.570692345630974	0.401723873530263	0.261396151283634	-0.0527149999999998	0.824218175636666
TMC4	0.167406217396322	0.570803629774848	0.0459778927467001	0.945364757579857	-0.0532273333333325	0.862509125961694
ROBO2	0.167394707087562	0.570803629774848	-0.316867629337210	0.431921760942787	-0.0821203333333332	0.710483929190267
FKBP14	0.167378692004602	0.570803629774848	0.0686257762152493	0.912149839732916	-0.410199666666666	0.0511690474567547
DCP2	0.167261873974608	0.571145991702399	0.122176106586493	0.829817978832854	0.620244333333335	0.0597108047277182
UBL7	0.167242531548563	0.571145991702399	-0.0707619735365707	0.909764940148113	-0.230354999999999	0.241725325535274
CCDC142	0.167184815698656	0.571436930284476	0.130809888050582	0.812271254601992	-0.0317789999999995	0.90945379489823
GRIK1	0.167043562006547	0.571958795505077	0.0523428368009419	0.937831566264837	-0.0955043333333332	0.641606997692605
TM9SF2	0.167041754751424	0.571958795505077	-0.204813092984309	0.673728362275223	0.0981700000000003	0.631192833987568
ZNF407	0.167033998677149	0.571958795505077	-0.242037870345715	0.59465236351643	0.0534953333333337	0.814273596067945
MAPK10	0.167032003513735	0.571958795505077	0.0555104470207661	0.933409139126562	0.596960666666668	0.0516401497931705
CD300A	0.166987919126881	0.57216363616719	-0.249661261318858	0.575370998761354	-0.072571333333333	0.764973469318093
USP9Y	0.166947507732684	0.572271044055225	0.133402701519313	0.808992949393043	0.0177700000000002	0.948778075888142
GOLGA6L2	0.166907901472383	0.572447584317643	0.139312144175491	0.798615265561297	0.0191373333333344	0.95197590164177
GINS2	0.166781712840774	0.573016081609163	0.240314987514477	0.597862631330416	0.0897650000000013	0.720355976568153
SCRN1	0.166781489153129	0.573016081609163	0.154784130500703	0.771310656070265	-0.0512153333333315	0.831638423014372
U2AF1	0.166733180898399	0.573063388724469	0.056339452077828	0.932140720270928	-0.0419013333333314	0.885895416574211
ELP2	0.166728251150368	0.573063388724469	-0.214308438160709	0.652991165086813	-0.00168733333333304	0.99628178244892
GPBP1L1	0.166716902178376	0.573063388724469	0.0442864394974491	0.948385499132766	-0.048424333333332	0.850914163608035
SYNE3	0.166677033260079	0.573241711051369	0.0946614094376186	0.8739154222556	-0.0396289999999998	0.890094615232462
TRIM27	0.166643551768838	0.573311766785947	0.0534544092383673	0.936250424462674	-0.0547686666666661	0.8257633904865
MYO7B	0.166642540057385	0.573311766785947	0.122790916799690	0.828721578450338	-0.138976666666666	0.528890172622518
FBXO46	0.166616950282132	0.573399657478269	-0.293642364716345	0.480614908214975	-0.0921276666666658	0.710004432586908
DBIL5P2	0.166586283292175	0.573519724602673	0.116309587695574	0.84217383062547	-0.131863333333333	0.688145991467449
PHC2	0.166502121200520	0.57369013909459	0.0797703201807291	0.897892885174022	0.148155333333334	0.581153506516024
ZNF571	0.166500816648497	0.57369013909459	-0.0320093688474727	0.963011981866235	0.0431826666666668	0.890511682678135
AAAS	0.166448702849301	0.573946150001301	-0.139701929039393	0.797957231657366	-0.146576333333333	0.455326357137964
THRAP3	0.166402995942449	0.574161608341810	-0.189147207263542	0.706450470188434	-0.221856666666665	0.294300395506211
HOXA7	0.166330826448396	0.574396362822699	0.105493657343913	0.85979009081468	0.0725330000000005	0.766776438263996
C2orf81	0.166296815149861	0.574537743776411	0.0421692413372494	0.951903861352535	-0.0470633333333335	0.847083064840772
DNAJC4	0.166183025556662	0.575048006722114	-0.114733808792369	0.844162087612813	-0.0680309999999994	0.828025063334271
CDK15	0.166119457659717	0.575291230361796	0.161339347925535	0.759429543907954	-0.860971999999999	0.030025317565228
MAP4	0.165960561258269	0.575691360158352	-0.283126577490952	0.502129505146473	-0.180122999999999	0.451580494926556
TACSTD2	0.165946017494742	0.575691360158352	0.113439122144951	0.845608298802641	-0.215443666666666	0.333881200421942
ADSS	0.165939310966641	0.575691360158352	0.110633171060639	0.84940885580427	0.101755000000000	0.677376696022552
DUSP14	0.165734758100218	0.576381531833468	-0.129495586736205	0.814099225370639	0.133125333333334	0.537105380052834
ZNF575	0.165730659519412	0.576381531833468	-0.112899773305187	0.846769743490891	-0.0577329999999988	0.847165781298603
AGPAT4	0.165713535765500	0.576381531833468	0.356921823969497	0.345138088652208	-0.141084666666666	0.608105979296521
KIF17	0.165651257962836	0.57670261229839	-0.0121428866731099	0.985739212424168	-0.142176666666666	0.558807324932864
METAP2	0.165602844915113	0.576776139010994	0.094120765066562	0.875126060855988	0.0309280000000012	0.902555205061281
C6orf223	0.165601439269058	0.576776139010994	0.221290466237513	0.637956894209002	-0.0994246666666668	0.661264586003921
DDHD2	0.165493433695604	0.577184803459183	0.198526645471157	0.685952362965345	0.243239333333335	0.230692922278365
AIPL1	0.165466264114713	0.577274094856667	0.0776700444631132	0.900452756580846	-0.00573133333333264	0.988133186272453
P2RY14	0.165397861345648	0.577274094856667	-0.0555392043390932	0.933409139126562	0.00884966666666712	0.97810230878021
VASH1	0.165397518616924	0.577274094856667	-0.0383413785960099	0.954545200586067	-0.129737666666665	0.60378773986659
DIRAS2	0.165343875795398	0.577466266417395	-0.0842674142242459	0.892396784625427	0.0326743333333338	0.896446774489185
CCR5	0.165322116538730	0.577508343561084	-0.296367444852128	0.476715216862939	-0.0196393333333326	0.946623914607177
NOS1	0.165312456238062	0.577508343561084	-0.00828709427917327	0.989989309601498	-0.154419000000000	0.540811550863698
PTGDR	0.165302185153119	0.577508343561084	-0.131524913971244	0.811042872465441	-0.223262	0.258098817610 54
PRTG	0.165201017284566	0.577926948562990	-0.0565946504268429	0.93176886339657	0.170106000000001	0.390410184343886
TYMS	0.165162317398771	0.577945371410434	0.148081457384548	0.784080588385358	0.113106666666668	0.592937025021788
EHBP1L1	0.165045937032443	0.578468023675744	-0.318433271770050	0.429140535727328	-0.0541496666666658	0.885345988320645
SLC9A3	0.164959940465446	0.578791790554682	0.222435419816296	0.636029818392136	-0.101287333333333	0.748388402768556
COLEC10	0.164892033563784	0.579074921036525	-0.0340183027727221	0.95993706312687	-0.0234563333333327	0.929594956567283
C20orf201	0.164839696528608	0.57931243070622	0.174395904841504	0.734267769889068	-0.095990999999999	0.74212040995637
ZBTB49	0.164815035583529	0.579330408981646	0.0698180406318064	0.910931201239754	0.00290733333333313	0.993805540619435
OSBPL5	0.164774188490107	0.579378544437305	0.198739959908499	0.685648577129052	-0.0620379999999992	0.83559019443239
DDX6	0.164721929337304	0.57953767367695	-0.214160164266812	0.653042977738486	-0.0134499999999988	0.962371956441728
RHOJ	0.164716602062902	0.57953767367695	-0.288571224519917	0.489962317647236	-0.0950403333333325	0.741134448923316
DDIT4L	0.164702448293762	0.57953767367695	-0.00770139860059404	0.990405674923721	-0.822101	0.0140473413958925
FIP1L1	0.164633285574184	0.579620913171064	-0.221149864221764	0.638281520637887	0.295063000000000	0.124121156456357
CUL9	0.164630768676379	0.579620913171064	-0.0344416872029213	0.959404955370931	-0.134734	0.566282142073858
DEGS1	0.164594164897406	0.579620913171064	0.18337766729151	0.718067440349544	0.260327000000001	0.188082633937580
FOXE3	0.164571375989202	0.579620913171064	-0.0435877311959712	0.94933169799234	-0.164273999999999	0.584153892885306
PRLHR	0.164549301393850	0.579620913171064	-0.0657078882101663	0.917495226662096	-0.045050666666666	0.919359825156123
TPO	0.164459977317020	0.580016379472673	0.208122583659839	0.667327688576615	-0.0724673333333325	0.855321176509099
COPB1	0.164452091495854	0.580016379472673	-0.162302931849592	0.758164869707887	0.0432950000000022	0.86011149680202
PDGFRA	0.164386829751312	0.580282991924784	-0.0477098215871173	0.943560556552289	1.09138933333333	0.00501765297023503
MAPK7	0.164362297066011	0.580290652761879	0.371536733347155	0.319541753509769	-0.025940333333333	0.927795934389862
PLEC	0.164203320024354	0.581079306161993	0.139953534928170	0.7976681602188	-0.029860666666666	0.919456505549688
ARHGEF26	0.16410212912761	0.581649152447826	0.0972595785321492	0.870020870354202	0.0399740000000003	0.895093529187836

SNX27	0.164028341263192	0.581896124070737	-0.0308725200561432	0.96529904314994	0.0995616666666665	0.673514912541661
FLJ11292	0.163934052134350	0.582145426472867	-0.126034664625097	0.822164329801713	0.0353090000000005	0.921455143042509
FOXF2	0.163899800997706	0.5822690038349	0.184200607425787	0.717436396325728	-0.00670333333333327	0.987937064305561
TREH	0.163836247422109	0.58259754049008	0.222107962431229	0.636477566065103	-0.0153906666666661	0.962762677953737
C2CD4A	0.163824899220149	0.58259754049008	-0.225378953530684	0.630067587788191	-0.198269666666666	0.537636136387445
DHX34	0.163802758326988	0.582650708933399	-0.281508161652952	0.506335763951991	-0.186135666666666	0.414405354802607
SLC25A51	0.163793227792611	0.582650708933399	-0.381788574765846	0.296921777642531	-0.0374466666666662	0.904507944803624
KLHL22	0.163773582653214	0.582701569817349	0.0137943350964429	0.983872723592937	0.179759333333334	0.408369243753251
STARD9	0.163685085216915	0.58295504947435	-0.0100723830772603	0.988421999436642	0.0239140000000002	0.934016415336754
IFNG	0.163656039136230	0.58295504947435	-0.333443814503250	0.396821740334178	-0.0589600000000002	0.803131392712617
SEC31A	0.163610899332227	0.58295504947435	-0.0271942557435823	0.970207612777188	0.108333333333333	0.607898776363481
AGAP3	0.163608808087895	0.58295504947435	0.0852015502737591	0.890458025676316	-0.0312186666666652	0.904236796123256
VGLL3	0.163599470828674	0.58295504947435	-0.0604669402484327	0.925565513580637	0.0393373333333339	0.903774117860245
GAL3ST2	0.163561562814603	0.583072462637914	0.598114863903599	0.0337939911292242	0.0524796666666672	0.83575292572393
NOTCH3	0.163481603522670	0.583373629107629	-0.0840625303916669	0.892634402174414	0.00788233333333456	0.985484787924072
XRCC6	0.163446175639080	0.583408259360654	0.107762547195294	0.856450662791083	-0.0706833333333328	0.750929580167368
SMARCB1	0.163431589391309	0.583408259360654	0.219831936911581	0.641035290381005	0.00416600000000113	0.990402175833635
GOLGB1	0.163429573729853	0.583408259360654	-0.139463962876414	0.798450177373409	0.0300166666666681	0.91131019859749
SULT4A1	0.163410311085903	0.583456779857505	-0.0641158076689042	0.920005468161721	-0.0150133333333328	0.964688677076962
KRT222	0.163387673556823	0.583460998793952	0.155512894273863	0.770174160140798	0.209549333333334	0.274121181062751
ZC3H7B	0.163340042391781	0.583533537424062	0.354855355980387	0.348722583996092	-0.103275333333332	0.6460143883793
ZNF239	0.163254761490708	0.583854342309044	-0.183428920162738	0.718067440349544	0.144247666666667	0.52128449277411
CPSF2	0.163234988048084	0.583906159911432	-0.210581517970516	0.661670522548494	0.188418	0.322753005561608
RPL41	0.163088580410019	0.584097582794797	0.0907306453754889	0.881323965029954	0.0347043333333344	0.891410328517388
CLDN11	0.163037564066731	0.584200294774187	-0.0429345531601805	0.950243031196165	-0.173552333333333	0.528409919089401
XPR1	0.162995059314170	0.584274714115258	0.104798616965014	0.86067831050617	-0.228753666666665	0.248864745773935
ARVCF	0.162940378614815	0.584374966811463	0.153665808810730	0.773358453580566	0.0686846666666676	0.780717071872908
PABPC5	0.162905289923564	0.584419769587582	-0.0146457006579231	0.983444353656142	0.0201050000000005	0.950980037243398
TLR1	0.162738344244248	0.584995659056984	-0.322326147012850	0.417538034812104	0.233392	0.376224594108747
CASP14	0.162719350793106	0.585033101068823	-0.0416794860093459	0.951989245458556	0.0186940000000003	0.958416953173227
DIO3OS	0.162704530286138	0.585033101068823	0.21554187073938	0.649546282524329	-0.0958973333333324	0.712653771000543
TRPV2	0.162697610435284	0.585033101068823	-0.208636959280321	0.666278181721133	0.173152666666667	0.469753357257375
C21orf88	0.162629001087719	0.585185395858916	0.276976685132410	0.515587194962102	0.0510550000000004	0.859299487874574
SUMO1	0.162627782575263	0.585185395858916	0.1836034819295	0.718067440349544	0.00890233333333428	0.981672346979786
HSPBAP1	0.162604784461253	0.585185395858916	-0.141806089403891	0.795069777846995	-0.090542666666666	0.68135180147671
AGAP11	0.162593471266141	0.585185395858916	-0.0394176166768004	0.953905313456582	-0.0417346666666663	0.865655368707012
SLC25A42	0.162592400789587	0.585185395858916	0.138144232974241	0.800478820487963	-0.098256999999999	0.656324975591247
CIRBP-AS1	0.162541312976965	0.585362910219145	0.111713070927488	0.848331918863767	-0.0187380000000000	0.950564200758787
GIPC3	0.162349556967682	0.586289055159345	0.303015793567758	0.462105017205825	-0.179534999999999	0.592716584553539
RPL37	0.162267544371196	0.586603154570441	0.384129596578627	0.292263251507162	-0.128251000000000	0.541952482941864
GGNBP2	0.162265464010872	0.586603154570441	0.106123201242583	0.858770922770743	0.035937666666667	0.884175799031047
KCNK13	0.162243177868384	0.586671231047169	-0.151548812032591	0.777101208632856	0.0954430000000006	0.727809376593958
NFYC	0.162221685382736	0.586734250808924	0.0410187888970399	0.952394447098818	-0.0461403333333329	0.852208015389387
PON1	0.162137420334614	0.587123657595299	-0.128028526186004	0.817554677762205	0.0105050000000002	0.973770798978734
ZNF557	0.162075462249169	0.58729672487233	0.0911039688513388	0.880960609633754	0.153818000000000	0.508507145194664
DPY19L2	0.162030984023676	0.587347505174131	-0.052848117239375	0.937009489777134	0.464894666666667	0.031687055072673
PNISR	0.161933666329892	0.587831204387909	0.182172594096621	0.71964467602874	-0.0443373333333324	0.856868322249419
STX17	0.161895281054065	0.588002137705721	0.293697709172643	0.480614908214975	0.185570666666668	0.337307065334106
HDGF	0.161845102366708	0.588174269827829	0.0951396523120337	0.872962271153999	-0.251419999999998	0.218478495014124
ERCC3	0.161772342614914	0.58835054179732	0.0275546219436961	0.969821510572266	0.0218531666666685	0.945904449997854
SLC24A2	0.161767564373080	0.58835054179732	-0.00121629829615308	0.998348925592729	0.118188333333334	0.553892046464523
MED24	0.161689343991719	0.588427791682219	0.00562422362839815	0.99251028338798	-0.0378699999999983	0.882342951312785
MAP3K13	0.161638662965066	0.588677309754282	0.377237423408632	0.307297021174057	0.204665333333334	0.527465067495661
SDR16C5	0.161580103785320	0.588725573433038	0.026613189823917	0.970207612777188	-0.103858666666666	0.660199472343126
SPINK5	0.161496196150946	0.588994516126126	-0.126502233668945	0.821473247266105	-0.0606893333333327	0.815156860161333
CNTN4	0.161430542195984	0.589051003277096	0.26841361026454	0.535217216332192	0.563946000000002	0.0895407923270921
MEP1A	0.161415368508766	0.589066450988932	-0.430610293390982	0.206606138978772	0.00306066666666751	0.993677488355417
GPRC5D	0.161392893065920	0.589135946316643	-0.182118057175728	0.71964467602874	-0.194067	0.514420666507349
RNF182	0.161350973973769	0.58925559056145	-0.132527725471185	0.809866082339655	0.201641333333334	0.452256892553908
SEC61A2	0.161308585467375	0.589396343512822	0.0974697077790039	0.86975668481123	-0.0495706666666654	0.86183426299397
PAOX	0.161301483716909	0.589396343512822	0.384527725025306	0.291180853572672	-0.009425666666666571	0.980944165465284
HECW1-IT1	0.161285668069427	0.589396343512822	0.0935945179500382	0.875932944246867	-0.0550029999999994	0.813038858174245
PPP1CC	0.161282578734623	0.589396343512822	0.174306105110903	0.734267769889068	-0.244659999999997	0.205838922584493
SKIV2L	0.161173103604396	0.589913990953418	0.196309678083805	0.691590431497479	-0.0872424444444443	0.68135180147671
PROSER1	0.161149341658176	0.589913990953418	-0.100817490054449	0.866035166924059	0.345604333333334	0.210845715069665
APOA1	0.161043828730428	0.590477363468414	0.34200104858449	0.377158872804021	-0.0960788333333336	0.770261991339376
NR2F1	0.160945269627363	0.59073701765132	-0.372222760239192	0.318055515780537	-0.0247046666666656	0.931859006463093
GULP1	0.160926370853359	0.590783765711432	0.229812473839409	0.619381301776937	0.548209666666668	0.0185304620090284
HOXA3	0.160881717432271	0.590995147158906	0.332926976030818	0.397658808533403	-0.0250793333333333	0.937717709824356
CSN1S1	0.160738904201570	0.591612037175829	0.239872136630281	0.598134271398487	0.0301580000000001	0.904507944803624
BRAT1	0.160513039954395	0.592744464023904	0.102389802531988	0.863428757305077	-0.0891906666666673	0.6846855637534
ZNF512B	0.160312169036611	0.593305229940031	0.171445991766391	0.739541495555634	-0.282197666666665	0.136476606225025
SLC35A1	0.160170726301613	0.59398809397981	0.482678278078681	0.135560525489292	-0.294058666666666	0.378876286708405
PRPF6	0.160159237803467	0.59398809397981	-0.380225019954294	0.300705577749487	-0.0095639999999989	0.974451420920923
ASPM	0.160137325695673	0.594054277810102	0.017268886292583	0.980723199123135	0.0337910000000011	0.910004971096712
TACC3	0.16009653147625	0.594167243552827	-0.00012238989971459	0.999860038008296	0.0849036666666671	0.720355976568153
MAX	0.160070837464892	0.594257679273084	-0.31847615698733	0.4290520704081	-0.213764999999999	0.313220411537404
DYNC1H1	0.160045421792455	0.594346338390519	0.062441826103453	0.92264749701958	0.0225746666666681	0.949376459031393
RAD18	0.160015341171191	0.59446490474432	-0.183402436974440	0.718067440349544	0.00798700000000103	0.982375192575954
BDP1	0.159986771673685	0.594499566948031	0.0663016950873762	0.916518252419605	-0.0522533333333341	0.822980493051179

PRSS21	0.159849119648208	0.595085081946662	-0.30756911938819	0.452689746287922	-0.207651666666666	0.43441407814235
ZIC1	0.159684020217696	0.595698381709174	0.592257462540168	0.0355984111824064	0.337045	0.120066554405991
GPR21	0.159623599705489	0.595865844161438	-0.0211440167511977	0.975917488338947	0.0102046666666672	0.974484855799046
SEMA4A	0.159612724404275	0.595865844161438	-0.176338132396529	0.7303866161159	0.0644460000000006	0.779485206901945
ANO9	0.159611606773427	0.595865844161438	0.0733058850515543	0.90588212168674	-0.185773666666666	0.477169587518779
LRRC71	0.159557475031490	0.59613887621942	0.177281162796318	0.728735147897621	-0.0606673333333332	0.855886648212195
CCL8	0.159475471498729	0.596590882110678	-0.203361934061187	0.676646782959283	0.154262666666667	0.661145927185587
SERTAD4	0.159299344928852	0.597209501733381	0.0469694331645572	0.944081286115546	0.249332333333334	0.310158645250256
MAP2K5	0.159232552613555	0.597416642081719	-0.0699586981108781	0.910885619751085	-0.0617889999999989	0.808253941454262
ALPL	0.159219527950341	0.597416642081719	-0.184641412656391	0.716351619471715	-0.413846666666666	0.0631498594066912
ASUN	0.159178212098486	0.597607670732576	0.0137408230672145	0.983872723592937	0.0573750000000012	0.839139657595693
PCIF1	0.159010201901225	0.598090704606163	0.0140858575700910	0.983444353656142	0.0734103333333348	0.736851545067772
GRAMD3	0.158990364499819	0.598090704606163	0.220027054848857	0.640746645143474	0.229452333333334	0.370208571402686
PKN2	0.158987304292290	0.598090704606163	-0.224163450507696	0.633180978990857	0.0821993333333344	0.712601873916151
SLC25A44	0.158786090418618	0.598698976244614	0.0899709653522437	0.88250679197721	-0.482897666666666	0.0308637336054088
NEO1	0.158757574059706	0.598698976244614	-0.211693622008017	0.658991855878698	0.166175000000001	0.445609276803149
TRAPPC6B	0.158753849316965	0.598698976244614	0.186401030865656	0.711640652971159	-0.198014666666667	0.40610776759241
SVIL	0.158727058006529	0.598723620158138	-0.254051010076791	0.567694846153007	0.120821000000000	0.589826330962094
DNMBP	0.158653525410279	0.598777388948049	0.0302604103306486	0.966191618983294	0.123174000000001	0.572207354314478
MTF2	0.158637629041219	0.598777388948049	0.0763465086911188	0.902928663998623	0.0584010000000002	0.799372147044399
MGC39372	0.158598113680239	0.598957005268319	-0.0416526955492828	0.951989245458556	0.0591750000000005	0.828279845160572
SIPA1L3	0.158472814388146	0.599539412068711	0.190681578090238	0.703262855228403	-0.100516999999999	0.711082533709514
LOC100288637	0.158434773789923	0.59954392913783	-0.0352908448902049	0.95869292666698	0.0131890000000005	0.969513647149429
SNAI1	0.158426766583430	0.59954392913783	0.0546159444065724	0.9344692810234	0.0101180000000003	0.981191596773742
GPR55	0.158405179161055	0.59954392913783	0.0967015468391245	0.870857630743202	-0.196711666666666	0.549323798856043
MANF	0.158340327635668	0.599722752473177	-0.308694854680094	0.449759364777049	-0.0871256666666663	0.68717273201429
CDC42	0.158310063314429	0.599808342517602	-0.179915713780676	0.723716289903531	0.0245620000000002	0.927365532267852
TBCD	0.158283660618708	0.599864305533342	0.284808596105509	0.498591817620473	-0.0179780000000001	0.947542885546135
EIF4E	0.158252294180275	0.599979780485236	-0.120741536795956	0.833602134657439	0.00903900000000065	0.978192116090894
TRIO	0.158221845553644	0.599979780485236	-0.147064664184747	0.785734908309155	0.224690333333334	0.267638034014719
OSBP	0.158219514605803	0.599979780485236	-0.0548900819192679	0.934255557320745	-0.113249666666667	0.626635388688185
RAB3D	0.158165341417010	0.600179566692836	0.0601620964411814	0.925649366494154	-0.00674266666666608	0.986290211615582
GABBR1	0.158070318005594	0.600491837136566	0.273349802373551	0.523887082296009	0.0278900000000012	0.911623909907545
USP47	0.158035974257249	0.600491837136566	-0.157382093638568	0.767770230680061	0.151179666666667	0.438758057008766
C20orf112	0.157997426031348	0.600528147382781	-0.186513773238630	0.711334804690432	-0.118930333333333	0.58058332386745
YPEL2	0.157995715137879	0.600528147382781	0.194770247121686	0.695696493534439	-0.104672999999999	0.662751767068109
PRDM15	0.157980759795849	0.600546862354767	0.0242301198647293	0.97415099097231	-0.0765026666666656	0.741438256509195
SFSWAP	0.157823864192111	0.60096344084338	-0.0782478736570219	0.899647854618521	-0.0875203333333332	0.696927295330661
ATXN2L	0.157813689559311	0.60096344084338	0.212019917575391	0.658350230734701	0.0236463333333336	0.927671110581394
ARHGEF7	0.157812662177670	0.60096344084338	-0.107428220336889	0.857065724607536	0.189005666666667	0.329908083538015
PKD1P1	0.157733499085478	0.601236967833506	0.191616722191907	0.701635983922402	0.0894320000000016	0.674286385759267
ANGPTL7	0.157723992426348	0.601236967833506	0.325738078282615	0.409206882509236	-0.128432666666666	0.614221661998915
ZRANB2	0.157632528367822	0.601602637583853	-0.172017301989382	0.738897289340835	-0.0228893333333322	0.93278021276477
FAM204A	0.157589166593950	0.601802147466037	0.0745258679465894	0.904832407812985	-0.0561420000000007	0.849384482495249
C10orf118	0.157543169428732	0.601802147466037	0.200533334533610	0.680830337398347	0.0907016666666678	0.718749100405876
REG3A	0.157539329232700	0.601802147466037	0.181189523322138	0.721382789731275	-0.135624333333333	0.567355756887702
CYCS	0.157425046141965	0.601971168723392	-0.230255843993512	0.618707227156037	-0.0257913333333323	0.926815497753858
STAT3	0.157371551758091	0.601971168723392	-0.362243230506534	0.337908913488640	-0.136338333333332	0.500226891090192
SFXN2	0.157365734860729	0.601971168723392	0.243678314208316	0.590086964098531	-0.213382333333333	0.257194631784673
OLFML1	0.157357606188267	0.601971168723392	0.0346477190971512	0.959404955370931	0.113009333333333	0.607898776363481
FLII	0.157344821288963	0.601971168723392	0.159438593130952	0.764209767000699	-0.00833333333333277	0.980779858267938
WNK4	0.15722267172792	0.602316295843635	-0.0477495689851891	0.943547842937191	-0.0406599999999989	0.866339388825863
GMIP	0.157222064216405	0.602316295843635	0.000922777509462902	0.998745415362821	-0.106264666666666	0.635069698575895
FAR1	0.157061477414045	0.602535123043039	-0.180150914004386	0.723525599963752	0.0879526666666678	0.67320648871569
ZYX	0.157029374004355	0.602648971723316	-0.368907933293258	0.32362669860138	0.129831666666667	0.534196070908831
LIN7A	0.156975818671965	0.602731333106124	0.573624562887378	0.0442317804409017	0.0911253333333341	0.789228734235839
COX3	0.156973455005262	0.602731333106124	0.184241402716564	0.717436396325728	0.160230000000002	0.436636858796753
ND6	0.156876863476235	0.603204339482139	0.0560930133256789	0.932663091036367	-0.257836666666666	0.265612333098174
PPP1R12A	0.156851817119594	0.603291440763405	-0.203187657564919	0.676760640226365	0.0702383333333335	0.778247870247617
RPP30	0.156838327659197	0.60330425444403	0.036823094476719	0.956918184553224	0.0242910000000004	0.924813456209956
DCUN1D1	0.156780752137797	0.603470260870189	-0.217078734421415	0.646775608530967	0.124644666666667	0.571070613702241
RBM15	0.156685133690041	0.603877059330022	-0.0730032481192669	0.906592046281011	-0.324273666666667	0.0942538294765004
LHX2	0.156658141455622	0.603877059330022	-0.243667233339999	0.590086964098531	-0.0594186666666651	0.809907899558073
CA2	0.156656740678832	0.603877059330022	0.046435561038432	0.944852355718197	-0.00330866666666622	0.993634447740042
TFCP2	0.156645789054181	0.603877059330022	-0.122517670678187	0.82920723741687	-0.0280509999999993	0.915761046438965
FOXP4	0.156605627797430	0.60398752200376	0.201070221965970	0.680143077995217	-0.0692656666666663	0.766434517050646
PRDX2	0.156515928589267	0.604379112918135	0.241900630970257	0.595018350907538	-0.0154016666666658	0.955620005467488
ALDH1L1	0.156503011639578	0.604379112918135	0.130822793359108	0.812271254601992	0.0176440000000005	0.949295980201336
PRR11	0.156422713192509	0.604607798755542	0.0813445813571606	0.896271792537737	-0.123618666666666	0.546434057395997
TDP1	0.156420252098776	0.604607798755542	-0.438221353802752	0.193398158224439	0.0876853333333334	0.755697432495201
GALNS	0.156415156221896	0.604607798755542	0.0950826981615119	0.872962271153999	-0.00101099999999909	0.997144862160692
KCTD2	0.156394294723607	0.604607798755542	0.268574262426588	0.535014466593144	0.175666333333334	0.356206927896033
MLLT3	0.156375457629196	0.604655083668601	0.322602208074592	0.416987214962026	0.119830000000002	0.580149967309238
SLC17A2	0.156312497778143	0.604983207884105	-0.134474652365492	0.808009477165608	-0.160614666666666	0.456799968498263
OVOL2	0.156275292390552	0.604983207884105	-0.125085368981142	0.824230922676958	-0.0816303333333326	0.824548552404467
XPO4	0.156255543404487	0.604983207884105	-0.405450569530963	0.254747594046254	-0.0582686666666655	0.824257394304182
KIF25	0.156184305569756	0.605182333361167	0.275187572519690	0.519867577693334	-0.0597333333333327	0.856640814502011
CLDN10	0.156178664854218	0.605182333361167	-0.0916432371492401	0.879272912391142	-0.00957599999999969	0.98147292539484
TSSK1B	0.156095500871603	0.605421959134304	-0.0466745066503798	0.944567208068726	-0.0352423333333331	0.904898244447396
EIF4E2	0.156050325956863	0.605538954242519	0.247732876148213	0.579471329472972	-0.0877216666666653	0.72802494241369

IL23R	0.156035536253096	0.605538954242519	0.302741825038019	0.462105017205825	-0.123659666666667	0.777406710539424
ZNF500	0.155975609947039	0.605669225490812	0.407715314856476	0.251655786339022	0.0347000000000005	0.903788355106891
FIZ1	0.155965267015219	0.605669225490812	0.0152495873290406	0.982921609770846	0.0140573333333343	0.960201918224867
EIF3M	0.15589318246412	0.605718744030191	0.456923334179613	0.167195938822765	-0.0865163333333325	0.680455232178275
DHRS13	0.155875253477116	0.605718744030191	0.270540290694267	0.529728353891894	0.0992916666666673	0.635267430402483
PIGW	0.155793137761556	0.605891285268995	0.201516245654953	0.680054545720343	-0.0536859999999999	0.843152489417868
ATAT1	0.155721499833415	0.606130219843663	0.0529093382737686	0.937009489777134	0.115669444444445	0.56909288260534
SEMA3D	0.155675658444954	0.60621789317646	0.0610939269307176	0.92470690387802	0.639112666666667	0.0204315499063131
BIRC5	0.155653768669527	0.606223135568862	0.159268115139118	0.764789523460873	0.054754833333334	0.823699339501946
ZBTB24	0.155620542465438	0.606264834080511	0.379606350586377	0.302065502142393	-0.0716023333333328	0.798344353938564
DMC1	0.155601970831386	0.606310055272322	-0.154622933179143	0.771310656070265	0.191103666666667	0.329908083538015
DSEL	0.155580010441213	0.606354099994578	0.217491690156596	0.645392535370888	0.280559666666668	0.364670331125492
IRX5	0.155542632474075	0.606354099994578	0.387092474161564	0.287581226843956	-0.469351999999999	0.0599913479443629
C10orf107	0.155502361212766	0.606508696461883	0.317525671239838	0.431166148274203	-0.0771949999999997	0.727311316699018
R3HDM4	0.155446246565993	0.606692758459855	0.0140910261741646	0.983444353656142	0.00682833333333333	0.983159241812861
IGFBP7	0.155302393856966	0.607280484256428	-0.0923410481024926	0.879133517463076	0.154003333333334	0.482760288609588
ILKAP	0.155208723351467	0.607638616494022	-0.0426370017358942	0.951071128215665	-0.0241566666666658	0.933412982728621
ZKSCAN2	0.155163527642754	0.607695794911339	0.00139849320436502	0.998303187767855	0.0541510000000012	0.853219850381138
MAGEA6	0.155135389426743	0.607764590035877	-0.0408608311620611	0.952426531595115	-0.104026333333332	0.61423776713215
NAV1	0.155073574665584	0.608015067862261	0.257308375628108	0.563116080558022	-0.151348666666667	0.481989564291462
ARHGAP31	0.155007533731303	0.608097705761365	-0.366977012625758	0.327869177739765	0.0505773333333341	0.891410328517388
RPL13	0.154900581273400	0.608345803896549	0.132952237165912	0.809578511927685	0.0323506666666671	0.898979600726666
FAF1	0.154899226855543	0.608345803896549	-0.0896744498992197	0.88319189898944	0.0678683333333348	0.762898679429892
CSNK1G3	0.154883876621468	0.608345803896549	-0.229539793024623	0.620183562399927	0.163549000000000	0.459624813256835
DYM	0.154853284139549	0.608345803896549	0.102293274172653	0.86390358875224	0.132991666666667	0.499850659274909
PFN1	0.154829900932797	0.608345803896549	-0.179494630517896	0.724770967840177	0.0919606666666681	0.661383805087653
ZBTB9	0.154827706083100	0.608345803896549	-0.0764229806044239	0.90277020200366	-0.156320999999999	0.491578070092
LRIG2	0.154800491375230	0.608370037775526	-0.214967017572063	0.651262360178562	-0.00862333333333234	0.983690244124512
CGRRF1	0.154706296810703	0.608728031892527	0.100228608072964	0.86681045025669	-0.0635276666666656	0.809505390888975
LTB4R	0.154675380050302	0.608811025711033	0.283571301245634	0.501852406168029	0.11782	0.613418255499423
AKR7A3	0.154595633971997	0.609115122601608	-0.115887849658861	0.842733236705133	-0.0523313333333327	0.849032599945
TRABD2B	0.154590682538953	0.609115122601608	0.075745691382796	0.903510413986113	-0.139013999999999	0.594374666543557
LRRC59	0.154525965516994	0.609184211582294	0.182891516904421	0.718289071039953	-0.312952666666665	0.137453276113987
CAPZA3	0.154430197914666	0.609615692757607	-0.0365192425658244	0.957361979121658	0.0235843333333339	0.947020470290463
DNAJB14	0.154407474824289	0.609615692757607	-0.197652563046867	0.688640225556359	0.238818666666667	0.211495399841293
TSKU	0.154369440896378	0.609615692757607	0.130376513168143	0.812271254601992	-0.209602999999998	0.270097728053891
LASP1	0.154357951096395	0.609615692757607	-0.276328360942937	0.517374509850433	-0.049137666666666	0.839408394792728
INO80B	0.154318859778737	0.609634972814063	-0.0205961358698130	0.976404959469803	0.104155666666669	0.635069698575895
RXRG	0.154276741199187	0.609832539824108	0.278713651652047	0.511025440770733	-0.112280333333333	0.704871474914214
RBPJL	0.154253610277256	0.609907925618759	0.115082584505880	0.844120246770255	-0.183060333333332	0.395806187250994
MTSS1L	0.154182244347738	0.610146811120858	0.270687740204674	0.529418563170514	-0.143544833333332	0.472124270069641
SCML2	0.154161668943095	0.610205767531998	0.171347469529367	0.73955239116035	0.784884000000001	0.0739218716203512
DOC2A	0.154150109525966	0.610206693852449	0.290281002423416	0.488568550695131	0.0243996666666672	0.935271974478465
PMS2P4	0.154119930260154	0.610327477504616	0.123014836585902	0.827904654392021	0.136571666666667	0.500875207139132
TAAR9	0.154092097361423	0.610359691387372	-0.163538581889753	0.755130122263395	-0.161034333333333	0.463103460780694
RB1	0.154063517913189	0.610470190902766	0.0716406530580764	0.908546712333622	0.0124186666666669	0.969104903637376
CCT2	0.153986235923232	0.610649326360155	0.0416127749658509	0.951989245458556	-0.0612799999999992	0.799725367501923
PDIA3	0.153978631534945	0.610649326360155	-0.383778595254489	0.293447764611617	0.120318333333333	0.565148387675909
NBR2	0.153964449217410	0.610667174033182	-0.02803212942832	0.96879496669932	0.185946333333333	0.340052241399374
TLX2	0.153890620676688	0.610995564908636	0.252928896877567	0.568985221714455	-0.185508333333333	0.627791771823967
CREB3L2	0.153863706835202	0.611095394650550	0.210296683133199	0.661935596679852	-0.0273846666666659	0.922697964464038
RAD54L	0.153769730893315	0.611440479260086	-0.0377665669888135	0.955190852940141	-0.0149829999999987	0.964192013934086
EDEM3	0.153764484114987	0.611440479260086	0.110555222779615	0.84940885580427	-0.0134939999999990	0.968356730754677
C12orf42	0.153708973635378	0.611631192418435	0.0433489226593124	0.94974708351052	-0.107822666666666	0.602084409940976
ARSF	0.153659340435399	0.611823802310667	0.147446679357814	0.785497496933202	0.0238649999999999	0.936557228489982
PPP2R5D	0.153643506451055	0.611852320096486	0.0339868683484955	0.95993706312687	0.016087666666668	0.955460918122607
SCN11A	0.153581033315105	0.612129845716746	0.0462368118378426	0.94512688522026	-0.132838333333333	0.502832431909902
LPPR2	0.153543963699459	0.612129845716746	0.437700373435698	0.194173491891936	0.143292333333334	0.566075188962
CCDC82	0.153543398809895	0.612129845716746	-0.114675445778271	0.844162087612813	0.271631666666667	0.205336588312115
ZNF14	0.153482555970605	0.612448382873663	-0.388081357715249	0.285763378960147	0.0881610000000014	0.78780036542932
RANGAP1	0.153425340170092	0.612682249233951	0.0156608609693153	0.982266426585123	-0.163781666666666	0.48174711509713
ZC3H13	0.153423456804680	0.612682249233951	-0.134020013362147	0.80854627520514	0.000684000000000058	0.997953056544486
PAQR7	0.153390536336541	0.61278579196294	0.395549310321418	0.272692742937895	0.318199000000001	0.163986811016470
ZNF282	0.153384582525001	0.61278579196294	0.315181027271214	0.435703778828941	-0.017868333333332	0.95197590164177
NTRK2	0.153354877444189	0.612903751923554	0.152937693602987	0.774656266040138	-0.00629799999999942	0.990385063260966
RPL27	0.153307738103426	0.613134115015201	0.242911265403146	0.592178030679976	-0.040693999999999	0.867268897859745
FBXO22	0.153267403850067	0.613320639102166	0.0187031387762639	0.978981596219275	-0.0132719999999992	0.963206882178337
STRN3	0.153192961418402	0.613727178860531	0.0865246222192964	0.887493600482322	-0.0233253333333323	0.928897428735123
MTR	0.153162190076695	0.61377851865632	0.272448885820172	0.526175116911121	0.274434333333334	0.235054998483403
CHAD	0.153116853104420	0.613879306845009	-0.09654864498131	0.870936619256698	0.0346256666666671	0.901808198966559
CRISPLD2	0.153100948346162	0.613879306845009	0.125268998087845	0.824230922676958	-0.374628666666667	0.100026144425143
AREG	0.153055683051223	0.613985801374944	0.330734443001292	0.401940724198604	-0.164467333333333	0.5488879250835
KIAA1430	0.153042899351448	0.613985801374944	-0.064003523131727	0.92003791975205	0.0412863333333344	0.877597838720525
STXBP5L	0.153022989759754	0.613985801374944	-0.0165837138077879	0.981654072941606	0.467398000000001	0.0351167468854759
DYNC1I2	0.153016034392179	0.613985801374944	0.100721329039312	0.86628046156792	0.0434493333333344	0.883189085233712
IGFBP6	0.152911483344009	0.614513144682209	0.107698758098208	0.856450662791083	-0.00155999999999916	0.996285985364494
RTN4R	0.152830191141348	0.614686781074613	0.323050952315732	0.415854621854132	-0.147511666666666	0.499957077244054
KLHL36	0.152823744692439	0.614686781074613	0.211428274091782	0.659576946537835	-0.0942576666666652	0.658976306010765
ZNF570	0.15280004041125	0.614686781074613	-0.306318903187448	0.456028340595575	0.0539553333333345	0.879265091272724
DNAJC3	0.152766460574553	0.614787028876594	-0.263991669463539	0.544711371453131	0.0427560000000011	0.889275143490577

FAM104A	0.152732194978204	0.614861085422025	0.285576687114861	0.497161201540639	-0.156127000000000	0.433950673944394
ZNF182	0.152686385354142	0.61508313695143	-0.152374453528929	0.775863712880333	0.000458333333333369	0.998556628992385
TTC5	0.152632906229509	0.615205800698938	0.211513422489803	0.659412013514194	-0.135754666666666	0.630743046685015
GALNT3	0.152601128650791	0.615205800698938	0.152473086361883	0.775863712880333	0.126118333333334	0.585987887254773
RBFOX3	0.152600512930500	0.615205800698938	0.113507973695536	0.84538474875759	-0.1201255	0.615722818909839
MYO1D	0.152575793705918	0.615205800698938	0.202400840240819	0.67896916637466	-0.134024000000000	0.695654544311453
TSPAN11	0.152483791598583	0.615402018161614	0.135362730559679	0.807369064541864	-0.869372666666666	0.0184916897836653
FBXL19	0.152483178485954	0.615402018161614	-0.0285507655415594	0.967880249275467	-0.0431400000000003	0.862626061994523
GRIN2D	0.15243297702715	0.615402018161614	-0.0486337701779396	0.94236319435931	-0.287350666666666	0.210845715069665
HHIPL2	0.152412888402674	0.615402018161614	-0.0753106928232	0.904137822927282	-0.100651333333333	0.677277330785112
AGPAT3	0.152411438943325	0.615402018161614	0.101170069370337	0.866005093242444	0.00402866666666724	0.99117823918328
RPL22	0.152409558927114	0.615402018161614	-0.251129123282063	0.572882168047464	-0.136217666666666	0.631192833987568
WDR52	0.152409243745323	0.615402018161614	0.0439706631653407	0.94865610372986	-0.0554646666666662	0.86218518452039
DIO2	0.152372969718145	0.615562584336544	-0.0155441681458583	0.982505387450863	-0.0221303333333332	0.954608215091566
MAGEC2	0.152336985882540	0.61560085677717	-0.225044155962943	0.63103711743427	0.0121503333333338	0.96681311917387
RGS5	0.152321529999892	0.61560085677717	0.0616189641539104	0.923246185283376	0.314806666666667	0.148479926866127
IQGAP1	0.152271324756202	0.615851298767444	-0.352009444307036	0.354183999067581	0.268370000000000	0.191850325697419
YBX1	0.152244997700060	0.615851935505223	-0.0445907878306257	0.947920610570169	-0.0361399999999981	0.890094615232462
SRPX	0.152229023933947	0.615851935505223	0.351802602796204	0.354413251589651	0.770540333333334	0.131369372059974
SAR1A	0.152225730738583	0.615851935505223	0.019213529861938	0.978232639595454	-0.602012666666665	0.0250150663657547
NAGA	0.152171372162548	0.615968427849402	0.0441068717547045	0.948520928173175	0.110180666666668	0.589703432017128
XRRA1	0.152123059147648	0.616147436498265	-0.0659365518510832	0.917090201751967	-0.0383973333333328	0.904507944803624
CDC42EP4	0.152103497057091	0.616192548068422	0.367558765539669	0.326948811683298	0.0622850000000012	0.814780717126634
MALAT1	0.152093326822068	0.616192548068422	-0.165873057116347	0.751869806279813	-0.0991949999999983	0.689661288408633
PIGM	0.152040469143052	0.616330822428300	-0.0926862733031925	0.877797216649453	-0.390142666666666	0.0931266433990784
SKIV2L2	0.151963302296106	0.616580957362935	-0.26959070488671	0.532107940833389	-0.0420733333333310	0.873454962131763
DLX1	0.151962819599431	0.616580957362935	0.424328204341353	0.216714808349232	0.317500666666667	0.121660357743656
PMS1	0.151895452406375	0.616735751069532	0.156932790844937	0.768401006769299	0.056151333333334	0.814956840733813
MAP2K7	0.151867155370978	0.616738274002298	0.39013806255803	0.282383744168943	-0.0238089999999989	0.934358485610289
EPS15L1	0.151816079927191	0.616738274002298	0.126268631426230	0.822164115743016	0.0161846666666672	0.95813639610796
SPTSSB	0.151764813122680	0.61684568978199	0.0058041666336819	0.99251028338798	0.0284116666666671	0.922463263763
AMT	0.151732761604124	0.616978178309509	-0.0246047866143317	0.973437611029098	-0.0074419999999992	0.98647849803298
TK1	0.151721558024878	0.616978178309509	0.220628516218259	0.639101135191794	-0.0473506666666652	0.849168149250465
PISD	0.151641008865624	0.617297279490192	0.071780716532733	0.90837606217316	-0.269696666666665	0.206251535561712
SKAP1	0.15163959709766	0.617297279490192	-0.520638530608366	0.0891512747500705	-0.0599649999999993	0.794486775271372
SPHK2	0.151619087833107	0.617297279490192	0.247554580329952	0.579745218830252	-0.102509333333333	0.622282705325595
SYTL1	0.151615311473652	0.617297279490192	-0.229812639349026	0.619381301776937	-0.0905013333333336	0.696927295330661
RNGTT	0.151603749782863	0.617298632369356	-0.0856320174037935	0.889156172508374	-0.760790333333333	0.00554643298010231
C1QTNF6	0.151563835356749	0.617409641733354	0.406991296222526	0.252941668074154	-0.113423000000000	0.575478632821557
ZMAT4	0.151462346053149	0.617599953100547	-0.264821005450623	0.542751754568497	0.182097000000000	0.342174534930341
DENND2A	0.151461741531354	0.617599953100547	0.136320082600947	0.804864448119512	0.357765000000001	0.151589765734136
ASB17	0.151453355669525	0.617599953100547	0.396540592351589	0.270980774578173	-0.00491100000000003	0.991175862027014
RIMKLB	0.151443543446835	0.617599953100547	0.170789499407554	0.741154777036791	0.149312333333334	0.523041310312964
ACTN4	0.151413470733915	0.617720725031559	0.0145065667617852	0.983444353656142	0.0308400000000004	0.912694388625723
GDI1	0.151301389258613	0.618297351144506	-0.225080079971185	0.63103711743427	0.0779300000000012	0.716517864791776
HES5	0.151264651960060	0.618305183665185	0.0300690147549878	0.966396286514145	-0.123899999999999	0.653123035053983
CDK2AP2	0.151255271951925	0.618305183665185	-0.214127018763619	0.653042977738486	-0.067373999999999	0.770261991339376
ERCC6L2	0.151215329195696	0.618413429555568	0.109938689530882	0.851013616427418	0.113137666666667	0.612697324680132
C20orf144	0.151202992053704	0.618419848293347	0.266359607403977	0.538833471023229	-0.127164999999999	0.624385634780367
PRSS53	0.151139211782757	0.618611823101421	0.11260870637905	0.847191484945575	-0.0990806666666667	0.733650645007746
MTHFD1	0.150992220063067	0.61926765838522	0.108846105354168	0.85348092441742	-0.130580333333333	0.57369089963002
ZNF461	0.150780810225001	0.620331763110547	-0.432307559915781	0.203737281652642	0.0114903333333342	0.97057157918193
TMEM39A	0.150770726920774	0.620331763110547	0.00919839227728687	0.98909166696601	-0.0209553333333333	0.945900449997854
SMARCD2	0.150703035056777	0.620475961349387	0.216061200339252	0.64844945813838	-0.190442999999999	0.321128515998283
LOC100294362	0.150653419287574	0.620708590237325	0.370842071764122	0.320399684386315	-0.104326333333334	0.618535434799748
MOB2	0.150605663562253	0.620708590237325	0.303405584520662	0.461534675086054	0.0839733333333334	0.705010402761235
SFI1	0.150601473379014	0.620708590237325	0.114025810407463	0.844272085764738	-0.0424919999999999	0.859420591955611
PM20D2	0.150547654204096	0.620877859853193	0.321423934905955	0.4207907841056	0.0390826666666678	0.880427463662501
GOLPH3	0.150543572492613	0.620877859853193	-0.0343181795443375	0.959581037407681	0.0781943333333348	0.716268843468132
GPS2	0.150505387652710	0.620877859853193	-0.141384557922275	0.796228722824663	-0.0587159999999998	0.814223077858884
FASN	0.150489494996104	0.620877859853193	0.504676737897744	0.106882834148277	-0.444878666666667	0.0649438674882112
PRR7	0.150481841445789	0.620877859853193	-0.0203978440519846	0.976452505880603	-0.0705413333333326	0.747694872257288
SMC5	0.150451487766904	0.620877859853193	-0.223279916114251	0.635113856809423	0.0970966666666682	0.635069698575895
EMCN	0.150428865949326	0.620877859853193	-0.165403465594832	0.752352242644389	-0.0312956666666668	0.922697964464038
EGFLAM	0.15042721469344	0.620877859853193	-0.28501151717182	0.49838641873573	0.339638666666667	0.520709741182581
BTN1A1	0.150348875047454	0.621210122502865	0.086950666991109	0.887065140370096	-0.165397333333333	0.40313895940962
GFPT1	0.150191625560293	0.621380473305381	-0.151078667749046	0.778007072419094	0.124357333333334	0.58391025729515
TBX20	0.150179361122143	0.621380473305381	0.0732835169957741	0.90588212168674	-0.184248999999999	0.48914578279316
NUDT13	0.150178887281875	0.621380473305381	0.224976960266538	0.631232672483489	0.0658466666666668	0.817550597555287
FKBP9	0.150171767220275	0.621380473305381	-0.177283896688723	0.728735147897621	0.410255000000002	0.120319574336004
AP1AR	0.150162109490205	0.621380473305381	0.0488581556878288	0.942180113539637	-0.0963813333333333	0.695654544311453
RNF40	0.150161997800187	0.621380473305381	0.238889343866482	0.600356188404063	0.00250333333333448	0.993756838831997
GNA11	0.150155123899069	0.621380473305381	0.372532749844455	0.317268019532456	-0.160797666666665	0.581811561322474
RSPRY1	0.150092874409047	0.621563196328304	-0.0398453377825081	0.953601323357358	0.066487666666668	0.790199988587959
SEC31B	0.150020596935292	0.621956881651606	-0.0344151632796023	0.959404955370931	0.0103470000000007	0.973686596735315
NUP54	0.149997300510736	0.622023201358892	-0.253355044248233	0.567705016149998	0.138522333333335	0.512869954128574
MEF2D	0.149943668127717	0.622110069797968	0.151236586858046	0.777604486471648	-0.0190403333333326	0.947455054282258
AKAP13	0.149896785691272	0.62223852646853	-0.18479767385395	0.716020884288485	-0.0681516666666655	0.791483318485292
ESM1	0.149886470404724	0.62223852646853	0.279790272207221	0.508836091265152	-0.0474800000000001	0.838927397112197
MARK1	0.149837580055357	0.622408177543805	0.134775258736973	0.807867938002244	-0.154480999999999	0.614221661998915

RNF8	0.149715358921921	0.622978558838723	0.219566761166142	0.641226345931986	-0.0492413333333328	0.863608092379234
EIF2AK2	0.149657584063542	0.623234939232207	-0.0138124893948393	0.983872723592937	0.0785343333333345	0.712110423183083
NT5C1A	0.149616491220262	0.62337281336052	0.24785340102274	0.579471329472972	0.016076333333335	0.954180927004992
FLT1	0.149609105445711	0.62337281336052	0.304474714443169	0.459334784763935	0.574971666666667	0.191847341402838
SET	0.149569004984476	0.623398195242054	-0.0301280070581786	0.966396286514145	-0.00047333333333335	0.998309716374356
SEC14L2	0.149552716605260	0.623398195242054	0.271428006458811	0.527984832801825	0.0481710000000002	0.893395132456423
TTBK1	0.149550932671646	0.623398195242054	0.177397244475948	0.728541130314952	-0.142526000000000	0.603978635563313
HEXDC	0.149464602014058	0.623614519145274	0.271610702858117	0.527940744091675	0.0567010000000005	0.849986993244602
ANGPT2	0.149433880696524	0.623628249691548	0.2565776112261	0.564398995459794	-0.129773666666666	0.595150543243344
RFX4	0.149416533649063	0.62366730080893	-0.226950455033975	0.625822145497039	-0.0521546666666663	0.879459918903629
GIPC2	0.149333316052742	0.624059027472953	-0.0143028287488610	0.983444353656142	-0.144414333333333	0.50103373645898
HS6ST2	0.149253648072603	0.624195775621733	-0.225068600056195	0.63103711743427	0.161023000000001	0.514420666507349
KCNN3	0.149205705682221	0.624195775621733	-0.153620740047421	0.773358453580566	-0.122020333333333	0.591037690220586
HERC4	0.149198583301168	0.624195775621733	0.150459912871966	0.77904503981756	0.0774516666666678	0.727065902741195
EIF2AK3	0.149194735914619	0.624195775621733	0.135800305369755	0.806188297553807	-0.0311483333333334	0.914034671247973
AURKB	0.149182494699689	0.624195775621733	0.0980089466098805	0.868852648210739	0.0812833333333349	0.725467874951849
SERPINE2	0.149132993290928	0.624227969966325	0.116705366823330	0.841809730226979	0.217545666666666	0.364895038300859
MKRN2	0.149126398510965	0.624227969966325	-0.233972611438989	0.60951860401502	0.0100290000000015	0.974097828643436
TYR	0.149086050025667	0.624263872564908	-0.00477317147464224	0.99311930576133	-0.0388428333333327	0.903274690789156
OR5E1P	0.149064389246013	0.624263872564908	0.140264734689033	0.797441782082887	0.156871333333333	0.446476080586417
SIRT1	0.149052298121054	0.624266251251278	0.0109155290306763	0.98774587825743	-0.0581696666666656	0.808511988397911
HJURP	0.149017429943597	0.624348473583563	0.110328880391149	0.850086645205643	0.0498140000000005	0.843840511995531
ASMTL	0.148913042228100	0.624690885013889	-0.0341132444480971	0.959808677091882	-0.0361591666666664	0.888285531317364
CDC7	0.148903394196769	0.624690885013889	-0.0254890809266549	0.972182886218393	0.0976213333333346	0.637530761419601
CHRM2	0.148888655862526	0.624690885013889	-0.00136294419501795	0.998320756202693	-0.113801333333333	0.572259462311624
ZNF451	0.148885428896758	0.624690885013889	0.0997846617873968	0.867558388686851	0.0576183333333332	0.813694870801638
SMCR8	0.148828854043079	0.62497942525456	-0.280533547944264	0.507460240190768	0.071538000000001	0.748989588811491
DCTD	0.14879977722534	0.625013161541119	0.0759535400471016	0.903510413986113	-0.00744233333333318	0.982523503616988
SMPD2	0.148790413496239	0.625013161541119	0.273261147799914	0.523997406191732	-0.178007666666667	0.48608244495005
ZWILCH	0.148749198016456	0.625133675536941	-0.0957574815563796	0.872537273851412	0.135747666666668	0.5530742296054
PEX10	0.148715889399767	0.625276003136465	0.0143132432315465	0.983444353656142	0.0867303333333341	0.693735487656277
CD82	0.148688431194021	0.625380538228599	0.126670773979209	0.820958242958398	0.0221140000000002	0.962552669872023
RPAP2	0.148506859848274	0.625887933920239	-0.228315379226417	0.623485483826317	0.156785000000001	0.441460337585051
CCL2	0.148376932275377	0.626374475785591	-0.255962139092492	0.565182535996794	0.146640000000000	0.549659494702918
LINC00518	0.148376754567781	0.626374475785591	-0.0225978153541058	0.97536722461972	0.0502703333333333	0.843152489417868
KLHDC8A	0.148220294090834	0.626941214033807	0.167428351654214	0.748960978799932	-0.242245666666667	0.361971299676959
ZNF134	0.148210176067921	0.626941214033807	-0.195496019039452	0.694043352975239	0.0293556666666675	0.91895269126233
STARD7	0.148184430067368	0.627034800812858	0.0530276655484463	0.936712858731269	-0.00548666666666539	0.986688186746568
EHHADH	0.148155018616505	0.627152098732504	0.363798495525478	0.335033160801809	0.0810786666666671	0.737850396206001
PXK	0.148120593277654	0.6273018346875	-0.189818415659616	0.704724567131716	-0.355828666666666	0.101980812453755
SLC5A12	0.148067914232066	0.627350927311438	-0.260121949994408	0.554748923785473	0.201131333333334	0.4261850174154
TAGLN	0.147952460920735	0.62790795326833	-0.00548425572011077	0.99251028338798	-0.0308649999999996	0.922697964464038
BAK1	0.147786089333086	0.628736899388862	0.210757094880973	0.661480633411348	0.0104620000000009	0.974895360062947
PCSK4	0.147729705833294	0.628981844282479	0.339400206709049	0.381991773838655	0.0435733333333341	0.891719978528798
C3orf65	0.147710376324659	0.628981844282479	0.223659656163040	0.634080386820485	-0.0732633333333328	0.754015160951226
PLSCR1	0.147677376376506	0.628981844282479	-0.296011375145277	0.47720428750326	0.579133666666668	0.0326806169766676
HNRNPA3	0.147668912737207	0.628981844282479	0.440088918697914	0.189842128278606	-0.0842555555555544	0.706965493695243
LEKR1	0.147627785747008	0.628981844282479	0.113323902271668	0.845747219093509	0.0598973333333341	0.786118160890663
ST7L	0.147625379373812	0.628981844282479	-0.151929446478301	0.776173016352835	-0.0337969999999989	0.942092082409632
SMC2	0.147619323302660	0.628981844282479	-0.089479739789312	0.883382963653617	0.146783666666668	0.492720036901389
KIF15	0.147587757273334	0.62899941584135	0.0499603157873692	0.941458038227918	0.181173000000001	0.520424642482869
ZNF787	0.147537808186078	0.629176891326632	0.498170304586322	0.114179995542754	-0.101633333333332	0.684569287653569
KLHL20	0.147383756042025	0.629882568684922	0.0605973968818717	0.925462896587765	-0.118639333333332	0.565864537714407
GUCY1B2	0.147363283080318	0.629942198633647	0.0525656350221296	0.937577440290918	0.0283026666666673	0.911242495488915
AKR1E2	0.147288733928017	0.630206156740303	-0.179745445950262	0.724074699453211	0.129762000000001	0.639837290428782
MAPK8	0.147228460331625	0.630450657411101	-0.0698576034084836	0.910885619751085	0.149855333333335	0.510927443048013
GAS1	0.147213096019574	0.63045132863169	0.0932559903496407	0.876856029801538	-0.0318686666666652	0.916575601310512
CAT	0.147089400886036	0.630991198110116	0.49965839888075	0.113777333040600	0.230372000000001	0.233528575266801
CTAGE5	0.147087903327269	0.630991198110116	0.553137445011699	0.0590850541024495	-0.96196	0.0216138709400044
ZNF416	0.147071737901679	0.630991198110116	-0.220602153260456	0.639149853200083	0.129317	0.576277303044571
NYNRIN	0.147051433995380	0.631013816997919	0.21095875716785	0.660645714735488	-0.00580333333333344	0.989265037176288
GUCY1A2	0.146975625480812	0.631193700472689	0.0642398646963865	0.919923865590619	-0.416987333333333	0.0631498594066912
DIRAS3	0.146898567053867	0.631566619423342	-0.159718224381029	0.763150272733332	-0.075040999999999	0.728911421670294
SAMD15	0.146790640905409	0.632193347030908	0.0576750023475451	0.929758936765496	-0.338921	0.099045346265795
MAGEA1	0.146746237852314	0.632260753923615	0.155676600476396	0.769837060156515	0.0914746666666675	0.667732731151074
RBMXL2	0.14669378645185	0.632383244620778	0.0911765566974062	0.880644955971727	-0.0750446666666658	0.830147563067484
SLC44A1	0.146668820296679	0.632399113025738	0.0917215849870098	0.879153312143409	0.2820710	0.137706210508003
HOXA4	0.146643139789843	0.632492634477969	0.224919564814433	0.631366594890923	0.127312000000001	0.536063442230912
TNFRSF10C	0.146599432238019	0.632557058296392	0.0920137567956879	0.879153312143409	0.107626666666667	0.6034967860712
TPX2	0.146571080522604	0.632625755174628	-0.0397600190518455	0.953647040142798	0.0227886666666671	0.944735546098178
KIF5B	0.146566326098954	0.632625755174628	0.179405855478584	0.72498617240364	-0.00956333333333161	0.974451420920923
sept-09	0.146478598417606	0.632975700196517	0.0675916278888268	0.914061739278386	0.112222666666668	0.597274793991978
DAZAP1	0.146369463483846	0.633194610632074	0.0966224359086728	0.870857630743202	-0.107415999999998	0.613258123073115
TEF	0.146344680594364	0.633194610632074	0.401086913620247	0.262127055693923	-0.189280333333333	0.330658906409802
SMCR5	0.146285809244355	0.633423304759469	0.247153870135874	0.580399307299869	-0.0425306666666663	0.922148106708426
PCDHB9	0.146162736434929	0.633809672338152	0.174222885362385	0.734267769889068	0.101852333333333	0.671766969198954
PCDHB6	0.146161295198869	0.633809672338152	-0.288459339187724	0.490230108819486	-0.212815333333332	0.266979834423163
PURB	0.146152636907603	0.633809672338152	-0.0952159613963026	0.872962271153999	-0.744531333333332	0.00964046731120165
ABCA2	0.146147504612146	0.633809672338152	0.113971039277623	0.844272085764738	-0.114697999999999	0.621638405081042
PRCC	0.146019473273313	0.63442139889097	0.187473386860621	0.709286896761914	0.0616193333333348	0.77895955075587

CSRNP1	0.14599584312209	0.634428771922548	-0.275038651846587	0.51990180188597	-0.326438333333333	0.144743493582474
SLC12A3	0.145808588006639	0.635065127924286	0.061624136940017	0.923246185283376	-0.00984499999999981	0.974451420920923
DENND4B	0.145807825709355	0.635065127924286	0.087612540604348	0.886302037299497	-0.280226666666666	0.182794399904883
CEACAM6	0.145743462634318	0.635294615551954	-0.0838089195703244	0.892833228561937	-0.0490523333333331	0.842903889380894
SPHK1	0.145738740041186	0.635294615551954	-0.094480336632529	0.874384897652873	0.0811536666666679	0.82138507507873
RASD2	0.145609971999188	0.635838647747924	0.162835074362435	0.757151893827501	-0.247739333333334	0.313220411537404
HUWE1	0.145582231306939	0.635945778164334	-0.249549860341965	0.575370998761354	-0.0468136666666653	0.883913953154694
TGM2	0.14556801968745	0.635965056768643	-0.0413758308531440	0.952098348256047	-0.0283989999999996	0.91143191097679
RNF175	0.145522890390360	0.635966131630973	0.0348191728487882	0.959404955370931	-0.0050033333333331	0.98893091110662
IL17D	0.145280167419881	0.636818071928564	0.114046495641176	0.844272085764738	-0.119593999999999	0.712653771000543
SLC1A5	0.145248586927965	0.636845582414223	-0.355150577599454	0.347834825198038	-0.0353436666666663	0.888476605628154
EIF2A	0.14523642564444	0.636845582414223	0.144976642566379	0.789227217144616	-0.0769566666666657	0.798151134664617
PLXNA1	0.145219395507122	0.636845582414223	0.368848756422928	0.32362669860138	-0.151872333333332	0.503308350574223
ZNF671	0.145207678059418	0.636845582414223	-0.216790491600512	0.647357580911858	0.147325333333334	0.580149967309238
SETD1B	0.145077367542845	0.63722527836202	-0.0871605590522198	0.887055939766881	-0.0287706666666659	0.910663650054071
KIAA1468	0.145005453067800	0.637351883323136	-0.0296586232173160	0.967094033447048	0.0100833333333347	0.974895360062947
DCAF13	0.144972759795651	0.637351883323136	-0.172847489738660	0.737853882640005	-0.0888033333333328	0.700976399025928
C3orf36	0.144972512113210	0.637351883323136	0.221987680683930	0.636647953876148	-0.0470923333333333	0.855240892932328
FZD3	0.144891380945812	0.637582792716122	-0.0397599257676134	0.953647040142798	0.104315333333334	0.74212040995637
SLC22A11	0.144887021216609	0.637582792716122	0.259731364319823	0.556147580300637	-0.0446096666666657	0.85184175589894
TNFRSF10D	0.144867118045852	0.637582792716122	-0.219106872866574	0.641699000980579	0.125661666666667	0.583036318848746
CNGB3	0.144847441579714	0.637582792716122	-0.0509719628949542	0.939617217812417	-0.0131859999999994	0.969104903637376
WIZ	0.144841302563415	0.637582792716122	0.261224516639418	0.552474056735769	-0.0959069999999995	0.670071773006358
ANP32A	0.144803594802100	0.637582792716122	0.163574330576937	0.755095478684029	-0.0902198333333315	0.6846855637534
PPP1R26	0.144761325374343	0.637582792716122	0.0792713959629672	0.898424765944956	-0.0232129999999991	0.928897428735123
C15orf60	0.144761127279830	0.637582792716122	-0.121025282332952	0.83275349524941	0.136493666666667	0.583708661347349
PLEKHO1	0.144746230589072	0.637582792716122	-0.386097864137512	0.289470192870154	-0.0619296666666666	0.791240721091965
C20orf195	0.144682112821109	0.637841947468087	0.00548618932488807	0.99251028338798	-0.0587609999999996	0.849384482495249
HOXB8	0.144652571422309	0.637841947468087	0.213391008254426	0.654550434157114	-0.264412333333333	0.309421300309741
UMPS	0.144633769990123	0.637841947468087	0.202692898045313	0.678079790105593	-0.105500333333332	0.637570808030689
ZNF302	0.144490240642106	0.638450070139276	0.318007345411812	0.430156985569412	0.196048666666667	0.320225451789804
TFAP2A	0.14448153467772	0.638450070139276	0.345186324159129	0.368530625231133	0.141962666666667	0.489894482040166
ZNF185	0.144478095001441	0.638450070139276	-0.348688464543714	0.361261311685225	-0.0559826666666662	0.837732816472933
BEST2	0.144464335188406	0.638466636239189	0.079356284390175	0.898323801514594	0.130746666666667	0.629657237462109
PRRT2	0.144432397870426	0.638570340856502	-0.0208825776649632	0.976330597778846	-0.112140333333333	0.639837290428782
C12orf74	0.144403534432899	0.638570340856502	0.0941000656695773	0.875126060855988	-0.197478666666666	0.512796060754107
PLK4	0.144374410035212	0.638613932111792	0.107433747223873	0.857065724607536	0.195353333333334	0.398554167047557
PNPLA3	0.144279179293629	0.639013963705338	-0.152969577621429	0.774642035123462	-0.0828046666666665	0.771941283177241
GRM4	0.144231927258632	0.639029815337622	0.115075838333806	0.844120246770255	-0.0317203333333323	0.925476944639214
RAB5A	0.144137157395527	0.639426951463028	-0.175497025165964	0.73194983434922	-0.000646666666666558	0.997953056544486
LYPLAL1	0.143993718319596	0.639873758284402	0.240963288252843	0.596648444172109	0.0206230000000007	0.95417877753931
sept-02	0.143987125538464	0.639873758284402	0.227226787472691	0.62581495163125	-0.0388996666666658	0.873663396186202
ALS2	0.143973589662800	0.639873758284402	0.231822818918342	0.613578980603213	0.0390853333333344	0.902744074046491
ANKRD11	0.143954971590591	0.639873758284402	-0.0519321869911318	0.938067483409541	-0.0594032499999981	0.808253941454262
ARPP19	0.143946278443285	0.639873758284402	0.00473441253234318	0.993222959527385	0.0847145000000002	0.688145991467449
PLEKHB1	0.143913742502543	0.639974227146072	-0.0458531642659937	0.945372627844176	0.00067533333333380	0.998281147912498
TUBG1	0.143907303209684	0.639974227146072	0.115403045644896	0.843658890260551	-0.0061233333333336	0.985263856374185
RIBC2	0.143840857007231	0.640333048390846	0.107329887134553	0.857128584932911	0.0198543333333341	0.94678260229888
ZMYND15	0.143729197604000	0.640767484378041	-0.0367719440775539	0.956918184553224	-0.129932666666667	0.546434057395997
PRKX	0.143649911900531	0.640912233896752	-0.376271989989565	0.309734308429584	0.0708566666666678	0.765276224002577
CNST	0.143544061719501	0.641088939967479	0.147884852760135	0.784090188380266	0.316912333333335	0.133094907391771
ISLR	0.143527985737481	0.641088939967479	-0.0212100592367594	0.975853964465421	-0.0749923333333333	0.778995157976597
ZSCAN12	0.143494700766970	0.641088939967479	0.341306656476502	0.378027240426398	0.121676333333333	0.626064244773985
PCDHB15	0.143457085689191	0.641088939967479	-0.175083645355794	0.733269860474173	0.351628	0.103301877951436
ROS1	0.143454310824102	0.641088939967479	0.0876660496459795	0.886302037299497	0.0428503333333335	0.863941738814148
C7orf31	0.143445853093652	0.641088939967479	0.00814264743048309	0.990248011892148	0.330993000000000	0.165619554708818
INTS2	0.143440912374276	0.641088939967479	0.132773420540108	0.809588765729788	0.104576000000001	0.635267430402483
XYLT2	0.143425841529079	0.641088939967479	0.279368724375336	0.510154465721482	-0.295918666666666	0.135327979780793
C17orf80	0.143407476821134	0.641088939967479	-0.0278434757391158	0.969317858168394	-0.0376786666666664	0.892921857967499
PYGO2	0.143405109570076	0.641088939967479	0.344220272146192	0.371106177422032	0.0684013333333335	0.779683585417535
EIF2S1	0.143279918539456	0.64140140648204	-0.0886795740101282	0.8841943128258	-0.289678666666666	0.150274389990121
FAM150B	0.143272342646666	0.64140140648204	0.315964411794778	0.433782024195460	0.000444333333333446	0.998351142601927
MTUS2-AS1	0.143225898655780	0.64140140648204	0.158610337732304	0.765791046741822	-0.199368333333333	0.306286104860031
TSPY1	0.143192128819635	0.641495485592949	0.0432948365355152	0.94995273206261	-0.0220801111111101	0.938282767814398
ACACA	0.143152727883057	0.641629352954776	0.400555884781714	0.262685489461853	0.0915280000000015	0.704370299831112
GRIN2C	0.143136716518990	0.641629352954776	0.458968815652509	0.165076020821271	-0.0784649999999994	0.794102100657862
NCOA4	0.143081516548461	0.641629352954776	-0.0868165043695526	0.88734162241363	0.0253973333333346	0.924674647382585
NMD3	0.143066512564017	0.641629352954776	0.031129387542753	0.965118873711308	0.0144353333333346	0.969387309763954
CCDC19	0.143026341565189	0.641629352954776	0.145664403301595	0.787804113309913	-0.169836666666666	0.431802976936949
TMEM25	0.142943013487714	0.641985729056844	0.201282838710098	0.680054545720343	0.185367000000000	0.361781227674926
FUBP3	0.142937936941906	0.641985729056844	0.0455691682186167	0.945769054147694	0.0200116666666677	0.941975814538135
CSNK2B	0.142825069347537	0.642500985447723	0.218239244942730	0.643344904659891	-0.0475024999999987	0.838927397112197
DIAPH3	0.142681861798687	0.643286004311135	0.0299075158208381	0.966686671402086	0.293724666666667	0.163725198409623
C3orf27	0.142511173229516	0.643959768377052	0.249523156137564	0.575370998761354	-0.145247	0.498454352317107
AGL	0.142464653663544	0.644189549520358	-0.276143176795932	0.517549833373363	0.0935556666666677	0.68641735550035
CAV1	0.142399640954129	0.644466995517224	-0.293190782312018	0.48133638278712	-0.0245069999999994	0.931494545972758
CCDC111	0.142358044665740	0.644483428214332	-0.079399278946814	0.898323801514594	0.324054333333334	0.126863181258387
MTMR4	0.142349050469466	0.644483428214332	0.191879158384504	0.701479891661163	-0.278747666666665	0.190569948330175
HMGN1	0.142318935596383	0.644483428214332	-0.0251615727133674	0.972358966414305	0.0348783333333345	0.891410328517388
FOXJ2	0.142287630799544	0.644483428214332	0.246674309515409	0.581438939050387	-0.232816666666666	0.245623058897326

KRT6A	0.142268443399986	0.644483428214332	0.0753399380002263	0.904120140003257	-0.098677666666666	0.644175841365564
AP4B1	0.142265088795082	0.644483428214332	-0.305923897193330	0.457192231066199	-0.554079999999999	0.0802890385571452
C21orf91	0.142134237733899	0.644741287801228	-0.221417802721897	0.637715957775489	-0.299057	0.267773920477442
GRP	0.142099228635836	0.644822942358535	0.049050252541827	0.942126968886265	-0.180826999999999	0.496462982918528
ITPKB	0.142088213494515	0.644822942358535	-0.0494893805145478	0.941686550321915	-0.0300876666666660	0.937717709824356
PABPC4	0.142057593645706	0.644863990753516	-0.0181221506145557	0.979375761502567	-0.0753389999999983	0.748388402768556
EVC	0.142047802444805	0.644863990753516	0.117115045159058	0.840993954510842	0.0349136666666674	0.908473154502781
VN1R2	0.141946672573182	0.645109323825881	-0.0887557565245441	0.8841943128258	-0.0213146666666663	0.952505889082874
AMFR	0.141905466738696	0.645109323825881	0.104334767246479	0.861150025460275	0.0446946666666674	0.861738550686595
C1orf180	0.141902234853769	0.645109323825881	0.139616930125980	0.797957231657366	0.0530733333333336	0.815309621716298
C16orf89	0.141877340796452	0.645109323825881	0.141248360333114	0.796285336930078	0.026000333333334	0.935223865987462
RMI1	0.141797186460066	0.645554953446382	0.209570832050857	0.664094875644384	0.0389166666666677	0.879610782084904
DONSON	0.141695866011701	0.645786074205067	0.148032377180198	0.784080588385358	0.224392666666667	0.234227600059511
LINC00308	0.141685816667997	0.645786074205067	0.0890334475393047	0.883824851493414	0.0328240000000002	0.904236796123256
ZNF548	0.141628775216042	0.646070041681342	-0.0456511890012634	0.945663463098983	0.0750446666666678	0.742840137118157
CGREF1	0.141552103204444	0.646278711344502	-0.285924853905264	0.496429042723156	-0.274957999999999	0.217068641960933
IHH	0.141491915931314	0.646525075175036	0.225493586384325	0.629710977741725	0.0568286666666676	0.871104061464043
CR2	0.141466632161047	0.646617007481215	-0.0296134343556853	0.967205787110068	0.0291633333333337	0.919753903246749
RCN3	0.141397474329840	0.646921736406921	0.0987148354205768	0.868042734188964	0.00682766666666628	0.98349947509937
DNAL1	0.141375363137503	0.646969381693755	-0.152740175915630	0.775138534286213	0.131243333333334	0.592716584553539
IFI27L2	0.141356729009877	0.646969381693755	0.456627646340627	0.16754724558121	0.0748436666666672	0.791599529156989
THOC2	0.141285881813983	0.64728335076677	-0.222273368703717	0.636309362271551	0.0899466666666679	0.671492528470281
CEBPD	0.141275020942745	0.64728335076677	-0.0789295302060321	0.899154242391172	0.0771490000000003	0.757990235154062
INSRR	0.141254270976532	0.647345848901796	0.134986010578725	0.80783889218961	-0.138081333333333	0.484446864118853
COX7A1	0.141118140050337	0.647796540688829	0.00876068588484225	0.98946753270812	-0.0760943333333326	0.826922793139517
VPS45	0.141068790734586	0.648040662604292	0.0570773574730649	0.930775493791569	0.120005666666667	0.549323798856043
SCYL2	0.141042894445253	0.648065332151138	-0.0448065848873490	0.947571875928317	0.0166730000000003	0.95285450244329
BCL2L14	0.140952115525601	0.648296979316259	-0.057656881140626	0.929758936765496	-0.0179819999999999	0.954021566744527
OVGP1	0.140908198122002	0.648440657327192	-0.0142862224061345	0.983444353656142	-0.114037333333332	0.611770847276966
NAT8L	0.140903154488939	0.648440657327192	0.028647338216274	0.96782691865619	-0.151282666666666	0.528409919089401
GLS2	0.140886441092247	0.648440657327192	-0.186911084052218	0.710476504107514	-0.0754076666666662	0.73481029572636
SLC35F3	0.140874366924028	0.648440657327192	0.111181348149030	0.849127967004266	-0.0601453333333327	0.840247972976129
POF1B	0.140732810397171	0.648852011402849	-0.167518397947549	0.748960978799932	-0.0936066666666664	0.68400971768044
MAP4K3	0.140725156344067	0.648852011402849	0.213527194866293	0.654198328177448	0.105255666666668	0.635069698575895
SNX1	0.140722089769500	0.648852011402849	0.156405288473631	0.768909201237015	0.133285666666667	0.547305665058354
OLFM1	0.140694542001103	0.648886455532056	0.098794076412385	0.868042734188964	-0.355697333333333	0.204005720059480
PTN	0.140676062561653	0.648906032795245	0.266411300386454	0.538833471023229	0.273578000000001	0.174033593336554
FAT1	0.140654460187497	0.648906032795245	0.181177325298587	0.721382789731275	-0.118718666666666	0.597274793991978
COMP	0.140650781860957	0.648906032795245	0.209350028422793	0.664703270325548	-0.190386000000000	0.419320988334059
KCTD12	0.140647037048626	0.648906032795245	-0.442456616620059	0.185282268884974	-0.177736666666666	0.427283471646461
UBE2J1	0.140603153873302	0.649119065018148	-0.0063276900682509	0.991853352023739	0.162994333333335	0.428953946992621
PPM1D	0.140574912803828	0.649230392205041	0.0783131789582599	0.89954686796206	0.253390000000001	0.174063898659918
PARP14	0.140530816459421	0.649308707427012	-0.435242961862762	0.197011021667727	0.169511000000002	0.393524204428715
mars-02	0.140474505955849	0.649485591155604	0.244274385407360	0.588630958916351	-0.301755333333332	0.181734378040088
JPH4	0.140457825737458	0.649485591155604	0.285399413464687	0.497701988631165	-0.094555666666667	0.77895955075587
FNBP4	0.140369756349153	0.649841432917991	0.245232544968667	0.585635671087268	0.113059000000000	0.61621014110361
RTN4RL2	0.140318274823390	0.649901107789831	0.0479359909428808	0.94314236584312	-0.154639666666666	0.552298643114782
C11orf87	0.140298032975372	0.649901107789831	-0.0642057855343869	0.919923865590619	-0.127412333333332	0.520709741182581
KLK5	0.140267814992490	0.649901107789831	0.0907177232961372	0.881323965029954	-0.200050666666666	0.44275000040488
CSRP3	0.140251947870383	0.649901107789831	-0.112002043560460	0.847604542536677	0.0973826666666674	0.64069646762551
FBXW4	0.140213024611708	0.649901107789831	0.497382277437643	0.114508695522455	-0.347114000000000	0.206909867235320
CASP8AP2	0.140204983603929	0.649901107789831	0.149693976460649	0.78143277453973	0.0485956666666674	0.85655790966096
DLX6	0.140192926557869	0.649907274105795	0.394734580588402	0.273836638126109	-0.0638260000000001	0.817083045365319
MASTL	0.140138028919757	0.650191969403441	0.105159824827507	0.8606258296021	0.247444666666667	0.320462604748547
CMIP	0.140053431263953	0.65060344582742	0.283678453002188	0.501852406168029	-0.300688333333333	0.249799910921436
AGAP1	0.140026326091506	0.650701664546099	0.431187453666437	0.205873334374071	0.0651336666666677	0.802470214652098
HTR7P1	0.139956822334839	0.650863362100047	-0.325133215663549	0.41045730743103	0.156167333333334	0.504170715090403
GIPR	0.139891980263084	0.650921837404149	-0.190449105505684	0.704011981544887	-0.217709333333333	0.313220411537404
ARHGAP6	0.139890254961826	0.650921837404149	-0.105644372327093	0.85956218633532	0.537554333333334	0.066097768061204
NME9	0.139881400494860	0.650921837404149	-0.0286026769856021	0.96782691865619	0.0301246666666676	0.904507944803624
TMEM204	0.139740428730168	0.651035132051217	0.163605597490815	0.755036658968701	0.0522396666666672	0.823061379322626
LOC728819	0.139692936085966	0.651035132051217	0.330637781399491	0.402284079938133	0.193042666666667	0.468377378486522
PLIN1	0.139673774315595	0.651035132051217	0.39064196549376	0.282005640075858	-0.126623666666666	0.711075151205956
LINC00242	0.139671430861473	0.651035132051217	0.0865636400483594	0.887493600482322	-0.0805503333333329	0.719304894613107
SPTAN1	0.139646687141138	0.651035132051217	-0.152648263911611	0.775258759376418	0.128420000000000	0.589687893028288
RABEP1	0.139622060612545	0.651035132051217	-0.212998310702135	0.655371496569188	0.02794066666667	0.911600329626215
PCDHB3	0.139619853165094	0.651035132051217	-0.255303605256688	0.566022340784541	-0.126931000000000	0.58391025729515
EPG5	0.139573587532120	0.651173977767592	-0.0808911392201952	0.896361013063453	-0.413816333333333	0.137297162355315
FAM120A	0.139557149365403	0.651173977767592	0.168172984126258	0.747936039510864	-0.0467478333333327	0.841174975800285
C8orf49	0.139554135021436	0.651173977767592	0.274251303071074	0.520857714835269	-0.00131233333333345	0.996285985364494
RNF165	0.139503422895803	0.651387844038414	0.124679929748334	0.82479991054473	-0.0772523333333328	0.734228369215232
TMPRSS15	0.139499049041917	0.651387844038414	0.0162815504370915	0.981980841998872	0.0930113333333336	0.698340448759983
KIF23	0.13945869413178	0.6514353909158	0.0831396645296452	0.893287218356307	0.152610000000000	0.492100665301584
HIST1H2AK	0.139456230832868	0.6514353909158	-0.13826254677339	0.800120930330156	0.0644760000000016	0.850897461752934
C19orf44	0.13944739230833	0.6514353909158	0.438971382430399	0.191636246400456	0.0998409999999998	0.635069698575895
NGFR	0.139387927033791	0.65173667313611	-0.204810843396275	0.673728362275223	-0.100186666666667	0.689267979966846
LRBA	0.139377794075689	0.65173667313611	-0.0486259063871642	0.94236319435931	-0.673481999999999	0.0169419371876331
OR5I1	0.139320947969674	0.651844538996729	0.0768703672267048	0.901856698918763	0.0747620000000003	0.766574854574416
PSMC3IP	0.139232667534832	0.65214027641347	0.240437539145122	0.597862631330416	-1.24255166666667	0.00295083342264811
MT1X	0.138821071461035	0.653994399226778	0.198253938471758	0.687317734296043	0.0800866666666674	0.772139923817113

LRRC4B	0.138707973529966	0.654467775823729	-0.0492865855881392	0.941842562680095	-0.0895993333333319	0.74687011643745
DDX3Y	0.138701713484338	0.654467775823729	0.090001816936521	0.882422779091356	0.0423503333333335	0.882517847404652
DR1	0.138687736095212	0.654467775823729	-0.165443274456867	0.752352242644389	-0.032001333333332	0.910625003107163
AXL	0.138593552558012	0.65487974852008	-0.251229455160123	0.572833531479119	0.316027666666667	0.0996515501712583
CPS1-IT1	0.138585286306345	0.65487974852008	0.253923979581639	0.567694846153007	-0.00576499999999994	0.987134800094338
SNRPN	0.138529376451204	0.654934455272035	-0.155035052724045	0.770747367396581	0.645132095238096	0.174063898659918
SLC25A18	0.138503306379696	0.654934455272035	0.0334051646981821	0.960784250392482	-0.0611376666666658	0.835884942206786
SYT15	0.138480216357461	0.654934455272035	0.115571161146284	0.843372362327421	0.0281883333333348	0.92484471634727
PPM1J	0.138463043784099	0.654934455272035	-0.170161982701004	0.74270446821332	0.0373206666666669	0.901863465434143
RFXANK	0.138427538087609	0.654934455272035	0.00343887283490901	0.994730189431381	0.072228	0.745245240629921
C8orf37	0.138421779382060	0.654934455272035	0.271729014876339	0.527608228552189	0.245010333333335	0.320875140358249
CDC45	0.138381310825688	0.654941206070384	-0.0325002956229245	0.961968358372373	-0.0805916666666662	0.704370299831112
PITPNM1	0.13836538280933	0.654941206070384	0.0564268656846195	0.931891677569814	-0.129581666666665	0.669530887411007
ZNF83	0.138189031997031	0.65558442673033	0.122132900221369	0.829990919492397	0.0494263333333341	0.836663486707778
HPS5	0.138149285894715	0.655626994541745	-0.142810296117984	0.794004622916083	-0.102499333333333	0.674531431277108
RALGAPA1	0.138094494690649	0.655875463183054	0.0823827793121097	0.894172508150787	0.367419333333335	0.158095862862508
SOX21	0.138088966549544	0.655875463183054	0.156366378783573	0.768909201237015	0.0432010000000001	0.9273350681791
SCAF8	0.138034150386461	0.656016131409846	0.134554757816306	0.808009477165608	-0.109495333333333	0.588052519326463
GABRP	0.137974506272051	0.656252692203676	-0.0743682147751289	0.904945422294164	-0.135227666666666	0.499554381454891
OPCML	0.137972044900931	0.656252692203676	0.197572877479187	0.688668717091024	-0.532792999999999	0.329321493912251
CNOT10	0.137931155607385	0.656252692203676	-0.200788899379831	0.680489843835436	0.0927193333333353	0.674780899769326
LEFTY1	0.137914458033855	0.656252692203676	0.275995882138241	0.517549833373363	-0.000870333333332083	0.99792238320329
FNDC9	0.137609237958282	0.657530768368233	0.138565230155069	0.799373390835754	0.0599296666666674	0.82667899725046
COL22A1	0.137577844753542	0.657530768368233	0.237723806781518	0.602664863375033	-0.0617219999999999	0.82844931076437
SLC22A14	0.137416694322548	0.658038000020068	0.0710740603103152	0.90935935829916	-0.0563159999999998	0.858560160567975
TRIM2	0.13739107390735	0.658114065674109	0.131230992242803	0.811695311619767	0.116154333333335	0.566075188962
ZFP36L1	0.137336741291129	0.658323869287807	0.186967389280242	0.710297140964086	-0.003614833333332	0.99106990984423
HAPLN3	0.137212139062349	0.659063573412741	-0.0137977887222328	0.983872723592937	-0.106520000000000	0.610847413222267
FLJ32790	0.137178785022577	0.659184368178927	0.208126350049504	0.667327688576615	-0.221676666666666	0.338777202459218
TAMM41	0.137121295176041	0.659295169063327	-0.0892697603228892	0.883460564717377	-0.0868723333333325	0.741438256509195
C6orf123	0.137071632079967	0.659474671425858	-0.0468949094247854	0.944274559688373	0.117254333333334	0.555088011807105
LMF1	0.137021101611074	0.65948784249837	0.547729267272537	0.0620641062369145	-0.118148666666666	0.584434983272196
RASL11B	0.137018756373532	0.65948784249837	0.287068815216717	0.494441582955552	-0.0364786666666663	0.905071365396373
CTSB	0.137005561794723	0.65948784249837	-0.059177318355029	0.927427607950847	0.109038333333335	0.637596150841031
CHIT1	0.136977284550987	0.659585091173146	0.0443628255012714	0.948385499132766	-0.0770626666666662	0.823458541833548
AHSA2	0.136852822728327	0.660199557485678	0.264340247201302	0.544049010305410	0.263671666666667	0.162124453550270
C15orf32	0.136722780695560	0.660739581613824	-0.127941365898603	0.817799920299584	0.0818780000000005	0.771373465365664
IL37	0.136656163044534	0.660951643912699	0.133092583574106	0.80942029599089	0.0501933333333342	0.828768475477959
BOLA1	0.136643408334004	0.660951643912699	0.130297280571348	0.812271254601992	0.195462333333334	0.374814376822818
LYSMD1	0.136619052221054	0.660983399289496	0.233951205142295	0.60951860401502	0.186088666666666	0.410917713955087
CLP1	0.136607288243986	0.660988021467853	-0.142275618267964	0.79450771779567	-0.117096333333333	0.578893615511221
TBX4	0.136536740353268	0.661231809586843	0.162462782481399	0.757685565672998	-0.0750693333333326	0.7711825248022
APOD	0.136522848580919	0.66125031823698	0.213642924101522	0.653708954135733	-0.0780756666666662	0.794911513166032
WDR11	0.136476222365260	0.661434990553551	0.226086908004441	0.62834683241336	0.0196030000000016	0.94622978991441
OR8D1	0.136417168497106	0.661434990553551	-0.0223384975263147	0.97536722461972	-0.0755623333333328	0.754488794029141
CEP76	0.136360293422563	0.661733814611913	0.0616199302714485	0.923246185283376	0.092508	0.732554472781933
PROX1	0.136174826781636	0.662727515154804	0.185987580667036	0.713284034966074	-0.255413666666667	0.408178621977204
SCRN2	0.136163421638367	0.662729803398929	0.457096652546596	0.166847523900473	0.0474263333333338	0.857015626494513
KIF5A	0.136133722719928	0.662851492966799	-0.154050593252174	0.772253934458676	0.0858756666666667	0.745967084434416
FCRLA	0.136103761433167	0.662856821515552	-0.0854055993977654	0.889779510878572	0.0402346666666675	0.903788355106891
ZFP41	0.136099451422505	0.662856821515552	0.427155124511018	0.21257721492727	-0.112324333333333	0.616036866881905
ISX	0.136044990025146	0.663069929524939	0.0141896297065168	0.983444353656142	-0.0707453333333331	0.769890867417328
MEGF6	0.135882060022018	0.663695092511455	0.0403548129141776	0.952720585102517	-0.113285333333333	0.704305129962527
SLC6A14	0.135853417928452	0.663738842730623	-0.0764010304396754	0.902817525124169	0.0582973333333334	0.802984596560166
SNRPB	0.135843056974686	0.663738842730623	0.0637567817138401	0.920576523393395	0.0659710000000003	0.764848773122078
PCDH12	0.135767826423662	0.664157901041985	-0.22483626163947	0.631777342904209	-0.120726000000000	0.549323798856043
BEND3	0.135753040314709	0.66418228891167	0.086170798455132	0.888051139742697	-0.0211550000000001	0.941559580750404
ICAM1	0.135689589183996	0.66432746675533	-0.542961228980195	0.0666521078484436	-0.144555666666666	0.721454370213253
STAT2	0.135639278408242	0.664461591190621	-0.329904577897546	0.40362877792936	-0.102965666666666	0.655063476133482
HYLS1	0.135629470862542	0.664461591190621	-0.08185126820034	0.89548747312664	0.246241333333333	0.309421300309741
PARP2	0.135616377534878	0.664461591190621	0.0705730348179769	0.91009246060164	0.120054000000001	0.575560239919437
CLEC12B	0.135599780644873	0.664461591190621	-0.151926069729264	0.776173016352835	-0.0358893333333331	0.899372153567648
TNN	0.135583987008912	0.664492591672631	-0.102564987658470	0.863124341151509	0.0380693333333335	0.883878395614985
CD3EAP	0.135453351042352	0.665173972566108	-0.364936011723593	0.331455689303192	-0.182542666666666	0.410296457977492
NAA35	0.135388770093886	0.66537655626436	0.0532536230022292	0.936629055455007	-0.0618933333333325	0.777585466017059
TEKT2	0.135382360446462	0.66537655626436	0.083671428742057	0.892833228561937	-0.00167333333333309	0.99597023247862
FBXL2	0.135261249450156	0.665951341771818	0.0366102105909267	0.957172973051355	0.213988666666668	0.503352066997863
PLA2G4D	0.135011423857391	0.666934431077886	-0.000680915891327850	0.999016032077037	-0.349810666666666	0.0966783833067472
COPB2	0.134966772395642	0.667154162706185	-0.0540174569268538	0.935109055935003	0.0501366666666681	0.825729064695823
MYO19	0.134937776989598	0.667181635107337	0.380297267285986	0.300705577749487	-0.124150333333334	0.536063442230912
PRSS55	0.134852004453019	0.667471205445311	0.00307779541173348	0.994986311144058	0.0189376666666669	0.94678260229888
RSPO4	0.134781534761875	0.667643105752503	0.0182266461403150	0.979327298872732	-0.119721333333332	0.706965493695243
C8A	0.134721795364917	0.667939842453807	-0.115497656150556	0.843609735991731	-0.112648000000000	0.641044966033937
HOXC13	0.134689620835065	0.667984690516311	0.00277866037283523	0.995617423523752	-0.391566333333332	0.139567607482897
TIGIT	0.134604592710118	0.668401427830757	-0.341051791145296	0.378272273903863	-0.169706000000000	0.596924734519002
SLC26A1	0.134556669328926	0.668463745331396	0.230131863520442	0.618978609135107	0.0139543333333332	0.976702491879288
MIER3	0.134487981517285	0.668624120576638	-0.181872084200916	0.720096350499662	0.146853333333334	0.49971129098408
ZNF836	0.134412797326522	0.668971523119448	0.344069048690667	0.371548592536918	0.137787333333335	0.509677952600171
HIPK4	0.134360100152069	0.669244102622457	-0.0327161094764305	0.961351562552925	0.00519433333333409	0.988133186272453
VCAM1	0.134255133620545	0.669641989229582	0.088333715168879	0.885142944149088	-0.289495333333333	0.329156959330247

GPR132	0.134237256621306	0.669665192765859	-0.176835294654187	0.729794187782856	-0.0844689999999994	0.777406710539424
THPO	0.134129452707352	0.669954580770996	0.198763405351137	0.685648577129052	-0.0748096666666657	0.762981436878551
GUCY2F	0.134129040805801	0.669954580770996	0.204411023075721	0.674817810302676	-0.140503666666666	0.520245449442202
SLC38A3	0.134036539906867	0.670350104743365	0.140410403760976	0.79743101967094	0.0187920000000003	0.97002499251073
VSIG10	0.133922379089705	0.67088074482925	0.389706933722198	0.282848386985187	0.0289946666666677	0.919314110828047
ACPP	0.133838396182072	0.671286186904916	-0.219771458363799	0.641035290381005	-0.403808333333332	0.05368634135411
RRP1B	0.133814563998006	0.671369967468384	0.0329476140041007	0.961277493638149	-0.053277333333332	0.84100404224146
SENP6	0.133774326877655	0.671493586421994	0.266424716254277	0.538833471023229	0.190671666666668	0.397798624983652
SP4	0.133719555467845	0.671630678434077	-0.0199025618349475	0.977526645675126	0.139887666666667	0.588512630072945
IPO8	0.133626885064457	0.671947888808701	-0.151620073123502	0.777101208632856	0.0689346666666662	0.765339817431245
ESRP2	0.133615509764069	0.671947888808701	0.0154400997247667	0.982725338620588	0.00640699999999985	0.984646946725923
GALNT12	0.133601265027250	0.671947888808701	-0.124185880115551	0.825382714207684	0.167206666666667	0.43400381736769
CARS2	0.133593853898262	0.671947888808701	-0.264103594419513	0.544525995334757	-0.00119866666666551	0.996826292964745
DCLRE1C	0.133487894211168	0.672369160942727	-0.140604682400402	0.79686373498152	0.101842333333334	0.735665165720299
HSH2D	0.133485750453034	0.672369160942727	0.281045032606079	0.506886267079183	-0.150089333333333	0.513967209547063
EML3	0.133461897032840	0.672387426418417	0.102624058687093	0.862863830017748	0.00655666666666694	0.986480140342539
RPS16	0.133459094377800	0.672387426418417	-0.00354774977082511	0.994730189431381	-0.0321733333333321	0.903182673566864
CUL4B	0.133449500754340	0.672387426418417	-0.511353753748168	0.0987450924554118	-0.00531766666666517	0.987134800094338
KIF4A	0.133369259856285	0.672623931112915	-0.142729811016188	0.794004622916083	-0.00275833333333185	0.994738356997797
RXFP1	0.133296802364496	0.672737187795882	0.104698525527814	0.86067831050617	-0.00249799999999971	0.993869242746475
CREB1	0.133156731731590	0.673295072298334	-0.0209869583246832	0.976103466334817	-0.0521819999999996	0.870543028294187
KRT32	0.133123910727887	0.673295072298334	0.121908225216449	0.830496327847296	0.0379256666666668	0.904507944803624
NR2C1	0.133118357278848	0.673295072298334	0.190127583814859	0.704292435545605	0.349237333333334	0.0896797344789652
ENO2	0.133074968120530	0.673395602095796	0.157172671383286	0.76810607001971	0.0575056666666676	0.810423232842053
PRKY	0.133033522362344	0.6735331010416	0.120351671410250	0.834595811109828	-0.0262806666666661	0.931446587444396
RNPS1	0.132986497933167	0.673751455020525	-0.135082441609535	0.807470535721699	-0.0663799999999998	0.764545897602815
NR1H2	0.132865360886777	0.674206340588878	0.110803094697832	0.849357727620213	0.0204616666666669	0.945904449997854
CAPNS1	0.132818341652994	0.674354643624273	-0.413562079851966	0.2391639242947	0.0640666666666675	0.779485206901945
TMTC2	0.132788391174679	0.674469516898462	-0.0840709405257503	0.892634402174414	0.138121000000001	0.551125574415782
TSPY26P	0.132773182567842	0.674469516898462	-0.111515149165576	0.848875498955953	-0.220982666666667	0.388303383752735
MAD2L1BP	0.132767075096898	0.674469516898462	-0.190643122791629	0.703402857980256	-0.00708366666666545	0.987163226136842
MYOCD	0.132659045762766	0.674838120156235	0.0487568613535157	0.94220484638146	-0.109910333333333	0.616961986237355
HIST1H2BN	0.132600765765484	0.674985796872349	-0.0227226671139564	0.97536722461972	0.196670000000000	0.410343763683663
HIST1H4J	0.132588055306374	0.67499684779374	0.0141594746940193	0.983444353656142	0.165321000000001	0.45895048781174
IRAK1BP1	0.132512595408980	0.675202460791617	0.248263163597443	0.577730323259556	0.337190333333334	0.1780215342276
RPS6KA5	0.132304580796751	0.675820355948717	-0.31469328036935	0.436900127228272	0.136023000000000	0.578893615511221
HEYL	0.132297110471514	0.675820355948717	0.143221236765564	0.79294618815764	-0.0984643333333327	0.697094236295306
FUT6	0.132248968046445	0.675991499303254	0.185465306273700	0.714427536588157	-0.125812666666666	0.539568472169283
LOC100130275	0.132183255065681	0.676076766978119	0.382475036461132	0.295177945739812	0.0831513333333343	0.766569862858904
FGFR3	0.131997489631737	0.676613340763984	0.383472770358371	0.293474457479736	0.0615386666666674	0.815540776675037
CTNNBL1	0.131989072693778	0.676613340763984	-0.0811671869809174	0.896346444707454	-0.0476563333333324	0.843152489417868
RILPL2	0.131929969065912	0.676750892212455	-0.208796820425077	0.665878812648868	-0.216438666666665	0.25470355352902
TNFRSF11B	0.131855321001233	0.677115328492982	-0.103444553100979	0.861943478503987	-0.0400569999999997	0.873663396186202
FRAT1	0.131812816455637	0.677169353232024	-0.150157430094059	0.779734405920598	0.0511136666666669	0.85184175589894
TCTN3	0.131812750186066	0.677169353232024	-0.0519170910680768	0.938067483409541	0.0998253333333332	0.640267000021599
TMEM95	0.131748804969979	0.677273036636075	0.00767041583103073	0.990405674923721	-0.0824519999999996	0.731007359044756
TLR10	0.131745122040019	0.677273036636075	-0.221091983664488	0.638286782439236	-0.0395869999999998	0.892810620000722
LOC442075	0.131712399195989	0.677415402767854	0.0403159370564851	0.952720585102517	-0.0947693333333322	0.647065102430804
LSM6	0.131660507316158	0.677539119302871	-0.0991606678748175	0.868042453226125	0.0539043333333339	0.851568943534661
SLC6A18	0.131639934859047	0.677601836090063	0.0458204653145645	0.945372627844176	-0.108508999999999	0.633561720825521
EYA2	0.131609909092259	0.677654377133094	-0.136038561685136	0.805641810268282	-0.326087999999999	0.155319778701693
MAFB	0.131507513930342	0.678027102115901	-0.0163865431923123	0.981892085795004	-0.343098000000000	0.128872002711120
PCDHB16	0.131501069898356	0.678027102115901	-0.141318044998745	0.796285336930078	0.163697666666669	0.57851821618347
TRPM4	0.131498036125167	0.678027102115901	-0.0859846241184448	0.888306347447303	-0.0652003333333331	0.77740028876925
RPS10P7	0.131430422849834	0.678253983107765	-0.490486982982584	0.124558098726319	-0.107565666666666	0.721039266334933
CSTF2	0.131342447443562	0.678707955209739	-0.412739836192726	0.241609582743514	-0.216818000000000	0.353471671280201
ARHGEF9	0.131339192703550	0.678707955209739	-0.208735472863667	0.666024041170443	-0.230600999999999	0.278938500161599
TAGAP	0.131325650520493	0.67872459678067	-0.0659550045938481	0.917090201751967	-0.0571623333333328	0.799725367501923
NLRP5	0.131262472089431	0.679066849552167	0.107355539461449	0.857128584932911	-0.137356999999999	0.516217364739058
NONO	0.131220841099066	0.679267783642918	-0.248383691311835	0.577626262735828	-0.0104099999999996	0.972395918043445
CLMP	0.131177513841345	0.67929334144288	0.0958339895464322	0.872499931194687	-0.116676666666666	0.764133293709815
HMGN2P46	0.131166667442562	0.67929334144288	-0.316169252090585	0.433381822396051	-0.132689333333332	0.623357154900356
ARID5A	0.131045661327968	0.679984034657302	-0.120418294776131	0.83453066829804	-0.0524561666666669	0.82700027112124
FBXO28	0.130730310452752	0.681115032592615	-0.120582512936514	0.833850618726547	0.281311333333334	0.145985985395390
ANKRD40	0.130691134288711	0.681300032806741	0.171902234717635	0.739043760943627	0.0636393333333333	0.82616467676523
NKX6-1	0.130609360785647	0.681498143157741	0.282343296524657	0.503790591247595	-0.272109666666666	0.278744378442307
DEFB126	0.130605942417521	0.681498143157741	-0.312976152412087	0.438673188880418	0.108965666666667	0.759682045828681
ANO2	0.130559487124042	0.681586437582039	0.0185228148096545	0.97902968742576	0.0225493333333331	0.94375604597120
PRR14	0.130499293322327	0.681909510389078	-0.264670159717601	0.54347643041286	-0.0726116666666664	0.748579513438275
ST6GALNAC4	0.130471687519018	0.681946283944242	0.103380442505476	0.861949447881126	-0.136577333333331	0.525917496526617
PRPSAP1	0.130447827829817	0.681958466715684	0.428604464187993	0.210173511664670	0.107653000000001	0.670113640649168
FAM171A2	0.130396857694432	0.682148720261065	-0.00365169844989228	0.994588964700772	0.0139693333333347	0.978192116090894
CD34	0.130385249163315	0.682152708659858	-0.0967808243061737	0.870762069561258	-0.0291693333333328	0.936557228489982
GPR4	0.130186138683574	0.682950787464635	0.130235794933573	0.812271254601992	-0.233583000000000	0.306750175723678
NR2F2	0.130126918361560	0.683127099119157	-0.0130335189593873	0.984547546298896	0.0817613333333345	0.731303939999377
TMEM75	0.130091897230708	0.683220022269031	-0.080952128687379	0.896361013063453	-0.091954666666666	0.704370299831112
KDM5C	0.130059793727759	0.683293890703655	-0.205006781959428	0.673623956972285	-0.229360999999999	0.296197666389862
ZNF587	0.130002919793589	0.683293890703655	-0.0705479016346304	0.91009246060164	-0.0291586666666663	0.91627892449973
INCENP	0.129991005513389	0.683293890703655	-0.206007235231555	0.672284583020815	-0.0139129999999984	0.963815889459617
FKBP1B	0.129981142589096	0.683293890703655	-0.271326600651666	0.527984832801825	0.0674703333333342	0.78606666960761

PEX26	0.129968398034885	0.683293890703655	0.168558796858466	0.746758286599055	-0.0360416666666660	0.888151072264636
BUB1	0.129965774559807	0.683293890703655	-0.00158167307814452	0.998066886004786	0.0965916666666667	0.6846855637534
FRZB	0.129960825115796	0.683293890703655	0.00271271498517124	0.995791124790592	0.0846006666666667	0.710917898234342
PPP2R2A	0.129945495321407	0.683293890703655	0.145863432715416	0.787755441629161	-0.00742233333333332	0.98256318768706
ASB13	0.129940773680179	0.683293890703655	0.57568078192646	0.0441256193514982	-0.390211333333332	0.0601168126740641
TRMT11	0.129936589938644	0.683293890703655	0.00954346084832312	0.98909166696601	-0.0832669999999993	0.692494286263795
NUFIP2	0.129820693794993	0.683549800792998	0.138140353460148	0.800478820487963	-0.191279666666664	0.390435015799203
ZNF776	0.129791655517555	0.683668336989706	0.154151569846514	0.77207588593583	0.261483000000002	0.195927187569286
POM121L2	0.129729718409052	0.683818702671633	-0.112134294164914	0.847454473507881	-0.0236356666666666	0.941559580750404
MBNL1	0.129724809365587	0.683818702671633	0.0373392206538175	0.955937551268326	0.155415000000001	0.441366313859876
CENPJ	0.129658064544618	0.68418491104014	-0.204870027118904	0.673728362275223	0.00297600000000076	0.99529569362957
DIO3	0.129542683367421	0.68487082060348	0.0645261026459201	0.919501506803908	-0.0848926666666664	0.760810252329096
GTPBP4	0.129510572037715	0.684900785854669	0.0779878297469146	0.899705053748212	-0.246266	0.187231535349616
ZNF645	0.129508722608963	0.684900785854669	-0.0128395148005664	0.984985516091596	-0.111354000000000	0.741809049318412
TMTC4	0.129501914189276	0.684900785854669	-0.0544707385852705	0.9344692810234	-0.563155999999999	0.0392329313827969
OR10D3	0.129472183138878	0.684900785854669	0.0328631446963416	0.961351562552925	-0.242070333333333	0.228292762690892
SARM1	0.129443075228715	0.684953945104925	0.233726067572674	0.610436189314218	-0.0897613333333333	0.774210549173745
MC5R	0.129442117587506	0.684953945104925	0.135436280118597	0.80704358190743	0.0267873333333334	0.92469922248949
ATXN3	0.129290814005278	0.685772635257597	-0.0661144339433405	0.916792605561869	-0.135872999999999	0.611770847276966
GLOD4	0.129284587718093	0.685772635257597	0.135480916641149	0.806930030832744	0.107587333333333	0.631650146912655
TMEM132D	0.129211560101865	0.685997510941375	-0.155182287737489	0.77066107821265	-0.103245999999999	0.731069486458256
APBA1	0.129206423873285	0.685997510941375	-0.252207304960493	0.570465803488487	-0.0121143333333324	0.974451420920923
MRFAP1L1	0.129185834886368	0.68602444960146	-0.303933168270640	0.460052080110635	-0.00203466666666579	0.99529569362957
HBD	0.129148007884893	0.686164832047574	-0.114366525506297	0.844272085764738	0.0765816666666667	0.766569862858904
POLR1E	0.129134605785192	0.686180703866193	-0.141218674554432	0.796285336930078	-0.258216	0.258517764941643
LOC440173	0.129093153573543	0.686375053827508	-0.369225137520448	0.323285574998587	0.276126000000001	0.158095716859654
UPK1B	0.129044715190987	0.68638239484696	-0.00477924267016113	0.99311930576133	0.0761963333333336	0.823062219441786
EEF1A1	0.129020566001155	0.68638239484696	0.0883556243370532	0.885142944149088	0.00486500000000196	0.987937064305561
LOC100506730	0.129016773589216	0.68638239484696	-0.0161431657081249	0.982057247916055	-0.174350333333333	0.426518032099216
LRFN1	0.129000735154146	0.686410801894367	0.0586412189959556	0.928586335857223	-0.128855000000000	0.588365247524911
CABP5	0.128936128699355	0.686674310746265	-0.290483540560128	0.488219138098698	-0.190578666666667	0.461494497197158
IRF9	0.128927699642146	0.686674310746265	0.110759234320452	0.849357727620213	0.0117545000000001	0.970030485008698
ANXA2P1	0.128792506917237	0.686985529575936	-0.155919259144966	0.76972820876143	0.356469333333334	0.162505104948410
ZNF197	0.128763710640114	0.687102647699536	0.0526790489756701	0.937357975161644	0.108423666666667	0.641391983149645
FAS	0.128747657285047	0.687107430942243	0.164702426925066	0.754002787599457	0.441815333333334	0.034523754310686
YIF1B	0.128691568195798	0.68721937888013	0.0933183135548902	0.876656471145851	-0.0363675	0.887363245747406
C15orf54	0.128668352365403	0.68721937888013	-0.0232502064599494	0.975348968561756	0.137893000000000	0.531469495552605
GPRC5B	0.128664793352937	0.68721937888013	0.155056173442398	0.770708441022583	0.133364666666668	0.55155082188871
TNXB	0.128658134094619	0.68721937888013	0.191359442638580	0.701753730088091	-0.133204666666666	0.68171195530621
ARHGAP11A	0.128626429492264	0.687297607619081	-0.159246506091625	0.764789523460873	-0.0441179999999992	0.870843154725153
NAT10	0.128624281934892	0.687297607619081	0.188155690746494	0.707635058271509	-0.20003	0.390497309173453
WISP3	0.128588052938345	0.687463587607856	-0.104928551999759	0.86067831050617	-0.00351899999999919	0.991883362600632
C12orf75	0.128525217647590	0.687727698012963	0.0934076604983724	0.8764557345753	0.0454230000000002	0.854488714029133
TTLL1	0.128524810231025	0.687727698012963	0.0892181437365943	0.883460564717377	-0.0534366666666661	0.826163946239814
FAM162B	0.128482358002414	0.687727698012963	-0.126665945574035	0.820958242958398	-0.0977933333333329	0.712601873916151
CHMP6	0.128482029129072	0.687727698012963	0.299690962586098	0.467649102112712	0.147033333333334	0.503308350574223
UG0898H09	0.128436767115472	0.687892899728614	0.193614905121744	0.6978139054698	-0.0828979999999997	0.818043942279884
ATP12A	0.128411130924868	0.687977342396888	0.0946296007731395	0.8739154222556	-0.103185999999999	0.68135180147671
BTNL2	0.12824073657941	0.688736882359778	0.0539103146973671	0.935390656567557	-0.0238143333333328	0.95482028291900
NEFL	0.128203897585543	0.688783588520571	-0.0408857472617915	0.952426531595115	0.092263666666668	0.748715075113364
ALDH6A1	0.128200716211569	0.688783588520571	-0.0379081689502645	0.954958350556272	-0.0738079999999995	0.754656494947715
CCNO	0.128105888113436	0.689221118646485	0.152852912047849	0.774725957219946	-0.118569333333333	0.63046946440408
MYH16	0.128095455741492	0.689221118646485	0.188372921024352	0.707361603786947	-0.161392333333333	0.449693583707481
LILRA5	0.128090292891737	0.689221118646485	0.00748230329762786	0.990795532161521	0.0671413333333335	0.791198225931664
BLZF1	0.127995621313515	0.689699474179867	-0.152097857255173	0.775926738317144	-0.0108263333333323	0.973783155960644
WDR6	0.127934135191225	0.689959598239135	0.198742503604579	0.685648577129052	-0.210487	0.26379864393003
GPR3	0.127861843351296	0.69033357397711	-0.0308192090742766	0.96529904314994	-0.0878963333333334	0.731069486458256
ANP32C	0.127840616828218	0.69033357397711	0.224446689261192	0.632710354180004	-0.119155999999999	0.592716584553539
SLC17A7	0.127786023311320	0.6905009408709	-0.124237386209044	0.825382714207684	-0.0109399999999996	0.97184501043952
POLA1	0.127742326738752	0.690650819938834	-0.223687929952815	0.63401557109874	-0.0214963333333327	0.952916086133035
NPFFR2	0.127673619240143	0.690650819938834	0.00322336438502344	0.994730189431381	-0.0819386666666663	0.705543977023539
KHDRBS1	0.127669900695118	0.690650819938834	-0.103925001109930	0.861570109612412	-0.00537499999999877	0.987134800094338
TMEM212	0.127668771925221	0.690650819938834	0.196853159158319	0.690061983084583	0.0649653333333337	0.80355218052314
LOC654433	0.127655669373946	0.690650819938834	-0.072395091898384	0.907556175729082	0.029073666666667	0.943605992246673
CSK	0.127549580275445	0.690937441916763	-0.1792477861521	0.725276394376351	0.0457096666666676	0.856640814502011
PRMT5	0.127544170985149	0.690937441916763	0.097078441796332	0.870143694994237	-0.163495999999999	0.446756640099031
TRUB1	0.127483932237739	0.691132305236648	0.173549468756435	0.735851467282891	0.157585000000001	0.44275000040488
HSD3B1	0.12741899900253	0.691368214259075	0.235742437914108	0.606716022115166	-0.143842	0.534910593395784
ACAD8	0.127403777657578	0.691368214259075	-0.482710815591007	0.135560525489292	-0.0689633333333316	0.771373465365664
ARHGEF16	0.127313211125313	0.691559121838698	-0.056212218265742	0.932459880997552	-0.162024333333332	0.410584580608825
SSBP3	0.127307147180477	0.691559121838698	-0.170215496405	0.74270446821332	0.082173000000002	0.76368723681351
RPUSD1	0.127253097855897	0.691698373298102	0.170153725556433	0.74270446821332	0.0621820000000003	0.782886750366221
C5orf15	0.127225876799866	0.69180534680697	0.0490484196033857	0.942126968886265	0.248984666666668	0.271129799442592
TELO2	0.127192118839607	0.691865737368823	0.450636771538036	0.176663460992485	-0.201196999999998	0.324937679238988
ZMYM5	0.12716751843898	0.691865737368823	-0.133235979701490	0.808992949393043	0.118970666666668	0.58969057474622
KLHDC2	0.127127674488116	0.691865737368823	0.372620219306664	0.317205263174137	-0.0745906666666652	0.754015160951226
ARG1	0.127120431009809	0.691865737368823	-0.0158443679061384	0.982102356159698	0.667656666666667	0.324443655582244
KLK11	0.127118081851984	0.691865737368823	-0.0771396821458103	0.90125257838581	0.0626923333333339	0.792778259205127
GRAMD1A	0.127077685570099	0.692059368877965	0.216281071967359	0.648391670659269	-0.0512073333333328	0.843134844119548
GINS1	0.126969533941484	0.692410471244923	-0.0723730492286411	0.907556175729082	0.203032666666668	0.302530834830147

DUSP4	0.12687705728111	0.692682697321704	-0.0140219764731719	0.983461004910902	-0.0413553333333319	0.888476605628154
FARP2	0.126873382593412	0.692682697321704	0.128620323162767	0.815811316696798	-0.0137573333333325	0.966342921970461
CKM	0.126834801153011	0.69286208891999	-0.0117875895400886	0.986418801521542	-0.0597659999999994	0.799725367501923
KIAA0100	0.126792169693229	0.69286208891999	-0.192409523091784	0.700450711372106	-0.365076333333332	0.124984536231968
IFNAR1	0.126780403339355	0.69286208891999	-0.0955619137652733	0.872754579338908	0.00580833333333349	0.987134800094338
CHODL	0.126745143134249	0.692935815024135	0.360099091624933	0.34143422019372	0.0450823333333337	0.859530555826076
NCAM2	0.126683671933253	0.692935815024135	-0.103303253155452	0.862065338914705	-0.145955333333333	0.46282835887768
HYDIN	0.126681615485469	0.692935815024135	0.235478970831507	0.60678764664537	0.120740666666667	0.619932346687646
ALG9	0.126645393919287	0.693071792256622	-0.0840295733838194	0.89265118468217	0.243414000000001	0.213135306400917
S1PR1	0.126496105884483	0.693795940224646	-0.155123008593317	0.77066107821265	-0.254295	0.192973194605635
SLC17A9	0.126409638173751	0.694132836544359	0.119734663821395	0.835531661413366	-0.186621333333334	0.57356096971983
LYAR	0.126390163772618	0.694132836544359	-0.361290935644404	0.339627732901974	-0.301913333333331	0.159421950325134
C3orf37	0.126348287900069	0.694150335650663	0.101097214443895	0.866005093242444	-0.172527999999999	0.398883894731131
CHORDC1	0.126335456368007	0.694150335650663	-0.119265569462425	0.836360753951164	-0.0186506666666648	0.949314722489396
SLC20A1	0.126322182535429	0.694150335650663	0.175111623491054	0.733192115929191	0.366740333333334	0.0884768351275245
MBD1	0.126301492006853	0.694150335650663	-0.145436766179537	0.787896872604863	-0.0441716666666662	0.861690060758414
C2orf50	0.126299956767368	0.694150335650663	0.00867321875690303	0.98946753270812	-0.0522429999999998	0.825729064695823
CACUL1	0.126222156073037	0.694417788555655	0.126061194962163	0.822164329801713	-0.0211909999999989	0.936239636794556
IGF2BP2	0.126216431121287	0.694417788555655	0.0223753995781228	0.97536722461972	0.0484996666666675	0.887363245747406
FAM179A	0.126168331875441	0.694646995451288	-0.156678045242389	0.768752873219075	-0.0106926666666665	0.972334209448278
VMO1	0.126123473274347	0.694735470336455	-0.114674023716000	0.844162087612813	-0.0644556666666657	0.830530508494463
SETDB1	0.126082515719955	0.694859284749551	0.00127805670274297	0.998348925592729	0.0930996666666666	0.665196715536232
OPALIN	0.126065167154455	0.694859284749551	-0.0884507766795468	0.884893243593597	-0.151286000000000	0.46336719380894
TINF2	0.126029565242761	0.694993419136248	-0.0311049433915250	0.965118873711308	0.0759293333333342	0.772520347452753
SGOL1	0.125947661595402	0.695388308643726	-0.0548399515410504	0.934255557320745	0.173145333333334	0.5530742296054
ZNF35	0.125922324405388	0.695445278178477	0.0918865446715959	0.879153312143409	-0.149470333333332	0.485054643787725
FAM160B1	0.125842909998879	0.695501838169025	0.249206894716909	0.576149573608393	0.119420000000001	0.575378149225683
TMEM133	0.125784941726699	0.695801408669718	-0.0220978465062913	0.97536722461972	0.399436000000001	0.124721634754001
GPR128	0.125745152717710	0.695911161292238	0.183017072985796	0.718277789823975	0.0552140000000004	0.855533429360007
TMED3	0.125624978383686	0.696415227326658	-0.210457019044905	0.661670522548494	-0.0591766666666658	0.799725367501923
RGS6	0.125520103821014	0.696897286651041	0.103002125569583	0.86257932725575	-0.0221863333333331	0.933248182961486
PARN	0.125503783633680	0.696897286651041	-0.0309687548368340	0.965250976084263	0.125874333333333	0.524648943468618
C19orf10	0.125499607997807	0.696897286651041	0.130907039735627	0.812271254601992	0.184275666666668	0.376020235114735
FLRT3	0.125376521094476	0.697563614518638	-0.22067872794975	0.639008702745457	0.0686143333333334	0.777392889079628
AMBRA1	0.125324551981252	0.697733202040456	0.0401627381634061	0.953057205323657	0.00393200000000096	0.991175862027014
PIP5KL1	0.125308371104021	0.697733202040456	0.114769523150393	0.844162087612813	0.106088333333334	0.667144126671051
ENPEP	0.125307014639131	0.697733202040456	0.127466829023941	0.81893660870295	0.100013333333333	0.637426515050706
MPHOSPH9	0.125260511723693	0.697862656205186	0.157400757723031	0.767770230680061	0.164815666666668	0.428953946992621
ZNF432	0.125238448457802	0.697880464743099	-0.100883562703408	0.866005093242444	-0.00621399999999824	0.988133186272453
KIAA0586	0.125215540153947	0.697880464743099	0.0102652134919929	0.988174744960397	-0.224022333333332	0.376882447735926
RBM17	0.125201512686741	0.697880464743099	0.00381663971932487	0.994588964700772	0.00917033333333359	0.976702491879288
PLN	0.125190374736868	0.697880464743099	-0.25181102620285	0.570957449196347	0.082530333333334	0.729912257977231
GPR137	0.125186233446759	0.697880464743099	0.0224930079253275	0.97536722461972	-0.00839266666666687	0.98025379113353
CPNE2	0.125122938545281	0.698009281533624	0.03750636618277	0.955411256179887	0.202719000000000	0.489894482040166
TRIP13	0.125078355574120	0.698145639004657	0.277107267962812	0.515333497848934	-0.306513333333333	0.156827757693885
CASC5	0.125057963994533	0.698149191996322	-0.170763367115806	0.741154777036791	0.161787666666668	0.541939010010167
KLHDC7A	0.124945381043754	0.698386735123518	0.0304266434584207	0.96583351705897	-0.0527483333333336	0.84290135427471
LOC652276	0.124913488532407	0.69852473081082	0.0338447282677282	0.960223960238245	-0.232874666666667	0.592903969429707
RFPL3	0.124865003341388	0.698651896697349	-0.116075267812626	0.842714411517451	-0.138898333333333	0.568659275949231
LINC00085	0.124826499913129	0.698809641750692	0.400281764013314	0.263139454802378	-0.0104929999999989	0.972327702257384
C21orf90	0.124803068560793	0.698891961564471	-0.0748548205664449	0.904466414650249	-0.214709666666666	0.262632881930563
SLCO1A2	0.124791122403203	0.69889868255838	-0.263338537402759	0.545883385328225	0.407620333333334	0.128450477696566
MDGA2	0.124731114341252	0.699101516100063	-0.218813103569888	0.642137448592891	0.00570300000000062	0.987163226136842
LUM	0.124703063843971	0.699101516100063	0.213438204130882	0.654550434157114	0.0315709999999998	0.906093399285191
RPGRIP1	0.124698051142476	0.699101516100063	-0.0267636487942966	0.970207612777188	-0.0680833333333329	0.76819424073258
CLPTM1	0.124683843024622	0.699101516100063	-0.0464529646406625	0.94484527463814	0.0110833333333346	0.970456964810804
CYB5R2	0.124631657711076	0.699177512183398	0.261121978409294	0.552696861759865	-0.547651333333333	0.114599211633843
NEIL3	0.124617693996546	0.699177512183398	-0.0780582622870619	0.899705053748212	0.305396333333334	0.257194631784673
INO80C	0.124592880701065	0.699268945064698	-0.222921548317140	0.635247978870818	0.117938	0.600135947031633
RPH3A	0.124573616181675	0.699323866149192	-0.0631972069423346	0.921359867071909	0.0519226666666669	0.87095450632169
WDR12	0.124505935543061	0.699528866424843	0.139952141503480	0.7976681602188	-0.255889333333333	0.198299581791991
FOXA1	0.124503431358371	0.699528866424843	-0.327290142331238	0.40846136066764	-0.0156806666666672	0.966879505998417
PLXNA2	0.124432864236896	0.699819048741485	0.412632859040769	0.241609582743514	-0.0798979999999988	0.712406292376982
UTY	0.124382178602441	0.700008934642897	0.143470583515228	0.792621058154238	-0.0903166666666662	0.733867173296405
CER1	0.124242447835604	0.700569404983347	-0.0206716983599587	0.976334100237874	0.0750556666666667	0.729368274679147
ATP2C1	0.124196480396501	0.70065640347438	0.271320839736722	0.527984832801825	-0.00487433333333158	0.987937064305561
CEP85L	0.124155675498937	0.700853174959123	0.193449537898953	0.698131592467487	0.254923666666667	0.242636078655898
TSLP	0.124136434366121	0.700907979054184	-0.121241759490295	0.83256316766566	0.204798333333333	0.309421300309741
RHOU	0.124104598060807	0.700946007305817	0.549570796003159	0.0608648191629941	-0.410433666666665	0.0697845693837847
SCGB1D1	0.124079871739953	0.700946007305817	0.0796411480630212	0.898031113962014	0.0800916666666672	0.78222483684508
DDN	0.123904000296766	0.70171967553804	0.0990039022674126	0.868042453226125	-0.176346999999999	0.549659494702918
NEK10	0.123867956456368	0.701813271295766	0.106807338896792	0.857800958803773	0.215212666666667	0.397798624983652
GPATCH8	0.123839907530911	0.701926107296376	-0.0109167646629222	0.98774587825743	-0.0197853333333335	0.946392308016584
TRPV5	0.123811110786307	0.701978518396593	0.141188376905859	0.796285336930078	0.0348980000000002	0.899484889138756
IQCC	0.123734854971669	0.702258554113845	0.172461051959463	0.738421727152922	-0.0200866666666659	0.94678260229888
CXCL2	0.123698586355165	0.702425549134902	-0.373842351687	0.3153356348575	0.0374856666666671	0.911715211050394
OLFML2A	0.123631173576498	0.702528099877908	-0.121193592722673	0.83256316766566	-0.0925416666666666	0.670071773006358
SLC6A9	0.123628523988300	0.702528099877908	0.270655834668219	0.529418563170514	-0.239658666666665	0.321696396000061
PSD3	0.123621367695773	0.702528099877908	0.391045704987661	0.281310802292141	0.188811500000000	0.406211996897819
PNO1	0.123617535416481	0.702528099877908	-0.117421008884168	0.839825318851589	-0.143645666666666	0.516217427447249

WNT16	0.123589652202102	0.702639871111902	-0.267362587025041	0.536380012714446	-0.0100279999999990	0.977997080364813
KDM5D	0.123495079238280	0.702900891988945	0.0537017009852044	0.935777496327359	-0.0744423333333328	0.745455303567133
SHCBP1	0.123404158091538	0.703149936009818	-0.0174300874787450	0.980603313490195	0.0426880000000003	0.862744795576892
OSTC	0.123355795293595	0.703245964143763	0.0133313257724232	0.98419257407872	-0.130808000000000	0.514420666507349
SCARF2	0.123239068310760	0.703719487640125	0.281251943848031	0.506749495296958	-0.0409149999999998	0.924674647382585
CFHR3	0.123229360796218	0.703719487640125	-0.109844072678898	0.851073392194999	-0.0956426666666663	0.696927295330661
HAND1	0.123102390477970	0.704124774637857	0.194457044798114	0.696294554451457	-0.131894333333333	0.551642602536241
FZD10	0.123036777609421	0.704341462293288	-0.184628367362501	0.716351619471715	-0.0546729999999998	0.867640898095998
PTPN21	0.122923925030211	0.705013192376124	-0.277516303764531	0.514204199305983	0.349358666666667	0.211495399841293
PXT1	0.122875588682694	0.705187967013898	-0.302990557350396	0.462105017205825	-0.00746799999999965	0.982398683576954
AFF1	0.122840110462971	0.705278000994486	0.0382810906603075	0.954545200586067	0.117264333333334	0.614221661998915
STRAP	0.122810893429643	0.705398674725919	0.0267010140561317	0.970207612777188	0.0592936666666671	0.788201431210286
FANCB	0.122763204023787	0.705561241434282	-0.177511140960708	0.728529406441478	-0.0883183333333327	0.812358226571296
CCDC90B	0.122752797413351	0.705561241434282	0.216905327910903	0.647212711808862	0.213856666666667	0.284033466227373
FLJ16124	0.122742596629200	0.705561241434282	0.179218229991397	0.725276394376351	-0.0416089999999997	0.914418029309221
VWF	0.122706590949194	0.705726673882723	0.0548168579666232	0.934303341981145	-0.0328903333333334	0.896255571019035
LIN37	0.122683439438907	0.705807381807808	0.229307101710944	0.620358409975039	0.0743700000000018	0.745967084434416
RDH16	0.122661320684465	0.705809387588053	0.170373294570290	0.742671224116098	-0.119884000000000	0.578893615511221
FAM124A	0.122613902487598	0.705850468136394	-0.134811354476960	0.807867938002244	-0.0599973333333325	0.786973845403943
ZNF414	0.122585062259477	0.705850468136394	0.101007101378841	0.866005093242444	0.0955526666666666	0.6846855637534
MAN1B1	0.122573852034791	0.705850468136394	0.10870094901168	0.8536426264533	-0.334954333333333	0.0888564451939157
ERVK13-1	0.122556952239581	0.705850468136394	0.000126974791706484	0.999860038008296	-0.0471693333333324	0.880254449461876
IQSEC2	0.122544339949925	0.705861744534477	-0.0550214073294895	0.93406507597279	-0.117513666666666	0.612697324680132
PADI1	0.122497900508466	0.706024085599851	0.170263172669227	0.74270446821332	0.0677173333333344	0.814211123519969
SNX20	0.122449397880417	0.706251956280252	-0.190346837641697	0.704208582824765	-0.0830379999999996	0.774104133965233
CTRC	0.122324834801162	0.706589907949361	0.340508078342383	0.378350877728096	-0.0635516666666663	0.890094615232462
LZTFL1	0.122306141071826	0.706641274410755	0.0286515382211142	0.96782691865619	-0.239743333333333	0.25721699788802
ADD1	0.122277739438873	0.70667419514649	-0.241992892982889	0.594802118766439	0.0899023333333347	0.685254219232839
TGM7	0.122259988029237	0.70667419514649	0.0163106041477362	0.981907598420222	0.0388379999999998	0.9035064073154
TANC2	0.122246565761113	0.70667419514649	-0.216701100600664	0.647357580911858	0.180247666666668	0.45504615767131
FAM46C	0.122225962567391	0.70667419514649	-0.478888136508434	0.140193860716139	-0.0625793333333321	0.78960166957155
MAN2B1	0.122208429739425	0.70667419514649	0.0738624517752806	0.905506084101768	0.20326	0.423347587771162
MEF2A	0.122188296730095	0.706699824738825	-0.0726900044003573	0.90746837333842	0.0695720000000018	0.750408632709156
HEY2	0.122161940921705	0.706801685288009	0.0404916893184104	0.952720585102517	-0.0919426666666655	0.752735751974352
MGC21881	0.122085035640251	0.706949545174499	0.186451914151982	0.711421618891571	0.112746916666667	0.60302310939506
PRMT8	0.122002229254459	0.707230732675409	0.171945277483906	0.738962506786205	-0.135345999999999	0.627524319098374
UBQLN2	0.121989816292603	0.707230732675409	-0.389737448021676	0.282848386985187	0.0523050000000011	0.822390330537826
TCEAL2	0.121986330312815	0.707230732675409	-0.00530647945879796	0.99251028338798	-0.193973333333333	0.33770171093777
KRTAP17-1	0.121974148966394	0.707230732675409	-0.114907808993142	0.844162087612813	-0.173162000000000	0.483840860674189
ATG16L2	0.121955231588677	0.707230732675409	-0.326650660089011	0.409206882509236	-0.0683583333333327	0.7879955379643
TTC9B	0.121850480201058	0.707777303071722	0.253457009745264	0.567705016149998	-0.0546726666666658	0.86011149680202
PPWD1	0.121813175949170	0.707930724925892	-0.169627241795085	0.743463590096836	0.0125500000000012	0.96757929696675
FANCM	0.121802222605401	0.707930724925892	0.107647279700819	0.856590369736358	-0.113952999999999	0.639002829892966
ZNF280D	0.121791202035866	0.707930724925892	0.220784687571915	0.6389751639565	0.225441333333334	0.253356882479072
C7orf62	0.121783636849435	0.707930724925892	-0.109747201912144	0.851195083614214	0.0254653333333335	0.934697874124054
OR12D2	0.121734313609131	0.708040481035214	-0.237612124999335	0.602664863375033	-0.119779833333333	0.571364362269605
RNF38	0.121703932585105	0.708067227023447	-0.183345803056496	0.718067440349544	0.293888333333334	0.14208977383533
GPR22	0.121697594701682	0.708067227023447	0.346512031819915	0.365349151543041	-0.338844000000000	0.112438115304648
SLC24A3	0.121647228083492	0.708327311134094	0.193103616545964	0.699002603433222	0.00044933333333388	0.998351142601927
COL6A2	0.121626175761148	0.708350241305407	0.172213308126418	0.738567943867944	0.166445000000000	0.443378989641463
SMEK1	0.121621975602475	0.708350241305407	0.0908725400746021	0.881323965029954	0.0591280000000007	0.798038248389079
KCNRG	0.121485874025094	0.708816622566664	-0.0520847752397919	0.938067483409541	0.111999000000000	0.611725132091384
ZNF446	0.121398142045922	0.709251262648646	0.313070055164354	0.438459883259885	-0.0994179999999996	0.656324975591247
NFATC3	0.121346253081311	0.709314662424659	0.221503846308310	0.637706908132848	0.0636620000000001	0.796433280371074
CERS6	0.121343965012638	0.709314662424659	0.176924037331852	0.729794187782856	0.116389000000001	0.596617523400524
ZNF766	0.121334633703971	0.709314662424659	0.0856589559327495	0.889114985071002	0.0341586666666679	0.897291389279896
ABCC4	0.121334096294444	0.709314662424659	-0.216002336003442	0.64844945813838	-0.425310666666666	0.0492053003500414
TAS2R10	0.121286601036757	0.709484193056979	0.144503276978463	0.790345129882953	-0.0172286666666663	0.974451420920923
DSCC1	0.121160295572656	0.709875366212611	0.232546156250357	0.612395190522005	0.196832666666666	0.442294185443201
KRT12	0.121130928790156	0.709924038368359	0.247563839321075	0.579745218830252	-0.0508626666666659	0.830926049084239
SLC26A6	0.121121911145201	0.709924038368359	0.38545447762851	0.290201439895691	-0.0468706666666659	0.839998150752089
CCDC33	0.121029816604618	0.710140288244206	-0.00139381456815711	0.998303187767855	-0.0200509999999993	0.953075282897994
NUP98	0.121018647649889	0.710140288244206	-0.194676464599038	0.69591561649809	-0.0606656666666651	0.833546318326642
SLC25A16	0.121002058095621	0.710140288244206	0.0221752775747416	0.97536722461972	-0.185174000000000	0.490110576940323
REG1P	0.120963233070087	0.710278544785477	0.059724737455113	0.926352922534113	-0.0635726666666666	0.79977286075522
LBR	0.120943092095515	0.710278544785477	-0.185605833660631	0.714210278087368	0.155303000000001	0.513141966218084
C16orf72	0.120937566115436	0.710278544785477	-0.209891253998397	0.663436500730575	-0.071054333333333	0.756338676348025
SLC12A4	0.120911863981430	0.710366451467214	0.228103316952802	0.623935503350068	0.0924873333333344	0.724603790871296
NRN1	0.120902471379061	0.710366451467214	0.187155699135676	0.709833349594307	0.195538333333335	0.346165986086181
CYP2F1	0.120844170038645	0.71050769347915	-0.0305897393747819	0.965652334162082	-0.0470756666666661	0.838927397112197
N4BP1	0.120834169940731	0.71050769347915	-0.107855731350803	0.856307629977434	0.0336903333333343	0.91869635463323
RAB8B	0.120826660507137	0.71050769347915	-0.309702073489327	0.447222629729729	0.384721666666668	0.183310525125595
GCNT7	0.120812141019448	0.710531715577606	-0.0170057108726364	0.98105020869911	-0.238731	0.204586504492032
SLC22A25	0.120775179377869	0.710541958128532	-0.00534574755219757	0.99251028338798	-0.00725566666666613	0.98349947509937
LRRC40	0.120767101809339	0.710541958128532	-0.29461983360514	0.4789900136588	-0.00806199999999948	0.983159241812861
SLC30A7	0.120703437317333	0.710768927575761	-0.238953485373237	0.600195828925044	0.00483800000000083	0.987937064305561
COX4I2	0.120700029238543	0.710768927575761	0.0979897719231831	0.868852648210739	-0.213238999999999	0.442294185443201
PYGO1	0.120638237721221	0.711032724124954	0.0682491760984632	0.912364243071055	0.296650000000001	0.384626801306227
ZNF318	0.120500224600121	0.711837597455395	-0.076099239374058	0.903296922694955	0.0648170000000004	0.774104133965233
MAPK8IP2	0.120447692205521	0.712001646386153	0.229362117368455	0.620183562399927	0.0170653333333337	0.955968113642085

GLI1	0.120254172315109	0.712918931440917	0.0112795190582299	0.98731277141449	0.0235716666666672	0.949173055617325
TIA1	0.120209497469757	0.713070079938746	0.209292573340295	0.66471227046382	-0.0895329999999992	0.670473415437522
SLC4A5	0.120168571482268	0.713268243545766	-0.176848010390991	0.729794187782856	0.0270109999999996	0.919456505549688
APOBEC2	0.120091715486537	0.713551495744817	0.226880556062015	0.625901142430324	-0.169755333333333	0.48174711509713
CNTROB	0.120075397861519	0.713551495744817	0.220142011573512	0.640598540561765	0.118954666666667	0.558045577505427
PPOX	0.120067828192077	0.713551495744817	0.131923836498710	0.810418188352907	0.0641080000000017	0.790848042413284
SYPL1	0.120060363575321	0.713551495744817	0.149134910425517	0.782612598081927	0.334399666666668	0.0894613209101226
PIEZO1	0.119885775714178	0.714191736778815	0.164620876633053	0.754105947879839	-0.0358896666666656	0.895093529187836
HPSE	0.119878842238505	0.714191736778815	-0.0426007121773884	0.951071128215665	0.509677	0.0895276393548003
OSR2	0.119876288445756	0.714191736778815	-0.186790237538447	0.710705126059778	0.442106000000001	0.0338015945178966
SPRR4	0.119849955693204	0.714232591567531	-0.0909429748198187	0.881187233358984	-0.0118709999999990	0.97184501043952
HOXC12	0.119827576750040	0.71429785796918	0.0509147408320014	0.939708336427265	0.090080333333335	0.677647679541427
AIP	0.119767204118125	0.71454102559288	-0.0828602440480097	0.89362638370288	-0.027512	0.922148106708426
OLFM4	0.119756701559354	0.71454102559288	-0.0971985019492853	0.870020870354202	-0.0873673333333329	0.686516408582019
DNMT1	0.119697158997077	0.71454102559288	0.155439012333018	0.770224269831267	0.0210903333333332	0.942092082409632
SLC9B2	0.119634964755943	0.714613340058283	-0.2394561540158	0.599103609129687	-0.0895069999999986	0.689661288408633
PLEKHA7	0.119633585410175	0.714613340058283	-0.137481703762824	0.802039543563725	-0.0241016666666662	0.959032046952242
PKN1	0.119615652197806	0.714613340058283	-0.0252907891101151	0.972310017823397	0.0410820000000004	0.86946178874091
CD19	0.11961457515984	0.714613340058283	0.0512891261587264	0.938934809036451	-0.0985063333333331	0.641957306407734
CAND2	0.119593119526881	0.714613340058283	0.141063173429237	0.796353139796933	-0.00183166666666661[illegible]	0.995321141422816
ZNF598	0.119584089572225	0.714613340058283	-0.0505028763320886	0.940625181975713	-0.0536963333333333	0.838927397112197
ASCC3	0.119544533923917	0.714802480799935	-0.0198294120616253	0.977526645675126	-0.0433399999999991	0.871104061464043
SCN1A	0.119528708882197	0.714835181242333	0.192712181725868	0.699904185007497	-0.122778000000000	0.548643967646122
PRKCSH	0.119505401083545	0.714917214687111	0.240596116599271	0.597542015483042	0.0876766666666676	0.675687379893745
KRTAP4-4	0.119490879844458	0.714941322909202	-0.0688507087334976	0.912149839732916	0.00556800000000089	0.987134800094338
GPD2	0.119446788287110	0.715110906873912	-0.341024248199032	0.378272273903863	0.102700000000001	0.745703398753986
BMP2	0.119400229752929	0.715180847154351	-0.200408985446774	0.680995659089999	-0.495854999999999	0.10978993977345
TRMU	0.119311894228884	0.715620013570657	-0.182540435053995	0.719179495974011	-0.117867999999999	0.558807324932864
IVNS1ABP	0.119256033181371	0.715701824923321	0.263989590122882	0.544711371453131	0.143883666666668	0.492100665301584
DDHD1	0.119191202311147	0.715760539805575	-0.238293428015594	0.601773482805025	0.260677666666668	0.236989286061345
HSPB9	0.119184664996518	0.715760539805575	0.237818862966735	0.60243420134202	-0.0865766666666657	0.811401072147441
SLC24A1	0.119181962281723	0.715760539805575	-0.163326176489442	0.755791379610975	0.135375333333334	0.649420534269272
FIGN	0.119141556463357	0.71595531891629	-0.0907235909913999	0.881323965029954	0.165646333333334	0.486933147969243
PBX4	0.119088305403752	0.716006642125779	-0.118606879343872	0.837517381587206	-0.0191496666666670	0.958727447107698
AKR1D1	0.119039374007727	0.716006642125779	-0.35926311157516	0.342292001700374	-0.096786666666666	0.637426515050706
FUT4	0.119037821141887	0.716006642125779	0.161105873638362	0.760354001455808	-0.0165576666666660	0.956567100065073
HPCAL1	0.118986892917800	0.716259118693749	0.32177074738407	0.419622143161389	0.065653666666667	0.77740028876925
ZNF549	0.118962823586165	0.716274667340199	-0.416025369536054	0.234713120194856	0.00539933333333505	0.987937064305561
CD47	0.118934407751461	0.716275404765345	0.323664111253641	0.414215860139795	0.457608666666668	0.0351167468854759
SDAD1	0.118930156646752	0.716275404765345	-0.409861376311564	0.247376671929043	0.0812625000000009	0.766132481270445
TMEM200B	0.118884916997820	0.716451848090248	-0.0390820342438922	0.95397534100479	-0.136325000000000	0.58391025729515
FSHR	0.118843229371344	0.716451848090248	0.0974817113208266	0.86975668481123	-0.151382333333333	0.51010196507179
LOH12CR1	0.11882028749997	0.716498816456009	0.156211349688629	0.768909201237015	-0.0554196666666662	0.827168548756117
GSDMD	0.118801265381323	0.716537212744705	0.513987380605431	0.0962522730025878	0.0757830000000009	0.724384070314197
FAM19A5	0.118775587482262	0.716546544049802	0.244386925625235	0.588171246207334	-0.0627749999999988	0.809704159744507
MICALCL	0.118769648060467	0.716546544049802	0.0984523264232077	0.868308803285993	-0.0694306666666663	0.814956840733813
SMEK3P	0.118725006905264	0.71671807727321	-0.228810273142297	0.62186917609878	0.174921000000001	0.353701605651176
CDK16	0.118662953790252	0.716892155259683	0.0195715772526729	0.977673457110824	0.0443990000000005	0.85184175589894
ERVH48-1	0.118581158456166	0.717288175986544	-0.0312132130479388	0.96506747451534	0.0224040000000003	0.933412982728621
TOR4A	0.118534686102218	0.717379965354467	0.121949097955991	0.830347805834363	-0.0195173333333326	0.951715387188896
SGSM2	0.118472441931597	0.717647189640219	0.0926944133654553	0.877797216649453	0.0240153333333334	0.930627582383128
TYW5	0.118459616249166	0.71766022635388	0.262131954092582	0.549752906834892	0.211343666666667	0.327240483600249
PGAP1	0.118385749189261	0.717997619664188	-0.215485780560459	0.649753331562286	0.0114596666666679	0.974451420920923
KCNG4	0.118378027211471	0.717997619664188	-0.0376114234974926	0.955365651737247	0.0665840000000012	0.772139923817113
GPR20	0.11836804697436	0.717997619664188	-0.253414850861843	0.567705016149998	-0.110011333333334	0.681151028675655
GPR137C	0.118346247182108	0.717997619664188	-0.216064812164432	0.64844945813838	0.147777333333334	0.498454352317107
SYNPO2	0.118281251286897	0.718263872393012	-0.330482236512006	0.402284079938133	0.213559666666667	0.255595078844894
RIC8A	0.118239572020586	0.718382943969183	-0.182448922781707	0.719419078472232	-0.0672996666666655	0.762898679429892
NVL	0.118220738210120	0.718382943969183	0.00837938352474333	0.989949154584002	0.0427426666666678	0.883114887563545
HNRNPA1	0.118219802735532	0.718382943969183	0.197577808761071	0.688668717091024	-0.0308555999999985	0.910935860136448
MPZL1	0.118184798623634	0.718470698226979	0.00578094295285313	0.99251028338798	0.204000666666668	0.332502783413088
DDX27	0.118158218519463	0.718561038481528	-0.239910143872592	0.598134271398487	-0.0269790000000007	0.928988192507851
RPS6KA3	0.118116465749028	0.71863518592559	0.146995526547978	0.785782367062857	0.158960333333333	0.445566557024313
RPL7	0.118078863227376	0.718727842030234	0.342726358590028	0.374882411235977	-0.0918513333333319	0.710004432586908
KCTD10	0.118069878971677	0.718727842030234	-0.219238091835703	0.641608739373952	-0.0332379999999996	0.90945379489823
ENO1	0.117864456791785	0.7195818775888	-0.0788234351116655	0.899154242391172	-0.121853333333331	0.558045577505427
MYD88	0.117864406556457	0.7195818775888	-0.093781460655048	0.875355472414883	-0.323054333333333	0.100679552334348
DCTN5	0.117749019916935	0.719985156419642	-0.0921454494190694	0.879153312143409	0.0116766666666674	0.970030485008698
STAG2	0.117720416719443	0.720030769232987	-0.363081679946054	0.336332757540532	-0.0908049999999998	0.68499503971094
PHYHIPL	0.117625466397674	0.720342619746835	0.00863469773093599	0.98946753270812	0.175984	0.437698813460044
ETNK1	0.117601991762942	0.720342619746835	0.104704534655710	0.86067831050617	-0.0883596666666661	0.672980097690282
RGCC	0.117597226365174	0.720342619746835	0.124924653666252	0.824457843812434	-0.382936666666667	0.166543996066754
CCNB2	0.117553747654063	0.720402514366216	0.154601095882664	0.771310656070265	0.171515000000001	0.422934701797817
KIR3DX1	0.117549301228706	0.720402514366216	-0.176780189064572	0.729837424743184	0.162515333333334	0.627523379761431
SAMD3	0.117410269141625	0.720793830391426	0.476092921781817	0.143137411589934	-0.0656923333333328	0.766132481270445
SAAL1	0.117383652375972	0.720793830391426	-0.154949831256133	0.771179692342142	0.0323769999999997	0.925845244747948
ZNF37A	0.117382470922025	0.720793830391426	-0.251679349435544	0.571279752918483	-0.0498719999999994	0.827851662633248
TFG	0.117344245313532	0.720793830391426	-0.0133717824780904	0.98412883278237	0.182600000000001	0.349094758813838
AEN	0.117335486700108	0.720793830391426	0.253797781583567	0.567694846153007	0.742925666666668	0.0238147039217215
SYCE2	0.117294605652366	0.720793830391426	0.136093203604578	0.805628922205698	0.152194666666667	0.450203306181989

HOXB13	0.117294297621898	0.720793830391426	0.157913222969615	0.767373487136803	0.394487666666668	0.190569948330175
PPP2R2D	0.117259384136727	0.720793830391426	0.358952627464358	0.342742323643068	0.109935000000002	0.63969794643309
FLJ25694	0.117222404607275	0.720839821866445	-0.114310176288251	0.844272085764738	0.045866000000001	0.902744074046491
MATN3	0.117190857675141	0.720839821866445	0.458542392711574	0.165666571940874	0.107126333333334	0.634587745028953
TMEM194A	0.117180418004947	0.720839821866445	0.07592019226111	0.903510413986113	0.0343300000000002	0.895510661232872
CCDC122	0.117164277860432	0.720839821866445	0.106917251647873	0.857671866148626	-0.0114623333333331	0.97184501043952
TRMT44	0.117151356679912	0.720839821866445	0.0434451568367785	0.949521794694691	-0.160327999999999	0.443378989641463
PGC	0.117105762029430	0.720839821866445	0.148854155476781	0.783041584899534	-0.139543999999999	0.517746794831249
PPP1R16B	0.117096836798929	0.720839821866445	0.0247604976397611	0.973136696879552	-0.179284	0.451746572873908
C10orf90	0.117082886649834	0.720839821866445	-0.00479531901437237	0.99311930576133	0.00396499999999995	0.991121790806362
LINC00282	0.117041493183086	0.720936148017322	0.125384389677401	0.824013463206763	-0.00469833333333299	0.99106990984423
KRTDAP	0.117015839078153	0.720976992705946	0.0938017059194058	0.875355472414883	-0.0373156666666664	0.914066650169675
C18orf25	0.117009367488839	0.720976992705946	-0.212686361658062	0.656111996220908	0.00318533333333379	0.993756838831997
GPR61	0.116944940562001	0.721039212664324	-0.061328727382608	0.92421768673053	-0.0715313333333331	0.764973469318093
PAFAH1B3	0.116918529630466	0.721039212664324	0.0127357964283125	0.985156239185327	0.124722666666667	0.563937037048725
ABHD2	0.116917780520713	0.721039212664324	-0.0184205336796177	0.979063297753127	0.382426000000001	0.097583449418131
SFR1	0.116910446118185	0.721039212664324	-0.184210178211936	0.717436396325728	0.0648503333333342	0.787230398823895
PLCB3	0.116906870318124	0.721039212664324	-0.00353909870061537	0.994730189431381	-0.367355999999999	0.206203758496048
RPL35A	0.116863185532230	0.72125553111527	0.353166732509292	0.352407344218756	-0.0421633333333314	0.867690146364162
ST8SIA2	0.116838954896000	0.721343834776764	-0.104367618134727	0.861121906453898	-0.0508299999999994	0.852958509217732
SPATA20	0.116623599196324	0.72208004597085	0.374052771634480	0.314975789873702	0.191454666666667	0.415110347922019
PCSK9	0.116550893440171	0.722243511416982	-0.349277758249203	0.359794558968334	-0.055161666666665	0.852346905702153
CACNA1D	0.116516122032913	0.722330068301684	-0.138981450513820	0.79864602540281	-0.302763000000000	0.124096550131011
NXPH1	0.116422315492793	0.722805223265632	-0.182251799839256	0.719419078472232	0.0232556666666673	0.95784037718285
C5orf64	0.116362008780278	0.722988719248825	0.096914741745297	0.870644879522268	-0.0450323333333327	0.871735940334629
TNIK	0.116192135572734	0.723822524988782	0.27951980252585	0.509655886591018	0.754049333333334	0.00706146994541395
CSAD	0.116154255523150	0.723858350783633	0.174418188993745	0.734267769889068	-0.213075333333333	0.262910099772857
PPP1R14C	0.116096624306647	0.72402427423259	-0.289594987822058	0.488870852256136	-0.101047000000000	0.719993821355515
DTX4	0.116052503341239	0.724106414411064	-0.0316604023723182	0.963712086545628	-0.065900333333333	0.812792750346224
AWAT1	0.116051717267010	0.724106414411064	-0.0653199389268752	0.918295693698803	-0.126744333333332	0.734228369215232
PREPL	0.115972916958216	0.724319636051439	0.155911127085545	0.76972820876143	0.00903266666666794	0.975988780928093
TFAP4	0.115911323141107	0.724532643217107	0.0964074035470238	0.871504823647028	-0.125860999999999	0.592716584553539
KCNK1	0.115855527898700	0.724828919766309	0.176905351168026	0.729794187782856	-0.749228666666666	0.0252836057158779
ZBTB34	0.115748831165238	0.725246892961326	0.171763465892989	0.739232511855443	0.386608333333334	0.0775561412231076
ZER1	0.115655884253866	0.725515919451903	0.0897029499893851	0.88318195439136	-0.0441433333333324	0.859299487874574
MARK3	0.115607095920902	0.725515919451903	0.0492925982079159	0.941842562680095	0.0636513333333342	0.774210549173745
UGGT2	0.115547502645596	0.725515919451903	-0.0371514134583593	0.956363028822848	0.0870710000000016	0.710004432586908
BLM	0.115529433397796	0.725515919451903	-0.167429856705356	0.748960978799932	-0.0328289999999996	0.930627582383128
ARSJ	0.115513804108435	0.725515919451903	0.259717989298171	0.556147580300637	0.685702333333334	0.00917425090054953
C16orf54	0.115480569581719	0.725515919451903	-0.230297482166400	0.618707227156037	-0.215341666666666	0.523453039510001
MRPL46	0.115455310441006	0.725515919451903	0.088786291406294	0.8841943128258	0.0551496666666677	0.811544767127089
FIBIN	0.115435604724793	0.72553072673316	0.22264514817694	0.635668764102271	-0.122761333333333	0.60814016771842
HOXA5	0.115418520369067	0.72557210066831	0.00721225333593993	0.991025604847553	0.211481666666666	0.268919232288051
MARCH10	0.115361040236306	0.72572601765204	0.247446621492993	0.579745218830252	0.0440376666666674	0.852208015389387
LIG1	0.115352803627231	0.72572601765204	-0.0987445158484513	0.868042734188964	-0.085059666666665	0.720071550811076
STK25	0.115352779888824	0.72572601765204	0.177594524745798	0.728492631264289	-0.112045333333333	0.614221661998915
GABPB2	0.115342856511519	0.72572601765204	-0.232273211495613	0.612658112747496	0.291557666666667	0.187209474302320
CEP128	0.115308761926778	0.72572601765204	-0.0718904425906743	0.90837606217316	0.0174363333333340	0.958133242434411
QRICH2	0.115263892266207	0.725829032050097	0.344908014806025	0.369280599599459	0.238695000000001	0.226054670808826
TDRD3	0.115240356601476	0.725829032050097	-0.136740873778633	0.803874269165519	0.130769666666667	0.578156984559118
SMAD6	0.115238369698695	0.725829032050097	0.339841091738202	0.380248604746386	0.0150493333333339	0.956567100065073
NKPD1	0.115135186782327	0.726157960314319	0.157727390129247	0.767770230680061	0.105579333333334	0.717906134812783
TSC1	0.115074595051422	0.726414633016046	0.112219112533513	0.847454473507881	-0.189756666666667	0.520250587274909
NUFIP1	0.114991802572013	0.726888486311357	0.00801198442539945	0.990405674923721	-0.119284999999999	0.555689256408654
TRPC5	0.114958002659903	0.726986345167162	-0.0340222811056192	0.95993706312687	-0.0188996666666665	0.946747601432857
B4GALT3	0.114869647253354	0.727358154900114	-0.202586295495984	0.678404399740367	0.157429000000000	0.473694625379344
GSDMB	0.114866482019172	0.727358154900114	0.0300599137067311	0.966396286514145	0.0663706666666674	0.791240721091965
B3GNT2	0.114777727427146	0.727572498664874	-0.0600714630032558	0.925676268175378	-0.426464333333332	0.048170505761132
RHBDL2	0.114720167708821	0.727572498664874	-0.137167624732850	0.802743708223464	-0.0485996666666666	0.856854911614454
PATZ1	0.114715865293363	0.727572498664874	0.055257898874219	0.933521481800723	0.0541663333333352	0.812232870389155
AQP6	0.114698635788005	0.727572498664874	0.0582023682376332	0.928879154519933	-0.104036666666666	0.731974410873657
POLR2E	0.114652974117811	0.727667409727304	-0.300937251566525	0.464913571526964	-0.0547563333333325	0.809505390888975
FRMPD1	0.114607769157011	0.727770319986321	-0.0333313239077182	0.960784250392482	-0.0184236666666665	0.955701951169976
FHOD1	0.114606146280195	0.727770319986321	0.320705494373459	0.421921269743406	0.079957666666667	0.73097308185803
MOK	0.114530781109980	0.728076186913904	0.0645865213084009	0.919313088327921	0.120545333333334	0.543819286485342
CCNL1	0.114499727458634	0.728185882815925	0.0901890012610375	0.882249601181978	0.0232000000000005	0.950884054684253
OASL	0.114492537947073	0.728185882815925	-0.346155182033699	0.366023660299226	-0.0335423333333328	0.90616464808104
GRB14	0.114371319556188	0.728719640672422	0.148724162661069	0.783041584899534	-0.00122433333333259	0.996826292964745
CCDC144A	0.114368289511893	0.728719640672422	-0.180398798345946	0.722985510661031	0.263755000000000	0.342825265121927
MED16	0.114271464593790	0.728921789474859	0.215969336073857	0.64844945813838	0.148108000000001	0.4584541407185
SPAG5	0.114269447501646	0.728921789474859	0.197104090757044	0.689805951602859	0.0105693333333346	0.973906823746008
NUP214	0.114257809614457	0.728921789474859	-0.127239049735085	0.819791676572254	-0.0121153333333320	0.97167131557531
GJB6	0.114217726437677	0.728921789474859	-0.085664664291587	0.889114985071002	-0.123530999999999	0.575378149225683
TEX19	0.114197848780431	0.728921789474859	-0.0690707797788246	0.911928203725685	-0.0804773333333323	0.707966006319856
DCAF8	0.114177441280261	0.728937109744323	0.180910834893320	0.721866914445283	-0.569484666666666	0.0382989968555555
CAPS	0.114116961827483	0.729140140084521	0.211659195944608	0.659100072823846	-0.137112666666667	0.557700710345762
BSG	0.113949597762975	0.729553642305836	-0.29574515406122	0.47761182409076	-0.0109233333333327	0.97184501043952
SLC47A2	0.113930940987662	0.729553642305836	0.00788228587904857	0.990405674923721	-0.110666666666666	0.625510328921698
FBXO21	0.113925300679140	0.729553642305836	0.254074902808998	0.567694846153007	0.0372066666666673	0.891719978528798
PKN3	0.113918604596850	0.729553642305836	-0.266275595139905	0.538833471023229	0.0928513333333342	0.65498201079628

SEMG2	0.113901431770737	0.729553642305836	-0.254177398178758	0.567694846153007	0.122440000000000	0.550563293736455
ATP1A2	0.113862101117221	0.7297091975961	-0.256639149116392	0.564398995459794	-0.0369809999999992	0.886782227572443
DENND5A	0.113791500464738	0.729912004053216	-0.087945842671567	0.885848000380851	0.157879666666668	0.491853844738248
EPC1	0.113776744983448	0.729912004053216	0.274245625482639	0.520857714835269	0.0963216666666668	0.716517864791776
NPAS2	0.113772204543504	0.729912004053216	0.553544222952452	0.0587020070494168	-0.255581333333333	0.315859935065171
LRCH3	0.113755846778737	0.729912004053216	0.0322710965763915	0.96237922674393	0.234716333333334	0.381152785222697
USP48	0.113742774490713	0.729927127650243	-0.0107692306507273	0.98774587825743	0.0283230000000005	0.915761046438965
EP400NL	0.113668643943371	0.73023514055474	0.194457570925098	0.696294554451457	-0.0389896666666654	0.899845504615556
C19orf55	0.113576518670741	0.73059345665792	0.0110381592347757	0.98774001793194	-0.108470666666666	0.662857774732191
RBM5	0.113555284672746	0.73059345665792	0.0846931103147493	0.891309254245872	0.219065333333335	0.271872170281604
NUSAP1	0.113526103562760	0.730621261157777	0.150996604813246	0.778205892965504	0.136920000000002	0.525594458227739
UTP18	0.113507972189662	0.730621261157777	0.0702053981968403	0.910394157189308	0.090903666666668	0.68271691249359
ARAP1	0.113453841751433	0.730764766893159	0.0380744247317738	0.95468180561467	-0.229708999999999	0.306145675725963
CD300LF	0.113419523307250	0.730919639214411	-0.290922121374988	0.486880079690803	0.0672366666666671	0.830987625642722
SLC25A10	0.113378624663854	0.731107441347613	0.343427247786951	0.373956385371539	-0.167878333333332	0.386320548003750
NEK4	0.113356466577116	0.731107441347613	0.029685792634492	0.967057202964766	-0.0942446666666654	0.691560871680326
ARFIP2	0.113349091899255	0.731107441347613	0.132467069028054	0.809866082339655	0.0399880000000007	0.87796421789874
FLJ32955	0.113337121999814	0.731107441347613	-0.304698862534541	0.459197740121543	-0.0585890000000001	0.819026323982093
TOP2A	0.113324487577446	0.731119715306497	0.0418075561294719	0.951989245458556	0.0383766666666667	0.888476605628154
UTP3	0.113287570675631	0.731220868045555	-0.405572797994642	0.25473834018135	-0.196587333333333	0.398349038294794
ZNF804A	0.113272385008148	0.73124992450369	-0.148127653761255	0.784080588385358	0.226211333333333	0.520470860856309
ARMC3	0.113211885538413	0.731435362951468	0.0967215919155152	0.870857630743202	0.00596833333333313	0.987937064305561
CENPE	0.113186888941715	0.731474349566299	-0.124259309324724	0.825382714207684	0.0968073333333339	0.717898224756017
DHPS	0.113184424737635	0.731474349566299	0.234920664386790	0.607880535500655	0.100967000000002	0.649420534269272
HEATR6	0.113133845631815	0.731736164087102	0.192891747157671	0.699590438092569	-0.0977366666666664	0.652608565667976
MTRF1L	0.113116251372927	0.731781066797111	0.151824884588451	0.776343233798271	-0.0794321666666659	0.712688984009924
POFUT1	0.113070870272643	0.732008723002131	-0.083911647662228	0.892716669164583	-0.0341046666666654	0.890511682678135
NDRG1	0.112954629532999	0.732459174424375	0.208971489016179	0.665595909869988	0.0241850000000014	0.96868314803423
RWDD2A	0.112876037223975	0.732640819005133	-0.00299014684984400	0.995177530008123	0.158528333333334	0.58969057474622
VLDLR	0.112875048660870	0.732640819005133	0.289681105691748	0.488870852256136	0.194992333333334	0.536810332635776
METTL3	0.112856338470264	0.732640819005133	0.184099439915075	0.717872053085112	0.0613480000000005	0.815156860161333
SCLT1	0.112810450347748	0.732695208054167	0.0725068131421799	0.907556175729082	0.283654666666668	0.189095480222886
FAM122A	0.112794019625080	0.732696371912362	0.116457115827563	0.842082118939645	0.257111333333334	0.274121181062751
RUNDC3B	0.112740482766322	0.732776407300487	0.580288418387598	0.0418641499872607	-0.0917686666666653	0.729912257977231
ARHGEF18	0.112627701667775	0.733295039391707	0.073105532310138	0.906556667984864	-0.156428666666665	0.464309613067155
SLC35E2	0.112575202300158	0.733549581195831	-0.207409510217646	0.668625825887966	-0.203031333333333	0.365126455342703
NLRP4	0.112539320309376	0.733549581195831	-0.0276609204005639	0.969691489996656	-0.0951629999999993	0.719839702715467
MAP3K1	0.112535188447202	0.733549581195831	0.116678071875269	0.841809730226979	0.375930666666667	0.114869100066458
HIVEP3	0.112496965870339	0.733659412314188	-0.0769315476991875	0.901805121558435	-0.00394366666666663	0.990124830455448
MIP	0.112460668970540	0.733681833723204	0.0599174631854928	0.926038115028264	-0.00010566666666661	0.999729972650062
SPAG4	0.112432205887971	0.733681833723204	-0.0562187351836964	0.932459880997552	-0.085249666666666	0.704370299831112
LYPD3	0.112415405432131	0.733681833723204	0.0481624660449142	0.943089870033472	-0.0600103333333333	0.824257394304182
SYTL4	0.112396726712478	0.733681833723204	-0.0821305883297271	0.894840896036786	0.313238666666668	0.141989461843871
MS4A1	0.112347485956581	0.73379341906053	-0.112312022978872	0.847454473507881	0.099450666666667	0.665452409821856
HSD17B10	0.112327860776485	0.733851730230801	0.00422953741175858	0.994111873745308	-0.0931103333333337	0.659906583837718
CNRIP1	0.112239570854664	0.734164574335833	-0.116319725977563	0.84217383062547	0.0459493333333342	0.844766787725698
FBL	0.112228086415143	0.734164574335833	0.223748669389100	0.633972830146615	-0.119693333333333	0.607789868477511
USHBP1	0.112213780825212	0.734164574335833	0.0996638094557319	0.867603998426271	-0.145934999999999	0.630743046685015
ZSCAN30	0.112212204886833	0.734164574335833	0.00939274851840917	0.98909166696601	0.181661000000000	0.394715834237743
EIF2B4	0.112090517237140	0.734423647436009	0.0785850120184935	0.899281198071059	-0.0918213333333316	0.660936100042198
SLC5A7	0.112090312463863	0.734423647436009	-0.167759242504347	0.748242818061289	-0.0730533333333331	0.786460365353421
OR1F2P	0.111993978369968	0.734859394933363	0.175121960111906	0.733192115929191	-0.105185333333333	0.720071550811076
MYO5A	0.111991768476821	0.734859394933363	-0.0979622821211202	0.868852648210739	0.325205333333334	0.113460612523657
NCAPG	0.111890669794523	0.735099931953258	-0.0517934307832799	0.938168091114785	0.0145286666666679	0.961145507903953
FAM222A-AS1	0.111840867151119	0.735215310933672	0.237637337263351	0.602664863375033	-0.133371666666666	0.534060420301683
LHB	0.111806587611991	0.735369982784632	0.324387451470329	0.412678992507379	-0.0974356666666663	0.774104133965233
ESRP1	0.111790291262105	0.735406443090876	0.333286655992222	0.396894500849691	-9.76666666666697e-0	0.999729972650062
BRD1	0.111753889322807	0.735441596340961	0.143308728937222	0.79287526984225	0.0529223333333342	0.830902619187654
GALNT9	0.111752696238710	0.735441596340961	0.288645937444873	0.489813374112312	-0.0929166666666663	0.747245436362979
SLC31A1	0.111695520168033	0.73560547149416	0.196703792443618	0.69035327148255	-0.314565999999998	0.097583449418131
DIRC1	0.111598962196632	0.736028229835286	-0.0831648838568839	0.893287218356307	-0.0434399999999993	0.870489343487753
SAMD1	0.111573964464616	0.736051244827683	-0.175036048031953	0.733276438413475	-0.0811799999999998	0.736987042909868
STK11	0.111450765198942	0.736450752701266	0.270499763075732	0.529843880806928	-0.0446829999999989	0.853541852582193
NCOA5	0.111448705447762	0.736450752701266	-0.284438419216255	0.500253126094934	-0.152878999999999	0.471989789810989
CDC27	0.111327184835424	0.736967041909643	-0.249637935669340	0.575370998761354	0.152309000000001	0.555404460523821
RPS18	0.111257301289842	0.73728520849087	0.180071446744685	0.723716289903531	-0.077393999999999	0.754015160951226
PPP1CB	0.111213695539728	0.737430588859046	-0.0901468651223674	0.882249601181978	0.146643333333334	0.461494497197158
PPP1R17	0.111168351058219	0.737516724605651	-0.121597193327379	0.831310831659304	-0.145696666666666	0.685488482699362
PARP3	0.110988657190850	0.738116316186204	-0.0495280568995678	0.94156962266198	0.217529000000001	0.315543124546046
CD93	0.110969940545875	0.738116316186204	-0.392052336750298	0.279798157524773	-0.0371943333333331	0.914205835266868
CMC1	0.110965665353054	0.738116316186204	0.101544204138787	0.865707430582435	0.0978313333333342	0.69414649287771
ZIC3	0.110964762322778	0.738116316186204	-0.182369959414648	0.719419078472232	0.0123526666666667	0.972395918043445
FSTL1	0.110960867347593	0.738116316186204	0.111437400458443	0.848968422336654	0.318043333333335	0.113700575160835
ZFYVE27	0.110946479080262	0.738116316186204	-0.067541310850579	0.914119377247673	-0.235696333333333	0.241273257416922
DCUN1D5	0.110939364248803	0.738116316186204	0.0755726755889144	0.903517361619051	-0.0823573333333328	0.699953171808848
HOXB4	0.110861308902136	0.738300223105469	0.256492496564414	0.564430426783556	-0.0430893333333328	0.874908057954497
LSP1	0.110821379609667	0.738300223105469	0.366330323244331	0.328765884278342	-0.369626444444444	0.0832004986783026
ECHS1	0.110800649283571	0.738300223105469	0.0346953120764761	0.959404955370931	-0.160456666666666	0.425882414194225
LOC100128751	0.110792969946161	0.738300223105469	-0.0483342590695773	0.942820948997969	-0.220076333333333	0.536063442230912
VWCE	0.110762949265678	0.738416086545705	0.312638654445257	0.439245998265575	-0.0656773333333326	0.786973845403943

NLRP7	0.110727277032419	0.738517759878263	-0.087109930824305	0.887055939766881	-0.121454666666667	0.704370299831112
HEG1	0.110684768260843	0.738517759878263	0.0122545032872166	0.985532757224888	-0.236896333333332	0.580149967309238
CLUAP1	0.110656969803606	0.738517759878263	-0.0641964300393183	0.919923865590619	0.0829516666666665	0.70224156146001
FAM92A1	0.110627784934661	0.738528146076702	-0.0714767067400853	0.909000765759042	0.218958000000001	0.294300395506211
NRSN2	0.110546803683642	0.738848636460768	0.120934674116755	0.832982209969764	0.0672056666666693	0.769997189923324
FKBP15	0.110507306889261	0.73903763438726	0.0284976764619897	0.967951385746003	-0.021290999999999	0.935991946407154
ACTN3	0.110489883075845	0.739081584771845	-0.16787205139829	0.748216580540664	-0.00753699999999977	0.987119618784287
PDCD1	0.110404922369488	0.73918460994212	0.157357919583474	0.767770230680061	-0.0615599999999993	0.851568943534661
RBM26	0.110402335885823	0.73918460994212	0.0420120140766272	0.951989245458556	0.138704333333335	0.523762186913243
UBTD2	0.110397191697666	0.73918460994212	0.00683392300500953	0.99129507664977	0.330685333333334	0.0994203414387535
ZDHHC5	0.110388332663747	0.73918460994212	-0.0261055656854792	0.970632644458507	-0.100067000000000	0.669391045681572
TBCCD1	0.110320811428181	0.73930308114069	-0.325417490981487	0.409402478681488	-0.188519333333333	0.492100665301584
KLC2	0.110294932055941	0.73930308114069	0.195992229675897	0.692325064566574	0.129653666666666	0.513530370066639
PRM1	0.110263203973179	0.73930308114069	0.0565057009165021	0.93176886339657	-0.164848333333333	0.516217427447249
SPACA4	0.110251024431579	0.73930308114069	0.260791751883270	0.553351910336626	-0.101818333333333	0.681765404528423
GSTM5	0.110244657403957	0.73930308114069	-0.0551614277808435	0.93374866322822	0.0509573333333339	0.85272965579086
CYTH2	0.110219761105186	0.73930308114069	0.295699457061910	0.47761182409076	-0.0377333333333333	0.880427463662501
CLCN2	0.110215106356828	0.73930308114069	0.216989882227486	0.647113940467726	0.135443333333334	0.515490602743194
MEG3	0.110095009807556	0.739794031351068	0.271135496736388	0.528206193453632	-0.0450789999999996	0.886782247572443
MRPS11	0.110049557212041	0.739838770578987	0.0110021958555885	0.98774001793194	0.235343000000000	0.335884836278273
SNUPN	0.110041994782255	0.739838770578987	-0.164731144941900	0.753918587639435	0.0746583333333344	0.728708142582036
COLEC11	0.110001897911091	0.74003115367181	0.355976201943310	0.346552539524285	-0.185660666666666	0.625510328921698
EMX2	0.109954714290569	0.74003115367181	0.214297520965635	0.652991165086813	0.615756666666668	0.0185304620090284
ENDOU	0.109947679594470	0.74003115367181	-0.294700211879086	0.478800228795472	-0.0946039999999997	0.716517864791776
CARD18	0.109920290435375	0.74003115367181	0.0137300068174611	0.983872723592937	0.0815100000000002	0.815309621716298
BAHCC1	0.109831305851299	0.740179801775906	0.295984449714555	0.47721976572363	-0.268992	0.233528575266801
SH3D21	0.109829237374339	0.740179801775906	0.0427244058343523	0.95079489434051	-0.242498000000000	0.443378989641463
CDC42BPB	0.109786108023387	0.74021780005114	0.0796340348991269	0.898031113962014	0.0894053333333346	0.719304894613107
STXBP4	0.109748200478624	0.74021780005114	-0.149457829419612	0.78215339162259	0.0579666666666677	0.816134952712854
RBM39	0.10972740329288	0.74021780005114	-0.00013537692376240	0.999860038008296	-0.159316666666666	0.456799968498263
MKI67IP	0.109707784211145	0.74021780005114	-0.158086652334497	0.767373487136803	-0.148524333333333	0.481094083070727
ATAD2B	0.109660647071502	0.74021780005114	0.229488223488656	0.620183562399927	0.0484773333333337	0.888476605628154
CYP26B1	0.109583827194037	0.740545533789453	0.272529604090177	0.526175116911121	-0.0714483333333336	0.809409373518692
FLJ40288	0.109571119592456	0.740545533789453	-0.161346091740496	0.759429543907954	0.0376393333333333	0.880844178274306
CCDC88A	0.109513428890328	0.7407988139827	0.140055394211555	0.7976681602188	0.284070333333334	0.135650972591574
GRIN2A	0.109500396320271	0.7407988139827	-0.140567835494474	0.79693828563394	-0.0505969999999992	0.870133635372032
ZADH2	0.109492452366411	0.7407988139827	-0.194058565502053	0.696771449352284	-0.271156666666666	0.344866669191371
RBM41	0.109464888513034	0.7407988139827	-0.190501374832836	0.703920416197528	0.0539983333333334	0.813345796866388
BYSL	0.109436322634166	0.74086005296032	0.052489145648975	0.937768017367375	-0.409048333333332	0.0663250682564305
NTN4	0.109403079984764	0.740937634587546	0.113294396662027	0.845839106750552	0.599352333333334	0.0133271541061395
GGT7	0.109391831328567	0.740941189881061	0.188129795918562	0.707635058271509	0.0688850000000003	0.754702252946525
EXT2	0.109315528471354	0.741268208741642	-0.224644314114001	0.632376156419106	0.167230000000001	0.379096889881337
POFUT2	0.109275986340730	0.741420122058845	0.173662377693407	0.735662921375731	-0.0150409999999998	0.956567100065073
RPL36AL	0.109230633304156	0.741428661576256	0.141541518001035	0.795705351419283	0.0501333333333345	0.835752276645858
NAA16	0.109217359144319	0.741428661576256	0.0887660333254416	0.8841943128258	0.0129746666666676	0.970250590875359
TCEA1	0.109130669002342	0.741600894680418	0.164450593987658	0.754189116283047	0.164015833333335	0.399012880214421
ZCCHC5	0.109090917443422	0.741729995518199	-0.14203566692807	0.79450771779567	-0.0625973333333333	0.788201431210286
MYL4	0.109056489622489	0.741732147906891	-0.0263950661229502	0.97050493100216	-0.0184903333333333	0.94678260229888
SLC39A9	0.109046443070560	0.741732147906891	-0.0524105881535223	0.937831566264837	-0.117710333333332	0.608287291470058
ICAM5	0.109012121323129	0.741887021496831	-0.075650319384575	0.903515868940619	-0.102479666666666	0.624457186990323
BAX	0.108977085373193	0.742041066047009	-0.0519035108639026	0.938067483409541	0.0709370000000023	0.760810252329096
C1orf198	0.108955121734473	0.742041066047009	-0.0564380880779553	0.931891677569814	-0.113332333333333	0.639002829892966
ZNF621	0.108938408466979	0.742041066047009	-0.150470998190002	0.77904503981756	0.0488923333333335	0.873398028647672
FOSL2	0.108883974309931	0.74214205420677	-0.00429243037297665	0.99405344216434	0.139080000000002	0.533475280376028
GABARAP	0.108876929321334	0.74214205420677	0.290324489795753	0.488568550695131	0.147506666666668	0.471741080987819
KCNE4	0.108852877573894	0.742229596962668	0.398029318419404	0.268643641218029	0.416722333333333	0.0489929905034798
ZNF532	0.108797175270032	0.74238437459387	-0.376141345381203	0.309828524690001	0.369884	0.0802641563431964
SLC2A3	0.108751548275290	0.742419475653791	-0.0958981736936548	0.872499931194687	0.0778453333333341	0.759887627318327
POLL	0.108737740867476	0.742419475653791	0.358905932250064	0.342742323643068	0.157709666666667	0.420336551307248
PEX5	0.108718116465325	0.742419475653791	0.184226350565074	0.717436396325728	-0.00152499999999949	0.996601352479184
CAD	0.108716050123213	0.742419475653791	0.116548070055506	0.841887188288611	-0.0866766666666656	0.697096570878995
JOSD1	0.108697845076768	0.742419475653791	-0.0576894914520278	0.929758936765496	0.0782903333333344	0.720355976568153
KRT37	0.108695568192108	0.742419475653791	0.155170127712802	0.77066107821265	-0.193036666666666	0.487761541713127
KCNQ4	0.108560388539483	0.742845827422467	0.144905439969168	0.78939605015755	-0.165988999999999	0.62405411820911
SENP8	0.108543251633017	0.742845827422467	0.0571521573783301	0.930775493791569	0.222866000000001	0.271361162960907
VANGL2	0.108529904931806	0.742845827422467	0.0434726172567522	0.949521794694691	0.276886666666667	0.157416230067645
BCL3	0.108508626226168	0.742845827422467	-0.205519569112467	0.672695616352514	0.144429666666668	0.580149967309238
PIH1D1	0.108502212689627	0.742845827422467	0.232068856141593	0.613186703369632	-0.230596666666665	0.372679505356532
NSRP1	0.108491073130291	0.742848783091248	0.0556419968363323	0.933409139126562	0.0221296666666681	0.93278021276477
ZNF793	0.108411359548316	0.743230990878106	0.0684745214972889	0.912149839732916	-0.0130143333333328	0.969387309763954
FAM43B	0.108387220224453	0.74331912032709	0.0299359202799421	0.96664589910922	-0.159121999999999	0.466098392080126
LOC100131541	0.108364178722868	0.74340005746261	-0.35650218162581	0.345989229646028	0.194250333333334	0.313093065378681
TCERG1	0.108281881882424	0.743711731224701	-0.0960356603840014	0.872311810447594	-0.200086666666666	0.295708667353848
ZBTB42	0.108271998379043	0.743711731224701	-0.176960472181444	0.729794187782856	0.0608496666666667	0.873329245675404
SLITRK2	0.108271948530449	0.743711731224701	-0.0249180030214633	0.972871752171633	-0.060458333333333	0.819626240637794
CYFIP1	0.108258292079939	0.743711731224701	-0.238599936143776	0.600861960738847	-0.120141333333332	0.601372293483574
TAF5L	0.108241790689667	0.743711731224701	-0.363174649012748	0.336332757540532	0.119371000000001	0.626742243562072
MAGI3	0.108217052473718	0.74380379118686	-0.293603367898926	0.480614908214975	-0.0892176666666664	0.79082065189004
TAPT1	0.108167039789670	0.74400860695872	0.273819644736549	0.522187256281732	0.240400666666668	0.287154886987160
MOGAT2	0.108131711275857	0.7440828536689	0.0906193888037968	0.881574626542805	0.0175103333333336	0.949492888401158

FOXE1	0.108090631106981	0.744282005877855	0.292672104194440	0.483287060229305	-0.0851176666666665	0.685012925741245
CUL3	0.108040631601540	0.744539629037417	-0.0289431597587541	0.96782691865619	0.0845606666666667	0.692723628522594
SMG6	0.108000851693642	0.744644941454626	-0.131379787220381	0.811227868886268	0.108778333333334	0.630227347736008
WFDC9	0.107981806279779	0.744644941454626	0.00578057475480323	0.99251028338798	0.158313333333334	0.416412641573293
HTR1A	0.107934575851930	0.744839787558042	0.132734894662384	0.809588765729788	-0.0538059999999993	0.84060196086617
ZNF326	0.107930698022086	0.744839787558042	-0.215309787479060	0.650576096954446	-0.0409393333333312	0.874353540431336
ADC	0.107814341732255	0.74515948618741	0.0070058233989453	0.991263132354674	0.159036333333334	0.462024330182512
TBC1D4	0.107806151734895	0.74515948618741	0.155639161737342	0.769837060156515	-0.952026333333333	0.00421637924026936
SIX3	0.107799909096946	0.74515948618741	-0.252909624444037	0.568985221714455	0.0605603333333337	0.790626524535316
SCN10A	0.107796413963251	0.74515948618741	0.103847972488213	0.861570109612412	-0.066341333333333	0.780619140960946
NRARP	0.107735651167897	0.745347582169282	-0.313711436131926	0.438046311563112	0.0239933333333332	0.925969997068921
C12orf10	0.107670300793435	0.745434764726546	0.171298769694315	0.739682729639583	-0.0251829999999996	0.929919927302465
UBR1	0.107651938804772	0.74547602881476	0.0091103018060575	0.98909166696601	0.170904333333333	0.405689711040978
MEX3B	0.107631170009064	0.74551311025658	-0.306965470139031	0.453768045270662	0.207775000000000	0.290799750864839
WARS2	0.107624912313228	0.74551311025658	-0.302560607767324	0.462229407853213	0.0140406666666675	0.967879244181712
GPR82	0.107584490562066	0.74563795550912	-0.277006198302901	0.515587194962102	-0.0950219999999999	0.676024903789639
WASF3	0.107529182943774	0.745741487097789	0.214689894846741	0.652003104177839	-0.161604666666665	0.48174711509713
RTP1	0.107525959109899	0.745741487097789	0.132910717015118	0.809578511927685	-0.0990223333333326	0.775273488714107
DNAJB4	0.107468540785975	0.745899251627909	-0.0246647147111696	0.973370943834497	-0.066972	0.765339817431245
PTP4A3	0.107448472393332	0.745899251627909	0.0652540892623846	0.918295693698803	-0.0748673333333327	0.772139923817113
RFX2	0.10741714177116	0.746034519459776	0.0216857763735032	0.97536722461972	-0.0139199999999989	0.967798161116417
MCPH1	0.107392866989874	0.746053618196042	0.0159012912668757	0.982102356159698	-0.00327533333333279	0.993756838831997
GNRHR2	0.107310501429887	0.746383276702675	-0.0219879037191309	0.97536722461972	0.0093513333333333	0.97554220862608
PIP5K1A	0.107299131009956	0.746387803212645	-0.331506428561327	0.400099845811371	-0.0307899999999985	0.91148338318481
MLH1	0.107277301634317	0.746396414296798	-0.153521612463805	0.773553722129288	0.195654333333335	0.299695825369762
ATR	0.107270572189668	0.746396414296798	0.103391166355384	0.861949447881126	0.206984000000001	0.314504177654909
ATP7A	0.107265781189548	0.746396414296798	-0.227041226302980	0.62581495163125	-0.354388666666667	0.22316376350853
CTDP1	0.107253663836891	0.746405845274631	-0.146700923736131	0.786187758946602	-0.088897666666665	0.683326025818219
ZFP112	0.107183085784246	0.74665830269739	-0.0190504817735849	0.97832242527852	0.149498000000002	0.476636323255549
ACE	0.107153598104495	0.746746408562783	-0.161824632919533	0.758930446817487	-0.0245453333333328	0.945904449997854
ENDOV	0.10714015871428	0.746746408562783	0.228561405131203	0.622857918814168	0.0313946666666671	0.904955321847437
PRKAR2B	0.107137602987651	0.746746408562783	0.369135380550571	0.323453907657934	0.0653733333333346	0.792168081595258
HHLA2	0.107082742678738	0.746994521354267	0.095860440122407	0.872499931194687	9.33333333337795e-05	0.999729972650062
KIFC2	0.107052388976917	0.746994521354267	0.0765790798384794	0.902425586962571	-0.0100979999999994	0.980770032498656
ARHGEF10	0.107051598245448	0.746994521354267	-0.0592968003504767	0.92721549851508	-0.130707666666666	0.513024230069645
BAG4	0.106808302295895	0.748414163170639	-0.252068476459187	0.570473184967855	-0.0604066666666663	0.79462828227058
TMEM43	0.106759549929025	0.748593671018958	-0.0548640643906003	0.934255557320745	-0.0304393333333325	0.904955321847437
SLC2A2	0.106738835651450	0.748657862404605	-0.0492948682017148	0.941842562680095	-0.158716333333333	0.514076428873743
CTR9	0.106718900045742	0.748657862404605	-0.353939300732433	0.351837195910859	0.0638240000000006	0.77288533293416
RTN4RL1	0.106717707204548	0.748657862404605	0.237941055330022	0.602142642649819	-0.0899939999999998	0.73480251716231
IMP3	0.106664398966948	0.748871411747	0.126533584720385	0.821473247266105	-0.0947056666666658	0.668408423975192
DBR1	0.106660540105638	0.748871411747	0.0253614742815416	0.972264538231849	0.145546	0.536063442230912
ZIC5	0.106653079510141	0.748871411747	0.227245564739242	0.62581495163125	-0.106819000000000	0.636490807097692
CYR61	0.106641479857731	0.748877424752691	-0.139592879150709	0.797957231657366	0.586623666666668	0.0725137013180337
ADM2	0.106549667868509	0.749339213109643	0.0567939843636909	0.93142484205481	-0.127228666666666	0.640836927155663
KLHL26	0.106448035352482	0.749585266317357	0.0140266529013980	0.983461004910902	-0.0911199999999999	0.671492528470281
B4GALT5	0.106309837812808	0.750214596083824	0.0992553186612417	0.868037677687382	0.00326100000000115	0.993677488355417
RAF1	0.106282657932388	0.750319313127835	0.0186365992385297	0.97902968742576	-0.140705333333332	0.479014333115247
SSR4P1	0.106216297446523	0.750404168500845	0.0893027157251496	0.883460564717377	0.0568786666666677	0.838603547123369
STXBP6	0.106118978411728	0.750692893394691	-0.0127550412750948	0.985156239185327	-0.187377333333333	0.52041472962553
TTC28-AS1	0.106111228992073	0.750692893394691	0.126312096671807	0.822011156288607	-0.0999223333333331	0.667697330885416
RFWD3	0.106104520223185	0.750692893394691	0.0990066134858561	0.868042453226125	-0.138420666666666	0.557700710345762
NEFM	0.106101050003507	0.750692893394691	0.0543127305875114	0.93460245621169	-0.262451	0.255595078844894
UGDH	0.106056152901650	0.750893832903709	-0.316051548906973	0.433592288295757	0.401740333333334	0.0601168126740641
SERPINB13	0.106011603036415	0.751028232469648	0.0801248834885193	0.897577172229156	0.0246240000000006	0.945904449997854
VPS4B	0.106003761799334	0.751028232469648	-0.191666406398015	0.701560521853157	-0.00729033333333322	0.98256318768706
SKA1	0.106003608632354	0.751028232469648	-0.0127383425892514	0.985156239185327	-0.467506666666666	0.0312984093958667
RRP12	0.105925624811396	0.751301373313753	-0.0105929749394143	0.98774587825743	-0.305218333333332	0.109706365148046
MACROD2	0.105919222297522	0.751301373313753	0.00356362036415539	0.994730189431381	0.117015000000001	0.646350580563869
TEAD1	0.105868627938307	0.751423005233297	-0.0363999456527317	0.957597361653107	0.215919333333335	0.375861361444218
RAB28	0.105705567100865	0.752090885818024	0.142731409368462	0.794004622916083	0.226029666666669	0.446756640099031
ANKRD27	0.105702677659916	0.752090885818024	-0.0266552615794311	0.970207612777188	-0.512303999999999	0.0686922836072744
ZNF574	0.10565648621885	0.752183656555968	0.0733485119310335	0.90588212168674	-0.0493876666666666	0.88487182863547
AIM1	0.105613006090484	0.752301760973892	-0.391693153529071	0.280141098619812	0.332101666666667	0.25804020045686
CCDC91	0.105588170438015	0.752301760973892	0.207418263480762	0.668625825887966	0.121582666666667	0.541270965348748
C8orf46	0.105586511113380	0.752301760973892	-0.266276257343825	0.538833471023229	0.111557333333333	0.60217016768875
FLVCR1	0.105507791497733	0.752315062743478	0.124438195694929	0.825021694154683	0.276929000000001	0.165655690750296
DNHD1	0.105486957447787	0.752315062743478	0.0587242575753789	0.928472484757714	-0.0051433333333332	0.987499363290182
OS9	0.105446215547351	0.752512112518203	0.221050556422901	0.638286782439236	0.0689733333333341	0.755697432495201
SIGLEC11	0.105417251373183	0.752597049912045	-0.00787598667915182	0.990405674923721	-0.235702666666667	0.220405584107617
PAPD7	0.105239686152713	0.753166098068876	-0.283316616541466	0.502129505146473	0.128105999999999	0.672980097690282
ZNF518B	0.105202679732205	0.753292038307237	0.148805340459913	0.783041584899534	0.146835333333335	0.462024330182512
TECPR2	0.105199122510237	0.753292038307237	-0.0376627411863892	0.955365651737247	0.0583460000000009	0.837732816472933
FBXO5	0.105095671322307	0.75369915939647	0.138919725701317	0.798791960356084	0.215643333333335	0.27721812275707
PRR3	0.105082751048593	0.75369915939647	0.271290296847087	0.527984832801825	-0.279656333333333	0.149612190211527
ZNF334	0.105072963346072	0.75369915939647	0.00816358325941404	0.990248011892148	0.110588333333334	0.670113640649168
SLC22A18AS	0.104962348558573	0.754144340680844	-0.112517736824077	0.847191484945575	-0.00738366666666667	0.983159241812861
NEUROD1	0.104905223120285	0.754308889845413	-0.157583158212737	0.767770230680061	-0.00839166666666637	0.980779858267938
CDC34	0.104844989685404	0.75452504632429	0.135159907390909	0.807470535721699	-0.0563103333333327	0.808657226656366
CCDC85B	0.104805477638837	0.75452504632429	0.0688993513028995	0.912149839732916	-0.118888666666666	0.583036318848746

ZFAND1	0.104784145930905	0.754542852465965	0.0950570256392016	0.872962271153999	-0.032143999999999	0.898863720449539
FOXR2	0.104765258910312	0.754586138086428	0.146534696180663	0.786539458660834	-0.0334316666666657	0.899614658619676
ZFP14	0.104659695388234	0.755145895013777	-0.297044356604238	0.475138844815997	0.378960000000001	0.084174670978616
NFKBIL1	0.104591240779834	0.755247629149678	0.460909910351465	0.161789531338644	0.144884333333335	0.522830837265953
WFDC8	0.104524631959916	0.755544331915507	0.0883613933785217	0.885142944149088	-0.208034666666667	0.27266488177
S1PR2	0.104493274993678	0.755609960944329	0.136305729141557	0.804864448119512	-0.106923333333332	0.73379884333301
SGOL2	0.104441656943197	0.755751315178799	0.0288183729915934	0.96782691865619	0.190165333333335	0.458006502146897
GTSE1	0.104439694436305	0.755751315178799	-0.0253223495673846	0.972285580751433	0.140877000000001	0.494484578122119
BUD13	0.104425578951843	0.755773889447617	-0.319349422456537	0.426142369255984	0.0538483333333334	0.821421133422134
FAM111B	0.104293766752688	0.756228302542497	-0.0719014766559696	0.90837606217316	0.248963000000001	0.245948767014956
PITPNC1	0.104265671266871	0.756230956393264	0.239742408486024	0.598468009092338	0.135661000000001	0.600146208272329
CAPSL	0.104224305846089	0.756393722917556	0.0591191010036202	0.927663042275419	0.0118620000000005	0.967441088545168
RTKN	0.104190807627947	0.756446230361476	0.314271236950936	0.437675147805387	0.0660893333333336	0.766247450031517
CNR2	0.104167764014699	0.756446230361476	-0.155685988211053	0.769837060156515	-0.00550099999999972	0.991625835822726
RPUSD3	0.104143577467298	0.756446230361476	0.182363124165096	0.719419078472232	0.0262563333333343	0.926989565501108
ARG2	0.104094224023109	0.756686870962441	0.279245387997456	0.510629614226119	-1.48966733333333	0.00311122808725912
DNAH17	0.104056679838473	0.756723186014334	-0.206428081035763	0.671220485448239	-0.0106911111111110	0.973686596735315
PRSS23	0.103896086363407	0.75750558269117	-0.151221696324696	0.777604486471648	-0.155697999999999	0.426484252681993
CNTN5	0.103886401683485	0.75750558269117	-0.191930633229563	0.70143383946588	0.0141703333333339	0.959910474847156
ZNF324	0.103872187156410	0.75750558269117	0.148217563578610	0.784080588385358	0.0636626666666668	0.77288533293416
CIB4	0.103871322935271	0.75750558269117	0.232235383907721	0.612658112747496	0.0742000000000006	0.825729064695823
ACTL8	0.103824807726978	0.757547696634754	0.0238306481883089	0.97479225206569	-0.141640333333333	0.580149967309238
N4BP2L2	0.103824192437094	0.757547696634754	-0.220798134331335	0.6389751639565	0.201703333333334	0.354143788711296
RASA3	0.103790566647304	0.757698162452286	0.0861809313678856	0.888051139742697	-0.438894999999998	0.106113992549159
SEC11A	0.103367377570664	0.759566391765485	0.00407197111772219	0.994485841304070	-0.0786699999999999	0.743876813090318
AKT1	0.103357234596664	0.759566391765485	0.0145825682417783	0.983444353656142	-0.0676709999999988	0.775273488714107
TCF19	0.103356226419387	0.759566391765485	-0.0208881559383069	0.976330597778846	0.0893970000000008	0.70581327936471
THAP6	0.103248778515222	0.759851097298433	0.0341502617180191	0.95969480339665	0.100065000000001	0.636605507883166
NACC2	0.103145213211883	0.760390360663275	0.376208938961068	0.309828524690001	0.0971286666666675	0.636490807097692
DCLRE1B	0.103113263032240	0.760529926266164	-0.270797284703156	0.529082314945774	-0.238229666666666	0.481098006194455
GOLGA4	0.103092162248577	0.760540522602517	-0.0889004984967655	0.88392988973275	0.282801000000001	0.205940838078582
PRKCZ	0.103086370353390	0.760540522602517	0.110736244315249	0.849357727620213	-0.108495666666667	0.61890657732825
PRRG3	0.103079624533879	0.760540522602517	-0.112355378772675	0.847454473507881	0.222830333333334	0.276850820859682
NUF2	0.102977468574032	0.761000587676047	0.09611845231677	0.872262037328287	0.106348333333333	0.6846855637534
FZD8	0.102899923675194	0.761369273002455	0.342876055483702	0.374882411235977	-0.104102333333333	0.689966051286086
GAPDHS	0.102829191413362	0.761553170575446	0.195467735908149	0.694043352975239	-0.0288453333333334	0.930547487203917
DAZAP2	0.102769462879491	0.761875039928547	-0.154701082728281	0.771310656070265	0.0414100000000006	0.868127376045146
GRSF1	0.102660827171811	0.762027448542839	-0.088069096776932	0.885848000380851	-0.229465333333332	0.226573267757968
LYPD4	0.102567810538166	0.762497682235044	0.0052052328299196	0.99251028338798	-0.0212313333333328	0.954736472265512
CARKD	0.102494080789966	0.76263121890583	0.142644181487212	0.794004622916083	0.0409116666666667	0.899845504615556
TRAK1	0.102353185526268	0.763065398562093	-0.119511609456265	0.836013196419542	-0.0645143333333322	0.772139923817113
TGFBRAP1	0.102331189960533	0.763133080236898	-0.0878380834583839	0.885957779199557	-0.097630333333333	0.708763045307279
PROP1	0.102291141948655	0.763133080236898	0.114848976531596	0.844162087612813	-0.151365666666667	0.501839684921699
ODF4	0.102264455113293	0.763227383090455	0.138782835527991	0.798926084240744	0.0531840000000005	0.848281050282272
STARD13	0.102154618438053	0.763741803623969	-0.0830340019949075	0.893446201797764	0.379984000000001	0.0763141016054713
LGALS1	0.102142051918808	0.763750351597364	0.167721035778842	0.748278912029834	0.482133333333335	0.131369372059974
C1orf216	0.102079974266809	0.764001342827076	-0.0947775767223904	0.873804326353411	-0.0733839999999994	0.748715075113364
MYOZ1	0.102071777983004	0.764001342827076	0.183711479580924	0.718067440349544	-0.0989876666666663	0.704904955554555
LOC100130264	0.102031178562972	0.764117763610336	0.0716134122940512	0.908620839893657	-0.0336133333333329	0.913644671932166
BCL6B	0.101992091961651	0.764244058962858	-0.30463032753272	0.459197740121543	-0.134838000000000	0.547839860241723
HOMER3	0.101904178732506	0.764657660995579	0.120714065365285	0.833602134657439	-0.207307666666666	0.344576368488222
RHD	0.101897039580895	0.764657660995579	-0.262004752071354	0.5499549817547	-0.075437666666667	0.729180627146151
MKI67	0.101692365694146	0.76559586607964	0.0729669733755113	0.906592046281011	0.00595300000000071	0.987937064305561
GHSR	0.101669380713095	0.76559586607964	0.140795760696715	0.796714214272984	0.167867666666668	0.492100665301584
ASB16	0.101649625137456	0.76565090122481	0.0153239613220999	0.982725338620588	-0.0613700000000001	0.862156400976549
HEY1	0.101607232004775	0.765789006666739	-0.243947133658185	0.589344144320884	-0.123702333333332	0.567454544833907
ERLIN1	0.101566790679866	0.7658420030114	-0.137130098916885	0.802818136248682	-1.19076833333333	0.00347244136732606
CENPO	0.101493696601774	0.766084819379979	0.341036284099115	0.378272273903863	0.0227896666666680	0.93901998985171
SPG21	0.101461633079356	0.766084819379979	-0.162642680401326	0.757524249707797	0.0416910000000018	0.867537917755829
ROM1	0.101455406517272	0.766084819379979	0.0336010311107801	0.960670911981755	-0.227385666666666	0.328178286792292
DUOXA1	0.101376757633633	0.766530969087398	0.137304950646514	0.802366200871977	-0.0675996666666657	0.797534995180308
ABI1	0.101288924833729	0.766897293484357	-0.132078925663200	0.810300414153187	0.0879950000000007	0.716517864791776
WWC2	0.101236491504462	0.767031228710085	-0.0308236865698383	0.96529904314994	0.14964266666667	0.567468995323405
HUS1B	0.101222132331383	0.76705540230893	0.232227208471053	0.612658112747496	-0.00708899999999964	0.98256318768706
C15orf38	0.101185214570396	0.767062958752361	-0.0106799978993188	0.98774587825743	0.0204446666666677	0.943391353769848
ROMO1	0.101140795179818	0.767111547279507	0.0649437682376985	0.918489100363712	0.225930333333334	0.390561931914011
TNFRSF13B	0.101140158079224	0.767111547279507	0.147061017504339	0.785734908309155	-0.179064999999999	0.570550813984136
DDX31	0.101049720865458	0.767486373681957	0.139959527568875	0.7976681602188	-0.196734000000000	0.310277378475854
NUP133	0.101015845071476	0.767568595611581	-0.156983769985464	0.768401006769299	0.139588000000002	0.583036318848746
MOV10	0.100970784257303	0.767718122558895	0.0107681742373703	0.98774587825743	0.138878000000001	0.483840860674189
DROSHA	0.100949725439059	0.767722338083145	-0.330352916281753	0.402284079938133	-0.148927666666667	0.467448228941098
CCNC	0.100926636025488	0.767803825354933	0.126733534663562	0.820923134671156	0.262031666666668	0.190727419491685
EIF4A2	0.100881181475635	0.767962047781637	-0.133943619280796	0.808567812988011	0.0058314444444454	0.988133186272453
CARD8	0.100861781750498	0.768019320476483	-0.0583501249758964	0.928725686060612	0.0575190000000011	0.798038248389079
TMSB10	0.100771135375646	0.768334047011382	-0.287420495272927	0.493442130049965	0.411891666666669	0.0423240321874334
LHX1	0.100754566520986	0.768372741419125	0.238177816542134	0.60184463266698	0.0395066666666672	0.87709801222343
DNAJC7	0.100699418170072	0.768664657317216	0.139394460308333	0.798549995714663	-0.392115333333332	0.0571777796149344
NAP1L4	0.100656374060914	0.768774305366964	-0.0793422917348366	0.898323801514594	-0.0272239999999992	0.915761046438965
TAF1A	0.100650690989831	0.768774305366964	-0.0462960008436819	0.945019358156718	-0.0316033333333324	0.929556143651339
MLF1	0.100566828579347	0.769184663557339	0.133604947107700	0.808933084408149	0.126873000000001	0.557611184484231

RACGAP1	0.100448864085368	0.769678776236701	0.112696643266527	0.847080600929454	0.0515900000000007	0.82667899725046
RET	0.100431256039285	0.769724285688844	-0.0999645271330773	0.867290404865575	-0.0660466666666661	0.782886750366221
BRD9	0.100394557508400	0.769764313513796	0.287347944092805	0.493633004722576	0.000205000000000102	0.999244012762373
LIG3	0.100335227684646	0.769946760684123	0.223414880540853	0.63471624724109	0.0194583333333347	0.948386471366378
KRT71	0.100282303798607	0.770030518344802	-0.0213483673332746	0.97554687597484	0.00253200000000095	0.994114279008565
GLP2R	0.100270525470164	0.770030518344802	0.176242762299694	0.730407919148629	-0.0803626666666663	0.719304894613107
IL1RAPL1	0.100237814558120	0.770030518344802	0.290024200390772	0.488870852256136	0.449954000000001	0.0490477538807126
KRT73	0.100222987337820	0.770030518344802	0.099793520366839	0.867558388686851	-0.0170886666666665	0.95197590164177
IKZF3	0.100222787527416	0.770030518344802	-0.368980449662232	0.32362669860138	-0.269407666666667	0.250411220073775
RGS1	0.100203151825555	0.770030518344802	-0.38768695840579	0.286134842484356	0.0218310000000007	0.940880027113653
PLEKHH2	0.100190549766669	0.770043195315775	0.522045479596846	0.0876859028054308	0.466714000000001	0.031687055072673
NFU1	0.10016023485088	0.770172106614474	-0.0702016132758774	0.910394157189308	0.0938723333333346	0.704370299831112
RAB2B	0.100048132443047	0.770487628217194	0.0757308575800723	0.903510413986113	-0.0470666666666658	0.883775402587518
KLK12	0.100016687427797	0.770584163312265	0.0530228387089484	0.936712858731269	0.0423983333333338	0.885518223441274
APOL3	0.100012081995449	0.770584163312265	-0.331763918927442	0.39948883833865	-0.143375999999999	0.523762186913243
ABL2	0.0999455218159168	0.77074088498422	-0.24747166386154	0.579745218830252	0.0864509999999999	0.760710284799861
TBC1D17	0.0999118658237508	0.770809315012867	0.144041898542945	0.791654392218254	-0.133395000000000	0.551125574415782
THAP2	0.0998840889639536	0.770809315012867	0.079810131687897	0.897892885174022	0.0880590000000006	0.833065801030836
MTPAP	0.0998642117199971	0.770809315012867	0.074279575439516	0.905205116779936	-0.0562326666666665	0.816337381174918
TAB1	0.099856507558245	0.770809315012867	0.241253874016551	0.596019873098193	0.114388333333333	0.58043274631496
TAS2R4	0.0998478079983713	0.770809315012867	-0.051118253942969	0.939355624240953	-0.0666763333333331	0.766256558155699
BDKRB2	0.0998390936154784	0.770809315012867	-0.230274741031512	0.618707227156037	-0.163632666666666	0.467805195600209
FAM205A	0.0998227563923974	0.770846523386548	0.1645417596945	0.754189116283047	-0.0203046666666665	0.949401371056423
HAND2	0.0997664955204135	0.771005697347159	0.175656414589899	0.73187914820204	-0.115352666666666	0.637570808030689
CLK1	0.0997177916597822	0.771185249577256	0.0762966333445493	0.903025028995418	0.26958	0.187638103900419
UBE2E3	0.09970258462407	0.771215047426184	-0.0698922970062613	0.910885619751085	0.145307666666667	0.471680039257208
MTNR1A	0.0996713246841719	0.77125743681416	-0.107722188128155	0.856450662791083	-0.156561666666667	0.514076428873743
BEGAIN	0.0996641319977514	0.77125743681416	-0.00730973852051387	0.990873555460625	-0.0622886666666664	0.893151737593092
TIMELESS	0.0995601332511001	0.771869627813235	0.106193438869592	0.858601715235073	-0.160611999999999	0.410880322058341
CRISP1	0.0994849671620767	0.772281153289143	0.0766842742041388	0.90221365266889	0.0226139999999999	0.932154136447394
EGR4	0.0994547402717608	0.772281153289143	-0.232739050477748	0.611941461795609	-0.144981000000000	0.639002829892966
HOOK3	0.099423848058027	0.772376580770484	-0.130250976482187	0.812271254601992	-0.0264599999999993	0.930211818640573
BAZ1B	0.0993721032103734	0.772403448622169	0.154667289558584	0.771310656070265	-0.105920333333332	0.668862273626329
SYNGR3	0.099301793476923	0.772724102045008	-0.152923122563114	0.774656266040138	0.0218276666666675	0.949295980201336
UGT8	0.0993000042994072	0.772724102045008	-0.314193614708861	0.437675147805387	-0.0177986666666663	0.95805797537369
PPFIA3	0.0992907451390072	0.772724102045008	0.276177955152426	0.517549833373363	-0.158125333333332	0.458006502146897
CRTAC1	0.0992520519047013	0.772724102045008	0.0958495381412787	0.872499931194687	-0.0340963333333323	0.948768037250156
ZNF37BP	0.099248568835999	0.772724102045008	-0.0569330304117287	0.930901437476692	-0.138833666666665	0.513530370066639
MLF1IP	0.0991909794023122	0.772844468507672	0.0837605320765642	0.892833228561937	0.263754666666668	0.233528575266801
CLPX	0.09918756462164	0.772844468507672	0.036634180351193	0.957119663560524	-0.0337849999999992	0.899614658619676
TULP2	0.0991712316363635	0.772881658475007	-0.179251479601502	0.725276394376351	-0.231460333333333	0.410662263424299
CDH19	0.099134105434258	0.77288643073837	-0.104900388113884	0.86067831050617	0.0235120000000005	0.931446587444396
DSC1	0.0991278589917292	0.77288643073837	0.341834280078705	0.377158872804021	0.0480280000000004	0.884064277940346
CD247	0.099025293037599	0.773279369327846	-0.296673730582695	0.475941682821927	-0.220108999999999	0.458006502146897
G2E3	0.0989159306083433	0.773856767819903	-0.063782614873273	0.920521809324956	-0.00120533333333321	0.997134925912045
FAM154B	0.0988916777290875	0.773875959361554	-0.158944293234096	0.765528269194987	-0.135672000000000	0.538968176654309
EFHC2	0.09882905480153	0.773947885173774	0.0479581983153071	0.94314236584312	-0.0237696666666666	0.926483816193244
TMEM173	0.0988167393876043	0.773947885173774	-0.318998349589509	0.427443025560672	0.0455666666666674	0.874353540431336
GALK1	0.098802081020115	0.773974101128174	0.205222774353578	0.673296856274784	0.0070280000000009	0.985266742780184
ZNF154	0.0987632498439864	0.774158832312489	-0.207800346577530	0.667695745454683	-0.113182333333333	0.578064656314161
LPCAT4	0.0987428120849956	0.774222946463293	-0.233798295118073	0.610242151790862	-0.0172759999999994	0.962552669872023
PMCH	0.098701775822776	0.774284195776364	-0.0359460578964299	0.95830581556695	-0.0415083333333333	0.868791301779828
HYI	0.0986889284578662	0.774296667227657	0.200764911306550	0.680524286175603	-0.109144333333333	0.589692872920383
ZNF391	0.0985636815675002	0.774760122188811	-0.172978814004548	0.737510221688958	0.104025000000000	0.654911833752806
EFNA1	0.0985544378829795	0.774760122188811	0.16409508409238	0.754243267462143	-0.557279666666665	0.0286129614738536
CDC6	0.0985499595196855	0.774760122188811	-0.0785958024647835	0.899281198071059	0.0430600000000013	0.87709801222343
MUSK	0.0985436308609191	0.774760122188811	0.169994078235610	0.742983788546072	-0.0434456666666663	0.856854911614454
EFR3A	0.0984762218164085	0.775053191412877	0.130540255427162	0.812271254601992	0.177312	0.571070613702241
NACAD	0.0984425725960214	0.775073293525489	-0.0021879863160916	0.996532780077613	-0.0934543333333325	0.742738366366583
DCAKD	0.0983538455688039	0.775445401595894	0.149620248762893	0.781645288607494	0.283757000000001	0.220405584107617
CAMK2D	0.0983146615561114	0.775632452378212	-0.0139183518290281	0.98369342899447	0.385183000000001	0.0660125264725009
KIAA1211	0.0982217129417026	0.775842293180703	-0.107709068589673	0.856450662791083	-0.286373999999999	0.219457597945103
HMGA1	0.0981781966278918	0.775842293180703	-0.283850012303248	0.501172426622519	0.0401756666666665	0.89052375260746
USP19	0.0981624258770714	0.775842293180703	0.305197276372642	0.458013550008763	-0.0520563333333309	0.819757926662461
ZMYND19	0.0981412674917503	0.775842293180703	0.0782519510537021	0.899647854618521	0.0115116666666682	0.9715865162904
TMOD3	0.098080523373159	0.775977123104018	-0.0271697904482912	0.970207612777188	-0.168839999999999	0.47541375968935
KCNV1	0.0980683805203646	0.775977123104018	-0.146799183269312	0.786187758946602	0.0366039999999999	0.904201124848034
AVPR2	0.0980595960013467	0.775977123104018	0.201261265650304	0.680054545720343	-0.000426333333332966	0.99876308497056
ARFIP1	0.0980407726362008	0.775982073356616	-0.252494777873747	0.569562951596314	-0.871128333333333	0.00580262445524077
DCAF17	0.0979829690257598	0.775982073356616	0.227090308178162	0.62581495163125	0.133849333333335	0.517547647029412
NIPAL4	0.0979795834620397	0.775982073356616	0.112170546715635	0.847454473507881	-0.0957906666666655	0.696194439936211
SWI5	0.0979721434015133	0.775982073356616	0.282482265801837	0.503412540505745	-0.0274306666666658	0.919891803741744
RHOD	0.0979629734432997	0.775982073356616	-0.330979222466427	0.40155437088421	-0.0844396666666665	0.852020205196772
TMPRSS12	0.09792645732268	0.776058197058579	0.159026129041700	0.765392156938033	-0.0545059999999993	0.842158667368744
GGA1	0.0978948603857156	0.776058197058579	0.184577682568184	0.716469623322814	-0.0371463333333323	0.879843042066371
TTC38	0.0978644829047437	0.776138963858525	0.256546579977427	0.564430426783556	-0.314075999999999	0.130824456840031
TTYH2	0.0978489257440241	0.776154884552076	0.153372015942675	0.773870060547252	-0.184946	0.493413216077427
ZP4	0.0977886303353365	0.776426576469089	-0.040602374143943	0.952426531595115	-0.0224093333333328	0.952505889082874
SCAMP3	0.0977677254467481	0.776493762488144	0.0149892415994160	0.982921609770846	-0.0298013333333325	0.905448870718489
WDR62	0.0977198183528688	0.77652859911607	0.145990226967707	0.787402233709509	-0.0239459999999989	0.937717709824356

TSSK3	0.0977069630758803	0.776543067669788	-0.0355734986504831	0.958689318480951	-0.193693333333333	0.338804872769334
KBTBD6	0.0976830249115415	0.776630119619023	0.0521726419308485	0.938067483409541	-0.328206999999999	0.106113992549159
PPAP2C	0.0976477843488163	0.776791195207255	0.0184542165443117	0.97902968742576	-0.174702	0.402139666857366
CCDC66	0.097601936104656	0.77700380114971	-0.111516000165319	0.848875498955953	0.269236333333335	0.436132693279062
MOB3C	0.0975940300936818	0.77700380114971	-0.257219740333240	0.563116080558022	-0.0472116666666666	0.846906282289456
ROR1	0.0974965912590182	0.777421711511432	-0.435815325469920	0.196496490861455	0.309945666666667	0.230634542108013
EIF1	0.0974703499108204	0.777421711511432	-0.22656234705714	0.626953708249384	0.0241900000000008	0.927422712012045
SRPK1	0.097433191283762	0.777432656418967	-0.126134237175728	0.822164329801713	-0.146651000000000	0.480330266927924
TMSB4X	0.0974327327325584	0.777432656418967	-0.462575299680069	0.158924094451500	0.531469500000001	0.0216393910603585
CIDEC	0.0973547550359164	0.777741936737473	-0.142663183243903	0.794004622916083	0.0234623333333340	0.929594956567283
SMARCAL1	0.0973284697094853	0.77783660665393	0.00639225874881318	0.991853352023739	0.0269046666666676	0.923293854892615
PTDSS1	0.09723338332993	0.778060012411121	-0.0345387340098596	0.959404955370931	-0.181858999999999	0.353471671280201
ZNF781	0.0972265278065964	0.778060012411121	-0.227714563400746	0.62473940477579	0.100712333333334	0.659124451108183
IL27RA	0.0972261465370179	0.778060012411121	-0.187122196823193	0.709833349594307	-0.122716666666666	0.536063442230912
RLTPR	0.0972198374574727	0.778060012411121	0.285861435549710	0.496429042723156	-0.197500666666667	0.462024330182512
OSBPL7	0.097191165951217	0.77812453881578	0.194505582323974	0.696263722143486	-0.248676666666666	0.194304107445554
LRFN5	0.097173197730809	0.77812453881578	-0.229463772028171	0.620183562399927	-0.0557046666666664	0.861746271641311
USP18	0.097152614587014	0.77812453881578	-0.394590115503357	0.273906516278198	-0.0376866666666666	0.904788907158467
NUCB2	0.097045976709676	0.778390000770194	-0.136212324567342	0.805254942117114	0.296110666666667	0.175583831213141
NANOG	0.0970434173562292	0.778390000770194	-0.139816879000326	0.797957231657366	-0.0946396666666664	0.670071773006358
LACRT	0.097013880542853	0.778432941009002	0.187768022049882	0.708377771996406	0.0473893333333334	0.855533429360007
ISLR2	0.0969596704982554	0.778651453175387	0.181659947886782	0.720537767137049	-0.055953666666666	0.811039304529434
CPSF7	0.0969592183122597	0.778651453175387	0.0291860209912211	0.967686723974196	-0.109059333333332	0.609928365742218
SPNS2	0.0968957477967056	0.77879075277873	-0.0788180264042772	0.899154242391172	-0.148617333333333	0.466098392080126
MRPL12	0.0968953756184003	0.77879075277873	0.341445599161682	0.377754622823514	-0.005967666666666518	0.987134800094338
MRO	0.0968238491918487	0.779119659069235	0.257201429957049	0.563116080558022	-0.0244239999999996	0.924674647382585
HIPK1	0.0967524130348273	0.779344043716784	-0.118723425570311	0.83731413798382	-0.0546426666666663	0.846861040972959
UBE3A	0.0967389262043992	0.779344043716784	0.0101722270157538	0.988384322540392	0.00326133333333353	0.992405253506922
SLC27A3	0.0967274012087246	0.779344043716784	0.0745474003126074	0.904832407812985	-0.0834743333333328	0.715392853963174
SUMF1	0.096710186456129	0.779344043716784	-0.308747967643583	0.449759364777049	0.312648666666667	0.108684800104187
FGF17	0.0966825142930797	0.779344043716784	0.293428678181862	0.480778950366873	-0.196995333333333	0.41903055047835
OXNAD1	0.0965763408697444	0.779834346711265	-0.341302320814310	0.378027240426398	-0.114908999999999	0.592269447222472
LOC442028	0.0965415769134873	0.7799922556193	0.391883724043201	0.280021017230138	-0.130192333333333	0.728429595651334
EGLN1	0.0965100926465947	0.780062162009313	0.183221324226534	0.718067440349544	0.0626290000000009	0.804464256710878
NEK9	0.0964848687457226	0.780062162009313	0.0383894185720629	0.954545200586067	0.0217790000000009	0.945904449997854
PCID2	0.0964776978130168	0.780062162009313	-0.0760771709234222	0.903296922694955	-0.215980833333333	0.343729765439488
UTP20	0.0963977379856846	0.780407259952561	-0.166028701395034	0.751830402832405	-0.532291666666666	0.0492053003500414
FBXW8	0.0963930540934214	0.780407259952561	-0.0243434639328168	0.97380018049607	-0.0366409999999998	0.891410328517388
ZNF749	0.0963276681456037	0.780695962174721	0.156702070040442	0.768752873219075	-0.0488653333333328	0.895093529187836
GCK	0.0962987188330486	0.780777363716665	0.113971183007458	0.844272085764738	-0.0498416666666667	0.885345988320645
HOXA10	0.0962939514785807	0.780777363716665	0.252226562746691	0.570465803488487	0.198336333333334	0.319387382047648
TAS2R40	0.0962008541992807	0.781247494844351	0.239833905530523	0.598134271398487	0.171175333333334	0.443072320689246
ZNF260	0.096173743239399	0.781267675219179	-0.154796452914339	0.771310656070265	0.0461983333333344	0.866601971778042
OTX2	0.0961179876922994	0.781375777391933	0.0273890897839968	0.970045676424287	-0.0276223333333328	0.9273350681791
WFDC1	0.0960482999143455	0.781418902408474	0.0994692397783326	0.867651972215247	0.169852666666668	0.4261850174154
CKAP5	0.0960452384230834	0.781418902408474	-0.14178364900059	0.79509253036971	-0.0259299999999987	0.922697964464038
SLC25A39	0.0960385678222083	0.781418902408474	0.236779887533422	0.604321362942628	-0.0028296666666656	0.993677488355417
LYPD5	0.0959647777424266	0.781678403700881	0.158929106682204	0.765529751054686	-0.130294999999999	0.653495412055016
HGS	0.0959135606543707	0.781943976732173	-0.0609289447596897	0.925004281742147	-0.071171	0.743291289804212
ZNF546	0.0958022409507617	0.782231553914146	0.134285642702802	0.808009477165608	0.355768333333334	0.332423309568816
DPP4	0.095794134772854	0.782231553914146	0.359574424973461	0.341510139061518	-1.286193	0.130729956218524
CADPS	0.095700688148368	0.782362439326206	0.195183426254300	0.694852194384891	-1.09926266666667	0.0233091007249086
ALOX15	0.0955897173327943	0.782810154405707	0.0691983220662324	0.911837389834658	-0.0762053333333327	0.745967084434416
AP1S1	0.0955120560758387	0.783179138260545	-0.0805848658330214	0.896800115194319	-0.129135333333332	0.5293518047673
RANBP9	0.0954415322643119	0.783430966020182	0.177465484759595	0.728529406441478	0.121112666666667	0.555273681994882
LOC338799	0.0954170722957377	0.783452637825342	0.00917924855416903	0.98909166696601	-0.154989666666666	0.528409919089401
CXCR6	0.0953986997542887	0.783503253077086	-0.180713633055367	0.722213862845507	-0.00751233333333331	0.98256318768706
ZNF639	0.0953341913050926	0.783727941886156	0.153301529061038	0.774003008542746	0.170102666666667	0.46297633209186
CRTC1	0.0953328788670767	0.783727941886156	0.163452251132558	0.75547765390766	-0.154267999999999	0.520368312537146
GAA	0.0953210865967905	0.783727941886156	0.154189146818202	0.772042628013353	0.125980000000001	0.532604205131631
HMGB2	0.0952997514747726	0.783727941886156	0.147881805631756	0.784090188380266	0.323012	0.216858796679299
LRRC47	0.0952899024599833	0.783727941886156	-0.233107919613891	0.611659075898586	0.0166566666666676	0.955460918122607
USF2	0.0952547382290397	0.783749255234118	0.191695346071841	0.701544026774214	-0.009833666666666485	0.974451420920923
MEGF11	0.0952407064371609	0.783771478668637	0.120700475892492	0.833602134657439	-0.115389999999999	0.594374666543557
PHF20	0.0951396722728924	0.784223736773649	-0.190825597072159	0.702764646363039	-0.0078473333333328	0.983159241812861
TIMM50	0.0951028075004448	0.784325754460734	0.0933308350274127	0.876656471145851	-0.0554029999999989	0.80578153770169
RAP1GDS1	0.0950288788603179	0.784739893808422	-0.133586127330544	0.808933084408149	0.236048666666668	0.244059562675669
ATP11B	0.0949698910958096	0.785056314764667	-0.217705691849133	0.644820007196044	0.200550333333334	0.367206442400058
LDOC1	0.094912511860181	0.785247302220465	0.0144218152019193	0.983444353656142	0.142288666666667	0.610151958903093
HAPLN1	0.0949051369339747	0.785247302220465	0.319704700645362	0.425200875750207	0.00727500000000055	0.983159241812861
TPT1-AS1	0.094827695269236	0.78549951377478	0.0684580838440182	0.912149839732916	-0.0787698333333324	0.74568697846442
ANP32D	0.094796300721493	0.785538164817971	-0.0466422172629779	0.944599178367537	0.0862330000000003	0.690213956173797
NUDT4	0.0947908615300827	0.785538164817971	0.205413900752512	0.672934804602266	0.274233333333334	0.158572036652044
FGF2	0.094789871460768	0.785538164817971	-0.161619316942612	0.758955920988521	0.148504333333334	0.616472837170869
CMTM4	0.094753986481864	0.785633758648009	0.338325469999705	0.384701467775014	0.154190999999999	0.512796060754107
FAM26E	0.0947104822077962	0.785848837017216	-0.124921692413950	0.824457843812434	-0.0897859999999992	0.730330043083783
DAB1	0.0946147638764668	0.786266389785723	-0.051624765404669	0.938514797510096	0.022648888888889	0.93278021276477
DKK4	0.0945498915900986	0.786482055242189	0.195945869067568	0.692430369468218	-0.0253639999999999	0.924674647382585
SLC25A31	0.0941920318783706	0.78806759026872	-0.141223573431032	0.796285336930078	0.171682333333334	0.418466188851614
CA12	0.0941250263135594	0.78806759026872	-0.0620466948050747	0.923034515346352	-0.254708	0.186420644564045

ARGLU1	0.0941198869867849	0.78806759026872	-0.148912765977276	0.783041584899534	0.0135066666666676	0.962275697500938
LOC100130428	0.094094835186361	0.78806759026872	-0.0632975720059288	0.92105583497281	-0.0594023333333328	0.871104061464043
BCL9L	0.094068432124277	0.78806759026872	-0.110796059413556	0.849357727620213	-0.0370766666666658	0.882040270326032
WDR54	0.0940019520347458	0.78818148835494	0.0705544730451454	0.91009246060164	-0.493909333333331	0.0584824376865046
EXOC5	0.0939855719989162	0.78818148835494	-0.0195125706283608	0.977673457110824	0.125422000000001	0.565072000360364
DDX24	0.0939710183479793	0.78818148835494	0.111356794058756	0.848968422336654	0.104466666666668	0.610151958903093
FMR1	0.093946458761174	0.788272578953166	-0.469904882144304	0.150589570338477	0.124129333333335	0.550584871031795
ZW10	0.0938239574844363	0.788865367608489	-0.356953907643823	0.345138088652208	0.180782000000000	0.346977521971170
OXCT1	0.0937181013614732	0.789240030126654	0.157616769409148	0.767770230680061	-0.0011319999999995	0.99704422037925
MTX3	0.0937169370100895	0.789240030126654	0.0824610069925304	0.8939835772794	-0.260436	0.216591326893487
RNF149	0.0936741654529982	0.789427868407118	-0.333653744627609	0.396821740334178	0.114361000000001	0.608987229758959
PECR	0.0935845056275218	0.789875449061887	0.448732464692195	0.177860736739947	0.0962210000000008	0.731425088367868
NMUR2	0.0934863225911944	0.790239475854191	0.0765926682349674	0.902423698188628	-0.0422866666666663	0.882342951312785
CHRNA10	0.0934046011446377	0.790496581251142	0.0396199854311531	0.953729831316741	0.00269333333333402	0.994969303295258
ZNF514	0.0933346070550497	0.790496581251142	-0.241277295494984	0.596019873098193	0.271101333333334	0.255595078844894
SCAND3	0.0933298908835064	0.790496581251142	-0.039934583850568	0.953472916828921	0.089952333333334	0.672980097690282
TTTY12	0.0932884840491575	0.790496581251142	-0.0712114097668675	0.90935479990339	-0.0472409999999998	0.852346905702153
CTSK	0.0932391448622575	0.790603450077757	0.164497908290265	0.754189116283047	-0.0392683333333323	0.880427463662501
OXGR1	0.0932189131904613	0.790630101157522	-0.113102299393235	0.846200067907049	0.177757666666667	0.591142517306808
GPR113	0.0931883106433873	0.79065761044567	-0.140643772776877	0.796714214272984	-0.194898999999999	0.390645312906549
PADI4	0.0931662468243397	0.790732359728148	-0.039159039242402	0.953903510456582	0.0533460000000002	0.87709801222343
SLC39A12	0.0931373853328811	0.790785866355938	0.101266476083951	0.866005093242444	0.0356820000000002	0.889610038918956
DLG5	0.0931185223506319	0.790816847713267	0.10499815905162	0.8606536389692	-0.202283333333333	0.446756640099031
TRAM1	0.0930736479019503	0.790861267771643	-0.154406330950308	0.771310656070265	0.198753333333334	0.344576368488222
RPGR	0.09301767457055	0.79100841759749	0.300932525991469	0.464913571526964	0.0477223333333335	0.83822720696374
CCDC37	0.0930058845659882	0.791015968125222	0.264417977543366	0.544042395849675	0.071848	0.773270959529093
RBBP5	0.0929327492701513	0.791193007798194	-0.0289865598419728	0.96782691865619	-0.162541333333332	0.500886251638044
NYAP1	0.092883106166847	0.791193007798194	0.173956475389508	0.734793595952544	-0.222642333333333	0.26757087734089
PPY	0.0928652620189128	0.791240160222504	0.243016081621305	0.592178030679976	0.070049	0.804755486961935
GMNN	0.0927886692886678	0.791532391476019	0.174199237650067	0.734267769889068	0.397778333333334	0.0755898373594719
PDE6B	0.0927311503885947	0.791699907493083	0.238158825507365	0.60184463266698	0.00792066666666722	0.98256318768706
MYCNOS	0.0926787364240163	0.791862590157808	0.156519664298284	0.768909201237015	0.0749223333333342	0.903274690789156
ZNF732	0.0926743705557369	0.791862590157808	0.113585254779456	0.845326910026624	0.0241746666666667	0.928640283648112
ADAMDEC1	0.0926619417146765	0.7918743355404	-0.196245127803311	0.691685804764422	0.0700273333333336	0.759034160486144
ATP13A1	0.09265045436637	0.791879927831586	0.129104231650833	0.815076057740972	0.0372030000000004	0.891339318634358
OTUD6B	0.0926282894312466	0.791955326690677	-0.0039531142531043	0.994501615176666	0.104473666666668	0.636468072096347
FLJ45340	0.0925615018171296	0.792070104354506	-0.123731386586329	0.826475556843795	-0.0926349999999998	0.702097473889838
SHISA2	0.09255454007677	0.792070104354506	0.0946432549789906	0.8739154222556	-0.00507866666666660	0.988133186272453
ASGR1	0.0925410935765005	0.792070104354506	0.511865902436617	0.0985590060518004	-0.228114666666667	0.346020200924341
HCAR1	0.0925352212841612	0.792070104354506	0.314583759090019	0.437067919667049	-0.0129626666666666	0.965661221820942
HIC1	0.092515058373025	0.792070104354506	0.0306882339967086	0.965460387628839	-0.101062666666666	0.666642292286073
CD22	0.0924790155037753	0.792144444364472	-0.0901471322176204	0.882249601181978	0.0747556666666673	0.824548552404467
PPARG	0.0924563209406742	0.792144444364472	-0.0389675238864868	0.954097938114723	-0.0687293333333332	0.822043577898586
CDCP1	0.0924505441260847	0.792144444364472	-0.173539418264924	0.735851467282891	-0.0511843333333333	0.919614989927452
CYP2C19	0.0923947235946175	0.792377503001364	-0.152375308054114	0.775863712880333	0.0555526666666673	0.822657709336468
ATP6V0A4	0.0923899054141407	0.792377503001364	-0.215222027597683	0.650754464016263	0.0286576666666673	0.920908349612552
CPNE5	0.092376060125264	0.792377503001364	-0.384305690560542	0.291814756660665	-0.182466666666666	0.443378989641463
XPA	0.0923723693708638	0.792377503001364	-0.113095049991778	0.846200067907049	-0.0395299999999985	0.880347900939311
TMEM121	0.0923389908941736	0.792477770214262	0.235703868824361	0.6067637636079	0.00321433333333470	0.993756838831997
KRT77	0.0923134665422148	0.792513383935407	-0.0973205492982088	0.870020870354202	-0.135112999999999	0.518244955194698
ZXDC	0.0923016191262032	0.792513383935407	0.0685485726752466	0.912149839732916	-0.150094000000000	0.45895048781174
ARFGAP3	0.092283944302208	0.792513383935407	0.0523923374431747	0.937831566264837	0.128872666666667	0.705010402761235
CPEB3	0.0922671629418152	0.792513383935407	0.254341271028307	0.567694846153007	0.466897333333335	0.0309320552705188
EBF2	0.0920974734141684	0.793353846615746	-0.216865798669508	0.647342905940871	0.0587430000000003	0.794404527150583
TRMT1	0.092095421272921	0.793353846615746	0.314771640424602	0.436900127228272	-0.204927666666666	0.309421300309741
DEDD	0.092049290008297	0.793569748632441	-0.211913930806633	0.658364968650984	-0.0162689999999986	0.954736472265512
SAMD4A	0.0920411245031992	0.793569748632441	-0.192174152731144	0.700820605802046	0.315026666666667	0.14208977383533
NARS	0.0920067148835132	0.793620637754673	-0.221490946629665	0.637706908132848	-0.0730633333333331	0.74511187686951
CDS2	0.092001452573394	0.793620637754673	-0.281448850907757	0.506335763951991	0.0595570000000004	0.798803651237705
AGRN	0.091961678437444	0.793734353145514	-0.255645187230785	0.565656702091657	0.038631	0.883189085233712
TP53INP1	0.0918775290689001	0.794083147880427	0.378650507802256	0.304578046752771	0.263081666666668	0.234508116088724
COL13A1	0.0918418734568264	0.794246685570202	0.302461878809144	0.462229407853213	-0.0990563333333326	0.672240322786097
SLC25A32	0.0918199976560804	0.794246933303053	0.124711740522399	0.82479991054473	-0.144549666666666	0.518244955194698
ASIC1	0.0917574281292121	0.79431235739296	0.163065025920921	0.756720201996006	0.195305333333335	0.344576368488222
PARP1	0.0917143483152826	0.794524376704688	-0.151055337101327	0.778007072419094	-0.179789999999999	0.432456927987147
PROZ	0.0916643236510013	0.794771604330465	0.224508333981824	0.632705750635533	0.0733260000000003	0.762898679429892
DPT	0.0916552533142409	0.794771604330465	-0.00265107517777805	0.99580901762095	0.0493183333333338	0.846917872248932
PLK3	0.0915145433381045	0.795138978026973	-0.125643840819542	0.8236721238761	0.127833833333334	0.524845938106282
MOG	0.0915114533687965	0.795138978026973	0.131620714328220	0.811042872465441	-0.0163853333333330	0.958565523123178
CAP1	0.0914283296862412	0.795420953982236	-0.366002958229223	0.32927628703889	0.0676900000000018	0.76005598628861
AATK	0.0913339736973354	0.795914059750297	0.281238730652068	0.506749495296958	-0.05608	0.85184445019945
CRYGN	0.0912383902917411	0.796314741902574	0.214675171822991	0.652003104177839	0.0103973333333326	0.974451420920923
FBXW2	0.0910575675486482	0.797079577812873	-0.184647516791185	0.716351619471715	0.0277300000000011	0.915761046438965
GFOD1	0.091007854963629	0.797195994492546	-0.444181963840584	0.183294194447278	-0.0159003333333327	0.965329851466378
SKA3	0.0909748590862571	0.797260349838267	-0.275825932038721	0.517637664838512	0.222013000000001	0.313220411537404
KLHL6	0.0909711989437873	0.797260349838267	-0.300983974094471	0.464902980443452	-0.0992449999999992	0.630227347736008
CYP2E1	0.0909401302895696	0.797309421789882	-0.110923409465029	0.849357727620213	0.0531393333333338	0.849114834471057
SRGN	0.09092709655295	0.797309421789882	-0.323027205280737	0.415854621854132	-0.338152666666665	0.106113992549159
TPGS1	0.0909267038788533	0.797309421789882	0.295093045660395	0.478475354656325	-0.0446209999999987	0.871104061464043
SLC45A2	0.0908941926392697	0.797313465351559	0.0340095615533249	0.95993706312687	0.0553463333333344	0.82586230412069

NTRK1	0.090808040535385	0.797667988770364	0.288361670347063	0.490238421312485	0.0399923333333334	0.88487182863547
YJEFN3	0.0907847876530045	0.797720453413313	0.372266352239236	0.318024489042558	0.122948333333334	0.562784422738728
ZFP62	0.0907787488773203	0.797720453413313	-0.117012718239572	0.841189837368881	0.235633000000001	0.291501950143291
NME7	0.0907352575542688	0.797820408712173	-0.174433984617339	0.734267769889068	-0.123031333333332	0.670113640649168
OTUD7B	0.0907207529880352	0.797820408712173	0.0930136146172956	0.877331332619452	-0.114086999999999	0.67320648871569
CXorf24	0.0906550896107272	0.797860656978609	-0.0595361439195012	0.926789115926346	-0.220377999999998	0.320282845977203
MRPS18A	0.090648066582444	0.797860656978609	0.161091128468240	0.760354001455808	-0.0293699999999984	0.925845244747948
ZNF664	0.0906293077183957	0.797860656978609	-0.316068578980131	0.433592288295757	-0.209043999999999	0.306650933067113
PRDM6	0.0906231764436464	0.797860656978609	-0.0472506161571059	0.943981149721771	-0.246955333333333	0.213876860878204
HDAC8	0.0906084908208445	0.797860656978609	-0.504605432127028	0.106882834148277	-0.240853999999999	0.331900139634594
SLC22A31	0.0905759092488786	0.79793464740021	0.290588646870315	0.488016046822631	-0.0609463333333334	0.870002591217953
ARID4A	0.090512980766789	0.798008380976633	0.168131889980923	0.748024524669236	0.223091666666669	0.279380540476768
SUPT16H	0.0904912007620852	0.798008380976633	-0.0261042285764387	0.970632644458507	-0.0684066666666657	0.776013100927836
ART4	0.090480257162724	0.798008380976633	0.0145991077872127	0.983444353656142	0.0281373333333332	0.917325389804968
RBM43	0.0904666841304836	0.798008380976633	0.147999284667026	0.784080588385358	0.144458000000001	0.528409919089401
SEC63	0.0904277562009352	0.798008380976633	0.111665118902585	0.848451264169	0.235613333333334	0.21325573490485
NRAS	0.090350469957882	0.798088899549843	-0.209352942143147	0.664703270325548	0.0718266666666682	0.745967084434416
ANXA6	0.0903290938146465	0.79815909520986	-0.043062403259089	0.950146638225747	0.267621000000001	0.177509274522709
ALS2CR11	0.0902759354415353	0.798367383095246	0.004026809535526	0.994485841304070	0.165539333333334	0.411817051607043
SLC6A13	0.0901944677800368	0.798480720392787	-0.00255183908239856	0.99580901762095	-0.117756666666666	0.637138440988813
CXCL5	0.0901875209104638	0.798480720392787	-0.408384902597005	0.250550105343578	0.0341516666666672	0.922710832267249
MID2	0.0900781446983243	0.798895826550302	-0.100959780649337	0.866005093242444	0.343810666666668	0.173494642908599
DTX1	0.0899916695921851	0.799182910581732	0.52926635704563	0.0799297163112453	-0.0622143333333326	0.838813113208837
SELM	0.0899663047136764	0.799209857312788	-0.0886793864474214	0.8841943128258	-0.0796736666666672	0.826961866961896
RGMA	0.089846533514969	0.79950636821062	0.0775623093359867	0.900701176765499	-0.142507666666666	0.631408228698652
TBC1D12	0.0897843466201146	0.799646636960362	-0.272074837458822	0.527284725671175	0.106540000000001	0.704169495024335
ZCCHC4	0.0897722578431441	0.799646636960362	-0.363757694566323	0.335033160801809	0.238911666666667	0.336373925530627
KNTC1	0.0897298223822192	0.799713712825645	0.0338804459004026	0.96017797488544	0.127146333333334	0.55248070124039
ARHGAP23	0.0897007024745714	0.79976518074109	0.221433630416530	0.637715957775489	-0.0256171666666667	0.929066230830908
NFKBIZ	0.0896202497776968	0.800012953343996	-0.292099279648980	0.484470659050591	0.556519333333334	0.0758492851786713
MFAP5	0.0895871272572867	0.800056946961457	-0.119275511583333	0.836360753951164	-0.0688526666666665	0.86098720418783
CSRNP3	0.0895228691774335	0.800232702632003	0.0637110969082508	0.920685491148474	0.290912	0.139122737306306
PSMD12	0.0894157488689158	0.80044363245692	-0.0853893812349045	0.889779510878572	-0.0609053333333331	0.787739636244196
PDHB	0.0893959699251958	0.80044363245692	-0.0927059060784373	0.877797216649453	0.0457976666666682	0.845737357656109
MIS18BP1	0.0893917592783075	0.80044363245692	-0.0304699704435335	0.965815288453725	0.154233000000001	0.482760288609588
MYL12B	0.0893844035540065	0.80044363245692	-0.0771910883281353	0.90125257838581	0.168246666666667	0.403772861091022
PCYT1A	0.0892721507823795	0.800691768667511	-0.260515696392103	0.553650518260168	-0.207685333333332	0.321008129295804
BLVRA	0.0892544607431383	0.800691768667511	0.102714430246372	0.86257932725575	-0.153907999999999	0.452657932065446
HIRA	0.0892402454702693	0.800691768667511	-0.522133564209147	0.0876859028054308	-0.0861046666666653	0.720355613738393
SIX5	0.0891968734772934	0.800777566774382	0.0823058858462363	0.894398263791695	-0.0391959999999997	0.872930585008323
KRTAP1-5	0.0891952575426478	0.800777566774382	-0.0634692529385607	0.920855211745173	-0.0921189999999993	0.739507465050387
CHST15	0.089166524426119	0.800873669404062	-0.332533462301571	0.398142341492687	-0.0628606666666664	0.805850711608402
GRIA1	0.0891592940677094	0.800873669404062	-0.0861753564064576	0.888051139742697	0.065699333333334	0.850364148347928
COG8	0.089116458960287	0.8010145348641	-0.0668307664029818	0.915238138104257	-0.0400329999999999	0.870843154725153
AKAP7	0.0890443655613156	0.801138742713337	0.463849718119704	0.157277647666176	0.507538333333334	0.0401656304731054
SFTA2	0.0889642897336421	0.801453093075462	-0.0483514453202411	0.942820948997969	-0.108199999999999	0.68417809000625
LPPR4	0.0888171143938753	0.80213546349139	-0.0438446022580641	0.948907750739645	-0.0363216666666663	0.903433230704096
DNAH14	0.0887185914966483	0.802708308724998	-0.264899910434031	0.542477070548696	0.178806222222223	0.49038083653267
HNRNPUL2	0.0887043987012674	0.802731646580365	-0.0351727421463391	0.958873757674185	-0.123071999999999	0.548804275857029
TMF1	0.0886154272174013	0.80310399936747	0.075456265493147	0.903779020368292	0.0257570000000014	0.922463263763
FLJ35024	0.088579032483507	0.803272031141632	0.295728061166942	0.47761182409076	0.157215000000000	0.52162552805731
BLID	0.0884667897190257	0.803588533511916	0.153515226216873	0.773553722129288	0.215467333333334	0.290781031227171
DOCK4	0.0884550238449446	0.803596046879676	-0.254670260248218	0.567282506136306	-0.370520666666666	0.178162638616424
SLC16A5	0.0884238276908564	0.803730186577719	-0.278014223654499	0.512411130555293	-0.141919666666666	0.503095600478615
ZNF804B	0.0883861248512526	0.803858920477004	0.249682264281210	0.575370998761354	-0.168636666666666	0.376224594108747
SH3RF3	0.0883679952609631	0.803858920477004	-0.0587210858836183	0.928472484757714	-0.185147666666665	0.422927358730594
CNGA3	0.0883510095687532	0.803858920477004	0.306711901914746	0.454790495793644	0.135563000000001	0.592716584553539
COASY	0.0883376277274516	0.803876969474605	0.146161613621728	0.787402233709509	-0.0118236666666653	0.969513647149429
CTPS1	0.0882226797526447	0.804140008682085	0.0470978610770419	0.944081286115546	-0.172419000000000	0.442036873484362
SCN5A	0.0882089294872906	0.804140008682085	0.371855205268906	0.319073951161831	0.137777333333334	0.60378773986659
TERT	0.0881816503840244	0.804140008682085	0.195362890496726	0.694342510983283	-0.166775333333332	0.517547647029412
BOD1L1	0.088165532322191	0.804140008682085	-0.286920905781566	0.494604746861742	0.0504500000000017	0.856854911614454
NIP7	0.0881583078738981	0.804140008682085	-0.165964500161089	0.751869806279813	-0.291150999999999	0.124096550131011
CAMK2N2	0.088148714156912	0.804140008682085	-0.0238127421038717	0.974814447104635	-0.142616333333332	0.648442399981194
PRR22	0.0880831300316206	0.804353688223243	0.0788521174246168	0.899154242391172	-0.224122000000001	0.2852695849699
PACSIN2	0.0880514929909685	0.804399274612148	-0.172478116140580	0.738421727152922	0.0812380000000005	0.729912257977231
PLA2G4F	0.0880395914318042	0.804399274612148	0.118964934761642	0.836828045978697	-0.0978236666666661	0.68400971768044
TUBB3	0.0880343304008707	0.804399274612148	0.0451940341955249	0.946577404106596	0.056525000000001	0.814273596067945
APOF	0.0880240471341926	0.804399274612148	0.103734365056947	0.861866357553435	-0.00489633333333266	0.988942941807152
TIFA	0.0879670245880582	0.804632451774617	-0.0142293899541635	0.983444353656142	0.210946333333334	0.508556355177491
FLG2	0.0879067843916344	0.804817511750773	0.239785109204281	0.598320155622224	-0.212821666666666	0.409303621288484
ANKRD60	0.0878206668121841	0.805240153975602	0.169165987988450	0.74499234065498	-0.235973666666666	0.465948865963889
RUSC2	0.0877711868605448	0.805355124014853	0.271916579245518	0.527284725671175	0.0518796666666673	0.86194148699956
FBXO43	0.0877604664041319	0.805355855794463	-0.0114167541926695	0.987215779667937	-0.113820000000000	0.605491766505367
TOP3B	0.0876725087664936	0.805652305270094	0.151290875120080	0.777604486471648	-0.143875999999999	0.535171605667144
LGR6	0.0876531392865507	0.805709355696982	0.0799186992650455	0.897892885174022	-0.0746626666666662	0.764060212462792
TAF6	0.0874435886990785	0.806452150558868	0.192019858697621	0.700976254251101	0.157752666666667	0.479014333115247
GJA3	0.0873531309072765	0.806763488326238	-0.240188626017524	0.59795653507991	-0.129474	0.537105380052834
RPL23AP53	0.0873342301111024	0.806763488326238	0.111190305182861	0.849127967004266	-0.242201333333333	0.376020235114735
KIAA0368	0.0872952041755711	0.806763488326238	-0.05448721153608	0.9344692810234	0.083500000000001	0.697261622653146

ZNF80	0.0872896874239388	0.806763488326238	0.0393717676502676	0.953905313456582	-0.224344999999999	0.290878986887538
PIK3CD	0.0872646221031022	0.806763488326238	-0.282185218652491	0.504065779877366	-0.100218999999999	0.68951218434739
AXDND1	0.087148614330683	0.807267521633286	0.0226122468377291	0.97536722461972	-0.0272123333333329	0.91402300740264
APOBEC1	0.087134402340955	0.807289069697	0.0318808336718187	0.963211116393051	-0.0290789999999998	0.91935982515612З
TTTY11	0.0870460348521795	0.80758145513663	-0.111424303254706	0.848968422336654	-0.112411333333333	0.583036318848746
FSTL5	0.0870404285420011	0.80758145513663	-0.303931554910597	0.460052080110635	0.115407666666667	0.670368464901515
ZNF496	0.0869022935587848	0.808042304465408	0.259004975889689	0.558522272130827	0.0875506666666669	0.673921955474628
SLC5A3	0.0868618502854106	0.808195242041729	-0.0420344170608267	0.951989245458556	-0.0520673333333316	0.829188536848723
NFIL3	0.086857581258722	0.808195242041729	0.0086944196351041	0.98946753270812	-0.0827976666666656	0.753956061482057
ZNF91	0.0868294600340375	0.808240180805732	-0.340792364828959	0.378272273903863	0.344894333333334	0.0773605457360439
ATG7	0.0867779220430017	0.808368491265843	-0.0466626458653279	0.944567208068726	-0.099026333333333	0.660899980762829
FSIP2	0.0867540712841538	0.808371663804036	0.160053723255733	0.762902034786947	0.0521251111111112	0.852020205196772
PCNXL3	0.0867456122521119	0.808371663804036	-0.110092830955160	0.850662071774129	-0.0994003333333327	0.657217907037306
CCNE2	0.0866953391948767	0.808560774352989	0.00745697788948381	0.990795532161521	0.285535333333334	0.165359581005486
C14orf169	0.0866146215790461	0.808793050580426	-0.223727489417907	0.633992942859828	0.00172466666666726	0.995621063297892
PAPPA	0.0866046195540793	0.808793050580426	0.270154775360484	0.530185035699403	0.0454141666666666	0.861677398924522
GPI	0.0865774821015323	0.808793050580426	0.15629392598456	0.768909201237015	-0.097319999999999	0.656324975591247
NIPA2	0.0864157067103938	0.80920125871668	-0.206015936761268	0.672284583020815	-0.0157206666666659	0.955115242974667
AIFM3	0.0864029327119347	0.80920125871668	-0.0258146019134853	0.97138601285307	-0.0615306666666663	0.809505390888975
HSPB3	0.0863995421780448	0.80920125871668	-0.388401763484391	0.28486220160813	0.633795666666667	0.0147480919536708
PEX2	0.086389781058676	0.80920125871668	0.260569830198855	0.553540075799904	0.226986333333335	0.291481585177291
SMPDL3B	0.0863760309269098	0.80920125871668	-0.0771634556624365	0.90125257838581	-0.398837	0.160589525668831
RPN1	0.0863741864066668	0.80920125871668	-0.0600644012723771	0.925676268175378	0.106966666666668	0.614221661998915
TDP2	0.0863608116303077	0.809219287663126	-0.219730273157838	0.641035290381005	-0.133170666666666	0.534227067294365
SBNO1	0.0863334850382085	0.809328057379334	0.0730000641118869	0.906592046281011	-0.0350686666666659	0.903788355106891
ZBED4	0.086207657300916	0.809374369082773	-0.156150053359877	0.768989193721112	-0.176135999999999	0.462361390840849
NUP93	0.0861906393692106	0.809374369082773	-0.243007903682785	0.592178030679976	-0.104795000000000	0.639002829892966
SCEL	0.0861720541333306	0.809374369082773	0.0218212418423553	0.97536722461972	0.0325266666666665	0.907374738017166
PF4	0.0861631469894018	0.809374369082773	-0.252293870727152	0.570311620130916	-0.0546493333333326	0.80895954022293
FCER2	0.086134623716379	0.809374369082773	0.432914408786897	0.202301256144175	-0.0082289999999993	0.982416094939651
LUC7L	0.086131218260968	0.809374369082773	0.0662317779181761	0.916661988649658	-0.0725863333333317	0.762898679429892
MRPL38	0.0860423665894105	0.809374369082773	0.205072235149886	0.673595203634548	-0.0386546666666656	0.888181586513563
CIAO1	0.0860391133718308	0.809374369082773	-0.356563029990472	0.345989229646028	-0.103517666666665	0.616036686881905
ASPHD2	0.085976823038292	0.809374369082773	-0.0756922751220019	0.903510413986113	-0.0309346666666662	0.935261323444314
LIN52	0.0859760099863359	0.809374369082773	0.0227986640798492	0.97536722461972	0.0046863333333339	0.988133186272453
EPSTI1	0.0859351084866858	0.809374369082773	-0.272413005380775	0.526175116911121	0.0140326666666675	0.96003296204918
CNTNAP3	0.0859242894011938	0.809374369082773	0.203711988782744	0.676117692782917	0.706551733333334	0.170391424415032
GSDMC	0.085913087979677	0.809374369082773	-0.00439584455188883	0.99405344216434	-0.0649036666666664	0.816134952712854
EFS	0.085912904696085	0.809374369082773	0.0809312290894185	0.896361013063453	-0.171388333333332	0.440368942982395
NDOR1	0.0858202722725736	0.80956500444395	0.197312324078552	0.689256317041816	-0.176481333333333	0.353471671280201
SIGLEC17P	0.0857679122169107	0.809698745948189	0.122779112009603	0.828721578450338	-0.185720333333333	0.398349038294794
MAPKAPK2	0.0857469563448279	0.809746235420844	-0.108025850746048	0.85582330081028	0.114600000000001	0.614221661998915
TTC9	0.0857390300051488	0.809746235420844	0.293599998435282	0.480614908214975	-0.042852999999999	0.873398028647672
ZNF79	0.0857278435718728	0.809746235420844	0.0746946709051353	0.904466414650249	0.00552833333333473	0.987134800094338
IRGQ	0.0857011352208332	0.809766007358444	-0.185439868146904	0.714427536588157	-0.250057333333331	0.249910577465585
ENDOD1	0.0856710373285395	0.809766007358444	0.201132801808177	0.680143033775131	-0.0187946666666658	0.945904449997854
ITM2C	0.0856588080000695	0.809766007358444	0.253261725769607	0.567925260831937	0.136410333333333	0.493413216077427
ATG9A	0.085627105974765	0.809766007358444	0.370332100661501	0.320801726666339	0.0112086666666678	0.972573891494574
CDH13	0.0856199680546977	0.809766007358444	0.038476262920112	0.954545200586067	0.186944000000000	0.327240483600249
RAPGEF5	0.0856084088397254	0.809772357530164	0.144977319598265	0.789227217144616	-0.043680666666666	0.873454962131763
VPS36	0.0855275280036099	0.8100225008865	0.0182559400717895	0.979327298872732	0.224789333333334	0.270327918481764
GBP1	0.0855067415693369	0.810046296870725	-0.519199720632214	0.0902262536464163	0.255709333333333	0.320462604748547
KCMF1	0.0854916129989974	0.810049587369187	-0.135223748401671	0.807470535721699	0.0885233333333349	0.702709435280393
SLC4A10	0.0854270062209018	0.810262779601573	0.215988806121490	0.64844945813838	-0.0100709999999995	0.974895360062947
STOM	0.0854158382652934	0.810266597711247	-0.142216556402918	0.79450771779567	0.0428306666666678	0.86272805475777
WDR49	0.0853916632116607	0.810286168363905	-0.2052495546439	0.673296856274784	-0.00313966666666602	0.993677488355417
RGS22	0.0853789144985598	0.810300244303853	-0.153637514518154	0.773358453580566	-0.0500176666666663	0.834476346642783
C1orf127	0.0853343113509463	0.810425976538755	-0.0218226071989986	0.97536722461972	-0.127883999999999	0.616036866881905
PLAU	0.0853199822897833	0.810425976538755	-0.336422521329050	0.389256307522526	-0.144978666666666	0.549664615421682
OLFML3	0.0853105858435227	0.810425976538755	-0.333546484617597	0.396821740334178	-0.253971666666667	0.343572153228906
MYF5	0.0851427667835674	0.811184069653553	0.0349891545630853	0.959140268168928	-0.108756000000000	0.607898776363481
ZKSCAN1	0.0851368406289408	0.811184069653553	-0.109887556399495	0.851013616427418	-0.0678639999999994	0.772139923817113
KCNA2	0.0850771383331114	0.811289520639195	0.0662072156078867	0.916661988649658	-0.154926333333333	0.470695033055552
RNH1	0.0850686146793077	0.811289520639195	0.208454889337788	0.666544605217271	0.0307753333333346	0.915761046438965
NLRP8	0.0850333616885338	0.811443468524502	-0.0347464869034888	0.959404955370931	-0.203935333333333	0.324443655582244
LOC79999	0.084995293172602	0.811578635522533	0.253814283603068	0.567694846153007	-0.0320759999999993	0.903864363916236
TGM5	0.084991358003062	0.811578635522533	0.00962873543668382	0.98909166696601	-0.0238426666666662	0.930211818640573
HEMGN	0.0849251213376678	0.811746991716635	-0.0110169737062979	0.98774001793194	0.112598000000000	0.588666179294878
SLTM	0.084915796334317	0.811746991716635	-0.0569686990983847	0.930784376757936	-0.0272526666666663	0.917543593028956
PPP1R14D	0.084902220112404	0.811746991716635	0.015768227251886	0.982266426585123	-0.0517636666666663	0.890073383340154
RPS5	0.084901918803511	0.811746991716635	0.100388398070033	0.866570192459381	-0.0646806666666657	0.789228734235839
FIBP	0.0848082600897782	0.812011290205306	-0.199299591057040	0.684227048797313	-0.0635626666666662	0.785344276827705
ZNF281	0.0847778499770083	0.812070116289892	-0.298323343385453	0.471509618848387	0.0681786666666679	0.76409396614905
FBLN2	0.0847711719011989	0.812070116289892	-0.0795183701040505	0.89819077139711	-0.151979666666665	0.477235955589408
KCNC4	0.0847539381469673	0.812070116289892	-0.0315858971560161	0.9638977610518	-0.0399043333333327	0.8754328609757
MSANTD2	0.0847028781850564	0.812070116289892	0.0580834274813571	0.929293497981508	0.0219720000000007	0.949852419200473
HAUS5	0.0846554782539175	0.812070116289892	0.206755541144080	0.670360323581237	-0.0692303333333328	0.759034160486144
MED23	0.084621415836398	0.812070116289892	0.328141127307047	0.407365872456796	0.039913666666667	0.892921857967499
NCOA2	0.0844732800876405	0.812364878203395	0.174189204829374	0.734267769889068	0.157796666666668	0.45432073488215
TMEM241	0.084466492672686	0.812364878203395	-0.0497107643596523	0.941458038227918	-0.0236686666666665	0.948778075888142

LYG1	0.0844554290804403	0.812364878203395	0.112064628547090	0.847483801046528	0.0974920000000003	0.637756447806811
OBFC1	0.0844274910939916	0.812364878203395	0.427835109032616	0.211811431978425	0.159368666666667	0.416412641573293
MYNN	0.0844208700051306	0.812364878203395	-0.0256904563090672	0.97179002490987	0.101019000000002	0.675183563312084
GJB3	0.0844101101958838	0.812364878203395	0.173492581082043	0.735950802418789	0.0426103333333338	0.904507944803624
RPE	0.0843959633638402	0.812364878203395	0.0067984573652102	0.99129507664977	0.00289366666666669	0.994738356997797
CSPG5	0.0843942185390803	0.812364878203395	-0.167965088965985	0.748024524669236	-0.0720833333333334	0.741809049318412
TRAPPC8	0.0843770378681	0.81238725370711	-0.0355468263428967	0.958689318480951	0.255249000000001	0.244974557746985
PLAC4	0.084369629463029	0.81238725370711	-0.0473151314010699	0.943981149721771	0.00237666666666678	0.994114279008565
BMP1	0.0842073151050921	0.81310084645348	0.390507831214337	0.282383744168943	0.194005000000000	0.306286104860031
TP53AIP1	0.0842023409936178	0.81310084645348	0.213268447669631	0.654954204535388	-0.0189979999999999	0.959032046952242
DEPDC4	0.0841694526368585	0.81310084645348	0.0971336096959802	0.870020870354202	-0.00947766666666635	0.984334402946184
THOP1	0.0841553486186272	0.81310084645348	0.0323324224248193	0.962272964666618	-0.151700666666666	0.446756640099031
PEX14	0.0841321670458095	0.81310084645348	-0.17575645908459	0.731808176718647	0.162426333333333	0.458154400582976
ZNF607	0.0840421217941916	0.813412880772907	-0.318077999954545	0.430156985569412	0.215844000000001	0.323781628570750
PTPN5	0.0840270774651328	0.813432366552144	0.068173800463497	0.912364243071055	-0.0813506666666663	0.733867173296405
CCDC57	0.08401797830281	0.813432366552144	0.135133166067388	0.807470535721699	-0.0768783333333324	0.735938662411184
SLBP	0.0839556832449063	0.813698776083663	-0.165890475307818	0.751869806279813	0.100875666666668	0.635069698575895
KIAA1967	0.0838997724288923	0.813923863813111	0.081058372968754	0.896361013063453	-0.126481666666667	0.581502485736275
RBM23	0.0838685298134383	0.814057684371344	0.0415073207450454	0.952098348256047	0.134613000000000	0.498456714564834
ZZZ3	0.0838203372737615	0.814197234250314	-0.0897164607051436	0.88318195439136	0.0733646666666664	0.760564116489878
APOA2	0.0838152648767475	0.814197234250314	-0.152694691678730	0.775154530747777	-0.159662666666666	0.518836392563847
C19orf66	0.0837713901139566	0.814412814476968	0.0928012056609809	0.877797216649453	-0.0680503333333327	0.824044031896077
AP1M1	0.0837152286913582	0.8145231684629	0.194963036583010	0.695325543127847	0.05844	0.81742145099016
SLC14A1	0.0837080954519202	0.8145231684629	-0.0541416090858917	0.934943125030587	0.276113000000001	0.474782845456497
HDAC7	0.083671730987317	0.8145231684629	-0.172333796704208	0.738556507224474	0.0984383333333339	0.645951838267189
FADD	0.0836697940904813	0.8145231684629	-0.395155293195634	0.272692742937895	-0.120540666666667	0.58432240713412
TFDP1	0.0836152728944352	0.814534880738293	-0.0261071128838246	0.970632644458507	0.0652100000000013	0.774553415918773
CTDSPL	0.0836017801631053	0.814534880738293	-0.323503376439207	0.414288146732192	0.201402666666668	0.379431342074965
SH2B2	0.083592867655877	0.814534880738293	-0.116621719349860	0.841809730226979	-0.214718666666666	0.313220411537404
PRDX6	0.0835407483077002	0.814565021892598	0.156203216674564	0.768909201237015	0.0665500000000008	0.766563380016891
KLHL13	0.0834919994758835	0.814565021892598	-0.151490556145485	0.777104180604402	0.697862000000002	0.0208784877318267
ST8SIA4	0.083477724961884	0.814565021892598	-0.213058481546798	0.655185554924368	-1.04440100000000	0.042424592410041
CNNM2	0.0834731823711208	0.814565021892598	0.105035766741694	0.8606536389692	-0.0243763333333323	0.926246672706072
FHL2	0.08341183127291	0.81482515095008	-0.0265583921064015	0.970207612777188	0.108595	0.641280432549761
VPS26B	0.083376552501389	0.814850396015408	-0.174535097879962	0.734267769889068	-0.306695333333333	0.206257290355168
ECI2	0.083355126517538	0.814850396015408	0.335322423773744	0.391197106326829	-0.00778833333333266	0.981191596773742
CRB2	0.0832316185288386	0.815067443750836	-0.0365621662834838	0.957280095599186	-0.203844666666666	0.311657354663077
CLSPN	0.083225735786953	0.815067443750836	-0.180570483400346	0.722518525037744	0.0866653333333353	0.765339817431245
TPRXL	0.083215968383111	0.815067443750836	0.36745897881711	0.327265204385924	-0.0869436666666655	0.829828193711794
USP33	0.0831821097783427	0.81521803655944	-0.234002963907093	0.60951860401502	0.0773353333333339	0.734913395435333
GPX1	0.0831207920257227	0.815271505761703	0.0692303490887798	0.911837389834658	-0.0114763333333327	0.969387309763954
GALR3	0.083071632953055	0.815271505761703	0.142885710945491	0.793851492530198	-0.225673666666666	0.447498161237612
WDR65	0.0830509108764994	0.815271505761703	0.075101595697891	0.904410702136818	-0.0309146666666660	0.917298532777554
TSGA10IP	0.0830488594399131	0.815271505761703	0.0555334170417449	0.933409139126562	-0.130943333333333	0.66399337759607
ANKRD13D	0.0830483523267197	0.815271505761703	0.230183946852015	0.618767132693029	-0.0148659999999994	0.95805797537369
LHCGR	0.0830471734518015	0.815271505761703	-0.160248922875170	0.762461161181997	-0.0835986666666663	0.722426540875015
RSBN1	0.083019686304736	0.815334939912188	-0.092929569003789	0.87756010254812	-0.0228953333333331	0.937545603686416
MGLL	0.0828320560856698	0.815754768166546	0.0481451658878290	0.943111020516793	-0.0479243333333334	0.835745709777946
MVP	0.082814070492419	0.815754768166546	-0.00678333015368573	0.99129507664977	-0.229715333333332	0.300335967478278
FAM81B	0.0827936330713644	0.815818607364237	0.248765551125484	0.576962252457532	0.0249183333333345	0.92865132951951
RPL12	0.0827741128568465	0.815834046799631	0.239995580684815	0.598092982260025	0.0345496666666678	0.899614658619676
LINC00221	0.0827177628896483	0.815967882269572	0.225471810011804	0.629710977747725	0.165094333333335	0.450233254347116
CCDC124	0.0826697825500356	0.816093630448644	0.0288371142244522	0.96782691865619	-0.118159666666666	0.567901106219822
MMACHC	0.0826085884887112	0.816093630448644	0.232005041105278	0.613445765196643	-0.0451279999999999	0.855193302520046
USP32P2	0.0825578265272073	0.816115174191564	0.0167901216843668	0.981160061237418	0.213593166666667	0.343611277724373
ATF4	0.0824371874296942	0.816553368471072	0.161991259102276	0.758555563769973	-0.123579999999999	0.540614958546206
LOC143188	0.0822385070872022	0.817495180115421	0.356364774661366	0.345997909309632	-0.343185666666666	0.101631762784143
MECP2	0.0822082862576786	0.817557122520272	-0.363373486885336	0.335749276958773	-0.464552999999999	0.0718886378521549
AP5S1	0.0821972577342493	0.817557122520272	0.0997312134516852	0.867558388686851	0.0643390000000012	0.771153294405787
LRRC4	0.082175015387152	0.817580231291201	0.0485246066544288	0.942450765672493	0.0889446666666668	0.786973845403943
LOC100128840	0.0821253131821226	0.817661245768519	0.0269152891930408	0.970207612777188	0.0574186666666671	0.832554061619867
AP3M1	0.0821178254359582	0.817661245768519	6.30680209323912e-05	0.999900172295419	-0.128205666666666	0.528890172622518
ADAMTS3	0.0820856564946893	0.817770160883151	-0.153804018023298	0.773138261759031	0.331319666666668	0.120847102073075
RRM2	0.08207984813483	0.817770160883151	0.0207142438428176	0.976330597778846	0.327013666666668	0.177814128969052
FLJ42875	0.0820503614377738	0.817892353280381	0.425545796469892	0.215369960032963	-0.0159844999999998	0.966149176465358
SEPN1	0.0819884000598031	0.818004296451419	-0.0524317624217826	0.937831566264837	0.313908	0.149279098704022
ELF5	0.0819816707210146	0.818004296451419	0.257030999575847	0.5632303733857	-0.0486079999999996	0.842204606374644
GNLY	0.0819802681177661	0.818004296451419	-0.328269744687417	0.407301172667012	-0.00319066666666576	0.993756838831997
RFX6	0.0819463179472099	0.818048689475348	-0.114113719713938	0.844272085764738	0.0370516666666668	0.879843042066371
PRKACG	0.0818842177666396	0.818245513109162	0.0383691024237470	0.954545200586067	-0.108512333333333	0.619996256183009
BLCAP	0.0818690421834845	0.818245513109162	-0.0836937775294149	0.892833228561937	-0.0560003333333326	0.823699339501946
NLRP11	0.0818513323121928	0.818246119998384	0.0465167805482778	0.944839700668385	-0.0401569999999996	0.896446774489185
PHTF2	0.0817548543009451	0.818641876647327	-0.128263366809458	0.816825525793169	0.262574333333334	0.231059485569270
UBE2G2	0.0816979975408924	0.818748916169369	0.0171708224102734	0.980994443759145	-0.977812333333331	0.00706146994541395
DNAJB5	0.0816910839671893	0.818748916169369	-0.137849758755609	0.801736842296971	-0.138677999999999	0.496323393705678
ORC3	0.0815756383174256	0.819098087878614	0.195200633325123	0.694852194384891	-0.114967333333334	0.660936100042198
NME8	0.0815161012302302	0.819173577079238	-0.29086969012112	0.487050608234981	-0.105377333333333	0.626274169235363
ZNF141	0.081506368919433	0.819173577079238	-0.240738490167074	0.596798482806084	0.141581666666669	0.626500880282759
UTF1	0.0814847582800385	0.819173577079238	-0.112529694923109	0.847191484945575	-0.0948243333333331	0.729912257977231
DAO	0.081482568626571	0.819173577079238	0.0725952934081142	0.907556175729082	-0.141655000000000	0.583061615740711

ATP8B3	0.0814406817217956	0.819239680942778	0.518760143627791	0.0903775728003269	-0.0217486666666670	0.949376459031393
SDCBP	0.081364382312744	0.819618996757204	0.0233178961918538	0.975348968561756	0.143626666666668	0.484148891768163
TAS2R3	0.0813240981023048	0.819784209883643	-0.0977420067353334	0.869205493224509	-0.0162873333333328	0.96162571048779
UBXN10	0.0813109865478861	0.819784209883643	0.255244945379722	0.566104244002421	0.168544333333334	0.616036866881905
NUP50	0.0812929391984881	0.819784209883643	0.0415832422256637	0.952005386179745	0.150988666666667	0.51786180567444
SYBU	0.0812728066561381	0.819846009644904	0.115446766677647	0.843614348588468	0.175351999999999	0.383593963545918
GPSM1	0.0811291908061697	0.820340005129058	0.320583421624524	0.421958475934190	0.00836866666666717	0.981672346979786
SLC25A20	0.0811155607573352	0.820340005129058	0.180768139143934	0.722159001254958	0.136698666666669	0.523041310312964
MORN3	0.0810398004611716	0.820532198471244	-0.0516207327155829	0.938514797510096	-0.0272233333333332	0.93845553040494
CACTIN	0.0810122990181629	0.820573514586876	0.123113197183046	0.827789978865629	-0.119223333333334	0.58391025729515
HSF5	0.0809860656110715	0.820606662945023	-0.235422013068647	0.60678764664537	-0.0729353333333333	0.77394434422275
KARS	0.0808922782976196	0.821037886678547	-0.365536846912703	0.329800721443396	-0.110414999999999	0.629657237462109
LRRC36	0.0808858793895862	0.821037886678547	0.498227040662896	0.114179995542754	0.075894333333334	0.729912257977231
DUSP8	0.08088524539783	0.821037886678547	0.124940227442574	0.824457843812434	-0.0616536666666662	0.816331815948435
NCAPD2	0.0808664992373258	0.821037886678547	-0.0665756127578538	0.915858484030651	-0.102509333333333	0.67057283279814
HDC	0.0808083240056088	0.821140446469847	0.0442895340688657	0.948385499132766	0.0213613333333332	0.94622978991441
CDK18	0.0807346774045384	0.821411131894489	0.57111986067928	0.0443803345694495	0.0462369999999998	0.847964572928708
PHOX2B	0.0804859968475722	0.822469851312713	0.107414797306042	0.857065724607536	0.0130220000000005	0.967460065937822
PPP4R2	0.0804451099496081	0.822645269942716	-0.159476802627762	0.764179000198617	0.048437666666668	0.842829549490636
FGF1	0.0804208507237456	0.822645269942716	-0.0772988510369418	0.901065188226888	0.0671903333333338	0.829739520310338
SLC25A47	0.0803492114625746	0.82286679096218	0.256593782133153	0.564398995459794	-0.00220299999999991	0.99628178244892
THYN1	0.0803400003400931	0.82286679096218	-0.127042480565877	0.820257254890606	-0.0995419999999997	0.674028958837018
COL27A1	0.0802609797814297	0.823036076658762	0.234488651891593	0.608446568003374	-0.213748666666666	0.316419483204427
RNF31	0.0801465101364427	0.823433731057713	0.215203561577903	0.65076092912169	-0.236327333333333	0.220419507280706
PEAR1	0.0800445069388017	0.823886992199326	0.110423824756207	0.849774125907477	-0.0081766666666653	0.981582681132884
IGFBP5	0.0800290481966981	0.823894887558597	0.279891585159477	0.508631053547956	0.196087333333333	0.464637455466939
SCNN1D	0.080022178348101	0.823894887558597	0.132406429920608	0.810061580929916	-0.173311999999999	0.485054643787725
LPAR2	0.0799448200893605	0.824189341221448	0.132100870734690	0.810300414153187	-0.225870333333333	0.369839415873888
PRRG4	0.07989925863510 1	0.824346910803706	-0.140076513759030	0.79762984905169	-0.0710056666666663	0.842805895171716
RPL39	0.0798694959493204	0.824402690529472	-0.155442168844282	0.770224269831267	-0.0429228333333333	0.888285531317364
LAG3	0.0797633017513292	0.824425222681619	-0.339357962997235	0.382055447975346	-0.178933666666667	0.612712713498171
ING4	0.079745630823751	0.824425222681619	0.0868626518641282	0.887319922815896	0.149543000000000	0.465424659374538
KIAA1731	0.079738493026584	0.824425222681619	-0.0811919355725111	0.896346444707454	0.134287666666668	0.637426515050706
PSG3	0.0797239118986265	0.824425222681619	-0.183110919865969	0.718067440349544	0.0489856666666673	0.902419124106415
C4orf26	0.07971603533016	0.824425222681619	0.223104300558765	0.635247978870818	-0.0291246666666659	0.90945379489823
C1R	0.0797114360784263	0.824425222681619	-0.153366921951537	0.773870060547252	0.0336773333333337	0.896699926823174
GNA12	0.0796961176209779	0.824425222681619	-0.218734982851175	0.642137448592891	-0.0198103333333329	0.942844330601465
SMYD4	0.0796619609086827	0.824425222681619	-0.168342977959368	0.747543826192986	-0.0283779999999989	0.91402300740264
MICAL3	0.0796564246773398	0.824425222681619	0.00218317092365704	0.996532780077613	0.0279168333333342	0.917325389804968
CNEP1R1	0.0796532775558176	0.824425222681619	-0.0718818186869422	0.90837606217316	0.294913666666667	0.124721634754001
LOC100132354	0.07964697268441 9	0.824425222681619	-0.071823708262667	0.90837606217316	-0.0637083333333331	0.852208015389387
FAM120AOS	0.0796444846146979	0.824425222681619	-0.257527216244491	0.563116080558022	-0.0282393333333325	0.915761046438965
THBD	0.0795482973279204	0.824840709810723	-0.376317002446427	0.309701363545704	0.0251386666666675	0.93845553040494
SERTAD3	0.0793502856687992	0.825775900385533	-0.383579113938121	0.293474457479736	0.0445506666666681	0.873663396186202
UBE2L3	0.0793246785839392	0.825851423988258	-0.0378739656204912	0.954958350556272	-0.0460726666666657	0.843676733449892
DCAF5	0.0793168357133406	0.825851423988258	0.112240750944146	0.847454473507881	0.0549909999999997	0.82138507507873
PIK3R4	0.0792754423961434	0.825918124180435	0.0958699583165208	0.872499931194687	-0.0195759999999996	0.953862784105061
LRRIQ3	0.079161330396653	0.826354793489329	0.201303796242117	0.680054545720343	-0.0924326666666663	0.660962515038256
THAP7	0.0791258516831193	0.826392568298105	0.315828947856065	0.433880593421022	-0.0540389999999998	0.814485009599967
ABTB1	0.079112303164022	0.826392568298105	0.268343009749166	0.535217216332192	-0.086266333333333	0.718749100405876
METTL4	0.0789538882946279	0.826664857165768	-0.275626983111775	0.518318935959533	0.0533003333333348	0.824548552404467
MARCH1	0.0789309251917204	0.8267096752137	-0.394103419848741	0.274746792684958	-0.0187043333333331	0.95874970634395
XCR1	0.0788638865722358	0.82686954489093	0.144648122840964	0.790044831580319	0.058137333333334	0.794486775271372
CHST5	0.0788334193401792	0.826987644413982	0.0392481371234075	0.953905313456582	-0.184591666666667	0.344576368488222
A4GNT	0.0788045954152993	0.827047418157213	-0.0685555839438023	0.912149839732916	-0.000741999999999986	0.997953056544486
ZDBF2	0.0787073737375662	0.827537170960882	0.0683934188446274	0.912232638501567	0.225262000000001	0.340052241399374
ACOT12	0.0786144045442975	0.827859732601988	-0.313466040149284	0.438100620341757	-0.0387789999999999	0.88384908879607
PYCR1	0.0785763752211451	0.82790484381792	0.150197497188925	0.779734405920598	-0.473263999999999	0.0539987962707824
LTK	0.078548117587094	0.828018761322676	0.171637337521757	0.739411494892728	-0.0459213333333326	0.90457784770246
GMDS	0.0785277970905764	0.828081614747362	-0.276451371939529	0.517367360081815	-0.105132333333333	0.677667606291399
RBM46	0.0785051652670492	0.828159336274539	-0.16615577609054	0.751830402832405	0.0239116666666666	0.939496718849109
LSR	0.0784130115162604	0.828434154189902	0.25280784965552	0.568985221714455	-0.0724289999999995	0.772139923817113
CACNA1G	0.0784096911565425	0.828434154189902	0.460397009245405	0.162787294570657	0.140250000000001	0.59825322346796
LANCL3	0.0783526064465566	0.828594557264245	0.0792496030466945	0.898424765944956	-0.0245796666666661	0.944454301779885
MIER1	0.0783476811610858	0.828594557264245	-0.0461405085213146	0.945364757579857	0.111045333333334	0.58051867264498
FOXJ3	0.0782990402831633	0.828751453782627	-0.325912858504654	0.409206882509236	0.0709133333333335	0.748799115646098
GJC2	0.0782704700650386	0.828847594955247	0.150699415380291	0.778777977391526	-0.0168413333333322	0.962219686887808
GOLGA2P5	0.0782610081512104	0.828847594955247	0.342142942546862	0.376742544476676	-0.0713350000000005	0.759696248427462
ZNF99	0.0781909350350672	0.829060049879976	-0.548792096769915	0.0613152653558691	0.295200000000001	0.313220411537404
OGT	0.0781901123620454	0.829060049879976	-0.247444571525646	0.579745218830252	0.0333746666666674	0.899614658619676
CLN6	0.0781157462597351	0.829246761295502	0.0478112063408989	0.943472656931067	0.146542666666666	0.508034564998038
OLIG2	0.0781145355045524	0.829246761295502	0.0628296183404904	0.922290895114464	-0.122310000000000	0.607789868477511
PRDM8	0.0780779056950843	0.82934661901331	0.298022788962300	0.47226540109898	0.0170046666666672	0.955115242974667
LILRB1	0.078043236707305	0.829366028455963	-0.33625985882914	0.389442467555813	-0.0502856666666663	0.910190419163007
FOXN2	0.0779883033254388	0.829377112336399	-0.233601200112489	0.610436189314218	0.129468000000002	0.525218637875881
EAPP	0.077987889812688	0.829377112336399	-0.0653632244919658	0.918295693698803	0.245267000000001	0.364095872333505
APLN	0.0779108358954329	0.829658582627836	0.0601203469650597	0.925649366494154	0.195290000000001	0.337307065334106
GIT1	0.077838749084861	0.829855506783046	-0.0536606059506995	0.935777496327359	0.0675226666666679	0.762898679429892
NANP	0.0778038302683949	0.829855506783046	-0.163763287421291	0.754968203901817	0.117129333333335	0.567224087161503
C1orf56	0.0777983156335531	0.829855506783046	-0.188850923272290	0.707236456264125	0.168050000000000	0.414405354802607

STGC3	0.0777749447156848	0.829870140879399	0.0184409833259925	0.97902968742576	-0.0341483333333328	0.899843700231124
PHGDH	0.077727811394459	0.829914357380686	-0.167484450435182	0.748960978799932	-0.173173333333331	0.460374458843217
CCDC63	0.0776684937771161	0.830033939199431	0.202842458030466	0.677604697677494	-0.0953203333333328	0.686659637953633
MAGEB1	0.0776567089848747	0.830033939199431	-0.149610124505731	0.781645288607494	-0.220021333333333	0.438019817548948
ZNF821	0.0776097136426419	0.830033939199431	0.293688194282414	0.480614908214975	0.312743000000001	0.266896890079960
PCDHB17	0.0776062675221145	0.830033939199431	-0.148800726892987	0.783041584899534	0.117158333333335	0.617314304298331
GAB1	0.0775456298187541	0.83018333245354	0.0460422781707689	0.945364757579857	0.0430553333333339	0.863745180020314
TRIM67	0.077542677836099	0.83018333245354	-0.0570624339319227	0.930775493791569	0.0523826666666667	0.85244489457123
PRB3	0.0774952615331534	0.830316649560768	0.146883567101574	0.785920387079635	0.00438000000000074	0.991175862027014
LOC100287166	0.0774734645659416	0.830316649560768	0.152874042913570	0.774725957219946	0.123184333333334	0.553892046464523
MCM2	0.0774289451498485	0.830534824833924	0.0573981898589736	0.930490408235626	-0.099106333333333	0.66807888867384
AGBL4	0.077362255432404	0.830763943835194	0.0523328729588596	0.937831566264837	-0.0304816666666662	0.90778598212344
RNASEH2A	0.0773444720310521	0.830763943835194	0.0957095380057957	0.872537273851412	0.101877333333333	0.61744726500365
CMTM7	0.0773346762123405	0.830763943835194	-0.0392051921821778	0.953905313456582	-0.178539333333333	0.575378149225683
RPLP1	0.0773035109870981	0.830763943835194	0.377299914218081	0.307297021174057	-0.0482433333333347	0.839740465054742
PCDHB13	0.077292280977819	0.830763943835194	0.0146550443842514	0.983444353656142	0.307193000000001	0.184184161944404
NHLH1	0.0772877331021062	0.830763943835194	-0.0514007808490291	0.938729151196048	-0.0999943333333324	0.649420534269272
TEX12	0.077280527534794	0.830763943835194	-0.198387115230664	0.686723045322017	-0.0851929999999993	0.72003090084477
LOC100506965	0.0772525013613557	0.83078782121405	0.329434540181243	0.404474769300112	-0.0744273333333331	0.753937973093065
ESF1	0.077222406662445	0.83091333276398	-0.0513108877964914	0.938890620237414	-0.420388666666666	0.0558976805165229
EXOC8	0.0771196472552553	0.831336368451807	-0.139447079006364	0.798450177373409	0.0504270000000007	0.871104061464043
HOXD8	0.0771154876274830	0.831336368451807	0.454862789912752	0.169205181987923	-0.238092666666666	0.337994438406310
PLOD1	0.0770945242101777	0.831336368451807	0.119652902688796	0.835740437824474	-0.135093666666666	0.509441315337042
APOBEC3C	0.077011889870118	0.831432840044339	0.220929829543316	0.638522059865506	0.092085	0.673396156479105
NCDN	0.077004438601858	0.831432840044339	0.103470445362943	0.861943478503987	-0.055479666666667	0.811997756288597
IFIT5	0.0768769674031256	0.831980249035714	-0.0469718803100055	0.944081286115546	0.140178666666668	0.523453039510001
ARFGAP2	0.0768444189647088	0.8320857489809	0.399022160888048	0.266198592726045	-0.201941000000000	0.287956132210494
CUEDC1	0.0767073781694679	0.832459652004448	0.0201481471572350	0.976956866647027	0.00024066666666698	0.998839825970976
TRAPPC10	0.0766740695363762	0.832526815184028	0.211471683970397	0.659412013514194	0.134838333333334	0.51139718772887
PLCXD1	0.0766652851394938	0.832526815184028	-0.0944329713900276	0.874384897652873	-0.0854940000000001	0.705698790800484
CRLF1	0.076642813080957	0.832535734916579	0.483556487007372	0.135120152804230	-0.104047000000000	0.711180779277199
CCNYL2	0.0766157420796909	0.832641773137354	-0.235742553650996	0.606716022115166	0.159134666666668	0.631408228698652
RGS4	0.0765493306645525	0.83295699514086	-0.0569846141412464	0.930784376757936	0.0254060000000004	0.925457630845019
DCAF4	0.0765380431037964	0.83295699514086	-0.0777000882774794	0.900452756580846	-0.0905759999999982	0.716517864791776
APPL1	0.0765349784608949	0.83295699514086	-0.186855586022296	0.710596654649373	0.176361333333334	0.359771290730514
ACACB	0.0764908435662699	0.833018707242402	0.196967815227474	0.690061983084583	0.0119550000000011	0.971256095411335
METTL7B	0.0764402612892291	0.833184131209985	0.254581983084753	0.567579021017007	-0.365768666666667	0.0660125264725009
CCKAR	0.0764278105818268	0.833184131209985	0.0638447000706768	0.920459698398106	-0.014471	0.968819358319328
TBPL1	0.076419584242865	0.833184131209985	0.195081829830805	0.695157516663803	-0.0166949999999991	0.971623441171626
SLC17A8	0.076415235569178	0.833184131209985	-0.103409503676016	0.861949447881126	-0.0169939999999992	0.956398070618612
IGSF5	0.076380508775127	0.833305124077573	0.422534782355534	0.221428501305239	-0.0971046666666667	0.707459630816261
NAPRT1	0.0763752878096205	0.833305124077573	0.0938736790452109	0.875355472414883	-0.137493666666666	0.697592019153389
MAP7D1	0.076354794675965	0.833368948461845	-0.332298517910692	0.39832322900678	-0.0769173333333322	0.729912257977231
DCPS	0.0763121403326205	0.833485306849093	-0.0141655318532198	0.983444353656142	0.0846856666666684	0.700855603017082
GADD45GIP1	0.0763050274197217	0.833485306849093	0.43449774801959	0.199121860533382	-0.0437906666666652	0.880131955403697
CAPN11	0.0762427809061731	0.833614093365595	-0.175459227105571	0.731991966468784	-0.127109000000000	0.774104133965233
CMTM5	0.0762272960851692	0.833645797289519	0.0833104478668868	0.892894681640269	-0.196908333333333	0.43400381736769
AMZ1	0.0761975098001052	0.833687415434091	0.232850777591534	0.611711680668619	-0.194305666666666	0.309421300309741
ZNF556	0.0761963530334901	0.833687415434091	-0.0785496382886689	0.899281198071059	0.088003333333334	0.767958022875087
TSG101	0.0761260486947031	0.833889473147059	-0.0831936347749867	0.893287218356307	0.139726000000000	0.504686960017307
PCDH15	0.0760877243882435	0.834046828141361	-0.0693849336402226	0.91172160664395	-0.0174939999999998	0.959032046952242
TTLL10	0.0760726611475522	0.834046828141361	0.164965873676331	0.753120404549573	-0.0444509999999995	0.901507943996785
GLP1R	0.075995808172831	0.834237228905603	0.204619744268984	0.674108447036116	-0.0597583333333327	0.82667899725046
ZNF322	0.075994542847721	0.834237228905603	0.105082315568964	0.8606536389692	0.1840185	0.376882447735926
PTCRA	0.0759478983277407	0.834355829278852	0.229785622203711	0.61942454408784	-0.112580666666666	0.742452599762094
LONP2	0.0759018914488823	0.834583150624728	0.00870104889339358	0.98946753270812	0.111124666666667	0.637596150841031
KAZALD1	0.075866412454393	0.834727898218458	0.232740399726967	0.611941461795609	0.130056333333335	0.517547647029412
CALR	0.0758582268432493	0.834727898218458	-0.202299942761764	0.6793987774232	-0.0560033333333325	0.814062949894134
DSN1	0.0757783307096876	0.834954391437476	-0.326502929336094	0.409206882509236	0.0181746666666671	0.94678260229888
BCL2L13	0.0757589143043653	0.834954391437476	0.0240089432034452	0.974510809875905	-0.0991360000000004	0.637602236970228
METTL21B	0.075704831678693	0.834954391437476	0.479689740271496	0.138333709024807	-0.294958999999999	0.160589525668831
ORAOV1	0.0756786849993153	0.834954391437476	0.0534834616642771	0.936236208222134	-0.0293716666666656	0.910513033220957
DNM1	0.075641631101944	0.834954391437476	0.0571612220880745	0.930775493791569	0.0237530000000012	0.931446587444396
CYP26A1	0.0756289595209592	0.834954391437476	0.132209899192954	0.810300414153187	0.000805333333333389	0.997791479103696
RPRML	0.0756284670797453	0.834954391437476	0.136823436081081	0.803720303246183	-0.199193333333333	0.4423818632121
HP1BP3	0.0756244389767342	0.834954391437476	-0.313628958696947	0.438046311563112	0.0435200000000004	0.855886648212195
PPY2	0.0755835552740242	0.834954391437476	0.489352576071845	0.125920888922881	-0.0844826666666657	0.769472529709702
HTR3A	0.0755756260442777	0.834954391437476	-0.0472954390483894	0.943981149721771	-0.0986683333333332	0.759445936839873
KCNIP4	0.075575538442967	0.834954391437476	-0.107793539189155	0.856450662791083	-0.067422333333333	0.837681049708462
PPP5C	0.0754947275310971	0.83503158972655	0.141878965545327	0.794791289237846	-0.0642483333333317	0.771153294405787
XRCC3	0.0754841873152624	0.83503158972655	0.0759331901535247	0.903510413986113	-0.112640666666666	0.608679471465191
HS3ST3B1	0.0753882718211787	0.835219701184006	0.189625141207496	0.704928430617595	-0.078882666666666	0.721454370213253
GLIS3-AS1	0.0753787481549569	0.835219701184006	-0.0301033152321975	0.966396286514145	0.0672526666666675	0.81000174583549
IDH2	0.0753230685521912	0.835219701184006	0.0133907134754628	0.98412883278237	0.095493000000001	0.696194439936211
SASS6	0.075312320443608	0.835219701184006	-0.00480492788073124	0.99311930576133	0.142714000000001	0.656941730375963
CYFIP2	0.0753082852888902	0.835219701184006	-0.0116037500022117	0.986661750359244	-0.0888376666666659	0.723993424337833
TAF1D	0.0752941862159734	0.835219701184006	0.00169713084402816	0.997793696211823	-0.0834458333333331	0.759034160486144
SKIL	0.0752529181727578	0.835219701184006	0.0164261538781892	0.981892085795004	0.257966333333333	0.239053553226839
ST3GAL4	0.0752517299738485	0.835219701184006	0.347328285225594	0.363662467720282	-0.457976333333333	0.171771824059364
PWWP2B	0.0752377997993639	0.835219701184006	0.382982137455969	0.294467842756818	-0.0427403333333332	0.878139763624324

MAB21L2	0.0752354594491055	0.835219701184006	0.33321992107078	0.396894500849691	0.0843396666666676	0.78885077801625
COMMD2	0.0752005469972527	0.835219701184006	-0.167104524712680	0.749664019651347	-0.0180996666666657	0.950980037243398
FGF13	0.075171786373767	0.835219701184006	-0.180002938077866	0.723716289903531	-0.133808333333333	0.518836392563847
NLRP13	0.0751606532895647	0.835219701184006	0.125010771494591	0.824457843812434	-0.210145666666666	0.294899302599823
HINFP	0.0751531268020087	0.835219701184006	-0.115920350234470	0.842733236705133	-0.257880333333333	0.187638103900419
AMPH	0.0751197918097586	0.835219701184006	0.0550312028863261	0.93406507597279	0.0139373333333340	0.969328142683525
ACTN2	0.0751183860147105	0.835219701184006	-0.116797120897603	0.841562044027187	0.279355000000000	0.148262582993822
TBCK	0.0750870392664494	0.835219701184006	0.0316731477765526	0.963712086545628	-0.00310099999999873	0.993677488355417
ZNF436	0.0748350731445654	0.836320923926061	-0.274259360310781	0.520857714835269	0.0510160000000001	0.853219850381138
SNAPC4	0.0748273008415104	0.836320923926061	0.156843840323326	0.768401006769299	-0.241906999999999	0.194593860853488
AGBL1	0.0748193721477641	0.836320923926061	0.190250268512786	0.704208582824765	-0.0368463333333326	0.910190419163007
STIL	0.0747404134658764	0.836758407937524	-0.106491033860122	0.858072018696671	0.0336906666666674	0.913087973739129
DENND2C	0.0747197245587051	0.836823370251241	-0.297473104141881	0.473800338702402	0.049543333333334	0.83662536731961
FGF5	0.0746229075655494	0.837240425152728	0.0869966032626362	0.887055939766881	0.0463586666666670	0.874243670972648
XPOT	0.0745919870266276	0.837370798298244	-0.112557105189476	0.847191484945575	-0.433844333333332	0.0552583431571053
FST	0.0745464811517222	0.837556460984957	0.0865438061003127	0.887493600482322	-1.08413200000000	0.0364080466199869
CCDC148	0.0745146637243729	0.837556460984957	0.00580095864287109	0.99251028338798	-0.0261570000000002	0.925476944639214
SFRP4	0.0745124230973435	0.837556460984957	-0.0996735817015478	0.867603998426271	-0.362456000000000	0.159176629475828
POLR3H	0.0745035514021103	0.837556460984957	0.0399838515199914	0.953370115695118	-0.431274666666666	0.096678383306742
CSDE1	0.0744419897074753	0.837622682032203	-0.347865975421206	0.362015758823702	-0.115616666666666	0.572182764145299
SPRYD4	0.0744367584228513	0.837622682032203	0.327944713822367	0.407625498965711	-0.0212609999999994	0.936634943217749
ALG2	0.0743412800710967	0.838136707339257	0.178607180582444	0.726444926461881	-0.0702539999999977	0.760989915219907
USP44	0.0743352823373314	0.838136707339257	-0.148039664344519	0.784080588385358	-0.0343033333333327	0.929037423741365
WDR35	0.0743025398778165	0.838211365773705	0.217158952583434	0.646495351690352	-0.528119	0.0584908310916042
ULBP2	0.0742675916600908	0.838367443867554	-0.0998092341217574	0.867558388686851	0.112883333333334	0.580149967309238
GLT8D1	0.0742170346728612	0.838555968837431	0.175601507436648	0.73187914820204	0.0806376666666682	0.707459630816261
FAM120B	0.0742007141031134	0.838592965546641	0.141115813132537	0.796333933122002	-0.0812386666666671	0.752384210059315
SLC4A11	0.074146503940871	0.838804830677983	0.425018019122098	0.215733128998344	-0.195536666666665	0.310158645250256
LOC100507530	0.0740173223988328	0.839297971775227	-0.0748030538163932	0.904466414650249	-0.127300000000000	0.661470161772113
MELK	0.073804539036063	0.839913205493915	-0.0141007634675218	0.983444353656142	0.172540000000001	0.39835425096141
ADRBK1	0.0737756445396439	0.83996061166547	0.00120861856111213	0.998348925592729	-0.00764966666666483	0.98173288785111
RUNX3	0.0737489325387344	0.83996061166547	-0.274921662527831	0.520064783279337	-0.0923869999999993	0.798344353938564
ERMP1	0.0737444436805726	0.83996061166547	0.237945576632859	0.602142642649819	0.0746053333333335	0.798344353938564
SSX5	0.0737290017156953	0.839978097006361	0.187182802274176	0.709833349594307	-0.129967666666666	0.540614958546206
CTNND2	0.0736995707146204	0.839978097006361	0.0270503453275721	0.970207612777188	-0.122189999999999	0.5488879250835
LRP8	0.0736860083971377	0.839997448033998	-0.240279648098664	0.597862631330416	-0.186648333333333	0.373594956320212
RNF111	0.0736415412853913	0.84014693905218	-0.00613394263227027	0.992286213306377	0.00817133333333342	0.980779858267938
DEFB129	0.0736109712697452	0.840212689577223	-0.0513686647558564	0.938729151196048	0.0649630000000006	0.846457250180447
IGFBP3	0.0735883650764207	0.840212689577223	0.0165135631853263	0.981833953634016	-0.0220393333333324	0.935261323444314
MOCS1	0.073570788179515	0.840212689577223	0.0839020281178228	0.892716669164583	0.00287833333333406	0.993756838831997
TMEM132C	0.073443951051242	0.840742040916408	0.0984019752732596	0.868441567107477	-0.0178816666666658	0.960210715969161
NCAPG2	0.0734325051020203	0.840742040916408	-0.0565587772667666	0.93176886339657	0.0907503333333346	0.704370299831112
RPP40	0.0733570952646605	0.84115642607279	0.241811379321041	0.595095850165855	-0.230317333333333	0.249209120593054
IL12B	0.0733395167218964	0.841163714902093	-0.107008883645503	0.857671866148626	-0.0660126666666663	0.804741883326752
INTS5	0.0732996219688336	0.841163714902093	-0.163932479198769	0.75491314198126	-0.0503736666666665	0.837778415103206
SPHKAP	0.0732955709084594	0.841163714902093	-0.0482862889282660	0.942853417571722	-0.023424333333333	0.93682999997207
C9orf163	0.0732499603934668	0.841363831979142	0.285695060883946	0.497004323673415	-0.0117019999999991	0.981191596773742
EXO1	0.0732403342116346	0.841363831979142	0.180528440661998	0.722641197261641	-0.0519353333333324	0.849242779775804
ELOF1	0.073215169689398	0.841457256376994	0.209014059063560	0.665595909869988	-0.137840999999999	0.48608244495005
LILRB4	0.0731949181869406	0.841519302983025	-0.189685394793796	0.704928430617595	-0.0270609999999993	0.944218475609619
CLEC5A	0.0731409969597545	0.841796376771184	-0.265587719461993	0.540074581830336	-0.0395506666666664	0.870662337224757
SDPR	0.0730790264805263	0.8419429022166	-0.232327465562679	0.612658112747496	0.00761600000000008	0.983690244124512
TRIM72	0.0730592565833623	0.8419429022166	0.0709724937920223	0.909400532983434	-0.212722666666667	0.35332082621686
LSAMP	0.0730341554212784	0.8419429022166	0.183577989888258	0.718067440349544	0.170761333333334	0.631192833987568
TACC2	0.0730116240088301	0.841949690018274	-0.148940503894577	0.783041584899534	0.00872866666666758	0.980536641691963
MAPK3	0.0729503928083754	0.842189464215531	0.307457466347687	0.452689746287922	0.177487666666667	0.388303383752735
ANKRD30B	0.0729350972009153	0.842189464215531	-0.0737793510393478	0.905506084101768	0.0537998333333338	0.816134952712854
MAGOHB	0.0729319243680433	0.842189464215531	-0.00551952717816434	0.99251028338798	-0.00665166666666634	0.986290211615582
OLIG1	0.0729093273456987	0.84226646189219	-0.130435218273470	0.812271254601992	0.0515633333333344	0.862744795576892
OR51I2	0.0728573948191943	0.84246346781668	-0.104120646171853	0.861364842664127	-0.217268000000000	0.512138212509218
TOMM40	0.0727917570449465	0.842480165045862	0.089363676420983	0.883460564717377	-0.395457000000000	0.0525641773262741
ITIH2	0.0727701123255013	0.842480165045862	-0.0855269949182699	0.88947685957198	0.210499	0.28487334684302
ELOVL1	0.072768679095134	0.842480165045862	0.154921594292184	0.7712622972511	0.220106333333334	0.367368801980436
PTGER2	0.0727246810688744	0.842480165045862	-0.0392591693471005	0.953905313456582	0.00979733333333456	0.986623910782426
TMED8	0.0727137974504582	0.842480165045862	0.109050793279826	0.852975887741054	-0.0551049999999995	0.862509125961694
CST11	0.0726616973721307	0.842480165045862	-0.0937970122243676	0.875355472414883	0.0087316666666661	0.98256318768706
SIRPD	0.0726571882569422	0.842480165045862	-0.0665560263292895	0.915858484030651	-0.0119333333333334	0.973644794487795
RASA2	0.072650608852115	0.842480165045862	-0.30744197020306	0.452689746287922	0.647969333333334	0.0392329313827969
CHM	0.0726349602821158	0.842480165045862	-0.439314998240859	0.190921419072490	-0.0114029999999995	0.97097048443105
KLHDC3	0.0726298288758573	0.842480165045862	0.114416714848211	0.844272085764738	0.179774000000001	0.341508291794679
CCDC108	0.0726227762887569	0.842480165045862	0.222090641799951	0.636477566065103	0.0197233333333338	0.94375604597120
PCNA	0.0726117466270186	0.842480165045862	0.165409130438526	0.752352242644389	0.0507533333333342	0.835381092237856
CLCA3P	0.0726020020736598	0.842480165045862	0.192437409425702	0.700450711372106	0.00600166666666672	0.985884500368235
CYS1	0.0725309982031446	0.842583418781564	-0.280641988769122	0.507460240190768	-0.0495806666666663	0.895510661232872
POLK	0.0725020267996759	0.842583418781564	0.0834075084569599	0.892833228561937	0.237942000000001	0.297888842945994
HPS4	0.0724744576689239	0.842583418781564	0.0871510240600383	0.887055939766881	0.232368333333335	0.309421300309741
STOX2	0.0724700126572603	0.842583418781564	0.243531857596975	0.59036707181346	0.140377666666668	0.503217861354116
THY1	0.072411329972073	0.84280506661071	-0.105289912511422	0.86011776595122	-0.151233666666666	0.579470077554465
DLX5	0.07237242113578	0.84280506661071	0.07129464932082	0.90912843135453	0.113783666666667	0.570953730203949
CNOT1	0.0723720851910817	0.84280506661071	-0.0839971562209749	0.892678844847032	-0.162146666666665	0.443378989641463

MYT1L	0.0723200666739548	0.842868289116268	-0.157119943662994	0.768136591637483	-0.0213109999999995	0.94678260229888
NOL8	0.0722685325288065	0.842883521410073	-0.0988619113877635	0.868042453226125	-0.098561333333332	0.72926287896686
NTN3	0.0722591992453166	0.842883521410073	0.174844787443127	0.733598828800337	-0.00485633333333319	0.992493658646832
SIX1	0.0722117106872709	0.8429905660351	0.238037469468255	0.602142642649819	-0.0257526666666664	0.933506612109668
SNX21	0.0721897806956873	0.8429905660351	0.260613499451934	0.553540075799904	0.00609366666666696	0.986350009478474
VIP	0.0721770376460306	0.8429905660351	0.00085563140314679	0.998745415362821	-0.108658333333333	0.631192833987568
BARX1	0.072173853013157	0.8429905660351	0.143357118738140	0.79287526984225	-0.111569333333332	0.754488794029141
ADORA1	0.0721640853351581	0.8429905660351	0.273115031641200	0.524473593886977	-0.266294	0.246567852901023
FGF19	0.0720490158213877	0.843184248522535	-0.0340042839560545	0.95993706312687	-0.144510333333333	0.60814016771842
PAX9	0.0720354187398218	0.843184248522535	-0.099035841286501	0.868042453226125	-0.0988703333333326	0.670071773006358
TMEM109	0.0720296977946534	0.843184248522535	-0.331916391799349	0.399068746068573	-0.0326913333333321	0.907374738017166
MGAT2	0.0719784012759569	0.843366440646804	-0.0181563969384853	0.979327298872732	0.0305920000000001	0.90821618556002
MSH6	0.0719682983522028	0.843366440646804	-0.00115100196960806	0.998383694263455	0.00700900000000088	0.983159241812861
JUN	0.0719417507905217	0.843405837923246	-0.106040557725854	0.858902068936183	0.619745666666667	0.0140473413958925
DSPP	0.07192360622081	0.843405837923246	-0.0276030771180818	0.96977908357033	0.140483333333334	0.534227067294365
IRG1	0.0719146355481985	0.843405837923246	0.057048008012719	0.93078214097382	0.0488206666666668	0.861945621270498
ARL1	0.071906797755732	0.843405837923246	-0.211930016507523	0.658364968650984	0.0158086666666668	0.959161379322177
PLXNB2	0.0718815627755338	0.843405837923246	-0.0621724321574976	0.92301259447339	0.171269000000000	0.36541451620531
PWP2	0.0718522833199451	0.843405837923246	0.295405897055700	0.478306608395718	-0.352196666666666	0.252579022109626
DNAJB7	0.0718496143205454	0.843405837923246	0.0578060617174786	0.929715247340158	0.0899133333333333	0.680436423395844
SLIT3	0.0718351738857022	0.843405837923246	-0.0957651733329921	0.872537273851412	-0.0250333333333325	0.947805707169764
ZNFX1	0.0718156296743757	0.843405837923246	-0.515772041162772	0.0934594179158924	-0.151727333333332	0.602084409940976
BSND	0.0717938537412302	0.843405837923246	0.184774318795687	0.716020884288485	-0.176941666666666	0.523453039510001
SLC22A12	0.0717341748620216	0.843718584867517	0.171464084888430	0.739541495555634	-0.0750383333333341	0.853541852582193
POLR1D	0.0717021572605687	0.843791409319414	0.107947333975450	0.856008265865111	0.0608310000000011	0.780704356521475
NRIP1	0.0717016967373795	0.843791409319414	-0.262470590184521	0.548787322027919	0.276092333333335	0.162661087494939
TNFRSF4	0.0716580648319508	0.844002073836238	-0.328633820653222	0.40666876162787	-0.0427466666666666	0.903274690789156
FAM131A	0.0716168204303806	0.844197553483168	0.160847807198893	0.761016253411733	0.203751333333334	0.287558911797493
MUL1	0.071574185372003	0.84431105919992	-0.258956403212295	0.558685853472518	-0.0133573333333325	0.972777106291215
APOBEC4	0.0715357528080681	0.844445640939703	0.0319811314027273	0.963062370853298	0.0316543333333336	0.918760683387302
ACTRT3	0.0715091227762002	0.84454813669758	-0.197565386696436	0.688668717091024	0.0308093333333340	0.903774117860245
ASIC2	0.071469673051411	0.844615117680176	-0.237677023914838	0.602664863375033	-0.0507406666666662	0.829662675541773
MAB21L3	0.0714587187761295	0.844615117680176	-0.136592237119343	0.804254387037059	-0.00534366666666646	0.989433129523288
CCDC42B	0.071446004642057	0.844615117680176	0.0436375426619619	0.94915564770945	-0.155226666666666	0.479018181134391
RFT1	0.0713874264547789	0.844679351023016	-0.0210780270266493	0.97607156937294	0.0201708333333345	0.94367036893426
SPTY2D1	0.0713873609717573	0.844679351023016	-0.394648234785797	0.273838292759582	0.0377813333333336	0.877641904580634
PCDHB8	0.07138332256376	0.844679351023016	-0.346211600049668	0.366023660299226	0.201526333333334	0.60378773986659
KLHL29	0.0712569594819316	0.845148507953709	0.15035044841979	0.779266896045314	-0.0190953333333333	0.949295980201336
FMNL3	0.0712420246550827	0.8451765971474	-0.264134143232665	0.544525995334757	0.143537333333334	0.585584889305174
RRM2B	0.0711973006302844	0.845327224582509	0.507257832052517	0.103996171350646	-0.208192666666666	0.300274668021666
ARHGEF2	0.0711668253682848	0.845414342045116	-0.0738502094967492	0.905506084101768	0.0636216666666678	0.777406710539424
RASGRP2	0.0711549536898437	0.845414342045116	-0.0314245524751052	0.964445978064237	-0.142173666666665	0.614081498359193
DNAJC14	0.0711334594078565	0.845465646108057	-0.310145142593203	0.446321819288896	0.181361333333334	0.343143699851351
ZFP3	0.0711091796672372	0.845480763755973	-0.0948570613303233	0.87373454493148	0.127339333333334	0.546434057395997
CDH18	0.0710901461959252	0.845480763755973	0.0635666934220529	0.920855211745173	0.0703333333333334	0.770112696597648
SUSD2	0.0710612851090295	0.845523332776925	0.0565358712284736	0.93176886339657	0.0720993333333335	0.764792949321967
GPR6	0.0710112862251198	0.845717883263012	0.167630886776576	0.748585550487178	-0.0591893333333327	0.87152108397136
GLIS3	0.0709516767783089	0.845802102942599	-0.112049713213268	0.847490786325228	0.353133000000001	0.107866708545887
GTF2E2	0.0709500955047042	0.845802102942599	0.292620593438477	0.483293572397754	0.0611066666666679	0.795703940655181
PP12613	0.070880126794081	0.845995243433244	0.0280930474975189	0.968668367660302	-0.0331699999999996	0.903864363916236
KIAA1586	0.0708713570043444	0.845995243433244	-0.0492090523172473	0.941842562680095	0.0370526666666674	0.906093399285191
ZNF225	0.0707222459210958	0.846541783815339	-0.222265840544905	0.636309362271551	-0.0139939999999996	0.964514058161652
HHAT	0.0706168571506463	0.846716316371747	-0.0951497527519021	0.872962271153999	0.214225333333334	0.278559146219942
OR3A1	0.0705991367643041	0.846716316371747	-0.217496878834294	0.645392535370888	-0.219099666666666	0.255777756723362
KIF2A	0.0705941526177879	0.846716316371747	-0.302039585959185	0.462229407853213	0.0848276666666676	0.693849443371592
ANKRD16	0.070591862162244	0.846716316371747	0.256354594393764	0.564430426783556	-0.0554149999999989	0.829424296489523
ITGA7	0.0705901504272331	0.846716316371747	0.0999764986919333	0.867290404865575	-0.307194333333332	0.17804508288096
MCM4	0.0705823925316592	0.846716316371747	0.0418791641449218	0.951989245458556	-0.0554419999999998	0.818669300716516
TRPM8	0.070572000113277	0.846716316371747	0.0718817424076403	0.90837606217316	-0.0261746666666667	0.931446587444396
FGF18	0.0705616950476405	0.846716316371747	0.0142731932560773	0.983444353656142	-0.117754666666667	0.765276224002577
ANP32B	0.0705475492590082	0.846716316371747	0.149808644234466	0.781147126980188	0.0354240000000009	0.892562516616591
SMARCAD1	0.0705136567458209	0.846864781836474	-0.185650559671001	0.714043644840406	0.246911000000000	0.207686482144783
PSAPL1	0.0704993994478259	0.846888549093824	0.248449557811647	0.57746120283043	0.0783903333333341	0.776112195351357
KLF13	0.0704731789723083	0.84698828730374	-0.323181082425156	0.415461412498052	-0.0484956666666658	0.842903889380894
DDA1	0.0704250965460831	0.847160077750527	0.220949641377742	0.638522059865506	-0.00016299999999924	0.999418989735238
TRIM51	0.0703385829649021	0.847422181965673	-0.0344259177704719	0.959404955370931	0.161580666666667	0.512796060754107
SPRR1A	0.0703083080004185	0.847422181965673	-0.0366925310385472	0.95697345043597	-0.164654666666666	0.481989564291462
KIAA1432	0.0702496939329973	0.847521168699173	0.314646606676246	0.437013962884228	0.104800666666667	0.682880869852867
EMC1	0.0702364969119738	0.847521168699173	-0.347246197394092	0.363761036292222	-0.00743166666666456	0.982398683576954
STARD8	0.07020448555394	0.847559362726196	-0.0653748514604129	0.918295693698803	-0.183302999999999	0.486290502670036
KIAA1199	0.0701777994605996	0.847628350993701	-0.117132729054052	0.840972703975817	-0.207450666666667	0.324443655582244
TMEM115	0.0701549560933776	0.847628350993701	0.0698876938263644	0.910885619751085	0.118551333333334	0.567355756887702
NR1H3	0.0701330124208267	0.847628350993701	-0.0180660401313149	0.979542365367084	0.197522000000001	0.315903409877569
THAP4	0.0700530351130793	0.847986744002199	0.129592761422611	0.81383917135971	-0.0540593333333330	0.811039304529434
ZMAT3	0.0700097090339859	0.848137187520615	0.194733693707812	0.695823488943761	0.0375223333333342	0.929594956567283
TPTE2P6	0.0699838975833266	0.848137187520615	0.266004913751140	0.539310262140605	0.187667000000001	0.320296173114253
SPDYA	0.0699667370124014	0.848137187520615	0.280160788733072	0.508074118826263	0.0633980000000006	0.795446186721078
CDCA8	0.0698642006291902	0.848584515107334	-0.0536615757455613	0.935777496327359	0.00222633333333448	0.994917858972776
ADAMTSL4	0.0698441354417344	0.848606292295712	-0.290961100042152	0.486880079690803	-0.219158666666666	0.36541451620531
OR10A5	0.0698397379775084	0.848606292295712	0.00765060110375714	0.990405674923721	0.0542978333333334	0.843213673943937

RASSF10	0.0697215391605032	0.849038796330032	-0.0579419678149395	0.929551006206862	-0.110603666666666	0.694867958798056
FLJ26850	0.0697057632183412	0.849038796330032	0.130562535261905	0.812271254601992	-0.0505709999999998	0.841976773189511
SNX3	0.0696979581929166	0.849038796330032	0.193064745730329	0.699002603433222	0.131362	0.59717035375984
MT1M	0.0696733962686765	0.849127902355706	-0.236629065301194	0.604679221319698	0.0358570000000007	0.921287736444926
HIATL1	0.0696280033142427	0.849349159213877	0.0896896071808086	0.88318195439136	0.0981058888888886	0.659036556301843
HRCT1	0.0696061780560454	0.849363028106944	-0.258483138247175	0.560093304159111	0.399936333333335	0.194800671491001
IQSEC1	0.0695497774399949	0.849378408080382	-0.00513630443732426	0.99251028338798	-0.0702419999999989	0.774104133965233
APLF	0.0693980109787433	0.850007467706599	-0.094863383437666	0.87373454493148	0.123961333333334	0.68171195530621
RPS27	0.0693857368206151	0.85001848320928	0.175930841263938	0.731278459622201	0.0441191666666669	0.870002591217953
UCP1	0.0693616688953348	0.85001848320928	0.160882534720720	0.761016253411733	-0.0195803333333329	0.95831174299246
GPRC5C	0.0693255174418436	0.850133698540806	0.482934194127385	0.135560525489292	-0.0321896666666662	0.930620998877894
HOXA-AS2	0.0692376698812334	0.850521190776634	0.226331960252769	0.627376123087303	0.0117616666666669	0.982398683576954
ZBTB38	0.0692243857426653	0.850521190776634	0.0168430762511032	0.981160061237418	0.0819908333333336	0.761468932274728
ADAM3A	0.0692118774360338	0.850521190776634	0.0584080212107567	0.928725686060612	0.000905666666666686	0.997755470689145
PVRL4	0.0692034784268186	0.850521190776634	0.240844819112782	0.596773408433565	-0.0738436666666667	0.786973845403943
FLJ33534	0.0691630654349327	0.850521190776634	0.112726080294979	0.847051776433405	-0.146190666666666	0.465279557781548
TUBA1B	0.0690965522037139	0.85078520099093	-0.121716544076887	0.83097116342043	0.0929266666666675	0.664617185770066
MB21D1	0.0690611700856507	0.850899868605456	-0.417270733031772	0.234235464483992	0.00450666666666703	0.990496921312903
CDK2AP1	0.0689976100507441	0.850945686813869	0.242142505585294	0.594588225026249	-0.100521666666666	0.657217907037306
MAP7D3	0.0688784513981873	0.851219585640576	-0.182825055460873	0.718289071039953	-0.0646056666666662	0.831668247201974
ARSI	0.0688702551097597	0.851219585640576	-0.141684253401636	0.795111017039838	-0.0676493333333326	0.78960166957155
MFSD5	0.0688378506607454	0.851291639112072	0.0977185389599975	0.8692242996093	0.0199673333333338	0.950641499123018
MAEA	0.0688026914424675	0.851311061977258	-0.265742469894581	0.539878668275766	-0.176345333333332	0.355387672697857
NIF3L1	0.0687775926958032	0.851311061977258	0.0201041677361020	0.97701039355297	-0.0642043333333323	0.81562142081933
IQCF2	0.0687692607963321	0.851311061977258	0.00429518156408681	0.99405344216434	0.191403666666667	0.466018597177945
PIF1	0.0687615416241214	0.851311061977258	0.104799708252012	0.86067831050617	-0.152717333333332	0.484148891768163
ARMCX4	0.0687519503808877	0.851311061977258	-0.249610821766486	0.575370998761354	-0.133070999999999	0.625182495192502
CYP4F2	0.0687174397321248	0.851311061977258	0.00868932453452027	0.98946753270812	-0.160927333333333	0.611387735196207
CDC20	0.0687041464433568	0.851311061977258	0.0500158172841983	0.941458038227918	0.0600466666666673	0.784176378770158
OR51M1	0.0686660783464823	0.851311061977258	-0.187929244294418	0.70798562655309	-0.120725666666667	0.567376816263482
PLA2G2E	0.0685292125142963	0.851779482874226	0.132738932529123	0.809588765729788	-0.0859153333333322	0.692145913868422
CALCA	0.0685135799471782	0.851779482874226	0.155568023235367	0.769926535744838	-0.0490539999999996	0.846917872248932
TEK	0.0684985668334518	0.851779482874226	-0.303388551973392	0.461534675086054	1.566011	0.00347244136732606
CLTA	0.0684947636708108	0.851779482874226	-0.0271431853404156	0.970207612777188	0.0422510000000016	0.864974134126057
DUSP7	0.0684838485146675	0.851779482874226	0.285507189472761	0.497372582234299	-0.253588000000000	0.297916341729699
DHRS2	0.0684744111638154	0.851779482874226	0.102101706139126	0.864337135688791	-0.186699666666666	0.472124270069641
CNBD1	0.0684663337756631	0.851779482874226	-0.0814082034189951	0.89615326639288	0.217220333333334	0.480396469408299
GGN	0.068445408587423	0.851779482874226	0.192566706437645	0.700273993931252	-0.0583539999999999	0.798133257429747
FBXL12	0.0684328906254348	0.851792188867619	0.137526216715231	0.802039543563725	0.161755666666667	0.414405354802607
NIPAL1	0.06840467306736	0.85188091446614	-0.0544521684511652	0.9344692810234	-0.101894666666666	0.629710931024195
SERPINB10	0.0683877787034263	0.85188091446614	-0.0442656248902886	0.948385499132766	-0.121288333333333	0.605491766505367
SLC23A3	0.0683500725780499	0.851917228713171	0.107340047656317	0.857128584932911	-0.123930333333332	0.604966458785844
PKIG	0.0683007800683917	0.85207114525411	-0.151539053781933	0.777101208632856	0.00279766666666766	0.993756838831997
PPHLN1	0.0682254316905716	0.852107426537877	-0.0621504147631167	0.92301259447339	-0.133669333333334	0.523041310312964
ROPN1B	0.0681198486934974	0.852478070101735	0.288735434996919	0.489813374112312	0.01535	0.955460918122607
TSPAN31	0.068078211279671	0.852478070101735	-0.0656358817763602	0.917807146745076	0.228245333333335	0.309421300309741
IRF3	0.0680687805861452	0.852478070101735	-0.134440105422880	0.808009477165608	-0.252149	0.199991163409780
EXD2	0.0680653613432318	0.852478070101735	0.0101247171159556	0.988384322540392	0.155532666666668	0.438758057008766
PYGB	0.068041733154682	0.852478070101735	-0.280080356743463	0.508074118826263	0.075683000000001	0.763362609740299
TAS2R39	0.068040736543299	0.852478070101735	-0.209559408924738	0.664094875644384	0.0633293333333338	0.812232870389155
IL11RA	0.0679560163607178	0.852766389428583	0.0697125312849668	0.911167570018484	0.033875666666667	0.911796922065088
THBS3	0.0679280544704841	0.852858342868616	-0.0573461334791165	0.930490408235626	0.425113333333334	0.0411983064548969
KAT2A	0.0678684170161654	0.852997444871091	0.250498061463382	0.573763059271745	-0.0284969999999983	0.910190419163007
ATXN7L3B	0.0678640208280391	0.852997444871091	-0.0257633146312374	0.971568058192994	0.0565866666666669	0.829424296489523
ITIH4	0.0677751547708563	0.853426370155038	0.231135760221423	0.615929441239857	0.0143643333333334	0.9681321732097
TBC1D10B	0.0676840032857132	0.853781800458078	0.128731945896242	0.815407702581246	0.094511333333335	0.700075065613492
SYT9	0.0675759109356836	0.854287307111887	0.323831812371931	0.414201624246883	-0.103656666666665	0.657726934705567
ZNF747	0.0675272101042647	0.854395787639396	0.0882518251922241	0.885460067541563	-0.0867706666666662	0.751653256027624
LINC00471	0.0673765672558669	0.855148804198252	-0.134255817027180	0.808009477165608	-0.0957243333333326	0.639837290428782
LAPTM4B	0.0673212125224395	0.855365803796615	0.0334601519969428	0.960784250392482	-0.246873333333332	0.190569948330175
CH25H	0.067305736400891	0.855397144588895	0.0140865439815609	0.983444353656142	-0.200861333333333	0.370079759630929
KANK4	0.0672539468758315	0.855591582754247	-0.126943649167519	0.820587859614657	-0.0814999999999997	0.781866487624294
SSTR4	0.0672300819183383	0.85567596248652	0.0827390336415417	0.893753725133293	0.0295650000000004	0.950884054684253
NEK1	0.0671629088614548	0.856034205570884	-0.231359255290697	0.615205010562777	0.148640666666667	0.540255856426522
LMOD3	0.0670337822851022	0.856451467908748	0.172025774283106	0.738897289340835	0.117563333333334	0.610151958903093
MEI1	0.0669727207293064	0.856623973429035	-0.130984379528476	0.812271254601992	-0.0927316666666663	0.74424924420945
EFCAB2	0.0669647117020486	0.856623973429035	0.412012438290948	0.243188677508906	0.208570666666667	0.324937679238988
TMEM26	0.0669154188471364	0.85673405088055	0.135214952424136	0.807470535721699	-0.124559333333333	0.606512600974356
CDC14B	0.0668865241525362	0.856834714522648	-0.066994223896137	0.914998937781868	0.125681000000001	0.527683811694749
TTTY13	0.066788485922382	0.857248648399729	-0.00707668860361067	0.991086607331007	0.0570816666666674	0.858641906535933
ZNF71	0.066771652380078	0.857248648399729	0.0550586503696867	0.93406507597279	0.270535000000001	0.169027316227871
MYH4	0.066684653071943	0.857475476004394	0.0995498853186642	0.867603998426271	0.101534666666667	0.756712997772435
ZNF180	0.0666823558734156	0.857475476004394	-0.0333033134271776	0.960784250392482	0.217868333333334	0.257194631784673
SYK	0.0666210517649862	0.857663359450648	-0.297443074800921	0.473800338702402	-0.0622339999999997	0.861945621270498
OXSR1	0.0665393494637168	0.857980151284636	-0.253547726899081	0.567705016149998	-0.0260143333333334	0.919127710424584
SPDYE2	0.0664269919583955	0.858490688830318	-0.165850511917635	0.751869806279813	-0.140219888888888	0.499554381454891
MEOX2	0.0664149516919959	0.85850022909745	-0.259851562069242	0.555832878516908	-0.0909133333333333	0.692483917178266
RASGEF1B	0.066387674553746	0.858606081001013	-0.267306354515131	0.536380012714446	-0.0628089999999996	0.838927397112197
ZNF497	0.0663712311093227	0.858643450984489	0.179526316316570	0.724770967840177	-0.151939333333333	0.604266415892742
MCTP2	0.0662883405049348	0.85908144516222	0.186159243349635	0.712609598901976	-0.0146383333333329	0.971769349205369

FLJ38576	0.066203987475032	0.859102165420192	0.00853877762298842	0.989612051419321	-0.0722329999999993	0.819580848096427
FCRL2	0.066200024400748	0.859102165420192	0.165244185474347	0.752586940524596	0.0650013333333335	0.790626524535316
C10orf99	0.0661967540500527	0.859102165420192	0.182266281130855	0.719419078472232	-0.0655663333333327	0.856854911614454
RBL1	0.066184106113026	0.859102165420192	-0.0738247042076132	0.905506084101768	0.160452333333335	0.482760288609588
SPRY2	0.066178094500181	0.859102165420192	0.143293816271519	0.79287526984225	0.0109873333333343	0.974484855799046
CTBP1	0.0661644633490666	0.859102165420192	-0.221875384248668	0.636778528562165	-0.0369899999999988	0.880429228421837
SIPA1L1	0.06615204786296	0.859102165420192	-0.383543538857745	0.293474457479736	0.484213333333334	0.0477045298744847
PRR15L	0.0661496530634118	0.859102165420192	-0.0300499079694865	0.966396286514145	0.0521586666666672	0.869505365719039
CDC25B	0.0661069740432447	0.859102165420192	-0.168257961309564	0.747723416813184	0.209366333333335	0.300124665604706
MKS1	0.0661029723987081	0.859102165420192	0.121574303467866	0.831318338128973	-0.0202643333333324	0.95197590164177
WDR89	0.0660863676393669	0.859102165420192	-0.188691700920843	0.707361603786947	-0.208558999999999	0.338402409003611
RPL6	0.0660807666211676	0.859102165420192	0.150566251904301	0.779021144254203	-0.0205783333333322	0.942092082409632
SERPINB7	0.0660698662556838	0.859102165420192	-0.170747316211352	0.741159221263917	0.0257226666666668	0.924674647382585
RARB	0.0660560458926577	0.859102165420192	0.0871484446412296	0.887055939766881	0.0163143333333340	0.962275697500938
MEN1	0.0660240076670421	0.85917408662755	0.0740333865558172	0.905506084101768	0.00345066666666679	0.993346316662499
CDC25A	0.065980235852195	0.85928612517923	0.124099429051846	0.825526959189789	-0.0288516666666651	0.922799610581502
DERL3	0.0659777154589246	0.85928612517923	0.123741008539372	0.826475556843795	0.132812000000001	0.527465067495661
YIPF3	0.0659641557714715	0.85928612517923	0.047409985769216	0.943981149721771	0.118248	0.570805767288581
ASCL2	0.0659103392058153	0.859559460344693	0.105018702640952	0.8606536389692	-0.173699666666667	0.540614958546206
MIRLET7D	0.0658588491340529	0.859685138369617	0.177803174698717	0.728031191477223	0.0660313333333339	0.837071318894421
EIF1AY	0.0657837219781078	0.859798861316744	0.115918594880522	0.842733236705133	-0.0785976666666666	0.745703398753986
ANKRD19P	0.0657725210257785	0.859798861316744	0.201420756734408	0.680054545720343	-0.0370436666666662	0.88629962370345
GDA	0.0657681563802352	0.859798861316744	-0.262091146919102	0.549752906834892	1.22581466666667	0.00311122808725912
CDK17	0.065711334230407	0.85998783556428	-0.144610572118870	0.790044831580319	0.249399333333333	0.193877143823627
EIF3F	0.0657035127775203	0.85998783556428	0.049531567401212	0.94156962266198	-0.0371486666666662	0.892573269731487
TRIOBP	0.0656950898238855	0.85998783556428	0.145957649210618	0.787402233709509	0.140738333333334	0.503308350574223
BUB1B	0.0656811292757417	0.860008880735683	-0.00527994250926747	0.99251028338798	0.0540090000000012	0.853077816975915
TRPC1	0.0656000916402162	0.860193284859981	-0.17010251773305	0.742880989493217	0.406809333333334	0.0566164416372554
SRF	0.0655798286543933	0.860193284859981	-0.235640373458692	0.6067637636079	-0.201282	0.359771290730514
TACR1	0.0655721061058316	0.860193284859981	0.123975405140486	0.826013091664537	-0.0418239999999998	0.908590774398726
TBR1	0.0655658963887149	0.860193284859981	-0.0640049358048369	0.92003791975205	-0.0429433333333330	0.887363245747406
AGTR2	0.0655572651400442	0.860193284859981	0.294013306499871	0.480297745849088	-0.00162199999999981	0.99597023247862
PES1	0.065545207616413	0.860202948621508	-0.125560691075491	0.82367707928204	-0.458205333333334	0.0364080466199869
KIAA1804	0.0654704305131	0.860390313640872	-0.144429369805274	0.790414495161718	-0.186724333333332	0.344576368488222
KIAA2022	0.0654628785894965	0.860390313640872	-0.229810846653074	0.619381301776937	0.0655750000000003	0.835747501406204
HM13	0.0654416124403877	0.860426900425764	-0.233375829000115	0.610679292739473	-0.195514999999999	0.337168522063646
LGI3	0.065418800158151	0.860469173234058	0.426711523081681	0.213221804021091	-0.0255506666666664	0.929066230830908
TBL1Y	0.0651988688735492	0.861657323031288	-0.132735757567689	0.809588765729788	0.0881390000000007	0.705010402761235
ADRBK2	0.0651822301014141	0.861695846801322	-0.366351149463402	0.328765884278342	0.355086666666667	0.25616557267041
IDI2	0.0651129407839772	0.86198439739943	-0.0252350651314178	0.972358966514305	-0.0246579999999995	0.936688436284818
RASL12	0.0651023718373767	0.86198439739943	-0.115960054652730	0.842733236705133	-0.0948619999999992	0.715392853963174
CSNK1A1	0.06508382734676	0.86198439739943	-0.00678394171354913	0.99129507664977	-0.00705399999999989	0.9826267252384
SPPL3	0.0650168398548407	0.862256307730716	0.114318471379228	0.844272085764738	0.037082666666668	0.883189085233712
DNAJB13	0.0649946783367229	0.862256307730716	-0.139080339356759	0.79864602540281	-0.109082166666667	0.606164611038961
ZNF568	0.0649839502125453	0.862256307730716	-0.369921914030593	0.322377391230088	0.0913716666666673	0.671492528470281
GPR161	0.0649775204260307	0.862256307730716	0.0804541831395888	0.896952038996626	0.207751000000001	0.293767671859086
HDAC6	0.0648748691636827	0.862770927274484	-0.098666677130758	0.868080433714765	-0.0799559999999992	0.709458407711009
NIN	0.0648053809503672	0.862975708121381	-0.274728974361113	0.520095539214775	0.209570666666667	0.271568162967415
ITGB8	0.064800257537118	0.862975708121381	-0.271353463863346	0.527984832801825	1.24964366666667	0.00295083342264811
NUDT15	0.0647385609363535	0.863238670732167	-0.123173924060293	0.827789978865629	0.031367333333334	0.907615510059205
EIF3C	0.0647007546483287	0.863382826774928	0.0822494616030631	0.894635641425896	-0.125168333333332	0.549323798856043
PBK	0.0646935630429627	0.863382826774928	0.0922047338833138	0.879153312143409	0.112196000000001	0.607898776363481
CDKN2C	0.0646808548488694	0.863396508329455	0.084702015422051	0.891309254245872	-0.0395349999999985	0.892414900592065
ONECUT2	0.0646322654867193	0.863503557974191	0.325058538935442	0.41045730743103	0.0386383333333342	0.88177867737749
CIZ1	0.0645960491216021	0.863599033220385	-0.00763688848912676	0.990405674923721	0.0646023333333337	0.77394434422275
GABRD	0.0645721200169756	0.863683478498749	-0.0417411067284416	0.951989245458556	-0.0015263333333328	0.996894681472573
GLI4	0.0645179197374404	0.863908950640213	0.444218492683333	0.183294194447278	-0.157294333333334	0.5530742296054
ZNF669	0.064474368740402	0.863908950640213	-0.10555545824012	0.85956218633532	0.144654666666667	0.493152852604312
FAM166A	0.064457525704808	0.863908950640213	0.314933288560516	0.436301158221661	-0.000244666666666631	0.99883107916854
TJAP1	0.0644544877551715	0.863908950640213	-0.0441123831948413	0.948520928173175	0.0231960000000005	0.932944513468781
SLC37A1	0.0644520011286187	0.863908950640213	-0.0144827723691669	0.983444353656142	0.0175530000000009	0.964995783450258
RCOR2	0.0644497779174747	0.863908950640213	0.0851527272227983	0.890458025676316	-0.0726316666666656	0.750936504909256
REEP2	0.0644033487300714	0.864083118270872	0.0734999728385883	0.905838142287595	-0.0707769999999986	0.771153294405787
PRDM7	0.064303462798851	0.864538261319216	0.227422687420517	0.625658029930638	0.0517173333333338	0.838813113208837
CKS1B	0.0642995383842586	0.864538261319216	-0.0434428401008343	0.949521794694691	0.031140333333334	0.911129648924728
ATXN3L	0.0642565977947391	0.864549068411763	-0.00794996418324488	0.990405674923721	0.123876666666667	0.55490958019059
SLC39A8	0.064238613094584	0.864549068411763	-0.087107303179126	0.887055939766881	0.603495333333334	0.0338015945178966
TP63	0.0642333405812172	0.864549068411763	0.107220086063591	0.857401685651907	-0.0680943333333323	0.809911399406217
KCNN2	0.064224038610244	0.864549068411763	0.240791980564196	0.596773408433565	-0.0313719999999994	0.901737739185354
NPY	0.0641468199433063	0.864810232966738	0.236668831337008	0.604560022191981	-0.0216356666666663	0.948386471366378
TNFRSF21	0.0640764468351849	0.864943849420114	0.0468232308139276	0.944274559688373	-0.208681999999999	0.344663423870532
CENPB	0.0640728095801969	0.864943849420114	0.0693604680068769	0.91172160664395	-0.104339666666666	0.611103116031235
CASKIN2	0.0640665383717125	0.864943849420114	0.133308802603193	0.808992949393043	-0.127528666666667	0.615722818909839
MBD3L1	0.0639895948667995	0.865163020040338	-0.166688407164506	0.750443995356965	-0.0381313333333327	0.898863720449539
ZNF335	0.0639667524483736	0.865240531520776	0.100350527937932	0.866573451223644	-0.0556953333333328	0.808239992878146
BRD7P3	0.0639338240725364	0.86529275954933	0.115493258320381	0.843609735991731	0.0355355000000011	0.898411505899836
ATP11C	0.0639195918955727	0.86529275954933	-0.472553315201527	0.145443641689931	0.145393000000001	0.51632194713066
CUTC	0.0638820058627444	0.865413194731614	-0.0728080448179137	0.907092546541332	-0.0312703333333327	0.91402300740264
CHRDL1	0.0638173849505366	0.865534744818376	-0.144143000316571	0.791303782903891	-0.335192333333333	0.101751290905858
PROS1	0.0638146468005975	0.865534744818376	-0.540997644629697	0.0689812645080214	0.323035166666667	0.172764809646572

HTR2A	0.063773349293158	0.865580858136988	-0.0532746476975984	0.936629055455007	-0.065685333333333	0.816054928507559
MCL1	0.0637616393370677	0.865580858136988	-0.405709927558199	0.25473834018135	-0.0919233333333332	0.65907849208289
ZNF254	0.0637552736273967	0.865580858136988	-0.0765335892655652	0.902600864962302	0.152404000000001	0.456074677719062
HOMER2	0.0637307807925292	0.865580858136988	-0.229959406298299	0.619254569407113	0.258520000000001	0.253356882479072
WDR64	0.063657195723812	0.865597207467778	-0.0421404920598546	0.951916033831249	0.0964073333333336	0.652989236147115
TCF25	0.063640890875555	0.865633518953104	0.0874445359142204	0.886459652119148	-0.0700046666666662	0.754351658576291
RAD51B	0.0635965148866622	0.865780196243309	-0.080038993225876	0.897577172229156	0.0260680000000006	0.926246672706072
PCDH20	0.0633962418724234	0.866510093713391	-0.136344095432683	0.804864448119512	-0.487010666666666	0.134443467906163
CSTF2T	0.063375976633646	0.866571303066037	-0.094843295870445	0.87373649118985	-0.0311216666666662	0.919127710424584
NOM1	0.0633417285982645	0.866587853925712	-0.355877643069152	0.346552539524285	-0.161976000000000	0.438307351749832
FLJ20518	0.0633241950189226	0.866599295770736	-0.168878128394448	0.74582180262941	0.139800000000000	0.611770847276966
MAST2	0.0633166022307354	0.866599295770736	-0.145659731242808	0.787804113309913	-0.265797999999998	0.319387382047648
NCS1	0.063188647223143	0.867153182468404	0.248169770865447	0.577859441890662	-0.356349999999999	0.106019468292232
DMBT1	0.0630881762744891	0.86758638529174	-0.153003839689715	0.774522298110532	-0.0785306666666663	0.753865265860856
CETN1	0.0630651338150401	0.86766503473614	0.230296177998956	0.618707227156037	-0.0898133333333333	0.671766969198954
POLG2	0.0629973814430881	0.867783548500798	0.115196224298475	0.843900131281755	0.142506000000001	0.590147690656472
SPATA18	0.0629361068942078	0.868012461033443	0.0466980304086901	0.9445210535875	0.361474333333334	0.302316846105060
MTERFD2	0.062913434259539	0.868088763054993	0.176806731760952	0.729794187782856	-0.0362476666666664	0.888217263833458
ORC1	0.0628747510109521	0.868197487181392	-0.164508251180775	0.754189116283047	-0.165476	0.502814370970592
APBB1	0.0628645174971656	0.868197487181392	-0.139670593513605	0.797957231657366	0.216798333333334	0.479443005264132
MS4A15	0.0627954503902621	0.868497498556366	-0.320967503089638	0.421618886526906	-0.0622869999999993	0.812603088881849
NELL2	0.06278519018676	0.868497498556366	-0.307251421117222	0.453005595044643	-0.280676333333333	0.274121181062751
ZNF213	0.062727728860003	0.868634296412632	0.0471133641734146	0.944081286115546	-0.0419276666666668	0.870662337224757
STAG3L4	0.062687295275278	0.868634296412632	0.0976109854749336	0.86949016346201	0.0532806666666664	0.85325937945754
UBAC2	0.0626588369818932	0.868634296412632	-0.16407410290197	0.754243267462143	0.000293666666666825	0.99883107916854
ZNF317	0.0626474435112875	0.868634296412632	0.0130810008527235	0.984547546298896	-0.0735903333333334	0.760010763598572
CST4	0.0626086722061601	0.868645326050901	0.103744222405128	0.861866357553435	-0.294879999999999	0.361971299676959
SLC25A6	0.0625990887542945	0.868645326050901	0.0573576822859219	0.930490408235626	-0.278680666666665	0.154631928204474
TSEN2	0.0625831102167766	0.868645326050901	-0.349781089210987	0.358977715627648	0.0403343333333335	0.884789546675685
HIST1H2AB	0.0625514077567884	0.86872526522374	0.0685525053674386	0.912149839732916	-0.0644928333333327	0.824044031896077
MYH15	0.0625486289572193	0.86872526522374	0.231821822676728	0.613578980603213	-0.231164666666667	0.235054998483403
OR5P3	0.0624964625398807	0.868921358564838	-0.0907363060150859	0.881323965029954	-0.147238333333333	0.522830837265953
ANKZF1	0.0624833425369291	0.868936825659058	0.455461488682672	0.168217575057610	-0.121553333333333	0.592716584553539
DBNDD1	0.0624240420997769	0.869243287218231	0.229600212841471	0.620127559201892	-0.0494696666666667	0.885033162791957
UBASH3A	0.0623224291577257	0.869550559592978	0.0858050493059631	0.888748594817136	-0.240689666666667	0.306650933067113
PITX1	0.0622866292273050	0.869643000327144	0.328002267771883	0.407465492446943	0.0383376666666669	0.879843042066371
TSPYL5	0.0622291990954015	0.869738820210182	0.0305249354460759	0.965676710289184	0.187687333333333	0.338139826463602
FAM179B	0.0622044647668876	0.869827979338551	0.0162170421657271	0.982057247916055	0.279339333333334	0.139122737306306
SEC14L5	0.0621468015323621	0.870057780278952	0.158125336974411	0.767373487136803	-0.0614449999999989	0.850157189407213
ECH1	0.0620784926666283	0.870154532034732	0.136625383258351	0.80422630975164	-0.0810166666666659	0.709568037614009
TAS1R1	0.0620542949351582	0.870154532034732	-0.0341581996136596	0.95969480339665	0.0964966666666667	0.719304894613107
C1orf191	0.0620530030376744	0.870154532034732	-0.207878349825804	0.667695745454683	-0.0322863333333333	0.906093399285191
EPRS	0.0619883765604633	0.870376452297217	-0.0429636657836578	0.95022891501176	-0.253582666666665	0.322437401396896
MAP7D2	0.06198006635632	0.870376452297217	-0.108310922632531	0.854569005311953	-0.0393433333333328	0.918619049556974
SRP54	0.0618299775105451	0.870843244782754	0.0350273609956297	0.959140268168928	-0.0255463333333327	0.93535961048518
PPFIA1	0.0618215551054773	0.870843244782754	-0.167747660506959	0.748242818061289	-0.0982523333333329	0.689375614466094
ARFGEF1	0.0618176925735491	0.870843244782754	-0.229721698739600	0.619603028959455	0.0562113333333337	0.827433678445379
CCDC6	0.0618108245061953	0.870843244782754	-0.468137329186514	0.151936428121882	0.0106813333333342	0.974895360062947
OR1D2	0.0617932838951397	0.870887163020617	0.0713363893560436	0.909119908757507	-0.214290666666667	0.465279557781548
GPX5	0.0616806221257873	0.871130166673945	0.0359546784250679	0.95830581556695	0.0479276666666669	0.840247972976129
DIDO1	0.0616725026418098	0.871130166673945	-0.108525664232090	0.85395367181085	-0.0328749999999993	0.906346904081866
UBFD1	0.0616702074724394	0.871130166673945	-0.000406674218957140	0.999513431881539	0.00358400000000094	0.991175862027014
NOP2	0.0616528115119884	0.871140076379319	-0.112984738814561	0.84660273030137	-0.305858666666666	0.113574858451228
GSTM4	0.0616162317981658	0.871140076379319	-0.16506957225136	0.7529423475214	-0.100903666666666	0.639002829892966
HS3ST6	0.0616158996714171	0.871140076379319	0.0261064526648632	0.970632644458507	-0.185544333333333	0.532604205131631
OSR1	0.0615976628656637	0.871182677493147	0.296921265354295	0.475575866007561	-0.252302666666666	0.476307520847818
NUDT16P1	0.0615641803643045	0.871182677493147	-0.251918588210443	0.570584539772622	-0.0982453333333328	0.746548092073175
RHOBTB1	0.0615556815603831	0.871182677493147	0.441952903688724	0.186115306176759	0.204102000000001	0.626635388688185
LINC00525	0.0614923551454565	0.871302289530923	-0.281684129289634	0.505839445440437	-0.165978999999999	0.508536973978403
USP15	0.06148394499154	0.871302289530923	0.281168707329194	0.506886267079183	-0.0717236666666659	0.739188468398639
C5orf56	0.0614827294692453	0.871302289530923	-0.23639268687005	0.60511225037033	0.0144160000000007	0.964104511796897
KERA	0.0614529781307912	0.871302289530923	-0.126244359922872	0.822164115743016	-0.179640666666666	0.358792123436256
MYH2	0.0614191563199344	0.87138218436233	-0.145743510926022	0.787804113309913	0.0886230000000006	0.701302778587828
ARMC7	0.0613420770264818	0.871601137789368	-0.112873637145348	0.846769743490891	-0.0381463333333324	0.888476605628154
UBE2L6	0.0612768507812793	0.87174572365585	-0.249802988950105	0.57536723546666	-0.0817253333333334	0.70610473471702
PAX8	0.0612213430271154	0.87195966722456	-0.073585358112642	0.905838142287595	-0.153387666666667	0.687794959259872
NOTCH4	0.0611360592326105	0.872298020238873	0.0204371412179177	0.976412247012224	-0.155642666666667	0.568393891086758
RFFL	0.061092318249021	0.872368746416083	-0.00553172923729601	0.99251028338798	0.0386263333333342	0.880427463662501
GRIA4	0.0610825985686707	0.872368746416083	-0.308034372839875	0.451488724245047	-0.311854999999999	0.156312939631849
DUS2L	0.0610062755853743	0.872538725230214	0.257790375326547	0.562492892055072	0.194790333333334	0.346165986086181
POLD1	0.0609827068854354	0.872598341911146	-0.0327381783032494	0.961351562552925	-0.0164149999999981	0.95450472002063
GPR37L1	0.060928429074376	0.872851713364954	0.226173657772934	0.627915430852572	-0.0973093333333339	0.748388402768556
CPS1	0.0609106519199173	0.872851713364954	-0.182261179814507	0.719419078472232	-0.281754	0.206187117033303
ITGB7	0.0608609670359122	0.872917574203327	0.0427227645130627	0.95079489434051	-0.0941463333333325	0.682815224992534
CBR3	0.0608522277335125	0.872917574203327	-0.169302071399291	0.744676064883446	0.0299906666666662	0.93278021276477
TULP4	0.0608173200527547	0.872917574203327	0.128722208049495	0.815407702581246	-0.106196333333333	0.682815224992534
FOXM1	0.0608157817423649	0.872917574203327	0.0217542871829364	0.97536722461972	0.0430970000000001	0.8754328609757
PACS2	0.0607908691753878	0.873007609802105	-0.0517335603543341	0.938273022292017	-0.0445741666666660	0.870662337224757
CDKAL1	0.0607788030167578	0.873017144128203	-0.428372260876116	0.210578505651933	-0.0639096666666654	0.822390330537826
P2RY12	0.060750315767178	0.873098029678284	-0.208748345127398	0.666024041170443	0.0482163333333336	0.863646775961842

TRIM54	0.0607284569215908	0.873098029678284	0.176418595753560	0.730175791231993	-0.190974333333332	0.349242158282191
PCSK7	0.0607237165783234	0.873098029678284	-0.390438089908198	0.282383744168943	-0.310008333333333	0.120319574336004
SOX14	0.0606362507067327	0.873311542871549	0.215927218191764	0.648513803694991	-0.148099333333333	0.613258123073115
EAF1	0.0605505976137754	0.873651995864866	-0.532088613669548	0.077325951207548	-0.214709999999999	0.306059380422769
FGD5	0.060528507633434	0.873651995864866	-0.302431425872488	0.462229407853213	0.0201073333333335	0.94678260229888
CEBPE	0.0605262567410067	0.873651995864866	0.192076810147887	0.700820605802046	-0.211589666666666	0.324937679238988
THOC1	0.0604796026531281	0.873651995864866	-0.0826480250264517	0.893753725133293	0.118339666666668	0.5530742296054
POLR2D	0.0604770382380625	0.873651995864866	-0.0169782355639339	0.98105020869911	-0.265000666666667	0.343143699851351
ZNF419	0.0604709315774392	0.873651995864866	0.0822120188791613	0.894768534362947	0.0960260000000005	0.68171195530621
KLF7	0.0604602688255749	0.873651995864866	-0.420640746341149	0.225825225366294	0.293929000000000	0.27592359940594
CAPN1	0.0603411511681616	0.874173230025722	0.144354530042953	0.790546040025025	-0.289725333333332	0.229420039916763
MLNR	0.0602659661842613	0.874338337433313	0.158674347745778	0.765791046741822	-0.0199316666666663	0.959218131095776
BPESC1	0.0602655987958877	0.874338337433313	0.191704048549593	0.701544026774214	0.112317666666667	0.641044966033937
CD55	0.0602545592719049	0.874338337433313	0.289455840557042	0.488870852256136	0.330455333333334	0.150274389990121
CA7	0.0602263083615503	0.874341539027338	0.375731160614024	0.310953589377137	-0.247478333333333	0.271872170281604
AQR	0.0602193562323185	0.874341539027338	-0.211985066822267	0.658364968650984	-0.110218999999999	0.639837290428782
MAML2	0.0601560850711479	0.87451971763259	-0.499438436272161	0.113777333040600	0.652687666666667	0.0140473413958925
SFRP1	0.0601479721706203	0.87451971763259	-0.221852029486344	0.636778528562165	-0.0869953333333318	0.705698790800484
MCM3AP	0.0601364232228595	0.87451971763259	-0.101067104542114	0.866005093242444	-0.0533839999999993	0.847964572928708
IL13	0.060130032981926	0.87451971763259	0.134925058274434	0.807867938002244	0.0202036666666675	0.955460918122607
PPP1R9A	0.0601130124546892	0.87451971763259	0.139533024968618	0.798145654866315	-0.316341666666666	0.114193085140962
ZNF626	0.0601065300238892	0.87451971763259	-0.168515118774708	0.746758286599055	0.088917833333335	0.80578153770169
NUP153	0.0600822414509293	0.874529858042039	-0.0376572211340037	0.955365651737247	0.0574036666666678	0.826305377184894
FAM200B	0.0599947012406214	0.8747887913695	-0.0280875660210357	0.968668367660302	0.121340666666667	0.582111650674021
NR2C2AP	0.059991338451965	0.8747887913695	0.286820826394208	0.494604746861742	-0.0783806666666653	0.772520347452753
SCN8A	0.0599780332901081	0.8747887913695	-0.220151051974809	0.640598540561765	-0.0172566666666658	0.950884054684253
LMAN1L	0.0599333476846691	0.8747887913695	0.147745845160135	0.784475200791671	-0.0118469999999998	0.97184501043952
TESK1	0.0598991951353619	0.8747887913695	0.302647658781730	0.462172681353884	0.0207110000000006	0.949314722489396
CXorf23	0.0598964492060715	0.8747887913695	-0.0492782630098887	0.941842562680095	0.248363666666669	0.322437401396896
PLEKHG1	0.0598948307140119	0.8747887913695	-0.373504920691599	0.315811026719894	-0.208850000000000	0.367241810450146
MRPS7	0.0598559681510345	0.874834054771097	0.192079399932341	0.700820605802046	0.0539266666666669	0.811997756288597
FBLL1	0.0598290203805835	0.874912848135513	-0.00795501860447418	0.990405674923721	-0.0616323333333327	0.813694870801638
CYSLTR1	0.059809518473331	0.874912848135513	-0.321018479935809	0.421618886526906	-0.250957666666666	0.461494497197158
LY6G5C	0.0597649296182887	0.874912848135513	-0.40202435070876	0.260476951140907	-0.0568688333333329	0.850364148347928
OAS1	0.0597477919588241	0.874912848135513	0.0121924110855354	0.985695257551953	-0.296534333333333	0.172257177620526
INGX	0.0597451499104854	0.874912848135513	0.201869478943202	0.680054545720343	-0.208204333333332	0.281178505900635
TDRD1	0.0596920100041536	0.875068319090306	-0.141108643630810	0.796333933122002	-0.084084	0.74164843446719
FLJ37453	0.0596641317195776	0.875176680365726	-0.0337706350655425	0.960457447372477	-0.0770276666666663	0.74496131311871
ZHX2	0.0596264824313174	0.875280229493012	0.447274017860521	0.178249202877872	0.153328666666668	0.555088011807105
TBCE	0.0594874691577732	0.875549659040567	0.0604686485172715	0.925565513580637	0.0105720000000001	0.973791486752984
SPOPL	0.0594762128887022	0.875549659040567	0.0734688781957088	0.905838142287595	-0.277719999999999	0.337081276851993
ZNHIT6	0.0594518485911145	0.875549659040567	-0.575040450040723	0.0442317804409017	-0.003153	0.994216250363693
MAP6	0.0594303196108114	0.875549659040567	-0.0155178519467595	0.98256069835494	-0.0950526666666658	0.775858800113142
SLC38A5	0.0594154442669687	0.875549659040567	0.0695819964484321	0.911313423064389	-0.196319333333333	0.495283912463359
ZMYM1	0.0594125606616746	0.875549659040567	-0.0735063692002286	0.905838142287595	0.115731000000001	0.60378773986659
DYRK2	0.0594041938381419	0.875549659040567	0.242036082426064	0.59465236351643	0.368883666666667	0.0846385443172634
LINC00310	0.0593587279258995	0.875753840415294	-0.171956922323421	0.738962506786205	-0.00749833333333238	0.987908236336346
MARCH5	0.0593398840196836	0.875753840415294	0.139178341495108	0.79864602540281	0.135530333333334	0.49699337233765
PRKD1	0.0592454554819362	0.876036620645823	0.0429953003225332	0.950146638225747	-0.116695666666665	0.596617523400524
MT2A	0.0592419043137619	0.876036620645823	0.0607111395234922	0.925335786425382	0.0718266666666682	0.759818201272615
INPP5E	0.0591905408892292	0.876185376560496	0.0252228684338806	0.972358966414305	0.0717386666666672	0.752384210059315
AP1G2	0.0591855061833236	0.876185376560496	0.256290924894332	0.564430426783556	0.0120216666666681	0.9681321732097
CENPI	0.0591834539872367	0.876185376560496	-0.18828833666847	0.70742148547693	0.0981563333333342	0.672674698802727
DPPA4	0.0591505620265872	0.876185376560496	-0.302934194939288	0.462105017205825	0.0127410000000006	0.979590677970898
REPS1	0.0591443068006399	0.876185376560496	0.40752442380034	0.252272524515249	0.0632343333333349	0.787798433132403
C15orf37	0.0590616345543388	0.876569860609871	-0.347954869351115	0.361882864224644	-0.213038333333333	0.507469905101034
ZFYVE16	0.0590178280050164	0.876759796107375	0.217684484851415	0.644842900139291	0.263019333333333	0.163681343085520
FAM122B	0.0590101259607599	0.876759796107375	-0.225112866246535	0.63103711743427	0.008641	0.979105254223562
CUL5	0.0589624785472591	0.876991425086534	-0.263676471027887	0.545348103490451	0.0194893333333339	0.944218475609619
ADAM20	0.0588438933003854	0.877468415234613	-0.0624964509227303	0.92264749701958	0.0310543333333342	0.906453667774637
METTL21D	0.0588210164227968	0.877479566693157	0.211096565084204	0.660389781435267	-0.0218056666666655	0.942680762825505
LENG9	0.0587842470964567	0.877519253552292	0.254269782459571	0.567694846153007	-0.182109666666666	0.514889931507188
ZNF491	0.0587830238827443	0.877519253552292	-0.292555051795985	0.483448456260604	0.091211	0.738848535823302
CCT4	0.058745902347306	0.877619313294037	-0.170159154686398	0.74270446821332	-0.0118103333333325	0.968260590715545
RAI2	0.0586580724820961	0.877959835595507	-0.0473898491703727	0.943981149721771	0.0319320000000007	0.903864363916236
EXTL3	0.0586449756114696	0.877959835595507	0.117364554947345	0.839922145389918	-0.381185666666666	0.164204374758928
HHIPL1	0.058601799778591	0.878048636805355	-0.0310223400593186	0.965118873711308	-0.125287666666667	0.741438256509195
POLE3	0.0585889426976582	0.878048636805355	-0.0967281067229545	0.870857630743202	-0.0847559999999992	0.750961152956367
BTNL3	0.0585596627279136	0.878048636805355	0.213347479292906	0.654674857291449	0.127674666666667	0.666054079859859
REXO4	0.0585439707735199	0.878048636805355	0.104224903152746	0.86124543980304	-0.416811333333334	0.0769043559304575
NACA	0.0585424579485119	0.878048636805355	0.288070336094374	0.491376246980382	-0.0759633333333314	0.747689818135114
CALM1	0.0585400165837282	0.878048636805355	-0.0836289716083137	0.892833228561937	-0.0413543333333331	0.87279470391545
HOOK2	0.0584679289285275	0.878148931544193	0.152924644368241	0.774656266040138	0.0238790000000001	0.93466802430313
ZNF93	0.0584673541453851	0.878148931544193	-0.315099231365833	0.435958039071744	0.215156666666668	0.287956132210494
EBLN2	0.0584455753348442	0.878148931544193	0.323035158233609	0.415854621854132	-0.0613636666666658	0.811816884537132
MAD2L1	0.0584361862765462	0.878148931544193	0.0279346544168732	0.96912535812542	-0.0446099999999991	0.878427136726667
OPN3	0.058434793410341	0.878148931544193	-0.158182919167965	0.767370100148398	0.0563396666666674	0.856589789394798
LBX1	0.0584290445613655	0.878148931544193	0.159855082300267	0.763150272733332	-0.0783049999999998	0.779569065519332
GOLGA2	0.0582900548447475	0.878581001150834	-0.0744016823501968	0.904945422294164	-0.0353084999999997	0.890433216407555
GSS	0.0582824520836854	0.878581001150834	0.103074304018363	0.862488288780321	0.156261000000002	0.443378989641463

MSH2	0.0582779280553902	0.878581001150834	-0.241746883197999	0.595201219333254	0.151790333333334	0.4423818632121
CLGN	0.0582754016966377	0.878581001150834	-0.0797671863991965	0.897892885174022	0.283270000000001	0.187397740404124
MACC1	0.0582309605120724	0.878636642500432	-0.382650388707500	0.294988445038215	0.036912666666667	0.883775402587518
CA4	0.0582300802750973	0.878636642500432	0.373881034469758	0.3153356348575	-0.169147666666666	0.534910593395784
AMZ2	0.0582230668814512	0.878636642500432	0.180919673012204	0.721866914445283	0.0517960000000001	0.830147563067484
MKX	0.0582137596890511	0.878636642500432	0.0395203415528353	0.953905313456582	0.238059333333334	0.291073763966987
ZNF528	0.0581976068515613	0.878671609461293	-0.261327918497035	0.552187117311017	0.126112666666667	0.592255902760106
DACH2	0.0581755864392131	0.878700945473514	-0.143216886492466	0.79294618815764	-0.835690666666666	0.00629285391915396
C1orf43	0.0581559650757313	0.878700945473514	-0.0787035306806006	0.899281198071059	0.0306400000000009	0.910625003107163
RETN	0.058153697371007	0.878700945473514	0.411446913773183	0.243528138193926	-0.119530333333332	0.659124451108183
BTBD1	0.0581329791896486	0.878700945473514	-0.140217792892123	0.797441782082887	0.0660466666666684	0.774104133965233
DPH2	0.0581305913026507	0.878700945473514	-0.00030547225459920	0.999739851959534	-0.123068999999999	0.535098742175357
TRIM10	0.0581109498829071	0.878700945473514	0.145471446873763	0.787804113309913	-0.0261518888888884	0.919891803741744
CBFA2T2	0.058100355679484	0.878700945473514	-0.214391696655294	0.652991165086813	-0.0382816666666662	0.910920430866321
RPL13AP17	0.0580980249721642	0.878700945473514	0.190879278782455	0.702630917104531	0.0511380000000002	0.82667899725046
LAMP3	0.0580697460759159	0.87879155309967	-0.104366855890459	0.861121906453898	-0.0929056666666666	0.721454370213253
KRT23	0.0580623999823078	0.87879155309967	-0.00906114237711096	0.98909166696601	0.0615683333333343	0.850157189407213
CREBRF	0.0579596707362279	0.87928930473805	-0.187283562163473	0.709833349594307	0.310068666666668	0.113344928390947
INSIG2	0.0578124068853808	0.87968617247414	-0.0321357282796983	0.96274336578329	0.369732333333334	0.0619237323392494
BLOC1S3	0.0578121047818842	0.87968617247414	0.275421156060348	0.518987846340853	-0.0968623333333325	0.727701945074978
SLC28A3	0.0577868469747389	0.87968617247414	-0.0322567919788812	0.962384779621192	-0.0142706666666663	0.966813448118178
KCNAB1	0.057739218428389	0.879753908876766	-0.0738554824031793	0.905506084101768	0.0166080000000004	0.955460918122607
ZNF703	0.0576948166215226	0.879894380831512	0.382507939491277	0.295177945739812	-0.412455666666666	0.0456150738964454
BAIAP3	0.0576261348916184	0.879906756675797	0.468228088203303	0.151936428121882	-0.211580999999999	0.309421300309741
GAL3ST3	0.0576095420795611	0.879906756675797	0.100450779190237	0.866539251141083	-0.179117333333333	0.50260418651654
PRMT10	0.0575986984580589	0.879906756675797	-0.280498421403083	0.507460240190768	-0.0526976666666651	0.846463767685512
BTG2	0.0575579979078609	0.880094475003919	-0.228127046533206	0.623935503350068	0.105381333333333	0.675687379893745
MAP3K4	0.0574753735330408	0.88034718809921	-0.0503694166371438	0.940817551418531	0.0868303333333338	0.711082533709514
PXDNL	0.057463180573429	0.88034718809921	-0.359735529467119	0.341510139061518	-0.0566016666666658	0.826163946239814
RNF126P1	0.0574443486866067	0.88034718809921	-0.0270648301137026	0.970207612777188	-0.0435646666666661	0.883114887563545
MINK1	0.0574435929327385	0.88034718809921	0.23362195341313	0.610436189314218	0.0677860000000004	0.780904457213926
ENKUR	0.057340082329498	0.880820380948655	-0.178470551539508	0.726913675793783	-0.122331333333333	0.644004223235948
FKBP11	0.0573310104348526	0.880820380948655	0.0654732538887554	0.918111672450639	-0.104166666666666	0.616036866881905
CD96	0.057196886176884	0.881077430801614	-0.227030387725733	0.62581495163125	-0.0710299999999996	0.7744217439919
TCTN2	0.0571898027952515	0.881077430801614	0.0351440484143996	0.958931053746098	0.411865666666668	0.0660125264725009
CASP9	0.0571679252891117	0.881077430801614	0.0636847578763634	0.920748332386663	-0.439576666666665	0.0618388608777955
NCOR2	0.0571634970751805	0.881077430801614	0.191515451489604	0.701635983922402	0.110690833333334	0.584814173457916
NUBP2	0.0571575220703241	0.881077430801614	0.0759394046410344	0.903510413986113	-0.195729333333333	0.297916341729699
ZNF665	0.0570917033849885	0.881224472412945	-0.249416219138879	0.575393431980598	0.175164000000001	0.575076472049916
FBXL8	0.0570721387536847	0.88128057037603	0.205830422313116	0.672625944768829	-0.0417839999999982	0.86771131236451
TRABD2A	0.0570292065404955	0.88148198995779	-0.0942374649295405	0.874730296182874	-0.0161399999999998	0.961509682688243
PDZRN4	0.0570057156189988	0.881562497092206	-0.0932343764647192	0.876902144481654	0.0246216666666669	0.928203681921325
ZNF337	0.0569757002463093	0.88161798988607	0.0626448345494566	0.922350559812086	-0.00463333333333301	0.9879739922351
YPEL4	0.0569619649559303	0.881637822970807	0.176447401675925	0.730152680173573	-0.0549106666666664	0.813345796866388
CELF1	0.0568807566678625	0.882011646605674	0.123868655903555	0.826287181959559	-0.159724666666667	0.546433742140708
MYCBP2	0.0568661078466694	0.882037146668352	-0.132145593459163	0.810300414153187	0.0474806666666671	0.895093529187836
VPREB1	0.0568188578310854	0.88222445915465	0.411682078524897	0.243528138193926	-0.113546000000000	0.577592845567139
UBE2Z	0.0568148883309572	0.88222445915465	-0.188537890416536	0.707361603786947	0.0379699999999994	0.879265091272724
CDKN1C	0.0567455033825004	0.882562114917243	0.121062320903449	0.83265693609154	-0.127887666666666	0.58391025729515
PPFIA4	0.0566753157739761	0.882776443544957	0.244592386962415	0.587653287154633	-0.118219000000000	0.639837290428782
RPGRIP1L	0.0566328035376346	0.882776443544957	0.0439024989741186	0.94885721615997	-0.242106999999999	0.238780185222418
ARHGAP21	0.0566046165820798	0.882776443544957	-0.068461131957072	0.912149839732916	0.180299666666666	0.491578070092
PPP1R2	0.0565903923913908	0.882776443544957	0.0116534571299416	0.986654146365942	0.0917066666666668	0.669210565039564
LIMK2	0.0565871325279745	0.882776443544957	-0.337447056643979	0.386478628052631	0.0293366666666681	0.914418029309221
GFRA4	0.0565450327907537	0.882776443544957	0.150619657300188	0.779021144254203	-0.148552333333333	0.560413476606931
CLNS1A	0.0565362137729	0.882776443544957	-0.0286626925978545	0.96782691865619	0.0363733333333339	0.890073383340154
DET1	0.0565070440852153	0.882828095471031	-0.185715718335156	0.714043644840406	0.356095333333333	0.086885627133918
MIF	0.0564962565310288	0.882828095471031	0.223067751516061	0.635247978870818	0.140650000000001	0.621414476984274
CRIPAK	0.0564605971865826	0.882918579060384	-0.162769545852568	0.757417321756424	0.0217706666666674	0.95285450244329
KCNJ10	0.0563504708274746	0.883254006104839	-0.145696120798741	0.787804113309913	-0.218984000000000	0.618886975439257
KIF2C	0.0563433133539444	0.883254006104839	-0.172090382515607	0.738729794817225	0.00258966666666863	0.993824047902985
ASCL3	0.0562986413530658	0.883255416563194	0.055721957311268	0.933409139126562	0.145905333333334	0.613474321897644
POLR2G	0.0562797956091142	0.883255416563194	-0.100536252931497	0.866529683756364	-0.102315333333332	0.704370299831112
PEX11A	0.056238292854607	0.883316648445328	0.388459101758052	0.28486220160813	0.155824333333334	0.427283471646461
RHPN2	0.0562139786412419	0.883402132718734	0.36543751175638	0.329838587028393	0.0877190000000004	0.677570340702693
DMRTC2	0.0561799774253951	0.88354776393146	-0.0608383845947881	0.92506536457632	-0.160990999999999	0.633561720825521
HTR1F	0.0561401630593998	0.883623059795258	-0.0595391191972595	0.926789115926346	0.0207526666666672	0.949295980201336
PTGR1	0.0561362054048678	0.883623059795258	0.103466307025328	0.861943478503987	-0.252743000000000	0.217550258219234
GLTSCR1	0.0560759586640856	0.883828755352398	-0.199247627929796	0.684227048797313	-0.0379186666666669	0.895093529187836
VPS33B	0.0560527789535152	0.883828755352398	0.149138198564404	0.782612598081927	-0.341445333333333	0.0907526139873654
C4orf36	0.0560345533046763	0.883828755352398	-0.239509664495568	0.59909029358707	-0.0480743333333329	0.838927397112197
UMODL1	0.0559493950998417	0.884069029520075	-0.0724298794889719	0.907556175729082	-0.287317666666666	0.135004030972507
IDI2-AS1	0.055948328688039	0.884069029520075	0.485733604032016	0.131934855037098	-0.0892673333333332	0.81000174583549
ATF7IP	0.0558803397564814	0.88436068540162	-0.191874475305346	0.701479891661163	-0.0550999999999985	0.82667899725046
STAM	0.0558578357931054	0.88436068540162	-0.234282362563491	0.608813237170423	-0.222592999999999	0.320462604748547
C1orf220	0.0558557735369723	0.88436068540162	-0.242210339556760	0.594404778895365	-0.0115286666666663	0.969249447532345
CCDC158	0.0558384431158863	0.88436068540162	-0.25391501947635	0.567694846153007	-0.00512400000000029	0.990426818363066
DSG4	0.0558106169507547	0.88436068540162	-0.201639512358339	0.680054545720343	-0.0997306666666664	0.685618342866425
SOX1	0.0557716964416996	0.88436068540162	0.340519421209564	0.378350877728096	0.0273406666666673	0.938187566801362
RFWD2	0.0557452255877359	0.88436068540162	-0.254223774177341	0.567694846153007	0.157311333333334	0.49679816436215

NUDT21	0.0557291664882448	0.88436068540162	-0.103684488981045	0.861872724387044	-0.116656666666666	0.576277303044571
MRI1	0.0557239300934128	0.88436068540162	0.31360098684973	0.438046311563112	0.0257336666666674	0.942092082409632
ACSBG2	0.055699103212112	0.884377537954199	-0.0457076419607153	0.94547820490174	-0.129357666666666	0.552298643114782
CALCRL	0.0556981982543904	0.884377537954199	-0.284825017713252	0.498591817620473	0.884994000000002	0.0476932547477028
SLC3A2	0.0556167117649346	0.884696166751604	-0.0274150869062046	0.970045676424287	-0.211384666666666	0.294899302599823
FRK	0.0556151775792447	0.884696166751604	0.408849358220113	0.249852262368516	0.130175333333333	0.551125574415782
KRTAP19-3	0.0555327805369262	0.885008852189839	0.0493618494665199	0.941842562680095	-0.109825666666667	0.597161514418836
MGC15705	0.0555120104616135	0.885008852189839	-0.162268608920432	0.758164869707887	0.00810533333333407	0.983159241812861
FZR1	0.0554816002175385	0.885132002383809	-0.0319641149254351	0.963062370853298	0.00837433333333388	0.982375192575954
IER3	0.0553716588748244	0.885682887391673	-0.246311051063943	0.582725878221343	-0.0897267777777779	0.706115075578775
AKAP10	0.0552759990054871	0.885948847023938	0.343187307489368	0.374497295817252	-0.278779999999999	0.194593860853488
WDR73	0.0552441554833341	0.886060817636859	-0.114265044661314	0.844272085764738	-0.100320666666665	0.635069698575895
SOX5	0.0551077518777982	0.886548023869141	-0.0105610891756097	0.98774587825743	-0.0736343333333329	0.822657709336468
SERPINB2	0.0550085173833151	0.887018204342901	-0.324377610046946	0.412678992507379	-0.0515663333333328	0.838777437201588
SCO2	0.0549715042807394	0.887182217753003	-0.0987150223652426	0.868042734188964	-0.132049999999999	0.553892046464523
TPCN2	0.0548891877321285	0.887561637656812	-0.0175033723921025	0.980496266838056	-0.110486333333332	0.609689963253306
COPZ2	0.0548478641331996	0.887621363510506	-0.205819057731661	0.672625944768829	0.0678193333333334	0.83616423402744
RNF145	0.0548342789724272	0.88764009777518	-0.170085443599472	0.742891895517604	0.141476	0.615722818909839
HACE1	0.0546909956950759	0.888180313356502	-0.0435630752584544	0.94933169799234	0.216281666666668	0.337886644302564
PLEKHG4	0.0546837507989039	0.888180313356502	0.371011665998868	0.320357523974285	0.00949600000000012	0.97817310894537
GPR124	0.0546052153730466	0.888261894607364	-0.126486683314273	0.821473247266105	-0.087037333333333	0.692723628522594
BRF1	0.0545913247833357	0.888261894607364	0.268257059695313	0.53532907159569	-0.0796113333333324	0.724965653038374
KSR2	0.0545436925242732	0.888261894607364	0.156580792043102	0.768909201237015	0.00983433333333406	0.983159241812861
ZNF442	0.0545284231759828	0.888261894607364	-0.362332246619320	0.337635834513295	0.168924333333334	0.592975387754524
C11orf84	0.0545279610739571	0.888261894607364	0.271848670876948	0.52741540460909	-0.179046666666666	0.368449159208846
CYP2D6	0.0545226834126837	0.888261894607364	0.189558124983621	0.704935980001481	-0.0621296666666651	0.786261420559593
ZYG11B	0.0544679554783536	0.888386769462208	-0.396678779674102	0.270862229167822	0.0374670000000012	0.888108918943862
CRIP1	0.0544620127357369	0.888386769462208	-0.370431465665398	0.320801726666339	-0.0721579999999991	0.798344353938564
TRAPPC4	0.0544469459241726	0.888386769462208	-0.323557849087936	0.414288146732192	0.0368063333333348	0.890094615232462
EXOSC5	0.054445048598008	0.888386769462208	-0.119020630937405	0.836828045978697	-0.607166333333332	0.0244512278936471
NOL12	0.0544274670045003	0.888386769462208	0.274808212100869	0.520095539214775	-0.082305666666666	0.711082533709514
SUN1	0.0544180234553952	0.888386769462208	-0.0281282805628053	0.968668367660302	0.0426223333333334	0.86715815306237
ZNF101	0.054393901261811	0.888429735646467	-0.447470623856462	0.178249202877872	0.0879680000000006	0.756066672359783
PCBP1	0.0543844090061605	0.888429735646467	0.0851706027930995	0.890458025676316	0.057852333333334	0.829590360509491
TPPP2	0.0543592755162355	0.888429735646467	0.100863854035831	0.866005093242444	0.145685666666667	0.552298643114782
ANXA7	0.0543519476187473	0.888429735646467	0.0248954036493377	0.972871752171633	0.0681290000000014	0.761257561656748
C1orf27	0.0543477170644683	0.888429735646467	0.0134094236998955	0.98412883278237	-0.00864099999999912	0.98256318768706
GYG1	0.0542158226598457	0.888845981092287	-0.248606447442156	0.577339690903853	-0.362818333333333	0.183134786321360
RPL31	0.0542065499218447	0.888845981092287	0.433276255230897	0.201555549804162	-0.0927769999999985	0.675491463656719
MEIS3	0.0541911269991765	0.88885652835451	-0.0215144516520123	0.97536722461972	-0.0615873333333335	0.824700707442131
TPSD1	0.0540550290329332	0.889344857703458	0.147313629492215	0.78561060123541	-0.148357333333333	0.625427927897151
KIF1A	0.0540285921440331	0.889344857703458	-0.225726726797856	0.629473073224838	-0.0624843333333326	0.816002211548937
UBXN11	0.0540203174677498	0.889344857703458	0.0464500951016282	0.94484527463814	-0.0526189999999993	0.823458541833548
OR2F1	0.0539830060921056	0.889510378099962	0.217583108958812	0.645148962732605	0.0564926666666668	0.802146947589035
HCG9	0.0539324702890177	0.889757733512644	0.16114555912038	0.760348559928014	0.139947000000000	0.514420666507349
RSU1	0.0539090609442192	0.889771826790456	-0.00713507156465677	0.991086607331007	-0.228712333333332	0.25705973114043
MAFF	0.0537763914560274	0.89035073885202	-0.136825952019435	0.803720303246183	-0.213362999999999	0.486325323768433
PTCHD2	0.0537732380776822	0.89035073885202	0.0295805360069080	0.967205787110068	-0.111102666666667	0.76409396614905
EDA2R	0.0537014085565346	0.890533596154895	0.0106555856320254	0.98774587825743	0.113523666666668	0.589703432017128
MYCN	0.0536833207534180	0.890580112197813	0.174525380996155	0.734267769889068	-0.236014333333333	0.458006502146897
ANG	0.0536024754109039	0.890884103772413	0.128980638182843	0.815386814587013	0.241968000000000	0.291601624433203
NOX4	0.0535191201000576	0.89121232891689	0.088708380201745	0.8841943128258	-0.235849666666667	0.305855213710406
UNC119B	0.0535177065429774	0.89121232891689	0.439685494355253	0.190622420860054	-0.297678333333333	0.199156816145278
CALHM2	0.0534139347640696	0.891527232005408	0.0100957148244502	0.988421999436642	-0.164333666666666	0.462024330182512
WFDC10B	0.053367024989007	0.891643339176029	0.141773569803534	0.79509253036971	-0.0647893333333332	0.818514407749293
PDE6G	0.0533369042548795	0.891643339176029	0.213210460407426	0.655051855533403	-0.148125666666666	0.598430627659442
HNRNPAB	0.0533174964495039	0.891643339176029	-0.130107698074865	0.812445757604344	-0.313633333333333	0.121660357743656
PPP2R5E	0.0532865717153534	0.891643339176029	-0.0307178230066422	0.965444790690893	0.00183100000000154	0.995621063297892
PTPRB	0.0532865539289915	0.891643339176029	-0.271273315370727	0.527984832801825	-0.0106496666666662	0.97435171941994
CDH6	0.0532693509923035	0.891643339176029	-0.330868301870204	0.401780307922136	-0.0380856666666657	0.874908057954497
COL6A5	0.0532264117549561	0.891681403525438	-0.0490656232959415	0.942126968886265	0.00508666666666665	0.988942941807152
CDKL5	0.0531987224015088	0.891681403525438	0.129960575250932	0.812986631487699	0.169277000000001	0.385678201128102
TAL2	0.0531887133412417	0.891681403525438	0.132528591795211	0.809866082339655	-0.178315666666666	0.49971129098408
SPPL2A	0.0531666477125211	0.891681403525438	-0.147163414435663	0.785691396687707	0.0747503333333338	0.741134448923316
SSH1	0.0531241508303657	0.891681403525438	0.0214913582077696	0.97536722461972	-0.020138999999999	0.945904449997854
TBC1D10C	0.0529960578479419	0.89214967826652	-0.212458870049714	0.656785033957219	-0.111972333333332	0.781866487624294
SPP1	0.0529649898368571	0.89221099348644	0.0222258973195446	0.97536722461972	0.340603333333332	0.158572036652044
ANXA2	0.0529110481596577	0.892261446447933	-0.440080670296890	0.189842128278606	0.387928333333334	0.0562663106369921
LILRA3	0.0529067157000012	0.892261446447933	0.1423253566519	0.794365292705815	0.100812	0.626500880282759
DEPDC1	0.0529039658666192	0.892261446447933	-0.102665883043493	0.862779607455073	0.376118333333335	0.0757640455290112
DNTTIP2	0.0528592675811549	0.892381145428128	-0.695377335686896	0.00553622192861942	-0.0252746666666646	0.932533366180564
HMBOX1	0.0528528723126783	0.892381145428128	0.107510836134692	0.856954951691796	0.00119233333333403	0.99734800727507
MYBL2	0.0528138135383442	0.892426512282215	0.202053051773666	0.680054545720343	0.137606000000002	0.535492155031224
RAD23B	0.0527902865104141	0.892426514901464	0.0117547844620754	0.986418801521542	0.0297356666666669	0.907374738017166
USP49	0.0527715326902129	0.892426514901464	0.188681498720614	0.707361603786947	-0.0602276666666657	0.821391812005445
CA10	0.0527330307249548	0.892598962496953	-0.0162020846426372	0.982057247916055	-0.0390396666666666	0.899614658619676
TTC19	0.0526827292342241	0.892634934873876	0.00297157331559333	0.995177530008123	-0.0411586666666665	0.863867563453025
PCDH17	0.052656928400645	0.892634934873876	-0.356184277761047	0.346122612511296	-0.0852116666666662	0.822980493051179
GPR35	0.0526185024599845	0.892662444710085	0.0731513461002658	0.906395836076364	0.102994333333334	0.772139923817113
LOC100127888	0.0526170490370355	0.892662444710085	0.0227823599704346	0.97536722461972	-0.182639666666667	0.388109721982005

TPPP3	0.0525622403176941	0.892741107951461	0.0600413463923752	0.925677399480702	-0.0225023333333332	0.945904449997854
C7orf63	0.0525514586864471	0.892741107951461	-0.101502547606816	0.865765350834364	-0.00438166666666608	0.991175862027014
RPL29	0.0525236157242559	0.892847686695643	0.314719148048034	0.436900127228272	-0.201305666666666	0.320462604748547
DND1	0.0524927749179849	0.892972756202691	0.0938749651830957	0.875355472414883	0.0242470000000008	0.942680762825505
NOXA1	0.0524475404243188	0.893186625471981	-0.0749757223898044	0.904466414650249	-0.0331493333333323	0.907374738017166
ANXA10	0.0524217279009994	0.893280661486279	-0.169177582711359	0.74499234065498	2.93333333339523e-05	0.999822702792133
RNF17	0.0524031854098326	0.893309945712506	0.0946258229426053	0.8739154222556	0.0208876666666669	0.939071003700555
TSPAN10	0.052347605948828	0.893349352959632	0.170201893432403	0.74270446821332	-0.184217333333334	0.60378773986659
FLNC	0.0522893243695321	0.893372539887668	-0.0507052267519901	0.940271086498237	0.164881000000002	0.420976808369158
ACTR5	0.0522883119685562	0.893372539887668	-0.256330869549218	0.564430426783556	0.0537663333333338	0.822990638900131
PPP2R1B	0.0522861148026806	0.893372539887668	-0.246284656960013	0.582768925965831	0.140746333333335	0.606214492777216
TRIM46	0.0522694209033678	0.893372539887668	-0.240413828921015	0.597862631330416	0.0472056666666677	0.896403646226360
EHD1	0.0522309977130159	0.893520278299578	-0.556464877296305	0.0555982011257914	0.229509666666667	0.220405584107617
PROCR	0.0522243218945409	0.893520278299578	-0.254966072852133	0.566577861267331	-0.263551666666666	0.171851044800459
KLC4	0.0521714141133765	0.893666385325808	-0.0272613554318565	0.970207612777188	0.0495820000000003	0.828008720113345
MIIP	0.0521326793118457	0.893772448357514	-0.288477039609764	0.490230108819486	-0.0972583333333324	0.656941730375963
LRP2	0.0521305708048366	0.893772448357514	-0.0175956761965816	0.980428114126044	-0.139613666666667	0.476515795581148
HAUS7	0.0520420239841471	0.894122807540997	-0.248249361086354	0.577730323259556	-0.272347999999999	0.267522096313775
DLEU1	0.0519890335663396	0.89420307729556	0.183494322698971	0.718067440349544	0.149618666666667	0.553892046464523
IL7	0.0519803228114676	0.89420307729556	-0.500019238246036	0.113503971677511	-0.000591666666666653	0.998275219056485
FOXC2	0.0519249876353058	0.894232585036227	0.146272641815998	0.787339147446412	-0.101524666666666	0.719304894613107
CLDN8	0.0519184980399393	0.894232585036227	0.084187601273274	0.892396784625427	0.0165580000000003	0.967241125442187
CHKA	0.0518798370127816	0.894364194388683	0.090419203447488	0.881797721301484	-0.0340333333333324	0.928988192507851
OSGEPL1	0.0518156382134143	0.894482033925895	0.158018000670335	0.767373487136803	0.236112000000001	0.283747127048019
GLTSCR2	0.0517544445009214	0.894482033925895	0.213778220184622	0.653708954135733	-0.0418919999999987	0.888476605628154
ZNF367	0.0517511637899589	0.894482033925895	0.201916839990962	0.680054545720343	0.0762110000000011	0.765339817431245
SEC13	0.051714462535985	0.894577872255452	-0.202947114621476	0.677532075317915	-0.0236146666666652	0.93278021276477
RPS15A	0.0517031428266522	0.894582490541319	0.0678579372523330	0.91327502295287	-0.0798533333333325	0.742124582656713
EPHA7	0.0516749073326822	0.89462618844183	-0.279744909152193	0.508973927270509	0.0474723333333341	0.861945621270498
LRRN2	0.051612463202078	0.894796889684755	0.122678600367724	0.828930912293926	-0.0594783333333332	0.864165276005277
GOPC	0.0516013046357562	0.894796889684755	0.0452974205147501	0.946556809762233	0.0285736666666674	0.926828987420178
LXN	0.051591528656097	0.894796889684755	-0.0908144951850163	0.881323965029954	1.244579	0.0351167468854759
ASCL1	0.0515803124247233	0.894796889684755	-0.0739841201683049	0.905506084101768	-0.00851266666666609	0.982523503616988
CCT3	0.0515332405264216	0.89488620036876	0.0934586927125093	0.876319616688429	-0.111719999999999	0.592845916952892
GLTPD2	0.0514786751606437	0.89488620036876	0.227902631609922	0.624359290301343	-0.082324333333333	0.81000174583549
SLC26A9	0.0514737348545623	0.89488620036876	-0.215302690832083	0.650576096954446	0.0545680000000007	0.834470739028853
MLN	0.0514635692762689	0.89488620036876	0.206675979167603	0.670733295226775	0.0555406666666673	0.83568726766556
THRB	0.0512712915397457	0.895404864222497	0.0803962283817834	0.896965035803767	0.117539666666667	0.785476747850623
PUS3	0.0512624163036099	0.895404864222497	-0.148048466103008	0.784080588385358	-0.0727219999999998	0.811039304529434
PSMD3	0.0511918456619808	0.89542891922674	-0.227808075428932	0.624564310654617	-0.127733333333332	0.548138764076282
THSD4	0.0511452784743471	0.895517991517654	0.0552626940967202	0.933521481800723	-0.615725333333333	0.0286129614738536
TAF11	0.0510215931257606	0.896044506489494	-0.105575348943112	0.85956218633532	0.226114333333335	0.244426581435231
MDFI	0.0509433180744471	0.896460803033412	0.0343501988039726	0.959553064595381	-0.0295506666666655	0.91787726743953
ASB6	0.0509299472375128	0.896478000929866	0.106867510189380	0.857703168176671	0.000678333333334607	0.998275219056485
AVPR1A	0.0509152857387488	0.896503134625303	0.0245094047811854	0.973562415846915	0.751908666666668	0.0185304620090284
GBX2	0.0508644012732563	0.896640814018376	0.350024336043898	0.358969704130952	-0.164164333333334	0.597274793991978
FKBP3	0.0507619990244607	0.896665475803386	0.235425165724874	0.60678764664537	0.0684616666666678	0.769217868422514
KIF6	0.0507302126756582	0.896730871384917	0.200622924394015	0.680830337398347	-0.188062666666666	0.464393642723292
C4orf29	0.0507176668592709	0.896742998651665	-0.246020725420454	0.582912574644681	-0.0483716666666666	0.862457429222775
IL20	0.0506902623636415	0.896751201873248	0.0879274610307128	0.885848000380851	-0.0450859999999998	0.856502682928004
LPHN2	0.0506766741559969	0.896751201873248	-0.607726348462029	0.0292014996288439	0.135951	0.716517864791776
RPRD1A	0.0506740448762344	0.896751201873248	0.00190618912439373	0.99746293488369	0.0654090000000017	0.789149377548591
NOP58	0.0505875769947883	0.897087183823461	-0.0172022597266446	0.980909861560089	-0.137813333333333	0.512869954128574
MATN1	0.0505821411750692	0.897087183823461	-0.0140863762827064	0.983444353656142	-0.0471456666666656	0.857623516158136
NLGN4X	0.0505517242013363	0.897087183823461	-0.164183517705013	0.754189116283047	0.120644000000000	0.553892046464523
POLR2H	0.0505280352058838	0.897087183823461	0.241597980984617	0.595261877490935	-0.0278663333333313	0.911600329626215
LHX5	0.0504783199479482	0.897239115158815	0.347633518230727	0.362566587472102	-0.147255666666666	0.594074153534407
IL13RA1	0.0504522973402010	0.897269069853584	-0.341512093087850	0.377727715280457	0.0835066666666673	0.692545022319336
ARSE	0.05041460861541	0.897414664923213	0.0641171730044392	0.920005468161721	0.0929536666666672	0.673815991677713
IFIT2	0.0503515786139572	0.897692764115087	-0.292150727267548	0.484381575140535	0.239833000000001	0.268127341983809
CHADL	0.0503219163149004	0.897809971005758	0.219445307677787	0.641226345931986	-0.281088333333333	0.293255927517952
STMN3	0.0503074617347512	0.897833789913342	0.100709691628642	0.86628046156792	-0.216577666666667	0.249858906930330
ATPAF2	0.0502081869816518	0.898248536225815	0.188385017049530	0.707361603786947	-0.057137666666666	0.842052180873092
ZNF710	0.0501557404986976	0.898387113573054	0.0978558405369508	0.868852648210739	-0.0110836666666667	0.973324325276993
SGCD	0.050139606806895	0.898387113573054	0.175827638874095	0.731631458094528	-0.366085666666666	0.160589525668831
XPC	0.0500130865820647	0.898824551007336	-0.146742677308578	0.786187758946602	-0.185399666666666	0.337168522063646
MT1H	0.0500085725547381	0.898824551007336	0.0207096909622136	0.976330597778846	0.0655033333333333	0.797384658979621
TRPC4AP	0.0499866103547975	0.89884823151062	0.0874710325797376	0.886459652119148	0.0675226666666689	0.759696248427462
ATG2B	0.049899436879701	0.898974891633157	-0.179471652044050	0.724770967840177	0.247813500000001	0.218478495014124
CFI	0.0498523008700130	0.899026778693079	-0.0367633190596663	0.956918184553224	0.286767333333335	0.133614476543628
GNGT1	0.0498486629421469	0.899026778693079	-0.192933965193036	0.699427238052696	-0.0404006666666663	0.889610038918956
RAB14	0.049740264512269	0.899432175862187	-0.0620991129933812	0.923034515346352	0.100603333333335	0.655063476133482
SPATS2	0.0497063096913312	0.899485758206589	-0.180472767467636	0.722736027881253	0.0539126666666674	0.823699339501946
CYP4F12	0.0496846773255955	0.899485758206589	0.158639492839978	0.765791046741822	-0.0570379999999996	0.814223077858884
RBM27	0.0496716625899776	0.899485758206589	-0.135857851260387	0.806017283558846	-0.126353999999999	0.536810332635776
DIEXF	0.0496680437710986	0.899485758206589	-0.405013097317281	0.25480455106032	-0.404139999999999	0.0647596980686847
KLRF1	0.0496010068101546	0.899706278767185	-0.250473731172655	0.573763059271745	0.236070666666667	0.308781492431849
PKP1	0.0495856686891794	0.899706278767185	0.0998649295480827	0.867553004916133	-0.0868843333333327	0.777406710539424
DNM1L	0.049545935543073	0.900083400122944	-0.0859408144927211	0.888365513603835	-0.0459579999999983	0.843840511995531
CD6	0.0494541886532951	0.900083400122944	0.0714389288669656	0.909119908757507	-0.0341880000000000	0.914808689682189

AFAP1-AS1	0.0492783815332063	0.900317797548699	0.0887579972768173	0.8841943128258	0.00755266666666716	0.983159241812861
TMX4	0.049218922826134	0.900431218087819	-0.244544419191720	0.587653287154633	0.0092413333333348	0.976702491879288
RNF4	0.0492130991843711	0.900431218087819	-0.283166647625515	0.502129505146473	0.080132333333334	0.730777457405814
MED22	0.049207669427687	0.900431218087819	0.0921127582414968	0.879153312143409	0.0622233333333331	0.780717071872908
XPNPEP1	0.0492069639615752	0.900431218087819	0.207857660934410	0.667695745454683	-0.00427766666666557	0.988942941807152
OR2H1	0.0491699426569029	0.90049835037118	-0.0659135987956206	0.917090201751967	-0.0456499999999995	0.857663804897022
EXOSC4	0.0491683769553867	0.90049835037118	0.258787637925825	0.559347229880095	-0.185227333333333	0.367206442400058
TSPEAR	0.0491642624066365	0.90049835037118	0.315194468715402	0.435703778828941	-0.0983023333333328	0.73287100778903
C17orf99	0.0491389903192061	0.900588342224444	0.0881699598429487	0.885569889492087	-0.117256999999999	0.696194439936211
GPR144	0.0490985041584644	0.900623474125738	0.144797828485617	0.789598951980813	-0.0665576666666657	0.875624463432848
UBAP2	0.0490969123338445	0.900623474125738	0.254687714565719	0.567282506136306	-0.0884523333333332	0.673538419974363
KDM2B	0.0490803492559208	0.900623474125738	0.0189583463116775	0.97832242527852	0.0128443333333338	0.965647799452417
FKBP7	0.0490600933409617	0.900646410141522	0.440832222324089	0.188414468461186	-0.144203999999999	0.503217861354116
TRIM48	0.0490102760355051	0.900646410141522	0.0144987118755981	0.983444353656142	-0.0370287777777773	0.899614658619676
USP14	0.048987145583961	0.900646410141522	-0.302924522126337	0.462105017205825	-0.133599	0.528409919089401
WNT8B	0.0489831231016153	0.900646410141522	-0.00160837879249956	0.998066886004786	0.000388666666666723	0.99859819361966
CNGA1	0.0489645958707985	0.900646410141522	-0.0346724023099588	0.959404955370931	0.0583946666666671	0.823910138699963
FAM83D	0.0489193576009778	0.900646410141522	-0.0726813630362855	0.90746837333842	0.120459666666668	0.566075188962
SCG5	0.0488611194739812	0.900646410141522	-0.289853402394694	0.488870852256136	0.130958333333335	0.657217907037306
C22orf43	0.0488451251244017	0.900646410141522	0.358327620020977	0.343186459025557	0.100267333333334	0.661383805087653
ACSS3	0.0488438843711812	0.900646410141522	0.346107846621942	0.366042223836959	-0.206720333333333	0.301793253300179
PRAC	0.0488272558429914	0.900683438699964	0.0129982171365024	0.98456132886228	-0.0238686666666662	0.929624114746256
FOSL1	0.048797212305773	0.900739088841486	-0.568989043730489	0.0458289062312383	-0.0868703333333332	0.77718596107678
NLRP3	0.0487864753208426	0.900739088841486	-0.185933576233885	0.713471651085994	-0.0430919999999993	0.902787568004946
HILPDA	0.0487481766910573	0.900843701957162	-0.00219618101729566	0.996532780077613	-0.122727333333332	0.585641973157377
OR51B5	0.0487321905888256	0.900876791820545	-0.05105994035033	0.939432580299312	-0.174251000000000	0.49971129098408
FSD1	0.0486984880130236	0.901018231061055	-0.0168403586554713	0.981160061237418	-0.180747999999999	0.376882447735926
C10orf62	0.0486795204247234	0.901047971997178	0.241278281773935	0.596019873098193	-0.0263946666666663	0.941559580750404
SUMO3	0.0486509391640125	0.901114971069222	-0.0376464611050637	0.955365651737247	0.042737333333334	0.874857188244301
RYR3	0.0486172324599193	0.901124568175452	-0.380141914283335	0.300816333583957	0.121055333333334	0.577524803794493
ADH1C	0.0486032218278154	0.901124568175452	0.067995100357303	0.912895204191642	-0.0759243333333328	0.74424924420945
PDE6D	0.0485964883856804	0.901124568175452	0.341084015734108	0.378272273903863	0.0389286666666667	0.874960823800592
HBP1	0.0484158785201653	0.90164672752692	0.285259298056195	0.498216546497375	0.125929333333335	0.55011810398893
SNX6	0.0484006337767327	0.901663957325342	0.0812479108020887	0.896346444707454	0.131327333333336	0.543819286485342
S100A3	0.0483826533860026	0.901663957325342	-0.0180099971356173	0.979613786029786	0.00756300000000042	0.988133186272453
SH3YL1	0.0483533355070223	0.901770375122724	0.495163684487762	0.116831680799555	-0.0463993333333324	0.871104061464043
SOGA2	0.0483128202567652	0.901789492122327	-0.252587925382009	0.569357224558971	-0.0501826666666655	0.826922793139517
EN2	0.0483007424658355	0.901789492122327	0.334871293125481	0.392978591084696	-0.141082333333332	0.477955084845205
SP8	0.0482843281171611	0.901789492122327	0.026300798267885	0.970631762839502	-0.0809799999999999	0.716517864791776
PRTN3	0.0482699696344849	0.901789492122327	0.0829101626050213	0.89362638370288	-0.260235	0.215722751602022
CEP78	0.0482388684387475	0.901888119663829	-0.149814012845828	0.781147126980188	0.135160666666668	0.541939010010167
PDE6C	0.0480897590019479	0.902300019129042	0.0919254296287339	0.879153312143409	-0.0545183333333333	0.812274868326197
IST1	0.0480895955286085	0.902300019129042	-0.0189835509348935	0.97832242527852	0.0232710000000017	0.931612374241687
KCNH1	0.048067057367788	0.902300019129042	0.0664709366750353	0.916100593047005	0.297633333333334	0.460428849906031
HSPA14	0.0480133485503162	0.902490230646602	0.123425255046480	0.827264208621148	0.121365333333334	0.596617523400524
C19orf24	0.0479879761245579	0.902515967008671	0.0568613691080943	0.931205564121867	-0.0906119999999995	0.672689684469428
NSAP11	0.047945073834188	0.902593089816724	-0.0797643448235092	0.897892885174022	0.100534333333334	0.657217907037306
ZNF579	0.0479340468011735	0.902593089816724	0.147990640081940	0.784080588385358	-0.0464446666666655	0.899614658619676
GZMM	0.047933029905305	0.902593089816724	-0.0795330256771256	0.89819077139711	-0.118608666666666	0.674780899769326
KHNYN	0.0478878955144367	0.902678034524663	0.271210923052612	0.528032433754325	-0.136588666666665	0.513141966218084
TSPAN16	0.0478830665229474	0.902678034524663	0.233461182963712	0.610436189314218	-0.0653806666666666	0.785482517274791
SMARCA2	0.0478768011723789	0.902678034524663	0.285039590680150	0.49838641873573	0.0766643333333348	0.778995157976597
GABRA3	0.0478353611900465	0.902783279579702	-0.302712095644553	0.462105017205825	-0.164248999999999	0.506914816034461
GALNT5	0.0477653220502465	0.902976406231047	0.0828846284899204	0.89362638370288	0.150436000000000	0.492043348845002
NPTN	0.0477644403028397	0.902976406231047	0.0497072138445818	0.941458038227918	0.364900333333334	0.0778643319695396
LPCAT2	0.0477461520765554	0.902999245622703	-0.196252567312643	0.691685804764422	-0.322078999999999	0.268061430255324
CMBL	0.0477360121607747	0.902999245622703	0.0384675047434068	0.954545200586067	0.162280666666668	0.410343763683663
CCDC89	0.0475193427608594	0.90370782355127	-0.240942480693116	0.596648444172109	-0.137589666666667	0.528890172622518
CDNF	0.0474964292427847	0.90370782355127	0.0749098967341172	0.904466414650249	0.00319666666666659	0.993677488355417
C1QBP	0.0474035078781243	0.90421008505439	-0.0570765271402348	0.930775493791569	-0.0710256666666662	0.745225523902052
MYL7	0.0473580044847163	0.904332509614936	0.163887819193107	0.754916750929288	0.0346740000000012	0.912833899801715
MC2R	0.04734109481423	0.904332509614936	0.2267204707034	0.626530163978789	-0.109452000000000	0.628711947029009
CDAN1	0.0473303914877795	0.904333226918312	0.287208812298842	0.494100958493577	-0.272670333333333	0.197242705853315
PSME3	0.0472484885737416	0.904574483949539	-0.259012090259097	0.558522272130827	-0.0824569999999981	0.697094236295306
B3GALT5	0.0472272368331709	0.90457496483751	-0.265992712161718	0.539310262140605	-0.0819463333333336	0.761257561656748
EVC2	0.0471324086025188	0.9050886743964	-0.116681058639828	0.841809730226979	-0.253223333333333	0.174481584258959
LECT2	0.0471086365636889	0.905169062152817	-0.275914020133424	0.517549833373363	0.217372000000001	0.442526042885238
GPR110	0.0470709930081786	0.90526945035311	0.168252993143032	0.747723416813184	0.00634100000000053	0.987937064305561
MED28	0.0470429566926125	0.905374273565743	-0.339945153771154	0.380053463718756	0.0160833333333342	0.957243575945961
ZFHX3	0.0469496695102102	0.905765433691289	0.415075372329733	0.236975467659395	0.0106220000000006	0.97184501043952
CEACAM7	0.0469472858375001	0.905765433691289	-0.264970587835778	0.542477070548696	0.211629666666667	0.294300395506211
MEGF10	0.0468620898634575	0.906091112982566	0.326639543079581	0.409206882509236	-0.139129333333333	0.511381112724989
FOXD2	0.0468125310635797	0.90619846366203	-0.323702507315201	0.414215860139795	-0.0604943333333333	0.8257633904865
CNTRL	0.0468020984104350	0.90619846366203	-0.296254668417695	0.476773737766457	0.235018666666668	0.380154726640807
LINC00477	0.0467211069243401	0.906306111846112	-0.149750448144547	0.781413384330409	-0.204219333333332	0.31525316986747
DPP8	0.046711387089775	0.906306111846112	0.126108692450413	0.822164329801713	0.0408073333333342	0.87095450632169
TAF6L	0.0466831350400676	0.906306111846112	-0.0645748941281523	0.919313088327921	-0.00810949999999928	0.98025379113353
RPS14	0.0466701292159166	0.906306111846112	0.120835452338573	0.833217514204468	-0.136911666666666	0.561205824568141
TBC1D14	0.0466536759915664	0.906306111846112	0.0605915899648023	0.925462896587765	-0.191343666666666	0.340805625968811
DYNLL2	0.04664124523184	0.906306111846112	0.257100707811807	0.563116080558022	-0.0728286666666666	0.809409373518692

GNAQ	0.0465993821793916	0.906306111846112	0.0314068264327907	0.964468061403013	0.220668666666667	0.239584169389072
PRG4	0.0465385653902891	0.906528061302866	0.11666314703427	0.841809730226979	0.0126810000000005	0.969513647149429
UHRF1	0.0465050394985817	0.906595534538283	0.135680518222507	0.806567281799677	0.0598563333333341	0.790270367828216
PDCD7	0.0464940287936709	0.906595534538283	0.0769688265442511	0.901746412709424	-0.133640333333333	0.518178391451896
CSPG4	0.0464846313708898	0.906595534538283	0.237766027555626	0.602639741374674	-0.158761999999999	0.440506189605982
CAPNS2	0.0464826529609051	0.906595534538283	0.209639052862882	0.663917169288348	-0.0838253333333331	0.709458407711009
ANKRD28	0.0464464561689978	0.906751453403705	-0.127482705717912	0.81893660870295	0.242909000000001	0.215722751602022
FAM105B	0.0463803280436085	0.906934794258586	-0.244412344020624	0.58816484023304	-0.125885333333333	0.636490807097692
ARHGAP9	0.0463257724278138	0.90709921873924	-0.40774031335435	0.251655786339022	0.0162890000000002	0.965930252534257
ADARB2	0.04628601506285	0.90727677331851	0.168528721381452	0.746758286599055	-0.100969666666665	0.65498201079628
C1orf115	0.0462459721761960	0.907391527876063	0.50031871285869	0.113248709977844	-0.167291000000000	0.467798588118316
CDCA3	0.0461383169239249	0.90746626528439	0.0862646670420625	0.88794718020464	0.184304	0.371797773837046
DFNB31	0.0460448532584635	0.907665652428168	0.143605323113947	0.79255044057935	0.000927333333333401	0.99792238320329
SIGLEC6	0.0460313725664667	0.907665652428168	-0.0710333863606401	0.909387602167883	-0.0173999999999997	0.953929177066653
C10orf76	0.0459851264927694	0.907709203986762	0.245478885668687	0.584678706753837	0.0970540000000006	0.67609334973886
PVRL2	0.0459608193163139	0.907709203986762	-0.144513762516776	0.790345129882953	0.0672373333333348	0.774210549173745
LHX8	0.0459577365440050	0.907709203986762	-0.215634337408246	0.649367620811155	0.0385430000000003	0.883913953154694
LOC388906	0.0459507502380962	0.907709203986762	0.128951072495521	0.815386814587013	-0.143449000000000	0.481986611994676
SS18L1	0.0458541463305246	0.90806277682761	-0.0748630184996052	0.904466414650249	-0.0294783333333329	0.915761046438965
DOPEY1	0.0458495175677233	0.90806277682761	0.0216431610512143	0.97536722461972	0.0990176666666673	0.682390613806982
PTS	0.0458090287932244	0.908186353161824	0.298807955578376	0.470109568576009	0.140559333333334	0.49971129098408
TFPI2	0.0458080015282442	0.908186353161824	-0.0284534499415326	0.968037313642883	0.0746620000000019	0.880427463662501
GCAT	0.0457601823329113	0.908258989829727	0.325930874876719	0.409206882509236	0.00353433333333384	0.993523650516004
RPL7L1	0.0457536636817014	0.908258989829727	-0.146456132211551	0.786929225310127	-0.105343999999998	0.680436423395844
C6orf99	0.0457378434506486	0.908290741536706	0.0691596614772085	0.911837389834658	-0.049376333333333	0.924674647382585
MCM7	0.0456816141254353	0.908439295008711	-0.0381183724677068	0.95468180561467	0.0600453333333346	0.814956840733813
LAMB1	0.0456412853412938	0.908593952260806	0.287403272460508	0.493442130049965	0.0972973333333357	0.660122297714278
LINC00299	0.0456228086168772	0.908593952260806	-0.110149578020737	0.850412437492421	-0.0530909999999994	0.86098720418783
COL18A1-AS1	0.0456137774151219	0.908593952260806	0.0557428512011154	0.93339378096251	0.164245333333333	0.417125112561133
HIST1H2BA	0.0455230337577892	0.908887798088196	-0.0275056410747891	0.969866457453454	-0.121344666666667	0.661812056571718
TTI1	0.0454734049771988	0.909125002918804	-0.177230457771891	0.728803270870423	-0.0782893333333322	0.798170083215805
FBXW9	0.0454139519147468	0.909217385240736	0.250160589557072	0.574321063440325	-0.137303333333333	0.540614958546206
ALOX12B	0.0454126077431313	0.909217385240736	0.111754680097315	0.848253236274008	-0.136296333333333	0.589841732939668
TTC12	0.0454051933601448	0.909217385240736	-0.177105758184476	0.72928981985422	0.138397666666667	0.567558442908127
PYCR2	0.0453419864151158	0.909447403135353	0.302101649433699	0.462229407853213	-0.128614999999999	0.558807324932864
OTP	0.0453331959386311	0.909447403135353	-0.139628982967306	0.797957231657366	-0.00692633333333285	0.985544323496696
F12	0.0453257568593517	0.909447403135353	-0.176825796077999	0.729794187782856	-0.0721593333333324	0.76307213318662
ANKRD49	0.0452709626018411	0.909488792902415	-0.274513359798528	0.520473735870349	0.253604000000002	0.223260757343954
PLVAP	0.0452541591564972	0.909490867123342	-0.0070717399401309	0.991086607331007	-0.0377189999999988	0.923702459119057
ULBP1	0.0452208402187392	0.90953930337245	0.145754155126416	0.787804113309913	0.0510573333333343	0.857663804897022
WNT9A	0.0452115088003859	0.90953930337245	0.205122791780727	0.673382949111104	-0.0769390000000003	0.759034160486144
PDIA6	0.0451825746953591	0.90953930337245	0.134761581908823	0.807867938002244	-0.135599999999999	0.528409919089401
NFIB	0.0451572396189142	0.909596815073033	0.0224297572893535	0.97536722461972	0.116045000000001	0.577269890738407
GAPDH	0.0450953890829337	0.90976492709599	0.106902908968888	0.857671866148626	-0.0480433333333332	0.836626995068017
ZCCHC7	0.0450729313050298	0.90976492709599	-0.0106195281835358	0.98774587825743	0.0583063333333337	0.826922793139517
SLC36A4	0.0450606039915102	0.90976492709599	-0.0137199092265694	0.983872723592937	0.0122390000000006	0.966342921970461
PTPDC1	0.0450331924905124	0.909801744629618	-0.0600247586431784	0.925677399480702	-0.00765299999999899	0.98162913124242
LIPF	0.045012583490836	0.909801744629618	0.0789673149492104	0.899154242391172	0.0223823333333338	0.935991946407154
EDC4	0.0449957114123837	0.909801744629618	-0.0955289279130505	0.872754579338908	-0.0399556666666661	0.874908057954497
DDX20	0.0449799964397584	0.909801744629618	-0.164517150622436	0.754189116283047	-0.415949666666667	0.0647406940889148
RPL22L1	0.0448909217430162	0.909801744629618	0.560087225110629	0.0531223791775081	0.0713943333333349	0.741809049318412
MFSD11	0.0448482210123146	0.909831637622738	0.04961126569311	0.941552283107912	0.186563666666668	0.324443655582244
BAZ1A	0.0448110184248014	0.909928712847782	-0.313363259074559	0.438100620341757	0.139862333333334	0.488602547609567
UCHL5	0.0447594793851247	0.910054696195604	-0.0540339362455373	0.935109055935003	-0.0203266666666654	0.949295980201336
RSPO2	0.044684121598234	0.910054696195604	-0.256795838657158	0.563932442700726	-0.0899616666666659	0.774104133965233
PIN1	0.0446770686872636	0.910054696195604	0.220352187669025	0.639834350402762	0.087470333333333	0.704370299831112
BBS5	0.0446741950905524	0.910054696195604	-0.132008245071267	0.810300414153187	0.0838456666666671	0.711082533709514
PTTG2	0.044624693085907	0.910054696195604	0.0281433490659026	0.968668367660302	0.219395666666667	0.320462604748547
DCAF15	0.0446238307382102	0.910054696195604	0.283048465524147	0.502129505146473	-0.0262229999999997	0.925845244747948
YY1	0.0446206878931986	0.910054696195604	-0.0621033420934408	0.923034515346352	-0.00584133333333294	0.985884500368235
CHTF18	0.0445624484307458	0.910306120956641	0.258555613020280	0.56003769471698	-0.277014999999999	0.178800838368910
WFDC5	0.0445416505496651	0.910340997300374	0.0944341294480443	0.874384897652873	-0.0902179999999997	0.777568084061469
L3MBTL4	0.0445018639778875	0.91051786201862	-0.0902982095109893	0.882127593406303	0.143705000000000	0.562399033626288
RAPGEF6	0.0444779966228888	0.910564885753519	-0.169050070372945	0.745332742997188	-0.108827999999999	0.592937025021788
SAMD14	0.0444729061514133	0.910564885753519	0.166007855888579	0.751830402832405	0.00857100000000096	0.982523503616988
C17orf97	0.0444571129681984	0.91059635894576	0.255118109550364	0.566513418996824	-0.0678469999999996	0.77394434422275
EPHA3	0.0443953801855482	0.910777720679	-0.174164577027935	0.734267769889068	0.0853166666666673	0.791934055525056
CDCA2	0.044257446242217	0.911196336757092	-0.105757166216512	0.859545278949543	0.211641333333334	0.390966211084762
DOCK9	0.0442520692120378	0.911196336757092	0.219035869750031	0.641856878959206	-0.179771999999999	0.589306764532592
PLCZ1	0.0442100163425824	0.91131068260858	-0.0133914938191737	0.98412883278237	-0.0768259999999996	0.725237788753458
BPIFB1	0.0442013844273625	0.91131068260858	-0.0654146898974917	0.918281729166116	-0.00168266666666638	0.99628178244892
ACAA2	0.0441091448976924	0.911508885822493	0.176640622037254	0.730152680173573	0.214113333333334	0.255309811786564
IFNAR2	0.0440843693446303	0.911508885822493	-0.232824512832262	0.611711680668619	-0.21064	0.279049509382533
AMDHD1	0.0440838250104261	0.911508885822493	0.221669738035144	0.637352459162288	-0.0812086666666655	0.732898134943024
NKAIN2	0.0440778916401469	0.911508885822493	0.0912317344225567	0.880544969693834	-0.090558333333333	0.721454370213253
NAA30	0.0440656870434431	0.911508885822493	0.168660042640188	0.746712391928056	-0.112897666666664	0.615689623138822
MS4A12	0.0440520337130797	0.911508885822493	0.0180157789550539	0.979613786029786	-0.0911546666666668	0.705010402761235
NOP9	0.0440262678996394	0.911539751386506	0.318366883834534	0.429291355866025	-0.380997000000000	0.139567607482897
HIST1H4G	0.0439328141063339	0.911959113432829	0.237367779035518	0.603170893739822	-0.0833406666666664	0.708381059238031
ARHGAP15	0.0438651939496762	0.912190524085625	-0.437723602090693	0.194173491891936	0.0231740000000001	0.931446587444396

SEC16A	0.0437975930539165	0.912407085958589	-0.0361274750874761	0.958023954102604	-0.180851666666666	0.4254947046787
KDR	0.0436562382228959	0.912645371088858	-0.262197033943222	0.549752906834892	0.0888140000000004	0.68135180147671
CCDC130	0.0436322415752012	0.912645371088858	0.0995585690344648	0.867603998426271	-0.0514096666666669	0.824700707442131
NHP2L1	0.0436191384913428	0.912645371088858	0.110156819753753	0.850412437492421	-0.0997956666666667	0.667098538791373
ASB11	0.0436005410874622	0.912645371088858	0.0959822679121897	0.872311810447594	-0.112533333333333	0.581811561322474
TMEM105	0.0435765671600708	0.912659612484531	0.111509874413326	0.848875498955953	-0.304659999999999	0.338754799084584
CDH16	0.0435107529482318	0.912802933599338	0.158660146447178	0.765791046741822	-0.117700333333333	0.605928551077722
FKBPL	0.0435101096726956	0.912802933599338	-0.105119980246143	0.8606258296021	-0.0250266666666656	0.922463263763
COPS7B	0.0434840189099807	0.91289245751568	0.42280243962685	0.220881685540025	-0.151824333333332	0.450233254347116
PACS1	0.0433505352193377	0.913439676881168	0.201361678806881	0.680054545720343	0.211736000000001	0.309421300309741
KRT24	0.0433351297833036	0.913439676881168	0.0817651369166234	0.89548747312664	-0.0100843333333334	0.974451420920923
USP28	0.0433298583830495	0.913439676881168	-0.360655197850870	0.34034906917442	0.0939030000000014	0.710501625370744
IRX1	0.0432160415222188	0.913795481167956	0.212582553896272	0.656504774622482	-0.174315666666665	0.581502485736275
ABCG2	0.0431813617248793	0.913795481167956	-0.181512238304379	0.721064177689136	-0.914235	0.0273713913650908
SHISA5	0.0431555459860218	0.913795481167956	-0.125284101673569	0.824230922676958	0.117688666666668	0.616658572613329
ST3GAL6	0.0430782990655047	0.913994623324273	-0.054946928546495	0.934246366517164	-0.274086999999999	0.267900565260008
SSR3	0.0430067406462837	0.914117190323323	-0.0946493138746705	0.8739154222556	0.292261666666666	0.145814726785909
CCDC84	0.0430055431224862	0.914117190323323	-0.0917616662739327	0.879153312143409	-0.0686873333333324	0.777568084061469
HSPB6	0.0429569343348756	0.914237370035319	-0.0680582967784288	0.912698920271358	-0.123096000000000	0.737472059237828
RRAGB	0.0429288368027692	0.914237370035319	-0.151773627977281	0.77656696724428	-0.304682999999999	0.177509274522709
EIF3K	0.0429264785473001	0.914237370035319	-0.0370315350362559	0.95664230789783	0.00848600000000266	0.982375192575954
OTOGL	0.0429066683671	0.914237370035319	-0.332903956686066	0.397658808533403	-0.0117213333333327	0.970481529511925
SH3BP5	0.0428918703387277	0.914237370035319	0.397423747428515	0.269969669080247	0.0982010000000004	0.660936117491532
OR7A17	0.0428886347237889	0.914237370035319	-0.106171462785136	0.858649831553783	0.0568809999999999	0.838055013770737
TTF1	0.0428641913106730	0.914237370035319	-0.0749888705089873	0.904466414650249	-0.0621843333333314	0.821526499502248
HPCA	0.042862920336863	0.914237370035319	-0.0420225244936856	0.951989245458556	0.150852666666668	0.472599709268839
BFAR	0.0428518156192775	0.914237370035319	0.0193120282921041	0.978109081170804	-0.0472086666666658	0.846917872248932
BATF	0.0428230565680578	0.9142996920267	-0.148119354189967	0.784080588385358	0.0283580000000008	0.925845244747948
C16orf45	0.0428161736571612	0.9142996920267	0.26016773881308	0.554683096193634	0.236056333333334	0.274121181062751
OTC	0.042743615552216	0.914673623795577	-0.0261831743045219	0.970632644458507	0.0396766666666669	0.886819278471755
PNLIPRP1	0.0427057600667041	0.914774015264956	0.0497003509102481	0.941458038227918	-0.101301333333332	0.674028958837018
PPM1F	0.0426806671590127	0.914861418308283	-0.343219471439918	0.374497295817252	-0.172444666666667	0.408033886002958
SNW1	0.0426588808432608	0.914864805792675	-0.176003050507405	0.731162861882915	0.094461666666667	0.646975691115257
WDR86	0.0426413355904177	0.914894707419021	-0.220302559938622	0.640034899944517	-0.0896920000000002	0.77415898386052
GPC3	0.0426174693321201	0.914922587747512	0.0676143829505432	0.914061739278386	-0.0161319999999990	0.958565523123178
SI	0.0425249702521204	0.915030398473745	-0.0582280436161656	0.928879154519933	0.0343163333333338	0.890094615232462
MS4A3	0.0425092896065790	0.915030398473745	0.147718418432976	0.78455364012166	-0.0787883333333328	0.731974410873657
SCNN1B	0.0424563875785744	0.915030398473745	0.340547856874877	0.378350877728096	-0.0165236666666665	0.952448105846014
ZNF624	0.0424331587189831	0.915030398473745	-0.226519710454351	0.626993722302945	0.244483333333334	0.29098458066948
MAGEC1	0.0424290009971227	0.915030398473745	-0.242918681577944	0.592178030679976	-0.117211666666665	0.557611184484231
ZDHHC16	0.042427184259895	0.915030398473745	0.194239181391165	0.696562453325836	0.0252440000000004	0.931494545972758
HMX2	0.042404018790863	0.915030398473745	0.184271407276499	0.717436396325728	-0.114143666666665	0.661362028309397
AKAP14	0.0423433439494818	0.915030398473745	-0.0411701468821235	0.952098348256047	-0.00425499999999984	0.988133186272453
UPF1	0.0423362595777209	0.915030398473745	0.0335953733353924	0.960670911981755	-0.108025666666666	0.630743046685015
TEPP	0.0423334696601092	0.915030398473745	0.236546900968615	0.604679221319698	0.006021333333334	0.987499363290182
LCE2B	0.0423118180749171	0.915030398473745	-0.0674269862375717	0.914318618000915	-0.049274333333333	0.861677398924522
SPIN3	0.0423007790618415	0.915030398473745	-0.243866547586213	0.589605368171009	-0.123888333333332	0.558494409261702
DBH	0.0422891299640728	0.915030398473745	0.224247839128173	0.632870078168085	-0.0741276666666666	0.761257561656748
HTATSF1	0.0422829307373561	0.915030398473745	-0.557718034260194	0.0549063676899021	-0.0202623333333327	0.94678260229888
SH2B1	0.0422808690856749	0.915030398473745	0.168032277718314	0.748024524669236	-0.254307666666666	0.194072775206065
C22orf15	0.0422496002941739	0.915030398473745	-0.201786361114659	0.680054545720343	-0.233523000000000	0.412758598920913
DOM3Z	0.0422494835352039	0.915030398473745	0.213698689935687	0.653708954135733	-0.0765729999999999	0.730777457405814
MAOB	0.0422275928344301	0.915096110960594	0.238495483374885	0.601159664824234	-0.0771506666666657	0.729368274679147
PI15	0.0422173629544654	0.915096110960594	-0.148076433013298	0.784080588385358	-0.0834009999999992	0.698638671208267
WDFY4	0.0421856922276115	0.915207840219804	-0.138765410044511	0.798926084240744	-0.148179999999999	0.523453039510001
PPP1R1B	0.0421457343444461	0.915207840219804	0.0445645130264831	0.947930707937031	-0.0158023333333329	0.962275697500938
POLR1C	0.0421178276206546	0.915248441248404	-0.182340656216904	0.719419078472232	-0.216827666666666	0.27587475645008
TMEM247	0.041910328441684	0.916154962186334	0.0241536012113098	0.97420021343239	-0.176323333333333	0.5530742296054
VWA1	0.0419036397876801	0.916154962186334	-0.113201799978423	0.845978328141518	-0.111996000000000	0.696194439936211
VNN3	0.0418533539585618	0.916329955404901	0.0457157364091448	0.94547820490174	-0.102829666666666	0.639002829892966
DDX1	0.0418060731555157	0.916409430419327	0.102147632071037	0.864337135688791	0.109213333333335	0.60378773986659
KIAA1683	0.0418060704842072	0.916409430419327	0.0303268629716155	0.966100909843628	0.0996480000000007	0.719839702715467
TRIM47	0.0417224877788411	0.916643867864817	-0.122839496557649	0.828721578450338	-0.147046333333332	0.508080341765777
WDR4	0.0417056874570562	0.916643867864817	0.141866482985882	0.794791289237846	-0.272416999999999	0.226499104403529
ATP6V1B1	0.0415813613054328	0.916971078454599	0.395944313137	0.271850185265546	0.00755300000000042	0.983159241812861
NME4	0.0415728778266126	0.916971078454599	0.313358881304671	0.438100620341757	-0.00791166666666573	0.982375192575954
C1orf111	0.0415664003779099	0.916971078454599	0.109810991708736	0.851106621132981	-0.0411029999999995	0.87796421789874
BRS3	0.0414786077694761	0.917371612223244	-0.0990880224631478	0.868042453226125	0.153732666666668	0.491578070092
EIF4G2	0.0413417021740146	0.917737026097232	-0.0516351966185165	0.938514797510096	-0.0650866666666649	0.767958022875087
CCR7	0.0413036396990792	0.917737026097232	-0.110970581434690	0.849357727620213	0.0195080000000013	0.955460918122607
SLC25A17	0.0412886841140487	0.917737026097232	0.14242626220576	0.794346532964905	0.0517963333333345	0.829384095613517
PDLIM4	0.0412656304866589	0.917737026097232	-0.135147147716484	0.807470535721699	0.0144863333333344	0.95805797537369
SSFA2	0.0412578792473979	0.917737026097232	-0.107846485123451	0.856307629977434	0.432868666666668	0.0416860404261162
ZNF501	0.0412526801352721	0.917737026097232	-0.234256533128344	0.608813237170423	0.322671333333334	0.285116883835521
DHX37	0.0412513250628097	0.917737026097232	-0.114842937642224	0.844162087612813	-0.147547999999999	0.485727384606525
FOXO3	0.0412102287428463	0.917893325813482	0.0954250366402733	0.872828680033553	-0.243196999999999	0.476790385924269
ZNF829	0.0411375259761413	0.9182095750328	-0.229244159696195	0.620409044581731	-0.0286456666666656	0.90945379489823
CCL1	0.0410704416786659	0.918357470383983	0.134148881368220	0.808009477165608	0.0554860000000007	0.814780717126634
SYNE2	0.0410441567566626	0.918448445787078	-0.247642960198855	0.579577896184371	0.266786333333333	0.326830048540369
C1QTNF4	0.0410168676675282	0.918448445787078	0.0510370610695703	0.939432580299312	0.00689333333333406	0.985544323496696

TNKS1BP1	0.0410009341482777	0.918448445787078	0.0904055218635771	0.881797721301484	0.0163143333333348	0.959218131095776
LRRC34	0.0409971546017034	0.918448445787078	-0.105243119586023	0.860230380568262	0.0997290000000002	0.628711947029009
MMP15	0.0409433890055107	0.918610386662619	0.0506211087497999	0.940404217212903	-0.275600999999999	0.263064019049831
ZCCHC2	0.0409310016278135	0.918620992156228	-0.119580647437514	0.835862307448206	0.0678068333333336	0.759445936839873
MTL5	0.0408650553234048	0.918889642485193	0.474585799241452	0.144197096534399	0.0789846666666672	0.719304894613107
MMP26	0.040833072634981	0.918954147954213	0.0416729350561039	0.951989245458556	0.0940253333333335	0.758341714501375
FGFRL1	0.040723937391	0.919247908418663	0.244085888783422	0.589082004066092	-0.155155999999998	0.480330266927924
PNPO	0.0406747659926909	0.919415412797575	0.163655212013860	0.754968203901817	-0.0491553333333336	0.831668247201974
LSM14B	0.0406478905854238	0.919415412797575	-0.0151398104615565	0.982921609770846	-0.147365888888888	0.511381112724989
TAF2	0.040579475656942	0.919416353645952	0.130846881816035	0.812271254601992	-0.0548539999999994	0.808241799908975
TEKT3	0.0405747849145846	0.919416353645952	0.294375146050465	0.479822264798778	0.130299000000001	0.551095525785999
EEF1D	0.0405500588951085	0.919416353645952	0.394687644259236	0.273838292759582	-0.12239	0.541939010010167
TLN1	0.0405293443020845	0.919416353645952	0.157188149090568	0.76810607001971	0.0723600000000015	0.76648935876098
TRA2A	0.0405111291100843	0.919416353645952	0.175389700220687	0.73213520578756	0.0845825000000007	0.758341714501375
LDHA	0.0404832411596875	0.919416353645952	0.118166089297693	0.838480113978299	-0.120619999999999	0.5531802635914
SNTN	0.0404809715703742	0.919416353645952	-0.118392901120187	0.837937752076601	-0.0130763333333329	0.969513647149429
C1QL1	0.0404627008358507	0.919416353645952	-0.0041098049060852	0.994466794467494	0.0452016666666677	0.903864363916236
ENPP7	0.0404578650453489	0.919416353645952	0.315812858934108	0.433880593421022	-0.109300666666666	0.663776173049665
GABRB2	0.0404253992751214	0.919416353645952	-0.362954718567792	0.336796064934793	-0.00639266666666607	0.987163226136842
AK5	0.0404220313666079	0.919416353645952	0.228571468178753	0.622857918814168	0.0501900000000006	0.884789546675685
HECW1	0.0404002821689286	0.919416353645952	-0.172394937535370	0.738537617651621	-0.175194666666667	0.51786180567444
MGAT5	0.0403842826081734	0.919416353645952	0.0623893013523015	0.922722084234165	0.0700236666666672	0.785482517274791
LOC100128988	0.04035384054367 1	0.919428749564321	0.0634521334615809	0.920855211745173	-0.0919066666666663	0.685488482699362
SOD3	0.0403362147951197	0.919428749564321	0.0164033670816597	0.981892085795004	-0.00666133333333253	0.983159241812861
GSTM2	0.0403314439631918	0.919428749564321	-0.116902481136325	0.841512197646315	0.044745833333335	0.871242375260852
GSG1	0.0403284450819932	0.919428749564321	-0.0237642890353837	0.974918978309863	-0.0256289999999996	0.942092082409632
R3HDM2	0.040318488438388	0.919428749564321	0.224484314747347	0.632710354180004	0.0555543333333339	0.815156860161333
P4HA1	0.0402989500060709	0.919451394694095	0.325569029230134	0.409206882509236	0.112852666666668	0.596617523400524
GUCY2D	0.0402934714750752	0.919451394694095	0.135478429124520	0.806930030832744	-0.0840626666666667	0.748388402768556
PELI2	0.0402562024914917	0.919521790311193	-0.223006771543040	0.635247978870818	-0.076095333333333	0.729368274679147
TMEM216	0.0401113740709446	0.919805956552342	-0.227069715315823	0.62581495163125	0.0980673333333341	0.66929001968245
LECT1	0.040053052873402	0.919805956552342	0.170248818609868	0.74270446821332	-0.241486333333333	0.279265360641951
LARGE	0.0400427788333159	0.919805956552342	-0.118959662968598	0.836828045978697	0.143273666666668	0.499820889593952
DLL4	0.0400417925792951	0.919805956552342	0.0648431376587607	0.918590305362052	-0.154063333333333	0.461415076124779
GPR101	0.0399974393607762	0.919828573037843	-0.00240161348044632	0.996089341390155	-0.0892609999999993	0.705734010576397
POU6F2	0.0399958171097943	0.919828573037843	-0.0083074241586239	0.989970322441312	-0.0193179999999995	0.94678260229888
RIN2	0.0399930466957746	0.919828573037843	-0.0392092434779251	0.953905313456582	0.0184446666666671	0.957940366305124
SIX4	0.0399862898373433	0.919828573037843	-0.124858450224677	0.824472060590171	-0.129258333333333	0.587396808178803
BREA2	0.0399253699169256	0.920002668653756	0.0793744177518855	0.898323801514594	-0.0395566666666666	0.871825961559453
C1orf87	0.0399099459138270	0.920031444934698	0.125593746530765	0.82367707928204	0.0235716666666669	0.928897428735123
PI4KB	0.0398825405057502	0.920073874934438	-0.163000178135493	0.756782785150973	-0.287080666666667	0.188549331106419
RAD21L1	0.0398398123867527	0.920170541196217	-0.0895927792313056	0.883199811446942	0.113485333333333	0.626274169235363
SPTLC1	0.0398336210655225	0.920170541196217	0.0356435953738035	0.958689318480951	-0.0625669999999998	0.779668393144765
CPNE6	0.0397918400587398	0.920293545958486	0.323416140976769	0.414618178044634	0.0202980000000005	0.949295980201336
P2RY4	0.0396627441057522	0.920695170173492	-0.147423215812702	0.785497496933202	-0.152244999999999	0.441366313859876
TMEM127	0.0396610344237122	0.920695170173492	-0.306640166808180	0.455057534399465	-0.104033999999999	0.673921955474628
ESAM	0.0396398672559377	0.920758297704929	-0.118516434502436	0.837761659039664	-0.182487999999999	0.412670239383384
GPATCH4	0.0395720518903158	0.92093397621954	-0.132204418211778	0.810300414153187	-0.244689833333332	0.243677515322349
ITPKA	0.03952864741479	0.920958838495851	-0.0414827424428322	0.952098348256047	-0.112911333333333	0.595150543243344
FGFR1OP	0.0395001286245469	0.920958838495851	0.0916943073381988	0.879153676419416	0.0566860000000009	0.825852772242435
XPO7	0.039452302783091	0.921117888257308	0.0810508861856553	0.896361013063453	-0.124234333333331	0.580149967309238
CORO2A	0.0394140494637685	0.921168793244392	-0.0758240127965641	0.903510413986113	-0.411951666666667	0.114391489603784
LAMA1	0.0394119223528442	0.921168793244392	0.0941403246400006	0.8751259691816	-0.0419163333333326	0.883878395614985
ESCO2	0.0393840296422786	0.921208545153004	0.0901451020964996	0.882249601181978	-0.133928999999999	0.550904785888199
HRC	0.0393209368620193	0.921458874226097	-0.255578888029019	0.565794380350602	-0.0201073333333331	0.946953175174742
MDM1	0.0392783039784667	0.921561574063926	-0.0327059223891189	0.961351562552925	0.132640000000001	0.604153257694145
AHSA1	0.0392778453421636	0.921561574063926	0.0893027952951467	0.883460564717377	-1.05433266666667	0.00809874206560261
PTTG3P	0.0392610253642529	0.921561574063926	0.0724867551659094	0.907556175729082	0.139681000000001	0.528501553687416
RNF215	0.0392472763905433	0.921561574063926	0.160140407247968	0.762902034786947	0.127231666666667	0.541939010010167
CRTC2	0.0392230178054489	0.921599665705712	0.0588294905591985	0.928306114846356	0.153423666666666	0.441909622750662
SFMBT1	0.0391962336380343	0.921632752163081	-0.0134126929973860	0.98412883278237	0.266715666666668	0.213876860878204
DCLK2	0.0391724030928662	0.92167803679331	0.0986212938789132	0.868080433714765	0.0297290000000011	0.931025463699155
ADAMTSL3	0.0391674091788449	0.92167803679331	0.221979839637049	0.636647953876148	-0.0563043333333329	0.810625157938411
UBE2E1	0.0390830150828199	0.921950417641824	-0.0907957154799007	0.881323965029954	0.227360333333334	0.233528575266801
SRGAP3	0.0390762057760784	0.921950417641824	-0.203254340812003	0.676646782959283	0.0153160000000000	0.96589392686322
C6orf62	0.0389841650505106	0.921950417641824	-0.133514918668010	0.808933084408149	0.129641000000001	0.601564043046757
BCAR1	0.0389751211616526	0.921950417641824	-0.120911710384337	0.832982209969764	0.0155450000000016	0.957391035727797
GPR123	0.0389689791934312	0.921950417641824	0.150991095360496	0.778205892965504	-0.0364776666666661	0.902208543897795
THEMIS	0.0389676439184327	0.921950417641824	-0.216555293171424	0.647532674045148	-0.00506433333333284	0.987937064305561
ADAT3	0.0389205205246272	0.921950417641824	0.325508924295139	0.409206882509236	0.0731580000000014	0.751653256027624
DNM2	0.0388919350452744	0.921950417641824	0.117873386688318	0.838686136084666	0.105223666666667	0.607898776363481
LIF	0.0388549075727195	0.921950417641824	-0.0710942135709332	0.90935479990339	0.118317000000000	0.778477148290034
ZNF350	0.0388153646057555	0.921979061812168	-0.192602409740736	0.700179434713673	-0.0620629999999996	0.83023589034143
TNFSF11	0.0388089264442604	0.921979061812168	-0.0321304287742578	0.96274336578329	-0.0902116666666658	0.667697330885416
CDON	0.0387514365165086	0.92218814107511	-0.352356845501499	0.352921806679009	-0.287483666666665	0.188232886284507
UBXN2A	0.0387490387969364	0.92218814107511	0.0134891264137042	0.98412883278237	0.149093000000001	0.443378989641463
STX2	0.0387224688034492	0.92218814107511	0.0266448474597544	0.970207612777188	0.097052000000001	0.68171195530621
PIGS	0.0387155527536941	0.92218814107511	0.365728176763785	0.32927628703889	0.0607806666666671	0.80091417944586
C9orf37	0.0387101685422527	0.92218814107511	0.351903239534691	0.354396334441796	-0.0165756666666651	0.955460918122607
ALPK1	0.0386844270059327	0.922212933645214	-0.256068346927252	0.564792882906668	0.14322	0.668408423975192

COQ10B	0.0386703188295854	0.922212933645214	0.0731485810494264	0.906395836076364	-0.219180666666666	0.25507701188487
ACTL10	0.0386380719524112	0.922212933645214	-0.203275178649501	0.676646782959283	0.0045853333333336	0.990496921312903
CCNA2	0.0386236798175052	0.922212933645214	0.0910086609656712	0.881119366945433	0.0199766666666680	0.951767328727707
ATAD3B	0.038621801317176	0.922212933645214	-0.155277328706901	0.77058108906698	-0.228456333333333	0.227200366691512
ZNF782	0.0386210316584462	0.922212933645214	-0.078219079247458	0.899647854618521	0.185624000000000	0.437822740624322
OMP	0.0385902043675579	0.922249697595596	0.305448574055981	0.458013550008763	0.00854900000000042	0.98173288785111
HDAC5	0.0384570279087462	0.922684383667927	-0.368800796394815	0.32362669860138	-0.0900743333333325	0.667040000089664
FXR1	0.0384450135439219	0.922692673720954	0.118021205702674	0.838500126709059	0.0173400000000015	0.950820187177115
FAIM3	0.0383922891368519	0.922880423618572	-0.114544853276483	0.844272085764738	-0.00826533333333285	0.981191596773742
BNIP3L	0.0383813600961297	0.922882232415021	0.357768196862537	0.344139573942358	-0.195351666666666	0.299765846328222
RGL4	0.0383667242008842	0.922906151780716	0.228097743387279	0.623935503350068	-0.114003	0.574486578500537
CUX1	0.0382466071042872	0.923136856614383	0.178387160559831	0.727147198636309	-0.215150999999999	0.28651815312586
DDX46	0.0382248154487271	0.923136856614383	-0.137732486095694	0.801934376886443	-0.111702999999999	0.59513523612168
PKNOX2	0.0381620490963590	0.923255574293952	0.231528181951296	0.614724037644417	-0.0984706666666663	0.641957306407734
HSD17B14	0.0381294564060143	0.923255574293952	0.112202649597394	0.847454473507881	-0.257379333333333	0.196266313247769
PP13	0.0379818768791592	0.92361665674474	0.175506800751544	0.73194983434922	0.178038333333334	0.535492155031224
LINC00482	0.0379138908428541	0.923895072603978	0.291623287928475	0.485747109971932	-0.117815666666666	0.555273681994882
ARL5C	0.0378674701940214	0.923990970510774	0.149091688743807	0.782758815400076	0.0528240000000009	0.82362083475088
SPEM1	0.0378659059473962	0.923990970510774	0.189926762159935	0.704446739027045	0.104728666666668	0.678589880084092
BMP3	0.0378018884005544	0.924245676001756	0.103457204196277	0.861943478503987	0.116405000000001	0.60378773986659
TEDDM1	0.0377898920868441	0.924253807468905	0.120675459397918	0.833602134657439	-0.331720999999999	0.218627213160066
ZNF620	0.0376585952334652	0.924507962758051	-0.409052248448734	0.249802976172500	-0.158023333333332	0.508602469366771
FAM194A	0.0376560427852230	0.924507962758051	0.189801756092295	0.704724567131716	-0.0347099999999991	0.891410328517388
NECAP2	0.0376511873381405	0.924507962758051	-0.129114958446465	0.815076057740972	-0.0156069999999999	0.956846543507582
ATF7IP2	0.0376425293080064	0.924507962758051	-0.139551230346197	0.79812211463829	0.0185666666666672	0.95197590164177
KCNJ11	0.0375979159394927	0.924507962758051	-0.142165832376032	0.79450771779567	-0.000522666666666645	0.998281147912498
ERBB2IP	0.0375603358312646	0.924507962758051	-0.0618558310619977	0.923142970949889	0.244906666666668	0.194923895076377
CCNA1	0.0374986086028282	0.924547559819667	-0.126657412013973	0.820958242958398	-0.182857666666666	0.340805625968811
SWT1	0.037497176608013	0.924547559819667	0.0554173708651301	0.933409139126562	0.245070000000001	0.241555096435003
NUDT18	0.0374960523516429	0.924547559819667	0.00151643035477612	0.998236707563222	-0.0543303333333324	0.897639655731474
RUFY3	0.0374605947599916	0.924581588141701	-0.139968436770363	0.7976681602188	0.120080000000001	0.603884415419483
SMPX	0.0374506950100677	0.924581588141701	0.299059802629930	0.469643383772576	-0.206212333333333	0.385026964267459
C22orf39	0.0374312887955893	0.924581588141701	0.0147501869279170	0.98335083165959	0.00126733333333497	0.99704422037925
TIMM13	0.0374096033273081	0.924581588141701	0.157380011387653	0.767770230680061	-0.0835846666666663	0.694407056566956
OCIAD1	0.0374053009091733	0.924581588141701	0.0299291333181200	0.96664589910922	0.200812333333333	0.301793253300179
WDFY2	0.0373854556115415	0.924636406599814	0.224000269720761	0.633436730790476	0.0180570000000011	0.950980037243398
MOB1A	0.0373131986438291	0.924876491388884	-0.332524488665071	0.398142341492687	-0.0402999999999987	0.873263526318665
PRKAR2A	0.0372569049354974	0.924924345268357	0.0873042291594208	0.886868696973805	0.145938666666669	0.472920709533599
JMJD8	0.0372420801696657	0.924924345268357	-0.105137546836214	0.8606258296021	-0.0202536666666674	0.94375604597120
GPER	0.0372186223369264	0.924924345268357	0.100045197606976	0.867232592288188	-0.0639046666666666	0.818849076927228
SH3BGRL3	0.0372165613372536	0.924924345268357	-0.263842447932426	0.544971884819175	-0.309223	0.4423818632121
HMGN4	0.0372043118511625	0.924924345268357	-0.059662385349737	0.926534310457165	0.0458870000000017	0.86521320388291
ATCAY	0.0371882266980728	0.924924345268357	-0.142107313058964	0.79450771779567	-0.119900000000000	0.55155082188871
CDIPT	0.0371148554581117	0.925044818451391	0.0522752322125115	0.93792141648313	-0.120492999999999	0.632532249588464
DLEU2	0.0369376760265565	0.92571834738707	0.195851337593523	0.692857583007357	0.302057666666667	0.140269987064999
TBC1D30	0.0368970098065609	0.92589678755083	-0.203222526500003	0.676646782959283	-0.49649	0.0739218716203512
ALMS1P	0.0368337316609834	0.92604480662681	0.163920595583727	0.75491314198126	0.00115133333333338	0.997134925912045
EBF3	0.0368125918871247	0.92604480662681	0.192829811205505	0.699775002279648	0.0865403333333337	0.690641668678273
BASP1	0.0367976499042279	0.92604480662681	-0.136601906684566	0.804254387037059	-0.00811066666666618	0.98256318768706
THNSL2	0.0367721727773556	0.926092325211593	-0.000257148832980930	0.999860038008296	-0.0941993333333328	0.686659637953633
DHX38	0.0367528136668064	0.926092325211593	0.00639095780243327	0.991853352023739	-0.206017999999999	0.343729765439488
SLC27A6	0.0367471102930801	0.926092325211593	-0.031045364456027	0.965118873711308	0.224458333333333	0.440368942982395
CCR10	0.0367167066654818	0.92614791567249	-0.159842907087881	0.763150272733332	-0.151226000000000	0.443072320689246
DNAL4	0.0367058452577282	0.92614791567249	-0.112423855461962	0.847309711184787	0.218833666666666	0.311354677197697
ENTPD2	0.0366730401787466	0.926279555614712	0.136331811498096	0.804864448119512	-0.176457666666666	0.495283817122373
DRG1	0.0366409320764217	0.926343899587079	0.0068901578730223	0.99129507664977	0.0275013333333354	0.919164861512704
GYPE	0.0365917415257326	0.926400003141092	0.00379165120846937	0.994588964700772	-0.0315616666666662	0.944454301779885
CEP104	0.0365356843392761	0.926463595150192	-0.197408761100366	0.689134981159127	-0.0286446666666657	0.910333045729225
MGC32805	0.0365123153535406	0.926505966200868	0.066042255332013	0.917010884328	-0.163370666666666	0.478484283517744
FLJ38668	0.0364819120413952	0.926593358182052	0.1492976503409	0.782416617297835	-0.0910946666666656	0.76368723681351
ALKBH2	0.0363499315461239	0.926811979455277	0.0156610515698672	0.982266426585123	-0.191164999999999	0.438019817548948
RASSF1	0.0363445703292704	0.926811979455277	-0.278329700968316	0.511582984376517	0.0337263333333337	0.895093529187836
BARD1	0.0363387037700737	0.926811979455277	0.0804324658544955	0.896952038996626	0.154008000000001	0.430060390011002
PCNX	0.0362989300995103	0.926814168216285	0.129802451059980	0.813305249846504	0.0902430000000005	0.703699627914376
SLC25A34	0.0362575305899041	0.926825237408712	-0.0835046138758711	0.892833228561937	-0.220761000000000	0.249266035781889
STYX	0.0362176585464482	0.926825237408712	-0.165421529604055	0.752352242644389	-0.250436666666666	0.211849533372038
ITGA3	0.0361764066826204	0.926825237408712	-0.135907932833198	0.805895463708687	-0.0582963333333333	0.861677398924522
NPY1R	0.0361432986161456	0.926825237408712	0.189991288110155	0.704446739027045	-1.36288533333333	0.048170505761132
KANK3	0.0361114685960176	0.926825237408712	-0.0909219555434377	0.881229950034148	-0.201776333333333	0.388303383752735
CHRD	0.0360996449485704	0.926825237408712	0.519544265147182	0.0902262536464163	-0.157041333333332	0.48174711509713
OSM	0.0360812537325245	0.926825237408712	-0.150777000841686	0.778702262208655	-0.0843943333333321	0.701931918360678
ILK	0.0360603730629406	0.926885951329854	-0.339045418343115	0.382919428108811	0.122499333333334	0.540614958546206
GPR183	0.0359888714018898	0.927120546825993	-0.357155580380733	0.345098975152893	0.0179386666666664	0.952522040665323
ZNF43	0.0359302467405833	0.927169687374104	-0.209163988262353	0.66529158710294	0.601704333333334	0.0349945973792836
PRSS33	0.0358440548686827	0.927309654428104	0.100906005741437	0.866005093242444	-0.208613666666666	0.45895048781174
GUCY1B3	0.0358399686192944	0.927309654428104	-0.177378540293180	0.72856048713283	0.00519000000000036	0.987937064305561
MYO10	0.0358087062891392	0.927357414700096	-0.0862577645400736	0.88794718020464	-0.150608666666666	0.564757468001238
BAMBI	0.0357750358533862	0.927357414700096	0.345503475127618	0.367449782222739	0.165791000000000	0.526986765016044
TCEAL3	0.0357691487201776	0.927357414700096	-0.0590771693613366	0.927744704715697	-0.00637616666666602	0.985544323496696
GEMIN5	0.0357363398504144	0.927357414700096	-0.105967312002884	0.859008989305801	-0.205418999999999	0.368449159208846

PGM2L1	0.0357241183808627	0.927357414700096	-0.189860105670038	0.704724567131716	-0.148310666666666	0.65865472196943
CEACAM8	0.0357081316898342	0.927357414700096	0.0150914356642348	0.982921609770846	0.00952366666666682	0.983159241812861
PSD	0.0357067892314365	0.927357414700096	0.122071892808466	0.830036484464228	-0.0663236666666667	0.816134952712854
FKBP4	0.0357043080413872	0.927357414700096	-0.004149248590625	0.994350585522749	-0.22592400	0.280608379927999
CENPM	0.0356492419694977	0.927480443459624	0.169650039118902	0.743463590096836	0.0784953333333343	0.76368723681351
C5orf46	0.0356358369973015	0.927480443459624	-0.261583412870898	0.5514154594931	-0.0129493333333332	0.965077743699825
VAV1	0.0356165724712234	0.927480443459624	-0.256604247912419	0.564398995459794	-0.160525666666667	0.449950389559863
G3BP2	0.0356003799044937	0.927480443459624	-0.234735419671629	0.60798961802855	0.0256500000000017	0.926339187551963
NUDT22	0.0355550292966547	0.927663408115771	-0.196349206206918	0.691445158350448	-0.0794606666666654	0.712267479945689
KRT6B	0.0354682376193045	0.927750134382795	0.0232370335851085	0.975348968561756	0.243273666666668	0.332502783413088
VASN	0.0354336709373360	0.927750134382795	0.190113044541239	0.704292435545605	-0.0152933333333330	0.956567100065073
SELL	0.0354073737129333	0.927750134382795	-0.417356270742697	0.234235464483992	-0.0948006666666662	0.714382809933119
SHOX	0.0353775793255695	0.927750134382795	0.0326126979399712	0.96167737515129	-0.0792086666666666	0.727809376593958
TTYH1	0.0353415716403659	0.927750134382795	0.22685693276089	0.625901142430324	-0.0820269999999999	0.776112195351357
IRX2	0.0353408146746199	0.927750134382795	0.205618873582008	0.672695616352514	-0.0559993333333321	0.82823525844265
RSAD1	0.0353047418439983	0.927750134382795	0.159801260449572	0.763150272733332	-0.189799333333332	0.388303383752735
LY6G6D	0.035299167132885	0.927750134382795	0.342496883302029	0.375699706552901	-0.140358555555555	0.696927295330661
NFKBID	0.035295253131246	0.927750134382795	-0.391551184820265	0.280348316838292	-0.0980596666666661	0.68499503971094
FAM178B	0.03521215008966	0.927750134382795	0.0975939346730956	0.869510504043729	-0.114036999999999	0.728054565398526
ZNF540	0.0352069151493340	0.927750134382795	-0.0350380982793012	0.959140268168928	0.0313273333333338	0.90895380701166
GLIPR1	0.0351974792421551	0.927750134382795	0.149688772816206	0.78143277453973	0.349850000000001	0.106113992549159
PSAT1	0.0351835488438063	0.927750134382795	0.532153168700369	0.077325951207548	-0.393260999999999	0.0802364138234242
TRIM62	0.0351833723750300	0.927750134382795	0.141925925097661	0.794692635418897	-0.0176029999999999	0.95482028291900
ZNF653	0.0351807851039623	0.927750134382795	0.336533796879327	0.389099686779674	-0.108923666666666	0.589826330962094
ARAP3	0.0351620361127209	0.927750320951316	-0.235070772124738	0.607525465293757	-0.0887366666666668	0.748528107724507
ZSCAN4	0.0351418980532998	0.927750320951316	-0.357979351143969	0.343653896213616	0.0311163333333339	0.907374738017166
TCP11L2	0.0351304202879358	0.927750320951316	0.0691276734318948	0.911909697136242	0.302669000000001	0.297888842945994
TMEM132A	0.0351276168433256	0.927750320951316	0.0882210133048565	0.885557272034463	0.0193550000000011	0.95285450244329
FAU	0.0349919498011418	0.92840747747733	0.00103628489905373	0.998635071185654	-0.0793799999999984	0.707966006319856
CCDC34	0.0349838321253736	0.92840747747733	-0.056068041509866	0.932663091036367	0.285371666666667	0.19267746458517
ZNF474	0.0349361834740382	0.92840747747733	0.0672087604622462	0.914700759157169	0.062730666666667	0.815274179657758
GPAM	0.0348946179050230	0.928435382877912	0.555227337979242	0.0568702787223458	-0.338353333333333	0.255381448050109
RRBP1	0.0348412617874806	0.928562011746215	0.110580969745458	0.84940885580427	-0.100878333333332	0.639837290428782
PPP1R1C	0.0348192390534392	0.928589026536108	-0.143310230688954	0.79287526984225	-0.0648143333333338	0.772139923817113
LAIR2	0.0348014288130342	0.928591109465333	0.0575397056808085	0.930191499459057	-0.0332476666666666	0.903778056312437
PRPS1L1	0.0347616807046565	0.928631908360351	0.0401091192996974	0.953163386277443	-0.0377896666666664	0.87796421789874
RIOK3	0.0346991783524521	0.928698098804835	-0.0181834164060028	0.979327298872732	0.0337536666666672	0.892573269731487
RARRES1	0.0346947600098953	0.928698098804835	-0.00765347354440645	0.990405674923721	-0.00246066666666622	0.994738356997797
NXPE4	0.0346799833628044	0.928698098804835	0.0451842304165927	0.946577404106596	-0.115716000000000	0.637426515050706
TDRD12	0.0346489882257918	0.92875548630662	0.100350122981415	0.866573451223644	-0.0184566666666666	0.946623914057177
C14orf180	0.0346214431780546	0.928827822578361	0.153079832045621	0.774491384468427	-0.154062999999999	0.578893615511221
PRSS22	0.0346150078475171	0.928827822578361	-0.132248388851614	0.810300414153187	-0.134499	0.669640159813955
SMG8	0.0346034187005088	0.928827822578361	0.0162317327685321	0.982057247916055	-0.000555333333333196	0.998275219056485
LRRC46	0.0345632660485500	0.928827822578361	0.09840448784225	0.868441567107477	-0.0501053333333325	0.834148450369096
SP110	0.0345630665453362	0.928827822578361	-0.378575179777916	0.304578046752771	0.0882536666666681	0.808239992878146
C19orf53	0.0345516726429157	0.928827822578361	0.240969238008706	0.596648444172109	0.0238583333333357	0.931714916765969
LRP5L	0.0345188048610158	0.928958868310162	0.32693013238474	0.408851707589499	-0.114374333333333	0.602084409940976
CAND1	0.0344323787499384	0.929235833666713	0.0668110885380117	0.915272762844361	-0.0579699999999992	0.801352759417909
ZC3HAV1	0.0343367846272214	0.929358109891413	-0.243538981421801	0.59036707181346	0.126780666666668	0.541939010010167
TMC1	0.0342948363698766	0.929358109891413	0.0229718657328070	0.97536722461972	-0.00582666666666687	0.985884500368235
IFLTD1	0.0342684070429711	0.929358109891413	-0.200967162486112	0.680143077995217	-0.0969113333333331	0.696927295330661
BARHL1	0.0342683409550623	0.929358109891413	0.129857345742709	0.813100151511544	-0.124775666666666	0.581811561322474
AGXT2	0.0342680740718142	0.929358109891413	-0.138321521288696	0.799981150504262	-0.161125000000000	0.474413951968734
RECQL	0.034256569080374	0.929358109891413	-0.0491542246769064	0.94196835509895	-0.132222666666666	0.522830837265953
NPDC1	0.0342532296163286	0.929358109891413	-0.292450735989622	0.483593153641414	-0.0699736666666656	0.791934055525056
C19orf68	0.0342119447934756	0.929358109891413	0.132318861715422	0.810300414153187	-0.0970799999999994	0.639261828248761
FLG	0.0342118498474522	0.929358109891413	0.042248042320356	0.951772749589165	0.0270850000000004	0.919420614501031
GSN	0.0341938826635922	0.929358109891413	-0.428170575562397	0.210938728792546	-0.201633	0.512796060754107
FAM117B	0.0341833182334438	0.929358109891413	0.255480549389765	0.565900677438537	0.148377333333335	0.528501553687416
MYL12A	0.0341676592600441	0.929358109891413	-0.10314790398888	0.862401456058756	0.131945333333334	0.516217427447249
PCSK2	0.0341494643444925	0.929358109891413	-0.061455999410671	0.923894708452324	-0.0933749999999984	0.760564116489878
STK33	0.0341427287302807	0.929358109891413	-0.172644630777571	0.738421727152922	-0.264368666666666	0.198299581791991
C16orf3	0.0340882576755318	0.929373482972217	0.153360517171751	0.773870060547252	-0.221441666666666	0.338230698383994
SLC35G3	0.0340550830259770	0.929373482972217	0.107058234079648	0.8576027597496	-0.362445	0.397274061224943
GATA2	0.0340405523390503	0.929396436437419	0.172091087138295	0.738729794817225	-0.0965503333333332	0.64090250408923
ZWINT	0.0339559761671439	0.929690657599372	0.0686714146249171	0.912149839732916	-0.0696856666666666	0.76368723681351
RASGRF2	0.0339414560551587	0.929690657599372	-0.265815212878337	0.539878668275766	0.285795666666668	0.467798588118316
GLIS1	0.0339184936188625	0.929690657599372	0.0154762233310806	0.982635481361693	-0.172780666666666	0.50181000987305
GDF5	0.033890708664912	0.929690657599372	0.0492535606692069	0.941842562680095	-0.0642249999999998	0.87095450632169
SFT2D2	0.033876736964395	0.929690657599372	-0.0954148875872607	0.872828680033553	-0.182999333333333	0.433950673944394
CAMK2N1	0.0338736020652186	0.929690657599372	0.215975737461255	0.64844945813838	0.452732	0.134443467906163
CLEC3A	0.0338467090996232	0.929784695334063	-0.0397799860382835	0.953647040142798	-0.142120333333333	0.469591696339734
C3orf43	0.0338363472957227	0.929784695334063	0.101295653435759	0.866005093242444	-0.0538336666666661	0.830987625642722
TEX11	0.0338166942897326	0.929790884303285	-0.251074738888648	0.572882168047464	-0.099322666666666	0.706965493695243
OR2S2	0.0337406826128375	0.929925363719654	-0.149643018495549	0.7816230027112	-0.0501546666666665	0.874353540431336
GRID1	0.03373945288597	0.929925363719654	0.0182019016736204	0.979327298872732	0.0168400000000009	0.967985930883208
GKN1	0.0336692006196555	0.930258487792908	0.317446778171252	0.431166148274203	-0.0131806666666663	0.963537990151403
PRODH	0.0336600583908956	0.930258487792908	-0.157587813613945	0.767770230680061	-0.0191713333333328	0.959218131095776
NMU	0.0336051639782094	0.930430448360306	-0.228478705703752	0.623147838958091	0.297277333333335	0.210845715069665
EMD	0.033598141075901	0.930430448360306	-0.18992913629814	0.704446739027045	0.248648666666668	0.213025725172764

METAP1	0.0335882626980188	0.930430448360306	-0.142028390241679	0.79450771779567	0.235979000000000	0.210510817626933
BIRC2	0.0335326281888023	0.930666291692353	-0.135612476144097	0.806637220506547	0.180646000000002	0.354514755946533
EXOSC1	0.0335212081195884	0.930666291692353	0.0188286380415443	0.978704570516588	-0.00278199999999986	0.993756838831997
NTN5	0.0335076754916579	0.930666291692353	0.501226758877672	0.112878524194511	-0.0368769999999993	0.915761046438965
RPL11	0.0334949499668201	0.930666291692353	-0.0931986943000902	0.876951966086083	-0.030943333333332	0.910625003107163
ADH7	0.0333660668865906	0.930909328433122	-0.236902991748562	0.60400816456508	-0.0800493333333331	0.808149567980568
ACOXL	0.0333522271028955	0.930909328433122	0.0448879114047696	0.947228117515627	0.0183003333333339	0.949287545570912
RHOV	0.0333369671798028	0.930909328433122	0.22291772387463	0.635247978870818	-0.167504666666666	0.516265433652982
FGD3	0.0333365852715556	0.930909328433122	-0.327003174758966	0.408851707589499	-0.132893333333333	0.605491766505367
SLC39A14	0.0332216431631596	0.931115405456017	0.224339198427269	0.632829553438263	-0.62838	0.0185304620090284
TMED10	0.0332051480304977	0.931115405456017	0.0149129239948860	0.983028706010457	-0.0729826666666661	0.765339817431245
RAP2A	0.0331717141930841	0.931115405456017	-0.240110278397081	0.598073254573735	-0.292931333333333	0.130173404647118
PREX2	0.0331668788070915	0.931115405456017	0.00495970081405840	0.992823901271183	-0.0314966666666665	0.900101096868846
SHROOM2	0.0331442778029717	0.931115405456017	0.0961385062506682	0.872262037328287	-0.0825389999999997	0.789228734235839
ANAPC7	0.0331419546785897	0.931115405456017	-0.122781836706042	0.828721578450338	-0.0100599999999980	0.974451420920923
BAHD1	0.0331287907564176	0.931130246702926	-0.199897080534515	0.682647318705982	-0.131196	0.662666815705326
PCDHB5	0.0330917997058631	0.931190352491494	-0.0354845353073187	0.958689318480951	0.323229000000001	0.106403409888264
GPR68	0.033054188249079	0.931190352491494	-0.0230576965102908	0.97536722461972	0.108906666666667	0.655063476133482
EIF3E	0.0330534240000282	0.931190352491494	0.235609307778507	0.6067637636079	0.0990176666666687	0.73097308185803
MSN	0.0330496097223134	0.931190352491494	-0.360240677102465	0.34143422019372	0.0601876666666678	0.809409373518692
TTN	0.0330441058889040	0.931190352491494	0.37067117000093	0.320399684386315	-0.545492333333333	0.0309320552705188
FHL5	0.0330194852701389	0.931272381611505	-0.308459394522538	0.450795265233859	0.0518819999999999	0.821421133422134
HADHA	0.0329156087382276	0.931569698821572	-0.0455200704031438	0.94582511262151	0.0470263333333346	0.842903889380894
SNTG2	0.0328658947665147	0.931613552577265	0.282368511960962	0.503775207851973	0.0482293333333339	0.88384908879607
SUCLA2	0.0328655850292144	0.931613552577265	-0.0723053609987253	0.907667123198644	0.191914000000001	0.340805625968811
P2RX2	0.0328325776633245	0.93170750336574	0.113938221749938	0.844272085764738	-0.0691813333333328	0.800532222303294
GREB1L	0.0328282863564490	0.93170750336574	0.320617463921153	0.421921269743406	0.342869	0.162018979106236
SHROOM3	0.0327487811209033	0.932069847380926	0.125503386153381	0.823755601962118	-0.172315333333333	0.442640840368423
TROAP	0.032720382489897	0.932090580397175	0.0117592600457215	0.986418801521542	0.0407806666666682	0.88384908879607
RHOB	0.0325586680354111	0.932705009144033	0.330144804665455	0.403109575875798	0.253829	0.300124665604706
FGFBP3	0.0325516981876027	0.932705009144033	0.251414743587566	0.57205578271528	0.00236366666666809	0.994786566213798
WDR44	0.0324634006524199	0.932973246551555	-0.469065908130391	0.151936428121882	0.0589976666666673	0.843257172500914
DCBLD1	0.0324073931622302	0.933160743662018	0.389793807157161	0.282848386985187	0.122542666666668	0.616036866881905
UBXN4	0.0323972887621277	0.933160743662018	0.0225309261157383	0.97536722461972	-0.0104076666666658	0.979355199193915
RPL24	0.0323689765161115	0.93317673887949	0.290516868704960	0.488161540281804	-0.00112999999999985	0.997144862160692
ZFP64	0.0323647376182566	0.93317673887949	-0.273415558335973	0.523840034113308	-0.0115779999999988	0.968668619102887
SMC6	0.03233959476232	0.933245931443092	-0.175520187570894	0.73194983434922	0.0685820000000007	0.827433678445379
GNB5	0.0322626419240591	0.933375619204777	0.0938013320152967	0.875355472414883	0.124933333333335	0.558045577505427
DNER	0.0322557544660536	0.933375619204777	-0.120696407593528	0.833602134657439	0.314278333333334	0.290781031227171
RAB40B	0.0321988622907585	0.933375619204777	0.261084631854760	0.552696861759865	0.0146276666666674	0.96003296204918
DLGAP4	0.0321893440911762	0.933375619204777	-0.0884470838580579	0.884893243593597	0.0355186666666672	0.903788355106891
C17orf50	0.0321468829489415	0.933474508130587	-0.200582305019914	0.680830337398347	-0.0801546666666664	0.754488794029141
RNF121	0.0321403639738838	0.933474508130587	-0.162711374391265	0.757524249707797	-0.274770999999999	0.192639721677933
SCMH1	0.0321328997787219	0.933474508130587	0.0184794265998411	0.97902968742576	0.146088333333334	0.458006502146897
MIS12	0.0320708428808172	0.933565145784872	-0.163882762875161	0.754916750929288	0.294549333333335	0.159937050172911
C20orf96	0.0320654083297027	0.933565145784872	-0.0250008421839813	0.972747285652953	0.166161666666668	0.554622302038406
TRNT1	0.0320570813423739	0.933565145784872	-0.384558839453397	0.291180853572672	-0.0115619999999997	0.979527173532206
KRT5	0.0320487538148039	0.933565145784872	0.253259339996351	0.567925260831937	-0.080671666666666	0.808149567980568
ZNF420	0.0320428895624385	0.933565145784872	-0.326325714455555	0.409206882509236	0.0660380000000008	0.847929842355322
HNF4G	0.0319870541180817	0.933829554584553	-0.0198797509012043	0.977526645675126	-0.0229636666666664	0.93682999997207
CYP21A2	0.0318035910689203	0.93434212964076	0.353038979467578	0.352407344218756	-0.224671333333333	0.399012880214421
GRIP2	0.0317599498878553	0.934535090314058	0.164158337678202	0.754189116283047	-0.184766333333333	0.540255856426522
E2F8	0.0317169486313121	0.93458301766211	-0.0846949054082485	0.891309254245872	0.163217333333333	0.423306342680643
MTMR6	0.0317113644341438	0.93458301766211	-0.331525201941386	0.400099845811371	0.0897040000000003	0.677999095007544
CTSC	0.0316729469111739	0.93458301766211	-0.0602486535412402	0.925649366494154	-0.295798000000000	0.291481585177291
STRADA	0.0316566611847936	0.93458301766211	-0.0267627185082195	0.970207612777188	-0.0639569999999985	0.829625826427045
CKMT2	0.0316457498831469	0.93458301766211	0.305471892222217	0.458013550008763	-0.120826999999999	0.613030354362634
SYNC	0.0316376291651384	0.93458301766211	-0.181428583843287	0.721174140548176	0.296119333333335	0.178963558988056
RYR2	0.0316345794714863	0.93458301766211	0.0477696022311611	0.943547842937191	-0.0913406666666666	0.762898679429892
BLK	0.0316163470065922	0.934627333294512	0.230830769706389	0.616786032722883	-0.0753533333333321	0.80841656016322
RCBTB2	0.0316004311146478	0.934638526910487	-0.0928633249248787	0.877797216649453	-0.176641666666666	0.422927358730594
BDKRB1	0.0315822919507845	0.934638526910487	-0.0206878113317754	0.976330597778846	-0.136695	0.60814016771842
PRPF38B	0.0315330407128539	0.93473502060002	-0.260180783599431	0.554683096193634	-0.0716706666666656	0.785476747850623
ABCB6	0.0315195223767722	0.93473502060002	0.446826031166956	0.178661417730793	-0.05656	0.825269786103875
TSHR	0.03150908443787	0.93473502060002	0.157997001971786	0.767373487136803	0.227594000000000	0.274799196284777
MAP3K2	0.0314914055090415	0.93473502060002	-0.0824797863258011	0.8939835772794	-0.0168956666666658	0.953035846190505
RERGL	0.0314680154029648	0.934783739533187	-0.177493132807194	0.728529406441478	-0.057985333333333	0.814791818131879
C8orf31	0.0314085321235844	0.934945600907772	0.30120716431393	0.464501110297982	-0.236063333333334	0.280362360296366
KIF13A	0.0314045181119014	0.934945600907772	-0.178664796834933	0.726384604408355	0.288312333333334	0.141989461843871
SGIP1	0.0314021015786747	0.934945600907772	-0.120152312188985	0.835124910268666	0.0419773333333332	0.883189085233712
TFDP2	0.0313615141989911	0.935120462232988	0.0620584130229435	0.923034515346352	0.0382760000000008	0.879843042066371
TRMT61B	0.0313160526364199	0.935323785390852	0.00179585534950975	0.99760061742039	0.00510966666666735	0.988942941807152
IFT27	0.0312967059097723	0.935374553201808	0.206910121582255	0.669921654987559	0.0619446666666678	0.78606666960761
PSCA	0.0312561616123318	0.935549133122529	0.287791488898892	0.492287202244585	0.103641333333334	0.728894262229555
EIF5A2	0.0312052841034263	0.935597314043919	-0.015868764318421	0.982102356159698	0.276030666666667	0.164339180497304
ZG16	0.0311645641173401	0.93577287618534	0.169141362944057	0.745050364924297	0.102348000000001	0.657217907037306
AADACL2	0.0310795675552458	0.936082482961996	-0.338129381589727	0.384960636702034	-0.0625226666666665	0.821421133422134
NEK3	0.0309538108958719	0.936437752880266	-0.0582885844928443	0.928772369340702	0.113529666666667	0.626064244773985
ADRA1B	0.0309364063088568	0.936437752880266	-0.122632352270544	0.828930912293926	-0.0658673333333325	0.793596726500656
BTBD16	0.0307719596406823	0.936986605287477	-0.00178103901096921	0.99760061742039	-0.0681416666666658	0.770296367591727

CCDC18	0.0307710361233786	0.936986605287477	-0.268403074904713	0.535217216332192	0.0189983333333343	0.950980037243398
CNDP2	0.0307140959462778	0.937119764299634	-0.304348036532433	0.459458170903768	-0.0116676666666651	0.969513647149429
C12orf65	0.0306785419922916	0.937178361443884	0.116549340383098	0.841887188288611	0.0880120000000012	0.682390613806982
ADAM21	0.0306671933489822	0.937182336185693	0.0801673915790031	0.897577172229156	-0.0088131666666662	0.98147292539484
SREBF2	0.0306370429148069	0.937296005301586	0.357390080768671	0.344762106379469	-0.324040333333331	0.144613416019330
SNX29P2	0.0305615554643633	0.937356579300458	-0.190942227628304	0.702438908776299	0.149633333333333	0.443378989641463
ERMN	0.0305590130958031	0.937356579300458	0.00730607641087984	0.990873555460625	-0.0349743333333328	0.887780036680668
LOC400590	0.0305582161845929	0.937356579300458	0.249474842585345	0.575370998761354	-0.0956826666666671	0.86011149680202
VDR	0.0305417634414784	0.937356579300458	0.207245403178135	0.66888525756447	-0.319768333333333	0.148610516089671
RPL30	0.0304957421639827	0.937356579300458	0.263739107858974	0.545144326127723	0.0166900000000008	0.952154839195077
ZC4H2	0.0304775933721211	0.937356579300458	-0.218113196852720	0.64369487339336	0.119417000000002	0.600950363504414
DTX2P1-UPK3BP1	0.0304773125859876	0.937356579300458	-0.201036388723094	0.680143077995217	0.113520000000002	0.572207354314478
HBB	0.0304431294588124	0.937440915766522	0.11523173231041	0.843853826478772	-0.0309479999999996	0.904507944803624
NANOS1	0.0304402103990102	0.937440915766522	-0.0779148698162935	0.899840642834554	-0.132100666666666	0.670113640649168
ZNF517	0.0304308454318347	0.937440915766522	0.111016170652941	0.849357727620213	-0.116061333333333	0.639837290428782
MAGED1	0.0304185940193812	0.937450151869683	-0.0267397790234728	0.970207612777188	-0.0310933333333328	0.902761873816516
DNAJA2	0.0303355578499905	0.937502503190248	-0.200627735888561	0.680830337398347	-0.105915166666666	0.601564043046757
PPP1R3C	0.0303280719397594	0.937502503190248	0.0544775453114085	0.9344692810234	0.0525393333333334	0.855888714554296
TNNT3	0.0303136124827867	0.937502503190248	0.183901046946773	0.718067440349544	0.00870266666666721	0.978192116090894
P4HB	0.0302589778414176	0.937562770540962	0.00784014076174572	0.990405674923721	0.109403333333334	0.610151958903093
NUDT10	0.0302348615380153	0.937562770540962	0.0362493715783453	0.95784895623431	0.129085666666666	0.549323798856043
CCNB1	0.0302100939916302	0.937562770540962	-0.0675985992798534	0.914061739278386	0.0324766666666686	0.904507944803624
IFI27	0.0301700439564303	0.937562770540962	0.117087149281840	0.840993954510842	0.0178326666666673	0.948778075888142
TTL	0.0301646154869264	0.937562770540962	-0.289321757516321	0.488889656845544	0.0121653333333339	0.972334209448278
TM4SF18	0.0301053130848335	0.937721753882745	-0.390669267048911	0.282005640075858	0.090463	0.705698790800484
MED29	0.0300907937585234	0.937744188265283	-0.28328068382752	0.502129505146473	0.0460650000000013	0.843134844119548
KITLG	0.0300343821986901	0.93798502749796	-0.0652365264829952	0.918319001090575	0.950517666666668	0.0032803977969701
NOP14	0.0299989024292858	0.937989337941006	-0.132063793784378	0.810300414153187	-0.250735999999999	0.190246103576351
TCEAL7	0.0299573430387654	0.938056642905692	-0.289734261340326	0.488870852256136	0.0193013333333338	0.94909760991843
RAD51C	0.0298724187323379	0.938240233739979	0.0767939749221601	0.901856698918763	0.112142333333334	0.66632805122137
TIGD7	0.0298710919576848	0.938240233739979	-0.242091223631242	0.59465236351643	0.0468083333333336	0.874243670972648
KIAA1656	0.0298694460481431	0.938240233739979	0.186518603367824	0.711334804690432	-0.0016749999999998	0.996285985364494
PAX5	0.0298466217105270	0.938290678693267	-0.207345885599261	0.668877930282194	0.0404526666666682	0.871104061464043
INSR	0.0298013044597926	0.938493430715422	0.362364209062174	0.337635834513295	0.0873650000000004	0.681182934234933
PPP4R4	0.0297579775522676	0.938493430715422	0.298973562788193	0.469860024384247	-0.0062446666666668	0.987134800094338
RWDD4	0.0297566079653908	0.938493430715422	0.315661651695011	0.434276316037369	-0.0029036666666656	0.99521788734336
TMEM220	0.0295966082240365	0.939129697183682	0.325894278947661	0.409206882509236	0.121282000000001	0.544685122345675
RAP2B	0.0295532866363464	0.939195322917376	-0.337689979411434	0.385980325567681	-0.0648269999999989	0.772139923817113
GATA5	0.0295419999501379	0.939198881074168	0.546003827077443	0.0635875421378674	-0.0764493333333335	0.773303114426509
MRPS23	0.0294528682931126	0.939592996684597	0.0816028320999158	0.895851873508272	0.0552183333333339	0.837549067157779
MFAP1	0.0294162725793122	0.939681603753623	-0.329163561773184	0.405332872944013	-0.0184063333333331	0.95813639610796
DNAH1	0.029308148666657	0.940123949612265	0.00010911016456619	0.999860038008296	0.0568906666666679	0.804734312477607
OLA1	0.0292552611694981	0.940322619627203	0.140242830980963	0.797441782082887	-0.0567149999999994	0.818849076927228
TOMM22	0.0292526098486802	0.940322619627203	-0.0407516960150451	0.952426531595115	-0.0536201666666652	0.815156860161333
SLC22A2	0.0291043440255710	0.940998176312325	-0.0542664710971726	0.934705604877472	-0.122243000000000	0.580149967309238
TMEM181	0.0290884160243051	0.941028629930143	-0.0574278546416612	0.930447815322161	-0.152652666666667	0.524908901546083
PRKRIR	0.0290613144319582	0.941048804860598	-0.124435685975222	0.825021694154683	-0.0438919999999992	0.886099236060729
EXOC1	0.0290387370583660	0.941048804860598	-0.404754466323003	0.25480455106032	0.184039333333334	0.333881200421942
FAM216B	0.0289450689977587	0.941302585562577	-0.0849750889103582	0.890886878560183	0.0475130000000002	0.855857615773956
TBC1D9	0.0289193338363454	0.941390001570625	-0.146996410362104	0.785782367062857	0.308726666666667	0.162505104948410
WNK1	0.0288732207163349	0.941595814462479	-0.0636453518850531	0.920855211745173	-0.144147666666667	0.58391025729515
PNPLA6	0.0288020848556159	0.941723156087002	-0.0539777776539001	0.93518107282108	-0.0130086666666657	0.964880128671703
LAMP1	0.0287558523135828	0.94190491890289	-0.283028450027604	0.502129505146473	0.251531000000001	0.195340089722887
ERH	0.0287234982999308	0.941922706455035	-0.0125043071414227	0.985220352735461	0.0553923333333334	0.808933385359076
UBE2U	0.0287069600226537	0.941922706455035	0.273824955864946	0.522187256281732	-0.0387593333333328	0.880844178274306
WDR75	0.0286826961679879	0.941957120289815	0.0901240037331186	0.882249601181978	-0.163367333333332	0.549323798856043
POLR2J4	0.0285972194774884	0.942326541903048	-0.201628874360418	0.680054545720343	0.0420686666666667	0.867354667762818
FAM212A	0.0285696682273379	0.942326541903048	0.0286064824920002	0.96782691865619	0.0121813333333339	0.967460065937822
MMP17	0.0285408873920057	0.94232832865633	0.195979037920218	0.692325064566574	-0.130677999999999	0.581570366864416
CEP55	0.0285331979250312	0.94232832865633	0.0308298262616332	0.96529904314994	0.170549000000001	0.432960172695455
PTCH1	0.0284992668604839	0.942350859359627	-0.21253214242719	0.656647524059113	-0.107915666666665	0.669530887411007
GGPS1	0.0284828216252204	0.942350859359627	-0.0282919254901625	0.96843394074672	0.252137333333334	0.211495399841293
TKTL1	0.0283563892380729	0.94267153471507	0.0153553620871377	0.982725338620588	0.0291083333333334	0.937660160285902
G6PC3	0.0283336281025006	0.942741523417714	-0.178081805907687	0.727939088352802	0.0340560000000013	0.895093529187836
ADRA1D	0.0283154329505334	0.942785015538633	0.254966014062145	0.566577861267331	-0.0846173333333325	0.766658480627171
C1orf100	0.028254240658417	0.942881090640926	0.0446961752322805	0.947863413907075	0.0459343333333335	0.86011149680202
CENPL	0.0282523360040043	0.942881090640926	0.0394264671434167	0.953905313456582	-0.0731723333333331	0.775273488714107
RAB15	0.0282370568930269	0.942881090640926	0.183294389125615	0.718067440349544	-0.0264780000000001	0.93278021276477
ZBTB22	0.0282346760374317	0.942881090640926	0.180347577182377	0.722985510661031	-0.0202491666666652	0.942092082409632
TMC8	0.0281902756088498	0.943076554878777	-0.0672358998976202	0.914700759157169	-0.201581333333333	0.447070880722204
LDHAL6A	0.0281276324352846	0.94331573881032	0.0130628513048752	0.984547546298896	-0.0561686666666664	0.824548552404467
TAGLN2	0.0281127499921583	0.943339979110183	-0.165809065937757	0.751933348574587	0.0595616666666675	0.797348658979621
FH	0.0281012479747681	0.94334461425366	-0.127494271417078	0.81893660870295	0.0842743333333347	0.742124582656713
SCARB1	0.0279847369215415	0.943958216285706	0.335663835605526	0.389971284120091	0.0232220000000012	0.929542746325962
RGS2	0.0279608148428533	0.943983261635717	-0.137867611585674	0.801736842296971	0.763342	0.0166728696318732
IQCD	0.0279163882072618	0.943983261635717	0.177576743259677	0.728492631264289	-0.183042333333332	0.355902971812274
POU5F1P3	0.0278754117722172	0.943983261635717	0.392586197314961	0.278963043692709	0.117734666666666	0.667040000089664
KCTD11	0.0278640285327387	0.943983261635717	-0.314478645285332	0.437362953615875	0.0539070000000007	0.835752276645858
DNM1P46	0.027851927681463	0.943983261635717	0.0238630394559068	0.97479225206569	0.045158111111111	0.85994094150432
TMEM100	0.0278044538830679	0.944134292623551	0.161276116443531	0.759727404503678	0.463163000000001	0.0846385443172634

PCM1	0.0277562737972838	0.94429278400804	-0.171684058464707	0.739343956096839	0.202921666666667	0.352897567720880
CNTNAP5	0.0277435780782357	0.94429278400804	0.0187078136739761	0.978981596219275	0.138417666666667	0.495717992674359
PDZD7	0.0277237672394374	0.94429278400804	0.134908163802066	0.807867938002244	-0.0781673333333335	0.754488794029141
SDF4	0.0277001018116267	0.94429278400804	-0.300699671093181	0.465222095816255	0.0920746666666666	0.661383805087653
CYP4F3	0.0276807288055205	0.94429278400804	-0.388447163803304	0.28486220160813	-0.145418666666666	0.541759186109413
AASS	0.0276489469397628	0.94435647376786	0.106946961651921	0.857671866148626	-0.0167136666666659	0.955460918122607
DHRS1	0.0275495082232603	0.944613500470726	-0.0479191240555745	0.94314236584312	0.171073333333334	0.371797773837046
SPTBN5	0.0275289735035185	0.944613500470726	0.00011775702084325	0.999860038008296	-0.0224629999999991	0.942393983671961
ASIC4	0.0273997317729897	0.944951355101247	0.189472106718364	0.705315444616143	0.147389333333334	0.519406653645538
ATP1A4	0.0273048432748107	0.945206214944512	-0.0217406701659113	0.97536722461972	-0.112263000000000	0.626064244773985
CYP11B2	0.0272922459376647	0.945206214944512	0.325575093716312	0.409206882509236	-0.0727269999999999	0.769890867417328
SIRT7	0.0272055087614072	0.945369721294895	-0.251099011304822	0.572882168047464	-0.192690999999998	0.361086199977056
NEU2	0.0271022477095137	0.945671866100197	0.094494761651179	0.874384897652873	-0.0705959999999999	0.794486775271372
AP3M2	0.027088989617104	0.945671866100197	0.0919178071727043	0.879153312143409	-0.166596	0.474123250726025
DENND5B	0.0270665693661456	0.945700145502931	0.0219462411317265	0.97536722461972	-0.166085333333332	0.433563092628613
C1orf35	0.0270519518843784	0.945700145502931	-0.08631760785727	0.88794718020464	-0.121197666666666	0.614573658931972
RCCD1	0.0269889577239512	0.945816549517078	0.0460182518084435	0.945364757579857	0.0719260000000002	0.743139552172972
ARL6IP1	0.0269707837671454	0.945859669284727	-0.114511708750625	0.844272085764738	0.0819500000000027	0.710483929190267
PGPEP1	0.0269266252857128	0.94604508400373	0.134178501540181	0.808009477165608	-0.206733666666666	0.279049509382533
SLC22A23	0.0268918983906558	0.946098591145162	0.193236724570823	0.698676320436036	-0.193986333333333	0.33989570998241
TPGS2	0.0268865890380220	0.946098591145162	0.0638480737762549	0.920459698398106	0.0586423333333341	0.814863116417465
RANBP1	0.026853166023712	0.946229845649215	0.0594282905876785	0.92694538699714	-0.0667299999999976	0.78960166957155
NCAPH	0.0267691931754452	0.946367595948977	0.0712615211258158	0.909235435917347	0.0390243333333351	0.896186784012643
TAAR3	0.0267385237221566	0.94637057203132	-0.113524372768783	0.84538474875759	-0.0421946666666664	0.883189085233712
TMEM104	0.0267294094692180	0.94637057203132	0.0512020913489796	0.939111418302491	-0.112210666666666	0.692306221300676
KCNJ1	0.0266955368580153	0.946459348905969	0.0717226134617083	0.90838901602167	-0.00587733333333309	0.986623910782426
LYPD6	0.0266740752907993	0.946459472008867	0.0520257207364275	0.938067483409541	0.0595313333333338	0.791014715933453
ADRB3	0.0266179647011676	0.946659759639485	0.00620298332538636	0.992168210253	-0.0919856666666664	0.687794959259872
KIAA1462	0.0265889412358408	0.946682046744321	0.0801280190725686	0.897577172229156	0.468382333333334	0.119540326024184
C17orf103	0.0265641510513595	0.946692955899007	-0.125159991045983	0.824230922676958	-0.0873036666666669	0.718456537087952
HRH3	0.0265586106143443	0.946692955899007	0.130879327110558	0.812271254601992	-0.149477333333332	0.68414816517367
DYNLRB1	0.0264982457088480	0.946979663627732	-0.061038802982987	0.924772945035666	0.0476753333333347	0.840251723747548
LENEP	0.0264553070379426	0.94713629491249	0.0779881875323922	0.899705053748212	0.0872353333333334	0.781760032958938
KIAA1239	0.0263296427940826	0.947457790948142	-0.0139294651811935	0.98369342899447	-0.00874366666666623	0.978109825558428
CLIP4	0.0263066116503228	0.947528826143142	-0.485862282942204	0.131934855037098	0.394564333333334	0.065682075976559
MAMDC4	0.0262770309954306	0.947546118619241	0.176501733371192	0.730152680173573	0.106799333333334	0.639837290428782
GPLD1	0.0262714336779281	0.947546118619241	-0.145099516088275	0.788920346633765	0.0548383333333334	0.809505390888975
CRHBP	0.0262271752977347	0.947739667502743	-0.0332593198414067	0.960784250392482	-0.0633249999999992	0.826347200876756
CNTD1	0.0261878301668287	0.947904843442042	0.206645428733515	0.670733295226775	0.187000666666667	0.398554167047557
NEUROD2	0.0261663216110455	0.947967054517794	0.172621405960900	0.738421727152922	-0.0972509999999988	0.71936631462663
LMO2	0.0261062310016048	0.948251950895648	0.0224990216811002	0.97536722461972	-0.0867173333333333	0.68149943269806
PYHIN1	0.0260269363841793	0.948361405939703	-0.166024512267901	0.751830402832405	-0.0734676666666666	0.74499160363205
NGLY1	0.0260193195925465	0.948361405939703	-0.0359944540905364	0.958301184344066	-0.0733606666666657	0.76409396614905
RGS17	0.0260157986032892	0.948361405939703	-0.233477758781534	0.610436189314218	-0.0027569999999999	0.995364835892372
IL17RB	0.0260041529694053	0.948361405939703	-0.0270692877961294	0.970207612777188	-0.0900169999999995	0.6715789263798
ZSCAN5A	0.0259908681735379	0.948361405939703	0.340989807711059	0.378272273903863	-0.191923666666666	0.458154400582976
PMVK	0.0259891326718616	0.948361405939703	-0.280815655431228	0.507365125605885	-0.0665166666666657	0.77394434422275
GPR114	0.0259842460193088	0.948361405939703	0.0788092814568585	0.899154242391172	-0.0426496666666666	0.894444086329255
URB1	0.0259477319301521	0.948361405939703	0.145757674703972	0.787804113309913	-0.605701666666666	0.0802641563431964
CDX2	0.0258691659403067	0.948566919785722	0.138326363944117	0.799981150504262	-0.047599666666666	0.843152489417868
PDE1C	0.0258122628585532	0.948697258853674	-0.0335962644209168	0.960670911981755	-0.142395999999999	0.510451110608922
PDZD2	0.0258099936400023	0.948697258853674	-0.119812646322506	0.8354985445494	-0.0390326666666667	0.880131955403697
MB	0.0257942825277512	0.948697258853674	-0.0110657574493170	0.98774001793194	-0.0845403333333333	0.74164843446719
CDK1	0.0257928909239219	0.948697258853674	0.167960269417798	0.748024524669236	0.236385000000001	0.243615883115952
OR2A4	0.0257116429562954	0.949041902092118	0.106649442341353	0.857937967416038	0.0831193333333335	0.807097731653659
LOC285033	0.0256832890002790	0.949082763897123	0.0842113087510249	0.892396784625427	-0.0139419999999997	0.972026971510771
CDR2	0.0256763541824321	0.949082763897123	-0.146226400951320	0.787402233709509	0.00222966666666754	0.995613236440634
ITM2A	0.0256637747894297	0.949082763897123	-0.217357877007450	0.645781950047533	-0.135366333333333	0.499850659274909
IARS	0.0255887111850123	0.949110471602622	-0.077932191258602	0.899818425584914	-0.135926666666665	0.549323798856043
MICALL1	0.0255834267455360	0.949110471602622	-0.122232033871529	0.829569971668386	0.0909433333333329	0.707538975864338
TRMT5	0.0255492262980741	0.949235356285212	-0.0106903093615442	0.98774587825743	-0.108689000000000	0.623903073849364
PNLIP	0.0255204475716668	0.949289115921692	0.0807313849072772	0.896621311541396	-0.0375433333333333	0.885345988320645
HCN4	0.0254870485558937	0.949289115921692	0.166363152677089	0.751360233962663	-0.0377979999999994	0.915480183438261
ZC2HC1B	0.0254866029554659	0.949289115921692	0.289062291360017	0.48926451774682	-0.0306023333333334	0.91562522450879
SLCO1B3	0.0254862061274915	0.949289115921692	-0.227035604100699	0.62581495163125	0.118942333333333	0.599409959328955
PRAF2	0.0254203124234105	0.949410938549992	-0.190777299238346	0.702864526428586	0.0370216666666677	0.880429228421837
SHC4	0.0254036222306703	0.949410938549992	0.0455449752168261	0.945769054147694	0.119579000000000	0.732086971105268
CDH1	0.0253809542414124	0.949453135391646	-0.101607675480534	0.865421934557068	-0.0744899999999998	0.786460365353421
FGF8	0.0253718236824766	0.949453135391646	0.0839019831536742	0.892716669164583	-0.0862370000000003	0.776805598353461
IDS	0.0252553113694672	0.949691142494257	-0.34959068875678	0.359178920097907	0.0749421666666698	0.761406666779549
DOHH	0.0251762093458069	0.94983735015005	0.247420111268319	0.579745218830252	-0.0307166666666657	0.931714916765969
SGK494	0.0251269941580724	0.949996948972339	-0.0477438461172617	0.943547842937191	-0.313250333333333	0.27071513514891
WDR88	0.0251052899367989	0.95004076548618	0.328710688151221	0.406402952155872	-0.0286919999999996	0.923019543204612
BOC	0.0250843145352593	0.95004076548618	-0.150597821056199	0.779021144254203	-0.113453666666666	0.673995132867941
CBLN2	0.0250060669390824	0.950107081122055	-0.153067741739351	0.774491384468427	0.0924160000000007	0.704370299831112
TMEM54	0.0249257672297436	0.950107081122055	-0.00984704303430668	0.989040892573755	0.0340450000000017	0.895093529187836
SFTPD	0.0249193011192455	0.950107081122055	0.0889574098304352	0.883865906764221	-0.142737333333333	0.549323798856043
TTC9C	0.0248928034055703	0.950107081122055	-0.128869072837726	0.815391045978843	0.137567666666666	0.590841338570121
ITGA2B	0.0248884623203212	0.950107081122055	-0.0588559337220898	0.928306114846356	0.0306553333333342	0.931859006463093
RDM1	0.0248818730025414	0.950107081122055	-0.193486130680603	0.698131592467487	0.218162000000001	0.258700580229750

TBX15	0.0248771209728103	0.950107081122055	-0.00929105043684544	0.98909166696601	-0.0206653333333333	0.946623914607177
FUCA2	0.0248440614648489	0.950107081122055	0.402614703230939	0.259322048328011	0.0690590000000009	0.771847553133033
SEC14L3	0.0248285916506641	0.950107081122055	0.133540879238804	0.808933084408149	0.0278253333333332	0.94010374131705
SECTM1	0.0247751030497943	0.9501463230797	-0.407075843536756	0.252941668074154	-0.182714999999999	0.384179305818026
ALG13	0.0247682288447482	0.9501463230797	-0.438579401347489	0.192317896804555	0.160754333333334	0.523041310312964
TRMT10B	0.0247557408388873	0.9501463230797	-0.110394088403157	0.849867216915014	-0.0505426666666661	0.838336524351522
ZNF90	0.0247485065775368	0.9501463230797	-0.183455907113982	0.718067440349544	0.0751576666666677	0.748715075113364
PCDHB1	0.0247429020503857	0.9501463230797	0.0323850105098372	0.962269716253437	0.0914770000000006	0.671492528470281
DTNBP1	0.0247251107471804	0.9501463230797	-0.294334238028635	0.479822264798778	-0.306466333333332	0.132957584348716
CPXM2	0.0246863202381229	0.95030744571135	-0.287489699637381	0.493342381401319	0.0387170000000005	0.874351113566721
OR2L13	0.0246123055547001	0.950535626512187	0.0060679403822199	0.992450441941654	0.0213726666666666	0.935976721437737
ALDOC	0.0245918768927324	0.950535626512187	0.546164803231755	0.0635875421378674	-0.327994333333333	0.216591326893487
IKBKAP	0.0245885331148106	0.950535626512187	-0.271955596432522	0.527284725671175	-0.0243063333333321	0.930612078459836
SUV39H1	0.0245714029816803	0.950535626512187	-0.138557925057154	0.799373390835754	-0.150020666666666	0.442294185443201
MMP25	0.0245515414334147	0.950587992154554	-0.0401188498770679	0.953163386277443	-0.0844389999999992	0.80108268456699
FBXO42	0.0244552326748500	0.950732506402299	-0.257268484705745	0.563116080558022	0.204048000000000	0.302316846105060
FGF21	0.0244391909379331	0.950732506402299	0.269928894710113	0.530964613532458	-0.0397439999999997	0.870489343487753
LOC729164	0.0244226345033926	0.950732506402299	0.139276802499724	0.79864602540281	0.223824666666668	0.322893184569333
RAD1	0.024408207151388	0.950732506402299	0.339486046860988	0.381694849069185	-0.236315666666666	0.238780185222418
TMIGD2	0.0243109429080205	0.950982397095651	0.0215187926618656	0.97536722461972	-0.382740333333334	0.113700575160835
GOLGA1	0.0241789732825533	0.951616222882805	-0.0721548096457791	0.908192956075098	0.136163000000001	0.54201946574576
CITED4	0.02415913891876	0.951668344939702	0.0963095890731633	0.871610640556059	0.103777333333334	0.662124890319915
QSOX2	0.0241430063940147	0.951676474342177	0.221872602349073	0.636778528562165	-0.176095333333332	0.464422404884939
KLHL17	0.0241188507794168	0.951714465534496	0.127976808606095	0.817778554441316	-0.0683936666666658	0.80796582766673
sept-01	0.0240666836431882	0.95195203229021	-0.155130582707827	0.77066107821265	-0.0598283333333327	0.87152108397136
GNB1	0.0240410195667496	0.952037566778506	-0.458717557022710	0.165317675121864	0.0265700000000002	0.918760683387302
PARP4	0.0239957785650113	0.952143349057609	-0.519584646328279	0.0902262536464163	-0.152029333333333	0.491667718974619
TUT1	0.0239625855701926	0.952143349057609	0.358778308978444	0.343044374570877	-0.0919053333333333	0.677268057457651
FAM5C	0.0238377145246054	0.95234482269337	0.234789149311147	0.607957283325678	0.0771706666666671	0.762898679429892
CROT	0.0237809444480470	0.95234547684085	0.280928371142143	0.507259109959297	0.229071000000000	0.25470355352902
HAPLN4	0.0237698079096433	0.95234547684085	0.160797531503536	0.761016253411733	-0.0715153333333337	0.845737357656109
RBM33	0.0237377912452426	0.95234547684085	-0.0207159395577622	0.976330597778846	-0.0761774999999997	0.729303008652613
NKX6-2	0.0237211561950035	0.95234547684085	0.299427549308740	0.468245148036717	-0.0036266666666660З	0.993474375150267
ZNF319	0.0236748937277102	0.95234547684085	-0.0205287917395691	0.976412247012224	0.202237666666668	0.29098458066948
KCNT2	0.0236655667170303	0.95234547684085	-0.128939060960274	0.815386814587013	0.0174550000000003	0.95784037718285
PARD3B	0.0236575619929960	0.95234547684085	0.0446139177759039	0.947920610570169	0.157398666666667	0.469591696339734
ZDHHC22	0.0236554134211126	0.95234547684085	0.0589506420130947	0.928158451370015	-0.0558913333333326	0.847897923153334
AP4S1	0.0236539868611034	0.95234547684085	-0.0396219378976074	0.953729831316741	0.220337333333334	0.466833909493819
SLC52A1	0.0236160047100628	0.952501408992994	0.0492166859817937	0.941842562680095	-0.0754129999999994	0.781986317719904
PPP2R3C	0.023521112834701	0.952769895604238	-0.136491506093632	0.804536748810928	0.198603666666667	0.405379282739141
ZBTB32	0.0235153449817933	0.952769895604238	0.0873216107734147	0.88684628203837	-0.0211459999999991	0.95765714061609
CASQ2	0.0234595537303221	0.952773056149496	-0.309658179936355	0.447255805930312	0.102465666666667	0.673538419974363
SLAMF1	0.0234460043618882	0.952773056149496	-0.113954669776655	0.844272085764738	0.030641666666667	0.919420614501031
ABL1	0.023424562385747	0.952773056149496	0.0413539797806098	0.952098348256047	0.087359333333335	0.730907166061737
FOCAD	0.0234115588593218	0.952773056149496	0.115699221205005	0.843056141987295	-0.370177666666667	0.169027316227871
CRTAM	0.0233113692326861	0.953137056502014	-0.300040551909833	0.466362574195078	0.101026000000000	0.639002829892966
IFITM2	0.0233001666530096	0.95313960800642	-0.325418780339573	0.409402478681488	-0.229045333333333	0.231095714637061
RNF141	0.0232494368025363	0.953260521231606	-0.230710502714195	0.617240048465949	0.217863000000001	0.351907021382432
NUP210P1	0.0232252455459595	0.953260521231606	0.0501846821601772	0.941133149988298	-0.212360333333333	0.261011849103251
FUT5	0.0231975664664046	0.953295780308193	0.190075966438724	0.704322109927422	-0.310098	0.305655404008506
GOLGA3	0.0230534897043158	0.953605499580367	-0.107095232670856	0.8576027597496	-0.0202789999999986	0.941979039201472
NT5C	0.0230473796518446	0.953605499580367	0.408073241754542	0.251137048131401	0.126151333333334	0.546876652092462
GPRASP1	0.0230405268359499	0.953605499580367	0.0345868780409915	0.959404955370931	-0.0535643333333329	0.841643842869151
ZNF785	0.0230392872609894	0.953605499580367	-0.0688305494315089	0.912149839732916	-0.0903646666666665	0.70610473471702
RFX1	0.0230214723035483	0.953605499580367	0.141451773287618	0.795908917700498	0.0714723333333337	0.763010509046616
IBTK	0.0229852627295634	0.953648479152213	0.210067294991022	0.663000357794695	0.093007	0.682390613806982
FGF23	0.0229207950067818	0.953818662081652	-0.0588833841859177	0.928276034593633	-0.269974666666667	0.205336588312115
IL15	0.0228908381132135	0.953818662081652	0.0177721710169621	0.980232784584208	-0.0386469999999989	0.891664661404789
TTPA	0.0228783627402016	0.953818662081652	0.0442638929233096	0.948385499132766	0.00726000000000073	0.987134800094338
RAD51	0.0228625868382879	0.953818662081652	-0.0711607256560428	0.90935479990339	0.163408666666667	0.49038083653267
NLGN4Y	0.0228259650235821	0.953818662081652	0.0206942143382365	0.976330597778846	-0.193600666666666	0.394132743055997
ZNF544	0.0228102990748768	0.953818662081652	-0.267832199819108	0.53559545997875	0.133519000000001	0.516040564500327
RRN3P2	0.0227940908322097	0.953818662081652	-0.173448923090292	0.73612655430126	0.0239948333333329	0.925845244747948
ZBTB25	0.0227102970891319	0.954174282073425	-0.169063227803451	0.745332742997188	0.279277333333334	0.231998578744259
LOC728613	0.0226804043658805	0.954283524348967	-0.251109151007811	0.572882168047464	0.125236000000001	0.558858878468157
MCHR2	0.0226635995718938	0.954305007811725	-0.173037943413497	0.737325433935202	-0.138500999999999	0.492100665301584
GZMB	0.0226392718680327	0.954305007811725	-0.294232562378514	0.479894403496244	-0.155780333333332	0.549664615421682
BACH2	0.022628197690331	0.954305007811725	0.188350143858957	0.707361603786947	0.131856666666667	0.541939010010167
LGALS12	0.0226228281514703	0.954305007811725	0.0794899655130857	0.89819077139711	-0.0142573333333329	0.964514058161652
HTR2B	0.022568601875464	0.95449167718482	0.131271197601400	0.811539578433911	-0.00093866666666669(	0.997144862160692
SGCZ	0.0225181670791016	0.95471812526746	-0.0403111813101651	0.952720585102517	-0.150986000000000	0.459949741425718
RASGEF1A	0.0224867727003241	0.954835872713343	-0.309886149849617	0.4470577615309	-0.0381559999999997	0.911600329626215
GYS1	0.0224759812830476	0.954836011912786	0.295583714787199	0.478008201657401	0.0127160000000012	0.969387309763954
OR1F1	0.0224626117886734	0.954850866082341	0.0641000012810825	0.920005468161721	-0.0973253333333338	0.73097308185803
PDCL	0.0223942887554525	0.955117892736173	-0.0570199676613678	0.930784376757936	0.0268793333333347	0.915761046438965
SRSF5	0.0222775974697353	0.95546243589192	-0.0118255290631559	0.986418801521542	-0.0482106666666658	0.833915232044739
CARD10	0.0222534787255702	0.95546243589192	-0.0273910162248876	0.970045676424287	-0.0098013333333331З	0.974895360062947
FAM83C	0.0222489169553964	0.95546243589192	0.0913558329626283	0.880224854770858	-0.136169333333333	0.613737891484298
SCAP	0.0222397842469356	0.95546243589192	0.278602033985043	0.511025440770733	0.0400943333333341	0.870002591217953
AFF4	0.0221614060144126	0.955709135771916	-0.114933468213715	0.844162087612813	0.0483246666666673	0.847985525870412

SLC6A20	0.0221423375522225	0.955749722487123	-0.0389207208069925	0.954165422885166	0.00672700000000027	0.987134800094338
DGCR14	0.0220299955214713	0.955749722487123	0.148269077509243	0.784080588385358	-0.00347666666666571	0.991175862027014
TUSC3	0.0220218915808879	0.955749722487123	0.298273661140880	0.471657032118147	0.238116666666668	0.205838922584493
EPHA8	0.0220194314843679	0.955749722487123	0.198960906248166	0.685196748018683	-0.0877236666666661	0.748388402768556
RCC1	0.0219967695484632	0.955749722487123	-0.0466037933938344	0.944599178367537	-0.0563936666666656	0.830234611990443
POLQ	0.0219866408349495	0.955749722487123	-0.0155446733976657	0.982505387450863	0.0931063333333333	0.775688971019473
MAP2K1	0.0219699956611937	0.955749722487123	-0.0915645627527055	0.87943055126106	-0.0287203333333322	0.90947946741371
ERVH-6	0.0219684115884473	0.955749722487123	0.0887045377588472	0.8841943128258	-0.135834333333333	0.600310031030304
SPRR1B	0.0219603773269084	0.955749722487123	-0.109520897503892	0.851624566716206	-0.0704386666666659	0.760951989611575
RBBP8	0.0217857074173648	0.956376614892663	-0.240024416805624	0.598092982260025	0.123803333333335	0.549323798856043
L3MBTL2	0.0217531036500019	0.956378166974644	0.149449195353511	0.78215339162259	-0.0990746666666659	0.637224122022876
ADH4	0.0217092072562572	0.95643466602139	-0.190234752542238	0.704208582824765	0.0696763333333335	0.761257561656748
MRPL45	0.0217065773929225	0.95643466602139	0.115562405150327	0.843372362327421	-0.207602999999998	0.316090386778478
ZNF775	0.021687554771624	0.9564445996635	0.255663423256750	0.565656702091657	-0.113091000000000	0.597274793991978
MDS2	0.0216166193300453	0.956657481276069	0.139021823627310	0.79864602540281	-0.0359226666666659	0.910190419163007
CDH7	0.0216068036268561	0.956657481276069	-0.124179550325421	0.825382714207684	-0.142532000000000	0.60026500215056
PRPSAP2	0.0215764172996224	0.956657481276069	-0.07743036415521	0.90079793355475	0.156670000000002	0.426518032099216
IDUA	0.0215706653533052	0.956657481276069	0.156275993590755	0.768909201237015	0.0875980000000015	0.684816738812494
NFKBIA	0.0215657156902886	0.956657481276069	-0.347598630280309	0.362583631278157	0.061273000000001	0.814959196808566
VWA3B	0.0215531667420272	0.956657481276069	0.183839773191863	0.718067440349544	-0.141965999999999	0.646581321628762
ARHGAP36	0.0214829381488356	0.956934514930974	-0.317294037330918	0.431286094810965	-0.114632000000000	0.601564043046757
EVI5L	0.0214347102232246	0.957007775431525	0.218583320586533	0.642399486366091	0.00214366666666671	0.994738356997797
LINS	0.0214116058826481	0.957033704740626	0.0767605786390967	0.901870850601638	0.107713333333334	0.613418255499423
UCA1	0.0213228193631721	0.957416215259639	0.0418944865406605	0.951989245458556	0.0836856666666675	0.745703398753986
PLXND1	0.0212961770208268	0.957450351388028	-0.046842784705282	0.944274559688373	-0.0598549999999996	0.821421133422134
WFS1	0.0212905412447855	0.957450351388028	0.110178580282053	0.850412437492421	0.0171160000000004	0.95482028291900
ADAM18	0.0212715446826307	0.957450351388028	0.163198492368250	0.756115153109131	-0.0422883333333334	0.85994094150432
EPB41L1	0.0212419472402848	0.9575081847502	0.535814676104791	0.074032576515667	-0.262088333333333	0.248340336813126
APC2	0.0212272820901634	0.957523899883896	-0.016577646297992	0.981654072941606	-0.0981219999999995	0.7615367624187
CECR5	0.0211680054919266	0.95762358039021	0.0459963965384154	0.945364757579857	-0.139520999999999	0.517547647029412
EEF1A2	0.0211600865797796	0.95762358039021	0.0781896040120776	0.899647854618521	-0.107754666666666	0.657217907037306
DIABLO	0.0211597822577332	0.95762358039021	-0.460289471408713	0.162787294570657	-0.236861333333332	0.256849084629137
XG	0.0211408973812795	0.957630432409999	-0.162143517343642	0.75843356082254	-0.163380333333333	0.396820546738918
EXOSC9	0.0211282212750137	0.957630432409999	-0.160063074399010	0.762902034786947	0.0794583333333334	0.728708142582036
DMBX1	0.0211145533004197	0.957630432409999	0.0710669928598127	0.90935935829916	-0.00161033333333212	0.99628178244892
ZNF623	0.0211126046443683	0.957630432409999	0.521484631762982	0.0885204961957005	0.154196333333334	0.573320977469053
POMT1	0.021093717184717	0.957649403174803	0.0745408874045116	0.904832407812985	0.108193333333334	0.604966458785844
SHANK2-AS3	0.0208811036082038	0.958289669612965	-0.0463998170362825	0.944856324173183	-0.0488376666666657	0.858848223547254
OR1G1	0.0208195239276152	0.95851515344389	-0.0503159029288953	0.940973131693063	-0.0271076666666664	0.92484471634727
MYO16	0.0207686734929618	0.958542777254142	-0.188555659885839	0.707361603786947	-0.0514576666666664	0.824746506521392
DYNC1LI2	0.0207225332673841	0.958743586486025	0.104780611232555	0.86067831050617	0.147263333333336	0.514568255062501
MAGEE2	0.0207020235603302	0.958798781161336	-0.313365453673721	0.438100620341757	-0.184803333333333	0.426518032099216
CACNG1	0.0206339239691125	0.95896614219621	-0.0141744490312716	0.983444353656142	-0.0686006666666664	0.772139923817113
SLC39A5	0.0205781478445007	0.959195806013237	0.200172097273743	0.681552955146541	-0.0529486666666662	0.82616467676523
MFSD12	0.0205089373479783	0.959296505496252	0.196387451388719	0.691445158350448	-0.0750159999999992	0.734968991436953
MRRF	0.0204995325408142	0.959296505496252	0.086837653320092	0.887319922815896	-0.133525333333333	0.573445212873666
IL12RB2	0.0204956306855577	0.959296505496252	0.0806905370569734	0.896621311541396	-0.0892506666666664	0.690213956173797
SRSF6	0.0204525045036461	0.959418741885463	-0.174932717302880	0.733518674801325	-0.0229896666666653	0.930375308310121
MPP5	0.0203918124539091	0.95959732543061	-0.209467165573195	0.664420531246941	0.277011666666667	0.512796060754107
KCNK15	0.0203471176695238	0.959649152366328	-0.129327844056113	0.814413768142626	-0.0529979999999992	0.888181586513563
SH3BP5L	0.0203264568395243	0.959667348770983	0.236148639844330	0.605775058649527	-0.158060666666665	0.446756640099031
FMO6P	0.0203223113096243	0.959667348770983	-0.0747531920130046	0.904466414650249	-0.0417743333333329	0.880844178274306
QPCTL	0.0202976467539144	0.959684738937971	-0.0522875471856653	0.93792141648313	-0.239692999999999	0.202381362671074
CNN3	0.0202692307543239	0.959784693447544	-0.245362969830463	0.585114015659223	0.210643333333335	0.265444694653544
TUBGCP5	0.0201733724927053	0.959845247928216	-0.243615415818512	0.590086964098531	0.153462000000001	0.485767303362274
NCOA3	0.0201723978803067	0.959845247928216	-0.257025351898795	0.5632303733857	0.229619000000001	0.256082730455995
IP6K1	0.0200808586207065	0.960179210445776	-0.144336777347157	0.790566065644438	0.0667073333333343	0.815540776675037
CCRL2	0.0200628718257899	0.960191375553833	0.0880203228018064	0.885848000380851	0.0516305000000007	0.85013576481729
IPMK	0.0199826938474066	0.960314632390177	0.0482148382819572	0.943089870033472	0.243518000000001	0.343572153228906
SLC2A11	0.0199509154845551	0.960314632390177	-0.202107096826772	0.680054545720343	-0.0957329999999984	0.673701601183362
EEFSEC	0.0199489361921483	0.960314632390177	-0.30770027851001	0.452432607043264	0.0760643333333352	0.748579513438275
DCK	0.0197524700324174	0.960956454764939	-0.344801184829351	0.369332936498588	0.0335316666666685	0.899614658619676
LOC339240	0.0197456436222072	0.960956454764939	-0.0267455771639192	0.970207612777188	-0.617304999999999	0.107866708545887
TTLL12	0.0196927234453383	0.96098418711431	0.371505930134357	0.319541753509769	-0.263449333333333	0.159075946945580
CHTOP	0.0196296641933602	0.961090124125493	-0.00868062094148542	0.98946753270812	-0.148207333333333	0.449828629541335
ANKIB1	0.0195474003649101	0.961328395435104	-0.0442763268934549	0.948385499132766	0.0843080000000005	0.710004432586908
P2RX3	0.0194220026536357	0.961646312684535	0.101967641734527	0.864616357134757	-0.00165566666666636	0.99597023247862
PRSS12	0.0193962197106599	0.961689132239948	0.0682961778580744	0.912364243071055	-0.107369666666666	0.712601873916151
IFIT3	0.0193857041204507	0.961689132239948	-0.261396168805941	0.552183982410566	0.206641666666666	0.291481585177291
SMEK2	0.0193819958267576	0.961689132239948	0.207227572908338	0.66888525756447	-0.0900813333333324	0.671582238113164
TNKS	0.0193378674062699	0.961816471140383	0.307992432017538	0.451488724245047	-0.000727666666666552	0.997953056544486
MAGEB3	0.0193086168745699	0.961920800768656	0.111142107248609	0.849127967004266	-0.0822033333333329	0.746473454296225
LINC00028	0.0192587350540895	0.962019449181333	0.194380037524304	0.696294554451457	0.0311856666666669	0.904236796123256
GLUD1	0.0192191644695691	0.962059735692546	-0.232984828569498	0.611711680668619	-0.0408766666666666	0.866051984632626
SLC12A7	0.0191793249046563	0.962223924869981	0.271493932946876	0.527984832801825	-0.097354333333332	0.698340448759983
ARF6	0.0190723835264907	0.962492144001176	0.214224408901596	0.653004496563115	-0.0111973333333326	0.97002499251073
PSIP1	0.0190669912591826	0.962492144001176	-0.00332293939258355	0.994730189431381	0.103910333333334	0.657299463983387
POU1F1	0.0190108681758861	0.962711254550908	0.400879638549491	0.262138265619497	0.00305500000000033	0.992981946108554
PANK4	0.0190051830999894	0.962711254550908	-0.0217884628589666	0.97536722461972	-0.0620209999999995	0.780704356521475
POR	0.0189957833462831	0.962711254550908	0.176490958662876	0.730152680173573	-0.255435333333333	0.18952159968825

NYAP2	0.0189561925723168	0.962787391395818	-0.0809780636586831	0.896361013063453	-0.00205566666666652	0.995114774989911
ZFP82	0.0189172484138996	0.962799477103743	-0.237368075761274	0.603170893739822	0.261955333333334	0.205927392898039
MYL1	0.0189145279966138	0.962799477103743	0.105692039479392	0.85956218633532	0.0220253333333337	0.951767328727707
FFAR3	0.0189138329365284	0.962799477103743	0.248717812037091	0.5770020684641	-0.0829386666666665	0.766915576647578
STAT4	0.0188838110151132	0.962799477103743	-0.362136739943411	0.338093611220237	-0.160211333333333	0.514889931507188
AZU1	0.0188755919643633	0.962799477103743	0.156261581640616	0.768909201237015	-0.218539333333333	0.527465067495661
DDX3X	0.0188672439205042	0.962799477103743	-0.193812122769414	0.697189653813355	-0.133793333333332	0.512138212509218
TMEM132B	0.0188503135108782	0.962799477103743	0.00740915468289155	0.990873555460625	-0.0711689999999997	0.766132481270445
ADAMTS8	0.0187576714640242	0.963200677823382	0.0548783828127509	0.934255557320745	-0.262107999999999	0.175210652385112
OSMR	0.0187026060044558	0.963301804931118	-0.0261825234899408	0.970632644458507	0.0737263333333346	0.730776900973603
PURG	0.0186543801902803	0.963301804931118	0.00527479109090684	0.99251028338798	-0.0820356666666665	0.716057356951225
ITGB6	0.0186251440571646	0.963301804931118	-0.0330721086542497	0.961277493638149	0.0739623333333338	0.744191906125539
CD37	0.018612509019874	0.963301804931118	-0.300379941145978	0.465824202471203	0.0782960000000003	0.787739636244196
PLEK	0.0186028736768723	0.963301804931118	-0.326149432184709	0.409206882509236	-0.222845000000000	0.311439437711061
IRF2BPL	0.0185814114913328	0.963301804931118	-0.158127050035102	0.767373487136803	0.0600603333333342	0.786973845403943
FZD2	0.0185169171678566	0.963301804931118	-0.121134759763373	0.83265693609154	0.061886000000001	0.796708478797823
ENHO	0.0185101731893523	0.963301804931118	0.0286955493710948	0.96782691865619	-0.158316666666666	0.719909781429035
SOX18	0.0184992749042668	0.963301804931118	-0.304098461372142	0.459902255695663	-0.0304806666666663	0.937717709824356
SEMA3F	0.0184659245695056	0.963301804931118	0.107547632351929	0.856850627832179	-0.0833256666666661	0.789149377548591
KCNG3	0.0184532082621488	0.963301804931118	0.0518775847169589	0.938067483409541	-0.162416333333333	0.398554167047557
RPS28	0.0184357560336786	0.963301804931118	0.511992172698553	0.0985590060518004	0.028646666666668	0.911600329626215
SLC18A1	0.0184279396827511	0.963301804931118	0.0870319565900387	0.887055939766881	0.0334470000000003	0.904018282943947
DSCR10	0.0184205657509944	0.963301804931118	-0.153571066788006	0.773392057294058	-0.0551893333333334	0.83822720696374
GPR107	0.018405825426982	0.963301804931118	-0.105706094839764	0.85956218633532	0.106561666666668	0.643688901525294
OLFM3	0.018402046907407	0.963301804931118	-0.336067053350373	0.389476012645306	0.0153356666666670	0.972261709812365
CTCFL	0.0183920132844417	0.963301804931118	-0.314205960522902	0.437675147805387	-0.0208243333333323	0.944539774796585
ZNF343	0.0183870177837913	0.963301804931118	0.0394580058712039	0.953905313456582	0.150759666666667	0.592716584553539
HOXD12	0.0183837532448804	0.963301804931118	0.100294190915036	0.866744687214714	0.0264806666666674	0.955928393298083
LIG4	0.0183614419693364	0.963301804931118	-0.21172695728897	0.658890527313751	0.00428233333333474	0.988942941807152
APLNR	0.0183478970611164	0.963301804931118	-0.203399871262194	0.676646782959283	-0.0345203333333328	0.912081144914596
GUSBP5	0.0183471951458083	0.963301804931118	0.0245474558084282	0.973562415846915	-0.195812666666666	0.309421300309741
CABP1	0.0183438890622108	0.963301804931118	0.124192847056317	0.825382714207684	-0.243412333333333	0.222331780603294
RNASEH1	0.0183212419581784	0.963301804931118	-0.0558483040097003	0.933151248545599	-0.180744666666666	0.343729765439488
NLRX1	0.0183129768890544	0.963301804931118	0.27934691259459	0.510154465721482	0.0389496666666667	0.880131955403697
SLC25A24	0.0182989960289820	0.963301804931118	-0.338183298130537	0.384960636702034	0.431603666666667	0.0894144790631108
COLEC12	0.0182464690309256	0.963501882978123	-0.115723922657010	0.842992311631688	0.357970666666666	0.131549958663763
CLK4	0.0182388340884182	0.963501882978123	0.00665354141995554	0.991309497300929	0.0556781666666666	0.87709801222343
REXO1	0.0181706091608171	0.963776231519503	0.350979294237796	0.356846278147136	-0.0498993333333336	0.830926049084239
C11orf48	0.0181685197129521	0.963776231519503	-0.160560917617908	0.761610418244687	-0.0783286666666666	0.771412700555691
GOLPH3L	0.0181522633662313	0.963806838578465	-0.433867914943775	0.200414575936555	-0.0379493333333325	0.904507944803624
ZNF2	0.0180334684566757	0.964232296192729	-0.0859190626303503	0.888382447758042	0.0638216666666681	0.844766787725698
TMEM206	0.0180183416294206	0.96425650829575	-0.0444127162885869	0.948327980852394	-0.224664333333332	0.277100753732215
FAM64A	0.0179719458982644	0.964456944195958	-0.275561836521919	0.518536711387462	0.247916333333335	0.216469715681292
DBF4B	0.0179128461949669	0.964606843037455	-0.186514737069084	0.711334804690432	-0.00755799999999923	0.98256318768706
BCAR3	0.0178829814598839	0.964653026970045	-0.258062204570632	0.561580373803451	0.0104053333333345	0.974451420920923
CDCA7	0.0178521969482353	0.96475894813130	0.137434201613096	0.802039543563725	0.0833670000000015	0.762118416653633
SPTBN2	0.0178425122154233	0.96475894813130	0.215870363118533	0.648679360344509	-0.201732333333333	0.296952839921866
GTF2B	0.0178147088364652	0.964793497852556	-0.447862899838158	0.178249202877872	-0.00124266666666572	0.99704422037925
IFNA2	0.0177826851072807	0.964854784003152	-0.329301441611949	0.404890442264825	0.00818100000000016	0.985263856374185
TBL1X	0.0177816828574910	0.964854784003152	-0.091977503020104	0.879153312143409	0.327617666666667	0.243957990539057
MAFG	0.0177557460104677	0.964881483056387	-0.0344681583485716	0.959404955370931	0.0170388888888895	0.950884054684253
METTL14	0.0177104648908252	0.965075477145209	-0.263746160287276	0.545144326127723	-0.0365626666666659	0.883114887563545
GREM1	0.0176562039764296	0.965320028648597	-0.132535384132914	0.809866082339655	-0.0327103333333324	0.89637298686676
BCL2L12	0.0176250390786840	0.965434491312516	-0.172888251135485	0.73779222044119	0.0310850000000014	0.904507944803624
COMMD3	0.0174471137653420	0.966070016572635	-0.104215071355722	0.86124543980304	0.0316010000000002	0.90945379489823
DNAAF1	0.0174294654897478	0.966104878002195	-0.443273614930383	0.184589905413952	-0.122782666666667	0.545778786908619
FBRSL1	0.0174192280951338	0.966104878002195	0.296663890325489	0.475941682821927	-0.0571799999999991	0.846906282289456
ZFAND3	0.0173864716121282	0.966128799430076	0.0734061010051486	0.905861613520508	0.00798833333333342	0.983098154995337
C1orf52	0.0173781755378705	0.966128799430076	-0.193050328602427	0.699002603433222	0.00566366666666673	0.987937064305561
TIGD2	0.0173209811614384	0.96629408589371	0.0535851314960918	0.935847524102816	-0.150188666666666	0.518836392563847
TMEM207	0.0172681369982121	0.966466823559212	0.224632848613155	0.632376156419106	-0.0134109999999995	0.965608911524945
CTTN	0.0172251831798965	0.9666040945891	0.0208671622631336	0.976330597778846	-0.0373619999999995	0.887780036680668
FERMT3	0.0172125319562719	0.9666040945891	-0.348667655086408	0.361261311685225	-0.254775333333333	0.246594398491240
POU5F1B	0.0171606572328554	0.9666040945891	0.287298016868493	0.493791835164831	-0.0426730000000007	0.910252591150996
ATG14	0.0171461475235889	0.9666040945891	-0.0671785498958552	0.914700759157169	-0.192601999999998	0.472370507912389
C3orf18	0.0171282341191125	0.9666040945891	0.0533121785139991	0.936629055455007	-0.0887183333333319	0.758098310478085
LACC1	0.0171278585830920	0.9666040945891	-0.0686793936320177	0.912149839732916	-0.0234383333333327	0.949287545570912
RAB33B	0.0171211916379094	0.9666040945891	0.0357399129035438	0.958366440880488	0.0413500000000022	0.886909407509907
TAS2R8	0.0171014933106785	0.96661081214278	0.249973550583274	0.574968685145169	0.128630666666667	0.5293518047673
CLEC2D	0.0170252498978235	0.966735763564638	-0.135772500007423	0.806188297553807	0.378824666666667	0.0725770605949615
PARPBP	0.0169991014324239	0.966820467904382	-0.062868756471269	0.922206526818357	0.191145666666667	0.399012880214421
IPO11	0.0169671418692665	0.966878123303039	-0.0384453810034624	0.954545200586067	0.121744000000001	0.626750668682093
ENTPD1	0.0169520465440726	0.966901975595207	-0.302066481062801	0.462229407853213	-0.237069000000000	0.446756640099031
OR2B2	0.0168683341823050	0.966911154004547	-0.0734236544910903	0.905838142287595	0.0619986666666668	0.777406710539424
MFSD1	0.0168669648441433	0.966911154004547	-0.333585518774389	0.396821740334178	0.221568333333334	0.259772013363267
NKX6-3	0.0167757705741002	0.966911154004547	0.178755509152581	0.726057281492174	-0.0880456666666657	0.783918962405407
BARX2	0.0167575879956002	0.966911154004547	0.401606933631101	0.261512734265668	-0.063802	0.817592590695949
CTC1	0.0167184191408389	0.966911154004547	-0.235036666435175	0.60755186112057	0.0099243333333346	0.975878170822231
CHRNB4	0.0166945607049203	0.966911154004547	0.250024842702849	0.574968685145169	-0.141684666666666	0.637426515050706
PANX2	0.0166819888101469	0.966911154004547	0.17997472514601	0.723716289903531	-0.0176913333333335	0.95784037718285

TM2D3	0.0166362256052540	0.966911154004547	-0.0783910642422666	0.899281198071059	0.166622000000001	0.575378149225683
IFNB1	0.0166301324952872	0.966911154004547	0.167382129250485	0.749061933609388	-0.0147249999999999	0.96797990593595
ACAP2	0.0166176204441584	0.966911154004547	-0.0553976517213443	0.933409139126562	0.0217183333333338	0.937591579324882
ZNF561	0.0165849859244836	0.966911154004547	0.122235844012777	0.829569971668386	0.171892666666667	0.409994360794613
RNF183	0.0165641678721387	0.966911154004547	-0.083381084108844	0.892833228561937	-0.0766286666666663	0.723993424337833
XAF1	0.0165571741735981	0.966911154004547	-0.118505231712966	0.837761659039664	-0.0259870000000002	0.926246672706072
ADCY4	0.0165489099192609	0.966911154004547	-0.0926449201779568	0.877878897534788	-0.126718666666666	0.586777560503894
PDIA4	0.0165428101758825	0.966911154004547	-0.297927383950794	0.472546858700416	-0.376286666666666	0.0809230308622037
ANKRD45	0.0164699444546104	0.966989126933407	0.00406335597020922	0.994485841304070	-0.0803349999999996	0.769582581088788
SGTA	0.0164190454902764	0.967169906070395	0.116375343698537	0.84214534364593	-0.0710733333333318	0.766497698403098
GPR111	0.0163120519796063	0.967238376233878	-0.0349715864272514	0.95915073655001	-3.69999999993521e-0	0.999822702792133
GPR160	0.0162762187455809	0.967238376233878	0.0335199151312734	0.960782399177614	-0.147883666666666	0.589703432017128
CENPA	0.0162733303110900	0.967238376233878	-0.171795989499065	0.739221495717459	0.0417836666666669	0.872930585008323
GPX4	0.0162455324626422	0.967238376233878	0.0169778683610876	0.98105020869911	0.0272466666666682	0.914739856620534
FRAS1	0.0161675685652349	0.967489388985278	0.181891150729581	0.720096350499662	-0.139606666666666	0.766132481270445
CENPQ	0.016158141993576	0.967489388985278	-0.150503358887043	0.77904503981756	0.230152666666667	0.269739446716269
PAN2	0.0161462371354243	0.967489388985278	-0.0483659840482177	0.942820948997969	0.107505000000001	0.595928600780435
PDPR	0.0161419648935344	0.967489388985278	0.172824017805472	0.737903985814506	-0.239853666666666	0.237531127392486
OR5L2	0.0160999621754066	0.967603071981992	-0.119709879210555	0.835595913936405	-0.0783073333333332	0.733650645007746
CCDC115	0.0160695849665322	0.967651628550873	0.119797510438865	0.8354985445494	-0.0725773333333325	0.809505390888975
ALX4	0.0159945206769791	0.967870136965796	-0.0238761502380851	0.97479225206569	-0.302140666666666	0.175210652385112
ZNF493	0.0159317588117410	0.968005759837965	-0.257204528852214	0.563116080558022	0.488276333333334	0.0292750036633431
SLC1A6	0.0159292464366796	0.968005759837965	-0.0604835902912066	0.925565513580637	-0.163295333333333	0.484125153753832
DBF4	0.0159086387680399	0.968005759837965	0.246041031335575	0.582912574644681	0.102744000000001	0.623123457579993
NPB	0.015871260428993	0.968049528408183	0.137156717328626	0.802743708223464	-0.124161333333332	0.76368723681351
NEK6	0.0157687413710561	0.968173337120257	-0.0684956130686967	0.912149839732916	0.786174666666668	0.00784994780223591
HABP4	0.015748627238417	0.968173337120257	0.28249118707218	0.503412540505745	-0.105235666666665	0.693664816978074
NKD2	0.0156877102256569	0.968391401492085	0.226479499738625	0.62700289306617	-0.227015333333332	0.41785973815832
IFI35	0.0155595240205767	0.968622401356479	-0.294989966180027	0.478655242507888	0.267100333333335	0.281687717156474
PPP2R2B	0.0155349517145587	0.96863833924918	-0.128230309942319	0.816855571576772	-0.616718666666666	0.0870358983821552
KIAA1614	0.0154715486283649	0.968806084934982	0.235931234171975	0.606121203124993	-0.0380563333333324	0.909196271455697
DHRS9	0.0154599121487103	0.968806084934982	-0.161709343938717	0.758955920988521	-0.0589743333333332	0.798736789384715
ERP44	0.0153534036208314	0.969024902080884	0.198688352481353	0.685703984501304	0.0239200000000004	0.926246672706072
PAPSS1	0.0153354379075196	0.969024902080884	-0.529201781732362	0.0799297163112453	0.0149530000000004	0.959610850206085
CDK4	0.0152906497568714	0.969153732817218	0.0908004416793246	0.881323965029954	-0.219533666666664	0.270000135172916
LRRC28	0.0152238137748754	0.969405835575625	-0.0849658110564571	0.890886878560183	-0.136125333333333	0.584153892885306
FAM84B	0.0152237894791720	0.969405835575625	0.0724274129371556	0.907556175729082	-0.0491956666666659	0.846213226883859
ADAMTS14	0.0151886285352068	0.969420132377438	0.0390172792942164	0.954046704090622	-0.292103	0.307774837877941
ARRDC4	0.0151656255580796	0.969487910609013	0.0809521920675788	0.896361013063453	0.234445666666667	0.208325255115003
HMHA1	0.0151103671838504	0.969548451668253	-0.280184230112195	0.508074118826263	-0.0837613333333321	0.822595110194286
BRD2	0.0151089963555810	0.969548451668253	0.0333280665961421	0.960784250392482	0.0545253333333346	0.809462787517012
PPP4C	0.0150984277887983	0.969548451668253	-0.195593908961776	0.693715509921929	0.00935833333333365	0.978504228748436
MX1	0.0150758888638823	0.969548451668253	-0.118422826334250	0.837937752076601	-0.0262053333333334	0.92469922248949
TSC2	0.0150695253326414	0.969548451668253	0.0648721426848625	0.918590305362052	-0.353742999999999	0.0749561445182193
TSSK2	0.0150678417285865	0.969548451668253	0.116673380159603	0.841809730226979	-0.140828833333332	0.516217427447249
FAM63A	0.0149834901799725	0.969837296722518	-0.0316546313642279	0.963712086545628	0.0643390000000008	0.777585466017059
TF	0.0147959266286084	0.97056188399748	-0.0135753975943149	0.98412883278237	-0.000389666666666634	0.998643269342783
METRNL	0.014783733743551	0.97056188399748	-0.170885107076264	0.740779344877952	-0.0427906666666661	0.872930585008323
CRMP1	0.0147061016264231	0.970764859944607	-0.254791074983596	0.566909791913503	0.00139433333333372	0.997144862160692
CABP4	0.0146578869626285	0.970764859944607	0.218003550041172	0.643858306038742	-0.111823000000000	0.754439827606006
TEX33	0.0146501901542671	0.970764859944607	0.106512434365133	0.858072018696671	-0.0189619999999993	0.9681321732097
ZNF354A	0.0146323799549587	0.970764859944607	-0.302248909799832	0.462229407853213	0.247410333333334	0.358847935929216
CLASRP	0.0144449418013925	0.97155585986998	0.000301483317108849	0.999739851959534	-0.0649703333333326	0.776013100927836
KRTAP9-8	0.0144161066816296	0.97158352363764	-0.154433568299919	0.771310656070265	-0.0494166666666654	0.830987625642722
CYP20A1	0.0143764440914327	0.97158514566387	-0.0652862623184501	0.918295693698803	0.272321333333334	0.191847341402838
GRIK3	0.0143540707047111	0.971649180268666	0.0413671122742041	0.952098348256047	-0.170543999999999	0.459936388684371
PERP	0.0143303612339187	0.971660674648208	0.101852149779581	0.864616357134757	-0.0297329999999989	0.909450927730061
RSPH3	0.0143297172548692	0.971660674648208	0.154439233949754	0.771310656070265	0.125167333333334	0.663776173049665
CAP2	0.0143077095443696	0.971664876789717	-0.325559225684688	0.409206882509236	0.0925470000000001	0.6846855637534
BRIX1	0.0142924029857269	0.971689510417472	0.251234956158133	0.572833531479119	0.00478866666666812	0.988133186272453
TIMP4	0.0142307769967338	0.97185672327181	0.312721871137667	0.439221508111214	-0.0269826666666667	0.948469012194168
ACHE	0.0142297337564174	0.97185672327181	0.193159022158282	0.698832386916027	0.0241146666666668	0.946623914607177
ARMC8	0.0141953822006721	0.971874490916315	0.136702667706940	0.804018414279872	0.299104000000001	0.195651414003911
KIAA1919	0.0141829936325481	0.971874490916315	0.201202739330169	0.680054545720343	0.272020666666668	0.219457597945103
SMO	0.0141473126455812	0.971993462228412	0.145820910971756	0.787804113309913	-0.00245233333333176	0.994216250363693
DPEP1	0.0141272863654301	0.972002842836511	0.344850448625008	0.369280599599459	-0.200175333333333	0.349671738808994
FOXI1	0.0139588525462406	0.97260703889587	0.153890967210396	0.77284838227453	-0.118096000000000	0.626750668682093
EYA4	0.0139121837774009	0.97260703889587	-0.219353422804978	0.641314646479258	-0.0409609999999996	0.915056801542305
ADGB	0.0139115995479191	0.97260703889587	-0.225418230076289	0.62993424888296	0.0708166666666671	0.76564992393702
SLC8A2	0.0139113630610279	0.97260703889587	0.0241110727218507	0.974276665384912	-0.0352373333333333	0.908443677591815
TYSND1	0.0139098677203194	0.97260703889587	0.370681604808221	0.320399684386315	-0.0869009999999999	0.705010402761235
IGF2BP3	0.0138363612415011	0.972844867873652	-0.316745505446749	0.432098412327371	0.0442230000000005	0.868203945159707
ACOX3	0.0138066859035896	0.972901089876424	-0.276089048578874	0.517549833373363	0.0464233333333341	0.84547275205075
BVES	0.0138025641177423	0.972901089876424	-0.342690681003365	0.374903556474195	-0.00288433333333283	0.99411609755275
C2orf49	0.0137195486873548	0.97330246369763	-0.109245271577461	0.85256212710303	0.050167666666667	0.843945436588033
OR12D3	0.0134148457182571	0.974277617942146	0.0620988168081139	0.923034515346352	-0.0756658333333331	0.786670898149409
ZKSCAN4	0.0134134254937084	0.974277617942146	0.123120724094553	0.827789978865629	0.0102653333333346	0.976784701964987
FAT4	0.0133847430199090	0.974315790605524	-0.399848935940446	0.264509265838310	0.238253000000001	0.217583016023457
TMEM50B	0.0133710831080846	0.974331085166785	-0.0932101151591313	0.876951966086083	-0.141256999999999	0.550660741372463
AGER	0.0133147810164034	0.974434837447505	0.106989908179364	0.857671866148626	-0.109975222222221	0.619628921356451

DUSP18	0.0133045918211741	0.974434837447505	-0.346206397760421	0.366023660299226	0.0451900000000012	0.887363245747406
LAS1L	0.0132663190428052	0.974434837447505	-0.379556966648104	0.302115584913204	-0.0530009999999985	0.833894599883106
LCMT2	0.0132554221524785	0.974434837447505	-0.224322896984345	0.632829553438263	0.128664000000001	0.537865784181774
HERC5	0.0132542315549342	0.974434837447505	-0.345293307178000	0.36822404913581	0.235686333333334	0.215722751602022
MPPED2	0.0132298419837628	0.974509761293486	0.187433003642519	0.709286896761914	-0.0305293333333326	0.956567100065073
RGS11	0.0131779409877283	0.9745394613788	0.067387660795668	0.914376605659715	-0.00523133333333267	0.990909577872758
C7orf41	0.0131734508510404	0.9745394613788	-0.281563933589206	0.506288700696016	-0.0714643333333333	0.786970336786627
NAA60	0.0131605841890635	0.9745394613788	0.139837046229148	0.79795161794304	-0.312992999999999	0.128384813166245
MTO1	0.0131590317892277	0.9745394613788	0.00497776868660401	0.992802943754543	0.0462880000000009	0.863815665454511
SCML1	0.0131090774025699	0.974756402033941	0.156443249374921	0.768909201237015	0.389649333333334	0.0725137013180337
CDC42EP1	0.0130661465926162	0.974920054026426	0.102817665679649	0.86257932725575	0.0332796666666674	0.922697964464038
FBXO17	0.0130333498648213	0.974934590884107	0.231181300487617	0.615929441239857	0.0428761666666677	0.867851765975147
TMEM59L	0.0129171011117301	0.97525774892429	0.250189074338550	0.574321063440325	-0.0425049999999996	0.91143191097679
NARF	0.01291499318509	0.97525774892429	0.213670371883950	0.653708954135733	-0.128602999999999	0.593008996011892
BET1L	0.0128856960328717	0.975330276337274	-0.112765851763433	0.846997525752467	-0.0198103333333323	0.946899561823473
UPB1	0.0127961725307368	0.975766784142034	-0.0185336654073592	0.97902968742576	0.0741220000000008	0.786118160890663
FBXO7	0.0127248735518487	0.975875218744788	0.0161053907269905	0.982057247916055	0.0190840000000014	0.945904449997854
KRT83	0.0126831945194652	0.975875218744788	0.110874801361789	0.849357727620213	-0.120446333333333	0.622085027921794
ZNF222	0.0126790993011724	0.975875218744788	-0.221912314469408	0.636778528562165	0.146668000000001	0.49679816436215
PCDH11X	0.0126705321274705	0.975875218744788	0.194097069252526	0.696755619051339	-0.244554333333332	0.359771290730514
LIMCH1	0.0125725310578429	0.976220556917915	0.00813857694109498	0.990248011892148	0.730159333333334	0.0205866975799116
CRNKL1	0.0125432636090244	0.976231008201011	-0.211244123378224	0.660013379937732	-0.0505829999999978	0.834148450369096
SATB2-AS1	0.0125286387313007	0.976231008201011	-0.0983234945292657	0.86848759013952	-0.125998333333333	0.619996256183009
RRP8	0.0125176506803756	0.976231008201011	-0.152728505647323	0.775138534286213	0.211409666666668	0.443378989641463
NEUROG2	0.0125049748943863	0.976231008201011	-0.0071297825032634	0.991086607331007	0.00110633333333352	0.997134925912045
C9orf114	0.0124736909935982	0.976343940398358	0.101180509271033	0.866005093242444	-0.219829333333333	0.287847267933509
NDUFS3	0.0124492974124011	0.97641864026956	-0.195996482972589	0.692325064566574	-0.0175236666666662	0.951767328727707
ZNF76	0.0124325206683379	0.97645108359503	0.227915573137344	0.624359290301343	0.135320666666667	0.503227591466303
RGR	0.0124105191938077	0.976451891142746	0.128957536011313	0.815386814587013	-0.0541786666666666	0.833336742497873
CCND3	0.0122899057894785	0.976938939929853	0.226942101674662	0.625822145497039	-0.0424893333333324	0.885774077100386
NEFH	0.0122430026511689	0.977077773065996	-0.449560129786414	0.177460282309099	-0.0842483333333333	0.791240721091965
PPP2R1A	0.0122196816364383	0.977085830695953	0.188234432073637	0.70758322613272	-0.0247233333333319	0.927795934389862
TRIM26	0.0121398078756397	0.977325761716884	-0.137647507045553	0.801934376886443	-0.050432833333332	0.862156400976549
C1orf61	0.0121326622455090	0.977325761716884	0.016125579669009	0.982057247916055	-0.00332066666666583	0.991625835822726
MYSM1	0.0120554664374291	0.97753519558919	-0.138453661346081	0.799829206249938	0.0521040000000008	0.840953774907927
SLC25A43	0.0119832620530618	0.977719025053275	-0.0385964566959676	0.954545200586067	0.00199000000000128	0.995942523459928
PSD2	0.0119787931029583	0.977719025053275	0.128121885167928	0.817166760137831	0.0375726666666672	0.91143191097679
C12orf43	0.0119741998052879	0.977719025053275	0.041790947900548	0.951989245458556	-0.115173333333333	0.62405411820911
FAM110A	0.0119214378404211	0.97778883789978	-0.209981067078472	0.663167662486675	-0.183989999999999	0.376915404087931
CSMD1	0.0118929617242127	0.97778883789978	-0.183211973532830	0.718067440349544	-0.0634249999999994	0.824700707442131
HAP1	0.0118808971479964	0.97778883789978	0.117587931713915	0.839436826243045	-0.134530333333333	0.60216511573053
GP9	0.0118767406659593	0.97778883789978	-0.0426090815680741	0.951071128215665	-0.155843999999999	0.507962983295522
KCNH3	0.0118454585883888	0.97788060289885	0.0635154248968526	0.920855211745173	-0.203078333333332	0.408086593806833
VN1R4	0.0118048111827348	0.97788060289885	-0.0849197458524847	0.891068135506467	0.194165000000001	0.4254947046787
ARHGAP28	0.0117858444249971	0.97788060289885	-0.0532621092924758	0.936629055455007	-0.0466626666666669	0.84761697289985
ZNF486	0.0117348637863656	0.97788060289885	-0.243640585269656	0.590086964098531	0.299456000000001	0.124096550131011
GBA2	0.0117340933743268	0.97788060289885	-0.101656500123928	0.865262342519817	-0.285323999999999	0.233528575266801
LRIG1	0.0117262272648995	0.97788060289885	0.0451694747170882	0.946577404106596	-0.421012666666667	0.0446032270650573
FAM13A	0.0116374968015642	0.978249041368744	0.119458351541775	0.836013196419542	0.312738333333335	0.0993494729220131
PPP1R37	0.0116236099004937	0.978249041368744	0.192489781286711	0.700450711372106	-0.296341333333333	0.134354816258782
IGDCC3	0.0116093863299447	0.978249041368744	0.166638317413134	0.750457342031334	-0.301252333333333	0.336597634514043
SUMO2	0.0116049420131969	0.978249041368744	0.272610012433480	0.526081777027135	0.022123833333333	0.941559580750404
TBC1D1	0.0115917274094968	0.978261607999426	-0.061658914683793	0.923246185283376	0.783779333333334	0.0263910835019345
CDH5	0.0115783605569029	0.978275015910494	-0.192126808690909	0.700820605802046	-0.0244616666666664	0.924674647382585
EPHB3	0.0115538808095752	0.978313491558535	0.393290497457818	0.277262500114017	0.0593253333333333	0.868109658266916
IFT57	0.0115089676579086	0.978356190327824	0.0719403266659985	0.90837606217316	0.398152000000001	0.0533308741028597
ERI3	0.0114364705130884	0.9784544932837	-0.241423241458111	0.595802238885157	0.000419333333333441	0.998351142601927
RTF1	0.0114169964716806	0.978501654994846	0.0471371872797731	0.944081286115546	-0.105251999999999	0.604179762453377
ABCC5	0.0113345615236475	0.978836398637934	0.0556144890336955	0.933409139126562	-0.378167999999999	0.0714169560354366
SYF2	0.0112573136125866	0.979081859514567	-0.149168226742607	0.782612598081927	0.066322	0.766132481270445
RPUSD2	0.0111247792131522	0.979477734779718	-0.283002591873563	0.502129505146473	-0.0966076666666652	0.780732284998362
HCN2	0.0110861737011802	0.979477734779718	0.48812868989678	0.128047898243798	0.0439560000000002	0.883189085233712
SRRD	0.0110835999166345	0.979477734779718	-0.143217021087444	0.79294618815764	0.0972576666666677	0.721039266334933
ZNF585B	0.0110714832971413	0.979477734779718	-0.194109982737351	0.696755619051339	0.210100666666668	0.278205568286522
CPNE3	0.0110652031774655	0.979477734779718	0.120553611064693	0.833896466602933	0.303328666666668	0.119723060761242
PRIM2	0.0110338479519319	0.979494661661714	-0.102856985582850	0.86257932725575	0.268289666666667	0.180114381586694
SPTA1	0.0109337173123703	0.979840940183596	0.331884739499343	0.399068746068573	-0.0189359999999996	0.946392308016584
TANK	0.0109198002590176	0.979856908790827	0.0120963322420850	0.985769556168802	0.180734333333334	0.36569302637233
ANKMY1	0.0108815770416415	0.979877137508027	0.451347680722975	0.176663460992485	-0.127078666666666	0.661812056571718
SUPT3H	0.0108491506116782	0.979944860766812	-0.190496064872812	0.703920416197528	0.239612666666667	0.302732927341247
GALNT8	0.0108370767555996	0.979951019359991	-0.112344152294082	0.847454473507881	-0.0253236666666665	0.931055338143178
IGLON5	0.0108054765109014	0.980064978249205	-0.0515119935849495	0.938728344711776	0.187838666666668	0.477467429367264
ABCG8	0.0107815391622270	0.980136622425619	-0.0111353587524164	0.98774001793194	0.0712973333333337	0.773270959529093
SETMAR	0.0106986931002105	0.980412415020454	-0.278899839832371	0.511025440770733	-0.0424853333333328	0.873398028647672
VDAC2	0.0106616807716068	0.980492804016766	0.0123038129796670	0.9854287825221	0.0338061666666684	0.895093529187836
KAL1	0.0106538305375932	0.980492804016766	-0.317040636115578	0.431780281826320	0.0345803333333341	0.903864363916236
METTL13	0.0106402747871288	0.980492804016766	-0.351069131198138	0.356692743945773	-0.0426083333333326	0.857015626494513
FEZ1	0.0106286062516168	0.980496694796203	-0.403234336633627	0.258453301425503	0.115182333333334	0.662751767068109
ALDH1A3	0.0106102143415613	0.980498659430435	-0.246345149775903	0.582725878221343	0.365159000000001	0.390966211084762
UBE2W	0.0105915816880600	0.980498659430435	0.0141803856340029	0.983444353656142	-0.227578666666665	0.248900866475991

TRIP4	0.0105254818684736	0.980519354778903	-0.148773600562939	0.783041584899534	0.281647666666667	0.155294715110343
NAA11	0.0105135903771830	0.980519354778903	-0.280070826923638	0.508074118826263	0.133289000000001	0.53157737237227
MAST3	0.0105039024779326	0.980519354778903	0.178770659431719	0.726057281492174	-0.0535456666666657	0.821421133422134
RPS4Y1	0.0104837912937642	0.980546103975963	0.0761866836050604	0.903296922694955	-0.127330000000000	0.612618944235881
ADAMTS7	0.0104771253655063	0.980546103975963	0.253497661438355	0.567705016149998	0.0437793333333341	0.866729620800214
SUCLG2	0.0104532186322379	0.980617472323033	-0.165014133528668	0.753008953828571	0.0269213333333338	0.933412982728621
SLC25A11	0.0104269269803332	0.980678041197686	-0.0653157601488979	0.918295693698803	-0.0128333333333318	0.965329851466378
RFXAP	0.0104203047827836	0.980678041197686	-0.411194241313624	0.243981291680416	-0.127108	0.562399033626288
RPS25	0.0103785594430277	0.980795288009253	0.12492847767233	0.824457843812434	-0.0856853333333326	0.697090143987229
DMAP1	0.0103324073353898	0.980981246160538	0.309279648658475	0.448350131365446	-0.141369999999999	0.512796060754107
PKD2	0.0103064674099474	0.980995092339144	-0.153331389289371	0.773958313698473	0.322203333333334	0.136886121705214
PIRT	0.0102969930963622	0.980995092339144	0.173579050299287	0.735851467282891	-0.109247000000000	0.628477077807168
C6orf170	0.0102822791037597	0.98099580442042	0.0419438501406138	0.951989245458556	0.333278	0.0837916958855762
HES6	0.0101711097699955	0.981297612709604	0.304582773087965	0.459296694704882	-0.134380000000000	0.5530742296054
PRKACA	0.0101528701813231	0.981297612709604	0.274162662092854	0.521169068933632	-0.247675999999998	0.275779229553450
DMRTA2	0.0101323718757313	0.981297612709604	0.0895370695800246	0.883258404969667	-0.111123333333333	0.63895968420953
OR51E1	0.0101246076755326	0.981297612709604	-0.192347355788741	0.700450711372106	-0.0982079999999994	0.712601873916151
RAD54L2	0.0100228602910997	0.981604721330235	-0.116909340164502	0.841512197646315	-0.276924333333333	0.264889721029722
ANKRD18B	0.0100109520950262	0.981604721330235	-0.0497040595119674	0.941458038227918	0.189960000000000	0.537186705207113
ZNF212	0.0100087273309333	0.981604721330235	0.079533930211304	0.89819077139711	-0.118199000000000	0.55709893589146
CSF3	0.00997604107425403	0.981614930699881	0.130495500198406	0.812271254601992	-0.135109666666666	0.532358187914645
PCDHB11	0.00986435917485603	0.981937423943774	-0.00073075800892078	0.998964123154806	0.111152333333334	0.629064753412306
TUBA3FP	0.00986258894140758	0.981937423943774	0.0387631180050608	0.954263089081373	-0.0266623333333332	0.922463263763
SYT1	0.00982380835265476	0.981981070729278	-0.242787538103803	0.59251299743287	0.567810666666666	0.0782543184284344
OR5P2	0.00974983948273812	0.982135063379431	-0.0447799466041069	0.947639907523753	0.0289713333333337	0.931708923663607
ELFN2	0.00966566256091343	0.982537812346308	0.436915499559015	0.195288910691951	0.00969433333333377	0.974895360062947
CPA6	0.00954295246325005	0.982971202198771	0.148544350717998	0.783532632807022	0.00202333333333340	0.994956689715437
C14orf80	0.00950206376463244	0.983113707988696	0.0897514117797753	0.88318195439136	-0.179201666666667	0.406211996897819
SLC35E3	0.00949507282295915	0.983113707988696	0.152219029825594	0.775863712880333	0.155609166666668	0.427283471646461
C3orf55	0.00944222418868623	0.983171770848564	-0.343962003737182	0.371949479280594	-0.115339666666666	0.590841338570121
CORO1A	0.00944056560809193	0.983171770848564	-0.404393625372867	0.255554450438873	-0.329757999999999	0.112920657048548
TGFBR2	0.00940037376260906	0.983322526775671	-0.144571531920449	0.790194087288815	-0.0395886666666656	0.881948770503174
CRYGB	0.00939117355222838	0.983322526775671	0.0225274953885087	0.97536722461972	-0.0252039999999998	0.93535961048518
APCDD1L	0.00936607464820853	0.983352134400071	0.325596567944101	0.409206882509236	-0.101406999999999	0.738948205923422
PLA2G6	0.00932701732082075	0.983373160156804	0.227165466434307	0.62581495163125	-0.0597886666666663	0.85013576481729
LOC100506127	0.00927649332918489	0.983456659715844	-0.124228818615419	0.825382714207684	-0.0381979999999998	0.904201124848034
KCNK4	0.00919565679485873	0.983589224469531	0.201940069016661	0.680054545720343	-0.0943419999999993	0.780512234657452
MRPL50	0.0091805442144722	0.983589224469531	0.06165842541815	0.923246185283376	0.0899120000000017	0.671766969198954
CFL1	0.0091512769958395	0.983589224469531	-0.275139155312510	0.51990180188597	0.114476666666668	0.572207354314478
SDR9C7	0.0090868980837038	0.983589224469531	0.105959222979318	0.859008989305801	-0.0167486666666664	0.959218131095776
DOK3	0.00903548905699753	0.983589224469531	-0.255641719801682	0.565656702091657	-0.242067000000000	0.377817332451136
ERO1LB	0.00903267334027523	0.983589224469531	0.0732130053334082	0.906190940526261	-0.114811666666665	0.567291668977137
ZNF511	0.00901454102464704	0.983589224469531	0.304820926694966	0.458650414345775	0.0774040000000005	0.724802692763867
OR2J3	0.00901325096034837	0.983589224469531	-0.158799623883418	0.765730464375784	0.0102406666666672	0.974451420920923
TBKBP1	0.00901293474044312	0.983589224469531	0.168259844270777	0.747723416813184	-0.0154869999999999	0.965329851466378
SETD8	0.00898377763307558	0.98368894332925	-0.0397286639021248	0.953647040142798	0.169478000000002	0.451227267294744
HTT	0.00889822655534258	0.984066087511655	-0.342797636481241	0.374882411235977	0.0386236666666664	0.901019845009176
PRSS1	0.00885266078224996	0.984066087511655	0.00905334089655507	0.98909166696601	-0.0246606666666663	0.929594956567283
CDY1	0.0088503593661954	0.984066087511655	0.234770457258989	0.607957283325678	-0.0337448333333331	0.892414900592065
PRR19	0.00882434366741585	0.984066087511655	0.196978276204910	0.690061983084583	-0.0447666666666663	0.874896733239698
C10orf82	0.00881884301142904	0.984066087511655	0.0817216253286838	0.89548747312664	-0.321280666666666	0.112438115304648
CR1L	0.00879811794304495	0.984066087511655	-0.0549149628751934	0.934255557320745	-0.0262439999999998	0.925845244747948
SAE1	0.00878230877353273	0.984066087511655	-0.097688307702306	0.86925341204377	-0.145919999999998	0.472363982227685
FXYD6	0.00867622648643925	0.984350866380494	-0.350325148194188	0.358738646007386	0.353572666666668	0.162461129851414
EIF3H	0.00864171052066623	0.984479835987488	0.217972137582039	0.643916980501688	0.0754130000000022	0.728909820842191
UCMA	0.00858340133682351	0.984618622122584	0.00441008627437386	0.99405344216434	-0.0749649999999996	0.794486775271372
CCR8	0.00849682572556112	0.984750443927275	-0.155597044030916	0.769839195080653	0.0655400000000004	0.819757926662461
CTSL2	0.00849518016958734	0.984750443927275	-0.0789108217170446	0.899154242391172	0.314431666666668	0.114299940362607
OR10J1	0.00849186981520834	0.984750443927275	0.0672209488045101	0.914700759157169	0.0363333333333344	0.890073383340154
PRKG2	0.00848390783880888	0.984750443927275	0.264526174870867	0.543735458258403	0.101585666666667	0.681982823818184
ARHGEF40	0.00848234886178432	0.984750443927275	0.308354946817994	0.450974903594884	0.000655666666666765	0.998351142601927
FAM163A	0.0084177789784947	0.985011876976705	-0.0479180105933848	0.94314236584312	0.0109713333333340	0.974895360062947
CD8A	0.00838445155000187	0.985015329849845	-0.447469596433431	0.178249202877872	-0.102926000000000	0.719839702715467
BCAP29	0.00833693496373246	0.985015329849845	0.373555132927515	0.315811026719894	0.0783826666666684	0.738030576833288
MYLPF	0.00828004255754501	0.985015329849845	0.171823520835101	0.739167054766667	-0.0462299999999998	0.888151072264636
SLC22A18	0.00821523879526933	0.985015329849845	-0.130209479765015	0.812271254601992	0.0761169999999995	0.787739636244196
TTC14	0.0082074196026847	0.985015329849845	-0.13133474583118	0.811328589649422	0.104687666666667	0.631192833987568
ANKRD30A	0.00820407404042378	0.985015329849845	-0.152127377743710	0.775920338550028	-0.300498666666666	0.120319574336004
DRAM1	0.00820308026489343	0.985015329849845	-0.126142928377811	0.822164329801713	0.0610803333333334	0.872930585008323
BCL7A	0.00820149185727179	0.985015329849845	0.239845524976492	0.598134271398487	-0.238847666666666	0.275951503166653
C17orf64	0.00819080861099327	0.985015329849845	0.128829914767366	0.815407702581246	0.0464026666666667	0.866601971778042
NR2E3	0.00817257048205331	0.985015329849845	0.227039357338401	0.62581495163125	-0.0435253333333329	0.856854911614454
REG1B	0.00803068932091597	0.985549546480488	0.165741613049587	0.751952150889158	0.0139983333333333	0.962944481336953
GDPD3	0.0080237513169986	0.985549546480488	0.182803759048750	0.718323250150523	-0.192961666666666	0.386320548003750
BAD	0.00801202400734154	0.985549546480488	-0.0800292458337285	0.897577172229156	0.166394000000001	0.396454946335441
GSTTP1	0.0078303429165043	0.98615051490859	-0.147110736139457	0.785734908309155	-0.0457111111111104	0.847083064840772
ZNF223	0.00782530491993903	0.98615051490859	-0.105336644930306	0.86011776595122	0.476942333333335	0.0689724571534153
AIMP2	0.00781934810755829	0.98615051490859	0.166921365935044	0.750076857136007	-0.238459666666667	0.292543465906489
MRPS21	0.00780775965683794	0.986153654692053	-0.00364747299211986	0.994588964700772	-0.0188569999999986	0.949314722489396
COMMD1	0.00773672875341213	0.986328445818055	-0.0985265613609111	0.868278775296491	-0.112464666666666	0.575589336285762

EXTL2	0.00763536351991323	0.986517599770886	0.0960274409316657	0.872311810447594	-0.0467213333333327	0.861165447055876
MAP3K8	0.00763103755346466	0.986517599770886	-0.330850829446249	0.401780307922136	0.0404590000000000	0.935261323444314
ABCF1	0.00758538199939993	0.986706931282515	-0.0586562244249976	0.928586335857223	-0.165940666666665	0.399848490147388
EFNB2	0.0075510980647811	0.986726444902736	-0.291371132667459	0.486757482084406	0.203287666666667	0.334612338353501
TSPO	0.00754695889331456	0.986726444902736	-0.0461128138850175	0.945364757579857	-0.0805769999999996	0.742124582656713
PAM	0.00753773249648056	0.986726444902736	-0.323827727850903	0.414201624246883	-0.345579999999999	0.255595078844894
RPS29	0.00747312468890887	0.98695904788482	0.160424819688755	0.762022428895144	0.032768666666667	0.91894315004511
TRIM59	0.007421513934353	0.987067189056492	0.167625952163743	0.748585550487178	0.210201666666667	0.417718684231587
SLC25A1	0.00740857375527886	0.987070835396966	0.21881186116825	0.642137448592891	0.236473666666668	0.241273257416922
ZNRF1	0.00735690471184412	0.987135963060185	-0.236601001768996	0.604679221319698	0.0608770000000014	0.789105818888855
SMC1B	0.00734741986922068	0.987135963060185	-0.0442658459840482	0.948385499132766	0.099845	0.655063476133482
COL11A2	0.00731941649669768	0.987135963060185	0.0167004675611576	0.981391739387265	-0.199303111111110	0.49912108040986
TXNRD2	0.00730983308477442	0.987135963060185	-0.0381495017485797	0.95468180561467	-0.0179989999999986	0.950790590129332
GINS4	0.00730846245916175	0.987135963060185	0.0702098954608612	0.910394157189308	-0.107354666666666	0.700527074531695
TMEM125	0.00726308976114684	0.987323518789716	0.0974333896791012	0.869861570992203	-0.148529	0.566282142073858
SNRPG	0.00723614801352325	0.987350220965653	0.0602057546462872	0.925649366494154	-0.0149964999999981	0.966813448118178
CUBN	0.00714587300859074	0.987546161977487	-0.289540547410363	0.488870852256136	-0.0158443333333332	0.95784037718285
YTHDC2	0.0071377550171103	0.987546161977487	0.0518157458418509	0.938168091114785	-0.00251566666666572	0.994956689715437
OPHN1	0.00713409346893136	0.987546161977487	-0.0144389671614986	0.983444353656142	-0.268162333333333	0.260859460677011
WASF1	0.00710881079968474	0.987566663118613	0.188276262183676	0.70742148547693	0.212304333333334	0.314112513261693
ZBTB39	0.00697933554983674	0.987572466384162	0.212417183693424	0.656785033957219	-0.0298383333333327	0.915761046438965
LRRC3DN	0.00697557604831585	0.987572466384162	0.132161965552415	0.810300414153187	-0.197126	0.502191611703409
KLF8	0.00696449316063535	0.987572466384162	-0.327030978936244	0.408851707589499	0.189696000000001	0.498454352317107
ABCB11	0.00696386052782598	0.987572466384162	0.17261132259545	0.738421727152922	-0.102869999999999	0.623903073849364
ADSL	0.00685964027770108	0.9878810010193	-0.00397481307586997	0.994491789164038	-0.0662516666666662	0.78611926267931
C20orf194	0.00685212963962424	0.9878810010193	0.134314909962097	0.808009477165608	0.385804666666668	0.121952230275805
CDHR3	0.00681035297876994	0.987994738019668	-0.148725461623049	0.783041584899534	-0.105429666666666	0.68400971768044
BAG6	0.00677963104994281	0.98800316315684	0.223241375804554	0.635219381121494	-0.0570976666666651	0.809755698910303
C20orf62	0.00676704226819699	0.98800316315684	-0.0224210458282513	0.97536722461972	-0.174415666666667	0.370661878103321
C8orf58	0.0067635322490749	0.98800316315684	0.442681600725244	0.185211009605428	-0.091495	0.685488482699362
TMEM74	0.00669410389804372	0.988087137396497	-0.334289544872856	0.394691211498274	0.083405666666667	0.698638671208267
ABCC8	0.00668589570334298	0.988087137396497	0.0461217632295709	0.945364757579857	-0.0393379999999998	0.892540400522449
DCLRE1A	0.00668193033493416	0.988087137396497	-0.193216464267925	0.698676320436036	0.189808333333334	0.391940380720525
ZNF649	0.00664609507096552	0.988113854487886	-0.356457710502115	0.345989229646028	-0.268104333333333	0.218478495014124
C20orf166-AS1	0.0066328969633424	0.988113854487886	-0.0951482751372606	0.872962271153999	-0.0493879999999994	0.869882427637781
RXFP2	0.00661033544487738	0.9881527105702	-0.130805745947934	0.812271254601992	0.141836666666667	0.517547647029412
TSNARE1	0.00643155169852894	0.988873328857047	0.140222359797730	0.797441782082887	-0.138729666666665	0.516635548538775
ABHD8	0.0063896185385606	0.988926433852696	0.563064431658703	0.0506240759292818	0.0824620000000011	0.778083808586846
SSR4	0.0063603832047794	0.988926433852696	-0.217742211494688	0.644743872041817	0.097018666666668	0.68149943269806
FAM213B	0.0063528251620221	0.988926433852696	0.147020150021837	0.785782367062857	0.0317430000000003	0.900467460403227
HYAL1	0.00635122514775119	0.988926433852696	0.322323420283129	0.417538034812104	0.171716333333334	0.575378149225683
RNF135	0.00632936640236766	0.988964996922452	-0.164173943532020	0.754189116283047	0.104999000000001	0.653246784345488
RBM45	0.00632205880473305	0.988964996922452	0.253109808030526	0.568478137843739	0.0562713333333332	0.799725367501923
C2orf57	0.00630584739591365	0.98899313668754	-0.173128576713008	0.736967971812539	-0.087855333333333	0.752384210059315
UBE2NL	0.00625454784047135	0.989011371537802	-0.0811928010158512	0.896346444707454	-0.0844050000000001	0.716517864791776
MSL3	0.00621188833300202	0.989139143644408	-0.434970036709776	0.197598735713799	0.0120013333333335	0.97184501043952
ALG1	0.00614116238581899	0.989217421415637	-0.00468048690914655	0.993354024627105	0.0199010000000005	0.944428790510578
C6orf165	0.00611866204092686	0.989230536506509	-0.043506494690286	0.949521794694691	-0.115639	0.592256474753133
HDX	0.0060854192587058	0.989350377642095	-0.437211847280144	0.194979332046875	0.0315503333333353	0.909196271455697
ETS1	0.00603286773973887	0.98936337291968	-0.50970503632427	0.101208857808966	0.274845666666667	0.296952839921866
AQP2	0.00599716735450125	0.98936337291968	-0.149245565300924	0.782576259201979	-0.0807353333333336	0.780717071872908
SLCO5A1	0.00598650700910611	0.98936337291968	0.254387605910664	0.567694846153007	-0.0201333333333328	0.941640072637564
AIDA	0.00586374023808143	0.989775667694729	0.0545975334803193	0.9344692810234	-0.139459666666666	0.486837155232753
SYT2	0.00579369985305837	0.98999616939341	0.00833796536909053	0.989949154584002	-0.163864333333333	0.669391045681572
PPP6C	0.00577128758300229	0.98999616939341	-0.0649412307876214	0.918489100363712	0.112863333333334	0.610487413621737
RGS13	0.00575685729012467	0.98999616939341	0.154139491545331	0.77207588593583	-0.141025666666666	0.501298037624658
IGFBP4	0.00574364439736964	0.990007914123216	-0.0588017495644922	0.928358924757516	-0.0662226666666653	0.827851662633248
FAM102B	0.00571434632153905	0.990107024673168	-0.581583484408558	0.0410486958316017	0.384323666666667	0.142821378450256
KBTBD11	0.00567685018106321	0.990114749018861	0.00768889537174377	0.990405674923721	-0.195126333333333	0.694407056956956
CBR4	0.00567505558583566	0.990114749018861	0.256983242721487	0.5632303733857	0.117756666666666	0.607328673358370
DUSP11	0.00567497151226402	0.990114749018861	-0.263403459693544	0.545874639137454	0.109090333333334	0.610847413222267
PKD1L1	0.00566871681904263	0.990114749018861	-0.0514845904101424	0.938728344711776	0.237847000000001	0.233528575266801
RLIM	0.00552807909970434	0.990497930107648	-0.426108082049187	0.21395651906068	-0.0340709999999995	0.899614658619676
GRM8	0.00552093250536345	0.990497930107648	0.115120952144743	0.84409286304878	-0.0669323333333329	0.775236864461361
MUC16	0.00547666811103463	0.990497930107648	0.350125224822828	0.358738646007386	-0.0253843333333326	0.92927353315392
CIDEA	0.00523444445853833	0.991451469936525	-0.172134488526914	0.738646873992406	-0.0266826666666665	0.937660160285902
ALOX12P2	0.00511781433492335	0.99189378135978	0.0873372659480241	0.886833468731436	0.0726453333333341	0.761837433189273
MRAP2	0.00510863568712432	0.99189378135978	-0.161762186790356	0.758930446817487	2.49044533333333	0.0252836057158779
PTGES	0.00507614813207013	0.991903951519182	0.0685216055668091	0.912149839732916	-0.00790999999999847	0.985183831169496
C16orf71	0.00507522702542936	0.991903951519182	0.278639361593392	0.511025440770733	-0.154899333333332	0.568393891086758
ZNF227	0.00507354986525504	0.991903951519182	-0.183580868361463	0.718067440349544	0.119459000000001	0.552125911260862
FAM182A	0.00497923911512168	0.99226589146942	-0.0149284223671251	0.983028706010457	0.0389521666666674	0.875727306158077
DFFB	0.00494170996900507	0.99231864477279	-0.227095620780403	0.62581495163125	-0.0653303333333329	0.797384658979621
RPN2	0.00488924447728567	0.992543039831503	-0.261109310244691	0.552696861759865	0.156433333333335	0.42330981660586
PPIL3	0.0048245191996297	0.992679842799668	0.210977063627070	0.660645714735488	-0.0741383333333324	0.73287100778903
IGSF8	0.00481287861987814	0.992679842799668	0.578999173427941	0.0427567474426921	-0.0309519999999998	0.911880659501762
MON2	0.00480427754628066	0.992679842799668	0.227978505929246	0.624226251100122	0.0319553333333353	0.90945379489823
HLA-DRB4	0.00477114506833162	0.992702814207875	-0.375860873583872	0.310614989418258	0.0283292222222231	0.91131019859749
FKTN	0.00468338397561275	0.992912855549273	-0.121722221982919	0.83097116342043	-0.243778666666667	0.207461391299889
C12orf56	0.0046733140146745	0.992912855549273	0.0406224732019276	0.952426531595115	0.168434666666667	0.471680039257208

XRN2	0.00464862093164930	0.992912855549273	-0.19266888542507	0.699966126007173	0.0847623333333346	0.730777457405814
KCTD16	0.00464373948503848	0.992912855549273	-0.149439243765916	0.78215339162259	-0.229362333333332	0.482433569556636
MYO15B	0.00455421627312078	0.993145256200874	0.072117823415494	0.90824872013293	-0.0963212222222223	0.656222957956058
NUP210L	0.00454877499706553	0.993145256200874	0.16247311683581	0.757685565672998	0.0563843333333338	0.814485009599967
TMEM41B	0.00453800181282463	0.993145256200874	0.0353567842635085	0.95869292666698	0.254425000000001	0.175476022490732
NFKBIE	0.00452342964264563	0.993145256200874	-0.529072334464477	0.0799297163112453	-0.0353886666666658	0.886697154717476
PTPRH	0.00452325249554801	0.993145256200874	0.0421429292471653	0.951916033831249	0.146044	0.543819286485342
TUBGCP4	0.00443792177029632	0.99348720987549	-0.0919823653111633	0.879153312143409	0.00988233333333405	0.982375192575954
RNF32	0.00442455732743209	0.993499541483934	-0.0934733513921151	0.876319616688429	0.00487600000000038	0.9879739922351
BNC1	0.00440214979731708	0.993513370292626	-0.298068895001728	0.472138727602117	-0.112866333333333	0.635069698575895
ANKRD20A11P	0.00439982936754773	0.993513370292626	0.435337250629088	0.197011021667727	0.184340500000001	0.419081654123944
AP5B1	0.00437481860307082	0.99358873352318	-0.133341127799249	0.808992949393043	0.0728143333333342	0.80181116616697
CLEC11A	0.00434277763465905	0.993702136729624	0.355866993828936	0.346552539524285	-0.111212666666666	0.696098804897955
WDR93	0.00429239021302148	0.993901371195602	0.0476372880982434	0.943611925889785	-0.164704333333334	0.389015722000736
FNDC7	0.00428378369652791	0.993901371195602	-0.00848608662678821	0.989705545062205	-0.0467319999999995	0.859264381447494
MPHOSPH8	0.00425217627747643	0.99401239186341	-0.463442041608137	0.157742452427219	-0.212020666666666	0.288015995370752
C3orf49	0.00422563431859259	0.994095991136722	0.0249024510679158	0.972871752171633	-0.0281976666666666	0.92951359233244
RGL2	0.00414554204766772	0.994409286494661	0.0793325838064917	0.898323801514594	0.0372550000000012	0.879939647520798
MZT2B	0.00411679726125952	0.994454395746571	0.168000396361018	0.748024524669236	-0.134795499999999	0.626096362424745
EPB41L5	0.00409315897299257	0.994454395746571	-0.205000009282942	0.673623956972285	0.334903333333335	0.0837916958855762
NICN1	0.00404992092556582	0.994562419790897	-0.433306611648961	0.201555549804162	0.087919666666668	0.707538975864338
ROBO3	0.00403379157593122	0.994562419790897	0.170462183739771	0.742264770850917	0.0134830000000009	0.972327702257384
ARL13B	0.00402845673951929	0.994562419790897	-0.439559263127475	0.190622420860054	-0.0428166666666653	0.864703661154805
MRPS5	0.00396307344181443	0.994855974236935	-0.027870223409253	0.969256428779973	0.0805720000000006	0.719839702715467
TSPYL1	0.00391859774837743	0.995006473715595	0.260852549651333	0.553105175440615	-0.203589833333332	0.355387672697857
PEG3	0.0038577848622177	0.995177935471283	0.23034815471305	0.618707227156037	-0.608560666666666	0.034300939647511
C8orf34	0.00378862633370341	0.995290848579375	-0.206242526962001	0.671467763213228	0.040951	0.872231116211086
BCHE	0.0037096279666458	0.995290848579375	0.106503019245141	0.858072018696671	0.386	0.0777324007395152
MESP1	0.00369412975893412	0.995290848579375	-0.0341626727121066	0.95969480339665	-0.0735053333333325	0.859264381447494
ZNRF2P1	0.00367015664064025	0.995290848579375	-0.114973911119305	0.844162087612813	-0.0649079999999996	0.821421133422134
LZIC	0.00366107042084257	0.995290848579375	-0.437264889514737	0.194979332046875	0.347258000000001	0.113878759491804
MYCL1	0.00357726515892053	0.995290848579375	-0.0508053841300614	0.939978131035994	-0.174690333333332	0.523021362996592
IGFN1	0.00356556629351138	0.995290848579375	0.331025356051226	0.401460837383713	-0.0544569999999996	0.904507944803624
NUP43	0.00354550860030345	0.995290848579375	-0.0730233850643579	0.906592046281011	-0.114869999999998	0.573883464758972
FBLIM1	0.00352197658055271	0.995290848579375	-0.187165593940360	0.709833349594307	-0.140837999999999	0.520846786113276
CCDC81	0.00350392292673055	0.995290848579375	-0.196857914593822	0.690061983084583	-0.139051666666666	0.49971129098408
CHIC1	0.00349602856488286	0.995290848579375	-0.220191876752530	0.640508005020556	0.111925	0.580149967309238
IGFBP2	0.00349232442702767	0.995290848579375	0.000113518736766373	0.999860038008296	-0.466152999999999	0.282765572761291
PDSS1	0.0034887008320628	0.995290848579375	-0.151407074643922	0.777463276052907	-0.0380009999999994	0.903788355106891
NDST1	0.00338770455606431	0.995290848579375	-0.0452140872391518	0.946577404106596	0.147750333333335	0.501298037624658
EIF3B	0.00338080223357576	0.995290848579375	-0.0966534858614854	0.870857630743202	-0.163464999999999	0.40716732757632
KRT20	0.00337415052731929	0.995290848579375	-0.0817919836393563	0.89548747312664	0.0736730000000006	0.762118416653633
LINC00339	0.00337414336575996	0.995290848579375	-0.211945752190271	0.658364968650984	0.0186833333333343	0.955460918122607
DNPEP	0.00335651556953277	0.995290848579375	0.0784222727185933	0.899281198071059	-0.00749399999999999	0.982375192575954
AGPAT1	0.00334984473180982	0.995290848579375	-0.0532192019589447	0.936629055455007	0.0182093333333335	0.948621641545621
IAH1	0.00333415005989927	0.995290848579375	0.101990342580650	0.864616357134757	0.381030000000002	0.0751433048669344
C11orf92	0.00331414083977781	0.995290848579375	-0.0916545877427438	0.879272912391142	-0.95051	0.00421637924026936
CCDC83	0.00330024843628184	0.995290848579375	-0.177664251535446	0.728276800708888	-0.0938323333333333	0.757300156767767
NRG2	0.00326933936784433	0.995290848579375	0.0204300738480436	0.976412247012224	0.0146680000000013	0.95979629762472
RAB40C	0.00326369639570885	0.995290848579375	0.366149126698603	0.328778006159436	-0.074053666666666	0.741438256509195
C16orf62	0.00326308101850072	0.995290848579375	-0.103855373245183	0.861570109612412	0.0958840000000003	0.651315737851928
PPP1R21	0.00325862235997278	0.995290848579375	-0.325806570031260	0.409206882509236	0.0287620000000002	0.94375604597120
C3orf56	0.00319097719610526	0.99548748451334	0.175358733798454	0.732231540747892	0.0286733333333339	0.91143191097679
TBC1D8B	0.00312651810631665	0.995734135469547	0.166273743490693	0.75159489949777	-0.165191333333332	0.443378989641463
RPL35	0.00311188107325959	0.995734135469547	0.078397639489271	0.899281198071059	-0.0198666666666661	0.948621641545621
ZNF767	0.00307887554753417	0.99576407171632	-0.0991961356986444	0.868042453226125	0.152978333333333	0.508080341765777
KHDC3L	0.00307299680226052	0.99576407171632	0.116404227079199	0.842082118939645	-0.0848663333333328	0.741438256509195
FAM149B1	0.00304814232055125	0.995838115018967	0.0204400823600983	0.976412247012224	0.160169000000001	0.448692388508699
NDUFA12	0.00298035136529752	0.996128243378565	-0.0346860349838337	0.959404955370931	0.0687666666666671	0.754488794029141
UNQ6975	0.00296605720794969	0.996128243378565	0.177937262924674	0.727982464250053	-0.0813269999999994	0.756314439453732
PHF8	0.00294825147666975	0.996128243378565	-0.0700031516086093	0.910821504793275	-0.460600999999999	0.048170505761132
MYOC	0.00292841380110854	0.996128243378565	0.0519332156098273	0.938067483409541	-0.0826539999999997	0.749488819393983
SBSN	0.00292760448778939	0.996128243378565	-0.0272431405661125	0.970207612777188	0.0274146666666671	0.94271014940259
DHDH	0.00290848311490209	0.99616807064005	0.337930707489307	0.385303753646555	-0.249322666666666	0.327835090482928
NR6A1	0.0027644583213681	0.996361109216605	0.0134078837537131	0.98412883278237	0.107556666666668	0.705010402761235
SPOCK3	0.00275266842979285	0.996361109216605	0.242699367276158	0.592765380565798	0.106343333333334	0.629710931024195
SPSB4	0.00274212601535607	0.996361109216605	-0.0847996870607096	0.891309254245872	0.126197666666667	0.538968176654309
CTSE	0.00273355888738286	0.996361109216605	0.0652638491801019	0.918295693698803	0.0605103333333335	0.783604035243992
NDUFA3	0.00272994506755082	0.996361109216605	-0.121234485655145	0.83256316766566	0.0158593333333345	0.963224271516007
FAM122C	0.00272874164051767	0.996361109216605	-0.278868380175662	0.511025440770733	0.113135333333334	0.667732731151074
ING5	0.00270218112433613	0.996384401132993	0.287823658794879	0.492287202244585	0.0653023333333339	0.785435825986191
LINC00615	0.00266443597280138	0.99642953701276	-0.102218142009346	0.864178408340026	0.00871133333333395	0.982375192575954
SPRR2B	0.00265842088028891	0.99642953701276	0.00538851456170149	0.99251028338798	-0.073380666666666	0.733867173296405
CCDC134	0.00260319499057232	0.99652534584155	0.0870130103581486	0.887055939766881	0.183176333333334	0.379613779505161
SEZ6L2	0.00258090606781106	0.996558357312568	-0.0252912895746499	0.972310017823397	0.170876000000001	0.474455297935757
NKX2-1	0.00255088014551863	0.996600247081185	-0.205046919896349	0.673623956972285	-0.107598999999999	0.608679471465191
AMPD1	0.00248287415045707	0.996874438917765	-0.36260480765366	0.337143926897226	0.123268000000000	0.731972426004787
FBXL21	0.00242727443794749	0.996874438917765	-0.27471939280802	0.520095539214775	0.00362500000000076	0.993756838831997
LANCL1	0.00240392312898788	0.996904594352679	-0.273393393725047	0.523842407819134	0.186460000000001	0.324443655582244
SHCBP1L	0.00239418558850764	0.996904594352679	0.174441380952426	0.734267769889068	-0.120532333333333	0.548804275857029

FGD1	0.00234250449301435	0.99712255359259	-0.369898082142272	0.322377391230088	0.145518666666667	0.48608244495005
TRPM6	0.00223529115292596	0.997399734767838	0.162476707519809	0.757685565767492	0.0594286666666663	0.805901684673546
PRKG1	0.00214611143148889	0.997492256074277	-0.0970276255494992	0.870355054290092	0.0491466666666671	0.885895416574211
ZBED2	0.00211791690172644	0.997492256074277	-0.211000079275273	0.660645714735488	-0.0752513333333324	0.788993370936467
PPP1R32	0.0021010366463754	0.997492256074277	0.189707413542175	0.704928430617595	-0.115108000000000	0.671766969198954
NEDD1	0.00209566590549581	0.997492256074277	-0.294813427049214	0.478800228795472	0.116149333333334	0.583224592065676
FAM98B	0.00205611049835842	0.997585127088607	-0.204051394540181	0.675344930297902	-0.0697783333333324	0.78571117988648
ZNF345	0.00200229539486601	0.997669959287987	-0.0530887923973403	0.936712858731269	0.381808333333334	0.0592964386181794
PRDM2	0.00199578999026756	0.997669959287987	-0.358620080969938	0.343101042014771	-0.146195666666666	0.578402578384935
INF2	0.00196756499366251	0.997761704042866	-0.0684878757795266	0.912149839732916	0.0905088333333343	0.71936631462663
TOMM70A	0.00195459400628416	0.997771581265103	-0.0126496419592053	0.985214990982244	-0.104458999999999	0.616472837170869
PLCE1	0.00194035572935534	0.99778825757645	-0.11358144425804	0.845326910026624	0.622064000000001	0.0456150738964454
TNFRSF1A	0.00192412912406248	0.997815600602423	0.0387568542255319	0.954263089081373	0.0265473333333334	0.92484471634727
DENND2D	0.0019105425162514	0.997828776070588	0.24419284577948	0.588660529799743	-0.0441873333333324	0.871104061464043
ATRX	0.00187773112509861	0.99794509095174	-0.0341631628911017	0.95969480339665	0.0314761666666676	0.910625003107163
GTSF1	0.00175733544455778	0.99823372252405	-0.403846598962918	0.257216918236696	-0.0228829999999999	0.947860086718622
JAK1	0.00175711483210761	0.99823372252405	-0.433242665664296	0.201555549804162	-0.0468439999999979	0.883189085233712
DLEC1	0.00173025638407876	0.99823372252405	0.275870833929622	0.517601356486792	-0.0664836666666662	0.761583161312229
CLDN16	0.00169726676216075	0.99823372252405	0.238952939542605	0.600195828925044	-0.0265173333333335	0.935261323444314
IL1RAPL2	0.00167986589529157	0.99823372252405	0.304291731888929	0.459458170903768	-0.0429166666666664	0.86260982919627
TMEM179B	0.00167917144646851	0.99823372252405	-0.179410310305157	0.72498671240364	0.177490666666667	0.390967882720418
ADO	0.00165862005490510	0.99825610864842	0.176149664304603	0.730592176611963	-0.209641999999999	0.285620770305918
C20orf141	0.00165272439125651	0.99825610864842	0.219179056360089	0.641655489120978	-0.0210916666666663	0.95285450244329
CLPP	0.00159674738284808	0.99843676006187	0.0281557853055833	0.968668367660302	-0.0979053333333322	0.660962515038256
FAM216A	0.00145446734391548	0.998718563994004	0.0342585543753027	0.959623239761825	-0.149900999999999	0.552125911260862
CLNK	0.00143276227248906	0.998718563994004	0.313324221058016	0.438100620341757	0.141723000000000	0.52637364284281
LOC100134368	0.0013191923530646	0.998821273010604	0.0141788252076743	0.983444353656142	-0.0638973333333331	0.778477148290034
FCGRT	0.00131736071296660	0.998821273010604	0.00539480206038644	0.99251028338798	0.142702666666667	0.509471764056684
HP	0.00129466265401543	0.998821273010604	-0.144836983303695	0.78956185520301	-0.0186729999999997	0.946392308016584
BTN2A3P	0.00128725956362176	0.998821273010604	0.206415205172061	0.671220485448239	0.0674240000000003	0.813694870801638
C11orf72	0.00127717957794579	0.998821273010604	-0.300121220335918	0.466180114981519	0.0290410000000011	0.918363743182112
SMOX	0.00119668374679284	0.998924459934465	0.228382445020794	0.623306946452998	0.0918503333333334	0.671844709893521
OR11A1	0.00116151500964032	0.998924459934465	0.0460411146443777	0.945364757579857	-0.131206	0.523041310312964
NSUN4	0.00116032490915504	0.998924459934465	-0.0526451673346815	0.937357975161644	-0.105289333333332	0.646350580563869
GPNMB	0.00113843700143765	0.998973957967217	-0.290625871458808	0.487935421898319	-0.106452666666666	0.732554472781933
C6orf47	0.00107623935869107	0.998988408471048	0.182949349165015	0.718289071039953	0.125836555555556	0.583728961001425
KIF20A	0.00101892888762290	0.999144285445616	-0.0364512141715897	0.957567496533117	0.238895000000000	0.311354677197697
UCP3	0.000998252229586673	0.999195248053545	0.00332461988357643	0.994730189431381	-0.0296166666666654	0.911323900781612
LBH	0.000972528981353444	0.999196673120221	-0.305070725445194	0.458198595388891	0.144217333333334	0.62979571408014
LOC541472	0.000934393508416456	0.999196673120221	-0.0218461827749609	0.97536722461972	0.126035333333334	0.566937124812328
PDP2	0.00091150177405529	0.999196673120221	0.143996586850753	0.791666231156359	-0.322862333333333	0.176338492557783
CTAG2	0.000900023410657752	0.999196673120221	-0.00861874577043867	0.98946753270812	-0.201505000000000	0.413829649844041
DDX60	0.000873498886030797	0.999196673120221	-0.358131662710358	0.343395848463795	0.614399	0.0319449578583259
SNAP29	0.000857361319318111	0.999196673120221	-0.104780685823548	0.86067831050617	-0.0964663333333326	0.647253392806699
MYBPC1	0.0008554477663286	0.999196673120221	-0.339818830751328	0.380248604746386	0.105024666666667	0.675449330308211
POGK	0.00079832181157324	0.999196673120221	0.0441870981272952	0.948493391127553	0.160480000000000	0.446756640099031
RAD51AP1	0.000770264983697899	0.999196673120221	0.0742544779762208	0.905205116779936	0.0484383333333333	0.838603547123369
ITIH6	0.000741231589478204	0.999196673120221	0.0367731561226616	0.956918184553224	-0.128348333333333	0.543216543662577
MAGT1	0.000726224722564058	0.999196673120221	-0.524938578115626	0.0854578873278813	0.145949666666668	0.45895048781174
TMEM92	0.000720717585418909	0.999196673120221	0.0552988138209291	0.933513863139975	-0.180039999999998	0.471709876204528
PTOV1	0.000713320829213656	0.999196673120221	-0.0333240866083471	0.960784250392482	0.109622666666667	0.643889811074018
SURF6	0.000707295765172248	0.999196673120221	0.222723472673672	0.635495054621817	-0.482158666666665	0.0309320552705188
HOXD3	0.000703075521386345	0.999196673120221	0.267297761476209	0.536380012714446	-0.276461000000000	0.243957990539057
GAS6	0.00066432243299663	0.999196673120221	-0.274076614516692	0.521425198453909	-0.0280653333333325	0.950884054684253
GALE	0.000657459099544719	0.999196673120221	-0.181057827637551	0.72163737037735	-0.0324983333333328	0.903274690789156
APOL6	0.000634937241962993	0.999196673120221	-0.313677652545464	0.438046311563112	-0.229597333333333	0.219484011715032
SLC5A11	0.00062586367933535	0.999196673120221	0.119278644261633	0.836360753951164	-0.108346999999999	0.663358802403662
CDH3	0.000607889298205214	0.999196673120221	-0.140666425157805	0.796741421472984	-0.00968633333333326	0.974451420920923
IGFL1	0.000583537041967683	0.99920158298743	0.0272136545747768	0.970207612777188	0.111615333333333	0.711180779277199
KCTD18	0.000542430977642458	0.99924857725577	-0.0994179316048754	0.867651972215247	0.0337346666666663	0.905659119449126
SYT13	0.000500550782489641	0.99927870853899	-0.0751586985065127	0.904173044514532	-0.132622666666666	0.615722818909839
MRPL49	0.000499196203905594	0.99927870853899	-0.109760122172235	0.851195083614214	0.00685366666666612	0.9847596802354
RBX1	0.000440798863642581	0.999433780335459	0.189139705068199	0.706450470188434	0.163865666666667	0.417125112561133
EVI2A	0.000414303358910004	0.999433780335459	-0.442213244082391	0.185614496651228	0.0272276666666669	0.931446587444396
ZNF630	0.000393231477462160	0.999433780335459	-0.201622515460397	0.680054545720343	0.186702000000001	0.451481934260006
PLOD2	0.000378605183660634	0.999433780335459	0.183880828574098	0.718067440349544	-0.110442999999999	0.612712713498171
CNPY3	0.000324502059548916	0.99951897612736	-0.0220576754980112	0.97536722461972	0.0255536666666677	0.926815497753858
FADS2	0.000256038309058685	0.99971276231037	0.303850288028349	0.460052080110635	-0.39331	0.209567117558470
SLC2A12	0.000243535121398778	0.99971276231037	0.371886187188661	0.319073951161831	0.0799693333333344	0.761583161312229
ZC3H18	0.000200801891281238	0.999762459870634	-0.119411211342742	0.836099954217028	0.00210066666666878	0.994956689715437
KLRC3	0.000176443677009290	0.999762459870634	-0.129548556322256	0.814018501998363	-0.0631649999999999	0.81742145099016
ACP2	0.000170830978814453	0.999762459870634	0.0403548153994528	0.952720585102517	0.124961000000000	0.528890172622518
WARS	0.000144772164712614	0.999762459870634	-0.541490673971745	0.0683699479262842	-0.0246489999999997	0.941559580750404
IRF2BP2	0.000120414574451134	0.999762459870634	0.0953142093488819	0.872962271153999	-0.0262749999999993	0.919891803741744
PIK3CA	9.99064106915537e-05	0.999762459870634	0.051879997831549	0.938067483409541	0.0226566666666680	0.944218475609619
RNMTL1	7.35870829003104e-05	0.999762459870634	-0.0254455586869203	0.972264538231849	-0.0212046666666666	0.93646016234182
SAMD13	5.81499638751509e-05	0.999762459870634	-0.141711533210874	0.795109963623224	0.138715000000000	0.516150048081156
MIS18A	-4.46275680414751e-0	0.999762459870634	-0.126667160589731	0.820958242958398	-0.161818999999999	0.426518032099216
C2CD4B	-4.48674795408855e-0	0.999762459870634	0.249060604518776	0.576473471308405	-0.144769666666666	0.603409225888664
ZNF565	-4.66426507922167e-0	0.999762459870634	-0.231935245843407	0.613445765196643	-0.0387049999999998	0.90452008697883

STARD6	-7.63039602177274e-0	0.999762459870634	-0.092742336466997	0.877797216649453	-0.0220300000000000	0.93901998985171
ABP1	-0.00010091194692254	0.999762459870634	-0.157752272948062	0.767726202151986	0.0262386666666679	0.928897428735123
AIM1L	-0.00013040982686306	0.999762459870634	0.198528286780056	0.685952362965345	-0.0711799999999996	0.816899402862994
NPRL2	-0.00014516792380441	0.999762459870634	-0.00487450650762242	0.993025304536657	-0.137976333333333	0.493769389569969
DZIP3	-0.00016672700961479	0.999762459870634	0.254643964683207	0.567320220274185	0.236395000000001	0.209567117558470
LOC100288594	-0.00018890683743412	0.999762459870634	0.0205558164802266	0.976412247012224	-0.154757333333332	0.60283710701508
PTPRO	-0.00024891974112631	0.99971276231037	-0.352210368117463	0.353317846408010	0.0880803333333336	0.748579513438275
UBA52	-0.00024944140057320	0.99971276231037	0.111786542502442	0.848223659808244	-0.0326066666666656	0.897372284357512
MIR146A	-0.00033433981928567	0.99951897612736	0.29246731564269	0.483593153641414	-0.111103333333333	0.583708661347349
KCNG1	-0.00036278445538850	0.999433780335459	0.329507445804794	0.404377515671201	0.00328883333333315	0.993756838831997
CASP12	-0.00036480515758555	0.999433780335459	0.189417903528892	0.705411515598974	0.0852909999999999	0.745967084434416
SPAST	-0.00038040175465989	0.999433780335459	-0.299909533692938	0.466982636526558	0.170606333333335	0.390561931914011
HOXA1	-0.00041630006933089	0.999433780335459	0.0297807010457902	0.96695070705759	0.0183806666666665	0.967460065937822
SLC29A4	-0.00047739804894424	0.999298182644656	0.203234790219408	0.676646782959283	0.216744666666667	0.36523677764789
GOLGA6A	-0.00049219643307752	0.99927870853899	-0.282997159054952	0.502129505146473	-0.0417086666666651	0.874243298833041
RSPH6A	-0.00050093509668601	0.99927870853899	0.0607319403775313	0.925335786425382	-0.142937333333334	0.667293862238229
PAK1	-0.00054989254998381	0.99924857725577	-0.356997963050179	0.345138088652208	0.736064333333334	0.00784994780223591
JUND	-0.00057353232430394	0.99920158298743	-0.00093469026926169	0.998745415362821	0.116716000000001	0.565148387675909
IL22	-0.00057846912270586	0.99920158298743	-0.0842881815335894	0.892396784625427	-0.0478380000000001	0.843785188756119
ERBB2	-0.00061523637787493	0.999196673120221	-0.274757387137397	0.520095539214775	0.0936656666666678	0.666054079859859
TNFRSF10A	-0.00062541256258551	0.999196673120221	0.113601555191302	0.845326910026624	-0.0276973333333329	0.912833899801715
CXCR5	-0.00062836339640674	0.999196673120221	0.0920893027571831	0.879153312143409	0.0687530000000002	0.772139923817113
PDK3	-0.00063999258985222	0.999196673120221	-0.153605187771488	0.773363488500034	-0.160873333333332	0.478484283517744
NEURL4	-0.00067807977050620	0.999196673120221	0.034530067276548	0.959404955370931	-0.0882786666666656	0.724001791100896
ZFP28	-0.00067880178510489	0.999196673120221	-0.0528591180604244	0.937009489777134	0.11279700	0.60814016771842
ZFP30	-0.00068010680290418	0.999196673120221	0.0713231836161565	0.909119908757507	0.234983000000001	0.277641259535605
MGC2752	-0.00076208748155402	0.999196673120221	0.0845969700890878	0.891547139719321	-0.0842443333333332	0.779420961236402
CELF5	-0.00076857065348665	0.999196673120221	0.0896127593968588	0.883196389963269	-0.0353946666666663	0.91495346197678
EID1	-0.00081531828708145	0.999196673120221	0.257355201650271	0.563116080558022	0.0526400000000005	0.821587043214193
CCDC101	-0.00083907099769781	0.999196673120221	0.419885612610106	0.227417142008707	-0.035080666666667	0.895510661232872
RNASEL	-0.00083986227285654	0.999196673120221	-0.350499592255619	0.358430460089679	-0.119132999999999	0.607789868477511
LOC399900	-0.00088598576625148	0.999196673120221	0.145539784679440	0.787804113309913	-0.250928666666666	0.241115180230598
ZNF573	-0.00090628996253869	0.999196673120221	-0.129761749609329	0.813305249846504	-0.00218499999999907	0.995146554639975
CACNA1F	-0.00097522054278558	0.999196673120221	0.050163076029389	0.941133149988298	-0.0517086666666663	0.856316468856464
LHFPL5	-0.00105830758814130	0.998993310312685	-0.0559395038821769	0.932919548410715	0.0197513333333341	0.942800137888472
FAF2	-0.00107036767554172	0.998988408471048	-0.226037780873893	0.628541809779496	-0.106323666666667	0.601564043046757
RNF213	-0.00108848280220500	0.998988408471048	-0.199012889439198	0.685167881041453	-0.0945066666666659	0.764197723764219
SPRR3	-0.00110649964230650	0.998973957967217	-0.0215800637420624	0.97536722461972	0.160511333333334	0.408086593806833
ITGB1BP1	-0.00111080027703410	0.998973957967217	-0.0691986537692754	0.911837389834658	0.148596333333334	0.534060420301683
PRPF39	-0.00112466396813201	0.998973957967217	0.167353241001703	0.749061933609388	0.0226216666666672	0.94678260229888
GGA3	-0.00116835320754893	0.998924459934465	-0.172562322327964	0.738421727152922	0.0298676666666677	0.909728882572794
SSTR1	-0.00117773391493024	0.998924459934465	0.0792226385425668	0.898489802888428	-0.0750303333333325	0.853077816975915
AMN	-0.00118611028080883	0.998924459934465	0.267876684523396	0.53559545997875	-0.104714666666666	0.664196112369643
ZNF843	-0.00123868626308260	0.99886288877261	0.235022173966287	0.60755186112057	-0.128179666666666	0.67210569186526
PSMC3	-0.00123889410114505	0.99886288877261	-0.00940296154946896	0.98909166696601	-0.144097999999999	0.500875207139132
SLC35E1	-0.00126874561724746	0.998821273010604	-0.0749361707766163	0.904466414650249	-0.212788999999999	0.3353681567844
ZNF667	-0.00127002290285916	0.998821273010604	-0.116416874794283	0.842082118939645	-0.372265999999999	0.1780215342276
SPCS1	-0.00129610522676594	0.998821273010604	-0.0342379200103986	0.959623239761825	0.0894816666666678	0.672980097690282
DSCR8	-0.00131484995227088	0.998821273010604	-0.402328416156877	0.259895916604029	0.0528343333333334	0.839998150752089
LDLR	-0.00133232501251820	0.998821273010604	0.299397445228877	0.468245148036717	-0.0800809999999987	0.754351658576291
ARHGAP25	-0.00136707541488616	0.998821273010604	-0.154385909640819	0.771310656070265	-0.067048666666666	0.859264381447494
HIST1H1A	-0.00136794341212869	0.998821273010604	0.0838214321270417	0.892833228561937	-0.0585579999999987	0.824548552404467
NT5DC1	-0.00140341553012179	0.998816079728708	0.377911097593027	0.305766089617986	0.167409333333334	0.446476080586417
CHRND	-0.00144376927274770	0.998718563994004	-0.0358177176482039	0.958354725588542	-0.080655	0.766247450031517
UGT2B28	-0.00145991733888900	0.998718563994004	-0.189055108044144	0.706678053343727	0.00123600000000015	0.99704422037925
TNNC1	-0.00147616114837437	0.998718563994004	-0.0490481292160578	0.942126968886265	0.00480833333333383	0.987937064305561
CPN1	-0.00150160840832870	0.998648142673258	0.133877353815253	0.808567812988011	0.166057333333333	0.495283817122373
CBLB	-0.00154253707702895	0.998488522294027	0.190206038838294	0.704208582824765	0.265558333333335	0.234508116088724
MMP9	-0.00155326598910104	0.998488522294027	-0.0572388283176551	0.93071299016553	-0.217407333333333	0.446756640099031
TMEM30A	-0.00156498428860340	0.998487606190888	-0.109111828977309	0.85285991580488	0.206008000000002	0.274121181062751
KCNF1	-0.00158298847236921	0.998450808656193	0.310720631253807	0.444665888122633	0.0511193333333337	0.882040270326032
SIRT6	-0.00162071305485961	0.998368001876124	0.0302874330684893	0.966124606544196	-0.135514666666666	0.516467297561779
CACNA1B	-0.00169969421251812	0.99823372252405	0.134025617051076	0.80854627520514	0.00597200000000019	0.986471172041113
ZFY	-0.00169998343740958	0.99823372252405	0.121643717418036	0.831271741617649	-0.0111846666666666	0.974895360062947
OR10H1	-0.00174505636887685	0.99823372252405	0.300275967874516	0.465914681155138	0.00497333333333386	0.988133186272453
TRIML2	-0.00178782393483188	0.99818848659068	0.0917258442685067	0.879153312143409	0.0598683333333343	0.787230398823895
OR14J1	-0.00180805929344155	0.998139671514607	0.00229677591499537	0.996326683062937	0.084613	0.69907357310347
RGAG4	-0.00180841582464093	0.998139671514607	-0.378843985578858	0.304578046752771	0.0660333333333342	0.77394434422275
ZMAT5	-0.00184627743804049	0.998054108710541	0.0882019899012035	0.885557272034463	-0.0116586666666671	0.976702491879288
OTOR	-0.00200210220108154	0.997669959287987	0.199407526672074	0.684056115356325	-0.0668033333333334	0.764197723764219
CNPY4	-0.00202875095774649	0.997669959287987	0.170178721898302	0.74270446821332	0.0351170000000001	0.899912734474539
ADAT2	-0.00208137331985612	0.99750924772082	0.00787358521900353	0.990405674923721	-0.318744333333332	0.137453276113987
GRIN2B	-0.00209677678374308	0.997492256074277	0.021914423553163	0.97536722461972	0.0411599999999999	0.890511682678135
DTX3L	-0.00210380609226838	0.997492256074277	-0.292655366072639	0.483287060229305	0.232129333333334	0.229591548351276
CXorf67	-0.00216285755985870	0.99748996551965	0.163436963719692	0.7554789807435	-0.00176966666666550	0.99599108619222
RRS1	-0.00218229568051660	0.997445329662936	0.0810015522224618	0.896361013063453	-0.322332333333333	0.134309227552143
PDE4A	-0.00220078130312547	0.99740580477986	-0.123713396330911	0.826475556843795	-0.0424196666666663	0.892921857967499
RHEBL1	-0.00220717790676451	0.99740580477986	-0.0461077497654649	0.945364757579857	-0.0753679999999994	0.768091397859868
ZSCAN29	-0.00221003735616114	0.99740580477986	-0.0510342862213338	0.939432580299312	0.0948236666666672	0.684569287653569
PDK1	-0.00225033440100856	0.997378692472988	0.250999037597403	0.572882168047464	-0.104848666666666	0.631408228698652

FYN	-0.00225679524719243	0.997378692472988	-0.224156549464202	0.633180978990857	0.379587000000001	0.0711528556656884
VPS11	-0.00230118671437692	0.997225022535206	-0.262476499235422	0.548787322027919	0.0939623333333334	0.69206623851055
ZNF510	-0.00230356168299692	0.997225022535206	-0.119485002726170	0.836013196419542	0.115948000000001	0.596617523400524
NKIRAS1	-0.00242203875115017	0.996874438917765	0.18029206865494	0.723079097675399	0.253551000000001	0.195340089722887
SRMS	-0.00243334514228608	0.996874438917765	0.191906934095505	0.701479891661163	-0.100250666666666	0.726534731471962
L1CAM	-0.00244230950588558	0.996874438917765	-0.233772203092164	0.610289154293877	-0.0553873333333334	0.842903889380894
FBXO24	-0.00244442607145312	0.996874438917765	0.113269587403866	0.845903615736408	-0.0759829999999995	0.772139923817113
DMTF1	-0.00244814489806388	0.996874438917765	0.107699536717291	0.856450662791083	0.189629000000002	0.315859935065171
ODF3L2	-0.00255650921864237	0.996600247081185	0.136096894564002	0.805628922205698	-0.227966666666666	0.310960271128485
VPRBP	-0.00259816213209175	0.99652534584155	-0.173733456941710	0.735412641528423	-0.459956	0.0755898373594719
EHF	-0.00262757318628304	0.9964867474333	0.184780376283838	0.716020884288485	-0.127689333333333	0.592845916952892
OR10C1	-0.00264932641706837	0.99642953701276	-0.0109542435644848	0.98774587825743	0.0507168888888886	0.824548552404467
PPIG	-0.00265757470413956	0.99642953701276	-0.0714066281128847	0.909119908757507	0.186270333333333	0.359908305595919
EME1	-0.00270431359397308	0.996384401132993	0.103550982214831	0.861943478503987	0.0933850000000016	0.667040000089664
MAN2C1	-0.00273889140902259	0.996361109216605	0.0883228080772001	0.885142944149088	-0.0129166666666668	0.970456964810804
ATP2A2	-0.00273908188929004	0.996361109216605	-0.0396367034692974	0.953729831316741	0.0245533333333349	0.928295508521289
ST18	-0.00281382696870974	0.996361109216605	-0.03476304622692	0.959404955370931	0.0265603333333336	0.930620998877894
PPP1R15B	-0.00285050840577537	0.996244149011244	0.0090727647784458	0.98909166696601	-0.065745333333333	0.774210549173745
TBX10	-0.00285419516746999	0.996244149011244	0.203590003375008	0.676207233014711	-0.0883103333333331	0.760951989611575
ADAMTS16	-0.00285881365160224	0.996244149011244	0.138812307849322	0.798926084240744	-0.0697633333333329	0.760010763598572
PHKA2	-0.00289798120355282	0.99616807064005	0.273520948620011	0.523465946730304	0.122904666666666	0.580149967309238
PRDM11	-0.00295825439401928	0.996128243378565	0.298493163946340	0.471239854755259	0.117939000000001	0.575378149225683
SLC30A5	-0.00309130789702627	0.99576407171632	-0.309037033785786	0.449107398769815	0.0661900000000004	0.76307213318662
THTPA	-0.00311760430851359	0.995734135469547	-0.203261319744961	0.676646782959283	0.00262900000000030	0.993756838831997
PPP1R18	-0.00322756249006546	0.995350169860998	-0.365781642235473	0.32927628703889	-0.328718333333333	0.159240262526426
SPATS2L	-0.00324967399827334	0.995290848579375	-0.108986129217625	0.853033740855416	0.191527333333333	0.390561931914011
TCEAL1	-0.00328781052297919	0.995290848579375	-0.145036620867227	0.789126578958976	0.156003333333334	0.446756640099031
EN1	-0.00330239492157408	0.995290848579375	0.132922499303277	0.809578511927685	-0.116339333333332	0.596725125925172
CES4A	-0.00330368734449803	0.995290848579375	-0.165208094372242	0.752651476479399	-0.182767000000000	0.429283821148627
DHX16	-0.00334849003206283	0.995290848579375	0.0300384835760755	0.966396286514145	-0.101839166666666	0.678589880084092
ZBTB33	-0.00336132141640806	0.995290848579375	-0.416612286384226	0.234235464483992	-0.159310666666666	0.479014333115247
MRPS18B	-0.00336481334158792	0.995290848579375	0.0141570160094809	0.983444353656142	-0.0357993333333326	0.890073383340154
BANP	-0.00338220260672391	0.995290848579375	-0.0413973707300594	0.952098348256047	0.0670783333333346	0.808933385359076
BST1	-0.00339451322682001	0.995290848579375	-0.140259179823972	0.797441782082887	-0.0192306666666653	0.944218475609619
CADM2	-0.00340895624436346	0.995290848579375	-0.301923764842327	0.462618148484335	0.0275283333333337	0.95285450244329
ADORA2B	-0.00343899932299183	0.995290848579375	0.122677433189455	0.828930912293926	-0.211781666666666	0.408369243753251
ACTG2	-0.00344363091352363	0.995290848579375	-0.188587318322127	0.707361603786947	0.279657666666668	0.172571369175474
TAF5	-0.00347253502115831	0.995290848579375	0.00350511954024792	0.994730189431381	-0.0287923333333323	0.94375604597120
STAB1	-0.00348059694349034	0.995290848579375	-0.35969476949386	0.341510139061518	-0.0513343333333325	0.860864811530264
FAM134A	-0.00351696206774915	0.995290848579375	0.0555941716564171	0.933409139126562	-0.129580333333332	0.599629532408434
EYA3	-0.00357136323505974	0.995290848579375	-0.0472187086772824	0.943981149721771	-0.00090933333333246	0.99792238320329
BCAT2	-0.00358118847233415	0.995290848579375	0.271365491725358	0.527984832801825	-0.237001333333333	0.320225451789804
ETS2	-0.00364790436591649	0.995290848579375	-0.38840045752977	0.28486220160813	-0.471099333333333	0.158751033455501
ZIK1	-0.00365016319291549	0.995290848579375	-0.371524400251401	0.319541753509769	0.0269590000000012	0.928295508521289
SLC25A22	-0.00367388306424913	0.995290848579375	0.0117069392699336	0.986576763237112	-0.0510313333333328	0.856854911614454
WDR66	-0.00368153774452383	0.995290848579375	-0.5338066587286	0.0758152544692281	0.0940383333333334	0.741121617241292
SHKBP1	-0.0036950138174387	0.995290848579375	-0.176473347688889	0.730152680173573	0.0393926666666671	0.876164760107145
IGSF6	-0.00370646718272771	0.995290848579375	-0.402711011106179	0.259260852315461	-0.0540956666666661	0.811012164454065
TUBGCP6	-0.00373605851416704	0.995290848579375	0.0200420713962770	0.977115438186772	-0.0418816666666658	0.861986907584547
BECN1	-0.00380224821676966	0.995290848579375	-0.221490495019097	0.637706908132848	0.0077606666666672	0.98256318768706
COL21A1	-0.0038259373507271	0.995237251069472	-0.200911723841351	0.68016888532461	0.285926333333334	0.383716544590226
GALNT2	-0.00384801414152497	0.995177935471283	0.0883221073967704	0.885142944149088	0.447722333333334	0.0439646473428104
CWC25	-0.00388352459054768	0.995106014947525	-0.183813472418263	0.718067440349544	-0.0781026666666661	0.727239075127513
TBC1D2B	-0.00391303767991863	0.995006473715595	0.196560800384974	0.690838714578657	0.00121050000000121	0.99734800727507
SRSF8	-0.00405796221847815	0.994562419790897	0.0397519750053441	0.953647040142798	-0.500249333333333	0.0233091007249086
ALOXE3	-0.00409281812733155	0.994454395746571	-0.0498978738856651	0.941458038227918	-0.067617999999999	0.75988028828342
ZNF490	-0.00411448479142285	0.994454395746571	0.142585588431975	0.794041118363265	0.102093000000001	0.724384070314197
CYTH4	-0.00415887084617482	0.99439720639511	-0.267609616127482	0.535603758171522	-0.122713000000000	0.639837290428782
LHFPL2	-0.00449755383255405	0.993224385484252	0.00041383497677444	0.999513431881539	0.262509333333334	0.233528575266801
CHRNA9	-0.00454323466800767	0.993145256200874	-0.369581122797062	0.323237610582955	0.00940533333333404	0.978504228748436
KBTBD4	-0.00456172017693159	0.993145256200874	-0.0920393958225137	0.879153312143409	-0.247871999999999	0.206909867235320
HMGB3	-0.00464625370889402	0.992912855549273	-0.320502122692538	0.422260384043692	-0.199057333333334	0.390059778510774
MYBPC2	-0.00471276768622056	0.992839033415634	-0.140540708585518	0.79693828563394	-0.102529333333333	0.753937973093065
ADIG	-0.00474549826560427	0.992721723792606	0.331467658484047	0.400099845811371	-0.143297666666667	0.51786180567444
NPR1	-0.00475718020666705	0.992718454569635	-0.0266198271426950	0.970207612777188	0.429626333333334	0.294300395506211
AHI1	-0.00478456739804916	0.992690112260759	0.292300508537185	0.484076751426251	0.071673333333334	0.776112195351357
CA1	-0.00479754064292261	0.992679842799668	-0.184056781047325	0.717937553844852	-0.0469916666666663	0.847083064840772
TMTC3	-0.00485360252518936	0.992616155774728	-0.180818906985707	0.722159001254958	0.396768000000002	0.0582780972148087
TRMT2A	-0.00485633819347065	0.992616155774728	0.00351006439047149	0.994730189431381	-0.0757933333333328	0.759162700398522
ZNF214	-0.00495344232211322	0.992315078242738	0.0436894354908688	0.949043721172002	0.133785000000000	0.631961600239811
SLA2	-0.00497358811975677	0.99226589146942	-0.113218611322332	0.845978328141518	-0.0124193333333329	0.970094446214144
CCDC116	-0.00498246444887907	0.99226589146942	0.0287882172257898	0.96782691865619	0.0384233333333331	0.876963204487654
VIPR1	-0.00517816991893676	0.99163668542289	-0.0675230758260392	0.914146369077299	-0.908413666666665	0.0523892384037901
ENPP3	-0.00519358043181348	0.991613124845886	0.14349368007115	0.792614473650425	-0.451509999999999	0.0368932712496524
SUPV3L1	-0.00525150659961821	0.991418935359401	-0.0699553726879501	0.910885619751085	-0.154554666666667	0.481989564291462
GRIK1-AS1	-0.0053371460946164	0.991014309437414	0.172241286828494	0.738567943867944	-0.0098583333333335	0.978192116090894
DDB2	-0.00536051926045491	0.990947502535112	0.47290896937237	0.145443641689931	-0.170032666666666	0.491141191148714
FCGBP	-0.00540069377709959	0.99078951810165	-0.130260834635021	0.812271254601992	0.213034333333333	0.27071513514891
TCP11	-0.00543484573103115	0.990664198770304	0.124897598818477	0.824457843812434	-0.126748000000000	0.596617523400524
NIPSNAP1	-0.00547654076440687	0.990497930107648	-0.0113296586565073	0.987266335252239	0.00780533333333497	0.981191596773742

PRB1	-0.0054840366729731	0.990497930107648	0.0197119743878418	0.97763669370967	-0.136178499999999	0.575378149225683
HLTF	-0.00550486522104267	0.990497930107648	0.0610590295724184	0.924735826792669	-0.106763999999999	0.601564043046757
PEMT	-0.00550967147996435	0.990497930107648	-0.0357342471248946	0.958366440880488	-0.0356023333333333	0.901259733118065
PRPF4B	-0.0055852248605171	0.990328029322033	0.134739721176885	0.807867938002244	0.0080733333333342	0.98147292539484
CDH12	-0.00559854550295483	0.990315722612406	0.272953248144115	0.524926583987471	0.416289000000000	0.104622217543493
TULP3	-0.00560910139410165	0.990315722612406	0.0198029210400301	0.977554137803725	-0.0719066666666662	0.77718596107678
ADCY10	-0.00562302558265963	0.990302840769422	-0.0565427601945886	0.93176886339657	0.0204733333333335	0.94375604597120
MPV17L	-0.00576489371453742	0.98999616939341	-0.212406581840067	0.656785033957219	-0.212054666666666	0.512041798508902
SUDS3	-0.00576864346023496	0.98999616939341	0.0310974177794312	0.965118873711308	0.0549395000000004	0.820159966111813
SLC13A1	-0.00582333295706213	0.989935193555838	-0.097278298073467	0.870020870354202	-0.0141416666666666	0.96018064860593
GPR25	-0.0059284504708548	0.989484057065507	0.149131190908430	0.782612598081927	-0.185222666666666	0.467798588118316
FAM161A	-0.00593051367847492	0.989484057065507	0.0284733796774710	0.968004871923256	0.144209333333334	0.565148387675909
MUC1	-0.00594285234926525	0.989484057065507	0.0194089713201477	0.977910658942646	0.0613450000000005	0.798133257429747
PALB2	-0.0059837914371679	0.98936337291968	-0.114475139072310	0.844272085764738	0.0209873333333341	0.937545603686416
PEX13	-0.00598551765603846	0.98936337291968	0.0963080176822218	0.871610640556059	0.0295620000000008	0.907374738017166
C5orf24	-0.00599610140393958	0.98936337291968	-0.189350352272613	0.705531762385378	-0.201065666666664	0.359908305595919
RAMP3	-0.00600961833419603	0.98936337291968	-0.33107467086616	0.401413989019412	-0.120027666666666	0.648442399981194
PPP1CA	-0.00602428737370738	0.98936337291968	-0.0658141810954309	0.917339272986403	0.0646300000000025	0.786958961725064
GAK	-0.00607453647092322	0.989350377642095	-0.169569178747302	0.743722118234192	-0.0469836666666663	0.845891599383498
MDGA1	-0.00613211673973076	0.989217421415637	0.102014370393232	0.864616357134757	0.255301333333334	0.261668636036981
ZNF850	-0.0061430981075886	0.989217421415637	-0.442503305871491	0.185282268884974	0.0621420000000004	0.886500454815894
HBE1	-0.00617963677912635	0.989139143644408	0.0186300210267712	0.97902968742576	-0.117845333333333	0.590841338570121
ITK	-0.00618171496116484	0.989139143644408	-0.307463876512305	0.452689746287922	0.115870333333334	0.639214589273613
KRT74	-0.00619424480301155	0.989139143644408	0.23412313537066	0.609024819897621	-0.0440053333333326	0.919420614501031
C10orf10	-0.00624729085851841	0.989011371537802	-0.201926947544803	0.680054545720343	-0.238653000000000	0.336373925530627
CAMSAP1	-0.00625324534675444	0.989011371537802	-0.00322890976272462	0.994730189431381	-0.121265333333332	0.571364362269605
NFATC2IP	-0.0062744240791723	0.989011371537802	-0.00673204379046821	0.99129507664977	-0.259932333333333	0.168877300696392
ARID3B	-0.00628382735751317	0.989011371537802	-0.289914210098450	0.488870852256136	-0.0893809999999991	0.692492951777046
RSPO3	-0.0063546020552689	0.988926433852696	-0.108691094108358	0.8536426264533	0.202253	0.474709570893085
CADPS2	-0.00636048458055108	0.988926433852696	-0.105655201933698	0.85956218633532	0.802437666666668	0.00711541332977091
ELOVL3	-0.00642722143535005	0.988873328857047	0.118407382049278	0.837937752076601	-0.276822666666666	0.417125112561133
LOC146880	-0.00647204991522856	0.98874944991557	0.261745216970025	0.550803906509978	-0.0316803333333333	0.901808198966559
MYL2	-0.00647727357206164	0.98874944991557	-0.00526358115106629	0.99251028338798	0.0946523333333336	0.686147984639302
ZDHHC2	-0.0066036978409816	0.9881527105702	-0.0287696637792124	0.96782691865619	0.118169666666668	0.692043732062832
NCR2	-0.00664181191394955	0.988113854487886	0.214202964694746	0.653004496563115	-0.0500319999999992	0.89293783676224
SEC22A	-0.00666188551493743	0.988113854487886	-0.351118772468818	0.356603456853694	-0.0896173333333335	0.697261622653146
FIGNL1	-0.00669063996637965	0.988087137396497	-0.175836280452786	0.731631458094528	-0.117924333333333	0.568903633175651
RADIL	-0.00671774027247081	0.988087137396497	-0.154513448140765	0.771310656070265	-0.0914846666666662	0.745967084434416
TRAF3IP1	-0.00673484827716458	0.988087137396497	0.0955410209589995	0.872754579338908	-0.104383000000000	0.623988109816431
SERTAD1	-0.00677656344225955	0.98800316353684	-0.292851471514546	0.482775213764401	0.0545056666666676	0.856868322249419
NBLA00301	-0.00680920388834345	0.987994738019668	-0.160945600289493	0.761016253411733	0.0089856666666671	0.982893960080295
WFDC11	-0.00691573010994165	0.98765451544627	0.0324531433781857	0.962133837439606	-0.057415666666666	0.822390330537826
MYL3	-0.00693953450758003	0.987584851934686	0.146097113217476	0.787402233709509	-0.0856293333333332	0.704370299831112
SLC15A3	-0.00694039482010472	0.987584851934686	-0.385317515198241	0.290650609338649	-0.184209333333333	0.398204716330972
MRPL41	-0.00700676592371125	0.987572466384162	0.200223895253077	0.681532337836957	-0.0147143333333319	0.960386058967456
LRRTM2	-0.00700807263662404	0.987572466384162	0.0277914231245235	0.969317858168394	0.0754563333333336	0.774104133965233
RLBP1	-0.0070247209045325	0.987572466384162	-0.0809603335716624	0.896361013063453	-0.0938906666666657	0.649977903605799
NKRF	-0.00703931312873389	0.987572466384162	-0.435498961912345	0.196965277981193	-0.063339	0.780904457213926
C6orf163	-0.00704231353955473	0.987572466384162	0.204187050375239	0.675320698349846	0.0498333333333336	0.870543028294187
SNRNP48	-0.00706062050536553	0.987572466384162	0.107271216867982	0.857185026392178	-0.191529333333332	0.376224594108747
FOXP3	-0.00707507123511078	0.987567359122406	0.147892381438414	0.784090188380266	-0.160988666666667	0.458006502146897
MED26	-0.00707528599770588	0.987567359122406	0.0774646855547008	0.900797933355475	-0.145409000000000	0.517547647029412
TMCO5A	-0.0070796574919895	0.987567359122406	-0.248223670349074	0.577768154755719	-0.0896043333333329	0.733650645007746
C17orf51	-0.0071082817328532	0.987566663118613	0.0411429531891258	0.952102539249605	-0.244989666666666	0.27721812275707
KLF17	-0.00714997227400433	0.987546161977487	-8.5644867048354e-05	0.999860038008296	-0.311918666666667	0.157434925792422
UBE2E2	-0.00716096521731912	0.987546161977487	-0.440977896384615	0.188105704960787	0.192224333333334	0.327835090482928
BANF2	-0.00721052241278919	0.987429927395615	0.130907230153841	0.812271254601992	-0.0635396666666666	0.830320848383177
PARL	-0.00724500215432024	0.987350220965653	-0.205906045310723	0.672561084372914	-0.175609666666666	0.467798588118316
CCT8	-0.00732927540884917	0.987135963060185	0.00641459189406151	0.991853352023739	-0.239062666666665	0.205336588312115
NDRG2	-0.00736940967513906	0.987135963060185	0.143159634914361	0.793064973612007	0.0463126666666667	0.843751236104194
HPN	-0.00739219397889556	0.987100097105295	0.0846017281019038	0.891547139719321	-0.108927999999999	0.729912257977231
SSB	-0.00742026438943072	0.987067189056492	0.0756658816171286	0.903510413986113	-0.0227789999999987	0.932560895493725
ITGA4	-0.0074498588371606	0.987025947039941	-0.457651287840074	0.166671273245692	0.127452000000001	0.687839312342416
ZNF696	-0.00749327645457156	0.986909158646792	0.172447894840974	0.738421727152922	-0.0078229999999992	0.983690244124512
POU2F3	-0.0075493144113586	0.986726444902736	-0.0175092604708505	0.980496266838056	-0.215307666666666	0.439339755781636
RBP3	-0.00763625119074224	0.986517599770886	0.299540115708804	0.468131316642775	-0.0877396666666657	0.747694872257288
PFKL	-0.00763711829428636	0.986517599770886	0.0868878991032943	0.887253963583638	-0.0701456666666663	0.759970711908908
TNMD	-0.00764894403201538	0.986517599770886	0.0920441644632804	0.879153312143409	-0.0498143333333331	0.843152489417868
PDPN	-0.00768338633451049	0.986517599770886	-0.262002679733063	0.5499549817547	-0.113402000000000	0.637584278840114
CTNNA3	-0.00773173690987685	0.986328445818055	0.102757129876741	0.86257932725575	0.227900333333334	0.255651669220982
TSSK4	-0.00776683780381826	0.986256976293308	-0.0971580194148256	0.870020870354202	-0.0477999999999997	0.841976773189511
TSPAN18	-0.00779127067702101	0.98618359551115	-0.0658031157135432	0.917339272986403	-0.309504666666665	0.483089789079152
CYP39A1	-0.00790746159013388	0.985849251294474	0.18946547286919	0.705315444616143	0.0853693333333347	0.711082533709514
JAKMIP3	-0.00791753006558206	0.985849251294474	0.162624309267591	0.757524249707797	-0.112382333333332	0.6096510569534
HSPG2	-0.00792464592575046	0.985849251294474	-0.113554790544648	0.84536273772944	-0.018719666666666	0.956567100065073
BBS9	-0.00795148187125436	0.98578914217854	-0.261312646544879	0.552187117311017	-0.185311666666667	0.568659275949231
MSTO1	-0.00800628098833228	0.985549546480488	-0.0269572127075482	0.970207612777188	-0.071462999999999	0.763233638804164
NOA1	-0.00804787040589154	0.985549546480488	-0.362664913959033	0.337143926897226	-0.0258726666666658	0.919420614501031
UBE4A	-0.008087791257424	0.985404683111164	-0.343697036235587	0.373021436752685	0.0170466666666677	0.95197590164177
FBXW11	-0.00816993372418126	0.985015329849845	-0.147923374214824	0.784090188380266	0.114657666666668	0.605121621910816

AAED1	-0.00818788933789821	0.985015329849845	-0.0775449564120759	0.900701176765499	0.186575000000002	0.714244999058301
KCNIP1	-0.00820574372733677	0.985015329849845	-0.0415857413015079	0.952005386179745	-0.126950666666666	0.550660741372463
FLJ34503	-0.00820883080106893	0.985015329849845	0.00980693422427567	0.989090482697721	-0.0273856666666664	0.929594956567283
CALML6	-0.00821771558562797	0.985015329849845	0.0905088773540479	0.881794677754899	-0.297848999999999	0.195820572246296
TFIP11	-0.00823607135715487	0.985015329849845	-0.286301233329715	0.495728580458409	-0.138070333333333	0.496462982918528
KRR1	-0.00824266693367504	0.985015329849845	0.325060154338143	0.41045730743103	0.0103983333333337	0.97236309164807
C19orf59	-0.00826809057540331	0.985015329849845	-0.0698462305711789	0.910885619751085	0.0306096666666670	0.90945379489823
SLC29A3	-0.00826896587058171	0.985015329849845	-0.252175238849395	0.570465803488487	0.102212333333333	0.634587745028953
ASB5	-0.00828316648822937	0.985015329849845	-0.0517460155214088	0.938273022292017	0.117125666666667	0.714382809933119
RAB6B	-0.00834370831749001	0.985015329849845	-0.270118759401784	0.530185035699403	-0.0854646666666666	0.745967084434416
PGBD4	-0.0084016373108273	0.985011876976705	-0.143219879072941	0.79294618815764	0.0617556666666678	0.82667899725046
TEP1	-0.00840200023662389	0.985011876976705	-0.252965186036125	0.568985221714455	-0.055032666666666	0.83616423402744
TDG	-0.00849996672449942	0.984750443927275	0.0303055147330464	0.966100909843628	0.199404000000001	0.42608701620641
C11orf83	-0.00850256132490735	0.984750443927275	-0.0410306659552835	0.952394447098818	-0.151450666666666	0.450232636847034
GTF3C3	-0.00858828160740805	0.984618622122584	0.145595748111642	0.787804113309913	-0.00747166666666595	0.98256318768706
FASTKD2	-0.00860145741936082	0.984618622122584	0.072525071234977	0.907556175729082	-0.0818729999999993	0.705260317228378
MANBA	-0.00868468643327202	0.984350866380494	-0.209861936506690	0.663436500730575	0.419805000000001	0.0452032920023148
IQGAP3	-0.00868802824939308	0.984350866380494	0.0891450052600729	0.883460564717377	0.215173666666668	0.34043575412146
MECOM	-0.0086924241266828	0.984350866380494	-0.275372093786643	0.519151381619209	0.0565923333333331	0.874908057954497
BBOX1	-0.00877213436794708	0.984066087511655	0.127916540078592	0.817803951566117	-0.0518803333333329	0.825269786103875
DEGS2	-0.00878199506706469	0.984066087511655	0.166883794073558	0.750076857136007	-0.101818999999999	0.739465662522697
TMX3	-0.0087950917865004	0.984066087511655	-0.06218312398141	0.92301259447339	0.123412000000001	0.551352991056569
KIAA0195	-0.00880158852309209	0.984066087511655	0.448860843686381	0.177860736739947	0.216268000000001	0.290345868058148
B3GNT6	-0.00883646359856561	0.984066087511655	0.087063912734845	0.887055939766881	-0.0310006666666657	0.942800137888472
OR1J4	-0.00885590643852394	0.984066087511655	0.121557607414972	0.831334839383787	0.11353	0.677647679541427
CST9L	-0.00902440361712692	0.983589224469531	-0.3206208995358	0.421921269743406	-0.0315689999999998	0.915182438764358
SIGLEC1	-0.00905491170951715	0.983589224469531	-0.304276085865187	0.459458170903768	0.0122443333333339	0.97184501043952
TXNDC9	-0.0090685074252624	0.983589224469531	-0.0640150233932743	0.92003791975205	0.132132333333335	0.625684461384028
GRIP1	-0.00910158301192599	0.983589224469531	-0.0584174402899529	0.928725686060612	-0.0826776666666658	0.838927397112197
FLJ37201	-0.00911014318633333	0.983589224469531	0.127308675789495	0.819667248990242	0.361599666666667	0.0714536337053717
MARVELD2	-0.009111434291661	0.983589224469531	0.388743336650417	0.284800807361049	0.165674333333334	0.459051870120874
PANK1	-0.00912200702131983	0.983589224469531	0.175982673004424	0.731162861882915	0.120295666666668	0.583708661347349
AKAP1	-0.00913181009544451	0.983589224469531	-0.194944033630058	0.695325543127847	-0.230825666666666	0.272895060299504
SLX4	-0.00914304588378812	0.983589224469531	0.00919533164435597	0.98909166696601	0.00648433333333452	0.985884500368235
PSG6	-0.00919405367311064	0.983589224469531	-0.114641253913285	0.844162087612813	0.194433666666667	0.355387672697857
ZNF672	-0.00921508610550711	0.983589224469531	0.212994319134515	0.655371496569188	-0.0750373333333338	0.758341714501375
DIMT1	-0.00922114803919913	0.983589224469531	-0.458088307354775	0.166671273245692	-0.00505833333333103	0.987802480176853
GBA3	-0.00926786680744928	0.983456659715844	0.286013781818803	0.496429042723156	0.0365913333333334	0.911623909907545
CBLC	-0.00927243225212299	0.983456659715844	0.15466183101112	0.771310656070265	0.0411516666666674	0.886543370934382
AASDH	-0.00928728233964646	0.983456659715844	-0.245970815349869	0.582912574644681	0.243286000000001	0.233528575266801
TCEB3B	-0.00934183144458702	0.983352134400071	0.228427773455019	0.623306946452998	0.119169666666667	0.567490596854382
DBNL	-0.00935236845409835	0.983352134400071	-0.103950964907668	0.861570109612412	-0.140790666666666	0.535487400963825
SSH2	-0.0093615603514642	0.983352134400071	-0.104485663577812	0.86098619534505	-0.140794666666667	0.541284231249241
MYF6	-0.00944165745053524	0.983171770848564	-0.234573102070441	0.608421850871805	-0.137561000000000	0.54292132372707
MYCT1	-0.00945193166489953	0.983171770848564	-0.164168849566089	0.754189116283047	-0.095436	0.71173331874509
RPS6KA1	-0.00954882186343425	0.982971202198771	0.211092487916157	0.660389781435267	-0.00383599999999898	0.99106990984423
DNTT	-0.00956360323824339	0.982971202198771	0.272872303316727	0.525056200978613	-0.0921803333333333	0.73097308185803
CXCL11	-0.0096529778137938	0.982547182558307	-0.475889136771356	0.143137411589934	0.150370333333333	0.463362442026763
DIRC2	-0.00977255750790636	0.98207046030145	-0.286709840193258	0.494947392191481	-0.123823999999999	0.556248360059405
ASNA1	-0.00978938063190917	0.982038289452813	0.0221207825582767	0.97536722461972	-0.0690233333333324	0.763424044800146
WDFY1	-0.00979174265768151	0.982038289452813	0.0222632222879131	0.97536722461972	-0.0104513333333321	0.974451420920923
TP53BP1	-0.00982173081648143	0.981981070729278	-0.248507288459735	0.57746120283043	0.110982666666667	0.637426515050706
NRCAM	-0.00984518308639296	0.981972815453095	0.371042639661411	0.320357523974285	-0.385050333333334	0.157439494855318
AQP1	-0.00987886123587908	0.981937423943774	-0.291886206532447	0.485243087131469	-0.177464	0.397798624983652
DPP9	-0.00988122174200683	0.981937423943774	0.0384715314096176	0.954545200586067	0.0159693333333342	0.955968113642085
ARMC9	-0.0099466720152537	0.981716212468575	-0.0313002039710578	0.964858024544415	0.00194300000000113	0.995621063297892
GSTM1	-0.00998886806834704	0.981604721330235	-0.0891951928627898	0.883460564717377	-0.0469213333333326	0.874464844016715
ZNF200	-0.00999439918462504	0.981604721330235	-0.374428596525607	0.314207741629911	0.139161666666668	0.540255856426522
DNMT3L	-0.0100756833792394	0.981428745653337	0.166673184035976	0.750443995356965	-0.0246236666666664	0.92865132951951
FAM27E3	-0.0101104626296008	0.981297612709604	0.0584706763125343	0.928725686060612	0.0422289999999999	0.870662337224757
NXNL1	-0.0101128653158317	0.981297612709604	0.208596619751031	0.666278181721133	-0.0788349999999996	0.791934055525056
PLEKHA8P1	-0.0101217488444838	0.981297612709604	-0.369266718636925	0.323285574998587	0.287983000000001	0.309421300309741
TMCO3	-0.0101907119281736	0.98127855430179	0.0796248826263262	0.898031113962014	0.0594816666666677	0.808612489493649
WDR5B	-0.0102440450820381	0.981045119283448	-0.235377279278579	0.60678764664537	0.0326843333333334	0.919420614501031
EMP2	-0.010262063932387	0.981006263411718	-0.199807375550718	0.682777037002814	-0.145382333333333	0.516635548538775
DDX23	-0.0102749291891968	0.98099580442042	-0.323655144691686	0.414215860139795	-0.222669999999999	0.246561548460872
FAM210A	-0.0103056638688769	0.980995092339144	0.0532100226276589	0.936629055455007	0.117433000000001	0.755053635067348
MTFP1	-0.0103771073147943	0.980795288009253	0.0867452101297187	0.887450470745992	0.0367136666666667	0.883189085233712
MTRF1	-0.0105122534914837	0.980519354778903	-0.0869747306495498	0.887065140370096	-0.0179906666666662	0.948920875503732
WWP1	-0.0105143721598053	0.980519354778903	0.473360125459051	0.145443641689931	0.0164356666666678	0.958565523123178
UPK3A	-0.0105585279691402	0.980519354778903	0.0428145426826594	0.950593273212722	-0.105485333333333	0.648209367206976
AKT1S1	-0.0105628314838930	0.980519354778903	0.296843170877488	0.475891984962559	-0.136501333333332	0.560413476606931
HFM1	-0.0105843967249847	0.980498659430435	-0.166539884523768	0.750627442079791	-0.00120149999999924	0.997134925912045
HIGD1A	-0.0105858357183623	0.980498659430435	-0.029205182985995	0.967677112447108	-0.121054333333333	0.613717653457397
DLAT	-0.0106444177888947	0.980492804016766	-0.267623262706952	0.535603758171522	-0.117828	0.608311546956177
NXPH4	-0.0107221306490205	0.980343554959522	-0.124762989511007	0.824652597696219	0.104055333333334	0.636641774707093
RPS6	-0.0107427088242249	0.980290472308245	0.286716311658818	0.494947392191481	-0.00126666666666538	0.996826292964745
GPR158	-0.0108723740662639	0.979877137508027	-0.215059655904799	0.651257878577277	0.0172436666666673	0.949852419200473
TNFAIP2	-0.010875178993311	0.979877137508027	-0.0535971083568575	0.935847524102816	0.00328500000000042	0.993677488355417
LCLAT1	-0.0109089105445246	0.979856908790827	-0.035556828395796	0.958689318480951	-0.116064333333333	0.661467769041579

PLEKHA2	-0.0109503210866528	0.979809754166634	-0.0593359798716511	0.927079474246234	-0.214364	0.286939266819770
ZNF678	-0.0109540427121490	0.979809754166634	0.101843399376343	0.864616357134757	0.108135166666668	0.706892642645718
ZNF473	-0.0110292762660048	0.979494661661714	-0.133117587368005	0.809355276890887	0.0501140000000007	0.880427463662501
FANCI	-0.0110432993012021	0.979494661661714	-0.116054970985735	0.842733236705133	0.0681503333333348	0.795270214132327
EXOC6	-0.0110738235054315	0.979477734779718	-0.174055010810002	0.734757646402866	0.355729333333334	0.0698861985917494
TIAM1	-0.0110805977680444	0.979477734779718	-0.123695182195326	0.826475556843795	0.215715	0.467798588118316
IFNE	-0.0111262994823955	0.979477734779718	-0.0755488925281377	0.903517361619051	0.00675433333333368	0.987134800094338
LRRC18	-0.0111299937654478	0.979477734779718	0.0991672129843726	0.868042453226125	-0.00957166666666652	0.974484855799046
KCNA6	-0.0111690982790503	0.979448415447601	-0.284953361585940	0.498464716905649	0.0963673333333337	0.688145991467449
POPDC3	-0.0112059592929043	0.979305205199632	-0.278251882603832	0.511880330989176	0.0310736666666671	0.902550440604425
CXCL12	-0.0112908524854882	0.978956983665234	-0.284364767368574	0.500419415740203	0.184067000000001	0.330829143983533
IL24	-0.0113004756402365	0.978956983665234	0.0173406613905299	0.980670285500112	-0.103411666666666	0.704904955554555
TUBG2	-0.0113585454033185	0.97876432311904	-0.112638769066814	0.847191484945575	0.0664716666666674	0.762890197654898
BANK1	-0.0114374701509477	0.9784544932837	-0.0711022871867572	0.90935479990339	0.0624716666666667	0.782886750366221
CD52	-0.0114723576639527	0.978377083304226	-0.118239586898364	0.838218827550598	0.0720740000000004	0.74687011643745
AP1G1	-0.0114774378588130	0.978377083304226	0.130621409729656	0.812271254601992	-0.39223	0.0649438674882112
PRDM14	-0.0114868716848090	0.978377083304226	0.171437202811619	0.739541495555634	-0.147066333333333	0.480601492957535
DNAH2	-0.0115276309693768	0.978313491558535	-0.00331802658378383	0.994730189431381	-0.0489773333333337	0.86131429953963
PHLDA2	-0.0115359031872992	0.978313491558535	-0.128717991341431	0.815407702581246	-0.0672693333333325	0.859264381447494
SIRT2	-0.0115471589922901	0.978313491558535	0.064414522185097	0.919813134013881	-0.0665369999999984	0.76409396614905
ZNF589	-0.0116497441818128	0.978243352149037	-0.283559915157036	0.501852406168029	0.118112666666668	0.615879889576619
SNORD114-3	-0.0117354649164258	0.97788060289885	0.25403176138414	0.567694846153007	0.0451373333333342	0.855231744039032
CPOX	-0.0117465964372872	0.97788060289885	0.0942906911018997	0.874645913015335	-0.0184463333333335	0.955115242974667
MYH13	-0.0117718190846307	0.97788060289885	0.125402614006039	0.824013463206763	-0.112592000000000	0.741134448923316
GPR153	-0.0117798495643401	0.97788060289885	-0.123613487126584	0.826505964393248	0.0219126666666667	0.949194996342177
CHI3L1	-0.011785132614233	0.97788060289885	-0.246827804285429	0.581279386777	-0.139087666666667	0.572707943305372
UBE2K	-0.0118322623850504	0.97788060289885	-0.291345536953558	0.486757482084406	-0.111679999999999	0.614221661998915
DNAAF2	-0.0118740801120728	0.97778883789978	-0.0283316700320396	0.968433247556251	0.278856666666667	0.166614298092430
NAA10	-0.0118974523889428	0.97778883789978	-0.362420481950789	0.337635834513295	0.0450270000000011	0.86183426299397
CKS2	-0.0119125520328768	0.97778883789978	0.0992860255487392	0.867939814640162	0.131236666666667	0.533597777826448
SLC28A1	-0.0119488044603013	0.97778883789978	0.083679665738387	0.892833228561937	0.0764670000000002	0.774104133965233
C19orf57	-0.0120237668700177	0.977626210205838	0.108711065231324	0.8536426264533	-0.142637333333332	0.470065542518108
HORMAD2	-0.0120511281850874	0.97753519558919	-0.241648417683834	0.595201219333254	0.113968333333333	0.617079629797294
TNNT2	-0.0120768686680793	0.977513776008793	0.178590215523489	0.726444926461881	-0.0705879999999994	0.789228734235839
BPIFB4	-0.0120845867423472	0.977513776008793	-0.0277059581573275	0.969601930443497	-0.135994000000000	0.5683587002004
DHFR	-0.0121381381845752	0.977325761716884	0.117411084065629	0.839825318851589	0.191371666666668	0.381152785222697
PIP4K2A	-0.0121819436412243	0.977234434004161	-0.491094522765129	0.123841586691124	0.312106333333335	0.189095480222886
HTR2C	-0.0122197032466475	0.977085830695953	0.0818349963285021	0.89548747312664	-0.0418243333333325	0.862744795576892
FAM209A	-0.0122516385861460	0.977077773065996	0.124481650854661	0.8249971578264	-0.0442483333333328	0.880427463662501
ARHGAP33	-0.0123651998452916	0.976582010871407	0.294225829502523	0.479894403496244	0.103567000000001	0.657726934707567
FAM181B	-0.0123837815462228	0.976539570276836	0.353654270703318	0.352236748429479	-0.0727373333333321	0.846106605564736
DAPK2	-0.0124143180143371	0.976451891142746	0.266931913375394	0.537408478863488	0.0120729999999993	0.967285731317065
OPTC	-0.0125100363382498	0.976231008201011	0.0251615424132985	0.972358966414305	0.0234103333333338	0.942393983671961
PROX2	-0.0125257558337193	0.976231008201011	0.195148152966875	0.694971001057948	-0.0755803333333331	0.735614422262526
DDX26B	-0.0125724151069347	0.976220556917915	0.337782804118501	0.385642813426369	0.101245	0.74687011643745
TTR	-0.0126261343754890	0.976043695581085	-0.195187751106061	0.694852194384891	-0.114452333333333	0.664196112369643
NPY6R	-0.0126674137436407	0.975875218744788	-0.00963505352908948	0.98909166696601	-0.0297363333333313	0.915761046438965
POM121L8P	-0.01266883613661	0.975875218744788	-0.15229873733405	0.775863712880333	0.0282461111111125	0.919359825156123
MAD1L1	-0.0126694625402141	0.975875218744788	-0.126256303153941	0.822164115743016	-0.0946003333333323	0.670113640649168
HMGN2	-0.0126950006456086	0.975875218744788	-0.0616245553290571	0.923246185283376	0.00355333333333437	0.99209221913287
KRT1	-0.0127620703735809	0.975834844867146	0.0618489553851263	0.923142970949889	-0.142971666666666	0.473337739823928
CREBL2	-0.0127810678538214	0.975790007850366	0.280628840384652	0.507460240190768	0.231716333333334	0.288133796028617
MRGPRX1	-0.0129096736601886	0.97525774892429	0.0293099700112555	0.967655960277738	-0.0884783333333333	0.855533429360007
KCNQ2	-0.0129394924234048	0.97525774892429	-0.185352485351182	0.714711178831204	-0.125737666666666	0.562674320626673
MBD4	-0.0129770673716366	0.975125970375063	-0.0430558590130296	0.950146638225747	0.0261753333333345	0.93278021276477
DRD3	-0.0129979661154707	0.975070517877517	0.198640806490705	0.685751316700852	-0.0569089999999997	0.82704790320885
CCDC92	-0.0130400194167824	0.974934590884107	-0.274545511260742	0.520439317950715	0.158849666666667	0.437376716606078
TOR1AIP2	-0.0130577956937221	0.974920054026426	-0.082832113493182	0.89362638370288	0.00989550000000127	0.974451420920923
VEZT	-0.0131735211587001	0.9745394613788	0.198205588561089	0.687345015143924	0.135144333333335	0.496462982918528
TUBE1	-0.0132066199731942	0.9745394613788	0.420073985995117	0.227275870797822	0.242666666666666	0.288460725027709
NCALD	-0.0132701801703274	0.974434837447505	-0.177485063638294	0.728529406441478	-0.00902499999999987	0.977997080364813
KRT78	-0.0132804292323603	0.974434837447505	0.0996747349599684	0.867603998426271	0.0688963333333342	0.78606666960761
CECR2	-0.0132893774985058	0.974434837447505	0.0176928772410765	0.980278729770454	-0.0436223333333323	0.863608092379234
ABRACL	-0.0133539167430576	0.974365878530921	0.0635797974231174	0.920855211745173	-0.458403333333332	0.0338015945178966
SLC6A6	-0.0133907551733963	0.974315790605524	-0.155287648389061	0.77058108906698	-1.01850133333333	0.00361742898882783
KLHL10	-0.0134342426498837	0.974277617942146	0.205551129799193	0.672695616352514	0.0502396666666668	0.84547275205075
SPRN	-0.0134418640564733	0.974277617942146	0.0865880807968468	0.887493600482322	-0.0730389999999998	0.794467820741688
BAI2	-0.0134478774067873	0.974277617942146	-0.0696880998489625	0.911167570018484	0.136424000000001	0.546434057395997
ZNF57	-0.0134618777756113	0.974277617942146	-0.204763721077264	0.673728362275223	-0.094930666666666	0.706573150130005
RPLP0	-0.0135885268056016	0.973667557050144	0.118591537022821	0.837522371512839	-0.0200369999999984	0.941640072637564
ZNF527	-0.0136201179276970	0.973552449416494	-0.136254769500472	0.805085290634736	0.0667660000000007	0.787579438125618
DISP2	-0.0136308438936773	0.973552449416494	-0.0126606258246766	0.985214990982244	-0.072906666666666	0.817579508079708
PRKACB	-0.0136489260370820	0.97351348290722	-0.340766680764483	0.378272273903863	0.0849770000000009	0.694195087922618
RAB5C	-0.0136874598656638	0.973359714237556	-0.175041628293706	0.733276438413475	0.00170433333333588	0.99628178244892
FAT3	-0.0137013038874912	0.973343336117428	-0.226662496059167	0.626670684247721	-0.00450766666666609	0.988133186272453
MRPL30	-0.0138344581467923	0.972844867873652	0.0333166236692297	0.960784250392482	-0.333307	0.168877300696392
VPS54	-0.0138494936588666	0.972844867873652	0.149370054347848	0.782397515639509	0.217526666666669	0.363358899074113
PRSS30P	-0.0139209163058323	0.97260703889587	-0.135266083231770	0.807470535721699	-0.0982680000000002	0.670113640649168
KLK9	-0.0139506775708404	0.97260703889587	0.231276522329078	0.615492377862864	-0.0848956666666657	0.751922332718575
TRIM63	-0.0139576678228662	0.97260703889587	-0.133293142611359	0.808992949393043	0.0129703333333342	0.973783155960644

CLCA4	-0.0139864182156531	0.97260703889587	-0.158020975197671	0.767373487136803	-0.0138759999999994	0.960769410434396
HGC6.3	-0.0139936151905751	0.97260703889587	0.0377871968678605	0.955190852940141	-0.142566333333333	0.552454037685747
NXPE1	-0.0141398592592606	0.971993462228412	-0.21945494546613	0.641226345931986	-0.082192	0.714382809933119
ACTL6A	-0.0141840871000606	0.971874490916315	0.119464111794501	0.836013196419542	0.0298940000000003	0.930375308310121
HOXB2	-0.0142147332137193	0.971874490916315	0.260948893122680	0.553022075740773	0.266345333333334	0.164651944779315
NOX1	-0.0142546703764211	0.971839109318454	0.116038977491556	0.842733236705133	-0.156613333333333	0.447498161237612
ACVR2A	-0.0143193497249886	0.971660674648208	-0.191293229974317	0.701804778213811	-0.209727666666666	0.324598441931638
LOC100128830	-0.0143809674553533	0.97158514566387	0.222438567303382	0.636029818392136	-0.238276666666666	0.419120371026967
JAKMIP1	-0.0143812734486558	0.97158514566387	-0.0383947205518274	0.954545200586067	-0.286569666666665	0.311354677197697
MYO1A	-0.0144093925473031	0.97158352363764	0.082688596826533	0.893753725133293	-0.00444266666666657	0.989201907882392
PEX19	-0.0144361257652871	0.97155585986998	-0.162165339302947	0.758408726071139	0.0837403333333344	0.704370299831112
CCDC61	-0.0145511709512036	0.971035722601538	0.148499373893408	0.783612110350999	-0.0644339999999999	0.772139923817113
TREX2	-0.0145516587038332	0.971035722601538	-0.0208178734759276	0.976330597778846	-0.140042500000000	0.596617523400524
PDC	-0.0145942790824632	0.97091667551661	-0.076467500685713	0.902679569009162	-0.0239333333333329	0.931055338143178
SYNJ1	-0.0146371941921927	0.970764859944607	-0.204340842595090	0.674837470309368	0.0934613333333348	0.703920073585524
PPL	-0.0146591385089249	0.970764859944607	-0.344158108061356	0.371242887531111	-0.111642333333332	0.607737988432737
COBL	-0.0146690163682771	0.970764859944607	-0.0781987544946914	0.899647854618521	0.00243533333333446	0.994917858972776
NEXN	-0.0146760257212552	0.970764859944607	-0.184439102294504	0.717040007673828	0.0483836666666671	0.841206167691009
KIAA1715	-0.0146810629466138	0.970764859944607	0.356257827013053	0.346087819515164	0.250988333333334	0.187638103900419
RWDD3	-0.0147637606189947	0.970578086207372	-0.197991237371598	0.687663943268976	0.165484000000000	0.448208117903793
POU4F3	-0.0147775413383504	0.97056188399748	0.201988394418401	0.680054547720343	-0.0524643333333329	0.824548552404467
MGC39545	-0.014778779187061	0.97056188399748	0.0937911175404882	0.875355472414883	-0.158839000000000	0.61183851242112
RAC1	-0.0147913983737743	0.97056188399748	-0.245986262665373	0.582912574644681	0.0995466666666682	0.636490807097692
UBE2C	-0.0148359422706529	0.970539474038738	0.0927721739215464	0.877797216649453	0.0807033333333343	0.76368723681351
TNFRSF8	-0.0148661775276913	0.970431419918118	0.162921889324014	0.756987543983042	-0.0894073333333333	0.777406710539424
CCDC137	-0.0150009186988297	0.96980068865894	0.0232805530643701	0.975348968561756	-0.229844333333333	0.253650543658781
RRM1	-0.0150090890185776	0.96980068865894	-0.0468345773302817	0.944274559688373	-0.0812336666666664	0.723383077113569
FBXO27	-0.0150953371443495	0.969548451668253	0.153755313397103	0.773238461355017	-0.146700999999999	0.493152852604312
C20orf26	-0.0151230987363517	0.969548451668253	-0.0641166115548466	0.920005468161721	-0.00331433333333313	0.991625835822726
C15orf62	-0.0151973503362542	0.969420132377438	0.0818731210965303	0.89548747312664	-0.173898333333333	0.391452559786487
C19orf25	-0.0152094107683087	0.969420132377438	0.133122396117285	0.809355276890887	-0.138388999999999	0.503217861354116
PFN2	-0.0153027973609671	0.969146566294153	-0.0723928708706992	0.907556175729082	0.136755000000000	0.498454352317107
HMGB1	-0.0153356434877224	0.969024902080884	-0.106476161259673	0.858072018696671	0.147439	0.55200825053725
DGKI	-0.0153396068743187	0.969024902080884	0.0232418541584404	0.975348968561756	-0.0199759999999993	0.942092082409632
OR7A10	-0.0153446098559212	0.969024902080884	-0.141208368338482	0.796285336930078	-0.0507046666666662	0.828240868185377
SUN2	-0.0153779863565155	0.969024902080884	0.147996680195274	0.784080588385358	0.309487000000001	0.127822686124728
ECHDC2	-0.0153879976088411	0.969024902080884	0.157484694578964	0.767770230680061	0.0259853333333344	0.925845244747948
PNPLA5	-0.0154506356025256	0.968806084934982	0.113326369472039	0.845747219093509	-0.104606000000000	0.756712997772435
RLN1	-0.0154782681222852	0.968806084934982	-0.0553584793993005	0.933409139126562	-0.317177333333333	0.220419507280706
OR4D1	-0.0155213097864882	0.968653887707595	0.036837389586895	0.956918184553224	0.137547000000000	0.615689623138822
SP140	-0.0155440376522660	0.96863833924918	-0.241832546858763	0.595095850165855	-0.0487886666666666	0.843134844119548
COG5	-0.0155619490362317	0.968622401356479	-0.0285739794020775	0.967838039797494	-0.0247243333333332	0.924674647382585
ZNF558	-0.0155636142289350	0.968622401356479	0.146930759385539	0.78589154083134	0.133553666666667	0.538851849177128
MACROD1	-0.0155792868270429	0.968622401356479	0.199167632341329	0.684571461406347	-0.069803	0.752992371482229
LEAP2	-0.0156061969416162	0.968604343084538	-0.241160616549269	0.596285896132316	-0.151707000000000	0.5293518047673
FAIM2	-0.0156154969764457	0.968604343084538	0.0112839678717970	0.98731277141449	-0.0315736666666658	0.94506324669512
RPS12	-0.0156164383764671	0.968604343084538	0.214727048892835	0.652003104177839	-0.114466666666666	0.656222957956058
TIMP2	-0.0156718372957618	0.968419444853627	0.066974058751169	0.915036159909267	0.0897846666666684	0.705260317228378
ECSIT	-0.0157375529736234	0.968173337120257	0.385973101187008	0.289470192870154	-0.0358839999999988	0.88636147735798
VIT	-0.0157460411722403	0.968173337120257	0.185013838261064	0.715658781310868	-0.130237333333333	0.64003454727887
NPR2	-0.0157652281014539	0.968173337120257	-0.0387108180077059	0.954263089081373	0.346915333333334	0.343572153228906
CRCT1	-0.0157739249887086	0.968173337120257	0.159781642169191	0.763150272733332	-0.0670636666666661	0.823781939390912
PTCH2	-0.0158150955008139	0.968103960300582	-0.00560191169855572	0.99251028338798	0.0704763333333344	0.785482517274791
C16orf74	-0.0158356797150162	0.968049528408183	0.341586881086718	0.377673659904403	-0.120020333333333	0.700075065613492
KIAA0020	-0.0158371269363582	0.968049528408183	-0.0809941676730169	0.896361013063453	-0.0966599999999992	0.695383127153992
C15orf59	-0.0158392825060418	0.968049528408183	0.0831194984459032	0.893287218356307	0.0215336666666676	0.949376459031393
E2F6	-0.015850520072365	0.968049528408183	0.0419868457736196	0.951989245458556	0.0632813333333339	0.781866487624294
C2orf88	-0.0158637608068936	0.968049528408183	-0.312104821479352	0.440558341460703	-0.0514399999999997	0.898982394082228
IP6K2	-0.0159105704605542	0.968005759837965	0.0677581628374657	0.913665851517816	0.116958000000001	0.565148387675909
IGFL2	-0.0159575517163986	0.967975042071118	-0.0817580328968024	0.89548747312664	0.0534050000000007	0.825621906949353
ITPKC	-0.0159871443517614	0.967870136965796	-0.318666209864633	0.428704672123663	-0.0690013333333326	0.757158592293374
ATL3	-0.0160226607789139	0.96779283267224	-0.366571907829541	0.328455020444395	0.302906500000001	0.110127837552696
REC8	-0.0160440275449034	0.967733967566746	0.297364674269648	0.474117388908907	-0.0978519999999999	0.639193282533739
NPFF	-0.0160790198188155	0.967651628550873	0.0577750999384101	0.929745210212238	-0.30142	0.190569948330175
PDIA2	-0.0161039003217801	0.967603071981992	0.147287103889446	0.78561060123541	-0.0749416666666666	0.80841656016322
LRRC73	-0.0161470311014259	0.967489388985278	0.0391246327737486	0.953905313456582	-0.0874113333333334	0.772139923817113
TNFRSF14	-0.0162410298831809	0.967238376233878	0.0026087319249147	0.99580901762095	-0.0393709999999995	0.883189085233712
SPACA7	-0.0162476667370769	0.967238376233878	0.129733737478518	0.813305249846504	-0.104479000000000	0.608311546956177
IYD	-0.0162581735009886	0.967238376233878	-0.0748806063978784	0.904466414650249	-0.00343066666666611	0.993756838831997
KLRAP1	-0.0162597855501461	0.967238376233878	0.161785778155542	0.758930446817487	-0.0400799999999999	0.871104061464043
ADAM29	-0.0162738580096217	0.967238376233878	0.220695060415409	0.639008702745457	0.070942	0.770112696597648
PURA	-0.0162790844952668	0.967238376233878	0.0335456908255199	0.960782399177614	0.0662343333333334	0.766569862858904
MTMR9	-0.0162921574925242	0.967238376233878	0.121961353310563	0.830347805834363	0.299778333333335	0.300124665604706
AVPR1B	-0.0163154411656356	0.967238376233878	-0.00139755189051158	0.998303187767855	-0.0770286666666661	0.777585466017059
INHBE	-0.0163158545267545	0.967238376233878	0.135584940567724	0.80669742201648	0.0341310000000003	0.898979600726666
ITGB2	-0.0163402702224595	0.967238376233878	-0.416542853223919	0.234235464483992	-0.0458976666666664	0.867139953860248
C8orf74	-0.0163466191197907	0.967238376233878	0.238819176746115	0.600563482790016	-0.0138766666666656	0.974110729041314
ZIC2	-0.0164160053266124	0.967169906070395	0.220585264559558	0.639149853200083	-0.155438666666666	0.487761541713127
LELP1	-0.0164798174526956	0.966989126933407	0.280225463281682	0.507980347140282	0.00165733333333447	0.99628178244892
ZFYVE19	-0.0165031076333202	0.966924830759317	-0.114192968283501	0.844272085764738	-0.152775000000000	0.459236736587605

TULP1	-0.0165062788258341	0.966924830759317	0.0722284451954775	0.908009852705821	-0.0584189999999999	0.791934055525056
ERVMER61-1	-0.0165272438554493	0.966911154004547	-0.193066408928618	0.699002603433222	-0.215636666666667	0.33651414986765
LGALS9	-0.0165287788783200	0.966911154004547	-0.355308778440307	0.347833413629182	-0.157814666666666	0.41785973815832
TNNI1	-0.0165603765696834	0.966911154004547	0.435479072564474	0.196965277981193	-0.157555666666666	0.449828629541335
GABARAPL3	-0.0165776496943482	0.966911154004547	-0.0620013385466461	0.923034515346352	0.119277333333334	0.747245436362979
TMEM145	-0.0165777982899006	0.966911154004547	-0.0819696748514692	0.895297079510875	0.143965000000002	0.644484616776256
ARSA	-0.0165852722369121	0.966911154004547	-0.137084731295691	0.802919666967562	0.137033666666668	0.566937124812328
RHOG	-0.0165917443399378	0.966911154004547	-0.232108188324161	0.61317136092084	0.0424323333333349	0.870996259555823
FAM109B	-0.0165998368381925	0.966911154004547	-0.176241395719849	0.730407919148629	0.00209666666666789	0.99529569362957
WDSUB1	-0.0166441687721601	0.966911154004547	-0.0864951141155936	0.88755074549681	0.208063000000000	0.383716544590226
ACP5	-0.0166501533482825	0.966911154004547	-0.0452622401045774	0.946556809762233	0.0520670000000002	0.871104061464043
CAPRIN2	-0.0166759955957436	0.966911154004547	0.0488452502496841	0.942180113539637	0.413685000000001	0.0833725664556335
IQCF1	-0.0167100439543293	0.966911154004547	0.102095506026351	0.864337135688791	-0.0604139999999992	0.840279502106244
TP53INP2	-0.0167346838550306	0.966911154004547	0.139634876966421	0.797957231657366	0.122683333333334	0.624595423091977
PPBP	-0.0167437178351107	0.966911154004547	-0.216426907717014	0.648230279306266	-0.0797459999999996	0.764973469318093
GPM6B	-0.0167472043910464	0.966911154004547	-0.00508190449417257	0.99251028338798	0.243215333333334	0.203817488671181
CDC20B	-0.0167555432228159	0.966911154004547	0.208519699895246	0.666505547128851	-0.00625566666666649	0.986471172041113
ATP2B2	-0.0167757697497121	0.966911154004547	-0.0548419760133741	0.934255557320745	-0.00134766666666657	0.99704422037925
NIPSNAP3A	-0.0167801698712494	0.966911154004547	0.252068891441770	0.570473184967855	0.0830713333333344	0.73097308185803
ZBTB1	-0.0168389087409088	0.966911154004547	-0.117168863213944	0.840841729303422	0.0206460000000010	0.944809257114924
MPEG1	-0.0168543781682816	0.966911154004547	-0.353428151049839	0.352407344218756	0.00508300000000043	0.987937064305561
CCDC106	-0.0168776807863309	0.966911154004547	-0.100687320097628	0.86628046156792	0.0315846666666673	0.911600329626215
C6orf15	-0.0168790200471133	0.966911154004547	0.0307001506304177	0.965460387628839	-0.0779074999999996	0.760810252329096
LEMD2	-0.016880817734492	0.966911154004547	-0.179747926245312	0.724074699453211	0.0665036666666681	0.772520347452753
NUP205	-0.0168938372556711	0.966911154004547	-0.00081834075589788	0.998745415362821	-0.0165123333333321	0.957568862260213
WFDC12	-0.0169802033243577	0.966865702500295	-0.169009317720648	0.745491053322477	-0.0194033333333329	0.955620005467488
CDH15	-0.0170250192185731	0.966735763564638	0.239180241052258	0.599801655225887	-0.178311333333333	0.510372461062965
FUZ	-0.0170470840221453	0.966733722237492	-0.070337443557737	0.910394157189308	0.0852169999999998	0.690641668678273
DCSTAMP	-0.0170713488006172	0.966658296337554	-0.0411976784024638	0.952098348256047	-0.0479439999999998	0.838603547123369
KISS1R	-0.0170785991583568	0.966658296337554	-0.132618927155076	0.809803594453954	-0.0549929999999992	0.830889449880472
UAP1L1	-0.0171135378218031	0.9666040945891	0.155481212655467	0.770187031128419	-0.291589333333333	0.292543465906489
TOR1A	-0.0171241916713528	0.9666040945891	-0.0293902851352956	0.967591608870015	0.0021166666666677	0.994917858972776
EID2B	-0.0171314627214032	0.9666040945891	0.0991253068247147	0.868042453226125	-0.00862933333333298	0.98147292539484
SLC34A1	-0.0171646055360444	0.9666040945891	0.270352503171547	0.529999252995877	0.0626346666666672	0.85576203728561
IFRD2	-0.0171886617351868	0.9666040945891	0.0262237881253195	0.970632644458507	-0.0801253333333317	0.714382809933119
KIAA0391	-0.0173096807268714	0.96629408589371	-0.15751736874789	0.767770230680061	-0.0407339999999999	0.874589840804364
PYY	-0.0173107564578507	0.96629408589371	0.36372737843003	0.335033160801809	-0.179697999999999	0.396454946335441
C6orf25	-0.0173715912187653	0.966128799430076	0.0124505313474586	0.985220352735461	-0.108640888888888	0.628131452559002
IL6	-0.01737296253959	0.966128799430076	-0.210410346557669	0.661670522548494	-0.138762666666666	0.480550639606866
CKB	-0.0174516139552621	0.966070016572635	-0.0303166910862327	0.966100909843628	-0.222686999999999	0.332735004225723
OLAH	-0.0175017934001899	0.96588427718496	0.093984760471856	0.875355472414883	0.00660366666666707	0.985484787924072
FJX1	-0.0175135075308951	0.965879383691418	0.0789021605759443	0.899154242391172	0.0456753333333345	0.903774117860245
PAEP	-0.0175831304469635	0.965548390204113	-0.106146699634761	0.85871402550174	-0.0537606666666665	0.856627065232437
C3orf80	-0.0175864371830485	0.965548390204113	0.158779718363308	0.765730464375784	0.130906333333334	0.541939010010167
PTRH2	-0.0177713221435095	0.964854784003152	0.120050662504134	0.835142119002087	-0.267952333333333	0.17289362417525
IL17B	-0.0178194187694951	0.964793497852556	-0.268310652224785	0.535217216332192	0.0278713333333343	0.919456505549688
CPSF3L	-0.0178833333328240	0.964653026970045	-0.350167697862225	0.358738646007386	-0.115411999999999	0.568659275949231
VWA7	-0.0179292161963503	0.964575646550905	0.141522009719961	0.795705351419283	0.0113530000000009	0.98129363917602
C16orf92	-0.0179453564959505	0.964545741797343	0.0866240537758662	0.887493600482322	-0.243625333333333	0.275939802512171
TARP	-0.0180923583603427	0.963961410284281	-0.296758130326774	0.475937559514153	-0.00115366666666565	0.997134925912045
NTHL1	-0.018119933663192	0.96386701308848	0.269187879927184	0.533126014175154	-0.0177239999999990	0.949492888401158
CASD1	-0.0181344064840857	0.963846465064417	0.307992541015841	0.451488724245047	-0.0104476666666654	0.974451420920923
NGRN	-0.0182959676541909	0.963301804931118	0.0493771824873511	0.941842562680095	-0.0154906666666661	0.959928393298083
CPNE9	-0.0182964499895818	0.963301804931118	-0.0793649737831902	0.898323801514594	-0.0542986666666661	0.811354407458157
PPME1	-0.0183230399181782	0.963301804931118	0.0234847241773545	0.975348968561756	0.178558000000002	0.398883894731131
RHPN1	-0.0183576584937528	0.963301804931118	0.0998537849588723	0.867553004916133	-0.00795199999999927	0.986350009478474
MZF1	-0.0184168576253121	0.963301804931118	0.145966434523954	0.787402233709509	-0.065762666666667	0.772520347452753
OAF	-0.0184687220873050	0.963301804931118	-0.0636248155070626	0.920855211745173	-0.268460333333333	0.248864745773935
PLCG2	-0.0184782509417751	0.963301804931118	-0.0910352121356606	0.881053992915981	0.0788230000000007	0.724384070314197
ADCY7	-0.0185006465979729	0.963301804931118	-0.353048395775375	0.352407344218756	-0.0058599999999997	0.985884500368235
RAB3A	-0.0185470914250344	0.963301804931118	0.278213564465426	0.511880330989176	-0.122209666666666	0.549323798856043
FARSB	-0.0185838405005965	0.963301804931118	-0.0459386093705871	0.945364757579857	0.072684666666667	0.763935841204439
ERICH1	-0.0185963422851275	0.963301804931118	-0.0249313959566864	0.972871752171633	0.059897166666667	0.799725367501923
RXRA	-0.0186006299884741	0.963301804931118	0.0723786037456812	0.907556175729082	0.0743513333333346	0.741134448923316
CRIM1	-0.0186357817358547	0.963301804931118	-0.04983012323554	0.941458038227918	0.536103666666667	0.0245465707456744
FCGR2A	-0.0187154099856226	0.963301804931118	-0.380204253766310	0.300705577749487	0.037743666666667	0.888319685310725
ECHDC1	-0.0187174468141248	0.963301804931118	-0.140767326316738	0.796714214272984	-0.214788666666665	0.282014649550509
PRDX1	-0.0187847118046584	0.963109043962797	-0.0627520986148782	0.922350559812086	-0.0232133333333328	0.931714916765969
ZNF366	-0.0188537777691258	0.962799477103743	0.196798885064826	0.690061983084583	-0.158096333333333	0.492100665301584
TSPYL6	-0.0188974018176585	0.962799477103743	0.101494152922277	0.865765350834364	0.133803666666667	0.577706190283961
STXBP5	-0.0189498514449665	0.962787391395818	0.0295211828275466	0.967226229119616	0.140481000000001	0.528127287297636
OR51E2	-0.0189698195054881	0.962787391395818	-0.103877998073037	0.861570109612412	0.00547533333333394	0.98764466753384
SERPINB12	-0.0190784609455854	0.962492144001176	-0.0831207932899236	0.893287218356307	0.0426203333333335	0.880427463662501
TXNDC8	-0.0191001908436800	0.962487967951025	-0.10047305215046	0.866539251141083	0.211222333333333	0.332161310031752
RSL1D1	-0.019140446529838	0.962321458289978	-0.152006177973897	0.776146388301083	-0.240329333333332	0.199298592432685
NARS2	-0.0191493257879205	0.962321458289978	-0.256173674514811	0.564628461858759	-0.138019333333333	0.538968176654309
RMND5A	-0.0192199093397841	0.962059735692546	-0.159801623886201	0.763150272733332	-0.940925333333333	0.0273198654230939
SUPT7L	-0.0192223400337542	0.962059735692546	-0.0329363665353824	0.961277493638149	0.0567720000000004	0.829369385827456
HOXD1	-0.0192641020292569	0.962019449181333	-0.00537900544697871	0.99251028338798	-0.0727653333333329	0.809755698910303
MMP11	-0.0192649033644384	0.962019449181333	0.0459270787425155	0.945364757579857	0.243873666666668	0.450203306181989

GRIK1-AS2	-0.0193564302902713	0.961772621095594	0.276983469829302	0.515587194962102	0.0521466666666671	0.845737357656109
RBMS2	-0.0194369225479292	0.961623068675594	0.125199973932777	0.824230922676958	0.221006000000000	0.246443273561679
TMEM67	-0.0194559972434761	0.961576300676483	0.137241915351469	0.802488298241532	0.0807660000000003	0.740842174759002
CLEC4D	-0.0194570105254252	0.961576300676483	-0.288989517212043	0.489280593381909	-0.176999000000000	0.351182087586052
ORAI2	-0.0194777951671545	0.961575333849804	-0.359071461639300	0.342594959625489	-0.281043666666665	0.214479402832005
GRIK2	-0.0195231386185379	0.961379817636192	0.123647933532601	0.826475556843795	-0.0160113333333328	0.976072925695116
CHSY3	-0.0195430317956856	0.961328395435104	-0.181360512833125	0.721174140548176	-0.218112000000000	0.434946851013257
MAPK13	-0.0195500731669676	0.961328395435104	-0.340820640164977	0.378272273903863	-0.213070666666667	0.309421300309741
CLDN17	-0.0195609690255403	0.961328395435104	-0.0513234416846958	0.938890620237414	0.0529506666666673	0.827433678445379
XIAP	-0.0196283493460867	0.961090124125493	-0.441271220375143	0.187767509360398	-0.0609373333333326	0.815540776675037
TOP1MT	-0.0196366513028132	0.961090124125493	0.467830915549151	0.151936428121882	-0.194939333333333	0.309988753408147
PI16	-0.0196752506968006	0.961008148506823	0.176622042474126	0.730152680173573	-0.079963666666666	0.780717071872908
SYTL5	-0.0196902898360416	0.96098418711431	-0.134262151784901	0.808009477165608	-0.195601999999999	0.305855213710406
PPP1R3F	-0.0197168031377492	0.960956454764939	0.203227622351497	0.676646782959283	-0.0149609999999997	0.96051270333788
BID	-0.0197216194923352	0.960956454764939	-0.374259343707317	0.314207741629911	-0.396874666666667	0.0521959245019195
LARP4B	-0.0197267909612049	0.960956454764939	0.059999383362703	0.925741394228908	-0.0324519999999987	0.90232248061402
KCNS1	-0.0197467683227848	0.960956454764939	-0.221956785915340	0.636680705852294	-0.341880333333333	0.190071767502635
RGP1	-0.0197597794423555	0.960956454764939	0.290268763676217	0.488568550695131	0.0450383333333334	0.860388779364817
IGFBPL1	-0.0197728685191098	0.960956454764939	-0.0480946925707566	0.94314236584312	0.0787376666666675	0.799725367501923
ZNF426	-0.0198524007434902	0.96067804486628	-0.164609080749555	0.754105947879839	0.153213333333334	0.514076428873743
GPR98	-0.0199119494091133	0.960401794755318	-0.54860255639308	0.0613797453852446	-0.342865666666665	0.295467950792479
ARSD	-0.0199309333902535	0.96035543495853	0.0398981440651114	0.953525981777884	-0.0784703333333328	0.741809049318412
NCF4	-0.0199522384597401	0.960314632390177	-0.331371281939653	0.400361755253837	-0.158121666666666	0.614221661998915
BPNT1	-0.0199745368117381	0.960314632390177	-0.0943790534097354	0.874461873364967	0.0905273333333359	0.687794959259872
FAM186B	-0.0199935299943453	0.960314632390177	0.141482071203329	0.795812554605343	0.0755073333333336	0.79414394251212
KRTAP13-1	-0.0200244843116683	0.960253831524569	0.221170660553058	0.638281520637887	-0.197441333333333	0.32708253982017
SEPSECS	-0.0200370303017671	0.960243953445976	-0.213059920685906	0.655185554924368	-0.0576356666666667	0.849114834471057
PRORSD1P	-0.020057106435231	0.960191375553833	-0.101649133780451	0.865262342519817	-0.0396266666666667	0.887363245747406
MBIP	-0.0200926670467346	0.960173511524895	0.172137442462787	0.738646873992406	0.140407333333334	0.49971129098408
CD177	-0.0201613500307194	0.959845247928216	-0.0363013794630922	0.95778718265765	0.133969333333334	0.710004432586908
ZNF646	-0.0201689110527809	0.959845247928216	0.170152009997788	0.74270446821332	-0.138411333333333	0.631192833987568
KDM3B	-0.0201707884467185	0.959845247928216	-0.111200226055718	0.849127967004266	-0.0172226666666668	0.953335531487028
YEATS4	-0.020195410061773	0.959845247928216	0.181352615994809	0.721174140548176	0.166083333333334	0.397778330217719
PLIN3	-0.0202060339298502	0.959845247928216	-0.117806439588848	0.838884923620932	0.0506436666666676	0.825729064695823
GOSR2	-0.0202398972878702	0.959828560874985	-0.21372295892769	0.653708954135733	-0.184250666666666	0.336373925530627
GPR146	-0.020253909014174	0.959810344414236	0.189097465606165	0.706614656293357	-0.125583666666666	0.62979571408014
SIX6	-0.0203035952951384	0.959684738937971	0.0265565031727891	0.970207612777188	-0.120292666666667	0.655951370096932
DNAJC12	-0.020347612451765	0.959649152366328	0.240969664828287	0.596648444172109	0.179434333333334	0.503217861354116
KCNK16	-0.0203570185101257	0.959649152366328	0.00541699223699216	0.99251028338798	-0.0629826666666662	0.84207712482186
C1orf68	-0.0203886484831916	0.95959732543061	0.171690917805184	0.739343956096839	-0.112701000000000	0.660122297714278
TRPV3	-0.0204342830665793	0.959460881579169	0.175612132507454	0.73187914820204	8.9333333333821e-05	0.999729972650062
KIAA1279	-0.0204531072374002	0.959418741885463	-0.0200774431791207	0.977011537442717	0.124577000000000	0.5530742296054
PTTG1	-0.0204960485462826	0.959296505496252	0.0090523498672691	0.98909166696601	0.0446633333333339	0.860301862460696
PEBP1	-0.0205166185516538	0.959296505496252	0.250157201002204	0.574321063440325	-0.0602433333333327	0.824548552404467
ARF3	-0.020549144979837	0.959296505496252	-0.350229579855226	0.358738646007386	-0.118473333333333	0.554622302038406
CRYGA	-0.0206293812676385	0.95896614219621	0.299655737556705	0.467649102112712	-0.0400716666666663	0.904507944803624
LOC93622	-0.0206359522978865	0.95896614219621	-0.112469358885338	0.847284575037946	-0.282581999999998	0.189510951797408
APOBEC3D	-0.0206780292447859	0.958873764834549	-0.101945590692211	0.864616357134757	-0.0478833333333317	0.839998150752089
KLK2	-0.0207843274271527	0.95851515344389	-0.0555176453347541	0.933409139126562	-0.0522313333333333	0.877641904580634
CLIP2	-0.0207847820929403	0.95851515344389	-0.155729215260901	0.769837060156515	0.0366746666666681	0.890094615232462
WDR7	-0.0207951435079596	0.95851515344389	-0.280537949856455	0.507460240190768	0.0124543333333339	0.97057157918193
CMTM2	-0.020814102332911	0.95851515344389	-0.19815463055167	0.687384485387017	0.0866506666666676	0.745967084434416
AKR1C1	-0.0208779646573633	0.958289669612965	-0.183408427316048	0.718067440349544	0.196717333333334	0.305124460469442
TBXA2R	-0.0208901364335699	0.958289669612965	0.0603848205826897	0.925649366494154	-0.0954703333333319	0.693684551749667
RIN1	-0.0209211019648459	0.958228472818492	0.133597339353728	0.808933084408149	-0.159468666666665	0.503217861354116
MORN5	-0.0209281344320800	0.958228472818492	0.0859825997151793	0.888306347447303	0.0110646666666672	0.974451420920923
FRAT2	-0.0209935228321888	0.957939488988284	-0.0814675564035007	0.89615326639288	-0.0885923333333323	0.716057356951225
ZC3H12A	-0.0210494577322116	0.95768286542358	-0.42651218106645	0.213221804021091	-0.0395583333333336	0.875253923096376
HSD11B1L	-0.0210605807235505	0.957680942578279	0.267215808764679	0.536453197697168	-0.103068333333333	0.65692454979738
CLDND1	-0.0210769137879262	0.957649403174803	-0.0130280210150438	0.984547546298896	0.160282000000001	0.405565769377661
C9orf64	-0.0210778603515522	0.957649403174803	-0.0195847092577007	0.977673457110824	0.169283333333335	0.386142100380776
sept-03	-0.0211569491768636	0.95762358039021	-0.101110829664743	0.866005093242444	-0.0425833333333323	0.895093529187836
LINC00479	-0.0212176153349995	0.957523899883896	0.0137313472280765	0.983872723592937	-0.0116753333333330	0.970250590875359
ITPRIPL1	-0.0212628982289618	0.957450351388028	-0.313662941481076	0.438046311563112	-0.0986653333333332	0.691236434401544
C1QTNF9B-AS1	-0.0212680641630543	0.957450351388028	-0.122069563909156	0.830036484464228	-0.0338038333333331	0.923019543204612
HEXB	-0.0213413503255355	0.957372133631158	0.0918560052092246	0.879153312143409	0.189940666666667	0.367206442400058
NAV2	-0.0214269432505459	0.957007775431525	-0.0634014504787382	0.920885810365418	0.0985060000000004	0.751545115802791
RNF19B	-0.0214510406601396	0.956993344234768	-0.431653294233906	0.205154507229699	0.172312666666666	0.427283471646461
DNAJC21	-0.0214693519090667	0.95695048433164	0.213803303513162	0.653708954135733	0.0536226666666666	0.821587043214193
UCN2	-0.0215041737904925	0.95687500270646	0.217030165329207	0.646970835651094	-0.158407999999999	0.536320838519271
SPC24	-0.021561729434245	0.956657481276069	0.287599617068969	0.492965339038039	0.0386083333333347	0.890511682678135
GNPNAT1	-0.0216038276471505	0.956657481276069	-0.152366510122034	0.775863712880333	0.0381196666666667	0.903778056312437
AMOTL2	-0.0216139687858969	0.956657481276069	-0.289405149975644	0.488871711189599	-0.117830666666666	0.74511187686951
SLC52A3	-0.0217000760953765	0.95643466602139	0.0129433293075974	0.98463111107678	-0.0364749999999994	0.914418029309221
ASF1A	-0.0217252074857977	0.95643466602139	0.0748656932544505	0.904466414650249	0.113233000000001	0.65569105202195
OCA2	-0.0217583584931744	0.956378166974644	-0.207293800002264	0.66888525756447	-0.0548013333333331	0.837163621558114
EPB41L4A-AS1	-0.0217652120278763	0.956378166974644	0.0105548918933968	0.98774587825743	-0.0467369999999986	0.843152489417868
SPATA16	-0.021788223268425	0.956376614892663	0.162933577936847	0.756977498877294	-0.134826666666666	0.566282142073858
DAOA-AS1	-0.0218485089915020	0.956141600880902	-0.114651827297253	0.844162087612813	0.0385226666666671	0.890511682678135
TRAPPC1	-0.0218862574767764	0.95598791113266	-0.0025913933271990	0.99580901762095	0.102054333333335	0.638202369830969

THAP10	-0.0218923087544209	0.95598791113266	-0.102472258712504	0.863158472924381	-0.0378366666666668	0.899912734474539
OAS2	-0.0219351371762005	0.955832165838675	-0.271763475728989	0.527608228552189	0.0600470000000003	0.788418231875778
SNRPD1	-0.0219916591879988	0.955749722487123	-0.0716404055437483	0.908546712333622	-0.106846666666665	0.633166039956913
ZNF233	-0.0220129488684179	0.955749722487123	-0.220547763523658	0.639162466991523	-0.0659186666666661	0.78124591605939
IGJ	-0.0220129588455615	0.955749722487123	-0.200514774276639	0.680830337398347	-0.371970666666667	0.441909622750662
PANX3	-0.0220630208804479	0.955749722487123	0.0355059900075252	0.958689318480951	-0.101816333333333	0.654323362279664
FOXJ1	-0.0220634839847386	0.955749722487123	0.223432246887333	0.63471624724109	-0.119900999999999	0.581570366864416
ZHX3	-0.0220961030900087	0.955749722487123	0.0683791470384195	0.912232638501567	0.000418000000000146	0.99866434080529
FAM71A	-0.0220987513020289	0.955749722487123	0.166901826860089	0.750076857136007	-0.130669833333333	0.552955692473106
CYLD	-0.0221041662589721	0.955749722487123	-0.178561182339270	0.726444926461881	-0.0811016666666666	0.771847553133033
CFHR2	-0.0221080197107395	0.955749722487123	-0.190700497138804	0.703245816076572	0.0470000000000004	0.885866310496875
KRT15	-0.0221637639100728	0.955709135771916	-0.0438183486827218	0.948934431299106	-0.0919509999999998	0.673417177632382
TTLL6	-0.0221909557070564	0.955663485615852	0.00789260359068681	0.990405674923721	0.0928320000000005	0.689661288408633
GPR156	-0.0222369713734101	0.95546243589192	-0.00926259538444861	0.98909166696601	-0.254820333333333	0.233528575266801
ANAPC11	-0.0223162220717163	0.955317547769898	0.248962226240156	0.576871285544251	0.207573333333334	0.293767671859086
HEATR5A	-0.0223227753668751	0.955317547769898	-0.00652194197719885	0.99157602801676	0.0704950000000009	0.77895955075587
KLHL4	-0.0223299759395107	0.955317547769898	-0.363486914697946	0.335533086114911	0.293146666666668	0.124984536231968
NOBOX	-0.0223748486850146	0.955167375843355	-0.023309940907125	0.975348968561756	-0.0298516666666657	0.925845244747948
RFTN1	-0.0224382458002862	0.954928478871806	-0.38119490621594	0.298054845970601	0.246575000000000	0.297888842945994
CAB39L	-0.0226017534711801	0.954363875451164	-0.0489995650754412	0.942180113539637	0.0844420000000004	0.821421133422134
DUS3L	-0.0226477810739440	0.954305007811725	0.128499851348981	0.81616974067692	-0.00532566666666666	0.987851590533963
USP51	-0.0227229145840253	0.954163685765336	-0.0489957275244252	0.942180113539637	-0.0300043333333331	0.911129648924728
WRAP53	-0.0228021870354655	0.953818662081652	-0.101351840551544	0.865954921256394	0.0235396666666668	0.935179763869757
NEDD9	-0.0228032768552372	0.953818662081652	-0.426237707612512	0.213660668060841	0.0971703333333336	0.741438256509195
COL19A1	-0.022843574026271	0.953818662081652	0.253541092757339	0.567705016149998	-0.0778686666666666	0.733867173296405
CCDC153	-0.0228495598423802	0.953818662081652	0.0950344170442633	0.872962271153999	-0.132043333333333	0.589703432017128
ZNF256	-0.0228690967045489	0.953818662081652	-0.0637969752427921	0.920521809324956	-0.0202689999999995	0.948469012194168
AHNAK2	-0.0228761990201216	0.953818662081652	-0.366494313744663	0.328484373471503	-0.0334549999999995	0.926989565501108
BCAS4	-0.0229003275174004	0.953818662081652	-0.0424495815701203	0.951446425515852	0.328051666666668	0.09418645408026
T	-0.0229261929122464	0.953818662081652	-0.0124237643010015	0.985220352735461	0.174469000000001	0.41903055047835
SMG9	-0.0229916165368497	0.953648479152213	-0.0330341105147889	0.961277493638149	0.230305000000000	0.217750635011612
ANKRD52	-0.0230143002189661	0.953605499580367	0.0797297560199878	0.897892885174022	0.153283666666668	0.466098392080126
SPARCL1	-0.0230333379918095	0.953605499580367	0.210446455022472	0.661670522548494	0.171295	0.631511678860383
ATP1B1	-0.0230736293914822	0.953605499580367	-0.0441043085778275	0.948520928173175	0.0613433333333351	0.816134952712854
CRELD2	-0.0230763837709497	0.953605499580367	-0.118668576973944	0.83731413798382	-0.113299999999999	0.65441512437094
HDHD2	-0.0230796615158924	0.953605499580367	-0.038373438116911	0.954545200586067	0.0705883333333358	0.789228734235839
CYP2W1	-0.0231629052983449	0.95337096273507	0.222920539936166	0.635247978870818	-0.0229133333333318	0.954216641809453
ATP6V0D2	-0.0231678258359512	0.95337096273507	-0.246020742223613	0.582912574644681	-0.0980676666666666	0.648861254074544
CYB5D2	-0.0232066645899102	0.953295780308193	0.0993348401397813	0.867933660937568	0.110910000000001	0.633561720825521
TNFRSF13C	-0.0232292521491299	0.953260521231606	0.137475628576385	0.802039543563725	-0.00281633333333167	0.993756838831997
CHGB	-0.0232294114410347	0.953260521231606	-0.0658983545932082	0.917090201751967	0.131720333333334	0.536444100847478
PEF1	-0.0232728855966436	0.953234143949403	-0.219336976373061	0.641314646479258	0.0966596666666668	0.68135180147671
CYP2U1	-0.0233857442273776	0.952773056149496	-0.146665082389593	0.786187758946602	0.0948063333333338	0.723383077113569
POLR3F	-0.0233867087167963	0.952773056149496	-0.087971982416598	0.885848000380851	-0.0675473333333327	0.795446186721078
BANF1	-0.0234038460896905	0.952773056149496	-0.200095489836236	0.68192181800777	-0.114999999999999	0.609981941749066
PTPRT	-0.0234058846780445	0.952773056149496	-0.0464891780045567	0.944839700668385	-0.0292729999999997	0.93278021276477
C4orf19	-0.0234203081560464	0.952773056149496	-0.157584663308238	0.767770230680061	-0.202082999999999	0.343729765439488
HEATR3	-0.0234406743097547	0.952773056149496	0.00542820052786617	0.99251028338798	-0.0232333333333330	0.931055338143178
CEP152	-0.0234923890559383	0.952773056149496	0.233204363231364	0.611287729493682	0.0577140000000006	0.845891599383498
LMO1	-0.0235032354718318	0.952773056149496	0.104056374375726	0.861570109612412	-0.0381816666666659	0.901246264384169
SCAPER	-0.0235606027575778	0.952633921593231	-0.254520355084893	0.567585390815808	0.112869000000000	0.587691500804211
PKHD1	-0.0235852935296919	0.952554119825863	-0.120399257973976	0.834560994486918	0.102542333333334	0.651318171574714
GTF2I	-0.0235933594697022	0.952554119825863	-0.178681098782656	0.726380623752689	0.0903593333333341	0.676024903789639
PPT1	-0.0236683463870670	0.95234547684085	-0.200481727818502	0.680830337398347	-0.0419599999999995	0.873398028647672
ARF1	-0.023686931738105	0.95234547684085	-0.189834729719868	0.704724567131716	-0.0813466666666659	0.700855603017082
GPR174	-0.023694637176542	0.95234547684085	0.0244526770011137	0.973562415846915	0.149746000000001	0.573673650964407
MRAS	-0.0237219744971471	0.95234547684085	0.0210607685075480	0.97607156937294	0.141646333333333	0.488602547609567
GYPB	-0.0237884319571716	0.95234547684085	-0.328169062985371	0.407365872456796	0.0394023333333335	0.895093529187836
TAF1C	-0.0237968985107285	0.95234547684085	0.414572903120012	0.237522444879778	-0.0586809999999991	0.811867274062946
DRAP1	-0.0238153860409304	0.95234482269337	0.0323688723238483	0.962269716253437	-0.0819849999999995	0.716057356951225
RHBDL1	-0.0238155621454397	0.95234482269337	0.10575772350676	0.859545278949543	-0.251174666666666	0.2729560423704
ADAMTS20	-0.0238320322759752	0.95234482269337	-0.436788928106998	0.195288910691951	0.140920333333334	0.510372461062965
L3MBTL1	-0.0238407687846163	0.95234482269337	-0.125216827040104	0.824230922676958	-0.0315549999999995	0.935352983719623
C5orf51	-0.0238540327465546	0.95234482269337	-0.0297252917424323	0.96695070705759	0.0189800000000012	0.945904449997854
ARNTL	-0.0238929268843918	0.952270182743188	0.165393496243049	0.752352242644389	-0.113805000000000	0.617169282107924
BOD1L2	-0.0239002433482678	0.952270182743188	-0.142647611453344	0.794004622916083	-0.0507959999999991	0.883960545140055
NMNAT3	-0.0239235874776657	0.952217698647555	0.116151955827149	0.842507799216635	-0.0738799999999997	0.774104133965233
GCNT1	-0.0239473074828623	0.952143349057609	-0.0153563222876514	0.982725338620588	-0.0538199999999991	0.813858339366752
RBM24	-0.0239539278005033	0.952143349057609	-0.40529223553726	0.25480455106032	0.352899	0.131369372059974
AKAP8L	-0.0239544580922101	0.952143349057609	0.346522073308847	0.365349151543041	-0.0621449999999991	0.783138907861786
IDE	-0.0239570728190968	0.952143349057609	-0.289765620373784	0.488870852256136	-0.163311666666665	0.409270914061388
AFF2	-0.0239745219926793	0.952143349057609	0.0385630503123782	0.954545200586067	0.141554333333334	0.490929966167076
HBEGF	-0.0241362231958304	0.951676474342177	-0.226310184299761	0.62739910276142	0.0515290000000011	0.842903889380894
LPP	-0.0242254959036501	0.951410976356597	-0.109066056778705	0.852975887741054	0.371024666666667	0.154573540919994
TMLHE	-0.0243295501524074	0.95093727893136	-0.360820382422680	0.34034906917442	-0.0268709999999985	0.929195660123953
LINC00521	-0.0243352371707087	0.95093727893136	-0.109897114406660	0.851013616427418	-0.115366666666666	0.603463436419421
PON3	-0.0243445866689116	0.95093727893136	-0.178074064394188	0.727939088352802	-0.124573000000000	0.646581321628762
RAB3IL1	-0.0243548656179757	0.95093727893136	0.167211264232162	0.749363202500443	-0.0497913333333327	0.865748629809888
PAK7	-0.0244141425153418	0.950732506402299	0.153853287187201	0.772926602479237	-0.0970409999999992	0.68717273201429
CD200	-0.0244168916328022	0.950732506402299	-0.401211074431709	0.262072933179063	-0.051013666666666	0.85994094150432

RASEF	-0.0244416800150834	0.950732506402299	-0.00101053074891102	0.998689442034748	0.129215000000000	0.709400163383053
RNF138	-0.0244535802871929	0.950732506402299	-0.0288435068832134	0.96782691865619	0.310966333333333	0.114599211633843
ZC2HC1C	-0.0244697188234101	0.950732506402299	-0.359727868387314	0.341510139061518	-0.0375216666666661	0.900101096868846
COMMD6	-0.0244721002103308	0.950732506402299	-0.00764512583782264	0.990405674923721	0.0819743333333337	0.774104133965233
ORM1	-0.024509153271717	0.950732506402299	-0.228027414552674	0.624148135339169	-0.0770589999999994	0.834476346642783
NPM2	-0.0245752326692902	0.950535626512187	0.127144662914036	0.820136656695479	-0.09453	0.731069486458256
RNF148	-0.0246013924028951	0.950535626512187	-0.288796996091147	0.489813374112312	0.412177	0.0456150738964454
SLITRK6	-0.0246325806225354	0.950535626512187	0.2719963850065	0.527284725671175	0.0116536666666669	0.969104903637376
FBLN7	-0.024729276511956	0.9501463230797	0.230235597585779	0.618707227156037	0.272323000000001	0.266979834423163
MMP20	-0.0247898689155837	0.9501463230797	0.098613849309776	0.868080433714765	0.0650710000000011	0.774104133965233
SLC46A2	-0.0248117502921081	0.950142146010828	0.0232527988721152	0.975348968561756	0.108310333333334	0.634966811516658
KIAA1755	-0.0248371639154241	0.950107081122055	0.0619425072671377	0.923034515346352	0.0720916666666667	0.747694997696212
BMF	-0.0248449374843251	0.950107081122055	-0.143060860608171	0.793304811422389	0.0700163333333336	0.789105818888855
C9orf152	-0.0248638390880749	0.950107081122055	0.180191060742836	0.723415882806093	0.0371633333333335	0.912872646779014
SLCO4C1	-0.0248687258150056	0.950107081122055	-0.249487463544163	0.575370998761354	-0.0184124999999999	0.954736472265512
PMM1	-0.0248862796708601	0.950107081122055	0.155082482139098	0.770708441022583	-0.0808419999999985	0.764973469318093
SNAP91	-0.0249001652628909	0.950107081122055	-0.104295178953112	0.86124543980304	-0.0531846666666666	0.824700707442131
JMJD6	-0.0249169502197286	0.950107081122055	0.199668075852314	0.683147715897123	0.151325666666668	0.522830837265953
ATP2B1	-0.0249399152301387	0.950107081122055	0.201669943896401	0.680054545720343	0.500167333333335	0.030025317565228
PIGG	-0.0249677554916783	0.950107081122055	-0.051927245202909	0.938067483409541	-0.0112249999999989	0.973783155960644
CD33	-0.0249819331844736	0.950107081122055	-0.240468955463352	0.597862631330416	0.263983333333335	0.203817488671181
SEMA7A	-0.0250070231702272	0.950107081122055	0.140118607793010	0.797462217955029	-0.160502999999999	0.609858095838542
BCL10	-0.0250331807618399	0.950107081122055	-0.296737776090479	0.475937559514153	0.326415666666667	0.108927207186132
NTMT1	-0.0250598479038710	0.950074270804855	-0.116828554540569	0.841562044027187	-0.135473666666666	0.500047580338372
PRND	-0.0250764131866501	0.95004076548618	-0.276711797813496	0.516650180998052	0.100438666666667	0.69523606597876
NPFFR1	-0.0250913904310675	0.95004076548618	0.294428404949174	0.479692636619539	-0.108613333333333	0.73168347388773
NDUFA10	-0.0251388469609744	0.94999055412821	0.0493582085847928	0.941842562680095	-0.0854543333333317	0.689543334701612
TBL3	-0.0251859517837029	0.94983735015005	0.0203950356923926	0.976452505880603	-0.0700409999999999	0.779668393144765
PHKG2	-0.0251941953053734	0.94983735015005	0.286673427077974	0.495026397372472	0.0174356666666672	0.955396570818642
TBC1D22A	-0.0252168875585258	0.949788674085074	-0.0192509444805203	0.97820434113681	-0.0277259999999994	0.918192039123086
RAB40AL	-0.0252227109781804	0.949788674085074	0.078394034593708	0.899281198071059	0.0477583333333337	0.837163621558114
INPP5B	-0.0252567119710125	0.949691142494257	0.0634993927625728	0.920855211745173	0.0271023333333349	0.927671110581394
PRLH	-0.0252870092666177	0.949632315084737	0.0998723006482793	0.867553004916133	-0.153403999999999	0.610151958903093
CCS	-0.0252920554498316	0.949632315084737	0.0255419876160526	0.972057389914729	-0.0595883333333329	0.82667899725046
SLC1A7	-0.0253187293670318	0.949573350813487	0.216628804200948	0.647400684192295	-0.177162666666666	0.523453039510001
CCL7	-0.0253230682583827	0.949573350813487	-0.235978623793111	0.606121203124993	0.0463680000000006	0.871104061464043
MTUS2	-0.0253468641351841	0.949535021922546	0.0345996831018709	0.959404955370931	-0.0996323333333328	0.651904335623326
CYP4V2	-0.0254006277171345	0.949410938549992	0.271185707502241	0.528032433754325	0.287321666666667	0.158485868172990
ZNF681	-0.0254204118000118	0.949410938549992	0.0791823081334242	0.898637746363503	0.578838333333334	0.0942538294765004
FPR3	-0.0254392443148874	0.949410938549992	-0.370376884064283	0.320801726666339	0.0551443333333338	0.821421133422134
TARSL2	-0.0254652477316202	0.949347989990218	0.0281369009290295	0.968668367660302	0.128996	0.518741522051306
GPR84	-0.0255094685845533	0.949289115921692	-0.332441622748160	0.398142341492687	0.0835946666666672	0.739507465050387
DCHS2	-0.0255816371194867	0.949110471602622	-0.0536512928700109	0.935777496327359	0.046938666666667	0.841976773189511
PLIN4	-0.0255891403142447	0.949110471602622	0.154382306838158	0.771310656070265	-0.0392106666666656	0.883913953154694
ZDHHC15	-0.0255942532242499	0.949110471602622	-0.522275276128074	0.0876859028054308	0.182587666666667	0.484583000340636
LMLN	-0.0255973443985831	0.949110471602622	0.0549729341163909	0.934246366571164	-0.278619999999999	0.246462634148331
TIPRL	-0.0256508674036728	0.949082763897123	-0.178819204592724	0.726006906302736	0.0566173333333343	0.811816884537132
OR1E1	-0.0256579187752639	0.949082763897123	0.0223483570210261	0.97536722461972	0.065865333333334	0.768446434650364
DOK7	-0.0257361089918321	0.948962747651763	-0.051408609707125	0.938729151196048	-0.0593953333333326	0.847609712518279
UHMK1	-0.0257969371512777	0.948697258853674	-0.0789401014376936	0.899154242391172	0.138780000000000	0.486325323768433
KCNE1L	-0.0258703276739954	0.948566919785722	-0.033261698615778	0.960784250392482	-0.0066946666666663	0.987134800094338
HIGD1B	-0.0259034757032094	0.948492855087622	0.0305735267128091	0.965652334162082	-0.088611333333332	0.753182944057782
KCNMB2	-0.0259035410628785	0.948492855087622	-0.148351433840334	0.78389646294903	0.139875333333334	0.523041310312964
WDR24	-0.0259515023223843	0.948361405939703	-0.0330516089856517	0.961277493638149	-0.183828666666667	0.329705038929258
FUT10	-0.0259545402381304	0.948361405939703	0.160493038304984	0.761693615106737	0.158121333333334	0.427283471646461
SCAMP1	-0.0259643141215649	0.948361405939703	-0.309605764289691	0.447328073030462	0.0212493333333338	0.937717709824356
CYP2C18	-0.0259763358851007	0.948361405939703	0.160348221090724	0.762217937284925	0.178546000000001	0.406087692098599
CTPS2	-0.0260360828418067	0.948361405939703	-0.0431433090510622	0.950119302534786	-0.0895596666666663	0.737398208375175
LY6H	-0.0262906813410713	0.947546118619241	-0.446950362296012	0.178543100892855	-0.198026333333333	0.467798588118316
TSR3	-0.0263418766645955	0.947449091402114	0.0817174332905597	0.89548747312664	-0.0107656666666658	0.97184501043952
PRSS8	-0.0263467646318761	0.947449091402114	-0.157449927758718	0.767770230680061	-0.127909999999999	0.596924734519002
CCNB3	-0.0263824995474829	0.947338405699436	-0.455918148535271	0.167648587973919	0.111388666666668	0.661470161772113
TAB3	-0.0263921455611884	0.947338405699436	-0.0995689199935599	0.867603998426271	-0.238422666666666	0.220405584107617
MS4A5	-0.0264263190790682	0.947209241800167	0.0836482836698563	0.892833228561937	-0.0793049999999995	0.725570231024154
ANGPTL6	-0.0264496759345169	0.94713629491249	-0.0907220967331538	0.881323965029954	-0.0770983333333328	0.827433678445379
LRRC37A5P	-0.0265819420140965	0.946682046744321	0.0688414330906393	0.912149839732916	-0.0519629999999998	0.831668247201974
KDM4D	-0.0265827506232933	0.946682046744321	0.144622682513884	0.790044831580319	-0.0192756666666657	0.949295980201336
CCR1	-0.0266554978369756	0.946504865283881	-0.316442686900185	0.432844809779835	-0.253583333333333	0.190246103576351
ERGIC2	-0.0266776396443271	0.946459472008867	0.112139444572413	0.847454473507881	0.109617000000001	0.603463436419421
UVRAG	-0.0267216212948666	0.94637057203132	-0.205334977616008	0.67311221282925	0.160831333333334	0.480396469408299
DPCD	-0.0267366573960299	0.94637057203132	0.228503392287809	0.623107525588402	-0.119838333333332	0.592006286402195
SLC27A4	-0.0267650156111324	0.946367595948977	-0.102915946162662	0.86257932725575	0.059617333333334	0.831360434244133
CCR6	-0.0267747067555687	0.946367595948977	0.0099248457594237	0.9888083127414	-0.0212833333333330	0.94692575385424
UPK2	-0.0267887108654844	0.946367595948977	0.0359517985321486	0.95830581556695	-0.128323333333332	0.640398707036842
OR6A2	-0.0268171386668260	0.946314173776292	0.218749120675805	0.642137448592891	-0.0209226666666667	0.958098397518313
ZNF266	-0.0268356147822709	0.946269338137272	0.280943022094825	0.507259109959297	0.0634790000000008	0.789105818888855
DNAH10	-0.0269172807954052	0.94604508400373	0.127079390114509	0.820197496905935	0.0401985000000004	0.871476278070153
RBM38	-0.0270142899169599	0.945732032736787	-0.276279857620071	0.51752420454074	0.0809600000000007	0.752518458717797
RRP36	-0.0270255145997615	0.945729091271474	0.0737356508353425	0.905506084101768	0.0288876666666681	0.924482212111412
CABLES1	-0.0270325236017165	0.945729091271474	0.335893314452897	0.38984602770636	0.150112666666668	0.506814416082488

GFM2	-0.0270608387565669	0.945700145502931	-0.0548974248580441	0.934255557320745	-0.272018666666667	0.171771824059364
RNF207	-0.0270905168811000	0.945671866100197	-0.124503798315921	0.824974271393593	-0.0223173333333329	0.943676450608251
CD226	-0.0270961590628948	0.945671866100197	-0.114044230238173	0.844272085764738	0.184778000000001	0.528501553687416
WDR67	-0.0270975552056167	0.945671866100197	0.0598980482659245	0.926038115028264	0.229327333333334	0.272442920905288
ZNF229	-0.0271051267421961	0.945671866100197	-0.247244423602633	0.580200878259622	0.145030666666668	0.516217427447249
CYP4F22	-0.0272353863392019	0.945258840240937	0.232644881199436	0.61200913457472	-0.0142369999999995	0.966976043849334
ENOPH1	-0.0272551945274035	0.945206214944512	-0.134222891473662	0.808009477165608	-0.0329386666666657	0.902176097172346
MGAT3	-0.0272627178250772	0.945206214944512	0.202109789811401	0.680054545720343	-0.0339800000000001	0.895093529187836
IFT122	-0.0272672475302127	0.945206214944512	-0.162286879300112	0.758164869707887	0.0688846666666675	0.794486775271372
PTGS1	-0.0272694913310456	0.945206214944512	-0.172602159625026	0.738421727152922	-0.487041666666666	0.138412505594457
TMEM214	-0.0272941396628228	0.945206214944512	0.164596318193196	0.754105947879839	-0.186481666666665	0.338754799084584
TUSC5	-0.0273413457403478	0.945141635093826	0.218264290180481	0.643298063500002	-0.162106666666666	0.66807888867384
STK35	-0.0273849308333421	0.944951355101247	0.21600506316552	0.64844945813838	-0.388261666666667	0.107866708545887
PRPS1	-0.0273920412778967	0.944951355101247	-0.367085189460832	0.327869177739765	-0.160657999999998	0.445566557024313
FUT9	-0.02740407259405	0.944951355101247	0.153091495376773	0.774491384468427	0.0514453333333338	0.827433678445379
FOXO1	-0.0274544033836826	0.944797210581258	-0.221610862462567	0.637480881894777	0.056951	0.84100404224146
ICAM3	-0.0274609175044791	0.944797210581258	-0.115323339144718	0.843755596233057	-0.104018999999999	0.619712416873704
DHRS11	-0.0275043514580676	0.944632044359781	0.316318927912760	0.432990247663455	-0.0645483333333326	0.783223894269194
LARS2	-0.0275173582817030	0.94461874366458	-0.114642068137589	0.844162087612813	-0.045885333333333	0.843152489417868
PCMTD2	-0.0275324421411357	0.944613500470726	-0.0496384716657903	0.941529432256713	0.246053857142858	0.516715160469872
MSH3	-0.0275342255045768	0.944613500470726	-0.427512709337176	0.212163576776299	0.066797666666668	0.765020657072822
ZNF324B	-0.0275850757686642	0.944536690235707	0.404825503754951	0.25480455106032	-0.0266376666666661	0.922463263763
NHLH2	-0.0275968768279848	0.944530368972062	0.221295828844855	0.637956894209002	-0.0607836666666665	0.837759479233448
OTUD1	-0.027637609116091	0.94435647376786	-0.204139917145349	0.675320698349846	0.247127	0.206303581777675
RPL19	-0.0276423060782302	0.94435647376786	0.122234801458978	0.829569971668386	-0.0269006666666656	0.919420614501031
DTD1	-0.0276819727851969	0.94429278400804	0.195386387332405	0.694303704398562	-0.159589666666666	0.407050483403022
MRPL43	-0.0277087282742994	0.94429278400804	0.234944278404555	0.607849541498715	0.146786000000000	0.528409919089401
CLEC4G	-0.0277180490393861	0.94429278400804	-0.0561225600453687	0.932652183149857	-0.0496810000000004	0.86011149680202
CDKN2B	-0.0277442064555567	0.94429278400804	0.196127450681526	0.69221985268187	-0.00177733333333344	0.995735546504905
HSF4	-0.0278088183665432	0.944134292623551	0.137275705212657	0.802456421819405	-0.0791243333333327	0.745324730039331
WDR59	-0.0278602462775661	0.943983261635717	0.101890608878516	0.864616357134757	-0.00401933333333258	0.989386930304526
ZNF485	-0.0278732517243289	0.943983261635717	-0.123647589062255	0.826475556843795	-0.218930333333333	0.365126455342703
RPL34-AS1	-0.0279037661883054	0.943983261635717	0.324787942553452	0.411071217323391	0.124886000000000	0.610873928204162
ATG4B	-0.0279048476670601	0.943983261635717	-0.0103940070616754	0.98797403729164	-0.1262055	0.534631074286033
CRBN	-0.0279223772379047	0.943983261635717	-0.223396932691619	0.63471624724109	0.066000333333334	0.766132481270445
NME3	-0.0279293439932623	0.943983261635717	0.123632555143578	0.826475556843795	-0.0657366666666672	0.860569150331002
MIOX	-0.0279447536313045	0.943983261635717	0.156614949669989	0.768867001180986	-0.049518333333333	0.89117923971418
CLDN5	-0.0281584683937461	0.943198967841961	-0.196007527439891	0.692325064566574	-0.0889896666666667	0.6846855637534
OGFR	-0.0282858887321393	0.94283227358722	-0.0156488882408990	0.982266426585123	-0.0118333333333323	0.970094446214144
PARP9	-0.0282904415577878	0.94283227358722	-0.359921370043516	0.341510139061518	0.228257666666668	0.264616961153914
HSPA1L	-0.0283625937304208	0.94267153471507	0.176287883337981	0.730407919148629	0.0414650000000005	0.9061074113295
RGS9BP	-0.0283876220202986	0.942614459334878	0.104568882263805	0.86067831050617	-0.040238666666666	0.874908057954497
RGS9	-0.0283916185254850	0.942614459334878	0.277340284755833	0.5144731963081	0.132034333333334	0.528409919089401
PLA2G3	-0.0283976401460612	0.942614459334878	0.00511352422782694	0.99251028338798	-0.436011333333333	0.0863442910203928
ZNF423	-0.0284071500159333	0.942614459334878	-0.144730096103298	0.789758130538055	-0.093346666666666	0.688145991467449
NOL3	-0.0284758335398867	0.942350859359627	0.268315517523610	0.535217216332192	-0.111584000000000	0.585987887254773
CD2	-0.0284780182254571	0.942350859359627	-0.340141021139204	0.379495306350778	0.0640650000000004	0.780512234657452
DENND4A	-0.0285142280487418	0.942350859359627	-0.0699126926733796	0.910885619751085	-0.0823420000000001	0.74850706128476
CTXN3	-0.0285548995656825	0.942326541903048	0.0859821237086928	0.888306347447303	-0.0617439999999998	0.785482517274791
OXER1	-0.028560384984293	0.942326541903048	0.0895231819538609	0.883258404969667	-0.126522999999999	0.701212063387352
SDC2	-0.0285714867664676	0.942326541903048	0.0472732837840033	0.943981149721771	-0.0213523333333307	0.946392308016584
WBP2NL	-0.028598163370645	0.942326541903048	0.0577490904199615	0.929758936765496	0.170410000000001	0.485563739672775
SVOP	-0.0286993165454171	0.941922706455035	0.0806145826573114	0.896754372944873	0.151929666666667	0.574404052938371
RAC3	-0.0287061678788338	0.941922706455035	0.0215693799382701	0.97536722461972	-0.110505666666666	0.592716584553539
KCNE3	-0.0287161876305482	0.941922706455035	-0.00605989158056327	0.992450441941654	-0.0065146666666667	0.985230239434352
FXYD5	-0.0287978361538635	0.941723156087002	-0.336520512360884	0.389099686779674	-0.0610936666666658	0.78757492538011
MAGEE1	-0.0288089340366828	0.941723156087002	-0.287713905040635	0.492562582944134	0.0607486666666673	0.790270367828216
FYTTD1	-0.0288203344533613	0.941723156087002	-0.133522852556735	0.808933084408149	0.00834500000000024	0.980779858267938
C16orf86	-0.028851269628403	0.94166122657299	0.192998589771188	0.699138815299348	-0.0999663333333318	0.72926287896686
ADAM15	-0.0289562694961049	0.941299622985211	-0.339164496229168	0.382766745714799	-0.0204443333333315	0.939118632618649
HIRIP3	-0.0289884420498477	0.94117478754407	-0.0261621294087265	0.970632644458507	-0.114267999999999	0.612697324680132
OR51B4	-0.0290208104748622	0.941048804860598	0.0985064801891046	0.868278775296491	0.0196190000000005	0.944218475609619
KIAA1429	-0.0290247554151524	0.941048804860598	0.00076702937203404	0.99887348584855	-0.199791666666665	0.32631478390653
PPEF1	-0.0290273713206267	0.941048804860598	0.319303951213002	0.426142369255984	-0.033492333333333	0.896446774489185
GALP	-0.0290423955951473	0.941048804860598	0.207026565652215	0.669713208801491	-0.086542333333333	0.684569287653569
ASB9	-0.0291109609967745	0.940998176312325	-0.0845965657388085	0.891547139719321	-0.103042333333333	0.629657237462109
CHDH	-0.0291447473796859	0.940887569244923	0.0666142774104215	0.915828699649446	-0.0336136666666658	0.902859284837213
SLC5A9	-0.0293674982286429	0.93984100036726	-0.165059054054432	0.7529423475214	-0.118468666666667	0.657217907037306
CDH4	-0.0294048835559407	0.939685734987777	-0.292645542182881	0.483287060229305	-0.189078333333333	0.476488454941569
DCUN1D2	-0.0294326793878707	0.939648297270262	0.148207996779834	0.784080588385358	-0.0877326666666666	0.674028958837018
LEO1	-0.0294977015287419	0.939394389673704	-0.225696319230517	0.629536581746323	0.140726000000001	0.512796060754107
AP2A1	-0.0295706267554796	0.939156562363169	0.295186783925481	0.478475354656325	-0.00605999999999951	0.98647849803298
TEX101	-0.0295859209500771	0.939129697183682	0.157169513554979	0.76810607001971	-0.122095000000000	0.544685122345675
PRL	-0.0295886024045674	0.939129697183682	0.261360777878074	0.552187117311017	0.0940716666666667	0.673514912541661
TUBB4A	-0.0295915403282393	0.939129697183682	0.471593015868945	0.146991015246019	0.0910759999999997	0.738948205923422
GALK2	-0.02963122594544	0.93911454044388	0.0651880284661607	0.918436427185622	-0.0586113333333328	0.812230603035278
BPIFC	-0.029748681135646	0.938493430715422	0.203055617277817	0.6772404821028	-0.119088000000000	0.612943770867845
NPLOC4	-0.0297516602154687	0.938493430715422	0.105762951039525	0.859545278949543	-0.0483933333333331	0.838927397112197
HMG20B	-0.0297519908361496	0.938493430715422	0.191360289134412	0.701753730088091	-0.0362933333333326	0.887363245747406
TRIM23	-0.0298668887819030	0.938240233739979	-0.285992507894781	0.496429042723156	0.132360666666667	0.576277303044571

TMEM35	-0.0299411119851886	0.938056642905692	-0.0473757205908911	0.943981149721771	-0.126781333333333	0.546467222253371
CHRM3	-0.0299441386365284	0.938056642905692	-0.312203696869702	0.440385292108219	0.14364	0.481899892112649
LOXHD1	-0.0299846786852087	0.937989337941006	0.171137436938329	0.740062065190296	0.0190286666666677	0.946623914607177
TACO1	-0.0299897305029794	0.937989337941006	0.0255912020389107	0.97204937337922	0.0279216666666677	0.914256893620689
CYP4A11	-0.0300174209504373	0.93798502749796	-0.182855325930695	0.718289071039953	0.192766666666667	0.453167962703614
CARD9	-0.0300201902652409	0.93798502749796	-0.118301131141083	0.838117566591658	-0.0600069999999995	0.85013576481729
ZUFSP	-0.0301219230376210	0.937687146491655	0.225888334288009	0.629121375721303	0.209102000000000	0.490929966167076
SPINK2	-0.0301417376702784	0.937633878183051	0.00833678307915131	0.989949154584002	0.0215450000000001	0.94622978991441
PIM3	-0.0301681246711271	0.937562770540962	-0.386078049735042	0.289470192870154	-0.205396333333333	0.301708243694309
SHOX2	-0.0301829255887405	0.937562770540962	0.0523364907452694	0.937831566264837	0.0939650000000009	0.705698790800484
TRAF1	-0.0302033167322748	0.937562770540962	-0.332402160105593	0.398142341492687	0.0599916666666677	0.803481724951494
TSPAN9	-0.0302107365530350	0.937562770540962	-0.242901793883145	0.592178030679976	-0.311043333333332	0.175476022490732
ZNF124	-0.0302312228830246	0.937562770540962	-0.265499343926336	0.54048517038548	-0.00646966666666668	0.9879739922351
VHL	-0.0302339790906545	0.937562770540962	-0.214274633948252	0.652991165086813	-0.0889453333333323	0.703878203604573
FDX1	-0.0302746310308023	0.937562770540962	0.257439771049689	0.563116080558022	0.146956666666667	0.458006502146897
SERPINB4	-0.0302871090319373	0.937562770540962	-0.30746736268541	0.452689746287922	-0.129217999999999	0.600146208272329
DENND1C	-0.0303137606226853	0.937502503190248	0.135108449956799	0.807470535721699	-0.132553333333333	0.754015160951226
KCNK6	-0.0303263045743347	0.937502503190248	-0.440387706697761	0.189370436823798	-0.235875333333333	0.343572153228906
DESI1	-0.0303419961157666	0.937502503190248	0.261605571098787	0.551406891797878	-0.181910333333333	0.370661878103321
CCL4	-0.0303771514791679	0.937502503190248	-0.414133498576857	0.238217967147046	-0.168192444444444	0.481899892112649
ALDH7A1	-0.0303840082149004	0.937502503190248	-0.005818923276754	0.99251028338798	0.126192333333334	0.631961600239811
VEGFC	-0.0303985146244182	0.937502503190248	-0.0371991807498642	0.956300346368081	0.141036000000001	0.794486775271372
ITSN1	-0.0304834430072213	0.937356579300458	-0.298324511363165	0.471509618848387	0.235628000000001	0.207686482144783
RNF112	-0.0304888975900011	0.937356579300458	0.237195708989595	0.603350164283107	-0.0613643333333327	0.80841656016322
ACIN1	-0.0304904568887823	0.937356579300458	0.152212036407222	0.775863712880333	-0.0518159999999995	0.840616234128479
OR1Q1	-0.0305034975886333	0.937356579300458	0.247778405105731	0.579471329472972	-0.0603676666666664	0.816054928507559
MTF1	-0.0305238754862556	0.937356579300458	-0.313660556518875	0.438046311563112	-0.0194183333333323	0.958988235991247
CWC27	-0.0305331820523596	0.937356579300458	-0.350445058127016	0.358430460089679	-0.0537006666666655	0.826033987348661
MCOLN2	-0.0306054970251863	0.937356579300458	-0.0642428804910884	0.919923865590619	-0.396429999999999	0.048170505761132
TRABD	-0.0306900469142871	0.937173475271833	0.0520720182998086	0.938067483409541	-0.136156666666665	0.599605187020528
GNG5	-0.0307099190160556	0.937119764299634	-0.326948996962747	0.408851707589499	0.333090166666667	0.0852927646341535
KPNA1	-0.0307540713671586	0.936986605287477	-0.106951123810275	0.857671866148626	0.0164273333333354	0.95285450244329
DOK6	-0.0307562740258755	0.936986605287477	-0.0172760347377011	0.980723199123135	0.0930140000000007	0.688145991467449
MUM1	-0.0308177556270169	0.936863928068497	0.075222569600496	0.904173044514532	0.136619333333334	0.637570808030689
MUS81	-0.0308386060074316	0.936804480933644	-0.155061357532006	0.770708441022583	-0.0159953333333323	0.954021566744527
B3GNTL1	-0.0308484073687049	0.936804480933644	-0.121608046190354	0.831310831659304	-0.0152348333333325	0.961665293425945
ANGEL1	-0.0309227613963503	0.936437752880266	0.113756607569177	0.844907358669798	-0.125409666666667	0.526072695137576
VIL1	-0.0309234565181599	0.936437752880266	0.106660764029116	0.857937967416038	0.109879666666667	0.590841338570121
CASP5	-0.0309944637551732	0.936268145056359	-0.3176398220845	0.431016140580857	-0.0294326666666662	0.910704531889447
ADRB1	-0.0310330662505645	0.936105017437037	-0.07344741142753	0.905838142287595	-0.0922963333333337	0.721454370213253
SST	-0.0310386974358844	0.936105017437037	0.0564793995615555	0.93176886339657	-0.0148686666666663	0.967241125442187
LOC441178	-0.0310533039583399	0.936105017437037	0.197253486482429	0.689280772804818	0.248262333333333	0.340052241399374
SMCP	-0.0310680678689939	0.9360873824976	0.295378481675354	0.478306608395718	-0.0526703333333329	0.816054928507559
HIST1H2BB	-0.0310947503153690	0.936056075812983	0.0571281921293621	0.930775493791569	0.138555666666667	0.734593656619209
BEST4	-0.0311152562357561	0.935998580120428	0.087917698622451	0.885848000380851	-0.0144026666666664	0.965329851466378
ZCCHC18	-0.0312081356632326	0.935597314043919	-0.239992798762031	0.598092982260025	-0.0724489999999997	0.788732255334066
SIX2	-0.0312169055195273	0.935597314043919	0.22508284458302	0.63103711743427	0.0496576666666681	0.83726290347191
L1TD1	-0.0312308896225552	0.935597314043919	0.108113927440602	0.855467857668328	-0.0793966666666666	0.707966006319856
DKFZp779M0652	-0.0314617642037838	0.934783739533187	0.191486025698968	0.701635983922402	-0.0426413333333327	0.888347984740063
SDHAF2	-0.0315014698401415	0.93473502060002	-0.160012811619062	0.762902034786947	-0.182388666666665	0.342250925467046
OPN1SW	-0.0315509524009627	0.934698267085849	0.283234856352545	0.502129505146473	-0.00866899999999995	0.982375192575954
BOP1	-0.0315718257435731	0.934638526910487	0.243081015790025	0.592178030679976	-0.2302215	0.242374255107433
C21orf2	-0.0315822844203315	0.934638526910487	0.183785177147410	0.718067440349544	0.0319120000000009	0.907096813604642
TSHZ2	-0.0316381840315524	0.93458301766211	-0.0459371969513859	0.945364757579857	0.153854500000000	0.671417957355308
JMY	-0.0316814260636	0.93458301766211	-0.106231791820674	0.858459121671731	0.0238476666666678	0.947020470290463
PAX7	-0.0317081705005791	0.93458301766211	-0.148434121891024	0.78374265784053	-0.0114273333333323	0.983159241812861
JHDM1D	-0.0317370958658373	0.93458301766211	-0.552941948249671	0.0591423760225182	-0.423895333333334	0.273327127062166
LRIG3	-0.0318769583914803	0.933975285415805	0.187434512024168	0.709286896761914	0.0404753333333344	0.90595687094025
C19orf54	-0.0318823536042091	0.933975285415805	0.127237188542693	0.819791676572254	0.0603060000000012	0.809704159744507
ALX1	-0.0318824669387964	0.933975285415805	-0.132014770660113	0.810300414153187	-0.0371769999999992	0.9061074113295
MBNL2	-0.0319034064095864	0.933975285415805	-0.0267479129108323	0.970207612777188	-0.00358399999999991	0.99209221913287
IQCG	-0.0319085516428041	0.933975285415805	-0.0876265527532532	0.886302037299497	0.0713903333333344	0.766132481270445
SLAMF6	-0.0319301748986024	0.933975285415805	-0.0514074530661952	0.938729151196048	-0.0149229999999997	0.962924081933534
CFLAR	-0.0319516316671811	0.933912205024486	-0.382371468439019	0.295293127580539	0.068127333333334	0.79107229830676
SNORA74A	-0.0319557806834691	0.933912205024486	0.0496787438556452	0.941458038227918	-0.232352	0.367756122563697
PRG3	-0.0320739395664079	0.933565145784872	-0.00449451110182559	0.993885762793588	0.0613796666666676	0.811623820117383
MYO3B	-0.032095097575208	0.933565145784872	-0.181061363057048	0.72163737037735	0.0957110000000007	0.778083808586846
ZNF230	-0.0321817314589888	0.933375619204777	-0.137616170042104	0.801951364081469	0.101026666666667	0.742452599762094
LY6K	-0.0321835743454233	0.933375619204777	-0.311184970930811	0.443419531318142	-0.0872793333333334	0.769387487364442
ZNF862	-0.0321847481910808	0.933375619204777	-0.0379480343218924	0.954889910016503	0.0824770000000004	0.719839702715467
SLC8A1	-0.0321962378906891	0.933375619204777	-0.0546607745521657	0.9344692810234	0.527909333333333	0.0379238760858006
ZNF204P	-0.0322368728958257	0.933375619204777	0.123042810307804	0.827904654392021	0.952018	0.00421637924026936
FAM71B	-0.0322555358155032	0.933375619204777	0.0306378975686128	0.965640547092659	-0.0267533333333329	0.921287736444926
LOC149134	-0.0322671398785162	0.933375619204777	-0.0912738392446723	0.880426986815658	-0.0318596666666662	0.913360847453956
TTC26	-0.032272476727132	0.933375619204777	-0.165607981398539	0.752352242644389	-0.0831409999999999	0.696927295330661
TBC1D10A	-0.0323316326613108	0.933245931443092	0.0228728291016657	0.97536722461972	0.00785900000000017	0.983159241812861
EDF1	-0.0323887606397081	0.933160743662018	-0.0544114093572356	0.9344692810234	0.0136033333333349	0.962620058410129
SMC4	-0.0324165739706251	0.933160743662018	0.0147856211803263	0.98327723682819	0.321786333333334	0.124096550131011
WDR46	-0.032463345492669	0.932973246551555	-0.197675076276406	0.688640225556359	-0.226782000000000	0.241273257416922
CTSS	-0.032525077546787	0.932736263352902	-0.203321241742656	0.676646782959283	-0.595614666666666	0.031687055072673

ALG10	-0.0325264790780361	0.932736263352902	-0.075545545383224	0.903517361619051	0.130927333333335	0.527465067495661
ZBP1	-0.0325693209006137	0.932705009144033	0.075802668504471	0.903510413986113	-0.153246666666667	0.488602547609567
CEACAM3	-0.0325873770676847	0.932683045551196	-0.0069357020250806	0.99129507664977	-0.071408999999999	0.741134448923316
PSG5	-0.0326324029322506	0.932481518868208	-0.0603398636102329	0.925649366494154	0.0391753333333339	0.883878395614985
ITGA6	-0.0326692809750712	0.932327739050086	-0.190860828961489	0.702644983417708	0.256005000000001	0.183344879272687
KIAA0922	-0.0327273542510921	0.932090580397175	0.216175701276656	0.648391670659269	-0.382289333333333	0.216663334151092
ZNF157	-0.0327451980020803	0.932069847380926	-0.200980603863481	0.680143077995217	0.172854333333335	0.388303383752735
LMTK3	-0.0328665688663254	0.931613552577265	0.127625737809314	0.818717515586031	-0.142141999999999	0.519046309802939
PUF60	-0.0329039549841303	0.93157566591952	0.188697029073318	0.707361603786947	-0.0773766666666659	0.718749100405876
ZNF346	-0.0329222088501548	0.931569698821572	-0.00402917446488234	0.994485841304070	0.0845026666666678	0.728429595651334
GEMIN7	-0.03293902067558	0.931557153216692	-0.0578052470225024	0.929715247340158	-0.107347999999999	0.633166039956913
ZFPL1	-0.0329807624532561	0.93137473857549	-0.119147453660395	0.836702107561498	0.109029000000001	0.592716584553539
REG1A	-0.0329864554804717	0.93137473857549	0.0396271822927546	0.953729831316741	-0.190246999999999	0.426518032099216
ZNF19	-0.0330524727495299	0.931190352491494	-0.166978324882453	0.749950473710419	-0.0728219999999999	0.752992371482229
SERPINA9	-0.0331057348135205	0.931190352491494	0.0814017460547604	0.89615326639288	0.00666900000000039	0.983159241812861
FMR1NB	-0.033158543822509	0.931115405456017	0.29655484399472	0.476296748214996	-0.0212566666666655	0.94375604597120
TWIST1	-0.0331615004425727	0.931115405456017	-0.0106169364065467	0.98774587825743	0.0927783333333338	0.68951218434739
KLK4	-0.033178566788403	0.931115405456017	0.0262949879158582	0.970631762839502	-0.465365999999999	0.0895671235838677
CRY1	-0.0331936216006023	0.931115405456017	-0.146883289084734	0.785920387079635	0.141600666666667	0.481989564291462
C17orf82	-0.0332025388257236	0.931115405456017	0.574560510706705	0.0442317804409017	-0.0818013333333335	0.732261272587549
C10orf91	-0.0332632186917573	0.931090302117051	0.314432387471852	0.437474636732405	-0.166991666666666	0.397008798616527
PRNT	-0.033268940687849	0.931090302117051	0.170764358355582	0.741154777036791	-0.0940859999999997	0.664617185770066
APOBR	-0.0332867948869606	0.931076774888206	-0.113943306019489	0.844272085764738	-0.0677089999999998	0.814273596067945
MAPK8IP3	-0.0332995878801925	0.931064111470657	0.134548137670787	0.808009477165608	-0.0568229999999993	0.823910138699963
MMP10	-0.0333816347012684	0.930894598428973	-0.00463915474191454	0.993478880305783	0.095668000000001	0.720466212913449
CCER1	-0.0333979580491415	0.930861194157046	0.0108223931331025	0.98774587825743	-0.0153679999999993	0.955620005467488
PTH	-0.0334099224436533	0.930853388901026	0.091924546357585	0.879153312143409	-0.0949693333333333	0.671124336259655
GTF2H3	-0.0334148980474118	0.930853388901026	0.00128542418230581	0.998348925592729	-0.0768853333333332	0.724220168429137
GPM6A	-0.0334188552067813	0.930853388901026	0.104437936404118	0.861104196969571	0.0829123333333333	0.757964857510564
SLC7A2	-0.0334456539385224	0.930830923888817	-0.161411416227914	0.759262381997816	0.192788000000001	0.351839993215702
TAS2R9	-0.0334633644002794	0.930789364495334	0.0987461976669893	0.868042734188964	-0.0703573333333328	0.787587683071905
APH1A	-0.0334957530584588	0.930666291692353	-0.217777455264846	0.644743872041817	0.188821666666667	0.340341227423177
BPIFB6	-0.0336060744131333	0.930430448360306	-0.241204592809227	0.5961307882797	0.198686333333334	0.332502783413088
DDX25	-0.0337380060010804	0.929925363719654	0.0500719791246007	0.941330035957713	-0.131674999999999	0.531199471764537
CC2D1A	-0.0337593024844137	0.929925363719654	0.0588347678270642	0.928306114846356	-0.0872789999999988	0.684273678433179
SCO1	-0.0337990191333196	0.929816667616756	-0.140136316805997	0.797441782082887	0.054900333333335	0.809409373518692
FAM167A	-0.0338140351178762	0.929790884303285	0.169859450696259	0.743328907676018	0.0408180000000002	0.895510661232872
CCR9	-0.0339017949921099	0.929690657599372	-0.253414031194683	0.567705016149998	-0.0374316666666662	0.902194685260117
FLJ45825	-0.0339024491575393	0.929690657599372	-0.0510372538720205	0.939432580299312	0.386431000000001	0.341727195150614
LTB4R2	-0.0339163406243682	0.929690657599372	0.182869054732933	0.718289071039593	-0.0705696666666659	0.76368723681351
LMO4	-0.0339549794079119	0.929690657599372	-0.330815576455991	0.401809167762213	-0.458401666666666	0.104464051521211
TNF	-0.0339596334796785	0.929690657599372	-0.0933456337352848	0.876653644624479	0.00666166666666772	0.987633631970435
TSFM	-0.0340645866358488	0.929373482972217	0.0169361374512475	0.981160038076789	-0.159464999999999	0.427099983332003
GPRIN1	-0.0340730233914795	0.929373482972217	-0.0690094930391422	0.91204845019337	-0.219746999999999	0.285116883835521
LIMS2	-0.0340807093474569	0.929373482972217	-0.224024704304170	0.633436730790476	-0.150397999999999	0.576277303044571
KRTCAP3	-0.0340812612375978	0.929373482972217	0.238818246598054	0.600563482790016	-0.12463	0.717340634522568
TTC17	-0.0340919548693002	0.929373482972217	0.155862756679358	0.76972820876143	0.0425946666666674	0.865655368707012
TRIM65	-0.0341170953707759	0.929373482972217	0.0951146784188764	0.872962271153999	-0.0593466666666662	0.799204212638847
ENDOG	-0.0341486554631095	0.929358109891413	0.143170706934654	0.793064973612007	-0.0968613333333318	0.662666815705326
CTSF	-0.0341619355082914	0.929358109891413	-0.282913080602132	0.502129505146473	0.279098999999999	0.223260757343954
CCT7	-0.0341633340410886	0.929358109891413	0.161469925244046	0.759189376564965	-0.259173333333333	0.167526000762082
USP5	-0.0342363829307277	0.929358109891413	0.153036833333861	0.774500444636875	-0.157839000000000	0.422927358730594
MAPT-AS1	-0.0342373386869229	0.929358109891413	0.215018334175283	0.651257878577277	0.0162020000000004	0.965661221820942
SLC45A4	-0.0342841690226880	0.929358109891413	-0.0215629872270915	0.97536722461972	-0.105712833333333	0.76307213318662
SH3D19	-0.0342902407318818	0.929358109891413	-0.197806484840958	0.688295576981474	0.349309333333335	0.178162638616424
C7orf61	-0.0342999389682181	0.929358109891413	-0.232921812352778	0.611711680668619	-0.123982666666666	0.569721277878681
BACE2	-0.0343004258422310	0.929358109891413	0.0627701046427323	0.922350559812086	0.026628666666668	0.915761046438965
CABP2	-0.0343837280424837	0.929358109891413	0.00287283063198995	0.995437468924912	-0.155190333333333	0.608311546956177
OCRL	-0.034418642397953	0.929235833666713	-0.314845569495619	0.436641260893239	0.0975936666666671	0.657353585270084
C22orf31	-0.0344285553343833	0.929235833666713	0.183000543475661	0.718281994122754	-0.00601299999999972	0.986471172041113
NFKB2	-0.0344748688358799	0.92909247680907	-0.443417159757806	0.1843534401377	-0.0202066666666655	0.941559580750404
TNIP3	-0.0344946580341677	0.929038514329255	-0.171564005071867	0.739517143964292	-0.138430000000000	0.551040195813256
CALR3	-0.0345618191118135	0.928827822578361	0.0506852346322353	0.940271086498237	-0.0119653333333331	0.972261709812365
PPP6R1	-0.0345879971399185	0.928827822578361	0.221002210579675	0.638427112536057	0.0318170000000014	0.904018282943947
NKX3-2	-0.0346643266476972	0.928727731719557	0.457352461647345	0.166671273245692	0.0990723333333335	0.706573150130005
NEUROD6	-0.0346890497797318	0.928698098804835	-0.0594324565340394	0.92694538699714	-0.142077666666667	0.538968176654309
PIGZ	-0.0347007958290522	0.928698098804835	-0.114046502989134	0.844272085764738	-0.237818333333333	0.210845715069665
DPY19L3	-0.0347222629380546	0.928698098804835	0.103345805573379	0.862065338914705	0.420499000000001	0.0727726679861762
SCARA5	-0.0347549930342933	0.928631908360351	-0.182062027365233	0.71977894194971	0.168980666666667	0.481969285852349
TTC31	-0.0347674405533850	0.928631908360351	0.139338085016413	0.798615265561297	-0.00409499999999828	0.991175862027014
RPS6KA6	-0.0347938036404749	0.928591109465333	-0.346664623248965	0.365167159367406	0.509814333333333	0.128957627033936
SPO11	-0.0348154194539275	0.928589026536108	0.187774184253472	0.708377771996406	0.00515433333333376	0.9879739922351
NRK	-0.0348725943741014	0.928439877590857	0.0344219151920645	0.959404955370931	0.301270666666667	0.114599211633843
PAGE5	-0.0348778804488482	0.928439877590857	0.31184833635141	0.441314096241515	-0.227995666666667	0.499554381454891
PSMD7	-0.0349092434169320	0.928411812886386	-0.205204130737592	0.673296856274784	-0.0695866666666657	0.748388402768556
PNOC	-0.0349206091704467	0.92840747747733	-0.0411190006388193	0.952102539249605	-0.110988333333333	0.648381288631965
MET	-0.0349233054002250	0.92840747747733	-0.5150922804519	0.0944627871415069	0.171108000000001	0.417125112561133
GSG1L	-0.0349313293743948	0.92840747747733	0.145355579798573	0.788068309146682	-0.0754450000000002	0.824622063444784
POLDIP2	-0.0349365176577827	0.92840747747733	0.150166782557083	0.779734405920598	-0.128393666666667	0.537725274785612
PLAC1L	-0.0349548946061463	0.92840747747733	0.0217730353357281	0.97536722461972	-0.00926233333333277	0.983159241812861

SLC16A4	-0.0350592612690283	0.928091078025516	-0.0185204989983168	0.97902968742576	-0.155364	0.622282705325595
NODAL	-0.0351453944200345	0.927750320951316	0.059770560610936	0.926352922534113	-0.0504196666666657	0.83575292572393
MGMT	-0.035194399568027	0.927750134382795	0.095734010606548	0.872537273851412	0.0191870000000008	0.94678260229888
CYP1A1	-0.0352063096531722	0.927750134382795	-0.118817678336461	0.837182040755817	0.195994666666668	0.344576368488222
DCLK3	-0.0352110651878992	0.927750134382795	0.0757821248499051	0.903510413986113	-0.0777613333333331	0.754702252946525
EIF4E3	-0.0352170346624797	0.927750134382795	-0.402917232704395	0.258758728765188	0.246219333333333	0.340052241399374
CA9	-0.0352193608151045	0.927750134382795	0.085247813168189	0.890358825229141	-0.0269316666666655	0.946392308016584
SPAG1	-0.0352203649002263	0.927750134382795	-0.0135447272324730	0.98412883278237	0.0613886666666675	0.793590895155653
GPR15	-0.0352511276436338	0.927750134382795	-0.330868754793021	0.401780307922136	0.0284010000000006	0.91680951061779
METTL6	-0.0353211894205879	0.927750134382795	-0.369918441823928	0.322377391230088	0.0495446666666676	0.82844931076437
C12orf49	-0.0353469094894443	0.927750134382795	-0.459380982118813	0.164444235253496	-0.223066	0.286365632463950
CDK5RAP2	-0.0353704890907845	0.927750134382795	0.0664847375650791	0.916100593047005	0.0778836666666684	0.745685276815889
DPM3	-0.0354212355688785	0.927750134382795	-0.00491241006473137	0.992971227074603	-0.0192683333333328	0.944097469332832
sept-07	-0.0354465129250423	0.927750134382795	-0.0703676261689589	0.910394157189308	0.251726000000001	0.188082633937580
RBFA	-0.0354467479250168	0.927750134382795	0.0788534980930212	0.899154242391172	-0.195961999999998	0.307304414570957
FBP2	-0.0354657126343897	0.927750134382795	-0.00126149851735820	0.998348925592729	-0.154569666666666	0.428946901721219
ERO1L	-0.0354730133031421	0.927750134382795	0.129271139909577	0.814667324632963	-0.0952103333333328	0.657353585270084
OR5V1	-0.0354888897506784	0.927750134382795	0.0479024923319648	0.94314236584312	-0.0828606666666664	0.719304894613107
ANGPTL3	-0.0354964705803982	0.927750134382795	0.122414188971298	0.829398647947819	-0.082582333333333	0.707538975864338
ZNF296	-0.0355010197925646	0.927750134382795	-0.121879025397851	0.830586601001743	-0.0924393333333331	0.768446434650364
SCN1B	-0.0355462585032877	0.927663408115771	0.104809502876009	0.86067831050617	0.0145130000000008	0.970611255509034
RNASE1	-0.0355984581994061	0.927480443459624	0.106018660111912	0.858902068936183	0.360930	0.49971129098408
ST6GALNAC3	-0.0356186872176444	0.927480443459624	0.04535196312236	0.946411712874635	0.108497000000001	0.614081498359193
IFT140	-0.035650520963763	0.927480443459624	-0.0520677280961071	0.938067483409541	-0.0130766666666656	0.967256128104685
COL6A6	-0.0356579148996131	0.927480443459624	-0.413347766460284	0.239647690131862	0.0572013333333329	0.815309621716298
LTA4H	-0.0357053177190366	0.927357414700096	0.0541438079098299	0.934943125030587	0.0816396666666672	0.716517864791776
CLIC1	-0.0357092366278719	0.927357414700096	0.0773815141864179	0.90079793355475	0.0372836666666673	0.895093529187836
TAF7	-0.0357478447747355	0.927357414700096	-0.188329254219242	0.70739176191442	0.100871333333335	0.62420320780168
HELQ	-0.035749806358458	0.927357414700096	-0.185824555516573	0.713765959545662	0.247857000000000	0.246475973365341
HTR1B	-0.0358573382389343	0.927309654428104	0.140942486899112	0.796714214272984	-0.0603770000000004	0.867354667762818
TBX19	-0.0358908665129805	0.927197170376704	-0.191642382646931	0.701609399805282	-0.0510503333333322	0.8257633904865
RIPK4	-0.035892537963998	0.927197170376704	0.119759167553572	0.835531661413366	-0.178150000000000	0.465279557781548
FAM83H	-0.0359136217130441	0.927188353464158	0.623208901212629	0.0233874480131624	0.174394666666668	0.367209658745259
HSD17B11	-0.0359274070345430	0.927169687374104	0.154530964340439	0.771310656070265	0.214472000000002	0.251458682190479
TMSB4Y	-0.0359345942328236	0.927169687374104	-0.0728665605163108	0.906919586620395	0.0663823333333342	0.778655609650856
WTAP	-0.035948286719496	0.927169687374104	0.00381031906963992	0.994588964700772	0.084064166666667	0.696862585388398
ARC	-0.0359554585972186	0.927169687374104	-0.0746797349901027	0.90447571963354	-0.084018	0.835752276645858
MICU1	-0.0360232923628062	0.926979640498277	-0.100709123558557	0.86628046156792	-0.262420333333333	0.27721812275707
CLRN3	-0.0360414871877925	0.92693484432189	-0.0188924065342383	0.978468238339512	-0.055614	0.827433678445379
ASTN2	-0.0360908371886950	0.926825237408712	-0.0147162805730196	0.983444353656142	0.0154230000000001	0.961018173938676
CRIP2	-0.0361088614359804	0.926825237408712	-0.339296537964165	0.382221871047438	-0.00705166666666542	0.983098154995337
PLP1	-0.0361112670912299	0.926825237408712	0.245538326769761	0.584539225718403	1.00703833333333	0.0778643319695396
ALG3	-0.0361176987359354	0.926825237408712	-0.112546315475638	0.847191484945575	-0.191532333333332	0.330590852425541
C2CD2L	-0.036125948432344	0.926825237408712	-0.116258781432880	0.842216714279405	-0.117952333333332	0.574486578500537
FILIP1	-0.0361409419749637	0.926825237408712	-0.263067799569602	0.546774946007658	-0.0119246666666666	0.975988780928093
TRPM7	-0.0361683300916992	0.926825237408712	-0.230197767999759	0.618767132693029	0.0290260000000003	0.925845244747948
RSRC1	-0.0362044863896805	0.926825237408712	-0.100239726317216	0.86681045025669	0.150407000000001	0.477871006905274
ACOT9	-0.0362149010881983	0.926825237408712	-0.468042691045606	0.151936428121882	0.135193666666668	0.523453039510001
EPHA1	-0.0362239331145632	0.926825237408712	0.00347800449907746	0.994730189431381	-0.149096666666666	0.588666179294878
SRD5A2	-0.0362271201562304	0.926825237408712	0.132711341167843	0.809588765729788	0.662582666666668	0.154573540919994
TSPAN13	-0.0362690171292063	0.926825237408712	-0.0767914422396341	0.901856698918763	-0.00104966666666619	0.997953056544486
B3GNT7	-0.0362782642871463	0.926825237408712	0.216015269575405	0.64844945813838	-0.0395343333333329	0.928295508521289
CHMP4B	-0.0362957922790888	0.926814168216285	-0.361696263376339	0.338556116268406	0.00533066666666723	0.987163226136842
UBE2DNL	-0.0363171899145214	0.926813416493938	0.00143694423377872	0.998303187767855	-0.107131999999999	0.728894262229555
EPB41L3	-0.0363188442903975	0.926813416493938	-0.0232704020694588	0.975348968561756	-0.0331853333333326	0.899614658619676
TNNI2	-0.0363482345753351	0.926811979455277	0.208929457499007	0.665595909869988	-0.289773999999999	0.220714782714483
SRSF11	-0.0363593473325941	0.926811979455277	-0.185806733378346	0.713765959545662	0.0946166666666675	0.657217907037306
GPRC6A	-0.0363697599864237	0.926811979455277	0.00865337625313058	0.98946753270812	0.115601000000001	0.578139856834541
ITGAX	-0.0363756536894545	0.926811979455277	-0.279834378462134	0.508808219915286	-0.128991000000000	0.523453039510001
CD5	-0.0363983661511451	0.926811979455277	0.234212296867087	0.608813237170423	0.0576030000000007	0.87629402535202
ST5	-0.0364428667820923	0.92669882290479	-0.218090875581942	0.643724650334922	0.218519000000001	0.3444468741324
CXCR1	-0.0364601570175208	0.926659339354124	0.122972201482912	0.82807427260882	0.0383950000000007	0.890094615232462
MEGF9	-0.0365072742198155	0.926505966200868	0.078779780518633	0.899242938121775	0.190546000000002	0.3353681567844
SLC22A24	-0.0365568005697919	0.926401389300564	0.111877510005825	0.847933931535332	0.0787740000000001	0.771373465365664
RAB27B	-0.0365600206496202	0.926401389300564	-0.0173523605139015	0.980670285500112	0.119214666666668	0.549323798856043
SCT	-0.036564246984516	0.926401389300564	0.235197893930787	0.607340367904098	-0.0628603333333334	0.830093119549048
CYP46A1	-0.0365889395375413	0.926400003141092	0.0505600360721847	0.940582281041682	0.0416586666666665	0.890094615232462
APOB	-0.0365898770240557	0.926400003141092	-0.137224800367470	0.802505565242574	-0.0852336666666664	0.691633384710545
SOX17	-0.0366208371945002	0.926400003141092	-0.291046768337312	0.486880079690803	-0.0761306666666656	0.729912257977231
LAGE3	-0.0366488061541851	0.926343899587079	-0.483856355184668	0.134615454592634	-0.0670209999999986	0.778409880500903
RAB42	-0.0367069261649156	0.92614791567249	-0.0353465732911685	0.95869292666698	0.138790333333334	0.675687379893745
COMT	-0.0367558726822659	0.926092325211593	0.0885579874832876	0.884647486201376	0.114072333333333	0.590841338570121
NDUFV2	-0.0368006725323202	0.92604480662681	0.0507638573487702	0.940077179570464	-0.309086	0.222673436535864
SLC22A4	-0.0368008002907286	0.92604480662681	-0.327325719720729	0.40846136066764	-0.0830766666666663	0.707538975864338
RAPGEFL1	-0.0368623280328528	0.925976472518125	0.188745447191554	0.707361603786947	-0.0735533333333328	0.76648935876098
ELP3	-0.0368626105203213	0.925976472518125	0.204417475062493	0.674817810302676	-0.180567333333333	0.36783045574146
CRYAB	-0.0369752039142009	0.925558545511504	-0.0226558286486229	0.97536722461972	0.138049666666668	0.67674663145704
PDIA5	-0.0370042732418305	0.925448994228934	-0.0756262193047025	0.903517361619051	0.464875333333334	0.031687055072673
GUF1	-0.0370061736102381	0.925448994228934	-0.262082568102423	0.549752906834892	-0.132929666666666	0.560277303308715
TFF3	-0.0370490202524188	0.925309446169217	-0.0164145541713666	0.981892085795004	0.095756333333334	0.709568037614009

SPDEF	-0.0370842058951495	0.925163530610077	0.219143667423683	0.641699006980579	-0.0365086666666658	0.896557410231352
LOC154872	-0.0371148123100175	0.925044818451391	-0.0841251646421048	0.892538205608146	0.110427666666667	0.614081498359193
RGS20	-0.0371414153148073	0.925013051822315	-0.0555920202418515	0.933409139126562	-0.0983269999999999	0.663931919724114
BRCA2	-0.0371443151859818	0.925013051822315	-0.232270042709045	0.612658112747496	0.095237000000001	0.777406710539424
FOXA2	-0.0371499238793553	0.925013051822315	0.428585960760413	0.210173511664670	-0.124682999999999	0.529034261534788
HSD17B3	-0.0371946039278172	0.924924345268357	0.227319990559251	0.62581495163125	0.226248666666667	0.355387672697857
GRIK5	-0.0372388451044962	0.924924345268357	0.134785629725771	0.807867938002244	-0.172104666666667	0.512041798508902
MCF2L	-0.0372470513221016	0.924924345268357	0.130560843693057	0.812271254601992	-0.0447696666666663	0.855716804697638
C4orf45	-0.0372642590096195	0.924924345268357	-0.0961930855076985	0.872108108484887	-0.0657609999999998	0.772139923817113
CTH	-0.0372943128050692	0.924924345268357	-0.112793162632536	0.846997525752467	-0.351272333333332	0.23677255184246
PMP2	-0.0373237877724671	0.924876491388884	0.197447447970756	0.688995325061211	-0.0667299999999996	0.7615367624187
MTHFR	-0.0373703016336336	0.924663320583785	-0.328352705004887	0.407301172667012	-0.211915999999999	0.320592704550458
CHID1	-0.0374125367574086	0.924581588141701	-0.159160351021642	0.764932600126537	0.138139000000002	0.512796060754107
MAP9	-0.0374254687461917	0.924581588141701	-0.0723811408698642	0.907556175729082	0.0565256666666681	0.812617573218469
ANK2	-0.0374705270392307	0.924581588141701	-0.410166861012188	0.246706088283721	0.279580000000001	0.223260757343954
CYB561D2	-0.037522510744935	0.924547559819667	-0.160028736996590	0.762902034786947	0.0804903333333341	0.741438256509195
SLC28A2	-0.0375452253093016	0.924507962758051	0.024822431919242	0.972967477059037	0.0133210000000001	0.96701817021473
GPANK1	-0.0375554149542133	0.924507962758051	0.00282671845096512	0.99552313490055	0.107349666666668	0.621406232563173
HSF2	-0.0375600246926707	0.924507962758051	0.265667842609851	0.539970612810106	0.187463333333335	0.329908083538015
PHTF1	-0.0375741236905316	0.924507962758051	-0.327366428002338	0.40846136066764	0.0824253333333343	0.704370299831112
CA5B	-0.0375881195337994	0.924507962758051	0.090133407817654	0.882249601181978	-0.077991333333333	0.785166558910926
CCDC136	-0.0375939375301097	0.924507962758051	0.227590558769141	0.625175206123754	0.130694000000001	0.670113640649168
PKD1L2	-0.0376204078960924	0.924507962758051	-0.184917101147005	0.715941635290555	0.0390506666666679	0.872963640818847
PRPF40B	-0.0376313255466358	0.924507962758051	-0.0602918613363661	0.925649366494154	-0.115655333333333	0.577524803794493
C2orf40	-0.0376351105278827	0.924507962758051	-0.0952036173051021	0.872962271153999	0.00205299999999973	0.995022374966977
SULT1E1	-0.0376605220565128	0.924507962758051	0.205843776483374	0.672625944768829	0.0777250000000003	0.716057356951225
RAB7A	-0.0377007632986822	0.924507962758051	-0.0892483890123445	0.883460564717377	0.240722833333333	0.280362360296366
IL1B	-0.0377043131702939	0.924507962758051	-0.227047135843713	0.62581495163125	-0.218942666666666	0.294300395506211
DYRK1B	-0.0377153380572763	0.924507962758051	0.00184508048716298	0.99760061742039	-0.0457796666666658	0.847083064840772
ZNF577	-0.0378286503392178	0.9241495829693	-0.106926780996492	0.857671866148626	0.0663310000000013	0.80444630024318
NHLRC4	-0.037889800120351	0.923975272758718	0.329605729286149	0.404167398197091	-0.261747666666665	0.255532741865527
GPR50	-0.0379218882529843	0.923895072603978	-0.254225514432273	0.567694846153007	-0.0449709999999992	0.859299487874574
ALDH1A1	-0.0379926161321892	0.923615989588873	-0.0513695210331278	0.938729151196048	0.582982666666667	0.0392329313827969
UBQLN3	-0.0380027253565725	0.923615989588873	0.102450592187825	0.863158472924381	-0.144262666666666	0.55956313262575
THAP9	-0.0380245503298483	0.92355235339903	-0.0461185489137541	0.945364757579857	0.0929446666666674	0.655464996286716
TMEM40	-0.0380494598882767	0.92346724337713	0.0241613902662868	0.97420021343239	0.059130000000001	0.80758784150395
FAM129C	-0.0380557368197971	0.92346724337713	-0.0584314127092895	0.928725686060612	-0.0939669999999993	0.725673883230848
ABCD4	-0.0380568564908606	0.92346724337713	-0.0588250154194183	0.928306114846356	0.0434310000000006	0.85994094150432
VAMP7	-0.0380626393718114	0.92346724337713	-0.611991082007517	0.0277519681937494	0.020078000000002	0.94121588658818
CD163L1	-0.0381274822312632	0.923255574293952	-0.429082697287247	0.209103669076588	0.0081486666666675	0.982523503616988
SPEG	-0.0381392027068603	0.923255574293952	0.134527141333125	0.808009477165608	-0.111896999999999	0.648442399981194
HNF1B	-0.0381457603832688	0.923255574293952	0.188113621904361	0.707635058271509	-0.108650666666666	0.664841649345171
ALPP	-0.0381725955495372	0.923255574293952	0.0532389082363955	0.936629055455007	0.00159600000000087	0.996285985364494
LOX	-0.0381947441551774	0.92323467744195	-0.172710265678249	0.738420611944387	0.0620050000000001	0.798344353938564
LOC730441	-0.0382217782511679	0.923136856614383	0.00373064806415169	0.994588964700772	-0.0410906666666666	0.88384908879607
sept-15	-0.0382249601647619	0.923136856614383	-0.344381177605632	0.370887661638426	0.0639033333333345	0.774104133965233
BORA	-0.038232287109025	0.923136856614383	-0.212774812235285	0.656037270488756	0.152645333333335	0.50103373645898
TLE6	-0.0382356803635002	0.923136856614383	0.404774728639084	0.25480455106032	-0.195412999999999	0.365181410092519
RBM22	-0.0382654994196912	0.923136856614383	-0.154508335726563	0.771310656070265	0.140400000000001	0.568659275949231
SSX1	-0.0382847184903655	0.923136856614383	-0.219506547967923	0.641226345931986	0.419208333333333	0.280144599298465
LPIN3	-0.0383238329085619	0.923035214831819	-0.0379696988559629	0.954889910016503	0.117753666666667	0.58391025729515
CAPN12	-0.0383461242081839	0.922965641704101	0.190225193698597	0.704208582824765	-0.049518333333333	0.880254449461876
LOC284009	-0.0384092392723666	0.92284269759451	0.102027925185331	0.864616357134757	-0.221043666666666	0.458006502146897
APOE	-0.0384928331180774	0.922534160626713	-0.118997745362467	0.836828045978697	-0.106238000000000	0.785482517274791
UNKL	-0.0385407567995994	0.922311633774086	-0.143758372869967	0.792008469470523	-0.0929698333333328	0.682815224992534
TIPARP	-0.0385496736141502	0.922311633774086	-0.264328168269125	0.544049010305410	0.195113	0.398081243601509
MRPL42	-0.038559865484886	0.922311633774086	-0.0361874449985518	0.957959538423961	-0.0137743333333332	0.965329851466378
PKD2L1	-0.0385830040531159	0.922249697595596	0.373894838061722	0.3153356348575	0.00428466666666694	0.991048664094104
SH3TC1	-0.0385845138762297	0.922249697595596	0.0354631284822092	0.958689318480951	-0.0404229999999987	0.890094615232462
STYK1	-0.0386307262267743	0.922212933645214	0.117896807332964	0.838686136084666	0.00330466666666713	0.993756838831997
TAPBP	-0.0386413165733714	0.922212933645214	-0.522758209487826	0.0873718619016661	0.0994761111111118	0.631192833987568
GPD1L	-0.0387394310340974	0.92218814107511	0.41690784392832	0.234235464483992	-0.379787666666665	0.098483885847313
ALKBH7	-0.0388345100706068	0.921953158918332	0.149341541042306	0.782416617297835	0.0991533333333343	0.639002829892966
GABRR3	-0.0388455922002325	0.921950417641824	-0.136840457928903	0.803720303246183	0.0666046666666665	0.766132481270445
DMRT3	-0.0388503190876093	0.921950417641824	-0.176199084621834	0.73052037591204	-0.101199333333333	0.685271038967111
STK17B	-0.0388504237792048	0.921950417641824	-0.125555903988583	0.82367707928204	0.0673796666666672	0.776805598353461
GPSM3	-0.0388690276007064	0.921950417641824	-0.265383005224985	0.540682092130659	-0.143179777777777	0.476307520847818
CNKSR2	-0.0388820865244419	0.921950417641824	-0.0631070303337116	0.921562696294372	0.0149093333333334	0.963180491237413
HPS6	-0.0388914052135121	0.921950417641824	0.188363993244127	0.707361603786947	-0.0445466666666657	0.875727306158077
POMZP3	-0.0389181084109064	0.921950417641824	0.104349074983376	0.861145946230937	0.0522576666666684	0.843152489417868
BCCIP	-0.0389235699510887	0.921950417641824	0.0534895908986806	0.936236208222134	0.0085353333333339	0.979355014372038
HAS1	-0.0389307464888407	0.921950417641824	0.252679117573820	0.569339421777585	-0.184901999999998	0.516217427447249
GPHN	-0.0389551440960985	0.921950417641824	0.049184785831099	0.94189929181141	-0.182995666666666	0.498454352317107
BCAP31	-0.0389599496395736	0.921950417641824	-0.208976605981390	0.665595909869988	0.0704939999999997	0.766132481270445
CPM	-0.0389630141516477	0.921950417641824	-0.0567928805893409	0.93142484205481	0.132619666666668	0.550542499847466
ALDH3A2	-0.0389635335127053	0.921950417641824	0.0827821413215287	0.893709269554424	0.155153666666667	0.458612437736657
F2RL3	-0.03902550207201	0.921950417641824	-0.183173306967017	0.718067440349544	-0.185124666666666	0.571070613702241
CD2AP	-0.0390556617892210	0.921950417641824	0.175839702218119	0.731631458094528	-0.210871	0.261439422857188
DTYMK	-0.0391042210218594	0.921928785214623	0.0581006267662455	0.92927238835057	0.0849350000000002	0.716517864791776
STX8	-0.0391483029047476	0.921728744815811	-0.0841075134102631	0.892538205608146	0.0837853333333352	0.717906134812783

UBTD1	-0.0392017716961263	0.921632752163081	0.254747362593047	0.567055314468567	-0.00702566666666543	0.983351516933134
MYO1H	-0.03924001262983	0.921561574063926	-0.0884595284411357	0.884893243593597	-0.0540853333333323	0.824343883140698
SLC6A4	-0.0392715352948957	0.921561574063926	-0.085953200461114	0.888365513603835	-0.134889666666666	0.590147690656472
ABCC12	-0.039351374461417	0.921340346796112	0.0525111714964242	0.937722352048327	0.0131913333333343	0.967970038103825
PIWIL4	-0.0393969120574174	0.92119503516046	-0.0221009017127686	0.97536722461972	0.294413000000001	0.122514080403437
PYCRL	-0.039416366143235	0.921168793244392	0.34330685826605	0.374265187515147	-0.257260666666667	0.178395954523901
FAM101B	-0.0394609232467583	0.921117888257308	-0.228854741541629	0.621704818657675	0.110392000000001	0.656324975591247
CD72	-0.0395055692074545	0.920958838495851	-0.18552809018512	0.714210278087368	0.165055333333334	0.41666677641248
BTBD8	-0.0395112588503512	0.920958838495851	-0.443909194602344	0.184034989851268	0.31202066666666	0.249266035781889
TCF24	-0.0395359741586878	0.920958838495851	-0.229400813220281	0.620183562399927	-0.0635199999999994	0.830147563067484
SH3GL2	-0.0395466197270404	0.920958838495851	-0.0993169291319388	0.867933660937568	-0.0763243333333336	0.776805598353461
JAG2	-0.0395680262178865	0.92093397621954	-0.373632223643482	0.315811026719894	-0.0297003333333332	0.910190419163007
CHST3	-0.0396070889536917	0.920827331036759	0.272917501097015	0.525011448098239	0.00169866666666675	0.99628178244892
ZNF253	-0.0396228724693447	0.920796441274887	-0.151554937147978	0.777101208632856	0.398965333333334	0.0660125264725009
VMA21	-0.0396704954257204	0.920695170174392	-0.731876644063043	0.00165671448978994	0.0825706666666677	0.706429250016819
SCAI	-0.0397168264974697	0.920551926746323	-0.317981572190296	0.430156985569412	-0.0220856666666662	0.940880027113653
PROKR2	-0.0397339793544655	0.920512822872917	-0.116405829687944	0.842082118939645	0.115646333333333	0.587396808178803
ADAMTS4	-0.0397360353693958	0.920512822872917	-0.0073472766439502	0.990873555460625	-0.0743789999999996	0.811401072147441
GZMA	-0.0398209756966445	0.920182668271348	-0.355833618900618	0.346552539524285	0.00724433333333382	0.986471172041113
CRISPLD1	-0.0398438842667032	0.920170541196217	-0.246312860252573	0.582725878221343	-0.0364573333333322	0.915761046438965
LIPJ	-0.0398816219174341	0.920073874934438	-0.401558796090956	0.261525927335127	0.103005333333334	0.661362028309397
TMEM222	-0.0399495635581480	0.919921354977912	-0.204149135791149	0.675320698349846	0.0951026666666673	0.680455232178275
HRAS	-0.0399591228244498	0.919921354977912	-0.0368164128177931	0.956918184553224	0.0806503333333348	0.741134448923316
B3GAT3	-0.0400325428001002	0.919805956552342	0.0151574285520195	0.982921609770846	0.180796333333334	0.345433231197032
STK38L	-0.0400403770200087	0.919805956552342	-0.249152266877363	0.576149573608393	0.0773166666666675	0.717906134812783
PLRG1	-0.0400433017266366	0.919805956552342	-0.275766690659577	0.517831636036978	0.0185526666666664	0.947020470290463
MMP27	-0.0400717463598655	0.919805956552342	-0.204291281240294	0.674897948203614	0.0478283333333339	0.848144542252909
IARS2	-0.0400763853994992	0.919805956552342	0.0201729448200249	0.976956866647027	0.159285000000002	0.419500365886586
MLANA	-0.0401099724034267	0.919805956552342	-0.114940768106876	0.844162087612813	-0.0661499999999997	0.780732284998362
PNKD	-0.0401253401517524	0.919805956552342	0.222718424862764	0.635495054621817	0.0766583333333338	0.725237788753458
PRAP1	-0.0401348906959744	0.919805956552342	0.176525930315628	0.730152680173573	-0.0824179999999999	0.817550597555287
RAG2	-0.0401514869261208	0.919805956552342	0.0261565181712835	0.970632644458507	-0.217306666666666	0.302861002390456
MYOM1	-0.0402059244656205	0.919594255380834	-0.246721571253453	0.581387364514747	0.175332666666667	0.566230609307145
RUNDC1	-0.0402245934687991	0.919545996959004	-0.477089070121016	0.141885297989556	-0.0221126666666656	0.939551683168752
BMP5	-0.0402392508585695	0.919521790311193	-0.459874802581025	0.163342087268212	0.0410646666666669	0.874243298833041
TFB2M	-0.040247585964413	0.919521790311193	0.05403748089769	0.935109055935003	-0.160282333333333	0.440358761885343
RBM48	-0.0402761734565869	0.919491440958644	-0.254123760246948	0.567694846153007	-0.0238109999999981	0.944218475609619
EIF4EBP2	-0.0403581990865346	0.919428749564321	0.155939443798258	0.76972820876143	-0.107556333333332	0.616962463518288
PPP1R3D	-0.0403916570432620	0.919416353645952	-0.530248426633478	0.0799297163112453	-0.0457429999999987	0.843751236104194
RTDR1	-0.0404125847399273	0.919416353645952	0.131884653366486	0.810567865692284	-0.0234740000000003	0.933412982728621
RTKN2	-0.0404209074211857	0.919416353645952	0.0122781910593258	0.985485202753412	-0.171726666666665	0.474413951968734
DPP7	-0.0404262117381986	0.919416353645952	-0.0707744501146553	0.909764940148113	0.0124196666666676	0.974484855799046
TOR1B	-0.0404269869915516	0.919416353645952	0.124056105773292	0.825618375687582	-0.0336633333333327	0.910696784037738
STAC3	-0.0404650948906013	0.919416353645952	0.222282843974932	0.636309362271551	-0.0469003333333328	0.861690060758414
PTTG1IP	-0.0404687138722156	0.919416353645952	-0.203027527867377	0.6772404821028	0.102926666666668	0.639002829892966
DDIT3	-0.040479352546437	0.919416353645952	-0.117477616409676	0.839678370459363	0.0823693333333346	0.748603801948204
HMX1	-0.0404838605250038	0.919416353645952	0.0950409629589484	0.872962271153999	0.0343283333333335	0.896446774489185
GAL3ST4	-0.0405304017723519	0.919416353645952	-0.230051586082166	0.619232314946967	-0.0408866666666663	0.888476605628154
SEC61B	-0.0405935511676054	0.919416353645952	0.248709828597761	0.5770020684641	0.0102700000000016	0.972353176085335
FAM217A	-0.0406394199527283	0.919415412797575	-0.0485751844777037	0.94236319435931	-0.0112389999999997	0.974895360062947
NCSTN	-0.0406526205366142	0.919415412797575	-0.0173333283719878	0.980670285500112	0.229324333333334	0.218822168421039
DENND1A	-0.0406579537567311	0.919415412797575	-0.361526602945806	0.339145270424456	0.00543666666666733	0.987459024686908
CYP3A43	-0.0407204331015619	0.919247908418663	-0.213723604960592	0.653708954135733	0.0292700000000001	0.923375744130404
ZNF418	-0.0407468400969936	0.91921693628635	-0.371647193762057	0.319527591032365	0.0362006666666666	0.883189085233712
MKRN3	-0.0407805923835624	0.919077970445327	-0.103399033475307	0.861949447881126	-0.218279333333333	0.264889721029722
GPRIN2	-0.0407995516078183	0.919027890462281	0.223290832186759	0.635113856809423	-0.0401643333333322	0.895093529187836
LOC284379	-0.0408102218577151	0.919027619488046	0.0175710688450277	0.980428114126044	-0.138783333333333	0.600423004242557
ENTPD3	-0.0408532170451883	0.918896936614892	-0.0900622386335666	0.88237793095104	-0.378767333333333	0.0888564451939157
SERGEF	-0.0408744730945375	0.918889642485193	-0.112135344571226	0.847454473507881	-0.0399489999999987	0.900467460403227
ARMC1	-0.0409445893019523	0.918610386662619	0.0500787975456083	0.941330035957713	0.0069870000000009	0.987134800094338
SH3GL3	-0.0409915757454178	0.918448445787078	-0.293771640938774	0.480614908214975	0.010969333333334	0.97184501043952
RGL3	-0.0410308440296947	0.918448445787078	0.0328482561484396	0.961351562552925	0.0225783333333340	0.938187566801362
PRDM13	-0.0410748946348973	0.918357470383983	0.0836693875147792	0.892833228561937	-0.183108	0.59812387928379
IVL	-0.0410757861411936	0.918357470383983	0.038207587491036	0.95460795537904	0.0101140000000005	0.978504228748436
TCL1A	-0.041081936272343	0.918357470383983	-0.146039136996843	0.787402233709509	0.0301540000000003	0.910190419163007
PSMD2	-0.0412007372876773	0.917893325813482	-0.219770798239253	0.641035290381005	0.0160776666666680	0.962924081933534
HDAC9	-0.0412479669682176	0.917737026097232	-0.362688413429626	0.337143926897226	0.341739666666667	0.255595078844894
NOL7	-0.04127229941314	0.917737026097232	0.188782747662689	0.707361603786947	0.0357580000000008	0.886500454815894
DPCR1	-0.0413033321954116	0.917737026097232	0.100505003788944	0.866538548597168	0.0142901666666674	0.974451420920923
TRIP11	-0.0413060734337881	0.917737026097232	-0.259643969219796	0.556349654075559	0.0313490000000015	0.911623909907545
CLEC12A	-0.0413248743499412	0.917737026097232	-0.293380527766779	0.480921323328224	0.0776196666666668	0.72926287896686
OSBPL11	-0.041344870974745	0.917737026097232	0.0498214299788875	0.941458038227918	0.178420666666667	0.539361605666511
CCDC60	-0.0413487641725524	0.917737026097232	0.0227672012119426	0.97536722461972	-0.0740539999999994	0.754015160951226
GCHFR	-0.0413768183325623	0.917737026097232	0.0225204542851238	0.97536722461972	0.0063370000000013	0.986350009478474
GART	-0.0414617177439653	0.917409351134784	-0.0151561300752454	0.982921609770846	-0.264426666666665	0.203287190925869
TEX13B	-0.0414949729757015	0.917337029530571	0.0304879439125126	0.965792095735685	-0.255723333333333	0.281579610467498
MYH6	-0.0415828714662614	0.916971078454599	0.115896843740342	0.842733236705133	-0.0976389999999997	0.670719950296173
FPGS	-0.0416018993694985	0.916971078454599	0.146923818383026	0.78589154083134	-0.135229666666667	0.498456714564834
FANCF	-0.0416551098513488	0.916756628487031	-0.114297362046381	0.844272085764738	0.0596336666666672	0.813489848876388
SCLY	-0.0416724236612406	0.916716307442115	-0.163758000470176	0.754968203901817	-0.258729333333333	0.200971178623791

LAPTM4A	-0.0416740175160243	0.916716307442115	0.171291669069554	0.739682729639583	-0.201696666666665	0.297888842945994
NPRL3	-0.0417062381761510	0.916643867864817	0.0198333781715833	0.977526645675126	-0.0400666666666654	0.873398024647672
ZNF10	-0.0417420230566585	0.916616830075606	-0.183350764599436	0.718067440349544	0.216990666666667	0.290878986887538
MORC2-AS1	-0.0417786741069826	0.916460077137626	-0.101151236918342	0.866005093242444	-0.204045666666666	0.29986081041577
RASSF9	-0.0417977000860265	0.916409430419327	-0.251185797301569	0.572882168047464	0.0646963333333335	0.809627693841916
ACAP3	-0.0418009511776056	0.916409430419327	-0.080208698304986	0.897577172229156	-0.178406	0.361971299676959
CXorf64	-0.0418687779099958	0.91630098879349	-0.0930409102248583	0.877331332619452	-0.0139249999999999	0.963815889459617
CCL25	-0.0419599041690493	0.915943942823171	0.0904169799207889	0.881797721301484	-0.0741266666666662	0.798752256977623
CBS	-0.0419869703944208	0.915844843661207	-0.0279866108184815	0.968950505723444	0.0312226666666675	0.913386831328212
INTS1	-0.0420853910257706	0.915316031249834	-0.221801116203074	0.636874397609126	-0.080585333333333	0.707538975864338
ZNF385C	-0.0420925105730102	0.915316031249834	-0.0688216372972544	0.912149839732916	-0.00842666666666616	0.98147292539484
IFI44	-0.0421351755608474	0.915207840219804	-0.0791205985938817	0.898830040032591	0.0543710000000005	0.855533429360007
SLC38A2	-0.0421360226707168	0.915207840219804	-0.316963533449600	0.431780281826320	0.13455	0.503352066997863
IQCE	-0.0421582561101006	0.915207840219804	-0.0388614249373179	0.954228173733495	-0.0325303333333322	0.896186784012643
ZNF300	-0.0421718350753271	0.915207840219804	0.0208015317023248	0.976330597778846	-0.0567059999999999	0.864574541922917
AOC2	-0.0422519226210058	0.915030398473745	-0.0106212344774987	0.98774587825743	0.097631000000001	0.64079197248551
BCMO1	-0.0422861063621978	0.915030398473745	0.0737476683744444	0.905506084101768	0.0364530000000003	0.904201124848034
MUC13	-0.0423263293133721	0.915030398473745	0.171343335261350	0.73955239116035	-0.0709823333333329	0.763971031224737
IFI44L	-0.0423368099037902	0.915030398473745	-0.281975547882732	0.504775776425053	-0.065492333333333	0.830987625642722
FBXL16	-0.0423531468733527	0.915030398473745	-0.0116651302162013	0.986654146365942	0.213841666666667	0.365126455342703
ATP5B	-0.042353765361902	0.915030398473745	-0.219387248807288	0.641226345931986	0.00071000000000192	0.99792238320329
GGH	-0.0423772311931563	0.915030398473745	0.119655738454615	0.835740437824474	0.297409333333334	0.117653089562184
AGR2	-0.0423917761388263	0.915030398473745	-0.159726502588882	0.763150272733332	0.0525613333333335	0.838279488520094
CCNB1IP1	-0.0424288626794716	0.915030398473745	0.207232564892070	0.66888525756447	0.225636666666667	0.390967882720418
MARC1	-0.0424303692040324	0.915030398473745	0.188408637757983	0.707361603786947	0.201664000000001	0.286257650826517
MYO18B	-0.0424843256741663	0.915030398473745	0.0617988146685493	0.923246185283376	-0.0677659999999994	0.866450473811278
CHST8	-0.0424935765961715	0.915030398473745	-0.0592173873621324	0.927424336832778	-0.091532333333332	0.767461404513371
WDR96	-0.0424951075576694	0.915030398473745	-0.100029061438361	0.867247507420094	0.325158666666667	0.0942538294765004
DGKG	-0.0425168708193767	0.915030398473745	-0.162751852907507	0.757417321756424	-0.214015	0.280489645634120
FNIP2	-0.042537219329533	0.915030398473745	-0.409269769836605	0.249078359611911	-0.482461333333333	0.166543996066754
OPN4	-0.0425585676540662	0.915030398473745	0.276089923018913	0.517549833373363	-0.184759333333332	0.504028037830483
RNF212	-0.0426327016149884	0.914894707419021	0.0476917102089396	0.943560556552289	-0.078084	0.735938662411184
UQCRC2	-0.0426589909017448	0.914864805792675	-0.227744245514827	0.624667381871867	-0.310567333333333	0.101980812453755
TEAD3	-0.0427229630651102	0.914734232008643	0.0807049888564274	0.896621311541396	0.176401666666666	0.365126455342703
DLK2	-0.0428477158032254	0.914237370035319	-0.257144000687765	0.563116080558022	0.103326666666668	0.739507465050387
RTN1	-0.042917769824943	0.914237370035319	-0.143031404405046	0.793304811422389	0.155064666666668	0.640068046551018
ZC3H8	-0.0429356163693417	0.914237370035319	0.0170341245162830	0.98105020869911	-0.0755113333333322	0.724384070314197
FAM26D	-0.0429844097183779	0.914180749966283	-0.198900912749784	0.68526131681093	-0.0582176666666659	0.813694870801638
HIP1R	-0.0430214166404972	0.914117190323323	0.500370633672044	0.113248709977844	-0.111350000000000	0.595271860494793
CCL11	-0.0430576643276542	0.913994623324273	-0.00513544464223948	0.99251028338798	-0.0141249999999993	0.974451420920923
TNFSF15	-0.0430629729018702	0.913994623324273	-0.389302636829257	0.283516069208756	0.106527666666667	0.657217907037306
ALLC	-0.0431214816527812	0.913801372036015	0.0601666980800782	0.925649366494154	-0.163357999999999	0.612891410601889
PHF14	-0.0431330658386501	0.913795481167956	-0.209470814329933	0.664420531246941	0.0402223333333342	0.87629402535202
NANOS3	-0.0431336090893102	0.913795481167956	0.153880787548451	0.77284838227453	-0.00505566666666677	0.987937064305561
CD101	-0.0431415523396004	0.913795481167956	0.273687357879085	0.522854876695708	-0.0268676666666657	0.92590541082869
GRPEL2	-0.0431432514210836	0.913795481167956	-0.222552561955691	0.635668764102271	-0.130255000000000	0.526142390569419
TMED1	-0.0431491135245475	0.913795481167956	0.3282610596281	0.407301172667012	0.111390000000001	0.661812056571718
KIR2DS4	-0.0431791012043392	0.913795481167956	-0.0185735300102277	0.97902968742576	0.0225546666666675	0.944218475609619
SLC6A19	-0.0431846395795234	0.913795481167956	0.169307065875081	0.744676064883446	-0.028350666666666	0.945813875079248
CPXM1	-0.043192965484483	0.913795481167956	-0.133355913410114	0.808992949393043	-0.0974343333333329	0.644175841365564
FBXL3	-0.043256358490134	0.913755713285736	-0.204939299754251	0.673728362275223	0.0373220000000006	0.903774117860245
C11orf88	-0.0432605802926854	0.913755713285736	0.0887952199864814	0.8841943128258	0.0292330000000005	0.929624114746256
GSPT1	-0.0433649520845377	0.91341993177419	-0.0437233758526726	0.949043721172002	0.0213800000000005	0.936899235817306
DIS3L	-0.0433790426274792	0.9133988847461	-0.0268965814404915	0.970207612777188	0.165582000000000	0.39808310170806
XRCC6BP1	-0.0433888890331518	0.9133988847461	0.236225957918616	0.6055054064321	-0.0334556666666668	0.911242495488915
TFAP2D	-0.0435094314853315	0.912802933599338	0.0298256125302204	0.96695070705759	-0.104159333333333	0.631961600239811
RXRB	-0.0435507269454868	0.91274561448986	-0.142355291108956	0.794358425717977	0.154582	0.428647340632264
EDDM3B	-0.0435755544104675	0.912659612484531	0.125127752278529	0.824230922676958	0.106741666666667	0.719909781429035
SCFD1	-0.0435991193149101	0.912645371088858	0.0294577588855587	0.967394096882695	0.124166333333334	0.534196070908831
IPO4	-0.0436001409442783	0.912645371088858	0.281117046443002	0.506886267079183	-0.541335999999999	0.0216369771709286
UBR3	-0.0436009558416221	0.912645371088858	0.369865324224997	0.322377391230088	0.00139733333333412	0.99704422037925
MAP3K7	-0.0436288680693338	0.912645371088858	-0.0629407649180464	0.921896414411348	0.0187563333333352	0.949295980201336
DCAF4L2	-0.0436313390148747	0.912645371088858	0.159702815743904	0.763153072998024	0.0868573333333339	0.707538975864338
CLMN	-0.0436376824025406	0.912645371088858	-0.145243906151905	0.788344418401815	-0.127799666666666	0.711075151205956
PRG2	-0.0436690277572189	0.912645371088858	0.0685284186554649	0.912149839732916	-0.122581999999999	0.667697330885416
MPO	-0.0436923525860388	0.912645371088858	-0.00542897056216005	0.99251028338798	0.0461580000000002	0.863745180020314
HDAC3	-0.0437063760399032	0.912645371088858	-0.142078630768820	0.79450771779567	-0.170326999999999	0.433234748327389
CCDC149	-0.0437263865049398	0.912645371088858	0.038295475945952	0.954545200586067	0.0618223333333339	0.778912256576868
CCDC105	-0.043767215707426	0.91252673144234	0.0792589522104546	0.898424765944956	-0.104382666666666	0.657217907037306
ADAM7	-0.043806935271442	0.912407085958589	-0.0627605532218493	0.922350559812086	-0.0161703333333327	0.955460918122607
ZNF320	-0.0438142139632956	0.912407085958589	-0.269752525886789	0.531775404769437	0.166378333333334	0.4254947046787
MAGEB6	-0.0438679968821552	0.912190524085625	-0.358497344508964	0.343101042014771	-0.122468333333333	0.600928321695266
LAPTM5	-0.0439246378810176	0.911959113432829	-0.466774046056654	0.152524229105475	-0.223879333333333	0.284819435721451
MTG1	-0.0439547772021899	0.911905050810721	0.263086048045043	0.546774946007658	-0.0778709999999996	0.758966957076109
POLR1B	-0.0440257325614549	0.911539751386506	-0.109976406527459	0.851013616427418	-0.286618333333334	0.172571369175474
ERCC2	-0.0440574947835294	0.911508885822493	-0.275521119062282	0.518649818798976	-0.0326929999999994	0.918952691262331
TNK2	-0.0440847818619819	0.911508885822493	0.0222214531702481	0.97536722461972	-0.0881046666666662	0.688145991467449
REEP1	-0.0441004441197304	0.911508885822493	-0.207478685427486	0.668625825887966	0.182765666666667	0.436020392195898
C20orf196	-0.0441230758885993	0.911508885822493	-0.0721016436921344	0.90824872013293	-0.104642666666666	0.704370299831112
ONECUT1	-0.0441656947189855	0.911462613070617	0.131466720940445	0.811042872465441	-0.0664896666666657	0.821587043214193

GP1BA	-0.0442026372889344	0.91131068260858	0.0752423452828076	0.904173044514532	0.170881333333334	0.465279557781548
APOA4	-0.0442545897496937	0.911196336757092	0.182708839968379	0.718559738568628	-0.140513166666667	0.600950363504414
OLR1	-0.044283313224051	0.911196336757092	-0.330378524552819	0.402284079938133	-0.0143703333333334	0.964530776748387
AHSP	-0.0443271928330549	0.910998148559775	-0.0808743207908752	0.896361013063453	-0.0835026666666665	0.691482999769299
AASDHPPT	-0.0443430477121693	0.910966319485413	-0.33259052001817	0.398142341492687	-0.0334581666666660	0.893151737593092
AHR	-0.0443724692736403	0.910852302306992	-0.110821835082952	0.849357727620213	0.0615976666666676	0.848462566063264
TAF8	-0.0444200957790656	0.910692201490954	-0.131036257695218	0.812271254601992	-0.732680999999999	0.0126478660661103
PSENEN	-0.0444305097810987	0.910692201490954	-0.327123492252143	0.408851707589499	0.103797333333335	0.637596150841031
EPCAM	-0.0445580034697218	0.910306120956641	-0.346864759095725	0.364568189046910	0.4614295	0.141475366389837
CLCN1	-0.0446273854333448	0.910054696195604	0.104578180545471	0.86067831050617	0.0088103333333339	0.985230239434352
GPR27	-0.0446301392365012	0.910054696195604	-0.139052776710195	0.79864602540281	-0.0746863333333324	0.76307213318662
GNRH2	-0.044670410153691	0.910054696195604	0.176565739400107	0.730152680173573	-0.132459000000000	0.76564992393702
ICAM4	-0.0446783801112341	0.910054696195604	-0.430015847369840	0.20761765766777	0.0929500000000004	0.720355976568153
SMR3B	-0.0446936173450437	0.910054696195604	0.185664811068056	0.714043644840406	0.0485156666666671	0.835752276645858
PPM1H	-0.0446958602858974	0.910054696195604	-0.016729913084763	0.98131656289171	-0.265180666666666	0.162661087494939
PLD3	-0.0447272925121655	0.910054696195604	-0.09786264544518	0.868852648210739	0.134383333333334	0.52128449277411
SHE	-0.0447308438718205	0.910054696195604	-0.308774658719191	0.449759364777049	0.0498970000000008	0.895365434044622
KY	-0.0447642222252572	0.910054696195604	0.0677360174412471	0.913713661548934	-0.122408666666666	0.648963524582188
CYB561	-0.0448220887156557	0.909925849043369	0.153573636927899	0.773392057294058	-0.156625666666665	0.447131186662059
FBXO2	-0.0448549634312305	0.909831637622738	-0.112439598013952	0.847309711184787	0.00427733333333372	0.993756838831997
LRTM1	-0.0448743451465718	0.909801744629618	0.0592452830459043	0.927347284466018	-0.0304696666666663	0.910663650054071
C5AR1	-0.044876913799618	0.909801744629618	-0.322930228082347	0.415927283236740	0.080748333333334	0.742738366366583
MLKL	-0.0448784648364814	0.909801744629618	-0.280782769516091	0.507365125605885	-0.247900333333333	0.195927187569286
SMR3A	-0.0448800815002408	0.909801744629618	0.0184694882532694	0.97902968742576	0.0762483333333336	0.782130952601532
MESP2	-0.0448881511870066	0.909801744629618	-0.0975442205995703	0.869639592679605	-0.163695666666666	0.608096830091536
ARL8B	-0.0449087565003858	0.909801744629618	-0.192767183906048	0.699860991842232	0.0433336666666687	0.872930585008323
RHOXF1	-0.0449215637895471	0.909801744629618	-0.219014884411661	0.641856878959206	-0.00230133333333307	0.995891981162594
OTOS	-0.0449368828247228	0.909801744629618	0.38298275027807	0.294467842756818	-0.0956819999999987	0.716517864791776
PPP2R5A	-0.0449512687095507	0.909801744629618	0.048868733415422	0.942180113539637	0.521281666666668	0.030025317565228
IMPG1	-0.0449692574803426	0.909801744629618	0.0368446344550613	0.956918184553224	0.0751233333333336	0.764133293709815
SHANK1	-0.0449981265093901	0.909801744629618	0.266548292792880	0.538833471023229	-0.437221	0.0382989968555555
NKX2-5	-0.0450215839159972	0.909801744629618	0.0607975795795276	0.925141411805701	-0.0177749999999990	0.948814570944396
ANKRD33B	-0.0450611534485873	0.90976492709599	-0.403790525602801	0.257216918236696	-0.0705496666666655	0.759034160486144
PRF1	-0.045079983794335	0.90976492709599	-0.439419069919152	0.190755916694782	-0.154572	0.622282705325595
TSEN34	-0.0450946700681314	0.90976492709599	0.17113832459286	0.740062065190296	0.00511433333333411	0.987499363290182
IRF4	-0.0451519011389538	0.909596815073033	-0.114017177682236	0.844272085764738	-0.180229333333332	0.398794937632645
BEST3	-0.0451841112517837	0.90953930337245	-0.155689603389145	0.769837060156515	0.0286336666666671	0.910935860136448
ZNF425	-0.0452021884782120	0.90953930337245	0.21990070796943	0.641001372559671	0.0074896666666674	0.983159241812861
XDH	-0.0452182677610262	0.90953930337245	-0.309222792113229	0.448350131365446	-0.0111629999999997	0.970891562387519
CEP290	-0.0452651027141685	0.909488792902415	-0.295854164749225	0.47761182409076	0.119475666666666	0.634163156600322
RHEB	-0.0452740416963689	0.909488792902415	-0.0920658519213563	0.879153312143409	-0.0472119999999987	0.866671423175521
WT1	-0.0452760329784695	0.909488792902415	0.451776404178115	0.176663460992485	-0.183041666666666	0.575589336285762
SLC9C2	-0.0452886839767792	0.909488792902415	-0.0195566179480067	0.977673457110824	-0.0312823333333332	0.90945379489823
ISCA1	-0.045324928472869	0.909447403135353	0.00251317251149853	0.99580901762095	-0.0276940000000000	0.91962770573477
PRDM9	-0.0454251841799689	0.909217385240736	0.0412677610781594	0.952098348256047	0.204201	0.353471671280201
PLG	-0.0454334997335347	0.909217385240736	0.0814272541887382	0.89615326639288	-0.0401859999999995	0.87279470391545
C9orf135	-0.0455314764021773	0.908887798088196	-0.318720249912710	0.428596955589139	0.102272000000000	0.692043732062832
CLEC4F	-0.0455425969402139	0.908887798088196	0.127591875735014	0.818717515586031	-0.00389533333333285	0.993677488355417
PHAX	-0.0455486888057802	0.908887798088196	-0.354989429961982	0.348488415421134	-0.0221883333333331	0.93278021276477
RABEP2	-0.0456185305199034	0.908593952260806	0.243876495555700	0.589605368171009	-0.371473333333333	0.12148388169181
LNX2	-0.0456889219751244	0.908439295008711	-0.256970464098868	0.5632303733857	0.318913333333334	0.128872002711120
TM9SF4	-0.0456950530311328	0.908439295008711	-0.0204662182019042	0.976412247012224	0.000957000000001015	0.997144862160692
ADIPOQ	-0.0457817730708668	0.908216953462097	0.381051779521529	0.298110142491852	0.0520196666666674	0.838874519061208
RIPK3	-0.0457950414517797	0.908200715959733	-0.0856248647780869	0.889156172508374	0.115221333333334	0.657217907037306
KRT27	-0.0458523015857117	0.90806277682761	-0.155498105508096	0.770174160140798	-0.0156643333333326	0.9681321732097
NGDN	-0.04587763313336	0.90806277682761	0.00434484579212293	0.99405344216434	0.0778393333333346	0.719839702715467
ETV7	-0.0459500392439378	0.907709203986762	0.190086707078066	0.704322109927422	-0.109595999999999	0.648861254074544
MYL6	-0.0459543410169961	0.907709203986762	0.113416452600954	0.845608298802641	0.178790000000001	0.359908305595919
FGR	-0.0459824055375221	0.907709203986762	-0.251275710331731	0.572793151484699	-0.136685000000000	0.670071773006358
WDR25	-0.0460397469598857	0.907665652428168	-0.295297414213811	0.478332806543771	-0.110702000000000	0.65888705184376
PCGF3	-0.0460535643853178	0.907665652428168	-0.167259735845085	0.749156694551161	0.154997666666667	0.462444913819465
PHF5A	-0.0460804838522492	0.907624746586025	-0.030794067391572	0.96529904314994	-0.166380666666665	0.390561931914011
SH3GL1	-0.0460895953046312	0.907624746586025	0.073723713628292	0.905506084101768	0.0203773333333350	0.95285450244329
RLF	-0.0461017060650805	0.907624583017827	-0.183128071161334	0.718067440349544	0.00282733333333379	0.993756838831997
ZNF721	-0.0461603622627697	0.907396587809547	-0.201883144602849	0.680054545720343	0.141125000000001	0.546294202178525
CNNM3	-0.0461651819213246	0.907396587809547	-0.147988844922340	0.784080588385358	0.0535853333333351	0.8257633904865
C8orf82	-0.0461702428054679	0.907396587809547	0.348156230170878	0.361374443308161	-0.100539666666666	0.68271691249359
AMD1	-0.0461718339990941	0.907396587809547	-0.0891590795450136	0.883460564717377	-0.0280559999999984	0.919456505549688
SPTBN4	-0.0461994888036199	0.907396587809547	0.112169770882359	0.847454473507881	0.00232000000000079	0.994738356997797
PIK3C2A	-0.0462081347922693	0.907396587809547	-0.0333827551112059	0.960784250392482	0.0514156666666677	0.84547275205075
WRNIP1	-0.0462089421655752	0.907396587809547	0.378603217480359	0.304578046752771	-0.264084999999999	0.173286092065797
OR5K1	-0.0462204298352591	0.907396587809547	-0.366687228960412	0.328151450414815	-0.0447286666666662	0.85013576481729
KIAA1045	-0.0462519064736183	0.907391527876063	0.176892392713231	0.729794187782856	0.0370206666666674	0.895093529187836
TPTE	-0.0463320849827963	0.90709921873924	-0.403106090743987	0.258453301425503	0.0478446666666675	0.868046126197316
CALD1	-0.0463739678563187	0.906934794258586	-0.281035637643640	0.506886267079183	0.671768000000002	0.0140473413958925
KIRREL3	-0.046381105578562	0.906934794258586	0.280386248294212	0.507669977518963	0.0945276666666673	0.721719725765502
GRAPL	-0.0464139406035172	0.906884947334779	0.0556154131190205	0.933409139126562	0.141420666666667	0.535539620005815
GPR85	-0.0465361076715097	0.906528061302866	0.0163173718476229	0.981907598420222	0.0474703333333336	0.837759479233448
TPM3	-0.0465937326681728	0.906306111846112	-0.0840204396129734	0.89265118468217	-0.124430999999999	0.5293518047673
DCTN2	-0.0466077491766412	0.906306111846112	0.27877162082695	0.511025440770733	0.107063333333335	0.622282705325595

BTBD11	-0.046617385334964	0.906306111846112	-0.171466548171159	0.739541495555634	0.0975110000000003	0.688145991467449
PROM2	-0.0466186378270049	0.906306111846112	0.398942015383939	0.266357224165851	-0.0657676666666663	0.838443963436899
SLC4A8	-0.0466227336410267	0.906306111846112	0.185540446341667	0.714210278087368	-0.337429666666665	0.248900866475991
ADAMTS1	-0.0466795359655708	0.906306111846112	0.115056285645366	0.844153940531421	0.148716666666668	0.650983465855075
MRPL10	-0.0466829670324159	0.906306111846112	-0.0711912772511898	0.90935479990339	-0.138961000000000	0.485005653709636
FATE1	-0.0467119071043338	0.906306111846112	-0.136970737705454	0.803348305001716	-0.0417739999999995	0.883206615493913
GTF3C2	-0.0467413236465909	0.906306111846112	-0.156869662582083	0.768401006769299	-0.168599333333331	0.416412641573293
JUP	-0.046750673576706	0.906306111846112	-0.234784629705686	0.607957283325678	-0.143634666666666	0.49000765832939
MLC1	-0.0467619247081740	0.906306111846112	0.303524236090437	0.461038353800206	-0.0726413333333322	0.771373465365664
TOX2	-0.046802128446364	0.90619846366203	-0.0860069494690319	0.888306347447303	0.1784265	0.490974594771964
BHLHE23	-0.0468264791346383	0.90619846366203	0.140474974614428	0.797214176067984	-0.129729666666666	0.649420534269272
KIAA0319	-0.0468680373910909	0.906091112982566	-0.0612824812246229	0.924274691992681	-0.074389333333333	0.729912257977231
SELK	-0.0469086340670363	0.905936519979082	-0.103780757292729	0.861833996895603	-0.0794806666666663	0.721454370213253
ASIP	-0.0470326202908349	0.905374273565743	-0.147477161561641	0.785497496933202	-0.183113000000000	0.528890172622518
PWP1	-0.0470971645287593	0.905174437836173	-0.391464528876016	0.280540221838803	-0.159742666666666	0.423347587771162
KLF12	-0.047233580319715	0.90457496483751	-0.143052777543918	0.793304811422389	-0.240910333333332	0.207741624292248
PDZD9	-0.0472494375007078	0.904574483949539	-0.0637098219824866	0.920685491148474	0.0336253333333338	0.895093529187836
GP6	-0.0472574493577395	0.904574483949539	0.205214466182992	0.673296856274784	-0.190282999999999	0.35499403703554
S100Z	-0.0472680932401854	0.904574483949539	0.0633951722675986	0.920885810365418	-0.0471956666666667	0.889547401003742
EPYC	-0.0473440027406372	0.904332509614936	-0.0237345545373661	0.974934434225626	0.0295773333333337	0.92484471634727
HSD17B7P2	-0.0473444247478451	0.904332509614936	0.0733478306455792	0.90588212168674	-0.580729333333333	0.0273713913650908
RNF128	-0.0474985675525813	0.90370782355127	-0.0528288272458132	0.937034903634168	0.182552666666668	0.378005932140775
CCDC42	-0.0475076389545407	0.90370782355127	0.2068901756869	0.669940115008878	0.0341110000000003	0.911600329626215
FCF1	-0.047536099027407	0.90370782355127	-0.0552279427770961	0.933608511324566	-0.0339309999999993	0.914418029309221
CCL16	-0.047626007843031	0.903240104759882	0.0473140723138132	0.943981149721771	-0.0765319999999994	0.746803560287939
RPS27A	-0.047658137951996	0.903108636482053	-0.227258592647076	0.62581495163125	-0.0383162222222212	0.87709801222343
HELB	-0.0476617665444203	0.903108636482053	-0.329726699098697	0.403821204945523	-0.370802999999999	0.166598174349797
YWHAB	-0.0476813978133931	0.903095808454259	-0.277957327582334	0.512619564175259	0.0485933333333355	0.841643842869151
ZNF619	-0.047701245651148	0.903039257510045	-0.0391541382921856	0.953905313456582	0.166661666666666	0.452783209309392
C21orf59	-0.047709939388240	0.903039257510045	-0.182886127924268	0.718289071039953	-0.173908333333333	0.441909622750662
MMP13	-0.0477289607139061	0.902999245622703	0.074861569023817	0.904466414650249	0.0193803333333334	0.952460815972492
DKFZp451A211	-0.0478172444937445	0.902783279579702	-0.00364084175339688	0.994588964700772	-0.0824259999999998	0.818866970611819
CTSH	-0.0478189755243979	0.902783279579702	-0.207330947113203	0.668877930282194	-0.0633039999999993	0.776112195351357
CHRNA1	-0.0478661753878454	0.90267832634107	0.198081299705970	0.687502298735334	0.0165636666666676	0.954216641809453
ZNF84	-0.0479203966786551	0.90260563442946	-0.179956568368718	0.723716289903531	0.137926000000000	0.487761541713127
PIGL	-0.0479467887560922	0.902593089816724	0.298855026522252	0.469994970380678	0.0334766666666669	0.903788355106891
TTC23	-0.0480010029479505	0.902501018968836	0.0544947771945084	0.9344692810234	-0.0378379999999995	0.887760071161783
MAP3K9	-0.0480501073642544	0.902330366054723	-0.0798062692794112	0.897892885174022	-0.111586333333333	0.621414476984274
BAZ2A	-0.0480656548842363	0.902300019129042	0.303823329317244	0.460052080110635	-0.105767333333333	0.695527029078549
ABCB10	-0.0480857564665281	0.902300019129042	-0.177799941673905	0.728031191477223	0.0994126666666681	0.640799282945291
ALPK2	-0.0481129445177376	0.902300019129042	-0.0190891453258130	0.97832242527852	-0.134701	0.496323393705678
ERAP2	-0.048121618549581	0.902300019129042	-0.188602159537756	0.707361603786947	0.104066666666669	0.718749100405876
STAMBPL1	-0.04815815242607	0.90218733375047	-0.173328809177369	0.736740209571778	0.835773	0.0326806169766676
LSM12	-0.0481844883980644	0.902091078776354	-0.194410878442678	0.696294554451457	-0.109805166666665	0.614221661998915
MADCAM1	-0.0481870692788919	0.902091078776354	0.089632611701494	0.883196389963269	-0.102438333333332	0.748715075113364
SLC25A29	-0.0482655903204331	0.901789492122327	-0.413790516253375	0.238622785037791	-0.144880999999999	0.549323798856043
CSF1R	-0.0482680712197086	0.901789492122327	-0.425429747832635	0.215369960032963	-0.0287973333333331	0.935187555185002
C8orf4	-0.0482831699627307	0.901789492122327	-0.271204859203386	0.528032433754325	0.00858333333333344	0.981775663937206
ZNF600	-0.0483132462491687	0.901789492122327	-0.136891947166606	0.80364731913341	-0.0231633333333328	0.954908838133862
PASD1	-0.0483813267085527	0.901663957325342	0.0381179060569186	0.95468180561467	0.064601666666667	0.787248874966057
LAMTOR1	-0.0484516709268732	0.901492610691968	-0.253634794656259	0.567705016149998	0.246081666666668	0.236989286061345
LONRF1	-0.0484779210869125	0.901396811471346	0.0880125096804989	0.885848000380851	0.706693333333335	0.0100150058243058
CERS4	-0.0485033172725434	0.901306227049634	0.183874299194636	0.718067440349544	0.0711663333333346	0.74238434992823
ARRB2	-0.0485040173302593	0.901306227049634	-0.372859017401934	0.317134467774557	-0.259005999999999	0.259184854253064
PJA1	-0.0485216376618711	0.901306227049634	-0.278713622149683	0.511025440770733	0.139479666666668	0.525421163436372
CLSTN3	-0.0485224104451144	0.901306227049634	0.252092022455317	0.570473184967855	0.0308960000000008	0.914418029309221
ELMO2	-0.048532187529197	0.901306227049634	-0.290969690638027	0.486880079690803	0.0814523333333345	0.709445924150415
KIAA1009	-0.0485817664546262	0.901149916734929	-0.389178371050477	0.283561813452034	0.717292333333333	0.0216138709400044
USP12	-0.0486098389764601	0.901124568175452	-0.00664083695216852	0.991309497300929	0.0979896666666682	0.703425120035931
ZXDB	-0.0486403157142415	0.901115257113378	-0.378360251609374	0.304866746588854	-0.0408236666666668	0.873981409807198
PSORS1C1	-0.0486724717504132	0.901047971997178	0.0501768477553278	0.941133149988298	0.0187285000000002	0.966813448118178
CDC16	-0.0487758563584495	0.900739088841486	-0.109868062938061	0.851013616427418	0.076522333333334	0.741438256509195
NSUN2	-0.0487817947281748	0.900739088841486	-0.396922935152925	0.270862229167822	-0.204350666666666	0.289347186230224
GBP5	-0.0488496027622398	0.900646410141522	-0.36670567914057	0.328151450414815	-0.0298313333333329	0.92469922248949
ANXA2R	-0.0488504714631742	0.900646410141522	-0.0404386606813767	0.952720585102517	0.0430610000000005	0.88616294579686
GK2	-0.0488571198074141	0.900646410141522	0.271315426116034	0.527984832801825	0.0650950000000006	0.778995157976597
PTPN3	-0.0488798085982998	0.900646410141522	0.0254760643539568	0.972182886218393	0.0877236666666675	0.716268843468132
C15orf27	-0.0488838429752504	0.900646410141522	-0.0686540884699513	0.912149839732916	0.337736333333333	0.186555739539875
EDARADD	-0.0488854197382133	0.900646410141522	-0.220632235043157	0.639101135191794	-0.160821666666666	0.46297633209186
ZNF784	-0.0488959125493081	0.900646410141522	0.397294390630245	0.270351490318455	-0.126947666666666	0.564757468001238
ZDHHC19	-0.048910016036452	0.900646410141522	0.0413611940000224	0.952098348256047	-0.017061666666667	0.968696337092954
WRN	-0.0489702239909716	0.900646410141522	0.101323281373564	0.866005093242444	0.114968000000001	0.592716584553539
QTRTD1	-0.0489947441688046	0.900646410141522	0.30808647507314	0.451455657653181	-0.0395189999999983	0.871104061464043
ACMSD	-0.0490090837553248	0.900646410141522	0.0143569385924415	0.983444353656142	-0.0289316666666665	0.924900975298637
TXNDC2	-0.0490320582751793	0.900646410141522	0.0807597471554813	0.896540908727066	-0.0479976666666662	0.868751484886214
DGKZ	-0.0490403795240073	0.900646410141522	-0.224768811127896	0.63208838996993	-0.0777091666666664	0.728708142582036
RNF219	-0.0490900336729992	0.900623474125738	-0.00097919544836933	0.998745415362821	0.00356166666666742	0.99117823918328
POLR2I	-0.0490953700979443	0.900623474125738	-0.191058511847038	0.702217515036541	0.0193129999999995	0.944218475609619
PDXDC1	-0.0492288196223278	0.900431218087819	-0.0508370268858058	0.93996755948181	-0.301243333333333	0.120319574336004
SPATA2L	-0.049287029128781	0.900317797548699	-0.197609190233785	0.688668717091024	0.00617766666666691	0.986290211615582

ZNF300P1	-0.049300469660261	0.900312130112225	-0.0972405823151803	0.870020870354202	0.516124333333334	0.101374569798671
UBE3D	-0.0493195123954363	0.900260277091284	-0.198526068751399	0.685952362965345	-0.0898106666666668	0.73097308185803
IGLJ3	-0.0493206967782546	0.900260277091284	-0.275028414652619	0.51990180188597	0.0530153333333344	0.859264381447494
PPP1R12C	-0.0493214526026909	0.900260277091284	0.153477516238796	0.773604308443274	-0.134633666666666	0.51786180567444
CD1C	-0.0493238331591358	0.900260277091284	-0.305260209181855	0.458013550008763	0.0554803333333341	0.86183426299397
SP100	-0.0493367880641529	0.900260277091284	0.0128881754584290	0.98482322383325	0.336494666666667	0.099045346265795
ARRDC2	-0.0493604109553519	0.900260277091284	-0.179862042845627	0.723716289903531	-0.209193999999999	0.331900139634594
TCEAL4	-0.0493666740879787	0.900260277091284	-0.284094846618295	0.500557068353025	0.0318260000000012	0.903788355106891
FGF11	-0.0493776235012117	0.900260277091284	-0.0735380107609881	0.905838142287595	-0.0980820000000001	0.689661288408633
APBA2	-0.0494059192603126	0.900249573752227	-0.097156397461867	0.870020870354202	-0.138198333333332	0.49971129098408
GPIHBP1	-0.0494274812433783	0.900182262813224	0.081085434429645	0.896361013063453	-0.182068666666666	0.408178621977204
AP5M1	-0.0494896555289148	0.899995708893333	-0.204787433300361	0.673728362275223	0.155997666666668	0.420177286343464
CENPT	-0.0494916127295921	0.899995708893333	0.41383532995363	0.238622785037791	-0.176328666666665	0.388303383752735
GLIPR1L1	-0.049558020945949	0.899706278767185	0.233493817931460	0.610436189314218	-0.0283049999999996	0.919359825156123
RAMP2	-0.0495627807927508	0.899706278767185	-0.143057986502315	0.793304811422389	0.108908666666667	0.595150543243344
LINC00313	-0.0495744467725648	0.899706278767185	0.301639383605863	0.463258004901552	-0.00587400000000003	0.988133186272453
C1orf192	-0.049582855858401	0.899706278767185	0.143019116805362	0.793304811422389	-0.142727666666666	0.592716584553539
DMRT1	-0.0495858879610232	0.899706278767185	0.155254318623968	0.77058108906698	-0.00218299999999975	0.99597023247862
NBR1	-0.049689144028945	0.899485758206589	-0.266973643472881	0.537283176095935	0.164197333333334	0.450203306181989
UBR4	-0.049716301211253	0.899485758206589	-0.0492360874540052	0.941842562680095	-0.0704989999999999	0.828866908964934
LYPD1	-0.0497422901523352	0.899432175862187	-0.0270966508508463	0.970207612777188	0.0186726666666682	0.956398070618612
TXNDC15	-0.0497881207466564	0.899268402927746	-0.0473560463729943	0.943981149721771	-0.0247569999999980	0.929594956567283
RELL1	-0.0497973531192832	0.899268402927746	0.0941046077080723	0.875126060855988	0.421455333333334	0.0895407923270921
FAM135A	-0.0498560030442757	0.899026778693079	-0.0611581361863772	0.924684666171101	0.0118210000000003	0.978708202134023
GPRIN3	-0.0498888413815393	0.898974989385599	-0.387061649456951	0.287581226843956	0.0841140000000002	0.835958129188236
RNF126	-0.049911304345214	0.898966989576867	0.186616873797007	0.711306976302188	-0.130115999999999	0.51786180567444
MGST2	-0.0499176366486862	0.898966989576867	0.169269070002106	0.744731802612236	-0.0298173333333325	0.915761046438965
HRG	-0.0499255519818346	0.898966989576867	-0.100607862150088	0.866414040673375	-0.0189439999999996	0.957844701580613
SPINK7	-0.0499430059517545	0.898966989576867	-0.116507165295912	0.842046114075432	-0.377643333333333	0.252456561824252
PPFIA2	-0.0499698268801098	0.89886754905469	0.243776185993508	0.590048043664967	0.0306443333333333	0.922148106708426
TNFRSF17	-0.0499835542103293	0.89884823151062	-0.252317805698872	0.570311620130916	-0.0763336666666667	0.750936504909256
MAP2	-0.0500153080868442	0.898824551007336	-0.225537447321512	0.629710977741725	0.789709333333334	0.0917903726055603
FCHO1	-0.0500691529185512	0.898647505493185	-0.235955605157694	0.606121203124993	-0.120909333333333	0.691633384710545
AMICA1	-0.0500858786295703	0.898609773609494	-0.204851543889140	0.673728362275223	-0.162661333333333	0.41903055047835
RASSF6	-0.0501327258210364	0.898387113573054	-0.0484466335950747	0.942646982038187	0.00769866666666673	0.98173288785111
SHPK	-0.0501515917269336	0.898387113573054	0.0223809425195846	0.97536722461972	-0.182068333333333	0.458006502146897
ADH1B	-0.0501547447060857	0.898387113573054	0.0377612124750712	0.955190852940141	-0.259529666666666	0.250479739586124
S100A5	-0.0502115782250405	0.898248536225815	0.0353919087813556	0.95869292666698	-0.0400990000000003	0.907885119110266
FAM83B	-0.0502620275058127	0.89804783363722	0.0689569682264047	0.912149839732916	0.0274173333333332	0.93901998985171
PMCHL1	-0.0504074416428034	0.897414664923213	0.116459842881365	0.842082118939645	-0.0940893333333333	0.741809049318412
GP2	-0.0504527811441021	0.897269069853584	0.142409669312362	0.794346532964905	0.104476000000001	0.732554472781933
C3P1	-0.0504793822138422	0.897239115158815	0.213674034085632	0.653708954135733	-0.0942353333333333	0.668223322932086
RRP9	-0.0505242032761908	0.897087183823461	0.0777527084711174	0.900452756580846	-0.393544666666666	0.0669084902158622
NXF5	-0.0505372264198538	0.897087183823461	-0.0298003785611964	0.96695070705759	-0.118224333333333	0.724001791100896
NPBWR1	-0.050539530487351	0.897087183823461	0.096624944989816	0.870857630743202	-0.106405999999999	0.641044966033937
CHGA	-0.0505546121871035	0.897087183823461	-0.157539766076061	0.767770230680061	-0.0821626666666655	0.696862585388398
CCL5	-0.0505884864368347	0.897087183823461	-0.385033146896032	0.291180853572672	-0.087822	0.711082533709514
CDCA5	-0.0506782086763818	0.896751201873248	-0.00520177476838474	0.99251028338798	-0.0607299999999991	0.798736789384715
PVRL1	-0.050733604172065	0.896730871384917	0.124668361792138	0.82479991054473	-0.00622266666666651	0.987937064305561
CCDC15	-0.050770683996585	0.896665475803386	-0.168038014342868	0.748024524669236	0.369125666666667	0.16995621050072
UCN3	-0.050777064516249	0.896665475803386	0.204538549783700	0.674509419583968	-0.234097333333333	0.317705658063493
CYP2C9	-0.0507835252815398	0.896665475803386	-0.0914343263510279	0.880010161938784	0.0121320000000006	0.967241125442187
TMEM176A	-0.0507981170962244	0.896665475803386	-0.299359909613225	0.468320582546323	-0.0584529999999995	0.855317148872886
NRGN	-0.0508188755336592	0.896640814018376	-0.157370056137615	0.767770230680061	-0.00760733333333298	0.98256318768706
AFMID	-0.0508230253155002	0.896640814018376	0.132378078294376	0.810146647283787	-0.079816666666666	0.720576971539962
LINC00483	-0.0508241344665546	0.896640814018376	0.147934952199176	0.784090188380266	-0.0837866666666656	0.7996066102903
ZSCAN1	-0.0508353991465252	0.896640814018376	0.0971143331964017	0.870020870354202	-0.0650999999999991	0.778083808586846
PDGFRL	-0.0508383353693238	0.896640814018376	-0.095998003462124	0.872311810447594	0.501762666666667	0.0782543184284344
IFNGR1	-0.050893083341653	0.896574632803822	0.171403380910017	0.73955239116035	-0.0417068333333329	0.874589840804364
ZFAND5	-0.0510940792917691	0.895663822638835	-0.123340390514914	0.827531665631642	-0.010043333333332	0.974372609405579
MALL	-0.0511279350975601	0.895520646100727	-0.63238748656673	0.0208232988311238	-0.104237333333333	0.697775517025997
PHF16	-0.0511389342442682	0.895517991517654	-0.188414952210352	0.707361603786947	-0.486010666666666	0.034523754310686
SLC30A3	-0.0511634180563651	0.895497402843643	-0.31272625164005	0.439221508111214	-0.109711333333333	0.627452499444403
DEFB121	-0.0511851231099261	0.89542891922674	-0.366287300724007	0.328771652750088	0.0484133333333331	0.833097445346985
CRYM	-0.0511920213567136	0.89542891922674	-0.207747698554474	0.667695745454683	-0.00977566666666621	0.98173288785111
RHOT2	-0.0511970881013783	0.89542891922674	0.191231447729837	0.70194618646891	-0.128251666666666	0.522830837265953
HPRT1	-0.0512134424298452	0.89542891922674	-0.0160721160629427	0.982057247916055	0.243514000000001	0.234508116088724
TOMM34	-0.0512418748191096	0.895404864222497	-0.0504036246064717	0.940709772508288	-0.447483333333333	0.0354366517739437
STIM1	-0.0512480165432545	0.895404864222497	0.295286459583641	0.478332806543771	0.458767000000001	0.0338015945178966
BEX1	-0.0512648071618201	0.895404864222497	0.0253531987955488	0.972264538231849	0.153476000000000	0.5293518047673
PIK3C2G	-0.0513069274606803	0.895329768787806	-0.216677302256099	0.647357580911858	-0.170992000000000	0.469591696339734
SPI1	-0.051374949815481	0.894976330131856	-0.0539772151530639	0.93518107282108	-0.190190666666667	0.527320166803475
CDC40	-0.0513829435584053	0.894976330131856	0.156925814538119	0.768401006769299	-0.0767829999999988	0.731003031373733
GAL	-0.0513912423012373	0.894976330131856	-0.327868118319010	0.407732856921131	-0.208030666666666	0.443378989641463
SELT	-0.0514169946559737	0.894912692117592	0.110259509739150	0.8502509043242	0.135377833333333	0.499554381454891
GDF2	-0.0514251047037878	0.894912692117592	0.381260534874996	0.297930089233645	-0.196340000000000	0.575378149225683
SLC12A5	-0.0514292601626854	0.894912692117592	-0.0739586625524576	0.905506084101768	-0.0935039999999998	0.659835984620576
BRDT	-0.0514398166776198	0.894912692117592	0.0260744352768102	0.970711985190733	0.123718333333334	0.635117428927823
FAM205B	-0.0514764844033169	0.89488620036876	0.350778568940583	0.357367124206561	-0.042512333333333	0.861165447055876
CXorf40B	-0.0514769863220452	0.89488620036876	-0.456368426656180	0.16754724558121	-0.209163333333332	0.276699699174342

CDKN2AIP	-0.0514923523606788	0.89488620036876	0.206997698787174	0.669713208801491	0.0913136666666676	0.697816760927068
CXCL16	-0.0515258807410257	0.89488620036876	-0.254273212695019	0.567694846153007	0.196586000000001	0.314112513261693
ZNF25	-0.0515358913815372	0.89488620036876	0.0290304770054727	0.96782691865619	0.00795833333333459	0.983736605119045
PJA2	-0.0515731959783131	0.894796889684755	-0.325908864523156	0.409206882509236	0.204395000000002	0.279295754475649
SGTB	-0.0516305299243725	0.894769273276044	-0.384634731450469	0.291180853572672	0.341751333333334	0.0907526139873654
BCL6	-0.0516626074688822	0.894636843448582	-0.0778583999166386	0.900076100287813	0.216698666666668	0.376224594108747
NUDCD1	-0.0516806426373604	0.89462618844183	-0.124517719984446	0.824974271393593	-0.218201333333332	0.405044878280701
SLC35D2	-0.0517298407151265	0.894548260376555	0.0674493586177473	0.914318618000915	0.342617000000001	0.0804638987444807
ACTR1A	-0.0517527552386445	0.894482033925895	0.0466073059429348	0.944599178367537	-0.0207736666666651	0.937717709824356
FNDC5	-0.0517773866248994	0.894482033925895	-0.126427674977388	0.821657368966329	-0.0907566666666661	0.664617185770066
EHD3	-0.0517782937032856	0.894482033925895	-0.0745080505686972	0.904832407812985	0.0973800000000007	0.709458407711009
SNRPA1	-0.0517910182570493	0.894482033925895	0.0463339967688938	0.945000118027662	0.239431000000001	0.390452785696877
GCG	-0.0517928343680271	0.894482033925895	-0.328509308568417	0.406864543297514	0.0542200000000003	0.83859650506788
VPS4A	-0.0518202691173641	0.894482033925895	-0.142602830878604	0.794041118363265	0.0150649999999997	0.962692453690766
FAM132B	-0.0518654259420503	0.894364194388683	0.177130727746481	0.729231283096295	0.00898900000000091	0.983159241812861
RAB1B	-0.0518691113765946	0.894364194388683	-0.291063853059934	0.486880079690803	-0.112266666666666	0.589826330962094
MPND	-0.0519270323695496	0.894232585036227	-0.0382718072294858	0.954545200586067	-0.0835256666666654	0.741438256509195
ATP5G2	-0.0519508049169661	0.894228924964409	0.0275090685310355	0.969866457453454	0.078934000000001	0.729303008652613
FLJ90680	-0.0519655703692475	0.89420307729556	-0.0184450378422926	0.97902968742576	-0.0317726666666660	0.921546617328678
IRAK1	-0.0519696549867729	0.89420307729556	-0.370538294720179	0.320801726666339	-0.481801666666666	0.0674510226934292
CD320	-0.0519731869646317	0.89420307729556	0.0761566965595097	0.903296922694955	-0.074878999999999	0.741438256509195
SLC50A1	-0.0519785171290903	0.89420307729556	-0.0164394299595839	0.981892085795004	0.203721666666667	0.312084999110155
CAV3	-0.0520575086751692	0.894092523549163	0.00900897239255834	0.98909166696601	-0.0106950000000002	0.982375192575954
DLK1	-0.0521088168414907	0.893841391787749	0.0231970699426851	0.975348968561756	0.135558333333335	0.584153892885306
SNRPE	-0.0521689150905819	0.893666385325808	0.0241750705083155	0.97420021343239	-0.0998694999999992	0.696194439936211
GADL1	-0.0522050175183614	0.893574133942386	-0.0103248808553551	0.988080075330206	-0.0241339999999998	0.931055338143178
LOC100128644	-0.0522732784612603	0.893372539887668	0.236289865160969	0.605285787761649	-0.0522580000000001	0.81742145099016
FAM32A	-0.0523070033442517	0.893372539887668	0.181225067257687	0.721380104991863	0.0455696666666683	0.844766787725698
R3HDML	-0.0523122629177825	0.893372539887668	0.0704600109374516	0.910280811140495	-0.114396000000000	0.675449330308211
FAM221B	-0.0523471676998022	0.893349352959632	0.115562512722908	0.843372362327421	0.133009666666667	0.60378773986659
NECAP1	-0.052359964375935	0.893349352959632	0.0689508374691743	0.912149839732916	-0.0150256666666664	0.957940366305124
GRTP1	-0.0523673530630679	0.893349352959632	0.305159449546955	0.458013550008763	0.0500860000000009	0.8257633904865
LINC00472	-0.0523958388674959	0.893309945712506	-0.334315725457363	0.394691211498274	0.026348333333334	0.92127681920952
VPS35	-0.052555835698998	0.892741107951461	-0.314184098086453	0.437675147805387	0.0158746666666683	0.954180927004992
HIBCH	-0.052566515631808	0.892741107951461	0.0494050017200557	0.941842562680095	0.151022666666667	0.508507145194664
TYK2	-0.052575016587021	0.892741107951461	0.115480470528081	0.843609735991731	-0.176211333333331	0.413630180206496
COCH	-0.0526426443862146	0.892634934873876	-0.168785931256551	0.746214650681792	0.348949666666668	0.0777324007395152
C10orf85	-0.0526498916468626	0.892634934873876	0.040856293421484	0.952426531595115	0.00455866666666731	0.9879739922351
PDE8A	-0.0526615589213573	0.892634934873876	0.290985110314756	0.486880079690803	-0.0655679999999992	0.786118160890663
HTRA3	-0.0526689311733814	0.892634934873876	0.295793460032547	0.47761182409076	-0.0411356666666662	0.890511682678135
HEMK1	-0.0526963157147626	0.892634934873876	-0.096172208882782	0.872150099358002	-0.0230653333333325	0.932154136447394
PPEF2	-0.0527108126240112	0.892634934873876	-0.198853340445532	0.68535210394429	-0.158032666666666	0.612697324680132
GATSL3	-0.0527786250742417	0.892426514901464	-0.245533129731038	0.584539225718403	0.261834666666667	0.173286092065797
CCDC146	-0.0527791342428803	0.892426514901464	-0.225662329942274	0.629536581746323	0.210228333333334	0.271568162967415
DLX2	-0.0528278457954369	0.892405136303882	-0.0538287613249965	0.935404504538153	0.558930000000001	0.0417610099358502
IP6K3	-0.052839225888828	0.892400138598471	-0.0626735010898377	0.922350559812086	-0.143948666666666	0.536320838519271
TAF7L	-0.0528847717854461	0.892314706539295	-0.0540257419169922	0.935109055935003	0.0781080000000006	0.732554472781933
LRRK1	-0.0529192688248074	0.892261446447933	-0.208492167530952	0.666505547128851	0.109248666666667	0.647737415202745
SPPL2C	-0.0529298597830432	0.892261446447933	0.0895384597869691	0.883258404969667	-0.124692666666667	0.739738232644097
KRT4	-0.052982403256746	0.892168725194482	0.115608261151940	0.84334641337026	-0.0660466666666664	0.76368723681351
SULT2B1	-0.0530485158124144	0.891890890788231	0.0499332018193917	0.941458038227918	-0.157718666666666	0.484208511056468
LOC100130815	-0.0530486091145125	0.891890890788231	0.296330084867188	0.476715216862939	0.0429926666666668	0.908473154502781
RNF216	-0.0530564483067369	0.891890890788231	-0.214354839930689	0.652991165086813	0.0298606666666688	0.922074388102783
UXS1	-0.0531141313169802	0.891681403525438	-0.296369548612268	0.476715216862939	0.0915493333333352	0.670113640649168
REN	-0.0531219881844824	0.891681403525438	0.199248331654680	0.684227048797313	0.0161710000000002	0.966813448118178
LRG1	-0.0531370961205355	0.891681403525438	-0.246732257617247	0.581387364514747	-0.183786333333333	0.414405354802607
GAS2L1	-0.053143268727427	0.891681403525438	-0.162868362147138	0.75703912930782	-0.0222323333333337	0.938640008704917
SLC39A6	-0.0531464315598136	0.891681403525438	-0.1753961517867	0.73213520578756	0.076019333333334	0.756403310280114
MRPL19	-0.053179446126773	0.891681403525438	0.0757362368033955	0.903510413986113	-0.0271863333333329	0.91402300740264
C17orf104	-0.0531990700991532	0.891681403525438	-0.235886582774952	0.606283218699268	-0.0619863333333330	0.784195924611017
ZNF738	-0.05320342187825	0.891681403525438	-0.183728380258341	0.718067440349544	0.209432333333335	0.391462160139671
CLEC1B	-0.0532171733163773	0.891681403525438	-0.289699305121845	0.488870852256136	-0.193624333333334	0.376020235114735
MAG	-0.0532682795161658	0.891643339176029	0.035367700375973	0.95869292666698	-0.0743639999999995	0.770261991339376
IDNK	-0.053275591548482	0.891643339176029	-0.0788589797725364	0.899154242391172	0.088802666666667	0.701993852257083
RPF1	-0.0532862090005505	0.891643339176029	-0.368112947670288	0.325826074795982	-0.0930876666666656	0.705698790800484
ALAS2	-0.0533153776669542	0.891643339176029	-0.19572571349523	0.693267493630778	0.00314433333333377	0.993677488355417
RAB37	-0.0533202422100395	0.891643339176029	0.000963620623584468	0.998745415362821	-0.0121043333333328	0.96701817021473
ZCCHC3	-0.0533505304809761	0.891643339176029	0.176456703445141	0.730152680173573	-0.120792999999999	0.563374114368038
RPS19BP1	-0.0534187023924811	0.891527232005408	-0.283415567030405	0.502129505146473	0.0192916666666676	0.94375604597120
UBC	-0.0534315388562917	0.891527232005408	-0.300143746852865	0.466180114981519	-0.0147566666666637	0.95874970634395
ANAPC10	-0.053464530750813	0.891410478743677	-0.169293330621533	0.744676064883446	0.0515643333333339	0.83254569616527
UGP2	-0.0534800931280913	0.891379618887054	0.080925924179107	0.896361013063453	0.187616333333333	0.324937679238988
RRAD	-0.0535596963818737	0.891083369203719	-0.344215152407055	0.371106177422032	-0.00767733333333323	0.98349947509937
A2ML1	-0.0536525480856015	0.890639711287754	0.191709535367715	0.701544026774214	-0.136113333333333	0.588052519326463
MFSD10	-0.053659674220215	0.890639711287754	0.0663895428469475	0.916323048676587	0.0912880000000013	0.665288826265065
TMPRSS11B	-0.0537147025587437	0.890516740047738	0.301029477343725	0.464782507068736	0.124787666666667	0.535492155031224
PSEN2	-0.0537264768013027	0.890509288118915	0.0489340833109504	0.942180113539637	-0.346466000000000	0.167277848166228
GLRA3	-0.0537365409554835	0.890509288118915	0.126728381894546	0.820923134671156	-0.0137553333333331	0.974451420920923
DPPA2	-0.0538861319232563	0.889782951833827	-0.253399459086106	0.567705016149998	0.090555666666667	0.721039266334933
LYL1	-0.0538981493913932	0.889773967226979	-0.00832545432049305	0.989949154584002	-0.132117666666666	0.635267430402483

GSTM3	-0.0539118020296431	0.889771826790456	0.159378692610740	0.764323045210806	0.285799666666668	0.204717851336330
GH2	-0.0540245972129051	0.889344857703458	0.198699755084253	0.685703984501304	-0.104683333333333	0.735529468860672
ZNF383	-0.0540303206547669	0.889344857703458	0.0286182458157607	0.96782691865619	0.142937333333335	0.567291668977137
SCARF1	-0.0540369443560529	0.889344857703458	-0.308321664876778	0.450974903594884	-0.0688833333333325	0.827586713844745
CCNG2	-0.054146475210935	0.888956321425301	-0.196939309075984	0.690061983084583	0.0588636666666684	0.811816884537132
ULBP3	-0.0541622077401068	0.888924319241248	0.126099735728987	0.822164329801713	-0.26957	0.244815449905264
IL12A	-0.0541837221984839	0.88885652835451	-0.162197662147637	0.758353674758641	-0.566095666666666	0.191214943102167
TMC6	-0.054213319615188	0.888845981092287	0.0418626381811554	0.951989245458556	-0.090061999999999	0.726110499746352
SLC35F4	-0.0542219939398612	0.888845981092287	-0.232314740023888	0.612658112747496	-0.0156343333333335	0.966342921970461
RPE65	-0.0542552425373043	0.88880609606771	-0.154843756509353	0.771310656070265	0.0119623333333336	0.9681321732097
FAM189A1	-0.0542635611712247	0.88880609606771	0.0452749224361947	0.946556809762233	-0.126363333333332	0.661476512366005
CNTNAP4	-0.0543211360453723	0.888528919456341	-0.0396266201788165	0.953729831316741	0.136857666666667	0.535487400963825
RAPGEF1	-0.0543763577994243	0.888429735646467	-0.209082204557028	0.665595909869988	0.0670286666666668	0.786670898149409
WDR83OS	-0.0544325004507618	0.888386769462208	0.0953755079637972	0.872881588028418	0.0286623333333341	0.910625003107163
KIF9	-0.0544395662069368	0.888386769462208	-0.0553787286683293	0.933409139126562	-0.338375666666667	0.108563852880459
ARIH2	-0.05453137985579	0.888261894607364	-0.10577574941644	0.859545278949543	-0.0297643333333331	0.910190419163007
PTPN12	-0.0545493925000648	0.888261894607364	-0.269813542587901	0.531534905802895	0.322567000000002	0.0917903726055603
GJD3	-0.0545742772049074	0.888261894607364	-0.0896067663754754	0.883196389963269	-0.179592333333333	0.479990255884948
GJA4	-0.0545838149566223	0.888261894607364	-0.371625117930659	0.319527591032365	-0.188259999999999	0.59717035375984
GEMIN8	-0.0545973680178956	0.888261894607364	-0.198816955165923	0.685476830270855	-0.195245999999999	0.373594956320212
DNAJC27	-0.054597394825065	0.888261894607364	-0.0226390319223366	0.97536722461972	0.0568820000000018	0.805801105047125
INHBC	-0.0546092598678782	0.888261894607364	0.132491384348914	0.809866082339655	0.0842970000000011	0.710004432586908
DQX1	-0.054654732361142	0.888261894607364	-0.0054502950920905	0.99251028338798	0.118957333333334	0.640111005310948
ZNF599	-0.0547084151290163	0.888158417438098	0.298156524457724	0.471912379968758	-0.0511049999999994	0.823833399538487
NFKBIB	-0.0547179901473423	0.888158417438098	-0.194914951529067	0.695405358987753	0.0024806666666674	0.993756838831997
LGALS8	-0.0547851850834949	0.88781347702276	-0.269603166769793	0.532107940833389	-0.306065333333333	0.232564672197746
CLCN4	-0.054789675010936	0.88781347702276	0.161480314667491	0.759189376564965	-0.0476526666666667	0.883478349705756
BCL2L10	-0.0548578237302084	0.887621363510506	-0.207878949488892	0.667695745454683	0.410739333333334	0.114877475958037
TCEA2	-0.0548713270980052	0.88760688213753	0.200597358020275	0.680830337398347	0.146489000000001	0.480339629154742
ZP1	-0.0549582951314447	0.887198635358745	0.152338556678629	0.775863712880333	-0.103346666666667	0.674409229672538
FYCO1	-0.0550454434822331	0.88685472631319	-0.276020282529502	0.517549833373363	-0.195217666666667	0.4584541407185
OR10A4	-0.0551054697734395	0.886548023869141	-0.237507969873081	0.603017682513881	-0.0946319999999995	0.709692328724711
TNRC6A	-0.055130421417817	0.886524234006866	0.317477357183262	0.431166148274203	0.153282666666667	0.503400741258744
TNP1	-0.0551462753335275	0.886491393709598	0.288638543618296	0.489813374112312	-0.0825429999999997	0.752992371482229
CCDC141	-0.0551646240267413	0.886443082725492	0.344314787100365	0.371073436162504	-0.955645333333334	0.00629285391915396
LDHC	-0.0552368312268468	0.886060817636859	0.0583995466852746	0.928725686060612	0.122325333333333	0.574278554232414
HEATR2	-0.0552810404388852	0.885948847023938	-0.255673764877319	0.565656702091657	0.0144166666666682	0.959218131095776
KIF26A	-0.055288765695973	0.885948847023938	-0.130397010744344	0.812271254601992	-0.0636126666666665	0.788201431210286
IMPG2	-0.0553016126433266	0.885948847023938	0.140823725943821	0.796714214272984	0.196452333333333	0.301793253300179
TFDP3	-0.0553459423348733	0.88577691661589	-0.0668694606826273	0.91517239775041	-0.032217666666666	0.911393693073216
WNT2B	-0.055380023816322	0.885682887391673	0.165285950683102	0.752544465367249	-0.0101579999999998	0.982940344703817
FMNL1	-0.0555133720297108	0.885008852189839	-0.156431844662328	0.768909201237015	-0.102099666666667	0.724001791100896
CACNG7	-0.0555259908543204	0.885008852189839	-0.0228688325548656	0.97536722461972	0.0284913333333345	0.924469572710745
NRIP3	-0.0555568898841691	0.88499222404441	-0.0779794890230385	0.899705053748212	-0.0352989999999993	0.907374738017166
TINAGL1	-0.0556302691503865	0.884696166751604	-0.231946160061583	0.613445765196643	0.0208956666666666	0.944218475609619
FARS2	-0.0557220084380439	0.88436068540162	0.0160882783393156	0.982057247916055	0.291117333333334	0.167001732057148
TXNL1	-0.0557424445586487	0.88436068540162	-0.0586828256916656	0.928567813924053	-0.0727816666666653	0.741438256509195
MARK2	-0.0557464169870846	0.88436068540162	-0.280290765348358	0.507849432265103	-0.261384333333332	0.320462604748547
TGFBI	-0.0557707171768847	0.88436068540162	-0.358220082896439	0.343290799330351	0.544400333333334	0.119275755027164
B4GALT4	-0.0557769164811173	0.88436068540162	0.0495369554065788	0.94156962266198	0.224459	0.285116883835521
PSMD4	-0.0557868893886631	0.88436068540162	-0.139150420852332	0.79864602540281	-0.119081333333333	0.565215044097433
DDX53	-0.0557981846156574	0.88436068540162	-0.209290053460569	0.66471227046382	0.0707926666666672	0.78885077801625
TMED2	-0.0558237566129207	0.88436068540162	-0.0966031269503828	0.870857630743202	0.103650000000001	0.616036866881905
TECR	-0.0559963538727687	0.883901896114605	0.0407914833424813	0.952426531595115	0.0725370000000018	0.746803560287939
EVPL	-0.0560186858140674	0.883828755352398	0.24754855480805	0.579745218830252	-0.156546333333333	0.637224122022876
UBN2	-0.0560241570612065	0.883828755352398	0.0905942032744824	0.881640655873722	-0.143197	0.589826330962094
WFDC2	-0.0560501534825124	0.883828755352398	0.265412215080668	0.540632069395457	0.0220516666666668	0.944735546098178
KCNJ3	-0.0560636451736214	0.883828755352398	0.115212971984099	0.843882871027693	-0.0339209999999990	0.90945379489823
USP8	-0.0561172170754083	0.883675460521602	0.174584039457056	0.734267769889068	0.126722333333335	0.60026500215056
TTLL2	-0.0561668816041369	0.883563581166646	-0.138184540696923	0.800478820487963	-0.0653723333333329	0.818514407749293
BBS7	-0.0562400188820131	0.883316648445328	-0.141094162141270	0.796333933122002	0.152449333333333	0.49971129098408
ANKAR	-0.0562527678606787	0.883316648445328	-0.05805276494025	0.92929701257827	0.0256933333333339	0.931446587444396
CSHL1	-0.0562857224676003	0.883255416563194	0.204346382745452	0.674837470309368	0.0602250000000005	0.86715815306237
SAC3D1	-0.0563025830665753	0.883255416563194	-0.0667468528471643	0.915438681913279	-0.085999666666666	0.68149943269806
TCAP	-0.0563059002026313	0.883255416563194	0.208494723843906	0.666505547128851	-0.118620666666666	0.628477077807168
PGAM2	-0.0563107302163053	0.883255416563194	0.00775320837685143	0.990405674923721	-0.127731333333332	0.60217016768875
SLC6A7	-0.0563473590429695	0.883254006104839	0.111614924131655	0.84862264853383	-0.224605666666667	0.233528575266801
RABEPK	-0.056410569997481	0.883032784911896	-0.135186531800891	0.807470535721699	-0.113372333333333	0.600950363504414
ASF1B	-0.0564385171784585	0.882924692980274	0.0796373961973358	0.898031113962014	0.0999180000000018	0.634966811516658
C2orf61	-0.0564399934574769	0.882924692980274	-0.033657561723512	0.96061727828906	-0.0170109999999996	0.953335531487028
MTX1	-0.0564823640960192	0.882848875419211	0.156177889877231	0.768909201237015	-0.0369303333333327	0.887363245747406
SHANK3	-0.0564994796830596	0.882828095471031	-0.355295646505775	0.347833413629182	-0.105784666666667	0.694407056566956
ATXN7L2	-0.0565473179957078	0.882776443544957	-0.391950640408779	0.280021017230138	-0.00112299999999924	0.99704422037925
AIFM2	-0.056567400448934	0.882776443544957	-0.0523321510671691	0.937831566264837	-0.0889136666666665	0.752992371482229
TMA7	-0.0565751405595397	0.882776443544957	-0.0797345634532391	0.897892885174022	0.0920898333333355	0.657217907037306
LOC150622	-0.0565773869271255	0.882776443544957	-0.108395722667043	0.854315506430894	-0.0312696666666665	0.902539439290515
CYP2S1	-0.0566145142159841	0.882776443544957	0.126368808644634	0.821845554157948	0.00251266666666774	0.993756838831997
ZNF569	-0.056621666708194	0.882776443544957	-0.359094697559840	0.342594959625489	0.250126333333335	0.340052241399374
DDX47	-0.0566229845075691	0.882776443544957	0.0945842729612601	0.874061779990106	0.0290170000000003	0.922697964464038
SARNP	-0.056635347459245	0.882776443544957	-0.0234925027247351	0.975348968561756	-0.00971833333333233	0.974669893685766

LGALS8-AS1	-0.0567055052401828	0.88270782998285	0.0869485769435772	0.887065140370096	-0.122163999999999	0.687794959259872
STAU1	-0.056739489029295	0.882562114917243	-0.431338168536085	0.205718890053769	0.102874000000001	0.615807897956118
C1orf145	-0.0569219825846012	0.881820869573238	0.387877894840829	0.286134842484356	0.0248833333333337	0.931859006463093
TGM4	-0.0569914019376944	0.881585927894167	-0.062245426757616	0.92301259447339	0.0142846666666672	0.966879505998417
ASIC5	-0.0571072887947776	0.881193120289542	-0.158671989239778	0.765791046741822	-0.0562776666666662	0.808933385359076
MBLAC1	-0.0571280445191209	0.881129609913808	0.302951461714414	0.462105017205825	-0.00915366666666614	0.978173971760794
TONSL	-0.0571362430239262	0.881129609913808	0.384236509453733	0.29195109104449	-0.0819076666666659	0.735938662411184
VPS37C	-0.0571661658388611	0.881077430801614	-0.270100247820121	0.530185035699403	-0.0953393333333325	0.698368688760464
PAPPA2	-0.0571802890205311	0.881077430801614	-0.162130609149537	0.75843356082254	0.063134333333334	0.811816884537132
ERG	-0.0571853012701503	0.881077430801614	-0.203512886681525	0.676207233014711	0.110247666666667	0.66807888867384
DOLK	-0.0572135077565663	0.881077430801614	0.0853790536889123	0.889779510878572	-0.0218779999999978	0.937545603686416
KLHL1	-0.0572235789480327	0.881077430801614	-0.193166653577467	0.698832386916027	0.0446096666666667	0.883478349705756
LINC00612	-0.0572850498185841	0.880940082276584	-0.131935200475813	0.810418188352907	0.0624823333333335	0.78960166957155
TRIM5	-0.0572918142667051	0.880940082276584	-0.135163304371970	0.807470535721699	0.0112810000000011	0.970030485008698
SHISA4	-0.0573253770126552	0.880820380948655	0.318511266644408	0.4290520704081	0.0386963333333342	0.90945379489823
SEL1L2	-0.0574122542332519	0.880476598982832	0.309721068623599	0.447222629729729	0.0975783333333338	0.643688901525294
GARNL3	-0.0574527864668124	0.88034718809921	0.0660317515755266	0.917010884328	0.0517206666666681	0.828240868185377
C3orf79	-0.0574559294435257	0.88034718809921	-0.00548680231272215	0.99251028338798	-0.235133333333333	0.223260757343954
LCK	-0.0575269510551047	0.880222100304071	-0.376668336392360	0.308928897676536	-0.0142033333333330	0.963626273229967
KRTAP4-3	-0.0576008392504186	0.879906756675797	-0.0161568088035090	0.982057247916055	0.0781633333333334	0.728708142582036
KCNJ14	-0.0576130391588955	0.879906756675797	-0.386703782363947	0.288212923135036	-0.105369333333333	0.669391045681572
NFE2L1	-0.0576517590925863	0.879904587271025	0.0649449010836489	0.918489100363712	0.0883460000000008	0.695654544311453
THEM5	-0.0576581139512703	0.879904587271025	0.201354456075591	0.680054545720343	0.0292840000000002	0.914808689682189
TIMM9	-0.0576735503906613	0.879900200582135	0.114106287350733	0.844272085764738	0.272301333333335	0.164442445909549
PXDC1	-0.057685028795241	0.879894380831512	0.249153779023166	0.576149573608393	0.356911000000001	0.0769043559304575
PPP2R3B	-0.0577092609614115	0.8798748028063	-0.075761992709733	0.903510413986113	0.0510766666666678	0.830234611990443
GBP3	-0.0577606391176853	0.87968617247414	0.027235248049326	0.970207612777188	0.3851195	0.0899297181257792
C20orf85	-0.0577692859305943	0.87968617247414	0.0287369556826563	0.96782691865619	-0.0498713333333332	0.82667899725046
FKBP8	-0.0577695662035193	0.87968617247414	0.0496052796057248	0.941552283017912	0.190036000000002	0.353701605651176
NLE1	-0.0577807638666524	0.87968617247414	0.0619008349440471	0.92306630614464	-0.226743333333332	0.286939266819770
PPP3CC	-0.0578286479559103	0.87968617247414	0.272588781981754	0.526081777027135	0.0763930000000002	0.73168347388773
VGLL2	-0.0578296080041649	0.87968617247414	0.176341394405035	0.7303866161159	-0.0244233333333328	0.937471798283197
CRYBB1	-0.0578359330677298	0.87968617247414	0.177256964867548	0.728735147897621	-0.0781459999999997	0.867268897859745
LAX1	-0.0578694702834384	0.879665090052102	-0.250960677214462	0.572882168047464	-0.223135333333333	0.231095714637061
MAML1	-0.0579155188866881	0.879443894597112	0.296051980862176	0.477108218722608	-0.0868285000000003	0.768164585194196
ATG3	-0.0579508763324116	0.87928930473805	0.139286136028571	0.79864602540281	0.0380866666666669	0.87709801222343
SARS	-0.0580161928308097	0.879013745756697	-0.384561690229318	0.291180853572672	-0.0653823333333327	0.777766007902715
KL	-0.0581047889180675	0.878700945473514	-0.309832798637942	0.4470577615309	0.201741666666667	0.479138506340968
IDH1	-0.0582261539030251	0.878636642500432	0.412694324609751	0.241609582743514	0.068346000000001	0.805801105047125
IGBP1	-0.058294427553509	0.878581001150834	-0.25502295604359	0.566577861267331	-0.00288599999999920	0.993756838831997
VIPR2	-0.0583325209986357	0.878553580003431	0.134420660015007	0.808009477165608	-0.026323333333333	0.940972575250573
EXOSC10	-0.0583709585023716	0.878379647324196	-0.346873957680468	0.364568189046910	0.160821333333334	0.537186705207113
TRIL	-0.0583973743544125	0.878280673564666	0.0893655677609039	0.883460564717377	-0.0862169999999992	0.689267979966846
ICMT	-0.0584591793303177	0.878148931544193	-0.310146609715377	0.446321819288896	-0.0675139999999986	0.76397955844876
PCYOX1	-0.0585043511540665	0.878139564375156	-0.129220136009489	0.814793489052246	0.142965000000001	0.501839684921699
GOT1	-0.0585079319279029	0.878139564375156	0.00771869754763182	0.990405674923721	0.019235333333334	0.94678260229888
KRTAP10-11	-0.0585598153517346	0.878048636805355	-0.0497567969019084	0.941458038227918	-0.0647946666666651	0.840247972976129
DNTTIP1	-0.0586099191520038	0.878048636805355	-0.313422419198180	0.438100620341757	-0.0127253333333329	0.966553712068514
BMP8A	-0.0586386102606999	0.877959835595507	0.178161370760342	0.727939088352802	-0.262043999999999	0.206867875194377
LOC84931	-0.0586931958193498	0.877816625864622	0.259997466901525	0.555188100945053	-0.185436666666666	0.529127543770363
CD27	-0.0587009025583713	0.877816625864622	-0.310516470050392	0.445142823947765	-0.0878686666666665	0.704370299831112
CRYGS	-0.0587520629947203	0.877619313294037	-0.00324862595826513	0.994730189431381	-0.0418813333333327	0.87290879338693
CEP44	-0.058787486593544	0.877519253552292	-0.0133878598969443	0.98412883278237	-0.0379309999999991	0.910625003107163
NOXRED1	-0.0588291623405085	0.877479566693157	0.151349909521648	0.777604486471648	0.00359166666666704	0.993346316662499
OR2J2	-0.0588999902658241	0.877184057394415	-0.0630565805264009	0.92160309476589	0.0144888888888896	0.961993177180408
NEU4	-0.0589027540541374	0.877184057394415	0.301177129902946	0.464501110297982	-0.0985086666666665	0.774210549173745
RTP3	-0.0589252533860379	0.87715799096469	0.0719621319935873	0.90837606217316	0.103104000000000	0.685488482699362
INPP1	-0.0590962158577124	0.876419785181601	0.224292271330064	0.632829553438263	0.263303666666667	0.22316376350853
BTF3	-0.0591524909763132	0.876185376560496	-0.0185790967564579	0.97902968742576	0.00509133333333443	0.987937064305561
SH2D3C	-0.0591682873777842	0.876185376560496	-0.450243200290201	0.176663460992485	-0.141113666666666	0.648861254074544
KRT14	-0.0592472462829554	0.876036620645823	0.280503002857651	0.507460240190768	-0.181561666666666	0.667098538791373
ADAMTS18	-0.0592550369488846	0.876036620645823	-0.0413320265963511	0.952098348256047	-0.0348809999999995	0.931708923663607
C22orf24	-0.059313871634164	0.87585046854787	-0.202900995507527	0.677532075317915	-0.0659679999999993	0.789377975672973
C9orf66	-0.0593411298059337	0.875753840415294	0.0994508303069299	0.867651972215247	-0.243546333333333	0.211715611903437
F7	-0.0594351138475232	0.875549659040567	0.302146230600526	0.462229407853213	-0.061869	0.819757926662461
PAQR6	-0.0594399566214973	0.875549659040567	0.120100810205779	0.835124910268666	-0.00438499999999893	0.991175862027014
C14orf93	-0.0594574604530373	0.875549659040567	0.0163548007180519	0.981892085795004	-0.0772006666666661	0.752518458717797
PLXDC2	-0.0594840259578617	0.875549659040567	-0.169812161099444	0.743328907676018	0.615862333333334	0.0145588447152274
CCDC167	-0.059496913654831	0.875549659040567	0.141716379969220	0.795109963623224	0.0270090000000011	0.94622978991441
EXD3	-0.0595070786571236	0.875549659040567	0.204315998339108	0.674848467447322	-0.037454666666665	0.898979600726666
AQP5	-0.0595204118108398	0.875549659040567	-0.37493230208894	0.313089039968076	-0.134445333333333	0.624144610798661
SH2D4B	-0.0595855327857705	0.875338529978458	0.256451659467815	0.564430426783556	0.00671000000000026	0.985884500368235
LRRC52	-0.0595969698063721	0.87533292436403	0.064052900130631	0.92003791975205	-0.0879939999999996	0.73097308185803
AHDC1	-0.0596147646907831	0.875287588737857	0.37864091623853	0.304578046752771	-0.051675666666666	0.838927397112197
TNFRSF18	-0.0596333213285648	0.875280229493012	0.00662668927988359	0.991309497300929	-0.085560333333333	0.789135781833215
PIN1P1	-0.0597094340631011	0.875025291327684	0.163745118471367	0.754968203901817	-0.020357666666666	0.941559580750404
WIPI1	-0.059737964279775	0.874912848135513	0.414473677441961	0.237624740202977	0.229710666666667	0.330549671750340
DENR	-0.0597394709830827	0.874912848135513	-0.0983613807131864	0.86848759013952	-0.150791999999999	0.480167951483741
PEX6	-0.0597557407186416	0.874912848135513	-0.0354045120564194	0.95869292666698	0.0609113333333349	0.789105818888855
CMKLR1	-0.0597578952592893	0.874912848135513	-0.283344769604705	0.502129505146473	0.140664000000001	0.560413476606931

RPRD1B	-0.0598077097636973	0.874912848135513	0.0599104685796607	0.926038115028264	-0.160489333333331	0.447498161237612
ZNF232	-0.0598726248881241	0.874795817456957	-0.09939601615508	0.86769814401307	0.0759393333333341	0.813401054179134
GRK4	-0.059883315797643	0.874794878596055	-0.207425776460201	0.668625825887966	0.224922333333334	0.305101612380486
ASTE1	-0.0599148481338918	0.8747887913695	-0.0838508257291744	0.892833228561937	-0.0507773333333323	0.865753401628855
NDUFAF3	-0.0599214993987119	0.8747887913695	-0.133251044482260	0.808992949393043	0.160613000000001	0.422658139524950
MBTPS2	-0.0599350420427515	0.8747887913695	-0.0248558285009391	0.972934354938456	0.355331666666668	0.0689724571534153
FSTL4	-0.0599516096509472	0.8747887913695	-0.157898901759363	0.767373487136803	0.173967666666668	0.64056420850272
RIOK2	-0.0599691855438389	0.8747887913695	-0.200539687979917	0.680830337398347	-0.00258599999999875	0.994738356997797
MUC7	-0.0600627349633745	0.874529858042039	0.0407266738616041	0.952426531595115	0.208532333333334	0.288015995370752
SREK1	-0.060067663759888	0.874529858042039	-0.0635099551788103	0.920855211745173	-0.077629	0.772520347452753
APOL4	-0.0600874132708658	0.874529858042039	0.0175103736903432	0.980496266838056	-0.157821000000000	0.420336551307248
CMYA5	-0.060125300412337	0.87451971763259	-0.132465732181182	0.809866082339655	0.113265	0.635267430402483
SAT2	-0.0601864778978108	0.874481334384805	0.450728932138281	0.176663460992485	-0.0819359999999991	0.73257024190143
FAM136A	-0.0602369816471686	0.874341539027338	-0.229277365731131	0.620409044581731	-0.172947666666666	0.370208571402686
NDFIP1	-0.0602515041828952	0.874338337433313	-0.105243874630939	0.860230380568262	0.200538333333334	0.314112513261693
ZDHHC11	-0.060312310620428	0.874221744900014	0.207668835711475	0.668036634256494	0.188559333333335	0.397798624983652
MSH4	-0.060317906633784	0.874221744900014	-0.279053734011667	0.511025440770733	-0.0188199999999998	0.954021566744527
CD70	-0.0604276506080888	0.873697647229602	0.204779011774544	0.673728362275223	-0.188162000000000	0.441495128589173
SLC25A14	-0.0604307664327901	0.873697647229602	-0.488248637824466	0.127979276934222	-0.0831619999999987	0.695325192692824
SSH3	-0.060456028093441	0.873651995864866	0.0205329396765903	0.976412247012224	-0.231038333333332	0.380263208996996
MGC45800	-0.0604768901423386	0.873651995864866	-0.265103783393303	0.542001377902249	0.0241073333333336	0.957251386058617
DERL2	-0.0604901865276978	0.873651995864866	0.0334851276974508	0.960784250392482	0.062809666666668	0.778707619349979
KRT72	-0.0605980092272501	0.873423142538837	0.116815244740497	0.841562044027187	-0.0636933333333329	0.822390330537826
DPAGT1	-0.0606263698862632	0.873311542871549	-0.208604452468748	0.666278181721133	0.0489650000000005	0.84602179705003
IGSF21	-0.0606280808126185	0.873311542871549	-0.129968900054319	0.812986631487699	-0.163556666666667	0.529034261534788
RND2	-0.0606482223006684	0.873311542871549	-0.180378177035078	0.722985510661031	-0.137377000000000	0.572207354314478
DENND1B	-0.0606497348606449	0.873311542871549	-0.177309447065630	0.728735147897621	0.490020333333334	0.0656979579234737
FSHB	-0.0607000136570072	0.87318046999526	0.123859389651249	0.826287181959559	-0.10249	0.707538975864338
EPHA5	-0.0607415332868062	0.873098029678284	-0.0430099090433574	0.950146638225747	-0.0710766666666663	0.742452599762094
ASMTL-AS1	-0.0608164888220543	0.872917574203327	0.159068632582678	0.765314332211235	-0.101733166666667	0.7556715716883
TRUB2	-0.0608324113735046	0.872917574203327	-0.0552831144891963	0.933521481800723	-0.0826389999999996	0.695962292251784
FBXL19-AS1	-0.0608392747916225	0.872917574203327	0.182872872391878	0.718289071039953	-0.105933999999999	0.650380062072748
AP4E1	-0.0608888257784929	0.872917574203327	-0.0307638651782228	0.96529904314994	0.161702666666668	0.462444913819465
SPATA22	-0.0609169168011828	0.872851713364954	0.00948980708424275	0.98909166696601	-0.0402143333333334	0.891074831556373
WBP11	-0.0610027617265438	0.872538725230214	-0.00907459374626957	0.98909166696601	0.0134136666666667	0.96797990593595
MAP3K3	-0.0610074258547742	0.872538725230214	-0.292921130248809	0.482504793686366	0.302731000000002	0.186080448828299
AURKA	-0.0610151276519373	0.872538725230214	0.0650523957770209	0.918489100363712	-0.0613446666666656	0.807533339772738
LOC541473	-0.0610196282856251	0.872538725230214	-0.0469404901191396	0.944157809762463	-0.0472899999999995	0.849168149250465
AGPAT2	-0.0610943337251447	0.872368746416083	0.213823487883319	0.653708954135733	0.150604333333334	0.473543693383828
SLC35E4	-0.0611149120721886	0.872364501552795	-0.0831289121838027	0.893287218356307	-0.0359429999999991	0.887363245747406
LMBR1L	-0.0611617379291361	0.872203118133727	0.143607678390899	0.79255044057935	0.0707793333333336	0.770226766814377
TAF1L	-0.0612111042738031	0.87195966722456	0.22353102923396	0.63432439947046	0.0722173333333341	0.780619140960946
IGKC	-0.0612244765583878	0.87195966722456	-0.255488307897489	0.565900677438537	-0.0834938333333335	0.733867173296405
FUT3	-0.061285835976083	0.87174572365585	0.230639754357438	0.617417844559199	-0.242045333333333	0.343572153228906
B3GAT1	-0.0612918510045257	0.87174572365585	-0.0464842078211882	0.944839700668385	-0.0334089999999985	0.90945379489823
ANKRD34C	-0.0613072911110248	0.87174572365585	0.0406359466247546	0.952426531595115	-0.222195333333334	0.441909622750662
KYNU	-0.0613783821885972	0.871439557350463	-0.115300268706439	0.843755596233057	0.0985386666666667	0.719304894613107
TFAP2B	-0.0613793001684782	0.871439557350463	0.267076993626350	0.537040363221995	-0.166216333333333	0.498454352317107
MYOZ2	-0.0613948448791507	0.871439557350463	-0.164100967103711	0.754243267462143	-0.0286069999999995	0.910190419163007
ACTR10	-0.0614196739968888	0.87138218436233	0.0900475321590361	0.88237793095104	0.140946000000001	0.483452617329376
GRIK4	-0.0614551381526216	0.871302289530923	0.0536719175225088	0.935777496327359	0.198978	0.318538641774025
KCNB2	-0.0614570993530836	0.871302289530923	0.0639136808401397	0.920312799155406	0.0627203333333335	0.819757926662461
SYN2	-0.0614944211281257	0.871302289530923	-0.0134953815803862	0.98412883278237	-0.098952999999999	0.707538975864338
KIAA0232	-0.0615458449245181	0.871182677493147	-0.192284609209757	0.700640530210457	0.0433650000000005	0.866671423175521
HSPBP1	-0.0615562909196363	0.871182677493147	0.0358428355671943	0.958354725588542	-0.0522326666666655	0.853541852582193
GGTLC2	-0.0615570534233801	0.871182677493147	0.0166847161230587	0.981403248174786	-0.254118333333333	0.241273257416922
APOL2	-0.0616297873747962	0.871140076379319	-0.055864953903038	0.933151248545599	-0.211788333333332	0.348000484523924
STX19	-0.0616329935275222	0.871140076379319	-0.329492833812551	0.404377515671201	-0.00551566666666592	0.988133186272453
SLITRK3	-0.0616943160255314	0.871130166673945	0.194401002622727	0.696294554451457	-0.0216709999999994	0.962552669872023
PP2D1	-0.0617190267159619	0.871088531509913	0.155870348535078	0.76972820876143	-0.0716303333333328	0.774104133965233
BTBD10	-0.061743662745279	0.87100006692858	-0.499379963973655	0.113777333040600	0.164987000000002	0.4423818632121
LRIF1	-0.0617711619180069	0.87089362099147	-0.161820730393956	0.758930446817487	0.222964000000002	0.321629187299521
MYO1F	-0.0617714243781612	0.87089362099147	-0.359862694863172	0.341510139061518	0.0467416666666677	0.864702194246548
HAUS3	-0.0618329135446492	0.870843244782754	-0.138868117040762	0.79879462261805	-0.189724	0.348000484523924
PPM1G	-0.0618503638787233	0.870843244782754	-0.079753028945599	0.897892885174022	-0.142978666666666	0.479961400228675
C16orf82	-0.0618920229124423	0.870730735987101	-0.0338009721475184	0.960373493629314	0.0820860000000004	0.741438256509195
ZNF700	-0.0619075395181518	0.870699525460283	0.160189406667393	0.762736287560146	0.184017333333334	0.379613779505161
THAP11	-0.0619552117420901	0.870466327158775	0.167568961148550	0.748845004143384	-0.0437539999999996	0.868203945159707
XCL1	-0.0619833062748644	0.870376452297217	-0.365156312107328	0.330886935712135	0.307449333333334	0.414421695755813
KLHL9	-0.0620470327339954	0.870154532034732	-0.0394579566883354	0.953905313456582	-0.115965	0.578893615511221
PIP4K2C	-0.062089228591641	0.870154455394063	0.148438681751265	0.78374265784053	-0.157270333333333	0.432960172695455
ZNF408	-0.0620931021682331	0.870154455394063	0.0382717913536287	0.954545200586067	-0.0673409999999995	0.772520347452753
ZBTB4	-0.0620990500850772	0.870154455394063	0.0769931825994194	0.901685964013167	0.0394506666666677	0.902208543897795
CES3	-0.0621295758918771	0.870099746995218	-0.174965597669139	0.733518674801325	0.0394730000000002	0.87279470391545
KIAA1456	-0.0621835709900617	0.869893002386717	0.143928122637988	0.791730335952443	-0.0364809999999992	0.885895416574211
MAK	-0.0622406233540251	0.869733298487778	-0.308219061623621	0.451118841045994	-0.068066666666666	0.77394434422275
TRAF2	-0.0622443028338047	0.869733298487778	-0.154484173246672	0.771310656070265	0.106106333333334	0.671766969198954
FAM73B	-0.0622708058301716	0.869676167865766	0.0219770687396879	0.97536722461972	-0.0604769999999985	0.78960166957155
POU3F4	-0.0622928743269085	0.869643000327144	0.315778623120045	0.433922711983664	-0.0408099999999989	0.880131955403697
CECR6	-0.0623297285959286	0.869550559592978	-0.184062342373952	0.717937553844852	-0.0818513333333324	0.743876813090318

NMUR1	-0.0623577363052235	0.86946120972868	0.181912669844959	0.720096350499662	0.0564310000000012	0.867268897859745
FBXO44	-0.0623656048156443	0.86946120972868	0.190209233854631	0.704208582824765	0.112907000000001	0.692043732062832
NUMBL	-0.0624043695945414	0.869300660242297	0.0417867945681144	0.951989245458556	-0.000133333333333236	0.999663546823165
MMP24	-0.0624963463360203	0.868921358564838	-0.0514705972573938	0.938728344711776	-0.150926000000000	0.534206202440822
DPM2	-0.0625824307630087	0.868645326050901	-0.0594425514619474	0.92694538699714	0.0874056666666668	0.678446375164533
CAPZB	-0.0625982082767613	0.868645326050901	-0.386983836503649	0.287619781745386	-0.0379033333333318	0.878596645532389
DKK2	-0.0626365571408566	0.86863645050874	-0.481917339263158	0.135560525489292	-1.43295500000000	0.00639136733370728
SOX15	-0.0626553482562587	0.868634296412632	0.0277286607716678	0.969556260869396	0.0186836666666673	0.95482028291900
LOC100130691	-0.0626638916054814	0.868634296412632	-0.0309950760227499	0.96518721623229	0.186082000000000	0.479138506340968
CD300LB	-0.0626672445546419	0.868634296412632	0.0719949580098883	0.90837606217316	-0.210627333333333	0.310158645250256
RRH	-0.0626726882382232	0.868634296412632	0.0265095883702447	0.970209453484284	-0.0664373333333335	0.814780717126634
PKP2	-0.0626990267707446	0.868634296412632	-0.158052338108804	0.767373487136803	-0.196502333333332	0.520424642482869
ICA1L	-0.0627435289812232	0.868626886201154	0.175732601902239	0.731836596769375	0.432711000000001	0.0641972703246714
ENTPD4	-0.0627692518722348	0.868531418443601	0.0124566235594331	0.985220352735461	-0.0170646666666665	0.95285450244329
PROCA1	-0.0627969506068375	0.868497498556366	-0.0235192112923214	0.975348968561756	-0.0475300000000001	0.879730542916545
KRT40	-0.0628839446972245	0.868197487181392	0.00516980961239957	0.99251028338798	-0.0226919999999996	0.946623914607177
EIF2S2	-0.062942665467355	0.868012461033443	-0.321266246063926	0.421365232679782	-0.144595999999998	0.550660741372463
PAQR5	-0.062993579447439	0.867783548500798	-0.137938681827211	0.801555307281312	-0.0650003333333330	0.7996066102903
ZNF813	-0.0630217637712328	0.867738895598387	-0.216779176655552	0.647357580911858	0.197802000000002	0.562674320626673
STEAP3	-0.0630273235221096	0.867738895598387	0.0696621130278092	0.911236058387483	-0.296070333333333	0.152824066102361
STUB1	-0.0630522997612248	0.867679444962133	0.143752384436988	0.792008469470523	0.0182533333333340	0.952448105846014
OR52D1	-0.063099986323641	0.867578417716872	0.230426732260333	0.618511766646781	-0.0862596666666663	0.760810252329096
BRD7	-0.0631343947855599	0.867428236930924	-0.397186793687481	0.270636326528939	0.0271033333333347	0.919456505549688
COL4A6	-0.0631953823162053	0.867153182468404	0.126807229377409	0.820923134671156	-0.112501333333333	0.712318389828268
LOC646214	-0.0632707778914542	0.866769040041089	0.134717240151198	0.807917669829974	0.223008333333334	0.287956132210494
TTC32	-0.0633082945656875	0.866599295770736	-0.0251695851198590	0.972358966414305	0.250107000000001	0.305655404008506
ATF6B	-0.0633529468029551	0.866583580376694	0.135544394502062	0.806704486837958	-0.0676994999999981	0.76307213318662
NPTXR	-0.0633596592274754	0.866583580376694	0.177948518545349	0.727982464250053	-0.0792219999999999	0.767958022875087
KCTD5	-0.0634194937723924	0.866430084546039	0.0391317035431792	0.953905313456582	-0.128217666666666	0.5293518047673
CHRNE	-0.0634709315973859	0.866172680503647	0.278585515119009	0.511025440770733	-0.0652853333333327	0.814273596067945
RPH3AL	-0.0634840050285007	0.86615672200694	0.474874803278259	0.144197096534399	-0.039071666666665	0.891410328517388
SLC9A2	-0.0634897499524396	0.86615672200694	0.0990366582797786	0.868042453226125	-0.027658333333333	0.93901998985171
SND1	-0.0635282355146138	0.866010988096045	-0.239450515977637	0.599103609129687	-0.129960333333332	0.530500926161619
FAM102A	-0.0635326532558774	0.866010988096045	0.319520568879975	0.42581212454843	0.102831000000001	0.667732731151074
TNFRSF1B	-0.0635715055170855	0.865871288652923	-0.307452485427123	0.452689746287922	-0.277102666666666	0.194072775206065
RALGAPB	-0.0636245020310194	0.865670358154757	-0.383344593718251	0.29374936963418	0.0511036666666667	0.847083064840772
RABGGTB	-0.0636601609091395	0.865597207467778	-0.0640275753846088	0.92003791975205	0.0125363333333348	0.96797990593595
ZNF470	-0.0636789406991174	0.865592975111095	0.0635156868410624	0.920855211745173	0.103491333333333	0.671582238113164
C9orf41	-0.0636914021891888	0.865580858136988	-0.173517036838958	0.735894671908835	0.342268000000000	0.0917903726055603
TGS1	-0.0636974465732179	0.865580858136988	-0.158245188571602	0.767214373310291	0.0462690000000008	0.843152489417868
TMEM185A	-0.0636990797649016	0.865580858136988	-0.46220068443332	0.159445854532777	-0.0562406666666653	0.815156860161333
PLEKHG5	-0.06369991037662	0.865580858136988	0.303590884081255	0.460797293728221	-0.00496733333333316	0.988133186272453
TIMD4	-0.0637099504490944	0.865580858136988	-0.176437859732215	0.730152680173573	-0.0231706666666664	0.92980287143849
STARD3	-0.0637182538841149	0.865580858136988	-0.309004285682180	0.449146665366917	0.0783559999999998	0.714382809933119
TDRD10	-0.0637622603112577	0.865580858136988	-0.0479406356978468	0.94314236584312	0.260235666666667	0.338184384427605
PPP1R2P9	-0.0638469981620764	0.865463747656326	0.134210261401678	0.808009477165608	0.00707866666666755	0.988133186272453
ARMC4	-0.0638655635363558	0.865413194731614	0.0839754548427049	0.892678844847032	-0.096817333333333	0.656941730375963
ACADM	-0.063874922052558	0.865413194731614	-0.150460868658516	0.77904503981756	-0.0587333333333323	0.799725367501923
ADAM11	-0.0639163015604409	0.86529275954933	0.120366843133297	0.834588275061672	0.0924840000000012	0.669391045681572
ICT1	-0.0639212891093842	0.86529275954933	0.341183933401866	0.378167769116079	0.0133426666666678	0.96469777383274
SLC30A6	-0.0640022808969396	0.86514950108376	-0.0101259425326007	0.988384322540392	0.0934816666666664	0.65948664958637
MAGEA3	-0.064022579714385	0.86508801255782	-0.0352885078377899	0.95869292666698	-0.120030333333332	0.548291557744437
CAMKV	-0.0640431908269784	0.865024553889298	0.0926520743191308	0.877878897534788	-0.239241000000000	0.361022088027705
SREBF1	-0.0640787273533454	0.864943849420114	0.279360409806745	0.510154465721482	-0.35517	0.12148388169181
ATP6V0B	-0.0641149442029612	0.864904499107038	-0.140919784252492	0.796714214272984	0.0165190000000007	0.958098397518313
RASGRP3	-0.0641404417732597	0.864810232966738	-0.3354223047239	0.390956441033312	0.103554666666667	0.65043308229974
CXCL3	-0.0641744692566802	0.864728634546941	-0.348158551805645	0.361374443308161	0.115968666666668	0.608679471465191
PRIM1	-0.064184028619979	0.864728634546941	-0.0983368130799745	0.86848759013952	0.145926666666667	0.459624813256835
CD207	-0.0642430230887724	0.864549068411763	-0.245279507208605	0.585512019050625	0.181302333333334	0.479031378601423
CIB3	-0.0642466611745363	0.864549068411763	0.368010570536419	0.325963209716859	-0.209965333333333	0.466098392080126
PDXK	-0.0642490380870023	0.864549068411763	-0.150426972481228	0.77915688016178	-0.372810333333332	0.0905524993267201
SLC35D1	-0.0643699695465198	0.864227116177435	0.141980051639502	0.794626181422627	0.0262126666666671	0.961659302951852
SESTD1	-0.0644415128606418	0.863908950640213	0.190170118175840	0.704208582824765	-0.0482129999999991	0.85013576481729
ATOH7	-0.0644994681503226	0.863908950640213	0.131173046503848	0.811872928177906	-0.0151689999999993	0.966149176465358
C19orf80	-0.0645095828526332	0.863908950640213	0.227610149712840	0.625174304269387	-0.126941666666666	0.575589336285762
TRAF7	-0.0646024606216265	0.863599033220385	0.139120954040679	0.79864602540281	-0.0847043333333333	0.710449166505851
SPINT3	-0.064645329276876	0.863487634104821	0.135908565931066	0.805895463708687	-0.0901646666666661	0.681765404528423
ZFPM2	-0.0646625328415192	0.863445599206029	-0.265776069248193	0.539878668275766	-0.0424143333333333	0.872930585008323
IL17F	-0.0647374941348164	0.863238670732167	0.185145044047215	0.71531201287312	0.0232970000000008	0.943391353769848
H1FOO	-0.0648169899077822	0.862975708121381	0.216721105731769	0.647357580911858	-0.0335433333333326	0.919347721375043
CSPG4P1Y	-0.0648475059591817	0.862877073886372	0.267291334186981	0.536380012714446	-0.116769000000000	0.606083038776023
OLIG3	-0.0649265523819035	0.862511358324903	0.241241499964111	0.596019873098193	-0.00655533333333303	0.987937064305561
C11orf21	-0.0649847878163243	0.862256307730716	0.142662128884528	0.794004622916083	-0.0295169999999999	0.907096813604642
RHAG	-0.0650021518082562	0.862256307730716	0.0497467456601958	0.941458038227918	0.0957590000000002	0.712818102694915
GPR83	-0.0650839180433747	0.86198439739943	-0.156843358338409	0.768401006769299	0.0846686666666674	0.815156860161333
GNPDA2	-0.0651237771730969	0.86198439739943	-0.29424833076192	0.479894403496244	0.171142000000000	0.436132693279062
MBOAT1	-0.0652289324042039	0.86153409197305	-0.203687464288328	0.676117692782917	0.0210693333333339	0.937545603686416
C3orf30	-0.0652365494692526	0.86153409197305	-0.0805210119797278	0.896949986708202	0.0553816666666669	0.834899630577731
DICER1	-0.0654360275360579	0.860426900425764	-0.00126046856730538	0.998348925592729	0.459783333333335	0.0863442910203928
RWDD2B	-0.0654712522391784	0.860390313640872	-0.124445424601299	0.825021694154683	0.0457116666666687	0.885894725360922

FAM110B	-0.0655022917353677	0.860340883462547	0.0365488655003512	0.957280839007133	0.0832883333333342	0.83648575595752
TMEM190	-0.0655290967585963	0.860238184286519	0.0920064060517884	0.879153312143409	-0.220061	0.409335260696998
ZNF529	-0.0655851067674888	0.860193284859981	-0.306462454219721	0.455641777483291	0.0340800000000007	0.898863720449539
CST5	-0.0656250917313225	0.860163841011155	-0.0375568146719786	0.955365651737247	0.0371156666666673	0.895093529187836
CALCB	-0.0656465763958918	0.860094691633248	0.0176183384705162	0.980393315184537	-0.194882999999999	0.343572153228906
HOXD4	-0.0656707007585821	0.860008880735683	0.508019399405668	0.103188745786156	-0.0788229999999999	0.804090240889797
SLAMF7	-0.0657390770263232	0.859909542247073	-0.266084268425798	0.53910027569054	-0.0755586666666662	0.73097308185803
SLITRK1	-0.0657671391486564	0.859798861316744	0.0406117969146692	0.952426531595115	0.11131133333334	0.63249696767281
NLRP12	-0.0657945843001556	0.859798861316744	-0.0307610582052354	0.96529904314994	-0.113806	0.660122297714278
ANKRD39	-0.0657993119619075	0.859798861316744	0.0700705831553626	0.910672238013038	-0.183342666666666	0.353131201082797
FAM110C	-0.0658314731051975	0.859791509866736	0.29100924681387	0.486880079690803	-0.0950646666666664	0.852020205196772
BATF2	-0.0658715299377536	0.859671550401468	-0.373234546531425	0.316397872891759	-0.0645013333333327	0.799660020384009
FGF6	-0.0658753491249653	0.859671550401468	0.0344590196927701	0.959404955370931	-0.0365103333333329	0.895093529187836
TRIM11	-0.065967532418816 7	0.85928612517923	0.0819364646529008	0.895406635164068	0.0054733333333349	0.987937064305561
RIC8B	-0.0660459240278817	0.859102165420192	-0.0210578479309386	0.97607156937294	-0.101517333333332	0.628876482131754
IRS4	-0.0660504100226492	0.859102165420192	0.360477755170246	0.340511948667662	-0.00214699999999975	0.99529569362957
SCN2B	-0.0660788896996634	0.859102165420192	0.130523134523779	0.812271254601992	-0.431815333333333	0.0895407923270921
SIPA1L2	-0.0661155642426359	0.859102165420192	-0.0728327585394782	0.907030540882842	0.452256666666668	0.0652061301014601
GCN1L1	-0.0661505756561212	0.859102165420192	-0.165408333623609	0.752352242644389	-0.178989666666666	0.399982212877977
CCDC67	-0.0662032535904003	0.859102165420192	0.131177324348804	0.811872928177906	0.0797696666666668	0.707538975864338
SLC9A5	-0.0662598083476795	0.85908144516222	0.346500743373356	0.365349151543041	-0.0134419999999998	0.97161673663638
MFN1	-0.0662614582197058	0.85908144516222	-0.239939446970139	0.598134271398487	-0.0152453333333319	0.959218131095776
SCUBE1	-0.0662621726740608	0.85908144516222	0.216198780650308	0.648391670659269	-0.0346703333333335	0.892573269731487
GABRA6	-0.066486227823172	0.858182820087211	0.184239959089019	0.717436396325728	0.068598666666667	0.861677398924522
GTF3C5	-0.0665223596227724	0.85802099256628	0.186603755745229	0.711306976302188	-0.0064453333333328	0.9835653863402
EZH2	-0.0665845413385008	0.857761053856996	-0.101911033689945	0.864616357134757	0.262620666666668	0.262756877023918
EIF4G1	-0.0665850304565831	0.857761053856996	-0.100857121577676	0.866005093242444	-0.346461999999998	0.105971509018004
MINOS1	-0.066622360699531	0.857663359450648	-0.400649971637049	0.262685489461853	-0.114100333333333	0.584434983272196
CSF2RA	-0.0666356139543459	0.857663359450648	-0.263015235281852	0.546969225987707	-0.133909333333333	0.501298037624658
GYPC	-0.0666872491434567	0.857475476004394	-0.295227166270756	0.478475354656325	-0.0934670000000003	0.668223322932086
SELENBP1	-0.0666935451826885	0.857475476004394	-0.1374847480735	0.802039543563725	-0.0608626666666658	0.86618358328362
ZP2	-0.0667228404264589	0.857475476004394	0.130338236705135	0.812271254601992	0.0498150000000002	0.848896273601345
C2orf83	-0.0667708696519744	0.857248648399729	0.208944766255094	0.665595909869988	0.0855456666666673	0.742124582656713
SNRPF	-0.0668018472721143	0.857248648399729	-0.175596795418054	0.73187914820204	-0.0823963333333329	0.710102032473187
TDRD7	-0.0668784473775535	0.856834714522648	-0.505516524109608	0.105941223458825	0.339472000000002	0.211495399841293
B3GALT6	-0.0669433542281004	0.856623973429035	-0.107070660571110	0.8576027597496	0.044605000000001	0.872217548758012
APOA1BP	-0.0669453998290241	0.856623973429035	-0.228707210744030	0.622401336557288	0.151640000000001	0.469591696339734
DARC	-0.0669611781835108	0.856623973429035	-0.288694393789302	0.489813374112312	0.0952386666666671	0.657353585270084
ZNF750	-0.0669971645259463	0.856616467595064	-0.170049593667690	0.742964131766277	0.123589000000001	0.630001429750697
RNF113A	-0.0670386637747441	0.856451467908748	-0.239096772551963	0.599801655225887	-0.130645999999999	0.512796006754107
ZNF81	-0.0670605402907504	0.856415368112598	-0.236915562873568	0.60400816456508	-0.0600219999999989	0.810944395524465
HDAC10	-0.067062403590663	0.856415368112598	0.159223919858558	0.764789523460873	-0.0568619999999995	0.819865694093637
RSPH1	-0.0670842667546678	0.856398443182581	-0.110883766804352	0.849357727620213	0.0760166666666672	0.74212040995637
UFD1L	-0.0671112678104191	0.856294241040603	-0.0990199054740936	0.868042453226125	-0.063236333333332	0.778083808586846
GMPR2	-0.067277610253056	0.855508475041307	-0.0586351863011011	0.928586335857223	0.0103283333333339	0.972329862794158
WSCD2	-0.0673267261221546	0.855365803160391	0.0790685450160391	0.89896954521199	-0.153065333333332	0.541284231249241
PGK2	-0.0674683209873228	0.854635147077845	0.170979993697417	0.740570923133528	0.0524953333333335	0.868340672756718
POM121L12	-0.0674799603996556	0.854628041651617	0.119206252875123	0.836519237151553	-0.235849666666667	0.313220411537404
ARMC6	-0.0675341679242177	0.854395787639396	0.0835244634350359	0.892833228561937	-0.189434333333331	0.343052387008537
SLC38A4	-0.0675422236265575	0.854395787639396	-0.0227516336509359	0.97536722461972	-0.451677666666665	0.294300395506211
SH2D5	-0.0676161861122484	0.854099134690376	-0.123309707245421	0.827531665631642	-0.130866666666666	0.520245449442202
PCSK1N	-0.0676768913859322	0.853781800458078	-0.0999749964508087	0.867290404865575	0.109014666666668	0.619881356855698
GLE1	-0.0677118963456064	0.853693402458083	-0.146741483184776	0.786187758946602	-0.493993333333332	0.0577241178957641
KCNJ6	-0.0677551014816831	0.853486703128641	-0.0380782400721731	0.95468180561467	0.0165913333333345	0.960259300600013
ROR2	-0.0677847069580778	0.853426370155038	0.175602961399362	0.73187914820204	-0.0417650000000002	0.918952691262331
PRRG2	-0.0678768046559443	0.852997444871091	0.163598915026934	0.755036658968701	0.00292600000000061	0.993805540619435
YTHDF2	-0.0679008572196163	0.85296385697998	-0.131999667833395	0.810300414153187	0.176386666666668	0.363634739722717
SERAC1	-0.0679531063821431	0.852766389428583	0.193846160063711	0.697189653813355	0.124338000000001	0.641044966033937
SCGB2B2	-0.0679834093550017	0.85270766570649	0.197777501343286	0.688373983767595	-0.0868333333333328	0.810625157938411
GPR88	-0.0680147758492815	0.852575779766944	-0.0968956855212716	0.870644879522268	-0.0794683333333333	0.787230398823895
PHF17	-0.0680576607671744	0.852478070101735	-0.169856050904041	0.743328907676018	0.0178870000000004	0.948780758881 42
TIMP1	-0.0680869896391135	0.852478070101735	-0.319298658938166	0.426142369255984	0.269993333333334	0.168877300696392
IRAK2	-0.0681093155076181	0.852478070101735	-0.237949029942696	0.602142642649819	-0.0664656666666663	0.77224206607108
FAM86C1	-0.0681210983623586	0.852478070101735	-0.27506922907162	0.51990180188597	0.0407556666666674	0.868791301779828
WDR60	-0.0681618324311928	0.852376770081218	-0.0422862288403043	0.95164730753796	-0.086291333333333	0.734228369215232
BIK	-0.0681662483702405	0.852376770081218	0.406149753634352	0.254041953733213	-0.174501666666666	0.384743478073559
TLR3	-0.0682347203035592	0.852107426537877	0.119629835087523	0.835740437824474	-0.101308333333332	0.753937973093065
SCYL3	-0.0682360731919837	0.852107426537877	-0.0836666518245144	0.892833228561937	0.166196666666667	0.471680039257208
MTA3	-0.0682375736020532	0.852107426537877	0.265703957297103	0.539878668275766	0.126011666666668	0.533475280376028
CNTNAP2	-0.0682541830748733	0.852107426537877	0.0572350860737484	0.93071299016553	0.101632333333334	0.779485206901945
HOXA6	-0.0682837087394657	0.85207114525411	-0.0727186359186971	0.907431863377741	0.127331333333334	0.537186705207113
CTLA4	-0.0682904034190085	0.85207114525411	-0.154208837081278	0.772012314922122	-0.113428666666667	0.573673650964407
WDR27	-0.0683114435134233	0.85207114525411	0.500837724807354	0.113169357904212	0.0369190000000008	0.896403646226360
G6PC	-0.0683511173137099	0.851917228713171	-0.222983878311155	0.635247978870818	-0.177677333333333	0.353471671280201
SLC39A2	-0.0683566575858596	0.851917228713171	0.234885365707262	0.607957283325678	0.138182666666667	0.588666179294878
MMP28	-0.068387340131513	0.85188091446614	0.259352976965456	0.557254575867468	0.0143526666666671	0.963103527374278
RRN3P1	-0.068447437711026	0.851779482874226	0.0358250748911465	0.958354725588542	-0.112638333333333	0.575378149225683
FAM134C	-0.0684804411953237	0.851779482874226	0.0223032176050191	0.97536722461972	-0.143841999999999	0.575378149225683
CENPK	-0.0684967222871825	0.851779482874226	-0.00122933319729711	0.998348925592729	0.256724	0.175476022490732
PHYHIP	-0.0685100890963098	0.851779482874226	-0.0788977569938048	0.899154242391172	-0.0763293333333322	0.745324730039331

ARR3	-0.0685683302727526	0.851779482874226	-0.0978466368752416	0.868852648210739	0.0401623333333346	0.874353540431336
IPO13	-0.0686560476649309	0.851311061977258	0.0715139871677124	0.908871419280947	-0.0729396666666663	0.778995157976597
IL5	-0.0686627781071785	0.851311061977258	0.250728879535245	0.573353777103379	-0.140496333333333	0.498456714564834
SLC25A21	-0.0686700592672033	0.851311061977258	-0.239266042174875	0.599781323663208	0.0204020000000001	0.951767328727707
SENP2	-0.0686791114150674	0.851311061977258	-0.0668411973175123	0.915238138104257	0.145191333333334	0.503400741258744
MTMR10	-0.0686896282570628	0.851311061977258	-0.171999637164974	0.738911460376148	0.0874643333333332	0.682978179404224
GZMK	-0.0687005368688401	0.851311061977258	-0.208073421405812	0.66739374020401	-0.0187726666666663	0.94871117680979
GRK7	-0.0687489269798193	0.851311061977258	0.348614482764315	0.361261311685225	0.0504113333333343	0.8445457518207
C4orf3	-0.068776561348199	0.851311061977258	0.0363792081331356	0.957597361653107	0.0825410000000008	0.707538975864338
GYPA	-0.0687810476525075	0.851311061977258	0.068205521185661	0.912364243071055	-0.138636333333333	0.583036318848746
DYDC1	-0.0688433900990408	0.851291639112072	-0.142474857811749	0.794346532964905	0.0208340000000003	0.940972575250573
GPC4	-0.0688760344476073	0.851219585640576	-0.106680352485318	0.857937967416038	-0.819615	0.188549331106419
DPH5	-0.0689131120635927	0.851147938414717	-0.111406789348631	0.848968422336654	0.00663250000000061	0.983159241812861
C1orf173	-0.0689202103737182	0.851147938414717	-0.123895184839839	0.826287181959559	-0.105491666666666	0.668223322932086
MATN1-AS1	-0.0689378286423199	0.851124605099981	-0.179327313756380	0.725260813023794	-0.190787666666666	0.384248044508942
TMEM44	-0.0689592625750539	0.851055417336397	-0.0198314562685377	0.977526645675126	0.0561383333333335	0.827433678445379
RPS7	-0.0689628343799213	0.851055417336397	0.287086379499478	0.494441582955552	0.0838543333333334	0.710449166505851
IGF1R	-0.0689994311208885	0.850945686813869	-0.231589647492151	0.61468512938802	-0.0517601666666662	0.870002591217953
CFHR5	-0.0690059466415914	0.850945686813869	0.0905707543001534	0.88168896154906	0.0095436666666672	0.974895360062947
ST13	-0.0690079645967193	0.850945686813869	-0.0354602319126827	0.958689318480951	0.135411000000000	0.497056976730679
DNAJB11	-0.0690338457413484	0.850945686813869	-0.184980181953244	0.715658781310868	-0.0306583333333323	0.911623909907545
EBPL	-0.0690574281122812	0.850899868605456	0.170849914918661	0.740902769015583	-0.146973	0.562674320626673
C1orf194	-0.069101900987437	0.85078520099093	0.00632569763440337	0.991853352023739	-0.159621333333333	0.417715878267123
ITGAL	-0.0691592309644968	0.850521190776634	-0.382628118730109	0.294988445038215	-0.0780203333333328	0.75973877196122
HK3	-0.0691679195471223	0.850521190776634	-0.205013412545406	0.673623956972285	0.0188606666666673	0.952681994500534
TGDS	-0.069171824099435	0.850521190776634	0.105714466751811	0.85956218633532	0.0590156666666677	0.861165447055876
KIAA1875	-0.0691743637551685	0.850521190776634	0.0495945566056341	0.941552283017912	-0.0193273333333334	0.954897695204154
C15orf41	-0.0691878855194886	0.850521190776634	-0.201227555121689	0.680054545720343	0.291089999999999	0.158095716859654
SLMO2	-0.0693542031874164	0.85001848320928	-0.00930378446845731	0.98909166696601	0.232821666666668	0.229351174169897
KRT3	-0.0693590126562909	0.85001848320928	0.246756388962884	0.581387364514747	-0.121071000000000	0.541939010010167
HMGB3P1	-0.0694295338530066	0.849874242051031	-0.313840012450249	0.438046311563112	0.0358713333333337	0.895093529187836
FLJ33360	-0.0694421020815891	0.84986123688818	-0.0933043947056475	0.87665934259866	0.00248866666666673	0.99411609755275
HSP90AB1	-0.069484660495465	0.849658011123852	-0.104665719662967	0.86067831050617	-0.0742766666666654	0.751422775543186
MYOT	-0.0695259627107393	0.849462755013429	0.00601231217280329	0.992486213203714	0.333028000000001	0.313220411537404
IGF2-AS	-0.069558574797186	0.849378408080382	0.378175807256418	0.305379763637051	-0.133655999999999	0.600304644185307
GIPC1	-0.0695732335897594	0.849363028106944	0.163031119429266	0.756777795131744	0.0259783333333338	0.9299162364306
C1orf131	-0.0695736051128474	0.849363028106944	-0.0406572208878136	0.952426531595115	0.0863393333333335	0.704370299831112
KIAA0101	-0.0695772462117758	0.849363028106944	0.098139978717253	0.868852648210739	0.151079000000001	0.462361390840849
GPR17	-0.0695869853187871	0.849363028106944	0.0305302159511550	0.965676710289184	-0.0876056666666663	0.707538975864338
CD8B	-0.0697016158119018	0.849038796330032	0.0391766122958092	0.953905313456683	-0.165350166666667	0.541939010010167
IL2	-0.0697138330605868	0.849038796330032	-0.112939238784641	0.846627656290767	-0.0455206666666664	0.848896273601345
ABCB1	-0.0697450975749164	0.849038796330032	0.598837720145031	0.0336307287478746	-0.0623333333333313	0.77769170716506
CSF2	-0.0698045313584157	0.848762952906499	0.170914584143244	0.740768646795758	0.124774666666667	0.58043274631496
FAM131B	-0.069905610281357	0.848388485545887	-0.0459637104097521	0.945364757579857	-0.0772369999999997	0.802934878255011
TMEM150B	-0.0699289465827725	0.848307130029845	0.192331029803866	0.700450711372106	0.0218513333333345	0.933934713252084
FBXO8	-0.069966244428232	0.848137187520615	0.0543779434958815	0.9344692810234	0.211998333333334	0.420976808369158
KIAA1407	-0.0699703083932782	0.848137187520615	-0.0828493767246743	0.89362638370288	0.338241666666667	0.0888564451939157
RASD1	-0.0699728487736312	0.848137187520615	-0.229367341936174	0.620183562399927	-0.230315999999999	0.608311546956177
ELF2	-0.0700612325302364	0.847986744002199	-0.182163019780547	0.71964467602874	0.128892333333335	0.558494409261702
FLJ20464	-0.0701305380122637	0.847628350993701	0.174913306710548	0.733518674801325	-0.138851000000000	0.655951370096932
ZNF440	-0.0701306301950775	0.847628350993701	-0.163949716348623	0.75491314198126	0.145140666666668	0.640545993948933
OVOL1	-0.0701579415554683	0.847628350993701	-0.0930230278673465	0.877331332619452	-0.121011000000000	0.610151958903093
CPNE7	-0.070220946577347	0.847521612968328	0.0815558611526897	0.895963909776673	-0.128336666666666	0.670113640649168
ISG20	-0.0702315303880445	0.847521168699173	0.0611376301514147	0.924684666171101	-0.0183726666666657	0.958098397518313
ADAM33	-0.070278666242123	0.847422181965673	0.0743838429008808	0.904945422294164	0.0160766666666669	0.966149176465358
KCNK17	-0.0702808642413483	0.847422181965673	0.180255469625534	0.723209685016312	-0.124487000000000	0.616036866881905
PPIL2	-0.070281024783767	0.847422181965673	0.150099148714950	0.779908498326709	-0.0163656666666655	0.959910474847156
ALPI	-0.0703094406732165	0.847422181965673	0.269675348220378	0.531924063959685	-0.0631139999999996	0.830987625642722
TCTN1	-0.0703227266087936	0.847422181965673	0.0597796606817647	0.926352922534113	0.483946000000001	0.0262046791709735
LINC00574	-0.0703452360633284	0.847422181965673	0.0156638185001888	0.982266426585123	-0.0413149999999999	0.902718738113061
BRCC3	-0.0703536884090029	0.847422181965673	-0.364782190643787	0.332047752124584	0.170159666666669	0.49971129098408
EMC9	-0.0703945077212647	0.847287549732229	0.0733747355305674	0.90588212168674	0.131921000000001	0.648325366843053
GNG11	-0.0704464228061631	0.847091424993108	-0.0972286963359617	0.870020870354202	0.136090333333334	0.680455232178275
MRPL55	-0.0705484291929927	0.846716316371747	-0.0779523657282551	0.899780667006098	-0.0614659999999994	0.778083808586846
GTF2H4	-0.0705539996693832	0.846716316371747	-0.152083732913796	0.775926738317144	0.0313616666666674	0.905659119449126
GPATCH1	-0.0705599704644282	0.846716316371747	0.0412481506203771	0.952098348256047	0.139779000000001	0.502480362041859
PRPH	-0.0706075144381069	0.846716316371747	0.112596572571567	0.847191484945575	-0.273929666666666	0.231998578744259
USP26	-0.0706108094469864	0.846716316371747	0.123103831096555	0.827789978865629	0.124848666666667	0.671766969198954
C4orf17	-0.0707446139640873	0.846466482377905	0.00307360021899699	0.994986311144058	0.00757000000000061	0.98256318768706
C14orf39	-0.0707578365668066	0.846449292693517	-0.0441635856340494	0.948493391127553	0.163644666666667	0.50181000987305
FHIT	-0.0707599163781449	0.846449292693517	-0.21368478892455	0.653708954135733	0.146389	0.475944400862393
PAGE1	-0.0708240222226182	0.84616238042143	0.132786708282799	0.809588765729788	-0.0958649999999997	0.681982823818184
SDS	-0.0708478212606928	0.846077976557559	-0.180756898278112	0.722159001254958	0.0504853333333337	0.896406735156757
DAPP1	-0.0708789039957237	0.845995243433244	-0.386243887292530	0.289351981332176	0.140049	0.492100665301584
GJA10	-0.0709332975519558	0.845802102942599	0.191445468728366	0.701635983922402	-0.0291276666666662	0.924674647382585
SFRP5	-0.0709421816111338	0.845802102942599	0.253202209078019	0.56815451706573	-0.00147433333333364	0.996290446076447
LAT2	-0.0709557233719636	0.845802102942599	-0.422520139085108	0.221428501305239	-0.117546333333333	0.587396808178803
DEFA5	-0.0709991280144402	0.845717883263012	0.250857740073314	0.572924277154653	-0.138095666666666	0.671832782024645
FOXQ1	-0.0710044882832239	0.845717883263012	-0.522654636786321	0.0873718619016661	-0.0517606666666658	0.860388779364817
TBX21	-0.0710758037098482	0.845497890563929	-0.055868790427112	0.933151248545599	-0.0944379999999995	0.773702861764059

NXF3	-0.0710890141674617	0.845480763755973	-0.0567315018092756	0.931538794783139	0.000633333333333376	0.997953056544486
ATP5SL	-0.0711187596247914	0.845480763755973	0.118060804560261	0.838500126709059	0.0822470000000005	0.734997389745078
CNTLN	-0.0711520462773309	0.845414342045116	0.0768535414950385	0.901856698918763	0.159152000000001	0.485883178494067
PLAC1	-0.071225198550465	0.845216711826659	-0.158750405278565	0.765791046741822	0.344462333333334	0.0905524993267201
KRTAP19-1	-0.0712694564241185	0.845135921364112	-0.223075360372081	0.635247978870818	0.0286080000000012	0.915480183438261
GTPBP6	-0.0712899930988666	0.84507220897457	-0.283947163192074	0.500873210019812	-0.316196	0.242993293923012
KIAA1257	-0.071296762996201	0.84507220897457	0.0860764276975358	0.888306347447303	0.139809666666667	0.523453039510001
GRN	-0.0713393658475936	0.844892000228838	-0.523560657304035	0.0865342407426229	0.0896700000000003	0.670113640649168
LCAT	-0.0713948643964359	0.844679351023016	0.083008679794198	0.893446201797764	-0.0500403333333328	0.852020205196772
GALNT13	-0.0714318599671865	0.844638190649587	-0.348414464210857	0.361311804770494	0.081764000000001	0.702709435280393
ZNF880	-0.0714494923015267	0.844615117680176	-0.320196715033784	0.423227776324677	0.0654016666666672	0.780717071872908
TRMT1L	-0.0714658281289133	0.844615117680176	-0.0463953737572603	0.944856324173183	0.165295333333334	0.417596927032144
CYP27C1	-0.0715674263718394	0.84431105919992	-0.238190228434653	0.60184463266698	-0.117808333333333	0.683326025818219
TSPO2	-0.0716057592830252	0.844201014173141	0.134790269222728	0.807867938002244	-0.168066666666666	0.478484283517744
ZNF404	-0.0718042923434947	0.843405837923246	0.0697432664187379	0.911167570018484	-0.133302999999999	0.696217349388802
PATE1	-0.0718049661444629	0.843405837923246	0.36653325625555	0.32846893492891	0.0785610000000003	0.713714407278061
PARP15	-0.0718194531225954	0.843405837923246	-0.164296307761692	0.754189116283047	0.0301256666666672	0.904788907158467
RFNG	-0.0718354242251509	0.843405837923246	0.230398914967812	0.618570954716098	0.102111333333334	0.629657237462109
PRMT7	-0.0718601661431433	0.843405837923246	0.130349530559321	0.812271254601992	0.00617900000000062	0.987908236336346
PPP2R5B	-0.0718812254055252	0.843405837923246	0.125970518260881	0.822378909366501	-0.00558366666666659	0.9879739922351
SLC26A3	-0.0719726860244281	0.843366440646804	0.210419549003316	0.661670522548494	-0.0880829999999995	0.710872198247224
CA5A	-0.0720284742482036	0.843184248522535	0.195201644405879	0.694852194384891	0.0958806666666671	0.772139923817113
KCNN1	-0.0720506856668245	0.843184248522535	0.0310231298158918	0.965118873711308	-0.157341666666667	0.481122049034717
C2orf44	-0.0720506964152335	0.843184248522535	-0.104466203358109	0.861019880295917	-0.0453126666666658	0.861677398924522
BMP15	-0.072057494376688	0.843184248522535	-0.173606356029569	0.735819417685315	-0.0648179999999994	0.811997756288597
PHF1	-0.0721101580259318	0.84313299737878	0.0576480481429062	0.929758936765496	0.457007000000001	0.0364080466199869
EFCAB6	-0.0721162446696198	0.84313299737878	0.0966308473035866	0.870857630743202	0.0623433333333338	0.808592092362553
TEX9	-0.0721250982281817	0.84313299737878	-0.258285620887444	0.560815056132889	0.117838666666668	0.59851425430501
SIDT2	-0.0721944038101412	0.8429905660351	-0.132470628538750	0.809866082339655	-0.205199000000000	0.337307065334106
TMEM69	-0.0722075273315387	0.8429905660351	-0.107172432973648	0.857440276187559	-0.109009333333333	0.610847413222267
SORCS3	-0.0722545390927843	0.842883521410073	-0.356343279997803	0.345997909309632	0.105407000000000	0.616036866881905
GFI1B	-0.0722790282710994	0.842883521410073	-0.124133905717662	0.825526959189789	-0.102219666666666	0.637596150841031
SLC35C2	-0.0722791604999564	0.842883521410073	-0.0564828986425742	0.93176886339657	0.146925000000001	0.458006502146897
MAT2B	-0.0722926974460706	0.842883521410073	-0.205818641128745	0.672625944768829	0.151701333333333	0.445566557024313
DDX41	-0.0723236055839463	0.842868289116268	-0.0204740070173838	0.976412247012224	-0.278030333333333	0.152146083127957
LPXN	-0.0723303661871905	0.842868289116268	-0.297868830553353	0.472550555499985	0.312225	0.108563852880459
LHFPL3	-0.0723503664572354	0.842868289116268	-0.0606955394347297	0.925335786425382	0.00897366666666706	0.985884500368235
ZNF770	-0.072382304075391	0.84280506661071	-0.0403289970641373	0.952720585102517	-0.178994500000000	0.498456714564834
LGI2	-0.0723892086366137	0.84280506661071	-0.122659825990860	0.828930912293926	-0.0148153333333327	0.964502647998226
COG2	-0.0723902926417922	0.84280506661071	0.0398926985746958	0.953525981777884	-0.0708939999999993	0.760010763598572
CBLN3	-0.072477940995756	0.842583418781564	0.104999795142720	0.8606536389692	0.100562666666666	0.714382809933119
PTK6	-0.0724804950282796	0.842583418781564	0.252016952013302	0.570584539772622	-0.113183000000000	0.698368688760464
NPHS1	-0.0725023199908685	0.842583418781564	0.0871741510920165	0.887055939766881	0.0244976666666671	0.942298301774487
CCDC51	-0.0725157192425522	0.842583418781564	-0.138567662880891	0.799373390835754	-0.128575333333332	0.546294202178525
C5orf22	-0.0725420357469461	0.842583418781564	-0.472163194803718	0.146264731300646	0.138505333333335	0.484583000340636
FILIP1L	-0.0725487991339974	0.842583418781564	-0.496278861293289	0.115892102998482	0.0507233333333339	0.832554061619867
ZNF629	-0.072581580198834	0.842543253235932	0.126367982034920	0.821845554157948	-0.294483666666666	0.162247666710333
SOCS6	-0.0726042559910987	0.842480165045862	-0.553701479372732	0.0587020070494168	0.073222000000001	0.745245240629921
POLE2	-0.0726127003390795	0.842480165045862	0.0354260672576584	0.95869292666698	0.200786000000001	0.41666677641248
DPY19L4	-0.0726156016456714	0.842480165045862	0.0449407344448872	0.947094297257154	0.422761666666667	0.0508895417296966
PTPRZ1	-0.0726326029345578	0.842480165045862	-0.0213310246549431	0.97554687597484	-0.166299999999999	0.636490807097692
FUT7	-0.0726806942986062	0.842480165045862	0.0713226139085927	0.909119908757507	-0.0403646666666661	0.909453794898923
YARS	-0.0726876113390945	0.842480165045862	0.0920734647855374	0.879153312143409	-0.216218666666666	0.40744229803525
ACTRT2	-0.0726899810865166	0.842480165045862	0.24094691343426	0.596648444172109	-0.0798020000000003	0.849384482495249
LRPAP1	-0.072728231455148	0.842480165045862	-0.0598531277855855	0.926162673668662	-0.335499999999999	0.114193085140962
EPHB4	-0.0727633718277539	0.842480165045862	0.159213424999801	0.764789523460873	0.222301000000001	0.353471671280201
ZSWIM6	-0.0728113087730393	0.842480165045862	-0.360977103627442	0.339909950400083	0.0397690000000011	0.896446774489185
CXorf61	-0.0728420143227856	0.842480165045862	-0.131804705476913	0.810671652076995	-0.0794529999999992	0.729912257977231
TUBA1C	-0.0728654167720403	0.842463467681668	-0.254070815571042	0.567694846153007	0.109420000000001	0.600304644185307
IL2RA	-0.0729507879432167	0.842189464215531	-0.388651464838433	0.284843835728873	-0.0888099999999998	0.712601873916151
ART3	-0.0730213587816217	0.841949690018274	-0.139336042766119	0.798615265561297	-0.0216779999999994	0.946623914607177
DMP1	-0.0730337581123868	0.8419429022166	-0.156192151835937	0.768909201237015	-0.110742333333333	0.58391025729515
SPRY1	-0.0730487807607944	0.8419429022166	-0.155626989280649	0.769837060156515	-0.00312199999999988	0.993677488355417
USP40	-0.073070716524172	0.8419429022166	0.128386178678934	0.816345918941366	0.287803333333333	0.131549958663763
GABRB3	-0.0730811324466112	0.8419429022166	-0.109171754774781	0.85260191154502	0.151183333333335	0.527287587549928
PAAF1	-0.0731141781848594	0.841900360011964	0.0854906647544354	0.889604286579558	-0.108072333333333	0.616961986237355
NDN	-0.0732927398815531	0.841163714902093	0.281974884724254	0.504775776425053	0.0370810000000006	0.885866310496875
MCRS1	-0.0732963360989824	0.841163714902093	-0.178580476384223	0.726444926461881	-0.0554616666666663	0.809116195668422
AQP4	-0.0733204156998777	0.841163714902093	0.0607494435836925	0.925324864292945	-0.111638666666666	0.80355218052314
VSIG10L	-0.0734397349526609	0.840742040916408	-0.390116785182122	0.282383744168943	0.117702333333334	0.648442399981194
PLCB4	-0.0734468908590691	0.840742040916408	0.0431089454728926	0.950146638225747	0.0017553333333333	0.995621063297892
PSMD8	-0.0735366975596796	0.840345671949946	-0.0587142393492581	0.928472484757714	0.046306666666668	0.855533429360007
CEP63	-0.0735680510953068	0.840212689577223	-0.150572408172252	0.779021144254203	-0.0551886666666655	0.847929842355322
SNF8	-0.07357595048923	0.840212689577223	-0.125908801905612	0.822438385407788	-0.013517999999999	0.963180491237413
IBA57	-0.0736017399589355	0.840212689577223	-0.125504721825511	0.823755601962118	-0.134840333333332	0.506771274411823
UPF3A	-0.0736537752529567	0.840136077145167	-0.0265434753115713	0.970207612777188	0.0632533333333337	0.850157189407213
OBP2A	-0.0737058425885654	0.839978097006361	0.245457919661228	0.584678706753837	-0.00029999999999988	0.99883107916854
MYO1G	-0.0737194796000389	0.839978097006361	-0.140668893194592	0.796714214272984	-0.0095359999999997	0.979790222743567
LINC00173	-0.0737502849435285	0.83996061166547	-0.101953185185721	0.864616357134757	-0.00813233333333326	0.988133186272453
CHP1	-0.0737779548944716	0.83996061166547	-0.162236043858173	0.758250496829902	-0.367233333333331	0.0951773359513526

NCOA6	-0.0738183205126512	0.839892464226428	-0.180668795884672	0.722326759740446	0.160844333333334	0.523453039510001
RIPPLY2	-0.0738363027951835	0.839844873590553	-0.184526538634894	0.716590537152508	-0.0726206666666661	0.770050233924502
ZNF273	-0.073853771269743	0.839800566036415	-0.0583713396132063	0.928725686060612	0.212158000000001	0.447070880722204
KRTAP4-5	-0.0738539592097591	0.839800566036415	0.1545740385118	0.771310656070265	0.126032000000000	0.575378149225683
GLDC	-0.0738558847344475	0.839800566036415	-0.33156520632048	0.400099845811371	0.0111643333333335	0.97161673663638
EML2	-0.0738778895914105	0.839800566036415	-0.134258040681574	0.808009477165608	-0.27252	0.228256748148085
RYBP	-0.0739215487317542	0.83963672823977	-0.00915219574186837	0.98909166696601	0.240784666666668	0.349094758813838
APC	-0.0739515442624964	0.839512338443197	-0.201273446173802	0.680054545720343	0.239298000000001	0.226148687544232
RPL9	-0.0739599506864777	0.839512338443197	0.246220341376741	0.582912574644681	0.0250733333333349	0.930582603370544
VPS37B	-0.0740057241138301	0.839300700269985	0.0390414191004064	0.954042565039198	-0.00482933333333283	0.988133186272453
LATS2	-0.0740166863497022	0.839297971775227	-0.298928250840127	0.469981232748083	0.113327000000001	0.590147690656472
RNF220	-0.0740289573590491	0.839297971775227	-0.180510893487457	0.722641197261641	0.144515333333334	0.460428849906031
CPLX2	-0.0740806572283853	0.839091075907445	-0.134459553257967	0.808009477165608	-0.117101333333333	0.603043023754784
RABAC1	-0.0741350726006813	0.838810569283075	-0.119226719434893	0.836480397144275	0.132022000000002	0.639552116649016
BAIAP2	-0.0741833478305117	0.838636646068851	0.159729479443975	0.763150272733332	-0.075473333333332	0.787248874966057
TMEM176B	-0.0742302619852146	0.83853874594259	-0.442885067731122	0.184912742974902	0.107874333333334	0.617477739759122
SPG7	-0.074313207281458	0.838210498617081	-0.0474822796837318	0.94394191186189	-0.193008999999999	0.318078745001365
RAB21	-0.0744661093015277	0.837569674239696	0.084724981669111	0.891309254245872	-0.0162176666666657	0.954736472265512
HAPLN2	-0.0744787055958099	0.837556460984957	0.15887925093643	0.765560758742476	0.0535690000000004	0.838677358393546
MFGE8	-0.0744854761656736	0.837556460984957	0.250464808443096	0.573763059271745	-0.196304666666666	0.342825265121927
VSIG1	-0.0744989885511743	0.837556460984957	0.0140225605746972	0.983461004910902	0.00428900000000068	0.990009919740954
MAGEL2	-0.0745283233180723	0.837556460984957	0.0637803043863445	0.920521809324956	-0.0531406666666662	0.842201018871951
PTGDR2	-0.0746700799613461	0.837006142607322	0.347638903953777	0.362566587472102	0.0541133333333345	0.838409588013206
MRGPRF	-0.0746881278292638	0.836958069885962	0.0874460233482617	0.886459652119148	0.0974160000000004	0.719839702715467
CSRP1	-0.0749439062825414	0.835726713585639	-0.313274715851478	0.438174716561408	0.0784553333333346	0.789228734235839
ORMDL1	-0.074957914660532	0.835704431738221	0.218707126492553	0.642137448592891	0.0895876666666683	0.701993852257083
NTF3	-0.0749590803191345	0.835704431738221	0.0292958546587734	0.967655960277738	0.0913333333333338	0.666477212988496
KCNV2	-0.0749641489563349	0.835704431738221	0.0194403745378946	0.977825576987171	-0.117773333333333	0.57883453172984
HLA-DPA1	-0.0750188721999738	0.8355165714799	-0.444585624501696	0.183005576574781	-0.0531373333333326	0.862509125961694
OTX1	-0.0750382285655839	0.835460102653893	0.170011045811542	0.742973514512442	-0.101525333333332	0.617867263424706
ARHGAP27	-0.0750863609844521	0.835219701184006	0.0135142452984724	0.98412883278237	0.0115860000000003	0.974451420920923
KBTBD7	-0.0750916067393776	0.835219701184006	0.0833615317078156	0.892833228561937	0.141639666666668	0.68993253167322
TMEM80	-0.0750937390659105	0.835219701184006	0.0169730168055780	0.98105020869911	-0.174602000000000	0.37613621138023
FAM215A	-0.0751240025303596	0.835219701184006	0.285163886938630	0.49838641873573	-0.240392000000000	0.476515795581148
MAPRE1	-0.075129077198352	0.835219701184006	-0.247717869847525	0.579471329472972	-0.131786666666665	0.533058247369904
TSSK6	-0.0751408043163033	0.835219701184006	0.191578445360847	0.701635983922402	-0.0205339999999988	0.962414879079903
SIT1	-0.0751857771039923	0.835219701184006	0.110061546155722	0.850684853430578	-0.0969079999999997	0.787356957242818
STAG3L1	-0.0752260388415589	0.835219701184006	-0.00907313706048279	0.98909166696601	-0.00912133333333298	0.978192116090894
KCNJ9	-0.0752264380837259	0.835219701184006	0.109408840126450	0.851975798726468	-0.0296413333333332	0.926828987420178
ZNF48	-0.0752601520496469	0.835219701184006	-0.0467738811251374	0.944274732245152	-0.0663493333333327	0.830147563067484
CDKL2	-0.0752761837720447	0.835219701184006	-0.0124664071819756	0.985220352735461	0.215140000000001	0.397798624983652
MAP2K2	-0.0753038681156223	0.835219701184006	0.292546704931569	0.483448456260604	-0.241677999999999	0.203654244466101
TSPAN3	-0.0753054928400104	0.835219701184006	-0.155897394926737	0.76972820876143	0.0481933333333356	0.83682449377028
KRT82	-0.075311888537312	0.835219701184006	0.0705831887161018	0.91009246060164	0.119397333333334	0.58391025729515
PGGT1B	-0.0753674669693488	0.835219701184006	-0.179144538048142	0.725639227819855	-0.284226333333333	0.181167706041881
LOXL1	-0.0753708466219047	0.835219701184006	-0.187575832379644	0.709135075556051	-0.0224203333333334	0.941493821305792
LTF	-0.0754572344643466	0.835069289361336	-0.103900545096482	0.861570109612412	-0.232383333333333	0.398883894731131
IL2RB	-0.0754708481344922	0.83504956065867	-0.289013808934306	0.489280593381909	-0.256235333333333	0.312600912762022
ZNF610	-0.0754927494886266	0.83503158972655	-0.273922177063161	0.522049608558573	0.388619666666666	0.112438115304648
MAPK4	-0.0755163801936747	0.835027775963887	-0.0794762935446325	0.89819077139711	-0.144947333333333	0.512041798508902
ZNF341	-0.0755312089991016	0.835000273013659	-0.262805486476875	0.54748847064794	-0.148842666666666	0.570775035559897
PRMT6	-0.0755409371676754	0.835000273013659	-0.193341928491808	0.698337791293402	-0.00010999999999997	0.999729972650062
SERPINI2	-0.0755594416307672	0.834954391437476	-0.103934028036685	0.861570109612412	-0.0186546666666668	0.945904449997854
C22orf26	-0.0755642680191676	0.834954391437476	-0.0260193777779628	0.970840789910547	0.095274000000001	0.643620644819614
E2F7	-0.0756018394408823	0.834954391437476	-0.0113464924710776	0.987250302234215	0.0493026666666668	0.852969361639689
ELOVL7	-0.0756021818820625	0.834954391437476	-0.276326739577108	0.517374509850433	-0.202808333333332	0.552298643114782
MARCKS	-0.0756494243784332	0.834954391437476	-0.291126061215325	0.486880079690803	0.451748666666667	0.0560515070562022
SLCO2A1	-0.0756580593571322	0.834954391437476	-0.183608271331542	0.718067440349544	-0.124587666666666	0.654323362279664
CASKIN1	-0.075661955254554	0.834954391437476	0.225122126928327	0.63103711743427	-0.115482999999999	0.709937184587543
ZNF616	-0.0756670812267633	0.834954391437476	-0.0548621474020728	0.934255557320745	0.192534666666667	0.501298037624658
SLC5A5	-0.0756819806185795	0.834954391437476	-0.0616396778197199	0.923246185283376	0.0312166666666671	0.91318558400885
RPA4	-0.0756852483070983	0.834954391437476	0.0619708220372611	0.923034515346352	-0.0743839999999997	0.774210549173745
USP16	-0.075702330855629	0.834954391437476	-0.267661106746133	0.535603758171522	0.148974666666668	0.454686281387452
DOK2	-0.0757786252837236	0.834954391437476	-0.129785873740902	0.813305249846504	-0.125135999999999	0.629657237462109
ISL2	-0.075785174448435	0.834954391437476	-0.112082335159733	0.847483801046528	0.123625666666667	0.657914679607938
ZDHHC1	-0.0757956356333642	0.834954391437476	0.368736654585268	0.32362669860138	0.210012666666667	0.294300395506211
POLE	-0.0759769423402692	0.834237228905603	0.290247721711063	0.488568550695131	0.0230270000000009	0.942298301774487
XIRP2	-0.0759831346633777	0.834237228905603	0.0299305042471974	0.96664589910922	0.0158783333333336	0.965661221820942
KCND1	-0.0760019574393907	0.834237228905603	-0.330367470927642	0.402284079938133	0.0343246666666668	0.895093529187836
PDE12	-0.076038980810511	0.834177356636318	-0.226443249096035	0.627018556270882	-0.174464666666666	0.372958466530121
GCGR	-0.0760698830627855	0.834046828141361	0.0270465915679553	0.970207612777188	-0.0472686666666663	0.86715815306237
SOS2	-0.0761433053288755	0.833846412572166	-0.143208073120749	0.79294618815764	0.384419000000001	0.0562663106369921
FAM153A	-0.0761577467996386	0.833821399154697	0.117542331340453	0.839619129865365	0.217432500000001	0.28322823752779
YTHDF1	-0.0761891857030836	0.833687415434091	-0.0382566887167989	0.954545200586067	-0.0166359999999993	0.958763419236472
PMEPA1	-0.0762471893307555	0.833614093365595	-0.165750723078004	0.751952150889158	-0.148523333333333	0.520396791179343
CCL13	-0.0762567427171387	0.833614093365595	-0.255428309137947	0.566022340784541	-0.0183933333333326	0.947860086718622
PASK	-0.0762910159570726	0.833507563453109	-0.103135288769322	0.862401456058756	-0.136374333333332	0.493497627091521
SYCE1	-0.0763337601062477	0.833436243595464	-0.109714313130584	0.851195083614214	0.0985696666666668	0.689411219697146
MGC4294	-0.0764831915765102	0.833018707242402	0.21460170751579	0.652098715879581	-0.0884429999999992	0.702088376583556
IGHMBP2	-0.0764984341492664	0.833018707242402	-0.0499699819307313	0.941458038227918	-0.060835666666666	0.786861016364811

CFTR	-0.076504999925722	0.833018707242402	-0.160301981953727	0.762321667379314	-0.0725196666666661	0.784292975250979
GABRA5	-0.0766543432813067	0.832529386957116	-0.0798826514816945	0.897892885174022	0.151694	0.610151958903093
CDH20	-0.0766811816099796	0.832526815184028	0.0349868751102826	0.959140268168928	0.0350913333333337	0.892279052416171
IL3RA	-0.0767143781477954	0.832459652004448	0.0338492077548212	0.960223960238245	0.103857666666667	0.641044966033937
PSG7	-0.076747354797967	0.832338431740274	-0.147273026878328	0.78561060123541	0.100318000000001	0.630227347736008
HPR	-0.0767553818539467	0.832338431740274	-0.316417266993753	0.432844809779835	-0.40132	0.0487161951903312
ZNF530	-0.0767624325649215	0.832338431740274	-0.1272317317172	0.819791676572254	-0.0082639999999984	0.983690244124512
ADAP2	-0.0767637379316393	0.832338431740274	-0.401431784956445	0.261687361201831	-0.0331736666666662	0.895510661232872
GPR1	-0.0768108168251835	0.832201794741737	-0.234852427946637	0.607957283325678	0.220203000000001	0.422658139524950
EPB42	-0.0768394432427558	0.8320857489809	0.142339660781230	0.794365292705815	-0.0115399999999991	0.971448267683311
MAEL	-0.0768787862124337	0.831980249035714	-0.0951614988219992	0.872962271153999	0.175568000000000	0.408178621977204
MGP	-0.0769505316692306	0.831643478779273	-0.0501308101119233	0.941163653850686	1.35738233333333	0.0195270907849587
PPP1R12B	-0.0769569466314941	0.831643478779273	-0.156435681532951	0.768909201237015	0.146195333333335	0.518836392563847
EYS	-0.0770062681123048	0.831432840044339	-0.0067739564749766	0.99129507664977	0.0960290666666671	0.691702376599988
IL16	-0.077013807763373	0.831432840044339	4.89741311851543e-05	0.999902915051594	0.0385766666666667	0.889343227773276
TREML2	-0.077028599475847	0.831432840044339	-0.120287613055690	0.834809734605012	0.0346970000000008	0.904788907158467
SP7	-0.0770396819724157	0.831432840044339	-0.179301633142085	0.725260813023794	-0.143683333333333	0.49038083653267
WNT3	-0.0770659031563092	0.831432840044339	0.348366865011512	0.361311804770494	-0.0108216666666657	0.979355199193915
CNPPD1	-0.0770932524574486	0.831336368451807	0.281443142056761	0.506335763951991	-0.0628009999999994	0.780049873103571
U2AF1L4	-0.0771078967361802	0.831336368451807	0.0544396663558892	0.9344692810234	0.0943080000000002	0.712406292376982
USP24	-0.0771954079820193	0.831018971104964	-0.160549686444889	0.761610418244687	0.198275666666667	0.343572153228906
LTBP3	-0.0772667612419782	0.830763943835194	-0.0204376018364680	0.976412247012224	0.0891656666666673	0.762898679429892
DEFB124	-0.077273709326671	0.830763943835194	0.0748201044918316	0.904466414650249	0.0476470000000001	0.902208543897795
STK10	-0.0772861268883876	0.830763943835194	-0.342355654497219	0.376190347727420	-0.286968666666665	0.134443467906163
SGSM1	-0.0773270255692503	0.830763943835194	0.207427330765257	0.668625825887966	-0.045729666666667	0.866671423175521
IFNA10	-0.0773712215667231	0.830763943835194	-0.0971332014749336	0.870020870354202	-0.161352000000000	0.566282142073858
RAB11FIP1	-0.0774831266505847	0.830316649560768	-0.113418919709154	0.845608298802641	-0.171404000000000	0.368449159208846
OSTCP1	-0.0774872743629246	0.830316649560768	0.0330417773285094	0.961277493638149	0.0631033333333338	0.783285861566748
SORL1	-0.0775363932241924	0.83018333245354	-0.384793212981107	0.291180853572672	0.346036666666668	0.143763220356682
AP3B1	-0.0775556227742562	0.83018333245354	-0.324779901141529	0.411071217323391	0.129509666666667	0.532458701847411
TCEB3	-0.0776018247424006	0.830033939199431	-0.267384235556318	0.536380012714446	-0.0930583333333322	0.695962292251784
NPW	-0.0776108019370404	0.830033939199431	-0.116442513376113	0.842082118939645	-0.0087363333333334	0.982398683576954
PGD	-0.0776367556332175	0.830033939199431	-0.289432856261825	0.488870852256136	-0.0286719999999983	0.914418029309221
FPR1	-0.0776400979091244	0.830033939199431	-0.417617579036184	0.234149160737542	-0.0100976666666666	0.978037992435615
IL36A	-0.0776966588780579	0.829966593075974	0.0963932196769752	0.871509004988527	0.0928073333333342	0.68135180147671
STIM2	-0.0776988776853497	0.829966593075974	0.114880423822821	0.844162087612813	-0.0703599999999994	0.752992371482229
SLC6A5	-0.0777258795591996	0.829914357380686	-0.00337631591782071	0.994730189431381	-0.154649333333333	0.516217364739058
STK19	-0.0777299727923167	0.829914357380686	0.250178729043240	0.574321063440325	0.103697000000000	0.634587745028953
UBXN2B	-0.077744095084392	0.829914357380686	-0.179860111014181	0.723716289903531	-0.0303019999999984	0.93414152758924
CLTCL1	-0.0777765651142566	0.829870140879399	-0.0892823919964408	0.883460564717377	0.0344396666666681	0.890094615232462
SLC1A1	-0.077799852684659	0.829855506783046	0.101826902598747	0.864633209174609	0.472743	0.0295118531519703
ADAL	-0.0778056701522402	0.829855506783046	-0.186906772235565	0.710476504107514	0.00735866666666794	0.982462715783175
GABRB1	-0.0778186625209834	0.829855506783046	-0.125176200570369	0.824230922276958	0.092489666666667	0.661383805087653
ZBTB43	-0.0778366664232899	0.829855506783046	-0.0576598312494272	0.929758936765496	-0.120230333333333	0.603463436419421
UTP23	-0.0779027888948681	0.829658582627836	-0.0808457132973972	0.896361013063453	-0.137395666666666	0.483840860674189
CSE1L	-0.0779572054978704	0.829444400493641	-0.220196648657026	0.640508005020569	0.096386666666669	0.670113640649168
STK16	-0.0779782212006878	0.829377112336399	-0.134462829049175	0.808009477165608	0.234722333333334	0.338230698383994
USE1	-0.0779855506516723	0.829377112336399	0.096820015785547	0.870711896934693	-0.0424009999999987	0.868791301779828
TMEM38B	-0.078017913383807	0.829377112336399	-0.0376450808337031	0.955365651737247	0.235291333333333	0.255766698590661
ZNF597	-0.078028179540209	0.829377112336399	-0.17779119467665	0.728031191477223	-0.0465726666666653	0.861165447055876
CLCC1	-0.0780494016933548	0.829366028455963	-0.396605432712401	0.270885885233685	0.135077666666668	0.503217861354116
TTC27	-0.0780596485835655	0.829366028455963	-0.0438097276259294	0.948934431299106	0.359698	0.0683459106150144
MIER2	-0.078095929301726	0.829298563821772	0.084247348817209	0.892396784625427	0.239941666666668	0.224147588185176
XPNPEP2	-0.0781646705507568	0.829060049879976	-0.00555925332077079	0.99251028338798	-0.191422333333333	0.465333992920292
ZNF467	-0.0781653526093567	0.829060049879976	-0.076789658633743	0.901856698918763	-0.0170406666666665	0.965329851466378
IL1A	-0.0781722378839477	0.829060049879976	-0.0982138633474373	0.868820771084259	0.0500446666666674	0.83822720696374
LOC648987	-0.0782419660571962	0.828902208287575	-0.0796511992966857	0.898031113962014	0.309303333333335	0.199375971292473
C10orf71	-0.0782970574195454	0.828751453782627	0.087634299428521	0.886302037299497	-0.0766703333333329	0.729912257977231
CYP1A2	-0.0783425516945347	0.828594557264245	-0.0309538962098178	0.965259090841226	0.0341656666666676	0.924674647382585
DHRS3	-0.078392222236369	0.82847865583559	-0.038675183346725	0.9543445884646	0.103334000000000	0.653246784345488
TMEM120B	-0.0784204242359692	0.828434154189902	0.308313996292065	0.450974903594884	-0.297688666666666	0.142821378450256
ELAC2	-0.0784345349501138	0.828434154189902	0.0709963704531448	0.909387602167883	-0.0510566666666661	0.824700707442131
TG	-0.0784395624990134	0.828434154189902	0.0445796618782681	0.947920610570169	0.0589196666666674	0.812504992213931
KRT33B	-0.0785873125068406	0.827902354085427	0.253645069640376	0.567705016149998	-0.165608333333333	0.602084409940976
NYX	-0.0786044877558549	0.827859732601988	0.281420591532036	0.506335763951991	-0.174757	0.523453039510001
RANBP6	-0.0786165061426164	0.827859732601988	-0.0251687243848332	0.972358966414305	-0.134228666666665	0.511044163684822
MARCH11	-0.0786315244325241	0.827859732601988	-0.0418914651526082	0.951989245458556	-0.0633133333333324	0.800384927463055
SOSTDC1	-0.0786654069004821	0.82773931324695	-0.280224087473530	0.507980347140282	-0.0596449999999998	0.809505390888975
RHOC	-0.0787605755179937	0.827262752676621	-0.320192663329883	0.423227776324677	0.244533	0.226059303638588
SMYD1	-0.0788244343404148	0.826987644413982	0.0709264099034147	0.909476930273377	-0.128968666666667	0.640799282945291
EPS8L3	-0.0788966520886187	0.826726607033426	0.0622604357171721	0.92301259447339	-0.135471000000000	0.557700710345762
LOC728606	-0.078901531325014	0.826726607033426	-0.549375921355425	0.0608648191629941	-0.264997333333333	0.320901958611039
USP35	-0.0789107592078323	0.826726607033426	0.505671261047459	0.105941223458825	-0.00364066666666552	0.993346316662499
IL17C	-0.0789484385841203	0.826664857165768	0.0642324648021213	0.919923865590619	-0.150448000000000	0.575589336285762
MYCBPAP	-0.0789729429711479	0.826642925937737	-0.00932978910102024	0.98909166696601	-0.054831666666666	0.826347200876756
COMMD5	-0.0789862681839955	0.82662505780585	0.150581281703046	0.779021144254203	-0.181825999999999	0.344959227986463
DNAJC28	-0.0790290438706573	0.826417682862372	-0.243320165175436	0.591446362805643	0.390872666666668	0.324432624046557
TC2N	-0.0790434944586412	0.826392568298105	0.0345255393576077	0.959404955370931	0.184479000000000	0.430018092058755
INSL3	-0.0790493911140233	0.826392568298105	-0.0850075407359935	0.890877295424996	-0.242530666666665	0.274121181062751
SLC6A3	-0.0790544156075412	0.826392568298105	0.238162739371177	0.60184463266698	-0.0627483333333334	0.822657709336468

KCNC1	-0.0790716314089878	0.826392568298105	-0.212259422841375	0.657494257179496	-0.113797666666666	0.719909781429035
MOGS	-0.079074462021791	0.826392568298105	-0.185431564548688	0.714427536588157	0.0623486666666674	0.82667899725046
TMEM178A	-0.0790822461065532	0.826392568298105	-0.111432635966308	0.848968422336654	-0.107792666666666	0.648325366843053
DARS2	-0.0790834707380655	0.826392568298105	-0.414037486616804	0.238232786801887	0.198850666666667	0.424186863556771
MIPOL1	-0.0791443066271737	0.826354793489329	-0.349887696350303	0.358969704130952	-0.0963339999999993	0.664617185770066
SMURF1	-0.079151846848099	0.826354793489329	0.103292375679317	0.862065338914705	-0.105917666666666	0.689543334701612
OGFRL1	-0.0791866578683102	0.826285925135397	-0.158989364289952	0.765434558091042	0.192939000000001	0.405689711040978
ZNF765	-0.0792264906479082	0.82609741752045	-0.300051436083653	0.466362574195078	0.0559366666666673	0.809755698910303
SOX9	-0.0792443123517219	0.826050608408904	-0.139052155072202	0.79864602540281	0.133146333333333	0.517547647029412
ADCY2	-0.0792963534327652	0.825851423988258	-0.107998843247379	0.8559005029888	0.190893333333334	0.529628396179385
SSSCA1	-0.0793025959989596	0.825851423988258	-0.35523326752039	0.347833413629182	-0.0719029999999992	0.74511187686951
SCN7A	-0.079365579299545	0.825745364022863	0.127591504196677	0.818717515586031	-0.0825579999999998	0.712509439611678
IFITM3	-0.0794510324551168	0.825263133541192	-0.286550594848031	0.495619629661258	-0.0937896666666658	0.667697330885416
LTA	-0.0795174765036608	0.82490333126863	0.00592645289784883	0.99251028338798	-0.104449333333333	0.70610473471702
MRPL24	-0.0795355704554824	0.82485474776403	-0.124789841115696	0.824576228369528	0.0944506666666671	0.670071773006358
ADAM28	-0.0795847865110625	0.82467372116312	-0.294862567107896	0.478695647537866	0.153394	0.588052519326463
SCGB3A2	-0.0795862582136762	0.82467372116312	0.197206333845804	0.689473813330868	-0.0495023333333333	0.8754328609757
NDUFS1	-0.0796657002160807	0.824425222681619	-0.0589453547264248	0.928158451370015	-0.0360483333333327	0.88629962370345
INPP5D	-0.0796737458974862	0.824425222681619	-0.371828986261296	0.319073951161831	0.0606043333333335	0.800532222303294
PODNL1	-0.0796754149521925	0.824425222681619	0.073763622578518	0.905506084101768	-0.083541333333332	0.80340014472271
TMCC2	-0.0797091383112297	0.824425222681619	-0.0801376249311414	0.897577172229156	0.0404743333333334	0.870037639560767
ALX3	-0.0797252679262175	0.824425222681619	0.179023977196161	0.725806648980606	-0.216548666666667	0.459936388684371
PRPH2	-0.079730361784801	0.824425222681619	-0.251468941032195	0.571843458748	-0.0602113333333325	0.841976773189511
HMHB1	-0.0797501955759371	0.824425222681619	0.285003512649499	0.49838641873573	0.0029986666666672	0.993756838831997
FECH	-0.0797859474969115	0.824425222681619	-0.517176109335775	0.0916291648883415	0.0486510000000007	0.838603547123369
OTUD5	-0.0798360969500994	0.824425222681619	-0.293626322476841	0.480614908214975	0.0114943333333328	0.97222097877396
FAM124B	-0.0798708383970783	0.824402690529472	-0.390099602083149	0.282383744168943	-0.134594	0.518741522051306
MSR1	-0.0799128926280088	0.824327058081886	-0.409910231154727	0.247372798192455	-0.104082000000000	0.607898776363481
MED14	-0.0799895686994454	0.82396902432866	-0.413853329631263	0.238622785037791	0.115993000000000	0.614221661998915
UNC79	-0.0799980309163509	0.82396902432866	0.0531010598994973	0.936712858731269	0.142619333333334	0.655951370096932
ATG9B	-0.0800662056417812	0.823815170253002	0.0513775365368738	0.938729151196048	-0.0655513333333332	0.765209686566971
KIF1B	-0.0800876525851142	0.823744969136392	-0.121221128279183	0.83256316766566	0.0738236666666676	0.773603738414183
EIF2AK1	-0.080151983976982	0.823433731057713	-0.214061217029636	0.653131926870065	-0.196266333333332	0.346260444647043
DNAJC11	-0.0801927121949072	0.823272005006758	-0.327398370040634	0.40846136066764	-0.217864333333334	0.278320107413092
CLDN14	-0.080203433159	0.82327090142424	0.0841606348934963	0.892465571300622	0.0244850000000006	0.930620998877894
TACR3	-0.0802306380097172	0.823163594732295	-0.0497739515789872	0.941458038227918	0.0792010000000006	0.759445936839873
GPHB5	-0.0802635459258319	0.823036076658762	0.0486913743014445	0.94227068186505	0.00887133333333438	0.979297677025106
TNFSF9	-0.0802797286328242	0.823036076658762	-0.0227694603791946	0.97536722461972	-0.157823666666666	0.471741080987819
WT1-AS	-0.0803030199092738	0.822969119729287	0.380256369808473	0.300705577749487	-0.351127333333333	0.236080893333632
FRMD3	-0.0803053962346043	0.822969119729287	-0.300472397320592	0.465764240927686	0.411673333333334	0.0974090174618997
GNPAT	-0.0803954781158825	0.822645269942716	-0.160650702325860	0.761367458467566	0.282758666666667	0.138021116806123
CELSR3	-0.0804030807043497	0.822645269942716	-0.233915686992498	0.609624481075982	0.00277333333333405	0.993756838831997
TNNT1	-0.0804077726041576	0.822645269942716	-0.192625609899934	0.700136059561707	-0.214514000000000	0.25721699788802
GSTO2	-0.0804126522055799	0.822645269942716	0.145019754026128	0.789141135622353	0.0929670000000006	0.717929175548401
CHUK	-0.0805110555989405	0.822376348207365	0.0606023623984074	0.925462896587765	0.114113666666667	0.587396808178803
KPNA6	-0.0805159617320298	0.822376348207365	-0.237461387992936	0.603017682531881	-0.0785829999999976	0.719304894613107
RBP4	-0.0805209671809256	0.822376348207365	-0.157918332049590	0.767373487136803	-0.115901333333333	0.583036318848746
MAT2A	-0.0806519175476397	0.821672573402839	-0.189424758480515	0.705411515598974	0.0571633333333346	0.799725367501923
HSF1	-0.0806601771122639	0.821672573402839	0.306585823784777	0.455223825739952	0.0861816666666678	0.690826917301738
HDAC11	-0.080699819203671	0.82149983552118	0.290656003388481	0.487935421898319	0.0314780000000007	0.90232248061402
ANO6	-0.0807068164203441	0.82149983552118	-0.277643741513226	0.513837569240538	0.465355	0.0326806169766676
ACOT4	-0.080735457638355	0.821411131894489	-0.230855293121725	0.61674758240945	-0.140770000000000	0.49912108040986
PLD4	-0.0807585217882796	0.821393395038899	-0.126841960038338	0.820883225356572	-0.0258616666666667	0.938187566801362
FAM3B	-0.0808129642760451	0.821140446469847	0.0483136964961847	0.942853417571722	-0.120747333333333	0.639002829892966
BCDIN3D	-0.080815733051996	0.821140446469847	-0.187139441545523	0.709833349594307	0.183240333333334	0.449693583707481
C20orf27	-0.0808558759521362	0.821037886678547	-0.0386244111067440	0.95449583203831	-0.218952	0.302861002390456
PALM	-0.0808602724167805	0.821037886678547	0.0306232495022965	0.965647626065546	0.0577650000000008	0.863479649214073
ANKRD53	-0.0809893565767991	0.820606662945023	0.0477940108104592	0.943493260394976	0.0489630000000004	0.910920430866321
CXADR	-0.0810254244842171	0.820556885650424	-0.396421483975946	0.271136524502442	-1.08774066666667	0.0327193986189533
FCN1	-0.0810566531270845	0.820491549519488	-0.182065773809089	0.71977894194971	-0.0387273333333327	0.90232248061402
FANCG	-0.0810803781676707	0.820406607214205	-0.043884963895475	0.94885721615997	-0.0909826666666654	0.695134555947856
C6orf141	-0.0811012573654448	0.820340005129058	0.139852777783094	0.797943073857778	-0.0466733333333327	0.865571667607644
PRSS54	-0.0811081114565919	0.820340005129058	0.0150622570126807	0.982921609770846	-0.104722666666667	0.605491766505367
VAV3	-0.0811266693669194	0.820340005129058	-0.309860460786824	0.4470577615309	0.0928653333333343	0.734997389745078
ALDH16A1	-0.0811840200773583	0.820146417049954	-0.123586198157192	0.82658494329903	-0.0531646666666669	0.861165447055876
TPTEP1	-0.0812001499180004	0.820110423542542	-0.103968830107382	0.861570109612412	0.199082666666667	0.299328994319573
HTR7	-0.0812328396184356	0.819967684835816	0.074777097878264	0.904466414650249	0.00261533333333370	0.993756838831997
ZNF720	-0.0812539374632023	0.819899664926831	-0.0378777316125617	0.954958350556272	-0.0802296666666656	0.743139552172972
ZAK	-0.0812941994833948	0.819784209883643	-0.074209759772302	0.905252113490377	0.182259333333333	0.352237134604623
SFTPB	-0.0813607498853912	0.819618996757204	-0.0231141672134106	0.97536722461972	0.0351350000000001	0.903774117860245
PLEKHO2	-0.081446967802825	0.819239680942778	-0.1575786950474	0.767770230680061	0.0105840000000005	0.972570911594307
GTF2A2	-0.081456267020148	0.819239680942778	-0.213394722551521	0.654550434157114	0.130706666666668	0.523021362996592
MRPL23	-0.0814903192868371	0.819173577079238	0.0265628630471305	0.970207612777188	0.0470410000000005	0.884344884568493
WNT10A	-0.081496930526252	0.819173577079238	0.168619629038408	0.746758286599055	-0.108472999999999	0.670719950296173
IGF1	-0.0815434337265143	0.819173577079238	-0.0265526053893808	0.970207612777188	-0.285095333333332	0.306281656395861
FLCN	-0.0815680958253236	0.819098087878614	0.0957532432415452	0.872537273851412	0.0784426666666668	0.771983588857888
ZNF7	-0.0816404310405292	0.818767665588243	0.181332031671659	0.721196149497303	0.136582000000000	0.503217861354116
RPL8	-0.0816538840955475	0.818748916169369	0.361067474823015	0.339627732901974	-0.0506833333333331	0.848860592348058
PSORS1C2	-0.0816583259330821	0.818748916169369	0.186692729992428	0.711116322689443	-0.162211333333332	0.579618427881693
NPY5R	-0.0816654325982169	0.818748916169369	0.0624455742473653	0.92264749701958	-0.115450333333333	0.592716584553539

NOXO1	-0.0816824779151948	0.818748916169369	0.300675562964791	0.465222095816255	-0.160416666666667	0.543818940802043
IDH3B	-0.0817176073387659	0.818746052330418	-0.200038373393372	0.682072046670084	-0.151701666666666	0.439061344563948
PAIP2B	-0.081740966687809	0.818663419242971	0.252306602362212	0.570311620130916	-0.159704333333333	0.496323393705678
RASL11A	-0.0818267636193927	0.818246119998384	0.132947133776868	0.809578511927685	-0.217450666666666	0.313220411537404
PLEKHG6	-0.0818300372774958	0.818246119998384	-0.0271497411881614	0.970207612777188	0.0203646666666674	0.94622978991441
LGI1	-0.0818434658600227	0.818246119998384	-0.307164770735788	0.453060894675967	0.0118606666666670	0.98025379113353
ASB2	-0.0818775217128737	0.818245513109162	-0.294018465269828	0.480297745849088	-0.107692333333333	0.721454370213253
LOC100129476	-0.0818919758974097	0.818245513109162	0.303879807130891	0.460052080110635	-0.100325333333333	0.662751767068109
IL4R	-0.0819417439143543	0.818048689475348	-0.477582300436512	0.141622491285205	0.373081000000001	0.155294715110343
C4orf22	-0.0819610742196312	0.818048689475348	-0.128872539204718	0.815391045978843	-0.0287906666666663	0.915761046438965
TNFRSF9	-0.0819951145267866	0.818004296451419	0.142085865350293	0.79450771779567	-0.0569533333333334	0.812623432909957
FLJ30679	-0.0820269902109257	0.817975085559353	-0.0600866981862648	0.925676268175378	0.0356190000000008	0.95197590164177
STH	-0.082151478366104	0.817580231291201	0.143463718564175	0.792621058154238	-0.231240000000000	0.455226686708489
SNX14	-0.082157906056358	0.817580231291201	0.104992573079899	0.8606536389692	0.195963666666667	0.318250389687098
MT3	-0.0821628981318681	0.817580231291201	-0.127813488297175	0.818166345526178	-0.515975666666666	0.0309320552705188
TP53TG5	-0.0822025364475116	0.817557122520272	0.224120410451044	0.633218729479524	0.0247280000000006	0.941559580750404
ACTB	-0.0822389232368725	0.817495180115421	-0.60355789361485	0.0309735949886774	0.0830266666666695	0.705260317228378
ZNF708	-0.082248863316472	0.817495180115421	-0.0800980196961861	0.897577172229156	0.274961333333334	0.209300554554219
PARS2	-0.0822833043212366	0.817410343832058	-0.229647180168541	0.619947904227279	-0.0975933333333334	0.640921133526682
SLC25A19	-0.0823069821766113	0.817325612488403	-0.107347081830673	0.857128584932911	0.120321666666667	0.674531431277108
DOCK8	-0.0824801056063672	0.816344489375968	0.100669803406051	0.86629297357855	-0.062817666666666	0.841844674599552
LRTOMT	-0.0824833221584536	0.816344489375968	-0.181036637637054	0.72167134029303	0.058127666666668	0.853219850381138
DNASE2B	-0.082499582357256	0.816344489375968	-0.225490685717780	0.629710977741725	0.0198293333333335	0.95170733221857
ZNF613	-0.082505936549845	0.816344489375968	-0.0691907734343526	0.911837389834658	0.125993333333334	0.595937351595018
LOC399491	-0.082566072429759	0.816115174191564	0.196353225860032	0.691445158350448	0.144607888888890	0.460428849906031
CENPW	-0.082574189581086	0.816115174191564	-0.0573407688127265	0.930490408235626	0.0132050000000006	0.979790222743567
SETD3	-0.0825795306201836	0.816115174191564	-0.265867104456044	0.539781998644881	0.0303860000000007	0.90945379489823
MYBPC3	-0.0826033489783684	0.816093630448644	0.00283207598733621	0.99552313490055	-0.0526703333333323	0.82700027112124
LRAT	-0.0826052154193755	0.816093630448644	-0.304215626203583	0.459515290473612	0.00850000000000043	0.979527173532206
OR2H2	-0.0826130719496889	0.816093630448644	0.179319416985184	0.725260813023794	-0.277746333333333	0.441460337585051
MAP2K4	-0.0826230419863317	0.816093630448644	-0.130915413992277	0.812271254601992	0.121365000000002	0.561051487344043
FBXO45	-0.0826277368971126	0.816093630448644	-0.115883092255113	0.842733236705133	0.0417216666666667	0.880858809336225
COX6C	-0.0826378729598092	0.816093630448644	-0.267848418716132	0.53559545997875	0.304073333333335	0.150274389990121
PTGES3	-0.0826737071161025	0.816093630448644	-0.0417568086535787	0.951989245458556	0.143183333333334	0.536063442230912
RNF185	-0.0827387612357542	0.815900425462494	-0.161834019373731	0.758930446817487	-0.141718833333332	0.653246784345488
UTP11L	-0.0827443740376837	0.815900425462494	-0.0223717694409713	0.97536722461972	-0.193548999999999	0.313220411537404
DEFB132	-0.082770141795532	0.815834046799631	0.069890058850837	0.910885619751085	-0.0747059999999997	0.829384095613517
TNK1	-0.082819763660716	0.815754768166546	0.218338448351742	0.642971191969613	-0.0497363333333322	0.902208543897795
RENBP	-0.0828439785326456	0.815754768166546	-0.152487077473085	0.775863712880333	-0.0470953333333338	0.90945379489823
TCF21	-0.0828572907103701	0.815748207746208	-0.0332188680737992	0.960903266605349	-0.00730933333333284	0.983159241812861
DUSP13	-0.082874495097787	0.815705245112023	-0.0424173992019734	0.951446425515852	0.0489040000000014	0.838777437201588
NPVF	-0.0829018950174006	0.815596438055816	-0.281745832101081	0.505724230030045	0.0146263333333336	0.95787549045386
SDK2	-0.0829267943300943	0.815503779080687	0.109351164302701	0.85210721238532	-0.202499000000000	0.40610776759241
ARHGAP22	-0.0829327182258149	0.815503779080687	0.0278087544911974	0.969317858168394	0.0289876666666672	0.915761046438965
DYRK4	-0.0829699389417135	0.815361429470783	0.0585693080995264	0.928586335857223	-0.0371606666666664	0.890511682678135
SOAT2	-0.0829837200211587	0.815340570416145	0.162566170535234	0.757614806491095	-0.0257309999999996	0.942393983671961
SMCR7	-0.0829951433534232	0.815334939912188	-0.0951838719672596	0.872962271153999	-0.0832186666666661	0.730777457405814
LOC113230	-0.0829969006657715	0.815334939912188	-0.0169824306033820	0.98105020869911	-0.204185666666665	0.423540014909016
OPLAH	-0.0830074630851693	0.815334939912188	0.451231064771328	0.176663460992485	-0.128334666666666	0.662751767068109
LCP2	-0.0830635297127089	0.815271505761703	-0.438068021147044	0.193743994785626	-0.0672996666666665	0.811401072147441
MPI	-0.0831014984404371	0.815271505761703	-0.0438677961741026	0.94885721615997	0.0884056666666679	0.68724506397038
TMEM159	-0.0831097824397053	0.815271505761703	-0.295345542856299	0.478306608395718	0.272986666666667	0.286939266819770
AKNA	-0.0831189743681293	0.815271505761703	-0.253737982963383	0.567705016149998	-0.0913646666666657	0.705698790800484
YPEL1	-0.083142197088826	0.815271505761703	-0.27030032545952	0.530011828941681	-0.364903999999999	0.0984855525784605
IKBIP	-0.0831543556657094	0.815271505761703	-0.110644452821969	0.84940885580427	0.0295516666666665	0.92590541082869
ZNF235	-0.0832275980463153	0.815067443750836	-0.165108601187234	0.7529423475214	0.0142623333333339	0.96051270333788
KRTAP9-4	-0.0832629410382201	0.815036786665744	0.0862814770078761	0.88794718020464	-0.0012566666666666	0.99704422037925
MAGOH	-0.0832707724620024	0.815036786665744	-0.215031680154698	0.651257878577277	-0.0937409999999995	0.661574121674287
MIXL1	-0.083284508607939	0.81503387305182	0.176027290951268	0.731161648783055	0.0112893333333339	0.978109825558428
C17orf78	-0.0832901259336852	0.81503387305182	0.0319595014083629	0.963062370853298	-0.0565856666666667	0.825269786103875
NKX2-1-AS1	-0.08330660783475	0.815027532221299	0.305222779143250	0.458013550008763	-0.0430906666666661	0.902208543897795
CLRN1	-0.0833211120785022	0.815002030776523	0.0906706851275698	0.881370595149313	0.0828556666666667	0.831328608591573
EPX	-0.0833562390148128	0.814850396015408	0.0540622440916752	0.935109055935003	-0.0626893333333325	0.831076445763244
LEMD1	-0.0833638036418282	0.814850396015408	0.198902429893469	0.68526131681093	0.0477926666666666	0.852208015389387
FDXACB1	-0.0833641353402786	0.814850396015408	-0.333665685056523	0.396821740334178	-0.0739999999999995	0.848860592348058
RECQL5	-0.0834551329561649	0.814613447478324	0.456078181713838	0.167648587973919	-0.0116706666666651	0.9681321732097
GLT8D2	-0.0834798134977254	0.814565021892598	0.0292267807674175	0.967677112447108	0.365673333333334	0.195340089722887
MAGIX	-0.0834990005179958	0.814565021892598	-0.170148463414339	0.74270446821332	-0.196452333333333	0.559214038647867
CCDC155	-0.0835000060707252	0.814565021892598	-0.026993233696933	0.970207612777188	-0.0526286666666659	0.82616467676523
MYO1B	-0.0835182569650115	0.814565021892598	0.191853681532424	0.70150833118141	0.414794666666668	0.0649438674882112
CALY	-0.083527629382064	0.814565021892598	0.121168728507556	0.83256316766566	-0.239666000000000	0.475308057726924
CRNN	-0.0835594914246137	0.814565021892598	0.069529898088339	0.911449822745146	0.135201000000001	0.582734747347156
FGF7	-0.0835834612591057	0.814534880738293	-0.204067320703082	0.675344930297902	-0.0799706666666666	0.747173802433489
IL1R1	-0.083598837995326	0.814534880738293	0.0480188882594256	0.94314236584312	-0.318055999999999	0.173286092065797
CEP57L1	-0.0836154489891518	0.814534880738293	0.00446448827907458	0.993960037436622	0.197322666666667	0.309421300309741
TM9SF1	-0.0836321062280857	0.814534880738293	-0.0201645651839755	0.976956866647027	0.144782000000001	0.462226947084334
CPA3	-0.0836510618985344	0.814534880738293	-0.212873630860299	0.655695431812946	-0.0954376666666664	0.648325366843053
ATOH8	-0.0836737343275262	0.8145231684629	0.111296437339950	0.849080210054875	-0.0814199999999993	0.797534995180308
PFN4	-0.0836933228132606	0.8145231684629	0.071751315751176	0.90837606217316	-0.0381366666666654	0.88436256532321
GIMAP8	-0.083726484894358	0.8145231684629	-0.424436582289492	0.216714808349232	-0.144952999999999	0.476515795581148

CCDC64B	-0.0837306853998494	0.8145231684629	0.126837909976043	0.820883225356572	-0.0571176666666654	0.897210169322072
MRPL21	-0.0838432054785372	0.814153196483831	-0.255779931439391	0.565656702091657	-0.117543333333333	0.613418255499423
TSTD2	-0.0839154667676143	0.813890682407262	-0.0784979648723137	0.899281198071059	-0.128169333333332	0.528501553687416
RAB3C	-0.083996684360564	0.813501797357779	-0.0068890965779907З	0.99129507664977	-0.290773666666666	0.671766969198954
OTOP2	-0.0840543550721662	0.813402095002614	0.171463726665728	0.739541495555634	-0.120264333333333	0.631408228698652
LRRC42	-0.0840663148623092	0.813393081257621	-0.119038636296029	0.836828045978697	-0.0150776666666665	0.956015594359323
CR1	-0.0841220301968181	0.81310084645348	-0.252887222440982	0.568985221714455	-0.142712333333333	0.637602236970228
ETFA	-0.0841224814765276	0.81310084645348	-0.134430736538757	0.808009477165608	0.280303666666667	0.167461485379690
OR10A3	-0.084140272313782	0.81310084645348	-0.0229323981156578	0.97536722461972	-0.0193069999999993	0.948778075888142
CUL7	-0.0841586493959699	0.81310084645348	0.182625411697550	0.718746116296779	-0.101213666666665	0.64090250408923
PANK2	-0.0841709777269938	0.81310084645348	0.078859192166774	0.899154242391172	-0.074645	0.756484285494797
MMP8	-0.0841881509743072	0.81310084645348	-0.00356507972379478	0.994730189431381	0.0683646666666671	0.774104133965233
CAMK2G	-0.0842880299329154	0.812778706244708	0.148526984651844	0.783532632807022	-0.153774999999999	0.488602547609567
DUSP12	-0.084305703176244	0.812732703830493	0.0458358990857139	0.945372627844176	-0.199237333333333	0.370122454788249
LINC00478	-0.0843974016848869	0.812364878203395	-0.0935048187293893	0.876286451056853	0.216362666666668	0.326398785133011
SAMD12	-0.0844055520571624	0.812364878203395	0.0218757545647144	0.97536722461972	0.114373333333334	0.695654544311453
SOX13	-0.0844583946651586	0.812364878203395	-0.0491449560462115	0.94196835509895	-0.200661666666666	0.452846213844736
SUSD3	-0.084493974676356	0.812364878203395	0.0369090925289284	0.956918184553224	-0.0803776666666657	0.735938662411184
GGTA1P	-0.0845021194511419	0.812364878203395	-0.352337620312719	0.352921806679009	-0.084525333333333	0.73350847296189
TMCO4	-0.0845202285147193	0.812364878203395	-0.194837133943869	0.695517703853906	0.231469666666667	0.216848931674793
USH1G	-0.0845541126515994	0.81228765910092	0.301030987976988	0.464782507068736	0.0850780000000018	0.716517864791776
LRRC33	-0.0845911200131033	0.812116413950308	-0.178254736963289	0.727675849947241	-0.188914000000000	0.484148891768163
ACTL9	-0.0846021507618195	0.812113437700045	0.0478200805315573	0.943472656931067	-0.129890666666666	0.576277303044571
ATF6	-0.0846194098225795	0.812070116289892	-0.0887153356915564	0.8841943128258	-0.108547666666665	0.62570317772964
ALKBH1	-0.084621788159602	0.812070116289892	-0.232976340496518	0.611711680668619	0.175231000000000	0.49155002081903
HOMER1	-0.0846360806955094	0.812070116289892	0.169530037394038	0.743870299337847	0.211060333333334	0.302316846105060
GIMAP2	-0.0846402021897438	0.812070116289892	-0.356560622673844	0.345989229646028	0.221888666666668	0.407125415904361
REXO1L1	-0.0846474506472751	0.812070116289892	0.268317377546144	0.535217216332192	0.0146902857142865	0.957844701580613
C20orf173	-0.0846586886727651	0.812070116289892	0.205525491843530	0.672695616352514	-0.156856666666666	0.528488494367153
TCN2	-0.0847034415255524	0.812070116289892	-0.457514210850377	0.166671273245692	0.0609459999999999	0.789917107854685
FBXL6	-0.0847080049272936	0.812070116289892	0.456347840091408	0.16754724558121	-0.152595666666666	0.467798588118316
GOLIM4	-0.0847262912095645	0.812070116289892	-0.0939983190161376	0.875355472414883	0.0524623333333352	0.842867380083783
CARNS1	-0.084733303767077	0.812070116289892	-0.0194925440419883	0.977705240182506	-0.125868999999999	0.677999095007544
KRT75	-0.084748777658499	0.812070116289892	-0.0225351336790155	0.97536722461972	-0.085703333333333	0.686926936590232
TMEM167B	-0.0848361486432129	0.81189906976435	-0.222101083303791	0.636477566065103	-0.121391666666667	0.584809009907698
KIT	-0.0848623331313925	0.811797895678378	0.219422299002731	0.641226345931986	-0.141130666666666	0.500886251638044
MXD3	-0.0848743983638802	0.811788252000516	0.327093058998509	0.408851707589499	0.0594590000000005	0.845737357656109
PBLD	-0.084878559253888	0.811788252000516	0.290000838575482	0.488870852256136	0.141282666666667	0.566075188962
TRPC2	-0.084909606300812	0.811746991716635	-0.114229597987147	0.844272085764738	-0.0412519999999991	0.883739719585572
FAM110D	-0.0849400611646182	0.811746991716635	0.224005620131359	0.633436730790476	-0.0973996666666661	0.710581219632272
ECHDC3	-0.0850676819920749	0.811289520639195	-0.393470574189837	0.276819922369918	-0.0367806666666666	0.886767946328976
STK31	-0.0850905711555582	0.811289520639195	-0.338633112097376	0.384154741685876	0.0532750000000004	0.815540776675037
ZNF77	-0.0850989313389629	0.811289520639195	-0.00621985728953179	0.99215248935175	-0.172285333333333	0.461494497197158
CX3CR1	-0.0851645364043503	0.811141686683115	-0.33004246023386	0.403195021794343	-0.212784333333333	0.275939802512171
CXorf36	-0.0852454622375853	0.810685480074015	-0.115424454352095	0.843614348588468	-0.00968833333333283	0.982375192575954
KIF25-AS1	-0.0852960609098693	0.810425976538755	-0.338358435192951	0.384701467775014	-0.0553689999999994	0.854573398968221
NADSYN1	-0.0853060435740851	0.810425976538755	0.0262761168045132	0.970631762839502	-0.212795000000000	0.279049509382533
LRRC43	-0.0853353579330644	0.810425976538755	-0.157809927701942	0.767678318854251	-0.0870080000000002	0.69206623851055
PXMP2	-0.0853990817811262	0.810286168363905	0.0446012394216634	0.947920610570169	0.176773500000002	0.399072623771344
C9orf57	-0.0854318482008094	0.810262779601573	0.00654805623986467	0.99151871436261	0.048200333333334	0.873454962131763
NFAM1	-0.085477723162866	0.810071061226507	-0.0288412144778392	0.96782691865619	-0.0910406666666666	0.756484285494797
CD109	-0.0855026997141628	0.810046296870725	-0.238655946156517	0.600861960738847	0.546494	0.0185304620090284
RAB6A	-0.0855343772544936	0.8100225008865	-0.187785402817448	0.708377771996406	0.0253401666666681	0.92927353315392
MRPL33	-0.0855707971320865	0.809879080616285	0.0233305711288825	0.975348968561756	0.079405666666667	0.720071550811076
C20orf166	-0.0855778580533824	0.809879080616285	0.0907999770745554	0.881323965029954	0.0985510000000005	0.633166039956913
IL6ST	-0.0856215513055713	0.809766007358444	-0.478011068680479	0.140842478689288	0.349006666666667	0.0746299373409457
HYAL2	-0.0856396182526456	0.809766007358444	0.208907526479492	0.665595909869988	-0.0374563333333332	0.891410328517388
URB2	-0.0856446040982186	0.809766007358444	-0.144443870641156	0.790414495161718	-0.324822999999999	0.165359581005486
ZFYVE1	-0.0856699644106357	0.809766007358444	0.0503127156065575	0.940973131693063	-0.010293333333332	0.983159241812861
CSNK1A1P1	-0.0857182563074188	0.809746235420844	0.0896945960574512	0.88318195439136	-0.103554666666666	0.641044966033937
ODF1	-0.0857833047402868	0.80966754307854	0.134224917013551	0.808009477165608	0.100664666666667	0.698352866722226
AP1B1	-0.0857954751844193	0.809657256827929	-0.178874162674913	0.726006906302736	-0.0267326666666657	0.922106493541096
KDSR	-0.0858343969254152	0.809542032370036	-0.451679877236217	0.176663460992485	0.0468583333333332	0.85184175589894
IFNK	-0.0858425078556107	0.809542032370036	-0.033671596254663	0.96061727828906	-0.0527953333333324	0.823585702195789
RNF166	-0.085848837606719	0.809542032370036	-0.189575791147518	0.704928430617595	-0.105886999999999	0.65577702857622
ADAM32	-0.0858579886501558	0.809542032370036	0.204723378462105	0.673769742574452	0.342245333333334	0.159075946945580
MNX1	-0.0859025682430222	0.809374369082773	0.112249808744140	0.847454473507881	0.0720726666666674	0.758341714501375
EPN3	-0.0859046658292514	0.809374369082773	-0.1341579037012	0.808009477165608	-0.0826289999999987	0.780474051101386
FAM71D	-0.0859154795272617	0.809374369082773	0.0161717850889120	0.982057247916055	0.0532226666666671	0.839703258597695
FAM19A2	-0.0859603710671602	0.809374369082773	-0.00537409913175481	0.99251028338798	0.0323423333333333	0.933214984828529
KCND3	-0.0859635748270585	0.809374369082773	-0.179083384467247	0.725727384118373	0.174511333333334	0.410584580608825
SPRED3	-0.0859946640332701	0.809374369082773	0.053132517529069	0.936712858731269	-0.240366333333333	0.309421300309741
ME1	-0.0860031461446664	0.809374369082773	0.38764685845676	0.286134842484356	-0.00707066666666486	0.98256318768706
MITD1	-0.0860358294337165	0.809374369082773	-0.295712793525057	0.47761182409076	-0.0357986666666659	0.899614658619676
APOL1	-0.0860622269302088	0.809374369082773	-0.296116546826681	0.477051680229433	0.118073333333335	0.634966811516658
LYZ	-0.0860813401869296	0.809374369082773	-0.468073938211292	0.151936428121882	-0.0726186666666658	0.792778259205127
CA3	-0.0860853293960115	0.809374369082773	-0.117074624470260	0.840993954510842	-0.154991666666666	0.518836392563847
RHOBTB2	-0.0860866356119791	0.809374369082773	0.171662697836520	0.739381216022144	-0.0504796666666653	0.888151072264636
FOLR1	-0.0860871092287015	0.809374369082773	0.0388492656069480	0.954228173733495	-0.0310733333333331	0.906093399285191
CNDP1	-0.0861039065414743	0.809374369082773	0.00543457567539129	0.99251028338798	-0.0203439999999994	0.939858986124333

SETBP1	-0.0861175237525569	0.809374369082773	-0.143535101138694	0.792614473650425	0.0246733333333335	0.92469922248949
OCLN	-0.0861186736618152	0.809374369082773	-0.203510838448953	0.676207233014711	0.271761666666668	0.383716544590226
CA5BP1	-0.0861229336449722	0.809374369082773	-0.261966533319717	0.5499549817547	0.00584700000000048	0.986350009478474
YIPF2	-0.0861268847439595	0.809374369082773	-0.0755992294865257	0.903517361619051	-0.0811769999999995	0.712653771000543
FAM117A	-0.0861356084667301	0.809374369082773	0.250629424075175	0.573664917766258	-0.0664923333333327	0.81000174583549
CEP41	-0.0861562826392802	0.809374369082773	0.0567381811884777	0.931538794783139	0.144213000000001	0.481986611994676
NT5E	-0.0862096783182323	0.809374369082773	-0.105315516825656	0.86011776595122	0.842687333333334	0.00711541332977091
SH2D1A	-0.086221175884682	0.809374369082773	-0.0475565123027407	0.943765177272388	-0.104678666666667	0.616036866881905
ZFP90	-0.0862652241162036	0.809374369082773	-0.0959978579073095	0.872311810447594	0.125116000000000	0.551125574415782
OPA1	-0.0862797824162731	0.809374369082773	0.173215810622176	0.736740209571778	0.0223013333333327	0.933248182961486
POLM	-0.0862868966803277	0.809374369082773	0.0717735862604132	0.90837606217316	-0.0360966666666654	0.89637298686676
PRSS58	-0.0863105957226086	0.809374369082773	-0.136033031861758	0.805641810268282	-0.0279869999999999	0.91148338318481
SRBD1	-0.0864281747561983	0.80920125871668	-0.0937642598381637	0.875355472414883	-0.0906326666666654	0.707538975864338
MTCH2	-0.086438524909656	0.80920125871668	-0.0543865704546992	0.9344692810234	-0.0154013333333322	0.958988235991247
ZNF22	-0.0864654999655142	0.80915911901445	-0.427703474894638	0.212110620400585	0.134174666666668	0.520846786113276
AMELY	-0.0864759750241443	0.80915911901445	0.0645080000002549	0.919527638281551	0.0480146666666671	0.839243391791351
ANKRD46	-0.0865429774974276	0.808793050580426	0.252650151837478	0.569339421777585	0.319112000000001	0.193322450377971
ZNF697	-0.08654558798064	0.808793050580426	-0.239390299078772	0.599361065356699	-0.151179666666665	0.623759859798594
BRF2	-0.0865468803837734	0.808793050580426	-0.138370129539103	0.799981150504262	-0.256329333333332	0.220405584107617
HRK	-0.0865592648866342	0.808793050580426	-0.117384319694794	0.839901243665883	-0.123679666666666	0.610847413222267
CYTL1	-0.0865824294308729	0.808793050580426	-0.291611001047683	0.485747109971932	-0.204057666666666	0.549585138287524
CDH9	-0.0866044904031268	0.808793050580426	0.0951756657362945	0.872962271153999	1.26559033333333	0.0763141016054713
SLC39A13	-0.0866090280599847	0.808793050580426	-0.244757751038356	0.587430330647728	0.099056	0.688145991467449
DSCR4	-0.0866721998478554	0.808642331082258	-0.198243037873612	0.687317734296043	-0.0825476666666665	0.709400163383053
DSCAML1	-0.0866991160623647	0.808560774352989	0.143913774595215	0.791730335952443	-0.101974999999999	0.769890867417328
IL33	-0.0867667105877414	0.808371663804036	-0.199721916233169	0.683035956626567	-0.197332000000000	0.338230698383994
CSF2RB	-0.086784934160368	0.808368491265843	-0.4318903470031	0.204884729507949	-0.140160999999999	0.73097308185803
FAM69A	-0.086805393215822	0.808327781694212	-0.230951869716444	0.616488238055513	-0.429933999999999	0.0425543345284076
UBE2V2	-0.0868436069121806	0.808217149395868	0.256309354719043	0.564430426783556	-0.0197023333333331	0.94678260229888
IMPDH1	-0.0869096001593915	0.808042304465408	0.176480923893635	0.730152680173573	-0.100272333333334	0.705698790800484
NIPA1	-0.0869460327010142	0.80789569839597	-0.414371461485222	0.237843761183458	-0.217602999999998	0.351839993215702
SNAPIN	-0.0869468897045212	0.80789569839597	-0.203894746538497	0.675615338672331	-0.0510363333333329	0.84207712482186
TIPIN	-0.0869731847580202	0.80785706730271	-0.418440092779140	0.231615997677004	0.0221246666666674	0.949376459031393
PTGER4	-0.086996865263997	0.807771972650153	-0.367811842317351	0.326542639565994	0.0525123333333333	0.85900837597792
SLCO1C1	-0.0870367403068408	0.80758145513663	-0.19702290831298	0.690005458891775	-0.180769000000000	0.351182087586052
ADPGK	-0.0871134806094452	0.807289069697	-0.164298989869798	0.754189116283047	0.0891396666666667	0.672674698802727
SLC16A8	-0.0871193801691786	0.807289069697	0.021572120863552	0.97536722461972	0.0169680000000002	0.95784037718285
CCDC117	-0.0871740094298131	0.807171258232073	-0.0397316760940186	0.953647040142798	0.223010666666668	0.270886793220924
CD3D	-0.0871856872774396	0.807164295689694	-0.222943414445301	0.635247978870818	-0.0873709999999997	0.714382809933119
MUC19	-0.0872578652672376	0.806763488326238	0.167799378895769	0.748242818061289	-0.0932683333333332	0.670113640649168
GLUL	-0.087270573983154	0.806763488326238	-0.188965688084431	0.706965444256208	-0.0869196666666655	0.69414649287771
LPAR3	-0.087274835576799	0.806763488326238	-0.0108079750456463	0.98774587825743	0.0151406666666667	0.95805797537369
ALDH4A1	-0.0872755058915642	0.806763488326238	0.0539282717657912	0.935365825918255	-0.046214666666667	0.849384482495249
LINC00302	-0.0872816448334003	0.806763488326238	-0.00844997202472681	0.989783000376216	0.00127933333333402	0.99704422037925
DNAH7	-0.0873084201785458	0.806763488326238	-0.146153817647665	0.787402233709509	-0.0944633333333333	0.65692454979738
TRRAP	-0.0873908795117094	0.806657206281744	-0.231960987724338	0.613445765196643	0.0239130000000006	0.946623914607177
ZBTB48	-0.087413941176561	0.806576121895493	-0.385773345316077	0.289470192870154	0.0385763333333343	0.897492693610486
SCRG1	-0.087445139499781	0.806452150558868	-0.0310485199648750	0.965118873711308	0.00919300000000038	0.976784701964987
CADM3	-0.0874547253218422	0.806452150558868	0.086229537684848	0.88794718020464	0.0536033333333335	0.81338029478666
ARHGAP29	-0.087462671136522	0.806452150558868	-0.289532946507248	0.488870852256136	0.905159666666667	0.00347244136732606
DHRS12	-0.0875171574035879	0.806249432680582	0.0630961812528969	0.921562696294372	-0.213071666666667	0.348000484523924
LINC00266-1	-0.0875241757108172	0.806249432680582	-0.370676270908593	0.320399684386315	0.101793666666668	0.793596726500656
MAST1	-0.087529690966901	0.806249432680582	-0.193832503085307	0.697189653813355	0.077795	0.728429595651334
C19orf40	-0.0875553004051138	0.806208325293202	-0.210701482603164	0.66164244512346	-0.0464339999999995	0.89052375260746
SCGB3A1	-0.0876077567643045	0.805935799070425	-0.0195642168363121	0.977673457110824	-0.166414333333333	0.552125911260862
HCCS	-0.0876989356561345	0.805549298643646	-0.307770869520674	0.452235381982992	0.189574666666667	0.320462604748547
ATP6V1H	-0.0877043646067077	0.805549298643646	0.0990103226806733	0.868042453226125	0.188696333333334	0.379431342074965
KRT13	-0.0877341198133728	0.805458342368195	-0.0121194977003655	0.985742962398217	-0.20126	0.432456927987147
NUDT16L1	-0.087775772879938	0.805355124014853	0.394188637585977	0.274555703975637	-0.0122289999999992	0.966813448118178
NRM	-0.087802263090764	0.80529091842868	0.152206591712898	0.775863712880333	0.294500555555556	0.156315769387234
PRSS50	-0.0878938558585915	0.804832631212486	-0.147170918492601	0.785691396687707	-0.133437666666666	0.504458932531644
ABCC11	-0.0879282918363528	0.804746531255782	-0.0022313323556649	0.996497835300076	-0.173313666666666	0.388303383752735
NKG7	-0.0879382636069093	0.804746531255782	-0.443406235787459	0.1843534401377	-0.206896666666666	0.501298037624658
SOX6	-0.0880027410663896	0.804468946930826	-0.102840507640829	0.86257932725575	0.0739386666666673	0.7996066102903
C1orf210	-0.0880734740826028	0.804353688223243	-0.0137746916282118	0.983872723592937	-0.133731333333333	0.565148387675909
GSC2	-0.0880930704577477	0.804353688223243	0.0332946434244467	0.960784250392482	-0.219375333333332	0.551846041283715
SPINT1	-0.0881210823194072	0.804250883542097	0.0601186487059037	0.925649366494154	-0.130297333333333	0.669391045681572
ABCB7	-0.0881523949882056	0.804140008682085	-0.491409907493666	0.123299386340344	0.0429080000000008	0.871104061464043
TOLLIP	-0.0881587957095517	0.804140008682085	-0.158540320392573	0.765993392549069	-0.214294999999998	0.305101612380486
C7orf50	-0.08818205091253	0.804140008682085	-0.190358852484707	0.704208582824765	-0.0188916666666654	0.946947729561688
CCDC47	-0.088187387035831	0.804140008682085	-0.238552634886969	0.601039183653478	-0.189596666666666	0.338158546666693
SLC10A7	-0.0881884695470644	0.804140008682085	-0.137652376053893	0.801934376886443	0.376852666666667	0.0979662489851624
FEZF2	-0.0882235979881006	0.804140008682085	0.0719546097027525	0.90837606217316	0.0912833333333335	0.729912257977231
MBP	-0.0882565555926794	0.804140008682085	-0.0955109056560426	0.872754579338908	-0.0891046666666662	0.772750225643389
SOX3	-0.0883180407455479	0.803935450843687	-0.221382864855866	0.63782368399216	-0.122051000000000	0.697902295371389
PRR18	-0.088353804154277	0.803858920477004	0.147777514907845	0.784459616761312	0.0435153333333334	0.911600329626215
ACAD9	-0.0883754909523519	0.803858920477004	-0.105292570480899	0.86011776595122	-0.245626999999998	0.23765426508992
TLR2	-0.0884706805780821	0.803588533511916	-0.317790622751607	0.43078084496581	0.122737333333333	0.600304644185307
UBE2M	-0.0884729169257243	0.803588533511916	-0.104596467026663	0.86067831050617	0.058817000000001	0.794075220327397
ZNF271	-0.0884871318300986	0.803588533511916	-0.0381189727680437	0.95468180561467	0.364739000000002	0.0832004986783026

RNF167	-0.0885088575837674	0.803588533511916	-0.0150399125531998	0.982921609770846	0.131647666666668	0.528409919089401
NRL	-0.0885379269314267	0.803470772732792	0.193566754622805	0.698014315401802	0.0063560000000009	0.987459024686908
UBE2T	-0.08862376348408	0.80310399936747	0.0557564098643107	0.93339378096251	0.0742123333333341	0.754439827606006
TNFSF8	-0.088650716188834	0.803012343650093	-0.337467051151008	0.386478628052631	-0.0187793333333326	0.963383328073994
LPAL2	-0.0888901495839911	0.80172877747387	0.453894049457397	0.171517363379933	0.0360813333333339	0.902194685260117
C1orf95	-0.0888901984710825	0.80172877747387	-0.0241836168059806	0.97420021343239	0.0759740000000012	0.786118160890663
FTCD	-0.0889504203700535	0.801474355021425	0.0620119506912974	0.923034515346352	-0.101173999999999	0.674492060174496
TMA16	-0.0890030672133763	0.801269591929494	0.0120559137828328	0.985893675157733	-0.158359999999999	0.456074677719062
PHLDB2	-0.0890237529915966	0.801203929189576	-0.305051243328410	0.458198595388891	-0.272759666666665	0.653123035053983
FAM127A	-0.0890647334643116	0.801075149283312	-0.281477307496069	0.506335763951991	0.143741	0.503308350574223
ACTL7B	-0.089065238395509	0.801075149283312	0.288670731115964	0.489813374112312	-0.134039999999999	0.680305534766464
PGBD1	-0.0890711472184806	0.801075149283312	-0.205700454213563	0.672625944768829	0.143826000000001	0.477328898122926
JRK	-0.089084057266851	0.801075149283312	0.324892934508889	0.410909153213675	-0.0805863333333329	0.761078552848883
ADIPOR1	-0.089136053568086	0.800955977776706	0.0215360237459209	0.97536722461972	0.151065000000001	0.449436921437102
PPIL6	-0.0892029156448167	0.800777566774382	0.172926438193862	0.73765042590756	0.448531000000001	0.0335464404361495
HTRA2	-0.0892441028806864	0.800691768667511	0.0155842370981725	0.982442849983933	-0.0237213333333325	0.930180576815812
VPS52	-0.0892562175950039	0.800691768667511	-0.0217825884929391	0.97536722461972	-0.0076405555555548	0.981582681132884
PBX2	-0.0892701798468081	0.800691768667511	-0.0791271111920049	0.898830040032591	-0.068358999999999	0.79082065189004
CDKN3	-0.089281646021496	0.800691768667511	0.0223530708511094	0.97536722461972	0.317178666666668	0.153036603279840
GGCX	-0.0893259735043399	0.80061700422549	-0.108566588835883	0.85395367181085	-0.0408376666666661	0.889610038918956
ACRC	-0.0893516336216998	0.800518917170585	-0.0641854231196546	0.919923865590619	-0.343565333333333	0.0777324007395152
LRRC31	-0.0893559451877675	0.800518917170585	0.0701057713683934	0.910580282989425	-0.0216753333333332	0.945904449997854
AHCYL1	-0.0894103177808862	0.80044363245692	-0.219100172021619	0.641699006980579	0.110460000000001	0.583699608015136
ATG16L1	-0.089411429581997	0.80044363245692	0.242528101077296	0.593306823292819	0.194278666666667	0.343632393207337
LINC00305	-0.0894561551452217	0.800390900892933	-0.0140989603424369	0.983444353656142	-0.0687206666666665	0.780732284998362
CDC26	-0.0894722044179335	0.800355452919704	0.0243705912330492	0.973772197470206	-0.107488166666666	0.649130382513952
CLEC14A	-0.089475104528479	0.800355452919704	-0.0708207308538657	0.909760747616489	-0.0173779999999992	0.949314722489396
MIA2	-0.0894893525034208	0.800355452919704	0.273022941060936	0.524713596820663	-0.615754666666666	0.0466349386848077
COG3	-0.0895535330451776	0.800101979835518	0.0106567358843762	0.98774587825743	-0.0152316666666656	0.962334965334496
PABPC3	-0.0895637499350264	0.800101979835518	0.226661815710274	0.626670684247721	0.042373333333334	0.870002591217953
SIGLEC16	-0.0895816643301855	0.800056946961457	0.0383410615620892	0.954545200586067	-0.00469299999999951	0.988133186272453
GUSBP11	-0.0895846736477117	0.800056946961457	-0.128571461817763	0.815827854535765	0.0672581666666670	0.763935841204439
FMO2	-0.0896232172961938	0.800012953343996	0.000579926697875800	0.999231019369277	-0.0363923333333331	0.905362528819556
ABI3	-0.0896627616661038	0.799874165661019	-0.253509807231327	0.567705016149998	-0.0289419999999995	0.910605164649817
TMEM198B	-0.0896665022345436	0.799874165661019	-0.341583725010465	0.377673659904403	0.213258666666668	0.297888842945994
ELAVL4	-0.0897053256128059	0.79976518074109	-0.259549043149001	0.556680762840168	0.012904333333334	0.965930252534257
CTSO	-0.0897329082768697	0.799713712825645	-0.100377737271917	0.866570192459381	0.0647976666666675	0.831360434244133
DLEU7	-0.0897505497721994	0.799713712825645	0.0267097685709203	0.970207612777188	-0.179054333333333	0.447498161237612
MSRB2	-0.0897719505681892	0.799646636960362	0.412187404199558	0.242862788007169	0.292302333333334	0.167461485379690
C7orf57	-0.0897742170325857	0.799646636960362	-0.331062996632543	0.401413989019412	-0.0396383333333324	0.902550440604425
KBTBD2	-0.08977497324941	0.799646636960362	-0.384600479894492	0.291180853572672	-0.217016	0.271872170281604
ARHGAP44	-0.0898553660344742	0.79950636821062	-0.052526897779865	0.937709096743257	0.0391176666666662	0.881409122959208
RNF180	-0.0898668549355329	0.79950636821062	-0.111271125473535	0.849115053536469	0.305821666666667	0.409270914061388
RASGEF1C	-0.0898701786892965	0.79950636821062	-0.0173415714599967	0.980670285500112	-0.0462576666666657	0.87290879338693
C17orf105	-0.0898934377664924	0.799477417860285	0.129772791567899	0.813305249846504	0.0308233333333338	0.96078716202891
PDE1A	-0.089900147423991	0.799477417860285	-0.0890136497176794	0.883824851493414	-0.0196513333333327	0.945080050179073
HES4	-0.0899329577852096	0.799358133395002	-0.322277087908236	0.417538034812104	-0.153410666666666	0.608311546956177
ACVR2B	-0.089980415354212	0.799187066231524	-0.210523648353245	0.661670522548494	0.291558666666667	0.352237134604623
RNF151	-0.0900153805323877	0.799097491686686	0.167101926641658	0.749664019651347	-0.155668000000001	0.604244022194318
ZC3H12D	-0.090046433754151	0.798964174429514	-0.236744604003458	0.604321362942628	-0.110598999999999	0.696194439936211
SULT1A1	-0.0900590192812977	0.798951332315513	0.213432149918181	0.654550434157114	-0.0098143333333324	0.981210370531448
ZNF480	-0.090105938403711	0.798783770603078	-0.408985972694561	0.249852262368516	0.116441000000001	0.618401074037011
TFEC	-0.0901179556799837	0.79877746358028	-0.396379950012002	0.271136524502442	-0.0352633333333333	0.887760071161783
LINC00526	-0.0901736270688643	0.798480720392787	0.0688604200291119	0.912149839732916	0.0796753333333337	0.716268843468132
MAN2B2	-0.0901801199786048	0.798480720392787	-0.539262212291332	0.0705285823494608	0.157415000000001	0.441460337585051
LINC00161	-0.0902059768049982	0.798480720392787	0.123708782967803	0.826475556843795	0.114621333333333	0.676024903789639
HEATR5B	-0.0902278158909941	0.798473509962728	0.0532497628331186	0.936629055455007	0.113952000000001	0.614744265452795
HGF	-0.0902333372597172	0.798473509962728	-0.406569736361423	0.253947807177628	0.0179750000000000	0.953929177066653
CD86	-0.0902550874881129	0.798434130535544	-0.318476964301427	0.4290520704081	0.199872000000000	0.423030362322111
HPCAL4	-0.0903157314401486	0.798177003487506	0.213189199734475	0.655051855533403	0.0517963333333337	0.819449110075885
GP5	-0.0903734281341467	0.798008380976633	0.0342431457862482	0.959623239761825	-0.165072666666666	0.493413216077427
TMEM230	-0.0903810033941772	0.798008380976633	-0.193899399257585	0.697189653813355	0.103140333333335	0.617967324583825
KLF1	-0.0903834024645173	0.798008380976633	0.145504935930987	0.787804113309913	-0.131687666666666	0.641044966033937
PHYHD1	-0.0903960265993457	0.798008380976633	-0.261043831307899	0.552796181718632	0.195844333333334	0.514889931507188
PREP	-0.0904039173946895	0.798008380976633	0.0678837035864925	0.913207931893897	-0.0933883333333325	0.718749100405876
PAK3	-0.0904132934735752	0.798008380976633	0.114970624782432	0.844162087612813	-0.105532666666666	0.778995157976597
FCGR2B	-0.0904194453433253	0.798008380976633	-0.338867389037767	0.383664784230327	-0.200686499999999	0.321128515998283
ATP5H	-0.0904445726134934	0.798008380976633	0.398223817023953	0.268367555143542	0.0299020000000008	0.910190419163007
MAGEA8	-0.0904492318979982	0.798008380976633	0.028939263490209	0.96782691865619	-0.0795736666666664	0.742738366366583
KRT2	-0.0905094543146387	0.798008380976633	0.0101841508327323	0.988384322540392	0.039374	0.880347900939311
DTWD1	-0.0905120124602202	0.798008380976633	-0.0282137872274435	0.968668367660302	0.204400333333334	0.471989789810989
IL2RG	-0.0905439407091091	0.798004636535099	-0.312369039238932	0.440033473850785	-0.0630633333333333	0.813694870801638
NAPEPLD	-0.0905613642259005	0.797960244029958	0.389592653802193	0.282991634599374	0.475113333333334	0.0351167468854759
TMEM5	-0.0905796280883654	0.79793464740021	-0.173976027995048	0.734793595952544	0.0633986666666662	0.824674571093737
ENTHD1	-0.0906161006449755	0.797860656978609	0.0353201185133412	0.95869292666698	0.00332333333333369	0.993724132855888
LYST	-0.0906611539764791	0.797860656978609	-0.370410100391320	0.320801726666339	0.281658333333334	0.190366446478058
LRRIQ1	-0.0906890417379237	0.797820408712173	-0.041178675707093	0.952098348256047	0.120619333333333	0.606466210369769
G6PC2	-0.0906907265271627	0.797820408712173	0.0506734035298706	0.940271086498237	-0.0213840000000000	0.941559580750404
RASAL1	-0.0906916486528446	0.797820408712173	0.0100372880544940	0.9884787454138	-0.191571666666667	0.309421300309741
MAK16	-0.0907081597980034	0.797820408712173	-0.222930225347326	0.635247978870818	-0.0980126666666666	0.682978179404224

JSRP1	-0.0907485409931171	0.797820408712173	0.349716049364246	0.359027546043591	-0.0647299999999989	0.80758784150395
MPZL2	-0.0908581949291297	0.797409752738972	-0.32480730370285	0.411071217323391	-0.111989666666666	0.724001791100896
ARF4	-0.090873690061928	0.797377967039553	-0.253518744751123	0.567705016149998	0.0174100000000012	0.949376459031393
GNG8	-0.0908985674653598	0.797313465351559	0.182351798316599	0.719419078472232	-0.215016000000000	0.446756640099031
TMEM99	-0.0909053269475637	0.797313465351559	0.317776544493972	0.43078084496581	0.200572000000001	0.344576368488222
DHFRL1	-0.090966109108973	0.797260349838267	-0.0485868077956307	0.94236319435931	0.0279219999999998	0.921118351689873
IL27	-0.0910340445244639	0.797094323218338	0.241592859669943	0.595261877490935	-0.0610849999999989	0.790646397068178
AES	-0.0910370705243874	0.797094323218338	0.239109578319408	0.599801655225887	-0.117172999999999	0.645219822507582
IL1F10	-0.0910897753838614	0.796938568103909	-0.0186330840758472	0.97902968742576	0.00092100000000081	0.99792238320329
MARCH4	-0.0911270397693213	0.79676451314694	0.00774198243041179	0.990405674923721	-0.171472333333333	0.4637142212394
BCL7C	-0.091134133980407	0.79676451314694	-0.251522444751399	0.571758327024774	-0.0355983333333326	0.895093529187836
FMO3	-0.0911750085574138	0.796589990864977	-0.210560718687514	0.661670522548494	0.0731310000000003	0.835943848903295
ZSCAN22	-0.091195131799423	0.796527963373417	-0.128824134939938	0.815407702581246	-0.0651993333333321	0.767971007443344
FBXO39	-0.0912673205613647	0.796195160002593	-0.0764661414808429	0.902679569009162	-0.0921906666666663	0.688145991467449
PTDSS2	-0.0913105435321001	0.795982185140874	0.0442380634427707	0.948449358182873	-0.0528066666666658	0.838603547123369
NGEF	-0.0913315993635863	0.795914059750297	-0.0315769016085382	0.9638977610518	0.462329333333334	0.0574767229252545
DERL1	-0.0914307846050443	0.795420953982236	0.237880933123459	0.602400332700316	-0.073985666666665	0.76648935876098
MOS	-0.0914522080862733	0.79540381781475	0.0140668111976435	0.983444353656142	-0.146955333333334	0.614081498359193
FHAD1	-0.0915033743482883	0.795138978026973	0.0690943675255252	0.911928203725685	0.045073000000001	0.862509125961694
GATC	-0.091508818304181	0.795138978026973	-0.286906797064381	0.494604746861742	-0.0265133333333327	0.915761046438965
C2orf42	-0.0915540468803803	0.795085649647918	-0.083577706353505	0.892833228561937	0.0723936666666676	0.7703486664151
CHMP4C	-0.0915756697655424	0.795013814447858	-0.25098463991672	0.572882168047464	0.0202983333333336	0.944218475609619
HTRA4	-0.0915782439073636	0.795013814447858	-0.124366478412685	0.825255414308211	-0.0127753333333332	0.980770032498656
STRC	-0.0915875866548983	0.795013814447858	0.0102497626349717	0.98817928459664	-0.0608619999999995	0.855321176509099
SLC30A8	-0.091610575478832	0.794994084337636	-0.0756987878497805	0.903510413986113	0.0382046666666669	0.903864363916236
AGR3	-0.0917780691084972	0.794246933303053	-0.0384488103880404	0.954545200586067	-0.176222333333333	0.361908968091749
CISD1	-0.0917794044415129	0.794246933303053	-0.125500741574676	0.823755601962118	0.0381656666666668	0.876804123149866
LCT	-0.0917800611671965	0.794246933303053	0.0889386970729314	0.883865906764221	0.0359010000000004	0.902558514067126
TTTY5	-0.091794045631814	0.794246933303053	0.0621816356022288	0.92301259447339	0.161655666666667	0.469591696339734
DPP6	-0.0918085264431503	0.794246933303053	-0.068329184941812	0.912364243071055	0.481046000000001	0.321718549232674
BTBD9	-0.091920022211837	0.79387493920527	-0.289265857292922	0.488889656845544	-0.217471999999999	0.292442659257650
TRAPPC9	-0.0919521666388939	0.793734353145514	0.170146228045599	0.74270446821332	-0.0684856666666667	0.760951989611575
NCBP2	-0.0919722752689715	0.793734353145514	-0.0295829447087035	0.967205787110068	-0.241304	0.243957990539057
PMS2P3	-0.0920083123639326	0.793620637754673	-0.214852326977265	0.651655315777885	-0.133089666666666	0.610847413222267
ADAMTS10	-0.0921787462471124	0.792948284577759	0.140434427125032	0.797372514376909	0.0520973333333341	0.843134844119548
TGM3	-0.09223736557373	0.792634746456433	-0.134377034870031	0.808009477165608	0.0723426666666668	0.800230313083876
PCDH7	-0.0922665686070685	0.792513383935407	-0.182248956438892	0.719419078472232	0.00257533333333427	0.993801264598204
APOC1	-0.0922744127696426	0.792513383935407	-0.0768974935847441	0.901846292119635	0.123450666666667	0.580796638239637
GAR1	-0.0923357707113777	0.792477770214262	-0.513091326913619	0.0967475930522153	-0.239402333333332	0.341727195150614
PIK3CG	-0.0924678452901666	0.792144444364472	-0.436446598671488	0.195288910691951	-0.142652666666666	0.503352066997863
SLC30A9	-0.0924709226562523	0.792144444364472	-0.322254735283667	0.417538034812104	0.0903033333333345	0.670719950296173
GPR78	-0.0925220918012395	0.792070104354506	0.330070485515118	0.403195021794343	-0.0311049999999993	0.904236796123256
KDM6A	-0.092526969726521	0.792070104354506	-0.179861074473763	0.723716289903531	0.210124333333335	0.277855860891285
AMZ2P1	-0.0925410988855145	0.792070104354506	-0.116110200906915	0.842613538353187	0.296642	0.178162638616424
FBXO3	-0.0925561201720207	0.792070104354506	0.236066693817539	0.60601651443518	-0.0865736666666654	0.729912257977231
AMIGO1	-0.092701944699916	0.791821343662755	-0.349334592552717	0.359794558968334	-0.073021666666666	0.775273488714107
PKIA	-0.0927647563031272	0.791549703447851	-0.0491024782605631	0.942117943885137	0.300751000000001	0.501234066666842
HSPA6	-0.092767703210136	0.791549703447851	-0.179892303852789	0.723716289903531	-0.135718833333332	0.587396808178803
NPHP4	-0.0927928610933402	0.791532391476019	-0.0527032244940521	0.937357975161644	0.0324766666666665	0.903352632801889
PCDH1	-0.0928434429824111	0.791313301303332	0.00902562558712324	0.98909166696601	-0.0571679999999995	0.809755698910303
SYNPR	-0.0928836850544289	0.791193007798194	0.110272365661086	0.8502509043242	0.0559856666666668	0.845045439210304
RFPL2	-0.092889261263779	0.791193007798194	0.00783040273881152	0.990405674923721	0.0372133333333339	0.890073383340154
IMPACT	-0.0928922107475187	0.791193007798194	-0.0351855816817223	0.958873757674185	0.180537666666669	0.344576368488222
NSUN5	-0.0929000307083378	0.791193007798194	-0.361170691088928	0.339627732901974	-0.193033333333331	0.324443655582244
PTPN6	-0.0929221717097005	0.791193007798194	-0.252989560465509	0.568985221714455	-0.125612333333333	0.546219696447415
VPS25	-0.0929314374591199	0.791193007798194	-0.234175486899943	0.608813903134825	0.110719333333334	0.613915242226352
HS3ST2	-0.0929457856184855	0.791193007798194	-0.0109727246912824	0.98774001793194	-0.120121333333333	0.657217907037306
ATP2B4	-0.0930251943835209	0.79100841759749	-0.0308161464742396	0.96529904314994	-0.0145810000000002	0.970210792906847
KXD1	-0.0930412768953162	0.790993177595791	0.208874207555407	0.665595909869988	-0.0676559999999989	0.76307213318662
SMTNL2	-0.0930720800079408	0.790861267771643	0.353429390310173	0.352407344218756	-0.0568729999999995	0.844111424193041
CYBB	-0.0930957927610637	0.790845309412949	-0.464902381463296	0.155837099170663	-0.0113356666666662	0.974484855799046
OFD1	-0.0931107807957852	0.790816847713267	-0.174443953364420	0.734267769889068	0.0432726666666668	0.868203945159707
KIAA0087	-0.0931367917980237	0.790785866355938	0.103125182652685	0.862401456058756	-0.057132	0.872930585008323
FXR2	-0.0931976216024663	0.79065761044567	0.112070962381003	0.847483801046528	-0.287289333333333	0.142151034574214
METRN	-0.0932137927593166	0.790630101157522	-0.140846169975807	0.796714214272984	0.0307150000000013	0.90945379489823
LGI4	-0.0932420823483635	0.790603450077757	-0.0692639092935215	0.911837389834658	0.0466330000000003	0.888476605628154
ARL11	-0.0932517462039435	0.790603450077757	-0.294353041879782	0.479822264798778	-0.0919583333333322	0.665071722126052
TAC1	-0.0932873972532802	0.790496581251142	-0.0509339481010621	0.939677282439105	0.0759823333333336	0.74238434992823
PRMT3	-0.0932980114778064	0.790496581251142	-0.137415343038542	0.802044965635484	-0.081352666666666	0.76653306515103
TAL1	-0.0932997708460023	0.790496581251142	0.0511150268676145	0.939355624240953	-0.115525333333333	0.681151028675655
SPOCD1	-0.0933129616874211	0.790496581251142	-0.120297582926327	0.834809734605012	0.0166896666666669	0.964995783450258
MOSPD3	-0.093329015017696	0.790496581251142	0.0574923787123399	0.93030634693695	-0.101003666666666	0.622282705325595
MPST	-0.0933544689473644	0.790496581251142	0.358134550005936	0.343395848463795	0.0201036666666666	0.943391353769848
HARS	-0.0933699839014777	0.790496581251142	-0.17464360697477	0.734176810345161	-0.414856999999999	0.0431903372471131
TRH	-0.0933838081314806	0.790496581251142	0.253928633349513	0.567694846153007	-0.0801393333333328	0.794622958123968
MGC27345	-0.0933863348763834	0.790496581251142	0.0168282488294692	0.981160061237418	-0.0885503333333326	0.777406710539424
LALBA	-0.0934256598085158	0.790496581251142	0.0650955704022641	0.918489100363712	-0.262532333333333	0.274121181062751
FOXC1	-0.0934324224992224	0.790496581251142	0.033381929739026	0.960784250392482	-0.0120313333333322	0.971293288800343
SPACA3	-0.093498184283188	0.79023151498941	0.33124433358804	0.400898197523962	-0.0798930000000001	0.730907166061737
CAMK2A	-0.0935527406857621	0.789944029333058	-0.0329551180961032	0.961277493638149	0.0303690000000004	0.911715211050394

SERPINE1	-0.093565725585063	0.789928708520605	0.0979728111715796	0.868852648210739	0.416823333333335	0.0620787936501218
PFDN5	-0.0936033071368414	0.789822049036702	-0.0190602262893876	0.978322425527852	0.0513053333333335	0.833772578631961
PTPN1	-0.0937135055817287	0.789240030126654	-0.310443253540831	0.445358686230635	0.153408666666668	0.442294185443201
LARS	-0.0937279384201316	0.789240030126654	-0.478210807887187	0.14073566711294	-0.142511333333332	0.550660741372463
SLC38A1	-0.0937799121347455	0.789084004879525	-0.202831504045730	0.677604697677494	-0.0486853333333319	0.88629962370345
SLC7A3	-0.0938278331175512	0.788865367608489	-0.313932177166735	0.438046311563112	0.00187033333333342	0.99529569362957
RSAD2	-0.0938790687175368	0.788643961669951	-0.217105491309420	0.646717655108075	0.332104000000001	0.159075946945580
UBE2I	-0.0939713435100432	0.78818148835494	-0.106511101331828	0.858072018696671	-0.0910746666666662	0.667040000089664
LIMD1-AS1	-0.0940065437878398	0.78818148835494	-0.0245849354902232	0.973469173381696	0.475993000000001	0.232897341415308
TBC1D2	-0.0940238487829174	0.78818148835494	-0.000155712607557776	0.999860038008296	-0.160281000000000	0.410917713955087
SMARCE1	-0.0940381720037063	0.78815980736685	-0.0715791623673625	0.908733931243345	0.111088833333334	0.639002829892966
FABP4	-0.094062902759536	0.78806759026872	0.185530270466186	0.714210278087368	-0.0677799999999997	0.764973469318093
SNX18	-0.0940781517945537	0.78806759026872	-0.5981615272354	0.0337939911292242	-0.0466963333333324	0.85805372104488
B4GALNT4	-0.0940852079626854	0.78806759026872	0.118076521785799	0.838500126709059	-0.317533666666666	0.188549331106419
TRIM52	-0.0941095845903944	0.78806759026872	-0.181359821675003	0.721174140548176	-0.202284500000000	0.355387672697857
IL1RL2	-0.0941136538722342	0.78806759026872	0.0654667292965039	0.918111672450639	0.0125603333333338	0.969387309763954
FOXK1	-0.0941329079602949	0.78806759026872	0.222267099290646	0.636309362271551	0.0459909999999996	0.85013576481729
MKNK2	-0.0941534166084129	0.78806759026872	0.0297878527049658	0.96695070705759	0.299263000000002	0.116856969510577
LCP1	-0.0941796002079738	0.78806759026872	-0.337931654522447	0.385303753646555	0.173822333333335	0.399012880214421
SLC1A4	-0.0941853947280892	0.78806759026872	-0.373117803231579	0.316692858754573	-0.0564200000000001	0.855853956437739
TCEANC2	-0.0941977416949854	0.78806759026872	-0.124374918763864	0.825255414308211	0.259171000000001	0.171804245444023
OR7E14P	-0.0942384585819792	0.787963555361071	0.0293325012570716	0.967655960277738	-0.0224646666666665	0.944218475609619
ZPBP2	-0.0943286109520686	0.787442933944443	-0.0815183460556722	0.896097064649906	-0.0139346666666663	0.96681311917387
C19orf45	-0.0943581162522671	0.787319447516422	-0.150974152849975	0.778205892965504	0.0174296666666669	0.962620058410129
KLRD1	-0.0944028528943011	0.787096248722794	-0.415733263476374	0.235287899587884	0.108452333333334	0.60378773986659
TMEM150A	-0.0944191735023203	0.787059080515631	0.0619375388880686	0.923034515346352	-0.00175266666666648	0.99597023247862
ADH1A	-0.094479481116181	0.786733969020801	-0.0910474635037297	0.881053992915981	0.0203143333333337	0.96913732998656
EFCAB1	-0.0944850502939007	0.786733969020801	0.226985254207337	0.625822145497039	-0.213670666666666	0.304850986720083
CLCN7	-0.0945210773773405	0.786601006057697	0.0290995752543927	0.96782691865619	-0.153889000000000	0.443072320689246
CHMP7	-0.0945571220684915	0.786482055242189	0.180719833087668	0.722213862845507	0.0139046666666681	0.962275697500938
TRAPPC2	-0.0945899003688455	0.786359481731675	-0.181436469931379	0.721174140548176	-0.326042999999999	0.317651195904626
LSMD1	-0.0946283378400742	0.786247192882865	0.0682599814735275	0.912364243071055	0.0110193333333341	0.97184501043952
SNTB2	-0.0946802616719419	0.785976986838169	-0.132294100334119	0.810300414153187	0.193953000000002	0.351907021382432
WHAMM	-0.094776285315831	0.785557453798747	-0.143584111429414	0.792591521987966	0.244630666666667	0.256673063330227
HRASLS5	-0.094830433963299	0.78549951377478	-0.107116633756897	0.8576027597496	-0.0525599999999995	0.866601971778042
ASGR2	-0.094875414028731	0.785326474151847	-0.0271990161699572	0.970207612777188	-0.100536666666666	0.707538975864338
STAU2	-0.0948981506826117	0.785247302220465	-0.316630757858927	0.432178413930141	0.109454	0.66132749915999
SLC45A3	-0.0949112679753008	0.785247302220465	-0.116854512675809	0.841562044027187	0.0867269999999998	0.754702091676029
ZIM3	-0.0951211057191213	0.78427562338021	0.229211070606240	0.620501848247781	0.00262500000000014	0.99529569362957
SHANK2	-0.0951396792517131	0.784223736773649	0.509607512531181	0.101208857808966	-0.0170670000000000	0.961265941014135
OXA1L	-0.09514455357185	0.784223736773649	0.170366175086458	0.742671224116098	-0.188455999999999	0.379613779505161
AIMP1	-0.0952681177952571	0.783731300203463	-0.127024287778053	0.820257254890606	-0.116975666666667	0.592006286402195
C17orf74	-0.095277592249672	0.783731300203463	-0.162561900943826	0.757614806491095	0.0224206666666675	0.935261323444314
SLC39A7	-0.0953097631656355	0.783727941886156	-0.255378275424511	0.566022340784541	-0.805214666666667	0.0098546599387472
CRYZL1	-0.0953239271330908	0.783727941886156	-0.039071091965461	0.95397534100479	0.0499223333333339	0.833065801030836
ZNF444	-0.0954310220865193	0.783430960200182	0.400590840940803	0.262685489461853	-0.0453626666666666	0.855321176509099
CD209	-0.0954803718331271	0.783247249066944	-0.129903836817504	0.813020408185924	-0.0728084999999989	0.836925962343033
PRDM10	-0.0954919223463173	0.783241278772585	-0.199941185830312	0.682474360043197	-0.150965333333332	0.442668180230308
CECR5-AS1	-0.0955125347029458	0.783179138260545	0.188528107748816	0.707361603786947	-0.123478666666666	0.564757468001238
CRYBB2P1	-0.0956145828280917	0.782717050577124	0.033006562851527	0.961277493638149	-0.0975966666666662	0.641044966033937
LOC400940	-0.0956208058170978	0.782717050577124	0.282184100962311	0.504065779877366	-0.0256423333333331	0.923019543204612
DDX19B	-0.095634916506677	0.782717050577124	0.282972738074764	0.502129505146473	-0.0644089999999987	0.795446186721078
CCDC87	-0.0957063373202715	0.782362439326206	-0.201589253885281	0.680054545720343	0.0399123333333338	0.899614658619676
RPUSD4	-0.0957109964242603	0.782362439326206	0.000719589687496990	0.998964123154806	-0.099401666666666	0.635069698575895
CSN3	-0.0957203065011665	0.782362439326206	-0.107308544614423	0.857128584932911	-0.196612333333333	0.319130307138698
FA2H	-0.095722877084685	0.782362439326206	-0.0573528583693313	0.930490408235626	-0.117908	0.625510328921698
NKAPP1	-0.09573694125139	0.782362439326206	-0.128392081885930	0.816345918941366	0.0858703333333333	0.688145991467449
GTPBP2	-0.0957845109036918	0.782231553914146	0.0808456648620207	0.896361013063453	0.159583666666669	0.469591696339734
GADD45B	-0.0958158018235719	0.782231553914146	-0.215706827842662	0.649360323329869	0.140105000000000	0.546219696447415
PADI3	-0.095827340742658	0.782229750111769	-0.0651006955891003	0.918489100363712	-0.119007000000000	0.557611184484231
C1QC	-0.0958475893789871	0.782166857056103	-0.46210850726892	0.159516378927220	0.0485156666666667	0.907374738017166
FBXO30	-0.0958558017288704	0.782166857056103	-0.046485311157658	0.944839700668385	-0.0745319999999987	0.742452599762094
TTC16	-0.095874509105446	0.782129941326668	0.086281468024505	0.88794718020464	-0.161915333333333	0.415770907577367
ERAP1	-0.0959650805313317	0.781678403700881	-0.335103157798335	0.392042077410332	0.399962000000000	0.0688646074782801
CD3G	-0.0959799751089315	0.781678403700881	-0.160516719827097	0.761639889788631	0.0487173333333337	0.861690060758414
PSG11	-0.0960363451073118	0.781418902408474	0.0211839681266803	0.975853964465421	-0.131867333333333	0.635279488727338
CDCA4	-0.0960488774518806	0.781418902408474	-0.236409922419906	0.60511225037033	-0.202571666666666	0.39576473605983
LOC375196	-0.0960930035997294	0.781396228603349	-0.0504373900640804	0.94067156299105	-0.116345333333332	0.76307213318662
ALKBH6	-0.0961067681226862	0.781375777391933	0.23827615522989	0.601773482805025	-0.0192139999999994	0.944097469332832
PHOX2A	-0.0961070603728038	0.781375777391933	0.2193202103503	0.641314646479258	-0.102289666666666	0.68400971768044
TADA1	-0.0961530233127752	0.781281953046957	0.0703063212421536	0.910394157189308	0.0941643333333343	0.649977903605799
SYNPO2L	-0.0961658455891156	0.781267675219179	-0.168534951405675	0.746758286599055	-0.108116666666667	0.690213956173797
RAB5B	-0.0961711835302248	0.781267675219179	0.134537343719875	0.808009477165608	0.125594000000000	0.60378773986659
ANGPT1	-0.0962393784591876	0.78106495305633	-0.479898785456392	0.138290570952161	0.142524	0.49536461519745
C22orf34	-0.0963494355511356	0.780623126871804	0.138673864365363	0.79914918957102	-0.105965999999999	0.669225752878437
LOC284837	-0.096432799164066	0.780286395320596	0.363752844420459	0.335033160801809	-0.0656609999999999	0.808149567980568
ATP13A2	-0.0964863522418793	0.780062162009313	-0.280100694823464	0.508074118826263	0.0328600000000008	0.904788907158467
LCN10	-0.0964890264527321	0.780062162009313	-0.0420847994376555	0.951989245458556	-0.180536666666666	0.622346194806715
FBXO36	-0.0966314536412866	0.779543259464612	0.442425740903693	0.185282268884974	0.176440333333334	0.484583000340636
ZFYVE26	-0.0966725321640863	0.779344043716784	-0.241410482588962	0.595802238885157	-0.0573113333333325	0.807453618390844

LRRC8C	-0.0966758383301145	0.779344043716784	-0.0954927206134353	0.872754579338908	0.679126000000000	0.0347429504699193
ABTB2	-0.0967001233894265	0.779344043716784	-0.00367861030664203	0.994588964700772	0.132196333333334	0.523041310312964
CRYBG3	-0.0967185209721433	0.779344043716784	-0.288398953250487	0.490238022687012	0.251326333333335	0.195820572246296
PA2G4	-0.0967350099995807	0.779344043716784	0.135970618018030	0.805852580790973	-0.151826666666666	0.441366313859876
CPLX3	-0.0968109730436402	0.779134289550355	0.270111619965900	0.530185035699403	-0.0733799999999995	0.86045090010169
DEFB127	-0.0968581093353665	0.778965042257724	-0.0295534629647085	0.967226229119616	-0.0464406666666669	0.85994094150432
GPC2	-0.0969256520094404	0.778731869259705	0.1934508972306	0.698131592467487	-0.0281976666666655	0.914418029309221
SIGIRR	-0.0969412396482642	0.778699486935115	-0.129342485616596	0.814410035678712	-0.191244333333333	0.518836392563847
VAMP1	-0.097031080982767	0.778390000770194	-0.277338760893951	0.5144731963081	0.151021000000000	0.441460337585051
LRRC7	-0.0970415322945267	0.778390000770194	-0.0761009054586283	0.903296922694955	0.0137386666666669	0.96622567326855
LRP10	-0.0970587577485827	0.778390000770194	-0.0983130289407887	0.86848759013952	0.0210483333333346	0.955701951169976
CASK	-0.097064408730937	0.778390000770194	0.0687315297481335	0.912149839732916	0.296930000000001	0.156312939631849
HMOX1	-0.0970759315176944	0.778390000770194	-0.114728104549462	0.844162087612813	-0.0497326666666666	0.844766787725698
VTA1	-0.097146121749356	0.77812453881578	0.0134506453383158	0.98412883278237	0.130301666666667	0.535171605667144
CYBA	-0.0971640896107468	0.77812453881578	-0.34662996756691	0.365167159367406	-0.127546	0.726963562600218
ZNHIT2	-0.0971762553823754	0.77812453881578	0.0661036239783386	0.916792605561869	-0.222304	0.234366351979507
PPP3R2	-0.0972227697642536	0.778060012411121	-0.0451047171275335	0.94659867551232	0.0486796666666666	0.842903889380894
ACP1	-0.0972506969124705	0.778060012411121	0.193660438759966	0.69772764496956	-0.0776949999999994	0.716268843468132
THBS4	-0.0972611417395798	0.778060012411121	0.234491201743040	0.608446568003374	-0.0404726666666664	0.879268366993072
RBPJ	-0.0973535706943556	0.777741936737473	-0.166535952841013	0.750627442079791	0.0273075000000007	0.91402300740264
SCIMP	-0.097403491243543	0.777554441388316	-0.0897972247076953	0.883178696174202	-0.00265533333333331	0.993756838831997
CSH2	-0.09744868773666	0.777432656418967	0.210639371367786	0.661670522548494	-0.0871213333333321	0.883189085233712
CRABP1	-0.0974663429545093	0.777421711511432	0.200314784044848	0.681169882699841	-0.0470573333333337	0.893856182973485
CHTF8	-0.0974869160606763	0.777421711511432	-0.437605779391193	0.194173491891936	0.138464666666668	0.550904785888199
CERS5	-0.0975056476692744	0.777421711511432	0.0318407516313552	0.963211116393051	0.0586693333333337	0.802934878255011
MAP3K10	-0.0975419381418899	0.777275270993496	0.136564103165464	0.804337959832803	0.141484666666667	0.503217861354116
EXOC4	-0.0977306475220875	0.776527402606294	-0.084349197241382	0.892381223757468	0.0454563333333349	0.847985525870412
ZFP42	-0.097730785395621	0.776527402606294	-0.322512003222977	0.417333080386525	-0.222607666666666	0.337307065334106
FOXR1	-0.0977318973356104	0.776527402606294	0.0561671821711227	0.932557112093148	-0.231375666666665	0.286257286390700
SLC22A6	-0.0978407581281102	0.776154884552076	0.21843426782049	0.642707776478228	-0.112809666666667	0.664617185770066
TNIP2	-0.0978874612550043	0.776058197058579	-0.502728506845842	0.109981880394312	-0.193398333333332	0.41785973815832
CST7	-0.0978925152827195	0.776058197058579	-0.357094450615176	0.345138088652208	-0.254343666666665	0.333881200421942
PDE1B	-0.0978974300944738	0.776058197058579	0.143020577126429	0.793304811422389	-0.190736666666666	0.40525472059024
LOC257358	-0.0979331218976458	0.776058197058579	-0.0124360585737507	0.985220352735461	-0.0612386666666661	0.84487621944046
CLDN19	-0.0979739876558182	0.775982073356616	-0.0508247012402844	0.93996755948181	-0.245015333333333	0.368828018168299
FSCB	-0.097975490039497	0.775982073356616	0.205642878254416	0.672695616352514	-0.0598539999999995	0.791240721091965
PSG2	-0.0980250296499476	0.775982073356616	-0.131506452706259	0.811042872465441	0.180198000000000	0.525033012210884
NIT1	-0.0980259950845612	0.775982073356616	-0.0460245371642747	0.945364757579857	0.266929333333335	0.205838922584493
BRK1	-0.0980940858253582	0.775960569127726	-0.381508881971936	0.297683474654085	0.00928633333333472	0.977381766239087
EFCAB11	-0.0981227856620583	0.775842293180703	-0.257119067840096	0.563116080558022	0.0713963333333338	0.747245436362979
NECAB2	-0.0981322975931134	0.775842293180703	0.428645181142053	0.210173511664670	-0.0946563333333326	0.83616423402744
PPBPP2	-0.0981410462739936	0.775842293180703	-0.110675087399399	0.849359866036793	-0.0711559999999997	0.748579513438275
KRTAP11-1	-0.0981665055862573	0.775842293180703	0.0320408653420131	0.962985798405868	0.0298736666666669	0.90945379489823
SOX11	-0.0981851018927672	0.775842293180703	-0.119077317696034	0.836828045978697	-0.0435729999999997	0.890001991602223
TRMT10C	-0.0981928290908442	0.775842293180703	-0.304379299829582	0.4594472826444	0.0259336666666681	0.91962770573477
PEX16	-0.0982148552739831	0.775842293180703	-0.0988755991777499	0.868042453226125	-0.177634666666666	0.458006502146897
ZNF683	-0.0982190171610507	0.775842293180703	0.115771453857852	0.842992311631688	-0.0657566666666659	0.789228734235839
ATP13A5	-0.0982311639570402	0.775842293180703	0.0808689914511343	0.896361013063453	0.00469000000000052	0.988133186272453
PRDM1	-0.0982453661277752	0.775842293180703	-0.441248832130305	0.187767509360398	-0.0434669999999992	0.897372284357512
SAYSD1	-0.098292291458836	0.775709241682566	0.0173072770059504	0.980723199123135	0.0669476666666667	0.77394434422275
USP9X	-0.098395666809882	0.775241067826797	-0.403750392568694	0.257216918236696	0.0451840000000016	0.849578856396924
ZBTB7B	-0.098406164118874	0.775241067826797	0.211827641915247	0.658506848145678	0.0348270000000005	0.89052375260746
LYSMD4	-0.098447465450535	0.775073293525489	0.075002691071831	0.904466414650249	0.0249893333333346	0.929066230830908
OXT	-0.0984669572437591	0.775053191412877	0.214142361203919	0.653042977738486	-0.133518666666667	0.664617185770066
IL23A	-0.098491869928744	0.775029635955567	-0.0210318963461332	0.97607156937294	0.00550500000000148	0.987134800094338
RGS16	-0.0986409631130903	0.774401494917502	-0.221653781318640	0.637352459162288	0.902035333333335	0.048170505761132
KLHDC1	-0.0986574384476834	0.774363359110028	-0.0562702523133216	0.93229515673473	0.461100000000002	0.09418645408026
METTL2B	-0.0986780404702958	0.774298166294622	-0.349888742476359	0.358969704130952	0.0138040000000006	0.973783155960644
P4HTM	-0.0987014902303606	0.774284195776033	-0.273844944059943	0.522187256281732	0.237015333333336	0.219026555143427
ESD	-0.09871939776725	0.774284195776033	0.406338082792444	0.254041953733213	0.222430666666667	0.266896890079960
RCL1	-0.0988240521168665	0.773947885173774	-0.264217004941627	0.544472698836504	-0.162426999999999	0.499532424296362
MORF4L2	-0.0988272710477092	0.773947885173774	-0.257409217759541	0.563116080558022	-0.368469333333332	0.0896797344789652
GZMH	-0.0988401593226608	0.773947885173774	-0.255687015595335	0.565656702091657	0.109685333333334	0.677570340702693
NKX2-2	-0.0988777469885731	0.773897392737273	0.174482805411664	0.734267769889068	0.125638333333333	0.568903633175651
C1orf106	-0.0988953110807268	0.773875959361554	-0.291759596325688	0.485570493436069	-0.113902666666666	0.580149967309238
CHFR	-0.0989731134127677	0.77355164581824	-0.29885211836788	0.469994970380678	0.0868686666666685	0.678230502146332
SLC3A1	-0.099039724532595	0.773254644482358	-0.184303558799711	0.717436396325728	-0.103219666666666	0.697261622653146
CAPN6	-0.0990738748605786	0.77310061095422	-0.0233342277576379	0.975348968561756	0.0101710000000002	0.980770032498656
LINC00319	-0.0990768688119096	0.77310061095422	-0.096005060285252	0.872311810447594	-0.282543666666666	0.135004030972507
EXOC3L2	-0.0991457618399393	0.77288643073837	-0.0418206659940600	0.951989245458556	-0.205298333333332	0.428358173426632
MADD	-0.099148045192036	0.77288643073837	0.0101352799961862	0.988384322540392	-0.0523246666666659	0.83321169515101
ACER1	-0.0991950320922577	0.772844468507672	0.0729745003703382	0.906592046281011	-0.227078000000000	0.353471671280201
CYP4B1	-0.0992350062889817	0.772743117354513	-0.0271608676175484	0.970207612777188	0.0149400000000001	0.964880128671703
HIVEP1	-0.0992692901071874	0.772724102045008	-0.284490059095527	0.50019549608092	0.187542000000001	0.438934974822897
KCNS3	-0.0992730532288746	0.772724102045008	-0.276248865158767	0.517549833373363	-0.0227443333333329	0.937717709824356
TBP	-0.0993772112573747	0.772403448622169	-0.00902796058707476	0.98909166696601	-0.060290999999999	0.791394257895868
DCXR	-0.0993975294245587	0.772376580770484	0.321026800305437	0.421618886526906	-0.311464333333333	0.162505104948410
KCNQ3	-0.0994047068382359	0.772376580770484	-0.229457395609038	0.620183562399927	-0.184047666666667	0.344576368488222
KCTD17	-0.0994054319030569	0.772376580770484	0.154442756381119	0.771310656070265	-0.202316666666666	0.361971299676959
C4orf6	-0.0994569478288604	0.772281153289143	-0.0182753757692417	0.979327298872732	-0.0986093333333333	0.803131392712617

FAXC	-0.0994632964943797	0.772281153289143	-0.129892555858259	0.813020408185924	0.239349	0.287956132210494
ARL4D	-0.0996744140591191	0.77125743681416	-0.024508062458853	0.973562415846915	-0.0312333333333333	0.906093399285191
KCNA1	-0.0997363729928294	0.77113332120905	-0.23358225445314	0.610436189314218	-0.118292333333333	0.570550813984136
RFC4	-0.099769985597122	0.771005697347159	-0.0391746441565481	0.953905313456582	0.122415000000000	0.583036318848746
DSTN	-0.0998054733699163	0.770889935775236	-0.181416674990480	0.721174140548176	0.312045000000001	0.0996515501712583
IGSF9	-0.0998520403167233	0.770809315012867	-0.179049135419859	0.725806648980606	-0.226458333333332	0.234508116088724
USP6	-0.0998561683882663	0.770809315012867	0.116832822688369	0.841562044027187	-0.0912376666666666	0.748715075113364
B3GNT3	-0.0998720656172212	0.770809315012867	0.106404576035523	0.858284876027025	-0.165636999999999	0.425664293107569
CAPN10	-0.099959765753542	0.770717425216199	0.277110561263648	0.515333497848934	-0.063276666666666	0.777406710539424
HEATR4	-0.099982425691528	0.770638745382594	0.0302225664076886	0.96627173572612	-0.0147836666666657	0.969654650605151
THRSP	-0.0999954412744154	0.770623347008957	-0.018575275212657	0.97902968742576	-0.139924666666666	0.603463436419421
KIAA0895L	-0.100053913499695	0.770487628217194	0.0718299986875992	0.90837606217316	-0.0330739999999993	0.910190419163007
PIK3CB	-0.100059629697507	0.770487628217194	-0.424360445965534	0.216714808349232	-0.0341326666666660	0.898979600726666
UNC45A	-0.100096957690650	0.77037728367374	0.410163473179053	0.246706088283721	-0.083747666666666	0.693228432049376
NTPCR	-0.100132271905850	0.770215566288228	-0.166231185265457	0.751661811727111	0.0439413333333343	0.885345988320645
KTN1	-0.100141841840374	0.770215566288228	-0.362813545924616	0.337032280901653	0.250247333333335	0.178866129871872
FIG4	-0.100208728986698	0.770030518344802	-0.100533631732654	0.866529683756364	0.0447220000000011	0.856640814502011
PRKAR1B	-0.100223481704025	0.770030518344802	0.175648820134583	0.73187914820204	-0.0688126666666658	0.776112195351357
SLC13A3	-0.100251982210462	0.770030518344802	-0.193283105609218	0.698565332048737	-0.171928999999998	0.449693583707481
NKX3-1	-0.100287437322706	0.770030518344802	0.151232193625977	0.777604486471648	-0.090161333333332	0.670510815115172
LGALS3BP	-0.100322639107575	0.769946760684123	-0.0832004961487236	0.893287218356307	0.0158209999999997	0.961665293425945
MGRN1	-0.100333021937738	0.769946760684123	0.00398881333079675	0.994491789164038	0.0661280000000001	0.766247450031517
DKKL1	-0.100339662986237	0.769946760684123	0.133668209334040	0.808933084408149	-0.0271789999999992	0.92927353315392
LCN15	-0.100393130836291	0.769764313513796	0.0755442212304037	0.903517361619051	-0.058249333333333	0.873179602186302
CELF4	-0.100412050751926	0.769764313513796	0.482298560583195	0.135560525489292	-0.0138219999999998	0.9681321732097
NETO1	-0.100461849958185	0.769663617136945	0.0216247646034052	0.97536722461972	0.0205006666666674	0.940972575250573
C3orf20	-0.100490250641912	0.7695472434568	0.109645574609529	0.851352116283305	-0.0669186666666664	0.815540776675037
SGSH	-0.100521995037446	0.769408917849914	0.34283154455018	0.374882411235977	-0.00841033333333238	0.980941723187375
CCNK	-0.100632778845816	0.76882181620677	-0.420440966751691	0.22621473932707	0.213964666666667	0.36569302637233
PLCD1	-0.100663378448773	0.768774305366964	0.0865943742169481	0.887493600482322	-0.191045666666666	0.344663423870532
SERPINB1	-0.100778764465187	0.768334047011382	0.0287320027963716	0.96782691865619	0.137327333333335	0.503400741258744
PARP16	-0.100789841162960	0.768334047011382	-0.0203072677086248	0.97655393383994	-0.220087666666666	0.330549671750340
TADA2B	-0.100803858025647	0.768329449649658	-0.223066788855539	0.635247978870818	-0.0455386666666659	0.888476605628154
PPP6R2	-0.100888738770777	0.767962047781637	0.00765728086383915	0.990405674923721	-0.150542999999999	0.4423818632121
KRTCAP2	-0.100961041177842	0.767718122558895	-0.0302174893127797	0.96627173572612	0.0844809999999996	0.692043732062832
POLR2F	-0.100985645577964	0.767696750507135	0.150350813673015	0.779266896045314	0.00827699999999988	0.981191596773742
TNIP1	-0.101027848051904	0.767559875210826	-0.0668861738301324	0.91517239775041	0.00404033333333532	0.991175862027014
THRA	-0.101067163511413	0.767441950801777	-0.268108810566560	0.53559545997875	-0.102383333333331	0.673620633700871
AGMAT	-0.101094126770376	0.767335037926686	-0.0315926003850817	0.9638977610518	-0.0298063333333331	0.94678260229888
SLC52A2	-0.101138852720175	0.767111547279507	0.296975177665672	0.475402257779173	0.0345680000000011	0.904236796123256
GCLC	-0.101178278312883	0.767062958752361	0.0840501310622875	0.892634402174414	0.0918523333333338	0.729912257977231
PDCD6IP	-0.101199368806533	0.767062958752361	-0.403078455777504	0.258453301425503	0.189231666666669	0.340052241399374
C2orf73	-0.101199678581618	0.767062958752361	-0.0251836438375586	0.972358966414305	-0.0411066666666665	0.878411216446275
PGAP3	-0.101257334497402	0.766964490595483	-0.0417785906041888	0.951989245458556	0.0272660000000012	0.924482212111412
FXYD4	-0.101264106497384	0.766964490595483	-0.278092232376061	0.512275963576091	-0.187928000000000	0.351086080682962
ERLEC1	-0.101299736555131	0.76689641447281	-0.165882219688589	0.751869806279813	0.235495666666667	0.226499104403529
ERC1	-0.101326315076224	0.766792022147785	-0.187247443936426	0.709833349594307	0.158746333333333	0.48802292623238
IFNA5	-0.101464463301334	0.766084819379979	0.0726722491925177	0.90746837333842	-0.033119666666666	0.922697964464038
BFSP2	-0.101469467544626	0.766084819379979	-0.102715596452499	0.86257932725575	-0.0513676666666659	0.870002591217953
LITAF	-0.101535150038086	0.765911753962639	-0.381466566994103	0.297697989583648	0.224589666666667	0.234508116088724
KRT38	-0.101556452738534	0.7658420030114	-0.135931196536015	0.805895463708687	-0.133281666666667	0.639002829892966
LRRC2	-0.101563125035230	0.7658420030114	0.246096522911641	0.582912574644681	0.0327246666666669	0.904520086797883
TRIM35	-0.101595264247188	0.7657974798901	0.104771912668092	0.86067831050617	0.047414	0.850364148347928
KIAA1210	-0.101609073609851	0.765789006666739	0.0113571299271865	0.987250302234215	0.0188703333333343	0.954736472265512
CTF1	-0.101668686380253	0.76559586607964	-0.0375699461302254	0.955365651737247	0.115251333333334	0.592716584553539
CRISP2	-0.101681997694760	0.76559586607964	-0.0697210359096244	0.911167570018484	0.082280666666667	0.696194439936211
PHLDA3	-0.101719195331626	0.765544685586167	0.282966563571621	0.502129505146473	-0.00193599999999902	0.99628178244892
ATP4A	-0.101728778760219	0.765544685586167	0.0933724927105855	0.876578042632603	-0.0548733333333333	0.821421133422134
NTF4	-0.101784473778166	0.765256343479535	0.139189127435315	0.79864602540281	0.116654	0.668518246777557
KRTAP3-2	-0.101823893252088	0.765067672420985	0.103876051759203	0.861570109612412	0.0196950000000003	0.943464122996745
OR1C1	-0.101919688328223	0.764649144539873	0.0925864689894826	0.878131820116803	0.147517666666667	0.486945441486585
LRPPRC	-0.102022013610809	0.764117763610336	-0.00900860958631198	0.98909166696601	-0.0468699999999983	0.85013576481729
SLC36A1	-0.102054579778908	0.764044151949174	0.0686427355148941	0.912149839732916	-0.0522433333333317	0.82667899725046
DGKQ	-0.102090661339832	0.764001342827076	-0.0426897457991663	0.950877394286126	-0.0166286666666658	0.955928393298083
UFL1	-0.102154028853658	0.763741803623969	0.189615582317897	0.704928430617595	0.210152666666668	0.262756877023918
HSD11B1	-0.102242522550284	0.763301252746966	-0.0864321083830936	0.88771097663173	0.169670666666667	0.390967882720418
KCTD21	-0.102289502468923	0.763133080236898	0.342862841630558	0.374882411235977	0.0154798333333337	0.96195931267601
IL1RN	-0.102305504235911	0.763133080236898	-0.246130366024056	0.582912574644681	-0.0802043333333328	0.80841656016322
XKR8	-0.102313123235999	0.763133080236898	-0.307053134017675	0.453557988495973	0.0514930000000006	0.85184175589894
HRH4	-0.102358101331557	0.763065398562093	-0.0865454378884345	0.887493600482322	0.00877333333333344	0.977499755477523
MRPL40	-0.102369687575206	0.763065398562093	-0.077164337983838	0.90125257838581	0.194996666666667	0.449828629541335
WDR90	-0.102394923755909	0.763001663403923	0.278595908100588	0.511025440770733	0.0106226666666661	0.973686596735315
AZGP1	-0.102417371327403	0.762924416573352	-0.140253339621425	0.797441782082887	0.115184333333334	0.607898776363481
PQLC3	-0.102449348925199	0.76278463755606	-0.063628653874907	0.920855211745173	0.164088000000000	0.396454946335441
PCK1	-0.102450178968616	0.76278463755606	-0.0250753750109588	0.97251780790599	-0.125050666666667	0.606689609914879
TNS3	-0.102497368058383	0.76263121890583	-0.374786088270547	0.313141424748220	0.362620333333335	0.07521501709354
GIF	-0.102525105338348	0.762567743053736	-0.179722992072452	0.724082727095475	-0.138871000000000	0.523453039510001
INPP5K	-0.102533722242274	0.762567743053736	-0.268557176277832	0.535014466593144	-0.048437666666666	0.84214313436019
SNIP1	-0.102549693803820	0.762546506252205	-0.507274672449441	0.103996171350646	-0.000384333333332033	0.998643269342783
DOLPP1	-0.102575986555583	0.762497682235044	0.0196661111239749	0.97763669370967	-0.428576333333332	0.0495377766513025

SPG20	-0.102663572449244	0.762027448542839	0.0564835007872501	0.93176886339657	0.171966000000001	0.384691943014905
ANGPT4	-0.102665041964256	0.762027448542839	-0.0474160353911268	0.943981149721771	-0.112284	0.661362028309397
BIN2	-0.102667042666161	0.762027448542839	-0.331999438610533	0.399068746068573	0.0237423333333339	0.944097469332832
CNPY1	-0.102695403369965	0.762027448542839	0.413408580713881	0.239598082905779	0.00351300000000052	0.993756838831997
TMEM218	-0.102701695663578	0.762027448542839	-0.127518921649185	0.81893660870295	-0.0207393333333328	0.946623914607177
NRD1	-0.102713526012359	0.762027448542839	-0.0992172638256926	0.868042453226125	-0.003203333333333200	0.992405716883817
SLC30A4	-0.102732062152245	0.762027448542839	-0.44427505132441	0.183294194447278	0.185744333333333	0.414405354802607
NLGN1	-0.102854521452296	0.76145702161339	-0.296296645518586	0.476715216862939	0.273568666666667	0.347383249495151
SFRP2	-0.102860565318308	0.76145702161339	-0.111309808546061	0.849080210054875	0.00948200000000054	0.975441914935346
UEVLD	-0.102870907301208	0.76145702161339	-0.278525554047438	0.511074209324221	0.0850696666666667	0.696194439936211
HIAT1	-0.102945639280594	0.76113937151262	-0.161581066843169	0.758955920988521	0.142593333333335	0.488602547609567
CD1E	-0.102979030302765	0.761000587676047	-0.059890085983686	0.926038115028264	0.00176699999999996	0.99597023247862
FPR2	-0.103042513536441	0.760713938215099	-0.0985465019788246	0.868278775296491	-0.0225526666666661	0.942030040940208
PNP	-0.1031495656891	0.760390360663275	-0.175567279580195	0.73187914820204	-0.153757333333332	0.558807324932864
RALGAPA2	-0.103261625040325	0.759836838348561	0.260082700794263	0.554790262750304	0.0145833333333335	0.966813448118178
TMEM186	-0.103265543937077	0.759836838348561	-0.289680539587545	0.488870852256136	-0.185912666666666	0.427958580840173
RFC5	-0.103283575449506	0.75983287405576	-0.139229466086562	0.79864602540281	0.0302070000000008	0.90945379489823
RABIF	-0.103325312343199	0.759629143139726	-0.129913757955103	0.813020408185924	-0.0530499999999987	0.826922793139517
OR6W1P	-0.103330017694612	0.759629143139726	0.131790124551584	0.810671652076995	0.00276666666666702	0.993756838831997
FAM78A	-0.103381465576371	0.759566391765485	-0.268624499087800	0.535014466593144	0.0507826666666671	0.828240868185377
LRFN4	-0.103387402592518	0.759566391765485	0.0113940221566527	0.987215779667937	0.00775933333333433	0.982375192575954
DHX32	-0.103390538499249	0.759566391765485	0.192229575084046	0.700781927483785	0.137319666666667	0.495169090369889
CREB3	-0.103423750721712	0.759543590069798	-0.0159198219616331	0.982102356159698	0.0341363333333346	0.891410328517388
KCNAB2	-0.103476891811962	0.759265032501944	-0.0178738551292139	0.979959907954606	-0.237447	0.242351595840697
ZNF441	-0.103480819170127	0.759265032501944	-0.131850813859622	0.810671652076995	0.292062333333334	0.208043530647293
ATG4C	-0.103602779873837	0.75857945937837	-0.620269340549575	0.0237528986370316	0.0710386666666673	0.799725367501923
LYVE1	-0.103612072067517	0.75857945937837	-0.0158514048264827	0.982102356159698	0.0963616666666667	0.674780899769326
C9orf96	-0.103649378872738	0.758413862829533	-0.143917176027867	0.791730335952443	0.11542666666667	0.673538419974363
INTS8	-0.103670681966569	0.758344154909732	0.0223902395351469	0.97536722461972	0.255103333333334	0.243957990539057
SDK1	-0.103743908678684	0.757934055554348	-0.00246692019753751	0.995929168584036	-0.0669836666666663	0.774269136264649
TRIM3	-0.103847621833116	0.757533973588663	0.0604384882048374	0.925576058042088	0.114496333333334	0.631408228698652
SIVA1	-0.103862621879366	0.75750558269117	-0.147982246425022	0.784080588385358	0.0528563333333344	0.831432210225512
ARL6IP5	-0.104013351799333	0.75686731258993	-0.216565091430845	0.647532674045148	-0.133953333333333	0.534910593395784
ERC2-IT1	-0.104016235656591	0.75686731258993	-0.109209595357138	0.852601567492819	-0.00334866666666643	0.992405716883817
EEPD1	-0.10405889756755	0.756723186014334	-0.0955575202566463	0.872754579338908	0.164867666666667	0.496323393705678
DMRTB1	-0.104078195614122	0.756722000075434	-0.0498311053245683	0.941458038227918	-0.133295333333333	0.661362028309397
YWHAQ	-0.104141612927979	0.756446230361476	0.0670355461583631	0.914926078524511	-0.0353366666666656	0.887760071161783
CDYL2	-0.104160221734679	0.756446230361476	-0.411554472258106	0.243528138193926	0.266396	0.188549331106419
ALOX15B	-0.104180491032758	0.756446230361476	-0.0698613245161316	0.910885619751085	-0.179061000000000	0.567207019661951
KCNA5	-0.104201514035128	0.756446230361476	0.00629980932014885	0.991853352023739	-0.326272999999999	0.228292762690892
CFC1	-0.104259809335533	0.756230956393264	0.251994272149506	0.570584539772622	-0.0778233333333333	0.786861016364811
SNX16	-0.104281554769434	0.756228302542497	-0.133280135834521	0.808992949393043	0.125636000000001	0.620692670936735
CASP2	-0.104291911076206	0.756228302542497	-0.287783535441010	0.492287202244585	-0.065605666666666	0.772139923817113
TMEM102	-0.104299180544329	0.756228302542497	0.270223040237154	0.530185035699403	0.0148423333333340	0.95768473561192
EIF3J	-0.104311147952170	0.756228302542497	-0.0428586992843136	0.950491677163598	-0.118433999999998	0.603013238645841
ND2	-0.104335419159481	0.756224997891557	0.093703843372748	0.875544470382024	-0.122815666666666	0.854573398968221
HOPX	-0.104406668837544	0.755827891954673	-0.0165123829600265	0.981833953634016	-0.024978666666666	0.941559580750404
BEX4	-0.104461087579185	0.755750995862272	-0.0615694946295329	0.923436890462326	0.222823666666667	0.313220411537404
TRDN	-0.104507051831769	0.755589611496985	-0.099588477448394	0.867603998426271	-0.107731	0.661812056571718
TMEM223	-0.104565188889926	0.755348438695765	0.220755104594815	0.6389751639565	-0.084912333333333	0.696594849734788
MTHFSD	-0.104617433971337	0.755145895013777	-0.133284261787483	0.808992949393043	0.205845000000001	0.390561931914011
NXT1	-0.104619182019745	0.755145895013777	-0.434094583264492	0.199989310804553	-0.180551666666667	0.471680039257208
SLC9A6	-0.104624628533766	0.755145895013777	-0.436597463485961	0.195288910691951	0.118522000000001	0.628711857980637
C8orf17	-0.104639118652528	0.755145895013777	0.114229439908353	0.844272085764738	-0.163125333333333	0.503511357936433
ECI1	-0.104756199003969	0.754586138086428	0.0690415065592056	0.911947879137232	-0.0852360000000002	0.73097308185803
NDUFB8	-0.104797534404219	0.75452504632429	0.405070185658909	0.25480455106032	0.0793176666666675	0.720071550811076
LY6E	-0.104812346743779	0.75452504632429	0.524754204889934	0.0855836631223817	-0.185432666666666	0.666405059390122
LOC728024	-0.104823031390149	0.75452504632429	-0.117654426306070	0.83940078178718	-0.397322999999999	0.0896797344789652
FAM3C	-0.104833722189367	0.75452504632429	0.0320279208068016	0.962985798405868	-0.0285438333333328	0.910843143481127
FLJ20712	-0.104865822885643	0.754497193775528	0.201127839724364	0.680143033775131	-0.0390553333333333	0.898863720449539
GUCA1A	-0.104912342904221	0.754308889845413	-0.119839498252661	0.8354985445494	0.0135103333333337	0.963389126882705
ACAA1	-0.104919540461852	0.754308889845413	0.00772350987306102	0.990405674923721	-0.0163663333333327	0.960071163568134
SCRIB	-0.104982762650287	0.754080496957099	0.197936545344578	0.687681948030762	-0.133340333333332	0.517547647029412
CCDC140	-0.105011231623591	0.753963854969644	0.0213470205832449	0.97554687597484	0.0494683333333338	0.900268989412964
LYRM2	-0.105020505840046	0.753963854969644	-0.0258971651899023	0.971054398685485	-0.114328499999999	0.575589336285762
VPS18	-0.105087677931473	0.75369915939647	-0.247193095246713	0.58027714848259	-0.223206333333332	0.248900866475991
KRTAP3-1	-0.105127252424381	0.753623178534276	0.166341931995297	0.751360233962663	-0.0861199999999996	0.714244999058301
NCLN	-0.105166286893285	0.753437289461441	0.221332580293301	0.63791595940277	-0.119911666666666	0.580258530958089
ABHD12	-0.105241035295969	0.753166098068876	-0.0310524028574381	0.965118873711308	0.0286880000000007	0.911623909907545
TPD52L2	-0.105293165215465	0.752955515045374	-0.168032255264137	0.748024524669236	0.0436616666666671	0.8754328609757
SQLE	-0.105296108148922	0.752955515045374	0.276346819608139	0.517374509850433	0.0749033333333341	0.79019567212592
INIP	-0.105308107080738	0.752955515045374	-0.0122379106779602	0.98554780734199	0.0216750000000001	0.942092082409632
C7orf34	-0.105322027878836	0.752955515045374	0.24497196414174	0.586459457167736	0.0212970000000003	0.937950350023697
UQCR10	-0.105362606453401	0.752780235430102	0.0719262853175199	0.90837606217316	0.138116666666668	0.49679816436215
FER1L6-AS1	-0.105387691240884	0.752685791944987	0.00402030248569262	0.994485841304070	0.0508700000000003	0.834476346642783
HCN1	-0.105411906274240	0.752597049912045	0.134862629476239	0.807867938002244	-0.0185540000000001	0.961509682688243
NDUFA9	-0.105494888487875	0.752315062743478	-0.333251084686695	0.396894500849691	0.0305636666666686	0.910252591150996
MAPK6	-0.105521011539582	0.752301760973892	-0.168103812485881	0.748024524669236	0.128852333333335	0.62116564059668
FASTKD1	-0.105523486500941	0.752301760973892	0.00250745631012081	0.99580901762095	0.0517039999999997	0.883478349705756
RASSF7	-0.105530685043726	0.752301760973892	-0.0249704556194006	0.972829179061494	-0.0602639999999995	0.816002211548937

ANXA2P2	-0.105536530022570	0.752301760973892	-0.46503696213341	0.155837099170663	0.710390333333335	0.0229237543527331
RNF13	-0.105545875585967	0.752301760973892	-0.259115929347495	0.558209290204909	0.111295000000001	0.675449330308211
PROC	-0.105557162428669	0.752301760973892	0.227115558491936	0.62581495163125	-0.00324699999999974	0.993824047902985
HENMT1	-0.105573952255344	0.752301760973892	-0.443747108894028	0.18420454346518	0.193282666666668	0.543216543662577
VAMP3	-0.105623397883755	0.752301760973892	-0.241640938322585	0.595201219333254	0.178526666666667	0.408369243753251
CDRT15	-0.105669540676636	0.752168092387028	0.351455045164983	0.355703245660861	-0.119184333333333	0.595150543243344
RAET1E	-0.105687983218226	0.752117200368254	0.0446423676903561	0.947920610570169	-0.00974866666666671	0.982523503616988
DAPK3	-0.10575211453295	0.751906802903229	0.223439504531663	0.63471624724109	-0.067073999999999	0.779485206901945
MCCC2	-0.105786530656047	0.75175117936613	-0.199719046355977	0.683035956626567	-0.0239433333333319	0.927671110581394
RAD9A	-0.105787827155066	0.75175117936613	-0.153782673579754	0.773178753339693	-0.100610666666666	0.624073652680538
TMPRSS11D	-0.105789310722406	0.75175117936613	-0.00086287410434936	0.998745415362821	-0.127759999999999	0.536063442230912
C12orf39	-0.105877139727501	0.751423005233297	-0.158046296914603	0.767373487136803	-0.0522783333333326	0.824700707442131
NEDD4	-0.105889661171864	0.751423005233297	-0.353696292978106	0.352236748429479	0.267222666666667	0.274620788437103
CD4	-0.105946551756098	0.751262252421597	-0.353107458770384	0.352407344218756	-0.108029000000000	0.671766969198954
DPY19L1	-0.105969745229019	0.751180218990289	-0.227078352294453	0.62581495163125	0.270757333333335	0.25622310036454
LTBR	-0.106097484266413	0.750692893394691	0.314932002093133	0.436301158221661	0.118979000000000	0.583036318848746
SNAP23	-0.106182701851151	0.75048435483368	-0.331173344114765	0.401127919590714	0.184871000000000	0.381082408187926
CETP	-0.106204831997032	0.750409301549792	-0.309258129931353	0.448350131365446	-0.00508166666666642	0.987499363290182
GRHL1	-0.106220953710440	0.750404168500845	-0.4562097896505	0.167648587973919	-0.116491666666667	0.600146208272329
CANX	-0.106223074587946	0.750404168500845	-0.246992474413758	0.580916018784993	0.00419666666666818	0.988942941807152
B9D1	-0.106246768772603	0.750404168500845	-0.095761033874592	0.872537273851412	0.062643333333334	0.780717071872908
ASXL2	-0.106256826112356	0.750404168500845	0.115316357784064	0.843755596233057	-0.058399999999999	0.830147563067484
EMC10	-0.106309312287059	0.750214596083824	-0.289599608254106	0.488870852256136	0.038904000000001	0.883376555562104
ZNF502	-0.106388270246267	0.749837001426233	-0.0904302872026791	0.881797721301484	0.228269666666667	0.25470355352902
FGF16	-0.106403600690736	0.749806534668777	-0.0313006781824683	0.964858024544415	-0.0318766666666666	0.924674647382585
TBC1D15	-0.106449330122347	0.749585266317357	-0.0189081352889931	0.978456646972519	0.0673810000000005	0.763331507619331
TMEM50A	-0.106455780733178	0.749585266317357	-0.177868134629797	0.728031191477223	0.0907833333333348	0.682185125074327
FZD1	-0.106465309359999	0.749585266317357	-0.169813607201705	0.743328907676018	0.318886333333334	0.238974545470918
LINC00189	-0.106476632310602	0.749585266317357	-0.401061595763746	0.262127055693923	0.360615333333334	0.135004030972507
SPATA1	-0.106508650594637	0.749538084656149	-0.112322482454615	0.847454473507881	0.106511000000001	0.677570340702693
ATP6V1G3	-0.106584518914673	0.749180777979776	0.0124158264183344	0.985220352735461	0.0612270000000006	0.78960166957155
SUV420H2	-0.106777002179165	0.748549303622016	0.357184310939712	0.345098975152893	-0.0207229999999989	0.938783708088713
NMBR	-0.106961321804598	0.747481438301418	0.145478421232702	0.787804113309913	-0.0195569999999998	0.957844701580613
C1D	-0.107046338667199	0.746994521354267	-0.154424834700907	0.771310656070265	0.326828500000001	0.0929567349015668
CCDC38	-0.107067407966692	0.746994521354267	0.191286384728820	0.701804778213811	0.00098900000000027	0.997144862160692
GHRL	-0.107206970315511	0.746571801005848	0.132203299768850	0.810300414153187	-0.182016666666666	0.436020392195898
PQBP1	-0.107219724820999	0.746558196893852	-0.210038019771396	0.663025134165522	-0.137912333333332	0.500226891090192
NDUFAF5	-0.107324837879826	0.746359323362837	-0.0196163572921056	0.977673457110824	-0.0312303333333323	0.910920430866321
FAM71C	-0.107345650134360	0.74629294977234	0.0328586529397596	0.961351562552925	-0.0250509999999997	0.934473497709064
MTMR12	-0.107402174174170	0.746053618196042	-0.614840813730112	0.0266008531275411	0.294035333333333	0.159240262526426
ATG2A	-0.107455174571370	0.745899251627909	-0.25252433175798	0.569562951596314	-0.0274139999999994	0.919420614501031
LINC00315	-0.107465146254391	0.745899251627909	0.0222163886004780	0.97536722461972	0.124525000000001	0.565020605732227
PPDPF	-0.107467299928511	0.745899251627909	-0.266341763179097	0.538833471023229	0.280556333333335	0.238780185222418
MAD2L2	-0.107536260265362	0.745741487097789	-0.344396816559681	0.370887661638426	0.143386666666666	0.462361390840849
SLAMF8	-0.107560348725917	0.745726133008012	-0.300870405053193	0.464997080622275	0.0141003333333334	0.97289725310129
CST6	-0.107602744104579	0.745588355855226	0.353345553290542	0.352407344218756	-0.168328999999999	0.549557793610283
TRIM15	-0.107668920711110	0.745434764726546	0.268171943887063	0.535543061070273	-0.0699169999999989	0.786861016364811
HLA-DOB	-0.107683718154433	0.745434764726546	-0.148015851619352	0.784080588385358	0.0309178333333335	0.91402300740264
GLT1D1	-0.107687717049452	0.745434764726546	-0.0469767895334474	0.944081286115546	-0.0524696666666659	0.828344660369637
PIK3C2B	-0.107699623587826	0.745434764726546	-0.139807089915542	0.797957231657366	-0.372993666666666	0.0802890385571452
ZNF566	-0.107752239389477	0.74530890624244	-0.166836570839597	0.750272141744695	0.0220070000000015	0.950980037243398
TMEM185B	-0.107776823197247	0.745217776395324	-0.331987622368576	0.399068746068573	0.237825666666668	0.263064019049831
FHOD3	-0.107808500034114	0.74515948618741	0.0875129250034074	0.886302037299497	-0.326288666666666	0.246475973365341
ITGAM	-0.107837473654738	0.74515948618741	-0.28871283784154	0.489813374112312	0.0555403333333341	0.834148450369096
CD1A	-0.107893250549797	0.74494510998738	-0.171350039530347	0.73955239116035	-0.361549666666666	0.0894613209101226
POMT2	-0.107897995383155	0.74494510998738	-0.00543941100776772	0.99251028338798	0.0477010000000004	0.846457250180447
GALNT6	-0.10798571300905	0.744644941454626	-0.0400988118697378	0.953163386277443	-0.346186666666667	0.0749561445182193
SLC32A1	-0.108009261912059	0.744644941454626	0.277163424413575	0.515261763771907	-0.0189173333333333	0.967256128104685
RIMS4	-0.108139100309417	0.7440828536689	0.0957922770063877	0.872537273851412	0.122289333333334	0.603978635563313
MRPS10	-0.108164418349800	0.74400860695872	-0.0666470002363392	0.915724830490542	-0.287562333333331	0.158095716859654
PGLYRP2	-0.108250578631476	0.743711731224701	0.118496066963088	0.837761659039664	-0.0256926666666665	0.92590541082869
CENPV	-0.108276287414721	0.743711731224701	-0.0512258725137152	0.939111418302491	-0.0123063333333334	0.98173288785111
PFKM	-0.108453608532061	0.743024207226611	0.0902455308886778	0.882168609130864	-0.208083333333333	0.334527228768702
CALCOCO1	-0.108508518449751	0.742845827422467	-0.189260737881372	0.705993769232416	0.00388366666666743	0.990194019173407
CNTNAP1	-0.108551449951184	0.742845827422467	-0.254153700933972	0.567694846153007	-0.0982723333333325	0.772139923817113
PQLC1	-0.108579709317012	0.742828377214569	-0.106669647928061	0.857937967416038	-0.0561389999999993	0.811816884537132
SYT12	-0.108628559537969	0.742578325097294	-0.0158840550409616	0.982102356159698	0.0658183333333337	0.783846059037353
PLA2G4A	-0.108658957633330	0.742449196871061	-0.00824397663212612	0.990076134399615	-0.00760999999999994	0.982375192575954
PITRM1	-0.108667511718594	0.742449196871061	0.191177195464555	0.702083312665327	-0.106276333333333	0.60026500215056
MED30	-0.108678878492332	0.742449196871061	-0.168187913272184	0.747936039510864	-0.0432689999999991	0.868046126197316
BCKDHB	-0.108707778014087	0.742419475653791	-0.356996178898783	0.345138088652208	0.0632983333333337	0.811997756288597
MLEC	-0.108749663868233	0.742419475653791	-0.0201420475286492	0.976956866647027	-0.465263999999999	0.119487210509652
ZNF329	-0.108759512339633	0.742419475653791	-0.32972653170645	0.403821204945523	0.127954333333334	0.562674320626673
SH3BGRL2	-0.108820572810417	0.74230111816403	0.414637487171533	0.237522444879778	-0.0877053333333325	0.728909820842191
ANLN	-0.108838943745506	0.742250813526172	-0.0544975421515876	0.9344692810234	-0.045506666666665	0.883739719585572
NAA40	-0.108887815647210	0.74214205420677	-0.367263864556292	0.327869177739765	0.0436060000000012	0.853541852582193
H2AFV	-0.108924428173603	0.742041066047009	-0.201267758616563	0.680054545720343	0.00175333333333456	0.995621063297892
VSTM2L	-0.108926882239434	0.742041066047009	-0.0481086483523988	0.94314236584312	-0.00124399999999947	0.996826292964745
ZNF555	-0.108961295797908	0.742041066047009	-0.209456334973599	0.664420531246941	0.219545666666668	0.443072320689246
HAO1	-0.109052072802526	0.741732147906891	-0.227504774014258	0.62539640207912	-0.069671666666666	0.751653256027624

PBOV1	-0.109067632543074	0.741732147906891	0.0532160764894986	0.936629055455007	-0.0478863333333332	0.86715815306237
CLPTM1L	-0.109089576336859	0.741729995518199	0.0189500729878789	0.97832242527852	0.150910666666668	0.459936388684371
TTLL4	-0.109139663175910	0.741600894680418	0.292231814987695	0.48422498009582	-0.0735743333333323	0.735063355984464
ZSCAN16	-0.109158871073608	0.741556328309458	-0.290698170304601	0.487927261832191	0.0164793333333338	0.974451420920923
CUL4A	-0.109167168631730	0.741556328309458	-0.0196381199673866	0.977673457110824	0.0427320000000012	0.86045090010169
CCDC28A	-0.109199746108855	0.741428661576256	-0.0580643301703245	0.92929701257827	0.0057200000000007	0.987937064305561
VANGL1	-0.109210435893661	0.741428661576256	-0.600603932247343	0.0331255554751519	0.301371666666667	0.135569775932062
CATSPERG	-0.109227179387699	0.741428661576256	-0.104693430721305	0.86067831050617	0.0737540000000001	0.758341714501375
NDUFV3	-0.109237217528315	0.741428661576256	0.096004107012830	0.872311810447594	-0.0674416666666664	0.764848773122078
SLFN11	-0.109265220247019	0.741420509244322	-0.398496888189523	0.267349827447097	0.0152880000000004	0.963180491237413
ZNF257	-0.109309852818451	0.741268208741642	-0.401208776172874	0.262072933179063	0.702042666666668	0.105097444982544
STK32A	-0.109320815674728	0.741268208741642	-0.115739867538249	0.842992311631688	-0.135881333333333	0.5530742296054
TAAR5	-0.109408941669704	0.740937634587546	-0.0672666914416204	0.914700759157169	-0.00657900000000043	0.9879739922351
SF3A3	-0.109456366703305	0.7407988139827	-0.405964692904524	0.254336088055854	-0.146315999999999	0.498454352317107
CXCR3	-0.109465914273803	0.7407988139827	0.151161251522668	0.777711725504625	-0.145968333333333	0.578893615511221
SUN3	-0.109471400753562	0.7407988139827	-0.303154318193478	0.462105017205825	0.00500833333333383	0.987937064305561
LIN28B	-0.109569940166908	0.740545533789453	-0.361798574432477	0.338556116268406	0.085742666666667	0.697187294779476
BBS1	-0.109652031740016	0.74021780005114	0.065549800575436	0.918054091945491	0.0183493333333344	0.952100146370651
CD300C	-0.109652450989140	0.74021780005114	-0.182872003816842	0.718289071039953	-0.122300666666666	0.581570366864416
FAM43A	-0.109660784248413	0.74021780005114	0.103766529113879	0.86183768727892	-0.107313666666666	0.60378773986659
GPR143	-0.109662713820074	0.74021780005114	0.115775301748933	0.842992311631688	-0.0204666666666659	0.973783155960644
TRIT1	-0.109675875272046	0.74021780005114	-0.0662152198182699	0.916661988649658	0.106201000000001	0.639837290428782
LOC283588	-0.109684046408451	0.74021780005114	0.0897363569640012	0.88318195439136	0.032574666666667	0.924674647382585
MRPS22	-0.109713914016562	0.74021780005114	0.290454373070556	0.488252082376591	-0.0921159999999995	0.656941730375963
DPF1	-0.109738285550740	0.74021780005114	0.157315921600835	0.767774203843122	0.0134089999999998	0.963537990151403
IL17RC	-0.109741145625987	0.74021780005114	-0.081625597702814	0.895799810931455	-0.0663813333333321	0.77718596107678
CYorf17	-0.109784841594026	0.74021780005114	0.264950536655676	0.542477070548696	-0.0717313333333329	0.824110405462623
ZCCHC11	-0.109798252620751	0.74021780005114	-0.0519809428968209	0.938067483409541	0.0606236666666674	0.811867274062946
SCRN3	-0.109853235726995	0.740162985034513	-0.228373795986650	0.623306946452998	0.352098333333334	0.108927207186132
TRMT12	-0.109867336446952	0.740140785964375	0.238768319805315	0.600757014649216	-0.214867666666666	0.314112513261693
STRADB	-0.109890888483658	0.740056548007566	-0.0211885210071651	0.975853964465421	0.934415166666668	0.00441604774813744
WFDC6	-0.109903439731758	0.740044522491628	-0.0192077531249882	0.978232639595454	-0.0806919999999991	0.729912257977231
THOC6	-0.109916196014490	0.74003115367181	-0.19429015949059	0.696504707499023	-0.255452333333333	0.223260757343954
TM6SF1	-0.109932170900444	0.74003115367181	-0.397063425582886	0.270862229167822	0.0590070000000005	0.818730551027274
LAYN	-0.109944876460752	0.74003115367181	-0.0846895484691022	0.891309254245872	0.116577500000001	0.589703432017128
PVR	-0.109949102868520	0.74003115367181	-0.184554365038547	0.716515903916773	0.0853283333333336	0.691940834235362
OSBPL1A	-0.109964924724071	0.74003115367181	0.100293042562416	0.866744687214714	-0.00573833333333332	0.987459024686908
ATAD3C	-0.110042340146210	0.739838770578987	0.236955457929992	0.603972819955905	-0.223894999999999	0.267017625061681
OR51B6	-0.110075092996589	0.739832697141982	0.050209473845134	0.941133149988298	-0.0655296666666666	0.815274179657758
DSCR9	-0.110091706176585	0.739794031351068	0.198053331961697	0.687574233588603	-0.127074333333333	0.616472837170869
LAMTOR3	-0.110124813995209	0.739717482245035	-0.244204500728022	0.588660529799743	0.218581666666669	0.262002806497455
DUS1L	-0.110198647110853	0.73930308114069	0.317295097320457	0.431286094810965	0.0378423333333355	0.880790657394578
RAE1	-0.110206970554157	0.73930308114069	-0.133871567424135	0.808567812988011	0.0211113333333345	0.941559580750404
MYO15A	-0.110228159616014	0.73930308114069	0.130196228616495	0.812271254601992	-0.054593666666666	0.857169623339788
C21orf67	-0.110250932384098	0.73930308114069	0.0528916481314863	0.937009489777134	0.143310333333334	0.463987285756381
GNG4	-0.110254034845790	0.73930308114069	-0.00318873940095539	0.994730189431381	0.161985333333334	0.450667095645064
ALOX5	-0.110291759550055	0.73930308114069	-0.211850800265873	0.658506848169835	0.0090250000000007	0.984899942310247
ZNF567	-0.110298886785210	0.73930308114069	-0.148598008561113	0.783455328724491	0.138223000000001	0.673538419974363
TMEM167A	-0.110326281809872	0.73930308114069	-0.248951960052383	0.576871285544251	-0.205816666666665	0.352897567720880
EPS8L2	-0.110361919478580	0.739287632219753	-0.06907517306495	0.911928203725685	0.00067466666666726	0.998281147912498
CNOT7	-0.110392447298306	0.73918460994212	0.23364666696029	0.610436189314218	0.0414170000000011	0.862509125961694
OR8B8	-0.110415942728320	0.73918460994212	-0.0156546177783713	0.982266426585123	0.00071633333333340	0.997953056544486
CRAT	-0.110465485943561	0.739100816830125	0.0482746579233641	0.942853417571722	0.125270000000001	0.566282142073858
MED25	-0.110469783552732	0.739100816830125	-0.0210333438092912	0.97607156937294	0.0159006666666673	0.95589485717785
TMEM135	-0.110588680310706	0.738644015520269	-0.0399815424513145	0.953370115695118	-0.170348333333334	0.41903055047835
IGSF9B	-0.110608321488749	0.738585496408088	0.00175803868420838	0.99764296910735	-0.044139666666666	0.883189085233712
FGL1	-0.110636439666223	0.738528146076702	-0.0838585340809321	0.892833228561937	-0.0084126666666665	0.983159241812861
APOA5	-0.110650836288134	0.738517759878263	0.140942055768386	0.796714214272984	-0.083217666666666	0.717951095501435
SLC43A3	-0.110661902183044	0.738517759878263	-0.395093459948607	0.272692742937895	-0.263642666666666	0.198299581791991
LOC285696	-0.110681618898052	0.738517759878263	0.0918429358047209	0.879153312143409	-0.0829886666666664	0.774104133965233
ARHGEF10L	-0.110701071909452	0.738517759878263	-0.0800263106655016	0.897577172229156	0.0906953333333343	0.671479766417605
CARTPT	-0.110713120994669	0.738517759878263	0.00292590712153962	0.995261259442339	-0.150357333333333	0.561051487344043
CLEC1A	-0.110725105540302	0.738517759878263	-0.249657675069039	0.575370998761354	0.0881566666666665	0.68135180147671
NALCN	-0.110791317063931	0.738300223105469	-0.174498465823046	0.734267769889068	0.0069116666666668	0.983109651883712
SIRT3	-0.110796736255796	0.738300223105469	-0.35560579401641	0.347014488563088	-0.0715869999999983	0.755519603705424
NEUROG1	-0.110818796542045	0.738300223105469	0.321446922545118	0.4207907841056	-0.00784866666666658	0.986471172041113
CLIC3	-0.110831015017340	0.738300223105469	-0.105446885329975	0.859800688625324	-0.0760376666666662	0.798804424103298
VPS37D	-0.110834856241494	0.738300223105469	0.40673095748131	0.253455126075109	0.0106563333333338	0.977499755477523
PDLIM5	-0.110855923756716	0.738300223105469	-0.0969080852723876	0.870644879522268	0.638613000000001	0.0222121644428973
MANSC1	-0.110925930098570	0.738121474096279	-0.0234162883738505	0.975348968561756	-0.0496906666666663	0.842903889380894
SAMD10	-0.110937459180654	0.738116316186204	0.100607530365986	0.866414040673375	-0.117894333333332	0.625510328921698
ICA1	-0.110957557129293	0.738116316186204	-0.338845489854058	0.383664784230327	-0.0990313333333331	0.631912818412474
C18orf56	-0.110970874246057	0.738116316186204	0.157917865673926	0.767373487136803	0.026943333333334	0.941559580750404
SPATA21	-0.111066912280687	0.737971764977912	0.389229697339584	0.283516069208756	-0.0567936666666662	0.811867274062946
C1QTNF2	-0.111079245999961	0.737961346576892	0.253944025325258	0.567694846153007	0.0285466666666676	0.941876953554646
CREB3L3	-0.111116080667068	0.737789783306444	-0.0985003046520277	0.868278775296491	-0.019754333333333	0.967285731317065
TAF10	-0.111177709763983	0.737516724605651	-0.129407605746103	0.81427296395351	0.0482713333333346	0.846655457900969
ZNF205	-0.111193222915480	0.737494532756218	0.231474271557299	0.614876015201472	-0.00410800000000036	0.991175862027014
BTF3P11	-0.111244035349871	0.737301758952117	0.229819385562468	0.619381301776937	-0.0140313333333332	0.965290875803469
ZNF823	-0.111280722414862	0.737201878484572	-0.0737296036540156	0.905506084101768	0.0653863333333337	0.843152489417868

AMMECR1	-0.111348438828324	0.736897966577737	-0.450486681454756	0.176663460992485	0.212559000000001	0.270000135172916
SUN5	-0.111424623903926	0.736467766426904	-0.135906568497335	0.805895463708687	-0.154235333333333	0.443378989641463
BMX	-0.111431986377631	0.736467766426904	-0.376099900340992	0.309828524690001	0.167548666666667	0.451743047233008
CRX	-0.11144870863353	0.736450752701266	0.141962935350163	0.794642725104275	0.00492666666666671	0.990385063260966
EMC8	-0.111491648708277	0.736380410860028	-0.206345561568767	0.671467763213228	0.0187760000000000	0.94678260229888
C9orf62	-0.111529973204904	0.736199123146402	-0.0758770379325305	0.903510413986113	0.111479000000000	0.604966458785844
ATP10B	-0.111558503334196	0.73608222859492	-0.187160755915387	0.709833349594307	-0.0718376666666664	0.814273596067945
SCN3A	-0.111582113490102	0.736051244827683	-0.414650393974043	0.237522444879778	0.0231050000000008	0.936688436284818
DNAH3	-0.111600031058179	0.736028229835286	-0.0846934352415214	0.891309254245872	0.135556000000001	0.493413216077427
PCMTD1	-0.111663144059880	0.735747634586881	0.123743281852030	0.826475556843795	0.164210499999999	0.489999806074963
LDHAL6B	-0.111714757374607	0.73554967195889	-0.0647316960418504	0.918970974321351	0.0746720000000009	0.823910138699963
ARHGAP39	-0.111733994042003	0.735493877081724	-0.0479810297263699	0.94314236584312	-0.0492009999999991	0.850157189407213
CCDC27	-0.111767410739794	0.735441596340961	0.322875760727552	0.415927283236740	-0.0581949999999992	0.84207712482186
UFSP1	-0.111841473184092	0.735215310933672	0.193057311954619	0.699002603433222	-0.077398666666667	0.789105818888855
HN1	-0.111849855836537	0.735215310933672	-0.217656653932258	0.644891652669682	0.166993333333333	0.409335260696998
USP39	-0.111891991483321	0.735099931953258	-0.103652508440759	0.861872724387044	-0.0160519999999985	0.955928393298083
SLC6A12	-0.111927639822861	0.734998248405611	-0.0145053755411075	0.983444353656142	-0.092961333333333	0.68414816517367
PAFAH1B2	-0.111932262211663	0.734998248405611	-0.252663291718416	0.569339421777585	0.170273000000000	0.390561931914011
FTL	-0.111939534880486	0.734998248405611	-0.341708430044203	0.377337219880391	0.169739666666667	0.39576473605983
SPATA6L	-0.11196025229527	0.734995901397685	0.154713610257083	0.771310656070265	0.00562966666666642	0.986591122558806
FDPS	-0.112001868321340	0.734859394393363	0.225507622339474	0.629710977741725	-0.131021333333333	0.51139718772887
PABPC1	-0.112104877638884	0.734423647436009	0.0554014194790504	0.933409139126562	-0.0291733333333331	0.913184328748245
ITIH1	-0.112106224032916	0.734423647436009	0.00722777721543723	0.991025604847553	0.0944660000000005	0.733556041294047
UBE3C	-0.112111756007414	0.734423647436009	-0.248908909512708	0.576871285544251	-0.0924773333333324	0.667040000089664
DCST1	-0.112157641521337	0.734334460702509	0.102873752977375	0.86257932725575	0.0268476666666673	0.920540786765438
WFDC3	-0.112194238447232	0.734164574335833	-0.216282216161091	0.648391670659269	-0.12403	0.675221998008387
CASP10	-0.112201409067949	0.734164574335833	-0.0275577936340010	0.969821510572266	-0.147376000000000	0.5531802635914
UBR7	-0.112228684878861	0.734164574335833	0.0170401450672248	0.98105020869911	0.211058333333335	0.293767671859086
C9orf38	-0.112259655759215	0.734164574335833	0.0134404864907289	0.98412883278237	0.0729293333333334	0.774104133965233
LAMC2	-0.112370927367623	0.733710021113512	-0.00864029840227715	0.98946753270812	-0.138073333333333	0.566282142073858
STC2	-0.112382037671923	0.73370768975025	0.050282128718032	0.941062359630336	0.00381500000000037	0.990471306092672
ABT1	-0.112403283260215	0.733681833723204	-0.0740621430878935	0.905506084101768	-0.0120486666666664	0.974484855799046
VSIG4	-0.112423664297436	0.733681833723204	-0.283922958837635	0.500873210019812	-0.0512226666666663	0.895093529187836
KRT85	-0.112424081930672	0.733681833723204	0.147929402546202	0.784090188380266	0.054652666666667	0.81742145099016
ZSCAN10	-0.112436931404735	0.733681833723204	0.0428121466830952	0.950593273212722	-0.0350163333333329	0.911600329626215
DDX19A	-0.112464804851730	0.733681833723204	-0.0368692168712008	0.956918184553224	-0.0103459999999992	0.972327702257384
TSSC4	-0.112500619050033	0.733659412314188	-0.0904492227557736	0.881797721301484	-0.0924246666666664	0.669547980569952
OXSM	-0.112551853889702	0.733549581195831	-0.148749729624943	0.783041584899534	-0.122969666666666	0.653738419919818
SLITRK5	-0.112559683261798	0.733549581195831	0.0747048597622196	0.904466414650249	-0.232367666666666	0.337994438406310
CCL28	-0.112632754199288	0.733295039391707	-0.132479430596662	0.809866082339655	-0.00228366666666565	0.99492454715772
ARRB1	-0.112728091216359	0.732776407300487	-0.433226283305986	0.201555549804162	0.168886333333335	0.466985343927182
TBX6	-0.112732655679890	0.732776407300487	0.134470870511787	0.808009477165608	-0.0832939999999997	0.71173331874509
REG3G	-0.112733371323133	0.732776407300487	-0.0409802643426211	0.952426531595115	0.0248913333333335	0.949492888401158
PHKB	-0.112783308607298	0.732696371912362	-0.0328226480855266	0.961351562552925	-0.128437333333332	0.528488494367153
ABCB9	-0.112805011276441	0.732695208054167	0.184041193890418	0.717937553844852	-0.150324333333332	0.443378989641463
VAMP2	-0.112809358058311	0.732695208054167	-0.0148128208518937	0.98327723682819	0.253846333333334	0.182479537094754
GPR62	-0.112834368004072	0.732695208054167	0.159007254865892	0.765416392217362	-0.0331399999999996	0.904507944803624
RHOT1	-0.112864163572741	0.732640819005133	0.387758572042227	0.286134842484356	-0.395786333333333	0.0828974079436732
BTRC	-0.112872252764095	0.732640819005133	0.337566671672341	0.386297465201585	0.249245666666668	0.209567117558470
CD1B	-0.112905235620467	0.732640819005133	-0.142443144209262	0.794346532964905	0.00082000000000051	0.997844982819387
GLS	-0.112948552081861	0.732459174424375	-0.21210255051213	0.658047200841489	-0.0595563333333332	0.815309621716298
ZNF471	-0.112968586374486	0.732459174424375	-0.133026439769653	0.809578511927685	0.305127000000001	0.141626794658499
SPATA8	-0.113032566546162	0.732119021094204	-0.0884307356713514	0.88490989383909	-0.161505333333333	0.449436921437102
TMEM196	-0.113043535929919	0.732117691687143	0.0190108052604950	0.97832242527852	-0.104383000000000	0.777704292898672
CECR1	-0.113219316436363	0.731435362951468	-0.406030771336447	0.254252360501407	-0.091858666666666	0.704019823060907
HCFC1R1	-0.113232675837733	0.731435362951468	-0.191065181235531	0.702217515036541	-0.0742959999999994	0.728894262229555
SLC35A5	-0.113311447143860	0.731134661974963	-0.172583437931954	0.738421727152922	0.200922333333335	0.359908305595919
LILRA4	-0.113338859858031	0.731107441347613	0.204666585062881	0.674021386858844	0.00620266666666692	0.987134800094338
GNL3L	-0.113454070437942	0.730764766983159	-0.498463210540876	0.114105676690072	-0.203453999999999	0.508159273570429
CALCOCO2	-0.113475387466722	0.730764729315412	0.0483989964377748	0.942820948997969	0.278651	0.143763220356682
NEURL	-0.113516539894187	0.730621261157777	0.127742846593521	0.818440182600377	0.069155666666667	0.78960166957155
ERGIC3	-0.113542817626210	0.730604605410657	-0.0474046961099384	0.943981149721771	0.00192333333333557	0.995204546518148
PIP	-0.113560064446309	0.73059345665792	-0.0782121623774817	0.899647854618521	-0.0313469999999994	0.913644671932166
EDDM3A	-0.113574895876789	0.73059345665792	-0.097817767505794	0.868852648210739	0.109968333333334	0.745730849000278
PTH1R	-0.113650385111019	0.730251362970325	0.110600532789068	0.84940885580427	-0.260249666666666	0.266021084950461
GAL3ST1	-0.113663624157204	0.73023514055474	0.32131085435972	0.421269686148499	-0.114326000000000	0.692043732062832
NME6	-0.113684366876202	0.73023514055474	-0.431033400350855	0.206246644924294	-0.0070893333333318	0.98349947509937
FNDC8	-0.113756824395527	0.729912004053216	0.184993488649889	0.715658781310868	-0.0455529999999997	0.885345988320645
HPSE2	-0.113836621695437	0.729735041816594	0.0149134602318875	0.983028706010457	0.0118066666666667	0.9681321732097
HES7	-0.113844362883162	0.729735041816594	0.0584383029705167	0.928725686060612	-0.181816999999999	0.550563293736455
FBN3	-0.113896525914719	0.729553642305836	0.160869006811131	0.761016253411733	-0.146346333333333	0.523453039510001
ESX1	-0.113901735006981	0.729553642305836	-0.080035719855251	0.897577172229156	0.0158806666666674	0.955115242974667
LRCH1	-0.113903151900844	0.729553642305836	-0.412119471229395	0.242943598295198	0.151537000000000	0.458006502146897
SNORA71A	-0.113939507199683	0.729553642305836	0.212409817091745	0.656785033957219	0.0203456666666675	0.954908838133862
PFKFB2	-0.113982328830163	0.729553642305836	-0.101896335231891	0.864616357134757	-0.188574333333333	0.350056273654693
ZNF384	-0.114008794347373	0.72952411162731	0.00562884863377519	0.99251028338798	0.0348116666666675	0.888319685310725
POLD2	-0.114056756642666	0.729279469192489	0.082798023454642	0.893709269554424	-0.162446666666665	0.400749522225696
OR1A2	-0.11408871429993	0.729140140084521	0.140793122120038	0.796714214272984	-0.0404393333333328	0.868011655529718
PDYN	-0.114090908526747	0.729140140084521	-0.0100630939362824	0.988421999436642	-0.125225666666666	0.60217016768875
TRMT13	-0.114105232831948	0.729140140084521	-0.0770959468752715	0.901343076217132	0.182378000000001	0.503217861354116

ZGPAT	-0.114147974447027	0.729033957741401	-0.181529239881944	0.72105659643795	-0.081603999999999	0.730777457405814
PRKCE	-0.114173475622176	0.728937109744323	-0.0667272104362673	0.915438681913279	-0.156727666666666	0.590101586536522
CYP2A13	-0.114197366985707	0.728921789474859	0.223311750373988	0.635113856809423	-0.00194366666666663(	0.995928140100809
UBOX5	-0.114199390817832	0.728921789474859	-0.181445443934400	0.721174140548176	-0.0289733333333327	0.90945379489823
PSMA4	-0.114209407809174	0.728921789474859	-0.206240073668540	0.671467763213228	0.0620233333333342	0.795446186721078
FAM21C	-0.114232384593734	0.728921789474859	-0.205610773535925	0.672695616352514	0.0180348333333343	0.950238313474508
ZCCHC6	-0.114262589772674	0.728921789474859	-0.0865471694494872	0.887493600482322	0.0638086666666675	0.787798433132403
CASC1	-0.114298360685997	0.728921789474859	-0.164393321175183	0.754189116283047	0.134552	0.508529020331279
LIMD1	-0.114308628590274	0.728921789474859	-0.0542477621962754	0.934724166908893	0.0368970000000002	0.903274690789156
FMO1	-0.114320868390715	0.728921789474859	0.0631934563426988	0.921359867071909	-0.0358019999999998	0.890094615232462
PRSS37	-0.114417335257264	0.72853883068644	-0.0214645218088837	0.97536722461972	-0.0265706666666661	0.923273649346542
MRPL2	-0.114475759218992	0.728225314872647	-0.292224802128563	0.48422498009582	0.0164470000000006	0.952448105846014
USP30	-0.114588050199666	0.727770319986321	-0.191407771811213	0.701653985008336	0.156031333333333	0.450233254347116
SLC16A9	-0.114597858112478	0.727770319986321	-0.0944016750965043	0.874410047496143	0.368592333333333	0.090903987582686
UNC5CL	-0.114646837014887	0.727667409727304	0.221853921843607	0.636778528562165	-0.0322713333333329	0.90945379489823
REEP4	-0.114682836074231	0.727572498664874	-0.0178303834713346	0.980042218206248	0.0423396666666675	0.891410328517388
FTSJD1	-0.114688693402208	0.727572498664874	-0.265442161071498	0.540577577805463	0.151797333333334	0.443378989641463
C22orf42	-0.114689440511270	0.727572498664874	-0.218486518323190	0.642691336921378	-0.0928203333333326	0.68171195530621
GEN1	-0.114713220286371	0.727572498664874	0.0432476956171779	0.949991344015323	0.0209906666666664	0.942298301774487
TRAPPC2L	-0.1147571392347	0.727572498664874	-0.0268634212104652	0.970207612777188	-0.155559333333332	0.449627269673783
WNT9B	-0.114761284370064	0.727572498664874	-0.148678714627675	0.78320766974083	-0.0822589999999992	0.705312045318091
ZNF483	-0.114762733518946	0.727572498664874	-0.150001894062082	0.780413206123914	0.577358333333334	0.0195270907849587
IFIT1	-0.114776813617229	0.727572498664874	0.039177133895834	0.953905313456582	-0.0103336666666648	0.974451420920923
FGF4	-0.114777487259863	0.727572498664874	-0.144386834494562	0.790525434005598	-0.136220333333332	0.648381288631965
LIMA1	-0.114851134805984	0.727388125880798	-0.155989302044351	0.76972820876143	-0.262661666666666	0.198299581791991
PIGR	-0.114916734347342	0.727169463421516	-0.167083418470617	0.749671494148584	-0.0896516666666662	0.670071773006358
FAM20A	-0.114955347079179	0.726986345167162	-0.398605185228994	0.267272688443470	-0.191252000000000	0.471680039257208
SKP1	-0.115104960979990	0.726285833052822	-0.310166112418961	0.446321819288896	0.115355000000001	0.634966811516658
GAS2L2	-0.115153646738549	0.726107505590521	0.0728897901169642	0.90686556727651	-0.167343666666666	0.49000765832939
ADCK5	-0.115197707049204	0.725888620837929	0.455595695612374	0.168217575057610	-0.208176333333333	0.456074677719062
CCDC129	-0.115217555693240	0.725829032050097	0.235581728566542	0.6067637636079	-0.0982726666666664	0.636490807097692
TTI2	-0.115223749324140	0.725829032050097	0.0519914277805713	0.938067483409541	0.103372166666667	0.613915242226352
ZNF382	-0.115226808026728	0.725829032050097	-0.227920159960575	0.624359290301343	0.549279333333334	0.0557323350548905
COPS4	-0.115250756212964	0.725829032050097	-0.178031814934962	0.727982464250053	0.257785666666668	0.249885255869378
ALDOA	-0.115315768309508	0.72572601765204	0.0333794383135305	0.960784250392482	0.0655526666666673	0.766132481270445
TGIF2LY	-0.115321525850025	0.72572601765204	0.0200789301418692	0.977011537442717	-0.0753844999999992	0.766574854574416
DNASE1L3	-0.115335826516189	0.72572601765204	-0.200179029621873	0.681552955146541	0.0153073333333339	0.955460918122607
KIFC3	-0.115448653099583	0.725515919451903	0.268443497914926	0.535217216332192	-0.299147333333332	0.235054998483403
CCL17	-0.115466294386339	0.725515919451903	0.215979370282615	0.64844945818838	0.0520846666666668	0.831432210225512
LRRC29	-0.115468756352031	0.725515919451903	0.173276122419487	0.736740209571778	0.0317223333333344	0.911600329626215
TMEM33	-0.115470112726759	0.725515919451903	-0.187754355611204	0.708377771996406	0.0884470000000014	0.674028958837018
CREG2	-0.115476455447359	0.725515919451903	-0.122660006674957	0.828930912293926	-0.0529879999999990	0.856854911614454
KCNQ1DN	-0.115494736766266	0.725515919451903	0.133476233423096	0.808933084408149	-0.0194536666666657	0.965329851466378
FLJ32154	-0.115503059825982	0.725515919451903	0.156277780903786	0.768909201237015	-0.108098999999999	0.707538975864338
DAND5	-0.115504594299923	0.725515919451903	0.183746485564418	0.718067440349544	-0.0693753333333326	0.787798433132403
PPP1R27	-0.115507915210536	0.725515919451903	0.163829452597034	0.754916750929288	0.00092600000000001	0.997144862160692
H2AFY	-0.115529830455041	0.725515919451903	-0.298467568079632	0.471239854755259	0.00966133333333504	0.974451420920923
CAMTA1	-0.115570858536676	0.725515919451903	-0.149238473837948	0.782576259201979	0.0842053333333338	0.693228432049376
NOD2	-0.115572183337823	0.725515919451903	0.0488188879130108	0.942180113539637	-0.314789999999999	0.195804289605525
PHB	-0.1156128143652	0.725515919451903	-0.167292983674349	0.7491393962206	-0.200860999999998	0.306145675725963
ACR	-0.115634336333292	0.725515919451903	-0.107441083418214	0.857065724607536	-0.086228166666666	0.720021524916561
HAMP	-0.115658970674617	0.725515919451903	-0.126004327786796	0.822261327404759	-0.133377	0.667585734216380
CYP2B7P1	-0.115659048239308	0.725515919451903	-0.0351125130266322	0.95895015491824	0.603561666666667	0.0121741378444765
CCR3	-0.115702100311624	0.72548353241304	0.0718956559764198	0.90837606217316	0.104565333333334	0.689267979966846
USP42	-0.115793041746222	0.72502688017115	-0.097885528904018	0.868852648210739	0.111867666666668	0.628131452559002
FBRS	-0.115805589626978	0.72501543733252	0.116359295326585	0.84214534364593	-0.0847539999999983	0.729912257977231
WBSCR17	-0.115834079775756	0.724898984988658	0.103998544328504	0.861570109612412	0.203415000000001	0.286939266819770
C14orf166	-0.115940906252427	0.724409011958068	-0.00329212598028482	0.994730189431381	-0.104207666666665	0.659036556301843
AKNAD1	-0.115965288005926	0.724319636051439	-3.09391372580797e-0	0.999906934715157	0.0972826666666674	0.692494286263795
ERCC8	-0.115987531016386	0.724315531528154	-0.440305236495548	0.189456871688041	0.0729330000000012	0.772139923817113
AKAP4	-0.116007697725491	0.724253915957535	0.0236621563299180	0.975152846635334	-0.107633333333333	0.626635388688185
ELANE	-0.116038249239062	0.724123914355851	0.426836782433629	0.21316922601328	-0.186345666666665	0.515415448777249
INSM2	-0.116065696110849	0.724106414411064	0.0334640874005423	0.960784250392482	-0.00856133333333278	0.982375192575954
SLC26A7	-0.116107580038574	0.724023329917156	-0.297715801929117	0.473204540069408	0.778756333333334	0.0848737046559716
SLU7	-0.116113389854556	0.724023329917156	-0.310885380531243	0.444320505310503	-0.193433666666665	0.324443655582244
CST9	-0.116154356459531	0.723858350783633	0.285032462862424	0.49838641873573	-0.138783999999999	0.574042216179207
NOS3	-0.116167896649383	0.723858350783633	-0.353564980953081	0.352236748429479	-0.0361566666666668	0.890792289911257
DNAJC5B	-0.116215337676706	0.72374093329595	-0.0406983645377164	0.952426531595115	0.0134690000000004	0.974451420920923
EIF1B	-0.116243786186402	0.723624789757645	-0.116406377086944	0.842082118939645	0.219554333333334	0.41785973815832
ZNF396	-0.116306858570840	0.723280644927841	0.0423542231601529	0.951504349503947	0.0235896666666674	0.931446587444396
NR1I2	-0.116375650505930	0.722970085391372	0.0534544074680606	0.936250424462674	-0.0200923333333333	0.948621641545621
OR1D5	-0.11638314231225	0.722970085391372	-0.254332357696263	0.567694846153007	-0.149665333333333	0.5530742296054
NRG1	-0.116426750683415	0.722805223265632	-0.242481656493555	0.593398090075382	0.762323333333334	0.0218686868425037
MANBAL	-0.116519480340646	0.722330068301684	-0.282893389569927	0.502129505146473	0.116557666666667	0.620840471737699
FDXR	-0.116584037976034	0.722096519113894	0.423438204057469	0.218841338302193	0.071258000000002	0.812617573218469
SNX9	-0.116591753459185	0.722096519113894	0.057468184233437	0.93030634693695	0.253664666666668	0.182794399904883
HSD17B7	-0.116608161932001	0.72208004597085	0.102453822471061	0.863158472924381	-0.321936333333333	0.151776694305005
ZNF133	-0.116614923546494	0.72208004597085	0.0142038123078954	0.983444353656142	0.0476910000000001	0.83616423402744
OPA3	-0.116622832443613	0.72208004597085	-0.100685525755200	0.86628046156792	-0.139102333333333	0.501298037624658
SLC13A4	-0.116632033704152	0.72208004597085	0.25637587739646	0.564430426783556	-0.151899333333333	0.520709741182581

NAT2	-0.116689710839609	0.721899112745347	-0.0523337290068112	0.937831566264837	0.0517593333333339	0.855240892932328
FRMD4B	-0.116720255778001	0.721769234584617	-0.387367178788686	0.286979481895785	0.0984346666666669	0.66807888867384
ANKRD55	-0.116721927852492	0.721769234584617	-0.381260984253361	0.297930089233645	-0.0206629999999992	0.94010374131705
CDS1	-0.116757527342460	0.72166625502271	-0.0169981352964481	0.98105020869911	0.144473000000001	0.537186705207113
PLEKHM2	-0.116797095222768	0.721477001655221	-0.125091992670523	0.824230922676958	-0.0322583333333318	0.91143191097679
DDIT4	-0.116821124472689	0.721390019886265	0.174354296053022	0.734267769889068	-0.167332666666666	0.557700710345762
C9orf43	-0.116912110344006	0.721039212664324	-0.00673446923156169	0.99129507664977	0.0785066666666675	0.71325466407859
TLX3	-0.116948042249694	0.721039212664324	0.118884541138633	0.83702538133036	-0.0185373333333327	0.952460815972492
SLC34A3	-0.116950337604600	0.721039212664324	0.218947795080842	0.642050021576508	-0.00771899999999866	0.982375192575954
GOLM1	-0.11700283708524	0.720976992705946	-0.285019218519618	0.49838641873573	0.385220000000002	0.085481985741103
FBF1	-0.117056628677767	0.720907726452413	-0.0077550698875618	0.990405674923721	0.0714346666666671	0.765020657072822
TMEM87A	-0.117077762272488	0.720839821866445	0.173211447350142	0.736740209571778	0.0786446666666671	0.713007765552813
GIMAP6	-0.117080222947454	0.720839821866445	-0.496389909729345	0.115856499197964	-0.0224266666666660	0.944218475609619
TGM6	-0.117103517449651	0.720839821866445	0.0242765804355562	0.973992718287184	-0.0598389999999993	0.801352759417909
LPPR3	-0.117114156671658	0.720839821866445	0.348942133709618	0.36071884720096	-0.0352819999999993	0.914066650169675
CXXC4	-0.117151481079022	0.720839821866445	-0.141995709411542	0.794618470341742	0.188451333333334	0.442833812576453
HPD	-0.117205753437194	0.720839821866445	-0.169646691726323	0.743463590096836	0.224801000000001	0.300274668021666
POLB	-0.117211516905401	0.720839821866445	-0.00780122225927238	0.990405674923721	0.107556666666668	0.778912256576868
CHRNG	-0.117247086371466	0.720793830391426	0.416045375141142	0.234713120194856	-0.0860849999999995	0.774104133965233
TMBIM1	-0.117251182201202	0.720793830391426	0.039168753310026	0.953905313456582	-0.0267906666666663	0.922854901626297
CITED1	-0.117254049013531	0.720793830391426	-0.0965853783726028	0.870878919920942	-0.208421333333333	0.438162889121856
SYT3	-0.117289458030151	0.720793830391426	0.093755801574163	0.875355472414883	-0.167192333333333	0.425664293107569
SEC61G	-0.117322091574557	0.720793830391426	-0.13157857897348	0.811042872465441	0.0452670000000005	0.846023945154185
FTSJD2	-0.117322480382186	0.720793830391426	-0.131077120033521	0.812271254601992	-0.0687249999999993	0.760951989611575
ERVK3-2	-0.117340474457440	0.720793830391426	0.0944730379742955	0.874384897652873	0.0473636666666673	0.85409823183973
NPHS2	-0.117343635476850	0.720793830391426	-0.111718063371292	0.848331918863767	-0.0872456666666666	0.718749100405876
CLPS	-0.117349478663866	0.720793830391426	0.148793889752873	0.783041584899534	0.0156556666666674	0.95482028291900
CLUL1	-0.117353884116393	0.720793830391426	-0.130547947087328	0.812271254601992	1.13713833333333	0.00311122808725912
OR51I1	-0.117373332388782	0.720793830391426	-0.126193098171442	0.822164329801713	-0.0188373333333332	0.949173055617325
FSTL3	-0.117388907959996	0.720793830391426	-0.170619480774451	0.741623909905464	0.0118423333333338	0.969654650605151
ZDHHC3	-0.117402797706403	0.720793830391426	-0.0244588548823679	0.973562415846915	0.151168500000001	0.451528670919448
TMEM168	-0.117431121848130	0.720793830391426	-0.0902506279342471	0.882168609130864	0.221465333333334	0.270000135172916
TMEM71	-0.117544785467004	0.720402514366216	-0.305378997522627	0.458013550008763	0.0099180000000008	0.976702491879288
FOLH1	-0.117576224685499	0.720402514366216	-0.118186617786686	0.838440856484595	0.0427653333333341	0.85994094150432
BNIP3	-0.117604835346004	0.720342619746835	0.261018214256881	0.552796181718632	0.0557226666666673	0.814956840733813
IDO1	-0.117665687874023	0.720177152497369	-0.198539921755433	0.685952362965345	0.740096333333334	0.0689724571534153
RAI14	-0.117678415811468	0.720164714194212	0.415878182342311	0.235080055809448	0.724137	0.0241179875336435
LCMT1	-0.117706123438010	0.720053524399753	-0.0351871981902608	0.958873757674185	0.100110666666668	0.70916032632167
IPO7	-0.117735681401891	0.72000161237302	-0.065987171114807	0.917090201751967	0.0257303333333355	0.928295508521289
INA	-0.117778642968968	0.719861343407611	-0.0971432049565597	0.870020870354202	-0.0870693333333332	0.822980493051179
FZD4	-0.117799517263111	0.719795209691658	-0.181270220077791	0.721196149497303	-0.375386666666666	0.147305400857476
RBMX2	-0.117829747024913	0.719667404413815	-0.462702323024389	0.15879071503051	-0.124513666666665	0.528488494367153
UCK1	-0.117831974649365	0.719667404413815	-0.00925039267990419	0.98909166696601	-0.0120399999999997	0.974484855799046
UQCC	-0.117911357396357	0.719415331809497	-0.0937911123618918	0.875355472414883	-0.0386339999999991	0.883189085233712
RNF133	-0.117996462625276	0.718925877451062	-0.187932779995736	0.70798562655309	0.137902333333333	0.518244955194698
SYN1	-0.118016879274568	0.71886277461036	-0.246507920563551	0.582108346881299	-0.0136039999999991	0.962924081933534
NOC4L	-0.118018194104070	0.71886277461036	-0.134215686689237	0.808009477165608	-0.0631206666666659	0.77290215873365
CRISP3	-0.118019405321521	0.71886277461036	-0.16853577273908	0.746758286599055	-0.0595783333333331	0.824700707442131
NGB	-0.118089412530185	0.718727842030234	0.244569529889361	0.587653287154633	0.0217176666666675	0.948469012194168
PFKFB4	-0.118138559898970	0.718561038481528	0.188452363932523	0.707361603786947	-0.173798333333332	0.378344763124493
FBXL4	-0.118145654501996	0.718561038481528	-0.0701541771575032	0.910436470094634	0.134030000000002	0.577524803794493
STXBP1	-0.118189525164141	0.718470698286279	-0.114700658889100	0.844162087612813	-0.0417023333333315	0.883189085233712
ALB	-0.118231755486375	0.718382943969183	-0.117619352706513	0.83940078178718	-0.216930999999999	0.274635121257823
DUT	-0.118293379851210	0.718255423306035	0.00902992979923196	0.98909166696601	0.0387193333333343	0.890094615232462
SASH3	-0.118343341563672	0.717997619664188	-0.332818612320685	0.397725187718434	0.0573286666666676	0.837369354308588
ZSCAN18	-0.118361913673438	0.717997619664188	-0.361991730725992	0.338556116268406	0.0411363333333344	0.874960823800592
PON2	-0.118493578250454	0.717579383643926	0.439780351602728	0.190498298131724	0.263036666666667	0.162505104948410
MED7	-0.118544160046999	0.717379965354467	-0.0504094175724465	0.940709772508288	0.0704976666666676	0.765339817431245
ZNF677	-0.118549545373096	0.717379965354467	-0.361510223874029	0.339145270424456	0.256438333333333	0.426007592485647
F13B	-0.118591418169941	0.717288175986544	-0.0334202747756758	0.960784250392482	0.0526996666666668	0.880347900939311
RNF34	-0.118690973865472	0.716779010518411	-0.122503889632823	0.82920723741687	-0.0504913333333325	0.83568726766556
NAT9	-0.118699257834394	0.716779010518411	0.0635171185623908	0.920855211745173	-0.205891	0.284462281603797
PKHD1L1	-0.118721917023320	0.71671807727321	0.0379403640104151	0.954889910016503	0.036399666666667	0.883913953154694
FHDC1	-0.118792761201283	0.716537212744705	0.108485585802513	0.85395367181085	-0.0615509999999992	0.7996066102903
C9orf85	-0.118838265683376	0.716451848090248	0.133507007420944	0.808933084408149	0.0924650000000002	0.741438256509195
GUCA2A	-0.118850955752268	0.716451848090248	0.088727735693984	0.8841943128258	-0.0295819999999996	0.925792017089844
LOC149373	-0.118853497053521	0.716451848090248	0.0214602025024573	0.97536722461972	0.167278000000000	0.450203306181989
ZNF701	-0.118857190406913	0.716451848090248	-0.143922146483894	0.791730335952443	0.0182476666666669	0.94692575385424
ALDH1B1	-0.118947114300040	0.716275404765345	-0.0613392115803129	0.92421768673053	-0.358585666666666	0.107866708545887
HHEX	-0.118963048728517	0.716274667340199	-0.117353086655974	0.839922145389918	0.184246333333334	0.397798624983652
TLE4	-0.119036055024976	0.716006642125779	-0.14914851081787	0.782612598081927	-0.511960999999999	0.188232886284507
TUFM	-0.119058362235636	0.716006642125779	-0.282451858213258	0.503412540505745	0.0714900000000014	0.742452599762094
PCDH9	-0.119067937104629	0.716006642125779	-0.0834864563153233	0.892833228561937	-0.195923333333333	0.361190470706496
RNF123	-0.119081995777002	0.716006642125779	-0.00428634954288940	0.99405344216434	-0.0994709999999995	0.640836927155663
PHOSPHO1	-0.119102334061905	0.716006642125779	0.216671269533259	0.647357580911858	-0.344761666666667	0.107866708545887
EDN2	-0.119108712618931	0.716006642125779	0.00926329467431837	0.98909166696601	0.153013333333334	0.464959317742365
CXorf27	-0.119186305597503	0.715760539805575	-0.238172980786213	0.60184463266698	-0.144201666666666	0.685254219232839
MTMR1	-0.119208761020368	0.715760539805575	-0.529104473460528	0.0799297163112453	0.0985716666666676	0.641044966033937
XYLB	-0.119225518736436	0.715760539805575	0.0157528805745547	0.982266426585123	-0.174214999999999	0.470695033055552
TRAT1	-0.119261557471913	0.715701824923321	-0.302494276396253	0.462229407853213	0.0787410000000006	0.733750603134827

VGLL1	-0.119280615068343	0.715682994307794	0.0919418769824166	0.879153312143409	0.169388666666667	0.558807324932864
SIGLEC7	-0.11929735034253	0.71564427792638	-0.0495254302137902	0.94156962266198	-0.027497666666666	0.929646115212086
NEURL3	-0.119356886554627	0.715394982319093	-0.296489931112652	0.476535595649776	-0.0419166666666673	0.873398028647672
KLK10	-0.119403149839228	0.715180847154351	-0.382332314657802	0.295293127580539	-0.184045333333332	0.522830837265953
SLC9A7	-0.119425114591747	0.715160025258756	-0.623761076598263	0.0233850729680282	0.288434666666667	0.210845715069665
EMID1	-0.119443428741808	0.715110906873912	0.104075539084036	0.861545877009007	-0.225017333333332	0.244066141899848
MSTN	-0.119588908332679	0.714613340058283	0.140655498837715	0.796714214272984	0.130955666666667	0.592845916952892
MTUS1	-0.119613447898408	0.714613340058283	0.150963207641724	0.778205892965504	0.110246666666668	0.720247067628545
MAGEA10	-0.119644341126641	0.714613340058283	0.182049193485253	0.71977894194971	0.213589666666667	0.332315132116478
PXMP4	-0.119692860870167	0.71454102559288	-0.091708635105285	0.879153312143409	0.199406000000001	0.323963327709623
SPAG16	-0.119700489933479	0.71454102559288	-0.190577213333521	0.703715698557082	0.0894336666666676	0.696927295330661
SUB1	-0.119701376977082	0.71454102559288	-0.225885054693671	0.629121375721303	-0.0263133333333329	0.924674647382585
TRPV4	-0.119730337746766	0.71454102559288	0.157248021735707	0.767897349801793	-0.060410333333333	0.824548552404467
LOC284751	-0.11974081569350	0.71454102559288	-0.416712892388302	0.234235464483992	0.114346000000001	0.607009506431467
GIGYF1	-0.119786539399916	0.714496813987018	0.104248554964555	0.86124543980304	-0.117199666666667	0.596617523400524
F10	-0.119848345917065	0.714232591567531	0.538086097996225	0.0719192718188871	-0.116879999999999	0.690641668678273
RFPL1	-0.119905224299195	0.714191736778815	-0.121097790553195	0.83265693609154	0.00219600000000063	0.995718974481635
C11orf53	-0.119907431748628	0.714191736778815	0.184595689132205	0.716456739167429	-0.120685333333333	0.663515191078113
CCDC125	-0.119919730998638	0.714191736778815	-0.320092676441598	0.423328320249032	-0.0512634999999994	0.840657236939036
CSNK2A2	-0.119969842194587	0.714005074371032	-0.00697764522399089	0.991290883124684	-0.026360333333332	0.924674647382585
ZNF189	-0.120005670242384	0.713840488871451	-0.124893541857235	0.824457843812434	0.481323333333335	0.0313933135786716
C14orf79	-0.120094240839186	0.713551495744817	0.133013045781406	0.809578511927685	-0.194273333333333	0.443378989641463
SIRPG	-0.120107633100105	0.713551495744817	-0.278470777096914	0.511269463609085	-0.0780683333333337	0.772139923817113
CHRM1	-0.120224475917169	0.713043031965866	0.13969581874267	0.797957231657366	-0.081301999999999	0.714092841685447
HERC6	-0.120285109792960	0.712786653174362	-0.333427265376707	0.396821740334178	0.225814000000002	0.231998578744259
UBL4B	-0.120384565141796	0.712202707158723	0.0196053938241992	0.977673457110824	-0.0101336666666664	0.978504228748436
GXYLT2	-0.120406232790210	0.712131563869955	-0.275459133521754	0.518891193183863	-0.309498666666667	0.387687154733619
PGK1	-0.120418839027499	0.712120145027897	-0.134644744742683	0.808009477165608	-0.108783333333331	0.610151958903093
GDPD1	-0.120466023719470	0.711952496058065	-0.348107831969151	0.361374443308161	-0.169054666666667	0.511696593608944
CDK9	-0.120494331616786	0.711837597455395	-0.13088568647042	0.812271254601992	-0.103806666666666	0.685488482699362
LSM7	-0.120657079301876	0.710980217830381	0.0250935662546361	0.972494094458606	-0.246057333333332	0.199922586577398
SERPINF1	-0.120703912149407	0.710768927575761	-0.136488337051709	0.804536748810928	-0.366048666666665	0.436636858796753
TM6SF2	-0.120782349251797	0.710541958128532	0.426558035698793	0.213221804021091	0.0315846666666675	0.92590541082869
GLRX	-0.120792141599440	0.710541958128532	0.407417482574026	0.252285038466352	-0.259600333333333	0.443072320689246
LOC51145	-0.120871653164792	0.710426194614831	0.0773242815597873	0.901037327680555	-0.0424473333333326	0.864688646629146
METTL18	-0.120879102029744	0.710426194614831	-0.206516410253527	0.671162787418854	0.0945526666666675	0.672674698802727
UBE2D2	-0.120948598131287	0.710278544785477	-0.389644243279956	0.282945386998586	0.0618570000000006	0.791934055525056
COL14A1	-0.121003547823400	0.710140288244206	-0.199288782765137	0.684227048797313	0.0884939999999998	0.779569065519332
C8orf12	-0.121022716404037	0.710140288244206	0.152433117789070	0.775863712880333	0.00214566666666727	0.99628178244892
SEC14L4	-0.121058254749579	0.710128406089538	-0.108543722144431	0.85395367181085	-0.0191381666666664	0.956024791104257
FAM154A	-0.121079430235492	0.710060566997554	0.0185836814922736	0.97902968742576	0.0229816666666672	0.930383110415834
TAAR8	-0.121110130305309	0.709929971370271	0.266521234089916	0.538833471023229	-0.065314333333333	0.766569862858904
DLGAP2	-0.121151059968501	0.709875366212611	-0.00264613315391232	0.99580901762095	-0.116179333333333	0.575378149225683
ATP1A1	-0.121165013856876	0.709875366212611	-0.100131657174444	0.867048047646049	0.356660000000001	0.0689876351548137
MSRA	-0.121174157607220	0.709875366212611	-0.102516660155856	0.863158472924381	-0.0041246666666669	0.993474375150267
OCEL1	-0.121177675480046	0.709875366212611	0.102757088529317	0.86257932725575	-0.0674526666666654	0.766563380016891
SUGT1	-0.121179293766218	0.709875366212611	-0.222037583837969	0.636621575666471	0.115113000000001	0.589826330962094
CCDC157	-0.121272539127762	0.709505138747348	-0.039604845859614	0.953730904892187	-0.0033463333333333355	0.993677488355417
SNX29	-0.121319058294627	0.70934203761209	-0.373573267386059	0.315811026719894	-0.0084740000000005	0.982375192575954
SPCS3	-0.121350859068031	0.709314662424659	-0.611134339982882	0.0277519681937494	0.0626500000000009	0.792778259205127
SLC37A3	-0.121441860824845	0.7090349010138	-0.278682602979861	0.511025440770733	0.0939486666666675	0.659835984620576
C9orf40	-0.121492358248332	0.708816622566664	-0.12858983487525	0.815827854535765	0.0376596666666674	0.880131955403697
GMPPA	-0.121498836590731	0.708816622566664	0.0331624107822445	0.9610502944838	0.0309083333333345	0.903774117860245
CYP4A22	-0.121500589498273	0.708816622566664	-0.251697405493151	0.571279752918483	0.0666573333333335	0.780512234657452
C16orf59	-0.121527292875023	0.708816622566664	0.123241723857341	0.82767952783856	-0.0349649999999994	0.887363245747406
NDUFB7	-0.121554087578006	0.70872580174233	0.0182441679488799	0.979327298872732	0.0908870000000003	0.670113640649168
AP2A2	-0.121711394975504	0.708067227023447	-0.313561608700426	0.438046311563112	0.0135293333333344	0.963626568322179
LRR1	-0.121746055403906	0.708034852381525	0.134878001684949	0.807867938002244	0.0293110000000012	0.917153642391165
NXNL2	-0.121767650242171	0.707964314189054	0.0237079869764940	0.974997824325618	0.178798333333334	0.358471531901741
ZNF826P	-0.121925000695610	0.707358133061854	0.078546458525417	0.899281198071059	0.0266733333333337	0.929594956567283
LSM2	-0.121963598054292	0.707230732675409	-0.0183142542784754	0.97931205662978	0.113274000000001	0.670419470313263
MX2	-0.121967561753533	0.707230732675409	-0.391876426536853	0.280021017230138	-0.088861333333333	0.703148620232928
ODF3L1	-0.121970397459629	0.707230732675409	0.0633979338308717	0.920885810365418	-0.116264	0.592716584553539
OR1A1	-0.122105881425639	0.706883973011192	0.00523661642898214	0.99251028338798	-0.130354666666666	0.520709741182581
NDUFA8	-0.122106615584439	0.706883973011192	-0.0973074715346599	0.870020870354202	-0.0125819999999986	0.97053261035629
IL10	-0.122110584505162	0.706883973011192	-0.0361197225986002	0.958023954102604	-0.149540666666666	0.474788546852034
FLJ35816	-0.122121876427498	0.706883973011192	-0.39142481563789	0.280540221838803	-0.0441616666666673	0.893839463620913
SNRPD2	-0.122203080497761	0.70667419514649	-0.0813402938423008	0.896271792537737	-0.0957889999999995	0.649977903605799
CYP4F8	-0.122208631972347	0.70667419514649	0.152702962318073	0.775154530747777	-0.0602793333333327	0.78345865774329
SIRT5	-0.122239199487883	0.70667419514649	0.285021321807582	0.49838641873573	0.0060783333333337	0.985183831169496
MAPK15	-0.122294612751591	0.70664542514545	0.130731057341821	0.812271254601992	-0.0857146666666656	0.737704293354791
CNIH3	-0.122330389942555	0.706589907949361	0.0116367469417102	0.986654146365942	-0.567657666666665	0.0195270907849587
FLJ30403	-0.122337374901238	0.706589907949361	-0.0569702360462618	0.930784376757936	0.159463333333334	0.410937431848516
CFP	-0.122342926760277	0.706589907949361	-0.101139466479615	0.866005093242444	-0.109907999999998	0.625258262671142
WDR53	-0.122369141267581	0.706585259643523	0.183773270123664	0.718067440349544	0.128555333333334	0.549323798856043
RYR1	-0.122369179023186	0.706585259643523	0.123764116471785	0.826475556843795	-0.00705599999999961	0.985263856374185
SLC6A15	-0.122441518088566	0.706251956280252	-0.0768498263178904	0.901856698918763	-0.0906643333333329	0.6846855637534
ARNTL2	-0.122500627747055	0.706024085599851	-0.448844958258251	0.177860736739947	0.138990333333335	0.540614958546206
SH2D1B	-0.122562472047619	0.705850468136394	-0.191482186073935	0.701635983922402	0.0368413333333336	0.927204985660666
TJP3	-0.122577928747944	0.705850468136394	0.129492916637152	0.814099225370639	-0.0667096666666668	0.812358226571296

MASP2	-0.122600975465885	0.705850468136394	0.0619236277192209	0.923034515346352	-0.0215923333333328	0.93948504599535
LRSAM1	-0.122618603682978	0.705850468136394	-0.0630152284825884	0.921642681436352	-0.0926296666666662	0.73097308185803
TMEM169	-0.122631680205204	0.705850468136394	-0.0908049135947216	0.881323965029954	0.0219383333333337	0.949287545570912
VASP	-0.122665040655475	0.705809387588053	-0.326166890955928	0.409206882509236	-0.0129280000000009	0.96554120323216
HTR1E	-0.122748910198481	0.705561241434282	0.0268273619416959	0.970207612777188	-0.0594716666666666	0.834140029892063
TRMT112	-0.122848030617325	0.705278000994486	-0.119306719455801	0.836360753951164	0.0969333333333331	0.664425919683979
VIMP	-0.122884439098448	0.705187967013898	0.0318917536030892	0.963211116393051	-0.0616303333333335	0.778083808586846
BROX	-0.123056757711114	0.70428168674094	0.0387991998183349	0.954263089081373	-0.0469759999999997	0.842903889380894
RTP4	-0.123091133664799	0.70412708523399	-0.297203195600694	0.474635631314086	-0.0736226666666667	0.73097308185803
TFAP2C	-0.123104528332628	0.704124774637857	0.130128057556494	0.81240810100014	-0.161797333333332	0.398883894731131
ABCA6	-0.123121400826368	0.704124774637857	0.153241539715274	0.774029578907844	0.134244000000000	0.498454352317107
EDN1	-0.123130118716746	0.704124774637857	-0.396817280699399	0.270862229167822	0.161702000000002	0.655103900829872
PNPLA7	-0.123180600359112	0.703896975451411	0.3401673036271	0.379495306350778	-0.159969666666666	0.41666677641248
SLC35G2	-0.123183382080927	0.703896975451411	0.250270808871846	0.574107101982055	0.322363666666668	0.11129998147197
FAM129A	-0.123251007678300	0.703719487640125	-0.463270640708589	0.157837170342414	0.299900666666668	0.340072381100187
PMCHL2	-0.123311553254399	0.703393662973945	-0.165645386253179	0.752289873539669	-0.0497538333333329	0.839791315519305
TOMM40L	-0.123327870180635	0.703358037121381	-0.414552574935724	0.237522444879778	-0.316316666666666	0.151342644884613
KLKB1	-0.123364108125220	0.703245964143763	0.0246280149961349	0.973393389577498	0.0478556666666669	0.842903889380894
INPP5F	-0.123374660387242	0.703245964143763	-0.386614775121954	0.288385928289901	0.290319333333334	0.163725198409623
VWA5B1	-0.123403101168192	0.703149936009818	0.166067370901856	0.751830402832405	0.115220000000001	0.626500880282759
ZNF211	-0.123405782789725	0.703149936009818	-0.193825757681033	0.697189653813355	-0.00531133333333131	0.989433129523288
ABCA5	-0.123468404409700	0.702935398438797	0.213535028908654	0.654198328177448	0.296049000000001	0.399371411881998
IFT43	-0.123484553622325	0.702900891988945	-0.278439244069246	0.511325408819849	0.0534743333333346	0.822657709336468
AGBL2	-0.123506873688004	0.702897604380903	-0.192695032549652	0.699904185007497	-0.0470976666666659	0.838927397112197
HIBADH	-0.123519633814082	0.702885427622371	0.0582089132636965	0.928879154519933	0.193106	0.324937679238988
NPEPPS	-0.123533883535073	0.702863442627546	-0.346151809086411	0.366023660299226	-0.0495893333333325	0.830320848383177
CADM4	-0.123574947135587	0.702664854083872	-0.185657574707057	0.714043644840406	0.128482333333334	0.600950363504414
TRIM66	-0.123622023993886	0.702528099877908	-0.0515472880062698	0.938728344711776	0.0532193333333338	0.813754719427875
SS18L2	-0.123659035719877	0.702528099877908	-0.181281730822116	0.721196149497303	-0.283572666666667	0.142821378450256
MAGED2	-0.123755070375613	0.702197297304222	-0.266710838829198	0.538390048324742	0.174823333333333	0.353471671280201
BEND4	-0.123793491478438	0.702016142276972	-0.215154645424213	0.650959077206536	0.0462126666666676	0.90945379489823
DOK5	-0.123810119016370	0.701978518396593	-0.112590122951815	0.847191484945575	0.212965333333334	0.378089536203812
DCC	-0.123884436281993	0.701776628110263	-0.0521768777584497	0.938067483409541	0.102266000000000	0.739465662522697
HCAR3	-0.123905584452576	0.70171967553804	-0.245097409390577	0.586282246055992	-0.232282333333333	0.264889721029722
UBE2D1	-0.123941674962323	0.701615347621749	-0.109286967645911	0.852399817890342	0.258523	0.174876267009578
TRAPPC5	-0.123959700770574	0.70156853103645	0.183112848122266	0.718067440349544	-0.126363333333333	0.534196070908831
STRA13	-0.123992381706753	0.701425205699229	-0.0616529751392908	0.923246185283376	-0.243562666666667	0.195247660560017
GAD1	-0.124076079063263	0.700946007305817	0.098061090375729	0.868852648210739	-0.914729333333333	0.171804245444023
ESRRB	-0.124078641740967	0.700946007305817	0.0938416813390622	0.875355472414883	-0.109566666666666	0.626149483603197
NEBL	-0.124096305827716	0.700946007305817	-0.293463064460749	0.480778950366873	0.0384160000000001	0.874464844016715
DCUN1D3	-0.124209456404946	0.700642849501428	-0.395376367562016	0.272692742937895	0.125366333333335	0.573673650964407
MLH3	-0.124229373649409	0.700583600333823	-0.526585055883102	0.0838370074507502	0.217645666666667	0.316427161487764
INSL5	-0.124281679411745	0.700383007195242	0.312202298017498	0.440385292108219	0.0789300000000005	0.737104221885809
ZFR2	-0.124308729408419	0.700276810968045	0.251949034982022	0.570584539772622	-0.220088999999999	0.4254947046787
FOXG1	-0.124321926528600	0.700261807577119	-0.309534807903913	0.447586697202665	-0.107881000000000	0.696451555016774
PP14571	-0.124338321293976	0.700225757127322	0.275314606545127	0.519365785652351	-0.0461109999999994	0.87338435621334
GPR56	-0.124402557411979	0.699946662766024	-0.232265516505965	0.612658112747496	-0.203631666666666	0.329908083538015
TBL2	-0.124487875599911	0.699528866424843	-0.200228251140309	0.681532337836957	-0.0175253333333321	0.948970566425317
FAM50A	-0.124498438627460	0.699528866424843	-0.332488707413473	0.398142341492687	-0.0152806666666664	0.95874970634395
CCNYL1	-0.124544596930447	0.699442983462467	-0.468463699610408	0.151936428121882	0.00906166666666706	0.982398683576954
BIRC7	-0.124625298146387	0.699177512183398	0.00512136011648905	0.99251028338798	-0.101263666666667	0.685271038967111
GJD2	-0.124645876474673	0.699177512183398	0.180616730643692	0.722518525037744	-0.0924233333333325	0.668580841735184
N4BP2L1	-0.124661654829646	0.699175667633296	-0.201584710924811	0.680054545720343	0.00924666666666715	0.974895360062947
LRRC45	-0.124689109593252	0.699101516100063	0.25960339518439	0.556472703411837	-0.196565999999999	0.326387191859404
MSLN	-0.124713150342247	0.699101516100063	0.0404152180737339	0.952720585102517	0.0430590000000014	0.893389698096825
LRRN4CL	-0.124742620435682	0.699101516100063	-0.32757166007782	0.40846136066764	-0.153751000000000	0.661470161772113
GCC2	-0.12486139094402	0.698651896697349	-0.289453768644616	0.488870852256136	0.154342333333334	0.443378989641463
ZNF852	-0.124880675659346	0.698651896697349	-0.3012252824216	0.464501110297982	-0.0420460000000004	0.902723025549103
UCHL3	-0.124958487414369	0.698372386522312	-0.0973973524751913	0.869861570992203	-0.118756666666666	0.576277303044571
LRRC16B	-0.124958812777347	0.698372386522312	0.241480122444681	0.595717891213491	-0.0372629999999994	0.9061074113295
MOGAT1	-0.124961305612858	0.698372386522312	0.0635136288369587	0.920855211745173	0.00872266666666709	0.982375192575954
RPTN	-0.124994415592217	0.698351680546187	-0.0917838683605281	0.879153312143409	-0.0650766666666666	0.785449444416478
NFE2	-0.125017054504279	0.698274597486289	-0.132453278050618	0.809866082339655	-0.185437666666666	0.496462982918528
CXorf38	-0.125041792159312	0.698183702698577	-0.0628264270708257	0.922290895114464	-0.381530333333333	0.0660125264725009
PLEKHA8	-0.125069433076944	0.698145639004657	-0.26784893967895	0.53559545997875	0.0687973333333353	0.791934055525056
FAM209B	-0.125107093669383	0.698041630878718	0.0429439671460839	0.950243031196165	-0.101991	0.677570340702693
C1orf168	-0.125129822233152	0.698009281533624	-0.0456402719015877	0.945663463098983	0.0280346666666669	0.926246672706072
ZNF835	-0.125136285460857	0.698009281533624	-0.195471905752601	0.694043352975239	-0.103342333333333	0.662828468002357
CPD	-0.125138433899171	0.698009281533624	-0.356264996586849	0.346087819515164	0.215112000000002	0.28487334684302
ME3	-0.125202875558829	0.697880464743099	0.0781407273178346	0.899652641295827	0.0978190000000007	0.648325366843053
C19orf60	-0.125254544438043	0.697862656205186	-0.00944525436192278	0.98909166696601	-0.0121160000000001	0.967695194723143
DYNLT3	-0.125265477322926	0.697862656205186	-0.0656223650881824	0.917809015101472	0.205386000000001	0.380711188899471
KRTAP5-2	-0.125359838224439	0.697601439933544	-0.144419563147337	0.790414495161718	-0.0241329999999989	0.938316557959043
KIAA0247	-0.125389767899536	0.69754841834009	-0.182017182641850	0.719880442043382	0.109763000000001	0.671766969198954
DSE	-0.125532026048933	0.696897286651041	-0.172042855918873	0.738897289340835	0.210046333333334	0.290781031227171
ISG15	-0.125616598252974	0.696415227326658	-0.315542819489224	0.434653173759935	-0.0391483333333331	0.87279470391545
IL3	-0.125673811059277	0.696182670874515	-0.0262885209258655	0.970631762839502	-0.0275719999999991	0.920633271571572
TFAP2E	-0.125682063999553	0.696182670874515	0.0103041831697546	0.988113617387568	-0.159785666666667	0.564757468001238
NINL	-0.125736948141389	0.695911161292238	-0.0309233208799086	0.96529904314994	0.0754320000000002	0.72802494241369
AFG3L1P	-0.125775500660552	0.695801408669718	-0.0482756001115527	0.942853417571722	-0.0455263333333331	0.849909082952122

FABP1	-0.125851727288239	0.695501838169025	-0.0814658002673314	0.89615326639288	-0.114787666666666	0.575076472049916
NPBWR2	-0.125859559139124	0.695501838169025	0.106359159310685	0.858284876027025	-0.0830273333333331	0.770112696597648
MTHFD1L	-0.125884314276764	0.695445278178477	0.138131770194960	0.800478820487963	-0.0122449999999991	0.973686596735315
SLC25A41	-0.125891574125086	0.695445278178477	0.0288149917958235	0.96782691865619	-0.171977666666667	0.502480362041859
MRC1	-0.125892695157132	0.695445278178477	-0.327335491929208	0.40846136066764	0.0417713333333335	0.862744795576892
TP53I11	-0.125901487262661	0.695445278178477	0.0721804780002154	0.908125883214154	0.0960376666666661	0.712601873916151
FDFT1	-0.126007424555780	0.695067110542222	0.308664832732945	0.449759364777049	0.0181366666666682	0.947592797551224
TIMM8A	-0.126060895733284	0.694859284749551	-0.503159996723275	0.109167411145190	-0.161738333333333	0.5683587002004
RPAP1	-0.126093100335771	0.694859284749551	-0.0713399772874452	0.909119908757507	0.0512996666666673	0.850157189407213
SLC35A3	-0.126129499063832	0.694735470336455	-0.364990701987097	0.331359658841643	0.0980566666666675	0.631961600239811
CCDC150	-0.126158803221631	0.694646995451288	-0.0159940450164187	0.982102356159698	-0.228120999999999	0.319387382047648
CXXC11	-0.126215530395632	0.694417788555655	0.0717225254142769	0.90838901602167	-0.130234333333333	0.688360595864485
MFI2	-0.126230648883188	0.694417788555655	-0.102121125932560	0.864337135688791	0.151271666666667	0.548138764076282
COX11	-0.126311085516481	0.694150335650663	-0.187569559149330	0.709135057556051	-0.0938676666666659	0.675449330308211
CBFA2T3	-0.126335922254921	0.694150335650663	-0.0830165170866052	0.893446201797764	-0.106109000000000	0.721919898836306
E2F4	-0.126375670958648	0.694150335650663	0.119107321076683	0.836828045978697	-0.125296999999999	0.536320838519271
RASGRP1	-0.126390633385460	0.694132836544359	0.0545299669181111	0.9344692810234	-0.0080706666666661[illegible]	0.98349947509937
THNSL1	-0.126420947006358	0.694132836544359	-0.222764088259429	0.635495054621817	-0.208997999999999	0.356860503105293
LOC388692	-0.126450599971997	0.694023301157811	0.0528758469553279	0.937009489777134	0.0576670000000009	0.865831575285905
CHEK2	-0.126542220484508	0.69356460372453	-0.206980556776307	0.669713208801491	0.0855770000000012	0.718749100405876
PDK2	-0.126549747368846	0.69356460372453	-0.332094756840021	0.399068746068573	0.0559170000000015	0.821451977498078
TNS4	-0.126639046536971	0.693071792256622	0.167784520366821	0.748242818061289	-0.0787049999999999	0.746404861999487
DAG1	-0.126691522557246	0.692935815024135	-0.291961072463412	0.484940697058018	-0.209834666666666	0.315612257978327
ACSBG1	-0.126709623245865	0.692935815024135	-0.148872019956181	0.783041584899534	0.0732603333333333	0.743387302966358
KCNH6	-0.126712196961373	0.692935815024135	0.112114601542728	0.847454473507881	-0.830124666666667	0.02445797919947
ADAM2	-0.126731433666451	0.692935815024135	0.145959349546356	0.787402233709509	0.00764433333333365	0.986471172041113
GRAP2	-0.126768390076692	0.692869087264702	-0.0123756545814360	0.98534346981395	-0.218076333333333	0.25721699788802
GUSBP1	-0.126784263429023	0.69286208891999	-0.156178437641492	0.768909201237015	0.098191666666668	0.637426515050706
CD74	-0.126787892738762	0.69286208891999	-0.507519285851023	0.103996171350646	0.0110696666666676	0.972261709812365
LETM2	-0.126798953974454	0.69286208891999	-0.308406273288229	0.450952555153602	-0.00194233333333335	0.995146554639975
VPS39	-0.126930048690636	0.692454054895207	-0.160356527285790	0.762217937284925	-0.0220263333333328	0.935966965628843
LCE1E	-0.126932430105669	0.692454054895207	-0.0449524852106883	0.947094297257154	-0.0884596666666655	0.787248874966057
ATP8B5P	-0.126945530171768	0.692454054895207	-0.0586034493339585	0.928586335857223	0.0651743333333338	0.794911513166032
MRPS26	-0.126986333519112	0.692372020943444	0.0579650386812164	0.929499310087992	0.100296000000002	0.649118055813479
GCFC2	-0.127008716905456	0.692296844127893	-0.105816210121332	0.85951926075622	0.308691	0.174033593336554
MUC2	-0.127021446626406	0.692285168646525	0.313700066278989	0.438046311563112	-0.0914286666666666	0.732086971105268
ZBED3	-0.127024443848094	0.692285168646525	-0.155311876254275	0.77058108906698	0.199247333333335	0.310277378475854
TECRL	-0.127135131518606	0.691865737368823	-0.127654708569915	0.818717515586031	0.0143840000000005	0.959218131095776
MRVI1-AS1	-0.127143157681380	0.691865737368823	0.0348618439609333	0.959404955370931	-0.0338446666666666	0.89671811772164
FAM49B	-0.127144528994857	0.691865737368823	0.0642906494693413	0.919923865590619	-0.135722999999999	0.516217427447249
GEMIN6	-0.127204085938239	0.691865737368823	0.065517462417859	0.918111672450639	-0.0432959999999998	0.871104061464043
ZNF285	-0.127278252503145	0.691604997479841	-0.36274935834118	0.337123031034334	0.0169586666666669	0.96469777383274
EFCAB5	-0.127278750884279	0.691604997479841	0.0145271242786367	0.983444353656142	0.180959666666667	0.344911970257145
IFI16	-0.127358090677765	0.691368214259075	-0.0816538517879976	0.895717565835764	-0.0244506666666667	0.937695109226553
ZNF259	-0.127364766705861	0.691368214259075	-0.425153245173154	0.215733128998344	-0.125079666666666	0.551352991056569
ZNF268	-0.127376071500597	0.691368214259075	-0.386775232126732	0.288207962791151	0.0324113333333342	0.907885119110266
ATP5A1	-0.127384618095713	0.691368214259075	-0.257508666173174	0.563116080558022	0.0523366666666681	0.821421133422134
FBXL22	-0.127388031668044	0.691368214259075	-0.0850350602310166	0.890809718139705	-0.196479666666666	0.543436323296458
TRPM5	-0.127419143785712	0.691368214259075	0.200718537006018	0.680618255541013	-0.0822159999999998	0.8125682765811
HIST1H2BH	-0.127481655260667	0.691132305236648	-0.176485200289854	0.730152680173573	0.107703333333334	0.637426515050706
VBP1	-0.127496518121304	0.691132305236648	-0.446277214049354	0.179597775907560	0.213960000000001	0.25470355352902
RGSL1	-0.127553204219397	0.690937441916763	0.0889974951909889	0.883836081896294	-0.0455425	0.850157189407213
TTC39A	-0.127611270717937	0.690712444920774	-0.0993141085934272	0.867933660937568	-0.0179680000000000	0.948382942196278
ATP1A3	-0.127611528838549	0.690712444920774	-0.127612645796192	0.818717515586031	-0.162439999999999	0.546219696447415
FREM1	-0.127640923770139	0.690661660595962	0.412269605935900	0.242722273788294	0.0821263333333337	0.708080688946526
LOC441601	-0.127653539007408	0.690650819938834	0.0115749400489034	0.986661750359244	0.0985683333333334	0.670113640649168
HTR3B	-0.127660036138112	0.690650819938834	0.136687600362686	0.804023390429745	0.0851623333333342	0.76409396614905
ASPG	-0.127678920324492	0.690650819938834	0.0198816304592176	0.977526645675126	-0.106112333333332	0.670113640649168
KRTAP10-12	-0.127707676027266	0.690650819938834	0.0171229017793546	0.98105020869911	-0.127630333333332	0.641391983149645
SLC16A3	-0.127738489887058	0.690650819938834	-0.0279238712985519	0.96912535812542	-0.093471333333332	0.721454370213253
SPAG11B	-0.127818300880510	0.690360748402953	0.164917211176873	0.75320079407004	-0.0027996666666661[illegible]	0.993756838831997
SECISBP2L	-0.127833403948556	0.69033357397711	0.0756916197632302	0.903510413986113	0.172273000000000	0.392990708193759
FLYWCH1	-0.127834501038947	0.69033357397711	-0.231133091486592	0.615929441239857	-0.145727	0.471749971867924
ACP6	-0.127951296503783	0.689918889746268	0.0672428937759271	0.914700759157169	0.0219720000000008	0.937717709824356
PKP3	-0.128054998132867	0.689381083314711	0.0740112491560029	0.905506084101768	-0.070078333333333	0.817124522613393
MYH7	-0.128159084973288	0.688985190067925	0.239696535102017	0.598516926091973	0.00322500000000005	0.993756838831997
LRRC19	-0.128223813430982	0.688776008202445	-0.0876330008460838	0.886302037299497	0.080748666666667	0.733933387497966
PRICKLE3	-0.128243972535710	0.688736882359778	-0.307522291453741	0.452689746287922	-0.0347529999999989	0.903663573715062
CST3	-0.128280133084254	0.688622141120947	-0.216781170183559	0.647357580911858	0.177276000000001	0.36523677764789
SCAMP2	-0.128290421221651	0.688622141120947	-0.235615594535585	0.6067637636079	0.191374666666667	0.320225451789804
CRYZ	-0.128307708717680	0.68858514029915	0.190248528877162	0.704208582824765	0.0184733333333340	0.946623914607177
MRPS2	-0.128434949992922	0.687892899728614	-0.105483016569843	0.85979009081468	-0.130985999999998	0.510761978128889
MARVELD3	-0.128484440353646	0.687727698012963	0.178386232614418	0.727147198636309	-0.095108333333333	0.677570340702693
ZNF16	-0.128486977506704	0.687727698012963	0.195996254493682	0.692325064566574	0.153313666666668	0.443378989641463
MNS1	-0.128677659322595	0.68721937888013	-0.381828045440613	0.296920946139734	0.283881	0.333031501097915
RUSC1-AS1	-0.128730047652705	0.687107430942243	0.119095359272832	0.836828045978697	0.0139143333333334	0.962924081933534
C20orf203	-0.128735873196293	0.687107430942243	0.174217286640117	0.734267769889068	-0.0785826666666664	0.760951989611575
TUBA1A	-0.128815669625152	0.686905446848326	-0.0492149402611224	0.941842562680095	0.0447433333333352	0.872930585008323
DYSF	-0.128822660955906	0.686905446848326	-0.301524835976677	0.463490113383679	-0.229051333333333	0.323239466490959
COX6B2	-0.128863476879584	0.686735550796904	0.0507963418280924	0.939978131035994	0.069907666666667	0.777406710539424

FANCA	-0.128876217522343	0.686723978702498	-0.0245192182273918	0.973562415846915	0.0410863333333348	0.878427136726667
NELL1	-0.128884334020035	0.686723978702498	0.124329000599083	0.825322945798765	0.0864883333333334	0.743456988551011
NR5A2	-0.128885843814595	0.686723978702498	-0.36451538766822	0.332851567373657	-0.118103333333333	0.741438256509195
EIF2D	-0.128905820963688	0.686723978702498	-0.0653174374534612	0.918295693698803	0.301481000000001	0.114599211633843
DDOST	-0.128955552853311	0.686635612330892	-0.290097456256991	0.488870852256136	-0.0464159999999988	0.85030886582909
PCP2	-0.129016040092921	0.68638239484696	-0.0890342095822059	0.883824851493414	-0.0172656666666651	0.952448105846014
SPIC	-0.12904419735069	0.68638239484696	-0.0605493226282596	0.925565513580637	0.413931333333334	0.0769043559304575
DSTNP2	-0.129053960341933	0.68638239484696	-0.0559407710652924	0.932919548410715	0.195323333333334	0.313220411537404
C11orf68	-0.12908307007727	0.686375053827508	-0.0106147211506736	0.98774587825743	-0.0944916666666658	0.672674698802727
C22orf46	-0.12918034911658	0.68602444960146	0.141344468199818	0.796285336930078	0.137921333333333	0.506814416082488
POU3F2	-0.129251918763576	0.68584290759649	-0.32320464009498	0.415461412498052	-0.0412236666666657	0.870489343487753
FLJ36116	-0.129266889677316	0.685816734643358	-0.126491864964174	0.821473247266105	0.200002000000000	0.421535331289692
NLRP2	-0.129307717952117	0.68576508726117	-0.0403759367713533	0.952720585102517	-0.0163299999999998	0.954815312836258
LCORL	-0.129474897778102	0.684900785854669	0.0218295035675705	0.97536722461972	0.289765666666668	0.156312939631849
KCTD13	-0.129477339560092	0.684900785854669	-0.0177484494675385	0.980264244976172	-0.0782796666666654	0.733867173296405
RBM3	-0.129731349350998	0.683818702671633	-0.239580053660815	0.598769383991889	-0.289286000000000	0.216591326893487
PAX3	-0.129762568290908	0.683787201079115	0.262569517887908	0.548594673068337	-0.141629333333333	0.558807324932864
SNX32	-0.129854536452795	0.683399723558191	-0.107560949618582	0.856850627832179	-0.105334333333333	0.664417498316816
EPDR1	-0.129866852154174	0.683391006288132	0.136779824022692	0.803725653241604	0.204858666666668	0.295467950792479
GBAP1	-0.129871049655896	0.683391006288132	-0.136019900785429	0.805641810268282	0.207788666666667	0.388602018395846
CD14	-0.129894039474145	0.683356798597616	-0.427280736284424	0.212299656428884	-0.112663666666667	0.668862273626329
PIPOX	-0.129902118372746	0.683356798597616	0.117996510872835	0.838500126709059	-0.0760239999999996	0.797408129996359
PTPLB	-0.129922739989922	0.683312666492836	-0.0604958134609992	0.925565513580637	0.0838726666666676	0.717087875531756
ZNF585A	-0.129956643087407	0.683293890703655	-0.256897961614527	0.563552879481391	0.164727000000000	0.43970147205246
ELAVL2	-0.129958696957532	0.683293890703655	-0.074168808006378	0.905380356355707	-0.109329999999999	0.730526498411145
LMO3	-0.130029963074603	0.683293890703655	0.169850591411591	0.743328907676018	-0.0745723333333327	0.733867173296405
PRDM12	-0.130090776113743	0.683220022269031	-0.181144888204161	0.721390952113546	-0.146450999999998	0.584434983272196
FFAR1	-0.130138739956256	0.68312170581624	0.212694426374583	0.656111996220908	-0.328815666666667	0.332110682491978
GRM5	-0.130175753384558	0.682950787464635	0.123906715684457	0.826287181959559	-0.0200593333333330	0.958900728767525
NEUROG3	-0.130272869595930	0.682457334427165	0.463295668323268	0.157837170342414	-0.311612333333333	0.173802457326336
ATP6V1E1	-0.130318059720118	0.682232779373109	-0.189164360376076	0.706450470188434	0.129680666666668	0.516217427447249
FOLR2	-0.130338812432224	0.682168721385755	-0.0925563080660638	0.878225904097636	-0.0986503333333334	0.662828468002357
CD63	-0.130348711274889	0.682168721385755	-0.184760778286541	0.716020884288485	0.127713333333335	0.523453039510001
PSG1	-0.130356459049867	0.682168721385755	-0.139411414507353	0.79853399315398	-0.0101543333333333	0.982375192575954
FLT4	-0.130358844386015	0.682168721385755	-0.0927256222263518	0.877797216649453	-0.0682086666666667	0.755697432495201
LINC00301	-0.130409120219915	0.682140440300944	0.114520887633672	0.844272085764738	0.0207483333333335	0.940769759007954
NAPSA	-0.130449870808601	0.681958466715684	0.158909756967047	0.76554422775177	-0.0112326666666665	0.972327702257384
FLAD1	-0.130486796851781	0.681919314394357	-0.250980089713220	0.572882168047464	-0.200497333333333	0.322893184569333
SDCCAG3	-0.130569862752127	0.681586437582039	0.282865853582564	0.502158515645595	-0.192156333333333	0.309421300309741
C1orf65	-0.130579074757781	0.681586437582039	0.408934137794246	0.249852262368516	-0.0881223333333333	0.79760358406525
RAD17	-0.130617065991796	0.681498143157741	-0.383686436264141	0.293447764611617	0.241319	0.220511040537878
UNC93B1	-0.130667411606981	0.681311270636141	-0.096830411261849	0.870711896934693	0.109616222222224	0.63279195353715
FAM120C	-0.130667778531815	0.681311270636141	-0.340903589275250	0.378272273903863	-0.0419936666666666	0.871104061464043
MRTO4	-0.130743039320552	0.68110371988182	-0.398177876706687	0.268375319490435	-0.476577666666665	0.1593236943391
TMEM91	-0.130751085835775	0.68110371988182	0.00342786893079851	0.994730189431381	0.0897220000000004	0.675449330308211
SCARB2	-0.130828736672492	0.680685547242822	-0.356124680793925	0.346256298606433	0.0828606666666668	0.702088376583556
CMPK2	-0.130831412219172	0.680685547242822	-0.293723366387177	0.480614908214975	0.130012666666668	0.580149967309238
ATP7B	-0.130843079153022	0.680685547242822	-0.166716688784839	0.750443995356965	-0.200551666666666	0.491065854797839
SPIRE2	-0.130869602414773	0.680634022898366	-0.0969721616328114	0.870463314702701	-0.0655309999999992	0.778083808586846
OBSL1	-0.130876089687261	0.680634022898366	0.318145100357727	0.430001165944905	0.0365480000000013	0.883795010653297
MARCO	-0.130898371041083	0.680589677882401	-0.0696137329948273	0.911313423064389	-0.137012000000000	0.533160929228493
FER	-0.130946628469769	0.68034511028374	-0.218349604627022	0.642971191969613	0.112431333333334	0.60814016771842
SYPL2	-0.130951612413812	0.68034511028374	-0.43211251232443	0.204259658997476	0.304718666666667	0.130834595594896
ZNF689	-0.130981423350210	0.68026120934696	-0.054793999091729	0.934303341981145	0.162155666666667	0.4423818632121
SLC25A35	-0.130999551946340	0.680214462442403	-0.261832617682044	0.550522174266833	0.212289666666667	0.266004202201482
S100B	-0.131161919267146	0.67929334144288	-0.103055762003542	0.862488288780321	-0.0816919999999997	0.768446434650364
WNT5B	-0.131191354786806	0.67929334144288	-0.210948539591345	0.660645714735488	0.183306	0.443378989641463
MAN1A1	-0.131199222280669	0.67929334144288	0.103305148998132	0.862065338914705	0.602352666666668	0.0273713913650908
ATP6V0C	-0.131430683355470	0.678253983107765	-0.272407454820260	0.526175116911121	0.159686000000002	0.428358173426632
CXorf21	-0.131451995253039	0.678253983107765	-0.229353286443782	0.620183562399927	-0.108300666666667	0.623357154900356
KCND2	-0.131564611967627	0.677807050544785	-0.328498089620928	0.406864543297514	0.281874333333334	0.309430307801264
TNFRSF25	-0.131564819701459	0.677807050544785	0.385973852986105	0.289470192870154	-0.0952146666666658	0.699705925827209
NRSN1	-0.131625142678832	0.67762665817675	-0.0386680859636892	0.9543445884646	-0.110634000000000	0.624595423091977
FITM1	-0.131660910368896	0.677539119302871	-0.164946147038447	0.753148845430278	-0.117656000000000	0.675449330308211
UTP14A	-0.131671923405905	0.677539119302871	-0.568528298095571	0.0459649573896877	-0.163559666666665	0.504556661551619
NIPAL3	-0.131750074470981	0.677273036636075	-0.361727363734732	0.338556116268406	-0.177900000000000	0.46297633209186
TCP10	-0.131792226941620	0.67718073213027	0.285762446112295	0.496740194039826	-0.104644333333333	0.703639844225278
PNMAL1	-0.131804972087523	0.677169353232024	0.121083508593995	0.83265693609154	0.0541910000000013	0.814814057921361
GEMIN2	-0.131846244032996	0.677115328492982	-0.268695784508033	0.535014466593144	0.136709000000000	0.494484578122119
HHATL	-0.131923853112882	0.676750892212455	-0.163851553785848	0.754916750929288	-0.084007666666666	0.762479823818089
PIGA	-0.131933311189435	0.676750892212455	-0.258481804494362	0.560093304159111	-0.176468999999998	0.62556031242715
C1orf186	-0.131941922424389	0.676750892212455	-0.05532949881715	0.933423044209716	-0.0753716666666662	0.822980493051179
LILRA1	-0.131988881847174	0.676613340763984	-0.0256427441245296	0.971891734131306	0.112225666666666	0.617967324583825
MC3R	-0.131994149574213	0.676613340763984	0.0625673156025107	0.922632631649811	-0.123835666666666	0.697592019153389
SLC7A7	-0.132062997784457	0.676416257308348	-0.275939580392440	0.517549833373363	-0.0300283333333324	0.92440022209385
PATE2	-0.132068458156089	0.676416257308348	0.0181149951142689	0.979375761502567	0.282948333333334	0.550904785888199
LRRC63	-0.132095948352646	0.676344657792586	0.350663970104932	0.357785279959936	-0.0681763333333333	0.795733806881408
EXOSC8	-0.132105492921307	0.676344657792586	0.0412414451806077	0.952098348256047	0.108658666666667	0.614081498359193
NACA2	-0.132113475062677	0.676344657792586	0.420796359972303	0.225408319488849	-0.0419319999999997	0.866671423175521
FOXH1	-0.132132518356300	0.676321565703656	-0.0504460489912251	0.94067156299105	-0.0530883333333329	0.874353540431336

TRIM68	-0.132180874357960	0.676076766978119	-0.302989167322854	0.462105017205825	-0.0617523333333333	0.819757926662461
TAP2	-0.132183109145431	0.676076766978119	-0.505151835262549	0.106552973154479	-0.0768768888888879	0.753182944057782
KPNA3	-0.132190674412891	0.676076766978119	-0.186128032190958	0.712651787801067	-0.551524666666666	0.0257919352862336
FAN1	-0.132215433985154	0.676076766978119	-0.309644318023693	0.447255805930312	0.0155110000000002	0.95531649368174
TMED6	-0.132284883839815	0.675828273471036	-0.140548877695968	0.79693828563394	-0.128013666666666	0.561449277320479
LIN28A	-0.132297238792317	0.675820355948717	0.0703224439481004	0.910394157189308	-0.251531333333333	0.180114381586694
KDELR3	-0.132300077875880	0.675820355948717	-0.267668435485503	0.535603758171522	0.155022666666668	0.580149967309238
ZNF347	-0.132372790929705	0.675613185475054	-0.411516510764525	0.243528138193926	0.164665333333334	0.547305665058354
AP2S1	-0.13240193447255	0.675494377776453	-0.137750907778055	0.801912855829954	0.172253000000001	0.525143015861964
APOL5	-0.132409429926549	0.675494377776453	0.0195372081156555	0.977673457110824	0.00239233333333396	0.994738356997797
PIK3R3	-0.132418757400692	0.675494377776453	-0.338320832106134	0.384701467775014	-0.233175666666665	0.241897450530544
RAD52	-0.132448397008857	0.675406542936044	0.0327554695964143	0.961351562552925	0.0172526666666680	0.9622178119912
RALGPS1	-0.132474523638525	0.675307523278567	0.0229358588453058	0.97536722461972	-0.0912069999999993	0.759162700398522
NFX1	-0.132486763777834	0.675299541131751	-0.312132695475857	0.440558341460703	-0.00249666666666653	0.993756838831997
SLN	-0.132512764304107	0.675202460791617	0.0075102946120681	0.99077339592597	0.00481300000000069	0.990408946313272
PHACTR1	-0.132518135460890	0.675202460791617	0.0369347580380911	0.956918184553224	0.0491233333333334	0.841976773189511
TMEM144	-0.132555623401065	0.675137187767881	0.0934716376975942	0.876319616688429	0.131541000000001	0.629710931024195
MMADHC	-0.132609082484239	0.674985796872349	-0.00545531263298393	0.99251028338798	0.225894333333334	0.224147588185176
SAP30L	-0.132645343477066	0.674838120156235	0.265029291307372	0.542328069427947	0.170690000000002	0.450203306181989
XKR6	-0.132649966674229	0.674838120156235	0.331869859773592	0.399068746068573	0.112092666666667	0.577269890738407
DEPDC7	-0.132655363693340	0.674838120156235	-0.408848449058086	0.249852262368516	0.183079333333334	0.377406302425587
LHX3	-0.132700796713640	0.674763734926109	0.0479740001277959	0.94314236584312	-0.0269943333333325	0.928203681921325
VCL	-0.132756706227427	0.674469516898462	-0.349795932176747	0.358977715627648	0.397773666666669	0.048170505761132
ZNF578	-0.132828123066994	0.674354643624273	-0.220468990884419	0.63932304081136	0.0507070000000018	0.90945379489823
PSMB9	-0.132863024085576	0.674206340588878	-0.63162429654041	0.0208242349154619	0.520661000000001	0.0273713913650908
TIMM44	-0.132897091363676	0.674127628966466	-0.128787824796629	0.815407702581246	-0.118324999999999	0.551125574415782
TBC1D16	-0.132948849835190	0.673860681135264	0.00881441266327568	0.98946753270812	-0.292782999999999	0.177094944003107
TMEM126B	-0.132976545784385	0.673751455020525	-0.402293178051221	0.259895916604029	0.00569033333333338	0.988133186272453
DMRT2	-0.133031925356889	0.6735331010416	0.0406558320483938	0.952426531595115	-0.0534769999999996	0.83321169515101
TLL2	-0.133099200329269	0.673309095284743	-0.263458458101202	0.545874639137454	0.217368666666667	0.338804872769334
ZNF449	-0.133112370404462	0.673295072298334	0.181274872721257	0.721196149497303	0.622724333333334	0.0262046791709735
SH2B3	-0.133115713046378	0.673295072298334	-0.430810138055862	0.206533702654214	-0.0744729999999985	0.752518458717797
C11orf73	-0.133134568595209	0.673295072298334	-0.0609532752359254	0.924945644657065	-0.109721666666666	0.631408228698652
WDYHV1	-0.133183675717318	0.673261572536351	0.317902913199132	0.430448648258555	0.119926666666668	0.672665644724356
IMPA1	-0.133185876223134	0.673261572536351	-0.0214327954374924	0.97536722461972	0.145349666666667	0.572873019944314
RAMP1	-0.133190776145566	0.673261572536351	-0.0901519722074743	0.882249601181978	-0.0670646666666651	0.873663396186202
HECA	-0.133301615279830	0.672737187795882	0.112471943138486	0.847284575037946	0.0949440000000008	0.657217907037306
C21orf58	-0.133306097571347	0.672737187795882	-0.0347903126954306	0.959404955370931	0.143897	0.543665540100043
GRIN1	-0.133317466231213	0.672737187795882	0.136127019658993	0.805596764001989	-0.0737976666666658	0.771373465365664
KRT19	-0.133327586511956	0.672737187795882	0.0256485409156615	0.971891734131306	-0.178241666666666	0.510372461062965
FLJ32756	-0.133387816117768	0.672574655318886	0.0424198268508846	0.951446425515852	-0.0675853333333323	0.761688891804975
FLJ13224	-0.133424913897087	0.672403898806423	0.0614064481638966	0.924016783210879	-0.071381333333333	0.763131964319038
OTOF	-0.133438015849394	0.672390363300619	0.0994176338564787	0.867651972215247	-0.0994879999999993	0.670071773006358
VKORC1L1	-0.133485402374714	0.672369160942727	-0.239285109877924	0.599780892198807	0.109051166666667	0.596371995531053
CSTL1	-0.133521561979955	0.672349242331076	0.0784124306683734	0.899281198071059	-0.026995333333333	0.915761046438965
ZNF430	-0.133618285509452	0.671947888808701	-0.0487911288849365	0.942180113539637	0.127724	0.600737808923323
YIF1A	-0.133679949081833	0.671745483302237	-0.176176413662769	0.73052229857415	-0.0297000000000002	0.911623909907545
FAM91A1	-0.133707505101349	0.671637286857974	0.249872572234373	0.575310770360099	0.065732000000001	0.775273488714107
ZNF395	-0.133739948022563	0.671569418792002	0.249859065099796	0.575310770360099	0.0190860000000007	0.95482028291900
SETD1A	-0.133743659433957	0.671569418792002	0.172964371648151	0.737510221688958	-0.110803333333332	0.590147690656472
SPSB1	-0.133773608258791	0.671493586421994	-0.44176061451934	0.186378962686717	0.196166666666667	0.313340135008504
LTB	-0.133848989932530	0.671286186904916	0.0274010443887618	0.970045676424287	-0.0671694999999983	0.871104061464043
CDK20	-0.133945135516620	0.670804028530349	0.293356728719511	0.480921323328224	-0.227014333333332	0.349094758813838
VCP	-0.133988108173373	0.670594924813141	-0.184390794527261	0.717243476899105	-0.0106833333333319	0.971595940539944
BTG3	-0.134079544764894	0.670140847108705	0.0815733631902612	0.8959407929359	0.176706666666668	0.442294185443201
GHDC	-0.134119040980628	0.669954580770996	0.00136844926063979	0.998320756202693	-0.155161000000000	0.458412878458348
GPR176	-0.134119113284093	0.669954580770996	-0.509018318300868	0.101906025861329	0.0957033333333338	0.660827808176898
CAPS2	-0.134207418167653	0.669665192765859	0.433441622551697	0.201555549804162	0.139327333333333	0.547839860241723
DNAH8	-0.134211895005954	0.669665192765859	-0.0827377348016288	0.893753725133293	0.0178483333333337	0.949296991322515
CCDC13	-0.134220951833998	0.669665192765859	0.106264209109890	0.85835076936337	0.0135513333333332	0.962620058410129
CLIC4	-0.134296759170761	0.669441818421977	-0.333904850203147	0.395819987028803	0.0362033333333341	0.88616294579686
LYN	-0.134329455440497	0.669300106368886	-0.568524018875988	0.0459649573896877	-0.0854276666666661	0.688516383064018
SLC15A2	-0.134346196573086	0.669262817824287	-0.375362628023756	0.312106257634910	-0.125091333333333	0.549323798856043
FBXO32	-0.134471002210930	0.668662960880318	-0.118578318136328	0.837522371512839	-0.304950333333333	0.4423818632121
HLCS	-0.134508309358866	0.668563377471649	-0.0647605317969272	0.918888051727773	-0.422557333333332	0.0647596980686847
HLA-DPB1	-0.134515269356041	0.668563377471649	-0.419889438770551	0.227417142008707	0.0337715000000012	0.89637298686676
USP53	-0.13453638013539	0.668524228169158	-0.348666931693825	0.361261311685225	0.0559290000000013	0.861677398924522
KCTD19	-0.134568447200536	0.668458947225405	0.279550198793641	0.509655886591018	0.0381380000000006	0.881624966098742
SP6	-0.134569192023990	0.668458947225405	0.165403942287625	0.752352242644389	0.0125316666666672	0.970094446214144
CGN	-0.134599329677742	0.668401427830757	-0.272171291538766	0.52716618152144	-0.141169000000000	0.516217427447249
SLC25A26	-0.134685126370938	0.667984690516311	-0.22689587722049	0.625901142430324	-0.210681999999999	0.282721762943589
RIPK1	-0.13471407335492	0.667939842453807	-0.218468001451503	0.642691336921378	-0.0810273333333325	0.721454370213253
FES	-0.134809420517485	0.667532976542791	-0.430139949461588	0.20761765766777	-0.0515946666666654	0.888319685310725
RCBTB1	-0.134826212743462	0.667495396116656	-0.154493765091359	0.771310656070265	-0.353407	0.15288400069506
NAT1	-0.134837478628138	0.667493958289643	-0.248570205111319	0.577442595482698	0.0901106666666672	0.718749100405876
VSTM1	-0.134857915846171	0.667471205445311	-0.0244320459333237	0.973567262803464	-0.0322479999999997	0.916061376873503
VWDE	-0.134918379835910	0.667181635107337	0.135075747462682	0.807470535721699	-0.217252833333333	0.347897317258212
QRSL1	-0.134925673381934	0.667181635107337	0.150490126323301	0.77904503981756	0.0944076666666667	0.699953171808848
GPAT2	-0.134942907433651	0.667181635107337	-0.258624275605803	0.559935260647793	-0.0503686666666664	0.882132095695824
BRAP	-0.13501981938973	0.666934431077886	-0.430098481871116	0.20761765766777	0.121871666666668	0.635069698575895

FER1L5	-0.135038444437494	0.66690224438639	-0.0201716420744753	0.976956866647027	-0.169419166666666	0.410984651167943
LUC7L2	-0.135047785100064	0.66690224438639	-0.306015097044928	0.457192231066199	-0.0219286666666657	0.93760967383883
CCDC30	-0.135051356012963	0.66690224438639	-0.256718423970824	0.564159017531156	0.128197000000000	0.575837271491686
UHRF2	-0.135079702350998	0.666849236630207	-0.100893824791713	0.866005093242444	0.156521000000001	0.514076428873743
SYT6	-0.135092166005549	0.666840003934976	-0.0714149522833031	0.909119908757507	-0.0325179999999997	0.922697964464038
SURF4	-0.135179587050814	0.666340665299368	-0.105126463383320	0.8606258296021	-0.112719999999998	0.578139856834541
LARP7	-0.135233308310129	0.666061743686319	-0.140534982465450	0.79693828563394	0.134915333333334	0.503217861354116
SLC39A3	-0.135272723568111	0.665948562496122	0.181725239002311	0.720487308022801	-0.0356809999999992	0.893208798828208
HHLA1	-0.135291782774396	0.665896213845679	0.0899058250992952	0.882796073669874	0.00450066666666689	0.988494624623319
RICTOR	-0.135387489188408	0.66537655626436	0.158104582554228	0.767373487136803	-0.0271249999999993	0.92469922248949
FAM76A	-0.135446513702696	0.665173972566108	-0.301284124089986	0.464457039761058	0.276885000000001	0.224147588185176
STXBP2	-0.135487720408479	0.665049136254173	-0.0622233297845408	0.92301259447339	-0.172996333333332	0.36541451620531
HIF3A	-0.135602438844971	0.664461591190621	-0.203536705456589	0.676207233014711	0.0109003333333336	0.975909178207253
CDHR2	-0.135617519408831	0.664461591190621	0.178016823301502	0.727982464250053	-0.0683593333333331	0.769634675887294
DNAJB2	-0.135686607326179	0.66432746675533	0.0537111621530702	0.935777496327359	0.0706003333333334	0.745849610477079
CATSPERD	-0.135724334918299	0.664225413752637	0.0685312945236866	0.912149839732916	-0.126618333333333	0.543807244026371
ST14	-0.135724915694267	0.664225413752637	0.0578692568276732	0.929715247340158	-0.197045333333333	0.460428849906031
CD69	-0.135871859367922	0.663695092511455	-0.353189763104450	0.352407344218756	-0.201589666666667	0.336373925530627
SDC1	-0.135876967229287	0.663695092511455	0.275984289339233	0.517549833373363	0.186358000000001	0.438162889121856
NIM1	-0.135965037413848	0.663303252116753	-0.0514679927449529	0.938728344711776	-0.0688316666666664	0.759862924283654
NNT	-0.135972345627284	0.663303252116753	-0.0190586353322244	0.97832242527852	-0.00071666666666579	0.99792238320329
SH3TC2	-0.136003400064841	0.663197117539022	0.0129545707845030	0.98463111107678	0.0762176666666673	0.761824584181416
RNF144B	-0.136029527983491	0.663098714557669	-0.527448986206151	0.082638567898698	0.114488500000000	0.598430627659442
ST6GALNAC1	-0.136088694988050	0.662856821515552	-0.0523902272929482	0.937831566264837	-0.0515766666666665	0.8754328609757
KCNE1	-0.136100203402704	0.662856821515552	0.0146168864821587	0.983444353656142	-0.159357333333333	0.469563017756053
ZNF606	-0.136327394388159	0.661804241358507	-0.00967614853458912	0.98909166696601	0.197953333333334	0.321214942566141
LIN7B	-0.136347446565014	0.661745531525532	0.00082268382737113	0.998745415362821	-0.0304136666666662	0.909574903593925
AUH	-0.136417416495167	0.661434990553551	-0.234206935291909	0.608813237170423	-0.129355333333334	0.519295998650134
OSBPL6	-0.136419937760970	0.661434990553551	-0.191432719420001	0.701635983922402	0.375177000000001	0.0908960628407462
MC4R	-0.136439301972418	0.661434990553551	-0.116622499484533	0.841809730226979	-0.0848459999999998	0.742452599762094
ZSWIM2	-0.136451641654249	0.661434990553551	-0.132226660723784	0.810300414153187	-0.093818333333333	0.682390613806982
FXYD3	-0.136454830877180	0.661434990553551	0.0339691972182783	0.95993706312687	-0.239198333333333	0.260859460677011
PTPN18	-0.136558749687882	0.661160367832982	-0.0938431941158575	0.875355472414883	0.130484333333334	0.657506201473314
CCDC135	-0.136595861522176	0.660990451404944	-0.179234352875528	0.725276394376351	0.00529633333333421	0.987937064305561
COPE	-0.136634977011706	0.660951643912699	0.071802225715683	0.90837606217316	0.0623110000000006	0.794486775271372
TMEM61	-0.136651939922061	0.660951643912699	-0.0382096873662649	0.95460795537904	-0.119787666666666	0.546434057395997
CD300LG	-0.136698486500597	0.660825898642853	0.210514601037448	0.661670522548494	-0.126858666666666	0.535539620005815
KLK14	-0.136746140887860	0.660659359306797	0.122099656022069	0.830022997302667	-0.0352123333333332	0.896906769316595
RAVER2	-0.136822275287161	0.66023507150418	0.221579889369284	0.637480881894777	0.159884333333335	0.463362442026763
NCKAP1L	-0.136835897717556	0.660218344084591	-0.382403096245980	0.295293127580539	0.152118666666667	0.442294185443201
ZIC4	-0.136849836575403	0.660199557485678	0.248452056172897	0.57746120283043	-0.0997656666666659	0.776805598353461
PIGV	-0.136931758321	0.659809722769369	-0.144156730290589	0.791303782903891	0.255456	0.220290410040458
DUSP27	-0.137003264726606	0.65948784249837	0.0786683184350185	0.899281198071059	-0.122724333333333	0.590841338570121
HDAC1	-0.137003744594091	0.65948784249837	-0.133960841108091	0.808567812988011	-0.0600600000000001	0.785476747850623
NMI	-0.137042959960872	0.65948784249837	-0.244204687563724	0.588660529799743	0.355215000000001	0.118223789682607
CFD	-0.137078230244927	0.659474671425858	-0.0988447358318207	0.868042453226125	0.0293716666666677	0.914375586072641
PODXL2	-0.137137194429094	0.659263634669139	0.196388286290229	0.691445158350448	-0.0169536666666661	0.950980037243398
PSG9	-0.137142029120735	0.659263634669139	-0.0794473540538108	0.8982764907534	-0.0679153333333324	0.7920412344971
MPPE1	-0.137171479731188	0.659184368178927	-0.106488676353974	0.858072018696671	0.0315220000000006	0.917325389804968
ZNHIT3	-0.137338780478327	0.658323869287807	-0.0511897867421199	0.939111418302491	-0.177653	0.442294185443201
IGLL1	-0.137413813267296	0.658038000020068	0.151054329307721	0.778007072419094	0.00771916666666719	0.986350009478474
PSMD11	-0.137439500152672	0.658014761349915	0.00572311978716686	0.99251028338798	-0.176720000000000	0.364895038300859
SPRYD3	-0.137499318732686	0.657697267377176	0.0271370324668181	0.970207612777188	-0.304838333333332	0.135004030972507
CENPN	-0.137502016539478	0.657697267377176	-0.0790888049943258	0.898931312896838	0.127779333333334	0.523453039510001
ST6GALNAC6	-0.137515520785744	0.657697267377176	0.0479534432919103	0.94314236584312	0.124687000000002	0.538592208738828
EIF2B5	-0.137531537121477	0.657697267377176	0.171204681693927	0.739841869018052	-0.0610903333333321	0.785476747850623
STX10	-0.137569129551275	0.657530768368233	-0.0233219530084845	0.975348968561756	-0.0688606666666663	0.764848773122078
KMO	-0.137576507657021	0.657530768368233	-0.254035061453736	0.567694846153007	-0.019772333333333	0.944239514412068
PROM1	-0.137577802480075	0.657530768368233	-0.199493772663757	0.683755152819083	0.0320983333333336	0.900467460403227
SOX7	-0.137578920129525	0.657530768368233	-0.189688304783941	0.704928430617595	-0.239357333333332	0.411784621423647
CAMK1D	-0.137724565704556	0.656950923325352	0.0409890367818009	0.952426531595115	-0.405702666666666	0.177094944003107
DNAJB6	-0.137738067026095	0.656935040945278	-0.286305891724509	0.495728580458409	0.0955260000000008	0.709937184587543
MUT	-0.137790163208747	0.656667967055491	-0.183202629564192	0.718067440349544	0.0302943333333334	0.906350383359944
BATF3	-0.137851966818071	0.656337782542116	-0.344510702626525	0.37069795553645	0.0649903333333334	0.85272965579086
SFT2D3	-0.137871935053892	0.656279820980852	0.298228548943290	0.47177769150751	-0.00701233333333327	0.983159241812861
OR2B3	-0.137876996245988	0.656279820980852	-0.206472886629087	0.671220485448239	0.0707766666666666	0.774055839452275
TLCD2	-0.137897043436854	0.656260380020865	-0.121182020302239	0.83256316766566	-0.122422999999999	0.646590194500551
SENP5	-0.137909286167325	0.656252692203676	-0.220093336460581	0.64064333199733	-0.137585999999999	0.520846786113276
DNAJB1	-0.137921283249171	0.656252692203676	0.0547912676414255	0.934303341981145	-0.0439183333333326	0.871104061464043
COL7A1	-0.137934042651133	0.656252692203676	0.421681051607408	0.222348587392621	-0.128674000000000	0.577524803794493
KIAA0556	-0.137947897687004	0.656252692203676	-0.112724260132951	0.847051776433405	-0.0618179999999985	0.794404527150583
ESPNL	-0.138054706865488	0.655954373513459	0.255668396869203	0.565656702091657	-0.0139919999999993	0.962962781493936
MTFMT	-0.138057404003099	0.655954373513459	-0.109027208838788	0.853032135925272	0.145886333333335	0.462444913819465
GJB5	-0.138149307729447	0.655626994541745	0.145419918407920	0.787896872604863	-0.164962333333333	0.458006502146897
CYP2R1	-0.138175851810290	0.655598166389116	-0.218428640249013	0.642707776478228	-0.0160433333333330	0.973783155960644
TTLL9	-0.13820953415115	0.655523046105068	0.0856866053683673	0.889114985071002	-0.126339333333333	0.610847413222267
COL8A2	-0.138241904828071	0.65538444446077	-0.0981471651845566	0.868852648210739	-0.132780333333333	0.62979571408014
ASPHD1	-0.138270714808215	0.65526901923179	-0.164234694515346	0.754189116283047	-0.0558023333333337	0.82700027112124
GML	-0.138294202311851	0.655188227587855	-0.00183073052681004	0.99760061742039	-0.0505786666666661	0.887363245747406
ELL	-0.13829942536814	0.655188227587855	-0.201630380319107	0.680054545720343	-0.11059	0.62979571408014

RREB1	-0.138321472109177	0.65515485309164	0.207030492533136	0.669713208801491	0.137661666666666	0.566282142073858
KIF27	-0.138370785390268	0.654941206070384	-0.271872309306336	0.527403246687446	0.141908333333334	0.528890172622518
COL5A3	-0.138393985250633	0.654941206070384	0.0693881396988768	0.91172160664395	-0.131478000000000	0.580149967309238
FLJ22184	-0.138403439595043	0.654941206070384	-0.0713989047687441	0.909119908757507	-0.169059999999999	0.438307351749832
IL21R	-0.138427038023248	0.654934455272035	0.0318367249193871	0.963211116393051	0.131929000000000	0.513141966218084
HES2	-0.138433835023354	0.654934455272035	0.126498914279538	0.821473247266105	0.0203063333333346	0.954651715081705
C1orf63	-0.138465668695647	0.654934455272035	-0.340512549662443	0.378350877728096	-0.0240049999999996	0.948768037250156
CACNG6	-0.138479950187199	0.654934455272035	0.0220870044859190	0.97536722461972	-0.0130356666666668	0.974484855799046
MEIS1	-0.138488280166279	0.654934455272035	0.0963424137650209	0.87156820165807	0.341670000000001	0.217295976105745
PMS2L2	-0.138526845592712	0.654934455272035	-0.39609639342477	0.271439858675643	0.116695000000001	0.567291668977137
NMRAL1	-0.138539195808423	0.654934455272035	-0.324916321509648	0.410909153213675	-0.0336986666666656	0.913644671932166
TSPAN15	-0.138568969230234	0.654913819476455	-0.156830591649012	0.768401006769299	-0.0551619999999996	0.822657709336468
MZB1	-0.138602224938141	0.65487974852008	-0.110197679967938	0.850412437492421	-0.147587000000000	0.544685122345675
FAM211B	-0.138681827610884	0.654467775823729	-0.0216132990076246	0.97536722461972	-0.183121666666667	0.563378898293951
GOT1L1	-0.138723388849108	0.654467775823729	-0.0371294879287579	0.956363028822848	-0.174470000000000	0.611770847276966
KIAA1244	-0.138774032302245	0.65422837894744	-0.0280654893463610	0.968697939346434	-0.124695666666666	0.588378465070572
CERS3	-0.138906823531459	0.653508618902525	-0.0297210025488181	0.96695070705759	-0.0734853333333324	0.816134952712854
ITIH5	-0.138922767283089	0.653476998262714	0.282157644840325	0.504088721698071	0.00331133333333344	0.993756838831997
CHI3L2	-0.138944616384026	0.653406969232019	-0.0703058006002843	0.910394157189308	-0.0427739999999997	0.883739719585572
CSH1	-0.138992608840309	0.653166914060907	0.311280051485932	0.443258400646928	-0.00158866666666608	0.99628178244892
MOGAT3	-0.139021650736345	0.653050121585518	-0.0335383220175116	0.960782399177614	-0.0580106666666665	0.846457250180447
ACSL3	-0.139071952549096	0.652795106191784	-0.060331243888028	0.925649366494154	0.123601333333335	0.544685122345675
NABP1	-0.139135577240278	0.652453536500975	-0.103266468750917	0.862136056890247	0.615107	0.0656979579234737
C6orf195	-0.139146355729919	0.652453536500975	0.31368723143115	0.438046311563112	-0.279648666666666	0.13704666630053
IL12RB1	-0.139149824431869	0.652453536500975	-0.160797957775733	0.761016253411733	0.0384696666666669	0.903352632801889
ACRBP	-0.139168914618203	0.65245291935063	-0.107625366168157	0.856638088483079	-0.0107053333333325	0.971769349205369
TRIM41	-0.139228096862349	0.65214027641347	-0.0803902650031561	0.896965035803767	-0.0649976666666662	0.799372147044399
POC1A	-0.139239386617992	0.65214027641347	-0.0405916957730615	0.952426531595115	-0.0823196666666655	0.723993424337833
ZNF18	-0.139306848306274	0.651844538996729	-0.260900343296973	0.553100551096796	-0.0616426666666657	0.786118160890663
LAMA5	-0.139307336663821	0.651844538996729	-0.316623979767763	0.432178413930141	-0.00510866666666508	0.9879739922351
PLA2G2D	-0.139319238487506	0.651844538996729	-0.224652918618484	0.632376156419106	-0.161678666666666	0.52128449277411
S100A13	-0.139367776041993	0.65173667313611	-0.416280232783945	0.234677826887939	0.261892333333334	0.220405584107617
CYP7B1	-0.139450985051839	0.6514353909158	-0.345990956292034	0.366044985124016	0.0785466666666676	0.794533800092385
RHO	-0.139563828906064	0.651173977767592	0.0160525942247044	0.982057247916055	-0.107148666666666	0.74238434992823
ING2	-0.139631972463535	0.651035132051217	0.105648943561223	0.85956218633532	0.412695	0.048170505761132
STAMBP	-0.139646398411001	0.651035132051217	-0.133358998893787	0.808992949393043	-0.0684089999999998	0.760951989611575
CAMLG	-0.139682257089242	0.651035132051217	-0.177938776717019	0.727982464250053	0.0317170000000004	0.91492899794829
COPS3	-0.139701215793942	0.651035132051217	-0.148724406041505	0.783041584899534	0.0201676666666672	0.942092082409632
SHISA9	-0.139719339741033	0.651035132051217	0.0232604462692973	0.975348968561756	-0.223718666666666	0.309421300309741
SKIDA1	-0.139722743157443	0.651035132051217	-0.30024085030331	0.465914681155138	0.107036333333333	0.625510328921698
ZFYVE21	-0.139729594078869	0.651035132051217	-0.284773908942904	0.498662021974229	0.0749010000000004	0.74499160363205
C5orf30	-0.139753619408863	0.651035132051217	0.248188788781515	0.577859441890662	-0.00300233333333284	0.99529569362957
RCSD1	-0.139761212614296	0.651035132051217	-0.408483831810029	0.250484125644429	-0.0150863333333327	0.971208229448032
MCAT	-0.139778087135463	0.651035132051217	-0.259889174894386	0.555728240771379	-0.152240666666665	0.441909622750662
MYBPH	-0.139786902721150	0.651035132051217	0.0520787333586622	0.938067483409541	-0.186714333333332	0.422927358730594
UPP1	-0.139799642631946	0.651035132051217	-0.284938591828974	0.498464716905649	0.0113180000000008	0.971870692701632
PTPMT1	-0.139834382088403	0.651010981947381	0.0989017281944603	0.868042453226125	0.0133450000000006	0.964880128671703
FAM189A2	-0.139837085774728	0.651010981947381	0.130904102991817	0.812271254601992	0.341172666666667	0.110806424945014
WAS	-0.139846640153120	0.651010981947381	-0.00589330051942405	0.99251028338798	-0.0670846666666663	0.789105818888855
TXNDC16	-0.139894165503767	0.650921837404149	-0.139977478072460	0.7976681602188	0.0451070000000021	0.879265091272724
P2RY6	-0.139934814043268	0.650863362100047	-0.28612678791029	0.496429042723156	-0.185183999999999	0.623070283203198
C4orf33	-0.139943190978893	0.650863362100047	-0.172258267291762	0.738567943867944	0.326231666666667	0.120066554405991
PPP1R16A	-0.139946196271400	0.650863362100047	0.159086544749964	0.765296051164558	-0.126929666666666	0.5293518047673
ASNSD1	-0.139980311998755	0.650856438167996	-0.00367352244605789	0.994588964700772	0.0311623333333339	0.908473154502781
TMEM187	-0.139997403284658	0.650817530714047	-0.260881060416005	0.553100551096796	0.185406000000000	0.366576358704214
QDPR	-0.140052536836298	0.65060344582742	-0.305300213803523	0.458013550008763	-0.00908966666666641	0.981191596773742
IFT80	-0.140214268957567	0.649901107789831	-0.032775611333151	0.961351562552925	0.0892836666666676	0.707538975864338
CYP19A1	-0.140218471891903	0.649901107789831	0.10361250328438	0.861872724387044	-0.0819023333333331	0.74808495490855
TMEM11	-0.140222340128396	0.649901107789831	-0.0404189023994358	0.952720585102517	-0.0791486666666672	0.729541086266503
ZXDA	-0.140229716539230	0.649901107789831	-0.352939578422537	0.352407344218756	-0.0388063333333334	0.880427463662501
VAT1	-0.140235818089287	0.649901107789831	-0.177935551019916	0.727982464250053	0.238832666666667	0.274121181062751
HTR6	-0.140275913246485	0.649901107789831	0.0663001144415747	0.916518252419605	-0.185774666666666	0.498454352317107
FSCN2	-0.140300531523991	0.649901107789831	0.284096620369549	0.500557068353025	-0.133549666666666	0.774210549173745
UHRF1BP1	-0.140306525732922	0.649901107789831	-0.55075506854189	0.0603608035588276	0.0301983333333349	0.921546617328678
MRPL17	-0.140417291957089	0.649604578149754	-0.0572291848158269	0.93071299016553	0.0261903333333345	0.937545603686416
RAB11FIP3	-0.140417476531937	0.649604578149754	0.161814190420354	0.758930446817487	-0.120524666666666	0.572207354314478
AQPEP	-0.140458470791100	0.649485591155604	-0.0203659524799593	0.976488095728823	-0.103320333333333	0.623988109816431
NR3C2	-0.140489484884087	0.649485591155604	-0.082610668790207	0.893753725133293	-0.375528333333332	0.0850992125511933
PCDH19	-0.140529505135187	0.649308707427012	-0.1994885401542	0.683755152819083	0.0201393333333338	0.954180927004992
AGBL3	-0.140538798667458	0.649308707427012	-0.17252906374634	0.738421727152922	0.238325666666667	0.266896890079960
LHFPL4	-0.140696814544469	0.648886455532056	-0.0536372634046865	0.93578205440935	0.0757730000000009	0.777568084061469
FURIN	-0.140727922807725	0.648852011402849	-0.0875143191801588	0.886302037299497	0.109900666666666	0.692043732062832
MAP1LC3C	-0.140735144596467	0.648852011402849	0.063461808715688	0.920855211745173	-0.319098666666666	0.121660357743656
AACS	-0.140749995300823	0.648852011402849	0.264812973577707	0.542751754568497	-0.0229906666666667	0.941319092333563
TAAR2	-0.140787939468533	0.648852011402849	-0.0956072138389812	0.872684476951248	-0.0369169999999995	0.890511682678135
TMCO1	-0.140792477344145	0.648852011402849	-0.0312565724410899	0.96494188520489	-0.232409999999999	0.256715815454135
PRR5L	-0.140882779370308	0.648440657327192	-0.142127757631872	0.79450771779567	0.123239333333333	0.604966458785844
NMNAT2	-0.140954759217831	0.648296979316259	-0.0142246833702898	0.983444353656142	-0.143865999999999	0.503217861354116
MLPH	-0.141009614407093	0.648067846442444	0.0253940101652194	0.972264538231849	0.0652493333333338	0.809755698910303
TREM1	-0.141021134074664	0.648065332151138	-0.0734765263111343	0.905838142287595	-0.119889000000000	0.643792824295256

MTERFD3	-0.141031703478022	0.648065332151138	-0.00733272388477462	0.990873555460625	0.171738666666667	0.441366313859876
C11orf71	-0.141058330811941	0.648040662604292	-0.0976378362025003	0.869414326288135	0.189677666666667	0.316090386778478
IL1R2	-0.141128965226122	0.647796540688829	-0.323986465401410	0.413920364055098	0.101292000000001	0.742665333187322
SH3BGRL	-0.141132461427855	0.647796540688829	-0.592145517092782	0.0355984111824064	0.348900000000001	0.0824998900297137
TRIM69	-0.141190438803298	0.647543651531973	-0.267632310874518	0.535603758171522	-0.0100069999999994	0.973686596735315
TNFAIP8	-0.141215504148278	0.647453085029048	-0.182702662556395	0.718559738568628	0.100177000000000	0.746473454296225
ZNF704	-0.141232610924070	0.647414267619477	0.0668785790869813	0.91517239775041	-0.324482333333333	0.151776694305005
CCL20	-0.141341578490813	0.646995468573846	-0.314608493455039	0.437067919687049	0.0369673333333332	0.891339318634358
ATP8A1	-0.14136767860888	0.646969381693755	-0.158896871997266	0.76554422775177	0.207801666666667	0.442398656490212
ZCCHC17	-0.141448416655623	0.646663002500123	-0.284470978422038	0.50019549608092	-0.179214666666666	0.524845938106282
TMEM198	-0.141516368428726	0.646438550607292	0.0260078813910368	0.970840789910547	-0.178230333333332	0.542447274777
ZMIZ2	-0.141566978472025	0.646254435343745	-0.139032314696870	0.79864602540281	-0.0962513333333323	0.665288826265065
HCK	-0.141569774645760	0.646254435343745	-0.401068617520203	0.262127055693923	0.0500823333333334	0.899614658619676
DDX60L	-0.141582028014819	0.646254435343745	-0.182832611923497	0.718289071039953	0.381382666666668	0.140860001683260
PPA1	-0.141683619914471	0.645786074205067	-0.0968176686819721	0.870711896934693	-0.0147903333333331	0.959032046952242
ACBD5	-0.141702969041741	0.645786074205067	-0.0623397216275369	0.922906018065302	0.273126666666668	0.178162638616424
UTS2R	-0.141719434268804	0.645786074205067	-0.115289909397230	0.843755596233057	-0.0577183333333333	0.88620514629877
GNL2	-0.141732099162006	0.645786074205067	-0.269663794029913	0.531924063959685	-0.201283333333333	0.321755185134160
FAAH	-0.141759842874561	0.645725189044905	0.318506100971968	0.4290520704081	0.133210333333333	0.514831164533638
CAGE1	-0.141876929782794	0.645109323825881	0.0172832398110145	0.980723199123135	0.0956300000000007	0.652989236147115
RNF186	-0.141885357431366	0.645109323825881	0.00604543354595977	0.992455290816664	0.00546400000000113	0.987937064305561
WFDC10A	-0.141908351733004	0.645109323825881	0.0229536370256894	0.97536722461972	0.0609416666666665	0.895093529187836
DLGAP3	-0.141924221752115	0.645109323825881	0.0362328264334923	0.957865652225373	-0.0207116666666661	0.951723450594248
C5	-0.141932640841156	0.645109323825881	-0.060232498113818	0.925649366494154	0.703885000000001	0.0173248876443729
CCDC113	-0.141961829189906	0.645109323825881	0.0175815283015596	0.980428114126044	-0.221815333333332	0.4254947046787
C4orf27	-0.142010407839699	0.644966238743228	-0.225907165220056	0.629121375721303	0.126965000000001	0.5531802635914
PARP8	-0.142037295349349	0.644863990753516	-0.486729384497001	0.130950940658109	0.0286376666666672	0.947455054282258
SLC9A1	-0.142058289328355	0.644863990753516	-0.492435466217334	0.121640558692374	0.127756000000001	0.578893615511221
F9	-0.142114083485212	0.6447997737538	-0.202574285409133	0.678404399740367	0.0302270000000003	0.91143191097679
SMAP2	-0.142145294349758	0.644741287801228	-0.281812300618152	0.505544023338075	-0.172426333333333	0.3649510393715
TPSB2	-0.14214876794984	0.644741287801228	-0.268236899848896	0.53532907159569	-0.237833166666666	0.469591696339734
PPP2R3A	-0.142174831896268	0.644694948150608	-0.47475473771093	0.144197096534399	0.420969000000001	0.0619237323392494
RGS19	-0.142195755556137	0.644631474184758	-0.128616242165283	0.815811316696798	-0.0804456666666664	0.721454370213253
MGAM	-0.142215469428500	0.644575864656545	-0.227500728070103	0.62539640207912	-0.110736333333333	0.583036318848746
TYW1	-0.142240851536520	0.644483428214332	-0.184992996269067	0.715658781310868	-0.144697000000000	0.461494497197158
EFCAB3	-0.142242319089057	0.644483428214332	-0.0121727288587001	0.985724447971216	-0.0434006666666661	0.870662337224757
HNF1A	-0.142256357022442	0.644483428214332	0.310765023716901	0.444561109519731	0.159205333333333	0.535171605667144
NRP2	-0.142289160192683	0.644483428214332	-0.155628721017804	0.769837060156515	-0.160767666666666	0.659124451108183
ARHGEF38	-0.142289863611268	0.644483428214332	-0.107651028080465	0.856590369736358	0.00730733333333357	0.983159241812861
MPP3	-0.142311371222954	0.644483428214332	0.117269716976664	0.840330543440358	0.0235683333333334	0.94367036893426
CD53	-0.142360832196886	0.644483428214332	-0.441002005779454	0.188105704960787	0.0129726666666676	0.967241125442187
HLA-DQB2	-0.142360839087506	0.644483428214332	-0.227025407905155	0.62581495163125	-0.106537499999999	0.626806418302655
ZMIZ1	-0.142424328348031	0.644379096909145	-0.0839308787211249	0.892716669164583	0.186345333333334	0.460374458843217
C12orf5	-0.142564956547072	0.643682825882654	-0.143914397032919	0.791730335952443	-0.247276666666666	0.355594274905286
PSMD1	-0.142585413740213	0.643622450284105	-0.21411580613761	0.653042977738486	-0.0400883333333327	0.871104061464043
IRF8	-0.142592688656793	0.643622450284105	-0.380015194746023	0.300906670900397	-0.192009666666666	0.426518032099216
CDH26	-0.142627383711860	0.643494850948184	0.00236452546446235	0.9961452626501	-0.0843843333333332	0.718749100405876
NRBP1	-0.142662604327832	0.643338570797861	0.0574760534347348	0.930306346936950	0.013871	0.96469777383274
APOLD1	-0.142775085945847	0.642753036025127	-0.171613777105005	0.739411494892728	0.149670666666667	0.532604205131631
TOM1L2	-0.142851385842474	0.64240260768023	0.38755178357149	0.286316430333128	0.0468958333333332	0.849384482495249
C8B	-0.142898900303305	0.642166640586172	0.0806881568625343	0.896621311541396	0.0340426666666671	0.90945379489823
SAP18	-0.142960537060529	0.641983940243566	-0.443400803094194	0.1843534401377	-0.0381766666666663	0.877597838720525
NT5C2	-0.143027271074855	0.641629352954776	-0.0126543530424239	0.985214990982244	0.00835766666666787	0.98025379113353
TTK	-0.143032495029803	0.641629352954776	0.0215596886185654	0.97536722461972	0.159416000000001	0.492100665301584
CCDC11	-0.14304071695109	0.641629352954776	-0.0178704626050849	0.979959907954606	0.0204726666666674	0.946623914607177
UBE2A	-0.143050527645001	0.641629352954776	-0.589384084496219	0.0371136262345896	0.285208666666668	0.142821378450256
GPHA2	-0.143052509857911	0.641629352954776	-0.0148716146561352	0.983111125676367	-0.0760496666666654	0.75868822136318
LOC152586	-0.143085877217714	0.641629352954776	-0.41151290508366	0.243528138193926	-0.0749633333333333	0.743291289804212
GLMN	-0.143090002538759	0.641629352954776	-0.15738824349655	0.767770230680061	0.0199760000000013	0.946392308016584
ALG5	-0.143138650678645	0.641629352954776	-0.0988719500680445	0.868042453226125	0.0393086666666676	0.899614658619676
PHF11	-0.143217793692423	0.64140140648204	-0.0297282208706667	0.96695070705759	0.220542000000001	0.312084999110155
CCDC114	-0.143222565823689	0.64140140648204	-0.201557081814045	0.680054545720343	0.164279000000001	0.516901822735967
EMR1	-0.143224880154002	0.64140140648204	-0.235427682858670	0.60678764664537	0.0330706666666668	0.919891803741744
LMX1A	-0.143252973102255	0.64140140648204	0.0238368614515448	0.97479225206569	-0.198274666666666	0.406200303517967
AZI2	-0.143259962871599	0.64140140648204	-0.0468862752889222	0.944274559688373	0.362448666666668	0.0656979579234737
DUSP2	-0.143332837723861	0.641234866008731	-0.206284920407270	0.671467763213228	-0.157310000000000	0.535098742175357
UROS	-0.143354889152414	0.641164266403621	0.212428919910285	0.656785033957219	0.199298000000001	0.341661960881399
KBTBD12	-0.143360057868934	0.641164266403621	-0.1642151362674	0.754189116283047	-0.0328009999999999	0.917325389804968
TOP1	-0.143364321774761	0.641164266403621	-0.405545574077161	0.25473834018135	0.0945446666666683	0.675099159639102
ZNF691	-0.143400024348246	0.641088939967479	0.109657942167943	0.851352116283305	0.091131	0.719839702715467
SACM1L	-0.143409861680961	0.641088939967479	-0.191115038331107	0.702165449732036	-0.0294296666666657	0.907615510059205
NPAS4	-0.143462683729757	0.641088939967479	-0.0267255920496779	0.970207612777188	0.0127746666666676	0.97229794993378
ZNF524	-0.143462741315867	0.641088939967479	0.117859174823867	0.838686136084666	-0.0696309999999989	0.786970336786627
ZMPSTE24	-0.143464428673434	0.641088939967479	-0.175141722469134	0.733192115929191	-0.034073333333331	0.890511682678135
CCHCR1	-0.143481097959654	0.641088939967479	0.0867819585220612	0.88739776018523	-0.191397333333332	0.314112513261693
SBNO2	-0.143490789144837	0.641088939967479	0.185429575510379	0.714427536588157	0.0265070000000009	0.918952691262331
CHMP2A	-0.143577704310115	0.641088939967479	0.147822459897785	0.784363140818881	0.0106603333333348	0.976469566022416
SLC13A5	-0.143594163306338	0.641088939967479	0.174857651976552	0.733598828800337	-0.320871333333332	0.207514442292398
TRNAU1AP	-0.143639752965201	0.640912233896752	-0.351928879612482	0.354396334441796	0.0266626666666671	0.92484471634727
KRT18	-0.143653582637491	0.640912233896752	-0.500749560712837	0.113169357904212	-0.344837333333333	0.263053396870868

RPAIN	-0.143684142172084	0.640842006908658	0.0381929662545792	0.954615297084618	0.0839576666666667	0.721931888873006
MGAT1	-0.143685301487916	0.640842006908658	-0.283608602683724	0.501852406168029	0.0351090000000004	0.89052375260746
FKBP6	-0.143686153079708	0.640842006908658	0.0718486432866574	0.90837606217316	-0.289693333333333	0.146245958681541
UBQLN1	-0.143787099210645	0.64046407017727	-0.0810545504363316	0.896361013063453	0.0152266666666666	0.957243575945961
MPHOSPH10	-0.143788766592010	0.64046407017727	-0.1540220006131	0.772253934458676	0.0150773333333344	0.963537990151403
RPP38	-0.143813742531533	0.640436447372882	0.0564768149149236	0.93176886339657	-0.0492443333333335	0.843134844119548
ACLY	-0.143945165887037	0.639873758284402	0.073020235142149	0.906592046281011	-0.118944666666664	0.575589336285762
RNPEP	-0.143964295630412	0.639873758284402	-0.0217681722632117	0.97536722461972	0.0230076666666674	0.93414152758924
SPTB	-0.144050001400247	0.639701989856852	0.121499736508721	0.831512266439821	0.153623000000002	0.49971129098408
KLHL24	-0.144050459263908	0.639701989856852	-0.340785034380212	0.378272273903863	-0.0642676666666666	0.7744217439919
SPNS3	-0.144092795259793	0.639569555038631	-0.00928689351862213	0.98909166696601	0.0105580000000004	0.975988780928093
NINJ2	-0.144114410016847	0.639501928735165	-0.283967225105328	0.500873210019812	-0.0746633333333334	0.76564992393702
POM121	-0.144159446159729	0.639354956360557	-0.080350962752195	0.897107670307584	-0.0880854444444424	0.732554472781933
OPTN	-0.144173089246777	0.639339105081564	-0.20017076661138	0.681552955146541	0.160480666666668	0.518836392563847
LZTS1	-0.144239146179011	0.639029815337622	-0.237113199924199	0.603531426598988	-0.113850333333333	0.581467157766532
STX4	-0.144248191715331	0.639029815337622	-0.133749857348869	0.808879359838378	-0.281155999999999	0.230259858639898
FCAMR	-0.144261471777134	0.639029815337622	0.038890909978161	0.954165422885166	-0.129384000000000	0.568949109970314
SOX30	-0.144312469330100	0.638870568682282	-0.062328039432283	0.922906018065302	-0.0425219999999992	0.871104061464043
NRBP2	-0.144315973875150	0.638870568682282	0.181660277719975	0.720537767137049	0.127073	0.554946393495356
CCDC151	-0.144388135620066	0.638597565364482	0.201620367579798	0.680054545720343	-0.148663	0.657217907037306
EMX1	-0.144413018028119	0.638570340856502	0.255036421364759	0.566577861267331	-0.176589666666666	0.516217427447249
TEX14	-0.144422034214768	0.638570340856502	-0.240020324078802	0.598092982260025	-0.0425399999999996	0.895510661232872
SLC43A2	-0.144514121689880	0.638434500719525	-0.167531802282616	0.748960978799932	0.168777000000000	0.408369243753251
AVIL	-0.144525867234919	0.63843103039692	-0.368918320517581	0.32362669860138	0.115871500000001	0.592845916952892
LRRC8A	-0.144627825756147	0.637841947468087	0.0588926949929488	0.928276034593633	0.00293866666666704	0.993677488355417
MMP7	-0.144647831383724	0.637841947468087	0.00033080057555777764	0.999729358441599	-0.0072433333333327	0.98387459083709
SLC25A33	-0.144648070023438	0.637841947468087	-0.645602038451476	0.0167517957225989	-0.240146833333333	0.383716544590226
C14orf182	-0.144669529773632	0.637841947468087	-0.0252220858419821	0.972358966414305	-0.0119239999999993	0.97184501043952
LETMD1	-0.14475664792477	0.637582792716122	-0.0627354921451746	0.922350559812086	0.0623846666666676	0.781866487624294
CDK5RAP1	-0.144778860766051	0.637582792716122	-0.249790245202197	0.57536723546666	-0.123497333333333	0.600737808923323
FLRT1	-0.144780645827890	0.637582792716122	0.0176537121485666	0.980290246368296	-0.0553076666666663	0.84207712482186
LOC100131510	-0.144794154331896	0.637582792716122	-0.191818570309954	0.701524424520188	-0.0269466666666662	0.921546617328678
EEA1	-0.144805757609412	0.637582792716122	-0.0378030675711324	0.955190852940141	0.311269333333335	0.177814128969052
C1orf158	-0.144856778484084	0.637582792716122	0.106049750666388	0.858902068936183	-0.103420333333333	0.612618944235881
CYP2B6	-0.144901048146040	0.637582792716122	-0.115801487718021	0.842992311631688	0.0106593333333340	0.972209175539945
LMAN2	-0.144927081138350	0.63757401118806	-0.286073989430902	0.496429042723156	0.0663620000000012	0.77718596107678
LDHB	-0.144978260073133	0.637351883323136	-0.0218034709635045	0.97536722461972	-0.103226666666665	0.625258262671142
APOM	-0.144987483505677	0.637351883323136	-0.0520815737439148	0.938067483409541	0.0787090000000006	0.791599529156989
NAP1L2	-0.145036302011602	0.637302147677643	0.0494565883460961	0.94171972877161	0.00082566666666639	0.997953056544486
E2F1	-0.145046404744259	0.637302147677643	0.0265363838401357	0.970207612777188	0.00477633333333512	0.987937064305561
PSMC6	-0.145070595558623	0.63722527836202	-0.0597289673704709	0.926352922534113	0.0792513333333344	0.73168347388773
LOC100131496	-0.145095511241382	0.637209328057365	0.0870920334114775	0.887055939766881	-0.116216999999999	0.629956898894185
MED27	-0.145095595389317	0.637209328057365	0.329218656955995	0.405187926617223	-0.151472333333332	0.504243893162425
UBR2	-0.145116659394219	0.637209328057365	-0.00662131505911668	0.991309497300929	0.0475213333333334	0.849986993244602
FOPNL	-0.145136693862264	0.63716069547674	-0.219753205522539	0.641035290381005	0.079730666666667	0.711082533709514
ATP6V1G1	-0.145138283941880	0.63716069547674	-0.0531512735760813	0.936712858731269	0.0835206666666678	0.698054726653478
SNX17	-0.145213684340103	0.636845582414223	-0.0452587759409977	0.946556809762233	0.302419666666667	0.10978993977345
ATP6AP1	-0.145249866629824	0.636845582414223	-0.408463349577522	0.250484125644429	0.193810000000002	0.32760640417558
REM1	-0.145279321330451	0.636818071928564	0.206649027332739	0.670733295226775	-0.0286369999999993	0.940113574285485
CCRN4L	-0.145350155273389	0.636503986804805	-0.129145747856441	0.814995929697308	-0.0875046666666663	0.68135180147671
MYO5C	-0.145355548109631	0.636503986804805	0.122730478209531	0.828926646969347	0.234319333333333	0.4209781872917
PGLYRP1	-0.145388122614927	0.636403373632038	0.00682394907706625	0.99129507664977	-0.174116000000000	0.623750330148461
RNASEH2B	-0.145404972871831	0.636366926203691	0.0222018226079219	0.97536722461972	0.230886000000000	0.291501950143291
CLEC4C	-0.145447593103110	0.636163147619255	-0.0179976952204058	0.979613786029786	0.0733303333333339	0.741809049318412
ABCD3	-0.145456795826478	0.636163147619255	-0.165233652306280	0.752586940524596	-0.154075333333332	0.474455297935757
SAPCD2	-0.145477987225753	0.636111735961998	0.046812373850307	0.944274559688373	-0.0379486666666662	0.883189085233712
PSG4	-0.145498905388834	0.636048888076124	-0.153401844600630	0.773870060547252	0.128289333333334	0.68951218434739
EFEMP1	-0.145526200996437	0.635966131630973	-0.270191664812164	0.530185035699403	-0.00726433333333288	0.983159241812861
PEBP4	-0.145540694980865	0.635966131630973	-0.0392938929839918	0.953905313456582	-0.0921623333333328	0.84999270430897
SLC19A2	-0.145555089802278	0.635966131630973	0.0144771378099474	0.983444353656142	-0.00369199999999902	0.993523650516004
CELF2	-0.145624610238924	0.635816604616969	-0.380428590772272	0.300567145339266	-0.0291036666666665	0.910625003107163
CISD2	-0.145647429242201	0.635741442109084	-0.0174579405991682	0.980603313490195	0.074087000000001	0.731303939999377
HS3ST4	-0.145662442817371	0.635716968241936	0.0556849721696144	0.933409139126562	-0.108780999999999	0.686983131653892
LIN54	-0.145762268333593	0.63528785946623	-0.0654660055640594	0.918111672450639	0.211307666666667	0.348000484523924
ASS1	-0.145833651325327	0.635043472348377	-0.235601858903370	0.6067637636079	0.243663333333333	0.310277378475854
RECQL4	-0.145839596862857	0.635043472348377	0.376100030264993	0.309828524690001	-0.126172999999999	0.592716584553539
UMOD	-0.14586424196834	0.634990903317457	0.218696590041833	0.642137448592891	-0.119819666666666	0.688145991467449
MAGEH1	-0.145866661725194	0.634990903317457	-0.106034006568674	0.858902068936183	0.104586000000001	0.673538419974363
TIMM8B	-0.145950428118731	0.634577519267715	-0.0866093626376832	0.887493600482322	-0.134598333333332	0.528409919089401
SNX30	-0.145983576944205	0.63443539284409	-0.34909172557049	0.360273681203832	-0.267798333333332	0.188354899671930
SLC9B1	-0.146002694432539	0.634428771922548	-0.090945418235846	0.881187233358984	0.0189820000000002	0.955308921356544
SCFD2	-0.146041892652218	0.63434890576505	-0.416883860570054	0.234235464483992	-0.250165333333333	0.263064019049831
SLC16A7	-0.146055105652294	0.634336157461589	-0.098722618539693	0.868042734188964	-0.251256333333332	0.385612834202104
OR3A3	-0.146151371128752	0.633809672338152	0.144020660978180	0.791666231156359	-0.241331333333333	0.287956132210494
CHST7	-0.146199784199028	0.633809672338152	-0.389458728602456	0.283343454325927	-0.0748449999999994	0.815309621716298
PDX1	-0.146214426098600	0.633809672338152	0.166065558960436	0.751830402832405	-0.154042999999999	0.529875977727902
LOC554206	-0.146316378716695	0.633298006783983	-0.0267620957310267	0.970207612777188	-0.109718000000000	0.656682070833594
TMEM161B	-0.146343572238544	0.633194610632074	-0.0168121294569541	0.981160061237418	-0.0690469999999989	0.752992371482229
REM2	-0.146360306498092	0.633194610632074	0.105621313734243	0.85956218633532	-0.0537363333333326	0.826163946239814
C1orf222	-0.146377909580216	0.633194610632074	0.0800301999341613	0.897577172229156	0.165844333333333	0.484446864118853

SLC46A1	-0.146414740265730	0.633097901963546	0.305401446199688	0.458013550008763	0.0123476666666668	0.966342921970461
SYVN1	-0.146419212603839	0.633097901963546	0.0606684451566276	0.925335786425382	-0.0454023333333322	0.855121286257049
C11orf40	-0.146426554848205	0.633097901963546	0.146591381085699	0.786373631678209	0.0688920000000006	0.766788062425793
RASSF4	-0.146444307445347	0.633097901963546	-0.353568739147759	0.352236748429479	0.150671333333334	0.648442399981194
FBXW4P1	-0.146534949154501	0.632683253666034	0.166964460520282	0.749950473710419	-0.171285000000000	0.4261850174154
SEMA3B	-0.146547226487643	0.632676621566667	-0.0363015608413496	0.95778718265765	-0.0852373333333328	0.801761479877858
TMEM234	-0.146617525729615	0.632512726277614	-0.115750714222641	0.842992311631688	0.233557	0.314112513261693
MMP1	-0.146627605376679	0.632512726277614	-0.0131617653815031	0.984482935754244	-0.0799799999999994	0.706087144562033
EID3	-0.146675326325916	0.632399113025738	-0.277014022545251	0.515587194962102	-0.113504666666666	0.631192833987568
PHB2	-0.146723935790843	0.632260753923615	-0.183263587045579	0.718067440349544	-0.163632499999999	0.490974594771964
EXTL1	-0.146725887980966	0.632260753923615	0.219705107176352	0.641035290381005	-0.105492333333332	0.639837290428782
EXOC3	-0.146757478500397	0.632260753923615	0.151480467534970	0.777104180604402	0.0170253333333343	0.955460918122607
JAK2	-0.146758611379885	0.632260753923615	-0.20229918659072	0.6793987774232	0.0190833333333340	0.95531649368174
CSTB	-0.146967370505846	0.631193700472689	-0.134639164098019	0.808009477165608	0.0636233333333336	0.788201431210286
RBP2	-0.146977252521208	0.631193700472689	0.102459688463971	0.863158472924381	0.0260356666666668	0.922148106708426
PTPRCAP	-0.146986796033708	0.631193700472689	-0.0468718869044355	0.944274559688373	-0.133710333333333	0.606031399898535
FBXO40	-0.147031801445523	0.631068058754457	-0.217735370862412	0.644743872041817	0.0853073333333346	0.843134844119548
TMEM175	-0.147066186893451	0.630991198110116	-0.111154916779180	0.849127967004266	-0.0080339999999999	0.982803787737895
ZNF513	-0.147093533843386	0.630991198110116	0.190158738946792	0.704208582824765	-0.101285999999999	0.714970019441215
C6orf132	-0.147205828844665	0.63045132863169	-0.0459039400573987	0.945372627844176	-0.0110323333333329	0.972261709812365
FAM8A1	-0.147262885437652	0.630300623524041	-0.0624297833958769	0.92264749701958	-0.0202876666666659	0.941493821305792
TMCO6	-0.147326776694722	0.630032716763133	-0.290542594606851	0.488149066489322	0.0236496666666667	0.933412982728621
OR2M4	-0.147326930010598	0.630032716763133	0.0931029850758037	0.877130088568424	0.348342333333334	0.113700575160835
ARX	-0.147391473756627	0.629882568684922	-0.365747474011984	0.32927628703889	-0.0888299999999994	0.798649992368555
MLLT11	-0.147412904323186	0.629839726223566	-0.52988043883997	0.0799297163112453	0.0234466666666684	0.93414152758924
UBE2J2	-0.147500529049608	0.629345290671977	-0.231276772953995	0.615492377862864	0.0134760000000003	0.967069669375554
C19orf52	-0.147575311164425	0.629007062570199	0.123634190644671	0.826475556843795	-0.0901089999999994	0.692494286263795
CACNB3	-0.147601737499267	0.628981844282479	-0.146722055172970	0.786187758946602	-0.116983333333332	0.664714409479409
TMPRSS2	-0.147604065339614	0.628981844282479	0.106310871481749	0.858284876027025	-0.0310056666666661	0.91282404798885
PDCD4	-0.147609883847291	0.628981844282479	-0.0231532097333497	0.97536722461972	0.102540000000001	0.684151978569598
RAB23	-0.147612181120007	0.628981844282479	-0.427637463614609	0.212158677414418	0.834796000000001	0.00521513356459738
WDR47	-0.147648836735792	0.628981844282479	-0.481542898608667	0.135560525489292	-0.0791099999999988	0.771420519321125
MID1IP1	-0.147715405324631	0.628981844282479	-0.038487180024869	0.954545200586067	0.0610306666666682	0.802146947589035
BMPER	-0.147886492499165	0.628159985088912	-0.365480636886159	0.329800721443396	-0.0523789999999993	0.822657709336468
PPM1N	-0.147916333424386	0.628039829192623	-0.20461900559302	0.674108447036116	-0.0440466666666657	0.864974134126057
PUS1	-0.147947987280883	0.62790795326833	-0.186990167483632	0.710254767709493	-0.198012666666666	0.472124270069641
RAX	-0.148005877165844	0.62767928325848	0.0889396648736086	0.883865906764221	-0.0921596666666666	0.758966957076109
KIAA2026	-0.148070760524812	0.627350927311438	0.410371207421706	0.246641234675363	0.0673323333333335	0.782945304631328
NAALADL1	-0.148077237447554	0.627350927311438	-0.327374923423365	0.40846136066764	0.0168570000000012	0.95784037718285
CSF1	-0.148097803460471	0.627350927311438	-0.114142647106722	0.844272085764738	-0.0497703333333336	0.867354667762818
HCLS1	-0.148227416372693	0.626941214033807	-0.494653768130156	0.117667182355246	0.100310666666667	0.627452499444403
CTSG	-0.148235679854156	0.626941214033807	0.158602969644394	0.765791046741822	-0.160715333333333	0.442322325044862
PPP1R35	-0.148238151127089	0.626941214033807	-0.0580244217993852	0.92929701257827	-0.138294666666667	0.657850424949736
NRG3	-0.148317421591380	0.626612273782842	-0.216173786868792	0.648391670659269	-0.121194999999999	0.597164563493288
FAM214A	-0.148360898518539	0.62640408769764	-0.0570705005076949	0.930775493791569	0.0381116666666672	0.875244017858826
PANK3	-0.148404761754296	0.626339214923887	0.0585842335401248	0.928586335857223	-0.0162876666666664	0.95450472002063
GALNTL5	-0.148471332574493	0.625981795395423	0.252929220705017	0.568985221714455	-0.0536559999999994	0.812230603035278
TOM1L1	-0.14849713178949	0.625887933920239	-0.0348134337458927	0.959404955370931	0.205124666666667	0.293713036282908
MARS	-0.148521296685787	0.62587751822743	0.124821389559945	0.824472060590171	-0.105823666666667	0.657217907037306
APBB3	-0.148536715536020	0.625850753878663	-0.0903729264107	0.881905181382283	0.0795196666666672	0.76397955844876
CYP2A7	-0.148573772658287	0.62568414945585	-0.0601604949636456	0.925649366494154	0.0583326666666671	0.801761479877858
SLC16A6	-0.148580462496245	0.62568414945585	-0.0440818030273519	0.948520928173175	0.0301933333333348	0.910663650054071
CLCN5	-0.148583699456087	0.62568414945585	-0.078445169726822	0.899281198071059	0.0462203333333332	0.86785638597605
KIF21A	-0.148593888650407	0.62568414945585	0.104490932822138	0.86098619534505	0.397565666666668	0.117136312205538
C7orf13	-0.148640580629201	0.625543966515978	-0.065022345538452	0.918489100363712	0.0439343333333343	0.895476343718166
USP45	-0.148640703174495	0.625543966515978	0.264011726928799	0.544711371453131	0.100435666666668	0.648442399981194
DGCR6L	-0.148752911026685	0.625133675536941	0.104287404486725	0.86124543980304	-0.0314739999999986	0.903864363916236
ADCK2	-0.148818199915195	0.62497942525456	-0.336982318405276	0.388337351990747	0.147228666666667	0.516265433652982
FNBP1L	-0.148933064893638	0.624674749775325	-0.152024356487586	0.776146388301083	0.220079000000002	0.27071513514891
TMEM165	-0.148964955643343	0.624541632301184	-0.227782333688360	0.624564310654617	0.0401540000000015	0.871104061464043
SLC6A17	-0.149005550391815	0.62435230722635	-0.063787053744251	0.920521809324956	-0.0809066666666666	0.762898679429892
LMBRD2	-0.149041445865053	0.624266251251278	0.0651072995359516	0.918489100363712	0.258958000000001	0.198299581791991
ZNF287	-0.149073310783731	0.624263872564908	-0.0746295150803547	0.904649210128014	0.166107333333334	0.575986474440933
MRPS31	-0.149092588740695	0.624263872564908	-0.372737596601488	0.317205263174137	-0.0110053333333326	0.975988780928093
CRYBA1	-0.149112589537847	0.624244231702046	-0.0662453418102091	0.916661988649658	-0.00604033333333298	0.985230239434352
KIAA1377	-0.149126771799325	0.624227969966325	-0.150254365920716	0.779675068549263	0.168423666666667	0.499850659274909
CDH10	-0.149131847329288	0.624227969966325	-0.253811363140794	0.567694846153007	0.445253	0.166543996066754
EMR2	-0.149176556765081	0.624195775621733	-0.301526298984792	0.463490113383679	-0.0821303333333332	0.76368723681351
CCDC70	-0.149229310314203	0.624195775621733	0.198275283611583	0.687317734296043	-0.158612666666666	0.600737808923323
CMA1	-0.149235657254991	0.624195775621733	0.00773764717578851	0.990405674923721	-0.0367003333333333	0.885866310496875
F2RL1	-0.149244839366107	0.624195775621733	-0.232729635012024	0.611941461795609	0.380287333333334	0.210554200098174
TRIB3	-0.149280180421616	0.624183296318004	-0.133805361426478	0.808794757024095	0.0574780000000006	0.80935794907855
SLC18A3	-0.149302990161133	0.624108916973016	-0.0453815205101804	0.94632884488527	-0.0992523333333318	0.663776173049665
PTH2	-0.149312313538043	0.624108916973016	0.146590982251590	0.786373631678209	-0.177685000000000	0.5683587002004
ZNF155	-0.149392418625673	0.6237500919811	0.0432252253488843	0.950038150979272	0.135361000000002	0.49912108040986
NLRC4	-0.149447310084189	0.623614519145274	-0.165518054998538	0.752352242644389	-0.0176956666666667	0.951470510192659
NUDT1	-0.149457507777113	0.623614519145274	-0.133234702707836	0.808992949393043	0.172054666666667	0.470695033055552
C5orf34	-0.149514702865689	0.623398195242054	0.0601145029633225	0.925649366494154	0.120071333333334	0.687448770010242
TMEM138	-0.149520959408699	0.623398195242054	-0.177691418408515	0.728276800708888	-0.0220433333333333	0.934358485610289
ZSCAN21	-0.149522581600132	0.623398195242054	0.172364646638709	0.738556291013274	-0.130976333333332	0.540255856426522

ARAP2	-0.149543766085902	0.623398195242054	0.0740241329070994	0.905506084101768	1.021606	0.0098546599387472
ISL1	-0.149562975318196	0.623398195242054	0.232305932382548	0.612658112747496	-0.0384489999999997	0.929066230830908
APLP1	-0.149653062354929	0.623234939232207	-0.0744467611503891	0.904945422294164	0.0699333333333345	0.811867274062946
CLN8	-0.149767837974095	0.622712561119846	-0.117997825307	0.838500126709059	-0.35665	0.0828974079436732
CIR1	-0.14979279037739	0.622624438918517	-0.0533846221039560	0.93647464505741	0.124463666666668	0.58043274631496
ATP5D	-0.149853471936248	0.622378597411022	-0.0176747406580797	0.980278729770454	-0.133158666666665	0.518244955194698
AIF1L	-0.149914710965002	0.622202268200945	-0.0704433934080134	0.910299212550106	-0.158178333333333	0.442322325044862
SNX7	-0.149940299749234	0.622110069797968	-0.312017937055063	0.440789236061843	0.277253000000000	0.14448396872886
CACFD1	-0.149945983695583	0.622110069797968	-0.0133856140217957	0.98412883278237	-0.251313000000000	0.337307065334106
EPT1	-0.149987695310334	0.622023201358892	0.126800311596380	0.820923134671156	-0.335274999999998	0.133651630882053
NUBPL	-0.15011316909161	0.621505223385126	-0.132633727156056	0.809801552882903	0.231629333333335	0.356690395692733
PMPCA	-0.150126099881614	0.621494801549529	-0.080535625725724	0.896949986708202	-0.159684333333332	0.408033886002958
SELPLG	-0.150155227977195	0.621380473305381	-0.227608917077415	0.625174304269387	-0.260828000000000	0.262516228425933
ZC2HC1A	-0.150202932171523	0.621380473305381	0.0305688363142197	0.965652334162082	0.351472666666667	0.112146551360288
BEX2	-0.150229274833799	0.621380473305381	-0.173037302351884	0.737325433935202	-0.101864666666666	0.735614422262526
CFL2	-0.150257608144040	0.621380473305381	-0.101368668555710	0.865954921256394	0.345821000000002	0.0979662489851624
DEAF1	-0.150269000439207	0.621380473305381	-0.00505406086369157	0.992521617396072	0.111604000000001	0.607789868477511
TDRD5	-0.150291278224543	0.621378619023987	-0.319486758900440	0.42581212454843	0.296610666666667	0.224565182821064
FAM71E2	-0.150321774091114	0.621254799760141	0.106671616098109	0.857937967416038	-0.208412666666666	0.437131160280468
GRHL2	-0.150340018543414	0.621210122502865	-0.0761060491358532	0.903296922694955	-0.0840159999999993	0.768164585194196
BAG2	-0.150349388454206	0.621210122502865	-0.110238256102795	0.850294817523385	0.134926666666667	0.608311546956177
CHAT	-0.150425448290704	0.620877859853193	0.271772654470063	0.527608228552189	-0.0516909999999998	0.840175825309652
PITHD1	-0.150460918190705	0.620877859853193	-0.310769945774519	0.444561109519731	-0.235780333333333	0.218478495014124
LGMN	-0.150491320035338	0.620877859853193	-0.230703138915678	0.617240048465949	0.426472666666667	0.120319574336004
IL36RN	-0.150493028768040	0.620877859853193	0.146091291524291	0.787402233709509	-0.106002000000000	0.70341061296855
CUTA	-0.150511834322005	0.620877859853193	-0.263630359037605	0.545374660547732	0.0526120000000001	0.824746506521392
D2HGDH	-0.150598986690912	0.620708590237325	0.125353724228520	0.824047378954	-0.0966913333333324	0.716517864791776
IKBKE	-0.150611645759915	0.620708590237325	-0.202555197875384	0.67841894891616	-0.122307333333332	0.616961986237355
CCL23	-0.150636655609368	0.620708590237325	-0.0897319971553066	0.88318195439136	-0.155422666666666	0.496323393705678
ABCC10	-0.150720761516553	0.62043471520434	0.0132278308564909	0.984362682224315	0.0186730000000006	0.945904449997854
MRPL9	-0.150736635645805	0.620405439767571	0.002975638263592	0.995177530008123	0.0605470000000013	0.786261420559593
PELI1	-0.150742724611347	0.620405439767571	-0.137247378444196	0.802488298241532	0.113780000000000	0.614221661998915
GLRX3	-0.150806046557698	0.620250165435839	-0.229066869556528	0.62083288085142	-0.144869666666666	0.48174711509713
PTK2B	-0.150875128332119	0.61987721074646	-0.100954969593824	0.866005093242444	-0.0533786666666659	0.814780717126634
LNX1	-0.150923200581219	0.619640024201442	-0.34308834957844	0.37476553791689	0.246655	0.341545647784104
MOAP1	-0.151035184610206	0.619063548897216	0.00454205118064656	0.993733225898948	-0.0908696666666658	0.69061405137422
ANAPC15	-0.151046737853177	0.619062181377524	-0.304625178219037	0.459197740121543	-0.0667303333333325	0.78197285216323
LMOD2	-0.151079228335696	0.618925691680997	-0.165440597707469	0.752352242644389	0.0208926666666672	0.943952610325773
PEPD	-0.151162827069460	0.618532632748791	-0.00788412970990654	0.990405674923721	0.0293733333333340	0.90778598212344
SPP2	-0.151175131064487	0.618526421457259	0.158540501402491	0.765993392549069	-0.0966866666666661	0.721039266334933
SOX10	-0.151224157561705	0.618413429555568	0.0702476173756577	0.910394157189308	0.0543406666666669	0.85212888163999
FZD5	-0.151254794120661	0.618305183665185	-0.322926782238092	0.415927283236740	0.0117103333333344	0.976900320969229
CSPP1	-0.151282976641765	0.618305183665185	0.0163792803420453	0.981892085795004	0.161797000000002	0.620692670936735
TESPA1	-0.151401723681985	0.617723321471433	-0.274762228028807	0.520095539214775	0.0175626666666670	0.958727447107698
KIR3DL3	-0.151456034436568	0.617599953100547	-0.134239547068713	0.808009477165608	0.11042588888889	0.682390613806982
TIGD5	-0.151457306139118	0.617599953100547	0.341927485834639	0.377158872804021	0.102638000000001	0.634105753368746
TRAF3IP3	-0.151482349991903	0.617599953100547	-0.282185366902718	0.504065779877366	-0.0906463333333332	0.662828468002357
C8orf22	-0.1515183761631	0.617599953100547	0.0148685137699471	0.983111125676367	0.0172783333333333	0.958098397518313
BEND2	-0.151580823220727	0.617373285710888	0.171446349441569	0.739541495555634	-0.0162016666666666	0.967879244181712
HSPA12A	-0.151633412159968	0.617297279490192	0.101384309585677	0.865954921256394	0.192551666666668	0.359908305595919
AUP1	-0.151792826694120	0.616738274002298	0.0583175032058282	0.928766151140997	-0.0540579999999995	0.826922793139517
RNFT2	-0.151800912659934	0.616738274002298	-0.290343128182832	0.488568550695131	-0.0492996666666662	0.843152489417868
LINC00029	-0.151801167876035	0.616738274002298	0.321119980198316	0.421618886526906	-0.162429666666666	0.585260484981764
PPP2R4	-0.151804803458570	0.616738274002298	0.158077620660441	0.767373487136803	-0.271723333333331	0.191850325697419
NUDT9	-0.151804810286586	0.616738274002298	0.0358657009288723	0.958354725588542	0.085080666666668	0.728894262229555
SAG	-0.151847331566233	0.616738274002298	0.06673891555996	0.915438681913279	-0.0541709999999998	0.868257776244077
STAG3	-0.151863344600121	0.616738274002298	-0.254843540355910	0.566909791913503	0.219763333333334	0.271872170281604
PLA2G4C	-0.151930814155769	0.616580957362935	-0.463873418076284	0.157277647666176	0.159352333333333	0.609744707329995
LCN1	-0.151941143834374	0.616580957362935	0.0960182973963838	0.872311810447594	0.150061000000001	0.591037690220586
SDF2L1	-0.151947058942752	0.616580957362935	-0.260579678225125	0.553540075799904	-0.151597999999999	0.561324277458518
SLC13A2	-0.152006557143155	0.616458931088168	0.199687438620803	0.68313035362506	0.00522800000000045	0.990426818363066
DUSP26	-0.152037785504180	0.616330822428300	-0.0171065799739549	0.98105020869911	-0.0197526666666664	0.954736472265512
RB1CC1	-0.152052865954082	0.616330822428300	-0.392895009402199	0.278297610043666	0.0210210000000014	0.94164007263756
SNRNP35	-0.152162184471975	0.615968427849402	0.138272064918007	0.800120930330156	-0.0067046666666660[illegible]	0.983159241812861
MCHR1	-0.152188892576166	0.615942839299046	0.536071556616508	0.074032576515667	-0.118209666666667	0.556319218153487
WBP2	-0.152211312577296	0.61587158263317	0.247779617935296	0.579471329472972	0.0938560000000009	0.686908666024983
INTS9	-0.152245343319383	0.615851935505223	-0.094828118321241	0.873746394266418	-0.219822666666666	0.29920573922735
C15orf26	-0.152322787819948	0.61560085677717	-0.202946173035503	0.677532075317915	-0.112215000000000	0.634163156600322
EIF2B1	-0.152329302717313	0.61560085677717	-0.0541769797969698	0.934896231465586	0.0170913333333340	0.95075262428163
ARL6	-0.152446938276994	0.615402018161614	0.239189731040973	0.599801655225887	0.0327703333333339	0.92980287143849
LSM11	-0.152472999646468	0.615402018161614	-0.214914075625072	0.651376266020544	0.188812333333334	0.472711941762195
UBE2R2	-0.152485788156819	0.615402018161614	-0.0521595333299266	0.938067483409541	0.156075666666667	0.43923010114809
SLURP1	-0.152510218455683	0.615402018161614	0.0593678319467789	0.927064799234187	-0.0711253333333326	0.809418131417703
CCDC85C	-0.152564866477147	0.615205800698938	-0.145487592738283	0.787804113309913	-0.238854499999999	0.232897341415308
CD40LG	-0.152572340835099	0.615205800698938	0.0110956994873131	0.98774001793194	0.085311666666667	0.68499503971094
HLA-DOA	-0.152594131996899	0.615205800698938	-0.257997528017323	0.561818764500592	-0.135733499999999	0.648442399981194
NCEH1	-0.152630918472532	0.615205800698938	0.060205460713322	0.925649366494154	0.302994000000001	0.143931155913550
S100A16	-0.152633295292547	0.615205800698938	-0.192019535580059	0.700976254251101	-0.0132459999999997	0.973686596735315
SEC24A	-0.152748028943133	0.614832434335103	0.0671232931924768	0.914735297916921	-0.168256666666667	0.447498161237612
LRRC58	-0.152793394101216	0.614686781074613	-0.00775950661422057	0.990405674923721	0.0311073333333339	0.902859284837213

FAM167B	-0.152813705306238	0.614686781074613	-0.303769484429509	0.460052080110635	-0.107066333333333	0.613030354362634
TINAG	-0.152835322949763	0.614686781074613	0.165055160646590	0.7529423475214	0.0268856666666669	0.915761046438965
TIGD4	-0.152847349258567	0.614686781074613	-0.178772753701823	0.726057281492174	-0.0844953333333325	0.738628460817237
CYP1B1	-0.152848588212088	0.614686781074613	-0.240248533763428	0.597862631330416	1.02177766666667	0.00421637924026936
ACTR1B	-0.152967822380149	0.614223234217958	-0.048822673320839	0.942180113539637	0.0642166666666684	0.778477148290034
MAPK12	-0.153032472894972	0.613985801374944	-0.176953568448841	0.729794187782856	0.0608313333333341	0.7996066102903
HEPACAM2	-0.153058238720396	0.613985801374944	0.0573825167868044	0.930490408235626	-0.145843000000000	0.575378149225683
NDFIP2	-0.153106730483719	0.613879306845009	-0.0625509476181897	0.922632631649811	-0.00471866666666643	0.987937064305561
COX8A	-0.153142881459301	0.613829498861917	-0.278415137452622	0.511336855414802	0.168268666666669	0.479443005264132
RIT2	-0.153175707986039	0.61376489456984	0.116798008855814	0.841562044027187	0.034012666666667	0.903864363916236
MAP1LC3B	-0.153562056239195	0.612129845716746	-0.474190146661583	0.144358453800098	0.203598500000001	0.422927358730594
DNAJC17	-0.153567505378546	0.612129845716746	-0.0804442302275397	0.896952038996626	0.102391333333335	0.640836927155663
DSG3	-0.153700649963161	0.611631192418435	0.0161337574328341	0.982057247916055	-0.0265959999999996	0.924469572710745
MLLT4	-0.153752513508635	0.611444103521948	-0.0451314904457941	0.94659867551232	0.123316000000000	0.575589336285762
CCNL2	-0.153810871076647	0.61128868909482	0.122249062737144	0.829569971668386	-0.123417166666666	0.602492574011631
CYP2J2	-0.153841661945098	0.611163879034138	0.0119356228734394	0.986261540519832	-0.0809993333333326	0.758341714501375
SNRPB2	-0.153923831548289	0.610855201648062	-0.00688799137017281	0.99129507664977	0.121343333333334	0.566992889727905
MLST8	-0.153991714989066	0.610649326360155	-0.151933920579974	0.776173016352835	0.0217176666666686	0.93682999997207
GTPBP8	-0.153995661998941	0.610649326360155	0.0867596657572808	0.887447471805072	-0.00745599999999925	0.982398683576954
CASC2	-0.154049184569913	0.610488990466812	-0.134266544128337	0.808009477165608	-0.00924999999999986	0.97978580354354
KCTD9	-0.154096797341266	0.610359691387372	-0.192141946955203	0.700820605802046	-0.140095333333332	0.596617523400524
MRGPRX4	-0.154189436988295	0.610146811120858	0.219106883166006	0.641699006980579	-0.0266056666666667	0.967241125442187
VN1R1	-0.154233054540587	0.609966740325334	-0.0985701376289665	0.8682520067418	0.0863593333333339	0.777704292898672
C2orf69	-0.154325397059249	0.609634972814063	0.216037633699887	0.64844945813838	0.224180333333334	0.322893184569333
ZNF160	-0.154344692503552	0.609615692757607	0.0843900170072438	0.892302911210018	0.0085753333333345	0.98025379113353
ETV2	-0.154356070545883	0.609615692757607	0.416676512393911	0.234235464483992	-0.0582389999999993	0.826305377184894
PREB	-0.154365593174055	0.609615692757607	-0.0197021841684932	0.97763669370967	-0.0885576666666658	0.728054565398526
DGKB	-0.15438175612818	0.609615692757607	0.00485455661539015	0.993054657943085	-0.965508	0.00587117284252594
TNFSF14	-0.154388093957545	0.609615692757607	0.0360576866317179	0.958088522299884	-0.139436333333332	0.633561720825521
PLXNA4	-0.154400436610704	0.609615692757607	-0.0248820453664248	0.97287238458594	0.161367666666667	0.425664293107569
GPKOW	-0.154536776459982	0.609184211582294	-0.467375124492315	0.151936428121882	-0.0297183333333321	0.907739915890815
RCE1	-0.154541831626492	0.609184211582294	0.211221119042796	0.660013379937732	0.0796256666666666	0.729368274679147
ZFP2	-0.154554020548616	0.609184211582294	-0.0360560017277558	0.958088522299884	0.239987000000001	0.498080784053156
PACRGL	-0.154582408116904	0.609115122601608	-0.42218729870066	0.222319600995852	-0.0758243333333325	0.780717071872908
HMGCR	-0.154656332314155	0.608860046598632	0.150882149874922	0.778253782413218	-0.207870000000000	0.274121181062751
PM20D1	-0.154699708289357	0.608728031892527	-0.0220065852808413	0.97536722461972	0.0701953333333335	0.777406710539424
MAP3K6	-0.154747689275819	0.608566407899434	-0.198010211787365	0.687663943268976	0.107418666666667	0.597780076365645
ABCB5	-0.154789644183316	0.608370037775526	-0.0995280377282762	0.867603998426271	-0.0742456666666662	0.730777457405814
TRANK1	-0.154804625269402	0.608370037775526	-0.349670832697529	0.359095724048761	-0.00496400000000007	0.988133186272453
NDNL2	-0.154845361093480	0.608345803896549	-0.197485832090602	0.68896171032074	0.105256333333334	0.640799282945291
C11orf74	-0.154846417882217	0.608345803896549	-0.178869741949825	0.726006906302736	-0.121283333333333	0.550446282371936
TLR7	-0.154858445740524	0.608345803896549	-0.314715793435679	0.436900127228272	0.135007000000000	0.518388954029844
PAX1	-0.154921192173265	0.608345803896549	0.0543798297658158	0.9344692810234	-0.0990906666666666	0.735938662411184
SQRDL	-0.154964409759743	0.60820209296039	-0.397906848607929	0.268992868889265	-0.0648396666666666	0.86131429953963
BTBD3	-0.154975463862154	0.60820209296039	0.115015181339113	0.844162087612813	0.367242666666667	0.0896797344789652
ZNF195	-0.155003514131579	0.608097705751365	-0.24458668564286	0.587653287154633	0.0898623333333344	0.78780036542932
C10orf54	-0.155006021872820	0.608097705751365	-0.356006572700388	0.346552539524285	0.118631666666668	0.567244860894295
INSC	-0.15501766042376	0.608097705751365	-0.174949480282077	0.733518674801325	-0.0572259999999992	0.823458541833548
GDPD2	-0.155059075741900	0.608034719164288	0.109812226206877	0.851106621132981	-0.128123999999999	0.57356096971983
GPR155	-0.155089163259934	0.607988408014023	0.0239488360533398	0.974721039716014	-0.0580799999999992	0.809277598660077
DNASE1L2	-0.155157527133154	0.607695794911339	0.248848440941132	0.576871285544251	-0.0526686666666665	0.875253923096376
LOC728196	-0.155168020784657	0.607695794911339	-0.0682789927554452	0.912364243071165	-0.0811263333333331	0.703776964646383
C9orf173	-0.155200755762621	0.607638616494022	0.254241833497450	0.567694846153007	-0.0467833333333331	0.866115937003813
INO80	-0.155279315579751	0.607280484256428	-0.0878711623978475	0.885868927703827	0.00664833333333447	0.985263856374185
PFKFB1	-0.155289792305169	0.607280484256428	0.0416164122514828	0.951989245458556	-0.0152273333333326	0.95572434494623
KCNH7	-0.155290352882362	0.607280484256428	-0.159819019486691	0.763150272733332	0.184919000000001	0.345425110681774
BZW2	-0.155302785885082	0.607280484256428	-0.167742092206550	0.748242818061289	-0.421628999999999	0.114193085140962
CEACAM21	-0.155364976729742	0.607097705246182	0.161645350027612	0.758955920988521	-0.089132666666666	0.67574215232331
P2RY8	-0.155439386627495	0.606692758459855	-0.128742222950983	0.815407702581246	-0.343003666666666	0.0883090203953399
IRAK3	-0.155446536683725	0.606692758459855	-0.225162425687342	0.63103711743427	0.000692333333333363	0.998033679668046
FCRL5	-0.155537855932525	0.606354099994578	-0.349898950091246	0.358969704130952	0.0298400000000005	0.90945379489823
MRPS14	-0.155537947510076	0.606354099994578	-0.0431992952954498	0.950102331606548	0.0970800000000015	0.640398707036842
C1orf159	-0.155571845010304	0.606354099994578	-0.148559655940566	0.783532632807022	-0.0357956666666675	0.910625003107163
TUBGCP3	-0.155620458559474	0.606264834080511	-0.206977188903761	0.669713208801491	0.0426449999999998	0.881515139574123
NUDT5	-0.155649855871029	0.606223135568862	0.0274353790617171	0.970045676424287	0.271798333333334	0.258700580229750
ABHD15	-0.155673587241686	0.60621789317646	-0.409467303073311	0.248644676969744	0.0638796666666673	0.785482517274791
GNAT1	-0.155710138901954	0.606130219843663	0.183119505033036	0.718067440349544	0.0501733333333335	0.847165781298603
LOC100130285	-0.155732424413650	0.606130219843663	0.589902445900868	0.03694061604449	-0.159952999999999	0.416841339539021
CYTH3	-0.155757326384750	0.606047875455257	-0.0265167812252743	0.970209453484284	-0.110147666666665	0.667732731151074
FAM9A	-0.155800936334024	0.605891285268995	-0.144625196773009	0.790044831580319	0.0836856666666673	0.719839702715467
RSRC2	-0.155842895229315	0.605718744030191	-0.0894552845122562	0.883383266241373	0.0409690000000010	0.870810859108551
LRRD1	-0.155850663655799	0.605718744030191	-0.119396513507662	0.836099954217028	0.086615333333334	0.684151978569598
ERC2	-0.155851502889466	0.605718744030191	-0.283554519703875	0.501852406168029	-0.162550000000000	0.516856313887476
MATN4	-0.155854093004431	0.605718744030191	0.0476903181554502	0.943560556552289	-0.132176333333333	0.555689256408654
DECR2	-0.155892554231619	0.605718744030191	0.0406890652170124	0.952426531595115	0.122828000000000	0.5531802635914
SORD	-0.155913422246931	0.605718744030191	0.0390312549374642	0.954042565039198	-0.346203333333333	0.0769341038137651
UPK1A	-0.155919980546887	0.605718744030191	-0.279059108298713	0.511025440770733	-0.0868506666666664	0.745967084434416
IL21	-0.155932973000075	0.605718744030191	-0.341526877501543	0.377727715280457	-0.0187546666666663	0.949295980201336
CCR2	-0.155976645724627	0.605669225490812	-0.278757268056866	0.511025440770733	0.192324000000000	0.359908305595919
RLN3	-0.156002283119879	0.605652516210628	0.406459054296311	0.254041953733213	-0.169718999999999	0.4584541407185

ZC3H3	-0.156031410665407	0.605538954242519	0.318877847617418	0.427860345558585	-0.0132456666666667	0.964514058161652
IFNA14	-0.156073366531263	0.605490518765527	-0.0368994457610312	0.956918184553224	0.0808063333333336	0.752992371482229
MLXIP	-0.156097064311754	0.605421959134304	-0.282653996911805	0.502746534171315	-0.347333666666666	0.192973194605635
CHMP1A	-0.156144432023075	0.605254863619147	-0.0661808037939387	0.916732289592703	-0.0397199999999996	0.873398028647672
PSMC4	-0.156145928235523	0.605254863619147	-0.210603559249146	0.661670522548494	-0.0322253333333329	0.902555205061281
KLK13	-0.156181128738037	0.605182333361167	0.130631688034976	0.812271254601992	-0.169279666666666	0.531329379320408
PILRB	-0.156183096662532	0.605182333361167	-0.238286889308916	0.601773482805025	-0.0463593333333333	0.853541852582193
YBEY	-0.156260118693097	0.604983207884105	0.286430358706504	0.495728580458409	0.0924806666666666	0.706902198257264
COG4	-0.156277003962123	0.604983207884105	0.035487512551021	0.958689318480951	0.114363000000001	0.573673650964407
MOSPD2	-0.156279157926800	0.604983207884105	-0.466329767803544	0.153152263339311	0.155590000000001	0.541495508172268
GNN	-0.156400278437879	0.604607798755542	0.168889071979273	0.74582180262941	0.141457333333334	0.561206054420725
APAF1	-0.156418496970849	0.604607798755542	-0.342324487977237	0.376191808453700	0.186917666666668	0.438651234952961
RABL3	-0.156498775452018	0.604379112918135	-0.00726950387907717	0.990932082705863	-0.0176906666666655	0.953035846190505
ALOX5AP	-0.156562791706661	0.604189090906956	-0.503202288769345	0.109167411145190	-0.02548	0.927422712012045
RNF114	-0.156621606372126	0.603958664529876	-0.142251907222274	0.79450771779567	0.0863313333333337	0.697243147844363
AHRR	-0.156649085746222	0.603877059330022	0.047211774816897	0.943981149721771	-0.0788576666666652	0.727065902741195
FAM46D	-0.156729474890518	0.603708465305339	0.101120100735871	0.866005093242444	-0.0445949999999992	0.863667052826012
LFNG	-0.156778019169560	0.603470260870189	0.337045995289974	0.388318044855766	-0.0317593333333327	0.911623909907545
ALDH3B1	-0.15680118318424	0.603469142553157	-0.253886396113665	0.567694846153007	-0.0356703333333320	0.915761046438965
CTNNAL1	-0.156908583467531	0.603074348719052	0.231846927969379	0.613578980603213	0.119329333333335	0.622315801210008
TRAM1L1	-0.156974524662906	0.602731333106124	-0.0498103144873512	0.941458038227918	0.0529546666666667	0.815309621716298
LRP5	-0.157020762774154	0.602648971723316	-0.192768809250866	0.699860991842232	0.0614406666666671	0.830320848383177
KRT84	-0.157061856916772	0.602535123043039	-0.0229440902211664	0.97536722461972	-0.153194000000000	0.651311817374123
EIF6	-0.157070809330667	0.602535123043039	-0.147970739036724	0.784080588385358	-0.158496666666666	0.45432073488215
LOR	-0.157075847736064	0.602535123043039	0.208402748432802	0.666722617010087	-0.214663333333332	0.458006502146897
AP3B2	-0.157081250224989	0.602535123043039	-0.181986317579863	0.719974771046719	0.129363000000001	0.59712789155738
RAB25	-0.157084957577110	0.602535123043039	-0.0559451590274437	0.932919548410715	-0.0303726666666667	0.94690965769901
MRGPRX2	-0.157105389587572	0.602535123043039	-0.0284162160536231	0.968153117820898	-0.212877333333333	0.496883548839873
EPM2A	-0.157142369287524	0.602532696249978	0.00196309174240728	0.997329836762314	-0.0836266666666656	0.766486380731067
LRRN1	-0.157167444157374	0.602445482321285	-0.00308643246071321	0.994986311144058	-0.0649199999999993	0.842903889380894
CLDN6	-0.157186001813282	0.602400160280277	-0.175766108005272	0.731808176718647	0.0220223333333334	0.966342921970461
CRYGD	-0.157198213037984	0.602395631727851	0.327469198827567	0.40846136066764	-0.163997333333333	0.402604754314243
GBP6	-0.157230271471937	0.602316295843635	0.0851054427052607	0.890556609269654	0.0281560000000003	0.921546617328678
C14orf28	-0.157285450682170	0.602130807812884	0.249904303705883	0.575275130340142	0.337038333333334	0.103301877951436
DRG2	-0.157290188616355	0.602130807812884	-0.0232464288451966	0.975348968561756	-0.0655459999999992	0.777151231980106
SLC25A3	-0.157292599986263	0.602130807812884	-0.268864968560972	0.534295192322247	-0.0312499999999991	0.901317484877412
TMEM219	-0.157350924359803	0.601971168723392	0.0230837036270541	0.97536722461972	0.0665056666666678	0.762898679429892
TRAFD1	-0.157352863904082	0.601971168723392	-0.128712254769341	0.815407702581246	-0.288576333333333	0.178187755617055
NRAP	-0.157365284650076	0.601971168723392	0.0255568858020415	0.97204937337922	0.0491120000000008	0.842696653091951
TBC1D13	-0.157387751573094	0.601971168723392	-0.0979818516822141	0.868852648210739	0.0197956666666667	0.942800137888472
IFI27L1	-0.157409704895371	0.601971168723392	-0.183127995089869	0.718067440349544	0.376680000000001	0.120319574336004
OAZ2	-0.157410287130912	0.601971168723392	-0.326167263599302	0.409206882509236	0.0758846666666673	0.774104133965233
PLCL1	-0.157449544578303	0.601971168723392	-0.261334335280519	0.552187117311017	0.161231333333334	0.514076428873743
FASLG	-0.157471967954021	0.601971168723392	-0.140294258533554	0.797441782082887	0.0606173333333338	0.816134952712854
DNAJC6	-0.157486052710485	0.601971168723392	-0.142051240115948	0.79450771779567	-0.0642050000000002	0.79462828227058
STK11IP	-0.157532348909807	0.601802147466037	-0.114343805978039	0.844272085764738	-0.0171933333333321	0.955460918122607
RNASEH2C	-0.157543152008742	0.601802147466037	0.0984748357504392	0.868308803285993	-0.0661519999999991	0.764973469318093
TRPT1	-0.157567308178617	0.601802147466037	0.0565484227337089	0.93176886339657	0.0753043333333348	0.765864161519786
BCL2	-0.157680460737486	0.601368599205503	0.153111735322114	0.774491384468427	-0.0658156666666663	0.774210549173745
IRF7	-0.157687640725706	0.601368599205503	-0.305237862570288	0.458013550008763	-0.156225000000000	0.589841732939668
SCD	-0.157764210877076	0.601126651235264	0.222545730630512	0.635668764102271	-0.573330999999999	0.085481985741103
FAM207A	-0.157791573159689	0.60102488402626	0.294040593454846	0.480297745849088	-0.296486666666667	0.189095480222886
CNTD2	-0.157822501701952	0.60096344084338	-0.0214673222072861	0.97536722461972	-0.0454986666666657	0.896906769316595
FTHL17	-0.157828009651261	0.60096344084338	0.208026708732189	0.66739374020401	-0.0737219999999988	0.866115937003813
EHD4	-0.157858382075137	0.60096344084338	-0.376938092672703	0.308367511671725	-0.156768333333333	0.464365096963932
OGFOD1	-0.157871699977957	0.60096344084338	-0.073633350512966	0.905838142287595	-0.346821333333332	0.0917903726055603
ATOH1	-0.157906714962030	0.600877760606069	-0.0818028411000807	0.89548747312664	-0.0343413333333327	0.896186784012643
FAM187B	-0.157969734297479	0.600546862354767	0.273437498213914	0.523839044577006	-0.30322	0.321313627659195
LPAR4	-0.158021240034354	0.600512488402626	-0.101880294720102	0.864616357134757	-0.0854003333333324	0.754561358073123
KRT31	-0.15803842736142	0.600491837136566	0.488243863203182	0.127979276934222	-0.0249073333333330	0.930383110415834
DHDDS	-0.158054484024518	0.600491837136566	-0.176883857682538	0.729794187782856	-0.799946666666665	0.00409813928101502
TNFSF10	-0.158055781521413	0.600491837136566	-0.389391524045613	0.283343454325927	0.743095333333334	0.0282821352636199
NPPB	-0.158095031926799	0.600483109763568	-0.152241453062752	0.775863712880333	0.0549220000000006	0.853077816975915
PTER	-0.158147405022701	0.600220663675328	-0.12888051466548	0.815391045978843	0.234665666666667	0.337786259836080
EDEM2	-0.158199935339310	0.600031425854916	-0.235983343104015	0.606121203124993	-0.160963666666667	0.46297633209186
PPP1R15A	-0.158221585296442	0.599979780485236	-0.478786072598904	0.140251433851464	-0.115504999999999	0.592975387754524
ZPLD1	-0.158303915029324	0.599808342517602	-0.140749450322613	0.796714214272984	0.0889550000000006	0.732554472781933
AJUBA	-0.158374585801488	0.599576819177974	-0.301657131097411	0.463258004901552	0.141680333333335	0.579643371835585
SEMA6C	-0.158385965096749	0.599576819177974	0.196015906824629	0.692325064566574	-0.000747666666666657	0.997953056544486
GUCA1B	-0.158402800817958	0.59954392913783	0.36617175266633	0.328778006159436	-0.0233799999999997	0.935261323444314
GET4	-0.158402802146373	0.59954392913783	-0.0702142721732026	0.910394157189308	-0.228735333333332	0.223260757343954
PARD6A	-0.158454983437072	0.59954392913783	-0.113617770636317	0.845326910026624	-0.0672966666666661	0.789228734235839
FAM49A	-0.158541795427247	0.599170375523223	-0.239589660068401	0.598769383991889	0.0304920000000007	0.910581676882346
PHPT1	-0.158557701247548	0.599142410990391	0.389722523149743	0.282848386985187	0.223354000000002	0.287956132210494
LAIR1	-0.158641483164775	0.598777388948049	-0.423824895054357	0.217939507033383	-0.0677503333333326	0.840657236939036
CD200R1	-0.158657428726593	0.598777388948049	-0.184978054721514	0.715658781310868	-0.0155236666666667	0.955460918122607
EIF4H	-0.158687348232366	0.598754911183855	-0.0855897621307662	0.889266195398476	-0.0822049999999996	0.7095294172582
SCGB2A2	-0.158698674567786	0.598754911183855	0.12178466431483	0.83097116342043	0.0499826666666667	0.83575292572393
LOC151121	-0.158703904312347	0.598754911183855	-0.215954914463374	0.64844945813838	-0.00230366666666594	0.995364835892372
ORAI1	-0.158726893145878	0.598723620158138	-0.250923988977001	0.572882168047464	0.0336490000000010	0.904507944803624

PPP4R1L	-0.158776550757555	0.598698976244614	-0.10479479971803	0.86067831050617	0.0733013333333337	0.775273488714107
GPR19	-0.158786205913580	0.598698976244614	-0.0421834450231836	0.951898632477817	-0.0912569999999994	0.664067525814732
DNAH9	-0.158853722554633	0.598428745755899	-0.103976219866299	0.861570109612412	-0.0540803333333321	0.82667899725046
LRGUK	-0.15885764421713	0.598428745755899	-0.289122103862265	0.489048761714482	0.0998020000000004	0.6846855637534
AVEN	-0.158863523977007	0.598428745755899	0.136416516617436	0.80479744641537	0.0994916666666671	0.636490807097692
CNIH	-0.158872268615625	0.598428745755899	-0.0824620046774773	0.8939835772794	-0.0292089999999984	0.909728882572794
FAM73A	-0.158894239993027	0.598428745755899	-0.478407470727479	0.140664651993598	-0.103488999999998	0.662751767068109
HNRNPA3P1	-0.158899532109701	0.598428745755899	0.0504990526613627	0.940625181975713	-0.204903666666666	0.447489138604371
SNRNP27	-0.158955643403819	0.598219788143589	0.0893387175600809	0.883460564717377	0.0920476666666666	0.671766969198954
RASGRP4	-0.159000259671788	0.598090704606163	0.199159733100331	0.684571461406347	0.020572666666667	0.955460918122607
RRAS	-0.159007788939647	0.598090704606163	-0.19979361212047	0.682777037002814	0.407252666666668	0.233528575266801
BEND7	-0.159036058842020	0.598090704606163	-0.238680614568531	0.600861960738847	-0.0124116666666666	0.97201219963332
KCNK5	-0.159047475597620	0.598090704606163	0.450658814369215	0.176663460992485	-0.126261666666666	0.558494409261702
TPMT	-0.159049303816592	0.598090704606163	-0.250365347952402	0.574011354423951	-0.0211226666666662	0.942393983671961
DFFA	-0.159056052077979	0.598090704606163	-0.416139226702703	0.234713120194856	0.0434250000000012	0.874908057954497
FABP7	-0.159111011333832	0.597965042511959	0.00812584937732972	0.990248011892148	-0.0306083333333329	0.903788355106891
FMO9P	-0.159227270700312	0.597416642081719	0.159044647244066	0.765370124656741	0.0435026666666671	0.867268897859745
MBD3L2	-0.159255400987171	0.59740927699556	0.361840649492183	0.338556116268406	-0.168536866666667	0.477955084845205
IKZF1	-0.159298082552978	0.597209501733381	-0.341234997504952	0.378059079534561	0.079588666666667	0.755980648751432
VSX1	-0.159309750912171	0.597209501733381	0.184249218538255	0.717436396325728	0.0317656666666670	0.924674647382585
USP20	-0.159349237050648	0.597104037746032	-0.0662076357139888	0.916661988649658	-0.458754999999999	0.0802641563431964
SOST	-0.159379763939033	0.596982354574229	-0.0720784533617033	0.90824872013293	-0.273868999999999	0.443378989641463
ABCG4	-0.159382342872744	0.596982354574229	0.106717499229764	0.857937967416038	-0.126600333333333	0.522694084864109
AK3	-0.159423527143413	0.596850042904717	0.0730149943889032	0.906592046281011	0.0217536666666668	0.938783708088713
S100A6	-0.159639734017511	0.595865844161438	-0.348483002118045	0.361311804770494	0.318400666666667	0.104259732288170
PPAPDC1A	-0.159697144721396	0.595688466982995	-0.0726134918001024	0.907556175729082	0.0786726666666666	0.774104133965233
FICD	-0.159750015664455	0.595423602542082	-0.219569018399293	0.641226345931986	-0.189087666666666	0.343729765439488
TRMT61A	-0.159771173632410	0.595362166291943	-0.322010250927859	0.418593043824504	-0.165646999999999	0.450203306181989
PSKH2	-0.159805805258668	0.595214328102542	-0.0216406962922738	0.97536722461972	-0.242452	0.381082408187926
PTPRJ	-0.159822624269859	0.595180728041892	-0.572679324251756	0.0443803345694495	0.0212060000000003	0.93901998985171
NUDT14	-0.159865047486871	0.595057204895561	0.176547196347589	0.730152680173573	-0.0814699999999997	0.741438256509195
SOCS2	-0.159932861763544	0.594696670591727	-0.0403991656069088	0.952720585102517	-0.0365696666666651	0.896186784012643
FEZ2	-0.159969164966444	0.594538196677863	-0.178991271859506	0.725831949422059	0.281235000000001	0.158485868172990
SHC2	-0.159988142811635	0.594499566948031	0.0903217980335596	0.882116295100215	0.221360666666667	0.273606049050204
SEMA3A	-0.160109135022983	0.594160702726915	-0.282466443494672	0.503412540505745	0.142545333333334	0.514016836574791
NUP62CL	-0.160184744005524	0.593973123004289	0.0263457297133454	0.970617924205993	-0.105484666666666	0.626635388688185
SIAH3	-0.160210421067532	0.593882825094737	-0.0414377134334821	0.952098348256047	-0.118209999999999	0.705260317228378
CXCL17	-0.160314135026349	0.593305229940031	-0.295413485348323	0.478306608395718	0.0988806666666663	0.636490807097692
LOC400891	-0.160335055805859	0.593305229940031	0.087828489459325	0.885957779199557	-0.130058666666666	0.581570366864416
DNAJA4	-0.16036449124102	0.59319271290238	-0.323676761780537	0.414215860139795	-0.0626749999999996	0.78197285126323
C11orf65	-0.160405831707113	0.593002169279099	-0.1967727605349	0.690061983084583	0.101060000000001	0.62979571408014
KLC3	-0.160410606701833	0.593002169279099	0.0603870992622710	0.925649366494154	0.089604333333334	0.704370299831112
GPT2	-0.160427014066295	0.593002169279099	0.115113377072878	0.84409286304878	-0.272737666666668	0.1593236943391
TBC1D20	-0.160447946505302	0.592955066722877	-0.202735801203067	0.678020962437652	-0.123694666666666	0.584153892885306
GNAS	-0.160492424868541	0.592744464023904	-0.268660256251882	0.535014466593144	0.0933943333333347	0.653246784345488
PCSK6	-0.160496399634596	0.592744464023904	0.242321744124706	0.593949183900524	0.204906666666667	0.458097980931337
ALKBH8	-0.160523559731364	0.592744464023904	-0.194646203262893	0.69591561649809	0.0656003333333331	0.788418231875778
BTLA	-0.160559650482404	0.592610834906167	-0.103647862972876	0.861872724387044	-0.0108726666666665	0.977499755477523
NOP16	-0.160703968366608	0.591761398289023	-0.0421974457049833	0.95189442152251	-0.400927333333333	0.0618388608777955
DNAI2	-0.160750488689984	0.591612037175829	-0.0153642483299214	0.982725338620588	0.00112266666666768	0.99704422037925
SAMHD1	-0.160778361700037	0.591507873137673	-0.354478927157015	0.350084741815739	-0.0887610000000006	0.708811767049238
A2M	-0.160789991449579	0.591507873137673	-0.117491945315232	0.839675491536757	0.102998000000000	0.62420320780168
CCDC88C	-0.160945769339844	0.59073701765132	-0.230036345019833	0.619232314946967	-0.148299666666666	0.705698790800484
EIF1AD	-0.160966149623042	0.59073701765132	0.00853343751326165	0.989612051419321	-0.231306666666666	0.246462634148331
PLK5	-0.161020033517764	0.590481332072997	0.187969328862577	0.70798562655309	-0.106740000000000	0.613101809403562
CACNA1A	-0.161030436846332	0.590481332072997	0.0859111052219844	0.888382447758042	-0.0748803333333326	0.789917107854685
CNTN1	-0.161143591571883	0.589913990953418	0.104160587757581	0.861291561747018	0.356677666666667	0.251702996251189
FAM214B	-0.161163456734192	0.589913990953418	-0.218846095165166	0.642137448592891	-0.275906999999999	0.177614563220760
CCT5	-0.161231444911137	0.589648880523891	-0.469661918863115	0.150981051310306	-0.0768899999999992	0.758341714501375
SYAP1	-0.161377632291447	0.589159382660881	-0.0457316864040236	0.94547820490174	0.0138980000000005	0.9622178119912
MRPL28	-0.161429379421471	0.589051003277096	0.038315432412581	0.954545200586067	-0.0373533333333318	0.880858809336225
PIGB	-0.161432302153961	0.589051003277096	-0.0616708992073177	0.923246185283376	0.404281333333334	0.0562663106369921
MAP4K1	-0.161456052911381	0.589051003277096	-0.0560554325595552	0.932663091036367	-0.182882999999999	0.361971299676959
GNA15	-0.161465842597008	0.589051003277096	0.241253303061055	0.596019873098193	-0.140754333333333	0.646350580563869
NANOS2	-0.161549913788974	0.588725573433038	-0.0358459402664628	0.958354725588542	-0.109498666666666	0.764848773122078
RANGRF	-0.161552076016182	0.588725573433038	-0.109896034727931	0.851013616427418	0.152271666666668	0.449024658532967
OR51B2	-0.161570253479129	0.588725573433038	-0.0706920158295581	0.909955989390414	-0.286302666666666	0.226573267757968
AADAC	-0.161573868276319	0.588725573433038	-0.182661339762325	0.71862020095107	-0.151699333333333	0.441909622750662
EMC2	-0.161596212793659	0.588725573433038	0.0346868954789784	0.959404955370931	0.116360333333333	0.681182934234933
SLCO1B1	-0.161602606000173	0.588725573433038	-0.0780702132596308	0.899705053748212	0.045106666666667	0.870002591217953
ODF3B	-0.161703713481548	0.588410119528747	-0.0545568884676289	0.9344692810234	0.0605203333333335	0.838670919454043
RIMKLA	-0.161719599133654	0.58838277317846	-0.105584582984817	0.85956218633532	-0.0570179999999994	0.812792750346224
VARS	-0.161728389719713	0.58838277317846	0.00021424710569710	0.999860038008296	-0.421854	0.0525641773262741
TSEN54	-0.161742396950558	0.58838277317846	0.186523221715784	0.711334804690432	-0.0912599999999997	0.670071773006358
ENOSF1	-0.16175945942424	0.58835054179732	0.132797469523989	0.809588765729788	0.185413000000000	0.411784621423647
C5orf48	-0.161785556690351	0.58835054179732	0.00107664415054058	0.998516123023229	0.062627666666667	0.81742145099016
PLAC8	-0.161807287265016	0.58834160927066	-0.274840895913085	0.520095539214775	0.523827666666667	0.0600979871105513
SYNE4	-0.161863940376546	0.588128114199645	0.0290778075563588	0.96782691865619	-0.00425633333333196	0.991625835822726
ZDHHC9	-0.162021067713899	0.587347505174131	-0.15838134139287	0.766698150107389	-0.252238666666666	0.294300395506211
ARAF	-0.16203636262269	0.587347505174131	-0.248254936952536	0.577730323259556	-0.0422839999999982	0.859420591955611

ACBD4	-0.162063536195073	0.587298739743184	-0.0264310198126091	0.970427456927088	0.0793313333333342	0.722426540875015
KCNH8	-0.162089803585408	0.587279303604837	-0.267485608049741	0.536168282496512	0.0655640000000005	0.873454962131763
NDUFA11	-0.162111848824626	0.587212727858592	0.124679060517037	0.82479991054473	0.0622713333333345	0.776117761644999
CCNH	-0.162157746822779	0.587068059701643	-0.345708738196143	0.367005900580236	0.00640666666666803	0.9847596802354
CCNF	-0.162268522824169	0.586603154570441	-0.303004532876984	0.462105017205825	-0.159600333333333	0.458006502146897
CNKSR3	-0.162387625505647	0.586120376773977	-0.172287788162652	0.738567943867944	0.475366000000001	0.0755898373594719
C10orf113	-0.162493439030721	0.585519976670237	-0.204880618346149	0.673728362275223	-0.0818873333333333	0.759434553759299
CMAHP	-0.162510310995173	0.585486454403686	-0.296686900757583	0.475941682821927	0.0149490000000002	0.963180491237413
FOXD3	-0.162558564838293	0.585326976254543	0.124837290685832	0.824472060590171	0.105363333333335	0.620692670936735
ZNF839	-0.162599910904536	0.585185395858916	-0.122502086205689	0.82920723741687	0.213537666666668	0.261418363811501
METTL5	-0.162606598247084	0.585185395858916	0.00459954441916203	0.993596029218977	0.00253300000000059	0.993756838831997
LOC100506557	-0.16276511519034	0.584899129658514	0.098968011193225	0.868042453226125	-0.138025666666667	0.6846855637534
FAM50B	-0.162801612534830	0.584740646065524	0.0109784209632834	0.98774001793194	-2.33333333335026e-0	0.999822702792133
CDH8	-0.162805458633196	0.584740646065524	-0.183485967688204	0.718067440349544	0.0753280000000003	0.77377928175062
SELRC1	-0.162841649683546	0.584633659980496	-0.424747915670511	0.216172456323230	-0.245221666666666	0.220551269970760
TMEM151A	-0.162860676756716	0.584586490048986	0.0951331113841814	0.872962271153999	-0.0190443333333333	0.94678260229888
ZFPM1	-0.162898473618105	0.584419769587582	-0.0649412066724565	0.918489100363712	-0.218891333333332	0.338230698383994
PRSS27	-0.162905773434726	0.584419769587582	0.152411081111942	0.775863712880333	-0.0328683333333329	0.91148338318481
DPEP2	-0.162967743443920	0.584274714115258	-0.258457398680497	0.560117060712577	-0.0929079999999991	0.757015566207214
SCML4	-0.162976203176234	0.584274714115258	-0.141348055508120	0.796285336930078	-0.00638733333333254	0.988133186272453
SLC23A1	-0.162982482424772	0.584274714115258	-0.0789765232106906	0.899154242391172	-0.0562059999999985	0.81742145099016
RRAGA	-0.163002884607742	0.584274714115258	0.0966899860930748	0.870857630743202	-0.0633893333333326	0.788174907829831
SNX5	-0.163044130440927	0.584200294774187	0.0424303434254568	0.951446425515852	-0.26316	0.159421950325134
MED12	-0.163065608126249	0.584169825493692	-0.253486576688327	0.567705016149998	-0.00905899999999831	0.983159241812861
C6orf1	-0.163095268744741	0.584097582794797	-0.100855558629870	0.866005093242444	-0.176518	0.393699610707377
LPAR5	-0.163114181996033	0.584082701253007	-0.177039275740252	0.729521332004078	-0.107734333333332	0.768318188305035
ATP6V0E2-AS1	-0.163115425965743	0.584082701253007	-0.300557192141924	0.465512153299176	-0.166303333333332	0.4504504175482
RMI2	-0.163125145552045	0.584082701253007	0.117969645324370	0.83857664717318	-0.00872333333333317	0.982375192575954
UFM1	-0.163129349802272	0.584082701253007	-0.0602528675148075	0.925649366494154	-0.00360383333333287	0.99117823918328
HAUS6	-0.163134681697934	0.584082701253007	-0.111608215594573	0.84862264853383	0.123448000000001	0.562399033626288
HAS3	-0.163160055798132	0.584082701253007	-0.188918838062705	0.707129289207266	-0.0941053333333318	0.691940834235362
MST1	-0.163182883941249	0.584082701253007	0.0553319332276094	0.933423044209716	0.193375166666667	0.342825265121927
KIAA0141	-0.163203602906460	0.584031970012284	-0.205368806196764	0.673088798709623	-0.0914953333333326	0.691236434401544
PCTP	-0.163268790455602	0.583839129823375	0.201840548079498	0.680054545720343	-0.386204333333333	0.055099483894989
BDH2	-0.163288418757175	0.583788246012145	0.00118330084134402	0.998383694263455	0.163972666666667	0.428358173426632
NLRC3	-0.163348971285874	0.583533537424062	-0.28831216078239	0.490393840170573	-0.00557099999999907	0.987937064305561
KLF5	-0.16337117905295	0.583483522586652	-0.327907879068694	0.407671070974377	-0.158228666666666	0.449828629541335
BCORP1	-0.163386358626121	0.583460998793952	0.402951436972148	0.258758728765188	-0.731058	0.00629285391915396
CAPG	-0.163458465962598	0.583408259360654	-0.315635941215385	0.434276316037369	-0.0931179999999988	0.794273694600852
CYP4F11	-0.163492395875113	0.583373629107629	0.176561942999290	0.730152680173573	0.0396176666666673	0.880830734199068
PEX12	-0.163552178338646	0.583072462637914	-0.0610025374409051	0.924825206377811	0.230591333333334	0.274865377740992
ABLIM3	-0.163593913277913	0.58295504947435	-0.206780172885851	0.670360323581237	-0.234197999999999	0.472363982227685
GALM	-0.163605357001458	0.58295504947435	0.123041255076356	0.827904654392021	0.0923770000000004	0.687787402649246
UFC1	-0.163618736097742	0.58295504947435	-0.146678224950328	0.786187758946602	-0.104872333333331	0.625510328921698
TMEM8A	-0.163655966936314	0.58295504947435	-0.0549477981425556	0.934246366517164	0.123647333333333	0.560413476606931
ESPN	-0.163690080214256	0.58295504947435	0.178326413384548	0.727335836218988	-0.209291666666666	0.549323798856043
BOLL	-0.163741961357419	0.582754446936419	-0.396325672468856	0.271136524502442	-0.0332993333333328	0.897291389279896
TTLL13	-0.163752785369226	0.582754446936419	-0.190050211490070	0.704348376269734	0.0687661666666667	0.760710284799861
SLC7A9	-0.163930867808729	0.582145426472867	-0.163280945737857	0.755849656740422	0.0367123333333337	0.893159646595003
ANKRD22	-0.163947004238219	0.582145426472867	-0.363977258369809	0.334843683313236	0.144296333333334	0.461494497197158
LINC00167	-0.163987418107418	0.582008145648513	0.00050558712650181	0.999374039351805	0.0228730000000005	0.946627610228776
GPR26	-0.163997982076569	0.582003701992584	0.220707054561524	0.639008702745457	-0.0998053333333322	0.775858800113142
P2RX1	-0.164028649029520	0.581896124070737	0.00538860525046514	0.99251028338798	0.0873560000000002	0.750961152956367
CXorf56	-0.164036387071487	0.581896124070737	-0.430888444386607	0.206533702654214	-0.742191333333333	0.008129182209396
SCCPDH	-0.164259640446694	0.580795187164649	-0.327130147247164	0.408851707589499	0.0649633333333342	0.774104133965233
TCTE3	-0.164283036156113	0.580720560965735	0.533919160547722	0.0758152544692281	-0.100404000000000	0.675221998008387
RAB40A	-0.164369237705884	0.580290652761879	0.0710396963461298	0.909387602167883	0.0582209999999993	0.809979063354947
GABRA4	-0.16441187789198	0.580197892364265	-0.138657751905299	0.79914918957102	0.202437333333334	0.351980210440002
ACTC1	-0.164501911542709	0.579847982945758	0.00365971508470749	0.994588964700772	0.274797333333334	0.151097325334439
SLC26A8	-0.164555046217983	0.579620913171064	0.0216578560298757	0.97536722461972	-0.0160003333333332	0.956015594359323
NINJ1	-0.16456793834905	0.579620913171064	-0.0370263814487255	0.95664230789783	0.0807593333333341	0.76648935876098
WDR16	-0.164578826915322	0.579620913171064	0.254522816626318	0.567585390815808	0.0567296666666668	0.820422171682127
RNF2	-0.164592042903127	0.579620913171064	-0.200382499293638	0.681033026144457	0.357809666666667	0.195651414003911
P2RY13	-0.164601757789827	0.579620913171064	-0.330355519304491	0.402284079938133	0.0281613333333337	0.914174691010705
KDM8	-0.164609435653937	0.579620913171064	0.195745139762855	0.693267493630778	-0.0826358333333321	0.73035482056318
POLR3K	-0.164618002761503	0.579620913171064	-0.00638633351876183	0.991853352023739	-0.0540816666666649	0.821526499502248
GGT6	-0.164707547582831	0.57953767367695	0.287199621487847	0.494100958493577	-0.0336676666666652	0.91869635463323
HGFAC	-0.164778677255916	0.579378544437305	0.0436542993426592	0.949137499717956	-0.00342333333333233	0.993028550445218
P4HA3	-0.164805119932331	0.579330408981646	-0.157002674922275	0.768401006769299	-0.0345119999999988	0.93414152758924
MC1R	-0.164831311741287	0.57931243070622	0.094265873215180	0.874645913015335	0.0532510000000004	0.821421133422134
MT1E	-0.164892734507002	0.579074921036525	-0.0183922046180032	0.979133715162458	0.330978666666668	0.128957627033936
SLC39A4	-0.164981994127021	0.57872588071401	0.192852512718153	0.699734841350316	-0.0563803333333338	0.846213226883859
ULK1	-0.164986359669072	0.57872588071401	-0.088149445200221	0.885569889492087	-0.296778333333334	0.204717851336330
SFTPC	-0.165139892249422	0.577945371410434	0.179299173503747	0.725260813023794	0.0378183333333334	0.89945786942875
MYL9	-0.165140384833923	0.577945371410434	-0.144875986202368	0.789487069585542	-0.115519999999999	0.670113640649168
PLEKHS1	-0.165155241446673	0.577945371410434	0.146645003622741	0.78622159423212	-0.0184796666666663	0.96313758691619
SLFN5	-0.165189527805723	0.577926948562990	-0.302028120444171	0.462229407853213	0.134594666666668	0.653246784345488
CENPH	-0.165238586072913	0.577763801339212	-0.109731251957360	0.851195083614214	0.382399333333334	0.116390284444001
OR4D2	-0.165283284440380	0.577554153809517	-0.0367949080722837	0.956918184553224	-0.163267000000000	0.445344763359555
CAB39	-0.165373918391498	0.577349721884681	-0.174256894732770	0.734267769889068	0.0214440000000006	0.942393983671961

HLA-DQB1	-0.165408034888519	0.577274094856667	-0.276108686583302	0.517549833373363	-0.133886888888889	0.569721048383279
EIF4EBP1	-0.165420860316746	0.577274094856667	0.0748858440705509	0.904466414650249	0.0545793333333338	0.821587043214193
TUBAL3	-0.165436371996477	0.577274094856667	0.0626440514989482	0.922350559812086	-0.106328666666666	0.743648472199156
TMEM129	-0.165443023128930	0.577274094856667	-0.125105137866200	0.824230922676958	-0.0577373333333325	0.80181116616697
DNAJC15	-0.165548387138444	0.576910119736639	-0.268666597477261	0.535014466593144	-0.640305666666666	0.09418645408026
FAM90A1	-0.165559717075912	0.576910119736639	-0.396937185640415	0.270862229167822	-0.0303456666666667	0.94678260229888
HSBP1	-0.165592882668282	0.576776139010994	0.0439603607543238	0.94865610372986	0.0992410000000017	0.664196112369643
NAA25	-0.165606325223881	0.576776139010994	0.106341983659279	0.858284876027025	-0.187978999999998	0.468574104598679
SAMSN1	-0.165713658293561	0.576381531833468	-0.447784186349076	0.178249202877872	0.080044666666667	0.706573150130005
PTPN2	-0.165729736791018	0.576381531833468	-0.340521301932691	0.378350877728096	-0.166522666666666	0.42608701620641
TSPYL2	-0.165764582341131	0.576381531833468	-0.326027531338095	0.409206882509236	0.228125666666668	0.274121181062751
MTM1	-0.165770700850482	0.576381531833468	-0.448632241637069	0.177860736739947	0.332896000000000	0.189095480222886
MON1B	-0.165860008438229	0.575971770425256	-0.457765614208275	0.166671273245692	-0.602689999999999	0.0383100402447561
USF1	-0.165882642684528	0.575902405398672	0.302244315942021	0.462229407853213	-0.0756206666666656	0.739465662522697
CLCN6	-0.165889815110761	0.575902405398672	-0.136194039754036	0.805279303793879	0.0784913333333343	0.719839702715467
GPX3	-0.165969524904436	0.575691360158352	-0.134163138298766	0.808009477165608	-0.0710149999999992	0.76307213318662
PPP1R14B	-0.165989102042296	0.575672545291101	-0.130472177218736	0.812271254601992	-0.131091999999999	0.527636614093972
CD180	-0.165989427254375	0.575672545291101	-0.172342748378797	0.738556507224474	0.0113920000000001	0.97318264525334
EMP3	-0.166014859970421	0.57565770092062	-0.381551542199008	0.297667425121795	0.262856666666667	0.287956132210494
LIPT1	-0.166046865877845	0.575528907388846	-0.321037641161801	0.421618886526906	0.0997759999999998	0.718749100405876
PSMA8	-0.1660939947067	0.575304180117375	-0.0560546931971073	0.932663091036367	-0.0306226666666667	0.90945379489823
NEIL2	-0.166097647319109	0.575304180117375	0.36625615481597	0.328771652750088	-0.237736666666667	0.226499104403529
UBL3	-0.166157133795528	0.575126507908518	-0.244764431129259	0.587430330647728	0.175967333333334	0.416412641573293
DUSP19	-0.166181220727683	0.575048006722114	-0.230107366318668	0.619017120481033	0.303338666666666	0.112438115304648
LRRC23	-0.166199766435383	0.575048006722114	-0.357461990449746	0.344739210399732	0.00494133333333345	0.987937064305561
SEPW1	-0.166336428942718	0.574396362822699	-0.124117188930774	0.825526959189789	0.116099000000000	0.590515735789471
SLA	-0.166338251892230	0.574396362822699	-0.426315040659694	0.213566074403563	-0.0392869999999995	0.890511682678135
AXIN1	-0.166540217338615	0.573588923736049	-0.102750669219088	0.86257932725575	0.00131033333333395	0.996285985364494
VENTX	-0.166560124807313	0.573537011332261	0.0608673682286619	0.92506536457632	-0.166384666666665	0.629657237462109
DHX58	-0.166562706653553	0.573537011332261	0.0193042320652663	0.978109081170804	0.0921360000000008	0.687454633819577
TCTEX1D1	-0.166759528541413	0.573016081609163	-0.148795537078303	0.783041584899534	-0.0425733333333327	0.859299487874574
P2RX7	-0.166768444470550	0.573016081609163	-0.145974794287346	0.787402233709509	0.239370000000000	0.255595078844894
TMEM87B	-0.166842589204036	0.572786955239514	-0.313069786546156	0.438459883259885	0.298126333333335	0.199320456083337
SHH	-0.166962122126345	0.572252733927971	0.0317598490351202	0.963456776578697	-0.245414333333333	0.335948256761884
CDC25C	-0.167158223683578	0.571456819322852	-0.155892269437388	0.76972820876143	0.121193333333334	0.562399033626288
TRMT2B	-0.167162547298658	0.571456819322852	-0.622171182669386	0.0236222757736635	0.0125740000000003	0.97097048443105
NCF2	-0.167245046563094	0.571145991702399	-0.307897468137756	0.451652892672575	-0.0336869999999994	0.903274690789156
C2	-0.167261924890447	0.571145991702399	-0.277345032003064	0.5144731963081	-0.167183111111111	0.379613779505161
TMEM239	-0.167312856465882	0.570997846072943	0.0329995133000803	0.961277493638149	0.0719503333333336	0.787739636244196
LRRC15	-0.167331874284836	0.570951735387985	0.0176797592209471	0.980278729770454	-0.0616306666666668	0.841174975800285
PRB4	-0.167367019841876	0.570803629774848	0.0943398853176453	0.874531031957077	-0.114398666666666	0.584153892885306
ZCWPW2	-0.167368119763439	0.570803629774848	-0.192106558221987	0.700820605802046	0.0583706666666672	0.840671985408282
APOC3	-0.167431869454149	0.570764430226347	-0.0925200475964582	0.878354232996353	-0.0806030000000003	0.789406063308412
GRIN3A	-0.167437022840640	0.570764430226347	-0.080402265432762	0.896965035803767	0.0931180000000013	0.81562142081933
C3orf67	-0.167499611321371	0.570667673127201	-0.228688008967858	0.622407163662984	-0.0499979999999993	0.829384095613517
LOC100129427	-0.167603087606574	0.570201099881215	0.172469113894591	0.738421727152922	-0.0554109999999996	0.822657709336468
LIMD2	-0.167616894153788	0.570188011112987	0.0924352308115768	0.878679925849405	-0.145760666666667	0.503217861354116
TST	-0.167639369850304	0.570120112178033	0.122383799209978	0.829398647947819	0.082509000000001	0.710456701900109
ZNF675	-0.167666778626086	0.570021029729154	-0.155258491947140	0.77058108906698	0.382623666666667	0.0581863609400333
EPHA2	-0.167674304386928	0.570021029729154	-0.442924126254786	0.184912742974902	-0.0615639999999986	0.808149567980568
TTC18	-0.167677698944357	0.570021029729154	-0.121948050505467	0.830347805834363	0.0966993333333338	0.772139923817113
GPN1	-0.167690130266294	0.570021029729154	-0.189575472192959	0.704928430617595	0.00353700000000035	0.991503437936314
C11orf42	-0.167735082799264	0.56988606906824	0.152209621235647	0.775863712880333	-0.0067436666666666594	0.9879739922351
LOC729173	-0.167756967591864	0.56982193825055	0.0400121244031132	0.953370115695118	0.0146420000000006	0.973686596735315
GPR115	-0.167796101237352	0.56982193825055	-0.333580332290307	0.396821740334178	0.123707666666667	0.550838204655945
LACE1	-0.167807450296053	0.56982193825055	0.0657536197343171	0.917495226662096	-0.0116719999999990	0.968696337092954
TPI1	-0.167946460335589	0.56943535764532	0.214499103510887	0.652454084593524	-0.0098236666666666618	0.974451420920923
CTNNA1	-0.167998261009795	0.569336168744494	-0.248807862960556	0.576871285544251	0.0492833333333355	0.828866908964934
GUK1	-0.168036398236649	0.569169364738195	-0.196080406052837	0.692325064566574	0.0127710000000004	0.976450907307814
IFT172	-0.168147730148068	0.568688561841434	-0.227778872095062	0.624564310654617	-0.180314333333333	0.419081654123944
COL17A1	-0.168184473149763	0.568530662681084	0.3369214745185	0.38850384336282	-0.0950893333333333	0.733679380704515
TARBP1	-0.168216407336006	0.568403163477102	-0.322218449506613	0.417586514434567	-0.385905999999998	0.0660125264725009
GLIPR2	-0.168237811411384	0.568342195247301	-0.368844872810755	0.32362669860138	0.196743666666669	0.408649882702122
UNC50	-0.168304220890590	0.568071207221548	-0.114085018596965	0.844272085764738	0.0395613333333333	0.888289502674391
WDR61	-0.168334197731723	0.568030354633703	0.146694155888833	0.786187758946602	0.0246340000000006	0.924674647382585
MSRB1	-0.168357810514325	0.56795546725295	-0.225056730371616	0.63103711743427	-0.206061666666666	0.273473953027795
MKNK1	-0.168392331554927	0.567923455745915	-0.132254403576405	0.810300414153187	-0.107737999999999	0.639703992089908
C14orf166B	-0.168405473894885	0.567923455745915	0.160573388322995	0.761610418244687	-0.0100806666666656	0.973783155960644
ILVBL	-0.168410920953591	0.567923455745915	0.302294188403537	0.462229407853213	0.017039666666667	0.963180491237413
LYSMD3	-0.168431046260189	0.567923455745915	-0.471604444253268	0.146991015246019	-0.0108546666666657	0.978504228748436
GPC6	-0.168438385623919	0.567923455745915	0.0764802025163256	0.902679569009162	0.230959	0.370523714900868
UBAC1	-0.168454055381297	0.567923455745915	-0.142666281873783	0.794004622916083	0.102237666666668	0.68135180147671
TEX26	-0.168469422916523	0.567919011889665	-0.125922301938316	0.822438385407788	0.0216353333333336	0.942092082409632
DCST2	-0.168487227175523	0.567902137561487	0.198643558942916	0.685751316700852	0.0255243333333332	0.931714916765969
NRXN1	-0.168511007135715	0.56789770070593	-0.257383302851896	0.563116080558022	-0.0292736666666659	0.911600329626215
ORC6	-0.168609479997659	0.567703310397148	0.0125319960509530	0.985220352735461	-0.133499999999998	0.549323798856043
SPEF2	-0.168636793125912	0.567605120912938	-0.145493624339725	0.787804113309913	0.122819000000000	0.541939010010167
NPAS1	-0.168640552830344	0.567605120912938	0.203636933993436	0.676207233014711	0.00619033333333341	0.984897785247171
NDUFV1	-0.168682429219287	0.56746556439381	-0.381952826179399	0.296704090260013	-0.254374000000000	0.21325573490485
POLR3B	-0.168735518766825	0.567278950204243	0.124606329562827	0.824974271393593	-0.135080000000000	0.580149967309238

CDKL3	-0.168784784246916	0.567116523802183	-0.100452420494836	0.866539251141083	0.214084000000001	0.414405354802607
NDST3	-0.168844851901608	0.56700839212582	-0.0470342243001911	0.944081286115546	-0.0325849999999999	0.903274690789156
MOV10L1	-0.168881803532602	0.566875688209449	0.198998656953484	0.685167881041453	-0.0313439999999995	0.942369384411502
GSTT1	-0.168905607556972	0.566799747542342	0.144259520095102	0.7909473604752	-0.142024333333333	0.592255902760106
ARHGAP5	-0.168918804356096	0.566790801314169	0.0123538714132708	0.985382317080831	0.193976333333334	0.353131201082797
C14orf183	-0.16896922789688	0.566546779323342	-0.0807919837647364	0.896509784694312	-0.0371436666666661	0.88384908879607
NHSL1	-0.169181015295961	0.565441603545533	-0.249553204879766	0.575370998761354	-0.170997999999999	0.474123250726025
MICALL2	-0.169270918036531	0.565089189880341	-0.257211331064677	0.563116080558022	0.0424920000000008	0.87709801222343
NCAN	-0.169327066906841	0.564994441048022	0.227439366651574	0.625658029930638	0.00858600000000023	0.979895563919825
GCDH	-0.169334540649881	0.564994441048022	0.329650609390858	0.40407920884139	-0.099525	0.645783884392886
GRAMD2	-0.169350665598614	0.564994441048022	0.312547796591602	0.439613810927592	-0.0307479999999991	0.91148338318481
TRIM21	-0.169405895685286	0.564980947886495	-0.270569898037129	0.529679098673302	0.0427580000000007	0.899845504615556
ZDHHC17	-0.169439805990538	0.564841427394517	-0.144146761040099	0.791303782903891	0.0452646666666677	0.859681098303919
CPSF3	-0.169479046355265	0.564810888255011	-0.219810245244906	0.641035290381005	0.114162666666668	0.62774583137117
TRAIP	-0.169490717919435	0.564810888255011	-0.0255586786990252	0.97204937337922	0.0356396666666678	0.900638103014907
SERPINB8	-0.169505544075276	0.564810888255011	-0.308862571516150	0.449515205044353	-0.0599079999999998	0.816938826104806
TBC1D23	-0.169514453834572	0.564810888255011	-0.248890903803559	0.576871285544251	0.148581666666668	0.461708697641046
WDR31	-0.169547652696336	0.564775090476322	-0.0620203373493105	0.923034515346352	0.140325666666667	0.504243893162425
RSL24D1	-0.169613316353802	0.564565144882512	-0.110898565926560	0.849357727620213	-0.0266979999999998	0.919127710424584
FAM186A	-0.169624424802641	0.564565144882512	0.151234121888884	0.777604486471648	0.0100863333333338	0.974451420920923
CYP27A1	-0.169640620123014	0.564565144882512	0.0433920333984467	0.949662621515857	0.317397000000001	0.27703697704839
FCRL4	-0.169701688120648	0.564454257995365	-0.0870112015184997	0.887055939766881	-0.0337236666666666	0.899981310623965
CHST2	-0.169718312205894	0.564423839890946	0.0329316178226769	0.961277493638149	-0.155491666666666	0.468233917603533
MMRN2	-0.169800913438652	0.564103977676077	-0.357681082054843	0.344139573942358	-0.164218333333333	0.40610776759241
TRIM24	-0.169872104949113	0.563826155298314	0.041716633895431	0.951989245458556	0.0288900000000001	0.910663650054071
GLRA1	-0.169927100745188	0.563738915153322	-0.0625339056336394	0.922632631649811	-0.141748	0.558807324932864
TNNI3	-0.170015728804725	0.563292883761724	-0.0978203882638254	0.868852648210739	-0.104244666666666	0.718192552235644
DEC1	-0.170048199323229	0.563162659670178	0.125603352304233	0.82367707928204	0.0562903333333334	0.814485009599967
GAB3	-0.170116168359946	0.562888927395928	-0.174698533046813	0.733930905697091	-0.144606999999999	0.465948865963889
PFDN2	-0.170190945686141	0.562837385752006	-0.0550448357767978	0.93406507597279	-0.132238666666666	0.503352066997863
CHAMP1	-0.170206236224215	0.562836513431751	-0.181196188573552	0.721382789731275	-0.0511559999999999	0.829188536848723
DDRGK1	-0.170221351291019	0.562833597069082	-0.187727754101295	0.70844398068702	0.0440896666666677	0.85588286783827
CXXC1P1	-0.170258416400809	0.562731032077964	-0.0595950894280644	0.926662910513879	0.0462573333333335	0.850157189407213
PLA2G2F	-0.170269802831605	0.562731032077964	0.104642789942420	0.86067831050617	-0.209525666666666	0.503217861354116
WWC3	-0.170446605675723	0.561992152819201	-0.191512222648629	0.701635983922402	0.0828820000000023	0.765339817431245
CCDC68	-0.170484344916571	0.561828920430304	0.0611028016533654	0.92470690387802	1.289451	0.00311122808725912
CDC42EP5	-0.170571626176775	0.561502613307155	-0.0230329647624984	0.97536722461972	-0.0461159999999997	0.88652090319904
SLC4A3	-0.170639619667903	0.561364054139834	-0.146000907437986	0.787402233709509	0.0460083333333344	0.904201124848034
HPGDS	-0.170681113976071	0.561185413835225	-0.278114359147296	0.512275963576091	0.100712666666667	0.641044966033937
HMP19	-0.170714756300341	0.56104808147807	0.134791915822422	0.807867938002244	0.0618013333333336	0.809354786908034
RANBP17	-0.170720534974642	0.56104808147807	-0.359591235082399	0.341510139061518	0.118712000000000	0.592845916952892
HSD17B2	-0.170799705353729	0.560662345458606	0.193990030683058	0.696962749474912	-0.0143809999999995	0.96051270333788
SARS2	-0.170810256769396	0.560662345458606	0.201787408288711	0.680054545720343	-0.0338436666666659	0.896446774489185
CELP	-0.170853886139386	0.56051712072331	-0.183255692023997	0.718067440349544	-0.008473999999999971	0.987499363290182
NR2F6	-0.170880222228101	0.56051712072331	0.0545856109585802	0.9344692810234	0.288747333333334	0.151972204090845
GNA14	-0.170920718869001	0.560421686614734	-0.329726353880873	0.403821204945523	1.27843933333333	0.0305026159262473
IQCB1	-0.170954430726094	0.560284017217623	-0.306384658806318	0.455795528901388	0.0382396666666681	0.888181586513563
KLRG1	-0.171081073159454	0.559859459234287	-0.274095272689132	0.521425198453909	-0.0993413333333334	0.741438256509195
EBNA1BP2	-0.171175830137642	0.559486817376503	-0.152114135603419	0.775920338550028	-0.249887999999999	0.234508116088724
PLS3	-0.171246448187736	0.559117343433338	-0.246943249750685	0.581100266003797	0.382841000000001	0.0833725664556335
NRXN3	-0.171415341337953	0.558433331141246	-0.166628328293198	0.750457342031334	0.00240800000000051	0.993824047902985
ZBBX	-0.171517330252194	0.558010367408551	-0.222876061715408	0.635391356864593	0.00205033333333362	0.99597023247862
DDR2	-0.171528182329773	0.558010367408551	-0.11136655106453	0.848968422336654	0.0337553333333332	0.891410328517388
CCNJL	-0.171577438983377	0.558004357320309	-0.0265643605603557	0.970207612777188	-0.130092333333332	0.631408228698652
SUGT1P1	-0.171579950539963	0.558004357320309	-0.0240822461719888	0.974287863238062	0.660872333333334	0.0196564180365988
C17orf75	-0.171594084353356	0.558004357320309	0.0292847880915273	0.967655960277738	0.199443333333334	0.309421300309741
MRPL3	-0.171629181568233	0.557907551793801	-0.0927957195319307	0.877797216649453	-0.0746346666666654	0.747245436362979
DGKE	-0.171744876189868	0.557399056622211	0.0720312817204749	0.90837606217316	0.764994666666667	0.0218270528465094
COMTD1	-0.171769928098885	0.557316119881936	0.109170054003977	0.85260191154502	-0.0975920000000001	0.716517864791776
TBC1D28	-0.171788300809282	0.557275101983247	0.158862190881768	0.765573831304863	-0.00693733333333307	0.988133186272453
FN3K	-0.171812775394690	0.557195801087788	0.0359864665917586	0.958301184344066	-0.0343116666666664	0.896446774489185
SERPINB6	-0.171823852411177	0.557195801087788	0.166883226600667	0.750076857136007	0.0115293333333343	0.969895173471778
FAM193B	-0.171825752722562	0.557195801087788	-0.178056123014371	0.727939088352802	-0.0372730000000005	0.880586178469642
ABCC3	-0.171894947798848	0.55712048479011	-0.141119143837330	0.796333933122002	-0.0952593333333327	0.661362028309397
FYB	-0.171909171802637	0.55712048479011	-0.421854706640822	0.222348587392621	0.177811833333334	0.360275319863852
IMP4	-0.171948288175991	0.557024379667748	0.00185580195322233	0.99760061742039	-0.172286666666667	0.370208571402686
CTNNBIP1	-0.171949966407093	0.557024379667748	-0.289141113627905	0.489048761714482	0.047649666666667	0.850157189407213
LGALS4	-0.171970249383863	0.557024379667748	-0.218854624517052	0.642137448592891	-0.140664999999999	0.74424924420945
C12orf23	-0.171972685775773	0.557024379667748	-0.129944595347139	0.812998265961053	0.0219170000000024	0.950884054684253
SSX3	-0.172010197602327	0.557024379667748	-0.00916952776145756	0.98909166696601	0.0194606666666669	0.94871117680979
CD80	-0.172165104408200	0.556545927654593	-0.212618378345372	0.656431174441413	0.343635	0.0802890385571452
POLR3D	-0.172191382789097	0.556455418841198	0.111989010254435	0.847604542536677	0.0207760000000015	0.94622978991441
SSTR5-AS1	-0.172204165506617	0.556449602870108	-0.00254481400240011	0.99580901762095	-0.0334009999999996	0.90945379489823
ODC1	-0.172211964217120	0.556449602870108	-0.542918807728167	0.0666521078484436	0.140996666666667	0.503308350574223
ICOS	-0.172232237574757	0.55642225906403	-0.193656498740509	0.69772764496956	0.00443200000000016	0.990471306092672
COPS2	-0.172285661118568	0.556235850599864	-0.123088424452128	0.827798779676671	0.162113333333334	0.409335260696998
LOC151174	-0.172304760595319	0.556235850599864	0.237466128230854	0.603017682513881	-0.190695	0.648806832470653
PRAM1	-0.172332953943967	0.556235850599864	-0.00821015441916122	0.990168714322153	0.0045553333333341	0.991175862027014
AANAT	-0.172370840557172	0.556235850599864	0.152154642914698	0.775920338550028	-0.148749999999999	0.49971129098408
HMGB4	-0.172395855046663	0.556235850599864	0.327288200399686	0.40846136066764	-0.0297054999999996	0.91680951061779

NOVA1	-0.172437047418983	0.556235850599864	-0.32984797072366	0.40362877792936	0.323910666666668	0.120319574336004
PPM1K	-0.172444037164549	0.556235850599864	-0.286358139036197	0.495728580458409	0.428557000000001	0.100026144425143
KIAA1143	-0.172584832618746	0.555922578072389	-0.181933107532140	0.720096350499662	0.0807236666666673	0.713714407278061
CTRB2	-0.172606305272904	0.555862356443805	-0.0427193458569279	0.95079489434051	-0.08882116666666	0.707459630816261
DNALI1	-0.17263344020107	0.555766596021948	-0.147153435950590	0.785691396687707	-0.0504476666666663	0.838279488520094
JAKMIP2	-0.172641508393982	0.555766596021948	0.20359737354944	0.676207233014711	0.197246000000001	0.466098392080126
TMEM164	-0.172708844221588	0.555442468100264	0.00874536431917725	0.98946753270812	-0.478575333333332	0.113522165242196
DOCK11	-0.172731169174530	0.555376929285741	-0.505535493324243	0.105941223458825	0.246261666666667	0.259184854253064
PUSL1	-0.172810980172105	0.555078904679651	-0.206449048234665	0.671220485448239	-0.0633016666666651	0.789917107854685
ARRDC3	-0.172816225153504	0.555078904679651	0.0954070652772131	0.872828680033553	0.459988666666668	0.0366259498115423
PDCD1LG2	-0.172844508493631	0.555038573396773	-0.325650194796836	0.409206882509236	-0.0510766666666668	0.895093529187836
TPD52L3	-0.172868205493991	0.554964465315439	-0.00114695197484284	0.998383694263455	-0.0814603333333333	0.785482517274791
ERBB4	-0.172980816242022	0.554332767746228	-0.216348102983351	0.648391670659269	-0.102245000000000	0.623123457579993
TMEM174	-0.172984819661593	0.554332767746228	0.00342617545959250	0.994730189431381	0.0658433333333336	0.76896959884888
GMCL1	-0.173005508971888	0.554332767746228	-0.0292012102688767	0.967677112447108	-0.0655843333333318	0.82667899725046
KIAA2013	-0.173013470687647	0.554332767746228	-0.288168734083385	0.490967865482371	-0.0418175000000009	0.864702194246548
KLHDC7B	-0.173019325255461	0.554332767746228	-0.116351973839925	0.84214534364593	-0.0981809999999988	0.762750978903907
ABCA8	-0.173078148427717	0.554332767746228	0.298405631544814	0.471415509360565	0.166210333333334	0.390298381662209
MSC	-0.173084636086874	0.554332767746228	-0.140212119419506	0.797441782082887	0.00446833333333454	0.991175862027014
ZNF605	-0.173152376122437	0.554151506647409	-0.379903924922352	0.301206449134387	-0.00659733333333205	0.988133186272453
IL10RA	-0.173159248402322	0.554151506647409	-0.36308878445106	0.336332757540532	-0.0833579999999994	0.744787682877495
PDCL2	-0.173210384670127	0.553936927894716	-0.37529472224389	0.312251235927787	0.00403933333333314	0.991175862027014
TMEM106A	-0.173271417004742	0.553703440525446	-0.310522475314128	0.445142823947765	-0.0079274999999988	0.986350009478474
FSCN3	-0.173294035010385	0.553636223778826	0.0359136187350828	0.958354725588542	-0.177351666666667	0.461494497197158
VN1R5	-0.173328350026230	0.553495698920365	0.151928519616702	0.776173016352835	-0.0357399999999993	0.907648578460912
NDUFA4L2	-0.173331431028953	0.553495698920365	-0.13263338108961	0.809801552882903	-0.0698763333333326	0.808230250532513
CCDC127	-0.173419540122419	0.553222435032236	-0.0538613598046739	0.935404504538153	0.150543666666667	0.541939010010167
SERPINA10	-0.173425641073359	0.553222435032236	0.0676422295699753	0.914002587027341	0.0316860000000003	0.914174691010705
S100A14	-0.173442823900108	0.553222435032236	0.134368355939742	0.808009477165608	-0.0419803333333332	0.874960823800592
CNNM1	-0.173486009458379	0.553104179820668	0.0398430317032087	0.953601323357358	-0.0060906666666666	0.985263856374185
BPGM	-0.173521519324007	0.55295624349672	-0.400412421886835	0.262833489019055	0.0270726666666674	0.920633271571572
EXOSC3	-0.173562394255256	0.55277471684713	-0.260570706451293	0.553540075799904	-0.516389666666666	0.0265384577950610
FLJ38379	-0.173674100885988	0.552298691387173	0.0297518172530534	0.96695070705759	-0.107333666666666	0.612891410601889
ST3GAL1	-0.173721529615436	0.552225296995499	0.0404126592456921	0.952720585102517	-0.144832666666666	0.564495134161 5
GPR65	-0.173726668861334	0.552225296995499	-0.351142110731155	0.356603456853694	-0.0868036666666664	0.702512405158624
STAB2	-0.173796676826832	0.551903856313032	0.126778875171720	0.820923134671156	-0.0974116666666663	0.649118055813479
ARFGAP1	-0.173833103131697	0.551750354242422	-0.0984980412175066	0.868278775296491	-0.0603173333333335	0.782886750366221
CROCCP2	-0.173880388669888	0.55152891683729	-0.0521109859364575	0.938067483409541	0.0510620000000002	0.853219850381138
OGFOD2	-0.173915075448951	0.551386353909282	0.119643603816605	0.835740437824474	-0.166182333333333	0.503308350574223
TRPV6	-0.173931450419090	0.55135838014283	0.157486141446631	0.767770230680061	0.010102666666667	0.985183831169496
ZBTB7A	-0.174103038233723	0.550434184555867	0.0406580467825606	0.952426531595115	-0.126221333333333	0.535487400963825
ZNF215	-0.174421437809461	0.549040046654845	-0.140750743177080	0.796714214272984	-0.168698000000000	0.621406232563173
APRT	-0.174432148443491	0.549040046654845	0.0550577770953682	0.93406507597279	-0.0291869999999989	0.91148338318481
ADCYAP1R1	-0.174468899967069	0.548892359857139	-0.0152176580835285	0.982921609770846	-0.0660186666666663	0.879843042066371
C6orf118	-0.174502668472507	0.548824007709882	0.146422512822138	0.786957117162244	-0.072675666666666	0.756955502778872
COX6B1	-0.174580834327006	0.54849093669354	-0.167688117503998	0.748388724437592	0.0335976666666672	0.895093529187836
ERI1	-0.174654129543779	0.548182200724763	-0.0633411016755695	0.920965031490783	-0.150976333333333	0.622282705325595
KIAA1522	-0.174680604073997	0.548091313533899	-0.146080131614412	0.787402233709509	0.0647426666666676	0.811997756288597
RAC2	-0.174749324332175	0.547737013883157	-0.331510884742943	0.400099845811371	0.0272843333333335	0.922148106708426
KAT8	-0.174771784722783	0.54767118812667	-0.127878163164320	0.817865585528586	0.087977333333333	0.685271038967111
GRINA	-0.174795672779691	0.547596468946763	0.334115405853072	0.39515588300251	0.0396023333333338	0.90452008697883
PARK2	-0.174812654482499	0.547564809580266	-0.103520350728509	0.861943478503987	0.0217046666666670	0.957164262608967
PLCB2	-0.174863126167419	0.547398601488697	-0.1910401929857	0.702230712161583	-0.122310666666666	0.623988109816431
LARP1B	-0.17488563223592	0.547398601488697	-0.572367550756975	0.0443803345694495	0.0287003333333346	0.9299162364306
C7	-0.174908164601682	0.547398601488697	-0.114054170994401	0.844272085764738	1.23612266666667	0.0423336162071103
TEKT5	-0.174962277615764	0.547300142141731	-0.0929424634054726	0.87756010254812	-0.159149666666666	0.641068499284844
PIK3AP1	-0.175008160599817	0.547162629050547	-0.441766127019444	0.186378962686717	-0.200707333333333	0.479138506340968
SERPINA5	-0.175083897217720	0.546839080131259	0.126213993271094	0.822164329801713	0.0652200000000026	0.873454962131763
ZNF226	-0.175099390455995	0.546839080131259	-0.00055716070048906	0.999260526969206	0.253023000000001	0.2729560423704
KRTAP4-8	-0.175119506557902	0.546839080131259	-0.242467723062393	0.593398090075382	-0.123833083333332	0.575076472049916
ATP5S	-0.175157398580340	0.546678031632202	-0.143868930235416	0.791853933422817	-0.0984563333333331	0.717856757149127
ART1	-0.175163916225904	0.546678031632202	0.0476185785892042	0.943611925889785	-0.155421333333333	0.5293518047673
GBX1	-0.175229788346271	0.546375484688413	0.112205789858043	0.847454473507881	-0.143864999999999	0.613030354362634
IGFBP1	-0.175240754561403	0.546375484688413	-0.260488570244133	0.553690269045998	-0.0482070000000005	0.892921857967499
NAPB	-0.175255418781831	0.546364296630729	-0.121205149351116	0.83256316766566	-0.0878326666666658	0.74424924420945
TTF2	-0.175300164924393	0.546159791988469	-0.301294160374979	0.464457039761058	-0.229826333333333	0.310277378475854
ZNF438	-0.175320566789877	0.546106949800054	-0.393164960205332	0.277638470372668	0.125581333333334	0.656324975591247
EPHX4	-0.175367715390884	0.546036014932721	-0.207404977057186	0.668625825887966	0.0693266666666673	0.77130466329802
SERPINC1	-0.175446470098216	0.545631125100736	-0.136977019765958	0.803348305001716	-0.119866333333333	0.627930065742649
ALKBH4	-0.17561827999228	0.545218248214068	0.0579287962599258	0.929551121465474	-0.0298199999999993	0.909196271455697
COPS6	-0.175657175991156	0.545124617434911	-0.210522784473053	0.661670522548494	-0.00181999999999967	0.995321141422816
FOXP1	-0.175672622937693	0.545102717729181	-0.0582383522308256	0.928879154519933	0.399715333333335	0.0490477538807126
HSPA2	-0.175754585775221	0.544820489592269	-0.221352413726297	0.637903205057623	0.0671660000000007	0.798170083215805
ACVRL1	-0.175786409475337	0.544765772560862	0.00210876043957799	0.996803154560116	-0.204570666666666	0.285262512901751
HTATIP2	-0.175838750617738	0.544520527858095	-0.0317893084907977	0.96337759808105	-0.148438000000000	0.502766729725258
CCDC120	-0.175847458598007	0.544520527858095	-0.0685984417917083	0.912149839732916	-0.0375983333333335	0.878933944675937
HAUS1	-0.175866066063954	0.544520527858095	-0.151190790944208	0.777620728620974	0.182270333333334	0.338230698383994
APBA3	-0.175875307209328	0.544520527858095	0.00194766058889814	0.997338673493808	0.0656833333333335	0.809505390888975
RRNAD1	-0.175950859018319	0.544385633360112	-0.131468697649727	0.811042872465441	0.143422666666667	0.489999806074963
ATP5C1	-0.176030837533408	0.544136104622159	0.201818443516990	0.680054545720343	-0.0129059999999984	0.965329851466378

FAM46B	-0.176121879560980	0.543941206129447	-0.495199312962265	0.116831680799555	-0.128586000000000	0.590515735789471
H2AFJ	-0.176130530703260	0.543941206129447	-0.225474234653382	0.629710977741725	-0.00436199999999901	0.99106990984423
SCIN	-0.176216808386424	0.543647921402694	-0.262321029795926	0.549438168615748	-0.426385999999999	0.0579505597335047
EMG1	-0.176354865876690	0.543012268440046	-0.143293042035140	0.79287526984225	-0.132386333333333	0.635267430402483
SLC22A8	-0.176360280016853	0.543012268440046	0.230968001093738	0.616488238055513	-0.197352333333333	0.397798624983652
EVL	-0.176373832411492	0.543012268440046	-0.332390170494493	0.398142341492687	-0.138888333333332	0.513530370066639
JAGN1	-0.176434721630936	0.54280805500418	-0.083369428369081	0.892833228561937	0.0642140000000015	0.775273488714107
ADA	-0.176467892729915	0.542681058700327	-0.0887984042692224	0.8841943128258	-0.033483999999999	0.917325389804968
EPHB6	-0.176483285106581	0.54265966888729	0.0798008270369611	0.897892885174022	-0.0390346666666654	0.872231116211086
MED20	-0.176504614375682	0.542601383591983	-0.280664432945437	0.507460240190768	0.119852000000001	0.568903633175651
AP1S2	-0.176550560210092	0.542390121469643	-0.221123237989312	0.638286782439236	-0.0723033333333323	0.746473454296225
TRIP6	-0.176599830253625	0.542232510486825	-0.390369166712767	0.282383744168943	-0.095091	0.702709435280393
SH3GLB1	-0.176653121958736	0.54204994663482	-0.448486472412359	0.177860736739947	0.248919333333334	0.198429082320895
ETAA1	-0.176665273805988	0.542048720948448	-0.0156941303897726	0.982266426585123	0.349656333333334	0.100756942335889
TTC33	-0.176720308031169	0.541855377464398	0.130990634308655	0.812271254601992	0.201425333333334	0.430018092058755
CTIF	-0.176769931875761	0.541621423046491	-0.327019945882049	0.408851707589499	-0.00257666666666662	0.993756838831997
FOLR3	-0.176844753803768	0.541305307692555	0.176587362860252	0.730152680173573	-0.05411	0.891803584952102
ASB12	-0.176850776408082	0.541305307692555	0.0989383139299843	0.868042453226125	0.146463666666667	0.577524803794493
ZNF92	-0.176956451329073	0.541167829228148	0.0498929099196938	0.941458038227918	0.365021333333334	0.156315769387234
SPANXA2-OT1	-0.176974305765893	0.541167829228148	0.140137462800787	0.797441782082887	-0.0140066666666661	0.974451420920923
CLDN9	-0.177042604645381	0.541042385305938	0.205974841617814	0.672284583020815	-0.100374666666666	0.668862273626329
EOMES	-0.177080305459459	0.540956925815842	-0.336112573516455	0.389476012645306	0.183647000000001	0.340539387848675
LY6G6C	-0.177107332416689	0.5409377695393	-0.0371947755332386	0.956300346368081	-0.0174318888888886	0.954865522683112
C16orf58	-0.177155864015687	0.540785099697526	-0.077411818319982	0.90079793355475	0.0558073333333333	0.816134952712854
CYTIP	-0.177205339008129	0.540552270450162	-0.353583128927635	0.352236748429479	-0.0746443333333334	0.753531226165113
CHP2	-0.177208356754060	0.540552270450162	0.0944091822739746	0.874410047496143	-0.0222206666666659	0.955460918122607
PTGER1	-0.177226128485093	0.540552270450162	0.258610224708098	0.559935260647793	0.0402106666666669	0.885895416574211
NDST2	-0.177330876623472	0.54029337147072	-0.0594235290529432	0.92694538699714	-0.00792266666666664	0.98147292539484
SRPK3	-0.177425549742872	0.539928832141098	0.118382997618103	0.837937752076601	-0.166230333333333	0.39808310170806
MLYCD	-0.177435081273590	0.539928832141098	-0.095487343560288	0.872754579338908	-0.0157923333333323	0.962924081933534
KIF12	-0.177531911234206	0.539566246084202	0.234647294890137	0.608421850871805	-0.00978299999999924	0.978504228748436
CCDC88B	-0.177660231093001	0.538844825512782	-0.201673369241146	0.680054545720343	-0.0202419999999989	0.94622978991441
SLC22A5	-0.177773651090204	0.538438876476933	-0.0336971932064108	0.96061727828906	-0.188823333333333	0.441744486045496
SCRT1	-0.177782909894594	0.538438876476933	-0.122782122042731	0.828721578450338	-0.133905833333333	0.527636614093972
MT1F	-0.177850314620349	0.538186578681686	0.0232066531738115	0.975348968561756	0.109942666666668	0.654323362279664
PUS10	-0.177899558586902	0.53810420792964	-0.35852140740912	0.343101042014771	0.0141240000000006	0.960071163568134
GTF2H1	-0.177953505201114	0.537908903184623	-0.455237791059449	0.168366359836401	0.162695333333334	0.461494497197158
ITFG3	-0.177980759153552	0.537891957199365	0.0788476666344475	0.899154242391172	-0.180390999999999	0.415528744566332
SPNS1	-0.177983390030855	0.537891957199365	0.118744473108878	0.83731413798382	0.0221976666666670	0.93278021276477
CA6	-0.178007052050177	0.537883827145728	0.206251075943999	0.671467763213228	-0.0301609999999996	0.908590774398674
ABCD1	-0.178085591601497	0.53760444615845	-0.231989359107902	0.613445765196643	-0.348755666666666	0.0909486750308436
KDM4C	-0.178094762088899	0.53760444615845	0.373068045303461	0.316692858754573	-0.427869999999999	0.311354677197697
ATP1B4	-0.178101928618819	0.53760444615845	0.134882309453555	0.807867938002244	-0.0733763333333332	0.742665333187322
DDX55	-0.178215579627808	0.537112236273957	-0.093889146192912	0.875355472414883	-0.182192333333333	0.387438322663731
FCRL1	-0.178221737490134	0.537112236273957	-0.0828317706179894	0.89362638370288	-0.103547999999999	0.707538975864338
TMC7	-0.178260703807078	0.537055625125650	-0.209841116292262	0.663436500730575	-0.00785899999999994	0.98147292539484
RAG1	-0.17828652435021	0.536970033391514	0.272441342470362	0.526175116911121	-0.0192586666666666	0.946623914607177
SERPINA7	-0.178325639253041	0.536876416100451	0.00987716986834603	0.988964532103159	-0.160457	0.445566557024313
STAT6	-0.178465088253764	0.536310392396584	0.0218891079831989	0.97536722461972	-0.0464603333333321	0.864702194246548
PSMD9	-0.178483741243868	0.536310392396584	-0.416187719207737	0.234713120194856	-0.0162006666666655	0.958368815250687
CRELD1	-0.178548100657463	0.536310392396584	-0.132973729055919	0.809578511927685	-0.0300586666666664	0.920295464978618
ZNF668	-0.178555855341958	0.536310392396584	-0.165312078528201	0.752544465367249	-0.2581295	0.352237134604623
E2F3	-0.17858207202513	0.536254941469553	-0.207759207289258	0.667695745454683	0.0992706666666669	0.63011839593414
PPP1R3A	-0.178631995853957	0.536168930743189	-0.182705323130461	0.718559738568628	-0.149908666666666	0.496116634943666
CD79A	-0.178795959087669	0.535451733730357	0.137104366905753	0.802887234315834	-0.0760953333333326	0.761078552848883
LOC100499221	-0.178868050422174	0.535154478377253	-0.216629863252863	0.647400684192295	0.102397666666668	0.728894262229555
TCIRG1	-0.178917571131245	0.534996832312356	-0.163004300223386	0.756782785150973	-0.0368213333333329	0.92484471634727
TRPM2	-0.178934797007073	0.534964598434281	-0.152389572665138	0.775863712880333	-0.180785333333333	0.393584757087412
ANKS1B	-0.178958023849945	0.534964598434281	0.175714414953743	0.73185312470559	-0.0217600000000002	0.95450472002063
CS	-0.178967142328469	0.534964598434281	0.0413107517330937	0.952098348256047	0.00212333333333426	0.994956689715437
CCDC59	-0.178990536646574	0.534964598434281	-0.120119409299714	0.835124910268666	0.0989176666666677	0.73097308185803
TMEM64	-0.179026842249779	0.534841236014344	0.101269566973944	0.866005093242444	0.418255333333334	0.0411983064548969
MGST3	-0.179029854690785	0.534841236014344	0.0487086808155628	0.942249463542745	0.0750233333333336	0.764848773122078
COG7	-0.179115972893354	0.534587444941604	-0.168864395376973	0.74582180262941	0.160616666666668	0.465424659374538
ZNF354C	-0.179136821675286	0.534587444941604	-0.0834576128876343	0.892833228561937	0.152332000000001	0.489763389325619
AMIGO2	-0.179167580527955	0.534491378815025	0.235095366604939	0.607525465293757	0.600483333333335	0.0777324007395152
ATHL1	-0.179283155815834	0.533985936406026	0.0158437527936876	0.982102356159698	-0.0468369999999994	0.878139763624324
FAM9C	-0.179384303238328	0.533523780567667	-0.286336293867049	0.495728580458409	0.0251790000000002	0.931708923663607
CCNE1	-0.179409731660759	0.53351512426611	0.135044411788734	0.807573416774362	0.147607333333334	0.476169903463145
IMMP1L	-0.179422883675758	0.53350829745761	-0.0720949213219403	0.90824872013293	0.0249653333333338	0.942393983671961
TBCC	-0.179526643552295	0.532941932649583	-0.0238519597476235	0.97479225206569	0.0835763333333335	0.697187294779476
UCN	-0.179533288498454	0.532941932649583	0.326272068402787	0.409206882509236	-0.0772673333333327	0.732554472781933
THSD7B	-0.179601250990828	0.532667653170197	-0.440503447952031	0.189166199430598	0.360575666666667	0.120319574336004
NIPSNAP3B	-0.179623698341690	0.532639815649576	0.196415698107542	0.691445158350448	0.195843666666667	0.526986765016044
USH2A	-0.179639094092746	0.532639815649576	0.130177611184944	0.812280305294252	0.0590263333333339	0.7996066102903
LRRC57	-0.179682031599645	0.532639815649576	-0.16030146573367	0.762321667379314	-0.144518666666666	0.470695033055552
ZNF132	-0.179689749176494	0.532639815649576	-0.0797955646090114	0.897892885174022	0.226595666666668	0.310960271128485
PFDN6	-0.179704461378862	0.532639815649576	-0.361187231970383	0.339627732901974	-0.307137999999999	0.121420429767753
C1orf94	-0.179957209714136	0.531250738759822	-0.131697418008778	0.811012545371072	-0.0881656666666665	0.719839702715467
RCVRN	-0.179991314640295	0.53111470620826	-0.0762438055058221	0.903240096616933	-0.0690219999999995	0.833228738262614

MRPS25	-0.179996853169894	0.53111470620826	-0.457995703668949	0.166671273245692	0.0139213333333342	0.964241302457077
TXNDC11	-0.180021182345019	0.53111470620826	0.0604536506682157	0.925565513580637	0.0773896666666683	0.727239075127513
SLC19A3	-0.180028786880417	0.53111470620826	0.216710317979635	0.647357580911858	0.0358690000000006	0.910190419163007
C17orf66	-0.180104527803885	0.530861883293047	0.00941807030637506	0.98909166696601	0.171840666666667	0.410984651167943
CST2	-0.180110040903775	0.530861883293047	0.0602188120608077	0.925649366494154	-0.155802333333333	0.607009506431467
FGFR1OP2	-0.180115158685994	0.530861883293047	-0.0971331544558927	0.870020870354202	-0.159214999999999	0.506012676836737
KIAA0196	-0.180227127180231	0.530540509097309	-0.157595636304165	0.767770230680061	0.0729520000000012	0.777124097170335
SLC37A4	-0.180246940241497	0.530503553968386	-0.0326825803063480	0.96140116667763	-0.543115	0.0254667407680142
ZNF674	-0.180265347663983	0.530503553968386	-0.377921751469158	0.305766089617986	0.0407876666666669	0.911796922065088
SNCAIP	-0.180282572015099	0.530503553968386	-0.0248359179414397	0.972966238885768	0.405856	0.230276420854917
WFIKKN1	-0.180302432266083	0.530503553968386	0.352920255307795	0.352407344218756	-0.000925333333333264	0.997264570850507
ABHD10	-0.180337249540836	0.530392267655157	-0.289166111132684	0.489048761714482	0.149260000000001	0.477169587518779
DPPA5	-0.180347894231096	0.530392267655157	0.0833152737962811	0.892894681640269	-0.353678666666666	0.126748755872072
FIGLA	-0.180365336469345	0.530367640845098	0.140301366752364	0.797441782082887	0.138201333333334	0.511696593608944
EPHX3	-0.180366800509122	0.530367640845098	0.202848774076203	0.677604697677494	0.0411096666666669	0.890511682678135
TMEM225	-0.180413539758882	0.530219114477558	0.0117869184388599	0.986418801521542	0.0359380000000007	0.89052375260746
MS4A6E	-0.180438673586446	0.530212723284643	0.0445920553466368	0.947920610570169	-0.0644859999999999	0.769890867417328
ZNF28	-0.180446462511269	0.530212723284643	-0.109353941248494	0.85210721238532	-0.0750276666666643	0.778477148290034
EPHB1	-0.180460212787449	0.530212723284643	-0.411896258578369	0.243463779196104	1.17169866666667	0.00690186682374875
SLC5A2	-0.180464655344156	0.530212723284643	0.0621889034208777	0.92301259447339	-0.0382469999999997	0.914172701010275
ENPP5	-0.180465801793492	0.530212723284643	-0.109516519418964	0.851624566716206	0.720158333333334	0.0208784877318267
ZFAND2B	-0.180531849224636	0.530186306866615	-0.0107002084871136	0.98774587825743	-0.0340203333333328	0.891339318634358
UPRT	-0.180631027858801	0.529844605820954	-0.325675011021438	0.409206882509236	0.0490326666666675	0.845891599383498
IFNA8	-0.180655709045350	0.529841157089316	0.0275887760937500	0.96978432630736	0.0935833333333333	0.674780899769326
POP5	-0.180693312654616	0.529832451881243	0.0483542474792466	0.942820948997969	0.149076333333334	0.490110576940323
NAT16	-0.180717037055072	0.529832451881243	-0.0876428881309323	0.886302037299497	-0.338222666666666	0.188019918378354
RIIAD1	-0.180727630213399	0.529832451881243	-0.166110061264764	0.751830402832405	-0.123676999999999	0.595025868516587
TMCO2	-0.180781331120090	0.529587537337645	-0.0530592246922414	0.936712858731269	-0.0563126666666663	0.80972577068199
STXBP3	-0.180819465611928	0.529426964267317	-0.291271129623088	0.486802739867898	0.144558666666668	0.472370507912389
FAM65C	-0.180839943189374	0.529375168789793	-0.243006186424676	0.592178030679976	0.0852610000000008	0.762890197654898
C1QTNF9	-0.180863027117747	0.529307326174231	0.0947432592093462	0.873818349270144	-0.0865999999999997	0.83023589034143
LIPE	-0.18086625573764	0.529307326174231	0.00080948248909802	0.998745415362821	-0.107250999999999	0.597098546469434
TRIM33	-0.180875380423635	0.529307326174231	-0.390663094970935	0.282005640075858	-0.068935666666666	0.796894030375034
RPA2	-0.180922196760834	0.529215715986132	-0.355251296920214	0.347833413629182	-0.0567439999999982	0.824700707442131
GBP4	-0.180967714408072	0.529059740063638	-0.293985545144538	0.480319908043145	0.156966333333334	0.524845938106282
DHRS7C	-0.180990769814716	0.529041054612635	0.316623586811005	0.432178413930141	-0.070363333333333	0.829384095613517
CXCL13	-0.181012687152139	0.528980446020242	-0.299318825163594	0.468416778777124	0.0852090000000004	0.712688984009924
ETF1	-0.181082371778600	0.528700073235685	-0.473660685273498	0.145443641689931	-0.0386206666666664	0.873663396186202
PRKAG1	-0.181102179493847	0.528652479323775	0.0398844748091741	0.953525981777884	0.0804323333333347	0.709937184587543
DDX59	-0.181142695043645	0.52850274846159	-0.00021722219637658	0.999860038008296	0.212614333333334	0.269653172251380
PRRC1	-0.18116431795895	0.52850274846159	-0.268024181858847	0.53559545997875	-0.0158779999999984	0.95784037718285
GABRR1	-0.181175947060507	0.52850274846159	-0.173880013674820	0.735077316606986	-0.000561666666666620	0.998309716374356
GSTO1	-0.181193158775410	0.52850274846159	-0.00509993449039314	0.99251028338798	-0.0200743333333322	0.945904449997854
CD1D	-0.181221827404355	0.52850274846159	-0.288829801328351	0.489813374112312	-0.0779849999999992	0.73097308185803
RAB11A	-0.181303939032078	0.528377495776519	0.209342924655593	0.664703270325548	0.0826833333333344	0.694195087922618
PIGH	-0.181321709841703	0.528342523950722	0.122358395219980	0.829398647947819	-0.0451736666666655	0.846917872248932
PSAP	-0.181345900072242	0.52826802712842	-0.317395033154316	0.431166148274203	0.0792800000000002	0.723383077113569
CTAGE1	-0.181376423913621	0.528154540074411	-0.329049795523485	0.405801577460192	-0.0104446666666669	0.974451420920923
FAM83G	-0.181398689590171	0.528091910777086	-0.174555846473042	0.734267769889068	-0.0854543333333321	0.7615367624187
ADPRHL1	-0.181510602701021	0.527613362142924	-0.228001528688006	0.62419618079852	-0.0940216666666661	0.662666815705326
SOX2	-0.181550576045382	0.527529243337058	-0.302506383635531	0.462229407853213	0.153518666666667	0.5293518047673
COX8C	-0.181553923115508	0.527529243337058	-0.0267757271654865	0.970207612777188	0.102810000000001	0.68149943269806
DPYS	-0.181565075947805	0.527529243337058	-0.125373415603599	0.824013463206763	-0.142399999999999	0.572873019944314
SYCP2L	-0.181608744921098	0.527529243337058	-0.480943346727123	0.136628524900842	-0.0307153333333334	0.943464122996745
TFEB	-0.181733795081666	0.526848632613445	-0.39426323496053	0.274555703975637	-0.113120333333333	0.591142517306808
QTRT1	-0.181740794079066	0.526848632613445	0.213875564591479	0.653708954135733	-0.172162333333333	0.371797773837046
CLEC10A	-0.181838312610783	0.52635480236956	-0.178948537254044	0.72588422946971	0.00091766666666767	0.997144862160692
FAM192A	-0.181865485211701	0.526276981986147	-0.171227018687820	0.739841869018052	-0.103523666666666	0.628485785524001
EVI2B	-0.181879317008013	0.526276981986147	-0.426395438579345	0.213459875394229	-0.206781333333333	0.307774837877941
LINC00487	-0.181904001596510	0.526248516028622	-0.114033474268824	0.844272085764738	-0.0467953333333326	0.84060196086617
KRTAP1-3	-0.181917769360974	0.526238275581629	0.130287571470343	0.812271254601992	-0.0699399999999999	0.780732284998362
IL36G	-0.181980298286651	0.52600273450314	0.0861538553011514	0.888071173454082	0.0155610000000003	0.967241125442187
ZNF267	-0.181981989492094	0.52600273450314	-0.424344259213388	0.216714808349232	0.222468333333334	0.462956926523536
ZNF174	-0.182046728916806	0.525834989854053	-0.170219216654297	0.74270446821332	0.0117890000000005	0.969387309763954
C1orf170	-0.182097435338680	0.525834989854053	0.175964912974764	0.731162861882915	-0.0385736666666659	0.873663396186202
ANKS1A	-0.182253558449033	0.525155433180681	-0.0555904696669682	0.933409139126562	-0.0136936666666660	0.965586337346777
ALG8	-0.182270557066412	0.525155433180681	-0.0538667866551117	0.935404504538153	0.0242480000000010	0.931859006463093
DOPEY2	-0.182280887653979	0.525155433180681	-0.328106130349735	0.407365872456796	0.0255176666666672	0.94871117680979
PLCD3	-0.182293546949643	0.525155433180681	-0.141530847127882	0.795705351419283	-0.0295796666666659	0.915761046438965
A1BG	-0.182304705181952	0.525155433180681	-0.163055495348524	0.756720201996006	0.00639199999999992	0.987134800094338
RIOK1	-0.182313607561638	0.525155433180681	-0.112941346385860	0.846627656290767	-0.155537666666664	0.447498161237612
AQP10	-0.182365058912029	0.525052530191233	0.167781419412044	0.748242818061289	-0.140611666666666	0.532545234597001
FCN2	-0.182367523052606	0.525052530191233	0.106417330153343	0.858284876027025	-0.215650666666667	0.575076472049916
SYCN	-0.182438405139100	0.524750915097822	-0.155925444998107	0.76972820876143	-0.180303999999999	0.532982474830836
LRRCC1	-0.182440030346954	0.524750915097822	-0.260412617162666	0.554030512113929	0.353784666666668	0.103455104221207
LDLRAD3	-0.182520319843648	0.52447120080529	-0.203331122373842	0.676646782959283	0.294823333333335	0.143974369985014
FLJ39739	-0.182600661943931	0.524410881411505	-0.164628432818385	0.754105947879839	0.0718273333333342	0.766563380016891
NMNAT1	-0.182657126703549	0.524205590574305	-0.472850820613642	0.145443641689931	0.129851666666667	0.536810332635776
GNG13	-0.182845462472010	0.523368741338241	-0.023063496935162	0.97536722461972	-0.0856813333333332	0.772139923817113
ZNF140	-0.183159797692782	0.522036740666353	-0.218676152178095	0.642148207033593	0.272466666666667	0.229591548351276

UBQLNL	-0.183255889055805	0.521671099091626	0.299848553337816	0.467194454586528	-0.316498666666666	0.313260450520079
SYNM	-0.183257103923573	0.521671099091626	-0.221811115382347	0.636874397609126	-0.0116089999999989	0.97236309164807
ANO10	-0.183696849129309	0.520122032823158	-0.100810374123810	0.866035166924059	0.137772333333334	0.527465067495661
LINC00334	-0.183762030021796	0.519910664165853	0.312421494090897	0.440033473850785	-0.0360829999999999	0.94678260229888
CTSW	-0.183835114115045	0.519538167357992	-0.186658960144017	0.71122781122807	-0.198918	0.542447274777
LDB3	-0.184046040671479	0.518398421774979	-0.0174432210790857	0.980603313490195	-0.177783333333334	0.483428472458056
C1orf101	-0.184067500919928	0.518341498050332	0.0881421646147368	0.885569889492087	0.0719563333333333	0.777406710539424
FLYWCH2	-0.184074378645398	0.518341498050332	0.191316595137437	0.701804778213811	0.00507466666666836	0.987937064305561
EIF5	-0.184092062456298	0.51833968715537	-0.233656737963014	0.610436189314218	0.00492100000000154	0.987937064305561
MCTP1	-0.184095991649621	0.51833968715537	-0.375142605574228	0.312620690092402	0.0220370000000003	0.933412982728621
MFSD6L	-0.184210384514124	0.518062036559546	0.184786097462882	0.716020884288485	-0.306728000000000	0.347281904527567
AGRP	-0.184228264711179	0.518027010264243	-0.0251861482993891	0.972358966414305	-0.0215216666666658	0.937041609399277
BAALC	-0.184233998358103	0.518027010264243	-0.0161798416907356	0.982057247916055	0.0390083333333342	0.891410328517388
WDR85	-0.184284650463770	0.517831147744369	-0.0347799937519454	0.959404955370931	0.0567886666666667	0.809505390888975
PRAMEF12	-0.184299921874470	0.517812053881374	0.0392175673388913	0.953905313456582	-0.00308833333333322	0.993677488355417
GLYATL1	-0.184303740054757	0.517812053881374	0.0290689054121651	0.96782691865619	-0.103992333333333	0.688516383064018
KPTN	-0.184394533538931	0.517457177649657	-0.0929341252425731	0.87756010254812	-0.00855466666666654	0.98025379113353
LY86	-0.184489265794299	0.516953691467903	-0.396309285610871	0.271136524502442	-0.0634879999999999	0.811401072147441
C18orf21	-0.184505927298871	0.516926143113104	0.0821361752584298	0.894840896036786	0.210720333333334	0.357739714466843
TOM1	-0.184547206515480	0.516862460766073	-0.0826315831374068	0.893753725133293	-0.0278660000000003	0.9123656811945
RAB9BP1	-0.184610099659981	0.516793597097072	-0.160635751961046	0.761367458467566	0.0354560000000001	0.91680951061779
TAS1R2	-0.184668170391975	0.516650620374677	0.11996861194569	0.835280975936648	-0.0882416666666664	0.738876306321397
KIF24	-0.184687945362876	0.516631178432684	0.0585609180649536	0.928586335857223	0.0423406666666674	0.91627892449973
SLC5A10	-0.184764856848496	0.516384669654958	0.0554623387890574	0.933409139126562	-0.158787333333333	0.580733005287472
LINC00304	-0.184971669248210	0.515495211727661	0.0504733329646071	0.94062893311096	-0.092253666666666	0.710004432586908
CPEB2	-0.184987107371261	0.515475225621241	-0.302187789930508	0.462229407853213	0.511213333333334	0.0260292526812800
SGMS1	-0.184996794073638	0.515475225621241	-0.40457122916842	0.255354561597911	0.0838730000000016	0.799898384121681
ALDH2	-0.185032406779762	0.515347471357885	-0.037971101868447	0.954889910016503	-0.054561999999999	0.830093119549048
FAM218A	-0.185074559519519	0.515238899370784	-0.0223431975952624	0.97536722461972	-0.0579390000000001	0.874353540431336
SMAP1	-0.185083955412677	0.515238899370784	-0.140692580851618	0.796714214272984	0.0904363333333349	0.679607902830893
MMRN1	-0.185100629475751	0.515228246641464	-0.120903135982172	0.832982209969764	0.0949543333333338	0.715017687269704
ADM	-0.185255532719751	0.514581659225769	-0.0321524377242915	0.96274336578329	-0.0914813333333325	0.762166238494042
ATG4A	-0.185297593197702	0.514399803674560	-0.427469365318606	0.212163576776299	-0.0255663333333326	0.926898701124672
MFN2	-0.185301048322036	0.514399803674560	-0.495318799716944	0.116831680799555	-0.0459086666666663	0.845891599383498
SAP30	-0.185305210653770	0.514399803674560	-0.208968326249070	0.665595909869988	0.0295586666666673	0.915663592470468
CYP4Z2P	-0.185496899132935	0.513579340052052	-0.107142545858209	0.857534099343316	0.0772523333333333	0.808511988397911
UGT2B17	-0.185513837335372	0.513579340052052	-0.28290578081446	0.502129505146473	-0.0386753333333330	0.90945379489823
SLC2A8	-0.185537287116607	0.513557798456639	-0.197041983221278	0.689989273027356	-0.0552840000000001	0.82704790320885
C21orf33	-0.185552420611911	0.513557798456639	-0.0689549921815083	0.912149839732916	-0.204343666666666	0.291201249796023
CGA	-0.185581677356448	0.513557798456639	-0.118035475366578	0.838500126709059	-0.124420666666666	0.589826330962094
AFM	-0.1855876394164	0.513557798456639	-0.107201771198296	0.85742860011897	-0.0898080000000002	0.774104133965233
TFF2	-0.185594285409758	0.513557798456639	0.216910167002242	0.647212711808862	-0.0304546666666674	0.914172701010275
YPEL5	-0.185602832475299	0.513557798456639	-0.276940371299852	0.515672755860042	0.232374000000000	0.248327829087472
IQSEC3	-0.185679385997926	0.513336257292002	-0.178324754779852	0.727335836219889	-0.111048499999999	0.690213956173797
BBS2	-0.185683260034048	0.513336257292002	0.119260909976079	0.836360753951164	-0.123722666666665	0.538968176654309
ATP2C2	-0.185686530104735	0.513336257292002	-0.111958464342214	0.847677166378871	-0.190854333333333	0.329569648675875
GSTZ1	-0.185696686063209	0.513336257292002	-0.00338751533343974	0.994730189431381	0.221493	0.264164257292671
YIPF6	-0.185713838722839	0.513336257292002	-0.589232472510747	0.0371136262345896	-0.0590493333333322	0.809409373518692
C14orf105	-0.18572428254404	0.513336257292002	-0.218648752681341	0.642209701165374	0.0406723333333339	0.868046126197316
ARHGEF5	-0.185810168064496	0.513133552655508	0.0590410350676283	0.927796078662766	-0.000951999999999937	0.997144862160692
FAM203A	-0.185814388545124	0.513133552655508	0.328228071418144	0.407325085101284	-0.103074666666666	0.644349193176966
TFCP2L1	-0.185821722715466	0.513133552655508	0.399578388901385	0.265001036100484	0.514139000000000	0.0222121644428973
ABCF2	-0.185903104414340	0.512864944993374	-0.282185209388301	0.504065779877366	-0.159683333333332	0.450203306181989
ADD2	-0.185925664397978	0.512801908622059	0.0389701062056678	0.954097938114723	-0.342317333333333	0.242351595840697
CD48	-0.185942106297841	0.51277605494595	-0.29496593137457	0.478655242507888	-0.105462666666667	0.671492528470281
GPX8	-0.185953860286712	0.51277605494595	-0.103858481282395	0.861570109612412	0.151508666666668	0.461494497197158
C5orf54	-0.185973898601188	0.512730998571322	-0.177461553155526	0.728529406441478	0.0794516666666679	0.748799811961987
ANKS4B	-0.186022790167838	0.512508004413709	-0.0189566108489303	0.97832242527852	-0.00487199999999996	0.993523650516004
RNF7	-0.186066488668831	0.512316606593126	0.113933352330078	0.844272085764738	0.0832430000000008	0.699953171808848
GMPR	-0.186187858695685	0.511727710296166	-0.0376896818678498	0.955365651737247	0.0996820000000014	0.70889268529697
FOLH1B	-0.186196403738286	0.511727710296166	-0.305358650103159	0.458013550008763	0.0555163333333337	0.80845521378406
ARMC5	-0.186415665872977	0.510641629477513	0.245970000851503	0.582912574644681	-0.059360666666666	0.861945621270498
LCTL	-0.186453994651216	0.510557028776537	-0.0570669924145256	0.930775493791569	-0.268463666666667	0.195247660560017
C11orf95	-0.186507156647012	0.510382553971261	-0.295145641656705	0.478475354656325	-0.138466333333333	0.604179762453377
SLC5A4	-0.18652912205436	0.510323302280436	0.0509935221167269	0.939574106942441	0.0538963333333335	0.812358226571296
ZNF439	-0.186539170058018	0.510323302280436	-0.406335645256912	0.254041953733213	0.07899	0.735011575254263
TCEANC	-0.186590711265131	0.510097825349868	-0.192776124248140	0.699860991842232	0.196811666666667	0.314161977530487
GORASP1	-0.186590966355888	0.510097825349868	-0.107720120558086	0.856450662791083	-0.2820530	0.178866129871872
RIPK2	-0.186631761926072	0.510040952152954	-0.383584334001778	0.293474457479736	0.212934666666668	0.383216446088131
ELMOD2	-0.186669849203612	0.510040952152954	-0.0130065208582899	0.98456132886228	0.234644666666668	0.245948767014956
UGT2A3	-0.186729903368649	0.509845514473723	-0.369044306240616	0.323543779375802	-0.0275079999999992	0.932533366180564
FBXO33	-0.186748578258414	0.509845514473723	-0.31002931151613	0.446837764966153	0.0945030000000008	0.789228734235839
LRRC39	-0.186787948073174	0.509845514473723	-0.0946898663646055	0.8739154222556	-0.0297083333333331	0.90945379489823
ANKRD54	-0.186793814330435	0.509845514473723	-0.00790781365476717	0.990405674923721	-0.0430146666666651	0.861677398924522
TIE1	-0.186803388589656	0.509845514473723	-0.156431385486376	0.768909201237015	-0.086631333333333	0.758616114209321
PADI2	-0.186823870797639	0.509845514473723	-0.174904154802599	0.733518674801325	0.233928666666668	0.259772013363267
FNDC4	-0.186827033590372	0.509845514473723	-0.0352283307760171	0.958873757674185	0.0286883333333337	0.925845244747948
KCNIP3	-0.186853362268663	0.509845514473723	-0.0231259945819867	0.97536722461972	0.104255666666668	0.666642292286073
KIAA1191	-0.186870873466656	0.509845514473723	-0.374740427133921	0.313141424748220	-0.000616999999999786	0.998281147912498
KRTAP8-1	-0.18689847343033	0.509845514473723	0.0876955783683465	0.886302037299497	0.0425503333333336	0.899614658619676

YPEL3	-0.186901153026388	0.509845514473723	-0.180577518384121	0.722518525037744	0.0339586666666671	0.914172701010275
OXTR	-0.187000855480776	0.509609216391632	-0.451036194023084	0.176663460992485	-0.126679000000000	0.527043331237303
TTTY7	-0.187104272918951	0.509130597665554	0.0554441471965553	0.933409139126562	0.158893666666667	0.411784621423647
TPRN	-0.187187102221837	0.508702927018347	-0.285591318933738	0.497161201540639	-0.0184669999999991	0.954021566744527
ADPRHL2	-0.187225554785592	0.508618052773001	-0.268924301038249	0.534079394978669	-0.059745999999999	0.789228734235839
LYPLA2	-0.187270373513112	0.508420686452793	-0.106556468431354	0.858072018696671	0.0369043333333347	0.885075170442055
LOC728175	-0.187313087638349	0.508236101459072	0.0670961533112315	0.91480694057025	-0.0252409999999996	0.93535961048518
PSTK	-0.187419634669599	0.507813204691204	0.0630490502732707	0.92160309476589	0.00472000000000074	0.987937064305561
S100A1	-0.187490470873063	0.507458679821234	-0.0597609796789147	0.926352922534113	-0.311486666666666	0.336419053562174
COL28A1	-0.187553567329820	0.507151085943081	-0.143563797886598	0.792614473650425	-0.184466666666667	0.4254947046787
TYRO3	-0.187586076450486	0.507028439022694	0.337540821575988	0.386297465201585	-0.0535926666666656	0.853219850381138
GCKR	-0.187662471757196	0.506788281941879	0.0212988422309200	0.975562088587469	0.0122083333333339	0.9681321732097
ROBO4	-0.187691416673306	0.506687223689098	-0.104033821493049	0.861570109612412	-0.0705829999999991	0.772520347452753
ZNF844	-0.187729077317534	0.50658337977313	-0.385800595603357	0.289470192870154	0.331655000000001	0.243251795903212
EPHX1	-0.187745773485221	0.506580322792359	0.276657506988592	0.51671109845278	-0.0450309999999999	0.867690146364162
S100G	-0.187771835820330	0.506496711539788	0.0953254733061983	0.872962271153999	-0.0333096666666665	0.899614658619676
WDR70	-0.187829785274534	0.506220510252593	-0.115896912425793	0.842733236705133	0.131695000000001	0.568659275949231
TBC1D24	-0.188000591080403	0.505337080231803	-0.292816509761636	0.482840748549717	-0.203917333333332	0.309395681555918
RPS6KB2	-0.188019306453005	0.505297892491219	0.203794547502112	0.676010626864207	0.0191513333333324	0.943976980215752
SOLH	-0.188092515324110	0.505077449879068	0.177737570394554	0.728170995367724	-0.0857263333333332	0.754561358073123
ELF3	-0.188110217079663	0.505044401717371	-0.0103493183854007	0.988029495198616	-0.203919999999999	0.379613779505161
KCNK10	-0.188139656725223	0.504965830712371	0.0916165500731121	0.879289305583857	-0.264528	0.283218330631050
GNB2	-0.188212413002248	0.504722984119448	-0.31310907230558	0.438459883259885	0.163802000000001	0.4261850174154
CTRL	-0.188244979655430	0.504674062180860	0.205434592316935	0.672911131891944	-0.113497333333333	0.585260484981764
ASPDH	-0.188255488856951	0.504674062180860	0.257534284179938	0.563116080558022	0.0997073333333334	0.67941752968872
DLST	-0.188337515658260	0.504411076311861	-0.389917490715151	0.282726287492993	-0.00540166666666598	0.987134800094338
ABCA9	-0.188368954687728	0.504295286474228	0.0561799401746802	0.932557112093148	0.144073333333334	0.572207354314478
WSB2	-0.188397517413860	0.504295286474228	0.0644204717576216	0.919813134013881	-0.555508333333331	0.0351167468854759
VTCN1	-0.188407849634902	0.504295286474228	-0.211302856959542	0.659974817413141	0.332667666666667	0.171804245444023
RHOQ	-0.188466389469639	0.504150443655039	-0.406237138211912	0.254041953733213	0.198559833333334	0.372749412776795
AGPAT6	-0.188497831799605	0.504034682442414	-0.0393311456945978	0.953905313456582	-0.164274666666666	0.399873592558586
CCDC26	-0.188526787276757	0.50393392526247	-0.0388167424568377	0.954263089081373	-0.167665333333333	0.417125112561133
PDCD10	-0.188572379884588	0.503732927624159	-0.232863396091104	0.611711680668619	0.0961809999999997	0.659124451108183
FBXW12	-0.188603188656733	0.503621040343638	0.0624008998070524	0.922722084234165	-0.0173779999999996	0.949376459031393
TRIP10	-0.188619847566838	0.503594413733284	0.166064580270683	0.751830402832405	-0.0379173333333317	0.880429228421837
SGCA	-0.188669754243358	0.503441240087487	0.132002233089296	0.810300414153187	-0.0145559999999994	0.963103527374278
PTPRM	-0.188690297320842	0.503391233422362	-0.120081358537966	0.835124910268666	0.330374666666668	0.0908960628407462
NKX2-3	-0.188783019624296	0.502906576511745	0.0439882283072219	0.948648166759756	0.0657026666666674	0.774104133965233
GDF15	-0.188867571127483	0.502766077319409	0.100875741280672	0.866005093242444	0.00607766666666774	0.985263856374185
SLC2A9	-0.188957441615656	0.502446260273499	0.0759425660871254	0.903510413986113	0.00945833333333351	0.980779858267938
ADH5	-0.189011429130838	0.502342490057537	-0.342976089913481	0.374882411235977	0.222343333333334	0.269565418326957
KIAA1737	-0.189033325218313	0.502342490057537	-0.132975346182647	0.809578511927685	-0.290755999999999	0.151097325334439
EPS8L1	-0.189033572787184	0.502342490057537	0.0519799764423683	0.938067483409541	0.0550210000000004	0.815156860161333
RDH12	-0.189052428211877	0.502342490057537	0.0115684885502353	0.986661750359244	0.127341000000001	0.714382809933119
TDRD6	-0.189064394618629	0.502342490057537	0.107077482116041	0.8576027597496	-0.00424899999999931	0.991625835822726
FAM71F1	-0.189103827666325	0.502302646454023	-0.156824336093505	0.768401006769299	0.306539333333334	0.184184161944404
RAD9B	-0.189218484987789	0.501833945947225	-0.0220428306022570	0.97536722461972	0.0525943333333343	0.914172701010275
CYB561D1	-0.189266514090918	0.501766272726072	-0.341835234956503	0.377158872804021	0.0319160000000011	0.914736964579324
TBC1D21	-0.189269565179658	0.501766272726072	0.0469911928045839	0.944081286115546	-0.0593013333333328	0.82700027112124
PPA2	-0.189371189657452	0.50143165996929	0.0520861747198242	0.938067483409541	0.077131333333334	0.728402452458775
DAOA	-0.18941175419475	0.501335223547507	0.187096054659697	0.709833349594307	-0.100691333333333	0.68271691249359
KIAA0947	-0.189477447929586	0.501059950524392	-0.484586578164745	0.133772472339013	-0.0267159999999994	0.941559580750404
NME5	-0.189504438483467	0.500999166373614	-0.118391868321819	0.837937752076601	-0.138324999999999	0.503217861354116
JOSD2	-0.189510468923518	0.500999166373614	0.0224100995235796	0.97536722461972	-0.0160659999999991	0.953723188802355
ZNF100	-0.189550897847877	0.500867365516304	-0.348794912180752	0.361261311685225	0.511545333333334	0.0214024833516911
TCTE1	-0.189602117935310	0.500633225766455	0.0641994513684262	0.919923865590619	0.0762173333333336	0.724384070314197
IK	-0.189652185317482	0.500406076585647	-0.416779518553208	0.234235464483992	-0.139122666666666	0.482760288609588
ARFRP1	-0.189691971938621	0.500288124422093	-0.156222701892598	0.768909201237015	-0.172242	0.390967882720418
AK2	-0.189696425244981	0.500288124422093	-0.108759873049684	0.853552381097052	-0.146661833333333	0.483089789079152
C1QB	-0.189743441763434	0.500152686319536	-0.460789896616478	0.161952078770073	-0.198306666666666	0.327821461809525
ZMYND8	-0.189803958614832	0.49986290295585	-0.127013353365458	0.820257254890606	-0.0302336666666657	0.912180255288873
TTLL7	-0.18985672124741	0.499693451271898	-0.264603880010837	0.54347643041286	0.321567000000000	0.158095716859654
IER3IP1	-0.189895130046766	0.499536531610972	0.0831721513229899	0.893287218356307	0.0670611666666683	0.760571541020441
DUSP16	-0.189911356655858	0.499512797993475	0.325566911895184	0.409206882509236	0.266552666666668	0.225005951354877
PRKCG	-0.189917421829559	0.499512797993475	0.204736747748666	0.673769742574452	-0.0376289999999999	0.899121707273884
MAP7	-0.189975312766508	0.499349928154591	-0.0826740615489523	0.893753725133293	0.159028333333335	0.535539620005815
ZDHHC18	-0.189976165329649	0.499349928154591	-0.139091930623636	0.79864602540281	0.317566000000000	0.131369372059974
RAB27A	-0.189988241114492	0.499349928154591	-0.425059585817396	0.215733128998344	0.300460666666667	0.162908301904803
PHRF1	-0.190057741543613	0.499223668881433	-0.0149990954218031	0.982921609770846	-0.110516333333333	0.61621014110361
THOC7	-0.190079744139167	0.499165318539034	0.00375629652349679	0.994588964700772	0.0350196666666677	0.890073383340154
GALNT1	-0.190089505876857	0.499165318539034	-0.081765729836955	0.89548747312664	0.291187666666667	0.210845715069665
USO1	-0.190109544397952	0.499133908861504	-0.468326663459321	0.151936428121882	-0.00706133333333262	0.98256318768706
STMN2	-0.190207820705393	0.498617863020497	-0.362631638934213	0.337143926897226	0.104104333333333	0.696594849734788
RAPGEF4	-0.190209440226483	0.498617863020497	-0.0450289240670515	0.94678213523172	0.131892666666667	0.525218637875881
NSMCE2	-0.190256809128206	0.498617863020497	0.0114003268524899	0.987215779667937	0.0882956666666674	0.7108031545527
TOPORS	-0.190270499283383	0.498617863020497	0.0357555858277899	0.958366440880488	0.0423430000000011	0.875624463432848
PSMA1	-0.190431979010112	0.498260466091655	-0.271572542553856	0.527984832801825	-0.00652166666666559	0.984931636076206
GJB4	-0.190455520219505	0.498260466091655	-0.0168158892024413	0.981160061237418	-0.0892263333333324	0.708080688946526
UNC13C	-0.190470248949104	0.498260466091655	-0.336163027245504	0.389476012645306	-0.163070333333333	0.4584541407185
TFE3	-0.190516668963270	0.498166231248934	-0.480268663517277	0.138098271295931	0.128014333333334	0.55248070124039

RARRES3	-0.190530152654785	0.498159155141885	-0.481482275078291	0.135560525489292	0.092243	0.73097308185803
C7orf33	-0.190535083066745	0.498159155141885	0.158717649261977	0.765791046741822	0.0551313333333338	0.84207712482186
CHRAC1	-0.190570916167011	0.498062242335872	0.172192640487786	0.738572368791165	0.0603286666666668	0.791599529156989
SSNA1	-0.190599065951899	0.498041039931056	0.130543902521328	0.812271254601992	0.0162533333333345	0.961665293425945
PMFBP1	-0.190605032791344	0.498041039931056	-0.338369658508427	0.384701467775014	-0.0366163333333327	0.916061376873503
LOC283693	-0.190651351633871	0.497919385973029	-0.333254444406433	0.396894500849691	0.145193000000001	0.474709570893085
SIGLEC15	-0.190794229339171	0.497239665340625	-0.065070779027223	0.918489100363712	0.0873336666666674	0.780619140960946
TMEM180	-0.190848528885406	0.496987980963709	-0.201045936838987	0.680143077995217	-0.0606816666666647	0.798344353938564
MANEAL	-0.190914377501826	0.496706730064261	-0.00142847293321423	0.998303187767855	0.00630933333333498	0.987937064305561
BCKDK	-0.190919841046314	0.496706730064261	-0.0927008395791177	0.877797216649453	0.0445643333333340	0.880347900939311
IMMT	-0.190941653858895	0.496706730064261	-0.0723506737071692	0.907565339533404	0.00186633333333538	0.99529569362957
PRR15	-0.191055471664071	0.496357866358074	-0.306799492378413	0.454431725456335	-0.394209666666666	0.0493233390534422
PLA2G7	-0.191070308405837	0.496357866358074	-0.284186814460227	0.500557068353025	0.528356	0.284197615593923
SKAP2	-0.191156415055831	0.496028772493681	-0.273068691469462	0.52462247978305	0.163516000000000	0.436636858796753
LCE3D	-0.191178160976626	0.495982294494015	-0.00664700918954307	0.991309497300929	-0.0427249999999998	0.926246672706072
PAGE4	-0.191193112457618	0.495982294494015	0.0111004488573407	0.98774001793194	0.0766653333333336	0.727065902741195
UNC5A	-0.191193214082501	0.495982294494015	-0.0253581587640266	0.972264538231849	-0.057277	0.85184175589894
SLC25A23	-0.191250082432438	0.495910569667287	0.111316281425007	0.849080210054875	-0.0953426666666664	0.688145991467449
F2	-0.191261237704633	0.495910569667287	0.140036930283108	0.7976681602188	-0.103441666666666	0.63979843704752
ZNF692	-0.191348880111421	0.495466651243027	0.0989290485448684	0.868042453226125	-0.0992529999999996	0.671124336259655
GLIS2	-0.191368081975318	0.495425489968521	-0.101091379875533	0.866005093242444	-0.242593666666666	0.195101950782130
NKAIN1	-0.191381942465012	0.49541630442645	-0.34568206178176	0.367005900580236	-0.130850666666667	0.578893615511221
BRWD1-IT2	-0.191423591959774	0.495388383144317	-0.0672836501253423	0.914700759157169	-0.0435376666666663	0.87290879338693
NAIF1	-0.191431685480582	0.495388383144317	-0.468408658546502	0.151936428121882	-0.0112499999999987	0.97161673663638
LAT	-0.191462973491803	0.495300276509354	-0.223558085805991	0.634267193037326	-0.0413993333333324	0.863479649214073
CD79B	-0.191464710715298	0.495300276509354	0.132210878938799	0.810300414153187	0.0156586666666672	0.969387309763954
CNIH4	-0.191506179087426	0.495263140193328	-0.257760320112411	0.562492892055072	0.0337383333333343	0.899614658619676
ABCC13	-0.191698490875998	0.494386904812511	-0.0590588487522573	0.927771716033454	0.0802186666666665	0.716268843468132
ZNF583	-0.191722835819704	0.494335932863974	-0.326978962068243	0.408851707589499	0.0740423333333343	0.797408129996359
TDRG1	-0.191737861121519	0.494335932863974	0.13695017517011	0.803386314119404	-0.121781666666666	0.729912257977231
PGM1	-0.191817155994078	0.494067052083392	-0.448315913287149	0.177868014516622	0.263933000000001	0.176338492557783
FOXN1	-0.191833959209300	0.494040365316885	-0.0161233802832357	0.982057247916055	-0.0822516666666663	0.780717071872908
GNG3	-0.191855974427387	0.494027353567239	0.120214901064167	0.835118069372686	-0.0181673333333330	0.97701753319801
TMEM14B	-0.191859006531007	0.494027353567239	-0.190916211758963	0.702500565899024	0.303341	0.132957584348716
UBA5	-0.191954534814918	0.493859573825982	0.144153561818424	0.791303782903891	0.00793900000000104	0.981582681132884
CBX7	-0.191955478117155	0.493859573825982	-0.247694229062331	0.579471329472972	-0.167279000000000	0.397798624983652
RP2	-0.192068838269335	0.493733119384798	-0.537323625037476	0.0725534260816134	0.377998000000001	0.0689724571534153
DIAPH2	-0.192080725031071	0.493733119384798	-0.165897989852945	0.751869806279813	-0.0692429999999991	0.814211123519969
AK1	-0.192189765275256	0.493242227168817	-0.108251445067667	0.85483409051824	-0.0212996666666656	0.942487401552645
KRT81	-0.192204900546964	0.493242227168817	0.169398301992038	0.74449897358286	-0.120747333333333	0.720336393581905
AKIRIN1	-0.192322178828117	0.492884897985742	-0.268427118622343	0.535217216332192	0.112062666666667	0.578893615511221
RNF139	-0.192333747600614	0.492884897985742	0.0683149048766298	0.912364243071055	0.054102000000001	0.815382785026166
PIN4	-0.192427417055431	0.49256091848193	-0.57184425946348	0.0443803345694495	-0.0464579999999999	0.863479649214073
STX7	-0.192532201422822	0.492268698139862	-0.599280323671232	0.0334696884408380	0.00746700000000134	0.98349947509937
ZNF550	-0.192564898432527	0.49218305461625	-0.0538511264784125	0.935404504538153	-0.0361369999999988	0.88479080768387
ATP6V0A1	-0.192604816062400	0.49204553667483	-0.517294394592008	0.091617200949096	0.0343003333333345	0.899614658619676
SDR42E1	-0.192633878384253	0.491992803021012	-0.254794622916272	0.566909791913503	0.0144213333333338	0.962334965334496
C10orf53	-0.192646934792275	0.491988728439896	-0.0652685367610626	0.918295693698803	-0.0260990000000001	0.920908349612552
ZDHHC8P1	-0.192753768646506	0.49149878281433	0.0896975558459354	0.88318195439136	-0.227117666666667	0.471989789810989
SSBP2	-0.192753780075055	0.49149878281433	-0.361076991027459	0.339627732901974	0.189861666666667	0.383110390635042
ARHGEF1	-0.192814592263532	0.49140044772686	0.0674716204542623	0.914318618000915	-0.162619333333332	0.410984651167943
PLAC8L1	-0.192829944150777	0.49140044772686	0.0187158727985395	0.978981596219275	-0.165839999999999	0.425882414194225
FOXRED1	-0.192900228410184	0.491141875491008	-0.316398380253799	0.432844809779835	0.0977043333333335	0.648325366843053
TRIM22	-0.192968075279883	0.490811051371234	-0.353800359886904	0.352212335098984	-0.144627000000000	0.479014333115247
LOXL3	-0.192983841759908	0.490790856016433	0.143517487561221	0.792614473650425	-0.156291666666667	0.518836392563847
FAM223A	-0.193028559897184	0.49059804431744	-0.0668843623480334	0.91517239775041	0.187610000000001	0.367620405250047
SLC17A6	-0.193058431175834	0.49054375891092	-0.00109083809328225	0.998512847681928	-0.00628266666666644	0.9879739922351
MTNR1B	-0.193059066065401	0.49054375891092	0.061087867092413	0.92470690387802	-0.0239529999999997	0.936634943217749
RETSAT	-0.193088415496876	0.49054375891092	0.201297692613131	0.680054545720343	-0.31133	0.353471671280201
NT5DC3	-0.193144706534355	0.490496294961125	0.146747657769433	0.786187758946602	-0.314814333333332	0.148610516089671
WNT7B	-0.193191973138467	0.490288355075062	0.0984633043266554	0.868308803285993	-0.220788333333333	0.470330562991677
STAT5A	-0.193210522787079	0.490259352870944	-0.258611418839808	0.559935260647793	-0.182624333333333	0.471989789810989
CYP11B1	-0.193234811782644	0.490259352870944	0.0756673580489293	0.903510413986113	-0.0794356666666654	0.762898679429892
PGLYRP4	-0.193261092903215	0.490245700313404	-0.0416321530025911	0.951989245458556	-0.206773000000000	0.351907021382432
ISOC1	-0.193335353468063	0.490024706997109	-0.290640837987869	0.487935421898319	0.0143000000000004	0.967241125442187
HAVCR1	-0.193359943142097	0.490006268701849	0.301141469826130	0.464564112537358	-0.0217959999999996	0.95831174299246
RGS18	-0.193403007675866	0.489917100557571	-0.384827384324055	0.291180853572672	0.134255666666666	0.516265433652982
GLO1	-0.193488111150114	0.489705585301327	0.174260680362882	0.734267769889068	0.192633333333335	0.343729765439488
DDX56	-0.193504034216039	0.489684630091927	-0.104272269111812	0.86124543980304	-0.133907666666666	0.523174709089214
BRSK2	-0.193559866229200	0.489425837866597	0.0835505132483248	0.892833228561937	0.0268310000000018	0.921128981205696
PTGDS	-0.193600120138651	0.489333856391425	-0.105440306777145	0.859800688625324	-0.0144306666666666	0.96900347496287
ABHD1	-0.193720892077496	0.488910133000194	0.187459327390648	0.709286896761914	-0.0532163333333329	0.839998150752089
PROK2	-0.193777691308256	0.488645815509843	-0.267236141194215	0.536453197697168	-0.0929229999999995	0.705543977023539
OSGIN1	-0.19381779665723	0.488628748800418	-0.117774289244058	0.838911179690148	-0.0201449999999997	0.95787549045386
NOL10	-0.193873238787543	0.488446496818671	-0.068177289204003	0.912364243071055	-0.049274333333332	0.833097445346985
NCK1	-0.193889637336815	0.488446496818671	-0.180947426770248	0.721866914445283	0.248918333333333	0.188082633937580
DEF8	-0.194011815901424	0.488065161341674	-0.224581824239545	0.632583866408459	-0.0330089999999991	0.896446774489185
ADAD1	-0.194028481206703	0.488039907249962	0.158823305744321	0.765721652938003	-0.119105333333333	0.615722818909839
COX6A2	-0.194162078629408	0.487392923609756	0.0159436085241926	0.982102356159698	-0.203844000000000	0.386293615344476
ARSG	-0.194209527619137	0.487184653529708	0.00181783865311897	0.99760061742039	-0.303191666666667	0.109227250003629

KCNQ5	-0.194239175090052	0.487082246228223	0.079772398660318	0.897892885174022	0.0156546666666671	0.963180491237413
TFRC	-0.194245930708292	0.487082246228223	-0.0443972090628536	0.948327980852394	-0.147021666666666	0.503400741258744
ERVW-1	-0.194272965460594	0.487047000839996	-0.133020007173304	0.809578511927685	0.00672666666666717	0.986290211615582
AKR1B10	-0.194299373476850	0.487019798984193	-0.155751760052297	0.769837060156515	-0.0424173333333329	0.889343227773276
NMT1	-0.194327526836345	0.48692630786469	-0.481950378494589	0.135560525489292	-0.266169333333332	0.160856802716546
CEL	-0.194345415502597	0.486893842434501	-0.177886236516382	0.728023343709833	-0.327157333333333	0.131549958663763
HMGCS1	-0.194446233337944	0.486368611640389	0.159166560391435	0.764932600126537	-0.193603333333333	0.363634739722717
HSPB7	-0.194471245650886	0.486293848768887	-0.07176484753818	0.90837606217316	0.0336236666666678	0.93461792157888
OR6B1	-0.194692469996616	0.48527573536476	0.00613049357904231	0.992286213306377	-0.134231000000000	0.587971705142625
HRASLS2	-0.194721327491082	0.485266929499885	0.123248000053057	0.82767952783856	0.0396710000000006	0.931446587444396
FTSJ1	-0.194770782396931	0.485179762641762	-0.280104173010174	0.508074118826263	-0.209878666666665	0.363290539080083
SURF1	-0.194850697393338	0.485000688942271	0.012620952001449	0.985220352735461	-0.0614136666666653	0.789917107854685
GALR1	-0.194875761833192	0.484925772671323	0.115935022885328	0.842733236705133	-0.00853966666666625	0.98370311636196
ZNF622	-0.194956136836379	0.484596618078428	-0.35276171023354	0.352560848501456	-0.0424616666666659	0.917142577725532
ST20	-0.195008188053287	0.484435481199243	-0.138348671354894	0.799981150504262	0.00361166666666789	0.995621063297892
LIM2	-0.195054636352675	0.484233881485061	0.228098214249897	0.623935503350068	-0.0157296666666666	0.97167131557531
SLC39A1	-0.195093369127135	0.484078048361283	-0.485806733397169	0.131934855037098	0.0493476666666663	0.828240868185377
ART5	-0.195118551871965	0.484002525277334	0.128395657290574	0.816345918941366	-0.117027333333333	0.604966458785844
DENND3	-0.195121871406075	0.484002525277334	-0.36699724905586	0.327869177739765	-0.148025333333333	0.500226891090192
CNFN	-0.195170429667397	0.483849099031502	0.0159610398010952	0.982102356159698	0.138518666666667	0.481229240510743
APIP	-0.195216632623376	0.483831787395704	0.0926899537705725	0.877797216649453	-0.0627976666666656	0.789488464226224
MS4A2	-0.19525664040994	0.483700350444178	0.0476439236940201	0.943611925889785	0.119678333333334	0.600950363504414
FGF3	-0.195273586830002	0.483673682779917	0.249412182729132	0.575393431980598	-0.0286556666666662	0.933248182961486
KATNAL2	-0.195343949935788	0.483404340629282	-0.229391890158214	0.620183562399927	0.104886333333334	0.73481029572636
TMEM231	-0.195363864943574	0.483360111061253	-0.497890182608927	0.114179995542754	-0.0290856666666657	0.90945379489823
KPNA5	-0.195424386031306	0.483123891693579	-0.133752693745720	0.808879359838378	0.168162666666667	0.449828629541335
RND1	-0.195453989566413	0.483123891693579	-0.137896174680934	0.801725362274543	0.0158110000000011	0.97057157918193
C1orf105	-0.195507576683618	0.483025199696385	-0.169498444785176	0.743971575601932	-0.0596849999999995	0.812232870389155
KCNS2	-0.195521873335928	0.48301429439611	0.112249129706704	0.847454473507881	-0.081971666666666	0.709937184587543
KLK3	-0.195589570497428	0.482687257930644	0.242684592477673	0.592765380565798	0.143805333333334	0.498454352317107
BMP8B	-0.19569839343434	0.48226441983316	0.0735993873820383	0.905838142287595	-0.188382	0.498454352317107
CYLC2	-0.195780169683758	0.482001750885581	-0.0431088872249787	0.950146638225747	0.119765000000000	0.592269447222472
CLCA2	-0.195841942084315	0.481710141770467	-0.0354699585216285	0.958689318480951	0.123434666666667	0.595152359048539
ALK	-0.195926867074614	0.481502702291456	0.0898181499782561	0.883136531143569	0.180676333333334	0.340052241399374
ALDH3B2	-0.195932987023458	0.481502702291456	0.173957509527645	0.734793595952544	0.216230333333334	0.270098077393495
PLAG1	-0.195939215304551	0.481502702291456	-0.332885288247210	0.397658808533403	0.0695086666666672	0.777406710539424
TAS2R1	-0.19594364658794	0.481502702291456	0.079626196314968	0.898031113962014	0.066872333333334	0.776805598353461
PIK3IP1	-0.196039578064981	0.48135221492648	-0.450401995304397	0.176663460992485	-0.0287183333333332	0.941640072637564
TTC21A	-0.196080795333562	0.481182281423441	0.110454789250951	0.849673914564946	-0.0133346666666663	0.963180491237413
TLN2	-0.196092309200865	0.481182281423441	-0.186281971050388	0.712187471324402	-0.144283666666666	0.501606759648947
CD244	-0.196126715404212	0.481105804958761	-0.0423372260861956	0.951523595489103	0.0231983333333337	0.95285450244329
PPM1B	-0.196156039485263	0.481105804958761	-0.232073917807874	0.613186703369632	0.0293856666666667	0.921287736444926
RAP1GAP	-0.196283171160268	0.480797242664364	0.145739386503644	0.787804113309913	-0.134805333333333	0.522480635253951
CLDND2	-0.196330138129724	0.480593459597003	-0.00931762392603797	0.98909166696601	-0.0653340000000001	0.83616423402744
ZRSR2	-0.196352669902721	0.480534080525703	-0.167497354072961	0.748960978799932	0.0726076666666678	0.735063355984464
NTNG1	-0.196403424688360	0.480510710663098	-0.0755953440912808	0.903517361619051	0.0439270000000005	0.90778598212344
TRIM25	-0.196450753052102	0.480323409317405	-0.230009359643659	0.619254341957051	-0.273453666666666	0.210845715069665
MEIG1	-0.196452367293136	0.480323409317405	-0.246751604257134	0.581387364514747	0.115781333333334	0.616036866881905
GAD2	-0.196455932896030	0.480323409317405	-0.0268161769673285	0.970207612777188	-0.176977333333333	0.368449159208846
POLR3E	-0.196483464126123	0.480323409317405	-0.0237994113234692	0.974814869598688	-0.208311333333333	0.279049509382533
SLC4A2	-0.196521435740007	0.480323409317405	-0.190361404080187	0.704208582824765	-0.0083670000000001	0.979527173532206
AGA	-0.196574908224838	0.480106511212609	-0.668621796211356	0.00934833222290552	0.721608333333335	0.0105638761565426
NPC1	-0.196630158712727	0.479853974612947	0.081294048586954	0.896346444707454	-0.0274003333333325	0.928897428735123
SERPINB5	-0.196667670194292	0.479807820116055	-0.298496378862038	0.471239854755259	0.182738666666667	0.557611184484231
MKRN1	-0.196675806085751	0.479807820116055	-0.386798699947654	0.288207962791151	-0.229505666666666	0.234508116088724
GALR2	-0.196728284469799	0.479717347564433	0.234801864113859	0.607957283325678	-0.153239999999999	0.596725125925172
SVEP1	-0.196854249888158	0.479047551028439	-0.145856067134783	0.787755441629161	0.319649	0.443378989641463
C1orf21	-0.196999619610968	0.478264000937494	-0.0168805214911057	0.981160061237418	0.338491666666668	0.120319574336004
PPID	-0.197029159660156	0.478237188217809	-0.528565806843214	0.0806737922798841	0.0594993333333333	0.82700027112124
GNE	-0.197067903549411	0.478227730852761	-0.161084677124233	0.760354001455808	0.327264666666668	0.109922018659511
PTPLAD2	-0.197138283008191	0.477962392343193	-0.309220405139357	0.448350131365446	-0.067322333333333	0.842903889380894
NFYB	-0.197168828553397	0.477856070232083	-0.343487505635857	0.373801771219189	0.0337120000000001	0.902957732816259
BAAT	-0.197177161398456	0.477856070232083	-0.035015234087111	0.959140268168928	0.123898666666667	0.578368087150555
CHRNB1	-0.197198423565672	0.477829034700983	-0.0869972990231362	0.887055939766881	0.0245640000000008	0.93724165573711
PITPNM3	-0.197213908981471	0.477811474240075	-0.171485490146241	0.739541495555634	0.184719333333334	0.452735492315141
RAB18	-0.197298342254433	0.477535081302344	0.036108190810666	0.958023954102604	0.0933133333333335	0.67574215232331
CHRNB2	-0.197299388498168	0.477535081302344	0.239072946223271	0.599801655225887	-0.169310333333332	0.569252389178917
ATP5J	-0.197353818863141	0.477429355795668	0.028718830120688	0.96782691865619	-0.0536150000000004	0.853077816975915
CDKN1A	-0.197371768644946	0.477429355795668	0.147272600294866	0.78561060123541	-0.445441999999999	0.150274389990121
CDC23	-0.197506760050883	0.47687245681861	-0.210478754523547	0.661670522548494	-0.0319386666666665	0.898979600726666
CEP85	-0.197512149317788	0.47687245681861	-0.260644532687432	0.553540075799904	-0.0655673333333322	0.857953692324335
RBP7	-0.197519352500534	0.47687245681861	-0.105728221026387	0.85956218633532	0.105786000000000	0.601564043046757
ACCS	-0.197534274825008	0.47687245681861	-0.201632630518677	0.680054545720343	0.0787740000000012	0.74164843446719
C9orf89	-0.197560192365794	0.47687245681861	-0.0289526733953256	0.96782691865619	-0.154973999999998	0.504775542398532
CCDC109B	-0.197564276743031	0.47687245681861	-0.320859012566693	0.421618886526906	0.123678000000001	0.637596150841031
ARPC1B	-0.197604804481797	0.47687245681861	-0.247945768304432	0.579125113383471	-0.0476769999999987	0.85994094150432
DAD1	-0.197634262953958	0.476809857936961	-0.07264581550674	0.907539622340586	0.0320133333333354	0.89936551840597
NDUFS5	-0.197685081239264	0.47665814615908	-0.244792871184333	0.587430330647728	-0.00241666666666647	0.993801264598204
INSL4	-0.19772565442392	0.47649303188639	0.150894612734884	0.778253782413218	0.0890663333333337	0.771396688597852
EVI5	-0.19773405683363	0.47649303188639	-0.513652222460094	0.0962522730025878	0.303995000000000	0.156312939631849

GPBAR1	-0.197798277557208	0.476360566098201	0.0232076785686564	0.975348968561756	0.0131366666666676	0.971293288800343
KCNC3	-0.197805077463241	0.476360566098201	-0.103308218745736	0.862065338914705	-0.0668969999999991	0.784903505054688
UNC119	-0.197845530866087	0.476303756647883	0.233579323910511	0.610436189314218	-0.100100666666665	0.63835301231481
HMGCLL1	-0.197949088485115	0.476063212021981	0.0474133020610627	0.943981149721771	-0.176012	0.353471671280201
ARV1	-0.198025300165899	0.475836127713227	-0.286935442158075	0.494604746861742	-0.0891236666666663	0.706429250016819
HCRTR1	-0.198040105168188	0.475822824838838	0.188961014658751	0.706965444256208	-0.0210370000000004	0.937717709824356
DNASE2	-0.198064941828147	0.475822824838838	-0.377902466907665	0.305766089617986	-0.131739333333332	0.512796060754107
APBB2	-0.198175693076120	0.47539417795200	-0.595263774486818	0.0344667539792754	0.278159000000001	0.180114381586694
BRI3BP	-0.198202311564935	0.475311419495428	-0.31626730370072	0.433130552389881	0.0972126666666678	0.706290370786298
CHMP2B	-0.198221582691745	0.475271887531312	-0.46667640977569	0.152560208324424	0.0198296666666671	0.94622978991441
COPZ1	-0.1982230653763	0.475271887531312	-0.348520844151225	0.361311804770494	0.102466666666668	0.633042734875639
RBFOX1	-0.198242883475745	0.475271887531312	0.0178262894892931	0.980042218206248	-0.0251263333333325	0.94245778509664
HSD17B6	-0.198366297443567	0.474789972832566	0.0372490813930995	0.956162116576064	0.175462333333333	0.465279557781548
FOXA3	-0.198397269050578	0.474686144087388	0.145388049436766	0.787959014672532	-0.00995266666666533	0.980779858267938
P4HA2	-0.198452701222475	0.474503487491678	0.0596622309688318	0.926534310457165	0.0068980000000011	0.985544323496696
FGF22	-0.198471858617153	0.474464694991381	0.144542290044414	0.790283845502267	-0.176034333333333	0.676662919155189
CCK	-0.198587768400210	0.474005021550358	-0.0221477415846128	0.97536722461972	0.0228893333333336	0.955460918122607
B3GAT2	-0.198664227805743	0.473703502155349	-0.0564048547754343	0.931937690249408	-0.0367023333333331	0.904201124848034
HERPUD2	-0.198719666300135	0.47346633197412	-0.213978592121968	0.65332314935922	0.134567333333334	0.513141966218084
STAT5B	-0.198757036810696	0.473379856425847	-0.511213671604883	0.0987450924554118	0.324578000000000	0.0932203439363738
RPAP3	-0.198768650058016	0.473379856425847	-0.272748212619585	0.525510448868965	0.0175276666666684	0.955701951169976
FLJ37035	-0.198781458873995	0.473379856425847	0.083014091997794	0.893446201797764	0.0357686666666674	0.907385309385626
MPL	-0.198882919703547	0.473083464911757	-0.086279828703704	0.88794718020464	-0.102973333333333	0.648548661313887
SPTSSA	-0.198902386737039	0.473042974273171	0.109599476780613	0.851509079273215	-0.814095666666665	0.00986190328472675
TMEM68	-0.198913942551696	0.473042974273171	0.0667538671100809	0.915438681913279	0.1293575	0.562674320626673
PAPD5	-0.198996666920139	0.472711040345555	-0.126543274809348	0.821473247266105	0.109125000000000	0.657726934705567
HARBI1	-0.199010682679344	0.472702560224251	-0.286447870873013	0.495728580458409	0.0717336666666668	0.765276224002577
ATP6V1C1	-0.199105872198464	0.472365461182511	-0.305960960508870	0.457192231066199	0.169574999999999	0.411878887058133
ZNF274	-0.199143210688883	0.472220245351223	-0.244607623976143	0.587653287154633	0.141411333333334	0.48712543076516
PPP1R36	-0.199193501234069	0.471999136368711	-0.0112118536559717	0.987500171360631	0.0768343333333338	0.758250508013307
TAF13	-0.199280051415238	0.471575858329526	-0.500185980934336	0.113333037486310	0.0623803333333348	0.799725367501923
DCAF12L1	-0.199300569093472	0.471575858329526	-0.125026328730430	0.824457843812434	-0.157853999999999	0.651165924999259
GC	-0.199371930321503	0.471469403017844	-0.163686199418922	0.754968203901817	0.110146333333334	0.623988109816431
FRMPD4	-0.199402661115629	0.471436764116854	-0.078665005791949	0.899281198071059	-0.0469929999999993	0.839703258597695
GRIPAP1	-0.199404230801973	0.471436764116854	-0.363630468622662	0.335343372678869	0.0255466666666678	0.924674647382585
TRAPPC3	-0.199568315844431	0.470835040972832	-0.50580733421884	0.105941223458825	-0.176439666666665	0.477169587518779
HR	-0.199605120643986	0.470693250301018	0.250276661221462	0.574107101982055	-0.0633639999999994	0.824044031896077
PAPOLA	-0.199620427651568	0.470677314684511	-0.0253688864871248	0.972264538231849	-0.0910566666666657	0.68171195530621
GAMT	-0.19962242479801	0.470677314684511	0.205754368608219	0.672625944768829	0.172258000000002	0.368449159208846
MGC24103	-0.199651392685044	0.470643410459078	0.074510862938858	0.904832407812985	-0.0934109999999992	0.759034160486144
ANXA3	-0.199747564402984	0.470449862430044	-0.276424907212068	0.517367360081815	0.166661000000000	0.549557793610283
SRR	-0.199792530217040	0.470335936599847	-0.273907797265912	0.522049608558573	0.468943000000002	0.048170505761132
SERPINA6	-0.199855567018359	0.470185452010123	0.118239885397435	0.838218827550598	0.0109433333333334	0.972353176085335
DDX51	-0.199907119202974	0.469957516086636	-0.0333517064140966	0.960784250392482	-0.091961999999999	0.659130116239877
CXCL10	-0.199981139379196	0.469623805463508	-0.462476848606591	0.159013702598371	0.0529460000000002	0.819449110075885
AKR1C4	-0.20008867071431	0.469338166860963	-0.203530094451222	0.676207233014711	0.141234000000000	0.474413951968734
MUM1L1	-0.200116443995055	0.469322857318905	-0.00114715926286446	0.998383694263455	0.232770333333335	0.211495399841293
TIRAP	-0.200163562040865	0.46912108770892	-0.299647344213456	0.467649102112712	-0.000812666666666571	0.997509578474685
MED10	-0.200173186894459	0.46912108770892	-0.293433581489154	0.480778950366873	-0.0176633333333321	0.95285450244329
TAF1	-0.200177188090690	0.46912108770892	-0.35427995336256	0.350901200328296	-0.158446999999999	0.516265433652982
COQ4	-0.200183960718156	0.46912108770892	0.131281188229457	0.811539578433911	0.124991000000001	0.552298643114782
DPYSL3	-0.200188362193939	0.46912108770892	-0.303781992619947	0.460052080110635	-0.216818333333333	0.287956132210494
SH2D2A	-0.200234797055427	0.46912108770892	-0.0458283288323584	0.945372627844176	-0.184254000000000	0.461494497197158
GABARAPL2	-0.200295432155032	0.468866191873438	-0.295100632216406	0.478475354656325	0.235992333333335	0.313220411537404
LCN6	-0.200386072636200	0.468410271889375	0.221227362007862	0.638214394314926	0.0693990000000002	0.801761479877858
RASAL2	-0.200428915529670	0.468233704965737	-0.241078780450721	0.596648444172109	0.274443000000001	0.142821378450256
LSS	-0.200440246070836	0.468233704965737	0.225000492271567	0.631198241677861	-0.161198	0.575526309068521
DHH	-0.200445475844253	0.468233704965737	0.0642195546548098	0.919923865590619	-0.0933723333333332	0.691482997769299
REG4	-0.200459326128757	0.468233704965737	0.096968136293847	0.870463314702701	-0.0217646666666665	0.938316557959043
SPATA9	-0.200472072817459	0.468233704965737	-0.0294316156789597	0.96745667731626	-0.00549799999999956	0.987937064305561
C6orf10	-0.200495696135852	0.468212047491129	-0.0692224258169018	0.911837389834658	0.0863576666666676	0.719839702715467
CXCR2	-0.200505133224479	0.468212047491129	-0.288990123362097	0.489280593381909	-0.0837323333333330	0.7996066102903
PSMG2	-0.200570654497925	0.468069083069457	-0.00158893459082472	0.998066886004786	0.103833000000001	0.645039931542325
ZNF706	-0.200601369424508	0.467963421543927	-0.211248301095012	0.660013379937732	-0.0169086666666658	0.955460918122607
KIAA0825	-0.200614055708722	0.467963057507189	0.0502283226801049	0.941133149988298	0.358993000000001	0.239584169389072
DCD	-0.200661150191011	0.467953655368110	-0.0404279136289246	0.952720585102517	-0.0292810000000001	0.928295508521289
CHST4	-0.20075101221289	0.467550152449231	-0.325881405150326	0.409206882509236	-0.111781666666667	0.636490807097692
PDILT	-0.200755798949752	0.467550152449231	0.0135918292668451	0.98412883278237	-0.120486333333333	0.59584352007183
MIDN	-0.200828215378226	0.467523805117928	-0.264247003878072	0.544417334045275	0.192923000000001	0.340052241399374
TAS2R38	-0.200862559718066	0.467397067224662	-0.154169724684679	0.77207130150678	-0.140512000000000	0.514420666507349
PSMD14	-0.200925890470024	0.467269938707	-0.146993137523905	0.785782367062857	-0.0648933333333329	0.774104133965233
MINA	-0.200953974066807	0.467232367685339	-0.345141156318125	0.368530625231133	-0.568659333333332	0.0351167468854759
TMX1	-0.201071135364714	0.466696202673607	-0.0879023901522097	0.885848000380851	0.0783733333333346	0.724603790871296
C1orf109	-0.201107607377798	0.466557202430018	-0.214985154914174	0.651257878577277	-0.119992666666667	0.618162576854777
PCMT1	-0.201116583674717	0.466557202430018	-0.0375418810914624	0.955365651737247	-0.123351333333333	0.636468072096347
MSMB	-0.201157289822414	0.466414924124033	0.215611367053283	0.649402346163219	0.0547433333333338	0.827433678445379
TMEM19	-0.201171530551155	0.466405619443364	-0.0896218772357186	0.883196389963269	0.247881333333334	0.212445240967000
TECTB	-0.201256542866003	0.466057407426340	-0.0542798583896495	0.93470434607584	0.138469000000001	0.49971129098408
SERPING1	-0.201299211687281	0.465926802776661	-0.263643297330358	0.545374660547732	0.165671333333334	0.528060753831246
CMTM8	-0.201362557861727	0.46566066901954	-0.0432744564927634	0.94998904453514	0.369739666666667	0.144613416019330

IGFALS	-0.201401846746764	0.465534680801173	0.135102168301663	0.807470535721699	-0.162779999999999	0.549664615421682
RASL10A	-0.201464355451305	0.465362386696958	-0.215806526406071	0.648943337948563	-0.111582333333334	0.774104133965233
GPR45	-0.201471779083291	0.465362386696958	0.170611236363087	0.741623909905464	-0.239829666666667	0.202884869911086
MDH1B	-0.201547219271662	0.465027167311134	-0.204444258015173	0.674817810302676	0.118205666666667	0.552298643114782
GLRX5	-0.201579110461274	0.464915153980298	-0.185193144020482	0.715110052196475	-0.0813276666666663	0.704370299831112
GXYLT1	-0.201584469044126	0.464915153980298	-0.117705439361991	0.839234361388341	0.475468666666668	0.0469041914734957
SPATA12	-0.201586101040520	0.464915153980298	-0.0498293743154698	0.941458038227918	-0.161778333333333	0.442294185443201
MRPL4	-0.201623837723475	0.4648757676403	-0.0218805337818304	0.97536722461972	-0.125523666666666	0.544685122345675
ZNF554	-0.201647731866823	0.464810343023994	-0.0964631667233893	0.87126567794459	-0.0593229999999996	0.794631152235972
TMEM55B	-0.201651420887538	0.464810343023994	-0.0184498096211898	0.97902968742576	0.00674366666666747	0.984700985286246
SEL1L	-0.201672253502403	0.464810343023994	-0.471741363744207	0.146991015246019	-0.196065333333332	0.403629729651335
SYNGR4	-0.201729580620307	0.464554952068802	0.157033422311323	0.768401006769299	-0.095489999999999	0.781677540593488
FLJ11235	-0.201730120452425	0.464554952068802	-0.18165944218403	0.720537767137049	-0.0772279999999995	0.81827440735546
LIPG	-0.201767796463945	0.464492364108428	0.137462268111282	0.802039543563725	0.0893640000000004	0.717087875531756
TMEM203	-0.201767891136538	0.464492364108428	-0.347817924603295	0.362101028295762	0.0202648333333355	0.945904449997854
IVD	-0.201800204414273	0.464492364108428	-0.159654215038579	0.763360730475393	-0.413640333333332	0.0649438674882112
RNFT1	-0.201943306661024	0.463827176972634	0.0498957033501471	0.941458038227918	0.607496333333334	0.0756081546313796
PPP2CA	-0.201961572524507	0.463794603504122	-0.156454198944356	0.768909201237015	0.0736333333333342	0.734997389745078
LIPC	-0.201970478919048	0.463794603504122	-0.381016077692074	0.298110142491852	0.0323960000000007	0.915761046438965
OR5H1	-0.201988374353334	0.463794603504122	-0.266988425062604	0.537283176095935	0.0465423333333337	0.856854911614454
AMELX	-0.201994749467177	0.463794603504122	-0.247754840548890	0.579471329472972	-0.0357199999999995	0.907374738017166
NTN1	-0.202089543983879	0.463713594189307	0.00578791261379927	0.99251028338798	-0.0360846666666656	0.890094615232462
MOCOS	-0.202198144376223	0.463524474278789	0.199489712512962	0.683755152819083	0.0284466666666670	0.920540786765438
IL20RA	-0.202202749944652	0.463524474278789	-0.00083079233838267	0.998745415362821	0.098302000000001	0.690285432180092
DDX54	-0.202229092936094	0.463506877978689	0.0713519924334399	0.909119908757507	-0.148361499999998	0.48712543076516
DAXX	-0.202233182092145	0.463506877978689	-0.269384481246691	0.532580848023115	0.0603512222222236	0.804470652326676
PHKA1	-0.202271903911656	0.463506877978689	-0.220557733128032	0.639162466991523	-0.113682000000000	0.624647715880144
UGT2B15	-0.202314346669396	0.463506877978689	-0.168097111367876	0.748024524669236	0.359045333333334	0.110806424945014
PMS2P5	-0.202347606591682	0.463430839944666	-0.0980797078912106	0.868852648210739	0.106170000000002	0.60378773986659
FTSJ2	-0.202378600885618	0.46336077480947	-0.130603989904726	0.812271254601992	-0.0777176666666664	0.719304894613107
GAB4	-0.20239809945495	0.46336077480947	0.0526492201179118	0.937357975161644	-0.156709999999999	0.629657237462109
VILL	-0.202398311223481	0.46336077480947	-0.260748284335322	0.553492035976081	-0.120149333333333	0.577524803794493
KCNH4	-0.202404773279865	0.46336077480947	0.12341554947229	0.827264208621148	-0.145052999999999	0.459199400888618
TEFM	-0.202484304600355	0.463263243213915	0.238742840970226	0.600797772196361	-0.0270563333333333	0.92590541082869
ANAPC13	-0.202486457077591	0.463263243213915	-0.122237961008987	0.829569971668386	-0.0526449999999990	0.830320848383177
AGPAT4-IT1	-0.202502548006741	0.463263243213915	0.28438510423842	0.500419415740203	0.0528946666666672	0.824044031896077
CEACAM5	-0.202504127050701	0.463263243213915	0.107186847796509	0.857435906976285	-0.073173666666666	0.774553415918773
LTV1	-0.202607611093341	0.462915649261208	0.234325836270867	0.608803849126507	-0.0205863333333324	0.949295980201336
DEFA4	-0.202668248090991	0.462636921212931	0.116292479791275	0.84217383062547	-0.0215393333333328	0.947833660927513
SLC35D3	-0.202680538761547	0.462636921212931	0.067175301515698	0.914700759157169	-0.182989000000000	0.455317063332253
C1orf122	-0.202810789477728	0.46217788409071	-0.102736489686249	0.86257932725575	-0.0968959999999998	0.665071722126052
CLPB	-0.202832064248631	0.462128832741706	-0.237422524162546	0.603142755466432	-0.250181999999999	0.203889537242039
NEGR1	-0.202868060624107	0.462128832741706	-0.184202664913647	0.717436396325728	0.0881493333333336	0.714961810216865
GPBP1	-0.202927732967342	0.462015828833737	-0.249190603771427	0.576149573608393	0.118099666666667	0.574486578500537
ZNF354B	-0.202964410002775	0.462015828833737	-0.349548342752311	0.359232392104395	0.0386756666666676	0.874243670972648
PUS7L	-0.203040131971515	0.461732512859725	-0.328804191194122	0.406249092981339	-0.238583999999999	0.309421300309741
PAMR1	-0.203059737649199	0.461692577347343	-0.0911739604883191	0.880644955971727	-0.0336956666666663	0.930383110415834
C16orf70	-0.203081415197025	0.461692577347343	-0.0423083370492849	0.95160244805127	0.147890000000001	0.542096490709595
ATP5G3	-0.203113381083838	0.461676914165108	0.104376650706706	0.861121906453898	0.041627333333334	0.882040270326032
IFITM1	-0.203117179718168	0.461676914165108	-0.318352137649411	0.429291355866025	0.228566666666668	0.225162645598518
MMAA	-0.203162584493831	0.461608395672996	-0.455928238755247	0.167648587973919	-0.197887666666666	0.337435643925038
EXOC3L1	-0.203270376538717	0.461208918687481	-0.0231689800349554	0.97535657852997	-0.0733929999999998	0.738876306321397
TTC13	-0.203316547882883	0.461014759180263	-0.561056144006038	0.05252006717346	-0.190793333333333	0.338184384427605
SMYD5	-0.203336705882367	0.461007435085684	0.0354931829638326	0.958689318480951	-0.0657816666666653	0.777568084061469
SPRYD7	-0.203350705228541	0.461007435085684	-0.0952403778129868	0.872962271153999	0.120476000000002	0.578893615511221
DBT	-0.203383122087612	0.46099832366188	-0.401235413521730	0.262072933179063	-0.0807303333333324	0.797534995180308
BTF3L4	-0.203433518425781	0.460779682869612	-0.161994397987783	0.758555563769973	0.0723685000000004	0.772379251169911
THSD7A	-0.203468893204499	0.460662833375828	0.0173581181023437	0.980670285500112	0.253875333333334	0.267017625061681
BTN3A3	-0.203481737264677	0.460662833375828	-0.396654379438757	0.270862229167822	0.255429666666667	0.177614563220760
SLCO2B1	-0.203503330631413	0.460662833375828	-0.491891697750535	0.122707160872791	-0.0875620000000002	0.6846855637534
ITPA	-0.203636261510104	0.460269158082508	-0.227138306708536	0.62581495163125	0.00334400000000137	0.991625835822726
GRIA3	-0.203813490750876	0.459611066432177	-0.201191395803502	0.680054545720343	0.514518666666667	0.0711528556656884
C10orf35	-0.203910077022965	0.45927303662344	0.101752027770566	0.864906409423152	-0.226797333333333	0.381000610926241
GGA2	-0.203963726489357	0.459036038196187	0.157513417584096	0.767770230680061	0.0763750000000008	0.733679380704515
BFSP1	-0.204026665971552	0.458819258761801	-0.271301146848117	0.527984832801825	0.279507000000000	0.198504722214288
PPP1R1A	-0.204126462639327	0.458463016608099	-0.0736391727224517	0.905838142287595	-0.0499606666666661	0.852608879235174
CCDC58	-0.204131780259731	0.458463016608099	0.087959990076561	0.885848000380851	0.0437560000000007	0.915761046438965
HAX1	-0.20413897787985	0.458463016608099	-0.127117529445298	0.820137243446168	0.263182000000002	0.172870488227521
KLF4	-0.204152476072901	0.458463016608099	-0.352319975353967	0.352921806679009	0.377236000000002	0.110474730891376
MCTS1	-0.204197441054295	0.458347798649049	-0.464470556947727	0.156705484651498	0.0621650000000011	0.855240892932328
DGUOK	-0.204218911811359	0.458297431278457	-0.156356736437732	0.768909201237015	-0.081305999999999	0.73097308185803
TCOF1	-0.204325630155851	0.457975398937914	0.0224072403169323	0.97536722461972	-0.121957999999999	0.578893615511221
IDO2	-0.204362164672588	0.457847695225033	-0.280029339014404	0.508074118826263	-0.269841333333333	0.273327127062166
DICER1-AS1	-0.204369997204738	0.457847695225033	-0.0956297686108053	0.8726818955265	0.353916000000001	0.143931155913550
ADCY1	-0.204402753084665	0.457847695225033	-0.0872391182224574	0.887055939766881	0.0590060000000013	0.807258720089412
SYNDIG1L	-0.204432031600858	0.457803402040890	-0.0294873248282298	0.967314962305573	-0.214230666666666	0.344866669191371
TMUB1	-0.204440609719212	0.457803402040890	-0.0950932282867424	0.872962271153999	0.0804226666666667	0.710449166505851
MRPL16	-0.204527140509210	0.457400909509857	-0.294881544747363	0.478695647537866	0.138141000000000	0.523453039510001
FAM3D	-0.204542567224146	0.457385633697219	0.237823053438940	0.60243420134202	-0.173772666666667	0.583061615740711
STARD4	-0.204565298921791	0.457328094008081	0.266459370639749	0.538833471023229	-0.0895639999999994	0.671766969198954

MFSD2A	-0.204616704694637	0.457104670715014	-0.0274455624242269	0.970045676424287	0.0252986666666678	0.936115752055406
PGRMC2	-0.204774223390765	0.456415729889017	0.00775390639417392	0.990405674923721	0.0915686666666677	0.660936100042198
F11	-0.204786061421673	0.456415729889017	-0.0132375440090945	0.984362682224315	-0.109948666666666	0.714097156630726
GABARAPL1	-0.204801602168199	0.456415729889017	-0.199506236271577	0.683755152819083	0.182328000000000	0.343258557657641
TTC39C	-0.204806329119244	0.456415729889017	-0.162101179860146	0.75843356082254	-0.00723116666666653	0.98256318768706
EIF3I	-0.204833992599805	0.456415729889017	-0.392215947580075	0.279438850660868	-0.106343333333332	0.605491766505367
GFAP	-0.204931268457121	0.456025669436935	0.199289381034996	0.684227048797313	-0.171742999999998	0.488602547609567
ERCC4	-0.205027244047435	0.455619143132178	-0.293602694732046	0.480614908214975	0.266895333333335	0.179047856373835
C16orf80	-0.205126142035697	0.455286748412102	-0.294174875620029	0.480053141095177	0.125944000000000	0.536810332635776
LGALS13	-0.205136527419325	0.455286748412102	-0.09983324442773	0.867558388686851	-0.201274333333333	0.552476486760774
UGT2B4	-0.205247812229609	0.454907196375431	-0.244559413352127	0.587653287154633	0.0908006666666667	0.779485206901945
TMEM154	-0.205256214478388	0.454907196375431	-0.281128887598225	0.506886267079183	-0.116080999999999	0.620978598876035
NCAPH2	-0.205260727843757	0.454907196375431	0.198139229146147	0.687384485387017	-0.0192293333333330	0.94375604597120
PITX2	-0.205273937587579	0.454907196375431	-0.197459084580830	0.688995325061211	-0.150731333333333	0.528409919089401
ZNF560	-0.205306548625923	0.454893629098973	-0.198116551400295	0.687384485387017	0.132332333333334	0.636490807097692
TLR8	-0.205325887231520	0.454855946549694	-0.341843041803217	0.377158872804021	0.0375740000000005	0.885078623144221
MTBP	-0.205483465714651	0.454242563828121	0.178848243987034	0.726006906302736	-0.0531319999999992	0.855025841776156
SF3B5	-0.205514870116601	0.454201531148586	0.223036937527583	0.635247978870818	0.0526086666666675	0.826922793139517
GFM1	-0.205540337072402	0.454136271779594	-0.134742173874820	0.807867938002244	-0.101531666666667	0.68400971768044
NPAP1	-0.205564711670260	0.454069622589742	0.0856927280869832	0.889114985071002	-0.0981843333333323	0.772139923817113
KSR1	-0.205653642946967	0.453704767549341	0.412300831024450	0.242722273788294	-0.172055666666666	0.365126455342703
SLC35B3	-0.205771888551591	0.453176868516581	0.206259016886585	0.671467763213228	0.0854913333333343	0.813694870801638
GHITM	-0.205777781125324	0.453176868516581	-0.204851012145600	0.673728362275223	-0.0308033333333322	0.906093399285191
MAPKAPK5	-0.205783819759515	0.453176868516581	-0.289483728661018	0.488870852256136	-0.0228956666666653	0.933412982728621
FAT2	-0.206027882481799	0.452288136083943	0.199382002760152	0.68411230286818	-0.131393333333333	0.514889931507188
PIK3R6	-0.206064279791378	0.452226269592337	-0.148990266420476	0.782985332504358	0.0191633333333336	0.949287545570912
C9orf78	-0.206165414759587	0.451865907184461	-0.126448789770599	0.821615070538219	-0.148888999999999	0.447498161237612
VPS28	-0.206199603021747	0.451743089308255	0.199339790790453	0.684227048797313	0.0116610000000013	0.973783155960644
TCP10L	-0.206203421319618	0.451743089308255	0.00242768758408937	0.996043687131725	-0.00372083333333314	0.991357545611754
PTPN22	-0.206222240343847	0.451743089308255	-0.29675612768718	0.475937559514153	-0.130778000000000	0.518836392563847
HSPB8	-0.206245776191177	0.451743089308255	-0.223593962588686	0.634154463155482	0.0656480000000005	0.771373465365664
HCFC2	-0.20625603046416	0.451743089308255	-0.162429576144190	0.757797874336913	0.226732333333334	0.516635548538775
MORN4	-0.206379109270588	0.451301255575500	0.135652559109039	0.806567281799677	0.337854000000000	0.130076798248918
GPT	-0.206432620614873	0.451141273619072	0.184024840250976	0.71794014754511	0.0314663333333344	0.90945379489823
QPRT	-0.206457701174108	0.451141273619072	0.088747368185364	0.8841943128258	-0.209247999999999	0.277519340419717
KRTAP9-3	-0.206572168227569	0.450560986694626	-0.00634685296408865	0.991853352023739	-0.0240489999999997	0.943952610325773
SLC15A1	-0.206584208452087	0.450560986694626	-0.0597949900688444	0.926352922534113	0.0599140000000002	0.787768761656041
NCR1	-0.206631751300991	0.450553722245452	-0.131729875422079	0.810902972636175	-0.136732333333333	0.60378773986659
KRIT1	-0.206669896358485	0.450493184415040	-0.160804553709016	0.761016253411733	0.104444666666668	0.608105893667925
HADH	-0.206710828146640	0.450370486588258	-0.177797464315405	0.728031191477223	0.218007666666667	0.307774837877941
DCAF11	-0.206737692777762	0.450347958869976	-0.0545032490998147	0.9344692810234	-0.207139000000000	0.373444155303416
COL9A3	-0.206744372800154	0.450347958869976	0.43658562028423	0.195288910691951	-0.133466333333333	0.625065210774855
CDA	-0.206751009531282	0.450347958869976	-0.078447458023447	0.899281198071059	-0.139398333333332	0.589306764532592
ALG12	-0.20678446507224	0.450347958869976	-0.00368088403028049	0.994588964700772	-0.0111879999999993	0.971353351696823
GTPBP3	-0.206792493136987	0.450347958869976	0.08177691128224	0.89548747312664	-0.152259	0.46297633209186
GLUD2	-0.2068246593213	0.450347958869976	-0.376897598725490	0.308377960719105	0.262313333333334	0.32866979392804
LDLRAP1	-0.20691018538987	0.450169545926292	-0.400920183364823	0.262138265619497	0.244942000000001	0.190246103576351
FAM129B	-0.206942508808343	0.450057812502383	0.0775874134934474	0.900701176765499	0.0823880000000011	0.720904339419538
PCSK1	-0.206969635965727	0.449975929754729	-0.329308861094181	0.404890442264825	-0.150470333333333	0.488602547609567
GRAMD4	-0.207160932666157	0.449026066037809	-0.380006055264065	0.300906670900397	-0.156500000000000	0.477871006905274
RPS27L	-0.207194875496403	0.44890522174235	0.113877759491592	0.844361135286573	0.288732666666667	0.13704666630053
KIF2B	-0.207286129879154	0.448529706239361	0.054417247554497	0.9344692810234	0.0142123333333336	0.979527173532206
PRDX5	-0.207324216429806	0.448397666922222	-0.336837105835512	0.388635287154227	0.288323333333333	0.294300395506211
HTR5A	-0.207353477087214	0.448370768485554	-0.151334548022885	0.777604486471648	-0.0688799999999992	0.759696248427462
ISOC2	-0.207400972835434	0.44824032591262	0.359558784119838	0.341510139061518	0.0103763333333348	0.97435171941994
C18orf8	-0.207409825021374	0.44824032591262	-0.094984701951203	0.873166720419511	0.0184530000000012	0.946953175174742
COG6	-0.207443198908915	0.448145891362268	-0.3617107217039	0.338556116268406	0.0931760000000011	0.729736472326845
MRPS9	-0.207529490924772	0.447886843068816	0.0405762347989391	0.952438082414015	-0.0422766666666652	0.872259757014563
SNAPC3	-0.207620441223576	0.447499648564424	0.234599741782945	0.608421850871805	-0.0514093333333322	0.837163621558114
C20orf181	-0.207622173382475	0.447499648564424	0.287049592875411	0.494441582955552	-0.0531453333333332	0.821611372525586
TMEM171	-0.207681228527234	0.447299157964814	-0.0272832156954955	0.970207612777188	0.0484080000000003	0.870543028294187
SLC4A1AP	-0.207772301125362	0.446999164128786	-0.178647748807044	0.726393257762681	0.0444226666666684	0.85576203728561
THEM4	-0.207799254004172	0.446955625484715	-0.196038308254883	0.692325064566574	-0.0758033333333322	0.7226046641081
ATP5L	-0.207854814996080	0.446885212978983	-0.237984428587677	0.602142642649819	0.0361200000000008	0.88487182863547
CASQ1	-0.207868070357816	0.446885212978983	-0.0228226800268113	0.97536722461972	0.0818760000000007	0.707459630816261
WASL	-0.207920228607543	0.446742540827548	-0.325563126880131	0.409206882509236	0.0818453333333344	0.70916032632167
KLHL3	-0.208003916035657	0.446411432856291	0.149704983598821	0.78143277453973	0.184189333333334	0.410268789432262
CFHR4	-0.208008402759623	0.446411432856291	0.070888180475923	0.909485982367054	0.0190533333333339	0.956626092762687
HSF2BP	-0.208010571424711	0.446411432856291	0.128975757794327	0.815386814587013	0.137733666666667	0.549323798856043
SPINK4	-0.208011044526228	0.446411432856291	-0.291099118307466	0.486880079690803	0.0772983333333334	0.747245436362979
TTC22	-0.208034222378155	0.446411432856291	-0.00369015772540070	0.994588964700772	-0.0511499999999998	0.843134844119548
SLC7A6	-0.20803533170522	0.446411432856291	-0.0701009465692073	0.910580282989425	-0.188948666666665	0.368449159208846
ZNF837	-0.208050880086381	0.446411432856291	0.245044106535047	0.58629105649071	-0.146831333333333	0.607764514767022
WDR13	-0.208115144095012	0.446411432856291	-0.396769436635951	0.270862229167822	-0.00697433333333267	0.98256318768706
PIK3R5	-0.208178152136117	0.446300065946442	-0.240382306675358	0.597862631330416	-0.177920000000000	0.422571606196103
POLD3	-0.208188991598096	0.446300065946442	-0.0209874931749176	0.976103466334817	0.00573966666666746	0.988133186272453
MAP6D1	-0.208204304494616	0.446300065946442	0.137865587230695	0.801736842296971	0.0404290000000006	0.874960823800592
TXN2	-0.208231545855877	0.446289999349723	0.0694515119257814	0.91172160664395	0.0296826666666672	0.912223129892746
LOC93432	-0.208268910624747	0.446150165367509	0.0715274375794977	0.908870023167211	0.0641663333333337	0.787248874966057
ENOX2	-0.208338020253743	0.445828905491797	-0.589013884653043	0.0371136262345896	-0.208579000000000	0.390967882720418

E2F2	-0.208377245032358	0.445785214429039	-0.0320706665458692	0.962905980785005	-0.0664829999999996	0.777568084061469
TRIAP1	-0.208412095720566	0.445700766544151	0.0674117263520547	0.914318618000915	0.0199810000000006	0.94678260229888
ABCF3	-0.208475425684949	0.445486504558547	0.108356734284426	0.854386098374291	-0.0973409999999993	0.660122297714278
PRAMEF10	-0.208749272227847	0.4444393106884	0.0992971293835974	0.867939814640162	0.0556134444444453	0.830987625642722
PSMG3	-0.208757167603383	0.4444393106884	-0.378633550595012	0.304578046752771	0.0337313333333343	0.902718738113061
GIMAP4	-0.208768315658898	0.4444393106884	-0.447240423940823	0.178249202877872	0.0507913333333337	0.824700707442131
RPF2	-0.208777114309907	0.4444393106884	-0.101170610313032	0.866005093242444	-0.122152666666668	0.603043023754784
EZH1	-0.208801266724662	0.444437597252978	-0.478316835411318	0.140664651993598	0.000881000000000169	0.997264570850507
OR1I1	-0.20895372635999	0.443862895832274	0.303075783487016	0.462105017205825	-0.117286999999999	0.646350580563869
TMPRSS4	-0.208958632169072	0.443862895832274	0.0106498300276195	0.98774587825743	0.0122206666666668	0.974895360062947
JPX	-0.209003013249346	0.443862895832274	-0.304954863789938	0.458359470895422	-0.0477219999999996	0.904201124848034
NHP2	-0.209016689322154	0.443862895832274	-0.296198563099566	0.476960965340804	-0.238344000000000	0.296279771008449
SLC4A7	-0.209035785341246	0.443837458770733	0.000846880181412659	0.998745415362821	0.0403723333333342	0.898979600726666
FCRL3	-0.209047621918396	0.443837458770733	-0.196501375618947	0.691109282629346	-0.0586116666666661	0.794486775271372
KIAA0226	-0.209120642952247	0.443648705815115	-0.302651427026229	0.462172681353884	-0.0818639999999998	0.730777457405814
CYHR1	-0.209166890016660	0.443458808641621	0.270959905676595	0.52859316924857	-0.0469576666666658	0.862509125961694
DAAM1	-0.209177766197596	0.443458808641621	-0.016845120175576	0.981160061237418	0.375408333333335	0.0748141981193211
FSD1L	-0.209190230959077	0.443458808641621	-0.0663991414960853	0.916323048676587	0.154886333333334	0.466985343927182
KIAA1107	-0.209276619527840	0.443276093145644	-0.348236484965522	0.361374443308161	0.154049666666667	0.560413476606931
KLHDC10	-0.209311014226626	0.443153854271573	0.196836975257683	0.690061983084583	-0.488936111111111	0.0354366517739437
FAM133A	-0.209369144281438	0.442970186907166	-0.355689649981035	0.346946485404757	0.595157333333334	0.0140473413958925
COX5B	-0.209377013867427	0.442970186907166	-0.210316164680449	0.66192111529052	0.0423540000000022	0.890094615232462
ANKRD9	-0.209454808675277	0.442785848013971	0.07393964780093	0.905506084101768	0.173871000000001	0.419984075630105
GPR180	-0.20947596439017	0.442785848013971	-0.0417139066635192	0.951989245458556	0.565519666666667	0.0252836057158779
GNRH1	-0.209501138100003	0.442785848013971	0.057639088378426	0.929758936765496	-0.00226733333333265	0.99476816097137
STAC	-0.20955356317295	0.442657777592301	-0.352615613552959	0.352560848501456	0.0734699999999998	0.748388402768556
LRRC14	-0.209562964081872	0.442657777592301	0.358259615414967	0.343262129853326	-0.00831833333333298	0.98025379113353
DNAAF3	-0.209623357013709	0.442555677038948	-0.385846923937215	0.289470192870154	-0.182955	0.350536203215703
C2orf71	-0.209637160122346	0.442550944689242	-0.0518783087446677	0.938067483409541	-0.0722949999999991	0.82696895163688
UROD	-0.209711800483922	0.442199617717621	-0.185822282264245	0.713765959545662	0.0536310000000006	0.841432644293008
ASAP3	-0.209824079967672	0.44178190332389	-0.0243625704683010	0.973772197470206	0.155608666666668	0.530277423785419
LOC285074	-0.209827594135067	0.44178190332389	-0.039659885638818	0.953729831316741	-0.180077333333332	0.36301901523745
PPP1R11	-0.209851880108874	0.44178190332389	-0.196218984958719	0.691746760946447	-0.0720763333333329	0.763935841204439
KRT33A	-0.209914127105308	0.441688903551785	0.226388738055958	0.627133298652487	0.0844343333333344	0.873398028647672
AP1M2	-0.209925588040010	0.441688903551785	-0.208131412533708	0.667327688576615	-0.161876666666666	0.462956926523536
MBL2	-0.209945447417495	0.441688903551785	-0.187818697301805	0.708377185479843	0.176625333333334	0.35135795138426
N6AMT2	-0.209951230821628	0.441688903551785	-0.263550125040488	0.545610060822567	0.0570440000000003	0.823297424642592
ZDHHC12	-0.209958669999025	0.441688903551785	-0.111113811330420	0.849127967004266	0.122603666666668	0.583436892256128
SCN4A	-0.209972266879767	0.441688903551785	0.188421960109015	0.707361603786947	-0.175520000000000	0.420804920248363
PDCD2L	-0.209986634901598	0.441688903551785	-0.0102640934835201	0.988174744960397	-0.0900906666666665	0.693176364346567
KDELR2	-0.210006779217367	0.441688903551785	-0.249766881414399	0.57536723546666	0.207762000000001	0.309421300309741
NABP2	-0.210040497422455	0.441688903551785	-0.316968477395167	0.431780281826320	-0.118276999999999	0.555404460523821
ZSWIM1	-0.210145138323131	0.441581077994147	-0.200960616138424	0.680143077995217	-0.149968999999999	0.458006502146897
DCAF10	-0.210154007730526	0.441581077994147	-0.176540836257527	0.730152680173573	0.134596000000002	0.523453039510001
TSKS	-0.210172772239043	0.441571870902861	0.128336792342452	0.8164728691172	-0.0992586666666666	0.785482517274791
FAM81A	-0.210320347564852	0.441101964052298	0.238128288070205	0.601917317428488	0.0782970000000007	0.73168347388773
ETHE1	-0.210454473684007	0.440708975084358	-0.352908865708027	0.352407344218756	0.0433336666666667	0.861860658844305
B9D2	-0.210556937217365	0.440274185924392	0.116106286202354	0.842613538353187	-0.0502186666666653	0.84100404224146
CRCP	-0.210685101177994	0.439841609050723	-0.317128706671748	0.43145661636151	-0.196297999999999	0.337994438406310
MRPS34	-0.210775469366513	0.439550074402794	-0.0806702460476752	0.896621311541396	-0.131328333333333	0.51786180567444
TPRKB	-0.210808029737103	0.439513096091911	-0.0675519250368993	0.914119377247673	-0.0664023333333326	0.859412082013537
GLDN	-0.210831091469145	0.439456084532965	-0.172208747461893	0.738567943867944	-0.187104666666666	0.441366313859876
MTMR9LP	-0.210898160476746	0.439223097687139	-0.140722932290729	0.796714214272984	-0.145835000000000	0.60302310939506
NAALAD2	-0.210921999783933	0.439161703509699	-0.0382465495930089	0.954545200586067	-0.389161333333333	0.381938699863172
MYO1E	-0.210976641554431	0.438999399524847	-0.183131058088494	0.718067440349544	0.319663000000001	0.173494642908599
BDH1	-0.210999332965192	0.438999399524847	-0.0681813987933955	0.912364243071055	-0.391150000000000	0.0853703346605502
METTL20	-0.211010511823081	0.438999399524847	-0.267667131483728	0.535603758171522	0.270352	0.33989570998241
ADAM30	-0.211042886546939	0.438976500223113	0.134158152815739	0.808009477165608	0.139893666666667	0.541939010010167
CDK5R2	-0.211046140036718	0.438976500223113	-0.184816417020809	0.716020884288485	-0.120999999999998	0.580149967309238
DDC	-0.211057861907769	0.438976500223113	0.166738757492311	0.750443995356965	0.0490976666666673	0.837989744649002
PACSIN1	-0.211068531664044	0.438976500223113	-0.0288751930702718	0.96782691865619	-0.156307666666667	0.588052519326463
SERPINB11	-0.211111654850813	0.438976500223113	0.173761186982103	0.735353104758227	-0.111302000000000	0.603978635563313
ZNF311	-0.211178814972289	0.438814171744791	-0.189595721242551	0.704928430617595	0.352961333333335	0.0895407923270921
C1orf50	-0.211214782848201	0.438800976282356	-0.0900795786795017	0.88237793095104	-0.102604333333333	0.639837290428782
SLC37A2	-0.211252603477532	0.438691668299098	-0.0137300090291663	0.983872723592937	-0.000516333333333266	0.998351142601927
HLA-DQA1	-0.211256529970911	0.438691668299098	-0.368438984302637	0.324738935935866	0.209258666666667	0.361693444551121
MAGEC3	-0.211264700401774	0.438691668299098	-0.211509948393357	0.659412013514194	0.0164070000000006	0.952519513963942
CCR4	-0.211270157257466	0.438691668299098	-0.236679015349250	0.604560022191981	-0.131029000000000	0.661812056571718
MPDU1	-0.211275341403979	0.438691668299098	0.110568412989455	0.84940885580427	-0.167092333333334	0.40500623952892
HSPA12B	-0.211286006414977	0.438691668299098	-0.112512806022581	0.847191484945575	-0.105527333333333	0.66758573421638
ATP2A1	-0.211451385388030	0.438304691453926	0.138652242423248	0.79914918957102	-0.170324	0.42608701620641
PCBP3	-0.211458733883427	0.438304691453926	-0.181874664738181	0.720096350499662	0.137822000000000	0.528257160219719
R3HCC1	-0.21151731351816	0.43815301940318	0.159816435075938	0.763150272733332	-0.0421869999999998	0.860388779364817
DTWD2	-0.211520184728053	0.43815301940318	-0.334201417865498	0.394920851245792	0.224235000000001	0.294899302599823
DNAH5	-0.211541851856927	0.43815301940318	-0.192538547089966	0.700273993931252	0.0447866666666671	0.854212733579292
PRODH2	-0.211565301700917	0.438103299628475	0.162335950439724	0.758164869707887	-0.179625333333333	0.384156832156599
WDR81	-0.21171713710114	0.437467053354377	-0.265273882360603	0.541091405922744	-0.0648459999999997	0.777019091352957
PRKCD	-0.211728899304625	0.437467053354377	0.303789336220954	0.460052080110635	-0.144712333333332	0.71984456610521
B3GNT4	-0.211757769359209	0.437456307655562	0.0597501375260926	0.926352922534113	-0.0322936666666665	0.911880659501762
BRMS1	-0.211808089853296	0.437314314062237	-0.425373645825515	0.215369960032963	-0.0508919999999994	0.838813113208837

CARD11	-0.211831390828697	0.437314314062237	0.120055409545663	0.835142119002087	-0.246781999999999	0.347028131116938
CAV2	-0.211833443057996	0.437314314062237	-0.328106977025241	0.407365872456796	0.582705666666668	0.015124966128685
UBXN8	-0.211862855520091	0.437305634989108	-0.0978189859601352	0.868852648210739	-0.0599239999999999	0.828069064988993
RP1L1	-0.211871181539766	0.437305634989108	0.221471547335921	0.637714022834496	-0.059493666666666	0.827433678445379
ITFG1	-0.21189293945315	0.437283534969351	0.0323149416807857	0.962293977912377	0.225745166666667	0.348373106039088
PLEKHG4B	-0.211911066850239	0.437254988549062	0.162022666278022	0.75854722544558	-0.0944893333333328	0.683579075943666
TMEM55A	-0.211962665168266	0.437110856958438	-0.0533907237795748	0.93647464505741	0.0958040000000005	0.684275286725583
ELL2	-0.211966689958757	0.437110856958438	-0.201127300195499	0.680143033775131	0.582312333333335	0.0475135196981266
UBE2Q1	-0.212025572036403	0.436902621885458	-0.397791376700101	0.26911454304354	-0.150889999999999	0.451580494926556
STOX1	-0.212052556307516	0.436823879524112	-0.325739027111259	0.409206882509236	0.311492333333334	0.163986811016470
C1orf116	-0.212064280313184	0.436823879524112	-0.306198981266110	0.456406236471471	-0.147858000000000	0.579412709059421
BRAF	-0.212067722342799	0.436823879524112	-0.302625238818272	0.462172681353884	0.066865000000002	0.76276832167361
DHCR7	-0.212142283254987	0.436686637035925	0.119830692458644	0.8354985445494	-0.0206983333333319	0.946623914607177
KEAP1	-0.212224483959515	0.4363691934465	0.0118554299142302	0.986418801521542	-0.0338363333333327	0.895510661232872
LOC441204	-0.21229961239414	0.436017686439646	-0.160536474872822	0.761610418244687	0.183339333333333	0.343572153228906
ATP6V1D	-0.212313322092235	0.436014288004944	-0.130684199060777	0.812271254601992	0.0121750000000008	0.970481529511925
HIST3H3	-0.212384791505766	0.435683684929370	0.454852104054807	0.169205181987923	-0.0415326666666656	0.890792289911257
GLB1L2	-0.212394765120731	0.435683684929370	0.0131283258001307	0.984547546298896	-0.355575333333333	0.187388151358237
DNAJC16	-0.212469352494709	0.435419972497334	0.0747553839742518	0.904466414650249	0.111014666666667	0.623070283203198
KHK	-0.212469410667159	0.435419972497334	-0.0204508120915024	0.976412247012224	-0.0917689999999994	0.683149536498155
HBM	-0.212472469952512	0.435419972497334	-0.174723213303139	0.733916687337453	0.0171176666666675	0.959910474847156
KCP	-0.212476066507707	0.435419972497334	0.154059776086695	0.772253934458676	-0.127051166666666	0.674028958837018
BCAS1	-0.212524540599424	0.435419972497334	0.190943018804894	0.702438908776299	-0.063859666666666	0.81854356442803
UROC1	-0.212539548024443	0.435419972497334	0.313319358930952	0.438100620341757	-0.0419146666666658	0.883775402587518
TKT	-0.212552623621752	0.435419972497334	0.0099933653354577	0.9886179275502	0.0279000000000010	0.912884365217818
SETD6	-0.212732630012066	0.434754215609114	-0.112570965192651	0.847191484945575	0.068450333333334	0.76564992393702
GFRA1	-0.212737999032984	0.434754215609114	-0.15650593504521	0.768909201237015	-0.0975399999999996	0.73487643149197
CD5L	-0.212756868389720	0.434754215609114	0.0536744316585665	0.935777496327359	-0.0123016666666662	0.966753414674244
SEZ6	-0.212788613580301	0.434660285121471	0.0646958175992464	0.91904023678194	-0.0484819999999995	0.851568943534661
TREM2	-0.212806175340199	0.434635211945612	-0.29330320978142	0.481095955363282	-0.0317456666666666	0.904788907158467
CACNA2D3	-0.212856951301376	0.434569961880974	-0.212767427074301	0.656037270488756	0.289200000000001	0.335593399300607
FAM169B	-0.212868742198784	0.434569961880974	0.172513068655137	0.738421727152922	-0.156338666666667	0.614221661998915
SPATA17	-0.212930817116503	0.434450255884345	-0.15625333767008	0.768909201237015	0.164382666666667	0.479443005264132
ILDR1	-0.213007385014105	0.434091479788283	-0.0496931062851073	0.941458038227918	0.00770100000000071	0.982523503616988
RND3	-0.213133136684685	0.433529213777913	-0.445440793116462	0.181231004740944	-0.444495666666666	0.20763364966155
RNPEPL1	-0.213149373014585	0.433511716921192	0.0638704275482354	0.920459698398106	0.0595956666666675	0.808239992878146
CAPN2	-0.213238944085641	0.433302682570747	-0.211334867891643	0.659974817413141	0.495538000000001	0.0751433048669344
DCP1B	-0.213249405261307	0.433302682570747	0.0821192883280841	0.894840896036786	0.205737666666667	0.483327417766769
SPEF1	-0.213322723379397	0.433126510682533	0.384332991033278	0.291814756660665	-0.0616139999999997	0.843152489417868
CCDC39	-0.213353048816849	0.433029507641618	-0.065901735705606	0.917090201751967	-0.0886853333333329	0.709445924150415
CBLN4	-0.213357837142216	0.433029507641618	0.060383098069239	0.925649366494154	0.0208270000000005	0.946392308016584
LOC441242	-0.213380106936857	0.433025260436268	-0.248313984388868	0.57769394603049	0.0358056666666667	0.89662732250635
SQSTM1	-0.213408931879066	0.432986679032818	-0.277654387118744	0.513837569240538	0.0865213333333344	0.710004432586908
ZNF275	-0.213518343287288	0.432616141749638	-0.0300529793818564	0.966396286514145	0.0133700000000008	0.97057157918193
NSA2	-0.213523715206507	0.432616141749638	0.0208383082549042	0.976330597778846	0.155829000000001	0.447498161237612
GH1	-0.213581148623321	0.432410195014493	0.123885342690810	0.826287181959559	0.0617580000000003	0.785948076117221
ZNF394	-0.213596176910526	0.43239966274853	-0.577754621853809	0.0432751280177754	0.0234833333333338	0.927795934389862
INSIG1	-0.213629123411379	0.432288010217057	0.171901453513907	0.739043760943627	-0.0253279999999991	0.92469922248949
RBL2	-0.213714858455664	0.431878588753369	-0.458860078485401	0.165076020821271	-0.107199666666665	0.6034967860712
KIAA0146	-0.213730633038911	0.431863859826178	-0.0855783488506355	0.889266195398476	0.0555766666666681	0.809755698910303
CATSPER1	-0.213764097871920	0.431823622916507	-0.163679475115628	0.754968203901817	-0.0321076666666654	0.923494544470989
C1orf177	-0.213794877793045	0.431753615665365	0.0141217733601983	0.983444353656142	0.0031470000000005	0.993677488355417
FCAR	-0.213869063253532	0.431454510154961	-0.00750196352505085	0.99077339592597	0.0280536666666673	0.914172701010275
TOE1	-0.21389468486001	0.431384317281016	0.0368947828943543	0.956918184553224	-0.0244186666666649	0.933502474556197
FANCL	-0.213904831400495	0.431384317281016	-0.0197681828131533	0.97763669370967	0.146477666666667	0.484208511056468
LINC00115	-0.214028136007542	0.430855002598601	-0.0417146208584888	0.951989245458556	-0.104384333333333	0.705010402761235
NAGPA	-0.214045288354451	0.430855002598601	-0.0292183290953206	0.967677112447108	0.0600963333333342	0.806525311728398
HABP2	-0.214071183381892	0.430835130712432	0.0438744400037333	0.94885721615997	-0.0955776666666659	0.706573150130005
WWOX	-0.214072579640605	0.430835130712432	-0.100632859838054	0.866414040673375	-0.0189323333333332	0.94622978991441
TIMM10	-0.214087201404961	0.430835130712432	-0.347125850221358	0.363894788429779	0.191131666666667	0.392990708193759
ATF3	-0.214109909359288	0.430835130712432	-0.289173693190645	0.489048761714482	0.0292370000000002	0.910190419163007
KLHL15	-0.214445753554588	0.429542937801181	-0.0126440704170200	0.985214990982244	0.274422333333334	0.164238618805411
DHX35	-0.214454932216556	0.429542937801181	-0.224324942568753	0.632829553438263	0.100846000000000	0.659835984620576
CDADC1	-0.214516611663621	0.429441089924016	0.00946023547369169	0.98909166696601	-0.0609423333333329	0.883739719585572
IDI1	-0.214687686816006	0.428701996371204	0.224435442137194	0.632710354180004	0.108007000000002	0.614221661998915
DNASE1	-0.214716225518461	0.428615783155932	0.0938250377335131	0.875355472414883	-0.007464999999999879	0.98256318768706
CLIC5	-0.214861370151017	0.428319356886105	-0.078478660430896	0.899281198071059	0.150020333333334	0.627523379761431
TLR9	-0.214917876635513	0.428150318458195	-0.123706398229581	0.826475556843795	-0.339561	0.244059562675669
RCAN1	-0.214947748350183	0.428130857506988	-0.497812985406443	0.114179995542754	-0.112011666666665	0.633561720825521
SMG5	-0.215002298053615	0.428036591750010	-0.182408641100502	0.719419078472232	-0.0836003333333337	0.741809049318412
SEMA4B	-0.215136191425909	0.427517989805176	-0.192379212570981	0.700450711372106	0.116146000000000	0.640111005310948
MRPL52	-0.215152889996082	0.42749842026273	0.251156769283272	0.572882168047464	0.124048666666668	0.576277303044571
TBC1D7	-0.215187227228849	0.427379911539613	-0.283076129589654	0.502129505146473	-0.223188999999999	0.356860503105293
PTPRN	-0.215190608574338	0.427379911539613	-0.238723022506181	0.600803167481551	-0.194509333333333	0.450203306181989
BCKDHA	-0.215430783204387	0.426385043153186	-0.0440794286402475	0.948520928173175	-0.247383999999999	0.199107820683444
IPCEF1	-0.215485030197672	0.426229240095483	-0.210091414517656	0.663000357794695	0.0412423333333335	0.90616464808104
NT5DC4	-0.21564939567993	0.425605117068318	-0.0992103061431498	0.868042453226125	-0.0747343333333328	0.728429595651334
SPATA7	-0.215801899927321	0.424974018814019	0.0593554664867297	0.927064799234187	0.201624000000001	0.320462604748547
GABRA1	-0.215866171875865	0.424688711059196	0.0267093699741797	0.970207612777188	0.0016410000000003	0.99597023247862
GALNT14	-0.21590479816048	0.42454676062872	-0.140745934213724	0.796714214272984	0.000953000000001015	0.997144862160692

CAMK2B	-0.215928406774962	0.424488715778281	-0.125425811148945	0.824013463206763	0.141702666666667	0.503352066997863
UNC5D	-0.216009664074910	0.424378212690633	-0.108689622070693	0.8536426264533	-0.0882106666666662	0.727065902741195
GAS7	-0.216015383716405	0.424378212690633	0.0739127333517781	0.905506084101768	-0.104695666666666	0.707538975864338
ZFP57	-0.216020903487743	0.424378212690633	0.00364012948878837	0.994588964700772	0.00305233333333451	0.993677488355417
AADAT	-0.216034194394420	0.424378212690633	0.0212360008601541	0.975795855403795	0.441637000000001	0.126468086952493
GPA33	-0.216037739663886	0.424378212690633	-0.266370190570321	0.538833471023229	-0.0722263333333332	0.78837348455068
WBSCR28	-0.216098412338572	0.424277824500044	-0.0959219928514667	0.872499931194687	-0.119244999999999	0.65498201079628
TMEM52	-0.216108268910581	0.424277824500044	0.0923223329303317	0.879153312143409	-0.0873663333333326	0.76648935876098
UNC13D	-0.216140963605906	0.424277824500044	0.210654777276235	0.661670522548494	-0.128955999999999	0.546434057395997
CHST9	-0.216240084417379	0.423930139320689	-0.0487636432540605	0.94220484638146	-0.140301000000000	0.510489270790114
CRYL1	-0.216373951545929	0.423404634889896	-0.325643904466196	0.409206882509236	0.0926890000000002	0.702498982060037
PLB1	-0.216381961296280	0.423404634889896	-0.197664457714857	0.688640225556359	-0.0183819999999997	0.952154839195077
OTUD7A	-0.216473210972373	0.423072536452837	-0.0558290725241973	0.933151248545599	-0.00727199999999938	0.987134800094338
LOC284023	-0.216578133332645	0.422709058755777	0.0906826717548898	0.881370595149313	-0.186645999999999	0.374814376822818
SLITRK4	-0.216625801586284	0.42259103057904	-0.0863665064027254	0.88794718020464	-0.0141826666666665	0.966813448118178
NUP37	-0.216631610192325	0.42259103057904	-0.250928009874084	0.572882168047464	0.0447886666666678	0.873454962131763
LAP3	-0.216639698087731	0.42259103057904	-0.454425887504257	0.170182428228888	-0.187856999999999	0.322963996491853
MPHOSPH6	-0.216647881411516	0.42259103057904	-0.325011863877894	0.410560803218241	-0.0361819999999991	0.885345988320645
PVRIG	-0.216691808206449	0.42259103057904	-0.148061799742392	0.784080588385358	-0.110724333333332	0.64054750244519
CD3E	-0.216729655265592	0.422529283917339	-0.311010779321196	0.443901535667797	-0.135031666666667	0.530500926161619
ATP6AP2	-0.216759957288355	0.422434282753794	-0.46302649529358	0.158386420886203	-0.125902666666665	0.650637125741525
STK32C	-0.216816242841148	0.422194497011845	0.214742716372980	0.652003104177839	0.0229956666666680	0.930547487203917
SESN1	-0.216844409995446	0.422111441454209	0.403128001563112	0.258453301425503	0.457096000000001	0.0353807205776334
OCIAD2	-0.217008296405704	0.421346577173717	-0.144456948437366	0.790414495161718	-0.120278000000000	0.631961600239811
SHQ1	-0.217049893915194	0.421262679103346	-0.312483383145308	0.439831084453115	-0.127637666666666	0.544701772043015
MGC45922	-0.2170607747986	0.421262679103346	0.101252494196544	0.866005093242444	-0.0840373333333323	0.692545022319336
SDR39U1	-0.217097759543867	0.421143942273445	0.171958288851771	0.738962506786205	0.0532170000000012	0.831076445763244
CCL26	-0.217103631666173	0.421143942273445	0.0595085938052863	0.926864409932968	0.057949000000001	0.882040270326032
CPQ	-0.217150049278949	0.421054296605614	-0.00510618380487028	0.99251028338798	0.189486333333333	0.528409919089401
JMJD4	-0.217383772521096	0.420070155683044	0.00555068804161273	0.99251028338798	-0.239864333333332	0.275951503166653
ZNF85	-0.217460568324863	0.419772230038917	-0.0877716534118464	0.886199793361817	0.053212666666668	0.870662337224757
PAIP1	-0.217529593692801	0.41948150426291	0.188403480955834	0.707361603786947	0.254611666666667	0.177614563220760
LZTS1-AS1	-0.217547329012087	0.419456713055060	-0.0474650180160560	0.94396283143730	0.118379333333334	0.621638405081042
TGM1	-0.217559670262938	0.419456713055060	0.226869889346294	0.625901142430324	0.0334603333333344	0.919456505549688
ARMCX1	-0.217610909711336	0.419453048146725	-0.315040863514	0.436065505708053	0.0806810000000013	0.75988028828342
MAN2A2	-0.217671415348086	0.419431202404669	-0.265645647375394	0.539977750195292	0.00436733333333466	0.99024100084589
C9orf131	-0.217696244263735	0.419367114978726	0.187433265278123	0.709286896761914	0.119861333333334	0.623070283203198
RELT	-0.217737354784198	0.419212665693885	-0.0647874017888739	0.918815522665966	0.0723880000000013	0.772520347452753
C1orf64	-0.217748217478477	0.419212665693885	0.180025998203838	0.723716289903531	-0.00594466666666597	0.987937064305561
TARS2	-0.217778298899758	0.419212665693885	-0.424805943774687	0.216149711053592	-0.368567666666666	0.0944736471523537
CCDC85A	-0.217866118981498	0.418940607981883	0.142729823310798	0.794004622916083	-0.0984146666666663	0.637426515050706
SESN2	-0.217875929679766	0.418940607981883	-0.215309244965465	0.650576096954446	-0.157636333333332	0.41785973815832
PTPLAD1	-0.217887795539662	0.418940607981883	-0.167999009658109	0.748024524669236	0.187710000000001	0.33472722031995
CRADD	-0.217900675345615	0.418940607981883	0.0158436615900739	0.982102356159698	0.207880333333334	0.405375597663209
SLC2A4RG	-0.217988964738056	0.418849380306562	0.205781031389297	0.672625944768829	0.129913000000001	0.5531802635914
FAM46A	-0.218105412060233	0.418350994037738	-0.0229901929088108	0.97536722461972	0.449365666666668	0.0567061135792632
SLC25A27	-0.218208929151596	0.417924651826083	0.0845932441007947	0.891547139719321	-0.0868563333333335	0.688091879975642
DNAJC5G	-0.21822585471204	0.417904601313961	-0.0646101621026265	0.919265765276547	-0.0382143333333331	0.880427463662501
TOR3A	-0.218318181426093	0.41754053674676	-0.0611345427400773	0.924684666171101	0.0740450000000009	0.785166558910926
UQCRC1	-0.218341963305695	0.417482532601531	-0.298193741882373	0.471837952237768	0.026076666666668	0.919420614501031
ACY3	-0.218386439407702	0.41738365531347	0.0922658920181358	0.879153312143409	-0.0213453333333333	0.949807973269898
TRPA1	-0.218390502309255	0.41738365531347	-0.0218358804900353	0.97536722461972	-0.0387533333333332	0.873663396186202
FAM213A	-0.218418594711223	0.41738365531347	-0.0373440068071217	0.955937551268326	-0.395944666666667	0.0619237323392494
SNX4	-0.218433748657318	0.41738365531347	0.0520751513710318	0.938067483409541	0.0748336666666673	0.758008333232833
C11orf85	-0.218512173194729	0.417129857083093	-0.0693663585260044	0.91172160664395	0.117440666666667	0.604179762453377
ZNF217	-0.218517941695615	0.417129857083093	-0.274260276338355	0.520857714835269	-0.0186859999999986	0.956567100065073
CCDC93	-0.218650641612271	0.416805887217254	-0.167979734761922	0.748024524669236	-0.0123606666666661	0.97319570998424
ITFG2	-0.218656786384621	0.416805887217254	-0.148420484048057	0.78374265784053	-0.118198666666667	0.590147690656472
CD163	-0.218754046963828	0.416454943227955	-0.350442581012726	0.358430460089679	-0.0627533333333335	0.841976773189511
RBM11	-0.218798721037452	0.416281555301159	-0.127037494946867	0.820257254890606	0.147959333333334	0.518836392563847
GCA	-0.218814377566936	0.416268701540264	-0.284295544219695	0.500550458443644	0.209737000000001	0.304850986720083
ZSWIM3	-0.218865043290603	0.416062233657734	-0.111139224220412	0.849127967004266	0.176082999999999	0.350996401015483
AKAP6	-0.218876606212563	0.416062233657734	-0.00908176529263851	0.98909166696601	-0.0265046666666658	0.947020470290463
HBQ1	-0.218927701597643	0.415893567000567	-0.182118662072338	0.71964467602874	-0.113405333333334	0.756484285494797
DCT	-0.218961767012656	0.415822391255039	-0.175554930286995	0.73187914820204	-0.267824333333333	0.248764384117032
GOLGA5	-0.219059666348921	0.415428776799075	-0.258312864800105	0.560815056132889	0.162933666666667	0.397798624983652
DHTKD1	-0.219082265095196	0.415380214500838	-0.208274087783348	0.667000196762987	-0.272091333333333	0.353471671280201
HMMR	-0.219082490012625	0.415380214500838	-0.195050274722193	0.695157516663803	0.598490000000001	0.0175106182746974
MYEOV	-0.219110172189028	0.415380214500838	-0.0215037693078063	0.97536722461972	-0.0926859999999999	0.662502526900672
GRPEL1	-0.219148948816615	0.415377842950378	-0.236044089898148	0.60601651443518	-0.503234333333334	0.0303173110182138
SRP19	-0.21916190497348	0.415377842950378	-0.152320918088766	0.775863712880333	-0.208660999999999	0.514420666507349
SLC45A1	-0.219293984043903	0.415047408647271	0.0112603388241026	0.987339520470843	-0.189357333333333	0.381082408187926
TREML3P	-0.219296603776563	0.415047408647271	-0.307165921490447	0.453060894675967	-0.186354666666666	0.416841672160456
DTNB	-0.219299055394264	0.415047408647271	-0.0412045904152301	0.952098348256047	-0.00442833333333253	0.988133186272453
TYW3	-0.219353820393914	0.415047408647271	-0.429130682514423	0.209103669076588	0.143205000000000	0.479014333115247
COMMD10	-0.219359371974745	0.415047408647271	-0.194269617571122	0.696504707499023	0.125336000000001	0.58391025729515
ETFDH	-0.219423041150766	0.414822965202851	-0.25853193390919	0.56003769471698	0.172764000000001	0.411878887058133
LRP11	-0.219485200769031	0.414701170157615	0.395267488268298	0.272692742937895	0.0614053333333342	0.780704356521475
C8G	-0.219540541265390	0.414469410138863	-0.366890946520721	0.328118420233443	-0.0787713333333334	0.823062219441786
CXCR4	-0.219542497587233	0.414469410138863	0.198968859868448	0.685196748018683	0.915232333333335	0.0185304620090284

GAPT	-0.219567265681445	0.414469410138863	-0.26907845013937	0.533661607275425	0.042411333333334	0.861165447055876
PCK2	-0.21960453151364	0.414337633131693	0.153287621439925	0.774003008542746	0.039933666666668	0.874243298833041
MPZL3	-0.219728101082330	0.413729604290818	0.0115922351341628	0.986661750359244	-0.138177999999999	0.518836392563847
SPIB	-0.219734637273147	0.413729604290818	0.266145112178046	0.538986305036309	-0.140937333333333	0.625510328921698
SIDT1	-0.219743047177981	0.413729604290818	-0.286064973088484	0.496429042723156	-0.0273029999999997	0.940080097693299
DMPK	-0.219744062973476	0.413729604290818	-0.0590905479047906	0.927743508587488	0.0208936666666678	0.94622978991441
LCA5L	-0.219896703800437	0.413316287838692	-0.150734468114359	0.778708853878748	-0.089502666666666	0.712509439611678
CAMK4	-0.219916430658866	0.413281314867321	-0.149459725104319	0.78215339162259	0.0890213333333341	0.678589880084092
C15orf56	-0.219919606739944	0.413281314867321	-0.220716541644185	0.639008702745457	-0.083918333333332	0.790848042413284
LOC100130331	-0.219984470482891	0.413275242560644	0.0367541758188458	0.956918184553224	0.0686600000000019	0.809138260707169
UPF3B	-0.220003851882329	0.413266552696229	-0.474712481580793	0.144197096534399	-0.0223113333333329	0.951011480824118
TMEM155	-0.220012300390056	0.413266552696229	-0.126164154381026	0.822164329801713	-0.030364666666666	0.937088747554542
TMPRSS6	-0.220085068359636	0.413163776111415	0.112290896917969	0.847454473507881	-0.0247016666666663	0.94622978991441
C16orf87	-0.220124684302999	0.413019249025036	-0.157312640975438	0.767774203843122	0.255909000000001	0.184913381487752
PSMC1	-0.220233547588365	0.412567039955036	-0.34772694268024	0.362409633458069	0.0240800000000006	0.92980287143849
THEG	-0.220246966727506	0.412566952207831	0.322333723552064	0.417538034812104	-0.096880333333333	0.73097308185803
OSBPL2	-0.220299180944638	0.412427013488449	0.0700037780153156	0.910821504793275	-0.501005333333333	0.057177779614934
PLEKHA6	-0.220334161524974	0.412409159873773	0.0138529757169424	0.983872058957207	-0.0149336666666656	0.96433869918248
CCL19	-0.220367249074657	0.412347534364454	-0.188046576368691	0.70790719815331	0.089365333333334	0.764973469318093
CCDC96	-0.220394390077168	0.412333837085274	-0.255611235478916	0.565719761550949	-0.0340096666666663	0.896255571019035
KLHL35	-0.220394515157354	0.412333837085274	-0.171832449387747	0.739167054766667	-0.0912303333333331	0.775273488714107
GPR135	-0.220394857939863	0.412333837085274	0.224249667284305	0.632870078168085	0.0410776666666668	0.865571667607644
SIN3B	-0.220429238480764	0.412333837085274	-0.0380215514211913	0.95481328986492	-0.0494649999999993	0.858848223547254
NOD1	-0.220456854294595	0.412333837085274	-0.416451985103429	0.234235464483992	0.0498966666666669	0.859299487874574
MAT1A	-0.220457699972186	0.412333837085274	0.0978438806717727	0.868852648210739	-0.0649156666666658	0.798344353938564
METTL21C	-0.220481482372017	0.412333837085274	-0.362468929684496	0.337635834513295	0.0364976666666675	0.888285531317364
KIRREL3-AS3	-0.220501117724704	0.412333837085274	0.107339570926218	0.857128584932911	-0.133838666666666	0.5530742296054
WNT6	-0.220563239678857	0.412333837085274	0.142383537631962	0.794346532964905	-0.0549726666666663	0.862509125961694
LCN2	-0.220624989655425	0.41218515696756	-0.204405244505004	0.674817810302676	-0.094130333333332	0.676226154991835
CDSN	-0.220625688075911	0.41218515696756	0.0285040024783454	0.967951385746003	0.00206866666666772	0.99628178244892
HADHB	-0.220668248224176	0.412094937536418	-0.0150769454360274	0.982921609770846	0.108393666666668	0.604966458785844
CSNK1G2-AS1	-0.220913227888413	0.411042197135469	0.188385066944856	0.707361603786947	-0.0120096666666654	0.974451420920923
SLC6A2	-0.220942412641136	0.411029576498123	-0.223839422246745	0.633743682471632	-0.541242333333332	0.15724420804312
CCKBR	-0.220960172113246	0.411005856155205	0.0931695677431428	0.876995589714343	-0.206684333333333	0.511780844477390
C1S	-0.220986412657245	0.41093548752646	-0.267975935833557	0.53559545997875	0.133600333333335	0.544721221322874
TMPO	-0.220997927019217	0.41093548752646	-0.214305452720642	0.652991165086813	0.140583333333336	0.49679816436215
TM4SF1	-0.221028194789673	0.410853637137417	-0.556665696350652	0.0555982011257914	0.250401000000001	0.48608244495005
CHN2	-0.221031199621112	0.410853637137417	0.148981562605914	0.782985332504358	-0.159597666666665	0.583061615740711
FAM155B	-0.221141376088626	0.410400235925056	-0.340635489292259	0.378350877728096	-0.0824869999999988	0.719304894613107
KLHL32	-0.221143067018213	0.410400235925056	-0.35944517644206	0.341912831255326	-0.0862769999999993	0.694407056566956
TPST2	-0.221152392640516	0.410400235925056	-0.124901482461664	0.824457843812434	-0.293411000000000	0.147433353861458
RERG	-0.221393640946953	0.409591831550925	-0.496925935931175	0.114964366876551	1.08102800000000	0.00383531955221498
SIRPB2	-0.221422951880552	0.409591831550925	-0.163684966398372	0.754968203901817	-0.0585413333333329	0.813045722907269
RAD50	-0.221424959970436	0.409591831550925	-0.252745557189544	0.569123293812549	0.0854723333333334	0.728429595651334
C14orf178	-0.221472849922188	0.409591831550925	-0.103611020697489	0.861872724387044	-0.0372010000000001	0.915761046438965
MITF	-0.221476407940351	0.409591831550925	-0.477696577496503	0.141529695531073	-0.159003999999999	0.42608701620641
NDUFS2	-0.221546011917965	0.409487706478505	-0.298122910329162	0.471965705880214	0.0616986666666679	0.777406710539424
CELF6	-0.22159266088103	0.409400624711454	-0.124112611343920	0.825526959189789	0.0254000000000015	0.931467600666044
TMEM57	-0.221715149764297	0.409122458004943	-0.300708241073447	0.465222095816255	0.0093763333333345	0.97482729795816
SULT1C4	-0.221780139931421	0.409046409965906	-0.449008632706656	0.177860736739947	0.0714139999999998	0.774104133965233
ERN2	-0.221786491953482	0.409046409965906	0.149010054000275	0.782985332504358	-0.0453819999999997	0.855080191855446
ME2	-0.221868236407351	0.408829402264892	-0.315294938383147	0.435453514843947	0.146650333333335	0.460374458843217
MRPL44	-0.221953067620312	0.408720859262330	-0.151973688716103	0.776173016352835	0.110651666666667	0.670113640649168
C14orf159	-0.221956124702586	0.408720859262330	-0.168201559435006	0.747936039510864	-0.131053666666666	0.516217427447249
FAM53A	-0.221975160593973	0.408720859262330	-0.0583659033809025	0.928725686060612	-0.103079333333333	0.681312448752638
CCT8L2	-0.221997331306802	0.408720859262330	0.128405093148631	0.816345918941366	-0.00166533333333228	0.996606768451015
CASP4	-0.222008520311585	0.408720859262330	-0.365170121656449	0.330886935712135	0.0729386666666682	0.783223894269194
DDX4	-0.222213234566571	0.408315434946706	0.255031429452398	0.566577861267331	0.206432666666667	0.3382337893737
YIPF5	-0.222217136517128	0.408315434946706	-0.3568387787252	0.345138088652208	0.0684710000000012	0.759162700398522
ARL2BP	-0.222233484639317	0.408315434946706	-0.0706113829610832	0.91009246060164	0.217839666666667	0.249466878801630
WDR36	-0.222257009309896	0.408315434946706	-0.382594356869527	0.294988445038215	0.00361000000000033	0.992206704554549
SNX2	-0.222263986037407	0.408315434946706	-0.233452337351057	0.610436189314218	0.150065666666667	0.517547647029412
SIGLEC5	-0.222267008641767	0.408315434946706	-0.00353882383200793	0.994730189431381	-0.0365919999999988	0.902208543897795
SERPINF2	-0.222280894534115	0.408315434946706	-0.0265715880409483	0.970207612777188	-0.0310179999999992	0.941559580750404
NTS	-0.222331275641552	0.408291706641824	-0.137821735753441	0.801744061130323	0.25622	0.192973194605635
MRPS18C	-0.22241040412364	0.407937694179576	-0.251596245641369	0.571547753805264	0.149126666666667	0.457682901611326
CLCNKB	-0.22250901020317	0.407586532029621	-0.219905088049520	0.641001372559671	-0.189138666666667	0.476515795581148
SYP	-0.222634817755518	0.407220992395331	-0.186598291105925	0.711306976302188	-0.130203999999999	0.579113095252633
PPIE	-0.222705952212454	0.406905403371789	-0.0808696859750996	0.896361013063453	0.0288400000000005	0.90945379489823
USP27X	-0.222734721309799	0.406822024188731	-0.251911150816505	0.570584539772622	-0.0494890000000001	0.85994094150432
TECTA	-0.222746549447798	0.406822024188731	0.0090636258477582	0.98909166696601	-0.0798453333333323	0.769616032414453
DNAJB9	-0.222778320558617	0.406731663954343	0.00337180655375128	0.994730189431381	0.227996333333334	0.269653172251380
STOML3	-0.222817252216875	0.406592660588389	-0.119608997248404	0.835781442463157	-0.0878936666666665	0.696098804897955
COLQ	-0.222839423608570	0.406545472295097	0.0702248188866066	0.910394157189308	-0.0707933333333322	0.775712904419558
LYSMD2	-0.222843624122407	0.406545472295097	-0.120672982600006	0.833602134657439	0.056786333333334	0.843134844119548
TMEM107	-0.222960557358407	0.406179169508392	-0.109541162104632	0.851624566716206	0.412876333333334	0.0697146597616042
NAALADL2	-0.222989824071186	0.406093201623267	0.0402014033590345	0.952930192037229	0.147694	0.530783297878648
SNX24	-0.223023837854262	0.406046491719101	0.0323771710132260	0.962269716253437	0.336497333333334	0.105971509018004
HMGN5	-0.223229250276502	0.405006408285308	-0.563915709858854	0.0501997179061873	0.330012666666668	0.190569948330175
JAK3	-0.223369516452354	0.404654407024462	-0.167913548621785	0.748125243418359	-0.00654666666666636	0.987459024686908

CASC4	-0.223381577571522	0.404654407024462	0.0318491694065968	0.963211116393051	0.155457333333335	0.433659122703145
C11orf54	-0.223386333514407	0.404654407024462	-0.546493085009931	0.0635875421378674	-0.0241119999999990	0.935261323444314
ZBTB17	-0.223435925040508	0.404654407024462	-0.199851067047223	0.682777037002814	0.0204900000000011	0.93901998985171
MTX2	-0.223436441687951	0.404654407024462	0.169760057564610	0.743337921052762	0.240937666666668	0.209300554554219
ADTRP	-0.223453599561031	0.404654407024462	-0.0752250830237245	0.904173044514532	-0.168095333333333	0.459236736587605
AARS2	-0.223513704513079	0.404508192854289	-0.0554447947367135	0.933409139126562	-0.287038333333332	0.166543996066754
VRK3	-0.223602602402606	0.404227329222235	-0.236136563503367	0.605775058649527	0.166699000000002	0.422927358730594
ETFB	-0.223615055671677	0.404227329222235	-0.147124729514119	0.785734908309155	0.0931183333333335	0.673921955474628
KHDC1L	-0.223616154528852	0.404227329222235	-0.00434834530151121	0.99405344216434	0.0386810000000009	0.899614658619676
C11orf80	-0.223622216857834	0.404227329222235	-0.319823705419539	0.424685308100647	0.225777000000001	0.287956132210494
CIRH1A	-0.223774366742908	0.40373479353367	-0.257102968439340	0.563116080558022	-0.246141999999999	0.246446606133733
PLSCR2	-0.223777332246357	0.40373479353367	-0.158247698109504	0.767214373310291	0.207749000000001	0.296821581558449
TNFRSF11A	-0.223789452833538	0.40373479353367	-0.372288765955552	0.318024489042558	-0.0561513333333331	0.842903889380894
BSPRY	-0.223840873462947	0.403594419798368	-0.186196741117227	0.712609598901976	0.147837000000000	0.549659494702918
KRTAP4-12	-0.223853798386601	0.403594419798368	0.0430136576427035	0.950146638225747	-0.0857219999999988	0.696217349388802
WDR38	-0.223859648111759	0.403594419798368	0.257667243133304	0.562720155321314	-0.0726856666666656	0.747245436362979
ACER3	-0.223943829275641	0.403255354352458	-0.516800057229241	0.0919553206911016	0.308782000000001	0.108749304547981
OPRD1	-0.224105154186729	0.402746662053215	0.202417176496224	0.67896916637466	-0.129982333333333	0.677647679541427
C9orf91	-0.224122561645781	0.402725933570316	-0.0217788580494023	0.97536722461972	-0.000708999999999895	0.997953056544486
NLN	-0.224129240621163	0.402725933570316	-0.375179318637518	0.312620690092402	-0.186292666666666	0.471709876204528
NAT8B	-0.224276625528745	0.402109162970231	0.0226645366769803	0.97536722461972	-0.108909333333333	0.604966458785844
C1QA	-0.224294273547778	0.402087157429111	-0.443204005421177	0.184625836141753	-0.0778423333333328	0.726931357930535
PTPN11	-0.224333841911185	0.401962515788963	-0.213209593072553	0.655051855533403	-0.0322019999999988	0.903817894383617
DNAI1	-0.224366281147955	0.401917435006880	-0.0213728959047599	0.97554687597484	-0.0732676666666659	0.760700494137744
CPLX1	-0.224403583837506	0.401795734624349	-0.0578379010197448	0.929715247340158	-0.102190333333333	0.696194439936211
LGSN	-0.224477229868604	0.401610017085912	0.0179263987725107	0.979822697688271	0.0127876666666670	0.966149176465358
PYCARD	-0.224495573287181	0.401606737710959	-0.334306330253696	0.394691211498274	-0.224221333333334	0.337994438406310
EMC7	-0.224542701883715	0.401600882235195	-0.193507536127108	0.698131592467487	0.0933160000000012	0.679958892307394
CDC42EP2	-0.224592461985613	0.401427716387775	-0.394909980629583	0.273241538638709	-0.0227956666666656	0.9467761639719
MPP2	-0.224619789084339	0.401353186119477	0.153044742463071	0.774500444636875	-0.100054999999999	0.66807888867384
ZBTB8OS	-0.224755306974519	0.400796786994708	-0.0768115343347332	0.901856698918763	0.155772666666668	0.498454352317107
IFNA6	-0.224841032474784	0.400446921135611	0.113740087933002	0.844923516576119	-0.00973333333333317	0.978109825558428
ADAMTS17	-0.224871493766704	0.400355496253027	-0.0558303750908559	0.933151248545599	-0.291847333333332	0.139122737306306
CNPY2	-0.224892266477179	0.400316698396658	-0.000344369017095211	0.999728920942978	-0.150112666666666	0.481989564291462
CELA3A	-0.224916095619925	0.400261313358926	0.104583720911160	0.86067831050617	-0.0134256666666665	0.980779858267938
NR1I3	-0.225033340292458	0.399898699844754	0.0811830687825098	0.896346444707454	-0.0505803333333328	0.852130240791585
C6	-0.225153003577112	0.399488376192033	-0.0276163127763908	0.969778965077813	0.100839000000001	0.657217907037306
ZNF32	-0.225161200230065	0.399488376192033	0.0705061971682818	0.910169586639542	0.325696666666667	0.171804245444023
APEH	-0.225200937773044	0.399481117996288	0.0241145750190250	0.974276665384912	-0.0825519999999986	0.719500645075484
AKAP3	-0.225214906269190	0.399481117996288	-0.260874006047202	0.553100551096796	0.166761333333334	0.522830837265953
RELL2	-0.225259135298296	0.399435901054902	-0.0785056412493898	0.899281198071059	-0.00308366666666546	0.993677488355417
GRID2	-0.225323615105540	0.399234236199210	-0.25344258178715	0.567705016149998	0.216784666666668	0.390966211084762
PTRHD1	-0.225435302259299	0.398736518016279	0.186752477355073	0.710841653311676	0.163816000000001	0.436636858796753
TDRD9	-0.225448081933027	0.398736518016279	-0.472725919861818	0.145443641689931	-0.0705543333333327	0.744921965377944
ITLN1	-0.225451487000612	0.398736518016279	-0.208043293907652	0.66739374020401	-0.0756990000000003	0.72457851707683
COL23A1	-0.225503268976038	0.398736518016279	-0.0389122680414168	0.954165422885166	0.00913166666666676	0.983159241812861
ZNF788	-0.225543118194017	0.398710590262227	-0.294661839683868	0.478884401710241	-0.0654369999999998	0.776606595031724
CD84	-0.2255750102371	0.398685877352536	-0.353154571593956	0.352407344218756	-0.0337076666666666	0.891780340140922
HLA-DRA	-0.22559881957265	0.398630911325383	-0.576128163362603	0.044102721191954	-0.0955743333333328	0.741438256509195
TCEAL8	-0.22563501265304	0.398535919362169	-0.189000161151580	0.706922219214013	0.240983666666668	0.204586504492032
MAL	-0.225861223440109	0.397727980607894	-0.141105848083446	0.796333933122002	-0.0530573333333327	0.870662337224757
DNAJC8	-0.225884559261582	0.397676695183782	-0.271462452944919	0.527984832801825	-0.124713333333333	0.548291557744437
PLA2G2A	-0.225964078877272	0.397467428281066	-0.392321016607733	0.279346915677921	-0.151494999999999	0.502191611703409
PYY2	-0.226041198406706	0.397198242010102	0.388470599619356	0.28486220160813	-0.263180333333332	0.368449159208846
MYLK4	-0.226142043951185	0.396800920651418	0.0341665523057454	0.95969480339665	-0.419866666666666	0.100756942335889
IL9R	-0.226147595350104	0.396800920651418	-0.0113649565569550	0.987250302234215	-0.0627186666666654	0.830320848383177
OPN5	-0.226192100276516	0.396752271818644	0.152879735362659	0.774725957219946	-0.00708933333333303	0.987937064305561
MRPL39	-0.226349114966539	0.396051914728563	0.297138518647009	0.47472856434884	-0.0706076666666668	0.752518458717797
TCP11L1	-0.226438012596684	0.395719835433369	-0.285238620606108	0.498216546497375	-0.0231906666666662	0.951011480824118
STC1	-0.226438118661556	0.395719835433369	-0.106811821367140	0.857800958803773	0.395678000000001	0.135004030972507
ZNF789	-0.226450946421146	0.395719835433369	-0.447539484520414	0.178249202877872	0.0839870000000002	0.69291321690025
FRRS1	-0.226480271333903	0.395719835433369	-0.193688083680359	0.69772764496956	0.229534000000000	0.224147588185176
C17orf77	-0.226695939307438	0.394919543141293	-0.0251023589145852	0.972494094458606	-0.0413223333333327	0.866671423175521
TCHP	-0.226987835340531	0.393642945504446	-0.229380818982562	0.620183562399927	-0.287120999999999	0.135033058827820
GPC1	-0.226999951316135	0.393642945504446	-0.0285761673887622	0.967838039797494	-0.0857233333333315	0.6846855637534
CDH24	-0.227003915017646	0.393642945504446	-0.0718213089366594	0.90837606217316	-0.0545256666666655	0.819626240637794
COL24A1	-0.227029675493090	0.393638887768747	-0.121309259233551	0.83247225420367	-0.137777333333333	0.607886568089557
ATP6V1A	-0.227036616157667	0.393638887768747	-0.27253086469403	0.526175116911121	-0.0366943333333323	0.883189085233712
ZPBP	-0.227050523820617	0.393638887768747	-0.240026706088401	0.598092982260025	0.184209333333334	0.398349038294794
BTN3A1	-0.227088044454418	0.393638887768747	-0.250698270092686	0.573420630064112	0.129679000000001	0.570550813984136
SNCB	-0.22719049980889	0.393289885994827	0.0250029897115608	0.972747285652953	-0.2202	0.418950140369692
PNMT	-0.227212150350057	0.393247190998558	0.144363565123660	0.790546040025025	-0.053941333333333	0.861690060758414
NAP1L3	-0.227272009213763	0.39307282442897	-0.203685673009891	0.676117692782917	0.0376046666666671	0.888067040366836
SPAG7	-0.227309645412037	0.392944248951418	-0.111103765626740	0.849127967004266	-0.0571803333333331	0.849909082952122
DOK4	-0.227323228708981	0.392944248951418	0.191430006623602	0.701635983922402	-0.0176283333333323	0.967985930883208
ARMC12	-0.227429234867812	0.392596495699220	-0.190219189029478	0.704208582824765	0.0356166666666668	0.918363743182112
FAIM	-0.227487918984812	0.392502380056775	-0.139681203574888	0.797957231657366	0.00961533333333362	0.974895360062947
SERTM1	-0.227564028494903	0.392167391177714	-0.255219558153659	0.566136012063882	0.00489333333333404	0.987937064305561
SERPINA4	-0.227652290675090	0.391841018408228	-0.0337411698327053	0.96053713440028	0.0140763333333336	0.965661221820942
WBP4	-0.227674609965797	0.391794905590096	-0.257841790160152	0.562492892055072	-0.033236666666666	0.925476944639214

TREML4	-0.227716427655918	0.391717843322044	-0.208875111171539	0.665595909869988	-0.00397966666666698	0.994379224023514
SASH1	-0.227749878234029	0.391685705170899	-0.247097359578373	0.580507768773699	-0.303318666666666	0.241725325535274
ZFX	-0.227759310197578	0.391685705170899	-0.256318612900292	0.564430426783556	0.358621333333334	0.121660357743656
CNOT6	-0.227764947623763	0.391685705170899	-0.0848095085672787	0.891309254245872	-0.115845333333334	0.58391025729515
BABAM1	-0.227778275787725	0.391685705170899	-0.114681929189788	0.844162087612813	-0.0734349999999985	0.733679380704515
SLC2A5	-0.227824751364743	0.39157877423626	-0.214679094459398	0.652003104177839	-0.0233993333333335	0.962620058410129
FUNDC2	-0.227827705539516	0.39157877423626	-0.332878703351708	0.397658808533403	0.651438666666667	0.0135533679164567
CD36	-0.228002820453492	0.390992342251522	-0.109775844193405	0.851195083614214	0.288780000000001	0.133614476543628
MS4A6A	-0.228019316259888	0.39097759417329	-0.483953698018746	0.134615454592634	0.0265223333333337	0.919456505549688
FAM195A	-0.228024523028853	0.39097759417329	0.0920685337935195	0.879153312143409	-0.0965009999999995	0.719909781429035
SLC7A10	-0.228043866999833	0.39097759417329	0.0894529402590471	0.883383266241373	-0.115589333333334	0.575378149225683
SEMA4D	-0.22808559786842	0.390843395459405	-0.411645724798563	0.243528138193926	0.063015166666668	0.782236110556264
C5orf27	-0.228096001935348	0.390843395459405	0.183351124825077	0.718067440349544	0.0225460000000006	0.94692575385424
TGFA	-0.228117357204901	0.390843395459405	-0.109401194423001	0.851975798726468	0.057973333333334	0.801763490633
C1orf54	-0.228201304872364	0.390591871635824	-0.236217210563124	0.6055054064321	-0.290891	0.128450477696566
RGS14	-0.228215542920548	0.390589301752613	0.234476057676112	0.608446568003374	-0.065268999999999	0.819836239597076
ACAT2	-0.22822783231099	0.390589301752613	0.288924355651701	0.489528138271421	-0.0341793333333327	0.891339318634358
SULT1C2	-0.228300012601518	0.390459037337855	-0.0871398162970276	0.887055939766881	0.469992333333334	0.369542751253673
STARD5	-0.228320042143660	0.390459037337855	-0.313981756670982	0.438046311563112	-0.195101999999999	0.470132494599548
SLC4A4	-0.228384549901439	0.390418313244989	-0.0698558164333193	0.910885619751085	-0.0512363333333323	0.822657709336468
LGR4	-0.228418901617288	0.390418313244989	0.242782814912261	0.59251299743287	0.613968	0.0166166190922455
NGFRAP1	-0.228492577958757	0.390137994435163	-0.366668785611436	0.328151450414815	0.270867333333334	0.147699085494653
MYADML2	-0.228602197047324	0.389826376425784	-0.0311542637022780	0.965118873711308	-0.0969443333333334	0.695654544311453
GPR182	-0.228666852916735	0.389647712939183	-0.073499980979071	0.905838142287595	-0.0156443333333328	0.957243575945961
C19orf18	-0.228677974654151	0.389647712939183	-0.142149295064218	0.79450771779567	0.0364813333333335	0.9061074113295
FAM228A	-0.228698116185348	0.389647712939183	0.0455500592931544	0.945769054147694	-0.0445156666666667	0.896186784012643
FCHSD1	-0.228793519746203	0.38948663464112	-0.0353782026376487	0.95869292666698	-0.116140666666666	0.578893615511221
MEPE	-0.228864525472262	0.389245793438402	0.119567516328379	0.835862307448206	-0.107056333333333	0.600950363504414
ARFGEF2	-0.228921368128065	0.389156168388652	-0.102484218691790	0.863158472924381	0.181005000000001	0.425087070314654
SYT16	-0.229043587108748	0.388665828081085	-0.172162841131250	0.738646873992406	0.129787	0.527465067495661
PIWIL1	-0.229096756675293	0.388529249342852	0.231420171182207	0.614939073296746	-0.0255806666666664	0.942881285019803
FAM131C	-0.229097608909427	0.388529249342852	-0.112237470586424	0.847454473507881	-0.226795666666666	0.266962150412040
HOOK1	-0.229101027174163	0.388529249342852	-0.411104298879148	0.244156737438911	-0.226164333333333	0.422927358730594
RAB7L1	-0.229170637130877	0.388529249342852	-0.0761802122430137	0.903296922694955	-0.323907666666666	0.189095480222886
APCS	-0.229195721876383	0.388529249342852	-0.0987350603726048	0.868042734188964	0.0313080000000005	0.952448105846014
NMRK1	-0.229234457009262	0.388529249342852	-0.154429057404703	0.771310656070265	0.227321333333334	0.234366351979507
CD164	-0.229237758714103	0.388529249342852	-0.0091198286932606	0.98909166696601	0.202710000000001	0.284033466227373
PLD1	-0.229257406129513	0.388529249342852	0.115039493611814	0.844162087612813	-0.326811333333333	0.100026144425143
AURKC	-0.229329432564088	0.388489480836173	0.086112793510769	0.888224932417267	-0.0498923333333324	0.899614658619676
EPB41	-0.229371616570667	0.388489480836173	-0.422040682482744	0.222319600995852	-0.229079333333333	0.256673063330227
DHODH	-0.229398749171901	0.388489480836173	-0.0646369882278287	0.919198375420231	-0.0919243333333327	0.714244999058301
SYCP3	-0.229437526094422	0.388408560457674	-0.0103881572671632	0.98797403729164	-0.102542000000000	0.632087270447934
COPS5	-0.229454856168298	0.388389985398199	-0.0966025872404747	0.870857630743202	0.122997666666667	0.540255856426522
PSMD10	-0.229472064393672	0.388372071899916	-0.345150781298814	0.368530625231133	0.220606000000001	0.236989286061345
RFC2	-0.229479765575029	0.388372071899916	-0.399711246888357	0.264920245894559	0.0290933333333338	0.910577311070717
TMC5	-0.229616399588742	0.387822589005760	0.0228686466805794	0.97536722461972	-0.0693656666666665	0.754488794029141
PPP1R7	-0.229640825724512	0.387822589005760	-0.0958876319147979	0.872499931194687	-0.0852683333333322	0.684569287653569
MLLT4-AS1	-0.229743757645175	0.387438373594262	-0.0644774004424538	0.919619848669605	0.00499933333333396	0.99106990984423
PART1	-0.229780726544286	0.387314971342415	-0.0829232184143704	0.89362638370288	-0.383098666666666	0.0674510226934292
TNNC2	-0.229806793309006	0.387249820531523	-0.322804924245252	0.416073658452742	0.0173683333333339	0.955460918122607
C4BPA	-0.229831487220842	0.387192014212049	-0.00732120252595003	0.990873555460625	0.327723333333333	0.309395681555918
C2orf80	-0.229919051442894	0.386798598514444	0.0440526908277115	0.948520928173175	-0.184635666666666	0.344911970257145
LAMB3	-0.229996159007149	0.386609209106880	-0.298992868386343	0.469860024384247	0.599203333333334	0.0205866975799116
CD7	-0.230085761718648	0.386279287608371	-0.185310193687255	0.71487657566782	-0.0375846666666654	0.914256893620689
PIWIL2	-0.230225194640301	0.385884205352783	-0.00522429069645117	0.99251028338798	-0.177160666666667	0.596617523400524
LSM4	-0.23031449251757	0.385744829004273	0.156337113916715	0.768909201237015	-0.0733303333333323	0.75120129784636
AOAH	-0.230324330889938	0.385744829004273	-0.341256610034297	0.378059079534561	-0.0986730000000001	0.690641668678273
BZW1	-0.230334170411196	0.385744829004273	-0.00326465777435305	0.994730189431381	0.0896260000000025	0.674028958837018
HAGHL	-0.230432090760939	0.385543540117475	0.229959407977072	0.619254569407113	-0.109631666666666	0.614221661998915
ALDOB	-0.230636577866163	0.384885653409818	-0.187097137597827	0.709833349594307	0.0610100000000006	0.87279470391545
ICAM2	-0.230646514965657	0.384885653409818	-0.175246157675281	0.732740076365731	-0.150792666666667	0.571543418059249
MAGI2	-0.230657250046725	0.384885653409818	-0.245220818603939	0.585635671087268	0.243783666666668	0.193877143823627
MS4A4A	-0.230717214578111	0.384839693420646	-0.388307061397471	0.284913841222491	-0.0432439999999995	0.866266768093673
TPD52	-0.230820947743598	0.384583859689299	-0.256219663496996	0.564469720607515	-0.00452599999999941	0.988133186272453
CARS	-0.230861454658345	0.384442383417365	-0.457915769487971	0.166671273245692	-0.162341999999999	0.523453039510001
GNB3	-0.230965445085197	0.38403735829992	0.265714727734228	0.539878668275766	-0.097168666666666	0.767565307054335
ZC3H12C	-0.231122902076245	0.383635641192995	-0.205145529240411	0.673382949111104	0.0728640000000016	0.78197285216323
METTL22	-0.231138245910120	0.383635641192995	0.156836158920934	0.768401006769299	-0.0789746666666668	0.73097308185803
DEFB125	-0.231176873216409	0.383505792868717	0.243117270309893	0.592178030679976	-0.0262509999999996	0.920683589440809
OSCAR	-0.231186667269058	0.383505792868717	-0.129342600695529	0.814410035678712	-0.0743879999999996	0.774104133965233
KCNK12	-0.231197309052214	0.383505792868717	-0.165174597869208	0.752756950243373	-0.164720333333334	0.719909781429035
ASL	-0.231394045583191	0.382585921397521	-0.0938657372723044	0.875355472414883	-0.103024999999999	0.619881356855698
STEAP1B	-0.231409251885711	0.382585921397521	0.0183483002768996	0.979214953187634	0.213323	0.294676656181581
DOCK2	-0.23144914655294	0.382504187972498	-0.451450274171357	0.176663460992485	0.209123666666667	0.352897567720880
SLC7A14	-0.231454001950409	0.382504187972498	-0.073286243671263	0.90588212168674	0.0687618333333337	0.871724803785777
TYROBP	-0.23148093470701	0.382504187972498	-0.449057029699577	0.177860736739947	-0.112825666666667	0.6299281511525
PDSS2	-0.231530350526089	0.382416506067663	0.0338883052858397	0.960177797488544	0.0550453333333336	0.833941200090013
ACSL5	-0.231577208844391	0.382342620648132	-0.458883666509088	0.165076020821271	-0.0608843333333332	0.871242375260852
VKORC1	-0.231620759165666	0.382277154100897	0.0890128723088	0.883824851493414	0.00752833333333401	0.982523503616988
SHISA7	-0.231656436634662	0.382218423214532	-0.0367539594360828	0.956918184553224	-0.118072999999998	0.640143543029453

GABRA2	-0.231713205155609	0.381991388234254	0.00877818712239014	0.98946753270812	0.00756633333333376	0.982398683576954
ZMYND10	-0.231765655083234	0.381861323684531	0.0683826372188155	0.912232638501567	0.0166800000000008	0.953335531487028
LAMA3	-0.231774021604761	0.381861323684531	-0.0579040816002042	0.929611377711508	-0.0255443333333331	0.922854901626297
HLA-DMB	-0.231777595105452	0.381861323684531	-0.415833567286038	0.235080055809448	-0.0911093333333329	0.761335858444915
CD28	-0.231788814476846	0.381861323684531	0.118142468824053	0.838500126709059	-0.0691119999999993	0.820041994125866
TMEM37	-0.231813403096692	0.381861323684531	-0.100533611829118	0.866529683756364	-0.267090666666666	0.175763076550523
RPP25	-0.231864498986838	0.381781539217797	-0.135736389537890	0.806319865319553	0.0590780000000013	0.859719118838948
ODF2L	-0.231877163583287	0.381781539217797	-0.517875139963806	0.0911964988720938	-0.0245346666666657	0.947455054282258
MRPS30	-0.231927157885089	0.381671602684610	-0.0733667094759396	0.90588212168674	0.000893000000001231	0.99734800727507
FANCE	-0.231947333415550	0.381638745470306	-0.0684508336654477	0.912149839732916	0.061944	0.84207712482186
RPS6KA4	-0.231982886859496	0.381524383203159	0.151390110525754	0.777477357825446	0.00823733333333373	0.981237738522034
PTPRC	-0.232038922261196	0.381449710152119	-0.490050802994066	0.124999442942958	-0.00129733333333318	0.996390456305606
ERVFRD-1	-0.232123480948989	0.381075775115471	-0.0464209133229037	0.944856324173183	0.146255000000000	0.623903073849364
LMBRD1	-0.232154657063231	0.38104033097597	0.314371379897885	0.437553283968115	0.330645333333335	0.130729956218524
SWSAP1	-0.2321698506303	0.38104033097597	0.126148669450836	0.822164329801713	0.071374000000001	0.76648935876098
ATP6V0D1	-0.232312944003100	0.380368983440040	-0.0658178653054014	0.917339272986403	0.160864666666669	0.451408285415073
PLCH2	-0.232331963850100	0.380342357865905	0.235441922178583	0.60678764664537	-0.150105333333333	0.637426515050706
SERPINA1	-0.232357087073498	0.38028345581132	-0.466536573082377	0.152768530180247	-0.154536666666666	0.495283912463359
GNAI1	-0.232363574215592	0.38028345581132	0.0510785400814622	0.939432580299312	0.793619333333334	0.0139341065577702
PCCB	-0.232364330984912	0.38028345581132	-0.195331014973404	0.694439832288228	-0.152950333333332	0.432960172695455
RNASE3	-0.232367958536133	0.38028345581132	-0.101156071047014	0.866005093242444	0.0289283333333339	0.913360847453956
PIH1D2	-0.232425585138280	0.38028345581132	-0.182266010656079	0.719419078472232	0.0801156666666666	0.706892642645718
PTCHD1	-0.232546552443838	0.379799572546026	-0.0336458245025235	0.96061727828906	-0.0399499999999996	0.880427463662501
EXD1	-0.232579649975346	0.379698624027678	-0.116781742619838	0.841571096973212	-0.111172666666666	0.59851425430501
TIAM2	-0.232583079102993	0.379698624027678	0.163722836802334	0.754968203901817	0.308990333333334	0.216858796679299
CDH22	-0.232597367001235	0.379698624027678	-0.115335720980766	0.843755596233057	-0.0490503333333334	0.832650560777779
TBK1	-0.232659593037662	0.379646213373699	-0.18685625803334	0.710596654649373	0.272236333333334	0.153235579995233
ACVR1C	-0.232687474962794	0.379611757132558	-0.110285448051436	0.8502509043242	0.186875000000001	0.415770907577367
TMEM88	-0.232693133116974	0.379611757132558	-0.473166309914935	0.145443641689931	-0.0562236666666659	0.852969361639689
ZNF3	-0.232695393791784	0.379611757132558	-0.130139400331967	0.81240810100014	-0.0572126666666659	0.824548552404467
DRD2	-0.232779416011706	0.379611757132558	0.0795760678610401	0.89819077139711	-0.0625943333333325	0.804090240889797
OR7C2	-0.232811926318619	0.379611757132558	0.141522909191915	0.795705351419283	-0.099152666666666	0.74687011643745
GPR34	-0.233120283017118	0.378546096736491	-0.450417436849768	0.176663460992485	-0.0097076666666664	0.974484855799046
LEP	-0.233153016529812	0.37844741424104	-0.05313875511073	0.936712858731269	-0.126134333333333	0.592988797958646
POU3F3	-0.233236627147481	0.378302593708416	0.167929683827655	0.748119802053525	-0.0126496666666655	0.977997080364813
OAZ1	-0.233253010639564	0.378290265002155	-0.0817089925759192	0.89548747312664	0.177880000000001	0.349268687364205
WFIKKN2	-0.233318678011442	0.378017897332678	0.273565920050631	0.523325685448867	0.0679310000000012	0.866601971778042
PHF6	-0.23342538421516	0.377603372553530	-0.293601736395254	0.480614908214975	0.137124666666669	0.488602547609567
DUSP23	-0.233493235878824	0.377491595345758	0.0214464544286968	0.97536722461972	0.00231533333333435	0.994738356997797
C9orf24	-0.2335743325369	0.377262771452597	0.0585725034947407	0.928586335857223	-0.0918569999999994	0.757958218118621
PRSS16	-0.233587805721928	0.377262771452597	0.0770987775058247	0.901343076217132	0.0869080000000008	0.68135180147671
KLF10	-0.233877008443035	0.376446676267346	-0.218333921079774	0.642971191969613	-0.00983233333333265	0.978504228748436
ZFAT	-0.233910170494949	0.376446676267346	0.0687299340247998	0.912149839732916	0.0295273333333338	0.923019543204612
FAM181A	-0.233979362096346	0.376389557253839	0.145173515726969	0.788615482689992	-0.0783523333333328	0.824700707442131
TRIM61	-0.233985808522062	0.376389557253839	-0.217641604048264	0.644891652669682	0.108215666666667	0.817550597555287
PLEKHG3	-0.233995727400164	0.376389557253839	0.0342775492940668	0.959623239761825	0.144432666666667	0.503352066997863
HPS3	-0.234009792874148	0.376389557253839	-0.17952444067061	0.724770967840177	0.159376666666667	0.465948865963889
CDK5R1	-0.234094661578908	0.376079344104674	8.56921084537266e-05	0.999860038008296	-0.0858783333333327	0.710009054320066
RRAGC	-0.234165483961415	0.375855042321357	-0.305836773824088	0.457192231066199	0.0552113333333332	0.834140029892063
SLC7A11	-0.234238841091357	0.375681843708681	-0.185578891834503	0.714210278087368	0.379101333333334	0.0619237323392494
XRCC4	-0.234286928320284	0.375587076251692	-0.363590888689898	0.335364857752972	0.0619346666666666	0.789377975672973
IFNA17	-0.234428436166937	0.375139814730285	-0.0630551502942885	0.92160309476589	0.120813500000000	0.547723846565958
CHPF	-0.234435928190688	0.375139814730285	0.0670457991353466	0.914926078524511	0.0645806666666673	0.809409373518692
MRPL35	-0.234443077224848	0.375139814730285	-0.0351900839279567	0.958873757674185	0.0209083333333353	0.952460815972492
DNAH12	-0.234509714233479	0.374935176565478	-0.231450160010034	0.614876015201472	0.0177618888888899	0.95784037718285
SLC25A4	-0.23451485331285	0.374935176565478	-0.083136499444987	0.893287218356307	0.0240053333333341	0.928785692303482
PSMB4	-0.234581360101026	0.374707126912693	-0.0896071877797109	0.883196389963269	-0.0409966666666663	0.870622300341484
ABCA12	-0.234625360480763	0.374550211655564	-0.119069743168086	0.836828045978697	0.00754700000000039	0.98256318768706
PTPRN2	-0.23465312983862	0.37447853439455	-0.0266079375884636	0.970207612777188	-0.0799726666666662	0.733650645007746
ERP27	-0.234758869203701	0.374045043657601	-0.108900368772852	0.853371999394785	0.156085333333334	0.442294185443201
OPRK1	-0.234888822788169	0.373674453953311	-0.104201697836722	0.86124543980304	-0.0893216666666663	0.66866470442489
HAVCR2	-0.234925519637426	0.373674453953311	-0.41678117971461	0.234235464483992	-0.0930986666666665	0.66399337759607
YDJC	-0.235053392996561	0.373564616906327	-0.0414152775091923	0.952098348256047	-0.206257333333333	0.271568162967415
AICDA	-0.235065937545214	0.373564616906327	-0.392410365482811	0.279133352313495	-0.194105333333333	0.435157099583407
C1orf227	-0.23519718868809	0.373255756460079	-0.146373804234868	0.787103461358382	0.0114979999999999	0.969387309763954
C1orf213	-0.235222714039862	0.373253891391860	0.143813061551805	0.791963790519804	0.00138366666666729	0.996285985364494
SEMA4F	-0.235310748088891	0.373105774848295	-0.199797861572865	0.682777037002814	0.044949833333334	0.893856182973485
GCM2	-0.235365663973465	0.372966438620156	-0.0733112359331361	0.90588212168674	-0.0883646666666658	0.729527404290342
C16orf46	-0.235387379128274	0.372966438620156	-0.140004563468056	0.7976681602188	-0.0584133333333328	0.807883392282122
CATSPERB	-0.23540847075976	0.372966438620156	-0.0366504581583422	0.957104247386384	-0.0197736666666664	0.942298301774487
ACSM2A	-0.235439093236847	0.372966438620156	0.00521721511164214	0.99251028338798	-0.0139489999999992	0.97184501043952
SORT1	-0.235467448980264	0.372966438620156	-0.490854153964522	0.124057824658091	0.344745000000000	0.075243913539028
GMFG	-0.235482098025399	0.372966438620156	-0.387730759983017	0.286134842484356	-0.0403656666666664	0.873329245675404
N4BP3	-0.235491954389197	0.372966438620156	-0.0175328319018367	0.980496266838056	-0.118464333333333	0.671492528470281
GRHL3	-0.235584214051583	0.372808107799978	-0.138978448072193	0.79864602540281	-0.0962033333333331	0.659378397216567
GPR112	-0.235586036995059	0.372808107799978	0.130091590270788	0.812458158507233	-0.172875666666666	0.40610776759241
AAGAB	-0.235765877222778	0.372430391403263	-0.233025118411613	0.611711680668619	-0.0957216666666667	0.670071773006358
CRB1	-0.23579902381047	0.372373912065631	0.093099957045089	0.877130088568424	-0.0674986666666668	0.784812322947218
ANXA13	-0.235799259871462	0.372373912065631	0.142394722516237	0.794346532964905	0.0790580000000004	0.709454018799734
PLA2G1B	-0.235870104459571	0.372256657317942	0.0097961096612909	0.989090482697721	-0.246316000000001	0.209585689318078

SERINC3	-0.235870757465485	0.372256657317942	-0.287090484779504	0.494441582955552	-0.0751199999999992	0.731974410873657
HTR3C	-0.235887972732407	0.372256657317942	-0.00230164497677125	0.996326683062937	-0.114926000000000	0.60217016768875
VWA5A	-0.236004642349985	0.371998806336189	-0.360718004947407	0.34034906917442	0.165937000000001	0.614221661998915
DAP	-0.236023637296397	0.371973835910802	-0.246688082224850	0.581438939050387	-0.278956666666666	0.183344879272687
OIT3	-0.236060878284288	0.371891210945978	-0.0684562994457224	0.912149839732916	-0.00719333333333246	0.983159241812861
WIBG	-0.236071718960124	0.371891210945978	0.0837515317228483	0.892833228561937	-0.348378666666666	0.0748141981193211
SLC25A40	-0.236098194233758	0.371881510051436	0.079263570969291	0.898424765944956	0.196654666666666	0.452256892553908
CCNG1	-0.236147944237819	0.37184468103949	0.0417563834093654	0.951989245458556	0.317621333333336	0.120319574336004
RHCE	-0.236220026205350	0.371690972284344	-0.222278213916849	0.636309362271551	-0.0452943333333335	0.85075184108129
TMEM147	-0.236226279654851	0.371690972284344	0.0723655718985709	0.907556175729082	0.362467000000002	0.104464051521211
SEMA6A	-0.236271170510213	0.371572380017735	-0.138928028994887	0.798791960356084	-0.520055333333333	0.0852927646341535
RCAN3	-0.236282711291586	0.371572380017735	-0.367223428671058	0.327869177739765	0.161425333333334	0.633561720825521
PWWP2A	-0.236340084142115	0.371435442227637	-0.102832900813406	0.86257932725575	-0.0563619999999994	0.806863588982305
MTTP	-0.236349783330735	0.371435442227637	0.0457429259265898	0.94547820490174	0.0677810000000005	0.786973845403943
LOC153684	-0.236383971254957	0.371354967805647	-0.0431508955677304	0.950119302534786	0.213460333333334	0.392144513393944
CCL21	-0.236448962345585	0.371164067723717	-0.263292750092534	0.546035961835399	-0.0627656666666659	0.77740028876925
SPINT2	-0.236469954501725	0.371128852212289	-0.57116334998926	0.0443803345694495	-0.143255333333333	0.60627373290346
PIP5K1C	-0.236479573370378	0.371128852212289	0.0127065259111737	0.985214990982244	0.0245293333333333	0.937950350023697
C12orf52	-0.236484868319768	0.371128852212289	-0.0370202802839895	0.95664230789783	-0.152730666666666	0.450718526927142
HAAO	-0.236519487424276	0.371128852212289	-0.00564008590553927	0.99251028338798	-0.00701699999999953	0.987937064305561
PCED1A	-0.236558753492491	0.371128852212289	0.193417309201208	0.698131592467487	0.0629486666666673	0.77718596107678
MIR31HG	-0.236673711810945	0.370809540333996	-0.0432591835043811	0.949991344015323	0.0358096666666666	0.888476605628154
TUSC1	-0.236680289490484	0.370809540333996	0.153657808486212	0.773358453580566	0.0232393333333336	0.931612374241687
VRTN	-0.236683669305713	0.370809540333996	0.133470914728095	0.808933084408149	-0.0725543333333329	0.82283509793617
LLPH	-0.236744936246419	0.370735896319354	0.0408310832297801	0.952426531595115	-0.163224444444444	0.422571606196103
EPHA6	-0.236873280385131	0.370273712684461	-0.127194014918788	0.819932468027166	0.0497106666666672	0.843213673943937
CILP	-0.237010556997417	0.369720804677813	0.0818913696918561	0.89548747312664	-0.182713333333333	0.534910593395784
LGALS2	-0.237236181906098	0.368767126577385	-0.146557199458291	0.786490862211655	-0.0644956666666665	0.798344353938564
PNPT1	-0.237258336220310	0.368726137594062	-0.0956282054515446	0.8726818955265	-0.279789	0.206187117033303
TMED9	-0.237363611692394	0.368252010355658	-0.312243298625189	0.440385292108219	-0.0539653333333326	0.834476346642783
TMEM30B	-0.237456491703852	0.367842824242432	-0.0042912735299396	0.99405344216434	-0.157443333333333	0.584919645732877
ACTR3BP2	-0.237554725910162	0.367480429156396	0.272050813627858	0.527284725671175	0.0387886666666669	0.901080970962937
ZMYM3	-0.237764587672221	0.366538430122355	-0.324218770418708	0.413072828507872	0.0526780000000003	0.846917872248932
PPAPDC1B	-0.237778648247801	0.366538430122355	-0.174405554516382	0.734267769889068	-0.0674596666666666	0.766569862858904
LINC00311	-0.237880358341519	0.366160069262932	0.103435771489481	0.861943478503987	-0.172006999999999	0.426518032099216
IL25	-0.237887144303909	0.366160069262932	-0.0566450610132784	0.93176886339657	0.0532193333333336	0.839998150752089
LY9	-0.237889786831085	0.366160069262932	-0.0709567444826127	0.909400532983434	0.0447313333333335	0.896699926823174
SGK2	-0.237938001672921	0.366083469604504	0.120597889770949	0.833850618726547	-0.156933000000000	0.615722818909839
CDX1	-0.237966463435349	0.366083469604504	0.060640506091248	0.925413306065874	-0.0538889999999988	0.826961866961896
CLEC7A	-0.238221733645284	0.365428111204467	-0.379053026567066	0.304146578068964	0.0389390000000005	0.87279470391545
OBSCN	-0.238228325688661	0.365428111204467	0.348165709629634	0.361374443308161	-0.0996856666666654	0.629710931024195
PPP2R2C	-0.23831013796419	0.365217428127883	-0.331899677182445	0.399068746068573	0.00161166666666678	0.996155885952353
GCNT3	-0.238342015542527	0.36517576761336	-0.343046453431274	0.374823214518104	-0.0699773333333328	0.760700494137744
C7orf10	-0.238397406247122	0.364962996333434	-0.0606843226328307	0.925335786425382	-0.0837353333333325	0.721484595160786
RBKS	-0.238486880334593	0.364648008754115	-0.0633680307567691	0.920892398643354	-0.0436453333333327	0.861945621270498
PAQR9	-0.238509022467947	0.364607544098417	-0.256387630532180	0.564430426783556	-0.112662666666666	0.62979571408014
MIPEP	-0.238541267901507	0.364514792092047	-0.533279004881341	0.0759909410633651	-0.0897673333333327	0.765058274870877
HKDC1	-0.238614643180811	0.364283379693368	0.0792477998606516	0.898424765944956	0.000668666666666668	0.998351142601927
MMEL1	-0.238725809787402	0.363930769022377	0.079989126206452	0.897708122364667	-0.0922669999999993	0.741902389662432
PKIB	-0.238749905591313	0.363880296724168	-0.387721657539884	0.286134842484356	-0.0172459999999992	0.954736472265512
MYOF	-0.238861729120574	0.363557175748413	-0.482122811265429	0.135560525489292	0.441840000000001	0.0425296476131025
TYRP1	-0.238945556182513	0.363239702888745	-0.383058094119290	0.294467842756818	0.563871333333334	0.0351167468854759
SEPP1	-0.238949462502927	0.363239702888745	0.101809229095689	0.864656791615618	0.315638000000002	0.0973966522574197
PSME1	-0.238952150687107	0.363239702888745	-0.492869551428361	0.121594840868068	0.129854333333335	0.516217364739058
AVPI1	-0.238954649246229	0.363239702888745	0.0817229822347046	0.89548747312664	-0.0279476666666661	0.943336059867727
TNP2	-0.238978061345425	0.363239702888745	0.0875238844382651	0.886302037299497	-0.0280533333333333	0.922697964464038
TAS2R16	-0.239003343538518	0.363239702888745	-0.0777306104698904	0.900452756580846	-0.110195000000000	0.590841338570121
RGS3	-0.23905070273946	0.363239702888745	0.0587293561188505	0.928472484757714	-0.095371999999999	0.678271225356529
FNIP1	-0.239149133592754	0.363164267623624	-0.241252513090965	0.596019873098193	-0.0546253333333333	0.825979744677476
FAM98C	-0.239235731927532	0.362950803739833	0.00255448976703339	0.99580901762095	0.0793613333333351	0.743291289804212
AGGF1	-0.239273197883690	0.362882428524456	-0.399559432957191	0.265001036100484	0.125113000000001	0.536945695664816
PARP10	-0.239287999063216	0.362882428524456	0.273206741340237	0.524050233203661	0.0349896666666678	0.897669057202925
SLC22A15	-0.239374403903497	0.362656836296749	-0.154027463892952	0.772253934458676	-0.122316000000000	0.546219696447415
RAB2A	-0.239512077576933	0.362168979644315	-0.155107077357525	0.770668139243906	0.0333380000000009	0.902761873816516
L2HGDH	-0.239521321348472	0.362168979644315	-0.0681981500035904	0.912364243071055	-0.297518000000000	0.134732824217786
MSX2	-0.239560371468377	0.362068297212767	-0.0891555427709488	0.883460564717377	-0.157487333333333	0.427283471646461
PIM2	-0.239619933716285	0.361835320449884	-0.139730135754123	0.797957231657366	0.0257956666666676	0.931446587444396
PTGR2	-0.23964696664402	0.361804066880140	-0.138878588265838	0.79879462261805	-0.241395999999999	0.233528575266801
ZNRD1	-0.239824521066790	0.36122604231926	0.137692885256164	0.801934376886443	-0.0947708333333332	0.71626935665586
PDIK1L	-0.239923377000425	0.360865280290754	-0.278983518545065	0.511025440770733	0.101322000000001	0.676024903789639
GPR32	-0.239995734737821	0.360607836719231	-0.0163537853938129	0.981892085795004	-0.167580999999999	0.450479537895213
CUL1	-0.240043344329328	0.360470259332589	0.15770513660565	0.767770230680061	0.0500006666666671	0.832903171531715
ZNF582	-0.240060951157428	0.360453785232596	-0.085978144770644	0.888306347447303	0.083965000000001	0.775273488714107
FEM1C	-0.240061395359111	0.360453785232596	-0.0596506118155056	0.926534310457165	0.0784686666666666	0.725570231024154
ECE1	-0.24007503671326	0.360453785232596	0.077432096612985	0.90079793355475	0.213876333333335	0.329050968765465
SLC2A13	-0.240116035909444	0.36039277870744	0.160262291300850	0.762461161181997	0.331609333333334	0.112438115304648
IL32	-0.240287098595296	0.359587563569668	-0.316588857108087	0.432222606811314	0.188188666666667	0.558807324932864
BHLHE22	-0.240361231060373	0.359354870569428	-0.311809057203286	0.441314096241515	-0.116655000000000	0.625258262671142
IZUMO2	-0.240422501478795	0.359262287360129	-0.233119874929973	0.611659075898586	0.067939333333334	0.771597788088894
LONP1	-0.240527916862397	0.358795355680400	-0.00472320554934811	0.993222959527385	-0.214174333333333	0.311439437711061

SVIP	-0.240576368113723	0.358641842282902	-0.297337075878499	0.474135884848874	-0.0554706666666667	0.823699339501946
ITSN2	-0.240656984495211	0.358355369770552	-0.063457127306308	0.920855211745173	0.183217666666666	0.412227633479785
FITM2	-0.240730580972515	0.358126027795616	-0.197254611969331	0.689280772804818	-0.311149333333333	0.125231573477647
CA14	-0.240832082728495	0.357914958831651	-0.229103356681187	0.62075895683145	-0.0221953333333331	0.942092082409632
ASB14	-0.240901316545829	0.357914958831651	0.0182930271822025	0.979327298872732	0.124811666666667	0.607009506431467
KLHL7	-0.240995592144298	0.357700381940505	-0.0866662862957628	0.887493600482322	0.031716666666668	0.915761046438965
FCRLB	-0.241038040281450	0.357586157190534	0.0480940804460552	0.94314236584312	-0.0871713333333327	0.74511187686951
SHBG	-0.241039644310047	0.357586157190534	0.162746992323390	0.757417321756424	0.134690666666667	0.670113640649168
IGIP	-0.241133010970054	0.357321874764282	-0.209700026842751	0.663785844870086	-0.242979666666666	0.199107820683444
RNASE7	-0.241158187439972	0.357321874764282	0.0843225380015682	0.892396784625427	-0.0374163333333332	0.893856182973485
TMEM163	-0.241203936467634	0.357180794602653	0.498939829706027	0.114105676690072	-0.210679666666666	0.33472722031995
CACNG2	-0.241250552152366	0.357016256624952	-0.00089703977368931	0.998745415362821	-0.0827983333333326	0.731974410873657
EYA1	-0.241348706022806	0.356662179089221	0.0608946021966871	0.92506536457632	-0.0198633333333326	0.943645170365395
TPST1	-0.241376714231921	0.35659291349175	-0.477134416205974	0.141885297989556	0.198857000000001	0.336610038325944
IDH3G	-0.241411841122802	0.356487281568381	-0.406133048919978	0.254041953733213	0.0150186666666672	0.95784037718285
TREML1	-0.241438040929800	0.356427291717585	-0.195459665992947	0.694043352975239	-0.170698333333333	0.535492155031224
FAM160A2	-0.241474914852772	0.356386685633928	-0.405199427908105	0.25480455106032	-0.106459	0.604966458785844
ELMO3	-0.241595938694570	0.355990169723410	0.311629325356825	0.442009587230904	-0.164770666666666	0.541939010010167
CCDC8	-0.241602458840231	0.355990169723410	-0.221069421113912	0.638286782439236	0.0259163333333347	0.929594956567283
ADCYAP1	-0.24166197442424	0.355874669345157	-0.305851308117031	0.457192231066199	-0.100313333333334	0.809321475119642
TTC7A	-0.241668773030146	0.355874669345157	-0.204773197130079	0.673728362275223	0.139719666666668	0.575378149225683
GRASP	-0.241680858286852	0.355874669345157	0.043563720048092	0.94933169799234	-0.195964000000000	0.344576368488222
GAS8	-0.241732529771113	0.355872245015112	-0.162058159432499	0.75854722544558	-0.121010999999999	0.552316152798775
EXOC6B	-0.241864750414377	0.355283174240538	0.0939236453775198	0.875355472414883	-0.0819573333333325	0.734228369215232
TOR2A	-0.241915270086214	0.355173260515214	0.089999375586087	0.882422779091356	0.115465666666667	0.578689664169438
PSMB3	-0.241945913237012	0.355164770316389	-0.385950386942582	0.289470192870154	-0.123323333333332	0.60814016771842
KLHL12	-0.241999399562359	0.355009105312798	-0.117854116661947	0.838686136084666	0.145968333333334	0.535171605667144
ZDHHC13	-0.242003518460357	0.355009105312798	-0.172396594867008	0.738537617651621	0.196179000000001	0.306281656395861
XBP1	-0.242141578019536	0.354536764946258	-0.123233881805125	0.82767952783856	-0.0568823333333323	0.807296839573994
ITPK1	-0.242200620058109	0.35441645884311	-0.242521636903598	0.593306823292819	-0.0323839999999999	0.911600329626215
VTI1B	-0.24220758781888	0.35441645884311	-0.34585108390488	0.366612790157282	0.268049666666667	0.270369355293981
NSMAF	-0.242218944724663	0.35441645884311	-0.0966105896169613	0.870857630743202	0.285643666666667	0.268859860310995
GPR126	-0.242226993145766	0.35441645884311	-0.265738996447213	0.539878668275766	0.210793666666667	0.307927332709227
SYT5	-0.24223223646467	0.35441645884311	0.109013741669539	0.853032135925272	-0.071426666666667	0.746116817092925
E2F5	-0.242271373078955	0.35441645884311	-0.071304458485466	0.90912843135453	0.266869333333334	0.247218027506970
OR7E24	-0.242287906221567	0.35441645884311	-0.0239052740901481	0.97479225206569	0.170133000000001	0.399391776486682
EMC6	-0.242325749308236	0.35441645884311	0.206566882076114	0.67111196781183	-0.0205526666666661	0.938640008704917
KCNQ1	-0.242432773250940	0.354310395848755	-0.142241546631020	0.79450771779567	-0.0706356666666657	0.744787682877495
SUOX	-0.242478712603507	0.35415036719107	0.0104598222216295	0.987849393233007	-0.0338729999999991	0.90945379489823
SPATA4	-0.242479213520167	0.35415036719107	-0.147696498682418	0.78459852198821	-0.0723316666666666	0.742124582656713
LOC79015	-0.242479472670242	0.35415036719107	-0.410968287655708	0.244526229723610	-0.127190666666666	0.543819286485342
EFHB	-0.242615993084355	0.353673330903508	-0.274391223235409	0.52071143082909	-0.00705999999999959	0.983690244124512
PLA2G15	-0.242640949655052	0.353620302630615	-0.0927735111103639	0.877797216649453	0.101542666666668	0.637530761419601
GPR37	-0.242663388502326	0.353620302630615	-0.348084408114073	0.361374443308161	-0.282746333333333	0.582761730556127
ACPT	-0.242702704798730	0.353602094404790	0.193352997507044	0.698337791293402	-0.136334333333332	0.577786572116335
KNDC1	-0.242886349101856	0.35286994362405	0.281747647249839	0.506335763951991	-0.0796846666666666	0.754488794029141
CNGA4	-0.242897051945057	0.35286994362405	0.0759635400792681	0.903510413986113	-0.128339333333333	0.709458407711009
FAM206A	-0.242933196569695	0.352799684690769	-0.129074227674602	0.815171037361366	0.134074000000001	0.691937554141542
TBXAS1	-0.243044827895786	0.352380369460166	-0.312830903503613	0.439050300925307	0.067113666666667	0.817735562413299
ARMC10	-0.243049397747758	0.352380369460166	-0.106031623153717	0.858902068936183	0.121812000000001	0.631961600239811
RASSF5	-0.243128503900550	0.352103297587021	-0.343531899453487	0.373731310823846	-0.342488000000001	0.254350940121719
TBX22	-0.243265958406697 5	0.35160904058329	-0.489557502809564	0.125607960746097	0.183716000000000	0.479067209456976
SIGLEC8	-0.243282691166701	0.35160904058329	0.0891776641871833	0.883460564717377	-0.191988666666666	0.320592704550458
RHBDF2	-0.243366478945014	0.351339222764694	-0.244033925525281	0.589285232493116	-0.127855000000000	0.675449330308211
ADH6	-0.243396395191836	0.35126327317345	-0.125233105683137	0.824230922676958	-0.0328259999999991	0.896403646226360
SRM	-0.243412768417516	0.35126327317345	-0.272347553859512	0.52636032720919	0.00875366666666806	0.980770032498656
ZMYM6	-0.243435577847831	0.35126327317345	-0.144918010142429	0.78939605015755	-0.0445083333333325	0.855321176509099
RGS8	-0.243458108949886	0.351243978694924	-0.295834112032983	0.47761182409076	-0.00356933333333287	0.991129100069248
DPM1	-0.243620764288726	0.350567611677208	-0.303067775569095	0.462105017205825	-0.0413346666666654	0.870363204357393
TPK1	-0.243631691325246	0.350567611677208	-0.483315817628634	0.135421395277788	0.074534333333334	0.816134952712854
RNF10	-0.243660831999056	0.350512334141964	-0.0752049169261171	0.904173044514532	-0.109900999999999	0.625258262671142
RHOF	-0.243779809314732	0.349984100558812	0.171107882264942	0.740062065190296	-0.0644786666666669	0.847572035799702
GRM6	-0.243841499055056	0.349819579757587	-0.0755191891156606	0.903537954146139	-0.156916333333333	0.611690992275966
C14orf1	-0.243901319182086	0.349590908482742	0.254121751094293	0.567694846153007	-0.00735899999999952	0.987937064305561
ENY2	-0.243964721645069	0.349344249125509	-0.052776633402134	0.937235903571983	0.104517666666668	0.633166039956913
SLC17A4	-0.244012792316883	0.34917511552447	-0.177678813326491	0.728276800708888	0.0145476666666671	0.969387309763954
PPIH	-0.244124597531044	0.348905152102474	-0.200927598382694	0.68016888532461	-0.0214436666666654	0.945904449997854
USP43	-0.244207516092611	0.348629752009377	-0.0601165710660018	0.925649366494154	-0.153719666666666	0.464637455466939
HIP1	-0.244217617097672	0.348629752009377	-0.163670023820885	0.754968203901817	-0.199764999999999	0.349516958620214
ATMIN	-0.244261485851982	0.348582516255570	-0.283811680161067	0.501265029475938	0.094771333333335	0.649118055813479
ABCA13	-0.24428573711475	0.34853380412794	-0.137837199807960	0.801736842296971	-0.152450666666666	0.682334554712472
ZNF276	-0.24433634550141	0.348498833946937	-0.0139859442492526	0.983565756891305	-0.203493999999999	0.313220411537404
GIMAP7	-0.244354973675544	0.348479140739777	-0.366455416526917	0.328499662474147	-0.00152633333333294	0.99628178244892
SEMG1	-0.244363297238419	0.348479140739777	-0.386245102940905	0.289351981332176	0.123702666666667	0.5530742296054
FBXW5	-0.24437341468563	0.348479140739777	-0.244013020155811	0.589294998673326	-0.0444623333333324	0.85994094150432
IGF2R	-0.244405026118648	0.348479140739777	-0.219037854422761	0.641856878959206	0.389595666666666	0.0689724571534153
WRAP73	-0.24449177881935	0.348231270527001	-0.429452147455428	0.209103669076588	0.143977333333335	0.496323393705678
LOC151009	-0.24451579143781	0.348183889285189	-0.150367835203210	0.779266896045314	0.155736666666667	0.456625567004912
MCOLN1	-0.244526777282096	0.348183889285189	0.0446146391957484	0.947920610570169	-0.0546216666666657	0.813306588489053
C19orf33	-0.244539214843745	0.348183889285189	-0.0470338962419776	0.944081286115546	-0.0840199999999989	0.750936504909256

KIAA0125	-0.244553548909366	0.348183889285189	-0.0739112738202294	0.905506084101768	-0.104338333333333	0.612891410601889
GFOD2	-0.24457986601945	0.348155735953097	0.0150503318312864	0.982921609770846	-0.121299666666665	0.636490807097692
COA1	-0.244616138552107	0.348142522920088	-0.325760490772142	0.409206882509236	-0.0311766666666655	0.90232248061402
C1orf228	-0.244635138163489	0.348142522920088	-0.116651720955345	0.841809730226979	0.323493000000001	0.0905524993267201
PENK	-0.244744245176437	0.347917238689666	0.135213281383115	0.807470535721699	-0.203197333333333	0.376020235114735
FAM159A	-0.244787871659502	0.347771107258147	-0.0392567517353679	0.953905313456582	0.090676666666667	0.789228734235839
CACNG5	-0.244865642921785	0.34752675928804	0.0963429393352533	0.87156820165807	-0.207027999999999	0.364396238839714
SLC35A2	-0.244915820397148	0.347347748691911	-0.373458184504170	0.315855731800715	0.0335733333333344	0.896186784012643
MRVI1	-0.244974397356996	0.347200286343060	-0.358597407417357	0.343101042014771	-0.154520666666667	0.5488879250835
WBSCR22	-0.244979371697455	0.347200286343060	-0.323816084857054	0.414201624246883	-0.130444	0.52286033069649
KPNA4	-0.245004785001459	0.34719485407983	-0.156197840840651	0.768909201237015	0.133299333333334	0.536320838519271
SPERT	-0.245035498122934	0.347113991513429	0.163834510666098	0.754916750929288	0.0570403333333335	0.827433678445379
CEMP1	-0.245075157060832	0.347061930814469	0.235845573638644	0.606422487682006	-0.112171000000000	0.670510815115172
HTR1D	-0.245095805204475	0.347031794328994	0.0904863644488578	0.881794677754899	0.0429853333333333	0.877940692255388
BTC	-0.245156098923093	0.346986700150183	0.000867035432066073	0.998745415362821	-0.0321636666666666	0.90945379489823
CROCC	-0.245209840191946	0.346886591253061	0.0895154197965997	0.883258404969667	-0.183185666666666	0.425882414194225
NDUFB11	-0.245235426203853	0.346886591253061	-0.314365273160541	0.437553283968115	0.0277876666666678	0.91148338318481
B3GNT1	-0.245245376340712	0.346886591253061	-0.148160681591555	0.784080588385358	0.222695666666667	0.238530206826239
LOC401320	-0.245500471183996	0.346384505654818	-0.323530098207480	0.414288146732192	-0.00757633333333293	0.985021106767112
LINC00529	-0.245511469624522	0.346384505654818	0.23984070735603	0.598134271398487	-0.0824903333333333	0.705543977023539
ZNF707	-0.245517757123958	0.346384505654818	0.334524445205828	0.394347836736941	-0.0887763333333322	0.742124582656713
ELSPBP1	-0.245550109154776	0.346371419340077	-0.253383192507562	0.567705016149998	0.415111	0.154631928204474
ABCB8	-0.245668699769560	0.346092169442257	-0.00941422481202125	0.98909166696601	-0.126133666666666	0.566937124812328
S1PR4	-0.245681621010689	0.346092169442257	0.16582182828432	0.751933348574587	0.00627333333333424	0.987937064305561
LYZL6	-0.245692636501649	0.346092169442257	0.134853065702411	0.807867938002244	0.0107810000000005	0.973686596735315
GSTA4	-0.245719693434529	0.346092169442257	0.0648500515302171	0.918590305362052	0.447258666666668	0.0619126099646093
SPG20OS	-0.245721975158423	0.346092169442257	0.0747406090008008	0.904466414650249	0.0240743333333338	0.928295508521289
H6PD	-0.24579920069148	0.346081222403933	-0.190023259562300	0.704348376269734	0.109824333333334	0.664067525814732
IKBKB	-0.245810793419318	0.346081222403933	-0.335979604687351	0.389712931761332	0.0615576666666662	0.788201431210286
NDRG4	-0.245831895121775	0.346065073086972	-0.142990558921825	0.793357892012091	-0.0179176666666662	0.967256128104685
RUNDC3A	-0.245853227179389	0.346031921527086	-0.0649214815553683	0.918523850969451	0.0434636666666673	0.907374738017166
SYCP1	-0.245894695003959	0.345897432083958	-0.146756042842844	0.786187758946602	-0.0606430000000002	0.798344353938564
KIAA0895	-0.245905021724406	0.345897432083958	-0.00327590399911907	0.994730189431381	0.307405000000002	0.131549958663763
NEK7	-0.245925020600467	0.345893289529067	-0.0979315073516332	0.868852648210739	0.522964000000001	0.0267950289682554
CEPT1	-0.245999749131988	0.345591518908240	-0.0501978202319658	0.941133149988298	0.0323196666666670	0.910190419163007
NDUFB10	-0.246072833470058	0.345446587934248	-0.238902173667791	0.600356188404063	0.0081970000000003	0.982375192575954
UBL4A	-0.246082905319926	0.345446587934248	-0.449942375592791	0.177025296973857	-0.311690333333333	0.347225394893919
DCTPP1	-0.246201306734213	0.345023351481735	-0.148956495421743	0.783041584899534	-0.236215333333333	0.234508116088724
DIAPH1	-0.246226076060527	0.344973072548688	-0.0516056594149345	0.93852470081792	-0.0588706666666664	0.789377975672973
FAM170B	-0.246228936402185	0.344973072548688	0.202818616044788	0.677604697677494	-0.0160109999999988	0.967256128104685
LRRC48	-0.246362468178658	0.344510525212809	0.0239383031100656	0.974721039716014	-0.185781	0.517547647029412
PHF7	-0.246374631649522	0.344510525212809	-0.0761828915661836	0.903296922694955	0.170562333333335	0.52333876394493
GFPT2	-0.246392366776282	0.344508722449486	-0.0530684670382673	0.936712858731269	0.0195146666666672	0.94690965769901
PARP11	-0.246461526534832	0.344373033589398	0.0706702687347835	0.910001879770153	-0.0403479999999985	0.903864363916236
SRL	-0.246528320239891	0.34419715955947	0.110800666010043	0.849357727620213	-0.089258	0.811401072147441
IL5RA	-0.246613859147175	0.344064700597176	-0.00048076850819381	0.999424151270175	-0.0143476666666665	0.97184501043952
CCDC78	-0.246652340461431	0.34394583572351	0.270675349228955	0.529418563170514	-0.0987019999999992	0.643325492871585
PGAM5	-0.246689059221574	0.343900341644868	-0.310275431851313	0.446172472201571	-0.245883333333332	0.229591548351276
SEMA3E	-0.246903084290790	0.343282177141416	-0.153242903116166	0.774029578907844	0.0386743333333337	0.900467460403227
BEND6	-0.247035479068079	0.342778295450497	-0.0833870537258109	0.892833228561937	0.375975000000001	0.0944736471523537
LINC00116	-0.247083732235689	0.342674058785343	-0.0765159488248099	0.902624771576157	0.233102	0.4261850174154
SCTR	-0.247284499374451	0.342188411035889	0.202023821163863	0.680054545720343	0.0246340000000005	0.967879244181712
APOOL	-0.247430365430673	0.341681511952723	-0.537582217314335	0.072332911210698	0.067223333333335	0.759434553759299
ARHGEF19	-0.247459426861565	0.341610381450793	-0.254811275823087	0.566909791913503	-0.0924793333333336	0.73168347388773
FGFR1	-0.247475460470496	0.341610381450793	-0.147471105848056	0.785497496933202	-0.399877666666665	0.0469041914734957
ACSF2	-0.247536310734336	0.34144853874236	0.103069091021451	0.862488288780321	-0.0930103333333322	0.682815224992534
MYPOP	-0.247553136935488	0.341438581887129	-0.00666025052663646	0.991309497300929	0.00907433333333427	0.981191596773742
ASB8	-0.247575221189448	0.341402372545998	-0.220081691526179	0.64064333199733	-0.0185560000000006	0.945904449997854
SPATA2	-0.247596701364331	0.341369192336469	-0.0806259700654261	0.896754372944873	0.0721090000000004	0.746866891197215
RGMB	-0.247640706466425	0.341223534558064	-0.302821656276032	0.462105017205825	-0.177382333333333	0.499554381454891
EFTUD1	-0.247697651003820	0.341076741697524	-0.688914416728235	0.00652810604027768	0.00324800000000013	0.993516307401388
C11orf86	-0.247731445534926	0.341076741697524	0.156216746396105	0.768909201237015	-0.0674156666666661	0.760700494137744
TMPRSS5	-0.2477512350957	0.341076741697524	-0.0819039705811179	0.89548747312664	-0.0280833333333331	0.919420614501031
ARHGDIB	-0.247826085919525	0.340816913594396	-0.381100524158773	0.298110142491852	0.127853333333334	0.631961600239811
TMEM79	-0.247852508322522	0.340759184118839	0.151284287968846	0.777604486471648	0.00117666666666651	0.996826292964745
KIAA0930	-0.247863654719616	0.340759184118839	-0.0986672480620293	0.868080433714765	0.0042040000000005	0.98870044758797
C1RL-AS1	-0.248064195326619	0.339999972903208	0.0161157825156309	0.982057247916055	0.0247370000000007	0.937545603686416
TMEM41A	-0.248069364772741	0.339999972903208	0.142794232841777	0.794004622916083	0.144222000000000	0.501679403024387
AQP11	-0.248090668240074	0.339999972903208	-0.20677227888889	0.670360323581237	0.197821333333334	0.450418928637380
TMEM179	-0.248109014753561	0.339999972903208	0.377734343471779	0.306202811220100	-0.0529083333333333	0.81742145099016
PDZD11	-0.248213429678707	0.339886979722670	-0.549472842345568	0.0608648191629941	0.0171023333333335	0.955460918122607
AP3S1	-0.248246210751649	0.339886979722670	0.0189653784222640	0.97832242527852	0.00676900000000105	0.983159241812861
RPL39L	-0.248248586792982	0.339886979722670	-0.0860188704632848	0.888306347447303	0.237757999999999	0.278320107413092
FGB	-0.248264298173757	0.339886979722670	-0.169896290130420	0.743328907676018	0.0807220000000003	0.728054565398526
CHST13	-0.248295156223768	0.339886979722670	0.008020209072363	0.990405674923721	0.0418966666666666	0.91143191097679
CCND2	-0.248326769244021	0.339886979722670	0.0121463507087419	0.985739212424168	-0.958597	0.0326806169766676
STK3	-0.248401916658985	0.339886979722670	-0.283352420016802	0.502129505146473	0.274682666666667	0.158095716859654
CTU2	-0.248425084007996	0.339886979722670	0.133734601705706	0.808879359838378	-0.104078999999999	0.656612455150282
P2RY1	-0.248460300310918	0.339806470235979	-0.240271464661807	0.597862631330416	0.0378456666666675	0.88683920165247
SPAM1	-0.248508343550410	0.339715695089225	0.119791869262624	0.8354985445494	-0.140872666666666	0.551352991056569

DOK1	-0.248527583504726	0.339694165148744	0.149293834199589	0.782416617297835	0.0183343333333336	0.949295980201336
DEFB114	-0.248551132338489	0.339651165457778	0.126197684530163	0.822164329801713	-0.00444666666666663	0.990009919740954
MYO6	-0.248690211153515	0.339219876081509	0.0572438288587807	0.93071299016553	-0.022826333333333	0.955396570818642
PCGF1	-0.248730313586306	0.33913026052092	-0.102001722875372	0.864616357134757	-0.0627236666666662	0.777277975739661
PTGES2	-0.248844581909291	0.338751898114942	0.154460745804478	0.771310656070265	-0.159501333333332	0.435815094785787
GFER	-0.248849734217579	0.338751898114942	0.296814079667801	0.475919488007743	0.135592000000001	0.559991181594454
HSPA4L	-0.249003393981547	0.338407008998992	-0.289596651111803	0.488870852256136	-0.710724999999999	0.0105638761565426
PNPLA2	-0.249008571625618	0.338407008998992	0.164081340993147	0.754243267462143	0.116977666666668	0.594026307591911
PSMB7	-0.249136358515164	0.338017532217371	-0.0094898433419428	0.98909166696601	-0.107403333333333	0.600146208272329
REEP3	-0.249147426734973	0.338017532217371	-0.075938207910598	0.903510413986113	0.398160500000002	0.048170505761132
IFIH1	-0.249253904440519	0.337715240304143	-0.474310874950645	0.144263399197967	0.276147666666667	0.216926184391852
ZNF542	-0.249276649251434	0.337676564899158	-0.197690571825682	0.688640225556359	0.172952000000001	0.436942928497947
TM4SF4	-0.249311240518985	0.337579053938874	-0.431561760659318	0.205291718017247	0.660912333333334	0.0838858621204292
TEX35	-0.249311518485792	0.337579053938874	0.0136638757490363	0.984069404551287	-0.0850449999999998	0.767565307054335
FASTKD3	-0.249332547653291	0.337579053938874	-0.516851054958156	0.0919553206911016	-0.0065599999999984	0.98670424971527
PAX4	-0.249466045662076	0.337171770755572	-0.164649218358888	0.754105947879839	0.167721666666668	0.518244955194698
PGM2	-0.249484532958454	0.337164520660385	-0.228607797119646	0.622796068559319	0.492826333333334	0.183344879272687
GINS3	-0.249523263446802	0.337046612204397	-0.141277465110702	0.796285336930078	0.0897333333333331	0.682390613806982
GPR39	-0.249532234893465	0.337046612204397	0.0671671813124128	0.914700759157169	-0.0315823333333327	0.94622978991441
KCNMB1	-0.249603552189484	0.336796867590402	-0.257091125601731	0.563116080558022	-0.176250000000000	0.426484252681993
CPB2	-0.249633614154034	0.336722054288833	-0.248801791335796	0.576871285544251	0.00870333333333375	0.980779858267938
GADD45A	-0.249654187914648	0.336694316130425	-0.112715327625379	0.847051776433405	0.271027000000000	0.162661087494939
DPEP3	-0.249748429384666	0.336449916668767	-0.32649878756104	0.409206882509236	-0.0902716666666664	0.783096519994904
TMPRSS13	-0.249786561867667	0.336335187038708	0.156321965324159	0.768909201237015	-0.0137560000000000	0.96162571048779
ANXA2P3	-0.249839553257196	0.336158521554701	-0.193644950329989	0.69772764496956	-0.205288333333333	0.451746572873908
MRPL27	-0.249851309432063	0.336158521554701	-0.0123264515209832	0.985386024673793	-0.0696933333333326	0.755697432495201
SNTA1	-0.249878100640819	0.336158521554701	0.0085573659864203	0.989612051419321	0.00514666666666813	0.987937064305561
FAM151A	-0.249892576253137	0.336158521554701	0.200353174884323	0.681033026144457	-0.0527149999999995	0.816493365052524
IL18	-0.249962867022451	0.336158521554701	-0.232926743046854	0.611711680668619	0.206453666666667	0.443378989641463
LOC643837	-0.250100359495329	0.335672385733047	-0.403158456694222	0.258453301425503	0.251338000000002	0.387687154733619
LY6D	-0.250113666774151	0.335672385733047	0.0757693034432957	0.903510413986113	-0.104529999999999	0.616036866881905
MAGEB18	-0.250168739637373	0.335482510086623	-0.0412969257145148	0.952098348256047	0.0448733333333339	0.856854911614454
POLR2J2	-0.250236865844335	0.335293988432227	0.122663431996576	0.828930912293926	-0.0377043333333326	0.884789546675685
MEF2C	-0.250244539648673	0.335293988432227	-0.09418576116635	0.874945765417599	-0.230659333333333	0.235770973926881
ELTD1	-0.250400088576445	0.334858177672006	-0.156464538954344	0.768909201237015	-0.0266116666666661	0.927422712012045
FBXL18	-0.250411716546444	0.334858177672006	-0.0150172635903906	0.982921609770846	0.135584333333334	0.556248360059405
RNF208	-0.250416074513162	0.334858177672006	-0.163306020932964	0.755822838510534	-0.101088666666666	0.651792448627654
DUOX2	-0.250457119728575	0.334858177672006	-0.0267291992379657	0.970207612777188	0.103341333333334	0.616961986237355
C5orf45	-0.250570024774012	0.334389743497963	0.234257304461099	0.608813237170423	-0.0563543333333334	0.804755486961935
PSMD13	-0.250671880393667	0.334035231961692	-0.336341188692541	0.389372549180413	-0.181157999999999	0.409270914061388
AP1S3	-0.250677492585189	0.334035231961692	-0.0935975060624886	0.875932944246867	-0.0924989999999997	0.725237788753458
CEACAM1	-0.250683684619889	0.334035231961692	-0.127457368135701	0.81893660870295	0.493626666666667	0.0899297181257792
MFAP3L	-0.250739249636070	0.334035231961692	-0.301584570506842	0.463432085368295	0.141411666666667	0.63263903123177
DMXL2	-0.250749057696642	0.334035231961692	-0.307296261486117	0.453005595044643	0.059841333333334	0.813694870801638
LUZP4	-0.250807135059393	0.333887486374327	0.0139593794575642	0.983626896754915	-0.0415106666666663	0.866671423175521
ASXL3	-0.250815393209657	0.333887486374327	-0.160032822412217	0.762902034786947	-0.0635156666666666	0.789149377548591
ASZ1	-0.250922451437093	0.333607110215859	-0.0355677169976168	0.958689318480951	0.0875553333333338	0.69669232122609
ARID3A	-0.250945179027325	0.333577797227537	-0.147102013602657	0.785734908309155	-0.0259329999999995	0.925603409622752
CRYAA	-0.250964774254314	0.333555449348658	0.146674295865776	0.786187758946602	-0.169297333333333	0.493413216077427
HMGCS2	-0.251056837749048	0.333175653847719	-0.241462331574502	0.595717891213491	0.0851840000000003	0.805201268688175
SLC15A4	-0.251103974545957	0.33301755680607	-0.350189031418294	0.358738646007386	0.146814666666667	0.583708661347349
FADS3	-0.251129203977457	0.33301755680607	-0.244691315131891	0.587653287154633	-0.163524333333333	0.443072320689246
SLC35A4	-0.251137329135369	0.33301755680607	-0.201371359770373	0.680054545720343	-0.117190666666666	0.58051867264498
C3orf22	-0.251154067832014	0.33301755680607	0.162631861348048	0.757524249707797	-0.0108696666666666	0.97097048443105
SH3BGR	-0.251233257502715	0.332900488993039	-0.476700380348	0.142080890079208	0.0924786666666675	0.78197285216323
CDC5L	-0.251258457303002	0.332850635143261	-0.289800985841866	0.488870852256136	0.183005333333334	0.389117246104976
PDPK1	-0.251300401333106	0.332815045497043	-0.0998121460726126	0.867558388686851	-0.113351666666665	0.675687379893745
DEFB119	-0.251337513521181	0.33277931959564	0.00514769592306643	0.99251028338798	0.0208813333333334	0.95787549045386
EGLN3	-0.251341005624094	0.33277931959564	0.111263029957598	0.849115053536469	-0.0836806666666656	0.712601873916151
S100P	-0.251354739427631	0.33277931959564	-0.130218728521851	0.812271254601992	0.0305590000000003	0.911129648924728
FAM86A	-0.2513605009797	0.33277931959564	-0.201397301211014	0.680054545720343	-0.0950513333333324	0.72947339075282
NAPA	-0.251367398655402	0.33277931959564	-0.254987575274525	0.566577861267331	-0.168967999999999	0.537186705207113
CPA1	-0.251404694251719	0.33277931959564	0.168583867605797	0.746758286599055	-0.00934599999999985	0.98147292539484
F3	-0.251405253402739	0.33277931959564	-0.270367128654762	0.529999252995877	-0.0609579999999988	0.787768761656041
RBM18	-0.251448926508731	0.33277931959564	-0.194175909984615	0.696754707517212	-0.0922466666666652	0.666143393594486
FKBP5	-0.251504268968788	0.332681298657975	-0.237057698944754	0.603539900977931	0.146880000000001	0.459702144856649
HERPUD1	-0.251571906704080	0.332448310749887	-0.214553997328803	0.652218036941415	-0.322786666666666	0.116223919619021
SLC34A2	-0.251629887771863	0.33228579679387	-0.323602361971842	0.414288146732192	0.0916020000000006	0.670113640649168
LPIN2	-0.251634789856651	0.33228579679387	-0.137623145737927	0.801951364081469	0.280632333333335	0.172571369175474
LINGO2	-0.251642109608070	0.33228579679387	-0.0710964913235011	0.90935479990339	0.0413283333333333	0.890094615232462
CAMKK1	-0.251700262810258	0.33216270263134	-0.0444708424379284	0.948327980852394	-0.310412999999999	0.220405584107617
G0S2	-0.251807663109354	0.331708422368584	-0.351833118359099	0.354413251589651	-0.0660809999999996	0.780732284998362
ENO3	-0.251870310157222	0.331474656026083	0.157259448442215	0.767897349801793	0.0274520000000001	0.915761046438965
AZIN1	-0.252003300860291	0.331044020491299	-0.00045718320866690	0.999468814267431	0.122080000000001	0.541939010010167
CYSLTR2	-0.252052229565766	0.330908476137250	-0.244407309985485	0.58816484023304	0.136747000000000	0.692270762979636
SLC9A9	-0.252087700887098	0.330852457383852	-0.41538900239966	0.236224795296402	-0.086200666666666	0.702762636573042
IL22RA2	-0.252101121881750	0.330852457383852	-0.186164650553987	0.712609598901976	0.0372390000000005	0.879201876928015
ATG12	-0.252171001543644	0.330667865584114	-0.217200552229455	0.646495351690352	0.0839213333333332	0.716122332067559
PAPSS2	-0.252445112799330	0.329618944348487	0.168718914044039	0.746469057428344	0.852243333333334	0.0126478660661103
UCHL1	-0.252483047992472	0.329531831344002	-0.395075285651381	0.272692742937895	0.122601666666668	0.546219696447415

KIR2DL4	-0.252487522760234	0.329531831344002	-0.0556555367364346	0.933409139126562	0.141514833333334	0.473543693383828
PNLDC1	-0.252633050729176	0.329260849668460	-0.114641381158034	0.844162087612813	0.0676853333333335	0.761537039620744
PP7080	-0.252722509385025	0.329002669316492	0.217403750217148	0.645724712341553	0.075312000000002	0.758966957076109
EPAS1	-0.252795839722724	0.328718023333319	-0.240435359548426	0.597862631330416	0.463018666666668	0.0420276417271761
ARHGAP24	-0.252986245531511	0.32815789573337	-0.245829181785971	0.583500894076193	0.313163333333335	0.226251245193179
SHARPIN	-0.252999601059285	0.32815789573337	0.0978208415780455	0.868852648210739	-0.0058296666666656	0.986471172041113
IFT52	-0.253078521422028	0.327855360130163	-0.446556760886263	0.179056569503337	0.111588666666667	0.660962515038256
COX4I1	-0.253103561088587	0.327807196967644	-0.0375541552801901	0.955365651737247	0.0781710000000013	0.766132481270445
ERBB3	-0.253216037640333	0.327332273398900	0.0320787416895955	0.962905980785005	-0.185149333333333	0.422571606196103
C15orf52	-0.253416665536494	0.326576268209441	-0.114141023595558	0.844272085764738	-0.0254006666666666	0.9299162364306
GJB2	-0.253443515857053	0.326519423877806	-0.00371584937295998	0.994588964700772	-0.0977796666666657	0.671492528470281
SLC6A16	-0.253536759314373	0.326139415384935	-0.234449038437462	0.60849909922524	0.238687333333334	0.220405584107617
PIM1	-0.253563781547844	0.326081792844878	-0.228407028920960	0.623306946452998	-0.0605413333333315	0.781866487624294
ACSM5	-0.253571574472367	0.326081792844878	-0.101362052866644	0.865954921256394	-0.0313699999999994	0.904236796123256
SCGB1A1	-0.253590258209526	0.326081792844878	-0.203333494552215	0.676646782959283	-0.084477999999999	0.812603088881849
FLI1	-0.253734988319967	0.325840066335586	-0.439213584079145	0.191077692207340	-0.124413333333333	0.529577105702511
USMG5	-0.253766777136255	0.325833330273798	0.130075597803722	0.812469877647218	0.0552431666666676	0.809769116280204
AMBP	-0.253807823555859	0.325781607114025	-0.103491085166738	0.861943478503987	-0.0445909999999988	0.855321176509099
KRT36	-0.253978946486096	0.325347023875332	0.279207278935161	0.51072434479235	-0.0722393333333334	0.774680328717204
RGS10	-0.254070249842399	0.325097605948050	-0.459431821707175	0.164444235253496	0.00478966666666694	0.988133186272453
SURF2	-0.254319044789713	0.324111778297761	0.0290261554965544	0.96782691865619	-0.0634736666666648	0.777568084061469
CPA2	-0.254352153545379	0.324025077557245	0.149986555168995	0.780417569801375	-0.0245426666666659	0.961509682688243
YIPF4	-0.254356239088477	0.324025077557245	0.0777363578450832	0.900452756580846	0.143135666666668	0.46297633209186
STMN4	-0.254382230979904	0.324025077557245	0.240130032563157	0.598068935430681	-0.131326666666666	0.633285135456374
TMPRSS11E	-0.254382271983060	0.324025077557245	-0.138469691242321	0.799818599059477	-0.00356949999999966	0.991950018109245
TMEM45A	-0.254404775608162	0.324025077557245	0.0917421351209073	0.879153312143409	0.574841333333334	0.0178322921209300
PRDM4	-0.254489423545778	0.324024381560391	-0.46732629424286	0.151936428121882	0.0082630000000007	0.98238813140645
PHKG1	-0.254561108809734	0.323750786520158	-0.0723927186731874	0.907556175729082	0.0112163333333342	0.974895360062947
CCL24	-0.254585388839978	0.323707023007804	-0.227015867646956	0.62581495163125	-0.129261333333333	0.761837433189273
SCG2	-0.254677320069236	0.323631200378364	-0.183680083861061	0.718067440349544	0.316849	0.317651195904626
LHX9	-0.254724025244612	0.323527808570995	0.217158370804551	0.646495351690352	-0.0399596666666657	0.90945379489823
SLC11A1	-0.254784266126478	0.323334939054748	-0.115353582700660	0.843755596233057	0.183576333333334	0.435940401842335
GRK5	-0.254842886557054	0.323198950131300	-0.289270626303783	0.488889656845544	-0.0367688333333329	0.909007610199794
EPOR	-0.254879382874675	0.32309618872419	0.109536943998962	0.851624566716206	-0.0384590000000002	0.893841804508537
GUCY2C	-0.255020173081965	0.322562766521254	0.143546615488121	0.792614473650425	-0.200931333333333	0.412293112262459
PPAPDC3	-0.255084816287161	0.322528086409901	-0.118332230394173	0.838117566591658	-0.0443329999999995	0.873329245675404
BTNL9	-0.255110722414667	0.322494361786547	0.181542319794297	0.72105659643795	-0.0220443333333326	0.955928393298083
GLYR1	-0.255331146452819	0.321777835304585	-0.196593015883903	0.690838714578657	-0.154274666666665	0.439046689557474
HLA-J	-0.255362793192386	0.321738428180403	-0.571475962936814	0.0443803345694495	0.163633333333335	0.397798624983652
TMEM182	-0.255371902249739	0.321738428180403	-0.273302296854814	0.523997406191732	-0.0825726666666663	0.759445936849873
STK17A	-0.255386801039632	0.321738428180403	-0.407801000303627	0.251655786339022	0.441373666666667	0.0447239573107979
SERPINA3	-0.255482908551323	0.321509100085174	0.0814516426659692	0.89615326639288	-0.0751603333333326	0.87629402535202
CRH	-0.255623993672622	0.321049970985946	0.237269331304439	0.603275967173677	-0.0802936666666666	0.729303008652613
HDDC2	-0.255624124562261	0.321049970985946	0.00127871433991115	0.998348925592729	-0.0802156666666661	0.737850396206001
IFRD1	-0.255682135543505	0.321049970985946	-0.101493413508974	0.865765350834364	-0.0241936666666655	0.93803614462273
BTD	-0.255825060411053	0.320597594249016	-0.357789860511848	0.344139573942358	-0.0114279999999988	0.970030485008698
MPP1	-0.255890625810875	0.320355556669308	-0.177816121987894	0.728031191477223	0.0238930000000006	0.93760967383883
C12orf44	-0.255980081576391	0.320210590768495	-0.322794122599108	0.416073658452742	-0.251940333333333	0.190569948330175
SNTG1	-0.255993944315155	0.320210590768495	-0.0357238691030487	0.958366440880488	0.134071000000001	0.527320166803475
KAT2B	-0.256013807204988	0.320205382008313	-0.337931799516666	0.385303753646555	0.913192333333334	0.00580262445524077
CENPBD1	-0.256073252392511	0.320066892990322	-0.247073298797232	0.580536038674523	0.303424666666667	0.1409133773217
DMRTA1	-0.256074352903375	0.320066892990322	0.067939973295374	0.913048535898504	-0.0259629999999987	0.928223674996767
TMEM97	-0.256122479661631	0.320066892990322	0.346022817125827	0.366042223836959	-0.251029666666666	0.209300554554219
FAHD2A	-0.256135787381094	0.320066892990322	0.0376053854467882	0.955365651737247	0.0808513333333346	0.822043577898586
HSCB	-0.256196858701528	0.319989241758349	-0.13206193935539	0.810300414153187	-0.0400773333333325	0.874908057954497
IL18RAP	-0.256246940063759	0.319896067468206	-0.43651392678912	0.195288910691951	0.0678930000000004	0.782975208270571
PBXIP1	-0.256306264404371	0.319758452281769	-0.169688338647418	0.743463590096836	0.00489766666666836	0.988133186272453
AOX1	-0.256353979780137	0.319676748482963	-0.0455511219098752	0.945769054147694	0.145424666666667	0.572263328257281
VAMP4	-0.256503363092435	0.319106143094606	-0.4255578816188	0.215369960032963	0.198566666666667	0.490110576940323
ACTR6	-0.256520695268312	0.319096749421831	-0.237195452076467	0.603350164283107	0.00574666666666641	0.987499363290182
SAT1	-0.256550504226973	0.319027391751791	-0.376723648916422	0.308846814162417	0.401260333333333	0.203654244466101
NDUFB9	-0.256676223847237	0.318645080107900	0.100892635214956	0.866005093242444	-0.0388466666666654	0.891410328517388
ANAPC2	-0.256734686333628	0.318438235932487	-0.0752619802905165	0.904173044514532	-0.119497666666666	0.5530742296054
CLU	-0.256771948932436	0.318333230358188	0.124218723771027	0.825382714207684	0.246833666666668	0.215722751602022
TKTL2	-0.256846104379575	0.318257413064343	0.224639565249424	0.632376156419106	-0.0575829999999996	0.8754328609757
RNF146	-0.256896969561986	0.318176547715077	-0.0703544443379243	0.910394157189308	0.193592333333334	0.317651195904626
CLC	-0.256939753525910	0.318045161665227	-0.0741234566031154	0.905506084101768	-0.0610063333333335	0.782886750366221
TBATA	-0.256980183245966	0.31798129409601	0.00337472364757519	0.994730189431381	-0.0317970000000002	0.938282767814398
C15orf39	-0.257006151195060	0.317948381163062	-0.085033076042649	0.890809718139705	-0.021457999999999	0.94622978991441
ITGB3BP	-0.257048419438433	0.317819558632523	-0.133881088233537	0.808567812988011	-0.0118756666666669	0.969895173471778
SATB1	-0.257053137865148	0.317819558632523	-0.322358409168516	0.417538034812104	0.327908333333334	0.503352066997863
PRELID2	-0.257066980203419	0.317819558632523	-0.006740980980122	0.99129507664977	-0.0889549999999994	0.698352866722226
DAP3	-0.257077459514233	0.317819558632523	-0.252826826417942	0.568985221714455	-0.174237666666666	0.398883894731131
ADORA3	-0.257081143145154	0.317819558632523	-0.26210356536732	0.549752906834892	-0.0335579999999997	0.910513033220957
SPRNP1	-0.257120580567556	0.317819558632523	0.211315891965338	0.659974817413141	-0.279226999999999	0.184571704928446
KBTBD8	-0.257149223355331	0.317819558632523	-0.526187389832368	0.083920348931983	0.159150000000000	0.519406653645538
ERVK3-1	-0.257238035618415	0.317575974073764	-0.275934831759275	0.517549833373363	-0.198387666666666	0.295708667353848
PGLYRP3	-0.257253203026129	0.317575974073764	-0.0772078500974956	0.90125257838581	-0.0105193333333326	0.973770798978734
SLC24A4	-0.257303358059337	0.317410814126132	-0.210407474426709	0.661670522548494	-0.0232866666666657	0.931708923663607
PCBD2	-0.257388601762446	0.317200478226005	-0.25311971432502	0.568478137843739	-0.128825333333332	0.53825134776358

CRLF2	-0.257389358083386	0.317200478226005	-0.0680301616424729	0.912778637872411	0.280028666666667	0.137297162355315
ATP6V0A2	-0.257502779545552	0.317010623839367	-0.168946760736814	0.74568001679609	0.0792959999999998	0.714244999058301
MLX	-0.25754161888153	0.316935390456175	0.0561233504153339	0.932652183149857	-0.260257666666665	0.200242771131457
SLC25A2	-0.25762586861953	0.316754058839319	-0.00528910142070927	0.99251028338798	-0.0439959999999995	0.890094615232462
ZNF831	-0.257632936954588	0.316754058839319	-0.175615331845506	0.73187914820204	-0.0284776666666661	0.915761046438965
GKAP1	-0.25777382219081	0.316193994035056	-0.395096694518343	0.272692742937895	0.255811000000001	0.473337739823928
RNF150	-0.257802218611315	0.316132162279785	-0.00437118955514351	0.99405344216434	0.108243666666667	0.596617523400524
CYP1B1-AS1	-0.25781998159538	0.316121200168883	0.105411633047282	0.85988740225956	0.198333	0.355490219708139
NDST4	-0.258014784695419	0.315412099661004	-0.407379709933858	0.252285038466352	0.0924610000000008	0.656682070833594
SECISBP2	-0.258034653259896	0.315391118924201	0.0293536815379180	0.967641990928056	0.269601	0.178835113772546
EMB	-0.258171518343732	0.314826064051786	-0.499558920653471	0.113777333040600	-0.068405333333332	0.760951989611575
PDHA1	-0.258191005110803	0.314826064051786	-0.347025523322238	0.364253555784646	-0.129835333333333	0.512796060754107
FCER1G	-0.258206901076875	0.314826064051786	-0.41916757969931	0.229902159410375	-0.0264516666666658	0.931708923663607
THAP8	-0.258238843022652	0.314826064051786	-0.180142072374775	0.723525599963752	-0.0642676666666653	0.788201431210286
MFSD4	-0.2582741648546	0.314826064051786	0.00423381208149003	0.994111873745308	-0.147199666666666	0.535492155031224
ZNF541	-0.258314781153478	0.314773872951073	-0.307173167094255	0.453060894675967	0.00913899999999998	0.980067494752551
PSMD6	-0.258357466440398	0.314773872951073	-0.338410536059811	0.384701467775014	0.067814666666669	0.771373465365664
UNC13A	-0.258440765230293	0.314636152552439	-0.0583525993101402	0.928725686060612	-0.120232666666666	0.626500880282759
CNOT8	-0.258460773005849	0.314614722952448	-0.0695817692407996	0.911313423064389	0.083695666666668	0.705698790800484
RDH13	-0.258485925584961	0.314568767216216	-0.0659250147491746	0.917090201751967	0.0193349999999998	0.952460815972492
SCN9A	-0.258571826694721	0.314233210242435	-0.302020151837839	0.462229407853213	-0.0349083333333329	0.903788355106891
GUCA1C	-0.258593258232456	0.314205034474465	-0.108821871206357	0.85350993671449	0.30214	0.310277378475854
FBXO16	-0.258618889271846	0.314156859160595	-0.133245340547905	0.808992949393043	0.179312333333333	0.489894482040166
NDUFS4	-0.25864379779914	0.314112141230073	-0.216275924406473	0.648391670659269	-0.0112596666666659	0.972408449448159
A1CF	-0.258679507045568	0.314015978487315	-0.180948484714242	0.721866914445283	-0.0184146666666661	0.947805707169764
LCN12	-0.258759828846478	0.313707401227216	0.215284549659158	0.6505804894272	-0.0677186666666661	0.788201431210286
CCDC12	-0.258989265912787	0.312690301332064	-0.239130819046458	0.599801655225887	-0.161359000000000	0.443378989641463
C2orf53	-0.25900120805501	0.312690301332064	0.0481934763674023	0.943089870033472	-0.112527333333333	0.675183563312084
S100A7A	-0.259023997536697	0.312690301332064	0.207838550591930	0.667695745454683	0.0307320000000004	0.910190419163007
ABHD5	-0.259051642083187	0.312690301332064	-0.252169264139737	0.570465803488487	0.0999816666666667	0.63512186298002
TRMT10A	-0.259259347085566	0.311998075302631	-0.131576328543805	0.811042872465441	0.110375666666667	0.583287370155883
CHRNA2	-0.259272708291660	0.311998075302631	0.263415062162790	0.545874639137454	0.0157033333333339	0.9681321732097
CREB3L1	-0.259330210880138	0.311809405965946	-0.161468242690169	0.759189376564965	-0.0396309999999997	0.888476605628154
WDR55	-0.259373366253609	0.311678505566295	-0.150486823661675	0.77904503981756	-0.382983333333333	0.0649438674882112
PET112	-0.259641941493183	0.310680967928277	-0.148826029369561	0.783041584899534	-0.0299509999999991	0.906093399285191
RPS21	-0.259721044361419	0.310474355217187	-0.0288780125328146	0.96782691865619	-0.0799459999999993	0.70830847840715
PIGT	-0.259740287020645	0.310456882042219	-0.394361273200337	0.274550296475226	0.280590000000001	0.134354816258782
TRIM9	-0.259818767403816	0.310159540275168	-0.124279236997150	0.825382714207684	-0.0669326666666662	0.843152489417868
CCDC53	-0.259916915781983	0.309885300840273	0.159415010677111	0.764209767000699	0.0953543333333343	0.719304894613107
HAL	-0.259918003345236	0.309885300840273	-0.165240109117469	0.752586940524596	-0.0800906666666659	0.753381247301444
B4GALNT1	-0.259952526132961	0.309885300840273	-0.288432868646509	0.490230108819486	0.210687666666667	0.390967882720418
B3GALT4	-0.259955267045625	0.309885300840273	-0.186002146912642	0.713284034966074	-0.165044333333333	0.443378989641463
TBC1D25	-0.260033448016888	0.309805014409379	-0.233966924381587	0.60951860401502	-0.0719498333333321	0.76368723681351
SLC22A7	-0.260083353491202	0.309718428073204	0.139019571782838	0.79864602540281	-0.0580806666666659	0.804452238131942
GOLT1A	-0.260139664293806	0.309599683341335	-0.102821553030966	0.86257932725575	-0.194461333333333	0.360029821276094
CD9	-0.260338640352126	0.308881804480903	-0.159615548931867	0.763507083670776	0.405673333333333	0.198299581791991
ELOVL5	-0.260341168588143	0.308881804480903	0.369303996128364	0.323285574998587	0.0698400000000022	0.768446434650364
ELL3	-0.260377958089334	0.308843390424635	-0.201197044850032	0.680054545720343	-0.0047279999999996	0.987937064305561
AHCY	-0.260407181712627	0.308779140304064	-0.116291821128395	0.84217383062547	-0.0502413333333329	0.82667899725046
MTHFD2	-0.260482719443872	0.308496807312416	-0.405479243814505	0.254747594046254	-0.145502333333333	0.520424642482869
ATPIF1	-0.260505985894271	0.308460660752900	0.10127846106713	0.866005093242444	0.0337880000000008	0.901246264384169
PRRG1	-0.260622808696788	0.307986903403344	-0.350862240979916	0.357260848237483	0.388507666666668	0.0895407923270921
KCNA3	-0.260663423630759	0.307939999079416	0.0954935683926644	0.872754579338908	-0.0373656666666664	0.887363245747406
KIAA1324L	-0.260742278138538	0.307715923622699	-0.437607451281347	0.194173491891936	0.267861333333334	0.218478495014124
FRMD1	-0.260751522688456	0.307715923622699	0.285826512272262	0.496495911343846	-0.160034333333333	0.516179292727291
NSF	-0.260776708222179	0.307715923622699	-0.138617810308049	0.799270555590463	-0.0837033333333328	0.712601873916151
YARS2	-0.260810693710927	0.307715923622699	-0.0207332402225636	0.976330597778846	-0.0384783333333335	0.879843042066371
FAM83A	-0.260819184198135	0.307715923622699	0.131554606608992	0.811042872465441	0.0320966666666668	0.927671110581394
TARBP2	-0.260874212440132	0.307618758899095	-0.0264244514375193	0.970427456927088	-0.0658803333333335	0.768446434650364
PAPD4	-0.260881921862093	0.307618758899095	0.0119482682190987	0.986261540519832	0.221837333333334	0.244901399556031
GPATCH3	-0.260916568641114	0.307618758899095	-0.0409246900268045	0.952426531595115	-0.177340333333333	0.387356917839865
SLC5A6	-0.260951581397807	0.307618758899095	0.0183560668110656	0.979214953187634	-0.0914143333333328	0.758445077185308
RBP1	-0.261041545369270	0.307543737215194	0.0890725478370394	0.883790169331042	0.0233866666666677	0.937545603686416
ALDH3A1	-0.261092431501947	0.307391724628225	0.147748386425964	0.784475200791671	-0.150596000000000	0.523630556735617
OSBPL3	-0.261297661719532	0.306734681541711	-0.562700889377389	0.0508877229788208	0.0613806666666676	0.800816123802171
NTSR1	-0.261380399091976	0.306511098771663	-0.0825589161122039	0.893825951190383	0.0376190000000007	0.88629962370345
XKR4	-0.26140238796279	0.306511098771663	0.0181554613305623	0.979327298872732	-0.0703679999999984	0.828240868185377
CETN3	-0.261547136382441	0.305992373833073	-0.237326154779109	0.60323620145965	0.214929333333335	0.435982596289422
POU2AF1	-0.261590440698856	0.305992373833073	-0.260554315388278	0.553540075799904	-0.142107666666666	0.471741080987819
GPR97	-0.261679983587552	0.305663582702983	-0.245864867944763	0.583444118723645	-0.0866283333333326	0.770112696597648
MIOS	-0.26173181296792	0.305494284507066	-0.302015578686321	0.462229407853213	-0.187144	0.442036873484362
TMEM72	-0.261746546586676	0.305494284507066	0.0123282404040139	0.985386024673793	0.0641483333333334	0.780474051101386
PALLD	-0.261842573766412	0.305164491909189	-0.461313924709827	0.161386904160798	0.404958666666668	0.0490477538807126
C16orf52	-0.261962414474517	0.305002191328993	-0.0411358009069341	0.952102539249605	0.0240233333333329	0.942708941944282
KLB	-0.261967558321642	0.305002191328993	0.0441740589566161	0.948493391127553	-0.143379999999999	0.642251423610972
PDDC1	-0.261973623982580	0.305002191328993	0.0406860108318058	0.952426531595115	0.049485000000001	0.843751236104194
ILDR2	-0.262012691366863	0.304987224003126	0.0747131071722647	0.904466414650249	-0.234058	0.313220411537404
EPHX2	-0.262022894825487	0.304987224003126	0.0227348846342315	0.97536722461972	0.201872	0.306286104860031
TM4SF5	-0.262023030456776	0.304987224003126	0.104612073335336	0.86067831050617	-0.139988666666667	0.532604205131631
NDUFA13	-0.262147816569221	0.30472248274705	-0.0455008642851471	0.94582511262151	0.0576496666666685	0.794486775271372

PLCH1	-0.262167967740124	0.30472248274705	-0.070756502579207	0.909764940148113	0.0822420000000007	0.812792750346224
UBASH3B	-0.262171022169651	0.30472248274705	-0.328274186344112	0.407301172667012	-0.0756766666666666	0.7744217439919
IL36B	-0.262192842823034	0.30472248274705	-0.105310597158099	0.86011776595122	0.0839776666666672	0.717743913529804
ATAD3A	-0.262235560841618	0.30472248274705	-0.445467708839449	0.181231004740944	-0.116835666666666	0.567224087161503
RIMS1	-0.262309786635255	0.304553178163346	0.0670342550290619	0.914926078524511	-0.000531999999999991	0.998309716374356
BCS1L	-0.262350944021767	0.304434321032723	0.0830992737030275	0.893325358714619	-0.247320999999999	0.248900866475991
TCN1	-0.262387268322634	0.30433808747513	0.0440410656290499	0.948520928173175	-0.0545996666666662	0.824700707442131
IQUB	-0.26247899323054	0.304104990420902	-0.295504548800984	0.478189129303348	0.101474666666667	0.649712375357267
ABCC2	-0.262491969547562	0.304104990420902	0.289715536117443	0.488870852256136	0.0147900000000003	0.96741948433529
CAMKK2	-0.262516119610091	0.304103780102387	-0.262139442281874	0.549752906834892	-0.0301423333333323	0.918514302627986
ANKMY2	-0.26254757143462	0.304030424845065	-0.376041553009694	0.30992624456671	0.294693666666668	0.126806477863114
DYDC2	-0.262551301921309	0.304030424845065	0.052179841231204	0.938067483409541	-0.000541666666666623	0.998275219056485
PCSK5	-0.262705746921508	0.303512628689343	-0.148726575502236	0.783041584899534	0.166911666666667	0.653707021350129
WDR34	-0.262786805947475	0.303281519064891	-0.130996806414704	0.812271254601992	0.173310666666668	0.370394153991469
C11orf63	-0.262817728220246	0.303281519064891	-0.0238805159528823	0.97479225206569	0.121093000000001	0.639837290428782
ATOX1	-0.262967359255594	0.302954260384014	-0.303014352607961	0.462105017205825	0.0308486666666677	0.903788355106891
OR10H3	-0.262988276299697	0.302930294820916	0.00580303878133527	0.99251028338798	0.0554960000000006	0.840136012582989
CLEC4GP1	-0.263126279317551	0.302507931090051	-0.130692161920442	0.812271254601992	-0.221345333333332	0.397798624983652
HFE	-0.263138383247321	0.302507931090051	-0.449627863429895	0.177450556373669	-0.0282149999999996	0.924674647382585
HCRT	-0.263151579480314	0.302507931090051	0.00592049171975086	0.99251028338798	-0.127244666666667	0.601915646309729
RPS6KL1	-0.263178027872439	0.30248749375641	-0.230230220267225	0.618707227156037	-0.0537746666666659	0.846842551791738
IL18BP	-0.263183242736829	0.30248749375641	-0.330410826163276	0.402284079938133	-0.0975893333333332	0.634587745028953
CLVS1	-0.26321811007333	0.302447905905106	0.09562110372569	0.8726818955265	-0.119590000000000	0.719304894613107
TAAR1	-0.263231967318568	0.302447905905106	-0.170924735396783	0.740768646795758	-0.0199546666666664	0.955115242974667
STRBP	-0.263243277292534	0.302447905905106	-0.446975395105717	0.178543100892855	-0.0158373333333318	0.957844701580613
FRS3	-0.263331581647821	0.302140308308285	0.161665018394056	0.758955920988521	-0.0486893333333332	0.833637671948824
IRS2	-0.263372694450361	0.302022536202208	-0.0461186822466978	0.945364757579857	0.0199326666666681	0.945904449997854
MRPL11	-0.263383320051927	0.302022536202208	-0.383804515022063	0.293447764611617	-0.160033333333332	0.509869431796637
TTLL11	-0.263580636489939	0.301276345323245	0.0398239446815845	0.953630861782686	0.0217433333333343	0.935261323444314
CRIP3	-0.263600307815161	0.301258390093487	-0.0784341119768007	0.899281198071059	-0.0502626666666654	0.855240892932328
LIMK1	-0.263653821901848	0.301166025338068	-0.247223364823374	0.58021047968492	0.00448200000000082	0.988942941807152
VPS29	-0.263821531952769	0.300598623092132	-0.359368808715072	0.342126738931318	0.0608318333333345	0.783604035243992
CYP11A1	-0.263899568050906	0.300310190487752	0.151167049651478	0.77066107821265	0.411261000000001	0.0454459478729606
RNASE6	-0.263903341428512	0.300310190487752	-0.447834533316526	0.178249202877872	0.299075666666667	0.216591326893487
SFMBT2	-0.263907849895973	0.300310190487752	-0.595993940309623	0.0342864566106277	0.0916553333333332	0.716517864791776
HLA-DPB2	-0.263946441236954	0.300310190487752	-0.150498578260474	0.77904503981756	-0.00318433333333318	0.993756838831997
ARHGAP10	-0.263962407082125	0.300310190487752	-0.342829269223372	0.374882411235977	0.204418666666667	0.407642090578389
LOC344887	-0.263993020895364	0.300310190487752	-0.162032206646299	0.75854722544558	-0.0663843333333336	0.830669063826073
CLECL1	-0.26406532028663	0.300128787008842	-0.228157391431305	0.623935503350068	0.122460000000001	0.634105753368746
EARS2	-0.264130756103168	0.299972390295981	0.0206915536570042	0.976330597778846	-0.119655333333332	0.549323798856043
KCNA7	-0.264222420176751	0.299621317307826	0.0555147720084054	0.933409139126562	-0.336704000000000	0.442411592010919
GPR157	-0.264375940360063	0.299350941689627	-0.0105315465399905	0.98774587825743	-0.0489006666666668	0.831432210225512
KDM1B	-0.264412588453561	0.299298016235916	-0.245747263032506	0.583812428024107	-0.165603666666665	0.58391025729515
MATK	-0.264423353099054	0.299298016235916	-0.0813189582497599	0.896315978987498	-0.123237333333333	0.568393891086758
MAPKAPK3	-0.264424928907946	0.299298016235916	-0.304281751197854	0.459458170903768	-0.259377	0.177614563220760
WWP2	-0.264458612105082	0.299298016235916	-0.106078453805447	0.858902068936183	0.0715283333333334	0.771847553133033
KRTAP4-7	-0.264500574895105	0.299298016235916	0.0286336315084003	0.96782691865619	-0.127380666666666	0.628131452559002
CDKN1B	-0.264561151692737	0.299298016235916	0.0361498517275833	0.958015079993705	0.311116000000001	0.124422753228706
TMEM62	-0.264595451267236	0.299290180873038	-0.154584571435150	0.771310656070265	0.432167333333334	0.0428311881894919
C1orf162	-0.264609814689290	0.299290180873038	-0.362189664260512	0.338002847456404	-0.070013	0.784944347000133
MTIF2	-0.264716191647731	0.298952203302698	-0.307434645865375	0.452689746287922	0.0956546666666665	0.654911833752806
CAMSAP3	-0.264717498888899	0.298952203302698	0.151864155500888	0.776343233798271	0.0644790000000006	0.853705749031551
LAD1	-0.264921988135766	0.298368557598436	0.0116004737161607	0.986661750359244	-0.00267533333333327	0.993756838831997
FAM47B	-0.264940114632805	0.298358324317696	-0.00483326600118761	0.993090001117876	0.00380433333333338	0.993756838831997
TRADD	-0.265076693045822	0.297948167180388	0.037141990566372	0.956363028822848	-0.0769843333333318	0.735665165720299
DUSP1	-0.265090339792348	0.297948167180388	-0.185859096593569	0.713738661681529	0.385142666666668	0.0629160804357479
NMRK2	-0.265117417165643	0.297909067355924	0.132983268501615	0.809578511927685	-0.274085666666666	0.264927993735684
HSPA4	-0.265119915474145	0.297909067355924	-0.328289419383964	0.407301172667012	0.00255433333333466	0.993756838831997
GVINP1	-0.265154788663196	0.297909067355924	-0.335786959793666	0.389949066081534	0.093817	0.6846855637534
CHST6	-0.26515954005355	0.297909067355924	-0.488242912641888	0.127979276934222	-0.190142333333333	0.472599709268839
TES	-0.265243089157653	0.297874606857331	-0.173158468831680	0.736877912871223	0.137207666666669	0.488821844868242
FDCSP	-0.265289536608608	0.297848384622863	-0.0661096454442579	0.916792605561869	0.0401876666666673	0.870662337224757
MSI1	-0.265423549944796	0.297377275385931	0.374280445454469	0.314207741629911	0.00638933333333396	0.987134800094338
ZNF135	-0.265453155687448	0.297314335109747	-0.379713400818333	0.301761007063413	0.0312819999999999	0.933881253208642
COPS7A	-0.265511939782155	0.297116918151739	-0.172233522900294	0.738567943867944	-0.0529389999999988	0.823297424642592
PRKCH	-0.265587729986308	0.296841270277965	-0.0442647841180091	0.948385499132766	0.00738466666666665	0.982375192575954
DAK	-0.265592150361306	0.296841270277965	-0.0298230481597018	0.96695070705759	0.110611333333334	0.600950363504414
TWF1	-0.265643176313071	0.296841270277965	-0.277901769652205	0.512820026324921	0.0556716666666681	0.805964794527842
PKLR	-0.265679204572917	0.296841270277965	0.207603836700193	0.668339204922102	-0.0986346666666665	0.648442399981194
GSPT2	-0.265772141382852	0.296653962586001	-0.214824506096481	0.651720792906497	0.214497333333334	0.291501950143291
POPDC2	-0.265783960125984	0.296653962586001	-0.26410482976564	0.544525995334757	0.138191000000000	0.498456714564834
OR2W1	-0.265817935333205	0.296653962586001	0.282112480976677	0.504223489294266	-0.0297440000000003	0.907648578460912
C6orf203	-0.26590411249371	0.296340827079671	-0.112110816955468	0.847454473507881	0.0691673333333336	0.752992371482229
ANKRD24	-0.265934358784916	0.296275238713528	0.164234966311181	0.754189116283047	0.0951200000000002	0.656785927775364
CHIA	-0.266164962116802	0.295582735573979	-0.0367297233414105	0.956972826723385	0.0288660000000006	0.922697964464038
CBLL1	-0.266168376467172	0.295582735573979	-0.227252278930945	0.62581495163125	0.220526000000001	0.426237423530267
POLE4	-0.266179135228401	0.295582735573979	-0.0551987411009886	0.933691629547946	0.112019000000001	0.588666179294878
PKDCC	-0.266179768524036	0.295582735573979	0.145541814620093	0.787804113309913	0.0953983333333339	0.728680504498904
GRHPR	-0.266240584137682	0.295582735573979	0.089204440481159	0.883460564717377	0.0188486666666675	0.949295980201336
NARFL	-0.266307924749208	0.29548643514339	-0.121454374651905	0.831697898017694	-0.00413066666666603	0.988942941807152

MAPT	-0.266347269186645	0.29548643514339	-0.192707267648328	0.699904185007497	-0.161959666666666	0.482760288609588
AKTIP	-0.266375913310384	0.29548643514339	-0.427090454158499	0.212621410541582	-0.577681999999999	0.0660125264725009
GATA6	-0.266401915875716	0.29545108637009	0.209234390732194	0.664955375015254	-0.0827393333333335	0.776805598353461
PIGU	-0.266459482752563	0.295334015938103	-0.193979281882278	0.696962749474912	-0.224318333333333	0.315543124546046
NHLRC3	-0.266531874015160	0.295222669854865	-0.0587444456871466	0.928472484757714	-0.341990666666667	0.167001732057148
POMP	-0.266550619533722	0.295210322929806	-0.432939093597803	0.202301256144175	0.00109700000000161	0.99704422037925
TMBIM6	-0.266550743072094	0.295210322929806	-0.281058165092037	0.506886267079183	0.109770000000001	0.585584889305174
OSBPL9	-0.266565677331044	0.295210322929806	-0.155222554111841	0.77066107821265	0.121244333333335	0.577269890738407
XIRP1	-0.266566947858154	0.295210322929806	-0.232910056252308	0.611711680668619	-0.055044666666666	0.85576203728561
NLRP6	-0.266637633045917	0.295105795680037	0.0889054562797756	0.88392988973275	-0.000341333333333262	0.99883107916854
NAMPT	-0.266646065683417	0.295105795680037	-0.337891280227007	0.385303753646555	0.130416666666667	0.523762186913243
CEACAM4	-0.266672009943770	0.295105795680037	-0.145490831536264	0.787804113309913	0.0617800000000001	0.785009062971273
PLEKHB2	-0.266728057892521	0.295059712641432	-0.228161124490077	0.623935503350068	-0.135449999999999	0.501655787180246
CUZD1	-0.266748398242116	0.295059712641432	-0.105008824746530	0.8606536389692	0.0729149999999999	0.865748629809888
NECAB3	-0.266752168913924	0.295059712641432	-0.081053694152661	0.896361013063453	-0.319597	0.195399498685346
PPP1R13B	-0.266774811202158	0.295059712641432	-0.198681454380638	0.685703984501304	-0.0427373333333316	0.885518223441274
DENND4C	-0.26683105677517	0.295030199564941	-0.0263450527271307	0.970617924205993	-0.0484366666666654	0.866601971778042
NPC1L1	-0.266871262875104	0.294919457553592	0.0999916701804056	0.867290404865575	0.0977946666666676	0.704417585200123
ZNF429	-0.266908160264192	0.29482395054586	0.091369622346459	0.880222627280556	0.823883	0.00846148955906804
C8orf56	-0.267065570968389	0.294175400319633	0.111935382879536	0.847677166378871	0.00265533333333373	0.993756838831997
TUBA4A	-0.267088124121868	0.294145838816555	-0.403452764190867	0.258015858764176	-0.0369623333333322	0.900467460403227
PIGX	-0.267109774403468	0.294120431578585	-0.166812955711512	0.750324293110858	0.00838200000000126	0.984928710257663
SNRNP25	-0.267113001644765	0.294120431578585	0.0948067250387446	0.873791310803026	-0.0524676666666663	0.834476346642783
NUDT6	-0.267337392875364	0.293299137552393	-0.293232705368328	0.48133638278712	0.0847646666666675	0.765058274870877
C16orf93	-0.267342731965961	0.293299137552393	-0.00736137900546466	0.990873555460625	0.104089666666668	0.630176150998965
OR2C1	-0.267388583276411	0.293278884939845	0.125586611627351	0.82367707928204	0.0829903333333338	0.709568037614009
SETDB2	-0.267422699396127	0.293203786241296	-0.190028750448551	0.704348376269734	0.0311030000000003	0.929594956587283
C11orf35	-0.267427660383899	0.293203786241296	-0.00364194601305495	0.994588964700772	-0.040893666666667	0.904507944803624
DUSP3	-0.267434787359669	0.293203786241296	-0.319291554612357	0.426142369255984	0.0470866666666669	0.861860658844305
S100A7	-0.267484019087241	0.293144590401814	-0.287863312957168	0.492227498343947	-0.111334999999999	0.579470077554465
C11orf1	-0.267484828575440	0.293144590401814	-0.0663924702366542	0.916323048676587	0.0558759999999999	0.821587043214193
SLC10A3	-0.267487151529984	0.293144590401814	-0.620369680707138	0.0237528986370316	-0.129121333333333	0.520368312537146
VSTM2A	-0.267496242992583	0.293144590401814	-0.158238001126557	0.767214373310291	-0.135139333333333	0.516467297561779
TMOD2	-0.267608322585509	0.29305833272293	-0.236352296278538	0.605130123419417	-0.169464	0.67770267433214
IQCK	-0.267755448354339	0.292578523977335	-0.204001199075767	0.675344930297902	0.188464000000002	0.48843863773377
TMEM128	-0.267831301174508	0.292442582626094	-0.346051414533392	0.366042223836959	0.0371546666666677	0.902859284837213
PLA1A	-0.267965618855518	0.291902975201220	-0.360762099234839	0.34034906917442	0.0616960000000005	0.829055670438719
C16orf11	-0.268009050860892	0.291778537621201	-0.221111069234200	0.638286782439236	-0.0778059999999991	0.794467820741688
HCG4	-0.268025653913399	0.291776532954195	0.237290757267464	0.603259759765221	0.0410953333333335	0.877417966415793
TCF15	-0.268039519219635	0.291776532954195	0.205127573725775	0.673382949111104	-0.0624836666666655	0.87518023052865
SLC5A1	-0.268054989252818	0.291776532954195	-0.0751600512669113	0.904173044514532	0.128229000000001	0.633561720825521
NBPF4	-0.268055763301586	0.291776532954195	0.0346962596081803	0.959404955370931	0.0536687777777783	0.814211123519969
DCAF12L2	-0.268116431138635	0.291705608323769	0.417710676792543	0.233975473685118	0.0382703333333333	0.910004971096712
RAB9B	-0.268159802763102	0.291705608323769	-0.360583852763977	0.34034906917442	0.213448333333333	0.263064019049831
TOX3	-0.268175200414297	0.291705608323769	-0.305352519075061	0.458013550008763	0.0129643333333335	0.96455391684166
CNOT4	-0.268219422248467	0.291705608323769	-0.109872369022830	0.851013616427418	0.256469666666668	0.173494642908599
MRPS12	-0.268235968812817	0.291705608323769	-0.102465324999621	0.863158472924381	-0.257948	0.187638103900419
STX1A	-0.268239192956586	0.291705608323769	-0.359992877804138	0.34143422019372	-0.0758383333333324	0.738101469932177
NPL	-0.268246405206551	0.291705608323769	-0.292088086572856	0.484470659050591	0.171401666666667	0.398883894731131
EDAR	-0.268266210303843	0.291705608323769	0.134016799420982	0.80854627520514	-0.0196769999999997	0.955460918122607
POLR2C	-0.268302325252065	0.291705608323769	-0.100423697726582	0.866539444904289	-0.0704346666666661	0.748579513438275
LYNX1	-0.268330749567583	0.291705608323769	0.339045048353619	0.382919428108811	-0.0561286666666655	0.830093119549048
C21orf62	-0.26837064577823	0.291657423511015	0.108994737488752	0.853033740855416	-0.157897333333333	0.546219696447415
DLGAP1	-0.268552227344470	0.291222224339989	0.0273084487498908	0.970207612777188	-0.0654253333333326	0.78197285216323
GNG7	-0.268597769856506	0.291162671281836	0.176365705327015	0.7303866161159	0.079955666666667	0.741438256509195
ZNF169	-0.268599620540795	0.291162671281836	0.0772090332587402	0.90125257838581	-0.215249333333333	0.40500623952892
ELMOD3	-0.26864637744599	0.291089195489609	0.0610776939930753	0.92470690387802	-0.131251666666666	0.6034967860712
ASB10	-0.268700669910055	0.290989854084311	0.123107312130486	0.827789978865629	-0.0316253333333335	0.902642066851499
CBLN1	-0.268722436023673	0.290989854084311	-0.0705648710050694	0.91009246060164	-0.0405010000000000	0.902049890102179
EME2	-0.268788835269609	0.290884467620576	-0.240239326241037	0.597862631330416	-0.138711666666667	0.502766729725258
BEST1	-0.268795523588134	0.290884467620576	-0.0718798175347593	0.90837606217316	-0.0720876666666666	0.743456988551011
GLTPD1	-0.268870525303956	0.290660423437029	-0.102479140167668	0.863158472924381	0.189307333333333	0.469562766268737
DBI	-0.268881678465914	0.290660423437029	0.227840960994429	0.624564310654617	-0.0057239999999979	0.986688186746568
SIGLEC10	-0.268924302380492	0.290563700738713	-0.411619555327409	0.243528138193926	0.145863333333334	0.457124518738818
CDC42SE2	-0.268996153154431	0.290310554750871	-0.373416353794661	0.315876570724519	0.0522730000000003	0.829198827622024
SLC25A48	-0.269027669193023	0.290241180010769	0.135117355348313	0.807470535721699	0.070373666666668	0.787248874966057
A4GALT	-0.269080019957253	0.290132534928617	0.0151415979894657	0.982921609770846	-0.189114666666667	0.343729765439488
OMA1	-0.269248205578961	0.289608276072372	-0.445520966743281	0.181231004740944	0.144452333333334	0.516217427447249
PUS7	-0.269330754180204	0.289307151261041	-0.0411817073015821	0.952098348256047	0.0316600000000011	0.910190419163007
C17orf47	-0.269416342167621	0.289140435618476	0.0330726343210759	0.961277493638149	0.0764650000000005	0.721039266334933
HMGN3	-0.269425159998590	0.289140435618476	0.274496124794931	0.520473735870349	0.0084460000000011	0.979790222743567
CAMKMT	-0.269554462943634	0.288888635170179	-0.106664705175907	0.857937967416038	-0.111382333333332	0.771396688597852
IL11	-0.269683432768085	0.288668466653145	0.179952950305439	0.723716289903531	-0.0430489999999988	0.894649771256526
MTSS1	-0.269699405918378	0.288668466653145	0.248351392195831	0.577670812328515	-0.240958333333332	0.36541451620531
RIC3	-0.269710479568503	0.288668466653145	-0.0910543352429102	0.881053992915981	-0.104015000000000	0.716517864791776
TRIM29	-0.269736170378643	0.288651483355957	0.0916328631552075	0.879272912391142	-0.101856666666666	0.694259186138267
CHRDL2	-0.269749480523983	0.288651483355957	0.00306823905712204	0.994986311144058	0.0318643333333338	0.906754770223696
HSD17B8	-0.26979785862942	0.288519836413787	0.0202821259258406	0.976609856377422	0.0159263333333340	0.955815367168137
COX14	-0.269861410903481	0.288446408052970	-0.00974866107388575	0.98909166696601	-0.536754	0.0314339287318021
GNAO1	-0.270031623665952	0.288052932711159	-0.0251035528525097	0.972494094458606	-0.0138053333333321	0.972395918043445

PCYOX1L	-0.270044425785704	0.288052932711159	-0.0331739198330438	0.9610502944838	-0.174888999999999	0.397798624983652
PPIA	-0.270065219535039	0.288049021445731	0.239075753192849	0.599801655225887	0.0666840000000023	0.762681520573934
PMEL	-0.270069566706275	0.288049021445731	-0.0737503729004884	0.905506084101768	-0.0163823333333326	0.955460918122607
TRPC7	-0.270113248121414	0.287979732852095	-0.165730163031535	0.751952150889158	-0.0978853333333332	0.633166039956913
FAM118A	-0.270124248718601	0.287979732852095	0.115252405906180	0.84381357845296	-0.199932666666667	0.311354677197697
TRIM36	-0.270185537321443	0.287958980637516	-0.170262260381424	0.74270446821332	0.309519666666668	0.142821378450256
DBC1	-0.270202213118047	0.287958980637516	-0.262846856245347	0.547362601048006	0.763178666666668	0.030025317565228
OR2C3	-0.270378641305880	0.287594111524568	-0.146160602239371	0.787402233709509	0.169279666666667	0.403772861091022
BPIFA4P	-0.270399327563257	0.287574734221981	-0.0190160324334655	0.97832242527852	-0.102795666666666	0.718749100405876
COL9A1	-0.270511185400770	0.287142478144436	0.253896093469264	0.567694846153007	-0.00432699999999997	0.991175862027014
CACYBP	-0.270529989201085	0.287131650215100	-0.103207336033092	0.86231943896506	0.0813201666666664	0.706115075578775
PAPLN	-0.270566973649464	0.287038553947308	-0.0709009930414778	0.909485982367054	0.0336400000000002	0.903788355106891
SF3B14	-0.270650274880241	0.286810237921555	0.0794071488107851	0.898323801514594	-0.244156999999999	0.223108571904444
NKAIN4	-0.270736242721081	0.286506937713597	0.0631163338206352	0.921562696294372	-0.0958459999999994	0.765183265579617
C6orf226	-0.270745754953308	0.286506937713597	0.0312043484642631	0.96506747451534	0.120429333333334	0.580149967309238
CFDP1	-0.270772658211773	0.286506937713597	-0.0760744550177115	0.903296922694955	0.0614303333333344	0.829590360509491
ATG4D	-0.270817981516614	0.286423331647491	0.0957931648615944	0.872537273851412	0.188341666666667	0.320462604748547
LRRC3	-0.270819822013499	0.286423331647491	0.00549243505272115	0.99251028338798	0.0810950000000004	0.731974410873657
ECRP	-0.27087432507597	0.286317256158407	0.0813837566792913	0.89617906480153	0.0520943333333338	0.870662337224757
KIAA0513	-0.270887861712277	0.286317256158407	-0.345817742002836	0.366623371189736	0.574845666666668	0.0142639134811873
LETM1	-0.270930778721053	0.286285055483211	-0.0409562586650210	0.952426531595115	-0.515424999999999	0.0309320552705188
MAN2A1	-0.271025975329151	0.286077947446191	-0.383472106883562	0.293474457479736	-0.357454333333332	0.099031968776772
MDH1	-0.271040794352109	0.286077947446191	-0.0246272922462778	0.973393389577498	0.0507200000000008	0.824044031896077
VWA5B2	-0.271067446931908	0.286063164085840	0.088194292687496	0.885557272034463	-0.172554333333334	0.541939010010167
USP13	-0.271078150244476	0.286063164085840	-0.392852973706120	0.278297610043666	0.249647333333334	0.243957990539057
UXT	-0.271084117883159	0.286063164085840	-0.261673482445071	0.55111709539619	0.204068333333335	0.351839993215702
FAM13B	-0.271090503573949	0.286063164085840	0.0133003206359018	0.984212870768403	0.150278666666668	0.513141966218084
C6orf89	-0.271218403928007	0.285915022742951	0.0107311945575438	0.98774587825743	-0.0421436666666643	0.866027240978154
ORMDL2	-0.271312765159747	0.28567554709106	-0.249486265878101	0.575370998761354	0.0429090000000003	0.877417966415793
SCAMP5	-0.271351467585377	0.285649754370281	-0.232656645998757	0.61200913457472	0.0202419999999998	0.941559580750404
RUFY1	-0.271360361177246	0.285649754370281	-0.232839533993125	0.611711680668619	0.122388000000001	0.546219696447415
RAB3B	-0.271393974615929	0.285606842775680	-0.00377661067972074	0.994588964700772	-0.550632666666665	0.137582533106320
EXOC2	-0.271451606637308	0.285421208307357	-0.267039488323276	0.537139877377169	0.173583666666666	0.367206442400058
ARSB	-0.271469907060957	0.285413116262472	-0.361109676589978	0.339627732901974	0.132013333333334	0.596725125925172
TXNDC17	-0.271517188922433	0.285274272422978	-0.111096393092860	0.849127967004266	-0.118650666666666	0.664051537836463
TTC25	-0.271581343375961	0.285156355419801	0.0612025785349741	0.924603721716112	0.0525780000000004	0.82586230412069
MED9	-0.271603591754753	0.285156355419801	-0.289385879858406	0.488871711189599	-0.0414583333333325	0.862744795576892
PPP1R3E	-0.271676931175577	0.285000835606872	0.0158027647711222	0.982168834821517	0.00608300000000156	0.986471172041113
CYP8B1	-0.271682124147408	0.285000835606872	-0.0847678808051664	0.891309254245872	0.183788000000002	0.331900139634594
TMC2	-0.271682785832076	0.285000835606872	-0.0424641884477336	0.951446425515852	-0.0756109999999998	0.754561358073123
APOH	-0.271799710918436	0.284745471160953	-0.082593602952161	0.893766036626113	-0.0460989999999999	0.879377348071728
PKD2L2	-0.271964419161577	0.284376125680046	0.151208099247874	0.777604486471648	0.0709246666666667	0.754596031434316
MUC17	-0.271991695113184	0.284327838164811	-0.160110906726514	0.762902034786947	-0.0284033333333336	0.924315245272643
IGFLR1	-0.272014219584177	0.284300963119195	-0.00902590841227948	0.98909166696601	-0.0138529999999995	0.9622178119912
CSF3R	-0.272243871362915	0.283565803187508	-0.360592997905153	0.34034906917442	-0.0269673333333333	0.919456505549688
NUDT7	-0.272303881353788	0.283370573178198	-0.39025644334625	0.282383744168943	-0.00210633333333209	0.996285985364494
PNPLA1	-0.272311317012423	0.283370573178198	-0.00130105688339551	0.998348925592729	-0.218341999999999	0.338804872769334
NBEAL2	-0.272313612910469	0.283370573178198	-0.0919750354022137	0.879153312143409	-0.252092666666666	0.177614563220760
CIB1	-0.272375696277316	0.283345724835076	-0.00239055407863532	0.996089341390155	-0.0105926666666659	0.973215169933885
GKN2	-0.272388826067150	0.283345724835076	0.136664075560482	0.804079236721315	-0.0265746666666664	0.928988192507851
LARP1	-0.272416620606382	0.283310848783555	-0.0247022122942231	0.973288484737646	-0.092855666666666	0.662857774732191
SLC25A38	-0.272479372223373	0.283103469730965	-0.115357235974523	0.843755596423577	-0.193905666666666	0.387356917839865
SLC26A10	-0.272618054439533	0.282704355247740	0.430708536099578	0.206533702654214	0.0755756666666668	0.730776900973603
RDX	-0.272711030608680	0.282361814351943	-0.144834739142206	0.78956185520301	0.468363333333333	0.0309320552705188
SCN3B	-0.27277876852331	0.282132624876948	-0.289600693988541	0.488870852256136	0.0330526666666674	0.917723025232653
GLYAT	-0.272812117155496	0.282057569588856	-0.218599982466155	0.642399486366091	0.0245903333333336	0.934727271708501
C16orf91	-0.272843364921579	0.281991946193704	-0.138780485640742	0.798926084240744	-0.114437666666666	0.592988797958646
TFR2	-0.272866088710938	0.2819645140783	-0.213799340957562	0.653708954135733	0.0128146666666670	0.966149176465358
MAP1A	-0.272953591102782	0.281679791338077	-0.162299095457576	0.758164869707887	-0.353999666666665	0.167239997399651
ZFYVE28	-0.272956142934262	0.281679791338077	-0.282725638716216	0.502589413566017	-0.281550333333332	0.295467950792479
DAGLA	-0.27300658471019	0.281679791338077	-0.0375763340517093	0.955365651737247	-0.128271999999999	0.523453039510001
PDCD2	-0.273014085155809	0.281679791338077	0.0537649051402759	0.935666131096917	-0.121714333333332	0.589703432017128
SLC39A11	-0.273038928343533	0.281679791338077	0.0654599459083983	0.918111672450639	0.0177570000000006	0.95805797537369
H2BFM	-0.273371792554265	0.281038660449459	0.154276944027310	0.771685265099002	-0.115098333333333	0.704370299831112
TMEM116	-0.273467864727047	0.280760285324976	-0.300242189618839	0.465914681155138	0.244826333333334	0.212873167238858
MUTYH	-0.273602211174592	0.280362408714442	-0.0395934786097002	0.953730904892187	-0.0932689999999993	0.663776173049665
KRT9	-0.273719361754251	0.280009528923005	0.267787754224657	0.53559545997875	0.00507333333333389	0.991129100069248
KIAA1324	-0.273734175359301	0.280009528923005	-0.473330485020910	0.145443641689931	0.0158230000000007	0.966553712068514
RAB17	-0.273736535100524	0.280009528923005	0.178433209276176	0.727049269755538	-0.0798723333333329	0.790848042413284
EEF2K	-0.273752026735900	0.280009528923005	-0.507286619493176	0.103996171350646	-0.00197766666666527	0.996285985364494
NPTX2	-0.273862822863580	0.27989033178041	-0.118029435000103	0.838500126709059	0.201993333333334	0.427958580840173
GPR31	-0.273871402912411	0.27989033178041	0.0408077533771354	0.952426531595115	-0.103056999999999	0.648381288631965
ZNF783	-0.273987672821498	0.279705852975535	0.102273961204291	0.863936531316282	-0.0860343333333328	0.702709435280393
SLC17A1	-0.274141425208177	0.279169417322417	0.275931264387575	0.517549833373363	0.0619323333333336	0.830987625642722
CCDC112	-0.274150162330805	0.279169417322417	-0.188583228491187	0.707361603786947	0.242995666666667	0.372440180394709
ZDHHC21	-0.274153324828181	0.279169417322417	-0.703723292737156	0.00434969683222941	-0.314792666666665	0.159926616381325
CTBS	-0.274155531129822	0.279169417322417	-0.347125861385919	0.363894788429779	0.405442666666669	0.0766028512425147
MRPL51	-0.274282773680423	0.279061332841155	-0.242936988893356	0.592178030679976	0.0292180000000000	0.90945379489823
TTC37	-0.274327942238117	0.27900921090713	-0.323711690862112	0.414215860139795	0.118333333333334	0.564757468001238
VAMP8	-0.274572296012319	0.278156946255882	-0.414306670957423	0.237908245508916	-0.121027333333332	0.543819286485342

PSTPIP1	-0.274677864813894	0.277900024075102	-0.276499082773457	0.517266722508946	-0.0348703333333328	0.908443677591815
BRE	-0.274745250668981	0.277675265971723	-0.301837417450793	0.462852458460743	0.193645000000001	0.350950202066923
SLIRP	-0.274756124699846	0.277675265971723	-0.0842047369197663	0.892396784625427	-0.0372996666666657	0.887363245747406
CRYBB2	-0.274786483801605	0.277675265971723	0.200365021831014	0.681033026144457	-0.191991666666667	0.352897567720880
ANKRD13A	-0.274800096499418	0.277675265971723	-0.209705840573539	0.663785844870086	0.137989333333335	0.484446864118853
URM1	-0.274896318501364	0.277456846907581	-0.113340244081538	0.845747219093509	-0.146223333333332	0.477730273256987
MPP7	-0.275090218861871	0.276963133136439	-0.193335550184325	0.698337791293402	-0.354737666666665	0.141442533653629
LRRC1	-0.275100703221032	0.276963133136439	-0.387228480328474	0.287431505136616	0.307337666666667	0.135004030972507
PROL1	-0.275114681227756	0.276963133136439	0.0261194627095773	0.970632644458507	-0.038512666666666	0.874908057954497
KRT25	-0.275126654054627	0.276963133136439	0.222588064521275	0.635668764102271	-0.0615536666666662	0.820041994125866
TLX1	-0.27516561772468	0.276963133136439	0.127931215672077	0.817799920299584	-0.178125333333333	0.455122305861045
PHACTR3	-0.275179182123371	0.276963133136439	-0.179218254032666	0.725276394376351	-0.100413333333333	0.623903073849364
LIPI	-0.275242590147616	0.276808645696010	-0.113906775953551	0.844272085764738	0.00713966666666704	0.98349947509937
PF4V1	-0.275333053400644	0.276482454117300	-0.0664777920780257	0.916100593047005	0.0179350000000003	0.953336983324579
TSTA3	-0.275349271278002	0.276482454117300	0.208451772484277	0.666544605217271	-0.382768666666667	0.0769043559304575
FCN3	-0.275458587546809	0.276224381779812	-0.0119956806861245	0.986108499147084	-0.0569276666666657	0.872291063736238
FAM185A	-0.275467913973350	0.276224381779812	0.0826296071492979	0.893753725133293	-0.0876851666666655	0.710004432586908
LGALSL	-0.275476169613789	0.276224381779812	0.269527643699357	0.532361069863492	0.119659666666668	0.574278554232414
CEP120	-0.275493583534444	0.276224381779812	-0.27614867848569	0.517549833373363	0.145859333333334	0.521771474977985
IL22RA1	-0.275509404137968	0.276224381779812	0.374879383614494	0.313141424748220	0.101609333333334	0.650128617876381
MCM9	-0.27551821907991	0.276224381779812	-0.236761018603118	0.604321362942628	0.0652600000000001	0.813694870801638
OSGEP	-0.275559906225508	0.276222594779242	-0.307329446545915	0.453005595044643	-0.0658789999999988	0.808253941454262
IQCH	-0.275684018077585	0.275822562022099	-0.210747277330118	0.661480633411348	0.152291333333333	0.470855813271857
USP25	-0.275711703771394	0.275774594989137	-0.334188138662182	0.394920851245792	0.0384990000000008	0.885866310496875
WDR87	-0.27594212413683	0.275203190484879	0.0930105105324425	0.877331332619452	0.0695523333333335	0.762879150953755
PTPRD	-0.276149837440493	0.274435887358654	0.0999404294237912	0.867290404865575	-0.0764983333333328	0.761824584181416
KRCC1	-0.276158490610105	0.274435887358654	-0.0931549860320493	0.876995589714343	0.483505000000001	0.0656979579234737
SLC44A3	-0.276171295505742	0.274435887358654	-0.523570835696013	0.0865342407426229	0.184853666666668	0.367206442400058
ZNF385D	-0.276204321089613	0.274418607833627	-0.183059349630729	0.718112136382915	0.140212666666668	0.50393099142638
TMEM123	-0.276357900242897	0.273965160991352	-0.074557268367421	0.904832407812985	0.181280000000001	0.343572153228906
FLOT1	-0.276426455226422	0.273949517052422	-0.340798217847295	0.378272273903863	0.141740000000001	0.547637880318521
NDUFB4	-0.276448630686054	0.273937159010349	0.590026611985465	0.03694061604449	0.0865456666666672	0.682615907440032
ARHGEF37	-0.276503191913121	0.273845670374045	0.0118775930928546	0.986418801521542	-0.0278219999999995	0.942092082409632
ITGA5	-0.276794551116495	0.272917828639371	-0.368741419691075	0.32362669860138	0.431398333333335	0.0423336162071103
GCM1	-0.276814239116672	0.272917828639371	-0.0431679578137726	0.950119302534786	-0.0365283333333331	0.883478349705756
CLDN15	-0.276963068277317	0.272419509957671	-0.031922423917147	0.96317863582128	-0.0861026666666663	0.685271038967111
PRX	-0.276987055716646	0.272401363387609	0.128148764243982	0.817090309757872	0.00687000000000056	0.986290211615582
DMXL1	-0.276988955689521	0.272401363387609	-0.387632055526212	0.286134842484356	0.194360333333334	0.390561931914011
TEX15	-0.27702063111251	0.272401363387609	-0.343930306158051	0.371952624889347	0.216147333333334	0.390561931914011
WWC1	-0.27722107911289	0.271881869589543	-0.256475740850336	0.564430426783556	-0.200159	0.361971299676959
PLEK2	-0.277404259220922	0.271228578025712	0.106332650672461	0.858284876027025	0.00284500000000033	0.993756838831997
FOXN4	-0.277540505264482	0.271003124729684	-0.0979226540489984	0.868852648210739	-0.112385333333332	0.604179762453377
JAZF1	-0.277564807995977	0.270970958431909	0.148005297433094	0.784080588385358	0.346055666666668	0.0748141981193211
LINC00277	-0.277577345665201	0.270970958431909	-0.260345059351736	0.554191999148329	0.134965333333334	0.523741417815996
N6AMT1	-0.277613897455941	0.270970958431909	-0.0117822231247221	0.986418801521542	-0.151705999999999	0.472370507912389
NSUN5P1	-0.27774324341279	0.270496146422345	-0.336122382601419	0.389476012645306	-0.134309666666666	0.532982474830836
IL9	-0.278283276516564	0.269022391594142	0.0885759825990768	0.884621657203655	-0.0947966666666661	0.671492528470281
TAS2R41	-0.278319899921227	0.268974723654341	0.179938149820167	0.723716289903531	0.00495000000000001	0.988133186272453
SHMT1	-0.278499077324713	0.268453792753664	-0.151656194866631	0.777016024293745	0.0767710000000013	0.720355976568153
DCAF12	-0.278516946454249	0.26844997931612	-0.0786724235185218	0.899281198071059	-0.0162763333333331	0.95482028291900
SNX13	-0.278549756173619	0.268381323301682	-0.0138015999059635	0.983872723592937	-0.150445666666665	0.594374666543557
RTBDN	-0.278673250040367	0.267979770131987	-0.207467110928128	0.668625825887966	-0.00806633333333297	0.987937064305561
TDH	-0.27872247191277	0.267979770131987	0.142860706787112	0.793915668332488	-0.104408666666666	0.630907197885932
RAP2C	-0.278805398825137	0.267838337057856	-0.185228597441763	0.71508811352043	-0.0482913333333328	0.834140029892063
CALCR	-0.279015293090780	0.267174519317948	-0.408674340482046	0.250192979708184	-1.28387633333333	0.193702415859911
DUOXA2	-0.279040855473939	0.267137567183018	0.219468907969430	0.641226345931986	-0.0940563333333332	0.731425088367868
RPP25L	-0.279091429189026	0.266992423074550	-0.144929526768010	0.78939605015755	0.135524666666667	0.62418880357415
SLC6A11	-0.279135208989549	0.266876721194928	0.219919280927313	0.641001372559671	-0.0154686666666665	0.95784037718285
FFAR2	-0.279160541814982	0.266840821580040	-0.125904599461910	0.822438385407788	-0.111508666666666	0.5949348797385
UPK3B	-0.279205934968892	0.266791868783658	-0.165889201550196	0.751869806279813	0.0164616666666676	0.955460918122607
FGF10	-0.279207837535735	0.266791868783658	0.338319705900426	0.384701467775014	0.0743836666666673	0.765209686566971
CDKN2D	-0.279215436601132	0.266791868783658	0.131388372254304	0.811227868886268	0.127525666666667	0.525143015861964
NEU1	-0.279282618432953	0.266681400741383	-0.0196817874967891	0.97763669370967	-0.066683222222222	0.817735562413299
PPFIBP2	-0.279319390667555	0.266596148769044	-0.449498720593789	0.177460282309099	-0.161278666666666	0.495283817122373
ANAPC16	-0.279328277952839	0.266596148769044	0.197072116598946	0.689903407953139	0.236704000000000	0.205838922584493
ENAM	-0.279369579845493	0.266596148769044	-0.113503028437471	0.84538474875759	-0.0604929999999998	0.795703940655181
DEDD2	-0.279424319273058	0.266564041897205	-0.352434902121501	0.352921806679009	-0.0897653333333328	0.68171195530621
ATP5F1	-0.279444859906621	0.266564041897205	-0.468336164178779	0.151936428121882	-0.00765766666666626	0.98256318768706
EMR3	-0.279485178930433	0.266543547857762	-0.238615891479174	0.600861960738847	-0.163192666666666	0.417125112561133
C14orf119	-0.27953225310349	0.266474722312921	0.0232400111105676	0.975348968561756	0.0430896666666674	0.884064277940346
SP140L	-0.279585302474198	0.266406264829189	-0.244283682138877	0.588630958916351	0.138299000000001	0.522208227467442
TRPM1	-0.279671281337961	0.266256434726935	0.152003792631067	0.776146388301083	-0.103686000000000	0.654323362279664
ZNF492	-0.279693582404429	0.266256434726935	-0.519291309695772	0.0902262536464163	-0.491633666666665	0.0327266263473543
SLC27A5	-0.279723116319541	0.266254299424955	-0.0869902596952468	0.887055939766881	-0.0321726666666669	0.91148338318481
COQ7	-0.279758049914321	0.266177316048286	-0.0875169147081849	0.886302037299497	-0.252723999999998	0.204717851336330
DVL1	-0.279812287117109	0.266076173627559	-0.124845739877605	0.824472060590171	-0.228762666666666	0.2985680874726
CLDN3	-0.279838224402575	0.266052908248728	-0.0240684326180459	0.974290606776992	-0.125176333333333	0.716268843468132
TRAPPC6A	-0.279898436575657	0.265866825970844	0.210336242065355	0.661902887633655	-0.352530333333333	0.114599211633843
CDK7	-0.279947834700433	0.265801523078537	-0.266541551064960	0.538833471023229	-0.000432333333333288	0.99868162314374
SNCG	-0.280015071903419	0.265585269433136	-0.138911382574663	0.798791960356084	-0.0520080000000005	0.85272965579086

KEL	-0.280041154577193	0.265546695935226	0.0379514903417699	0.954889910016503	-0.0567526666666666	0.80578153770169
COPS8	-0.280045158044042	0.265546695935226	-0.082442192868831	0.894013947727154	0.0813990000000014	0.728911421670294
NLGN3	-0.280057461151699	0.265546695935226	-0.065961853925586	0.917090201751967	-0.133938	0.607009506431467
ALAS1	-0.280169262947339	0.265246526906541	0.125160308777554	0.824230922676958	-0.0334986666666661	0.898979600726666
FBXL15	-0.280278350826644	0.265041650411041	-0.197306908197662	0.689256317041816	0.0148886666666667	0.961657879696991
HSP90AA1	-0.280358309613067	0.264771098999370	-0.156831501513894	0.768401006769299	0.0161600000000022	0.956567100065073
ZNF75A	-0.280370978909322	0.264771098999370	-0.12788527445182	0.817865585528586	0.356167333333334	0.0917903726055603
CYP3A7-CYP3AP1	-0.280372298893384	0.264771098999370	-0.217177518625009	0.646495351690352	0.282775000000000	0.198155014578346
BSN	-0.28038140157073	0.264771098999370	-0.143803080369799	0.791963790519804	0.165716333333334	0.443378989641463
CLDN23	-0.280453421103401	0.264657330135584	0.0216509897538612	0.97536722461972	-0.264432	0.359908305595919
OR7D2	-0.280478030669684	0.264625346326138	-0.209835401373446	0.663436500730575	-0.0484336666666661	0.841976773189511
GJD4	-0.280513505956113	0.264546593089509	0.286370256335912	0.495728580458409	-0.0650145000000001	0.808253941454262
TMEM17	-0.28060987237871	0.264501881836858	-0.143376071601634	0.79287526984225	0.322277000000001	0.279049509382533
TSNAXIP1	-0.280683859778645	0.264469823623908	0.0990835336541968	0.868042453226125	0.0252553333333338	0.923019543204612
IRAK4	-0.280704738030134	0.264469823623908	-0.626785615412917	0.0225578375274624	0.0295336666666668	0.921287736444926
NOC2L	-0.280778314091614	0.264369781202308	-0.399638321950131	0.265001036100484	-0.27912566666666	0.138021116806123
EML1	-0.28086060988747	0.264163909406868	-0.248524396710715	0.57746120283043	0.0426696666666677	0.886909407509907
C5orf28	-0.280872663862809	0.264163909406868	0.194693196077450	0.69591561649809	0.168985333333335	0.373955108831303
FDX1L	-0.280903247657372	0.264128833735171	0.214931247128036	0.651376266020544	0.177724333333335	0.348661840021751
ANXA9	-0.280904532026642	0.264128833735171	-0.00123530235091022	0.998348925592729	-0.165418333333333	0.580149967309238
AQP3	-0.280935875154620	0.264128833735171	-0.387713205080224	0.286134842484356	0.201562666666668	0.408369243753251
NMB	-0.280937862729441	0.264128833735171	-0.180367236246204	0.722985510661031	-0.139849333333333	0.62420320780168
ZNF786	-0.28094815994833	0.264128833735171	-0.209863282375832	0.663436500730575	-0.202396999999999	0.462444913819465
RBMS1	-0.280971470848843	0.264128833735171	-0.363929916191873	0.334903767060811	0.298354166666668	0.212382159505009
PPP1R13L	-0.280999043621102	0.264128833735171	-0.445204217968972	0.181308964012049	0.181433333333334	0.386271329500871
WDTC1	-0.281029683642359	0.264128833735171	-0.518455819951108	0.0905875685853887	0.0786396666666672	0.741438256509195
COQ2	-0.281050363318683	0.264128833735171	-0.254469531701341	0.567650054720788	0.269221333333333	0.172629239571161
SGPP1	-0.281089224909816	0.264128833735171	-0.0974100978305327	0.869861570992203	0.339387000000001	0.0864039053489188
ACO2	-0.281126927267228	0.264058229483333	-0.335816695440347	0.389949066081534	-0.00267166666666659	0.993756838831997
GSTK1	-0.281139322816624	0.264058229483333	0.0784161675574822	0.899281198071059	0.0290113333333334	0.914418029309221
ZNF468	-0.281224939388025	0.263934066070848	-0.546204192528999	0.0635875421378674	0.0685446666666671	0.758920309140801
LCNL1	-0.281317239827349	0.263611353383299	0.0473289402247302	0.943981149721771	-0.14269	0.62570317772964
POU4F2	-0.281413042557137	0.263273889951982	0.173701776751628	0.735513734442976	-0.0664013333333333	0.831331052726554
KNG1	-0.281452653841155	0.263178066015452	-0.142048556622788	0.79450771779567	0.103782333333334	0.673701601183362
SLC7A13	-0.281641673495588	0.262663916736577	0.152245882471417	0.775863712880333	-0.191741999999999	0.344586016841321
GJB1	-0.28165432950463	0.262663916736577	0.197946180734783	0.687681948030762	-0.0631256666666662	0.839998150752089
RNPC3	-0.281788073853929	0.262258997988652	-0.54625328420858	0.0635875421378674	0.201181000000001	0.414405354802607
INO80E	-0.281929738746885	0.262140461694217	0.146917084008311	0.78589154083134	-0.0448643333333324	0.852020205196772
CHAC2	-0.281934461106447	0.262140461694217	-0.0375193820800705	0.955411256179887	-0.0720149999999989	0.758341714501375
CDK19	-0.281953148952073	0.262140461694217	-0.113233055863488	0.845978328141518	0.293188333333334	0.121310299227152
CYP7A1	-0.282072024519081	0.261934292468404	-0.340656178633099	0.378350877728096	0.216090000000001	0.305655404008506
KLHL2	-0.282161058630075	0.261627405026628	-0.175277707716836	0.732640079776942	0.136368333333334	0.692043732062832
SPG11	-0.282235327580789	0.261458260820562	-0.240075955679281	0.598092982260025	0.212188666666668	0.334527228768702
PI3	-0.28234054574211	0.261156897535426	-0.294435970450641	0.479692636619539	-0.0615633333333327	0.843152489417868
B4GALNT2	-0.282364216560057	0.26113000841191	0.140335177074443	0.797441782082887	0.0537153333333341	0.88616294579686
TMEM229B	-0.282594851432504	0.260293694382581	0.134578685371808	0.808009477165488	-0.328453333333332	0.117653089562184
MTRR	-0.282600230027668	0.260293694382581	-0.612821690478367	0.0277519681937494	-0.141367666666666	0.523741417815996
OGDHL	-0.282695036202584	0.260022393410511	-0.292297482188439	0.484076751426251	-0.140833333333334	0.590841338570121
C2orf76	-0.282695726117762	0.260022393410511	0.00702821090026437	0.991222489895419	0.293563333333334	0.264889721029722
CHMP4A	-0.282732396913681	0.260022393410511	-0.329874206980152	0.40362877792936	0.00220566666666690	0.994738356997797
LHPP	-0.282760572847079	0.260022393410511	0.0919807115695914	0.879153312143409	0.0400746666666673	0.879730542916545
GRIA2	-0.282763363964305	0.260022393410511	-0.185410926341618	0.714444278035968	0.128034666666667	0.621414476984274
METTL17	-0.282774553420099	0.260022393410511	-0.0879956645598355	0.885848000380851	0.0526960000000018	0.83616423402744
RAB20	-0.282816418276933	0.260022393410511	-0.148410070620552	0.78374265784053	-0.097034	0.661470161772113
ALDH9A1	-0.282855982700632	0.260022393410511	-0.0344340576254647	0.959404955370931	0.0552140000000005	0.813694870801638
ANAPC4	-0.283007959925205	0.259794375234072	-0.450713109694385	0.176663460992485	0.108655333333335	0.600403709971156
[truncated: 244,854 more chars]
